# Supplementary material for: The basic psychological needs satisfaction and frustration scale at work: A validation in the Polish language
Source: PLoS One. 2021 Nov 4;16(11):e0258948. doi: 10.1371/journal.pone.0258948 (PMC8568341; doi:10.1371/journal.pone.0258948)
Supplement: S1 File — (PDF) [file pone.0258948.s002.pdf]

C:\Users\nikmo_000\Downloads\MICHA LUKASZ INVARIANCE PAPER\SECOND ROUND INVARIANCE.amw

Analysis Summary

Date and Time

Date: Monday, January 4, 2021
Time: 10:32:14 PM

Title

Second round invariance: Monday, January 4, 2021 10:32 PM

Groups

Group number 1 (Group number 1)

Notes for Group (Group number 1)

The model is recursive.
Sample size = 439

Variable Summary (g1)

Your model contains the following variables (g1)

Observed, endogenous variables

BPNSF19
BPNSF13
BPNSF7
BPNSF1
BPNSF18
BPNSF15
BPNSF10
BPNSF5
BPNSF24
BPNSF16
BPNSF12
BPNSF4
BPNSF22
BPNSF20
BPNSF8
BPNSF2
BPNSF21
BPNSF14
BPNSF9
BPNSF3
BPNSF23
BPNSF17
BPNSF11
BPNSF6

Unobserved, exogenous variables

F1
e1
e2
e3
e4
F2
e5
e6
e7
e8
F3
e9
e10
e11
e12
F4
e13

e14
e15
e16
F5
e17
e18
e19
e20
F6
e21
e22
e23
e24

Variable counts (g1)

**Number of variables in your model:**	54
**Number of observed variables:**	24
**Number of unobserved variables:**	30
**Number of exogenous variables:**	30
**Number of endogenous variables:**	24

Parameter Summary (g1)

	Weights	Covariances	Variances	Means	Intercepts	Total
**Fixed**	30	0	0	30	0	60
**Labeled**	18	15	30	0	24	87
**Unlabeled**	0	0	0	0	0	0
**Total**	48	15	30	30	24	147

Sample Moments (g1)

Sample Covariances (g1)

	BPNSF6	BPNSF11	BPNSF17	BPNSF23	BPNSF3	BPNSF9	BPNSF14	BPNSF21	BPNSF2	BPNSF8	BPNSF20	BPNSF22	BPNSF4	BPNSF12	BPNSF16	BPNS[illegible]
**BPNSF6**	2.654															
**BPNSF11**	1.465	2.683														
**BPNSF17**	1.408	1.399	2.556													
**BPNSF23**	1.374	1.461	1.326	2.342												
**BPNSF3**	-.859	-.776	-.746	-.981	1.889											
**BPNSF9**	-.908	-.848	-.856	-.999	.937	1.929										
**BPNSF14**	-.803	-.718	-.857	-.922	.930	1.092	2.019									
**BPNSF21**	-.687	-.729	-.585	-.887	.742	.910	1.093	2.038								
**BPNSF2**	1.264	1.379	1.086	1.440	-.939	-.882	-.900	-.819	2.651							
**BPNSF8**	1.446	1.497	1.270	1.189	-.591	-.826	-.632	-.422	1.409	2.707						
**BPNSF20**	1.186	1.187	1.282	1.449	-.814	-.809	-.837	-.736	1.328	1.305	2.297					
**BPNSF22**	.910	1.031	.796	1.077	-.590	-.540	-.418	-.425	1.127	1.308	1.103	2.072				
**BPNSF4**	-.503	-.368	-.361	-.353	.627	.445	.693	.424	-.558	-.378	-.468	-.360	1.462			
**BPNSF12**	-.611	-.393	-.558	-.505	.603	.710	.893	.667	-.622	-.537	-.607	-.588	.674	1.699		
**BPNSF16**	-.691	-.555	-.613	-.659	.559	.719	.816	.700	-.604	-.585	-.618	-.489	.642	1.002	1.639	
**BPNSF24**	-.510	-.444	-.528	-.557	.612	.750	.593	.544	-.719	-.545	-.631	-.591	.580	.910	.836	1.5[illegible]
**BPNSF5**	.785	.933	.646	.650	-.184	-.294	-.214	-.282	.787	.775	.613	.611	-.042	-.418	-.466	-.4[illegible]
**BPNSF10**	.886	1.394	.795	.761	-.210	-.379	-.174	-.274	.910	1.093	.648	.553	-.250	-.446	-.381	-.2[illegible]
**BPNSF15**	.601	.779	.557	.367	-.210	-.159	.167	-.197	.376	.665	.462	.450	-.129	-.362	-.107	-.2[illegible]
**BPNSF18**	-.157	.062	.109	-.065	.186	.254	.056	.389	.051	.146	-.091	.131	.026	.108	.192	.0[illegible]
**BPNSF1**	-.280	-.269	-.086	-.246	.608	.394	.359	.587	-.164	-.135	-.219	-.183	.611	.552	.450	.4[illegible]
**BPNSF7**	-.269	-.432	-.130	-.468	.596	.799	.652	.880	-.555	-.090	-.229	-.185	.309	.386	.336	.3[illegible]
**BPNSF13**	-.641	-.575	-.581	-.669	.725	.850	1.295	1.050	-.649	-.383	-.647	-.378	.583	.939	.771	.6[illegible]
**BPNSF19**	-.566	-.679	-.684	-.848	.740	.771	1.023	1.252	-.811	-.426	-.740	-.428	.450	.735	.757	.5[illegible]

Condition number = 39.649

Eigenvalues
17.595 5.922 3.626 2.517 2.070 1.966 1.893 1.677 1.581 1.420 1.279 1.224 1.099 1.072 1.052 .971 .869 .824 .732 .687 .663 .575 .534 .444

Determinant of sample covariance matrix = 1688.756

Sample Correlations (g1)

	BPNSF6	BPNSF11	BPNSF17	BPNSF23	BPNSF3	BPNSF9	BPNSF14	BPNSF21	BPNSF2	BPNSF8	BPNSF20	BPNSF22	BPNSF4	BPNSF12	BPNSF16	BPNS[illegible]
**BPNSF6**	1.000															
**BPNSF11**	.549	1.000														
**BPNSF17**	.541	.534	1.000													
**BPNSF23**	.551	.583	.542	1.000												

	BPNSF6	BPNSF11	BPNSF17	BPNSF23	BPNSF3	BPNSF9	BPNSF14	BPNSF21	BPNSF2	BPNSF8	BPNSF20	BPNSF22	BPNSF4	BPNSF12	BPNSF16	BPNS
**BPNSF3**	-.384	-.345	-.340	-.466	1.000											
**BPNSF9**	-.401	-.373	-.386	-.470	.491	1.000										
**BPNSF14**	-.347	-.309	-.377	-.424	.476	.553	1.000									
**BPNSF21**	-.295	-.312	-.256	-.406	.378	.459	.539	1.000								
**BPNSF2**	.476	.517	.417	.578	-.420	-.390	-.389	-.352	1.000							
**BPNSF8**	.539	.556	.483	.472	-.261	-.361	-.270	-.180	.526	1.000						
**BPNSF20**	.480	.478	.529	.625	-.391	-.384	-.389	-.340	.538	.523	1.000					
**BPNSF22**	.388	.437	.346	.489	-.298	-.270	-.204	-.207	.481	.552	.505	1.000				
**BPNSF4**	-.255	-.186	-.187	-.191	.377	.265	.404	.246	-.283	-.190	-.255	-.207	1.000			
**BPNSF12**	-.288	-.184	-.268	-.253	.337	.392	.482	.358	-.293	-.250	-.307	-.314	.428	1.000		
**BPNSF16**	-.331	-.265	-.299	-.336	.318	.404	.449	.383	-.290	-.278	-.318	-.265	.414	.600	1.000	
**BPNSF24**	-.253	-.219	-.266	-.293	.359	.436	.337	.307	-.356	-.267	-.336	-.331	.387	.563	.527	1.0
**BPNSF5**	.316	.373	.265	.278	-.088	-.139	-.099	-.130	.317	.309	.265	.278	-.023	-.210	-.239	-.2
**BPNSF10**	.334	.522	.305	.305	-.094	-.168	-.075	-.118	.343	.408	.262	.236	-.127	-.210	-.183	-.1
**BPNSF15**	.237	.305	.223	.154	-.098	-.074	.075	-.088	.148	.259	.196	.201	-.069	-.178	-.053	-.1
**BPNSF18**	-.066	.026	.047	-.029	.093	.125	.027	.187	.021	.061	-.041	.062	.015	.057	.103	.0
**BPNSF1**	-.115	-.110	-.036	-.108	.297	.190	.170	.276	-.068	-.055	-.097	-.085	.339	.284	.236	.2
**BPNSF7**	-.108	-.172	-.053	-.200	.284	.376	.300	.403	-.223	-.036	-.099	-.084	.167	.194	.172	.2
**BPNSF13**	-.284	-.253	-.262	-.315	.380	.441	.657	.530	-.287	-.168	-.308	-.189	.348	.519	.434	.3
**BPNSF19**	-.241	-.287	-.297	-.384	.373	.384	.499	.607	-.345	-.179	-.338	-.206	.258	.391	.409	.3

Condition number = 35.474

Eigenvalues
7.988 2.668 1.577 1.302 .978 .928 .872 .720 .696 .642 .598 .558 .506 .493 .465 .435 .418 .393 .359 .331 .304 .278 .266 .225

Sample Means (g1)

	BPNSF6	BPNSF11	BPNSF17	BPNSF23	BPNSF3	BPNSF9	BPNSF14	BPNSF21	BPNSF2	BPNSF8	BPNSF20	BPNSF22	BPNSF4	BPNSF12	BPNSF16	BPNSF24	BP
	2.458	2.690	2.440	2.068	5.716	5.848	5.585	5.359	2.230	2.768	2.351	3.015	5.068	5.230	5.112	5.244	3.

Group number 2 (Group number 2)

Notes for Group (Group number 2)

The model is recursive.
Sample size = 437

Variable Summary (g2)

Your model contains the following variables (g2)

Observed, endogenous variables

BPNSF19
BPNSF13
BPNSF7
BPNSF1
BPNSF18
BPNSF15
BPNSF10
BPNSF5
BPNSF24
BPNSF16
BPNSF12
BPNSF4
BPNSF22
BPNSF20
BPNSF8
BPNSF2
BPNSF21
BPNSF14
BPNSF9
BPNSF3
BPNSF23
BPNSF17
BPNSF11
BPNSF6

Unobserved, exogenous variables

F1
e1
e2
e3

e4
F2
e5
e6
e7
e8
F3
e9
e10
e11
e12
F4
e13
e14
e15
e16
F5
e17
e18
e19
e20
F6
e21
e22
e23
e24

Variable counts (g2)

**Number of variables in your model:**	54
**Number of observed variables:**	24
**Number of unobserved variables:**	30
**Number of exogenous variables:**	30
**Number of endogenous variables:**	24

Parameter Summary (g2)

	Weights	Covariances	Variances	Means	Intercepts	Total
**Fixed**	30	0	0	30	0	60
**Labeled**	18	15	30	0	24	87
**Unlabeled**	0	0	0	0	0	0
**Total**	48	15	30	30	24	147

Sample Moments (g2)

Sample Covariances (g2)

	BPNSF6	BPNSF11	BPNSF17	BPNSF23	BPNSF3	BPNSF9	BPNSF14	BPNSF21	BPNSF2	BPNSF8	BPNSF20	BPNSF22	BPNSF4	BPNSF12	BPNSF16	BPNS[illegible]
**BPNSF6**	2.935															
**BPNSF11**	1.185	2.891														
**BPNSF17**	1.345	1.538	2.894													
**BPNSF23**	1.322	1.439	1.736	2.728												
**BPNSF3**	-.854	-.665	-.863	-.888	2.275											
**BPNSF9**	-.784	-.929	-1.155	-1.047	1.130	2.200										
**BPNSF14**	-.677	-.785	-1.129	-.857	.978	1.254	2.018									
**BPNSF21**	-.699	-.848	-.782	-.889	1.049	.994	1.117	2.225								
**BPNSF2**	1.189	1.116	1.365	1.372	-.765	-.749	-.802	-.754	2.610							
**BPNSF8**	1.291	1.254	1.426	1.311	-.682	-.792	-.711	-.753	1.481	3.053						
**BPNSF20**	1.076	1.292	1.480	1.739	-.851	-.825	-.888	-.998	1.512	1.610	2.918					
**BPNSF22**	1.001	1.163	1.294	1.299	-.461	-.760	-.713	-.820	1.306	1.405	1.609	2.740				
**BPNSF4**	-.645	-.592	-.667	-.699	1.404	.874	.826	1.090	-.856	-.776	-.958	-.764	2.153			
**BPNSF12**	-.578	-.708	-.851	-.796	.880	.977	1.055	1.080	-.904	-.845	-.930	-1.038	1.144	2.051		
**BPNSF16**	-.518	-.694	-.782	-.694	.757	.856	1.029	1.175	-.808	-.801	-.999	-.961	1.025	1.414	2.156	
**BPNSF24**	-.549	-.648	-.920	-.730	.629	.791	.974	.980	-.881	-.905	-1.055	-1.135	.934	1.186	1.250	2.1[illegible]
**BPNSF5**	.901	.664	.736	.895	-.443	-.324	-.358	-.518	.837	1.118	1.015	.953	-.726	-.488	-.400	-.5[illegible]
**BPNSF10**	.888	1.402	1.203	1.251	-.629	-.773	-.742	-.889	1.118	1.337	1.373	1.279	-.709	-.700	-.653	-.7[illegible]
**BPNSF15**	.868	1.167	1.001	.873	-.395	-.318	-.299	-.448	.856	1.100	1.114	1.167	-.530	-.489	-.445	-.5[illegible]
**BPNSF18**	.218	.641	.581	.366	.012	-.130	-.072	-.247	.353	.504	.663	.522	-.170	-.176	-.202	-.2[illegible]
**BPNSF1**	-.287	-.351	-.373	-.412	.816	.663	.765	.894	-.640	-.473	-.633	-.542	.863	.708	.639	.6[illegible]
**BPNSF7**	-.041	-.514	-.685	-.340	.691	.950	.850	.813	-.497	-.606	-.527	-.699	.763	.752	.749	.7[illegible]
**BPNSF13**	-.534	-.777	-.703	-.681	.907	.921	1.138	1.216	-.634	-.781	-.780	-.840	.917	1.205	1.040	1.0[illegible]
**BPNSF19**	-.609	-.855	-.898	-.873	.772	.981	1.032	1.154	-.562	-.693	-.843	-.797	.820	.823	.775	.6[illegible]

Condition number = 43.436

Eigenvalues
22.127 5.874 3.293 2.619 2.544 2.025 1.897 1.759 1.705 1.602 1.419 1.379 1.287 1.205 1.161 1.035 .944 .926 .877 .806 .767 .644 .599 .509

Determinant of sample covariance matrix = 17870.592

Sample Correlations (g2)

	BPNSF6	BPNSF11	BPNSF17	BPNSF23	BPNSF3	BPNSF9	BPNSF14	BPNSF21	BPNSF2	BPNSF8	BPNSF20	BPNSF22	BPNSF4	BPNSF12	BPNSF16	BPNS[illegible]
**BPNSF6**	1.000															
**BPNSF11**	.407	1.000														
**BPNSF17**	.462	.532	1.000													
**BPNSF23**	.467	.513	.618	1.000												
**BPNSF3**	-.331	-.259	-.336	-.356	1.000											
**BPNSF9**	-.309	-.369	-.458	-.427	.505	1.000										
**BPNSF14**	-.278	-.325	-.467	-.365	.457	.595	1.000									
**BPNSF21**	-.274	-.335	-.308	-.361	.466	.449	.527	1.000								
**BPNSF2**	.429	.406	.497	.514	-.314	-.312	-.349	-.313	1.000							
**BPNSF8**	.431	.422	.480	.454	-.259	-.306	-.286	-.289	.525	1.000						
**BPNSF20**	.368	.445	.509	.616	-.330	-.326	-.366	-.392	.548	.540	1.000					
**BPNSF22**	.353	.413	.460	.475	-.185	-.309	-.303	-.332	.489	.486	.569	1.000				
**BPNSF4**	-.257	-.237	-.267	-.289	.635	.402	.396	.498	-.361	-.303	-.382	-.314	1.000			
**BPNSF12**	-.236	-.291	-.350	-.337	.407	.460	.519	.506	-.391	-.338	-.380	-.438	.544	1.000		
**BPNSF16**	-.206	-.278	-.313	-.286	.342	.393	.493	.537	-.341	-.312	-.398	-.396	.476	.672	1.000	
**BPNSF24**	-.218	-.259	-.368	-.300	.284	.362	.466	.447	-.371	-.352	-.420	-.466	.433	.563	.579	1.0[illegible]
**BPNSF5**	.340	.252	.280	.350	-.190	-.141	-.163	-.224	.335	.414	.384	.372	-.320	-.220	-.176	-.2[illegible]
**BPNSF10**	.305	.486	.417	.446	-.246	-.307	-.308	-.351	.407	.451	.473	.455	-.284	-.288	-.262	-.3[illegible]
**BPNSF15**	.290	.393	.337	.303	-.150	-.123	-.121	-.172	.303	.360	.373	.404	-.207	-.196	-.173	-.2[illegible]
**BPNSF18**	.083	.246	.223	.145	.005	-.057	-.033	-.108	.143	.188	.254	.206	-.076	-.080	-.090	-.1[illegible]
**BPNSF1**	-.118	-.146	-.155	-.176	.382	.316	.380	.424	-.280	-.191	-.262	-.231	.416	.349	.307	.3[illegible]
**BPNSF7**	-.016	-.210	-.279	-.143	.318	.445	.415	.378	-.214	-.241	-.214	-.293	.361	.365	.354	.3[illegible]
**BPNSF13**	-.226	-.331	-.299	-.298	.435	.450	.580	.590	-.284	-.323	-.330	-.367	.453	.609	.513	.4[illegible]
**BPNSF19**	-.233	-.329	-.345	-.346	.335	.433	.475	.506	-.228	-.260	-.323	-.315	.366	.376	.346	.2[illegible]

Condition number = 36.760

Eigenvalues
8.949 2.424 1.298 1.095 1.043 .830 .794 .676 .658 .638 .594 .538 .487 .467 .459 .438 .412 .403 .361 .335 .321 .283 .254 .243

Sample Means (g2)

	BPNSF6	BPNSF11	BPNSF17	BPNSF23	BPNSF3	BPNSF9	BPNSF14	BPNSF21	BPNSF2	BPNSF8	BPNSF20	BPNSF22	BPNSF4	BPNSF12	BPNSF16	BPNSF24	BP[illegible]
	2.616	2.761	2.474	2.173	5.619	5.725	5.607	5.240	2.309	2.731	2.439	3.078	5.077	5.314	5.218	5.311	3.[illegible]

Group number 3 (Group number 3)

Notes for Group (Group number 3)

The model is recursive.
Sample size = 431

Variable Summary (g3)

Your model contains the following variables (g3)

Observed, endogenous variables

BPNSF19
BPNSF13
BPNSF7
BPNSF1
BPNSF18
BPNSF15
BPNSF10
BPNSF5
BPNSF24
BPNSF16
BPNSF12
BPNSF4
BPNSF22
BPNSF20
BPNSF8
BPNSF2
BPNSF21
BPNSF14
BPNSF9

BPNSF3
BPNSF23
BPNSF17
BPNSF11
BPNSF6

Unobserved, exogenous variables

F1
e1
e2
e3
e4
F2
e5
e6
e7
e8
F3
e9
e10
e11
e12
F4
e13
e14
e15
e16
F5
e17
e18
e19
e20
F6
e21
e22
e23
e24

Variable counts (g3)

**Number of variables in your model:**	54
**Number of observed variables:**	24
**Number of unobserved variables:**	30
**Number of exogenous variables:**	30
**Number of endogenous variables:**	24

Parameter Summary (g3)

	Weights	Covariances	Variances	Means	Intercepts	Total
**Fixed**	30	0	0	30	0	60
**Labeled**	18	15	30	0	24	87
**Unlabeled**	0	0	0	0	0	0
**Total**	48	15	30	30	24	147

Sample Moments (g3)

Sample Covariances (g3)

	BPNSF6	BPNSF11	BPNSF17	BPNSF23	BPNSF3	BPNSF9	BPNSF14	BPNSF21	BPNSF2	BPNSF8	BPNSF20	BPNSF22	BPNSF4	BPNSF12	BPNSF16	BPNS[illegible]
**BPNSF6**	3.011															
**BPNSF11**	1.647	2.909														
**BPNSF17**	1.558	1.765	3.203													
**BPNSF23**	1.535	1.775	1.902	3.025												
**BPNSF3**	-.869	-.886	-1.025	-1.073	2.157											
**BPNSF9**	-1.077	-1.288	-1.378	-1.173	1.127	2.169										
**BPNSF14**	-.761	-1.002	-1.167	-1.079	.953	1.357	2.200									
**BPNSF21**	-.652	-.925	-.677	-.966	.762	.938	.994	1.905								
**BPNSF2**	1.174	1.480	1.418	1.652	-.939	-.761	-.738	-.492	3.232							
**BPNSF8**	1.433	1.752	1.640	1.607	-.790	-1.042	-.672	-.660	1.754	3.160						
**BPNSF20**	1.285	1.553	1.896	1.961	-.935	-1.068	-.825	-.731	1.646	1.786	3.155					
**BPNSF22**	.915	1.206	1.122	1.416	-.682	-.641	-.507	-.637	1.229	1.534	1.457	2.468				
**BPNSF4**	-.554	-.587	-.503	-.587	1.039	.687	.564	.636	-.410	-.600	-.598	-.594	1.722			
**BPNSF12**	-.617	-.822	-.894	-.960	.815	1.162	1.203	1.001	-.741	-.730	-.838	-.827	.876	2.091		
**BPNSF16**	-.525	-.798	-.872	-.776	.627	.901	1.038	.937	-.632	-.665	-.889	-.738	.790	1.328	1.925	
**BPNSF24**	-.642	-.859	-.653	-.862	.581	.786	.823	.983	-.698	-.806	-.807	-.593	.682	1.064	.933	1.8[illegible]

	BPNSF6	BPNSF11	BPNSF17	BPNSF23	BPNSF3	BPNSF9	BPNSF14	BPNSF21	BPNSF2	BPNSF8	BPNSF20	BPNSF22	BPNSF4	BPNSF12	BPNSF16	BPNS
**BPNSF5**	1.069	1.246	1.035	1.074	-.395	-.537	-.417	-.343	1.464	1.350	1.054	1.025	-.466	-.573	-.575	-.5
**BPNSF10**	1.285	1.690	1.446	1.361	-.677	-.910	-.591	-.527	1.371	1.613	1.279	1.118	-.528	-.581	-.552	-.5
**BPNSF15**	.704	.886	.975	.646	-.341	-.426	-.357	-.222	.804	1.083	.776	.740	-.444	-.275	-.336	-.3
**BPNSF18**	.139	.300	.410	.104	-.100	.156	.256	.084	.210	.246	.242	.363	-.143	.179	.107	.2
**BPNSF1**	-.377	-.455	-.403	-.390	.956	.679	.510	.591	-.177	-.436	-.427	-.587	.772	.665	.537	.4
**BPNSF7**	-.164	-.566	-.474	-.501	.536	.747	.794	.831	-.273	-.259	-.357	-.417	.473	.824	.669	.5
**BPNSF13**	-.490	-.716	-.690	-.547	.605	.978	1.061	.882	-.441	-.505	-.566	-.654	.719	1.303	1.020	.8
**BPNSF19**	-.526	-.691	-.549	-.701	.637	.858	.886	1.123	-.318	-.510	-.502	-.458	.639	.829	.851	.7

Condition number = 42.540

Eigenvalues
21.971 6.463 3.648 2.594 2.336 2.139 1.950 1.854 1.837 1.500 1.357 1.312 1.219 1.157 1.091 1.037 .971 .873 .850 .753 .719 .620 .547 .516

Determinant of sample covariance matrix = 13000.023

Sample Correlations (g3)

	BPNSF6	BPNSF11	BPNSF17	BPNSF23	BPNSF3	BPNSF9	BPNSF14	BPNSF21	BPNSF2	BPNSF8	BPNSF20	BPNSF22	BPNSF4	BPNSF12	BPNSF16	BPNS
**BPNSF6**	1.000															
**BPNSF11**	.556	1.000														
**BPNSF17**	.502	.578	1.000													
**BPNSF23**	.509	.598	.611	1.000												
**BPNSF3**	-.341	-.354	-.390	-.420	1.000											
**BPNSF9**	-.422	-.513	-.523	-.458	.521	1.000										
**BPNSF14**	-.296	-.396	-.440	-.418	.437	.621	1.000									
**BPNSF21**	-.272	-.393	-.274	-.402	.376	.462	.485	1.000								
**BPNSF2**	.376	.483	.441	.528	-.356	-.287	-.277	-.198	1.000							
**BPNSF8**	.465	.578	.516	.520	-.303	-.398	-.255	-.269	.549	1.000						
**BPNSF20**	.417	.512	.596	.635	-.358	-.408	-.313	-.298	.515	.566	1.000					
**BPNSF22**	.336	.450	.399	.518	-.296	-.277	-.218	-.294	.435	.549	.522	1.000				
**BPNSF4**	-.243	-.262	-.214	-.257	.539	.356	.290	.351	-.174	-.257	-.257	-.288	1.000			
**BPNSF12**	-.246	-.333	-.345	-.382	.384	.546	.561	.501	-.285	-.284	-.326	-.364	.462	1.000		
**BPNSF16**	-.218	-.337	-.351	-.322	.308	.441	.505	.489	-.253	-.270	-.361	-.339	.434	.662	1.000	
**BPNSF24**	-.271	-.369	-.267	-.363	.290	.391	.406	.521	-.284	-.332	-.333	-.277	.380	.539	.492	1.0
**BPNSF5**	.364	.432	.342	.365	-.159	-.216	-.166	-.147	.481	.449	.351	.386	-.210	-.234	-.245	-.2
**BPNSF10**	.447	.597	.487	.472	-.278	-.373	-.240	-.230	.460	.547	.434	.429	-.243	-.242	-.240	-.2
**BPNSF15**	.242	.310	.326	.222	-.139	-.173	-.144	-.096	.267	.364	.261	.281	-.202	-.114	-.145	-.1
**BPNSF18**	.054	.120	.156	.041	-.046	.072	.117	.042	.079	.094	.093	.157	-.074	.084	.053	.1
**BPNSF1**	-.138	-.170	-.143	-.142	.414	.293	.218	.272	-.063	-.156	-.153	-.238	.374	.293	.246	.2
**BPNSF7**	-.066	-.231	-.184	-.200	.254	.353	.372	.419	-.106	-.101	-.140	-.184	.251	.396	.335	.2
**BPNSF13**	-.198	-.293	-.269	-.220	.288	.465	.500	.447	-.172	-.199	-.223	-.291	.383	.630	.514	.4
**BPNSF19**	-.217	-.289	-.219	-.288	.310	.416	.427	.582	-.126	-.205	-.202	-.208	.348	.410	.438	.3

Condition number = 36.904

Eigenvalues
8.603 2.743 1.478 1.069 .959 .827 .800 .791 .711 .617 .582 .565 .499 .474 .430 .400 .390 .338 .330 .317 .303 .293 .249 .233

Sample Means (g3)

	BPNSF6	BPNSF11	BPNSF17	BPNSF23	BPNSF3	BPNSF9	BPNSF14	BPNSF21	BPNSF2	BPNSF8	BPNSF20	BPNSF22	BPNSF4	BPNSF12	BPNSF16	BPNSF24	BPN
	2.632	2.806	2.828	2.503	5.491	5.461	5.183	4.999	2.644	2.916	2.762	3.274	5.204	5.107	5.019	5.117	3.

Models

Unconstrained (Unconstrained)

Notes for Model (Unconstrained)

Computation of degrees of freedom (Unconstrained)

**Number of distinct sample moments:**	972
**Number of distinct parameters to be estimated:**	261
**Degrees of freedom (972 - 261):**	711

Result (Unconstrained)

Minimum was achieved
Chi-square = 2242.555
Degrees of freedom = 711
Probability level = .000

g1 (g1 - Unconstrained)

Estimates (g1 - Unconstrained)

Scalar Estimates (g1 - Unconstrained)

Maximum Likelihood Estimates

Regression Weights: (g1 - Unconstrained)

			Estimate	S.E.	C.R.	P	Label
BPNSF19	<---	F1	1.000				
BPNSF13	<---	F1	1.107	.081	13.581	***	a1_1
BPNSF7	<---	F1	.815	.085	9.586	***	a2_1
BPNSF1	<---	F1	.614	.081	7.544	***	a3_1
BPNSF18	<---	F2	1.000				
BPNSF15	<---	F2	4.661	1.875	2.486	.013	a4_1
BPNSF10	<---	F2	5.678	2.271	2.500	.012	a5_1
BPNSF5	<---	F2	4.386	1.767	2.481	.013	a6_1
BPNSF24	<---	F3	1.000				
BPNSF16	<---	F3	1.096	.081	13.562	***	a7_1
BPNSF12	<---	F3	1.201	.084	14.328	***	a8_1
BPNSF4	<---	F3	.777	.074	10.470	***	a9_1
BPNSF22	<---	F4	1.000				
BPNSF20	<---	F4	1.189	.089	13.390	***	a10_1
BPNSF8	<---	F4	1.278	.096	13.287	***	a11_1
BPNSF2	<---	F4	1.243	.095	13.100	***	a12_1
BPNSF21	<---	F5	1.000				
BPNSF14	<---	F5	1.122	.075	15.036	***	a13_1
BPNSF9	<---	F5	.960	.072	13.286	***	a14_1
BPNSF3	<---	F5	.867	.071	12.194	***	a15_1
BPNSF23	<---	F6	1.000				
BPNSF17	<---	F6	.918	.062	14.894	***	a16_1
BPNSF11	<---	F6	1.044	.062	16.854	***	a17_1
BPNSF6	<---	F6	.970	.062	15.546	***	a18_1

Standardized Regression Weights: (g1 - Unconstrained)

			Estimate
BPNSF19	<---	F1	.667
BPNSF13	<---	F1	.768
BPNSF7	<---	F1	.513
BPNSF1	<---	F1	.397
BPNSF18	<---	F2	.139
BPNSF15	<---	F2	.606
BPNSF10	<---	F2	.706
BPNSF5	<---	F2	.582
BPNSF24	<---	F3	.701
BPNSF16	<---	F3	.743
BPNSF12	<---	F3	.800
BPNSF4	<---	F3	.558
BPNSF22	<---	F4	.662
BPNSF20	<---	F4	.748
BPNSF8	<---	F4	.741
BPNSF2	<---	F4	.728
BPNSF21	<---	F5	.693
BPNSF14	<---	F5	.781
BPNSF9	<---	F5	.684
BPNSF3	<---	F5	.625
BPNSF23	<---	F6	.785
BPNSF17	<---	F6	.690
BPNSF11	<---	F6	.766
BPNSF6	<---	F6	.716

Intercepts: (g1 - Unconstrained)

	Estimate	S.E.	C.R.	P	Label
**BPNSF19**	5.314	.069	77.042	***	i1_1
**BPNSF13**	5.196	.066	78.372	***	i2_1
**BPNSF7**	4.945	.073	67.677	***	i3_1

	Estimate	S.E.	C.R.	P	Label
**BPNSF1**	4.595	.071	64.504	***	i4_1
**BPNSF18**	4.383	.070	62.825	***	i5_1
**BPNSF15**	3.793	.074	50.934	***	i6_1
**BPNSF10**	3.294	.078	42.278	***	i7_1
**BPNSF5**	3.688	.073	50.579	***	i8_1
**BPNSF24**	5.244	.059	88.508	***	i9_1
**BPNSF16**	5.112	.061	83.560	***	i10_1
**BPNSF12**	5.230	.062	83.979	***	i11_1
**BPNSF4**	5.068	.058	87.716	***	i12_1
**BPNSF22**	3.015	.069	43.833	***	i13_1
**BPNSF20**	2.351	.072	32.463	***	i14_1
**BPNSF8**	2.768	.079	35.206	***	i15_1
**BPNSF2**	2.230	.078	28.665	***	i16_1
**BPNSF21**	5.359	.068	78.555	***	i17_1
**BPNSF14**	5.585	.068	82.257	***	i18_1
**BPNSF9**	5.848	.066	88.115	***	i19_1
**BPNSF3**	5.716	.066	87.036	***	i20_1
**BPNSF23**	2.068	.073	28.288	***	i21_1
**BPNSF17**	2.440	.076	31.935	***	i22_1
**BPNSF11**	2.690	.078	34.372	***	i23_1
**BPNSF6**	2.458	.078	31.577	***	i24_1

Covariances: (g1 - Unconstrained)

	Estimate	S.E.	C.R.	P	Label
F1 <--> F2	-.047	.023	-2.046	.041	ccc1_1
F2 <--> F3	-.059	.026	-2.258	.024	ccc2_1
F1 <--> F3	.624	.074	8.464	***	ccc3_1
F2 <--> F4	.114	.047	2.414	.016	ccc4_1
F3 <--> F4	-.454	.062	-7.265	***	ccc5_1
F1 <--> F4	-.406	.067	-6.080	***	ccc6_1
F2 <--> F5	-.036	.019	-1.855	.064	ccc7_1
F4 <--> F5	-.592	.075	-7.874	***	ccc8_1
F3 <--> F5	.635	.073	8.700	***	ccc9_1
F1 <--> F5	.916	.096	9.512	***	ccc10_1
F6 <--> F5	-.833	.093	-8.944	***	ccc11_1
F6 <--> F3	-.498	.071	-6.971	***	ccc12_1
F6 <--> F4	1.060	.107	9.924	***	ccc13_1
F6 <--> F2	.157	.064	2.442	.015	ccc14_1
F6 <--> F1	-.580	.083	-6.954	***	ccc15_1

Correlations: (g1 - Unconstrained)

	Estimate
F1 <--> F2	-.240
F2 <--> F3	-.335
F1 <--> F3	.746
F2 <--> F4	.590
F3 <--> F4	-.548
F1 <--> F4	-.442
F2 <--> F5	-.179
F4 <--> F5	-.627
F3 <--> F5	.739
F1 <--> F5	.962
F6 <--> F5	-.701
F6 <--> F3	-.477
F6 <--> F4	.925
F6 <--> F2	.645
F6 <--> F1	-.501

Variances: (g1 - Unconstrained)

	Estimate	S.E.	C.R.	P	Label
**F1**	.927	.126	7.371	***	vvv1_1
**F2**	.041	.033	1.263	.207	vvv2_1
**F3**	.755	.097	7.816	***	vvv3_1
**F4**	.909	.123	7.419	***	vvv4_1
**F5**	.979	.123	7.978	***	vvv5_1
**F6**	1.444	.153	9.465	***	vvv6_1
**e1**	1.157	.091	12.778	***	v1_1

	Estimate	S.E.	C.R.	P	Label
**e2**	.790	.074	10.720	***	v2_1
**e3**	1.724	.123	13.964	***	v3_1
**e4**	1.872	.130	14.373	***	v4_1
**e5**	2.090	.142	14.701	***	v5_1
**e6**	1.536	.130	11.785	***	v6_1
**e7**	1.334	.139	9.569	***	v7_1
**e8**	1.539	.127	12.148	***	v8_1
**e9**	.783	.064	12.184	***	v9_1
**e10**	.733	.064	11.421	***	v10_1
**e11**	.610	.062	9.917	***	v11_1
**e12**	1.007	.074	13.594	***	v12_1
**e13**	1.163	.088	13.206	***	v13_1
**e14**	1.012	.083	12.154	***	v14_1
**e15**	1.221	.100	12.272	***	v15_1
**e16**	1.246	.100	12.464	***	v16_1
**e17**	1.059	.080	13.301	***	v17_1
**e18**	.787	.065	12.033	***	v18_1
**e19**	1.027	.077	13.386	***	v19_1
**e20**	1.152	.083	13.801	***	v20_1
**e21**	.898	.075	12.001	***	v21_1
**e22**	1.338	.101	13.269	***	v22_1
**e23**	1.109	.090	12.353	***	v23_1
**e24**	1.294	.099	13.021	***	v24_1

Matrices (g1 - Unconstrained)

Residual Covariances (g1 - Unconstrained)

	BPNSF6	BPNSF11	BPNSF17	BPNSF23	BPNSF3	BPNSF9	BPNSF14	BPNSF21	BPNSF2	BPNSF8	BPNSF20	BPNSF22	BPNSF4	BPNSF12	BPNSF16	BPNS[illegible]
**BPNSF6**	.000															
**BPNSF11**	.002	.000														
**BPNSF17**	.121	.014	.000													
**BPNSF23**	-.027	-.047	.000	.000												
**BPNSF3**	-.157	-.021	-.082	-.258	.000											
**BPNSF9**	-.132	-.013	-.122	-.199	.122	.000										
**BPNSF14**	.104	.258	.002	.013	-.023	.038	.000									
**BPNSF21**	.122	.141	.181	-.053	-.108	-.030	-.006	.000								
**BPNSF2**	-.015	.003	-.125	.122	-.301	-.176	-.075	-.083	.000							
**BPNSF8**	.131	.083	.026	-.166	.066	-.100	.217	.335	-.036	.000						
**BPNSF20**	-.037	-.129	.125	.189	-.203	-.133	-.048	-.032	-.016	-.077	.000					
**BPNSF22**	-.119	-.075	-.177	.018	-.076	.028	.246	.167	-.003	.146	.022	.000				
**BPNSF4**	-.128	.035	-.006	.033	.199	-.028	.140	-.069	-.120	.072	-.049	-.008	.000			
**BPNSF12**	-.031	.231	-.009	.092	-.059	-.022	.037	-.096	.055	.160	.041	-.043	-.030	.000		
**BPNSF16**	-.162	.014	-.112	-.114	-.045	.051	.036	.005	.014	.051	-.027	.008	-.001	.009	.000	
**BPNSF24**	-.027	.076	-.071	-.059	.061	.141	-.119	-.092	-.155	.035	-.092	-.137	-.006	.003	.009	[illegible]
**BPNSF5**	.117	.214	.014	-.039	-.048	-.143	-.037	-.125	.165	.136	.019	.111	.158	-.107	-.183	[illegible]
**BPNSF10**	.021	.463	-.024	-.130	-.033	-.184	.054	-.070	.105	.266	-.121	-.094	.010	-.044	-.015	[illegible]
**BPNSF15**	-.109	.015	-.115	-.364	-.065	.001	.354	-.029	-.285	-.014	-.170	-.081	.084	-.032	.194	[illegible]
**BPNSF18**	-.309	-.102	-.035	-.222	.217	.289	.096	.425	-.091	.000	-.227	.017	.072	.179	.257	[illegible]
**BPNSF1**	.066	.103	.241	.111	.120	-.147	-.272	.024	.146	.183	.077	.067	.313	.091	.030	[illegible]
**BPNSF7**	.190	.062	.304	.005	-.051	.083	-.186	.133	-.144	.332	.164	.145	-.086	-.224	-.221	[illegible]
**BPNSF13**	-.018	.095	.009	-.028	-.154	-.123	.158	.036	-.090	.191	-.113	.071	.047	.109	.014	[illegible]
**BPNSF19**	-.003	-.074	-.152	-.268	-.055	-.109	-.005	.335	-.307	.093	-.258	-.023	-.034	-.015	.073	[illegible]

Residual Means (g1 - Unconstrained)

	BPNSF6	BPNSF11	BPNSF17	BPNSF23	BPNSF3	BPNSF9	BPNSF14	BPNSF21	BPNSF2	BPNSF8	BPNSF20	BPNSF22	BPNSF4	BPNSF12	BPNSF16	BPNSF24	BP[illegible]
	.000	.000	.000	.000	.000	.000	.000	.000	.000	.000	.000	.000	.000	.000	.000	.000	[illegible]

Standardized Residual Covariances (g1 - Unconstrained)

	BPNSF6	BPNSF11	BPNSF17	BPNSF23	BPNSF3	BPNSF9	BPNSF14	BPNSF21	BPNSF2	BPNSF8	BPNSF20	BPNSF22	BPNSF4	BPNSF12	BPNSF16	BPNS[illegible]
**BPNSF6**	.000															
**BPNSF11**	.013	.000														
**BPNSF17**	.870	.097	.000													
**BPNSF23**	-.199	-.337	-.003	.000												
**BPNSF3**	-1.403	-.184	-.752	-2.429	.000											
**BPNSF9**	-1.152	-.115	-1.090	-1.837	1.226	.000										
**BPNSF14**	.874	2.137	.014	.117	-.224	.354	.000									
**BPNSF21**	1.035	1.182	1.570	-.477	-1.056	-.287	-.052	.000								

	BPNSF6	BPNSF11	BPNSF17	BPNSF23	BPNSF3	BPNSF9	BPNSF14	BPNSF21	BPNSF2	BPNSF8	BPNSF20	BPNSF22	BPNSF4	BPNSF12	BPNSF16	BPNS
**BPNSF2**	-.107	.020	-.908	.908	-2.709	-1.557	-.635	-.716	.000							
**BPNSF8**	.919	.569	.188	-1.213	.583	-.871	1.827	2.837	-.249	.000						
**BPNSF20**	-.283	-.958	.972	1.496	-1.962	-1.262	-.437	-.296	-.118	-.566	.000					
**BPNSF22**	-.970	-.603	-1.481	.151	-.780	.279	2.390	1.631	-.024	1.159	.189	.000				
**BPNSF4**	-1.334	.364	-.063	.368	2.427	-.337	1.625	-.804	-1.242	.742	-.548	-.094	.000			
**BPNSF12**	-.296	2.169	-.089	.925	-.642	-.239	.381	-.997	.517	1.485	.411	-.466	-.358	.000		
**BPNSF16**	-1.578	.133	-1.112	-1.174	-.504	.562	.378	.049	.132	.484	-.277	.092	-.008	.093	.000	
**BPNSF24**	-.276	.756	-.726	-.629	.717	1.612	-1.314	-1.018	-1.546	.348	-.981	-1.558	-.079	.038	.108	.0
**BPNSF5**	.952	1.721	.116	-.331	-.478	-1.412	-.359	-1.197	1.352	1.097	.162	1.031	1.786	-1.117	-1.940	-1.8
**BPNSF10**	.156	3.426	-.181	-1.029	-.311	-1.695	.486	-.629	.790	1.982	-.980	-.810	.104	-.422	-.143	.9
**BPNSF15**	-.869	.114	-.935	-3.058	-.635	.009	3.336	-.276	-2.274	-.113	-1.452	-.739	.928	-.329	2.016	.5
**BPNSF18**	-2.717	-.893	-.313	-2.073	2.267	2.978	.972	4.265	-.798	.001	-2.139	.166	.854	1.964	2.870	1.4
**BPNSF1**	.561	.872	2.095	1.003	1.191	-1.435	-2.577	.227	1.247	1.552	.712	.647	3.590	.954	.320	1.1
**BPNSF7**	1.567	.506	2.561	.040	-.490	.775	-1.670	1.207	-1.192	2.727	1.457	1.364	-.951	-2.253	-2.268	-1.3
**BPNSF13**	-.161	.842	.078	-.260	-1.539	-1.195	1.448	.340	-.813	1.701	-1.094	.724	.558	1.151	.153	-.7
**BPNSF19**	-.028	-.631	-1.340	-2.460	-.537	-1.039	-.041	3.112	-2.670	.802	-2.406	-.223	-.396	-.150	.774	-.3

Standardized Residual Means (g1 - Unconstrained)

	BPNSF6	BPNSF11	BPNSF17	BPNSF23	BPNSF3	BPNSF9	BPNSF14	BPNSF21	BPNSF2	BPNSF8	BPNSF20	BPNSF22	BPNSF4	BPNSF12	BPNSF16	BPNSF24	BP
	.000	.000	.000	.000	.000	.000	.000	.000	.000	.000	.000	.000	.000	.000	.000	.000	.

Notes for Group/Model (g1 - Unconstrained)

The following covariance matrix is not positive definite (g1 - Unconstrained)

	F5	F4	F3	F2	F1	F6
**F5**	.979					
**F4**	-.592	.909				
**F3**	.635	-.454	.755			
**F2**	-.036	.114	-.059	.041		
**F1**	.916	-.406	.624	-.047	.927	
**F6**	-.833	1.060	-.498	.157	-.580	1.444

This solution is not admissible.

Modification Indices (g1 - Unconstrained)

Covariances: (g1 - Unconstrained)

			M.I.	Par Change
e24	<-->	F3	5.458	-.096
e23	<-->	F3	5.375	.090
e23	<-->	F2	23.666	.053
e22	<-->	e24	4.467	.146
e21	<-->	F4	4.317	.065
e21	<-->	F2	11.879	-.034
e19	<-->	F1	10.997	-.118
e19	<-->	e20	6.261	.139
e17	<-->	F3	6.010	-.090
e17	<-->	F1	19.799	.161
e17	<-->	e22	5.761	.149
e17	<-->	e20	4.797	-.124
e16	<-->	F5	4.462	-.074
e16	<-->	e22	7.468	-.189
e15	<-->	F2	6.000	.028
e15	<-->	F1	5.434	.096
e15	<-->	e24	5.276	.157
e15	<-->	e23	5.154	.146
e15	<-->	e21	17.286	-.244
e15	<-->	e19	6.692	-.156
e15	<-->	e17	4.805	.134
e14	<-->	F2	4.593	-.023
e14	<-->	e23	8.245	-.169
e14	<-->	e22	4.788	.138
e14	<-->	e21	16.666	.219
e13	<-->	F5	7.906	.094
e13	<-->	F4	5.027	.076
e13	<-->	F3	4.814	-.085
e13	<-->	e22	5.418	-.152
e13	<-->	e18	8.257	.149

			M.I.	Par Change
e13	<-->	e15	8.317	.185
e12	<-->	e20	10.990	.182
e12	<-->	e18	5.266	.109
e11	<-->	F2	6.200	-.022
e11	<-->	F6	4.776	.072
e11	<-->	e23	6.508	.126
e11	<-->	e21	4.158	.092
e11	<-->	e18	5.009	.093
e11	<-->	e13	4.281	-.102
e10	<-->	F4	5.381	.069
e10	<-->	F6	11.088	-.114
e10	<-->	e16	4.598	.117
e9	<-->	F4	5.488	-.070
e9	<-->	e19	11.892	.166
e9	<-->	e18	11.797	-.150
e8	<-->	e12	11.338	.222
e7	<-->	e23	25.993	.368
e7	<-->	e15	5.702	.182
e7	<-->	e14	5.089	-.157
e7	<-->	e13	7.030	-.191
e7	<-->	e9	6.151	.151
e7	<-->	e8	5.584	-.195
e6	<-->	e21	7.269	-.178
e6	<-->	e18	13.952	.228
e6	<-->	e16	5.658	-.183
e6	<-->	e11	5.487	-.137
e6	<-->	e10	11.573	.207
e5	<-->	F1	5.800	.120
e5	<-->	e24	5.788	-.200
e5	<-->	e18	12.080	-.230
e5	<-->	e17	7.167	.199
e5	<-->	e6	5.565	.219
e4	<-->	F3	5.315	.109
e4	<-->	F2	27.525	-.071
e4	<-->	e22	5.185	.183
e4	<-->	e20	10.878	.241
e4	<-->	e18	11.025	-.210
e4	<-->	e16	4.134	.161
e4	<-->	e12	14.948	.266
e4	<-->	e7	4.482	-.187
e4	<-->	e6	13.557	-.327
e3	<-->	F3	14.479	-.175
e3	<-->	F1	8.885	.133
e3	<-->	e22	9.290	.237
e3	<-->	e19	4.069	.136
e3	<-->	e18	10.838	-.201
e3	<-->	e17	4.301	.143
e3	<-->	e16	8.296	-.222
e3	<-->	e11	4.868	-.129
e3	<-->	e10	4.309	-.127
e3	<-->	e8	4.103	-.173
e3	<-->	e4	5.817	.215
e2	<-->	F2	6.565	.024
e2	<-->	e20	9.117	-.156
e2	<-->	e19	9.329	-.150
e2	<-->	e18	17.147	.183
e2	<-->	e11	10.410	.139
e1	<-->	e24	5.016	.146
e1	<-->	e19	5.752	-.137
e1	<-->	e17	31.877	.327
e1	<-->	e5	5.232	.180

Variances: (g1 - Unconstrained)

	M.I.	Par Change

Regression Weights: (g1 - Unconstrained)

			M.I.	Par Change
BPNSF11	<---	F5	5.310	.132
BPNSF11	<---	F3	4.147	.138

	M.I.	Par Change
BPNSF11 <--- F2	11.875	1.063
BPNSF11 <--- BPNSF10	5.421	.034
BPNSF23 <--- F5	4.270	-.108
BPNSF23 <--- F2	9.665	-.872
BPNSF3 <--- F4	4.705	-.128
BPNSF9 <--- F4	4.238	-.116
BPNSF9 <--- F6	4.080	-.089
BPNSF14 <--- F2	6.634	.668
BPNSF2 <--- F5	4.468	-.128
BPNSF2 <--- F1	6.793	-.166
BPNSF8 <--- F5	8.995	.181
BPNSF8 <--- F3	4.230	.147
BPNSF8 <--- F2	4.064	.656
BPNSF8 <--- F1	9.589	.197
BPNSF20 <--- F5	4.030	-.111
BPNSF10 <--- BPNSF11	4.035	.041
BPNSF15 <--- F4	4.825	-.157
BPNSF15 <--- F6	4.333	-.117
BPNSF18 <--- F5	14.257	.277
BPNSF18 <--- F3	7.696	.240
BPNSF18 <--- F1	13.534	.284
BPNSF1 <--- F2	9.530	-1.165
BPNSF7 <--- F3	5.487	-.188
BPNSF19 <--- F4	4.972	-.135
BPNSF19 <--- F6	5.070	-.107

Means: (g1 - Unconstrained)

	M.I.	Par Change

Intercepts: (g1 - Unconstrained)

	M.I.	Par Change

Bootstrap (g1 - Unconstrained)

Bootstrap standard errors (g1 - Unconstrained)

Scalar Estimates (g1 - Unconstrained)

Regression Weights: (g1 - Unconstrained)

Parameter	SE	SE-SE	Mean	Bias	SE-Bias
BPNSF19 <--- F1	.000	.000	1.000	.000	.000
BPNSF13 <--- F1	.102	.005	1.119	.012	.007
BPNSF7 <--- F1	.096	.005	.816	.002	.007
BPNSF1 <--- F1	.100	.005	.615	.001	.007
BPNSF18 <--- F2	.000	.000	1.000	.000	.000
BPNSF15 <--- F2	3.701	.185	5.268	.607	.262
BPNSF10 <--- F2	4.442	.222	6.403	.725	.314
BPNSF5 <--- F2	3.536	.177	4.913	.527	.250
BPNSF24 <--- F3	.000	.000	1.000	.000	.000
BPNSF16 <--- F3	.108	.005	1.100	.004	.008
BPNSF12 <--- F3	.092	.005	1.203	.002	.007
BPNSF4 <--- F3	.093	.005	.777	.000	.007
BPNSF22 <--- F4	.000	.000	1.000	.000	.000
BPNSF20 <--- F4	.099	.005	1.192	.003	.007
BPNSF8 <--- F4	.091	.005	1.282	.004	.006
BPNSF2 <--- F4	.095	.005	1.249	.006	.007
BPNSF21 <--- F5	.000	.000	1.000	.000	.000
BPNSF14 <--- F5	.085	.004	1.130	.009	.006
BPNSF9 <--- F5	.090	.004	.967	.007	.006
BPNSF3 <--- F5	.093	.005	.872	.005	.007
BPNSF23 <--- F6	.000	.000	1.000	.000	.000
BPNSF17 <--- F6	.057	.003	.931	.013	.004
BPNSF11 <--- F6	.067	.003	1.045	.001	.005
BPNSF6 <--- F6	.063	.003	.973	.003	.004

Standardized Regression Weights: (g1 - Unconstrained)

Parameter	SE	SE-SE	Mean	Bias	SE-Bias
BPNSF19 <--- F1	.045	.002	.668	.001	.003
BPNSF13 <--- F1	.037	.002	.769	.001	.003
BPNSF7 <--- F1	.054	.003	.510	-.002	.004
BPNSF1 <--- F1	.050	.003	.395	-.002	.004
BPNSF18 <--- F2	.064	.003	.158	.019	.005
BPNSF15 <--- F2	.053	.003	.611	.004	.004
BPNSF10 <--- F2	.045	.002	.705	.000	.003
BPNSF5 <--- F2	.047	.002	.577	-.005	.003
BPNSF24 <--- F3	.039	.002	.702	.001	.003
BPNSF16 <--- F3	.043	.002	.747	.004	.003
BPNSF12 <--- F3	.028	.001	.801	.000	.002
BPNSF4 <--- F3	.046	.002	.555	-.003	.003
BPNSF22 <--- F4	.035	.002	.660	-.003	.003
BPNSF20 <--- F4	.034	.002	.745	-.003	.002
BPNSF8 <--- F4	.037	.002	.738	-.003	.003
BPNSF2 <--- F4	.033	.002	.724	-.004	.002
BPNSF21 <--- F5	.035	.002	.692	-.001	.003
BPNSF14 <--- F5	.043	.002	.786	.004	.003
BPNSF9 <--- F5	.042	.002	.686	.002	.003
BPNSF3 <--- F5	.046	.002	.627	.003	.003
BPNSF23 <--- F6	.031	.002	.783	-.002	.002
BPNSF17 <--- F6	.037	.002	.694	.004	.003
BPNSF11 <--- F6	.029	.001	.762	-.004	.002
BPNSF6 <--- F6	.035	.002	.713	-.003	.002

Intercepts: (g1 - Unconstrained)

Parameter	SE	SE-SE	Mean	Bias	SE-Bias
**BPNSF19**	.070	.004	5.324	.010	.005
**BPNSF13**	.069	.003	5.201	.005	.005
**BPNSF7**	.077	.004	4.948	.002	.005
**BPNSF1**	.071	.004	4.594	-.001	.005
**BPNSF18**	.068	.003	4.379	-.004	.005
**BPNSF15**	.081	.004	3.790	-.002	.006
**BPNSF10**	.078	.004	3.299	.005	.005
**BPNSF5**	.083	.004	3.683	-.005	.006
**BPNSF24**	.064	.003	5.249	.005	.005
**BPNSF16**	.064	.003	5.117	.005	.005
**BPNSF12**	.067	.003	5.237	.007	.005
**BPNSF4**	.062	.003	5.074	.006	.004
**BPNSF22**	.072	.004	3.016	.000	.005
**BPNSF20**	.077	.004	2.345	-.006	.005
**BPNSF8**	.085	.004	2.766	-.001	.006
**BPNSF2**	.076	.004	2.229	-.001	.005
**BPNSF21**	.076	.004	5.363	.004	.005
**BPNSF14**	.071	.004	5.596	.011	.005
**BPNSF9**	.069	.003	5.852	.005	.005
**BPNSF3**	.069	.003	5.724	.009	.005
**BPNSF23**	.073	.004	2.059	-.009	.005
**BPNSF17**	.078	.004	2.437	-.003	.005
**BPNSF11**	.075	.004	2.689	-.002	.005
**BPNSF6**	.085	.004	2.455	-.003	.006

Covariances: (g1 - Unconstrained)

Parameter	SE	SE-SE	Mean	Bias	SE-Bias
F1 <--> F2	.024	.001	-.049	-.003	.002
F2 <--> F3	.026	.001	-.063	-.004	.002
F1 <--> F3	.083	.004	.618	-.006	.006
F2 <--> F4	.053	.003	.127	.013	.004
F3 <--> F4	.062	.003	-.448	.006	.004
F1 <--> F4	.068	.003	-.393	.013	.005
F2 <--> F5	.020	.001	-.038	-.002	.001
F4 <--> F5	.064	.003	-.577	.014	.005
F3 <--> F5	.076	.004	.633	-.002	.005
F1 <--> F5	.132	.007	.907	-.010	.009
F6 <--> F5	.090	.005	-.817	.016	.006
F6 <--> F3	.066	.003	-.497	.001	.005
F6 <--> F4	.112	.006	1.048	-.012	.008
F6 <--> F2	.073	.004	.175	.018	.005
F6 <--> F1	.086	.004	-.567	.012	.006

Correlations: (g1 - Unconstrained)

Parameter	SE	SE-SE	Mean	Bias	SE-Bias
F1 <--> F2	.076	.004	-.232	.007	.005
F2 <--> F3	.068	.003	-.324	.011	.005
F1 <--> F3	.054	.003	.742	-.004	.004
F2 <--> F4	.062	.003	.584	-.006	.004
F3 <--> F4	.050	.002	-.544	.004	.004
F1 <--> F4	.057	.003	-.433	.009	.004
F2 <--> F5	.063	.003	-.175	.004	.004
F4 <--> F5	.048	.002	-.620	.007	.003
F3 <--> F5	.040	.002	.740	.001	.003
F1 <--> F5	.038	.002	.958	-.004	.003
F6 <--> F5	.044	.002	-.696	.005	.003
F6 <--> F3	.049	.002	-.479	-.003	.003
F6 <--> F4	.033	.002	.925	.000	.002
F6 <--> F2	.053	.003	.639	-.005	.004
F6 <--> F1	.049	.002	-.496	.005	.003

Variances: (g1 - Unconstrained)

Parameter	SE	SE-SE	Mean	Bias	SE-Bias
**F1**	.146	.007	.923	-.004	.010
**F2**	.046	.002	.062	.021	.003
**F3**	.104	.005	.759	.004	.007
**F4**	.118	.006	.902	-.007	.008
**F5**	.137	.007	.973	-.006	.010
**F6**	.163	.008	1.429	-.015	.012
**e1**	.140	.007	1.134	-.023	.010
**e2**	.111	.006	.780	-.010	.008
**e3**	.182	.009	1.719	-.004	.013
**e4**	.136	.007	1.850	-.023	.010
**e5**	.134	.007	2.063	-.027	.009
**e6**	.174	.009	1.510	-.026	.012
**e7**	.163	.008	1.328	-.006	.012
**e8**	.152	.008	1.535	-.004	.011
**e9**	.096	.005	.776	-.007	.007
**e10**	.108	.005	.713	-.020	.008
**e11**	.081	.004	.605	-.005	.006
**e12**	.092	.005	1.010	.003	.006
**e13**	.105	.005	1.162	-.001	.007
**e14**	.125	.006	1.015	.003	.009
**e15**	.158	.008	1.221	.000	.011
**e16**	.145	.007	1.261	.015	.010
**e17**	.115	.006	1.051	-.008	.008
**e18**	.156	.008	.762	-.025	.011
**e19**	.157	.008	1.011	-.016	.011
**e20**	.167	.008	1.127	-.025	.012
**e21**	.128	.006	.894	-.004	.009
**e22**	.171	.009	1.323	-.015	.012
**e23**	.116	.006	1.114	.005	.008
**e24**	.160	.008	1.297	.004	.011

Matrices (g1 - Unconstrained)

Sample Covariances - Standard Errors (g1 - Unconstrained)

	BPNSF6	BPNSF11	BPNSF17	BPNSF23	BPNSF3	BPNSF9	BPNSF14	BPNSF21	BPNSF2	BPNSF8	BPNSF20	BPNSF22	BPNSF4	BPNSF12	BPNSF16	BPNS[illegible]
**BPNSF6**	.193															
**BPNSF11**	.149	.159														
**BPNSF17**	.156	.150	.186													
**BPNSF23**	.151	.153	.138	.180												
**BPNSF3**	.129	.111	.108	.107	.175											
**BPNSF9**	.118	.105	.108	.109	.115	.181										
**BPNSF14**	.128	.114	.118	.117	.102	.123	.157									
**BPNSF21**	.103	.105	.119	.113	.102	.114	.133	.160								
**BPNSF2**	.154	.154	.137	.149	.098	.105	.120	.115	.161							
**BPNSF8**	.151	.152	.155	.153	.114	.116	.111	.114	.152	.170						
**BPNSF20**	.159	.147	.159	.150	.099	.109	.131	.114	.128	.154	.167					
**BPNSF22**	.123	.130	.123	.127	.094	.091	.102	.103	.111	.126	.116	.117				
**BPNSF4**	.103	.106	.095	.085	.104	.086	.075	.093	.113	.106	.092	.091	.101			

	BPNSF6	BPNSF11	BPNSF17	BPNSF23	BPNSF3	BPNSF9	BPNSF14	BPNSF21	BPNSF2	BPNSF8	BPNSF20	BPNSF22	BPNSF4	BPNSF12	BPNSF16	BPNS[illegible]
**BPNSF12**	.117	.103	.106	.088	.091	.109	.129	.119	.106	.116	.098	.093	.086	.133		
**BPNSF16**	.123	.104	.110	.109	.095	.107	.124	.115	.105	.111	.103	.081	.081	.103	.121	
**BPNSF24**	.102	.092	.106	.096	.104	.109	.102	.107	.100	.099	.090	.091	.081	.096	.096	[illegible]
**BPNSF5**	.124	.123	.139	.115	.093	.091	.101	.108	.113	.139	.120	.112	.089	.099	.098	[illegible]
**BPNSF10**	.139	.144	.144	.135	.122	.112	.123	.122	.140	.151	.133	.129	.115	.113	.109	[illegible]
**BPNSF15**	.123	.129	.131	.114	.122	.112	.110	.124	.137	.148	.115	.123	.098	.113	.111	[illegible]
**BPNSF18**	.115	.112	.120	.102	.106	.114	.117	.115	.105	.122	.100	.106	.089	.114	.106	[illegible]
**BPNSF1**	.122	.109	.114	.094	.112	.097	.100	.109	.106	.117	.098	.100	.092	.092	.090	[illegible]
**BPNSF7**	.119	.113	.112	.092	.111	.109	.109	.121	.109	.116	.098	.101	.090	.107	.102	[illegible]
**BPNSF13**	.116	.116	.110	.110	.095	.115	.127	.130	.113	.116	.107	.096	.088	.130	.114	[illegible]
**BPNSF19**	.107	.112	.116	.113	.106	.108	.132	.140	.120	.126	.116	.102	.098	.107	.115	[illegible]

Sample Correlations - Standard Errors (g1 - Unconstrained)

	BPNSF6	BPNSF11	BPNSF17	BPNSF23	BPNSF3	BPNSF9	BPNSF14	BPNSF21	BPNSF2	BPNSF8	BPNSF20	BPNSF22	BPNSF4	BPNSF12	BPNSF16	BPNS[illegible]
**BPNSF6**	.000															
**BPNSF11**	.044	.000														
**BPNSF17**	.046	.047	.000													
**BPNSF23**	.044	.040	.047	.000												
**BPNSF3**	.053	.049	.049	.043	.000											
**BPNSF9**	.051	.044	.045	.047	.053	.000										
**BPNSF14**	.053	.048	.047	.049	.053	.047	.000									
**BPNSF21**	.043	.043	.052	.045	.050	.051	.050	.000								
**BPNSF2**	.051	.044	.048	.045	.044	.046	.048	.044	.000							
**BPNSF8**	.044	.044	.048	.051	.052	.049	.047	.050	.044	.000						
**BPNSF20**	.052	.046	.050	.041	.044	.047	.053	.050	.044	.048	.000					
**BPNSF22**	.045	.046	.048	.044	.045	.043	.051	.051	.040	.040	.044	.000				
**BPNSF4**	.048	.052	.048	.044	.056	.049	.036	.049	.053	.050	.045	.050	.000			
**BPNSF12**	.050	.049	.049	.042	.047	.049	.055	.051	.047	.053	.046	.047	.047	.000		
**BPNSF16**	.053	.047	.048	.048	.051	.049	.057	.049	.048	.050	.048	.045	.044	.043	.000	
**BPNSF24**	.047	.046	.051	.049	.051	.050	.055	.055	.048	.051	.048	.051	.047	.039	.052	[illegible]
**BPNSF5**	.044	.044	.054	.047	.045	.043	.047	.049	.042	.053	.050	.050	.048	.049	.047	[illegible]
**BPNSF10**	.051	.042	.051	.048	.056	.050	.053	.051	.047	.050	.050	.052	.058	.052	.050	[illegible]
**BPNSF15**	.047	.047	.051	.046	.056	.052	.050	.055	.054	.055	.047	.053	.052	.054	.056	[illegible]
**BPNSF18**	.048	.047	.051	.046	.053	.057	.057	.054	.045	.050	.046	.051	.050	.060	.057	[illegible]
**BPNSF1**	.050	.046	.048	.042	.045	.047	.048	.046	.043	.048	.044	.046	.046	.045	.046	[illegible]
**BPNSF7**	.049	.046	.047	.041	.053	.046	.053	.049	.045	.047	.043	.047	.049	.052	.052	[illegible]
**BPNSF13**	.050	.050	.049	.047	.048	.051	.037	.043	.047	.051	.047	.048	.047	.052	.050	[illegible]
**BPNSF19**	.045	.046	.048	.042	.051	.050	.052	.044	.048	.055	.047	.049	.053	.048	.052	[illegible]

Sample Means - Standard Errors (g1 - Unconstrained)

	BPNSF6	BPNSF11	BPNSF17	BPNSF23	BPNSF3	BPNSF9	BPNSF14	BPNSF21	BPNSF2	BPNSF8	BPNSF20	BPNSF22	BPNSF4	BPNSF12	BPNSF16	BPNSF[illegible]
**BPNSF6**	.085	.075	.078	.073	.069	.069	.071	.076	.076	.085	.077	.072	.062	.067	.064	[illegible]

Bootstrap Confidence (g1 - Unconstrained)

Percentile method (g1 - Unconstrained)

90% confidence intervals (percentile method)

Scalar Estimates (g1 - Unconstrained)

Regression Weights: (g1 - Unconstrained)

Parameter			Estimate	Lower	Upper	P
BPNSF19	<---	F1	1.000	1.000	1.000	...
BPNSF13	<---	F1	1.107	.976	1.310	.010
BPNSF7	<---	F1	.815	.671	.982	.010
BPNSF1	<---	F1	.614	.452	.772	.010
BPNSF18	<---	F2	1.000	1.000	1.000	...
BPNSF15	<---	F2	4.661	2.424	13.836	.010
BPNSF10	<---	F2	5.678	2.834	16.036	.010
BPNSF5	<---	F2	4.386	2.138	12.169	.010
BPNSF24	<---	F3	1.000	1.000	1.000	...
BPNSF16	<---	F3	1.096	.932	1.283	.010
BPNSF12	<---	F3	1.201	1.049	1.365	.010
BPNSF4	<---	F3	.777	.621	.935	.010
BPNSF22	<---	F4	1.000	1.000	1.000	...

Parameter			Estimate	Lower	Upper	P
BPNSF20	<---	F4	1.189	1.025	1.383	.010
BPNSF8	<---	F4	1.278	1.135	1.450	.010
BPNSF2	<---	F4	1.243	1.113	1.413	.010
BPNSF21	<---	F5	1.000	1.000	1.000	...
BPNSF14	<---	F5	1.122	1.005	1.280	.010
BPNSF9	<---	F5	.960	.819	1.133	.010
BPNSF3	<---	F5	.867	.717	1.015	.010
BPNSF23	<---	F6	1.000	1.000	1.000	...
BPNSF17	<---	F6	.918	.840	1.018	.010
BPNSF11	<---	F6	1.044	.937	1.165	.010
BPNSF6	<---	F6	.970	.873	1.074	.010

Standardized Regression Weights: (g1 - Unconstrained)

Parameter			Estimate	Lower	Upper	P
BPNSF19	<---	F1	.667	.586	.741	.010
BPNSF13	<---	F1	.768	.707	.826	.010
BPNSF7	<---	F1	.513	.417	.603	.010
BPNSF1	<---	F1	.397	.308	.476	.010
BPNSF18	<---	F2	.139	.051	.274	.010
BPNSF15	<---	F2	.606	.527	.696	.010
BPNSF10	<---	F2	.706	.634	.780	.010
BPNSF5	<---	F2	.582	.501	.651	.010
BPNSF24	<---	F3	.701	.633	.766	.010
BPNSF16	<---	F3	.743	.673	.815	.010
BPNSF12	<---	F3	.800	.746	.846	.010
BPNSF4	<---	F3	.558	.469	.630	.010
BPNSF22	<---	F4	.662	.598	.716	.010
BPNSF20	<---	F4	.748	.684	.802	.010
BPNSF8	<---	F4	.741	.674	.794	.010
BPNSF2	<---	F4	.728	.673	.778	.010
BPNSF21	<---	F5	.693	.629	.747	.010
BPNSF14	<---	F5	.781	.711	.853	.010
BPNSF9	<---	F5	.684	.609	.756	.010
BPNSF3	<---	F5	.625	.558	.704	.010
BPNSF23	<---	F6	.785	.727	.832	.010
BPNSF17	<---	F6	.690	.633	.758	.010
BPNSF11	<---	F6	.766	.707	.809	.010
BPNSF6	<---	F6	.716	.653	.771	.010

Intercepts: (g1 - Unconstrained)

Parameter	Estimate	Lower	Upper	P
**BPNSF19**	5.314	5.205	5.451	.010
**BPNSF13**	5.196	5.066	5.310	.010
**BPNSF7**	4.945	4.815	5.064	.010
**BPNSF1**	4.595	4.485	4.713	.010
**BPNSF18**	4.383	4.269	4.490	.010
**BPNSF15**	3.793	3.658	3.936	.010
**BPNSF10**	3.294	3.164	3.426	.010
**BPNSF5**	3.688	3.563	3.829	.010
**BPNSF24**	5.244	5.137	5.367	.010
**BPNSF16**	5.112	5.014	5.221	.010
**BPNSF12**	5.230	5.121	5.346	.010
**BPNSF4**	5.068	4.970	5.171	.010
**BPNSF22**	3.015	2.897	3.143	.010
**BPNSF20**	2.351	2.220	2.471	.010
**BPNSF8**	2.768	2.626	2.909	.010
**BPNSF2**	2.230	2.105	2.367	.010
**BPNSF21**	5.359	5.236	5.484	.010
**BPNSF14**	5.585	5.474	5.713	.010
**BPNSF9**	5.848	5.729	5.952	.010
**BPNSF3**	5.716	5.617	5.853	.010
**BPNSF23**	2.068	1.945	2.182	.010
**BPNSF17**	2.440	2.306	2.585	.010
**BPNSF11**	2.690	2.572	2.818	.010
**BPNSF6**	2.458	2.319	2.615	.010

Covariances: (g1 - Unconstrained)

Parameter	Estimate	Lower	Upper	P

Parameter	Estimate	Lower	Upper	P
F1 <--> F2	-.047	-.100	-.016	.010
F2 <--> F3	-.059	-.109	-.020	.010
F1 <--> F3	.624	.490	.760	.010
F2 <--> F4	.114	.040	.221	.010
F3 <--> F4	-.454	-.557	-.338	.010
F1 <--> F4	-.406	-.516	-.299	.010
F2 <--> F5	-.036	-.079	-.011	.010
F4 <--> F5	-.592	-.696	-.477	.010
F3 <--> F5	.635	.510	.762	.010
F1 <--> F5	.916	.704	1.133	.010
F6 <--> F5	-.833	-.973	-.660	.010
F6 <--> F3	-.498	-.600	-.382	.010
F6 <--> F4	1.060	.851	1.231	.010
F6 <--> F2	.157	.055	.297	.010
F6 <--> F1	-.580	-.725	-.450	.010

Correlations: (g1 - Unconstrained)

Parameter	Estimate	Lower	Upper	P
F1 <--> F2	-.240	-.360	-.111	.010
F2 <--> F3	-.335	-.446	-.221	.010
F1 <--> F3	.746	.654	.837	.010
F2 <--> F4	.590	.469	.678	.010
F3 <--> F4	-.548	-.624	-.456	.010
F1 <--> F4	-.442	-.534	-.335	.010
F2 <--> F5	-.179	-.283	-.065	.010
F4 <--> F5	-.627	-.708	-.544	.010
F3 <--> F5	.739	.670	.802	.010
F1 <--> F5	.962	.893	1.018	.010
F6 <--> F5	-.701	-.770	-.628	.010
F6 <--> F3	-.477	-.554	-.399	.010
F6 <--> F4	.925	.866	.975	.010
F6 <--> F2	.645	.553	.725	.010
F6 <--> F1	-.501	-.584	-.416	.010

Variances: (g1 - Unconstrained)

Parameter	Estimate	Lower	Upper	P
**F1**	.927	.707	1.173	.010
**F2**	.041	.005	.157	.010
**F3**	.755	.598	.945	.010
**F4**	.909	.709	1.082	.010
**F5**	.979	.733	1.207	.010
**F6**	1.444	1.159	1.706	.010
**e1**	1.157	.902	1.365	.010
**e2**	.790	.609	.986	.010
**e3**	1.724	1.425	2.022	.010
**e4**	1.872	1.641	2.123	.010
**e5**	2.090	1.845	2.280	.010
**e6**	1.536	1.214	1.815	.010
**e7**	1.334	1.041	1.584	.010
**e8**	1.539	1.286	1.784	.010
**e9**	.783	.611	.937	.010
**e10**	.733	.551	.920	.010
**e11**	.610	.467	.754	.010
**e12**	1.007	.863	1.158	.010
**e13**	1.163	1.000	1.337	.010
**e14**	1.012	.808	1.232	.010
**e15**	1.221	.995	1.474	.010
**e16**	1.246	1.011	1.528	.010
**e17**	1.059	.859	1.246	.010
**e18**	.787	.524	1.035	.010
**e19**	1.027	.784	1.299	.010
**e20**	1.152	.866	1.426	.010
**e21**	.898	.673	1.124	.010
**e22**	1.338	1.033	1.607	.010
**e23**	1.109	.938	1.315	.010
**e24**	1.294	1.029	1.580	.010

Matrices (g1 - Unconstrained)

Sample Covariances (g1 - Unconstrained)

Sample Covariances - Lower Bounds (PC) (g1 - Unconstrained)

	BPNSF6	BPNSF11	BPNSF17	BPNSF23	BPNSF3	BPNSF9	BPNSF14	BPNSF21	BPNSF2	BPNSF8	BPNSF20	BPNSF22	BPNSF4	BPNSF12	BPNSF16	BPNS[illegible]
**BPNSF6**	2.323															
**BPNSF11**	1.185	2.407														
**BPNSF17**	1.125	1.184	2.243													
**BPNSF23**	1.083	1.165	1.100	2.025												
**BPNSF3**	-1.076	-.940	-.930	-1.141	1.565											
**BPNSF9**	-1.080	-.991	-1.030	-1.177	.755	1.640										
**BPNSF14**	-1.018	-.933	-1.064	-1.118	.760	.897	1.730									
**BPNSF21**	-.852	-.876	-.765	-1.066	.564	.722	.869	1.773								
**BPNSF2**	1.006	1.134	.869	1.170	-1.103	-1.061	-1.120	-1.016	2.389							
**BPNSF8**	1.185	1.231	1.041	.912	-.772	-.990	-.808	-.579	1.133	2.418						
**BPNSF20**	.879	.932	1.022	1.198	-.951	-.986	-1.045	-.893	1.119	1.047	2.013					
**BPNSF22**	.684	.837	.622	.844	-.744	-.688	-.576	-.594	.941	1.082	.914	1.865				
**BPNSF4**	-.681	-.528	-.518	-.514	.484	.299	.568	.270	-.746	-.549	-.597	-.509	1.313			
**BPNSF12**	-.817	-.553	-.746	-.670	.431	.520	.695	.471	-.793	-.712	-.765	-.736	.523	1.453		
**BPNSF16**	-.869	-.720	-.816	-.847	.413	.545	.601	.519	-.779	-.767	-.793	-.638	.505	.829	1.413	
**BPNSF24**	-.650	-.606	-.722	-.724	.442	.557	.437	.371	-.890	-.704	-.760	-.751	.449	.761	.675	[illegible]
**BPNSF5**	.565	.702	.436	.444	-.332	-.443	-.372	-.455	.607	.513	.413	.420	-.182	-.572	-.612	[illegible]
**BPNSF10**	.634	1.130	.544	.537	-.396	-.544	-.394	-.481	.656	.811	.435	.322	-.434	-.635	-.538	[illegible]
**BPNSF15**	.388	.540	.348	.168	-.417	-.334	-.015	-.399	.141	.421	.272	.248	-.286	-.537	-.304	[illegible]
**BPNSF18**	-.330	-.081	-.059	-.218	.009	.049	-.142	.193	-.099	-.019	-.236	-.038	-.114	-.100	.019	[illegible]
**BPNSF1**	-.478	-.429	-.268	-.398	.420	.224	.191	.394	-.319	-.304	-.362	-.315	.446	.377	.291	[illegible]
**BPNSF7**	-.435	-.592	-.284	-.615	.376	.614	.459	.688	-.734	-.259	-.416	-.343	.140	.184	.157	[illegible]
**BPNSF13**	-.833	-.757	-.756	-.855	.577	.664	1.083	.826	-.832	-.574	-.814	-.522	.424	.715	.560	[illegible]
**BPNSF19**	-.745	-.870	-.871	-1.033	.570	.567	.822	1.006	-.998	-.625	-.931	-.571	.285	.573	.577	[illegible]

Sample Covariances - Upper Bounds (PC) (g1 - Unconstrained)

	BPNSF6	BPNSF11	BPNSF17	BPNSF23	BPNSF3	BPNSF9	BPNSF14	BPNSF21	BPNSF2	BPNSF8	BPNSF20	BPNSF22	BPNSF4	BPNSF12	BPNSF16	BPNS[illegible]
**BPNSF6**	2.941															
**BPNSF11**	1.697	2.941														
**BPNSF17**	1.666	1.686	2.870													
**BPNSF23**	1.606	1.683	1.594	2.617												
**BPNSF3**	-.631	-.574	-.544	-.793	2.150											
**BPNSF9**	-.701	-.653	-.678	-.829	1.128	2.239										
**BPNSF14**	-.591	-.550	-.661	-.733	1.096	1.310	2.241									
**BPNSF21**	-.496	-.527	-.375	-.688	.913	1.093	1.322	2.287								
**BPNSF2**	1.496	1.646	1.320	1.664	-.753	-.693	-.723	-.634	2.911							
**BPNSF8**	1.697	1.734	1.570	1.434	-.396	-.587	-.450	-.213	1.641	2.945						
**BPNSF20**	1.456	1.436	1.562	1.702	-.617	-.604	-.620	-.520	1.523	1.545	2.583					
**BPNSF22**	1.102	1.260	1.016	1.266	-.428	-.366	-.235	-.229	1.329	1.503	1.295	2.260				
**BPNSF4**	-.324	-.195	-.213	-.217	.800	.597	.822	.581	-.372	-.195	-.317	-.217	1.639			
**BPNSF12**	-.398	-.195	-.396	-.343	.746	.897	1.116	.880	-.444	-.331	-.446	-.430	.816	1.923		
**BPNSF16**	-.460	-.374	-.447	-.485	.718	.905	1.007	.876	-.434	-.410	-.452	-.352	.793	1.160	1.813	
**BPNSF24**	-.308	-.280	-.355	-.389	.793	.926	.791	.720	-.555	-.352	-.465	-.439	.710	1.069	.994	[illegible]
**BPNSF5**	.982	1.127	.886	.838	-.011	-.112	-.067	-.108	.969	.984	.814	.784	.109	-.263	-.285	[illegible]
**BPNSF10**	1.110	1.597	1.065	.988	-.013	-.158	.031	-.074	1.145	1.304	.859	.767	-.048	-.255	-.191	[illegible]
**BPNSF15**	.796	.991	.777	.541	.001	.040	.353	.014	.593	.910	.644	.638	.035	-.171	.100	[illegible]
**BPNSF18**	.042	.275	.328	.109	.375	.422	.246	.583	.231	.386	.106	.320	.179	.276	.359	[illegible]
**BPNSF1**	-.083	-.092	.112	-.076	.791	.538	.542	.771	.025	.089	-.056	-.003	.754	.672	.574	[illegible]
**BPNSF7**	-.055	-.221	.084	-.314	.750	.970	.834	1.079	-.392	.136	-.060	-.006	.430	.552	.497	[illegible]
**BPNSF13**	-.439	-.360	-.400	-.492	.898	1.033	1.491	1.272	-.450	-.173	-.437	-.195	.727	1.142	.953	[illegible]
**BPNSF19**	-.376	-.486	-.491	-.654	.926	.931	1.239	1.468	-.604	-.172	-.530	-.241	.613	.916	.947	[illegible]

Sample Covariances - Two Tailed Significance (PC) (g1 - Unconstrained)

	BPNSF6	BPNSF11	BPNSF17	BPNSF23	BPNSF3	BPNSF9	BPNSF14	BPNSF21	BPNSF2	BPNSF8	BPNSF20	BPNSF22	BPNSF4	BPNSF12	BPNSF16	BPNS[illegible]
**BPNSF6**	.010															
**BPNSF11**	.010	.010														
**BPNSF17**	.010	.010	.010													
**BPNSF23**	.010	.010	.010	.010												
**BPNSF3**	.010	.010	.010	.010	.010											
**BPNSF9**	.010	.010	.010	.010	.010	.010										
**BPNSF14**	.010	.010	.010	.010	.010	.010	.010									
**BPNSF21**	.010	.010	.010	.010	.010	.010	.010	.010								
**BPNSF2**	.010	.010	.010	.010	.010	.010	.010	.010	.010							
**BPNSF8**	.010	.010	.010	.010	.010	.010	.010	.010	.010	.010						

	BPNSF6	BPNSF11	BPNSF17	BPNSF23	BPNSF3	BPNSF9	BPNSF14	BPNSF21	BPNSF2	BPNSF8	BPNSF20	BPNSF22	BPNSF4	BPNSF12	BPNSF16	BPNS[illegible]
**BPNSF20**	.010	.010	.010	.010	.010	.010	.010	.010	.010	.010	.010					
**BPNSF22**	.010	.010	.010	.010	.010	.010	.010	.010	.010	.010	.010	.010				
**BPNSF4**	.010	.010	.010	.010	.010	.010	.010	.010	.010	.010	.010	.010	.010			
**BPNSF12**	.010	.010	.010	.010	.010	.010	.010	.010	.010	.010	.010	.010	.010	.010		
**BPNSF16**	.010	.010	.010	.010	.010	.010	.010	.010	.010	.010	.010	.010	.010	.010	.010	
**BPNSF24**	.010	.010	.010	.010	.010	.010	.010	.010	.010	.010	.010	.010	.010	.010	.010	[illegible]
**BPNSF5**	.010	.010	.010	.010	.077	.012	.044	.016	.010	.010	.010	.010	.671	.010	.010	[illegible]
**BPNSF10**	.010	.010	.010	.010	.087	.010	.183	.019	.010	.010	.010	.010	.039	.010	.010	[illegible]
**BPNSF15**	.010	.010	.010	.010	.100	.214	.163	.133	.016	.010	.010	.010	.176	.010	.371	[illegible]
**BPNSF18**	.207	.417	.353	.579	.088	.051	.609	.010	.662	.137	.410	.201	.793	.366	.078	[illegible]
**BPNSF1**	.027	.028	.476	.016	.010	.010	.010	.010	.146	.339	.023	.098	.010	.010	.010	[illegible]
**BPNSF7**	.025	.010	.258	.010	.010	.010	.010	.010	.010	.529	.010	.073	.010	.010	.016	[illegible]
**BPNSF13**	.010	.010	.010	.010	.010	.010	.010	.010	.010	.010	.010	.010	.010	.010	.010	[illegible]
**BPNSF19**	.010	.010	.010	.010	.010	.010	.010	.010	.010	.010	.010	.010	.010	.010	.010	[illegible]

Sample Correlations (g1 - Unconstrained)

Sample Correlations - Lower Bounds (PC) (g1 - Unconstrained)

	BPNSF6	BPNSF11	BPNSF17	BPNSF23	BPNSF3	BPNSF9	BPNSF14	BPNSF21	BPNSF2	BPNSF8	BPNSF20	BPNSF22	BPNSF4	BPNSF12	BPNSF16	BPNS[illegible]
**BPNSF6**	1.000															
**BPNSF11**	.469	1.000														
**BPNSF17**	.460	.469	1.000													
**BPNSF23**	.472	.506	.472	1.000												
**BPNSF3**	-.474	-.424	-.429	-.541	1.000											
**BPNSF9**	-.479	-.435	-.460	-.550	.403	1.000										
**BPNSF14**	-.444	-.394	-.456	-.503	.389	.474	1.000									
**BPNSF21**	-.364	-.375	-.341	-.486	.307	.376	.452	1.000								
**BPNSF2**	.390	.437	.335	.499	-.497	-.457	-.476	-.431	1.000							
**BPNSF8**	.461	.474	.413	.376	-.339	-.424	-.347	-.254	.446	1.000						
**BPNSF20**	.389	.398	.447	.543	-.453	-.456	-.475	-.418	.460	.437	1.000					
**BPNSF22**	.309	.363	.276	.408	-.378	-.339	-.287	-.288	.412	.474	.432	1.000				
**BPNSF4**	-.333	-.268	-.265	-.277	.290	.181	.347	.164	-.368	-.266	-.316	-.289	1.000			
**BPNSF12**	-.370	-.260	-.345	-.323	.251	.315	.393	.264	-.371	-.334	-.388	-.387	.347	1.000		
**BPNSF16**	-.412	-.342	-.383	-.426	.238	.326	.344	.306	-.369	-.363	-.400	-.343	.345	.527	1.000	
**BPNSF24**	-.314	-.301	-.350	-.376	.277	.348	.251	.217	-.434	-.349	-.419	-.422	.310	.493	.448	[illegible]
**BPNSF5**	.236	.294	.177	.192	-.158	-.208	-.174	-.203	.244	.210	.178	.194	-.100	-.296	-.309	[illegible]
**BPNSF10**	.248	.440	.217	.220	-.178	-.239	-.174	-.205	.256	.313	.179	.141	-.226	-.296	-.259	[illegible]
**BPNSF15**	.155	.217	.142	.072	-.193	-.157	-.007	-.174	.055	.167	.120	.109	-.153	-.263	-.147	[illegible]
**BPNSF18**	-.140	-.033	-.026	-.099	.005	.026	-.067	.093	-.042	-.008	-.109	-.018	-.069	-.051	.010	[illegible]
**BPNSF1**	-.193	-.184	-.116	-.173	.218	.111	.094	.195	-.132	-.123	-.162	-.145	.254	.191	.153	[illegible]
**BPNSF7**	-.178	-.240	-.119	-.258	.186	.297	.211	.318	-.296	-.105	-.180	-.156	.075	.099	.078	[illegible]
**BPNSF13**	-.373	-.333	-.344	-.395	.311	.356	.590	.443	-.365	-.252	-.384	-.270	.265	.432	.336	[illegible]
**BPNSF19**	-.309	-.374	-.368	-.449	.296	.302	.420	.536	-.423	-.266	-.413	-.282	.162	.317	.328	[illegible]

Sample Correlations - Upper Bounds (PC) (g1 - Unconstrained)

	BPNSF6	BPNSF11	BPNSF17	BPNSF23	BPNSF3	BPNSF9	BPNSF14	BPNSF21	BPNSF2	BPNSF8	BPNSF20	BPNSF22	BPNSF4	BPNSF12	BPNSF16	BPNS[illegible]
**BPNSF6**	1.000															
**BPNSF11**	.607	1.000														
**BPNSF17**	.611	.617	1.000													
**BPNSF23**	.616	.638	.622	1.000												
**BPNSF3**	-.295	-.262	-.254	-.388	1.000											
**BPNSF9**	-.308	-.297	-.307	-.394	.589	1.000										
**BPNSF14**	-.259	-.235	-.297	-.346	.574	.636	1.000									
**BPNSF21**	-.217	-.227	-.169	-.335	.470	.536	.626	1.000								
**BPNSF2**	.557	.588	.496	.651	-.347	-.307	-.318	-.284	1.000							
**BPNSF8**	.607	.619	.579	.550	-.173	-.264	-.197	-.088	.596	1.000						
**BPNSF20**	.563	.548	.612	.689	-.297	-.302	-.294	-.245	.608	.597	1.000					
**BPNSF22**	.455	.516	.432	.554	-.217	-.192	-.114	-.114	.548	.619	.575	1.000				
**BPNSF4**	-.167	-.100	-.112	-.122	.471	.352	.455	.327	-.192	-.098	-.179	-.125	1.000			
**BPNSF12**	-.198	-.091	-.188	-.183	.413	.476	.578	.442	-.211	-.150	-.227	-.226	.510	1.000		
**BPNSF16**	-.227	-.187	-.222	-.259	.409	.498	.541	.466	-.204	-.201	-.236	-.191	.491	.670	1.000	
**BPNSF24**	-.155	-.138	-.182	-.210	.441	.518	.435	.401	-.272	-.175	-.250	-.248	.463	.629	.610	[illegible]
**BPNSF5**	.393	.439	.364	.351	-.005	-.057	-.031	-.049	.385	.384	.351	.359	.060	-.133	-.147	[illegible]
**BPNSF10**	.411	.582	.403	.380	-.006	-.069	.013	-.031	.412	.477	.339	.314	-.024	-.115	-.093	[illegible]
**BPNSF15**	.305	.380	.309	.223	.000	.018	.158	.007	.230	.354	.266	.285	.019	-.083	.048	[illegible]
**BPNSF18**	.017	.119	.139	.049	.183	.215	.126	.277	.097	.159	.048	.144	.101	.145	.191	[illegible]
**BPNSF1**	-.033	-.041	.046	-.034	.370	.265	.254	.359	.010	.038	-.025	-.001	.413	.354	.303	[illegible]
**BPNSF7**	-.022	-.083	.033	-.132	.360	.449	.387	.479	-.153	.054	-.026	-.003	.238	.278	.250	[illegible]
**BPNSF13**	-.195	-.156	-.180	-.237	.470	.528	.712	.595	-.203	-.079	-.217	-.099	.416	.600	.512	[illegible]

	BPNSF6	BPNSF11	BPNSF17	BPNSF23	BPNSF3	BPNSF9	BPNSF14	BPNSF21	BPNSF2	BPNSF8	BPNSF20	BPNSF22	BPNSF4	BPNSF12	BPNSF16	BPNS[illegible]
**BPNSF19**	-.162	-.213	-.212	-.319	.466	.471	.589	.676	-.259	-.072	-.239	-.118	.346	.476	.496	.4[illegible]

Sample Correlations - Two Tailed Significance (PC) (g1 - Unconstrained)

	BPNSF6	BPNSF11	BPNSF17	BPNSF23	BPNSF3	BPNSF9	BPNSF14	BPNSF21	BPNSF2	BPNSF8	BPNSF20	BPNSF22	BPNSF4	BPNSF12	BPNSF16	BPNS[illegible]
**BPNSF6**	...															
**BPNSF11**	.010	...														
**BPNSF17**	.010	.010	...													
**BPNSF23**	.010	.010	.010	...												
**BPNSF3**	.010	.010	.010	.010	...											
**BPNSF9**	.010	.010	.010	.010	.010	...										
**BPNSF14**	.010	.010	.010	.010	.010	.010	...									
**BPNSF21**	.010	.010	.010	.010	.010	.010	.010	...								
**BPNSF2**	.010	.010	.010	.010	.010	.010	.010	.010	...							
**BPNSF8**	.010	.010	.010	.010	.010	.010	.010	.010	.010	...						
**BPNSF20**	.010	.010	.010	.010	.010	.010	.010	.010	.010	.010	...					
**BPNSF22**	.010	.010	.010	.010	.010	.010	.010	.010	.010	.010	.010	...				
**BPNSF4**	.010	.010	.010	.010	.010	.010	.010	.010	.010	.010	.010	.010	...			
**BPNSF12**	.010	.010	.010	.010	.010	.010	.010	.010	.010	.010	.010	.010	.010	...		
**BPNSF16**	.010	.010	.010	.010	.010	.010	.010	.010	.010	.010	.010	.010	.010	.010	...	
**BPNSF24**	.010	.010	.010	.010	.010	.010	.010	.010	.010	.010	.010	.010	.010	.010	.010	
**BPNSF5**	.010	.010	.010	.010	.077	.012	.044	.016	.010	.010	.010	.010	.671	.010	.010	.0[illegible]
**BPNSF10**	.010	.010	.010	.010	.087	.010	.183	.019	.010	.010	.010	.010	.039	.010	.010	.0[illegible]
**BPNSF15**	.010	.010	.010	.010	.100	.214	.163	.133	.016	.010	.010	.010	.176	.010	.371	.0[illegible]
**BPNSF18**	.207	.417	.353	.579	.088	.051	.609	.010	.662	.137	.410	.201	.793	.366	.078	.5[illegible]
**BPNSF1**	.027	.027	.476	.016	.010	.010	.010	.010	.145	.339	.023	.098	.010	.010	.010	.0[illegible]
**BPNSF7**	.025	.010	.258	.010	.010	.010	.010	.010	.010	.529	.010	.073	.010	.010	.015	.0[illegible]
**BPNSF13**	.010	.010	.010	.010	.010	.010	.010	.010	.010	.010	.010	.010	.010	.010	.010	.0[illegible]
**BPNSF19**	.010	.010	.010	.010	.010	.010	.010	.010	.010	.010	.010	.010	.010	.010	.010	.0[illegible]

Sample Means (g1 - Unconstrained)

Sample Means - Lower Bounds (PC) (g1 - Unconstrained)

	BPNSF6	BPNSF11	BPNSF17	BPNSF23	BPNSF3	BPNSF9	BPNSF14	BPNSF21	BPNSF2	BPNSF8	BPNSF20	BPNSF22	BPNSF4	BPNSF12	BPNSF16	BPNSF[illegible]
**BPNSF6**	2.317	2.572	2.306	1.945	5.617	5.729	5.474	5.236	2.103	2.626	2.215	2.897	4.967	5.121	5.014	5.13[illegible]

Sample Means - Upper Bounds (PC) (g1 - Unconstrained)

	BPNSF6	BPNSF11	BPNSF17	BPNSF23	BPNSF3	BPNSF9	BPNSF14	BPNSF21	BPNSF2	BPNSF8	BPNSF20	BPNSF22	BPNSF4	BPNSF12	BPNSF16	BPNSF[illegible]
**BPNSF6**	2.615	2.818	2.585	2.182	5.853	5.952	5.713	5.486	2.367	2.909	2.471	3.143	5.171	5.352	5.222	5.36[illegible]

Sample Means - Two Tailed Significance (PC) (g1 - Unconstrained)

	BPNSF6	BPNSF11	BPNSF17	BPNSF23	BPNSF3	BPNSF9	BPNSF14	BPNSF21	BPNSF2	BPNSF8	BPNSF20	BPNSF22	BPNSF4	BPNSF12	BPNSF16	BPNSF[illegible]
**BPNSF6**	.010	.010	.010	.010	.010	.010	.010	.010	.010	.010	.010	.010	.010	.010	.010	.01[illegible]

Bias-corrected percentile method (g1 - Unconstrained)

90% confidence intervals (bias-corrected percentile method)

Scalar Estimates (g1 - Unconstrained)

Regression Weights: (g1 - Unconstrained)

Parameter			Estimate	Lower	Upper	P
BPNSF19	<---	F1	1.000	1.000	1.000	...
BPNSF13	<---	F1	1.107	.968	1.307	.012
BPNSF7	<---	F1	.815	.671	.986	.009
BPNSF1	<---	F1	.614	.454	.783	.007
BPNSF18	<---	F2	1.000	1.000	1.000	...
BPNSF15	<---	F2	4.661	2.838	23.827	.001
BPNSF10	<---	F2	5.678	3.263	26.054	.002
BPNSF5	<---	F2	4.386	2.735	23.003	.001
BPNSF24	<---	F3	1.000	1.000	1.000	...
BPNSF16	<---	F3	1.096	.957	1.356	.006
BPNSF12	<---	F3	1.201	1.049	1.365	.010
BPNSF4	<---	F3	.777	.618	.925	.012

Parameter	Estimate	Lower	Upper	P
BPNSF22 <--- F4	1.000	1.000	1.000	...
BPNSF20 <--- F4	1.189	1.044	1.398	.007
BPNSF8 <--- F4	1.278	1.126	1.427	.014
BPNSF2 <--- F4	1.243	1.113	1.413	.009
BPNSF21 <--- F5	1.000	1.000	1.000	...
BPNSF14 <--- F5	1.122	.980	1.260	.016
BPNSF9 <--- F5	.960	.815	1.110	.016
BPNSF3 <--- F5	.867	.709	1.007	.013
BPNSF23 <--- F6	1.000	1.000	1.000	...
BPNSF17 <--- F6	.918	.803	.995	.034
BPNSF11 <--- F6	1.044	.940	1.169	.009
BPNSF6 <--- F6	.970	.873	1.074	.010

Standardized Regression Weights: (g1 - Unconstrained)

Parameter	Estimate	Lower	Upper	P
BPNSF19 <--- F1	.667	.574	.735	.012
BPNSF13 <--- F1	.768	.688	.819	.021
BPNSF7 <--- F1	.513	.409	.595	.014
BPNSF1 <--- F1	.397	.332	.506	.004
BPNSF18 <--- F2	.139	.028	.225	.044
BPNSF15 <--- F2	.606	.523	.690	.015
BPNSF10 <--- F2	.706	.637	.782	.007
BPNSF5 <--- F2	.582	.520	.677	.004
BPNSF24 <--- F3	.701	.626	.754	.016
BPNSF16 <--- F3	.743	.655	.800	.025
BPNSF12 <--- F3	.800	.735	.835	.023
BPNSF4 <--- F3	.558	.474	.636	.007
BPNSF22 <--- F4	.662	.610	.721	.006
BPNSF20 <--- F4	.748	.685	.802	.007
BPNSF8 <--- F4	.741	.674	.794	.009
BPNSF2 <--- F4	.728	.678	.794	.004
BPNSF21 <--- F5	.693	.630	.750	.007
BPNSF14 <--- F5	.781	.696	.836	.030
BPNSF9 <--- F5	.684	.605	.746	.015
BPNSF3 <--- F5	.625	.553	.702	.014
BPNSF23 <--- F6	.785	.727	.831	.011
BPNSF17 <--- F6	.690	.623	.749	.018
BPNSF11 <--- F6	.766	.720	.815	.004
BPNSF6 <--- F6	.716	.656	.771	.008

Intercepts: (g1 - Unconstrained)

Parameter	Estimate	Lower	Upper	P
**BPNSF19**	5.314	5.175	5.424	.019
**BPNSF13**	5.196	5.056	5.300	.016
**BPNSF7**	4.945	4.802	5.057	.018
**BPNSF1**	4.595	4.485	4.713	.010
**BPNSF18**	4.383	4.275	4.496	.007
**BPNSF15**	3.793	3.655	3.935	.011
**BPNSF10**	3.294	3.159	3.408	.015
**BPNSF5**	3.688	3.569	3.829	.006
**BPNSF24**	5.244	5.123	5.354	.016
**BPNSF16**	5.112	5.001	5.207	.016
**BPNSF12**	5.230	5.091	5.323	.025
**BPNSF4**	5.068	4.945	5.150	.032
**BPNSF22**	3.015	2.896	3.142	.011
**BPNSF20**	2.351	2.223	2.476	.007
**BPNSF8**	2.768	2.626	2.909	.010
**BPNSF2**	2.230	2.118	2.383	.005
**BPNSF21**	5.359	5.224	5.482	.014
**BPNSF14**	5.585	5.446	5.698	.019
**BPNSF9**	5.848	5.704	5.943	.021
**BPNSF3**	5.716	5.594	5.811	.023
**BPNSF23**	2.068	1.975	2.242	.003
**BPNSF17**	2.440	2.317	2.602	.006
**BPNSF11**	2.690	2.581	2.821	.005
**BPNSF6**	2.458	2.346	2.640	.004

Covariances: (g1 - Unconstrained)

Parameter	Estimate	Lower	Upper	P
F1 <--> F2	-.047	-.108	-.020	.004
F2 <--> F3	-.059	-.099	-.014	.025
F1 <--> F3	.624	.501	.776	.006
F2 <--> F4	.114	.032	.208	.025
F3 <--> F4	-.454	-.575	-.352	.005
F1 <--> F4	-.406	-.590	-.325	.001
F2 <--> F5	-.036	-.083	-.012	.006
F4 <--> F5	-.592	-.741	-.515	.001
F3 <--> F5	.635	.510	.762	.009
F1 <--> F5	.916	.731	1.187	.005
F6 <--> F5	-.833	-1.038	-.727	.002
F6 <--> F3	-.498	-.602	-.391	.008
F6 <--> F4	1.060	.877	1.262	.005
F6 <--> F2	.157	.038	.267	.034
F6 <--> F1	-.580	-.773	-.480	.002

Correlations: (g1 - Unconstrained)

Parameter	Estimate	Lower	Upper	P
F1 <--> F2	-.240	-.383	-.132	.004
F2 <--> F3	-.335	-.503	-.247	.003
F1 <--> F3	.746	.675	.848	.004
F2 <--> F4	.590	.468	.675	.012
F3 <--> F4	-.548	-.640	-.480	.004
F1 <--> F4	-.442	-.562	-.362	.003
F2 <--> F5	-.179	-.288	-.068	.009
F4 <--> F5	-.627	-.727	-.566	.003
F3 <--> F5	.739	.665	.799	.015
F1 <--> F5	.962	.900	1.035	.004
F6 <--> F5	-.701	-.774	-.636	.006
F6 <--> F3	-.477	-.553	-.397	.011
F6 <--> F4	.925	.866	.975	.010
F6 <--> F2	.645	.553	.725	.010
F6 <--> F1	-.501	-.598	-.437	.004

Variances: (g1 - Unconstrained)

Parameter	Estimate	Lower	Upper	P
**F1**	.927	.725	1.207	.005
**F2**	.041	.002	.113	.039
**F3**	.755	.607	.953	.007
**F4**	.909	.713	1.100	.008
**F5**	.979	.772	1.227	.007
**F6**	1.444	1.159	1.706	.010
**e1**	1.157	.955	1.468	.003
**e2**	.790	.649	1.070	.003
**e3**	1.724	1.392	2.000	.014
**e4**	1.872	1.694	2.172	.003
**e5**	2.090	1.867	2.351	.005
**e6**	1.536	1.317	1.872	.002
**e7**	1.334	1.031	1.571	.014
**e8**	1.539	1.258	1.756	.012
**e9**	.783	.616	.945	.007
**e10**	.733	.599	1.000	.002
**e11**	.610	.509	.784	.004
**e12**	1.007	.863	1.150	.012
**e13**	1.163	1.000	1.339	.009
**e14**	1.012	.812	1.233	.009
**e15**	1.221	1.003	1.483	.009
**e16**	1.246	.949	1.450	.023
**e17**	1.059	.879	1.265	.006
**e18**	.787	.595	1.209	.002
**e19**	1.027	.804	1.369	.003
**e20**	1.152	.890	1.460	.005
**e21**	.898	.723	1.150	.005
**e22**	1.338	1.051	1.618	.006
**e23**	1.109	.939	1.337	.009
**e24**	1.294	1.031	1.585	.009

Matrices (g1 - Unconstrained)

Sample Covariances (g1 - Unconstrained)

Sample Covariances - Lower Bounds (BC) (g1 - Unconstrained)

	BPNSF6	BPNSF11	BPNSF17	BPNSF23	BPNSF3	BPNSF9	BPNSF14	BPNSF21	BPNSF2	BPNSF8	BPNSF20	BPNSF22	BPNSF4	BPNSF12	BPNSF16	BPNS[illegible]
**BPNSF6**	2.396															
**BPNSF11**	1.225	2.459														
**BPNSF17**	1.090	1.185	2.241													
**BPNSF23**	1.084	1.215	1.117	2.033												
**BPNSF3**	-1.170	-.969	-.959	-1.166	1.673											
**BPNSF9**	-1.158	-1.197	-1.091	-1.240	.778	1.692										
**BPNSF14**	-1.043	-.994	-1.074	-1.182	.760	.899	1.783									
**BPNSF21**	-.905	-.893	-.800	-1.078	.565	.734	.899	1.778								
**BPNSF2**	1.008	1.158	.891	1.170	-1.117	-1.076	-1.127	-1.004	2.377							
**BPNSF8**	1.191	1.258	1.074	.928	-.858	-1.048	-.835	-.636	1.154	2.413						
**BPNSF20**	.947	.968	1.061	1.242	-.968	-1.011	-1.100	-.942	1.143	1.065	2.026					
**BPNSF22**	.685	.849	.617	.848	-.840	-.710	-.582	-.621	.975	1.087	.927	1.867				
**BPNSF4**	-.692	-.548	-.552	-.513	.487	.329	.575	.265	-.746	-.587	-.626	-.510	1.318			
**BPNSF12**	-.823	-.572	-.746	-.638	.411	.520	.698	.525	-.776	-.758	-.764	-.745	.540	1.454		
**BPNSF16**	-.876	-.748	-.821	-.854	.398	.570	.616	.521	-.799	-.777	-.821	-.642	.505	.834	1.424	
**BPNSF24**	-.650	-.614	-.722	-.721	.471	.595	.427	.397	-.912	-.723	-.773	-.758	.447	.782	.674	[illegible]
**BPNSF5**	.567	.758	.435	.444	-.325	-.454	-.367	-.458	.615	.521	.413	.448	-.190	-.572	-.669	[illegible]
**BPNSF10**	.652	1.131	.574	.561	-.383	-.552	-.407	-.493	.713	.832	.441	.332	-.455	-.666	-.620	[illegible]
**BPNSF15**	.367	.531	.338	.164	-.427	-.334	.005	-.404	.120	.421	.248	.220	-.285	-.596	-.315	[illegible]
**BPNSF18**	-.365	-.119	-.103	-.221	.002	.092	-.147	.174	-.097	-.069	-.248	-.081	-.095	-.077	.027	[illegible]
**BPNSF1**	-.479	-.460	-.261	-.410	.449	.248	.197	.409	-.347	-.350	-.415	-.338	.473	.392	.320	[illegible]
**BPNSF7**	-.474	-.632	-.319	-.660	.409	.617	.471	.689	-.715	-.306	-.446	-.395	.148	.237	.192	[illegible]
**BPNSF13**	-.871	-.792	-.771	-.894	.579	.652	1.123	.845	-.816	-.612	-.835	-.535	.427	.715	.585	[illegible]
**BPNSF19**	-.755	-.890	-.925	-1.035	.616	.609	.852	1.013	-1.001	-.679	-.977	-.580	.300	.591	.610	[illegible]

Sample Covariances - Upper Bounds (BC) (g1 - Unconstrained)

	BPNSF6	BPNSF11	BPNSF17	BPNSF23	BPNSF3	BPNSF9	BPNSF14	BPNSF21	BPNSF2	BPNSF8	BPNSF20	BPNSF22	BPNSF4	BPNSF12	BPNSF16	BPNS[illegible]
**BPNSF6**	2.992															
**BPNSF11**	1.768	3.020														
**BPNSF17**	1.604	1.688	2.867													
**BPNSF23**	1.606	1.693	1.600	2.630												
**BPNSF3**	-.687	-.602	-.599	-.806	2.236											
**BPNSF9**	-.758	-.742	-.703	-.846	1.155	2.344										
**BPNSF14**	-.617	-.582	-.674	-.774	1.096	1.311	2.341									
**BPNSF21**	-.546	-.548	-.412	-.705	.917	1.108	1.361	2.297								
**BPNSF2**	1.497	1.716	1.406	1.664	-.765	-.736	-.731	-.614	2.907							
**BPNSF8**	1.697	1.781	1.592	1.460	-.433	-.677	-.475	-.256	1.665	2.942						
**BPNSF20**	1.486	1.470	1.577	1.746	-.653	-.657	-.648	-.547	1.590	1.566	2.595					
**BPNSF22**	1.105	1.286	1.007	1.272	-.469	-.425	-.261	-.281	1.363	1.534	1.326	2.270				
**BPNSF4**	-.334	-.223	-.233	-.213	.805	.611	.830	.575	-.374	-.249	-.327	-.224	1.648			
**BPNSF12**	-.409	-.220	-.396	-.320	.724	.897	1.126	.913	-.436	-.359	-.445	-.435	.835	1.923		
**BPNSF16**	-.465	-.394	-.452	-.487	.714	.973	1.019	.881	-.442	-.415	-.458	-.358	.795	1.161	1.820	
**BPNSF24**	-.312	-.297	-.355	-.388	.809	.972	.761	.742	-.567	-.387	-.478	-.456	.709	1.088	.992	[illegible]
**BPNSF5**	.986	1.164	.879	.838	-.007	-.142	-.051	-.110	.997	.986	.814	.826	.106	-.263	-.323	[illegible]
**BPNSF10**	1.134	1.607	1.076	1.008	.001	-.173	.019	-.084	1.185	1.311	.883	.771	-.071	-.265	-.226	[illegible]
**BPNSF15**	.762	.986	.775	.541	-.013	.038	.389	.004	.581	.910	.616	.617	.035	-.202	.058	[illegible]
**BPNSF18**	.006	.236	.304	.097	.362	.433	.244	.555	.239	.322	.091	.285	.185	.304	.363	[illegible]
**BPNSF1**	-.091	-.112	.115	-.089	.843	.561	.545	.818	-.006	.037	-.095	-.037	.766	.707	.600	[illegible]
**BPNSF7**	-.091	-.267	.028	-.346	.774	.974	.850	1.080	-.366	.072	-.108	-.029	.444	.605	.518	[illegible]
**BPNSF13**	-.471	-.427	-.411	-.520	.907	1.010	1.593	1.289	-.446	-.233	-.495	-.224	.741	1.127	.979	[illegible]
**BPNSF19**	-.387	-.495	-.527	-.664	1.007	.972	1.305	1.479	-.611	-.247	-.588	-.249	.620	.945	1.019	[illegible]

Sample Covariances - Two Tailed Significance (BC) (g1 - Unconstrained)

	BPNSF6	BPNSF11	BPNSF17	BPNSF23	BPNSF3	BPNSF9	BPNSF14	BPNSF21	BPNSF2	BPNSF8	BPNSF20	BPNSF22	BPNSF4	BPNSF12	BPNSF16	BPNS[illegible]
**BPNSF6**	.006															
**BPNSF11**	.004	.003														
**BPNSF17**	.020	.009	.011													
**BPNSF23**	.009	.005	.007	.008												
**BPNSF3**	.003	.005	.004	.005	.002											
**BPNSF9**	.002	.001	.005	.003	.005	.003										
**BPNSF14**	.006	.002	.007	.004	.010	.009	.004									
**BPNSF21**	.002	.005	.004	.007	.009	.007	.003	.006								
**BPNSF2**	.009	.004	.004	.010	.008	.005	.007	.014	.012							
**BPNSF8**	.009	.005	.005	.006	.003	.002	.004	.004	.007	.012						

	BPNSF6	BPNSF11	BPNSF17	BPNSF23	BPNSF3	BPNSF9	BPNSF14	BPNSF21	BPNSF2	BPNSF8	BPNSF20	BPNSF22	BPNSF4	BPNSF12	BPNSF16	BPNS[illegible]
**BPNSF20**	.005	.005	.005	.003	.005	.003	.004	.004	.003	.007	.006					
**BPNSF22**	.009	.006	.012	.009	.002	.002	.006	.003	.003	.007	.005	.009				
**BPNSF4**	.007	.006	.004	.012	.009	.005	.007	.012	.009	.002	.005	.007	.007			
**BPNSF12**	.007	.007	.010	.015	.019	.010	.009	.003	.013	.004	.011	.007	.006	.009		
**BPNSF16**	.009	.005	.008	.009	.012	.004	.007	.009	.006	.007	.006	.007	.009	.008	.007	
**BPNSF24**	.009	.005	.010	.011	.005	.005	.014	.006	.005	.004	.007	.006	.011	.005	.011	[illegible]
**BPNSF5**	.009	.003	.012	.010	.086	.007	.059	.014	.006	.009	.010	.004	.617	.010	.003	[illegible]
**BPNSF10**	.008	.009	.007	.007	.102	.005	.145	.010	.004	.007	.005	.008	.029	.007	.003	[illegible]
**BPNSF15**	.019	.012	.011	.011	.090	.205	.095	.103	.020	.010	.016	.020	.184	.004	.262	[illegible]
**BPNSF18**	.112	.741	.531	.480	.098	.037	.663	.019	.626	.353	.317	.329	.626	.269	.070	[illegible]
**BPNSF1**	.019	.016	.524	.010	.003	.005	.008	.006	.088	.166	.007	.045	.006	.005	.004	[illegible]
**BPNSF7**	.008	.003	.159	.002	.005	.009	.006	.009	.016	.235	.003	.023	.007	.003	.006	[illegible]
**BPNSF13**	.004	.003	.009	.006	.009	.018	.004	.007	.014	.002	.003	.006	.006	.012	.005	[illegible]
**BPNSF19**	.007	.007	.003	.008	.002	.005	.004	.007	.009	.003	.002	.008	.007	.006	.003	[illegible]

Sample Correlations (g1 - Unconstrained)

Sample Correlations - Lower Bounds (BC) (g1 - Unconstrained)

	BPNSF6	BPNSF11	BPNSF17	BPNSF23	BPNSF3	BPNSF9	BPNSF14	BPNSF21	BPNSF2	BPNSF8	BPNSF20	BPNSF22	BPNSF4	BPNSF12	BPNSF16	BPNS[illegible]
**BPNSF6**	1.000															
**BPNSF11**	.483	1.000														
**BPNSF17**	.442	.468	1.000													
**BPNSF23**	.476	.509	.478	1.000												
**BPNSF3**	-.495	-.424	-.430	-.543	1.000											
**BPNSF9**	-.495	-.456	-.463	-.552	.403	1.000										
**BPNSF14**	-.439	-.411	-.456	-.522	.380	.463	1.000									
**BPNSF21**	-.366	-.377	-.348	-.477	.311	.377	.441	1.000								
**BPNSF2**	.382	.447	.337	.488	-.498	-.463	-.462	-.431	1.000							
**BPNSF8**	.457	.467	.420	.379	-.341	-.438	-.361	-.266	.464	1.000						
**BPNSF20**	.407	.406	.457	.545	-.460	-.459	-.484	-.418	.460	.449	1.000					
**BPNSF22**	.309	.363	.263	.404	-.403	-.358	-.289	-.292	.431	.491	.438	1.000				
**BPNSF4**	-.332	-.274	-.278	-.265	.288	.185	.342	.164	-.368	-.280	-.330	-.289	1.000			
**BPNSF12**	-.375	-.265	-.338	-.315	.241	.315	.367	.267	-.369	-.351	-.382	-.382	.338	1.000		
**BPNSF16**	-.416	-.346	-.377	-.431	.225	.323	.333	.288	-.368	-.363	-.393	-.344	.345	.525	1.000	
**BPNSF24**	-.316	-.287	-.340	-.372	.259	.358	.242	.221	-.434	-.355	-.421	-.423	.297	.491	.432	[illegible]
**BPNSF5**	.237	.300	.171	.187	-.154	-.208	-.172	-.203	.242	.210	.173	.200	-.103	-.293	-.323	[illegible]
**BPNSF10**	.253	.444	.227	.222	-.178	-.249	-.176	-.207	.260	.320	.185	.140	-.234	-.303	-.293	[illegible]
**BPNSF15**	.151	.209	.139	.072	-.194	-.159	-.001	-.182	.057	.169	.106	.103	-.151	-.270	-.159	[illegible]
**BPNSF18**	-.146	-.050	-.045	-.103	.001	.051	-.068	.085	-.041	-.027	-.115	-.034	-.053	-.050	.017	[illegible]
**BPNSF1**	-.199	-.187	-.112	-.189	.222	.114	.074	.194	-.138	-.137	-.179	-.152	.254	.223	.164	[illegible]
**BPNSF7**	-.203	-.256	-.130	-.282	.206	.297	.206	.314	-.296	-.123	-.191	-.178	.080	.114	.083	[illegible]
**BPNSF13**	-.377	-.343	-.344	-.385	.306	.348	.594	.439	-.366	-.262	-.399	-.273	.266	.430	.342	[illegible]
**BPNSF19**	-.318	-.374	-.391	-.445	.301	.302	.415	.522	-.424	-.289	-.418	-.280	.164	.317	.331	[illegible]

Sample Correlations - Upper Bounds (BC) (g1 - Unconstrained)

	BPNSF6	BPNSF11	BPNSF17	BPNSF23	BPNSF3	BPNSF9	BPNSF14	BPNSF21	BPNSF2	BPNSF8	BPNSF20	BPNSF22	BPNSF4	BPNSF12	BPNSF16	BPNS[illegible]
**BPNSF6**	1.000															
**BPNSF11**	.639	1.000														
**BPNSF17**	.600	.611	1.000													
**BPNSF23**	.631	.640	.628	1.000												
**BPNSF3**	-.313	-.265	-.255	-.398	1.000											
**BPNSF9**	-.329	-.309	-.309	-.395	.589	1.000										
**BPNSF14**	-.256	-.250	-.297	-.359	.558	.629	1.000									
**BPNSF21**	-.223	-.227	-.184	-.335	.476	.549	.620	1.000								
**BPNSF2**	.556	.593	.507	.637	-.348	-.308	-.307	-.284	1.000							
**BPNSF8**	.599	.616	.586	.552	-.176	-.279	-.205	-.108	.609	1.000						
**BPNSF20**	.584	.567	.617	.691	-.321	-.310	-.304	-.246	.608	.602	1.000					
**BPNSF22**	.457	.516	.421	.549	-.237	-.214	-.118	-.122	.559	.630	.578	1.000				
**BPNSF4**	-.167	-.113	-.119	-.110	.471	.352	.450	.327	-.189	-.118	-.192	-.125	1.000			
**BPNSF12**	-.206	-.106	-.185	-.163	.405	.477	.560	.442	-.206	-.173	-.225	-.225	.500	1.000		
**BPNSF16**	-.234	-.190	-.216	-.263	.395	.486	.531	.448	-.203	-.201	-.233	-.194	.484	.666	1.000	
**BPNSF24**	-.157	-.134	-.178	-.207	.429	.524	.423	.408	-.276	-.188	-.251	-.253	.460	.625	.603	[illegible]
**BPNSF5**	.394	.441	.353	.346	-.002	-.057	-.028	-.046	.384	.384	.342	.369	.058	-.126	-.163	[illegible]
**BPNSF10**	.422	.584	.409	.386	-.006	-.075	.009	-.032	.417	.482	.348	.313	-.039	-.126	-.110	[illegible]
**BPNSF15**	.295	.368	.304	.223	-.012	.018	.173	-.009	.232	.354	.259	.269	.026	-.087	.029	[illegible]
**BPNSF18**	.004	.099	.127	.042	.177	.221	.121	.266	.097	.134	.037	.137	.109	.151	.193	[illegible]
**BPNSF1**	-.041	-.043	.048	-.040	.379	.272	.245	.355	-.002	.016	-.038	-.015	.413	.378	.304	[illegible]
**BPNSF7**	-.034	-.106	.011	-.142	.373	.450	.386	.477	-.150	.030	-.035	-.012	.242	.284	.253	[illegible]
**BPNSF13**	-.201	-.182	-.180	-.232	.469	.518	.716	.587	-.204	-.095	-.234	-.112	.417	.599	.514	[illegible]

	BPNSF6	BPNSF11	BPNSF17	BPNSF23	BPNSF3	BPNSF9	BPNSF14	BPNSF21	BPNSF2	BPNSF8	BPNSF20	BPNSF22	BPNSF4	BPNSF12	BPNSF16	BPNS[illegible]
**BPNSF19**	-.167	-.214	-.231	-.314	.471	.471	.574	.673	-.261	-.103	-.260	-.118	.347	.476	.499	.4[illegible]

Sample Correlations - Two Tailed Significance (BC) (g1 - Unconstrained)

	BPNSF6	BPNSF11	BPNSF17	BPNSF23	BPNSF3	BPNSF9	BPNSF14	BPNSF21	BPNSF2	BPNSF8	BPNSF20	BPNSF22	BPNSF4	BPNSF12	BPNSF16	BPNS[illegible]
**BPNSF6**	...															
**BPNSF11**	.003	...														
**BPNSF17**	.023	.013	...													
**BPNSF23**	.005	.008	.007	...												
**BPNSF3**	.004	.007	.009	.007	...											
**BPNSF9**	.003	.003	.007	.008	.010	...										
**BPNSF14**	.012	.003	.010	.006	.023	.021	...									
**BPNSF21**	.006	.009	.005	.011	.007	.008	.014	...								
**BPNSF2**	.012	.005	.006	.023	.009	.009	.019	.010	...							
**BPNSF8**	.014	.015	.006	.009	.007	.005	.004	.004	.003	...						
**BPNSF20**	.003	.005	.003	.008	.005	.005	.006	.009	.010	.005	...					
**BPNSF22**	.009	.011	.016	.015	.003	.002	.009	.006	.003	.006	.006	...				
**BPNSF4**	.011	.006	.005	.019	.011	.007	.021	.010	.011	.003	.004	.010	...			
**BPNSF12**	.007	.007	.018	.020	.020	.009	.016	.009	.014	.004	.012	.012	.013	...		
**BPNSF16**	.009	.006	.016	.009	.019	.014	.018	.019	.011	.010	.014	.008	.013	.015	...	
**BPNSF24**	.009	.013	.014	.012	.016	.006	.019	.007	.007	.006	.009	.009	.012	.012	.019	
**BPNSF5**	.009	.007	.013	.013	.095	.012	.056	.017	.011	.010	.013	.005	.617	.012	.005	.0[illegible]
**BPNSF10**	.007	.007	.005	.008	.082	.006	.152	.015	.008	.007	.005	.011	.027	.007	.002	.0[illegible]
**BPNSF15**	.019	.026	.012	.010	.081	.188	.110	.088	.015	.009	.016	.023	.210	.007	.262	.0[illegible]
**BPNSF18**	.124	.761	.548	.465	.097	.033	.644	.021	.626	.340	.317	.293	.626	.315	.066	.4[illegible]
**BPNSF1**	.017	.021	.557	.010	.006	.008	.019	.011	.088	.166	.008	.051	.010	.003	.006	.0[illegible]
**BPNSF7**	.009	.003	.159	.004	.005	.009	.012	.014	.012	.267	.005	.030	.008	.005	.013	.0[illegible]
**BPNSF13**	.006	.004	.010	.012	.013	.020	.007	.013	.009	.004	.003	.007	.008	.012	.008	.0[illegible]
**BPNSF19**	.006	.008	.004	.014	.006	.011	.013	.016	.009	.003	.005	.012	.009	.010	.008	.0[illegible]

Sample Means (g1 - Unconstrained)

Sample Means - Lower Bounds (BC) (g1 - Unconstrained)

	BPNSF6	BPNSF11	BPNSF17	BPNSF23	BPNSF3	BPNSF9	BPNSF14	BPNSF21	BPNSF2	BPNSF8	BPNSF20	BPNSF22	BPNSF4	BPNSF12	BPNSF16	BPNSF[illegible]
**BPNSF6**	2.345	2.581	2.317	1.967	5.594	5.704	5.445	5.224	2.118	2.626	2.222	2.896	4.946	5.106	5.005	5.12[illegible]

Sample Means - Upper Bounds (BC) (g1 - Unconstrained)

	BPNSF6	BPNSF11	BPNSF17	BPNSF23	BPNSF3	BPNSF9	BPNSF14	BPNSF21	BPNSF2	BPNSF8	BPNSF20	BPNSF22	BPNSF4	BPNSF12	BPNSF16	BPNSF[illegible]
**BPNSF6**	2.642	2.822	2.602	2.233	5.811	5.943	5.698	5.483	2.383	2.909	2.476	3.142	5.152	5.330	5.210	5.34[illegible]

Sample Means - Two Tailed Significance (BC) (g1 - Unconstrained)

	BPNSF6	BPNSF11	BPNSF17	BPNSF23	BPNSF3	BPNSF9	BPNSF14	BPNSF21	BPNSF2	BPNSF8	BPNSF20	BPNSF22	BPNSF4	BPNSF12	BPNSF16	BPNSF[illegible]
**BPNSF6**	.004	.005	.006	.003	.023	.021	.019	.013	.005	.010	.007	.011	.028	.020	.015	.01[illegible]

g2 (g2 - Unconstrained)

Estimates (g2 - Unconstrained)

Scalar Estimates (g2 - Unconstrained)

Maximum Likelihood Estimates

Regression Weights: (g2 - Unconstrained)

			Estimate	S.E.	C.R.	P	Label
BPNSF19	<---	F1	1.000				
BPNSF13	<---	F1	1.170	.097	12.037	***	a1_2
BPNSF7	<---	F1	.910	.093	9.801	***	a2_2
BPNSF1	<---	F1	.870	.091	9.594	***	a3_2
BPNSF18	<---	F2	1.000				
BPNSF15	<---	F2	2.036	.320	6.365	***	a4_2
BPNSF10	<---	F2	2.249	.343	6.566	***	a5_2
BPNSF5	<---	F2	1.752	.278	6.314	***	a6_2
BPNSF24	<---	F3	1.000				
BPNSF16	<---	F3	1.099	.074	14.855	***	a7_2

	Estimate	S.E.	C.R.	P	Label
BPNSF12 <--- F3	1.132	.073	15.580	***	a8_2
BPNSF4 <--- F3	.949	.073	12.951	***	a9_2
BPNSF22 <--- F4	1.000				
BPNSF20 <--- F4	1.107	.072	15.402	***	a10_2
BPNSF8 <--- F4	1.021	.074	13.894	***	a11_2
BPNSF2 <--- F4	.951	.068	13.988	***	a12_2
BPNSF21 <--- F5	1.000				
BPNSF14 <--- F5	1.006	.065	15.501	***	a13_2
BPNSF9 <--- F5	.968	.068	14.265	***	a14_2
BPNSF3 <--- F5	.877	.069	12.674	***	a15_2
BPNSF23 <--- F6	1.000				
BPNSF17 <--- F6	1.006	.060	16.661	***	a16_2
BPNSF11 <--- F6	.871	.062	14.100	***	a17_2
BPNSF6 <--- F6	.799	.063	12.676	***	a18_2

Standardized Regression Weights: (g2 - Unconstrained)

	Estimate
BPNSF19 <--- F1	.581
BPNSF13 <--- F1	.753
BPNSF7 <--- F1	.561
BPNSF1 <--- F1	.546
BPNSF18 <--- F2	.354
BPNSF15 <--- F2	.632
BPNSF10 <--- F2	.718
BPNSF5 <--- F2	.614
BPNSF24 <--- F3	.706
BPNSF16 <--- F3	.777
BPNSF12 <--- F3	.821
BPNSF4 <--- F3	.671
BPNSF22 <--- F4	.723
BPNSF20 <--- F4	.776
BPNSF8 <--- F4	.699
BPNSF2 <--- F4	.704
BPNSF21 <--- F5	.724
BPNSF14 <--- F5	.765
BPNSF9 <--- F5	.705
BPNSF3 <--- F5	.628
BPNSF23 <--- F6	.789
BPNSF17 <--- F6	.771
BPNSF11 <--- F6	.668
BPNSF6 <--- F6	.608

Intercepts: (g2 - Unconstrained)

	Estimate	S.E.	C.R.	P	Label
**BPNSF19**	5.243	.073	71.655	***	i1_2
**BPNSF13**	5.197	.066	78.557	***	i2_2
**BPNSF7**	4.850	.069	70.290	***	i3_2
**BPNSF1**	4.867	.068	71.797	***	i4_2
**BPNSF18**	4.106	.073	56.019	***	i5_2
**BPNSF15**	3.605	.084	43.082	***	i6_2
**BPNSF10**	3.152	.081	38.759	***	i7_2
**BPNSF5**	3.637	.074	49.075	***	i8_2
**BPNSF24**	5.311	.070	75.410	***	i9_2
**BPNSF16**	5.218	.070	74.205	***	i10_2
**BPNSF12**	5.314	.069	77.486	***	i11_2
**BPNSF4**	5.077	.070	72.253	***	i12_2
**BPNSF22**	3.078	.079	38.836	***	i13_2
**BPNSF20**	2.439	.082	29.819	***	i14_2
**BPNSF8**	2.731	.084	32.644	***	i15_2
**BPNSF2**	2.309	.077	29.840	***	i16_2
**BPNSF21**	5.240	.071	73.352	***	i17_2
**BPNSF14**	5.607	.068	82.405	***	i18_2
**BPNSF9**	5.725	.071	80.596	***	i19_2
**BPNSF3**	5.619	.072	77.786	***	i20_2
**BPNSF23**	2.173	.079	27.469	***	i21_2
**BPNSF17**	2.474	.081	30.370	***	i22_2
**BPNSF11**	2.761	.081	33.908	***	i23_2
**BPNSF6**	2.616	.082	31.890	***	i24_2

Covariances: (g2 - Unconstrained)

	Estimate	S.E.	C.R.	P	Label
F1 <--> F2	-.269	.053	-5.036	***	ccc1_2
F2 <--> F3	-.265	.053	-4.952	***	ccc2_2
F1 <--> F3	.805	.093	8.630	***	ccc3_2
F2 <--> F4	.538	.091	5.888	***	ccc4_2
F3 <--> F4	-.845	.098	-8.595	***	ccc5_2
F1 <--> F4	-.649	.088	-7.366	***	ccc6_2
F2 <--> F5	-.264	.055	-4.845	***	ccc7_2
F4 <--> F5	-.787	.097	-8.111	***	ccc8_2
F3 <--> F5	.939	.099	9.518	***	ccc9_2
F1 <--> F5	.954	.104	9.145	***	ccc10_2
F6 <--> F5	-.972	.108	-8.993	***	ccc11_2
F6 <--> F3	-.717	.095	-7.575	***	ccc12_2
F6 <--> F4	1.380	.135	10.221	***	ccc13_2
F6 <--> F2	.526	.090	5.826	***	ccc14_2
F6 <--> F1	-.622	.089	-6.952	***	ccc15_2

Correlations: (g2 - Unconstrained)

	Estimate
F1 <--> F2	-.559
F2 <--> F3	-.470
F1 <--> F3	.873
F2 <--> F4	.829
F3 <--> F4	-.680
F1 <--> F4	-.611
F2 <--> F5	-.452
F4 <--> F5	-.609
F3 <--> F5	.838
F1 <--> F5	.994
F6 <--> F5	-.690
F6 <--> F3	-.530
F6 <--> F4	.885
F6 <--> F2	.744
F6 <--> F1	-.537

Variances: (g2 - Unconstrained)

	Estimate	S.E.	C.R.	P	Label
**F1**	.789	.124	6.347	***	vvv1_2
**F2**	.294	.085	3.438	***	vvv2_2
**F3**	1.077	.133	8.075	***	vvv3_2
**F4**	1.431	.171	8.374	***	vvv4_2
**F5**	1.167	.138	8.452	***	vvv5_2
**F6**	1.700	.181	9.408	***	vvv6_2
**e1**	1.545	.111	13.894	***	v1_2
**e2**	.827	.070	11.797	***	v2_2
**e3**	1.422	.102	13.997	***	v3_2
**e4**	1.407	.100	14.068	***	v4_2
**e5**	2.048	.144	14.259	***	v5_2
**e6**	1.834	.149	12.329	***	v6_2
**e7**	1.397	.130	10.722	***	v7_2
**e8**	1.493	.119	12.558	***	v8_2
**e9**	1.085	.085	12.836	***	v9_2
**e10**	.854	.073	11.764	***	v10_2
**e11**	.670	.063	10.671	***	v11_2
**e12**	1.183	.090	13.177	***	v12_2
**e13**	1.308	.102	12.772	***	v13_2
**e14**	1.162	.097	11.943	***	v14_2
**e15**	1.560	.120	13.035	***	v15_2
**e16**	1.316	.101	12.986	***	v16_2
**e17**	1.058	.081	13.008	***	v17_2
**e18**	.837	.067	12.426	***	v18_2
**e19**	1.106	.084	13.211	***	v19_2
**e20**	1.378	.100	13.784	***	v20_2
**e21**	1.028	.091	11.340	***	v21_2
**e22**	1.172	.100	11.752	***	v22_2
**e23**	1.602	.122	13.161	***	v23_2
**e24**	1.850	.136	13.606	***	v24_2

Matrices (g2 - Unconstrained)

Residual Covariances (g2 - Unconstrained)

	BPNSF6	BPNSF11	BPNSF17	BPNSF23	BPNSF3	BPNSF9	BPNSF14	BPNSF21	BPNSF2	BPNSF8	BPNSF20	BPNSF22	BPNSF4	BPNSF12	BPNSF16	[illegible]
**BPNSF6**	.000															
**BPNSF11**	.003	.000														
**BPNSF17**	-.021	.049	.000													
**BPNSF23**	-.036	-.041	.025	.000												
**BPNSF3**	-.173	.077	-.005	-.035	.000											
**BPNSF9**	-.032	-.110	-.208	-.106	.139	.000										
**BPNSF14**	.105	.067	-.145	.121	-.051	.117	.000									
**BPNSF21**	.077	-.002	.196	.083	.025	-.136	-.056	.000								
**BPNSF2**	.141	-.026	.045	.060	-.109	-.024	-.049	-.005	.000							
**BPNSF8**	.166	.027	.008	-.099	.024	-.014	.098	.052	.091	.000						
**BPNSF20**	-.145	-.038	-.058	.211	-.086	.019	-.011	-.126	.005	-.009	.000					
**BPNSF22**	-.101	-.039	-.094	-.081	.229	.003	.079	-.033	-.054	-.057	.023	.000				
**BPNSF4**	-.102	.001	.017	-.019	.622	.011	-.071	.198	-.094	.042	-.071	.038	.000			
**BPNSF12**	.070	-.002	-.035	.015	-.053	-.053	-.015	.017	.005	.131	.129	-.082	-.013	.000		
**BPNSF16**	.111	-.008	.010	.094	-.148	-.144	-.010	.143	.074	.147	.029	-.033	-.099	.073	.000	
**BPNSF24**	.024	-.024	-.199	-.013	-.195	-.119	.029	.041	-.078	-.043	-.119	-.290	-.088	-.034	.066	[illegible]
**BPNSF5**	.166	-.138	-.191	-.026	-.037	.125	.109	-.055	-.060	.156	-.029	.010	-.287	.036	.110	[illegible]
**BPNSF10**	-.057	.373	.013	.068	-.108	-.198	-.144	-.294	-.032	.101	.033	.069	-.144	-.027	.001	[illegible]
**BPNSF15**	.013	.235	-.076	-.197	.077	.203	.242	.091	-.186	-.019	-.099	.072	-.019	.120	.147	[illegible]
**BPNSF18**	-.202	.183	.052	-.160	.243	.126	.194	.017	-.159	-.046	.067	-.016	.081	.123	.088	[illegible]
**BPNSF1**	.145	.120	.172	.129	.088	-.140	-.070	.065	-.103	.104	-.008	.023	.199	-.084	-.131	[illegible]
**BPNSF7**	.411	-.021	-.115	.226	-.071	.109	-.024	-.056	.065	-.003	.127	-.108	.068	-.077	-.057	[illegible]
**BPNSF13**	.047	-.143	.029	.047	-.073	-.160	.014	.099	.088	-.005	.062	-.080	.024	.139	.004	[illegible]
**BPNSF19**	-.112	-.314	-.272	-.251	-.065	.057	.072	.199	.055	-.031	-.125	-.148	.056	-.088	-.109	[illegible]

Residual Means (g2 - Unconstrained)

	BPNSF6	BPNSF11	BPNSF17	BPNSF23	BPNSF3	BPNSF9	BPNSF14	BPNSF21	BPNSF2	BPNSF8	BPNSF20	BPNSF22	BPNSF4	BPNSF12	BPNSF16	BPNSF24	[illegible]
	.000	.000	.000	.000	.000	.000	.000	.000	.000	.000	.000	.000	.000	.000	.000	.000	[illegible]

Standardized Residual Covariances (g2 - Unconstrained)

	BPNSF6	BPNSF11	BPNSF17	BPNSF23	BPNSF3	BPNSF9	BPNSF14	BPNSF21	BPNSF2	BPNSF8	BPNSF20	BPNSF22	BPNSF4	BPNSF12	BPNSF16	[illegible]
**BPNSF6**	.000															
**BPNSF11**	.020	.000														
**BPNSF17**	-.136	.314	.000													
**BPNSF23**	-.240	-.269	.159	.000												
**BPNSF3**	-1.355	.604	-.039	-.279	.000											
**BPNSF9**	-.252	-.865	-1.611	-.840	1.182	.000										
**BPNSF14**	.855	.546	-1.157	.992	-.450	1.023	.000									
**BPNSF21**	.605	-.015	1.503	.657	.213	-1.144	-.486	.000								
**BPNSF2**	.995	-.181	.308	.424	-.899	-.199	-.422	-.045	.000							
**BPNSF8**	1.084	.178	.051	-.641	.180	-.105	.785	.395	.603	.000						
**BPNSF20**	-.953	-.251	-.369	1.373	-.669	.149	-.087	-.980	.035	-.052	.000					
**BPNSF22**	-.692	-.263	-.627	-.551	1.850	.024	.663	-.263	-.379	-.364	.151	.000				
**BPNSF4**	-.826	.006	.138	-.158	5.537	.098	-.655	1.753	-.791	.329	-.555	.311	.000			
**BPNSF12**	.578	-.014	-.284	.128	-.472	-.467	-.133	.145	.042	1.022	1.012	-.669	-.116	.000		
**BPNSF16**	.894	-.064	.083	.767	-1.296	-1.254	-.093	1.233	.614	1.123	.223	-.264	-.849	.614	.000	
**BPNSF24**	.192	-.192	-1.596	-.105	-1.720	-1.052	.261	.361	-.651	-.329	-.929	-2.353	-.770	-.290	.560	[illegible]
**BPNSF5**	1.256	-1.051	-1.426	-.201	-.324	1.114	1.010	-.485	-.468	1.133	-.215	.078	-2.587	.334	.985	[illegible]
**BPNSF10**	-.386	2.540	.089	.469	-.859	-1.597	-1.210	-2.361	-.226	.658	.218	.474	-1.179	-.221	.005	[illegible]
**BPNSF15**	.087	1.574	-.504	-1.338	.599	1.607	1.992	.710	-1.288	-.119	-.643	.486	-.148	.976	1.169	[illegible]
**BPNSF18**	-1.590	1.447	.411	-1.293	2.191	1.151	1.850	.158	-1.312	-.348	.522	-.130	.747	1.162	.814	[illegible]
**BPNSF1**	1.230	1.022	1.452	1.120	.815	-1.304	-.675	.595	-.918	.853	-.064	.199	1.908	-.810	-1.233	[illegible]
**BPNSF7**	3.424	-.174	-.955	1.927	-.648	.989	-.222	-.504	.563	-.020	1.042	-.920	.638	-.725	-.523	[illegible]
**BPNSF13**	.406	-1.228	.246	.410	-.660	-1.443	.132	.883	.782	-.042	.514	-.694	.223	1.287	.041	[illegible]
**BPNSF19**	-.879	-2.468	-2.123	-2.013	-.553	.483	.632	1.686	.452	-.233	-.961	-1.180	.495	-.775	-.946	[illegible]

Standardized Residual Means (g2 - Unconstrained)

	BPNSF6	BPNSF11	BPNSF17	BPNSF23	BPNSF3	BPNSF9	BPNSF14	BPNSF21	BPNSF2	BPNSF8	BPNSF20	BPNSF22	BPNSF4	BPNSF12	BPNSF16	BPNSF24	[illegible]
	.000	.000	.000	.000	.000	.000	.000	.000	.000	.000	.000	.000	.000	.000	.000	.000	[illegible]

Notes for Group/Model (g2 - Unconstrained)

The following covariance matrix is not positive definite (g2 - Unconstrained)

	F5	F4	F3	F2	F1	F6
**F5**	1.167					
**F4**	-.787	1.431				
**F3**	.939	-.845	1.077			
**F2**	-.264	.538	-.265	.294		
**F1**	.954	-.649	.805	-.269	.789	
**F6**	-.972	1.380	-.717	.526	-.622	1.700

This solution is not admissible.

Modification Indices (g2 - Unconstrained)

Covariances: (g2 - Unconstrained)

			M.I.	Par Change
e24	<-->	F1	4.957	.092
e23	<-->	F2	8.541	.088
e23	<-->	F1	4.899	-.086
e20	<-->	e24	8.208	-.233
e19	<-->	F6	5.058	-.104
e19	<-->	e20	6.326	.160
e18	<-->	e22	9.439	-.170
e18	<-->	e19	7.954	.144
e17	<-->	F5	4.887	-.069
e17	<-->	F6	4.727	.099
e17	<-->	e22	14.861	.237
e17	<-->	e19	8.253	-.163
e15	<-->	e24	5.633	.211
e15	<-->	e21	4.260	-.146
e14	<-->	e24	6.131	-.196
e14	<-->	e21	16.201	.254
e13	<-->	F5	8.876	.115
e13	<-->	F3	4.176	-.085
e13	<-->	F1	5.232	-.082
e13	<-->	e20	16.067	.282
e12	<-->	F5	14.423	.137
e12	<-->	F3	10.553	-.124
e12	<-->	F2	6.523	-.066
e12	<-->	F6	4.316	.101
e12	<-->	e22	5.130	.147
e12	<-->	e20	100.538	.662
e12	<-->	e18	8.730	-.158
e12	<-->	e13	4.161	.136
e11	<-->	e14	7.068	.140
e10	<-->	e20	7.284	-.159
e10	<-->	e17	7.004	.139
e10	<-->	e12	5.506	-.129
e10	<-->	e11	6.088	.109
e9	<-->	F4	4.430	-.086
e9	<-->	e22	4.043	-.127
e9	<-->	e20	11.188	-.214
e9	<-->	e13	8.152	-.184
e8	<-->	e24	8.948	.262
e8	<-->	e23	7.996	-.234
e8	<-->	e22	4.328	-.154
e8	<-->	e12	14.959	-.275
e7	<-->	F5	8.442	-.121
e7	<-->	e23	8.323	.243
e6	<-->	F5	4.698	.099
e6	<-->	e23	6.634	.238
e6	<-->	e21	4.034	-.157
e5	<-->	e23	4.513	.196
e5	<-->	e6	4.451	.212
e4	<-->	F6	9.777	.160
e4	<-->	e22	4.488	.146
e4	<-->	e20	4.329	.145
e4	<-->	e16	5.294	-.161
e4	<-->	e12	7.121	.176
e4	<-->	e7	9.490	.236
e4	<-->	e6	13.342	-.306
e4	<-->	e5	5.179	-.191

			M.I.	Par Change
e3	<-->	e24	18.908	.357
e3	<-->	e22	9.162	-.209
e3	<-->	e21	5.901	.159
e3	<-->	e19	6.708	.165
e3	<-->	e13	5.469	-.165
e2	<-->	F3	5.677	.078
e2	<-->	e23	5.567	-.145
e2	<-->	e19	10.272	-.161
e2	<-->	e12	5.495	-.123
e2	<-->	e11	13.858	.160
e1	<-->	F6	6.539	-.138
e1	<-->	e17	5.053	.147
e1	<-->	e16	4.658	.159

Variances: (g2 - Unconstrained)

	M.I.	Par Change

Regression Weights: (g2 - Unconstrained)

			M.I.	Par Change
BPNSF4	<---	BPNSF3	4.646	.020
BPNSF10	<---	F5	6.394	-.155
BPNSF10	<---	F1	5.143	-.169
BPNSF7	<---	BPNSF6	4.702	.040
BPNSF19	<---	F6	7.750	-.138

Means: (g2 - Unconstrained)

	M.I.	Par Change

Intercepts: (g2 - Unconstrained)

	M.I.	Par Change

Bootstrap (g2 - Unconstrained)

Bootstrap standard errors (g2 - Unconstrained)

Scalar Estimates (g2 - Unconstrained)

Regression Weights: (g2 - Unconstrained)

Parameter			SE	SE-SE	Mean	Bias	SE-Bias
BPNSF19	<---	F1	.000	.000	1.000	.000	.000
BPNSF13	<---	F1	.100	.005	1.171	.001	.007
BPNSF7	<---	F1	.098	.005	.906	-.004	.007
BPNSF1	<---	F1	.132	.007	.878	.008	.009
BPNSF18	<---	F2	.000	.000	1.000	.000	.000
BPNSF15	<---	F2	.313	.016	2.080	.044	.022
BPNSF10	<---	F2	.364	.018	2.296	.048	.026
BPNSF5	<---	F2	.303	.015	1.807	.054	.021
BPNSF24	<---	F3	.000	.000	1.000	.000	.000
BPNSF16	<---	F3	.069	.003	1.113	.013	.005
BPNSF12	<---	F3	.074	.004	1.143	.010	.005
BPNSF4	<---	F3	.094	.005	.959	.010	.007
BPNSF22	<---	F4	.000	.000	1.000	.000	.000
BPNSF20	<---	F4	.070	.004	1.104	-.003	.005
BPNSF8	<---	F4	.073	.004	1.017	-.005	.005
BPNSF2	<---	F4	.081	.004	.954	.003	.006
BPNSF21	<---	F5	.000	.000	1.000	.000	.000
BPNSF14	<---	F5	.079	.004	1.001	-.005	.006
BPNSF9	<---	F5	.079	.004	.967	-.002	.006
BPNSF3	<---	F5	.088	.004	.882	.004	.006
BPNSF23	<---	F6	.000	.000	1.000	.000	.000
BPNSF17	<---	F6	.059	.003	1.011	.004	.004
BPNSF11	<---	F6	.067	.003	.874	.004	.005
BPNSF6	<---	F6	.065	.003	.802	.004	.005

Standardized Regression Weights: (g2 - Unconstrained)

Parameter			SE	SE-SE	Mean	Bias	SE-Bias
BPNSF19	<---	F1	.050	.002	.584	.002	.004
BPNSF13	<---	F1	.030	.001	.753	.000	.002
BPNSF7	<---	F1	.050	.003	.559	-.002	.004
BPNSF1	<---	F1	.047	.002	.548	.003	.003
BPNSF18	<---	F2	.047	.002	.352	-.002	.003
BPNSF15	<---	F2	.042	.002	.631	.000	.003
BPNSF10	<---	F2	.043	.002	.718	.000	.003
BPNSF5	<---	F2	.052	.003	.616	.002	.004
BPNSF24	<---	F3	.038	.002	.705	-.001	.003
BPNSF16	<---	F3	.031	.002	.779	.002	.002
BPNSF12	<---	F3	.024	.001	.823	.002	.002
BPNSF4	<---	F3	.045	.002	.671	.000	.003
BPNSF22	<---	F4	.030	.001	.722	-.001	.002
BPNSF20	<---	F4	.034	.002	.776	.001	.002
BPNSF8	<---	F4	.039	.002	.696	-.004	.003
BPNSF2	<---	F4	.041	.002	.708	.004	.003
BPNSF21	<---	F5	.032	.002	.724	.000	.002
BPNSF14	<---	F5	.043	.002	.764	-.001	.003
BPNSF9	<---	F5	.041	.002	.706	.001	.003
BPNSF3	<---	F5	.052	.003	.631	.003	.004
BPNSF23	<---	F6	.033	.002	.788	-.002	.002
BPNSF17	<---	F6	.034	.002	.773	.001	.002
BPNSF11	<---	F6	.040	.002	.666	-.001	.003
BPNSF6	<---	F6	.043	.002	.607	-.001	.003

Intercepts: (g2 - Unconstrained)

Parameter	SE	SE-SE	Mean	Bias	SE-Bias
**BPNSF19**	.075	.004	5.248	.005	.005
**BPNSF13**	.070	.003	5.210	.013	.005
**BPNSF7**	.072	.004	4.864	.014	.005
**BPNSF1**	.071	.004	4.877	.010	.005
**BPNSF18**	.072	.004	4.102	-.004	.005
**BPNSF15**	.093	.005	3.605	.000	.007
**BPNSF10**	.092	.005	3.139	-.013	.007
**BPNSF5**	.077	.004	3.642	.005	.005
**BPNSF24**	.078	.004	5.320	.009	.005
**BPNSF16**	.071	.004	5.224	.006	.005
**BPNSF12**	.067	.003	5.319	.004	.005
**BPNSF4**	.073	.004	5.081	.004	.005
**BPNSF22**	.089	.004	3.067	-.012	.006
**BPNSF20**	.088	.004	2.426	-.013	.006
**BPNSF8**	.093	.005	2.726	-.006	.007
**BPNSF2**	.080	.004	2.301	-.008	.006
**BPNSF21**	.074	.004	5.252	.012	.005
**BPNSF14**	.068	.003	5.618	.012	.005
**BPNSF9**	.074	.004	5.733	.008	.005
**BPNSF3**	.074	.004	5.625	.006	.005
**BPNSF23**	.076	.004	2.167	-.005	.005
**BPNSF17**	.088	.004	2.464	-.010	.006
**BPNSF11**	.090	.005	2.752	-.009	.006
**BPNSF6**	.078	.004	2.613	-.003	.005

Covariances: (g2 - Unconstrained)

Parameter			SE	SE-SE	Mean	Bias	SE-Bias
F1	<-->	F2	.055	.003	-.272	-.003	.004
F2	<-->	F3	.062	.003	-.267	-.003	.004
F1	<-->	F3	.099	.005	.799	-.005	.007
F2	<-->	F4	.091	.005	.535	-.003	.006
F3	<-->	F4	.114	.006	-.847	-.003	.008
F1	<-->	F4	.096	.005	-.654	-.005	.007
F2	<-->	F5	.058	.003	-.266	-.002	.004
F4	<-->	F5	.096	.005	-.789	-.002	.007
F3	<-->	F5	.111	.006	.932	-.007	.008
F1	<-->	F5	.129	.006	.957	.003	.009
F6	<-->	F5	.109	.005	-.972	.001	.008
F6	<-->	F3	.100	.005	-.715	.002	.007
F6	<-->	F4	.126	.006	1.368	-.012	.009

Parameter	SE	SE-SE	Mean	Bias	SE-Bias
F6 <--> F2	.088	.004	.517	-.009	.006
F6 <--> F1	.103	.005	-.629	-.007	.007

Correlations: (g2 - Unconstrained)

Parameter	SE	SE-SE	Mean	Bias	SE-Bias
F1 <--> F2	.057	.003	-.565	-.006	.004
F2 <--> F3	.062	.003	-.477	-.007	.004
F1 <--> F3	.040	.002	.869	-.003	.003
F2 <--> F4	.033	.002	.826	-.003	.002
F3 <--> F4	.047	.002	-.684	-.004	.003
F1 <--> F4	.051	.003	-.612	-.002	.004
F2 <--> F5	.064	.003	-.455	-.004	.005
F4 <--> F5	.049	.002	-.609	.000	.003
F3 <--> F5	.039	.002	.834	-.004	.003
F1 <--> F5	.035	.002	.991	-.003	.002
F6 <--> F5	.046	.002	-.692	-.001	.003
F6 <--> F3	.055	.003	-.534	-.004	.004
F6 <--> F4	.035	.002	.880	-.004	.003
F6 <--> F2	.049	.002	.737	-.006	.003
F6 <--> F1	.059	.003	-.543	-.006	.004

Variances: (g2 - Unconstrained)

Parameter	SE	SE-SE	Mean	Bias	SE-Bias
**F1**	.137	.007	.799	.010	.010
**F2**	.084	.004	.298	.004	.006
**F3**	.150	.007	1.070	-.007	.011
**F4**	.150	.008	1.436	.005	.011
**F5**	.143	.007	1.174	.007	.010
**F6**	.194	.010	1.690	-.010	.014
**e1**	.185	.009	1.536	-.009	.013
**e2**	.095	.005	.820	-.008	.007
**e3**	.156	.008	1.417	-.005	.011
**e4**	.118	.006	1.387	-.020	.008
**e5**	.133	.007	2.054	.005	.009
**e6**	.180	.009	1.832	-.002	.013
**e7**	.189	.009	1.384	-.013	.013
**e8**	.159	.008	1.493	.000	.011
**e9**	.133	.007	1.076	-.009	.009
**e10**	.107	.005	.844	-.010	.008
**e11**	.077	.004	.656	-.014	.005
**e12**	.149	.007	1.182	.000	.011
**e13**	.126	.006	1.315	.007	.009
**e14**	.180	.009	1.145	-.017	.013
**e15**	.195	.010	1.569	.010	.014
**e16**	.179	.009	1.285	-.032	.013
**e17**	.114	.006	1.058	-.001	.008
**e18**	.137	.007	.830	-.007	.010
**e19**	.167	.008	1.094	-.012	.012
**e20**	.205	.010	1.364	-.014	.015
**e21**	.155	.008	1.026	-.001	.011
**e22**	.187	.009	1.157	-.015	.013
**e23**	.190	.009	1.603	.001	.013
**e24**	.199	.010	1.847	-.003	.014

Matrices (g2 - Unconstrained)

Sample Covariances - Standard Errors (g2 - Unconstrained)

	BPNSF6	BPNSF11	BPNSF17	BPNSF23	BPNSF3	BPNSF9	BPNSF14	BPNSF21	BPNSF2	BPNSF8	BPNSF20	BPNSF22	BPNSF4	BPNSF12	BPNSF16	BPNS[illegible]
**BPNSF6**	.172															
**BPNSF11**	.148	.172														
**BPNSF17**	.156	.161	.206													
**BPNSF23**	.150	.150	.164	.194												
**BPNSF3**	.134	.125	.124	.135	.183											
**BPNSF9**	.124	.136	.149	.132	.135	.193										
**BPNSF14**	.113	.138	.136	.120	.136	.153	.162									
**BPNSF21**	.120	.131	.139	.136	.121	.129	.138	.155								
**BPNSF2**	.144	.127	.139	.150	.106	.110	.114	.106	.166							

	BPNSF6	BPNSF11	BPNSF17	BPNSF23	BPNSF3	BPNSF9	BPNSF14	BPNSF21	BPNSF2	BPNSF8	BPNSF20	BPNSF22	BPNSF4	BPNSF12	BPNSF16	BPNS[illegible]
**BPNSF8**	.158	.147	.159	.146	.125	.127	.131	.135	.139	.169						
**BPNSF20**	.162	.141	.154	.172	.141	.134	.141	.148	.140	.153	.201					
**BPNSF22**	.146	.135	.139	.137	.120	.130	.129	.137	.125	.149	.138	.144				
**BPNSF4**	.132	.122	.119	.137	.137	.118	.121	.119	.110	.126	.146	.128	.141			
**BPNSF12**	.126	.127	.134	.134	.139	.135	.139	.132	.115	.127	.136	.129	.120	.142		
**BPNSF16**	.120	.123	.128	.131	.136	.126	.118	.137	.118	.136	.157	.134	.127	.134	.160	
**BPNSF24**	.124	.123	.135	.131	.132	.125	.122	.125	.128	.138	.141	.136	.125	.136	.132	.1[illegible]
**BPNSF5**	.139	.134	.144	.148	.136	.119	.109	.135	.110	.148	.162	.156	.139	.133	.123	.1[illegible]
**BPNSF10**	.146	.142	.149	.152	.138	.130	.123	.138	.131	.144	.152	.150	.137	.134	.135	.1[illegible]
**BPNSF15**	.138	.146	.157	.150	.137	.121	.121	.134	.135	.155	.151	.153	.136	.134	.135	.1[illegible]
**BPNSF18**	.138	.141	.140	.134	.119	.110	.105	.124	.123	.139	.135	.139	.132	.138	.124	.1[illegible]
**BPNSF1**	.101	.112	.101	.117	.107	.111	.111	.108	.112	.143	.138	.136	.115	.112	.108	.1[illegible]
**BPNSF7**	.130	.130	.139	.118	.131	.135	.122	.118	.102	.139	.118	.126	.113	.126	.120	.1[illegible]
**BPNSF13**	.115	.125	.124	.119	.111	.123	.132	.118	.104	.118	.124	.121	.104	.117	.116	.1[illegible]
**BPNSF19**	.128	.138	.132	.138	.120	.151	.132	.135	.103	.131	.141	.126	.124	.131	.121	.1[illegible]

Sample Correlations - Standard Errors (g2 - Unconstrained)

	BPNSF6	BPNSF11	BPNSF17	BPNSF23	BPNSF3	BPNSF9	BPNSF14	BPNSF21	BPNSF2	BPNSF8	BPNSF20	BPNSF22	BPNSF4	BPNSF12	BPNSF16	BPNS[illegible]
**BPNSF6**	.000															
**BPNSF11**	.047	.000														
**BPNSF17**	.051	.047	.000													
**BPNSF23**	.046	.045	.045	.000												
**BPNSF3**	.053	.050	.051	.053	.000											
**BPNSF9**	.050	.047	.051	.047	.059	.000										
**BPNSF14**	.046	.054	.046	.049	.060	.050	.000									
**BPNSF21**	.045	.049	.053	.047	.045	.048	.051	.000								
**BPNSF2**	.048	.046	.046	.048	.045	.046	.047	.041	.000							
**BPNSF8**	.048	.049	.045	.045	.048	.047	.051	.049	.043	.000						
**BPNSF20**	.053	.045	.045	.043	.053	.049	.053	.050	.047	.043	.000					
**BPNSF22**	.050	.047	.046	.043	.050	.049	.052	.051	.042	.045	.038	.000				
**BPNSF4**	.050	.049	.045	.050	.036	.047	.049	.040	.046	.048	.050	.050	.000			
**BPNSF12**	.051	.051	.050	.050	.059	.052	.048	.042	.046	.049	.049	.046	.047	.000		
**BPNSF16**	.048	.047	.049	.052	.061	.053	.043	.046	.046	.049	.052	.045	.053	.037	.000	
**BPNSF24**	.050	.048	.050	.051	.061	.053	.046	.046	.047	.049	.049	.044	.051	.045	.042	.0[illegible]
**BPNSF5**	.047	.050	.051	.049	.055	.050	.047	.054	.039	.047	.052	.053	.052	.058	.052	.0[illegible]
**BPNSF10**	.049	.043	.043	.043	.053	.048	.047	.047	.046	.044	.044	.047	.052	.053	.051	.0[illegible]
**BPNSF15**	.044	.046	.052	.049	.052	.047	.050	.051	.047	.048	.046	.047	.051	.053	.051	.0[illegible]
**BPNSF18**	.052	.050	.051	.052	.051	.048	.049	.053	.049	.051	.048	.052	.059	.063	.054	.0[illegible]
**BPNSF1**	.041	.047	.042	.049	.046	.051	.048	.044	.046	.055	.053	.054	.049	.051	.049	.0[illegible]
**BPNSF7**	.053	.054	.052	.051	.058	.050	.051	.049	.045	.053	.048	.052	.049	.054	.054	.0[illegible]
**BPNSF13**	.049	.050	.050	.049	.049	.051	.047	.038	.045	.045	.047	.046	.043	.035	.047	.0[illegible]
**BPNSF19**	.049	.052	.048	.052	.051	.056	.053	.047	.043	.048	.050	.049	.048	.052	.050	.0[illegible]

Sample Means - Standard Errors (g2 - Unconstrained)

	BPNSF6	BPNSF11	BPNSF17	BPNSF23	BPNSF3	BPNSF9	BPNSF14	BPNSF21	BPNSF2	BPNSF8	BPNSF20	BPNSF22	BPNSF4	BPNSF12	BPNSF16	BPNSF[illegible]
**BPNSF6**	.078	.090	.088	.076	.074	.074	.068	.074	.080	.093	.088	.089	.073	.067	.071	.07[illegible]

Bootstrap Confidence (g2 - Unconstrained)

Percentile method (g2 - Unconstrained)

90% confidence intervals (percentile method)

Scalar Estimates (g2 - Unconstrained)

Regression Weights: (g2 - Unconstrained)

Parameter			Estimate	Lower	Upper	P
BPNSF19	<---	F1	1.000	1.000	1.000	...
BPNSF13	<---	F1	1.170	1.021	1.348	.010
BPNSF7	<---	F1	.910	.752	1.084	.010
BPNSF1	<---	F1	.870	.678	1.130	.010
BPNSF18	<---	F2	1.000	1.000	1.000	...
BPNSF15	<---	F2	2.036	1.649	2.689	.010
BPNSF10	<---	F2	2.249	1.760	2.955	.010
BPNSF5	<---	F2	1.752	1.411	2.355	.010
BPNSF24	<---	F3	1.000	1.000	1.000	...

Parameter	Estimate	Lower	Upper	P
BPNSF16 <--- F3	1.099	.986	1.229	.010
BPNSF12 <--- F3	1.132	1.027	1.269	.010
BPNSF4 <--- F3	.949	.797	1.131	.010
BPNSF22 <--- F4	1.000	1.000	1.000	...
BPNSF20 <--- F4	1.107	.990	1.231	.010
BPNSF8 <--- F4	1.021	.902	1.144	.010
BPNSF2 <--- F4	.951	.817	1.099	.010
BPNSF21 <--- F5	1.000	1.000	1.000	...
BPNSF14 <--- F5	1.006	.880	1.128	.010
BPNSF9 <--- F5	.968	.835	1.088	.010
BPNSF3 <--- F5	.877	.735	1.020	.010
BPNSF23 <--- F6	1.000	1.000	1.000	...
BPNSF17 <--- F6	1.006	.910	1.098	.010
BPNSF11 <--- F6	.871	.769	.987	.010
BPNSF6 <--- F6	.799	.697	.916	.010

Standardized Regression Weights: (g2 - Unconstrained)

Parameter	Estimate	Lower	Upper	P
BPNSF19 <--- F1	.581	.500	.661	.010
BPNSF13 <--- F1	.753	.702	.803	.010
BPNSF7 <--- F1	.561	.480	.652	.010
BPNSF1 <--- F1	.546	.462	.623	.010
BPNSF18 <--- F2	.354	.276	.429	.010
BPNSF15 <--- F2	.632	.561	.693	.010
BPNSF10 <--- F2	.718	.645	.784	.010
BPNSF5 <--- F2	.614	.535	.697	.010
BPNSF24 <--- F3	.706	.638	.768	.010
BPNSF16 <--- F3	.777	.721	.829	.010
BPNSF12 <--- F3	.821	.780	.855	.010
BPNSF4 <--- F3	.671	.591	.741	.010
BPNSF22 <--- F4	.723	.671	.769	.010
BPNSF20 <--- F4	.776	.716	.830	.010
BPNSF8 <--- F4	.699	.633	.749	.010
BPNSF2 <--- F4	.704	.636	.777	.010
BPNSF21 <--- F5	.724	.675	.778	.010
BPNSF14 <--- F5	.765	.692	.834	.010
BPNSF9 <--- F5	.705	.631	.775	.010
BPNSF3 <--- F5	.628	.537	.719	.010
BPNSF23 <--- F6	.789	.737	.844	.010
BPNSF17 <--- F6	.771	.717	.835	.010
BPNSF11 <--- F6	.668	.598	.733	.010
BPNSF6 <--- F6	.608	.536	.678	.010

Intercepts: (g2 - Unconstrained)

Parameter	Estimate	Lower	Upper	P
**BPNSF19**	5.243	5.111	5.366	.010
**BPNSF13**	5.197	5.086	5.326	.010
**BPNSF7**	4.850	4.739	4.976	.010
**BPNSF1**	4.867	4.762	4.990	.010
**BPNSF18**	4.106	3.979	4.222	.010
**BPNSF15**	3.605	3.443	3.752	.010
**BPNSF10**	3.152	2.975	3.296	.010
**BPNSF5**	3.637	3.513	3.763	.010
**BPNSF24**	5.311	5.192	5.438	.010
**BPNSF16**	5.218	5.108	5.337	.010
**BPNSF12**	5.314	5.201	5.424	.010
**BPNSF4**	5.077	4.966	5.211	.010
**BPNSF22**	3.078	2.913	3.216	.010
**BPNSF20**	2.439	2.279	2.604	.010
**BPNSF8**	2.731	2.584	2.891	.010
**BPNSF2**	2.309	2.160	2.432	.010
**BPNSF21**	5.240	5.129	5.388	.010
**BPNSF14**	5.607	5.518	5.728	.010
**BPNSF9**	5.725	5.612	5.860	.010
**BPNSF3**	5.619	5.506	5.742	.010
**BPNSF23**	2.173	2.042	2.284	.010
**BPNSF17**	2.474	2.325	2.613	.010
**BPNSF11**	2.761	2.604	2.904	.010
**BPNSF6**	2.616	2.464	2.725	.010

Covariances: (g2 - Unconstrained)

Parameter	Estimate	Lower	Upper	P
F1 <--> F2	-.269	-.362	-.174	.010
F2 <--> F3	-.265	-.382	-.173	.010
F1 <--> F3	.805	.647	.971	.010
F2 <--> F4	.538	.405	.682	.010
F3 <--> F4	-.845	-1.025	-.666	.010
F1 <--> F4	-.649	-.823	-.507	.010
F2 <--> F5	-.264	-.370	-.178	.010
F4 <--> F5	-.787	-.940	-.645	.010
F3 <--> F5	.939	.755	1.140	.010
F1 <--> F5	.954	.751	1.187	.010
F6 <--> F5	-.972	-1.159	-.809	.010
F6 <--> F3	-.717	-.881	-.559	.010
F6 <--> F4	1.380	1.162	1.591	.010
F6 <--> F2	.526	.392	.692	.010
F6 <--> F1	-.622	-.812	-.471	.010

Correlations: (g2 - Unconstrained)

Parameter	Estimate	Lower	Upper	P
F1 <--> F2	-.559	-.646	-.460	.010
F2 <--> F3	-.470	-.578	-.379	.010
F1 <--> F3	.873	.797	.934	.010
F2 <--> F4	.829	.771	.879	.010
F3 <--> F4	-.680	-.753	-.598	.010
F1 <--> F4	-.611	-.697	-.529	.010
F2 <--> F5	-.452	-.563	-.340	.010
F4 <--> F5	-.609	-.683	-.524	.010
F3 <--> F5	.838	.771	.892	.010
F1 <--> F5	.994	.922	1.046	.010
F6 <--> F5	-.690	-.765	-.607	.010
F6 <--> F3	-.530	-.625	-.439	.010
F6 <--> F4	.885	.824	.937	.010
F6 <--> F2	.744	.655	.822	.010
F6 <--> F1	-.537	-.644	-.448	.010

Variances: (g2 - Unconstrained)

Parameter	Estimate	Lower	Upper	P
**F1**	.789	.579	1.040	.010
**F2**	.294	.171	.444	.010
**F3**	1.077	.817	1.345	.010
**F4**	1.431	1.185	1.667	.010
**F5**	1.167	.943	1.415	.010
**F6**	1.700	1.356	2.013	.010
**e1**	1.545	1.217	1.834	.010
**e2**	.827	.653	.993	.010
**e3**	1.422	1.131	1.703	.010
**e4**	1.407	1.201	1.581	.010
**e5**	2.048	1.819	2.292	.010
**e6**	1.834	1.533	2.145	.010
**e7**	1.397	1.077	1.710	.010
**e8**	1.493	1.258	1.776	.010
**e9**	1.085	.832	1.288	.010
**e10**	.854	.657	1.039	.010
**e11**	.670	.541	.787	.010
**e12**	1.183	.932	1.434	.010
**e13**	1.308	1.108	1.511	.010
**e14**	1.162	.860	1.486	.010
**e15**	1.560	1.292	1.904	.010
**e16**	1.316	1.007	1.590	.010
**e17**	1.058	.882	1.252	.010
**e18**	.837	.611	1.070	.010
**e19**	1.106	.865	1.388	.010
**e20**	1.378	.999	1.708	.010
**e21**	1.028	.775	1.316	.010
**e22**	1.172	.862	1.487	.010
**e23**	1.602	1.294	1.944	.010
**e24**	1.850	1.490	2.152	.010

Matrices (g2 - Unconstrained)

Sample Covariances (g2 - Unconstrained)

Sample Covariances - Lower Bounds (PC) (g2 - Unconstrained)

	BPNSF6	BPNSF11	BPNSF17	BPNSF23	BPNSF3	BPNSF9	BPNSF14	BPNSF21	BPNSF2	BPNSF8	BPNSF20	BPNSF22	BPNSF4	BPNSF12	BPNSF16	BPNS[illegible]
**BPNSF6**	2.640															
**BPNSF11**	.891	2.605														
**BPNSF17**	1.111	1.287	2.524													
**BPNSF23**	1.081	1.178	1.439	2.356												
**BPNSF3**	-1.078	-.866	-1.056	-1.127	1.966											
**BPNSF9**	-1.015	-1.175	-1.431	-1.265	.901	1.894										
**BPNSF14**	-.865	-1.012	-1.348	-1.044	.749	1.010	1.748									
**BPNSF21**	-.912	-1.066	-1.031	-1.110	.864	.789	.899	1.961								
**BPNSF2**	.965	.899	1.151	1.114	-.951	-.940	-1.008	-.937	2.284							
**BPNSF8**	1.025	.971	1.151	1.056	-.891	-.999	-.946	-.985	1.217	2.765						
**BPNSF20**	.804	1.037	1.209	1.445	-1.053	-1.035	-1.137	-1.258	1.291	1.354	2.521					
**BPNSF22**	.752	.931	1.034	1.071	-.676	-1.006	-.936	-1.043	1.090	1.177	1.390	2.498				
**BPNSF4**	-.894	-.814	-.880	-.948	1.180	.679	.637	.916	-1.049	-.999	-1.238	-1.002	1.923			
**BPNSF12**	-.790	-.940	-1.092	-1.000	.657	.749	.837	.888	-1.124	-1.045	-1.155	-1.267	.938	1.828		
**BPNSF16**	-.719	-.914	-.978	-.911	.523	.671	.831	.936	-1.042	-1.033	-1.288	-1.198	.810	1.192	1.895	
**BPNSF24**	-.753	-.847	-1.146	-.933	.426	.578	.735	.777	-1.115	-1.137	-1.285	-1.369	.738	.940	1.029	[illegible]
**BPNSF5**	.684	.456	.496	.656	-.665	-.524	-.550	-.775	.645	.884	.725	.707	-.987	-.715	-.620	[illegible]
**BPNSF10**	.657	1.177	.943	.995	-.873	-.978	-.940	-1.130	.888	1.085	1.109	1.050	-.987	-.940	-.880	[illegible]
**BPNSF15**	.656	.891	.709	.574	-.635	-.542	-.504	-.677	.622	.886	.881	.937	-.779	-.735	-.706	[illegible]
**BPNSF18**	-.021	.403	.358	.120	-.204	-.332	-.254	-.435	.163	.257	.408	.279	-.392	-.396	-.398	[illegible]
**BPNSF1**	-.469	-.552	-.534	-.620	.636	.476	.604	.740	-.829	-.743	-.854	-.792	.649	.520	.474	[illegible]
**BPNSF7**	-.244	-.762	-.915	-.539	.442	.741	.658	.623	-.661	-.820	-.754	-.921	.589	.531	.564	[illegible]
**BPNSF13**	-.710	-.995	-.929	-.885	.717	.729	.930	1.031	-.832	-.991	-.971	-1.061	.747	1.025	.837	[illegible]
**BPNSF19**	-.851	-1.074	-1.109	-1.157	.574	.743	.832	.928	-.719	-.930	-1.087	-.986	.648	.617	.555	[illegible]

Sample Covariances - Upper Bounds (PC) (g2 - Unconstrained)

	BPNSF6	BPNSF11	BPNSF17	BPNSF23	BPNSF3	BPNSF9	BPNSF14	BPNSF21	BPNSF2	BPNSF8	BPNSF20	BPNSF22	BPNSF4	BPNSF12	BPNSF16	BPNS[illegible]
**BPNSF6**	3.244															
**BPNSF11**	1.409	3.169														
**BPNSF17**	1.595	1.810	3.245													
**BPNSF23**	1.584	1.676	2.000	3.053												
**BPNSF3**	-.650	-.453	-.650	-.676	2.571											
**BPNSF9**	-.586	-.722	-.942	-.818	1.353	2.548										
**BPNSF14**	-.497	-.572	-.892	-.654	1.202	1.519	2.302									
**BPNSF21**	-.503	-.620	-.567	-.649	1.279	1.249	1.355	2.472								
**BPNSF2**	1.441	1.343	1.584	1.621	-.604	-.578	-.618	-.590	2.901							
**BPNSF8**	1.571	1.498	1.634	1.551	-.487	-.588	-.498	-.546	1.697	3.333						
**BPNSF20**	1.316	1.532	1.729	2.000	-.612	-.602	-.646	-.758	1.752	1.857	3.234					
**BPNSF22**	1.237	1.372	1.534	1.533	-.280	-.558	-.484	-.599	1.499	1.653	1.824	2.983				
**BPNSF4**	-.438	-.375	-.489	-.484	1.640	1.085	1.067	1.304	-.653	-.569	-.730	-.549	2.385			
**BPNSF12**	-.357	-.523	-.657	-.582	1.094	1.200	1.305	1.332	-.736	-.614	-.695	-.842	1.341	2.296		
**BPNSF16**	-.306	-.496	-.573	-.467	.968	1.080	1.215	1.390	-.639	-.581	-.747	-.757	1.232	1.657	2.448	
**BPNSF24**	-.323	-.448	-.713	-.502	.853	.968	1.163	1.190	-.691	-.684	-.825	-.896	1.138	1.393	1.468	[illegible]
**BPNSF5**	1.141	.911	.966	1.164	-.245	-.151	-.188	-.314	.999	1.377	1.261	1.229	-.509	-.294	-.202	[illegible]
**BPNSF10**	1.143	1.619	1.428	1.509	-.401	-.566	-.553	-.667	1.337	1.598	1.645	1.547	-.504	-.507	-.437	[illegible]
**BPNSF15**	1.106	1.392	1.251	1.116	-.142	-.136	-.089	-.218	1.052	1.361	1.366	1.436	-.316	-.281	-.243	[illegible]
**BPNSF18**	.441	.903	.793	.586	.215	.062	.096	-.034	.551	.728	.858	.745	.053	.064	.006	[illegible]
**BPNSF1**	-.144	-.178	-.189	-.209	1.004	.837	.964	1.074	-.469	-.269	-.387	-.324	1.047	.897	.833	[illegible]
**BPNSF7**	.184	-.296	-.439	-.139	.899	1.184	1.051	1.006	-.326	-.376	-.344	-.487	.975	.980	.950	[illegible]
**BPNSF13**	-.356	-.563	-.515	-.489	1.090	1.143	1.358	1.452	-.481	-.588	-.584	-.642	1.103	1.400	1.247	[illegible]
**BPNSF19**	-.424	-.587	-.680	-.646	.975	1.252	1.271	1.407	-.380	-.494	-.628	-.587	1.049	1.075	.966	[illegible]

Sample Covariances - Two Tailed Significance (PC) (g2 - Unconstrained)

	BPNSF6	BPNSF11	BPNSF17	BPNSF23	BPNSF3	BPNSF9	BPNSF14	BPNSF21	BPNSF2	BPNSF8	BPNSF20	BPNSF22	BPNSF4	BPNSF12	BPNSF16	BPNS[illegible]
**BPNSF6**	.010															
**BPNSF11**	.010	.010														
**BPNSF17**	.010	.010	.010													
**BPNSF23**	.010	.010	.010	.010												
**BPNSF3**	.010	.010	.010	.010	.010											
**BPNSF9**	.010	.010	.010	.010	.010	.010										
**BPNSF14**	.010	.010	.010	.010	.010	.010	.010									
**BPNSF21**	.010	.010	.010	.010	.010	.010	.010	.010								

	BPNSF6	BPNSF11	BPNSF17	BPNSF23	BPNSF3	BPNSF9	BPNSF14	BPNSF21	BPNSF2	BPNSF8	BPNSF20	BPNSF22	BPNSF4	BPNSF12	BPNSF16	BPNS[illegible]
**BPNSF2**	.010	.010	.010	.010	.010	.010	.010	.010	.010							
**BPNSF8**	.010	.010	.010	.010	.010	.010	.010	.010	.010	.010						
**BPNSF20**	.010	.010	.010	.010	.010	.010	.010	.010	.010	.010	.010					
**BPNSF22**	.010	.010	.010	.010	.010	.010	.010	.010	.010	.010	.010	.010				
**BPNSF4**	.010	.010	.010	.010	.010	.010	.010	.010	.010	.010	.010	.010	.010			
**BPNSF12**	.010	.010	.010	.010	.010	.010	.010	.010	.010	.010	.010	.010	.010	.010		
**BPNSF16**	.010	.010	.010	.010	.010	.010	.010	.010	.010	.010	.010	.010	.010	.010	.010	
**BPNSF24**	.010	.010	.010	.010	.010	.010	.010	.010	.010	.010	.010	.010	.010	.010	.010	[illegible]
**BPNSF5**	.010	.010	.010	.010	.010	.021	.010	.010	.010	.010	.010	.010	.010	.010	.013	[illegible]
**BPNSF10**	.010	.010	.010	.010	.010	.010	.010	.010	.010	.010	.010	.010	.010	.010	.010	[illegible]
**BPNSF15**	.010	.010	.010	.010	.016	.010	.023	.010	.010	.010	.010	.010	.010	.010	.010	[illegible]
**BPNSF18**	.121	.010	.010	.016	.946	.249	.578	.050	.010	.010	.010	.010	.152	.276	.108	[illegible]
**BPNSF1**	.010	.010	.010	.010	.010	.010	.010	.010	.010	.010	.010	.010	.010	.010	.010	[illegible]
**BPNSF7**	.692	.010	.010	.016	.010	.010	.010	.010	.010	.010	.010	.010	.010	.010	.010	[illegible]
**BPNSF13**	.010	.010	.010	.010	.010	.010	.010	.010	.010	.010	.010	.010	.010	.010	.010	[illegible]
**BPNSF19**	.010	.010	.010	.010	.010	.010	.010	.010	.010	.010	.010	.010	.010	.010	.010	[illegible]

Sample Correlations (g2 - Unconstrained)

Sample Correlations - Lower Bounds (PC) (g2 - Unconstrained)

	BPNSF6	BPNSF11	BPNSF17	BPNSF23	BPNSF3	BPNSF9	BPNSF14	BPNSF21	BPNSF2	BPNSF8	BPNSF20	BPNSF22	BPNSF4	BPNSF12	BPNSF16	BPNS[illegible]
**BPNSF6**	1.000															
**BPNSF11**	.309	1.000														
**BPNSF17**	.380	.452	1.000													
**BPNSF23**	.391	.435	.542	1.000												
**BPNSF3**	-.420	-.354	-.418	-.454	1.000											
**BPNSF9**	-.397	-.450	-.547	-.507	.412	1.000										
**BPNSF14**	-.358	-.418	-.538	-.449	.364	.496	1.000									
**BPNSF21**	-.351	-.406	-.399	-.437	.394	.365	.446	1.000								
**BPNSF2**	.358	.335	.422	.432	-.398	-.399	-.437	-.389	1.000							
**BPNSF8**	.344	.325	.406	.376	-.351	-.389	-.368	-.371	.450	1.000						
**BPNSF20**	.279	.367	.424	.538	-.411	-.405	-.452	-.473	.475	.467	1.000					
**BPNSF22**	.262	.323	.376	.399	-.270	-.388	-.386	-.417	.421	.417	.501	1.000				
**BPNSF4**	-.348	-.332	-.349	-.374	.569	.323	.320	.437	-.438	-.379	-.467	-.398	1.000			
**BPNSF12**	-.331	-.383	-.434	-.413	.306	.359	.439	.438	-.472	-.416	-.457	-.511	.457	1.000		
**BPNSF16**	-.285	-.356	-.390	-.372	.247	.312	.421	.457	-.424	-.397	-.492	-.468	.383	.620	1.000	
**BPNSF24**	-.296	-.348	-.443	-.383	.191	.267	.389	.353	-.453	-.430	-.507	-.538	.354	.482	.508	[illegible]
**BPNSF5**	.262	.169	.187	.266	-.281	-.221	-.243	-.324	.266	.336	.290	.289	-.416	-.320	-.261	[illegible]
**BPNSF10**	.230	.412	.344	.371	-.341	-.386	-.389	-.427	.331	.382	.397	.378	-.380	-.385	-.348	[illegible]
**BPNSF15**	.218	.309	.251	.208	-.242	-.205	-.206	-.252	.223	.286	.295	.327	-.302	-.296	-.273	[illegible]
**BPNSF18**	-.008	.160	.129	.050	-.085	-.143	-.119	-.191	.068	.098	.160	.109	-.171	-.182	-.174	[illegible]
**BPNSF1**	-.193	-.234	-.220	-.256	.306	.226	.310	.351	-.362	-.300	-.351	-.335	.328	.268	.240	[illegible]
**BPNSF7**	-.101	-.309	-.362	-.227	.207	.364	.333	.296	-.289	-.333	-.297	-.376	.286	.273	.260	[illegible]
**BPNSF13**	-.304	-.421	-.391	-.382	.350	.362	.500	.527	-.366	-.398	-.411	-.444	.384	.540	.432	[illegible]
**BPNSF19**	-.323	-.417	-.431	-.444	.252	.341	.394	.434	-.300	-.339	-.414	-.383	.294	.294	.258	[illegible]

Sample Correlations - Upper Bounds (PC) (g2 - Unconstrained)

	BPNSF6	BPNSF11	BPNSF17	BPNSF23	BPNSF3	BPNSF9	BPNSF14	BPNSF21	BPNSF2	BPNSF8	BPNSF20	BPNSF22	BPNSF4	BPNSF12	BPNSF16	BPNS[illegible]
**BPNSF6**	1.000															
**BPNSF11**	.477	1.000														
**BPNSF17**	.548	.610	1.000													
**BPNSF23**	.540	.586	.697	1.000												
**BPNSF3**	-.249	-.180	-.248	-.272	1.000											
**BPNSF9**	-.230	-.289	-.376	-.350	.604	1.000										
**BPNSF14**	-.205	-.239	-.388	-.288	.550	.674	1.000									
**BPNSF21**	-.196	-.252	-.218	-.265	.542	.530	.618	1.000								
**BPNSF2**	.510	.488	.573	.589	-.248	-.240	-.271	-.243	1.000							
**BPNSF8**	.509	.499	.545	.524	-.189	-.234	-.199	-.208	.595	1.000						
**BPNSF20**	.448	.525	.576	.684	-.245	-.237	-.281	-.302	.629	.605	1.000					
**BPNSF22**	.432	.482	.529	.546	-.112	-.225	-.210	-.236	.559	.554	.625	1.000				
**BPNSF4**	-.175	-.157	-.198	-.207	.693	.481	.473	.559	-.287	-.224	-.300	-.229	1.000			
**BPNSF12**	-.148	-.219	-.276	-.254	.501	.546	.590	.578	-.324	-.251	-.287	-.354	.621	1.000		
**BPNSF16**	-.119	-.204	-.238	-.192	.441	.484	.565	.606	-.277	-.227	-.308	-.322	.568	.732	1.000	
**BPNSF24**	-.123	-.178	-.281	-.213	.384	.437	.541	.513	-.299	-.271	-.347	-.397	.525	.633	.646	[illegible]
**BPNSF5**	.425	.335	.357	.429	-.112	-.065	-.084	-.134	.399	.497	.466	.467	-.235	-.135	-.090	[illegible]
**BPNSF10**	.388	.556	.481	.519	-.153	-.227	-.234	-.276	.483	.528	.552	.537	-.211	-.214	-.179	[illegible]
**BPNSF15**	.361	.469	.421	.379	-.054	-.052	-.036	-.085	.376	.441	.453	.481	-.130	-.115	-.095	[illegible]
**BPNSF18**	.165	.335	.312	.227	.091	.028	.045	-.014	.220	.264	.324	.285	.025	.029	.003	[illegible]
**BPNSF1**	-.061	-.074	-.083	-.091	.462	.409	.464	.495	-.207	-.111	-.164	-.146	.491	.442	.389	[illegible]

	BPNSF6	BPNSF11	BPNSF17	BPNSF23	BPNSF3	BPNSF9	BPNSF14	BPNSF21	BPNSF2	BPNSF8	BPNSF20	BPNSF22	BPNSF4	BPNSF12	BPNSF16	BPNSI
**BPNSF7**	.071	-.119	-.188	-.062	.408	.537	.493	.457	-.136	-.151	-.142	-.208	.444	.455	.438	.4
**BPNSF13**	-.150	-.243	-.220	-.215	.521	.534	.652	.657	-.216	-.248	-.256	-.287	.521	.660	.585	.5
**BPNSF19**	-.160	-.226	-.262	-.266	.421	.529	.572	.582	-.158	-.185	-.248	-.229	.451	.466	.420	.3

Sample Correlations - Two Tailed Significance (PC) (g2 - Unconstrained)

	BPNSF6	BPNSF11	BPNSF17	BPNSF23	BPNSF3	BPNSF9	BPNSF14	BPNSF21	BPNSF2	BPNSF8	BPNSF20	BPNSF22	BPNSF4	BPNSF12	BPNSF16	BPNSI
**BPNSF6**	...															
**BPNSF11**	.010	...														
**BPNSF17**	.010	.010	...													
**BPNSF23**	.010	.010	.010	...												
**BPNSF3**	.010	.010	.010	.010	...											
**BPNSF9**	.010	.010	.010	.010	.010	...										
**BPNSF14**	.010	.010	.010	.010	.010	.010	...									
**BPNSF21**	.010	.010	.010	.010	.010	.010	.010	...								
**BPNSF2**	.010	.010	.010	.010	.010	.010	.010	.010	...							
**BPNSF8**	.010	.010	.010	.010	.010	.010	.010	.010	.010	...						
**BPNSF20**	.010	.010	.010	.010	.010	.010	.010	.010	.010	.010	...					
**BPNSF22**	.010	.010	.010	.010	.010	.010	.010	.010	.010	.010	.010	...				
**BPNSF4**	.010	.010	.010	.010	.010	.010	.010	.010	.010	.010	.010	.010	...			
**BPNSF12**	.010	.010	.010	.010	.010	.010	.010	.010	.010	.010	.010	.010	.010	...		
**BPNSF16**	.010	.010	.010	.010	.010	.010	.010	.010	.010	.010	.010	.010	.010	.010	...	
**BPNSF24**	.010	.010	.010	.010	.010	.010	.010	.010	.010	.010	.010	.010	.010	.010	.010	
**BPNSF5**	.010	.010	.010	.010	.010	.021	.010	.010	.010	.010	.010	.010	.010	.010	.013	.0
**BPNSF10**	.010	.010	.010	.010	.010	.010	.010	.010	.010	.010	.010	.010	.010	.010	.010	.0
**BPNSF15**	.010	.010	.010	.010	.015	.010	.023	.010	.010	.010	.010	.010	.010	.010	.010	.0
**BPNSF18**	.121	.010	.010	.016	.946	.249	.578	.050	.010	.010	.010	.010	.152	.275	.108	.0
**BPNSF1**	.010	.010	.010	.010	.010	.010	.010	.010	.010	.010	.010	.010	.010	.010	.010	.0
**BPNSF7**	.692	.010	.010	.017	.010	.010	.010	.010	.010	.010	.010	.010	.010	.010	.010	.0
**BPNSF13**	.010	.010	.010	.010	.010	.010	.010	.010	.010	.010	.010	.010	.010	.010	.010	.0
**BPNSF19**	.010	.010	.010	.010	.010	.010	.010	.010	.010	.010	.010	.010	.010	.010	.010	.0

Sample Means (g2 - Unconstrained)

Sample Means - Lower Bounds (PC) (g2 - Unconstrained)

	BPNSF6	BPNSF11	BPNSF17	BPNSF23	BPNSF3	BPNSF9	BPNSF14	BPNSF21	BPNSF2	BPNSF8	BPNSF20	BPNSF22	BPNSF4	BPNSF12	BPNSF16	BPNSF
**BPNSF6**	2.464	2.604	2.325	2.042	5.506	5.612	5.518	5.129	2.160	2.584	2.279	2.913	4.966	5.201	5.108	5.19

Sample Means - Upper Bounds (PC) (g2 - Unconstrained)

	BPNSF6	BPNSF11	BPNSF17	BPNSF23	BPNSF3	BPNSF9	BPNSF14	BPNSF21	BPNSF2	BPNSF8	BPNSF20	BPNSF22	BPNSF4	BPNSF12	BPNSF16	BPNSF
**BPNSF6**	2.725	2.904	2.613	2.284	5.742	5.860	5.728	5.388	2.432	2.891	2.604	3.216	5.211	5.424	5.337	5.43

Sample Means - Two Tailed Significance (PC) (g2 - Unconstrained)

	BPNSF6	BPNSF11	BPNSF17	BPNSF23	BPNSF3	BPNSF9	BPNSF14	BPNSF21	BPNSF2	BPNSF8	BPNSF20	BPNSF22	BPNSF4	BPNSF12	BPNSF16	BPNSF
**BPNSF6**	.010	.010	.010	.010	.010	.010	.010	.010	.010	.010	.010	.010	.010	.010	.010	.01

Bias-corrected percentile method (g2 - Unconstrained)

90% confidence intervals (bias-corrected percentile method)

Scalar Estimates (g2 - Unconstrained)

Regression Weights: (g2 - Unconstrained)

Parameter			Estimate	Lower	Upper	P
BPNSF19	<---	F1	1.000	1.000	1.000	...
BPNSF13	<---	F1	1.170	1.035	1.393	.006
BPNSF7	<---	F1	.910	.762	1.109	.005
BPNSF1	<---	F1	.870	.682	1.131	.009
BPNSF18	<---	F2	1.000	1.000	1.000	...
BPNSF15	<---	F2	2.036	1.663	2.697	.009
BPNSF10	<---	F2	2.249	1.739	2.899	.012
BPNSF5	<---	F2	1.752	1.403	2.290	.014
BPNSF24	<---	F3	1.000	1.000	1.000	...
BPNSF16	<---	F3	1.099	.982	1.219	.020

Parameter			Estimate	Lower	Upper	P
BPNSF12	<---	F3	1.132	1.004	1.248	.021
BPNSF4	<---	F3	.949	.795	1.115	.013
BPNSF22	<---	F4	1.000	1.000	1.000	...
BPNSF20	<---	F4	1.107	1.015	1.255	.004
BPNSF8	<---	F4	1.021	.916	1.160	.004
BPNSF2	<---	F4	.951	.815	1.093	.011
BPNSF21	<---	F5	1.000	1.000	1.000	...
BPNSF14	<---	F5	1.006	.894	1.171	.005
BPNSF9	<---	F5	.968	.832	1.087	.012
BPNSF3	<---	F5	.877	.722	1.019	.012
BPNSF23	<---	F6	1.000	1.000	1.000	...
BPNSF17	<---	F6	1.006	.884	1.087	.023
BPNSF11	<---	F6	.871	.770	.987	.009
BPNSF6	<---	F6	.799	.691	.915	.012

Standardized Regression Weights: (g2 - Unconstrained)

Parameter			Estimate	Lower	Upper	P
BPNSF19	<---	F1	.581	.499	.659	.011
BPNSF13	<---	F1	.753	.702	.803	.010
BPNSF7	<---	F1	.561	.499	.664	.004
BPNSF1	<---	F1	.546	.445	.606	.025
BPNSF18	<---	F2	.354	.275	.428	.011
BPNSF15	<---	F2	.632	.560	.690	.012
BPNSF10	<---	F2	.718	.641	.783	.013
BPNSF5	<---	F2	.614	.525	.691	.016
BPNSF24	<---	F3	.706	.637	.766	.011
BPNSF16	<---	F3	.777	.715	.821	.020
BPNSF12	<---	F3	.821	.772	.851	.032
BPNSF4	<---	F3	.671	.589	.737	.013
BPNSF22	<---	F4	.723	.674	.770	.009
BPNSF20	<---	F4	.776	.715	.829	.012
BPNSF8	<---	F4	.699	.633	.751	.008
BPNSF2	<---	F4	.704	.627	.768	.018
BPNSF21	<---	F5	.724	.679	.782	.008
BPNSF14	<---	F5	.765	.689	.828	.012
BPNSF9	<---	F5	.705	.617	.768	.016
BPNSF3	<---	F5	.628	.526	.696	.021
BPNSF23	<---	F6	.789	.738	.849	.007
BPNSF17	<---	F6	.771	.717	.835	.010
BPNSF11	<---	F6	.668	.598	.731	.011
BPNSF6	<---	F6	.608	.534	.675	.012

Intercepts: (g2 - Unconstrained)

Parameter	Estimate	Lower	Upper	P
**BPNSF19**	5.243	5.097	5.357	.019
**BPNSF13**	5.197	5.040	5.288	.044
**BPNSF7**	4.850	4.674	4.940	.046
**BPNSF1**	4.867	4.705	4.959	.034
**BPNSF18**	4.106	4.000	4.244	.005
**BPNSF15**	3.605	3.457	3.768	.006
**BPNSF10**	3.152	3.014	3.342	.004
**BPNSF5**	3.637	3.493	3.745	.020
**BPNSF24**	5.311	5.163	5.422	.021
**BPNSF16**	5.218	5.100	5.334	.013
**BPNSF12**	5.314	5.196	5.422	.013
**BPNSF4**	5.077	4.964	5.195	.013
**BPNSF22**	3.078	2.935	3.223	.007
**BPNSF20**	2.439	2.318	2.619	.003
**BPNSF8**	2.731	2.603	2.912	.005
**BPNSF2**	2.309	2.186	2.446	.005
**BPNSF21**	5.240	5.106	5.362	.023
**BPNSF14**	5.607	5.474	5.694	.036
**BPNSF9**	5.725	5.602	5.855	.012
**BPNSF3**	5.619	5.500	5.740	.011
**BPNSF23**	2.173	2.053	2.313	.005
**BPNSF17**	2.474	2.327	2.614	.009
**BPNSF11**	2.761	2.620	2.929	.005
**BPNSF6**	2.616	2.453	2.721	.012

Covariances: (g2 - Unconstrained)

Parameter	Estimate	Lower	Upper	P
F1 <--> F2	-.269	-.361	-.172	.011
F2 <--> F3	-.265	-.397	-.186	.006
F1 <--> F3	.805	.661	1.003	.005
F2 <--> F4	.538	.406	.686	.008
F3 <--> F4	-.845	-1.025	-.666	.010
F1 <--> F4	-.649	-.828	-.510	.009
F2 <--> F5	-.264	-.371	-.179	.009
F4 <--> F5	-.787	-.944	-.651	.008
F3 <--> F5	.939	.766	1.167	.005
F1 <--> F5	.954	.763	1.195	.009
F6 <--> F5	-.972	-1.190	-.816	.006
F6 <--> F3	-.717	-.893	-.565	.007
F6 <--> F4	1.380	1.192	1.603	.005
F6 <--> F2	.526	.416	.737	.003
F6 <--> F1	-.622	-.817	-.472	.009

Correlations: (g2 - Unconstrained)

Parameter	Estimate	Lower	Upper	P
F1 <--> F2	-.559	-.628	-.420	.032
F2 <--> F3	-.470	-.559	-.349	.023
F1 <--> F3	.873	.790	.927	.015
F2 <--> F4	.829	.777	.882	.007
F3 <--> F4	-.680	-.745	-.588	.023
F1 <--> F4	-.611	-.696	-.520	.012
F2 <--> F5	-.452	-.548	-.328	.023
F4 <--> F5	-.609	-.677	-.512	.018
F3 <--> F5	.838	.774	.896	.008
F1 <--> F5	.994	.922	1.046	.010
F6 <--> F5	-.690	-.765	-.607	.010
F6 <--> F3	-.530	-.610	-.415	.023
F6 <--> F4	.885	.836	.956	.003
F6 <--> F2	.744	.679	.838	.003
F6 <--> F1	-.537	-.641	-.436	.016

Variances: (g2 - Unconstrained)

Parameter	Estimate	Lower	Upper	P
**F1**	.789	.561	1.021	.015
**F2**	.294	.159	.429	.014
**F3**	1.077	.860	1.388	.004
**F4**	1.431	1.189	1.672	.009
**F5**	1.167	.943	1.415	.010
**F6**	1.700	1.405	2.051	.007
**e1**	1.545	1.230	1.882	.007
**e2**	.827	.705	1.021	.003
**e3**	1.422	1.122	1.670	.012
**e4**	1.407	1.257	1.671	.002
**e5**	2.048	1.815	2.285	.011
**e6**	1.834	1.559	2.171	.006
**e7**	1.397	1.146	1.764	.004
**e8**	1.493	1.279	1.831	.004
**e9**	1.085	.866	1.301	.007
**e10**	.854	.667	1.054	.008
**e11**	.670	.560	.802	.004
**e12**	1.183	.933	1.440	.009
**e13**	1.308	1.085	1.508	.013
**e14**	1.162	.863	1.506	.007
**e15**	1.560	1.295	1.950	.007
**e16**	1.316	1.068	1.688	.003
**e17**	1.058	.892	1.263	.007
**e18**	.837	.624	1.084	.006
**e19**	1.106	.873	1.417	.006
**e20**	1.378	1.107	1.743	.004
**e21**	1.028	.793	1.317	.009
**e22**	1.172	.866	1.493	.009
**e23**	1.602	1.294	1.944	.010
**e24**	1.850	1.555	2.271	.006

Matrices (g2 - Unconstrained)

Sample Covariances (g2 - Unconstrained)

Sample Covariances - Lower Bounds (BC) (g2 - Unconstrained)

	BPNSF6	BPNSF11	BPNSF17	BPNSF23	BPNSF3	BPNSF9	BPNSF14	BPNSF21	BPNSF2	BPNSF8	BPNSF20	BPNSF22	BPNSF4	BPNSF12	BPNSF16	[illegible]
**BPNSF6**	2.619															
**BPNSF11**	.993	2.610														
**BPNSF17**	1.114	1.290	2.598													
**BPNSF23**	1.112	1.209	1.476	2.338												
**BPNSF3**	-1.071	-.875	-1.037	-1.124	1.965											
**BPNSF9**	-1.023	-1.198	-1.464	-1.308	.913	1.909										
**BPNSF14**	-.852	-1.041	-1.402	-1.087	.764	1.039	1.789									
**BPNSF21**	-.896	-1.108	-1.058	-1.105	.828	.836	.916	1.931								
**BPNSF2**	.965	.899	1.153	1.105	-.948	-.917	-1.040	-.935	2.371							
**BPNSF8**	1.018	.976	1.155	1.120	-.890	-.993	-.946	-.985	1.214	2.796						
**BPNSF20**	.817	1.084	1.257	1.508	-1.033	-1.035	-1.202	-1.264	1.305	1.404	2.550					
**BPNSF22**	.765	.973	1.126	1.076	-.673	-1.024	-.945	-1.042	1.090	1.183	1.376	2.470				
**BPNSF4**	-.871	-.834	-.868	-.922	1.171	.703	.640	.922	-1.020	-.964	-1.238	-.961	1.903			
**BPNSF12**	-.764	-.915	-1.091	-.993	.647	.779	.887	.918	-1.082	-1.058	-1.150	-1.227	.932	1.844		
**BPNSF16**	-.686	-.914	-.978	-.911	.543	.684	.854	.939	-1.029	-1.060	-1.285	-1.179	.813	1.192	1.862	
**BPNSF24**	-.704	-.847	-1.171	-.953	.439	.628	.792	.777	-1.149	-1.170	-1.282	-1.375	.733	.996	1.007	[illegible]
**BPNSF5**	.676	.459	.497	.633	-.652	-.493	-.539	-.730	.619	.895	.720	.742	-.952	-.712	-.597	[illegible]
**BPNSF10**	.666	1.238	.959	1.085	-.866	-1.021	-.978	-1.130	.911	1.149	1.117	1.080	-.944	-.956	-.886	[illegible]
**BPNSF15**	.657	.898	.742	.639	-.594	-.536	-.497	-.666	.630	.801	.889	.964	-.710	-.680	-.700	[illegible]
**BPNSF18**	-.009	.403	.310	.127	-.192	-.332	-.262	-.473	.161	.257	.401	.286	-.402	-.396	-.424	[illegible]
**BPNSF1**	-.418	-.543	-.520	-.594	.649	.503	.614	.739	-.828	-.732	-.820	-.777	.618	.537	.448	[illegible]
**BPNSF7**	-.218	-.788	-.961	-.549	.437	.750	.675	.635	-.653	-.815	-.756	-.936	.616	.590	.592	[illegible]
**BPNSF13**	-.704	-.996	-.945	-.872	.720	.759	.936	1.055	-.832	-.979	-.973	-1.061	.751	1.031	.832	[illegible]
**BPNSF19**	-.854	-1.084	-1.158	-1.093	.560	.740	.853	.948	-.710	-.915	-1.103	-.987	.640	.628	.556	[illegible]

Sample Covariances - Upper Bounds (BC) (g2 - Unconstrained)

	BPNSF6	BPNSF11	BPNSF17	BPNSF23	BPNSF3	BPNSF9	BPNSF14	BPNSF21	BPNSF2	BPNSF8	BPNSF20	BPNSF22	BPNSF4	BPNSF12	BPNSF16	[illegible]
**BPNSF6**	3.222															
**BPNSF11**	1.494	3.170														
**BPNSF17**	1.613	1.832	3.300													
**BPNSF23**	1.631	1.701	2.021	3.040												
**BPNSF3**	-.638	-.468	-.634	-.676	2.564											
**BPNSF9**	-.589	-.750	-.960	-.842	1.363	2.560										
**BPNSF14**	-.479	-.587	-.942	-.698	1.222	1.615	2.368									
**BPNSF21**	-.479	-.652	-.595	-.638	1.241	1.294	1.417	2.456								
**BPNSF2**	1.441	1.342	1.589	1.582	-.601	-.568	-.646	-.585	2.915							
**BPNSF8**	1.562	1.505	1.644	1.597	-.486	-.576	-.498	-.545	1.696	3.369						
**BPNSF20**	1.332	1.569	1.772	2.075	-.578	-.602	-.708	-.780	1.764	1.906	3.254					
**BPNSF22**	1.249	1.414	1.594	1.536	-.279	-.575	-.491	-.594	1.512	1.664	1.820	2.972				
**BPNSF4**	-.399	-.411	-.474	-.473	1.624	1.108	1.079	1.316	-.648	-.549	-.730	-.520	2.370			
**BPNSF12**	-.329	-.516	-.655	-.571	1.076	1.253	1.366	1.363	-.714	-.620	-.693	-.808	1.339	2.341		
**BPNSF16**	-.251	-.495	-.573	-.467	.968	1.106	1.223	1.401	-.638	-.594	-.743	-.743	1.233	1.657	2.400	
**BPNSF24**	-.286	-.448	-.725	-.525	.855	1.060	1.194	1.190	-.711	-.700	-.818	-.920	1.127	1.432	1.461	[illegible]
**BPNSF5**	1.136	.921	.988	1.126	-.224	-.112	-.174	-.292	.991	1.381	1.250	1.246	-.496	-.284	-.179	[illegible]
**BPNSF10**	1.144	1.686	1.433	1.566	-.395	-.573	-.570	-.667	1.351	1.627	1.650	1.600	-.470	-.514	-.442	[illegible]
**BPNSF15**	1.111	1.392	1.253	1.155	-.119	-.126	-.083	-.216	1.054	1.333	1.377	1.451	-.256	-.229	-.236	[illegible]
**BPNSF18**	.448	.903	.782	.590	.225	.062	.074	-.046	.550	.723	.857	.751	.006	.064	-.024	[illegible]
**BPNSF1**	-.040	-.168	-.186	-.208	1.008	.880	.998	1.070	-.464	-.268	-.330	-.303	1.031	.908	.782	[illegible]
**BPNSF7**	.226	-.311	-.476	-.177	.876	1.190	1.066	1.021	-.315	-.367	-.348	-.517	.989	1.002	1.015	[illegible]
**BPNSF13**	-.331	-.566	-.533	-.473	1.100	1.182	1.377	1.461	-.481	-.584	-.590	-.642	1.113	1.444	1.230	[illegible]
**BPNSF19**	-.433	-.630	-.719	-.643	.958	1.242	1.285	1.420	-.371	-.492	-.633	-.589	1.041	1.077	.966	[illegible]

Sample Covariances - Two Tailed Significance (BC) (g2 - Unconstrained)

	BPNSF6	BPNSF11	BPNSF17	BPNSF23	BPNSF3	BPNSF9	BPNSF14	BPNSF21	BPNSF2	BPNSF8	BPNSF20	BPNSF22	BPNSF4	BPNSF12	BPNSF16	[illegible]
**BPNSF6**	.013															
**BPNSF11**	.003	.009														
**BPNSF17**	.008	.007	.004													
**BPNSF23**	.003	.005	.006	.013												
**BPNSF3**	.012	.007	.014	.012	.013											
**BPNSF9**	.009	.006	.005	.005	.009	.006										
**BPNSF14**	.013	.007	.003	.004	.007	.004	.003									
**BPNSF21**	.018	.005	.005	.012	.020	.004	.006	.020								

	BPNSF6	BPNSF11	BPNSF17	BPNSF23	BPNSF3	BPNSF9	BPNSF14	BPNSF21	BPNSF2	BPNSF8	BPNSF20	BPNSF22	BPNSF4	BPNSF12	BPNSF16	BPNS[illegible]
**BPNSF2**	.010	.011	.009	.012	.011	.014	.005	.012	.003							
**BPNSF8**	.011	.008	.009	.002	.011	.012	.010	.011	.012	.006						
**BPNSF20**	.008	.005	.003	.003	.016	.010	.003	.008	.005	.003	.007					
**BPNSF22**	.007	.004	.002	.007	.012	.006	.007	.012	.009	.007	.012	.012				
**BPNSF4**	.016	.006	.015	.015	.012	.006	.009	.007	.014	.018	.010	.020	.014			
**BPNSF12**	.019	.013	.011	.014	.013	.005	.004	.004	.025	.008	.011	.018	.012	.005		
**BPNSF16**	.018	.011	.010	.010	.009	.007	.006	.009	.012	.007	.011	.015	.009	.010	.021	
**BPNSF24**	.021	.010	.007	.005	.009	.002	.004	.010	.004	.007	.014	.009	.012	.004	.012	[illegible]
**BPNSF5**	.012	.007	.008	.016	.021	.047	.013	.020	.016	.009	.012	.006	.020	.013	.022	[illegible]
**BPNSF10**	.009	.003	.008	.002	.011	.005	.005	.010	.006	.004	.007	.004	.015	.007	.009	[illegible]
**BPNSF15**	.007	.009	.008	.004	.029	.012	.034	.012	.007	.019	.007	.006	.028	.019	.012	[illegible]
**BPNSF18**	.109	.010	.018	.014	.926	.249	.434	.032	.011	.012	.011	.009	.120	.287	.063	[illegible]
**BPNSF1**	.030	.014	.015	.013	.007	.007	.006	.011	.012	.011	.020	.014	.014	.006	.030	[illegible]
**BPNSF7**	.985	.007	.005	.009	.013	.007	.005	.007	.013	.012	.009	.004	.005	.005	.005	[illegible]
**BPNSF13**	.015	.009	.006	.013	.008	.005	.007	.006	.010	.012	.009	.010	.007	.005	.013	[illegible]
**BPNSF19**	.007	.007	.003	.013	.015	.011	.005	.007	.016	.013	.007	.009	.012	.008	.009	[illegible]

Sample Correlations (g2 - Unconstrained)

Sample Correlations - Lower Bounds (BC) (g2 - Unconstrained)

	BPNSF6	BPNSF11	BPNSF17	BPNSF23	BPNSF3	BPNSF9	BPNSF14	BPNSF21	BPNSF2	BPNSF8	BPNSF20	BPNSF22	BPNSF4	BPNSF12	BPNSF16	BPNS[illegible]
**BPNSF6**	1.000															
**BPNSF11**	.342	1.000														
**BPNSF17**	.379	.451	1.000													
**BPNSF23**	.406	.436	.556	1.000												
**BPNSF3**	-.409	-.356	-.413	-.446	1.000											
**BPNSF9**	-.396	-.441	-.544	-.507	.416	1.000										
**BPNSF14**	-.345	-.418	-.539	-.447	.362	.500	1.000									
**BPNSF21**	-.345	-.415	-.400	-.432	.355	.384	.446	1.000								
**BPNSF2**	.358	.337	.412	.420	-.387	-.370	-.412	-.385	1.000							
**BPNSF8**	.351	.321	.402	.397	-.345	-.377	-.367	-.374	.442	1.000						
**BPNSF20**	.278	.373	.445	.544	-.404	-.400	-.469	-.471	.471	.469	1.000					
**BPNSF22**	.262	.337	.405	.399	-.269	-.385	-.386	-.420	.415	.418	.497	1.000				
**BPNSF4**	-.334	-.337	-.347	-.363	.569	.322	.320	.435	-.427	-.373	-.451	-.390	1.000			
**BPNSF12**	-.297	-.372	-.432	-.403	.301	.363	.437	.435	-.453	-.411	-.452	-.507	.449	1.000		
**BPNSF16**	-.277	-.360	-.389	-.379	.238	.311	.426	.461	-.414	-.397	-.475	-.463	.384	.608	1.000	
**BPNSF24**	-.286	-.331	-.442	-.392	.192	.275	.394	.352	-.447	-.429	-.487	-.535	.346	.482	.475	[illegible]
**BPNSF5**	.261	.176	.183	.265	-.266	-.208	-.241	-.302	.263	.337	.277	.295	-.395	-.316	-.247	[illegible]
**BPNSF10**	.233	.417	.344	.394	-.339	-.398	-.392	-.430	.322	.392	.397	.390	-.365	-.388	-.348	[illegible]
**BPNSF15**	.226	.311	.254	.229	-.222	-.205	-.198	-.247	.223	.274	.305	.331	-.276	-.268	-.271	[illegible]
**BPNSF18**	-.003	.153	.122	.051	-.085	-.143	-.127	-.203	.072	.092	.162	.112	-.178	-.182	-.183	[illegible]
**BPNSF1**	-.173	-.222	-.213	-.248	.306	.223	.302	.348	-.354	-.281	-.336	-.317	.300	.259	.220	[illegible]
**BPNSF7**	-.093	-.313	-.369	-.227	.204	.374	.345	.302	-.276	-.318	-.310	-.393	.294	.284	.275	[illegible]
**BPNSF13**	-.293	-.418	-.384	-.370	.349	.363	.496	.528	-.359	-.397	-.409	-.445	.376	.540	.432	[illegible]
**BPNSF19**	-.318	-.426	-.447	-.433	.244	.332	.395	.434	-.277	-.327	-.413	-.382	.293	.292	.258	[illegible]

Sample Correlations - Upper Bounds (BC) (g2 - Unconstrained)

	BPNSF6	BPNSF11	BPNSF17	BPNSF23	BPNSF3	BPNSF9	BPNSF14	BPNSF21	BPNSF2	BPNSF8	BPNSF20	BPNSF22	BPNSF4	BPNSF12	BPNSF16	BPNS[illegible]
**BPNSF6**	1.000															
**BPNSF11**	.486	1.000														
**BPNSF17**	.546	.607	1.000													
**BPNSF23**	.550	.589	.704	1.000												
**BPNSF3**	-.236	-.188	-.246	-.260	1.000											
**BPNSF9**	-.226	-.283	-.373	-.349	.606	1.000										
**BPNSF14**	-.190	-.244	-.388	-.283	.542	.675	1.000									
**BPNSF21**	-.189	-.254	-.221	-.260	.524	.536	.613	1.000								
**BPNSF2**	.510	.490	.566	.583	-.240	-.224	-.259	-.242	1.000							
**BPNSF8**	.510	.498	.544	.545	-.186	-.231	-.198	-.210	.592	1.000						
**BPNSF20**	.447	.530	.602	.686	-.240	-.237	-.288	-.300	.623	.608	1.000					
**BPNSF22**	.426	.488	.552	.547	-.105	-.222	-.211	-.245	.550	.563	.622	1.000				
**BPNSF4**	-.160	-.164	-.197	-.203	.693	.478	.473	.558	-.267	-.214	-.293	-.220	1.000			
**BPNSF12**	-.122	-.206	-.261	-.245	.493	.548	.588	.577	-.302	-.251	-.282	-.341	.617	1.000		
**BPNSF16**	-.118	-.205	-.230	-.202	.440	.483	.570	.611	-.261	-.227	-.294	-.311	.571	.724	1.000	
**BPNSF24**	-.115	-.170	-.278	-.216	.385	.446	.555	.512	-.297	-.271	-.333	-.384	.519	.633	.628	[illegible]
**BPNSF5**	.421	.350	.356	.429	-.094	-.047	-.081	-.128	.397	.498	.458	.478	-.223	-.115	-.063	[illegible]
**BPNSF10**	.392	.560	.481	.533	-.149	-.237	-.236	-.278	.477	.545	.551	.541	-.182	-.215	-.179	[illegible]
**BPNSF15**	.363	.472	.429	.385	-.042	-.049	-.030	-.083	.376	.439	.454	.496	-.095	-.087	-.092	[illegible]
**BPNSF18**	.168	.332	.300	.229	.091	.028	.034	-.022	.230	.262	.326	.287	.002	.029	-.008	[illegible]
**BPNSF1**	-.017	-.066	-.076	-.083	.462	.394	.456	.488	-.205	-.107	-.144	-.126	.480	.437	.378	[illegible]

	BPNSF6	BPNSF11	BPNSF17	BPNSF23	BPNSF3	BPNSF9	BPNSF14	BPNSF21	BPNSF2	BPNSF8	BPNSF20	BPNSF22	BPNSF4	BPNSF12	BPNSF16	BPNS[illegible]
**BPNSF7**	.089	-.125	-.203	-.064	.404	.548	.518	.467	-.132	-.131	-.147	-.214	.467	.464	.464	[illegible]
**BPNSF13**	-.136	-.240	-.212	-.205	.509	.535	.651	.657	-.208	-.246	-.256	-.288	.517	.660	.585	[illegible]
**BPNSF19**	-.147	-.243	-.289	-.255	.415	.516	.579	.582	-.130	-.182	-.247	-.227	.448	.465	.419	[illegible]

Sample Correlations - Two Tailed Significance (BC) (g2 - Unconstrained)

	BPNSF6	BPNSF11	BPNSF17	BPNSF23	BPNSF3	BPNSF9	BPNSF14	BPNSF21	BPNSF2	BPNSF8	BPNSF20	BPNSF22	BPNSF4	BPNSF12	BPNSF16	BPNS[illegible]
**BPNSF6**	...															
**BPNSF11**	.003	...														
**BPNSF17**	.012	.012	...													
**BPNSF23**	.005	.007	.006	...												
**BPNSF3**	.019	.008	.012	.014	...											
**BPNSF9**	.012	.019	.013	.011	.007	...										
**BPNSF14**	.019	.009	.009	.012	.013	.009	...									
**BPNSF21**	.016	.007	.009	.014	.032	.005	.011	...								
**BPNSF2**	.010	.008	.015	.016	.014	.026	.026	.015	...							
**BPNSF8**	.007	.012	.013	.003	.015	.016	.011	.009	.013	...						
**BPNSF20**	.012	.007	.004	.006	.018	.012	.005	.011	.015	.006	...					
**BPNSF22**	.012	.005	.002	.009	.013	.013	.009	.008	.015	.007	.018	...				
**BPNSF4**	.020	.006	.012	.014	.010	.013	.010	.012	.019	.015	.021	.015	...			
**BPNSF12**	.034	.019	.018	.019	.016	.007	.012	.011	.036	.012	.021	.018	.019	...		
**BPNSF16**	.012	.009	.012	.007	.012	.011	.007	.006	.019	.010	.015	.016	.009	.018	...	
**BPNSF24**	.020	.021	.012	.007	.009	.006	.005	.016	.015	.011	.034	.015	.013	.012	.039	
**BPNSF5**	.012	.005	.011	.011	.032	.047	.012	.028	.012	.009	.016	.006	.025	.016	.030	[illegible]
**BPNSF10**	.008	.007	.011	.004	.014	.006	.007	.007	.015	.004	.012	.005	.026	.009	.010	[illegible]
**BPNSF15**	.007	.008	.007	.004	.032	.011	.038	.013	.011	.012	.008	.007	.030	.023	.012	[illegible]
**BPNSF18**	.109	.012	.018	.015	.946	.249	.434	.032	.007	.015	.008	.009	.114	.275	.075	[illegible]
**BPNSF1**	.030	.016	.018	.018	.010	.013	.018	.019	.014	.012	.023	.019	.018	.015	.026	[illegible]
**BPNSF7**	.965	.009	.005	.015	.015	.005	.003	.008	.018	.016	.006	.005	.004	.008	.006	[illegible]
**BPNSF13**	.021	.012	.013	.019	.013	.009	.013	.009	.019	.012	.011	.009	.013	.010	.011	[illegible]
**BPNSF19**	.016	.006	.003	.015	.014	.019	.009	.011	.036	.021	.011	.012	.014	.011	.011	[illegible]

Sample Means (g2 - Unconstrained)

Sample Means - Lower Bounds (BC) (g2 - Unconstrained)

	BPNSF6	BPNSF11	BPNSF17	BPNSF23	BPNSF3	BPNSF9	BPNSF14	BPNSF21	BPNSF2	BPNSF8	BPNSF20	BPNSF22	BPNSF4	BPNSF12	BPNSF16	BPNSF[illegible]
**BPNSF6**	2.453	2.620	2.327	2.053	5.500	5.602	5.474	5.106	2.186	2.603	2.318	2.935	4.964	5.196	5.100	[illegible]

Sample Means - Upper Bounds (BC) (g2 - Unconstrained)

	BPNSF6	BPNSF11	BPNSF17	BPNSF23	BPNSF3	BPNSF9	BPNSF14	BPNSF21	BPNSF2	BPNSF8	BPNSF20	BPNSF22	BPNSF4	BPNSF12	BPNSF16	BPNSF[illegible]
**BPNSF6**	2.721	2.929	2.614	2.319	5.740	5.855	5.694	5.362	2.446	2.912	2.619	3.223	5.195	5.422	5.334	[illegible]

Sample Means - Two Tailed Significance (BC) (g2 - Unconstrained)

	BPNSF6	BPNSF11	BPNSF17	BPNSF23	BPNSF3	BPNSF9	BPNSF14	BPNSF21	BPNSF2	BPNSF8	BPNSF20	BPNSF22	BPNSF4	BPNSF12	BPNSF16	BPNSF[illegible]
**BPNSF6**	.012	.005	.009	.005	.011	.012	.036	.023	.005	.005	.003	.007	.013	.013	.013	[illegible]

g3 (g3 - Unconstrained)

Estimates (g3 - Unconstrained)

Scalar Estimates (g3 - Unconstrained)

Maximum Likelihood Estimates

Regression Weights: (g3 - Unconstrained)

			Estimate	S.E.	C.R.	P	Label
BPNSF19	<---	F1	1.000				
BPNSF13	<---	F1	1.183	.096	12.298	***	a1_3
BPNSF7	<---	F1	.892	.091	9.769	***	a2_3
BPNSF1	<---	F1	.837	.098	8.559	***	a3_3
BPNSF18	<---	F2	1.000				
BPNSF15	<---	F2	2.171	.481	4.515	***	a4_3
BPNSF10	<---	F2	3.437	.706	4.870	***	a5_3
BPNSF5	<---	F2	2.917	.612	4.766	***	a6_3

			Estimate	S.E.	C.R.	P	Label
BPNSF24	<---	F3	1.000				
BPNSF16	<---	F3	1.189	.090	13.264	***	a7_3
BPNSF12	<---	F3	1.368	.096	14.229	***	a8_3
BPNSF4	<---	F3	.849	.081	10.503	***	a9_3
BPNSF22	<---	F4	1.000				
BPNSF20	<---	F4	1.270	.094	13.575	***	a10_3
BPNSF8	<---	F4	1.319	.094	13.993	***	a11_3
BPNSF2	<---	F4	1.184	.094	12.664	***	a12_3
BPNSF21	<---	F5	1.000				
BPNSF14	<---	F5	1.164	.083	14.017	***	a13_3
BPNSF9	<---	F5	1.195	.083	14.439	***	a14_3
BPNSF3	<---	F5	.927	.081	11.461	***	a15_3
BPNSF23	<---	F6	1.000				
BPNSF17	<---	F6	.995	.060	16.677	***	a16_3
BPNSF11	<---	F6	.988	.056	17.521	***	a17_3
BPNSF6	<---	F6	.832	.060	13.971	***	a18_3

Standardized Regression Weights: (g3 - Unconstrained)

			Estimate
BPNSF19	<---	F1	.628
BPNSF13	<---	F1	.727
BPNSF7	<---	F1	.545
BPNSF1	<---	F1	.467
BPNSF18	<---	F2	.256
BPNSF15	<---	F2	.487
BPNSF10	<---	F2	.779
BPNSF5	<---	F2	.648
BPNSF24	<---	F3	.652
BPNSF16	<---	F3	.763
BPNSF12	<---	F3	.842
BPNSF4	<---	F3	.576
BPNSF22	<---	F4	.669
BPNSF20	<---	F4	.752
BPNSF8	<---	F4	.780
BPNSF2	<---	F4	.693
BPNSF21	<---	F5	.686
BPNSF14	<---	F5	.743
BPNSF9	<---	F5	.768
BPNSF3	<---	F5	.598
BPNSF23	<---	F6	.784
BPNSF17	<---	F6	.758
BPNSF11	<---	F6	.789
BPNSF6	<---	F6	.653

Intercepts: (g3 - Unconstrained)

	Estimate	S.E.	C.R.	P	Label
**BPNSF19**	4.926	.067	73.012	***	i1_3
**BPNSF13**	4.783	.069	69.372	***	i2_3
**BPNSF7**	4.717	.069	68.009	***	i3_3
**BPNSF1**	4.672	.076	61.590	***	i4_3
**BPNSF18**	4.474	.071	63.105	***	i5_3
**BPNSF15**	3.730	.081	46.228	***	i6_3
**BPNSF10**	3.118	.080	38.993	***	i7_3
**BPNSF5**	3.901	.082	47.826	***	i8_3
**BPNSF24**	5.117	.066	77.673	***	i9_3
**BPNSF16**	5.019	.067	75.003	***	i10_3
**BPNSF12**	5.107	.070	73.245	***	i11_3
**BPNSF4**	5.204	.063	82.253	***	i12_3
**BPNSF22**	3.274	.076	43.219	***	i13_3
**BPNSF20**	2.762	.086	32.243	***	i14_3
**BPNSF8**	2.916	.086	34.018	***	i15_3
**BPNSF2**	2.644	.087	30.500	***	i16_3
**BPNSF21**	4.999	.067	75.099	***	i17_3
**BPNSF14**	5.183	.072	72.471	***	i18_3
**BPNSF9**	5.461	.071	76.899	***	i19_3
**BPNSF3**	5.491	.071	77.537	***	i20_3
**BPNSF23**	2.503	.084	29.846	***	i21_3
**BPNSF17**	2.828	.086	32.764	***	i22_3
**BPNSF11**	2.806	.082	34.109	***	i23_3

	Estimate	S.E.	C.R.	P	Label
**BPNSF6**	2.632	.084	31.456	***	i24_3

Covariances: (g3 - Unconstrained)

	Estimate	S.E.	C.R.	P	Label
F1 <--> F2	-.158	.040	-3.949	***	ccc1_3
F2 <--> F3	-.135	.035	-3.829	***	ccc2_3
F1 <--> F3	.700	.081	8.625	***	ccc3_3
F2 <--> F4	.331	.074	4.487	***	ccc4_3
F3 <--> F4	-.510	.072	-7.135	***	ccc5_3
F1 <--> F4	-.368	.067	-5.485	***	ccc6_3
F2 <--> F5	-.158	.040	-3.957	***	ccc7_3
F4 <--> F5	-.581	.077	-7.513	***	ccc8_3
F3 <--> F5	.718	.081	8.915	***	ccc9_3
F1 <--> F5	.746	.084	8.856	***	ccc10_3
F6 <--> F5	-.980	.106	-9.224	***	ccc11_3
F6 <--> F3	-.674	.089	-7.587	***	ccc12_3
F6 <--> F4	1.304	.132	9.859	***	ccc13_3
F6 <--> F2	.417	.092	4.552	***	ccc14_3
F6 <--> F1	-.567	.088	-6.476	***	ccc15_3

Correlations: (g3 - Unconstrained)

	Estimate
F1 <--> F2	-.478
F2 <--> F3	-.403
F1 <--> F3	.895
F2 <--> F4	.838
F3 <--> F4	-.545
F1 <--> F4	-.398
F2 <--> F5	-.444
F4 <--> F5	-.583
F3 <--> F5	.852
F1 <--> F5	.897
F6 <--> F5	-.759
F6 <--> F3	-.556
F6 <--> F4	.909
F6 <--> F2	.815
F6 <--> F1	-.474

Variances: (g3 - Unconstrained)

	Estimate	S.E.	C.R.	P	Label
**F1**	.771	.114	6.791	***	vvv1_3
**F2**	.141	.057	2.466	.014	vvv2_3
**F3**	.792	.110	7.210	***	vvv3_3
**F4**	1.106	.148	7.486	***	vvv4_3
**F5**	.897	.115	7.786	***	vvv5_3
**F6**	1.858	.197	9.418	***	vvv6_3
**e1**	1.186	.090	13.127	***	v1_3
**e2**	.964	.083	11.638	***	v2_3
**e3**	1.455	.106	13.734	***	v3_3
**e4**	1.934	.137	14.074	***	v4_3
**e5**	2.020	.140	14.462	***	v5_3
**e6**	2.135	.155	13.747	***	v6_3
**e7**	1.082	.115	9.387	***	v7_3
**e8**	1.660	.133	12.446	***	v8_3
**e9**	1.074	.081	13.211	***	v9_3
**e10**	.806	.068	11.870	***	v10_3
**e11**	.608	.063	9.639	***	v11_3
**e12**	1.151	.084	13.695	***	v12_3
**e13**	1.362	.104	13.139	***	v13_3
**e14**	1.372	.113	12.168	***	v14_3
**e15**	1.238	.106	11.642	***	v15_3
**e16**	1.681	.130	12.923	***	v16_3
**e17**	1.008	.076	13.189	***	v17_3
**e18**	.984	.079	12.539	***	v18_3
**e19**	.889	.073	12.126	***	v19_3
**e20**	1.386	.101	13.773	***	v20_3
**e21**	1.166	.095	12.265	***	v21_3

	Estimate	S.E.	C.R.	P	Label
**e22**	1.362	.108	12.657	***	v22_3
**e23**	1.097	.090	12.167	***	v23_3
**e24**	1.726	.127	13.593	***	v24_3

Matrices (g3 - Unconstrained)

Residual Covariances (g3 - Unconstrained)

	BPNSF6	BPNSF11	BPNSF17	BPNSF23	BPNSF3	BPNSF9	BPNSF14	BPNSF21	BPNSF2	BPNSF8	BPNSF20	BPNSF22	BPNSF4	BPNSF12	BPNSF16	BPNS[illegible]
**BPNSF6**	.000															
**BPNSF11**	.120	.000														
**BPNSF17**	.020	-.061	.000													
**BPNSF23**	-.010	-.061	.052	.000												
**BPNSF3**	-.113	.011	-.120	-.164	.000											
**BPNSF9**	-.104	-.131	-.212	-.002	.133	.000										
**BPNSF14**	.187	.125	-.032	.062	-.015	.110	.000									
**BPNSF21**	.163	.043	.298	.014	-.069	-.133	-.050	.000								
**BPNSF2**	-.110	-.044	-.118	.108	-.301	.061	.063	.196	.000							
**BPNSF8**	.004	.054	-.071	-.112	-.080	-.127	.220	.106	.027	.000						
**BPNSF20**	-.092	-.082	.248	.306	-.251	-.186	.034	.007	-.017	-.065	.000					
**BPNSF22**	-.169	-.081	-.176	.112	-.143	.053	.169	-.056	-.080	.076	.053	.000				
**BPNSF4**	-.078	-.022	.067	-.015	.474	-.040	-.146	.026	.103	-.029	-.048	-.161	.000			
**BPNSF12**	.150	.089	.024	-.038	-.095	-.012	.060	.018	.085	.191	.049	-.129	-.044	.000		
**BPNSF16**	.142	-.006	-.074	.025	-.165	-.119	.045	.083	.086	.135	-.119	-.132	-.010	.039	.000	
**BPNSF24**	-.081	-.194	.018	-.188	-.084	-.072	-.012	.265	-.094	-.133	-.159	-.083	.009	-.020	-.009	[illegible]
**BPNSF5**	.057	.044	-.177	-.143	.032	.013	.119	.118	.320	.077	-.172	.059	-.132	-.036	-.109	[illegible]
**BPNSF10**	.092	.273	.018	-.073	-.174	-.261	.041	.016	.023	.112	-.166	-.020	-.136	.052	-.001	[illegible]
**BPNSF15**	-.049	-.009	.074	-.260	-.023	-.016	.042	.121	-.047	.135	-.136	.021	-.196	.125	.011	[illegible]
**BPNSF18**	-.208	-.112	-.005	-.314	.047	.345	.440	.242	-.182	-.191	-.178	.031	-.028	.363	.267	[illegible]
**BPNSF1**	.017	.013	.069	.085	.377	-.067	-.217	-.033	.187	-.031	-.036	-.279	.275	-.136	-.160	[illegible]
**BPNSF7**	.256	-.066	.029	.005	-.081	-.047	.020	.166	.115	.173	.059	-.089	-.056	-.030	-.073	[illegible]
**BPNSF13**	.068	-.053	-.022	.124	-.213	-.076	.034	-.001	.074	.068	-.014	-.219	.017	.170	.036	[illegible]
**BPNSF19**	-.055	-.131	.015	-.134	-.054	-.033	.018	.377	.118	-.025	-.036	-.090	.045	-.128	.019	[illegible]

Residual Means (g3 - Unconstrained)

	BPNSF6	BPNSF11	BPNSF17	BPNSF23	BPNSF3	BPNSF9	BPNSF14	BPNSF21	BPNSF2	BPNSF8	BPNSF20	BPNSF22	BPNSF4	BPNSF12	BPNSF16	BPNSF24	BP[illegible]
	.000	.000	.000	.000	.000	.000	.000	.000	.000	.000	.000	.000	.000	.000	.000	.000	[illegible]

Standardized Residual Covariances (g3 - Unconstrained)

	BPNSF6	BPNSF11	BPNSF17	BPNSF23	BPNSF3	BPNSF9	BPNSF14	BPNSF21	BPNSF2	BPNSF8	BPNSF20	BPNSF22	BPNSF4	BPNSF12	BPNSF16	BPNS[illegible]
**BPNSF6**	.000															
**BPNSF11**	.750	.000														
**BPNSF17**	.117	-.358	.000													
**BPNSF23**	-.063	-.360	.296	.000												
**BPNSF3**	-.883	.088	-.897	-1.258	.000											
**BPNSF9**	-.785	-.985	-1.526	-.013	1.163	.000										
**BPNSF14**	1.416	.937	-.228	.454	-.133	.906	.000									
**BPNSF21**	1.334	.348	2.329	.113	-.654	-1.202	-.455	.000								
**BPNSF2**	-.679	-.268	-.687	.643	-2.296	.456	.468	1.575	.000							
**BPNSF8**	.022	.324	-.405	-.655	-.612	-.949	1.637	.859	.156	.000						
**BPNSF20**	-.565	-.495	1.438	1.810	-1.929	-1.400	.255	.054	-.096	-.367	.000					
**BPNSF22**	-1.195	-.565	-1.177	.770	-1.255	.456	1.445	-.516	-.533	.503	.351	.000				
**BPNSF4**	-.695	-.199	.572	-.129	4.897	-.406	-1.457	.287	.884	-.253	-.420	-1.587	.000			
**BPNSF12**	1.182	.699	.180	-.292	-.855	-.098	.512	.172	.649	1.449	.372	-1.128	-.429	.000		
**BPNSF16**	1.178	-.054	-.588	.204	-1.562	-1.078	.408	.826	.689	1.077	-.952	-1.206	-.099	.340	.000	
**BPNSF24**	-.691	-1.657	.143	-1.575	-.829	-.679	-.116	2.719	-.768	-1.093	-1.316	-.780	.101	-.181	-.087	[illegible]
**BPNSF5**	.381	.290	-1.123	-.934	.263	.108	.962	1.030	2.044	.489	-1.098	.432	-1.224	-.297	-.941	[illegible]
**BPNSF10**	.614	1.789	.114	-.471	-1.449	-2.142	.337	.143	.146	.703	-1.049	-.146	-1.273	.436	-.011	[illegible]
**BPNSF15**	-.342	-.061	.488	-1.769	-.195	-.136	.349	1.074	-.313	.896	-.908	.161	-1.837	1.054	.101	[illegible]
**BPNSF18**	-1.679	-.916	-.041	-2.510	.446	3.293	4.166	2.469	-1.416	-1.491	-1.398	.280	-.305	3.526	2.709	[illegible]
**BPNSF1**	.131	.101	.503	.634	3.283	-.571	-1.843	-.306	1.360	-.225	-.267	-2.325	2.686	-1.168	-1.444	[illegible]
**BPNSF7**	2.098	-.550	.233	.040	-.763	-.434	.183	1.646	.915	1.385	.471	-.807	-.595	-.275	-.710	[illegible]
**BPNSF13**	.553	-.435	-.171	.999	-1.962	-.672	.296	-.007	.587	.543	-.110	-1.983	.171	1.495	.334	[illegible]
**BPNSF19**	-.459	-1.106	.121	-1.115	-.517	-.309	.165	3.781	.956	-.207	-.291	-.838	.489	-1.185	.184	[illegible]

Standardized Residual Means (g3 - Unconstrained)

	BPNSF6	BPNSF11	BPNSF17	BPNSF23	BPNSF3	BPNSF9	BPNSF14	BPNSF21	BPNSF2	BPNSF8	BPNSF20	BPNSF22	BPNSF4	BPNSF12	BPNSF16	BPNSF24	BP[illegible]

	BPNSF6	BPNSF11	BPNSF17	BPNSF23	BPNSF3	BPNSF9	BPNSF14	BPNSF21	BPNSF2	BPNSF8	BPNSF20	BPNSF22	BPNSF4	BPNSF12	BPNSF16	BPNSF24	BP[illegible]
	.000	.000	.000	.000	.000	.000	.000	.000	.000	.000	.000	.000	.000	.000	.000	.000	[illegible]

Notes for Group/Model (g3 - Unconstrained)

The following covariance matrix is not positive definite (g3 - Unconstrained)

	F5	F4	F3	F2	F1	F6
**F5**	.897					
**F4**	-.581	1.106				
**F3**	.718	-.510	.792			
**F2**	-.158	.331	-.135	.141		
**F1**	.746	-.368	.700	-.158	.771	
**F6**	-.980	1.304	-.674	.417	-.567	1.858

This solution is not admissible.

Modification Indices (g3 - Unconstrained)

Covariances: (g3 - Unconstrained)

			M.I.	Par Change
e24	<-->	F4	4.113	-.092
e23	<-->	F2	9.024	.050
e23	<-->	e24	4.058	.148
e21	<-->	F4	9.373	.117
e21	<-->	F2	12.692	-.061
e20	<-->	F4	5.788	-.098
e20	<-->	e23	5.403	.153
e19	<-->	e22	4.996	-.135
e19	<-->	e21	6.878	.148
e19	<-->	e20	7.515	.161
e18	<-->	e19	7.597	.141
e17	<-->	F5	11.679	-.096
e17	<-->	F1	14.539	.126
e17	<-->	e22	13.601	.231
e17	<-->	e19	10.584	-.166
e16	<-->	F2	4.885	.044
e16	<-->	F1	5.380	.101
e16	<-->	e20	11.367	-.269
e15	<-->	F2	4.467	.038
e15	<-->	e21	8.218	-.196
e15	<-->	e19	6.255	-.149
e14	<-->	F2	13.811	-.069
e14	<-->	F6	8.081	.137
e14	<-->	e23	6.140	-.170
e14	<-->	e22	13.871	.281
e14	<-->	e21	18.389	.303
e13	<-->	F5	8.979	.105
e13	<-->	F1	10.798	-.128
e13	<-->	e21	6.506	.175
e13	<-->	e19	10.661	.195
e13	<-->	e18	6.472	.158
e12	<-->	F2	5.991	-.040
e12	<-->	e20	69.585	.537
e12	<-->	e18	11.116	-.187
e11	<-->	e20	4.106	-.109
e10	<-->	e20	8.482	-.166
e9	<-->	e22	5.245	.149
e9	<-->	e17	17.256	.228
e8	<-->	F3	4.783	-.089
e8	<-->	e16	16.188	.364
e8	<-->	e14	4.182	-.171
e7	<-->	e23	10.709	.214
e7	<-->	e19	7.647	-.162
e7	<-->	e14	4.484	-.155
e6	<-->	e21	7.589	-.231
e6	<-->	e12	4.975	-.178
e5	<-->	F2	5.586	.050
e5	<-->	e22	4.889	.189
e5	<-->	e18	4.628	.156
e5	<-->	e12	7.176	-.204

			M.I.	Par Change
e5	<-->	e9	9.898	.235
e5	<-->	e8	17.553	.396
e5	<-->	e6	6.058	.254
e4	<-->	F2	16.559	-.085
e4	<-->	F6	6.304	.137
e4	<-->	e20	36.874	.500
e4	<-->	e18	6.388	-.182
e4	<-->	e16	5.492	.219
e4	<-->	e13	6.077	-.206
e4	<-->	e12	17.849	.318
e4	<-->	e6	5.704	-.244
e4	<-->	e5	27.993	-.516
e3	<-->	e24	6.888	.213
e3	<-->	e17	5.650	.148
e3	<-->	e7	6.663	-.185
e2	<-->	F5	6.414	-.074
e2	<-->	F3	7.345	.082
e2	<-->	e20	11.752	-.210
e2	<-->	e13	6.474	-.158
e2	<-->	e11	14.666	.177
e1	<-->	e17	37.926	.352
e1	<-->	e11	11.760	-.172

Variances: (g3 - Unconstrained)

	M.I.	Par Change

Regression Weights: (g3 - Unconstrained)

			M.I.	Par Change
BPNSF3	<---	BPNSF2	4.052	-.037
BPNSF18	<---	F5	14.473	.291
BPNSF18	<---	F3	14.943	.318
BPNSF18	<---	F1	10.906	.276

Means: (g3 - Unconstrained)

	M.I.	Par Change

Intercepts: (g3 - Unconstrained)

	M.I.	Par Change

Bootstrap (g3 - Unconstrained)

Bootstrap standard errors (g3 - Unconstrained)

Scalar Estimates (g3 - Unconstrained)

Regression Weights: (g3 - Unconstrained)

Parameter			SE	SE-SE	Mean	Bias	SE-Bias
BPNSF19	<---	F1	.000	.000	1.000	.000	.000
BPNSF13	<---	F1	.119	.006	1.182	-.002	.008
BPNSF7	<---	F1	.114	.006	.898	.006	.008
BPNSF1	<---	F1	.141	.007	.840	.003	.010
BPNSF18	<---	F2	.000	.000	1.000	.000	.000
BPNSF15	<---	F2	1.016	.051	2.416	.246	.072
BPNSF10	<---	F2	1.676	.084	3.859	.421	.118
BPNSF5	<---	F2	1.344	.067	3.251	.334	.095
BPNSF24	<---	F3	.000	.000	1.000	.000	.000
BPNSF16	<---	F3	.108	.005	1.186	-.003	.008
BPNSF12	<---	F3	.106	.005	1.360	-.008	.007
BPNSF4	<---	F3	.094	.005	.849	.000	.007
BPNSF22	<---	F4	.000	.000	1.000	.000	.000
BPNSF20	<---	F4	.087	.004	1.271	.001	.006
BPNSF8	<---	F4	.089	.004	1.316	-.003	.006
BPNSF2	<---	F4	.105	.005	1.182	-.002	.007
BPNSF21	<---	F5	.000	.000	1.000	.000	.000

Parameter	SE	SE-SE	Mean	Bias	SE-Bias
BPNSF14 <--- F5	.095	.005	1.171	.007	.007
BPNSF9 <--- F5	.117	.006	1.200	.005	.008
BPNSF3 <--- F5	.123	.006	.937	.009	.009
BPNSF23 <--- F6	.000	.000	1.000	.000	.000
BPNSF17 <--- F6	.059	.003	.994	-.001	.004
BPNSF11 <--- F6	.067	.003	.987	-.001	.005
BPNSF6 <--- F6	.066	.003	.833	.001	.005

Standardized Regression Weights: (g3 - Unconstrained)

Parameter	SE	SE-SE	Mean	Bias	SE-Bias
BPNSF19 <--- F1	.048	.002	.629	.002	.003
BPNSF13 <--- F1	.034	.002	.724	-.002	.002
BPNSF7 <--- F1	.050	.003	.547	.002	.004
BPNSF1 <--- F1	.061	.003	.467	.000	.004
BPNSF18 <--- F2	.060	.003	.250	-.005	.004
BPNSF15 <--- F2	.047	.002	.486	-.002	.003
BPNSF10 <--- F2	.034	.002	.779	.000	.002
BPNSF5 <--- F2	.040	.002	.646	-.002	.003
BPNSF24 <--- F3	.042	.002	.652	.001	.003
BPNSF16 <--- F3	.037	.002	.762	-.001	.003
BPNSF12 <--- F3	.023	.001	.840	-.002	.002
BPNSF4 <--- F3	.054	.003	.574	-.001	.004
BPNSF22 <--- F4	.034	.002	.671	.002	.002
BPNSF20 <--- F4	.037	.002	.754	.002	.003
BPNSF8 <--- F4	.030	.001	.777	-.003	.002
BPNSF2 <--- F4	.039	.002	.691	-.002	.003
BPNSF21 <--- F5	.044	.002	.689	.003	.003
BPNSF14 <--- F5	.038	.002	.748	.004	.003
BPNSF9 <--- F5	.034	.002	.771	.003	.002
BPNSF3 <--- F5	.062	.003	.601	.003	.004
BPNSF23 <--- F6	.028	.001	.785	.001	.002
BPNSF17 <--- F6	.033	.002	.758	.000	.002
BPNSF11 <--- F6	.030	.001	.788	-.002	.002
BPNSF6 <--- F6	.042	.002	.652	-.001	.003

Intercepts: (g3 - Unconstrained)

Parameter	SE	SE-SE	Mean	Bias	SE-Bias
**BPNSF19**	.074	.004	4.923	-.004	.005
**BPNSF13**	.066	.003	4.783	.000	.005
**BPNSF7**	.076	.004	4.718	.001	.005
**BPNSF1**	.078	.004	4.674	.002	.006
**BPNSF18**	.072	.004	4.476	.002	.005
**BPNSF15**	.074	.004	3.733	.003	.005
**BPNSF10**	.079	.004	3.118	-.001	.006
**BPNSF5**	.080	.004	3.895	-.006	.006
**BPNSF24**	.072	.004	5.115	-.002	.005
**BPNSF16**	.071	.004	5.023	.004	.005
**BPNSF12**	.071	.004	5.113	.006	.005
**BPNSF4**	.064	.003	5.204	-.001	.005
**BPNSF22**	.075	.004	3.273	-.001	.005
**BPNSF20**	.084	.004	2.754	-.008	.006
**BPNSF8**	.082	.004	2.920	.004	.006
**BPNSF2**	.087	.004	2.648	.003	.006
**BPNSF21**	.063	.003	5.000	.002	.004
**BPNSF14**	.073	.004	5.180	-.003	.005
**BPNSF9**	.080	.004	5.462	.001	.006
**BPNSF3**	.071	.004	5.487	-.004	.005
**BPNSF23**	.074	.004	2.504	.001	.005
**BPNSF17**	.087	.004	2.825	-.003	.006
**BPNSF11**	.076	.004	2.810	.004	.005
**BPNSF6**	.080	.004	2.641	.008	.006

Covariances: (g3 - Unconstrained)

Parameter	SE	SE-SE	Mean	Bias	SE-Bias
F1 <--> F2	.040	.002	-.152	.006	.003
F2 <--> F3	.032	.002	-.130	.005	.002
F1 <--> F3	.085	.004	.698	-.002	.006

Parameter			SE	SE-SE	Mean	Bias	SE-Bias
F2	<-->	F4	.082	.004	.322	-.009	.006
F3	<-->	F4	.086	.004	-.517	-.006	.006
F1	<-->	F4	.070	.003	-.369	-.002	.005
F2	<-->	F5	.035	.002	-.151	.007	.002
F4	<-->	F5	.080	.004	-.580	.001	.006
F3	<-->	F5	.100	.005	.719	.001	.007
F1	<-->	F5	.115	.006	.745	-.001	.008
F6	<-->	F5	.100	.005	-.982	-.002	.007
F6	<-->	F3	.098	.005	-.678	-.004	.007
F6	<-->	F4	.119	.006	1.303	.000	.008
F6	<-->	F2	.097	.005	.405	-.013	.007
F6	<-->	F1	.087	.004	-.572	-.005	.006

Correlations: (g3 - Unconstrained)

Parameter			SE	SE-SE	Mean	Bias	SE-Bias
F1	<-->	F2	.062	.003	-.478	.000	.004
F2	<-->	F3	.055	.003	-.404	-.002	.004
F1	<-->	F3	.036	.002	.891	-.004	.003
F2	<-->	F4	.038	.002	.837	-.001	.003
F3	<-->	F4	.058	.003	-.549	-.004	.004
F1	<-->	F4	.062	.003	-.399	-.001	.004
F2	<-->	F5	.061	.003	-.443	.002	.004
F4	<-->	F5	.053	.003	-.580	.003	.004
F3	<-->	F5	.044	.002	.848	-.004	.003
F1	<-->	F5	.051	.003	.890	-.007	.004
F6	<-->	F5	.036	.002	-.758	.001	.003
F6	<-->	F3	.046	.002	-.556	-.001	.003
F6	<-->	F4	.031	.002	.907	-.003	.002
F6	<-->	F2	.044	.002	.814	.000	.003
F6	<-->	F1	.051	.003	-.476	-.003	.004

Variances: (g3 - Unconstrained)

Parameter	SE	SE-SE	Mean	Bias	SE-Bias
**F1**	.127	.006	.777	.006	.009
**F2**	.064	.003	.142	.001	.005
**F3**	.109	.005	.797	.005	.008
**F4**	.138	.007	1.112	.007	.010
**F5**	.126	.006	.903	.006	.009
**F6**	.177	.009	1.866	.007	.012
**e1**	.143	.007	1.173	-.013	.010
**e2**	.107	.005	.960	-.004	.008
**e3**	.163	.008	1.438	-.017	.012
**e4**	.184	.009	1.915	-.019	.013
**e5**	.147	.007	1.998	-.022	.010
**e6**	.154	.008	2.124	-.010	.011
**e7**	.158	.008	1.074	-.008	.011
**e8**	.166	.008	1.662	.002	.012
**e9**	.131	.007	1.070	-.003	.009
**e10**	.114	.006	.797	-.009	.008
**e11**	.080	.004	.607	-.001	.006
**e12**	.151	.008	1.152	.001	.011
**e13**	.122	.006	1.347	-.016	.009
**e14**	.202	.010	1.350	-.022	.014
**e15**	.158	.008	1.249	.011	.011
**e16**	.206	.010	1.681	.000	.015
**e17**	.142	.007	.994	-.014	.010
**e18**	.146	.007	.964	-.020	.010
**e19**	.125	.006	.872	-.017	.009
**e20**	.222	.011	1.377	-.008	.016
**e21**	.138	.007	1.158	-.008	.010
**e22**	.175	.009	1.352	-.010	.012
**e23**	.138	.007	1.102	.005	.010
**e24**	.213	.011	1.737	.011	.015

Matrices (g3 - Unconstrained)

Sample Covariances - Standard Errors (g3 - Unconstrained)

	BPNSF6	BPNSF11	BPNSF17	BPNSF23	BPNSF3	BPNSF9	BPNSF14	BPNSF21	BPNSF2	BPNSF8	BPNSF20	BPNSF22	BPNSF4	BPNSF12	BPNSF16	BPNS[illegible]
**BPNSF6**	.172															
**BPNSF11**	.147	.150														
**BPNSF17**	.155	.155	.154													
**BPNSF23**	.141	.138	.141	.159												
**BPNSF3**	.128	.126	.124	.133	.187											
**BPNSF9**	.139	.121	.142	.136	.139	.176										
**BPNSF14**	.118	.113	.143	.130	.121	.136	.149									
**BPNSF21**	.111	.130	.133	.115	.121	.119	.118	.136								
**BPNSF2**	.167	.155	.165	.139	.132	.130	.133	.112	.181							
**BPNSF8**	.144	.147	.162	.141	.144	.145	.135	.133	.169	.164						
**BPNSF20**	.153	.154	.157	.153	.129	.140	.136	.133	.155	.154	.177					
**BPNSF22**	.139	.131	.153	.135	.134	.136	.137	.121	.129	.138	.139	.130				
**BPNSF4**	.113	.117	.114	.099	.133	.108	.106	.112	.124	.114	.114	.114	.128			
**BPNSF12**	.121	.118	.133	.125	.120	.129	.125	.104	.119	.138	.138	.124	.094	.135		
**BPNSF16**	.120	.114	.119	.118	.110	.124	.113	.111	.108	.132	.141	.118	.093	.111	.118	
**BPNSF24**	.110	.103	.121	.111	.120	.114	.112	.103	.123	.123	.123	.116	.102	.105	.093	[illegible]
**BPNSF5**	.139	.131	.140	.128	.131	.111	.125	.121	.163	.153	.154	.138	.110	.121	.113	[illegible]
**BPNSF10**	.154	.142	.144	.126	.121	.131	.114	.113	.147	.146	.136	.134	.111	.106	.116	[illegible]
**BPNSF15**	.166	.136	.157	.132	.126	.125	.130	.132	.133	.149	.156	.137	.112	.122	.114	[illegible]
**BPNSF18**	.125	.119	.134	.114	.111	.109	.119	.103	.140	.140	.127	.119	.103	.116	.112	[illegible]
**BPNSF1**	.147	.126	.146	.125	.143	.125	.131	.126	.139	.152	.142	.130	.114	.122	.121	[illegible]
**BPNSF7**	.129	.119	.133	.112	.133	.121	.119	.108	.134	.149	.123	.135	.116	.112	.116	[illegible]
**BPNSF13**	.129	.108	.124	.117	.122	.118	.120	.103	.125	.149	.136	.129	.097	.115	.109	[illegible]
**BPNSF19**	.104	.104	.126	.100	.098	.119	.120	.107	.113	.128	.124	.105	.096	.113	.103	[illegible]

Sample Correlations - Standard Errors (g3 - Unconstrained)

	BPNSF6	BPNSF11	BPNSF17	BPNSF23	BPNSF3	BPNSF9	BPNSF14	BPNSF21	BPNSF2	BPNSF8	BPNSF20	BPNSF22	BPNSF4	BPNSF12	BPNSF16	BPNS[illegible]
**BPNSF6**	.000															
**BPNSF11**	.044	.000														
**BPNSF17**	.048	.044	.000													
**BPNSF23**	.042	.035	.038	.000												
**BPNSF3**	.052	.053	.049	.052	.000											
**BPNSF9**	.050	.041	.044	.046	.058	.000										
**BPNSF14**	.046	.045	.050	.046	.051	.041	.000									
**BPNSF21**	.048	.051	.053	.045	.055	.053	.054	.000								
**BPNSF2**	.052	.047	.049	.042	.050	.049	.051	.049	.000							
**BPNSF8**	.043	.039	.046	.040	.054	.050	.052	.054	.048	.000						
**BPNSF20**	.047	.045	.039	.037	.049	.048	.048	.054	.045	.045	.000					
**BPNSF22**	.050	.042	.052	.041	.055	.059	.059	.055	.041	.040	.046	.000				
**BPNSF4**	.050	.052	.049	.044	.053	.054	.054	.059	.055	.050	.049	.056	.000			
**BPNSF12**	.047	.046	.048	.048	.053	.041	.047	.043	.046	.055	.052	.051	.049	.000		
**BPNSF16**	.050	.046	.046	.049	.057	.052	.046	.050	.044	.054	.052	.052	.053	.037	.000	
**BPNSF24**	.044	.041	.049	.043	.058	.052	.051	.044	.050	.050	.050	.053	.057	.043	.045	[illegible]
**BPNSF5**	.043	.039	.043	.039	.053	.044	.050	.053	.044	.044	.047	.047	.049	.048	.047	[illegible]
**BPNSF10**	.052	.038	.042	.039	.052	.048	.048	.050	.047	.042	.044	.049	.052	.045	.051	[illegible]
**BPNSF15**	.054	.044	.049	.045	.051	.049	.051	.057	.044	.046	.049	.048	.051	.051	.050	[illegible]
**BPNSF18**	.049	.048	.049	.045	.051	.050	.054	.051	.053	.053	.047	.049	.052	.055	.055	[illegible]
**BPNSF1**	.054	.049	.052	.046	.051	.051	.056	.058	.050	.055	.051	.051	.052	.053	.056	[illegible]
**BPNSF7**	.052	.050	.052	.045	.065	.055	.055	.050	.053	.059	.049	.060	.059	.051	.057	[illegible]
**BPNSF13**	.051	.046	.047	.050	.058	.047	.050	.048	.050	.061	.054	.055	.049	.036	.047	[illegible]
**BPNSF19**	.044	.042	.050	.042	.049	.051	.057	.038	.047	.051	.051	.049	.050	.051	.047	[illegible]

Sample Means - Standard Errors (g3 - Unconstrained)

	BPNSF6	BPNSF11	BPNSF17	BPNSF23	BPNSF3	BPNSF9	BPNSF14	BPNSF21	BPNSF2	BPNSF8	BPNSF20	BPNSF22	BPNSF4	BPNSF12	BPNSF16	BPNSF[illegible]
**BPNSF6**	.080	.076	.087	.074	.071	.080	.073	.063	.087	.082	.084	.075	.064	.071	.071	[illegible]

Bootstrap Confidence (g3 - Unconstrained)

Percentile method (g3 - Unconstrained)

90% confidence intervals (percentile method)

Scalar Estimates (g3 - Unconstrained)

Regression Weights: (g3 - Unconstrained)

Parameter	Estimate	Lower	Upper	P

Parameter			Estimate	Lower	Upper	P
BPNSF19	<---	F1	1.000	1.000	1.000	...
BPNSF13	<---	F1	1.183	1.003	1.398	.010
BPNSF7	<---	F1	.892	.714	1.067	.010
BPNSF1	<---	F1	.837	.610	1.103	.010
BPNSF18	<---	F2	1.000	1.000	1.000	...
BPNSF15	<---	F2	2.171	1.619	3.742	.010
BPNSF10	<---	F2	3.437	2.425	6.002	.010
BPNSF5	<---	F2	2.917	2.209	4.936	.010
BPNSF24	<---	F3	1.000	1.000	1.000	...
BPNSF16	<---	F3	1.189	1.012	1.381	.010
BPNSF12	<---	F3	1.368	1.216	1.573	.010
BPNSF4	<---	F3	.849	.688	1.000	.010
BPNSF22	<---	F4	1.000	1.000	1.000	...
BPNSF20	<---	F4	1.270	1.126	1.432	.010
BPNSF8	<---	F4	1.319	1.159	1.458	.010
BPNSF2	<---	F4	1.184	1.022	1.352	.010
BPNSF21	<---	F5	1.000	1.000	1.000	...
BPNSF14	<---	F5	1.164	1.028	1.353	.010
BPNSF9	<---	F5	1.195	1.027	1.434	.010
BPNSF3	<---	F5	.927	.728	1.129	.010
BPNSF23	<---	F6	1.000	1.000	1.000	...
BPNSF17	<---	F6	.995	.904	1.083	.010
BPNSF11	<---	F6	.988	.873	1.099	.010
BPNSF6	<---	F6	.832	.725	.949	.010

Standardized Regression Weights: (g3 - Unconstrained)

Parameter			Estimate	Lower	Upper	P
BPNSF19	<---	F1	.628	.544	.706	.010
BPNSF13	<---	F1	.727	.663	.780	.010
BPNSF7	<---	F1	.545	.461	.625	.010
BPNSF1	<---	F1	.467	.371	.574	.010
BPNSF18	<---	F2	.256	.148	.345	.010
BPNSF15	<---	F2	.487	.406	.556	.010
BPNSF10	<---	F2	.779	.724	.835	.010
BPNSF5	<---	F2	.648	.578	.713	.010
BPNSF24	<---	F3	.652	.577	.727	.010
BPNSF16	<---	F3	.763	.697	.818	.010
BPNSF12	<---	F3	.842	.799	.876	.010
BPNSF4	<---	F3	.576	.477	.654	.010
BPNSF22	<---	F4	.669	.614	.731	.010
BPNSF20	<---	F4	.752	.694	.810	.010
BPNSF8	<---	F4	.780	.726	.827	.010
BPNSF2	<---	F4	.693	.627	.753	.010
BPNSF21	<---	F5	.686	.617	.760	.010
BPNSF14	<---	F5	.743	.680	.805	.010
BPNSF9	<---	F5	.768	.713	.835	.010
BPNSF3	<---	F5	.598	.488	.702	.010
BPNSF23	<---	F6	.784	.740	.830	.010
BPNSF17	<---	F6	.758	.707	.810	.010
BPNSF11	<---	F6	.789	.733	.831	.010
BPNSF6	<---	F6	.653	.577	.718	.010

Intercepts: (g3 - Unconstrained)

Parameter	Estimate	Lower	Upper	P
**BPNSF19**	4.926	4.800	5.056	.010
**BPNSF13**	4.783	4.680	4.890	.010
**BPNSF7**	4.717	4.587	4.838	.010
**BPNSF1**	4.672	4.530	4.800	.010
**BPNSF18**	4.474	4.362	4.602	.010
**BPNSF15**	3.730	3.606	3.847	.010
**BPNSF10**	3.118	2.986	3.241	.010
**BPNSF5**	3.901	3.773	4.058	.010
**BPNSF24**	5.117	5.001	5.238	.010
**BPNSF16**	5.019	4.900	5.142	.010
**BPNSF12**	5.107	5.000	5.235	.010
**BPNSF4**	5.204	5.093	5.299	.010
**BPNSF22**	3.274	3.135	3.388	.010
**BPNSF20**	2.762	2.601	2.890	.010
**BPNSF8**	2.916	2.779	3.059	.010

Parameter	Estimate	Lower	Upper	P
**BPNSF2**	2.644	2.510	2.809	.010
**BPNSF21**	4.999	4.893	5.097	.010
**BPNSF14**	5.183	5.051	5.295	.010
**BPNSF9**	5.461	5.333	5.592	.010
**BPNSF3**	5.491	5.375	5.607	.010
**BPNSF23**	2.503	2.385	2.629	.010
**BPNSF17**	2.828	2.685	2.973	.010
**BPNSF11**	2.806	2.681	2.931	.010
**BPNSF6**	2.632	2.499	2.772	.010

Covariances: (g3 - Unconstrained)

Parameter	Estimate	Lower	Upper	P
F1 <--> F2	-.158	-.216	-.084	.010
F2 <--> F3	-.135	-.179	-.080	.010
F1 <--> F3	.700	.556	.843	.010
F2 <--> F4	.331	.189	.461	.010
F3 <--> F4	-.510	-.666	-.378	.010
F1 <--> F4	-.368	-.489	-.248	.010
F2 <--> F5	-.158	-.201	-.095	.010
F4 <--> F5	-.581	-.724	-.463	.010
F3 <--> F5	.718	.567	.911	.010
F1 <--> F5	.746	.556	.958	.010
F6 <--> F5	-.980	-1.131	-.811	.010
F6 <--> F3	-.674	-.832	-.526	.010
F6 <--> F4	1.304	1.104	1.495	.010
F6 <--> F2	.417	.245	.567	.010
F6 <--> F1	-.567	-.701	-.428	.010

Correlations: (g3 - Unconstrained)

Parameter	Estimate	Lower	Upper	P
F1 <--> F2	-.478	-.583	-.365	.010
F2 <--> F3	-.403	-.505	-.315	.010
F1 <--> F3	.895	.825	.948	.010
F2 <--> F4	.838	.769	.900	.010
F3 <--> F4	-.545	-.648	-.450	.010
F1 <--> F4	-.398	-.509	-.295	.010
F2 <--> F5	-.444	-.538	-.334	.010
F4 <--> F5	-.583	-.667	-.485	.010
F3 <--> F5	.852	.770	.914	.010
F1 <--> F5	.897	.805	.971	.010
F6 <--> F5	-.759	-.825	-.704	.010
F6 <--> F3	-.556	-.631	-.475	.010
F6 <--> F4	.909	.846	.952	.010
F6 <--> F2	.815	.738	.888	.010
F6 <--> F1	-.474	-.552	-.396	.010

Variances: (g3 - Unconstrained)

Parameter	Estimate	Lower	Upper	P
**F1**	.771	.556	.970	.010
**F2**	.141	.048	.260	.010
**F3**	.792	.622	.982	.010
**F4**	1.106	.898	1.380	.010
**F5**	.897	.702	1.138	.010
**F6**	1.858	1.553	2.148	.010
**e1**	1.186	.954	1.404	.010
**e2**	.964	.796	1.139	.010
**e3**	1.455	1.168	1.718	.010
**e4**	1.934	1.628	2.234	.010
**e5**	2.020	1.782	2.248	.010
**e6**	2.135	1.870	2.371	.010
**e7**	1.082	.835	1.367	.010
**e8**	1.660	1.394	1.933	.010
**e9**	1.074	.831	1.283	.010
**e10**	.806	.608	1.008	.010
**e11**	.608	.476	.749	.010
**e12**	1.151	.927	1.467	.010
**e13**	1.362	1.133	1.555	.010

Parameter	Estimate	Lower	Upper	P
**e14**	1.372	1.046	1.693	.010
**e15**	1.238	.972	1.508	.010
**e16**	1.681	1.342	2.098	.010
**e17**	1.008	.773	1.239	.010
**e18**	.984	.737	1.215	.010
**e19**	.889	.672	1.069	.010
**e20**	1.386	1.051	1.787	.010
**e21**	1.166	.932	1.369	.010
**e22**	1.362	1.049	1.630	.010
**e23**	1.097	.888	1.339	.010
**e24**	1.726	1.410	2.091	.010

Matrices (g3 - Unconstrained)

Sample Covariances (g3 - Unconstrained)

Sample Covariances - Lower Bounds (PC) (g3 - Unconstrained)

	BPNSF6	BPNSF11	BPNSF17	BPNSF23	BPNSF3	BPNSF9	BPNSF14	BPNSF21	BPNSF2	BPNSF8	BPNSF20	BPNSF22	BPNSF4	BPNSF12	BPNSF16	BPNS[illegible]
**BPNSF6**	2.743															
**BPNSF11**	1.349	2.668														
**BPNSF17**	1.289	1.488	2.920													
**BPNSF23**	1.291	1.528	1.661	2.752												
**BPNSF3**	-1.083	-1.079	-1.205	-1.282	1.804											
**BPNSF9**	-1.293	-1.465	-1.607	-1.391	.896	1.840										
**BPNSF14**	-.981	-1.200	-1.410	-1.277	.779	1.116	1.963									
**BPNSF21**	-.839	-1.153	-.933	-1.145	.574	.751	.818	1.654								
**BPNSF2**	.909	1.221	1.125	1.401	-1.146	-.933	-.956	-.654	2.898							
**BPNSF8**	1.172	1.519	1.360	1.366	-1.027	-1.284	-.909	-.865	1.443	2.852						
**BPNSF20**	.999	1.299	1.625	1.706	-1.148	-1.322	-1.074	-.940	1.357	1.506	2.811					
**BPNSF22**	.662	.991	.860	1.198	-.919	-.860	-.732	-.818	1.025	1.300	1.226	2.221				
**BPNSF4**	-.745	-.794	-.705	-.753	.837	.506	.367	.458	-.612	-.789	-.804	-.777	1.526			
**BPNSF12**	-.819	-.996	-1.115	-1.163	.631	.924	.987	.826	-.925	-.950	-1.094	-1.015	.712	1.822		
**BPNSF16**	-.728	-1.007	-1.080	-.975	.431	.697	.841	.735	-.817	-.919	-1.149	-.926	.632	1.141	1.723	
**BPNSF24**	-.835	-1.018	-.843	-1.042	.391	.590	.663	.792	-.893	-1.015	-1.010	-.763	.519	.899	.767	[illegible]
**BPNSF5**	.837	1.035	.784	.864	-.603	-.724	-.633	-.517	1.186	1.098	.797	.778	-.635	-.784	-.744	[illegible]
**BPNSF10**	1.020	1.468	1.237	1.177	-.882	-1.125	-.776	-.721	1.108	1.378	1.053	.885	-.716	-.766	-.742	[illegible]
**BPNSF15**	.415	.670	.684	.429	-.548	-.619	-.563	-.433	.560	.831	.520	.532	-.637	-.447	-.509	[illegible]
**BPNSF18**	-.061	.075	.164	-.094	-.281	-.012	.079	-.052	-.056	.007	.017	.161	-.295	.009	-.080	[illegible]
**BPNSF1**	-.630	-.669	-.641	-.604	.708	.479	.301	.391	-.414	-.686	-.675	-.834	.589	.469	.326	[illegible]
**BPNSF7**	-.373	-.775	-.674	-.707	.312	.517	.616	.652	-.490	-.486	-.562	-.629	.288	.628	.471	[illegible]
**BPNSF13**	-.698	-.895	-.905	-.729	.406	.788	.864	.681	-.643	-.759	-.808	-.870	.536	1.091	.821	[illegible]
**BPNSF19**	-.721	-.859	-.760	-.880	.466	.669	.688	.945	-.522	-.721	-.739	-.637	.463	.612	.649	[illegible]

Sample Covariances - Upper Bounds (PC) (g3 - Unconstrained)

	BPNSF6	BPNSF11	BPNSF17	BPNSF23	BPNSF3	BPNSF9	BPNSF14	BPNSF21	BPNSF2	BPNSF8	BPNSF20	BPNSF22	BPNSF4	BPNSF12	BPNSF16	BPNS[illegible]
**BPNSF6**	3.307															
**BPNSF11**	1.863	3.156														
**BPNSF17**	1.813	2.010	3.439													
**BPNSF23**	1.794	1.990	2.120	3.260												
**BPNSF3**	-.659	-.682	-.834	-.858	2.437											
**BPNSF9**	-.825	-1.065	-1.124	-.938	1.357	2.458										
**BPNSF14**	-.593	-.820	-.922	-.875	1.177	1.571	2.426									
**BPNSF21**	-.473	-.703	-.465	-.773	.974	1.142	1.213	2.113								
**BPNSF2**	1.451	1.718	1.678	1.867	-.712	-.538	-.503	-.290	3.519							
**BPNSF8**	1.664	2.010	1.869	1.840	-.548	-.797	-.480	-.410	2.022	3.416						
**BPNSF20**	1.524	1.790	2.155	2.208	-.739	-.850	-.621	-.491	1.912	2.033	3.413					
**BPNSF22**	1.124	1.403	1.360	1.659	-.460	-.401	-.269	-.416	1.452	1.757	1.712	2.691				
**BPNSF4**	-.361	-.388	-.305	-.429	1.288	.878	.723	.835	-.188	-.406	-.427	-.389	1.950			
**BPNSF12**	-.428	-.610	-.662	-.729	1.005	1.373	1.415	1.160	-.526	-.492	-.635	-.614	1.038	2.278		
**BPNSF16**	-.334	-.596	-.664	-.596	.821	1.106	1.227	1.099	-.455	-.443	-.676	-.542	.946	1.485	2.089	
**BPNSF24**	-.457	-.681	-.430	-.656	.778	.976	1.024	1.156	-.507	-.599	-.609	-.386	.854	1.240	1.097	[illegible]
**BPNSF5**	1.294	1.448	1.258	1.280	-.149	-.359	-.213	-.119	1.709	1.583	1.312	1.241	-.266	-.369	-.367	[illegible]
**BPNSF10**	1.549	1.937	1.684	1.584	-.469	-.704	-.381	-.337	1.620	1.849	1.526	1.344	-.313	-.412	-.363	[illegible]
**BPNSF15**	.959	1.134	1.223	.871	-.137	-.202	-.131	-.007	1.008	1.287	1.016	.972	-.272	-.030	-.132	[illegible]
**BPNSF18**	.343	.484	.618	.305	.093	.344	.454	.271	.410	.455	.461	.562	.020	.383	.299	[illegible]
**BPNSF1**	-.133	-.238	-.144	-.182	1.181	.888	.742	.810	.034	-.185	-.192	-.379	.974	.864	.732	[illegible]
**BPNSF7**	.060	-.381	-.236	-.313	.769	.925	.991	1.017	-.034	-.008	-.147	-.191	.662	.982	.849	[illegible]
**BPNSF13**	-.247	-.528	-.482	-.358	.823	1.163	1.256	1.038	-.233	-.254	-.357	-.446	.866	1.454	1.177	[illegible]

	BPNSF6	BPNSF11	BPNSF17	BPNSF23	BPNSF3	BPNSF9	BPNSF14	BPNSF21	BPNSF2	BPNSF8	BPNSF20	BPNSF22	BPNSF4	BPNSF12	BPNSF16	BPNS
**BPNSF19**	-.367	-.528	-.354	-.548	.794	1.060	1.088	1.290	-.148	-.304	-.295	-.280	.784	1.010	1.001	.8

Sample Covariances - Two Tailed Significance (PC) (g3 - Unconstrained)

	BPNSF6	BPNSF11	BPNSF17	BPNSF23	BPNSF3	BPNSF9	BPNSF14	BPNSF21	BPNSF2	BPNSF8	BPNSF20	BPNSF22	BPNSF4	BPNSF12	BPNSF16	BPNS
**BPNSF6**	.010															
**BPNSF11**	.010	.010														
**BPNSF17**	.010	.010	.010													
**BPNSF23**	.010	.010	.010	.010												
**BPNSF3**	.010	.010	.010	.010	.010											
**BPNSF9**	.010	.010	.010	.010	.010	.010										
**BPNSF14**	.010	.010	.010	.010	.010	.010	.010									
**BPNSF21**	.010	.010	.010	.010	.010	.010	.010	.010								
**BPNSF2**	.010	.010	.010	.010	.010	.010	.010	.010	.010							
**BPNSF8**	.010	.010	.010	.010	.010	.010	.010	.010	.010	.010						
**BPNSF20**	.010	.010	.010	.010	.010	.010	.010	.010	.010	.010	.010					
**BPNSF22**	.010	.010	.010	.010	.010	.010	.010	.010	.010	.010	.010	.010				
**BPNSF4**	.010	.010	.010	.010	.010	.010	.010	.010	.010	.010	.010	.010	.010			
**BPNSF12**	.010	.010	.010	.010	.010	.010	.010	.010	.010	.010	.010	.010	.010	.010		
**BPNSF16**	.010	.010	.010	.010	.010	.010	.010	.010	.010	.010	.010	.010	.010	.010	.010	
**BPNSF24**	.010	.010	.010	.010	.010	.010	.010	.010	.010	.010	.010	.010	.010	.010	.010	.0
**BPNSF5**	.010	.010	.010	.010	.010	.010	.010	.010	.010	.010	.010	.010	.010	.010	.010	.0
**BPNSF10**	.010	.010	.010	.010	.010	.010	.010	.010	.010	.010	.010	.010	.010	.010	.010	.0
**BPNSF15**	.010	.010	.010	.010	.010	.010	.016	.080	.010	.010	.010	.010	.010	.066	.014	.0
**BPNSF18**	.334	.022	.010	.387	.401	.145	.010	.346	.207	.090	.074	.012	.149	.085	.301	.0
**BPNSF1**	.019	.010	.010	.010	.010	.010	.010	.010	.185	.010	.010	.010	.010	.010	.010	.0
**BPNSF7**	.188	.010	.010	.010	.010	.010	.010	.010	.052	.095	.010	.010	.010	.010	.010	.0
**BPNSF13**	.010	.010	.010	.010	.010	.010	.010	.010	.010	.010	.010	.010	.010	.010	.010	.0
**BPNSF19**	.010	.010	.010	.010	.010	.010	.010	.010	.019	.010	.010	.010	.010	.010	.010	.0

Sample Correlations (g3 - Unconstrained)

Sample Correlations - Lower Bounds (PC) (g3 - Unconstrained)

	BPNSF6	BPNSF11	BPNSF17	BPNSF23	BPNSF3	BPNSF9	BPNSF14	BPNSF21	BPNSF2	BPNSF8	BPNSF20	BPNSF22	BPNSF4	BPNSF12	BPNSF16	BPNS
**BPNSF6**	1.000															
**BPNSF11**	.471	1.000														
**BPNSF17**	.419	.502	1.000													
**BPNSF23**	.441	.542	.549	1.000												
**BPNSF3**	-.426	-.441	-.482	-.508	1.000											
**BPNSF9**	-.507	-.570	-.601	-.533	.425	1.000										
**BPNSF14**	-.382	-.472	-.526	-.496	.365	.551	1.000									
**BPNSF21**	-.349	-.475	-.371	-.484	.285	.389	.402	1.000								
**BPNSF2**	.290	.406	.347	.462	-.439	-.363	-.359	-.278	1.000							
**BPNSF8**	.387	.506	.435	.446	-.387	-.482	-.353	-.358	.463	1.000						
**BPNSF20**	.339	.440	.532	.576	-.442	-.497	-.401	-.387	.441	.496	1.000					
**BPNSF22**	.240	.386	.310	.445	-.382	-.366	-.320	-.377	.372	.481	.454	1.000				
**BPNSF4**	-.323	-.353	-.303	-.327	.449	.263	.185	.251	-.266	-.346	-.350	-.382	1.000			
**BPNSF12**	-.326	-.409	-.421	-.465	.306	.465	.484	.434	-.366	-.382	-.425	-.450	.376	1.000		
**BPNSF16**	-.304	-.419	-.438	-.403	.211	.336	.427	.402	-.329	-.376	-.461	-.430	.351	.591	1.000	
**BPNSF24**	-.342	-.437	-.344	-.430	.195	.307	.336	.447	-.374	-.418	-.412	-.360	.278	.473	.412	1.0
**BPNSF5**	.285	.374	.270	.302	-.242	-.291	-.247	-.221	.401	.377	.273	.305	-.286	-.308	-.321	-.2
**BPNSF10**	.361	.538	.416	.408	-.361	-.453	-.320	-.309	.382	.477	.369	.350	-.331	-.322	-.320	-.2
**BPNSF15**	.143	.240	.235	.145	-.225	-.243	-.223	-.193	.185	.287	.180	.207	-.283	-.190	-.221	-.2
**BPNSF18**	-.023	.031	.064	-.036	-.126	-.006	.039	-.025	-.021	.003	.007	.072	-.156	.004	-.039	.0
**BPNSF1**	-.229	-.257	-.233	-.223	.327	.207	.133	.179	-.159	-.248	-.234	-.331	.294	.211	.152	.1
**BPNSF7**	-.151	-.314	-.264	-.275	.141	.251	.286	.332	-.191	-.193	-.219	-.276	.149	.310	.252	.1
**BPNSF13**	-.279	-.378	-.348	-.298	.189	.385	.416	.360	-.253	-.305	-.319	-.377	.292	.559	.432	.3
**BPNSF19**	-.292	-.361	-.307	-.363	.227	.332	.338	.520	-.209	-.289	-.298	-.290	.266	.313	.348	.3

Sample Correlations - Upper Bounds (PC) (g3 - Unconstrained)

	BPNSF6	BPNSF11	BPNSF17	BPNSF23	BPNSF3	BPNSF9	BPNSF14	BPNSF21	BPNSF2	BPNSF8	BPNSF20	BPNSF22	BPNSF4	BPNSF12	BPNSF16	BPNS
**BPNSF6**	1.000															
**BPNSF11**	.622	1.000														
**BPNSF17**	.579	.644	1.000													
**BPNSF23**	.576	.652	.677	1.000												
**BPNSF3**	-.258	-.268	-.304	-.327	1.000											
**BPNSF9**	-.325	-.438	-.444	-.380	.613	1.000										
**BPNSF14**	-.222	-.326	-.344	-.346	.529	.687	1.000									

	BPNSF6	BPNSF11	BPNSF17	BPNSF23	BPNSF3	BPNSF9	BPNSF14	BPNSF21	BPNSF2	BPNSF8	BPNSF20	BPNSF22	BPNSF4	BPNSF12	BPNSF16	BPNS[illegible]
**BPNSF21**	-.193	-.308	-.188	-.332	.475	.562	.592	1.000								
**BPNSF2**	.455	.551	.515	.602	-.259	-.201	-.190	-.116	1.000							
**BPNSF8**	.523	.645	.575	.584	-.210	-.320	-.184	-.176	.617	1.000						
**BPNSF20**	.495	.580	.661	.701	-.276	-.337	-.242	-.199	.590	.640	1.000					
**BPNSF22**	.406	.519	.484	.585	-.205	-.172	-.121	-.195	.506	.614	.601	1.000				
**BPNSF4**	-.156	-.175	-.130	-.190	.627	.438	.370	.450	-.078	-.167	-.175	-.189	1.000			
**BPNSF12**	-.172	-.250	-.264	-.299	.470	.607	.630	.569	-.202	-.198	-.251	-.284	.537	1.000		
**BPNSF16**	-.133	-.263	-.278	-.255	.406	.532	.575	.571	-.173	-.177	-.284	-.256	.516	.718	1.000	
**BPNSF24**	-.194	-.296	-.171	-.283	.387	.484	.498	.593	-.205	-.243	-.255	-.177	.475	.613	.571	1.0[illegible]
**BPNSF5**	.434	.491	.410	.428	-.061	-.139	-.085	-.049	.550	.511	.434	.463	-.115	-.156	-.166	-.1[illegible]
**BPNSF10**	.539	.659	.561	.541	-.190	-.296	-.156	-.146	.534	.615	.511	.508	-.146	-.173	-.163	-.1[illegible]
**BPNSF15**	.332	.383	.405	.298	-.058	-.084	-.052	-.003	.332	.436	.339	.368	-.127	-.012	-.057	-.0[illegible]
**BPNSF18**	.132	.194	.229	.116	.042	.159	.212	.138	.152	.178	.174	.231	.011	.182	.143	.2[illegible]
**BPNSF1**	-.048	-.093	-.051	-.067	.497	.378	.315	.377	.012	-.066	-.071	-.154	.465	.382	.337	.3[illegible]
**BPNSF7**	.023	-.152	-.089	-.121	.361	.442	.469	.505	-.014	-.003	-.057	-.086	.346	.473	.429	.3[illegible]
**BPNSF13**	-.102	-.215	-.192	-.137	.387	.538	.587	.522	-.091	-.103	-.141	-.205	.457	.678	.584	.4[illegible]
**BPNSF19**	-.152	-.222	-.145	-.224	.393	.494	.522	.639	-.060	-.118	-.123	-.126	.421	.491	.503	.4[illegible]

Sample Correlations - Two Tailed Significance (PC) (g3 - Unconstrained)

	BPNSF6	BPNSF11	BPNSF17	BPNSF23	BPNSF3	BPNSF9	BPNSF14	BPNSF21	BPNSF2	BPNSF8	BPNSF20	BPNSF22	BPNSF4	BPNSF12	BPNSF16	BPNS[illegible]
**BPNSF6**	...															
**BPNSF11**	.010	...														
**BPNSF17**	.010	.010	...													
**BPNSF23**	.010	.010	.010	...												
**BPNSF3**	.010	.010	.010	.010	...											
**BPNSF9**	.010	.010	.010	.010	.010	...										
**BPNSF14**	.010	.010	.010	.010	.010	.010	...									
**BPNSF21**	.010	.010	.010	.010	.010	.010	.010	...								
**BPNSF2**	.010	.010	.010	.010	.010	.010	.010	.010	...							
**BPNSF8**	.010	.010	.010	.010	.010	.010	.010	.010	.010	...						
**BPNSF20**	.010	.010	.010	.010	.010	.010	.010	.010	.010	.010	...					
**BPNSF22**	.010	.010	.010	.010	.010	.010	.010	.010	.010	.010	.010	...				
**BPNSF4**	.010	.010	.010	.010	.010	.010	.010	.010	.010	.010	.010	.010	...			
**BPNSF12**	.010	.010	.010	.010	.010	.010	.010	.010	.010	.010	.010	.010	.010	...		
**BPNSF16**	.010	.010	.010	.010	.010	.010	.010	.010	.010	.010	.010	.010	.010	.010	...	
**BPNSF24**	.010	.010	.010	.010	.010	.010	.010	.010	.010	.010	.010	.010	.010	.010	.010	
**BPNSF5**	.010	.010	.010	.010	.010	.010	.010	.010	.010	.010	.010	.010	.010	.010	.010	.0[illegible]
**BPNSF10**	.010	.010	.010	.010	.010	.010	.010	.010	.010	.010	.010	.010	.010	.010	.010	.0[illegible]
**BPNSF15**	.010	.010	.010	.010	.010	.010	.016	.080	.010	.010	.010	.010	.010	.066	.014	.0[illegible]
**BPNSF18**	.334	.022	.010	.388	.401	.145	.010	.346	.207	.090	.074	.012	.149	.085	.301	.0[illegible]
**BPNSF1**	.019	.010	.010	.010	.010	.010	.010	.010	.185	.010	.010	.010	.010	.010	.010	.0[illegible]
**BPNSF7**	.188	.010	.010	.010	.010	.010	.010	.010	.052	.095	.010	.010	.010	.010	.010	.0[illegible]
**BPNSF13**	.010	.010	.010	.010	.010	.010	.010	.010	.010	.010	.010	.010	.010	.010	.010	.0[illegible]
**BPNSF19**	.010	.010	.010	.010	.010	.010	.010	.010	.019	.010	.010	.010	.010	.010	.010	.0[illegible]

Sample Means (g3 - Unconstrained)

Sample Means - Lower Bounds (PC) (g3 - Unconstrained)

	BPNSF6	BPNSF11	BPNSF17	BPNSF23	BPNSF3	BPNSF9	BPNSF14	BPNSF21	BPNSF2	BPNSF8	BPNSF20	BPNSF22	BPNSF4	BPNSF12	BPNSF16	BPNSF[illegible]
**BPNSF6**	2.499	2.681	2.685	2.385	5.375	5.333	5.051	4.893	2.510	2.779	2.601	3.135	5.093	4.999	4.900	5.00[illegible]

Sample Means - Upper Bounds (PC) (g3 - Unconstrained)

	BPNSF6	BPNSF11	BPNSF17	BPNSF23	BPNSF3	BPNSF9	BPNSF14	BPNSF21	BPNSF2	BPNSF8	BPNSF20	BPNSF22	BPNSF4	BPNSF12	BPNSF16	BPNSF[illegible]
**BPNSF6**	2.772	2.931	2.973	2.629	5.607	5.592	5.295	5.097	2.809	3.059	2.890	3.388	5.300	5.235	5.142	5.23[illegible]

Sample Means - Two Tailed Significance (PC) (g3 - Unconstrained)

	BPNSF6	BPNSF11	BPNSF17	BPNSF23	BPNSF3	BPNSF9	BPNSF14	BPNSF21	BPNSF2	BPNSF8	BPNSF20	BPNSF22	BPNSF4	BPNSF12	BPNSF16	BPNSF[illegible]
**BPNSF6**	.010	.010	.010	.010	.010	.010	.010	.010	.010	.010	.010	.010	.010	.010	.010	.01[illegible]

Bias-corrected percentile method (g3 - Unconstrained)

90% confidence intervals (bias-corrected percentile method)

Scalar Estimates (g3 - Unconstrained)

Regression Weights: (g3 - Unconstrained)

Parameter			Estimate	Lower	Upper	P
BPNSF19	<---	F1	1.000	1.000	1.000	...
BPNSF13	<---	F1	1.183	1.020	1.447	.004
BPNSF7	<---	F1	.892	.713	1.063	.012
BPNSF1	<---	F1	.837	.618	1.130	.008
BPNSF18	<---	F2	1.000	1.000	1.000	...
BPNSF15	<---	F2	2.171	1.627	3.797	.008
BPNSF10	<---	F2	3.437	2.425	6.144	.009
BPNSF5	<---	F2	2.917	2.223	5.246	.008
BPNSF24	<---	F3	1.000	1.000	1.000	...
BPNSF16	<---	F3	1.189	1.043	1.456	.003
BPNSF12	<---	F3	1.368	1.232	1.604	.004
BPNSF4	<---	F3	.849	.684	.997	.012
BPNSF22	<---	F4	1.000	1.000	1.000	...
BPNSF20	<---	F4	1.270	1.126	1.432	.010
BPNSF8	<---	F4	1.319	1.170	1.474	.008
BPNSF2	<---	F4	1.184	1.034	1.375	.005
BPNSF21	<---	F5	1.000	1.000	1.000	...
BPNSF14	<---	F5	1.164	1.010	1.332	.015
BPNSF9	<---	F5	1.195	1.033	1.439	.007
BPNSF3	<---	F5	.927	.719	1.104	.015
BPNSF23	<---	F6	1.000	1.000	1.000	...
BPNSF17	<---	F6	.995	.902	1.080	.011
BPNSF11	<---	F6	.988	.885	1.113	.005
BPNSF6	<---	F6	.832	.721	.935	.012

Standardized Regression Weights: (g3 - Unconstrained)

Parameter			Estimate	Lower	Upper	P
BPNSF19	<---	F1	.628	.533	.700	.018
BPNSF13	<---	F1	.727	.671	.781	.007
BPNSF7	<---	F1	.545	.455	.614	.021
BPNSF1	<---	F1	.467	.372	.574	.009
BPNSF18	<---	F2	.256	.143	.342	.014
BPNSF15	<---	F2	.487	.406	.557	.009
BPNSF10	<---	F2	.779	.720	.829	.014
BPNSF5	<---	F2	.648	.580	.714	.009
BPNSF24	<---	F3	.652	.575	.716	.013
BPNSF16	<---	F3	.763	.681	.811	.018
BPNSF12	<---	F3	.842	.804	.878	.007
BPNSF4	<---	F3	.576	.469	.648	.015
BPNSF22	<---	F4	.669	.607	.725	.019
BPNSF20	<---	F4	.752	.680	.806	.015
BPNSF8	<---	F4	.780	.727	.830	.007
BPNSF2	<---	F4	.693	.625	.752	.011
BPNSF21	<---	F5	.686	.590	.753	.019
BPNSF14	<---	F5	.743	.673	.802	.018
BPNSF9	<---	F5	.768	.712	.833	.012
BPNSF3	<---	F5	.598	.469	.685	.023
BPNSF23	<---	F6	.784	.740	.830	.011
BPNSF17	<---	F6	.758	.703	.806	.015
BPNSF11	<---	F6	.789	.733	.831	.010
BPNSF6	<---	F6	.653	.577	.718	.010

Intercepts: (g3 - Unconstrained)

Parameter	Estimate	Lower	Upper	P
**BPNSF19**	4.926	4.803	5.056	.009
**BPNSF13**	4.783	4.686	4.896	.007
**BPNSF7**	4.717	4.582	4.828	.015
**BPNSF1**	4.672	4.526	4.792	.013
**BPNSF18**	4.474	4.362	4.602	.010
**BPNSF15**	3.730	3.595	3.844	.014
**BPNSF10**	3.118	2.984	3.240	.011
**BPNSF5**	3.901	3.786	4.070	.004
**BPNSF24**	5.117	4.976	5.216	.015
**BPNSF16**	5.019	4.898	5.123	.018
**BPNSF12**	5.107	4.987	5.209	.023
**BPNSF4**	5.204	5.093	5.299	.010
**BPNSF22**	3.274	3.133	3.386	.012

Parameter	Estimate	Lower	Upper	P
**BPNSF20**	2.762	2.645	2.915	.004
**BPNSF8**	2.916	2.760	3.041	.023
**BPNSF2**	2.644	2.510	2.798	.012
**BPNSF21**	4.999	4.869	5.078	.021
**BPNSF14**	5.183	5.059	5.311	.007
**BPNSF9**	5.461	5.326	5.575	.018
**BPNSF3**	5.491	5.383	5.614	.008
**BPNSF23**	2.503	2.387	2.631	.008
**BPNSF17**	2.828	2.701	3.021	.004
**BPNSF11**	2.806	2.674	2.911	.015
**BPNSF6**	2.632	2.485	2.760	.019

Covariances: (g3 - Unconstrained)

Parameter	Estimate	Lower	Upper	P
F1 <--> F2	-.158	-.222	-.093	.006
F2 <--> F3	-.135	-.199	-.091	.003
F1 <--> F3	.700	.556	.843	.010
F2 <--> F4	.331	.208	.482	.004
F3 <--> F4	-.510	-.665	-.376	.011
F1 <--> F4	-.368	-.489	-.248	.010
F2 <--> F5	-.158	-.214	-.101	.004
F4 <--> F5	-.581	-.743	-.470	.007
F3 <--> F5	.718	.568	.916	.009
F1 <--> F5	.746	.556	.959	.009
F6 <--> F5	-.980	-1.130	-.805	.011
F6 <--> F3	-.674	-.832	-.526	.010
F6 <--> F4	1.304	1.079	1.488	.016
F6 <--> F2	.417	.265	.579	.005
F6 <--> F1	-.567	-.698	-.416	.013

Correlations: (g3 - Unconstrained)

Parameter	Estimate	Lower	Upper	P
F1 <--> F2	-.478	-.590	-.389	.006
F2 <--> F3	-.403	-.498	-.306	.013
F1 <--> F3	.895	.834	.952	.006
F2 <--> F4	.838	.769	.900	.010
F3 <--> F4	-.545	-.641	-.444	.015
F1 <--> F4	-.398	-.508	-.291	.013
F2 <--> F5	-.444	-.540	-.335	.009
F4 <--> F5	-.583	-.673	-.490	.009
F3 <--> F5	.852	.772	.917	.009
F1 <--> F5	.897	.815	.980	.006
F6 <--> F5	-.759	-.825	-.704	.011
F6 <--> F3	-.556	-.627	-.471	.012
F6 <--> F4	.909	.849	.957	.007
F6 <--> F2	.815	.733	.883	.012
F6 <--> F1	-.474	-.551	-.393	.012

Variances: (g3 - Unconstrained)

Parameter	Estimate	Lower	Upper	P
**F1**	.771	.539	.959	.014
**F2**	.141	.047	.259	.011
**F3**	.792	.605	.970	.012
**F4**	1.106	.880	1.354	.015
**F5**	.897	.697	1.113	.012
**F6**	1.858	1.545	2.109	.014
**e1**	1.186	.954	1.404	.010
**e2**	.964	.808	1.162	.007
**e3**	1.455	1.227	1.837	.003
**e4**	1.934	1.645	2.248	.005
**e5**	2.020	1.825	2.312	.002
**e6**	2.135	1.871	2.373	.009
**e7**	1.082	.836	1.372	.009
**e8**	1.660	1.378	1.918	.012
**e9**	1.074	.873	1.286	.007
**e10**	.806	.650	1.040	.003
**e11**	.608	.488	.761	.007

Parameter	Estimate	Lower	Upper	P
**e12**	1.151	.935	1.493	.007
**e13**	1.362	1.159	1.581	.005
**e14**	1.372	1.071	1.728	.004
**e15**	1.238	.955	1.494	.016
**e16**	1.681	1.341	2.092	.011
**e17**	1.008	.814	1.268	.003
**e18**	.984	.781	1.280	.004
**e19**	.889	.679	1.085	.005
**e20**	1.386	1.078	1.800	.006
**e21**	1.166	.941	1.407	.007
**e22**	1.362	1.063	1.650	.007
**e23**	1.097	.878	1.317	.016
**e24**	1.726	1.383	2.082	.013

Matrices (g3 - Unconstrained)

Sample Covariances (g3 - Unconstrained)

Sample Covariances - Lower Bounds (BC) (g3 - Unconstrained)

	BPNSF6	BPNSF11	BPNSF17	BPNSF23	BPNSF3	BPNSF9	BPNSF14	BPNSF21	BPNSF2	BPNSF8	BPNSF20	BPNSF22	BPNSF4	BPNSF12	BPNSF16	BPNS[illegible]
**BPNSF6**	2.723															
**BPNSF11**	1.422	2.681														
**BPNSF17**	1.312	1.486	2.921													
**BPNSF23**	1.329	1.522	1.661	2.748												
**BPNSF3**	-1.115	-1.099	-1.232	-1.270	1.766											
**BPNSF9**	-1.282	-1.467	-1.621	-1.362	.896	1.820										
**BPNSF14**	-.945	-1.206	-1.410	-1.268	.781	1.114	1.981									
**BPNSF21**	-.845	-1.160	-.933	-1.145	.573	.730	.794	1.699								
**BPNSF2**	.910	1.236	1.146	1.385	-1.190	-.945	-.953	-.677	2.909							
**BPNSF8**	1.219	1.561	1.360	1.413	-1.039	-1.281	-.925	-.914	1.437	2.888						
**BPNSF20**	1.017	1.291	1.640	1.695	-1.134	-1.307	-1.080	-.921	1.355	1.506	2.843					
**BPNSF22**	.666	.996	.858	1.234	-.890	-.895	-.747	-.843	1.036	1.326	1.228	2.241				
**BPNSF4**	-.764	-.758	-.708	-.796	.837	.525	.372	.460	-.626	-.833	-.831	-.779	1.528			
**BPNSF12**	-.905	-1.029	-1.115	-1.188	.664	.969	1.029	.846	-.960	-.968	-1.087	-1.015	.727	1.899		
**BPNSF16**	-.728	-1.007	-1.076	-.989	.471	.695	.888	.737	-.822	-.922	-1.166	-.962	.634	1.152	1.740	
**BPNSF24**	-.849	-1.004	-.818	-1.038	.370	.582	.630	.803	-.891	-1.023	-.983	-.771	.522	.921	.786	1.6[illegible]
**BPNSF5**	.817	1.064	.784	.876	-.588	-.731	-.659	-.508	1.217	1.082	.816	.751	-.679	-.787	-.775	-.7[illegible]
**BPNSF10**	1.046	1.513	1.237	1.180	-.878	-1.131	-.752	-.699	1.126	1.417	1.062	.909	-.711	-.766	-.716	-.6[illegible]
**BPNSF15**	.453	.682	.713	.443	-.551	-.624	-.584	-.518	.575	.795	.528	.532	-.645	-.483	-.531	-.4[illegible]
**BPNSF18**	-.051	.101	.207	-.064	-.319	-.009	.079	-.091	-.023	-.004	.030	.182	-.295	-.010	-.106	.1[illegible]
**BPNSF1**	-.604	-.629	-.641	-.593	.707	.503	.304	.387	-.476	-.774	-.609	-.851	.615	.478	.346	.3[illegible]
**BPNSF7**	-.368	-.742	-.668	-.713	.312	.517	.612	.630	-.496	-.493	-.535	-.670	.282	.659	.469	.3[illegible]
**BPNSF13**	-.695	-.898	-.931	-.740	.418	.811	.874	.746	-.638	-.792	-.808	-.888	.580	1.111	.847	.6[illegible]
**BPNSF19**	-.651	-.868	-.740	-.872	.464	.669	.700	.952	-.489	-.693	-.709	-.639	.464	.608	.673	.5[illegible]

Sample Covariances - Upper Bounds (BC) (g3 - Unconstrained)

	BPNSF6	BPNSF11	BPNSF17	BPNSF23	BPNSF3	BPNSF9	BPNSF14	BPNSF21	BPNSF2	BPNSF8	BPNSF20	BPNSF22	BPNSF4	BPNSF12	BPNSF16	BPNS[illegible]
**BPNSF6**	3.280															
**BPNSF11**	1.880	3.191														
**BPNSF17**	1.824	2.006	3.441													
**BPNSF23**	1.806	1.984	2.122	3.257												
**BPNSF3**	-.682	-.704	-.836	-.831	2.420											
**BPNSF9**	-.821	-1.073	-1.156	-.893	1.357	2.455										
**BPNSF14**	-.545	-.828	-.922	-.869	1.178	1.570	2.454									
**BPNSF21**	-.476	-.707	-.465	-.773	.974	1.117	1.160	2.175								
**BPNSF2**	1.453	1.740	1.684	1.858	-.752	-.542	-.502	-.325	3.527							
**BPNSF8**	1.693	2.047	1.869	1.878	-.552	-.795	-.496	-.453	2.002	3.431						
**BPNSF20**	1.534	1.780	2.157	2.198	-.670	-.848	-.622	-.461	1.904	2.033	3.447					
**BPNSF22**	1.136	1.440	1.358	1.699	-.457	-.414	-.298	-.456	1.516	1.781	1.716	2.708				
**BPNSF4**	-.389	-.358	-.311	-.438	1.288	.889	.729	.839	-.217	-.438	-.448	-.391	1.953			
**BPNSF12**	-.440	-.630	-.662	-.758	1.052	1.403	1.438	1.175	-.575	-.505	-.626	-.616	1.055	2.357		
**BPNSF16**	-.336	-.596	-.663	-.604	.867	1.104	1.255	1.105	-.455	-.459	-.682	-.557	.948	1.523	2.152	
**BPNSF24**	-.480	-.665	-.395	-.651	.772	.962	1.013	1.167	-.504	-.628	-.548	-.400	.861	1.254	1.107	2.0[illegible]
**BPNSF5**	1.289	1.497	1.257	1.288	-.147	-.367	-.247	-.110	1.719	1.562	1.339	1.222	-.287	-.376	-.414	-.3[illegible]
**BPNSF10**	1.573	2.051	1.684	1.600	-.461	-.711	-.355	-.333	1.639	1.912	1.546	1.354	-.311	-.412	-.323	-.3[illegible]
**BPNSF15**	1.001	1.160	1.259	.902	-.140	-.207	-.172	-.046	1.043	1.276	1.040	.972	-.279	-.086	-.152	-.1[illegible]
**BPNSF18**	.385	.497	.630	.339	.061	.350	.457	.241	.433	.449	.504	.573	.023	.371	.267	.4[illegible]
**BPNSF1**	-.121	-.200	-.144	-.180	1.181	.934	.758	.807	.014	-.225	-.161	-.383	.989	.868	.740	.6[illegible]

	BPNSF6	BPNSF11	BPNSF17	BPNSF23	BPNSF3	BPNSF9	BPNSF14	BPNSF21	BPNSF2	BPNSF8	BPNSF20	BPNSF22	BPNSF4	BPNSF12	BPNSF16	BPNS
**BPNSF7**	.070	-.342	-.227	-.322	.759	.925	.973	1.007	-.038	-.015	-.126	-.213	.658	1.019	.846	.6
**BPNSF13**	-.245	-.530	-.500	-.362	.836	1.193	1.270	1.084	-.232	-.310	-.358	-.458	.891	1.468	1.200	1.0
**BPNSF19**	-.268	-.528	-.325	-.543	.792	1.060	1.090	1.302	-.112	-.279	-.257	-.286	.786	1.006	1.003	.9

Sample Covariances - Two Tailed Significance (BC) (g3 - Unconstrained)

	BPNSF6	BPNSF11	BPNSF17	BPNSF23	BPNSF3	BPNSF9	BPNSF14	BPNSF21	BPNSF2	BPNSF8	BPNSF20	BPNSF22	BPNSF4	BPNSF12	BPNSF16	BPNS
**BPNSF6**	.015															
**BPNSF11**	.006	.006														
**BPNSF17**	.007	.012	.009													
**BPNSF23**	.005	.012	.009	.012												
**BPNSF3**	.006	.007	.009	.015	.016											
**BPNSF9**	.012	.009	.006	.021	.010	.012										
**BPNSF14**	.021	.008	.010	.018	.009	.011	.006									
**BPNSF21**	.007	.009	.010	.010	.011	.025	.019	.005								
**BPNSF2**	.009	.006	.007	.012	.007	.006	.011	.004	.008							
**BPNSF8**	.004	.004	.010	.003	.006	.011	.006	.005	.011	.008						
**BPNSF20**	.007	.013	.007	.012	.018	.012	.009	.018	.011	.010	.005					
**BPNSF22**	.009	.009	.012	.004	.013	.006	.005	.005	.005	.007	.009	.005				
**BPNSF4**	.007	.021	.008	.005	.010	.004	.009	.009	.005	.005	.004	.009	.009			
**BPNSF12**	.004	.005	.010	.005	.004	.004	.004	.006	.005	.007	.012	.009	.005	.003		
**BPNSF16**	.009	.010	.011	.007	.004	.011	.003	.009	.009	.009	.007	.008	.008	.005	.004	
**BPNSF24**	.006	.013	.021	.012	.015	.013	.015	.008	.011	.005	.030	.008	.008	.005	.006	.0
**BPNSF5**	.012	.005	.011	.007	.013	.008	.005	.012	.006	.014	.006	.019	.005	.008	.005	.0
**BPNSF10**	.006	.003	.010	.009	.012	.009	.021	.012	.007	.003	.007	.009	.012	.010	.016	.0
**BPNSF15**	.006	.007	.005	.005	.009	.008	.006	.038	.006	.013	.007	.010	.008	.039	.008	.0
**BPNSF18**	.275	.015	.006	.254	.243	.132	.009	.539	.165	.105	.053	.007	.157	.122	.419	.0
**BPNSF1**	.030	.020	.010	.012	.011	.005	.009	.012	.127	.005	.026	.006	.006	.007	.007	.0
**BPNSF7**	.224	.023	.014	.008	.011	.009	.013	.012	.044	.081	.018	.006	.011	.003	.013	.0
**BPNSF13**	.012	.009	.006	.007	.005	.004	.007	.004	.011	.005	.009	.006	.004	.004	.005	.0
**BPNSF19**	.036	.009	.019	.015	.011	.010	.008	.007	.041	.014	.020	.007	.009	.011	.008	.0

Sample Correlations (g3 - Unconstrained)

Sample Correlations - Lower Bounds (BC) (g3 - Unconstrained)

	BPNSF6	BPNSF11	BPNSF17	BPNSF23	BPNSF3	BPNSF9	BPNSF14	BPNSF21	BPNSF2	BPNSF8	BPNSF20	BPNSF22	BPNSF4	BPNSF12	BPNSF16	BPNS
**BPNSF6**	1.000															
**BPNSF11**	.481	1.000														
**BPNSF17**	.403	.497	1.000													
**BPNSF23**	.447	.541	.540	1.000												
**BPNSF3**	-.436	-.445	-.483	-.506	1.000											
**BPNSF9**	-.491	-.562	-.577	-.530	.401	1.000										
**BPNSF14**	-.363	-.470	-.520	-.482	.344	.551	1.000									
**BPNSF21**	-.344	-.470	-.371	-.481	.278	.375	.364	1.000								
**BPNSF2**	.291	.412	.344	.468	-.431	-.356	-.357	-.287	1.000							
**BPNSF8**	.401	.510	.435	.473	-.389	-.480	-.354	-.368	.463	1.000						
**BPNSF20**	.340	.434	.530	.561	-.433	-.490	-.395	-.373	.441	.496	1.000					
**BPNSF22**	.240	.393	.308	.446	-.379	-.364	-.332	-.384	.375	.488	.454	1.000				
**BPNSF4**	-.327	-.340	-.303	-.341	.443	.269	.193	.252	-.269	-.352	-.356	-.382	1.000			
**BPNSF12**	-.326	-.417	-.420	-.468	.316	.464	.485	.435	-.358	-.383	-.407	-.451	.378	1.000		
**BPNSF16**	-.302	-.418	-.431	-.412	.220	.334	.429	.396	-.332	-.367	-.455	-.438	.357	.590	1.000	
**BPNSF24**	-.353	-.426	-.338	-.428	.181	.304	.318	.446	-.372	-.421	-.397	-.361	.290	.472	.411	1.0
**BPNSF5**	.277	.383	.259	.304	-.238	-.291	-.257	-.227	.400	.375	.273	.297	-.287	-.307	-.326	-.2
**BPNSF10**	.356	.540	.408	.407	-.343	-.453	-.295	-.303	.382	.487	.367	.350	-.323	-.321	-.317	-.2
**BPNSF15**	.151	.239	.242	.152	-.221	-.239	-.229	-.226	.191	.288	.182	.207	-.288	-.193	-.221	-.2
**BPNSF18**	-.022	.040	.083	-.030	-.137	-.009	.034	-.049	-.007	-.003	.008	.075	-.152	-.008	-.050	.0
**BPNSF1**	-.218	-.236	-.229	-.212	.333	.205	.135	.176	-.164	-.267	-.218	-.343	.299	.216	.151	.1
**BPNSF7**	-.148	-.308	-.253	-.274	.132	.238	.285	.313	-.192	-.200	-.215	-.293	.149	.311	.218	.1
**BPNSF13**	-.281	-.378	-.350	-.305	.203	.394	.411	.360	-.253	-.316	-.308	-.388	.312	.563	.434	.3
**BPNSF19**	-.279	-.354	-.295	-.361	.222	.328	.334	.517	-.198	-.285	-.291	-.303	.268	.310	.331	.3

Sample Correlations - Upper Bounds (BC) (g3 - Unconstrained)

	BPNSF6	BPNSF11	BPNSF17	BPNSF23	BPNSF3	BPNSF9	BPNSF14	BPNSF21	BPNSF2	BPNSF8	BPNSF20	BPNSF22	BPNSF4	BPNSF12	BPNSF16	BPNS
**BPNSF6**	1.000															
**BPNSF11**	.625	1.000														
**BPNSF17**	.570	.644	1.000													
**BPNSF23**	.582	.650	.672	1.000												
**BPNSF3**	-.258	-.272	-.308	-.326	1.000											

	BPNSF6	BPNSF11	BPNSF17	BPNSF23	BPNSF3	BPNSF9	BPNSF14	BPNSF21	BPNSF2	BPNSF8	BPNSF20	BPNSF22	BPNSF4	BPNSF12	BPNSF16	BPNS
**BPNSF9**	-.319	-.432	-.431	-.373	.594	1.000										
**BPNSF14**	-.209	-.324	-.340	-.342	.516	.687	1.000									
**BPNSF21**	-.185	-.306	-.188	-.329	.464	.554	.559	1.000								
**BPNSF2**	.458	.555	.512	.605	-.252	-.195	-.184	-.125	1.000							
**BPNSF8**	.544	.646	.575	.612	-.213	-.319	-.185	-.178	.617	1.000						
**BPNSF20**	.497	.580	.660	.693	-.262	-.324	-.239	-.175	.590	.642	1.000					
**BPNSF22**	.406	.527	.483	.587	-.203	-.167	-.129	-.206	.507	.617	.600	1.000				
**BPNSF4**	-.160	-.163	-.127	-.192	.616	.443	.378	.454	-.085	-.184	-.185	-.189	1.000			
**BPNSF12**	-.174	-.253	-.261	-.301	.477	.602	.631	.570	-.198	-.198	-.244	-.284	.538	1.000		
**BPNSF16**	-.133	-.263	-.272	-.257	.417	.530	.575	.564	-.179	-.175	-.284	-.262	.523	.718	1.000	
**BPNSF24**	-.208	-.289	-.165	-.281	.373	.481	.493	.589	-.203	-.250	-.221	-.189	.487	.613	.571	1.0
**BPNSF5**	.429	.507	.397	.431	-.056	-.142	-.094	-.053	.547	.509	.437	.459	-.116	-.155	-.175	-.1
**BPNSF10**	.532	.660	.559	.540	-.180	-.296	-.125	-.129	.534	.620	.508	.507	-.134	-.168	-.151	-.1
**BPNSF15**	.339	.381	.410	.302	-.055	-.075	-.073	-.018	.346	.436	.346	.357	-.130	-.035	-.060	-.0
**BPNSF18**	.136	.202	.240	.129	.033	.157	.211	.117	.170	.170	.175	.244	.016	.166	.132	.2
**BPNSF1**	-.040	-.076	-.049	-.064	.501	.378	.323	.371	.006	-.076	-.056	-.162	.469	.387	.337	.3
**BPNSF7**	.030	-.141	-.086	-.119	.353	.432	.468	.481	-.015	-.006	-.055	-.095	.346	.480	.418	.3
**BPNSF13**	-.105	-.215	-.197	-.144	.397	.546	.576	.522	-.091	-.116	-.126	-.207	.459	.681	.588	.5
**BPNSF19**	-.120	-.218	-.123	-.218	.386	.493	.518	.639	-.043	-.117	-.102	-.137	.421	.479	.497	.4

Sample Correlations - Two Tailed Significance (BC) (g3 - Unconstrained)

	BPNSF6	BPNSF11	BPNSF17	BPNSF23	BPNSF3	BPNSF9	BPNSF14	BPNSF21	BPNSF2	BPNSF8	BPNSF20	BPNSF22	BPNSF4	BPNSF12	BPNSF16	BPNS
**BPNSF6**	...															
**BPNSF11**	.007	...														
**BPNSF17**	.018	.011	...													
**BPNSF23**	.006	.011	.013	...												
**BPNSF3**	.008	.009	.007	.012	...											
**BPNSF9**	.018	.016	.021	.015	.023	...										
**BPNSF14**	.026	.011	.014	.021	.018	.009	...									
**BPNSF21**	.015	.015	.010	.014	.015	.019	.028	...								
**BPNSF2**	.009	.007	.013	.007	.014	.014	.013	.006	...							
**BPNSF8**	.003	.006	.010	.002	.008	.011	.008	.007	.010	...						
**BPNSF20**	.009	.013	.013	.016	.019	.021	.012	.026	.010	.009	...					
**BPNSF22**	.010	.005	.012	.009	.012	.012	.006	.006	.007	.007	.012	...				
**BPNSF4**	.009	.019	.011	.006	.014	.007	.008	.009	.007	.005	.006	.010	...			
**BPNSF12**	.009	.007	.011	.007	.005	.012	.009	.009	.015	.009	.019	.009	.008	...		
**BPNSF16**	.011	.011	.015	.007	.007	.012	.009	.013	.008	.012	.011	.007	.007	.011	...	
**BPNSF24**	.004	.018	.015	.012	.016	.012	.015	.012	.012	.008	.034	.009	.007	.011	.012	
**BPNSF5**	.013	.003	.019	.007	.013	.009	.006	.008	.012	.015	.009	.014	.009	.012	.005	.0
**BPNSF10**	.014	.009	.013	.012	.025	.010	.034	.016	.010	.005	.012	.011	.018	.012	.012	.0
**BPNSF15**	.007	.012	.006	.007	.011	.012	.006	.040	.006	.009	.009	.012	.008	.041	.012	.0
**BPNSF18**	.298	.015	.005	.276	.275	.183	.012	.556	.150	.110	.070	.010	.172	.148	.419	.0
**BPNSF1**	.034	.020	.012	.014	.007	.012	.008	.014	.140	.007	.032	.005	.007	.007	.011	.0
**BPNSF7**	.234	.018	.020	.012	.018	.016	.012	.030	.044	.077	.015	.006	.010	.007	.018	.0
**BPNSF13**	.009	.010	.008	.008	.006	.005	.016	.010	.010	.006	.018	.007	.005	.008	.006	.0
**BPNSF19**	.028	.014	.025	.012	.013	.012	.012	.012	.041	.012	.020	.005	.009	.016	.019	.0

Sample Means (g3 - Unconstrained)

Sample Means - Lower Bounds (BC) (g3 - Unconstrained)

	BPNSF6	BPNSF11	BPNSF17	BPNSF23	BPNSF3	BPNSF9	BPNSF14	BPNSF21	BPNSF2	BPNSF8	BPNSF20	BPNSF22	BPNSF4	BPNSF12	BPNSF16	BPNSF
**BPNSF6**	2.485	2.674	2.701	2.386	5.382	5.326	5.059	4.869	2.510	2.760	2.645	3.133	5.092	4.987	4.896	4.97

Sample Means - Upper Bounds (BC) (g3 - Unconstrained)

	BPNSF6	BPNSF11	BPNSF17	BPNSF23	BPNSF3	BPNSF9	BPNSF14	BPNSF21	BPNSF2	BPNSF8	BPNSF20	BPNSF22	BPNSF4	BPNSF12	BPNSF16	BPNSF
**BPNSF6**	2.760	2.911	3.021	2.631	5.608	5.577	5.311	5.078	2.798	3.041	2.915	3.386	5.299	5.209	5.123	5.21

Sample Means - Two Tailed Significance (BC) (g3 - Unconstrained)

	BPNSF6	BPNSF11	BPNSF17	BPNSF23	BPNSF3	BPNSF9	BPNSF14	BPNSF21	BPNSF2	BPNSF8	BPNSF20	BPNSF22	BPNSF4	BPNSF12	BPNSF16	BPNSF
**BPNSF6**	.019	.015	.004	.009	.009	.016	.007	.021	.012	.023	.004	.012	.011	.023	.018	.01

Minimization History (Unconstrained)

Iteration	Negative eigenvalues	Condition #	Smallest eigenvalue	Diameter	F	NTries	Ratio

Iteration		Negative eigenvalues	Condition #	Smallest eigenvalue	Diameter	F	NTries	Ratio
0	e	41		-4.542	9999.000	104470.396	0	9999.000
1	e	38		-.654	3.956	38479.116	18	.444
2	e	30		-.207	4.055	17199.698	6	.607
3	e*	14		-.033	1.493	11233.180	5	1.022
4	e	9		-.025	.970	8790.221	4	.939
5	e	4		-.046	2.415	5774.330	7	.828
6	e	4		-.010	2.158	4103.697	5	.855
7	e*	3		-.005	3.322	2779.516	5	.896
8	e	0	612.873		1.646	2395.231	4	.794
9	e	0	1522.939		1.366	2295.836	1	1.000
10	e	0	3534.857		.952	2263.393	1	1.133
11	e	0	21490.494		.997	2258.085	1	.499
12	e	0	41342.218		1.073	2250.138	1	.692
13	e	0	108593.224		.655	2244.136	1	1.024
14	e	0	91149.878		.651	2243.499	2	.000
15	e	0	342346.456		.433	2242.926	1	1.197
16	e	0	294931.582		.794	2242.870	1	.250
17	e	0	1512801.304		.239	2242.592	1	1.036
18	e	0	1144446.542		.496	2242.577	1	.458
19	e	0	2890561.979		.063	2242.555	1	1.006
20	e	0	2958076.917		.037	2242.555	1	1.004
21	e	0	2992440.598		.000	2242.555	1	1.000

Pairwise Parameter Comparisons (Unconstrained)

Variance-covariance Matrix of Estimates (Unconstrained)

	a1_1	a2_1	a3_1	a4_1	a5_1	a6_1	a7_1	a8_1	a9_1	a10_1	a11_1	a12_1	a13_1	a14_1	a15_1	a16_1	a17_1	a18_1	ccc1_1	ccc2_1
**a1_1**	.007																			
**a2_1**	.003	.007																		
**a3_1**	.002	.002	.007																	
**a4_1**	.000	.000	.000	3.514																
**a5_1**	.000	.000	.000	4.118	5.160															
**a6_1**	.000	.000	.000	3.182	3.876	3.124														
**a7_1**	.000	.000	.000	.000	.000	.000	.007													
**a8_1**	.000	.000	.000	.000	.000	.000	.004	.007												
**a9_1**	.000	.000	.000	.000	.000	.000	.003	.003	.006											
**a10_1**	.000	.000	.000	.000	.000	.000	.000	.000	.000	.008										
**a11_1**	.000	.000	.000	.000	.000	.000	.000	.000	.000	.005	.009									
**a12_1**	.000	.000	.000	.000	.000	.000	.000	.000	.000	.005	.005	.009								
**a13_1**	.000	.000	.000	.000	.000	.000	.000	.000	.000	.000	.000	.000	.006							
**a14_1**	.000	.000	.000	.000	.000	.000	.000	.000	.000	.000	.000	.000	.003	.005						
**a15_1**	.000	.000	.000	.000	.000	.000	.000	.000	.000	.000	.000	.000	.003	.002	.005					
**a16_1**	.000	.000	.000	.000	.000	.000	.000	.000	.000	.000	.000	.000	.000	.000	.000	.004				
**a17_1**	.000	.000	.000	.000	.000	.000	.000	.000	.000	.000	.000	.000	.000	.000	.000	.002	.004			
**a18_1**	.000	.000	.000	.000	.000	.000	.000	.000	.000	.000	.000	.000	.000	.000	.000	.001	.002	.004		
**ccc1_1**	.000	.000	.000	.034	.041	.032	.000	.000	.000	.000	.000	.000	.000	.000	.000	.000	.000	.000	.001	
**ccc2_1**	.000	.000	.000	.043	.052	.040	.000	.000	.000	.000	.000	.000	.000	.000	.000	.000	.000	.000	.001	.001
**ccc3_1**	-.002	-.002	-.001	.000	.000	.000	-.002	-.002	-.001	.000	.000	.000	.000	.000	.000	.000	.000	.000	.000	.000
**ccc4_1**	.000	.000	.000	-.083	-.101	-.078	.000	.000	.000	.000	.000	.000	.000	.000	.000	.000	.000	.000	-.001	-.001
**ccc5_1**	.000	.000	.000	.000	.000	.000	.002	.002	.001	.002	.002	.002	.000	.000	.000	.000	.000	.000	.000	.000
**ccc6_1**	.002	.001	.001	.000	.000	.000	.000	.000	.000	.002	.002	.002	.000	.000	.000	.000	.000	.000	.000	.000
**ccc7_1**	.000	.000	.000	.026	.032	.025	.000	.000	.000	.000	.000	.000	.000	.000	.000	.000	.000	.000	.000	.000
**ccc8_1**	.000	.000	.000	.000	.000	.000	.000	.000	.000	.002	.003	.003	.002	.002	.001	.000	.000	.000	.000	.000
**ccc9_1**	.000	.000	.000	.000	.000	.000	-.002	-.002	-.001	.000	.000	.000	-.002	-.002	-.002	.000	.000	.000	.000	.000
**ccc10_1**	-.004	-.003	-.002	.000	.000	.000	.000	.000	.000	.000	.000	.000	-.003	-.002	-.002	.000	.000	.000	.000	.000
**ccc11_1**	.000	.000	.000	.000	.000	.000	.000	.000	.000	.000	.000	.000	.003	.002	.002	.001	.001	.001	.000	.000
**ccc12_1**	.000	.000	.000	.000	.000	.000	.002	.002	.001	.000	.000	.000	.000	.000	.000	.001	.001	.001	.000	.000
**ccc13_1**	.000	.000	.000	.000	.000	.000	.000	.000	.000	-.004	-.005	-.005	.000	.000	.000	-.002	-.002	-.002	.000	.000
**ccc14_1**	.000	.000	.000	-.114	-.139	-.107	.000	.000	.000	.000	.000	.000	.000	.000	.000	.000	.000	.000	-.001	-.002
**ccc15_1**	.002	.002	.001	.000	.000	.000	.000	.000	.000	.000	.000	.000	.000	.000	.000	.001	.001	.001	.001	.000
**vvv1_1**	-.007	-.005	-.004	.000	.000	.000	.000	.000	.000	.000	.000	.000	.000	.000	.000	.000	.000	.000	.000	.000
**v1_1**	.001	.001	.001	.000	.000	.000	.000	.000	.000	.000	.000	.000	.000	.000	.000	.000	.000	.000	.000	.000
**v2_1**	-.001	.000	.000	.000	.000	.000	.000	.000	.000	.000	.000	.000	.000	.000	.000	.000	.000	.000	.000	.000
**v3_1**	.000	-.001	.000	.000	.000	.000	.000	.000	.000	.000	.000	.000	.000	.000	.000	.000	.000	.000	.000	.000
**v4_1**	.000	.000	-.001	.000	.000	.000	.000	.000	.000	.000	.000	.000	.000	.000	.000	.000	.000	.000	.000	.000
**vvv2_1**	.000	.000	.000	-.060	-.073	-.056	.000	.000	.000	.000	.000	.000	.000	.000	.000	.000	.000	.000	-.001	-.001
**v5_1**	.000	.000	.000	.015	.019	.014	.000	.000	.000	.000	.000	.000	.000	.000	.000	.000	.000	.000	.000	.000
**v6_1**	.000	.000	.000	-.017	.003	.001	.000	.000	.000	.000	.000	.000	.000	.000	.000	.000	.000	.000	.000	.000
**v7_1**	.000	.000	.000	.002	-.028	.001	.000	.000	.000	.000	.000	.000	.000	.000	.000	.000	.000	.000	.000	.000

	a1_1	a2_1	a3_1	a4_1	a5_1	a6_1	a7_1	a8_1	a9_1	a10_1	a11_1	a12_1	a13_1	a14_1	a15_1	a16_1	a17_1	a18_1	ccc1_1	ccc2_1
**v8_1**	.000	.000	.000	.001	.002	-.015	.000	.000	.000	.000	.000	.000	.000	.000	.000	.000	.000	.000	.000	.000
**vvv3_1**	.000	.000	.000	.000	.000	.000	-.005	-.006	-.004	.000	.000	.000	.000	.000	.000	.000	.000	.000	.000	.000
**v9_1**	.000	.000	.000	.000	.000	.000	.001	.001	.001	.000	.000	.000	.000	.000	.000	.000	.000	.000	.000	.000
**v10_1**	.000	.000	.000	.000	.000	.000	-.001	.000	.000	.000	.000	.000	.000	.000	.000	.000	.000	.000	.000	.000
**v11_1**	.000	.000	.000	.000	.000	.000	.000	-.001	.000	.000	.000	.000	.000	.000	.000	.000	.000	.000	.000	.000
**v12_1**	.000	.000	.000	.000	.000	.000	.000	.000	-.001	.000	.000	.000	.000	.000	.000	.000	.000	.000	.000	.000
**vvv4_1**	.000	.000	.000	.000	.000	.000	.000	.000	.000	-.007	-.008	-.008	.000	.000	.000	.000	.000	.000	.000	.000
**v13_1**	.000	.000	.000	.000	.000	.000	.000	.000	.000	.001	.001	.001	.000	.000	.000	.000	.000	.000	.000	.000
**v14_1**	.000	.000	.000	.000	.000	.000	.000	.000	.000	-.001	.000	.000	.000	.000	.000	.000	.000	.000	.000	.000
**v15_1**	.000	.000	.000	.000	.000	.000	.000	.000	.000	.000	-.001	.000	.000	.000	.000	.000	.000	.000	.000	.000
**v16_1**	.000	.000	.000	.000	.000	.000	.000	.000	.000	.000	.000	-.001	.000	.000	.000	.000	.000	.000	.000	.000
**vvv5_1**	.000	.000	.000	.000	.000	.000	.000	.000	.000	.000	.000	.000	-.006	-.005	-.005	.000	.000	.000	.000	.000
**v17_1**	.000	.000	.000	.000	.000	.000	.000	.000	.000	.000	.000	.000	.001	.001	.000	.000	.000	.000	.000	.000
**v18_1**	.000	.000	.000	.000	.000	.000	.000	.000	.000	.000	.000	.000	-.001	.000	.000	.000	.000	.000	.000	.000
**v19_1**	.000	.000	.000	.000	.000	.000	.000	.000	.000	.000	.000	.000	.000	-.001	.000	.000	.000	.000	.000	.000
**v20_1**	.000	.000	.000	.000	.000	.000	.000	.000	.000	.000	.000	.000	.000	.000	-.001	.000	.000	.000	.000	.000
**vvv6_1**	.000	.000	.000	.000	.000	.000	.000	.000	.000	.000	.000	.000	.000	.000	.000	-.004	-.005	-.005	.000	.000
**v21_1**	.000	.000	.000	.000	.000	.000	.000	.000	.000	.000	.000	.000	.000	.000	.000	.001	.001	.001	.000	.000
**v22_1**	.000	.000	.000	.000	.000	.000	.000	.000	.000	.000	.000	.000	.000	.000	.000	-.001	.000	.000	.000	.000
**v23_1**	.000	.000	.000	.000	.000	.000	.000	.000	.000	.000	.000	.000	.000	.000	.000	.000	-.001	.000	.000	.000
**v24_1**	.000	.000	.000	.000	.000	.000	.000	.000	.000	.000	.000	.000	.000	.000	.000	.000	.000	-.001	.000	.000
**i1_1**	.000	.000	.000	.000	.000	.000	.000	.000	.000	.000	.000	.000	.000	.000	.000	.000	.000	.000	.000	.000
**i2_1**	.000	.000	.000	.000	.000	.000	.000	.000	.000	.000	.000	.000	.000	.000	.000	.000	.000	.000	.000	.000
**i3_1**	.000	.000	.000	.000	.000	.000	.000	.000	.000	.000	.000	.000	.000	.000	.000	.000	.000	.000	.000	.000
**i4_1**	.000	.000	.000	.000	.000	.000	.000	.000	.000	.000	.000	.000	.000	.000	.000	.000	.000	.000	.000	.000
**i5_1**	.000	.000	.000	.000	.000	.000	.000	.000	.000	.000	.000	.000	.000	.000	.000	.000	.000	.000	.000	.000
**i6_1**	.000	.000	.000	.000	.000	.000	.000	.000	.000	.000	.000	.000	.000	.000	.000	.000	.000	.000	.000	.000
**i7_1**	.000	.000	.000	.000	.000	.000	.000	.000	.000	.000	.000	.000	.000	.000	.000	.000	.000	.000	.000	.000
**i8_1**	.000	.000	.000	.000	.000	.000	.000	.000	.000	.000	.000	.000	.000	.000	.000	.000	.000	.000	.000	.000
**i9_1**	.000	.000	.000	.000	.000	.000	.000	.000	.000	.000	.000	.000	.000	.000	.000	.000	.000	.000	.000	.000
**i10_1**	.000	.000	.000	.000	.000	.000	.000	.000	.000	.000	.000	.000	.000	.000	.000	.000	.000	.000	.000	.000
**i11_1**	.000	.000	.000	.000	.000	.000	.000	.000	.000	.000	.000	.000	.000	.000	.000	.000	.000	.000	.000	.000
**i12_1**	.000	.000	.000	.000	.000	.000	.000	.000	.000	.000	.000	.000	.000	.000	.000	.000	.000	.000	.000	.000
**i13_1**	.000	.000	.000	.000	.000	.000	.000	.000	.000	.000	.000	.000	.000	.000	.000	.000	.000	.000	.000	.000
**i14_1**	.000	.000	.000	.000	.000	.000	.000	.000	.000	.000	.000	.000	.000	.000	.000	.000	.000	.000	.000	.000
**i15_1**	.000	.000	.000	.000	.000	.000	.000	.000	.000	.000	.000	.000	.000	.000	.000	.000	.000	.000	.000	.000
**i16_1**	.000	.000	.000	.000	.000	.000	.000	.000	.000	.000	.000	.000	.000	.000	.000	.000	.000	.000	.000	.000
**i17_1**	.000	.000	.000	.000	.000	.000	.000	.000	.000	.000	.000	.000	.000	.000	.000	.000	.000	.000	.000	.000
**i18_1**	.000	.000	.000	.000	.000	.000	.000	.000	.000	.000	.000	.000	.000	.000	.000	.000	.000	.000	.000	.000
**i19_1**	.000	.000	.000	.000	.000	.000	.000	.000	.000	.000	.000	.000	.000	.000	.000	.000	.000	.000	.000	.000
**i20_1**	.000	.000	.000	.000	.000	.000	.000	.000	.000	.000	.000	.000	.000	.000	.000	.000	.000	.000	.000	.000
**i21_1**	.000	.000	.000	.000	.000	.000	.000	.000	.000	.000	.000	.000	.000	.000	.000	.000	.000	.000	.000	.000
**i22_1**	.000	.000	.000	.000	.000	.000	.000	.000	.000	.000	.000	.000	.000	.000	.000	.000	.000	.000	.000	.000
**i23_1**	.000	.000	.000	.000	.000	.000	.000	.000	.000	.000	.000	.000	.000	.000	.000	.000	.000	.000	.000	.000
**i24_1**	.000	.000	.000	.000	.000	.000	.000	.000	.000	.000	.000	.000	.000	.000	.000	.000	.000	.000	.000	.000
**a1_2**	.000	.000	.000	.000	.000	.000	.000	.000	.000	.000	.000	.000	.000	.000	.000	.000	.000	.000	.000	.000
**a2_2**	.000	.000	.000	.000	.000	.000	.000	.000	.000	.000	.000	.000	.000	.000	.000	.000	.000	.000	.000	.000
**a3_2**	.000	.000	.000	.000	.000	.000	.000	.000	.000	.000	.000	.000	.000	.000	.000	.000	.000	.000	.000	.000
**a4_2**	.000	.000	.000	.000	.000	.000	.000	.000	.000	.000	.000	.000	.000	.000	.000	.000	.000	.000	.000	.000
**a5_2**	.000	.000	.000	.000	.000	.000	.000	.000	.000	.000	.000	.000	.000	.000	.000	.000	.000	.000	.000	.000
**a6_2**	.000	.000	.000	.000	.000	.000	.000	.000	.000	.000	.000	.000	.000	.000	.000	.000	.000	.000	.000	.000
**a7_2**	.000	.000	.000	.000	.000	.000	.000	.000	.000	.000	.000	.000	.000	.000	.000	.000	.000	.000	.000	.000
**a8_2**	.000	.000	.000	.000	.000	.000	.000	.000	.000	.000	.000	.000	.000	.000	.000	.000	.000	.000	.000	.000
**a9_2**	.000	.000	.000	.000	.000	.000	.000	.000	.000	.000	.000	.000	.000	.000	.000	.000	.000	.000	.000	.000
**a10_2**	.000	.000	.000	.000	.000	.000	.000	.000	.000	.000	.000	.000	.000	.000	.000	.000	.000	.000	.000	.000
**a11_2**	.000	.000	.000	.000	.000	.000	.000	.000	.000	.000	.000	.000	.000	.000	.000	.000	.000	.000	.000	.000
**a12_2**	.000	.000	.000	.000	.000	.000	.000	.000	.000	.000	.000	.000	.000	.000	.000	.000	.000	.000	.000	.000
**a13_2**	.000	.000	.000	.000	.000	.000	.000	.000	.000	.000	.000	.000	.000	.000	.000	.000	.000	.000	.000	.000
**a14_2**	.000	.000	.000	.000	.000	.000	.000	.000	.000	.000	.000	.000	.000	.000	.000	.000	.000	.000	.000	.000
**a15_2**	.000	.000	.000	.000	.000	.000	.000	.000	.000	.000	.000	.000	.000	.000	.000	.000	.000	.000	.000	.000
**a16_2**	.000	.000	.000	.000	.000	.000	.000	.000	.000	.000	.000	.000	.000	.000	.000	.000	.000	.000	.000	.000
**a17_2**	.000	.000	.000	.000	.000	.000	.000	.000	.000	.000	.000	.000	.000	.000	.000	.000	.000	.000	.000	.000
**a18_2**	.000	.000	.000	.000	.000	.000	.000	.000	.000	.000	.000	.000	.000	.000	.000	.000	.000	.000	.000	.000
**ccc1_2**	.000	.000	.000	.000	.000	.000	.000	.000	.000	.000	.000	.000	.000	.000	.000	.000	.000	.000	.000	.000
**ccc2_2**	.000	.000	.000	.000	.000	.000	.000	.000	.000	.000	.000	.000	.000	.000	.000	.000	.000	.000	.000	.000
**ccc3_2**	.000	.000	.000	.000	.000	.000	.000	.000	.000	.000	.000	.000	.000	.000	.000	.000	.000	.000	.000	.000
**ccc4_2**	.000	.000	.000	.000	.000	.000	.000	.000	.000	.000	.000	.000	.000	.000	.000	.000	.000	.000	.000	.000
**ccc5_2**	.000	.000	.000	.000	.000	.000	.000	.000	.000	.000	.000	.000	.000	.000	.000	.000	.000	.000	.000	.000
**ccc6_2**	.000	.000	.000	.000	.000	.000	.000	.000	.000	.000	.000	.000	.000	.000	.000	.000	.000	.000	.000	.000
**ccc7_2**	.000	.000	.000	.000	.000	.000	.000	.000	.000	.000	.000	.000	.000	.000	.000	.000	.000	.000	.000	.000
**ccc8_2**	.000	.000	.000	.000	.000	.000	.000	.000	.000	.000	.000	.000	.000	.000	.000	.000	.000	.000	.000	.000
**ccc9_2**	.000	.000	.000	.000	.000	.000	.000	.000	.000	.000	.000	.000	.000	.000	.000	.000	.000	.000	.000	.000

	a1_1	a2_1	a3_1	a4_1	a5_1	a6_1	a7_1	a8_1	a9_1	a10_1	a11_1	a12_1	a13_1	a14_1	a15_1	a16_1	a17_1	a18_1	ccc1_1	ccc2_1
ccc10_2	.000	.000	.000	.000	.000	.000	.000	.000	.000	.000	.000	.000	.000	.000	.000	.000	.000	.000	.000	.000
ccc11_2	.000	.000	.000	.000	.000	.000	.000	.000	.000	.000	.000	.000	.000	.000	.000	.000	.000	.000	.000	.000
ccc12_2	.000	.000	.000	.000	.000	.000	.000	.000	.000	.000	.000	.000	.000	.000	.000	.000	.000	.000	.000	.000
ccc13_2	.000	.000	.000	.000	.000	.000	.000	.000	.000	.000	.000	.000	.000	.000	.000	.000	.000	.000	.000	.000
ccc14_2	.000	.000	.000	.000	.000	.000	.000	.000	.000	.000	.000	.000	.000	.000	.000	.000	.000	.000	.000	.000
ccc15_2	.000	.000	.000	.000	.000	.000	.000	.000	.000	.000	.000	.000	.000	.000	.000	.000	.000	.000	.000	.000
vvv1_2	.000	.000	.000	.000	.000	.000	.000	.000	.000	.000	.000	.000	.000	.000	.000	.000	.000	.000	.000	.000
v1_2	.000	.000	.000	.000	.000	.000	.000	.000	.000	.000	.000	.000	.000	.000	.000	.000	.000	.000	.000	.000
v2_2	.000	.000	.000	.000	.000	.000	.000	.000	.000	.000	.000	.000	.000	.000	.000	.000	.000	.000	.000	.000
v3_2	.000	.000	.000	.000	.000	.000	.000	.000	.000	.000	.000	.000	.000	.000	.000	.000	.000	.000	.000	.000
v4_2	.000	.000	.000	.000	.000	.000	.000	.000	.000	.000	.000	.000	.000	.000	.000	.000	.000	.000	.000	.000
vvv2_2	.000	.000	.000	.000	.000	.000	.000	.000	.000	.000	.000	.000	.000	.000	.000	.000	.000	.000	.000	.000
v5_2	.000	.000	.000	.000	.000	.000	.000	.000	.000	.000	.000	.000	.000	.000	.000	.000	.000	.000	.000	.000
v6_2	.000	.000	.000	.000	.000	.000	.000	.000	.000	.000	.000	.000	.000	.000	.000	.000	.000	.000	.000	.000
v7_2	.000	.000	.000	.000	.000	.000	.000	.000	.000	.000	.000	.000	.000	.000	.000	.000	.000	.000	.000	.000
v8_2	.000	.000	.000	.000	.000	.000	.000	.000	.000	.000	.000	.000	.000	.000	.000	.000	.000	.000	.000	.000
vvv3_2	.000	.000	.000	.000	.000	.000	.000	.000	.000	.000	.000	.000	.000	.000	.000	.000	.000	.000	.000	.000
v9_2	.000	.000	.000	.000	.000	.000	.000	.000	.000	.000	.000	.000	.000	.000	.000	.000	.000	.000	.000	.000
v10_2	.000	.000	.000	.000	.000	.000	.000	.000	.000	.000	.000	.000	.000	.000	.000	.000	.000	.000	.000	.000
v11_2	.000	.000	.000	.000	.000	.000	.000	.000	.000	.000	.000	.000	.000	.000	.000	.000	.000	.000	.000	.000
v12_2	.000	.000	.000	.000	.000	.000	.000	.000	.000	.000	.000	.000	.000	.000	.000	.000	.000	.000	.000	.000
vvv4_2	.000	.000	.000	.000	.000	.000	.000	.000	.000	.000	.000	.000	.000	.000	.000	.000	.000	.000	.000	.000
v13_2	.000	.000	.000	.000	.000	.000	.000	.000	.000	.000	.000	.000	.000	.000	.000	.000	.000	.000	.000	.000
v14_2	.000	.000	.000	.000	.000	.000	.000	.000	.000	.000	.000	.000	.000	.000	.000	.000	.000	.000	.000	.000
v15_2	.000	.000	.000	.000	.000	.000	.000	.000	.000	.000	.000	.000	.000	.000	.000	.000	.000	.000	.000	.000
v16_2	.000	.000	.000	.000	.000	.000	.000	.000	.000	.000	.000	.000	.000	.000	.000	.000	.000	.000	.000	.000
vvv5_2	.000	.000	.000	.000	.000	.000	.000	.000	.000	.000	.000	.000	.000	.000	.000	.000	.000	.000	.000	.000
v17_2	.000	.000	.000	.000	.000	.000	.000	.000	.000	.000	.000	.000	.000	.000	.000	.000	.000	.000	.000	.000
v18_2	.000	.000	.000	.000	.000	.000	.000	.000	.000	.000	.000	.000	.000	.000	.000	.000	.000	.000	.000	.000
v19_2	.000	.000	.000	.000	.000	.000	.000	.000	.000	.000	.000	.000	.000	.000	.000	.000	.000	.000	.000	.000
v20_2	.000	.000	.000	.000	.000	.000	.000	.000	.000	.000	.000	.000	.000	.000	.000	.000	.000	.000	.000	.000
vvv6_2	.000	.000	.000	.000	.000	.000	.000	.000	.000	.000	.000	.000	.000	.000	.000	.000	.000	.000	.000	.000
v21_2	.000	.000	.000	.000	.000	.000	.000	.000	.000	.000	.000	.000	.000	.000	.000	.000	.000	.000	.000	.000
v22_2	.000	.000	.000	.000	.000	.000	.000	.000	.000	.000	.000	.000	.000	.000	.000	.000	.000	.000	.000	.000
v23_2	.000	.000	.000	.000	.000	.000	.000	.000	.000	.000	.000	.000	.000	.000	.000	.000	.000	.000	.000	.000
v24_2	.000	.000	.000	.000	.000	.000	.000	.000	.000	.000	.000	.000	.000	.000	.000	.000	.000	.000	.000	.000
i1_2	.000	.000	.000	.000	.000	.000	.000	.000	.000	.000	.000	.000	.000	.000	.000	.000	.000	.000	.000	.000
i2_2	.000	.000	.000	.000	.000	.000	.000	.000	.000	.000	.000	.000	.000	.000	.000	.000	.000	.000	.000	.000
i3_2	.000	.000	.000	.000	.000	.000	.000	.000	.000	.000	.000	.000	.000	.000	.000	.000	.000	.000	.000	.000
i4_2	.000	.000	.000	.000	.000	.000	.000	.000	.000	.000	.000	.000	.000	.000	.000	.000	.000	.000	.000	.000
i5_2	.000	.000	.000	.000	.000	.000	.000	.000	.000	.000	.000	.000	.000	.000	.000	.000	.000	.000	.000	.000
i6_2	.000	.000	.000	.000	.000	.000	.000	.000	.000	.000	.000	.000	.000	.000	.000	.000	.000	.000	.000	.000
i7_2	.000	.000	.000	.000	.000	.000	.000	.000	.000	.000	.000	.000	.000	.000	.000	.000	.000	.000	.000	.000
i8_2	.000	.000	.000	.000	.000	.000	.000	.000	.000	.000	.000	.000	.000	.000	.000	.000	.000	.000	.000	.000
i9_2	.000	.000	.000	.000	.000	.000	.000	.000	.000	.000	.000	.000	.000	.000	.000	.000	.000	.000	.000	.000
i10_2	.000	.000	.000	.000	.000	.000	.000	.000	.000	.000	.000	.000	.000	.000	.000	.000	.000	.000	.000	.000
i11_2	.000	.000	.000	.000	.000	.000	.000	.000	.000	.000	.000	.000	.000	.000	.000	.000	.000	.000	.000	.000
i12_2	.000	.000	.000	.000	.000	.000	.000	.000	.000	.000	.000	.000	.000	.000	.000	.000	.000	.000	.000	.000
i13_2	.000	.000	.000	.000	.000	.000	.000	.000	.000	.000	.000	.000	.000	.000	.000	.000	.000	.000	.000	.000
i14_2	.000	.000	.000	.000	.000	.000	.000	.000	.000	.000	.000	.000	.000	.000	.000	.000	.000	.000	.000	.000
i15_2	.000	.000	.000	.000	.000	.000	.000	.000	.000	.000	.000	.000	.000	.000	.000	.000	.000	.000	.000	.000
i16_2	.000	.000	.000	.000	.000	.000	.000	.000	.000	.000	.000	.000	.000	.000	.000	.000	.000	.000	.000	.000
i17_2	.000	.000	.000	.000	.000	.000	.000	.000	.000	.000	.000	.000	.000	.000	.000	.000	.000	.000	.000	.000
i18_2	.000	.000	.000	.000	.000	.000	.000	.000	.000	.000	.000	.000	.000	.000	.000	.000	.000	.000	.000	.000
i19_2	.000	.000	.000	.000	.000	.000	.000	.000	.000	.000	.000	.000	.000	.000	.000	.000	.000	.000	.000	.000
i20_2	.000	.000	.000	.000	.000	.000	.000	.000	.000	.000	.000	.000	.000	.000	.000	.000	.000	.000	.000	.000
i21_2	.000	.000	.000	.000	.000	.000	.000	.000	.000	.000	.000	.000	.000	.000	.000	.000	.000	.000	.000	.000
i22_2	.000	.000	.000	.000	.000	.000	.000	.000	.000	.000	.000	.000	.000	.000	.000	.000	.000	.000	.000	.000
i23_2	.000	.000	.000	.000	.000	.000	.000	.000	.000	.000	.000	.000	.000	.000	.000	.000	.000	.000	.000	.000
i24_2	.000	.000	.000	.000	.000	.000	.000	.000	.000	.000	.000	.000	.000	.000	.000	.000	.000	.000	.000	.000
a1_3	.000	.000	.000	.000	.000	.000	.000	.000	.000	.000	.000	.000	.000	.000	.000	.000	.000	.000	.000	.000
a2_3	.000	.000	.000	.000	.000	.000	.000	.000	.000	.000	.000	.000	.000	.000	.000	.000	.000	.000	.000	.000
a3_3	.000	.000	.000	.000	.000	.000	.000	.000	.000	.000	.000	.000	.000	.000	.000	.000	.000	.000	.000	.000
a4_3	.000	.000	.000	.000	.000	.000	.000	.000	.000	.000	.000	.000	.000	.000	.000	.000	.000	.000	.000	.000
a5_3	.000	.000	.000	.000	.000	.000	.000	.000	.000	.000	.000	.000	.000	.000	.000	.000	.000	.000	.000	.000
a6_3	.000	.000	.000	.000	.000	.000	.000	.000	.000	.000	.000	.000	.000	.000	.000	.000	.000	.000	.000	.000
a7_3	.000	.000	.000	.000	.000	.000	.000	.000	.000	.000	.000	.000	.000	.000	.000	.000	.000	.000	.000	.000
a8_3	.000	.000	.000	.000	.000	.000	.000	.000	.000	.000	.000	.000	.000	.000	.000	.000	.000	.000	.000	.000
a9_3	.000	.000	.000	.000	.000	.000	.000	.000	.000	.000	.000	.000	.000	.000	.000	.000	.000	.000	.000	.000
a10_3	.000	.000	.000	.000	.000	.000	.000	.000	.000	.000	.000	.000	.000	.000	.000	.000	.000	.000	.000	.000
a11_3	.000	.000	.000	.000	.000	.000	.000	.000	.000	.000	.000	.000	.000	.000	.000	.000	.000	.000	.000	.000
a12_3	.000	.000	.000	.000	.000	.000	.000	.000	.000	.000	.000	.000	.000	.000	.000	.000	.000	.000	.000	.000

	a1_1	a2_1	a3_1	a4_1	a5_1	a6_1	a7_1	a8_1	a9_1	a10_1	a11_1	a12_1	a13_1	a14_1	a15_1	a16_1	a17_1	a18_1	ccc1_1	ccc2_1
a13_3	.000	.000	.000	.000	.000	.000	.000	.000	.000	.000	.000	.000	.000	.000	.000	.000	.000	.000	.000	.000
a14_3	.000	.000	.000	.000	.000	.000	.000	.000	.000	.000	.000	.000	.000	.000	.000	.000	.000	.000	.000	.000
a15_3	.000	.000	.000	.000	.000	.000	.000	.000	.000	.000	.000	.000	.000	.000	.000	.000	.000	.000	.000	.000
a16_3	.000	.000	.000	.000	.000	.000	.000	.000	.000	.000	.000	.000	.000	.000	.000	.000	.000	.000	.000	.000
a17_3	.000	.000	.000	.000	.000	.000	.000	.000	.000	.000	.000	.000	.000	.000	.000	.000	.000	.000	.000	.000
a18_3	.000	.000	.000	.000	.000	.000	.000	.000	.000	.000	.000	.000	.000	.000	.000	.000	.000	.000	.000	.000
ccc1_3	.000	.000	.000	.000	.000	.000	.000	.000	.000	.000	.000	.000	.000	.000	.000	.000	.000	.000	.000	.000
ccc2_3	.000	.000	.000	.000	.000	.000	.000	.000	.000	.000	.000	.000	.000	.000	.000	.000	.000	.000	.000	.000
ccc3_3	.000	.000	.000	.000	.000	.000	.000	.000	.000	.000	.000	.000	.000	.000	.000	.000	.000	.000	.000	.000
ccc4_3	.000	.000	.000	.000	.000	.000	.000	.000	.000	.000	.000	.000	.000	.000	.000	.000	.000	.000	.000	.000
ccc5_3	.000	.000	.000	.000	.000	.000	.000	.000	.000	.000	.000	.000	.000	.000	.000	.000	.000	.000	.000	.000
ccc6_3	.000	.000	.000	.000	.000	.000	.000	.000	.000	.000	.000	.000	.000	.000	.000	.000	.000	.000	.000	.000
ccc7_3	.000	.000	.000	.000	.000	.000	.000	.000	.000	.000	.000	.000	.000	.000	.000	.000	.000	.000	.000	.000
ccc8_3	.000	.000	.000	.000	.000	.000	.000	.000	.000	.000	.000	.000	.000	.000	.000	.000	.000	.000	.000	.000
ccc9_3	.000	.000	.000	.000	.000	.000	.000	.000	.000	.000	.000	.000	.000	.000	.000	.000	.000	.000	.000	.000
ccc10_3	.000	.000	.000	.000	.000	.000	.000	.000	.000	.000	.000	.000	.000	.000	.000	.000	.000	.000	.000	.000
ccc11_3	.000	.000	.000	.000	.000	.000	.000	.000	.000	.000	.000	.000	.000	.000	.000	.000	.000	.000	.000	.000
ccc12_3	.000	.000	.000	.000	.000	.000	.000	.000	.000	.000	.000	.000	.000	.000	.000	.000	.000	.000	.000	.000
ccc13_3	.000	.000	.000	.000	.000	.000	.000	.000	.000	.000	.000	.000	.000	.000	.000	.000	.000	.000	.000	.000
ccc14_3	.000	.000	.000	.000	.000	.000	.000	.000	.000	.000	.000	.000	.000	.000	.000	.000	.000	.000	.000	.000
ccc15_3	.000	.000	.000	.000	.000	.000	.000	.000	.000	.000	.000	.000	.000	.000	.000	.000	.000	.000	.000	.000
vvv1_3	.000	.000	.000	.000	.000	.000	.000	.000	.000	.000	.000	.000	.000	.000	.000	.000	.000	.000	.000	.000
v1_3	.000	.000	.000	.000	.000	.000	.000	.000	.000	.000	.000	.000	.000	.000	.000	.000	.000	.000	.000	.000
v2_3	.000	.000	.000	.000	.000	.000	.000	.000	.000	.000	.000	.000	.000	.000	.000	.000	.000	.000	.000	.000
v3_3	.000	.000	.000	.000	.000	.000	.000	.000	.000	.000	.000	.000	.000	.000	.000	.000	.000	.000	.000	.000
v4_3	.000	.000	.000	.000	.000	.000	.000	.000	.000	.000	.000	.000	.000	.000	.000	.000	.000	.000	.000	.000
vvv2_3	.000	.000	.000	.000	.000	.000	.000	.000	.000	.000	.000	.000	.000	.000	.000	.000	.000	.000	.000	.000
v5_3	.000	.000	.000	.000	.000	.000	.000	.000	.000	.000	.000	.000	.000	.000	.000	.000	.000	.000	.000	.000
v6_3	.000	.000	.000	.000	.000	.000	.000	.000	.000	.000	.000	.000	.000	.000	.000	.000	.000	.000	.000	.000
v7_3	.000	.000	.000	.000	.000	.000	.000	.000	.000	.000	.000	.000	.000	.000	.000	.000	.000	.000	.000	.000
v8_3	.000	.000	.000	.000	.000	.000	.000	.000	.000	.000	.000	.000	.000	.000	.000	.000	.000	.000	.000	.000
vvv3_3	.000	.000	.000	.000	.000	.000	.000	.000	.000	.000	.000	.000	.000	.000	.000	.000	.000	.000	.000	.000
v9_3	.000	.000	.000	.000	.000	.000	.000	.000	.000	.000	.000	.000	.000	.000	.000	.000	.000	.000	.000	.000
v10_3	.000	.000	.000	.000	.000	.000	.000	.000	.000	.000	.000	.000	.000	.000	.000	.000	.000	.000	.000	.000
v11_3	.000	.000	.000	.000	.000	.000	.000	.000	.000	.000	.000	.000	.000	.000	.000	.000	.000	.000	.000	.000
v12_3	.000	.000	.000	.000	.000	.000	.000	.000	.000	.000	.000	.000	.000	.000	.000	.000	.000	.000	.000	.000
vvv4_3	.000	.000	.000	.000	.000	.000	.000	.000	.000	.000	.000	.000	.000	.000	.000	.000	.000	.000	.000	.000
v13_3	.000	.000	.000	.000	.000	.000	.000	.000	.000	.000	.000	.000	.000	.000	.000	.000	.000	.000	.000	.000
v14_3	.000	.000	.000	.000	.000	.000	.000	.000	.000	.000	.000	.000	.000	.000	.000	.000	.000	.000	.000	.000
v15_3	.000	.000	.000	.000	.000	.000	.000	.000	.000	.000	.000	.000	.000	.000	.000	.000	.000	.000	.000	.000
v16_3	.000	.000	.000	.000	.000	.000	.000	.000	.000	.000	.000	.000	.000	.000	.000	.000	.000	.000	.000	.000
vvv5_3	.000	.000	.000	.000	.000	.000	.000	.000	.000	.000	.000	.000	.000	.000	.000	.000	.000	.000	.000	.000
v17_3	.000	.000	.000	.000	.000	.000	.000	.000	.000	.000	.000	.000	.000	.000	.000	.000	.000	.000	.000	.000
v18_3	.000	.000	.000	.000	.000	.000	.000	.000	.000	.000	.000	.000	.000	.000	.000	.000	.000	.000	.000	.000
v19_3	.000	.000	.000	.000	.000	.000	.000	.000	.000	.000	.000	.000	.000	.000	.000	.000	.000	.000	.000	.000
v20_3	.000	.000	.000	.000	.000	.000	.000	.000	.000	.000	.000	.000	.000	.000	.000	.000	.000	.000	.000	.000
vvv6_3	.000	.000	.000	.000	.000	.000	.000	.000	.000	.000	.000	.000	.000	.000	.000	.000	.000	.000	.000	.000
v21_3	.000	.000	.000	.000	.000	.000	.000	.000	.000	.000	.000	.000	.000	.000	.000	.000	.000	.000	.000	.000
v22_3	.000	.000	.000	.000	.000	.000	.000	.000	.000	.000	.000	.000	.000	.000	.000	.000	.000	.000	.000	.000
v23_3	.000	.000	.000	.000	.000	.000	.000	.000	.000	.000	.000	.000	.000	.000	.000	.000	.000	.000	.000	.000
v24_3	.000	.000	.000	.000	.000	.000	.000	.000	.000	.000	.000	.000	.000	.000	.000	.000	.000	.000	.000	.000
i1_3	.000	.000	.000	.000	.000	.000	.000	.000	.000	.000	.000	.000	.000	.000	.000	.000	.000	.000	.000	.000
i2_3	.000	.000	.000	.000	.000	.000	.000	.000	.000	.000	.000	.000	.000	.000	.000	.000	.000	.000	.000	.000
i3_3	.000	.000	.000	.000	.000	.000	.000	.000	.000	.000	.000	.000	.000	.000	.000	.000	.000	.000	.000	.000
i4_3	.000	.000	.000	.000	.000	.000	.000	.000	.000	.000	.000	.000	.000	.000	.000	.000	.000	.000	.000	.000
i5_3	.000	.000	.000	.000	.000	.000	.000	.000	.000	.000	.000	.000	.000	.000	.000	.000	.000	.000	.000	.000
i6_3	.000	.000	.000	.000	.000	.000	.000	.000	.000	.000	.000	.000	.000	.000	.000	.000	.000	.000	.000	.000
i7_3	.000	.000	.000	.000	.000	.000	.000	.000	.000	.000	.000	.000	.000	.000	.000	.000	.000	.000	.000	.000
i8_3	.000	.000	.000	.000	.000	.000	.000	.000	.000	.000	.000	.000	.000	.000	.000	.000	.000	.000	.000	.000
i9_3	.000	.000	.000	.000	.000	.000	.000	.000	.000	.000	.000	.000	.000	.000	.000	.000	.000	.000	.000	.000
i10_3	.000	.000	.000	.000	.000	.000	.000	.000	.000	.000	.000	.000	.000	.000	.000	.000	.000	.000	.000	.000
i11_3	.000	.000	.000	.000	.000	.000	.000	.000	.000	.000	.000	.000	.000	.000	.000	.000	.000	.000	.000	.000
i12_3	.000	.000	.000	.000	.000	.000	.000	.000	.000	.000	.000	.000	.000	.000	.000	.000	.000	.000	.000	.000
i13_3	.000	.000	.000	.000	.000	.000	.000	.000	.000	.000	.000	.000	.000	.000	.000	.000	.000	.000	.000	.000
i14_3	.000	.000	.000	.000	.000	.000	.000	.000	.000	.000	.000	.000	.000	.000	.000	.000	.000	.000	.000	.000
i15_3	.000	.000	.000	.000	.000	.000	.000	.000	.000	.000	.000	.000	.000	.000	.000	.000	.000	.000	.000	.000
i16_3	.000	.000	.000	.000	.000	.000	.000	.000	.000	.000	.000	.000	.000	.000	.000	.000	.000	.000	.000	.000
i17_3	.000	.000	.000	.000	.000	.000	.000	.000	.000	.000	.000	.000	.000	.000	.000	.000	.000	.000	.000	.000
i18_3	.000	.000	.000	.000	.000	.000	.000	.000	.000	.000	.000	.000	.000	.000	.000	.000	.000	.000	.000	.000
i19_3	.000	.000	.000	.000	.000	.000	.000	.000	.000	.000	.000	.000	.000	.000	.000	.000	.000	.000	.000	.000
i20_3	.000	.000	.000	.000	.000	.000	.000	.000	.000	.000	.000	.000	.000	.000	.000	.000	.000	.000	.000	.000
i21_3	.000	.000	.000	.000	.000	.000	.000	.000	.000	.000	.000	.000	.000	.000	.000	.000	.000	.000	.000	.000

	a1_1	a2_1	a3_1	a4_1	a5_1	a6_1	a7_1	a8_1	a9_1	a10_1	a11_1	a12_1	a13_1	a14_1	a15_1	a16_1	a17_1	a18_1	ccc1_1	ccc2_1
i22_3	.000	.000	.000	.000	.000	.000	.000	.000	.000	.000	.000	.000	.000	.000	.000	.000	.000	.000	.000	.000
i23_3	.000	.000	.000	.000	.000	.000	.000	.000	.000	.000	.000	.000	.000	.000	.000	.000	.000	.000	.000	.000
i24_3	.000	.000	.000	.000	.000	.000	.000	.000	.000	.000	.000	.000	.000	.000	.000	.000	.000	.000	.000	.000

Correlations of Estimates (Unconstrained)

	a1_1	a2_1	a3_1	a4_1	a5_1	a6_1	a7_1	a8_1	a9_1	a10_1	a11_1	a12_1	a13_1	a14_1	a15_1	a16_1	a17_1	a18_1	ccc1_1
a1_1	1.000																		
a2_1	.444	1.000																	
a3_1	.349	.247	1.000																
a4_1	.000	.000	.000	1.000															
a5_1	.000	.000	.000	.967	1.000														
a6_1	.000	.000	.000	.960	.965	1.000													
a7_1	.000	.000	.000	.000	.000	.000	1.000												
a8_1	.000	.000	.000	.000	.000	.000	.583	1.000											
a9_1	.000	.000	.000	.000	.000	.000	.427	.452	1.000										
a10_1	.000	.000	.000	.000	.000	.000	.000	.000	.000	1.000									
a11_1	.000	.000	.000	.000	.000	.000	.000	.000	.000	.608	1.000								
a12_1	.000	.000	.000	.000	.000	.000	.000	.000	.000	.599	.595	1.000							
a13_1	.000	.000	.000	.000	.000	.000	.000	.000	.000	.000	.000	.000	1.000						
a14_1	.000	.000	.000	.000	.000	.000	.000	.000	.000	.000	.000	.000	.551	1.000					
a15_1	.000	.000	.000	.000	.000	.000	.000	.000	.000	.000	.000	.000	.506	.447	1.000				
a16_1	.000	.000	.000	.000	.000	.000	.000	.000	.000	.000	.000	.000	.000	.000	.000	1.000			
a17_1	.000	.000	.000	.000	.000	.000	.000	.000	.000	.000	.000	.000	.000	.000	.000	.416	1.000		
a18_1	.000	.000	.000	.000	.000	.000	.000	.000	.000	.000	.000	.000	.000	.000	.000	.384	.434	1.000	
ccc1_1	.096	.066	.052	.793	.799	.791	.000	.000	.000	.000	.000	.000	.000	.000	.000	.000	.000	.000	1.000
ccc2_1	.000	.000	.000	.875	.882	.873	.093	.100	.070	.000	.000	.000	.000	.000	.000	.000	.000	.000	.869
ccc3_1	-.399	-.275	-.216	.000	.000	.000	-.347	-.376	-.264	.000	.000	.000	.000	.000	.000	.000	.000	.000	-.183
ccc4_1	.000	.000	.000	-.936	-.943	-.934	.000	.000	.000	-.111	-.110	-.108	.000	.000	.000	.000	.000	.000	-.827
ccc5_1	.000	.000	.000	.000	.000	.000	.298	.323	.227	.334	.331	.326	.000	.000	.000	.000	.000	.000	.152
ccc6_1	.286	.197	.155	.000	.000	.000	.000	.000	.000	.279	.277	.273	.000	.000	.000	.000	.000	.000	.274
ccc7_1	.000	.000	.000	.719	.724	.717	.000	.000	.000	.000	.000	.000	.077	.068	.062	.000	.000	.000	.882
ccc8_1	.000	.000	.000	.000	.000	.000	.000	.000	.000	.362	.359	.354	.328	.289	.265	.000	.000	.000	.158
ccc9_1	.000	.000	.000	.000	.000	.000	-.357	-.387	-.272	.000	.000	.000	-.363	-.319	-.292	.000	.000	.000	-.105
ccc10_1	-.448	-.309	-.242	.000	.000	.000	.000	.000	.000	.000	.000	.000	-.397	-.349	-.320	.000	.000	.000	-.139
ccc11_1	.000	.000	.000	.000	.000	.000	.000	.000	.000	.000	.000	.000	.373	.328	.301	.219	.249	.229	.177
ccc12_1	.000	.000	.000	.000	.000	.000	.286	.310	.218	.000	.000	.000	.000	.000	.000	.171	.194	.178	.185
ccc13_1	.000	.000	.000	.000	.000	.000	.000	.000	.000	-.456	-.452	-.446	.000	.000	.000	-.243	-.277	-.254	-.121
ccc14_1	.000	.000	.000	-.946	-.954	-.944	.000	.000	.000	.000	.000	.000	.000	.000	.000	-.060	-.068	-.062	-.838
ccc15_1	.327	.226	.177	.000	.000	.000	.000	.000	.000	.000	.000	.000	.000	.000	.000	.170	.194	.178	.304
vvv1_1	-.680	-.483	-.380	.000	.000	.000	.000	.000	.000	.000	.000	.000	.000	.000	.000	.000	.000	.000	-.167
v1_1	.151	.111	.088	.000	.000	.000	.000	.000	.000	.000	.000	.000	.000	.000	.000	.000	.000	.000	.016
v2_1	-.183	.003	.003	.000	.000	.000	.000	.000	.000	.000	.000	.000	.000	.000	.000	.000	.000	.000	-.014
v3_1	-.002	-.102	.001	.000	.000	.000	.000	.000	.000	.000	.000	.000	.000	.000	.000	.000	.000	.000	-.003
v4_1	-.001	.000	-.078	.000	.000	.000	.000	.000	.000	.000	.000	.000	.000	.000	.000	.000	.000	.000	-.001
vvv2_1	.000	.000	.000	-.978	-.984	-.976	.000	.000	.000	.000	.000	.000	.000	.000	.000	.000	.000	.000	-.815
v5_1	.000	.000	.000	.057	.057	.057	.000	.000	.000	.000	.000	.000	.000	.000	.000	.000	.000	.000	.046
v6_1	.000	.000	.000	-.071	.009	.002	.000	.000	.000	.000	.000	.000	.000	.000	.000	.000	.000	.000	-.012
v7_1	.000	.000	.000	.007	-.088	.006	.000	.000	.000	.000	.000	.000	.000	.000	.000	.000	.000	.000	-.031
v8_1	.000	.000	.000	.002	.007	-.069	.000	.000	.000	.000	.000	.000	.000	.000	.000	.000	.000	.000	-.010
vvv3_1	.000	.000	.000	.000	.000	.000	-.639	-.683	-.490	.000	.000	.000	.000	.000	.000	.000	.000	.000	-.076
v9_1	.000	.000	.000	.000	.000	.000	.207	.230	.154	.000	.000	.000	.000	.000	.000	.000	.000	.000	.000
v10_1	.000	.000	.000	.000	.000	.000	-.219	.021	-.004	.000	.000	.000	.000	.000	.000	.000	.000	.000	.000
v11_1	.000	.000	.000	.000	.000	.000	.008	-.274	-.008	.000	.000	.000	.000	.000	.000	.000	.000	.000	.000
v12_1	.000	.000	.000	.000	.000	.000	.001	.006	-.139	.000	.000	.000	.000	.000	.000	.000	.000	.000	.000
vvv4_1	.000	.000	.000	.000	.000	.000	.000	.000	.000	-.679	-.674	-.664	.000	.000	.000	.000	.000	.000	-.075
v13_1	.000	.000	.000	.000	.000	.000	.000	.000	.000	.137	.136	.134	.000	.000	.000	.000	.000	.000	.000
v14_1	.000	.000	.000	.000	.000	.000	.000	.000	.000	-.149	.001	.001	.000	.000	.000	.000	.000	.000	.000
v15_1	.000	.000	.000	.000	.000	.000	.000	.000	.000	.001	-.146	.001	.000	.000	.000	.000	.000	.000	.000
v16_1	.000	.000	.000	.000	.000	.000	.000	.000	.000	.001	.001	-.142	.000	.000	.000	.000	.000	.000	.000
vvv5_1	.000	.000	.000	.000	.000	.000	.000	.000	.000	.000	.000	.000	-.661	-.585	-.537	.000	.000	.000	-.053
v17_1	.000	.000	.000	.000	.000	.000	.000	.000	.000	.000	.000	.000	.106	.096	.088	.000	.000	.000	.000
v18_1	.000	.000	.000	.000	.000	.000	.000	.000	.000	.000	.000	.000	-.118	.000	.001	.000	.000	.000	.000
v19_1	.000	.000	.000	.000	.000	.000	.000	.000	.000	.000	.000	.000	-.002	-.095	.000	.000	.000	.000	.000
v20_1	.000	.000	.000	.000	.000	.000	.000	.000	.000	.000	.000	.000	-.001	.000	-.085	.000	.000	.000	.000
vvv6_1	.000	.000	.000	.000	.000	.000	.000	.000	.000	.000	.000	.000	.000	.000	.000	-.467	-.529	-.488	-.119
v21_1	.000	.000	.000	.000	.000	.000	.000	.000	.000	.000	.000	.000	.000	.000	.000	.136	.155	.143	.000
v22_1	.000	.000	.000	.000	.000	.000	.000	.000	.000	.000	.000	.000	.000	.000	.000	-.124	.000	.000	.000
v23_1	.000	.000	.000	.000	.000	.000	.000	.000	.000	.000	.000	.000	.000	.000	.000	-.001	-.151	-.001	.000
v24_1	.000	.000	.000	.000	.000	.000	.000	.000	.000	.000	.000	.000	.000	.000	.000	-.001	.000	-.132	.000
i1_1	.000	.000	.000	.000	.000	.000	.000	.000	.000	.000	.000	.000	.000	.000	.000	.000	.000	.000	.000

	a1_1	a2_1	a3_1	a4_1	a5_1	a6_1	a7_1	a8_1	a9_1	a10_1	a11_1	a12_1	a13_1	a14_1	a15_1	a16_1	a17_1	a18_1	ccc1_1
i2_1	.000	.000	.000	.000	.000	.000	.000	.000	.000	.000	.000	.000	.000	.000	.000	.000	.000	.000	.000
i3_1	.000	.000	.000	.000	.000	.000	.000	.000	.000	.000	.000	.000	.000	.000	.000	.000	.000	.000	.000
i4_1	.000	.000	.000	.000	.000	.000	.000	.000	.000	.000	.000	.000	.000	.000	.000	.000	.000	.000	.000
i5_1	.000	.000	.000	.000	.000	.000	.000	.000	.000	.000	.000	.000	.000	.000	.000	.000	.000	.000	.000
i6_1	.000	.000	.000	.000	.000	.000	.000	.000	.000	.000	.000	.000	.000	.000	.000	.000	.000	.000	.000
i7_1	.000	.000	.000	.000	.000	.000	.000	.000	.000	.000	.000	.000	.000	.000	.000	.000	.000	.000	.000
i8_1	.000	.000	.000	.000	.000	.000	.000	.000	.000	.000	.000	.000	.000	.000	.000	.000	.000	.000	.000
i9_1	.000	.000	.000	.000	.000	.000	.000	.000	.000	.000	.000	.000	.000	.000	.000	.000	.000	.000	.000
i10_1	.000	.000	.000	.000	.000	.000	.000	.000	.000	.000	.000	.000	.000	.000	.000	.000	.000	.000	.000
i11_1	.000	.000	.000	.000	.000	.000	.000	.000	.000	.000	.000	.000	.000	.000	.000	.000	.000	.000	.000
i12_1	.000	.000	.000	.000	.000	.000	.000	.000	.000	.000	.000	.000	.000	.000	.000	.000	.000	.000	.000
i13_1	.000	.000	.000	.000	.000	.000	.000	.000	.000	.000	.000	.000	.000	.000	.000	.000	.000	.000	.000
i14_1	.000	.000	.000	.000	.000	.000	.000	.000	.000	.000	.000	.000	.000	.000	.000	.000	.000	.000	.000
i15_1	.000	.000	.000	.000	.000	.000	.000	.000	.000	.000	.000	.000	.000	.000	.000	.000	.000	.000	.000
i16_1	.000	.000	.000	.000	.000	.000	.000	.000	.000	.000	.000	.000	.000	.000	.000	.000	.000	.000	.000
i17_1	.000	.000	.000	.000	.000	.000	.000	.000	.000	.000	.000	.000	.000	.000	.000	.000	.000	.000	.000
i18_1	.000	.000	.000	.000	.000	.000	.000	.000	.000	.000	.000	.000	.000	.000	.000	.000	.000	.000	.000
i19_1	.000	.000	.000	.000	.000	.000	.000	.000	.000	.000	.000	.000	.000	.000	.000	.000	.000	.000	.000
i20_1	.000	.000	.000	.000	.000	.000	.000	.000	.000	.000	.000	.000	.000	.000	.000	.000	.000	.000	.000
i21_1	.000	.000	.000	.000	.000	.000	.000	.000	.000	.000	.000	.000	.000	.000	.000	.000	.000	.000	.000
i22_1	.000	.000	.000	.000	.000	.000	.000	.000	.000	.000	.000	.000	.000	.000	.000	.000	.000	.000	.000
i23_1	.000	.000	.000	.000	.000	.000	.000	.000	.000	.000	.000	.000	.000	.000	.000	.000	.000	.000	.000
i24_1	.000	.000	.000	.000	.000	.000	.000	.000	.000	.000	.000	.000	.000	.000	.000	.000	.000	.000	.000
a1_2	.000	.000	.000	.000	.000	.000	.000	.000	.000	.000	.000	.000	.000	.000	.000	.000	.000	.000	.000
a2_2	.000	.000	.000	.000	.000	.000	.000	.000	.000	.000	.000	.000	.000	.000	.000	.000	.000	.000	.000
a3_2	.000	.000	.000	.000	.000	.000	.000	.000	.000	.000	.000	.000	.000	.000	.000	.000	.000	.000	.000
a4_2	.000	.000	.000	.000	.000	.000	.000	.000	.000	.000	.000	.000	.000	.000	.000	.000	.000	.000	.000
a5_2	.000	.000	.000	.000	.000	.000	.000	.000	.000	.000	.000	.000	.000	.000	.000	.000	.000	.000	.000
a6_2	.000	.000	.000	.000	.000	.000	.000	.000	.000	.000	.000	.000	.000	.000	.000	.000	.000	.000	.000
a7_2	.000	.000	.000	.000	.000	.000	.000	.000	.000	.000	.000	.000	.000	.000	.000	.000	.000	.000	.000
a8_2	.000	.000	.000	.000	.000	.000	.000	.000	.000	.000	.000	.000	.000	.000	.000	.000	.000	.000	.000
a9_2	.000	.000	.000	.000	.000	.000	.000	.000	.000	.000	.000	.000	.000	.000	.000	.000	.000	.000	.000
a10_2	.000	.000	.000	.000	.000	.000	.000	.000	.000	.000	.000	.000	.000	.000	.000	.000	.000	.000	.000
a11_2	.000	.000	.000	.000	.000	.000	.000	.000	.000	.000	.000	.000	.000	.000	.000	.000	.000	.000	.000
a12_2	.000	.000	.000	.000	.000	.000	.000	.000	.000	.000	.000	.000	.000	.000	.000	.000	.000	.000	.000
a13_2	.000	.000	.000	.000	.000	.000	.000	.000	.000	.000	.000	.000	.000	.000	.000	.000	.000	.000	.000
a14_2	.000	.000	.000	.000	.000	.000	.000	.000	.000	.000	.000	.000	.000	.000	.000	.000	.000	.000	.000
a15_2	.000	.000	.000	.000	.000	.000	.000	.000	.000	.000	.000	.000	.000	.000	.000	.000	.000	.000	.000
a16_2	.000	.000	.000	.000	.000	.000	.000	.000	.000	.000	.000	.000	.000	.000	.000	.000	.000	.000	.000
a17_2	.000	.000	.000	.000	.000	.000	.000	.000	.000	.000	.000	.000	.000	.000	.000	.000	.000	.000	.000
a18_2	.000	.000	.000	.000	.000	.000	.000	.000	.000	.000	.000	.000	.000	.000	.000	.000	.000	.000	.000
ccc1_2	.000	.000	.000	.000	.000	.000	.000	.000	.000	.000	.000	.000	.000	.000	.000	.000	.000	.000	.000
ccc2_2	.000	.000	.000	.000	.000	.000	.000	.000	.000	.000	.000	.000	.000	.000	.000	.000	.000	.000	.000
ccc3_2	.000	.000	.000	.000	.000	.000	.000	.000	.000	.000	.000	.000	.000	.000	.000	.000	.000	.000	.000
ccc4_2	.000	.000	.000	.000	.000	.000	.000	.000	.000	.000	.000	.000	.000	.000	.000	.000	.000	.000	.000
ccc5_2	.000	.000	.000	.000	.000	.000	.000	.000	.000	.000	.000	.000	.000	.000	.000	.000	.000	.000	.000
ccc6_2	.000	.000	.000	.000	.000	.000	.000	.000	.000	.000	.000	.000	.000	.000	.000	.000	.000	.000	.000
ccc7_2	.000	.000	.000	.000	.000	.000	.000	.000	.000	.000	.000	.000	.000	.000	.000	.000	.000	.000	.000
ccc8_2	.000	.000	.000	.000	.000	.000	.000	.000	.000	.000	.000	.000	.000	.000	.000	.000	.000	.000	.000
ccc9_2	.000	.000	.000	.000	.000	.000	.000	.000	.000	.000	.000	.000	.000	.000	.000	.000	.000	.000	.000
ccc10_2	.000	.000	.000	.000	.000	.000	.000	.000	.000	.000	.000	.000	.000	.000	.000	.000	.000	.000	.000
ccc11_2	.000	.000	.000	.000	.000	.000	.000	.000	.000	.000	.000	.000	.000	.000	.000	.000	.000	.000	.000
ccc12_2	.000	.000	.000	.000	.000	.000	.000	.000	.000	.000	.000	.000	.000	.000	.000	.000	.000	.000	.000
ccc13_2	.000	.000	.000	.000	.000	.000	.000	.000	.000	.000	.000	.000	.000	.000	.000	.000	.000	.000	.000
ccc14_2	.000	.000	.000	.000	.000	.000	.000	.000	.000	.000	.000	.000	.000	.000	.000	.000	.000	.000	.000
ccc15_2	.000	.000	.000	.000	.000	.000	.000	.000	.000	.000	.000	.000	.000	.000	.000	.000	.000	.000	.000
vvv1_2	.000	.000	.000	.000	.000	.000	.000	.000	.000	.000	.000	.000	.000	.000	.000	.000	.000	.000	.000
v1_2	.000	.000	.000	.000	.000	.000	.000	.000	.000	.000	.000	.000	.000	.000	.000	.000	.000	.000	.000
v2_2	.000	.000	.000	.000	.000	.000	.000	.000	.000	.000	.000	.000	.000	.000	.000	.000	.000	.000	.000
v3_2	.000	.000	.000	.000	.000	.000	.000	.000	.000	.000	.000	.000	.000	.000	.000	.000	.000	.000	.000
v4_2	.000	.000	.000	.000	.000	.000	.000	.000	.000	.000	.000	.000	.000	.000	.000	.000	.000	.000	.000
vvv2_2	.000	.000	.000	.000	.000	.000	.000	.000	.000	.000	.000	.000	.000	.000	.000	.000	.000	.000	.000
v5_2	.000	.000	.000	.000	.000	.000	.000	.000	.000	.000	.000	.000	.000	.000	.000	.000	.000	.000	.000
v6_2	.000	.000	.000	.000	.000	.000	.000	.000	.000	.000	.000	.000	.000	.000	.000	.000	.000	.000	.000
v7_2	.000	.000	.000	.000	.000	.000	.000	.000	.000	.000	.000	.000	.000	.000	.000	.000	.000	.000	.000
v8_2	.000	.000	.000	.000	.000	.000	.000	.000	.000	.000	.000	.000	.000	.000	.000	.000	.000	.000	.000
vvv3_2	.000	.000	.000	.000	.000	.000	.000	.000	.000	.000	.000	.000	.000	.000	.000	.000	.000	.000	.000
v9_2	.000	.000	.000	.000	.000	.000	.000	.000	.000	.000	.000	.000	.000	.000	.000	.000	.000	.000	.000
v10_2	.000	.000	.000	.000	.000	.000	.000	.000	.000	.000	.000	.000	.000	.000	.000	.000	.000	.000	.000
v11_2	.000	.000	.000	.000	.000	.000	.000	.000	.000	.000	.000	.000	.000	.000	.000	.000	.000	.000	.000
v12_2	.000	.000	.000	.000	.000	.000	.000	.000	.000	.000	.000	.000	.000	.000	.000	.000	.000	.000	.000
vvv4_2	.000	.000	.000	.000	.000	.000	.000	.000	.000	.000	.000	.000	.000	.000	.000	.000	.000	.000	.000

	a1_1	a2_1	a3_1	a4_1	a5_1	a6_1	a7_1	a8_1	a9_1	a10_1	a11_1	a12_1	a13_1	a14_1	a15_1	a16_1	a17_1	a18_1	ccc1_1
v13_2	.000	.000	.000	.000	.000	.000	.000	.000	.000	.000	.000	.000	.000	.000	.000	.000	.000	.000	.000
v14_2	.000	.000	.000	.000	.000	.000	.000	.000	.000	.000	.000	.000	.000	.000	.000	.000	.000	.000	.000
v15_2	.000	.000	.000	.000	.000	.000	.000	.000	.000	.000	.000	.000	.000	.000	.000	.000	.000	.000	.000
v16_2	.000	.000	.000	.000	.000	.000	.000	.000	.000	.000	.000	.000	.000	.000	.000	.000	.000	.000	.000
vvv5_2	.000	.000	.000	.000	.000	.000	.000	.000	.000	.000	.000	.000	.000	.000	.000	.000	.000	.000	.000
v17_2	.000	.000	.000	.000	.000	.000	.000	.000	.000	.000	.000	.000	.000	.000	.000	.000	.000	.000	.000
v18_2	.000	.000	.000	.000	.000	.000	.000	.000	.000	.000	.000	.000	.000	.000	.000	.000	.000	.000	.000
v19_2	.000	.000	.000	.000	.000	.000	.000	.000	.000	.000	.000	.000	.000	.000	.000	.000	.000	.000	.000
v20_2	.000	.000	.000	.000	.000	.000	.000	.000	.000	.000	.000	.000	.000	.000	.000	.000	.000	.000	.000
vvv6_2	.000	.000	.000	.000	.000	.000	.000	.000	.000	.000	.000	.000	.000	.000	.000	.000	.000	.000	.000
v21_2	.000	.000	.000	.000	.000	.000	.000	.000	.000	.000	.000	.000	.000	.000	.000	.000	.000	.000	.000
v22_2	.000	.000	.000	.000	.000	.000	.000	.000	.000	.000	.000	.000	.000	.000	.000	.000	.000	.000	.000
v23_2	.000	.000	.000	.000	.000	.000	.000	.000	.000	.000	.000	.000	.000	.000	.000	.000	.000	.000	.000
v24_2	.000	.000	.000	.000	.000	.000	.000	.000	.000	.000	.000	.000	.000	.000	.000	.000	.000	.000	.000
i1_2	.000	.000	.000	.000	.000	.000	.000	.000	.000	.000	.000	.000	.000	.000	.000	.000	.000	.000	.000
i2_2	.000	.000	.000	.000	.000	.000	.000	.000	.000	.000	.000	.000	.000	.000	.000	.000	.000	.000	.000
i3_2	.000	.000	.000	.000	.000	.000	.000	.000	.000	.000	.000	.000	.000	.000	.000	.000	.000	.000	.000
i4_2	.000	.000	.000	.000	.000	.000	.000	.000	.000	.000	.000	.000	.000	.000	.000	.000	.000	.000	.000
i5_2	.000	.000	.000	.000	.000	.000	.000	.000	.000	.000	.000	.000	.000	.000	.000	.000	.000	.000	.000
i6_2	.000	.000	.000	.000	.000	.000	.000	.000	.000	.000	.000	.000	.000	.000	.000	.000	.000	.000	.000
i7_2	.000	.000	.000	.000	.000	.000	.000	.000	.000	.000	.000	.000	.000	.000	.000	.000	.000	.000	.000
i8_2	.000	.000	.000	.000	.000	.000	.000	.000	.000	.000	.000	.000	.000	.000	.000	.000	.000	.000	.000
i9_2	.000	.000	.000	.000	.000	.000	.000	.000	.000	.000	.000	.000	.000	.000	.000	.000	.000	.000	.000
i10_2	.000	.000	.000	.000	.000	.000	.000	.000	.000	.000	.000	.000	.000	.000	.000	.000	.000	.000	.000
i11_2	.000	.000	.000	.000	.000	.000	.000	.000	.000	.000	.000	.000	.000	.000	.000	.000	.000	.000	.000
i12_2	.000	.000	.000	.000	.000	.000	.000	.000	.000	.000	.000	.000	.000	.000	.000	.000	.000	.000	.000
i13_2	.000	.000	.000	.000	.000	.000	.000	.000	.000	.000	.000	.000	.000	.000	.000	.000	.000	.000	.000
i14_2	.000	.000	.000	.000	.000	.000	.000	.000	.000	.000	.000	.000	.000	.000	.000	.000	.000	.000	.000
i15_2	.000	.000	.000	.000	.000	.000	.000	.000	.000	.000	.000	.000	.000	.000	.000	.000	.000	.000	.000
i16_2	.000	.000	.000	.000	.000	.000	.000	.000	.000	.000	.000	.000	.000	.000	.000	.000	.000	.000	.000
i17_2	.000	.000	.000	.000	.000	.000	.000	.000	.000	.000	.000	.000	.000	.000	.000	.000	.000	.000	.000
i18_2	.000	.000	.000	.000	.000	.000	.000	.000	.000	.000	.000	.000	.000	.000	.000	.000	.000	.000	.000
i19_2	.000	.000	.000	.000	.000	.000	.000	.000	.000	.000	.000	.000	.000	.000	.000	.000	.000	.000	.000
i20_2	.000	.000	.000	.000	.000	.000	.000	.000	.000	.000	.000	.000	.000	.000	.000	.000	.000	.000	.000
i21_2	.000	.000	.000	.000	.000	.000	.000	.000	.000	.000	.000	.000	.000	.000	.000	.000	.000	.000	.000
i22_2	.000	.000	.000	.000	.000	.000	.000	.000	.000	.000	.000	.000	.000	.000	.000	.000	.000	.000	.000
i23_2	.000	.000	.000	.000	.000	.000	.000	.000	.000	.000	.000	.000	.000	.000	.000	.000	.000	.000	.000
i24_2	.000	.000	.000	.000	.000	.000	.000	.000	.000	.000	.000	.000	.000	.000	.000	.000	.000	.000	.000
a1_3	.000	.000	.000	.000	.000	.000	.000	.000	.000	.000	.000	.000	.000	.000	.000	.000	.000	.000	.000
a2_3	.000	.000	.000	.000	.000	.000	.000	.000	.000	.000	.000	.000	.000	.000	.000	.000	.000	.000	.000
a3_3	.000	.000	.000	.000	.000	.000	.000	.000	.000	.000	.000	.000	.000	.000	.000	.000	.000	.000	.000
a4_3	.000	.000	.000	.000	.000	.000	.000	.000	.000	.000	.000	.000	.000	.000	.000	.000	.000	.000	.000
a5_3	.000	.000	.000	.000	.000	.000	.000	.000	.000	.000	.000	.000	.000	.000	.000	.000	.000	.000	.000
a6_3	.000	.000	.000	.000	.000	.000	.000	.000	.000	.000	.000	.000	.000	.000	.000	.000	.000	.000	.000
a7_3	.000	.000	.000	.000	.000	.000	.000	.000	.000	.000	.000	.000	.000	.000	.000	.000	.000	.000	.000
a8_3	.000	.000	.000	.000	.000	.000	.000	.000	.000	.000	.000	.000	.000	.000	.000	.000	.000	.000	.000
a9_3	.000	.000	.000	.000	.000	.000	.000	.000	.000	.000	.000	.000	.000	.000	.000	.000	.000	.000	.000
a10_3	.000	.000	.000	.000	.000	.000	.000	.000	.000	.000	.000	.000	.000	.000	.000	.000	.000	.000	.000
a11_3	.000	.000	.000	.000	.000	.000	.000	.000	.000	.000	.000	.000	.000	.000	.000	.000	.000	.000	.000
a12_3	.000	.000	.000	.000	.000	.000	.000	.000	.000	.000	.000	.000	.000	.000	.000	.000	.000	.000	.000
a13_3	.000	.000	.000	.000	.000	.000	.000	.000	.000	.000	.000	.000	.000	.000	.000	.000	.000	.000	.000
a14_3	.000	.000	.000	.000	.000	.000	.000	.000	.000	.000	.000	.000	.000	.000	.000	.000	.000	.000	.000
a15_3	.000	.000	.000	.000	.000	.000	.000	.000	.000	.000	.000	.000	.000	.000	.000	.000	.000	.000	.000
a16_3	.000	.000	.000	.000	.000	.000	.000	.000	.000	.000	.000	.000	.000	.000	.000	.000	.000	.000	.000
a17_3	.000	.000	.000	.000	.000	.000	.000	.000	.000	.000	.000	.000	.000	.000	.000	.000	.000	.000	.000
a18_3	.000	.000	.000	.000	.000	.000	.000	.000	.000	.000	.000	.000	.000	.000	.000	.000	.000	.000	.000
ccc1_3	.000	.000	.000	.000	.000	.000	.000	.000	.000	.000	.000	.000	.000	.000	.000	.000	.000	.000	.000
ccc2_3	.000	.000	.000	.000	.000	.000	.000	.000	.000	.000	.000	.000	.000	.000	.000	.000	.000	.000	.000
ccc3_3	.000	.000	.000	.000	.000	.000	.000	.000	.000	.000	.000	.000	.000	.000	.000	.000	.000	.000	.000
ccc4_3	.000	.000	.000	.000	.000	.000	.000	.000	.000	.000	.000	.000	.000	.000	.000	.000	.000	.000	.000
ccc5_3	.000	.000	.000	.000	.000	.000	.000	.000	.000	.000	.000	.000	.000	.000	.000	.000	.000	.000	.000
ccc6_3	.000	.000	.000	.000	.000	.000	.000	.000	.000	.000	.000	.000	.000	.000	.000	.000	.000	.000	.000
ccc7_3	.000	.000	.000	.000	.000	.000	.000	.000	.000	.000	.000	.000	.000	.000	.000	.000	.000	.000	.000
ccc8_3	.000	.000	.000	.000	.000	.000	.000	.000	.000	.000	.000	.000	.000	.000	.000	.000	.000	.000	.000
ccc9_3	.000	.000	.000	.000	.000	.000	.000	.000	.000	.000	.000	.000	.000	.000	.000	.000	.000	.000	.000
ccc10_3	.000	.000	.000	.000	.000	.000	.000	.000	.000	.000	.000	.000	.000	.000	.000	.000	.000	.000	.000
ccc11_3	.000	.000	.000	.000	.000	.000	.000	.000	.000	.000	.000	.000	.000	.000	.000	.000	.000	.000	.000
ccc12_3	.000	.000	.000	.000	.000	.000	.000	.000	.000	.000	.000	.000	.000	.000	.000	.000	.000	.000	.000
ccc13_3	.000	.000	.000	.000	.000	.000	.000	.000	.000	.000	.000	.000	.000	.000	.000	.000	.000	.000	.000
ccc14_3	.000	.000	.000	.000	.000	.000	.000	.000	.000	.000	.000	.000	.000	.000	.000	.000	.000	.000	.000
ccc15_3	.000	.000	.000	.000	.000	.000	.000	.000	.000	.000	.000	.000	.000	.000	.000	.000	.000	.000	.000
vvv1_3	.000	.000	.000	.000	.000	.000	.000	.000	.000	.000	.000	.000	.000	.000	.000	.000	.000	.000	.000

	a1_1	a2_1	a3_1	a4_1	a5_1	a6_1	a7_1	a8_1	a9_1	a10_1	a11_1	a12_1	a13_1	a14_1	a15_1	a16_1	a17_1	a18_1	ccc1_1
v1_3	.000	.000	.000	.000	.000	.000	.000	.000	.000	.000	.000	.000	.000	.000	.000	.000	.000	.000	.000
v2_3	.000	.000	.000	.000	.000	.000	.000	.000	.000	.000	.000	.000	.000	.000	.000	.000	.000	.000	.000
v3_3	.000	.000	.000	.000	.000	.000	.000	.000	.000	.000	.000	.000	.000	.000	.000	.000	.000	.000	.000
v4_3	.000	.000	.000	.000	.000	.000	.000	.000	.000	.000	.000	.000	.000	.000	.000	.000	.000	.000	.000
vvv2_3	.000	.000	.000	.000	.000	.000	.000	.000	.000	.000	.000	.000	.000	.000	.000	.000	.000	.000	.000
v5_3	.000	.000	.000	.000	.000	.000	.000	.000	.000	.000	.000	.000	.000	.000	.000	.000	.000	.000	.000
v6_3	.000	.000	.000	.000	.000	.000	.000	.000	.000	.000	.000	.000	.000	.000	.000	.000	.000	.000	.000
v7_3	.000	.000	.000	.000	.000	.000	.000	.000	.000	.000	.000	.000	.000	.000	.000	.000	.000	.000	.000
v8_3	.000	.000	.000	.000	.000	.000	.000	.000	.000	.000	.000	.000	.000	.000	.000	.000	.000	.000	.000
vvv3_3	.000	.000	.000	.000	.000	.000	.000	.000	.000	.000	.000	.000	.000	.000	.000	.000	.000	.000	.000
v9_3	.000	.000	.000	.000	.000	.000	.000	.000	.000	.000	.000	.000	.000	.000	.000	.000	.000	.000	.000
v10_3	.000	.000	.000	.000	.000	.000	.000	.000	.000	.000	.000	.000	.000	.000	.000	.000	.000	.000	.000
v11_3	.000	.000	.000	.000	.000	.000	.000	.000	.000	.000	.000	.000	.000	.000	.000	.000	.000	.000	.000
v12_3	.000	.000	.000	.000	.000	.000	.000	.000	.000	.000	.000	.000	.000	.000	.000	.000	.000	.000	.000
vvv4_3	.000	.000	.000	.000	.000	.000	.000	.000	.000	.000	.000	.000	.000	.000	.000	.000	.000	.000	.000
v13_3	.000	.000	.000	.000	.000	.000	.000	.000	.000	.000	.000	.000	.000	.000	.000	.000	.000	.000	.000
v14_3	.000	.000	.000	.000	.000	.000	.000	.000	.000	.000	.000	.000	.000	.000	.000	.000	.000	.000	.000
v15_3	.000	.000	.000	.000	.000	.000	.000	.000	.000	.000	.000	.000	.000	.000	.000	.000	.000	.000	.000
v16_3	.000	.000	.000	.000	.000	.000	.000	.000	.000	.000	.000	.000	.000	.000	.000	.000	.000	.000	.000
vvv5_3	.000	.000	.000	.000	.000	.000	.000	.000	.000	.000	.000	.000	.000	.000	.000	.000	.000	.000	.000
v17_3	.000	.000	.000	.000	.000	.000	.000	.000	.000	.000	.000	.000	.000	.000	.000	.000	.000	.000	.000
v18_3	.000	.000	.000	.000	.000	.000	.000	.000	.000	.000	.000	.000	.000	.000	.000	.000	.000	.000	.000
v19_3	.000	.000	.000	.000	.000	.000	.000	.000	.000	.000	.000	.000	.000	.000	.000	.000	.000	.000	.000
v20_3	.000	.000	.000	.000	.000	.000	.000	.000	.000	.000	.000	.000	.000	.000	.000	.000	.000	.000	.000
vvv6_3	.000	.000	.000	.000	.000	.000	.000	.000	.000	.000	.000	.000	.000	.000	.000	.000	.000	.000	.000
v21_3	.000	.000	.000	.000	.000	.000	.000	.000	.000	.000	.000	.000	.000	.000	.000	.000	.000	.000	.000
v22_3	.000	.000	.000	.000	.000	.000	.000	.000	.000	.000	.000	.000	.000	.000	.000	.000	.000	.000	.000
v23_3	.000	.000	.000	.000	.000	.000	.000	.000	.000	.000	.000	.000	.000	.000	.000	.000	.000	.000	.000
v24_3	.000	.000	.000	.000	.000	.000	.000	.000	.000	.000	.000	.000	.000	.000	.000	.000	.000	.000	.000
i1_3	.000	.000	.000	.000	.000	.000	.000	.000	.000	.000	.000	.000	.000	.000	.000	.000	.000	.000	.000
i2_3	.000	.000	.000	.000	.000	.000	.000	.000	.000	.000	.000	.000	.000	.000	.000	.000	.000	.000	.000
i3_3	.000	.000	.000	.000	.000	.000	.000	.000	.000	.000	.000	.000	.000	.000	.000	.000	.000	.000	.000
i4_3	.000	.000	.000	.000	.000	.000	.000	.000	.000	.000	.000	.000	.000	.000	.000	.000	.000	.000	.000
i5_3	.000	.000	.000	.000	.000	.000	.000	.000	.000	.000	.000	.000	.000	.000	.000	.000	.000	.000	.000
i6_3	.000	.000	.000	.000	.000	.000	.000	.000	.000	.000	.000	.000	.000	.000	.000	.000	.000	.000	.000
i7_3	.000	.000	.000	.000	.000	.000	.000	.000	.000	.000	.000	.000	.000	.000	.000	.000	.000	.000	.000
i8_3	.000	.000	.000	.000	.000	.000	.000	.000	.000	.000	.000	.000	.000	.000	.000	.000	.000	.000	.000
i9_3	.000	.000	.000	.000	.000	.000	.000	.000	.000	.000	.000	.000	.000	.000	.000	.000	.000	.000	.000
i10_3	.000	.000	.000	.000	.000	.000	.000	.000	.000	.000	.000	.000	.000	.000	.000	.000	.000	.000	.000
i11_3	.000	.000	.000	.000	.000	.000	.000	.000	.000	.000	.000	.000	.000	.000	.000	.000	.000	.000	.000
i12_3	.000	.000	.000	.000	.000	.000	.000	.000	.000	.000	.000	.000	.000	.000	.000	.000	.000	.000	.000
i13_3	.000	.000	.000	.000	.000	.000	.000	.000	.000	.000	.000	.000	.000	.000	.000	.000	.000	.000	.000
i14_3	.000	.000	.000	.000	.000	.000	.000	.000	.000	.000	.000	.000	.000	.000	.000	.000	.000	.000	.000
i15_3	.000	.000	.000	.000	.000	.000	.000	.000	.000	.000	.000	.000	.000	.000	.000	.000	.000	.000	.000
i16_3	.000	.000	.000	.000	.000	.000	.000	.000	.000	.000	.000	.000	.000	.000	.000	.000	.000	.000	.000
i17_3	.000	.000	.000	.000	.000	.000	.000	.000	.000	.000	.000	.000	.000	.000	.000	.000	.000	.000	.000
i18_3	.000	.000	.000	.000	.000	.000	.000	.000	.000	.000	.000	.000	.000	.000	.000	.000	.000	.000	.000
i19_3	.000	.000	.000	.000	.000	.000	.000	.000	.000	.000	.000	.000	.000	.000	.000	.000	.000	.000	.000
i20_3	.000	.000	.000	.000	.000	.000	.000	.000	.000	.000	.000	.000	.000	.000	.000	.000	.000	.000	.000
i21_3	.000	.000	.000	.000	.000	.000	.000	.000	.000	.000	.000	.000	.000	.000	.000	.000	.000	.000	.000
i22_3	.000	.000	.000	.000	.000	.000	.000	.000	.000	.000	.000	.000	.000	.000	.000	.000	.000	.000	.000
i23_3	.000	.000	.000	.000	.000	.000	.000	.000	.000	.000	.000	.000	.000	.000	.000	.000	.000	.000	.000
i24_3	.000	.000	.000	.000	.000	.000	.000	.000	.000	.000	.000	.000	.000	.000	.000	.000	.000	.000	.000

Critical Ratios for Differences between Parameters (Unconstrained)

	a1_1	a2_1	a3_1	a4_1	a5_1	a6_1	a7_1	a8_1	a9_1	a10_1	a11_1	a12_1	a13_1	a14_1	a15_1	a1[illegible]
a1_1	.000															
a2_1	-3.326	.000														
a3_1	-5.298	-1.960	.000													
a4_1	1.894	2.050	2.157	.000												
a5_1	2.011	2.140	2.228	1.540	.000											
a6_1	1.853	2.018	2.132	-.526	-1.773	.000										
a7_1	-.098	2.396	4.194	-1.900	-2.016	-1.860	.000									
a8_1	.805	3.236	5.019	-1.844	-1.970	-1.800	1.401	.000								
a9_1	-2.996	-.337	1.473	-2.070	-2.157	-2.040	-3.836	-5.107	.000							
a10_1	.682	3.046	4.769	-1.850	-1.975	-1.806	.779	-.098	3.564	.000						
a11_1	1.361	3.613	5.268	-1.802	-1.935	-1.755	1.456	.608	4.130	1.088	.000					
a12_1	1.092	3.366	5.030	-1.821	-1.951	-1.775	1.187	.336	3.875	.660	-.407	.000				
a13_1	.135	2.716	4.594	-1.886	-2.005	-1.845	.238	-.706	3.280	-.580	-1.287	-1.008	.000			
a14_1	-1.352	1.300	3.171	-1.973	-2.076	-1.937	-1.254	-2.181	1.767	-2.004	-2.650	-2.380	-2.331	.000		

	a1_1	a2_1	a3_1	a4_1	a5_1	a6_1	a7_1	a8_1	a9_1	a10_1	a11_1	a12_1	a13_1	a14_1	a15_1	a16
**a15_1**	-2.213	.476	2.340	-2.022	-2.117	-1.989	-2.119	-3.034	.883	-2.827	-3.435	-3.170	-3.508	-1.223	.000	
**a16_1**	-1.843	.989	2.977	-1.995	-2.095	-1.961	-1.742	-2.715	1.470	-2.503	-3.150	-2.871	-2.100	-.433	.542	.00
**a17_1**	-.611	2.183	4.201	-1.928	-2.039	-1.889	-.504	-1.503	2.768	-1.337	-2.047	-1.757	-.799	.889	1.874	1.88
**a18_1**	-1.328	1.478	3.470	-1.967	-2.072	-1.931	-1.225	-2.205	1.999	-2.013	-2.685	-2.403	-1.555	.114	1.089	.75
**ccc1_1**	-13.983	-9.955	-7.924	-2.536	-2.541	-2.534	-13.606	-14.361	-10.608	-13.477	-13.400	-13.215	-14.976	-13.284	-12.235	-14.67
**ccc2_1**	-13.622	-9.826	-7.873	-2.549	-2.552	-2.548	-13.984	-14.779	-10.869	-13.483	-13.415	-13.230	-14.938	-13.262	-12.225	-14.59
**ccc3_1**	-3.716	-1.502	.080	-2.152	-2.224	-2.126	-3.715	-4.410	-1.297	-4.894	-5.398	-5.153	-4.744	-3.250	-2.375	-3.06
**ccc4_1**	-10.541	-7.207	-5.316	-2.370	-2.403	-2.358	-10.490	-11.299	-7.537	-10.229	-10.421	-10.222	-11.414	-9.800	-8.824	-10.35
**ccc5_1**	-15.199	-12.026	-10.407	-2.727	-2.699	-2.736	-17.989	-19.049	-14.399	-18.274	-18.081	-17.848	-16.193	-14.802	-13.957	-15.63
**ccc6_1**	-16.930	-12.562	-10.521	-2.701	-2.677	-2.709	-14.328	-14.996	-11.850	-16.785	-16.715	-16.488	-15.260	-13.884	-13.053	-14.57
**ccc7_1**	-13.642	-9.758	-7.768	-2.524	-2.531	-2.521	-13.621	-14.378	-10.600	-13.478	-13.392	-13.207	-15.311	-13.546	-12.451	-14.76
**ccc8_1**	-15.321	-12.397	-10.884	-2.800	-2.759	-2.814	-15.292	-15.924	-12.959	-19.089	-18.974	-18.717	-19.742	-17.644	-16.438	-15.53
**ccc9_1**	-4.312	-1.603	.189	-2.146	-2.219	-2.120	-3.633	-4.329	-1.207	-4.819	-5.327	-5.081	-3.994	-2.752	-2.005	-2.96
**ccc10_1**	-1.256	.693	2.151	-1.995	-2.095	-1.960	-1.424	-2.227	1.150	-2.080	-2.658	-2.418	-1.432	-.310	.358	-.01
**ccc11_1**	-15.673	-13.068	-11.699	-2.927	-2.864	-2.949	-15.642	-16.232	-13.519	-15.712	-15.767	-15.614	-20.537	-18.410	-17.218	-17.54
**ccc12_1**	-14.809	-11.823	-10.268	-2.750	-2.718	-2.761	-17.465	-18.517	-13.992	-14.804	-14.824	-14.660	-15.683	-14.350	-13.545	-16.46
**ccc13_1**	-.351	1.795	3.316	-1.918	-2.031	-1.878	-.268	-1.041	2.176	-.774	-1.264	-1.071	-.477	.776	1.498	1.04
**ccc14_1**	-9.150	-6.171	-4.408	-2.327	-2.367	-2.313	-9.090	-9.882	-6.313	-9.414	-9.691	-9.477	-9.796	-8.300	-7.409	-8.30
**ccc15_1**	-17.638	-13.311	-11.296	-2.793	-2.753	-2.806	-14.431	-15.062	-12.155	-14.521	-14.595	-14.432	-15.208	-13.955	-13.204	-15.78
**vvv1_1**	-.945	.613	1.795	-1.988	-2.089	-1.952	-1.131	-1.816	1.027	-1.706	-2.223	-2.012	-1.336	-.228	.409	.05
**v1_1**	.449	2.926	4.665	-1.867	-1.989	-1.824	.509	-.354	3.251	-.251	-.917	-.657	.303	1.706	2.517	2.18
**v2_1**	-2.650	-.217	1.603	-2.063	-2.151	-2.033	-2.792	-3.680	.129	-3.456	-4.028	-3.771	-3.161	-1.641	-.754	-1.33
**v3_1**	4.168	5.798	7.504	-1.563	-1.738	-1.502	4.259	3.504	6.577	3.517	2.846	3.085	4.174	5.343	6.011	5.83
**v4_1**	4.981	6.802	7.916	-1.484	-1.673	-1.418	5.069	4.335	7.310	4.335	3.668	3.903	5.001	6.129	6.771	6.61
**vvv2_1**	-12.145	-8.501	-6.538	-2.423	-2.447	-2.415	-12.108	-12.901	-9.082	-12.139	-12.183	-11.984	-13.278	-11.596	-10.564	-12.58
**v5_1**	6.001	7.701	9.007	-1.373	-1.582	-1.300	6.083	5.389	8.191	5.377	4.729	4.954	6.032	7.090	7.692	7.56
**v6_1**	2.794	4.638	5.998	-1.655	-1.821	-1.608	2.874	2.164	5.065	2.202	1.592	1.816	2.760	3.870	4.504	4.28
**v7_1**	1.407	3.181	4.457	-1.771	-1.899	-1.722	1.480	.818	3.530	.877	.328	.537	1.343	2.385	2.981	2.72
**v8_1**	2.867	4.746	6.138	-1.662	-1.821	-1.599	2.949	2.223	5.191	2.260	1.635	1.864	2.835	3.971	4.620	4.40
**vvv3_1**	-2.786	-.465	1.111	-2.081	-2.166	-2.051	-2.120	-2.695	-.148	-3.310	-3.842	-3.609	-3.007	-1.699	-.939	-1.42
**v9_1**	-3.124	-.300	1.622	-2.068	-2.155	-2.037	-3.393	-4.491	.066	-3.708	-4.286	-4.021	-3.445	-1.831	-.884	-1.52
**v10_1**	-3.601	-.765	1.146	-2.094	-2.176	-2.065	-3.188	-4.476	-.443	-4.160	-4.714	-4.453	-3.948	-2.343	-1.401	-2.08
**v11_1**	-4.863	-1.948	-.041	-2.160	-2.230	-2.135	-4.798	-5.058	-1.720	-5.357	-5.851	-5.598	-5.289	-3.682	-2.734	-3.53
**v12_1**	-.906	1.706	3.566	-1.948	-2.055	-1.910	-.808	-1.739	2.059	-1.574	-2.235	-1.964	-1.091	.458	1.359	.91
**vvv4_1**	-1.345	.632	2.002	-1.997	-2.097	-1.962	-1.271	-1.967	.923	-1.443	-1.844	-1.684	-1.484	-.356	.293	-.06
**v13_1**	.472	2.849	4.577	-1.864	-1.986	-1.821	.568	-.308	3.358	-.220	-.948	-.664	.361	1.789	2.614	2.27
**v14_1**	-.813	1.659	3.414	-1.944	-2.053	-1.907	-.720	-1.599	2.111	-1.357	-2.095	-1.834	-.981	.476	1.321	.90
**v15_1**	.890	3.107	4.719	-1.832	-1.960	-1.788	.981	.156	3.582	.242	-.386	-.162	.800	2.128	2.892	2.58
**v16_1**	1.076	3.285	4.896	-1.819	-1.950	-1.774	1.168	.343	3.768	.424	-.237	.015	.993	2.319	3.083	2.78
**vvv5_1**	-.865	1.103	2.477	-1.960	-2.066	-1.923	-.791	-1.491	1.413	-1.384	-1.918	-1.702	-.787	.112	.651	.44
**v17_1**	-.421	2.097	3.903	-1.920	-2.033	-1.880	-.324	-1.229	2.593	-1.092	-1.759	-1.490	-.610	.970	1.877	1.39
**v18_1**	-3.058	-.256	1.655	-2.065	-2.152	-2.035	-2.966	-3.891	.107	-3.643	-4.222	-3.957	-3.190	-1.769	-.830	-1.46
**v19_1**	-.711	1.856	3.689	-1.937	-2.047	-1.898	-.613	-1.529	2.347	-1.379	-2.042	-1.772	-.883	.613	1.527	1.10
**v20_1**	.388	2.832	4.610	-1.870	-1.991	-1.828	.486	-.413	3.361	-.304	-.993	-.723	.270	1.743	2.493	2.25
**vvv6_1**	1.950	3.604	4.797	-1.710	-1.860	-1.658	2.019	1.396	3.934	1.444	.918	1.116	1.898	2.870	3.425	2.77
**v21_1**	-1.891	.733	2.561	-2.006	-2.104	-1.972	-1.798	-2.700	1.148	-2.510	-3.125	-2.862	-2.122	-.597	.292	-.23
**v22_1**	1.782	3.968	5.582	-1.770	-1.909	-1.722	1.876	1.045	4.483	1.108	.426	.682	1.723	3.050	3.813	3.36
**v23_1**	.014	2.378	4.078	-1.893	-2.010	-1.852	.108	-.753	2.850	-.638	-1.292	-1.033	-.113	1.293	2.105	1.74
**v24_1**	1.455	3.664	5.288	-1.794	-1.929	-1.747	1.548	.714	4.170	.786	.110	.366	1.384	2.720	3.489	3.20
**i1_1**	39.404	41.107	44.035	.348	-.160	.525	39.713	37.891	44.794	36.686	34.087	34.693	41.261	43.599	44.876	47.50
**i2_1**	38.922	40.647	43.628	.285	-.212	.458	39.236	37.383	44.420	36.157	33.526	34.138	40.821	43.209	44.513	47.24
**i3_1**	35.068	36.855	39.582	.152	-.323	.316	35.343	33.674	40.035	32.664	30.350	30.904	36.614	38.792	39.987	42.11
**i4_1**	32.223	34.088	36.787	-.035	-.477	.118	32.489	30.853	37.125	29.916	27.700	28.239	33.669	35.833	37.024	39.01
**i5_1**	30.537	32.452	35.141	-.148	-.570	-.002	30.798	29.177	35.413	28.282	26.120	26.650	31.927	34.089	35.282	37.20
**i6_1**	24.330	26.356	28.802	-.463	-.830	-.335	24.550	23.117	28.695	22.467	20.665	21.131	25.340	27.310	28.406	29.72
**i7_1**	19.398	21.504	23.774	-.729	-1.049	-.617	19.588	18.289	23.399	17.818	16.279	16.698	20.137	21.972	22.999	23.90
**i8_1**	23.603	25.659	28.117	-.519	-.876	-.394	23.822	22.387	27.989	21.749	19.958	20.424	24.600	26.583	27.688	29.00
**i9_1**	41.058	42.752	45.967	.311	-.191	.485	41.408	39.388	47.054	37.984	35.092	35.752	43.268	45.860	47.272	50.58
**i10_1**	39.301	41.036	44.153	.240	-.249	.410	39.634	37.688	45.086	36.377	33.619	34.256	41.356	43.866	45.237	48.27
**i11_1**	40.200	41.907	45.021	.304	-.197	.477	40.534	38.586	45.980	37.258	34.477	35.117	42.275	44.778	46.143	49.19
**i12_1**	39.654	41.391	44.604	.217	-.269	.386	40.000	37.989	45.643	36.617	33.768	34.421	41.824	44.421	45.838	49.10
**i13_1**	17.894	20.126	22.521	-.877	-1.172	-.775	18.092	16.732	22.127	16.257	14.683	15.114	18.658	20.608	21.704	22.69
**i14_1**	11.412	13.759	15.935	-1.231	-1.464	-1.150	11.572	10.382	15.186	10.140	8.906	9.277	11.823	13.603	14.614	15.06
**i15_1**	14.667	16.870	19.023	-1.009	-1.281	-.915	14.834	13.634	18.420	13.311	11.985	12.367	15.187	16.936	17.923	18.50
**i16_1**	9.970	12.285	14.345	-1.296	-1.517	-1.218	10.116	8.999	13.521	8.818	7.691	8.039	10.283	11.968	12.926	13.21
**i17_1**	40.007	41.697	44.659	.372	-.141	.550	40.321	38.474	45.467	37.237	34.594	35.207	41.913	44.279	45.569	48.28
**i18_1**	42.220	43.857	46.882	.493	-.041	.678	42.546	40.646	47.818	39.329	36.572	37.205	44.248	46.662	47.975	50.88
**i19_1**	45.108	46.676	49.814	.633	.074	.827	45.455	43.464	50.948	42.023	39.091	39.755	47.330	49.833	51.191	54.41
**i20_1**	44.035	45.632	48.761	.562	.016	.752	44.379	42.400	49.853	40.986	38.090	38.747	46.221	48.720	50.078	53.25
**i21_1**	8.782	11.183	13.285	-1.382	-1.589	-1.310	8.928	7.799	12.401	7.644	6.536	6.885	9.062	10.788	11.772	12.02
**i22_1**	11.931	14.220	16.346	-1.184	-1.425	-1.100	12.089	10.922	15.617	10.676	9.451	9.817	12.342	14.077	15.061	15.49
**i23_1**	14.013	16.234	18.378	-1.050	-1.315	-.958	14.178	12.987	17.745	12.682	11.382	11.760	14.506	16.249	17.234	17.78

	a1_1	a2_1	a3_1	a4_1	a5_1	a6_1	a7_1	a8_1	a9_1	a10_1	a11_1	a12_1	a13_1	a14_1	a15_1	a16
**i24_1**	11.988	14.259	16.364	-1.174	-1.417	-1.090	12.144	10.989	15.636	10.745	9.530	9.893	12.392	14.110	15.083	15.50
**a1_2**	.501	2.754	4.383	-1.859	-1.983	-1.817	.592	-.238	3.219	-.142	-.791	-.538	.396	1.740	2.514	2.18
**a2_2**	-1.589	.761	2.397	-1.998	-2.097	-1.964	-1.504	-2.322	1.125	-2.168	-2.752	-2.507	-1.774	-.418	.368	-.07
**a3_2**	-1.945	.443	2.095	-2.020	-2.115	-1.987	-1.859	-2.682	.795	-2.516	-3.092	-2.847	-2.146	-.775	.020	-.44
**a4_2**	2.815	3.690	4.307	-1.380	-1.588	-1.308	2.851	2.526	3.835	2.552	2.268	2.376	2.784	3.283	3.566	3.43
**a5_2**	3.244	4.064	4.643	-1.266	-1.493	-1.187	3.277	2.972	4.201	2.995	2.728	2.829	3.215	3.683	3.949	3.82
**a6_2**	2.232	3.230	3.934	-1.535	-1.716	-1.472	2.272	1.902	3.396	1.933	1.613	1.735	2.194	2.764	3.089	2.93
**a7_2**	-.068	2.526	4.407	-1.898	-2.015	-1.858	.035	-.909	3.079	-.776	-1.476	-1.197	-.213	1.351	2.259	1.87
**a8_2**	.232	2.839	4.744	-1.881	-2.000	-1.839	.337	-.620	3.423	-.496	-1.214	-.931	.100	1.684	2.603	2.24
**a9_2**	-1.440	1.197	3.054	-1.979	-2.081	-1.943	-1.344	-2.263	1.653	-2.085	-2.724	-2.456	-1.652	-.103	.799	.31
**a10_2**	.006	2.631	4.539	-1.894	-2.011	-1.853	.111	-.846	3.202	-.714	-1.423	-1.142	-.138	1.451	2.374	1.99
**a11_2**	-.780	1.839	3.709	-1.940	-2.049	-1.902	-.680	-1.612	2.342	-1.456	-2.125	-1.851	-.960	.598	1.504	1.07
**a12_2**	-1.471	1.250	3.170	-1.978	-2.080	-1.942	-1.372	-2.319	1.730	-2.131	-2.782	-2.508	-1.695	-.090	.846	.35
**a13_2**	-.966	1.791	3.762	-1.948	-2.056	-1.911	-.862	-1.837	2.328	-1.662	-2.346	-2.063	-1.169	.480	1.441	.98
**a14_2**	-1.305	1.414	3.339	-1.968	-2.073	-1.932	-1.205	-2.156	1.907	-1.974	-2.633	-2.357	-1.520	.089	1.027	.54
**a15_2**	-2.147	.571	2.459	-2.017	-2.113	-1.984	-2.052	-2.978	.991	-2.769	-3.385	-3.117	-2.402	-.823	.099	-.44
**a16_2**	-.992	1.837	3.864	-1.949	-2.056	-1.911	-.886	-1.885	2.399	-1.703	-2.397	-2.109	-1.204	.494	1.487	1.01
**a17_2**	-2.310	.532	2.507	-2.021	-2.116	-1.988	-2.212	-3.173	.973	-2.944	-3.568	-3.293	-2.594	-.937	.034	-.54
**a18_2**	-2.992	-.152	1.789	-2.059	-2.148	-2.028	-2.898	-3.837	.225	-3.586	-4.172	-3.905	-3.309	-1.680	-.724	-1.35
**ccc1_2**	-14.118	-10.795	-9.070	-2.629	-2.618	-2.632	-14.089	-14.789	-11.439	-14.069	-14.061	-13.886	-15.156	-13.676	-12.774	-14.55
**ccc2_2**	-14.072	-10.751	-9.024	-2.626	-2.616	-2.630	-14.043	-14.744	-11.390	-14.026	-14.020	-13.845	-15.108	-13.626	-12.724	-14.49
**ccc3_2**	-2.440	-.079	1.537	-2.055	-2.144	-2.023	-2.358	-3.161	.235	-2.985	-3.536	-3.298	-2.655	-1.314	-.535	-1.01
**ccc4_2**	-4.647	-2.218	-.625	-2.197	-2.261	-2.174	-4.572	-5.348	-2.029	-5.111	-5.581	-5.355	-4.950	-3.621	-2.846	-3.45
**ccc5_2**	-15.286	-12.772	-11.432	-2.933	-2.869	-2.955	-15.252	-15.838	-13.168	-15.355	-15.437	-15.284	-15.938	-14.794	-14.113	-15.19
**ccc6_2**	-14.629	-11.956	-10.530	-2.829	-2.784	-2.845	-14.594	-15.212	-12.378	-14.693	-14.774	-14.613	-15.338	-14.119	-13.391	-14.57
**ccc7_2**	-13.980	-10.684	-8.964	-2.626	-2.616	-2.630	-13.950	-14.651	-11.305	-13.945	-13.948	-13.772	-14.997	-13.521	-12.624	-14.36
**ccc8_2**	-14.945	-12.417	-11.063	-2.902	-2.844	-2.922	-14.910	-15.503	-12.802	-15.023	-15.115	-14.958	-15.594	-14.439	-13.750	-14.83
**ccc9_2**	-1.309	.957	2.539	-1.982	-2.084	-1.947	-1.225	-2.021	1.317	-1.881	-2.461	-2.221	-1.475	-.166	.591	.17
**ccc10_2**	-1.153	1.036	2.567	-1.974	-2.078	-1.938	-1.072	-1.844	1.386	-1.715	-2.285	-2.051	-1.307	-.043	.687	.29
**ccc11_2**	-15.356	-12.994	-11.722	-3.000	-2.925	-3.026	-15.321	-15.886	-13.339	-15.448	-15.551	-15.401	-15.941	-14.858	-14.215	-15.19
**ccc12_2**	-14.602	-12.041	-10.663	-2.865	-2.813	-2.883	-14.566	-15.171	-12.421	-14.686	-14.785	-14.626	-15.258	-14.082	-13.382	-14.47
**ccc13_2**	1.732	3.544	4.856	-1.746	-1.889	-1.696	1.808	1.127	3.916	1.182	.612	.827	1.674	2.745	3.359	3.10
**ccc14_2**	-4.777	-2.330	-.728	-2.203	-2.267	-2.181	-4.703	-5.481	-2.147	-5.238	-5.705	-5.479	-5.089	-3.752	-2.972	-3.59
**ccc15_2**	-14.285	-11.642	-10.219	-2.815	-2.772	-2.830	-14.249	-14.870	-12.035	-14.367	-14.465	-14.302	-14.969	-13.755	-13.031	-14.17
**vvv1_2**	-2.136	-.168	1.177	-2.061	-2.149	-2.030	-2.065	-2.745	.087	-2.616	-3.111	-2.903	-2.293	-1.184	-.545	-.93
**v1_2**	3.180	5.220	6.753	-1.659	-1.817	-1.604	3.272	2.472	5.749	2.503	1.814	2.064	3.162	4.416	5.134	4.92
**v2_2**	-2.598	.116	1.983	-2.043	-2.135	-2.012	-2.506	-3.417	.498	-3.195	-3.788	-3.525	-2.874	-1.312	-.400	-.97
**v3_2**	2.420	4.585	6.202	-1.725	-1.872	-1.674	2.515	1.678	5.130	1.726	1.025	1.284	2.382	3.709	4.471	4.23
**v4_2**	2.324	4.511	6.143	-1.733	-1.879	-1.683	2.420	1.576	5.059	1.627	.923	1.183	2.283	3.624	4.394	4.15
**vvv2_2**	-6.883	-4.320	-2.714	-2.327	-2.369	-2.312	-6.816	-7.576	-4.265	-7.262	-7.649	-7.433	-7.296	-5.948	-5.157	-5.92
**v5_2**	5.700	7.391	8.683	-1.390	-1.595	-1.318	5.781	5.095	7.865	5.088	4.452	4.674	5.724	6.771	7.366	7.22
**v6_2**	4.288	5.951	7.192	-1.503	-1.689	-1.439	4.363	3.709	6.361	3.724	3.137	3.348	4.281	5.288	5.863	5.68
**v7_2**	1.888	3.743	5.093	-1.737	-1.882	-1.686	1.966	1.265	4.137	1.318	.731	.952	1.833	2.935	3.567	3.31
**v8_2**	2.677	4.640	6.095	-1.687	-1.840	-1.633	2.763	2.005	5.110	2.046	1.400	1.638	2.643	3.832	4.513	4.28
**vvv3_2**	-.189	1.660	2.961	-1.907	-2.022	-1.867	-.117	-.785	1.969	-.698	-1.223	-1.015	-.291	.775	1.388	1.08
**v9_2**	-.183	2.258	4.012	-1.905	-2.021	-1.865	-.087	-.971	2.744	-.846	-1.508	-1.244	-.323	1.131	1.972	1.59
**v10_2**	-2.316	.353	2.197	-2.029	-2.123	-1.997	-2.223	-3.128	.746	-2.920	-3.521	-3.259	-2.571	-1.031	-.131	-.67
**v11_2**	-4.245	-1.368	.541	-2.128	-2.204	-2.101	-4.158	-5.069	-1.097	-4.772	-5.295	-5.038	-4.632	-3.025	-2.080	-2.82
**v12_2**	.625	2.977	4.689	-1.853	-1.978	-1.810	.721	-.149	3.486	-.051	-.729	-.466	.521	1.936	2.752	2.42
**vvv4_2**	1.714	3.231	4.315	-1.716	-1.864	-1.664	1.777	1.211	3.514	1.259	.780	.962	1.661	2.543	3.046	2.82
**v13_2**	1.538	3.708	5.302	-1.786	-1.922	-1.738	1.630	.810	4.202	.879	.211	.463	1.471	2.781	3.534	3.25
**v14_2**	.437	2.690	4.317	-1.864	-1.986	-1.821	.528	-.301	3.151	-.203	-.849	-.597	.330	1.672	2.446	2.11
**v15_2**	3.129	5.077	6.532	-1.651	-1.811	-1.595	3.216	2.457	5.563	2.488	1.832	2.071	3.107	4.294	4.974	4.76
**v16_2**	1.612	3.794	5.399	-1.781	-1.918	-1.734	1.705	.879	4.297	.946	.272	.526	1.547	2.867	3.626	3.35
**vvv5_2**	.373	2.171	3.446	-1.859	-1.983	-1.816	.444	-.213	2.488	-.137	-.665	-.459	.286	1.329	1.927	1.64
**v17_2**	-.422	2.071	3.856	-1.920	-2.033	-1.881	-.325	-1.222	2.558	-1.086	-1.748	-1.481	-.575	.907	1.766	1.37
**v18_2**	-2.550	.208	2.108	-2.038	-2.130	-2.006	-2.456	-3.383	.604	-3.157	-3.757	-3.490	-2.831	-1.240	-.309	-.89
**v19_2**	-.008	2.441	4.208	-1.894	-2.012	-1.854	.089	-.802	2.943	-.681	-1.353	-1.087	-.142	1.323	2.171	1.80
**v20_2**	2.100	4.292	5.920	-1.749	-1.892	-1.699	2.196	1.355	4.829	1.411	.715	.974	2.052	3.390	4.159	3.91
**vvv6_2**	2.993	4.434	5.478	-1.572	-1.746	-1.512	3.055	2.506	4.728	2.539	2.060	2.238	2.959	3.805	4.288	4.09
**v21_2**	-.649	1.715	3.392	-1.936	-2.046	-1.897	-.559	-1.403	2.144	-1.271	-1.897	-1.644	-.801	.588	1.391	.99
**v22_2**	.509	2.730	4.333	-1.858	-1.982	-1.815	.599	-.219	3.183	-.125	-.765	-.516	.406	1.727	2.489	2.16
**v23_2**	3.381	5.304	6.743	-1.628	-1.792	-1.571	3.467	2.714	5.790	2.741	2.085	2.323	3.364	4.539	5.210	5.00
**v24_2**	4.690	6.458	7.797	-1.495	-1.682	-1.430	4.772	4.065	6.931	4.071	3.433	3.659	4.697	5.784	6.404	6.24
**i1_2**	37.766	39.490	42.279	.311	-.191	.485	38.054	36.331	42.867	35.235	32.799	33.374	39.441	41.663	42.878	45.19
**i2_2**	38.969	40.693	43.678	.286	-.212	.459	39.284	37.427	44.476	36.198	33.562	34.175	40.874	43.265	44.571	47.31
**i3_2**	35.057	36.866	39.685	.101	-.364	.263	35.343	33.615	40.210	32.558	30.168	30.738	36.691	38.950	40.189	42.48
**i4_2**	35.472	37.277	40.135	.110	-.357	.272	35.764	34.009	40.706	32.923	30.490	31.068	37.155	39.447	40.703	43.08
**i5_2**	27.361	29.326	31.866	-.296	-.692	-.158	27.598	26.089	31.925	25.332	23.375	23.869	28.533	30.575	31.706	33.27
**i6_2**	21.386	23.395	25.611	-.563	-.912	-.441	21.575	20.298	25.292	19.800	18.244	18.662	22.150	23.931	24.925	25.84
**i7_2**	17.764	19.871	22.049	-.804	-1.112	-.697	17.940	16.706	21.579	16.302	14.871	15.269	18.396	20.156	21.144	21.88
**i8_2**	22.971	25.031	27.452	-.546	-.898	-.423	23.185	21.775	27.280	21.167	19.421	19.878	23.921	25.874	26.962	28.19

	a1_1	a2_1	a3_1	a4_1	a5_1	a6_1	a7_1	a8_1	a9_1	a10_1	a11_1	a12_1	a13_1	a14_1	a15_1	a16
**i9_2**	39.031	40.737	43.621	.347	-.162	.523	39.333	37.542	44.329	36.369	33.818	34.415	40.832	43.133	44.390	46.92
**i10_2**	38.194	39.920	42.786	.297	-.203	.471	38.492	36.717	43.453	35.570	33.057	33.647	39.956	42.244	43.495	45.97
**i11_2**	39.501	41.202	44.142	.348	-.160	.525	39.811	37.981	44.915	36.766	34.155	34.763	41.371	43.720	45.002	47.66
**i12_2**	36.895	38.653	41.489	.222	-.265	.391	37.187	35.439	42.088	34.335	31.882	32.462	38.594	40.860	42.101	44.48
**i13_2**	17.343	19.480	21.682	-.843	-1.144	-.739	17.521	16.275	21.203	15.874	14.439	14.839	17.976	19.758	20.760	21.50
**i14_2**	11.541	13.774	15.811	-1.184	-1.425	-1.100	11.689	10.575	15.057	10.357	9.193	9.545	11.902	13.560	14.501	14.84
**i15_2**	13.909	16.072	18.131	-1.028	-1.297	-.935	14.066	12.923	17.481	12.642	11.395	11.760	14.359	16.029	16.973	17.44
**i16_2**	10.697	13.001	15.084	-1.254	-1.483	-1.174	10.847	9.713	14.294	9.508	8.345	8.700	11.045	12.747	13.714	14.05
**i17_2**	38.137	39.860	42.698	.309	-.193	.483	38.432	36.675	43.340	35.545	33.057	33.642	39.870	42.134	43.372	45.79
**i18_2**	42.384	44.017	47.043	.504	-.032	.690	42.711	40.810	47.984	39.489	36.727	37.361	44.418	46.831	48.145	51.05
**i19_2**	42.718	44.332	47.292	.567	.020	.757	43.037	41.177	48.180	39.889	37.179	37.802	44.687	47.040	48.320	51.09
**i20_2**	41.435	43.076	45.975	.511	-.026	.697	41.744	39.931	46.770	38.702	36.079	36.686	43.310	45.615	46.870	49.49
**i21_2**	9.386	11.698	13.727	-1.326	-1.542	-1.251	9.528	8.433	12.875	8.273	7.180	7.522	9.667	11.326	12.271	12.50
**i22_2**	11.866	14.096	16.145	-1.165	-1.410	-1.080	12.016	10.893	15.406	10.664	9.484	9.839	12.242	13.910	14.856	15.22
**i23_2**	14.358	16.536	18.639	-1.013	-1.284	-.918	14.519	13.349	18.014	13.047	11.761	12.134	14.843	16.549	17.512	18.03
**i24_2**	13.053	15.252	17.317	-1.090	-1.347	-1.000	13.208	12.067	16.632	11.805	10.580	10.942	13.478	15.156	16.105	16.54
**a1_3**	.607	2.872	4.514	-1.853	-1.977	-1.809	.699	-.138	3.347	-.043	-.699	-.444	.506	1.860	2.640	2.31
**a2_3**	-1.758	.618	2.267	-2.008	-2.106	-1.974	-1.672	-2.495	.978	-2.335	-2.916	-2.671	-1.951	-.584	.210	-.24
**a3_3**	-2.119	.173	1.750	-2.037	-2.129	-2.005	-2.038	-2.825	.492	-2.665	-3.217	-2.982	-2.315	-1.008	-.251	-.70
**a4_3**	2.182	2.777	3.192	-1.287	-1.511	-1.209	2.205	1.987	2.866	2.008	1.820	1.892	2.156	2.491	2.682	2.58
**a5_3**	3.280	3.689	3.973	-.611	-.942	-.498	3.296	3.146	3.749	3.160	3.031	3.080	3.262	3.492	3.622	3.55
**a6_3**	2.932	3.402	3.729	-.884	-1.174	-.785	2.950	2.778	3.471	2.794	2.644	2.702	2.911	3.176	3.326	3.24
**a7_3**	.677	3.029	4.743	-1.850	-1.975	-1.806	.773	-.099	3.542	-.002	-.682	-.419	.575	1.991	2.809	2.48
**a8_3**	2.073	4.313	5.981	-1.754	-1.896	-1.705	2.170	1.310	4.870	1.368	.659	.922	2.024	3.396	4.186	3.93
**a9_3**	-2.250	.289	2.041	-2.032	-2.125	-1.999	-2.161	-3.027	.656	-2.836	-3.421	-3.168	-2.484	-1.025	-.175	-.68
**a10_3**	1.314	3.601	5.284	-1.807	-1.939	-1.761	1.410	.548	4.130	.626	-.065	.198	1.237	2.624	3.424	3.13
**a11_3**	1.700	3.972	5.654	-1.781	-1.918	-1.733	1.797	.933	4.519	1.001	.298	.562	1.638	3.024	3.821	3.55
**a12_3**	.625	2.925	4.596	-1.852	-1.977	-1.809	.718	-.133	3.415	-.037	-.702	-.444	.523	1.901	2.697	2.37
**a13_3**	.491	2.940	4.725	-1.864	-1.986	-1.821	.591	-.313	3.478	-.206	-.901	-.630	.378	1.857	2.712	2.37
**a14_3**	.757	3.204	4.999	-1.847	-1.973	-1.803	.858	-.053	3.762	.047	-.660	-.387	.655	2.141	3.000	2.67
**a15_3**	-1.565	.958	2.724	-1.990	-2.090	-1.955	-1.473	-2.351	1.370	-2.181	-2.795	-2.537	-1.769	-.300	.554	.08
**a16_3**	-1.103	1.740	3.773	-1.954	-2.061	-1.917	-.997	-1.998	2.297	-1.810	-2.500	-2.213	-1.323	.382	1.378	.89
**a17_3**	-1.203	1.696	3.768	-1.959	-2.065	-1.922	-1.096	-2.112	2.264	-1.915	-2.608	-2.318	-1.435	.305	1.324	.82
**a18_3**	-2.726	.164	2.154	-2.042	-2.133	-2.010	-2.630	-3.592	.578	-3.343	-3.949	-3.675	-3.039	-1.367	-.386	-1.01
**ccc1_3**	-13.933	-10.355	-8.512	-2.570	-2.569	-2.570	-13.907	-14.633	-11.091	-13.831	-13.786	-13.606	-15.119	-13.537	-12.565	-14.64
**ccc2_3**	-13.986	-10.321	-8.444	-2.558	-2.559	-2.557	-13.963	-14.694	-11.101	-13.859	-13.794	-13.615	-15.233	-13.621	-12.628	-14.83
**ccc3_3**	-3.540	-.978	.743	-2.111	-2.190	-2.083	-3.457	-4.297	-.700	-4.068	-4.598	-4.354	-3.829	-2.392	-1.554	-2.14
**ccc4_3**	-7.056	-4.297	-2.578	-2.308	-2.353	-2.292	-6.988	-7.790	-4.259	-7.431	-7.814	-7.589	-7.536	-6.088	-5.233	-6.10
**ccc5_3**	-14.914	-11.928	-10.377	-2.757	-2.723	-2.768	-14.883	-15.531	-12.490	-14.904	-14.920	-14.756	-15.791	-14.461	-13.658	-15.12
**ccc6_3**	-13.974	-10.923	-9.311	-2.681	-2.661	-2.687	-13.940	-14.616	-11.446	-13.992	-14.038	-13.866	-14.851	-13.470	-12.637	-14.12
**ccc7_3**	-13.936	-10.358	-8.516	-2.570	-2.569	-2.570	-13.911	-14.637	-11.095	-13.835	-13.789	-13.610	-15.123	-13.541	-12.569	-14.65
**ccc8_3**	-15.023	-12.146	-10.644	-2.794	-2.754	-2.807	-14.992	-15.625	-12.670	-15.032	-15.063	-14.902	-15.846	-14.559	-13.784	-15.16
**ccc9_3**	-3.393	-.825	.905	-2.101	-2.182	-2.073	-3.310	-4.155	-.536	-3.929	-4.467	-4.221	-3.678	-2.233	-1.390	-1.97
**ccc10_3**	-3.079	-.575	1.123	-2.086	-2.170	-2.057	-2.996	-3.830	-.274	-3.621	-4.165	-3.921	-3.340	-1.926	-1.102	-1.65
**ccc11_3**	-15.584	-13.191	-11.910	-3.004	-2.928	-3.030	-15.551	-16.116	-13.557	-15.664	-15.756	-15.607	-16.189	-15.097	-14.448	-15.45
**ccc12_3**	-14.772	-12.109	-10.691	-2.843	-2.795	-2.859	-14.737	-15.351	-12.535	-14.832	-14.909	-14.749	-15.480	-14.268	-13.544	-14.72
**ccc13_3**	1.267	3.111	4.438	-1.786	-1.923	-1.739	1.343	.656	3.476	.719	.154	.370	1.198	2.283	2.905	2.64
**ccc14_3**	-5.621	-3.179	-1.607	-2.261	-2.314	-2.242	-5.551	-6.309	-3.047	-6.046	-6.480	-6.261	-5.960	-4.647	-3.879	-4.53
**ccc15_3**	-13.994	-11.323	-9.880	-2.786	-2.747	-2.799	-13.956	-14.587	-11.710	-14.082	-14.186	-14.021	-14.682	-13.451	-12.716	-13.87
**vvv1_3**	-2.401	-.306	1.122	-2.071	-2.158	-2.041	-2.327	-3.044	-.040	-2.898	-3.408	-3.190	-2.580	-1.400	-.718	-1.13
**v1_3**	.653	2.996	4.701	-1.851	-1.976	-1.808	.748	-.119	3.503	-.022	-.699	-.436	.550	1.959	2.772	2.44
**v2_3**	-1.227	1.260	3.011	-1.970	-2.074	-1.934	-1.135	-2.009	1.686	-1.851	-2.475	-2.217	-1.413	.041	.886	.44
**v3_3**	2.605	4.715	6.291	-1.707	-1.857	-1.655	2.698	1.881	5.245	1.924	1.234	1.487	2.572	3.864	4.605	4.37
**v4_3**	5.177	6.927	8.261	-1.451	-1.646	-1.383	5.259	4.554	7.411	4.553	3.907	4.134	5.194	6.276	6.892	6.74
**vvv2_3**	-9.695	-6.572	-4.753	-2.410	-2.437	-2.400	-9.639	-10.441	-6.782	-9.918	-10.158	-9.944	-10.427	-8.880	-7.953	-9.23
**v5_3**	5.648	7.373	8.694	-1.405	-1.607	-1.334	5.731	5.029	7.863	5.022	4.373	4.600	5.674	6.745	7.354	7.21
**v6_3**	5.861	7.457	8.670	-1.343	-1.557	-1.269	5.936	5.291	7.891	5.286	4.687	4.897	5.879	6.861	7.419	7.27
**v7_3**	-.176	1.866	3.313	-1.906	-2.021	-1.865	-.097	-.835	2.227	-.736	-1.309	-1.082	-.290	.899	1.584	1.25
**v8_3**	3.540	5.346	6.691	-1.597	-1.766	-1.538	3.621	2.915	5.788	2.940	2.321	2.545	3.523	4.618	5.244	5.04
**vvv3_3**	-2.299	-.161	1.300	-2.060	-2.149	-2.029	-2.224	-2.957	.117	-2.808	-3.329	-3.107	-2.481	-1.273	-.574	-1.00
**v9_3**	-.288	2.203	3.992	-1.912	-2.026	-1.872	-.191	-1.090	2.699	-.958	-1.626	-1.359	-.436	1.049	1.909	1.52
**v10_3**	-2.838	-.081	1.806	-2.055	-2.144	-2.024	-2.745	-3.663	.290	-3.428	-4.014	-3.750	-3.132	-1.551	-.626	-1.22
**v11_3**	-4.840	-1.952	-.062	-2.161	-2.231	-2.136	-4.757	-5.652	-1.732	-5.334	-5.827	-5.576	-5.259	-3.667	-2.728	-3.51
**v12_3**	.378	2.815	4.586	-1.870	-1.992	-1.828	.477	-.420	3.340	-.310	-.997	-.728	.261	1.728	2.576	2.23
**vvv4_3**	-.007	1.708	2.913	-1.891	-2.009	-1.849	.060	-.561	1.990	-.484	-.980	-.785	-.097	.888	1.453	1.17
**v13_3**	1.937	4.084	5.672	-1.757	-1.898	-1.708	2.029	1.210	4.593	1.269	.592	.845	1.882	3.186	3.935	3.67
**v14_3**	1.909	3.950	5.449	-1.751	-1.893	-1.701	1.996	1.220	4.413	1.277	.634	.875	1.854	3.082	3.787	3.53
**v15_3**	.979	3.109	4.655	-1.823	-1.953	-1.778	1.066	.273	3.557	.353	-.283	-.039	.894	2.165	2.896	2.59
**v16_3**	3.742	5.577	6.951	-1.586	-1.757	-1.526	3.825	3.104	6.040	3.125	2.490	2.719	3.731	4.850	5.489	5.29
**vvv5_3**	-1.488	.574	2.003	-2.004	-2.102	-1.970	-1.412	-2.134	.877	-2.009	-2.542	-2.322	-1.639	-.461	.218	-.16
**v17_3**	-.883	1.693	3.526	-1.947	-2.055	-1.909	-.786	-1.699	2.173	-1.544	-2.200	-1.931	-1.063	.462	1.348	.91
**v18_3**	-1.081	1.468	3.272	-1.959	-2.065	-1.923	-.986	-1.885	1.924	-1.726	-2.367	-2.102	-1.268	.233	1.105	.66

	a1_1	a2_1	a3_1	a4_1	a5_1	a6_1	a7_1	a8_1	a9_1	a10_1	a11_1	a12_1	a13_1	a14_1	a15_1	a16
**v19_3**	-1.990	.659	2.503	-2.011	-2.108	-1.977	-1.897	-2.805	1.074	-2.609	-3.223	-2.959	-2.229	-.690	.208	-.31
**v20_3**	2.154	4.336	5.959	-1.745	-1.888	-1.695	2.249	1.411	4.872	1.466	.770	1.029	2.108	3.440	4.206	3.96
**vvv6_3**	3.521	4.858	5.828	-1.487	-1.675	-1.421	3.578	3.067	5.131	3.094	2.642	2.809	3.492	4.277	4.725	4.54
**v21_3**	.475	2.757	4.408	-1.862	-1.985	-1.819	.567	-.274	3.230	-.175	-.829	-.575	.368	1.730	2.516	2.18
**v22_3**	1.891	3.992	5.540	-1.757	-1.898	-1.708	1.980	1.181	4.478	1.240	.579	.826	1.835	3.105	3.834	3.57
**v23_3**	-.083	2.277	3.971	-1.899	-2.015	-1.858	.010	-.846	2.742	-.729	-1.378	-1.121	-.213	1.187	1.997	1.63
**v24_3**	4.104	5.965	7.370	-1.562	-1.737	-1.501	4.190	3.452	6.456	3.466	2.810	3.045	4.104	5.247	5.900	5.72
**i1_3**	36.099	37.890	40.770	.142	-.331	.306	36.395	34.621	41.382	33.510	31.040	31.624	37.822	40.131	41.397	43.84
**i2_3**	34.437	36.262	39.066	.065	-.394	.225	34.721	33.005	39.560	31.968	29.607	30.172	36.041	38.290	39.525	41.77
**i3_3**	33.732	35.572	38.350	.030	-.423	.187	34.011	32.316	38.798	31.308	28.987	29.545	35.292	37.520	38.744	40.92
**i4_3**	32.020	33.860	36.457	.006	-.443	.162	32.273	30.704	36.714	29.822	27.696	28.217	33.368	35.442	36.584	38.39
**i5_3**	31.172	33.064	35.745	-.099	-.530	.050	31.433	29.815	36.033	28.910	26.737	27.269	32.572	34.724	35.910	37.84
**i6_3**	22.874	24.879	27.178	-.496	-.857	-.371	23.075	21.739	26.947	21.179	19.524	19.961	23.736	25.584	26.613	27.68
**i7_3**	17.618	19.741	21.938	-.822	-1.126	-.716	17.796	16.552	21.468	16.145	14.706	15.107	18.256	20.033	21.031	21.78
**i8_3**	24.234	26.202	28.515	-.405	-.782	-.274	24.439	23.088	28.339	22.493	20.792	21.236	25.144	26.999	28.030	29.16
**i9_3**	38.264	40.008	42.982	.243	-.247	.413	38.576	36.730	43.746	35.522	32.915	33.523	40.139	42.524	43.827	46.52
**i10_3**	37.099	38.867	41.786	.191	-.290	.358	37.402	35.598	42.464	34.444	31.915	32.509	38.887	41.227	42.507	45.06
**i11_3**	37.298	39.048	41.906	.238	-.251	.408	37.593	35.828	42.537	34.704	32.221	32.806	39.029	41.314	42.563	45.00
**i12_3**	39.715	41.431	44.507	.290	-.209	.463	40.043	38.122	45.414	36.827	34.092	34.723	41.736	44.206	45.554	48.51
**i13_3**	19.478	21.603	23.912	-.739	-1.058	-.628	19.672	18.350	23.555	17.863	16.296	16.721	20.243	22.112	23.159	24.11
**i14_3**	13.998	16.137	18.169	-1.012	-1.283	-.918	14.152	13.024	17.519	12.748	11.515	11.876	14.438	16.085	17.014	17.46
**i15_3**	15.298	17.410	19.468	-.930	-1.215	-.830	15.458	14.308	18.874	13.994	12.710	13.080	15.791	17.455	18.393	18.91
**i16_3**	12.921	15.070	17.065	-1.075	-1.335	-.984	13.070	11.969	16.368	11.726	10.545	10.897	13.311	14.929	15.844	16.22
**i17_3**	36.985	38.758	41.683	.180	-.299	.346	37.289	35.482	42.362	34.328	31.797	32.392	38.776	41.121	42.405	44.96
**i18_3**	37.594	39.329	42.153	.279	-.218	.451	37.886	36.142	42.764	35.030	32.570	33.150	39.298	41.551	42.783	45.16
**i19_3**	40.282	41.955	44.855	.427	-.096	.608	40.589	38.780	45.617	37.573	34.977	35.580	42.131	44.441	45.701	48.30
**i20_3**	40.606	42.272	45.185	.443	-.083	.625	40.915	39.097	45.968	37.876	35.260	35.867	42.476	44.797	46.062	48.69
**i21_3**	11.941	14.142	16.157	-1.150	-1.397	-1.064	12.089	10.983	15.420	10.759	9.595	9.946	12.307	13.946	14.874	15.22
**i22_3**	14.498	16.621	18.653	-.977	-1.254	-.880	14.654	13.523	18.023	13.234	11.987	12.350	14.955	16.600	17.528	18.00
**i23_3**	14.672	16.834	18.931	-.989	-1.264	-.893	14.833	13.665	18.318	13.356	12.065	12.438	15.164	16.864	17.823	18.35
**i24_3**	13.060	15.241	17.282	-1.081	-1.340	-.991	13.213	12.086	16.595	11.829	10.618	10.977	13.475	15.132	16.070	16.48

Bootstrap (Unconstrained)

Summary of Bootstrap Iterations (Unconstrained)

(Unconstrained)

Iterations	Method 0	Method 1	Method 2
**1**	0	0	0
**2**	0	0	0
**3**	0	0	0
**4**	0	0	0
**5**	0	0	0
**6**	0	0	0
**7**	0	0	0
**8**	0	0	0
**9**	0	0	1
**10**	0	0	1
**11**	0	0	0
**12**	0	0	1
**13**	0	0	3
**14**	0	0	1
**15**	0	0	2
**16**	0	0	2
**17**	0	1	2
**18**	0	1	1
**19**	0	164	20
**Total**	0	166	34

0 bootstrap samples were unused because of a singular covariance matrix.
14 bootstrap samples were unused because a solution was not found.
200 usable bootstrap samples were obtained.

Bootstrap Distributions (Unconstrained)

ML discrepancy (implied vs sample) (Unconstrained)

	|--------------------
2821.912	|**
2887.804	|*

```
                    2953.695  |***
                    3019.587  |*******
                    3085.479  |*********
                    3151.370  |*******
                    3217.262  |**************
N = 200             3283.154  |***********
Mean = 3206.141     3349.045  |*******
S. e. = 11.544      3414.937  |****
                    3480.829  |***
                    3546.720  |*
                    3612.612  |*
                    3678.504  |*
                    3744.395  |*
                              |--------------------
```

ML discrepancy (implied vs pop) (Unconstrained)

```
                              |--------------------
                    2533.966  |**
                    2553.481  |******
                    2572.997  |*********
                    2592.512  |*********
                    2612.027  |***************
                    2631.542  |**************
                    2651.057  |**************
N = 200             2670.573  |**********
Mean = 2639.209     2690.088  |***********
S. e. = 3.872       2709.603  |*****
                    2729.118  |****
                    2748.634  |***
                    2768.149  |*
                    2787.664  |
                    2807.179  |**
                              |--------------------
```

K-L overoptimism (unstabilized) (Unconstrained)

```
                              |--------------------
                   -1361.555  |*
                   -1032.183  |*
                    -702.812  |*
                    -373.441  |*****
                     -44.069  |*****
                     285.302  |***********
                     614.674  |************
N = 200              944.045  |**************
Mean = 787.684      1273.417  |******
S. e. = 51.599      1602.788  |*******
                    1932.160  |****
                    2261.531  |**
                    2590.903  |*
                    2920.274  |
                    3249.645  |*
                              |--------------------
```

K-L overoptimism (stabilized) (Unconstrained)

```
                              |--------------------
                     351.591  |**
                     423.382  |**
                     495.173  |***
                     566.964  |******
                     638.756  |******
                     710.547  |***********
                     782.338  |***************
N = 200              854.129  |********
Mean = 775.769       925.921  |*******
S. e. = 12.695       997.712  |****
                    1069.503  |****
                    1141.294  |**
                    1213.086  |*
                    1284.877  |
```

1356.668	|*
	|--------------------

Measurement weights (Measurement weights)

Notes for Model (Measurement weights)

Computation of degrees of freedom (Measurement weights)

**Number of distinct sample moments:**	972
**Number of distinct parameters to be estimated:**	225
**Degrees of freedom (972 - 225):**	747

Result (Measurement weights)

Minimum was achieved
Chi-square = 2309.884
Degrees of freedom = 747
Probability level = .000

g1 (g1 - Measurement weights)

Estimates (g1 - Measurement weights)

Scalar Estimates (g1 - Measurement weights)

Maximum Likelihood Estimates

Regression Weights: (g1 - Measurement weights)

			Estimate	S.E.	C.R.	P	Label
BPNSF19	<---	F1	1.000				
BPNSF13	<---	F1	1.146	.052	21.901	***	a1_1
BPNSF7	<---	F1	.872	.051	16.991	***	a2_1
BPNSF1	<---	F1	.774	.051	15.177	***	a3_1
BPNSF18	<---	F2	1.000				
BPNSF15	<---	F2	2.522	.319	7.905	***	a4_1
BPNSF10	<---	F2	3.235	.395	8.191	***	a5_1
BPNSF5	<---	F2	2.579	.322	8.003	***	a6_1
BPNSF24	<---	F3	1.000				
BPNSF16	<---	F3	1.125	.047	24.171	***	a7_1
BPNSF12	<---	F3	1.221	.048	25.585	***	a8_1
BPNSF4	<---	F3	.861	.044	19.667	***	a9_1
BPNSF22	<---	F4	1.000				
BPNSF20	<---	F4	1.188	.049	24.383	***	a10_1
BPNSF8	<---	F4	1.203	.050	23.925	***	a11_1
BPNSF2	<---	F4	1.115	.049	22.903	***	a12_1
BPNSF21	<---	F5	1.000				
BPNSF14	<---	F5	1.090	.042	25.682	***	a13_1
BPNSF9	<---	F5	1.038	.043	24.375	***	a14_1
BPNSF3	<---	F5	.889	.042	20.973	***	a15_1
BPNSF23	<---	F6	1.000				
BPNSF17	<---	F6	.977	.035	27.907	***	a16_1
BPNSF11	<---	F6	.972	.034	28.226	***	a17_1
BPNSF6	<---	F6	.870	.036	24.502	***	a18_1

Standardized Regression Weights: (g1 - Measurement weights)

			Estimate
BPNSF19	<---	F1	.646
BPNSF13	<---	F1	.762
BPNSF7	<---	F1	.521
BPNSF1	<---	F1	.462
BPNSF18	<---	F2	.237
BPNSF15	<---	F2	.579
BPNSF10	<---	F2	.700
BPNSF5	<---	F2	.593
BPNSF24	<---	F3	.689
BPNSF16	<---	F3	.743
BPNSF12	<---	F3	.795

	Estimate
BPNSF4 <--- F3	.590
BPNSF22 <--- F4	.679
BPNSF20 <--- F4	.765
BPNSF8 <--- F4	.733
BPNSF2 <--- F4	.700
BPNSF21 <--- F5	.687
BPNSF14 <--- F5	.764
BPNSF9 <--- F5	.710
BPNSF3 <--- F5	.631
BPNSF23 <--- F6	.796
BPNSF17 <--- F6	.723
BPNSF11 <--- F6	.747
BPNSF6 <--- F6	.681

Intercepts: (g1 - Measurement weights)

	Estimate	S.E.	C.R.	P	Label
**BPNSF19**	5.314	.068	78.264	***	i1_1
**BPNSF13**	5.196	.066	78.795	***	i2_1
**BPNSF7**	4.945	.073	67.509	***	i3_1
**BPNSF1**	4.595	.073	62.603	***	i4_1
**BPNSF18**	4.383	.071	61.714	***	i5_1
**BPNSF15**	3.793	.073	51.736	***	i6_1
**BPNSF10**	3.294	.078	42.312	***	i7_1
**BPNSF5**	3.688	.073	50.353	***	i8_1
**BPNSF24**	5.244	.059	89.461	***	i9_1
**BPNSF16**	5.112	.061	83.563	***	i10_1
**BPNSF12**	5.230	.062	84.305	***	i11_1
**BPNSF4**	5.068	.059	85.906	***	i12_1
**BPNSF22**	3.015	.070	43.118	***	i13_1
**BPNSF20**	2.351	.074	31.882	***	i14_1
**BPNSF8**	2.768	.078	35.485	***	i15_1
**BPNSF2**	2.230	.076	29.467	***	i16_1
**BPNSF21**	5.359	.068	78.937	***	i17_1
**BPNSF14**	5.585	.067	83.876	***	i18_1
**BPNSF9**	5.848	.068	85.675	***	i19_1
**BPNSF3**	5.716	.066	86.931	***	i20_1
**BPNSF23**	2.068	.074	27.951	***	i21_1
**BPNSF17**	2.440	.080	30.674	***	i22_1
**BPNSF11**	2.690	.077	35.091	***	i23_1
**BPNSF6**	2.458	.075	32.669	***	i24_1

Covariances: (g1 - Measurement weights)

	Estimate	S.E.	C.R.	P	Label
F1 <--> F2	-.077	.024	-3.188	.001	ccc1_1
F2 <--> F3	-.099	.023	-4.275	***	ccc2_1
F1 <--> F3	.582	.061	9.583	***	ccc3_1
F2 <--> F4	.206	.035	5.904	***	ccc4_1
F3 <--> F4	-.462	.057	-8.079	***	ccc5_1
F1 <--> F4	-.398	.062	-6.430	***	ccc6_1
F2 <--> F5	-.061	.024	-2.575	.010	ccc7_1
F4 <--> F5	-.611	.069	-8.891	***	ccc8_1
F3 <--> F5	.612	.062	9.918	***	ccc9_1
F1 <--> F5	.857	.078	11.011	***	ccc10_1
F6 <--> F5	-.853	.086	-9.914	***	ccc11_1
F6 <--> F3	-.499	.067	-7.428	***	ccc12_1
F6 <--> F4	1.133	.097	11.633	***	ccc13_1
F6 <--> F2	.277	.045	6.174	***	ccc14_1
F6 <--> F1	-.563	.077	-7.306	***	ccc15_1

Correlations: (g1 - Measurement weights)

	Estimate
F1 <--> F2	-.238
F2 <--> F3	-.330
F1 <--> F3	.750
F2 <--> F4	.588
F3 <--> F4	-.549
F1 <--> F4	-.436

			Estimate
F2	<-->	F5	-.178
F4	<-->	F5	-.630
F3	<-->	F5	.741
F1	<-->	F5	.956
F6	<-->	F5	-.709
F6	<-->	F3	-.479
F6	<-->	F4	.925
F6	<-->	F2	.637
F6	<-->	F1	-.498

Variances: (g1 - Measurement weights)

	Estimate	S.E.	C.R.	P	Label
**F1**	.841	.095	8.849	***	vvv1_1
**F2**	.124	.032	3.938	***	vvv2_1
**F3**	.715	.072	9.931	***	vvv3_1
**F4**	.988	.100	9.891	***	vvv4_1
**F5**	.954	.096	9.975	***	vvv5_1
**F6**	1.519	.137	11.116	***	vvv6_1
**e1**	1.178	.090	13.085	***	v1_1
**e2**	.799	.073	10.991	***	v2_1
**e3**	1.711	.123	13.968	***	v3_1
**e4**	1.855	.131	14.206	***	v4_1
**e5**	2.085	.143	14.551	***	v5_1
**e6**	1.564	.124	12.580	***	v6_1
**e7**	1.355	.130	10.401	***	v7_1
**e8**	1.524	.123	12.428	***	v8_1
**e9**	.789	.063	12.592	***	v9_1
**e10**	.734	.062	11.779	***	v10_1
**e11**	.619	.059	10.542	***	v11_1
**e12**	.995	.074	13.521	***	v12_1
**e13**	1.154	.088	13.170	***	v13_1
**e14**	.988	.082	12.023	***	v14_1
**e15**	1.234	.098	12.556	***	v15_1
**e16**	1.280	.099	12.963	***	v16_1
**e17**	1.065	.079	13.403	***	v17_1
**e18**	.808	.065	12.438	***	v18_1
**e19**	1.013	.077	13.190	***	v19_1
**e20**	1.140	.083	13.803	***	v20_1
**e21**	.879	.074	11.907	***	v21_1
**e22**	1.321	.101	13.039	***	v22_1
**e23**	1.138	.089	12.751	***	v23_1
**e24**	1.329	.099	13.423	***	v24_1

Matrices (g1 - Measurement weights)

Residual Covariances (g1 - Measurement weights)

	BPNSF6	BPNSF11	BPNSF17	BPNSF23	BPNSF3	BPNSF9	BPNSF14	BPNSF21	BPNSF2	BPNSF8	BPNSF20	BPNSF22	BPNSF4	BPNSF12	BPNSF16	BPNS[illegible]
**BPNSF6**	.174															
**BPNSF11**	.180	.109														
**BPNSF17**	.116	-.044	-.214													
**BPNSF23**	.052	-.017	-.158	-.057												
**BPNSF3**	-.199	-.039	-.006	-.223	-.005											
**BPNSF9**	-.137	.013	.009	-.114	.057	-.111										
**BPNSF14**	.006	.186	.052	.009	.005	.013	.077									
**BPNSF21**	.055	.100	.248	-.034	-.106	-.080	.053	.020								
**BPNSF2**	.164	.150	-.149	.176	-.334	-.175	-.157	-.138	.142							
**BPNSF8**	.259	.171	-.062	-.175	.063	-.062	.170	.313	.083	.042						
**BPNSF20**	.014	-.122	-.033	.102	-.169	-.055	-.046	-.010	.020	-.107	-.084					
**BPNSF22**	-.077	-.071	-.311	-.056	-.046	.094	.248	.186	.026	.120	-.070	-.069				
**BPNSF4**	-.129	.049	.059	.077	.159	-.102	.119	-.103	-.115	.100	.004	.037	-.062			
**BPNSF12**	-.081	.199	.037	.104	-.061	-.066	.078	-.081	.006	.141	.062	-.025	-.077	.013		
**BPNSF16**	-.203	-.009	-.064	-.098	-.053	.004	.065	.012	-.025	.040	-.001	.031	-.051	.019	.000	
**BPNSF24**	-.076	.042	-.040	-.057	.068	.115	-.075	-.069	-.204	.011	-.083	-.129	-.036	.036	.031	.0[illegible]
**BPNSF5**	.164	.239	-.050	-.063	-.044	-.131	-.042	-.124	.195	.136	-.018	.080	.176	-.108	-.181	-.1[illegible]
**BPNSF10**	.107	.524	-.079	-.134	-.034	-.174	.041	-.076	.166	.292	-.143	-.113	.024	-.057	-.023	.0[illegible]
**BPNSF15**	-.007	.100	-.125	-.331	-.073	.001	.335	-.042	-.204	.040	-.155	-.070	.085	-.059	.173	.0[illegible]
**BPNSF18**	-.398	-.207	-.161	-.342	.241	.318	.123	.450	-.179	-.102	-.336	-.075	.111	.228	.303	.1[illegible]
**BPNSF1**	.099	.155	.339	.190	.019	-.295	-.364	-.076	.179	.236	.147	.126	.224	.002	-.057	.0[illegible]
**BPNSF7**	.158	.046	.349	.023	-.067	.025	-.162	.133	-.168	.327	.183	.161	-.127	-.233	-.234	-.1[illegible]

	BPNSF6	BPNSF11	BPNSF17	BPNSF23	BPNSF3	BPNSF9	BPNSF14	BPNSF21	BPNSF2	BPNSF8	BPNSF20	BPNSF22	BPNSF4	BPNSF12	BPNSF16	BPNS[illegible]
**BPNSF13**	-.079	.053	.049	-.024	-.147	-.169	.225	.069	-.140	.166	-.106	.078	.010	.125	.021	[illegible]
**BPNSF19**	-.076	-.132	-.134	-.285	-.021	-.118	.090	.395	-.367	.053	-.267	-.030	-.050	.025	.102	[illegible]

Residual Means (g1 - Measurement weights)

	BPNSF6	BPNSF11	BPNSF17	BPNSF23	BPNSF3	BPNSF9	BPNSF14	BPNSF21	BPNSF2	BPNSF8	BPNSF20	BPNSF22	BPNSF4	BPNSF12	BPNSF16	BPNSF24	BP[illegible]
	.000	.000	.000	.000	.000	.000	.000	.000	.000	.000	.000	.000	.000	.000	.000	.000	[illegible]

Standardized Residual Covariances (g1 - Measurement weights)

	BPNSF6	BPNSF11	BPNSF17	BPNSF23	BPNSF3	BPNSF9	BPNSF14	BPNSF21	BPNSF2	BPNSF8	BPNSF20	BPNSF22	BPNSF4	BPNSF12	BPNSF16	BPNS[illegible]
**BPNSF6**	1.042															
**BPNSF11**	1.327	.626														
**BPNSF17**	.833	-.306	-1.146													
**BPNSF23**	.393	-.120	-1.114	-.350												
**BPNSF3**	-1.840	-.347	-.051	-2.062	-.036											
**BPNSF9**	-1.209	.107	.071	-.998	.555	-.808										
**BPNSF14**	.053	1.613	.433	.076	.054	.117	.589									
**BPNSF21**	.492	.865	2.074	-.297	-1.041	-.744	.495	.144								
**BPNSF2**	1.259	1.111	-1.071	1.335	-3.087	-1.545	-1.409	-1.225	.839							
**BPNSF8**	1.916	1.222	-.428	-1.273	.563	-.533	1.478	2.696	.600	.236						
**BPNSF20**	.110	-.910	-.240	.782	-1.590	-.495	-.417	-.089	.149	-.776	-.525					
**BPNSF22**	-.639	-.570	-2.430	-.463	-.465	.903	2.421	1.797	.209	.938	-.579	-.479				
**BPNSF4**	-1.363	.511	.588	.817	1.882	-1.153	1.371	-1.175	-1.199	1.008	.039	.417	-.604			
**BPNSF12**	-.799	1.925	.349	1.037	-.674	-.691	.817	-.849	.058	1.331	.617	-.262	-.913	.115		
**BPNSF16**	-2.044	-.092	-.609	-.991	-.599	.040	.705	.124	-.252	.385	-.012	.329	-.618	.209	.001	
**BPNSF24**	-.801	.429	-.397	-.610	.803	1.287	-.851	-.780	-2.127	.107	-.880	-1.460	-.456	.419	.373	[illegible]
**BPNSF5**	1.381	1.959	-.399	-.535	-.438	-1.244	-.407	-1.192	1.629	1.099	-.155	.722	1.936	-1.120	-1.906	[illegible]
**BPNSF10**	.838	3.980	-.579	-1.044	-.317	-1.559	.380	-.684	1.297	2.196	-1.136	-.958	.248	-.552	-.227	[illegible]
**BPNSF15**	-.055	.821	-.988	-2.794	-.721	.007	3.270	-.404	-1.707	.320	-1.327	-.634	.928	-.613	1.823	[illegible]
**BPNSF18**	-3.538	-1.809	-1.355	-3.083	2.462	3.131	1.241	4.459	-1.583	-.876	-3.046	-.722	1.265	2.469	3.324	[illegible]
**BPNSF1**	.849	1.295	2.740	1.646	.181	-2.682	-3.369	-.700	1.528	1.945	1.283	1.158	2.418	.018	-.583	[illegible]
**BPNSF7**	1.350	.381	2.815	.198	-.637	.222	-1.486	1.210	-1.429	2.699	1.591	1.488	-1.371	-2.336	-2.393	[illegible]
**BPNSF13**	-.741	.478	.434	-.226	-1.473	-1.592	2.139	.656	-1.307	1.499	-1.007	.787	.113	1.331	.230	[illegible]
**BPNSF19**	-.695	-1.175	-1.158	-2.629	-.211	-1.116	.856	3.772	-3.352	.470	-2.495	-.300	-.574	.264	1.109	[illegible]

Standardized Residual Means (g1 - Measurement weights)

	BPNSF6	BPNSF11	BPNSF17	BPNSF23	BPNSF3	BPNSF9	BPNSF14	BPNSF21	BPNSF2	BPNSF8	BPNSF20	BPNSF22	BPNSF4	BPNSF12	BPNSF16	BPNSF24	BP[illegible]
	.000	.000	.000	.000	.000	.000	.000	.000	.000	.000	.000	.000	.000	.000	.000	.000	[illegible]

Notes for Group/Model (g1 - Measurement weights)

The following covariance matrix is not positive definite (g1 - Measurement weights)

	F5	F4	F3	F2	F1	F6
**F5**	.954					
**F4**	-.611	.988				
**F3**	.612	-.462	.715			
**F2**	-.061	.206	-.099	.124		
**F1**	.857	-.398	.582	-.077	.841	
**F6**	-.853	1.133	-.499	.277	-.563	1.519

This solution is not admissible.

Modification Indices (g1 - Measurement weights)

Covariances: (g1 - Measurement weights)

			M.I.	Par Change
e24	<-->	F3	4.958	-.089
e23	<-->	F3	5.294	.087
e23	<-->	F2	25.876	.098
e21	<-->	F2	12.051	-.060
e19	<-->	F1	12.451	-.120
e19	<-->	e20	5.084	.125
e17	<-->	F3	5.901	-.087
e17	<-->	F1	21.028	.160
e17	<-->	e22	5.528	.147
e17	<-->	e20	5.217	-.129

			M.I.	Par Change
e16	<-->	F5	5.027	-.078
e16	<-->	e22	8.514	-.203
e15	<-->	F2	6.894	.053
e15	<-->	F1	5.478	.093
e15	<-->	e24	6.417	.174
e15	<-->	e23	6.221	.162
e15	<-->	e21	17.631	-.246
e15	<-->	e19	7.945	-.170
e15	<-->	e17	4.829	.135
e14	<-->	F2	5.559	-.044
e14	<-->	e23	8.630	-.173
e14	<-->	e21	14.580	.203
e13	<-->	F5	7.560	.091
e13	<-->	F3	4.188	-.077
e13	<-->	e22	6.078	-.162
e13	<-->	e18	8.261	.150
e13	<-->	e15	8.028	.182
e12	<-->	e20	10.824	.180
e12	<-->	e19	4.265	-.108
e12	<-->	e18	4.945	.106
e11	<-->	F2	5.838	-.038
e11	<-->	F6	4.539	.072
e11	<-->	e23	6.830	.130
e11	<-->	e18	4.967	.094
e11	<-->	e13	4.379	-.104
e10	<-->	F4	5.380	.072
e10	<-->	F6	10.889	-.116
e10	<-->	e16	4.186	.112
e9	<-->	F4	5.175	-.071
e9	<-->	e19	11.553	.164
e9	<-->	e18	11.407	-.148
e8	<-->	e12	12.395	.233
e8	<-->	e10	4.066	-.122
e7	<-->	e23	28.190	.387
e7	<-->	e15	6.318	.192
e7	<-->	e14	5.171	-.158
e7	<-->	e13	7.186	-.194
e7	<-->	e9	5.936	.149
e7	<-->	e8	5.701	-.198
e6	<-->	F2	5.510	.050
e6	<-->	e21	7.543	-.181
e6	<-->	e18	16.530	.250
e6	<-->	e16	5.772	-.185
e6	<-->	e11	5.337	-.135
e6	<-->	e10	10.944	.201
e5	<-->	F1	5.888	.117
e5	<-->	e24	7.297	-.226
e5	<-->	e18	12.488	-.236
e5	<-->	e17	6.937	.197
e4	<-->	F3	4.408	.097
e4	<-->	F2	26.086	-.121
e4	<-->	e22	4.181	.165
e4	<-->	e20	10.240	.233
e4	<-->	e18	13.396	-.233
e4	<-->	e16	5.437	.186
e4	<-->	e12	14.343	.261
e4	<-->	e7	4.419	-.187
e4	<-->	e6	14.361	-.337
e3	<-->	F3	15.812	-.177
e3	<-->	F1	8.454	.124
e3	<-->	e22	8.379	.226
e3	<-->	e18	10.188	-.196
e3	<-->	e17	4.214	.141
e3	<-->	e16	7.419	-.210
e3	<-->	e11	5.170	-.133
e3	<-->	e10	4.507	-.129
e3	<-->	e8	4.302	-.177
e3	<-->	e4	4.764	.194
e2	<-->	F2	7.517	.046
e2	<-->	e20	9.373	-.158
e2	<-->	e19	10.315	-.158
e2	<-->	e18	20.678	.203

			M.I.	Par Change
e2	<-->	e11	10.523	.140
e1	<-->	e19	6.374	-.144
e1	<-->	e17	33.587	.338
e1	<-->	e5	6.851	.208

Variances: (g1 - Measurement weights)

	M.I.	Par Change

Regression Weights: (g1 - Measurement weights)

			M.I.	Par Change
BPNSF6	<---	F3	4.070	-.149
BPNSF11	<---	F2	18.226	.763
BPNSF11	<---	BPNSF10	6.814	.039
BPNSF23	<---	F2	12.138	-.562
BPNSF14	<---	F2	4.631	.324
BPNSF2	<---	F5	8.993	-.185
BPNSF2	<---	F1	10.869	-.222
BPNSF8	<---	F5	6.998	.162
BPNSF8	<---	F2	5.341	.434
BPNSF8	<---	F1	7.740	.186
BPNSF20	<---	F2	5.119	-.387
BPNSF10	<---	BPNSF11	4.558	.044
BPNSF18	<---	F5	18.585	.322
BPNSF18	<---	F4	7.684	-.206
BPNSF18	<---	F3	12.539	.316
BPNSF18	<---	F1	17.725	.343
BPNSF18	<---	F6	9.549	-.184
BPNSF18	<---	BPNSF6	4.403	-.050
BPNSF1	<---	F5	9.300	-.216
BPNSF1	<---	F2	6.751	-.566
BPNSF1	<---	F6	4.444	.119
BPNSF7	<---	F4	4.455	.145
BPNSF7	<---	F3	6.416	-.208
BPNSF19	<---	F4	7.559	-.161
BPNSF19	<---	F6	7.954	-.131

Means: (g1 - Measurement weights)

	M.I.	Par Change

Intercepts: (g1 - Measurement weights)

	M.I.	Par Change

Bootstrap (g1 - Measurement weights)

Bootstrap standard errors (g1 - Measurement weights)

Scalar Estimates (g1 - Measurement weights)

Regression Weights: (g1 - Measurement weights)

Parameter			SE	SE-SE	Mean	Bias	SE-Bias
BPNSF19	<---	F1	.000	.000	1.000	.000	.000
BPNSF13	<---	F1	.058	.003	1.141	-.005	.004
BPNSF7	<---	F1	.060	.003	.871	.000	.004
BPNSF1	<---	F1	.073	.004	.774	.000	.005
BPNSF18	<---	F2	.000	.000	1.000	.000	.000
BPNSF15	<---	F2	.413	.021	2.575	.053	.029
BPNSF10	<---	F2	.549	.027	3.306	.072	.039
BPNSF5	<---	F2	.437	.022	2.636	.057	.031
BPNSF24	<---	F3	.000	.000	1.000	.000	.000
BPNSF16	<---	F3	.054	.003	1.126	.001	.004
BPNSF12	<---	F3	.049	.002	1.216	-.005	.003
BPNSF4	<---	F3	.052	.003	.860	-.001	.004
BPNSF22	<---	F4	.000	.000	1.000	.000	.000
BPNSF20	<---	F4	.051	.003	1.186	-.001	.004

Parameter	SE	SE-SE	Mean	Bias	SE-Bias
BPNSF8 <--- F4	.050	.003	1.198	-.005	.004
BPNSF2 <--- F4	.055	.003	1.114	-.002	.004
BPNSF21 <--- F5	.000	.000	1.000	.000	.000
BPNSF14 <--- F5	.047	.002	1.092	.002	.003
BPNSF9 <--- F5	.054	.003	1.040	.002	.004
BPNSF3 <--- F5	.061	.003	.894	.005	.004
BPNSF23 <--- F6	.000	.000	1.000	.000	.000
BPNSF17 <--- F6	.034	.002	.980	.004	.002
BPNSF11 <--- F6	.038	.002	.973	.001	.003
BPNSF6 <--- F6	.037	.002	.870	.000	.003

Standardized Regression Weights: (g1 - Measurement weights)

Parameter	SE	SE-SE	Mean	Bias	SE-Bias
BPNSF19 <--- F1	.039	.002	.650	.004	.003
BPNSF13 <--- F1	.032	.002	.760	-.002	.002
BPNSF7 <--- F1	.038	.002	.521	-.001	.003
BPNSF1 <--- F1	.033	.002	.462	.000	.002
BPNSF18 <--- F2	.036	.002	.238	.001	.003
BPNSF15 <--- F2	.038	.002	.581	.001	.003
BPNSF10 <--- F2	.036	.002	.699	-.001	.003
BPNSF5 <--- F2	.030	.001	.593	.000	.002
BPNSF24 <--- F3	.029	.001	.691	.002	.002
BPNSF16 <--- F3	.033	.002	.747	.004	.002
BPNSF12 <--- F3	.025	.001	.794	-.001	.002
BPNSF4 <--- F3	.028	.001	.588	-.002	.002
BPNSF22 <--- F4	.027	.001	.678	-.001	.002
BPNSF20 <--- F4	.026	.001	.763	-.002	.002
BPNSF8 <--- F4	.031	.002	.729	-.004	.002
BPNSF2 <--- F4	.032	.002	.695	-.005	.002
BPNSF21 <--- F5	.030	.002	.686	-.002	.002
BPNSF14 <--- F5	.041	.002	.766	.002	.003
BPNSF9 <--- F5	.034	.002	.712	.002	.002
BPNSF3 <--- F5	.039	.002	.633	.003	.003
BPNSF23 <--- F6	.027	.001	.795	-.001	.002
BPNSF17 <--- F6	.030	.001	.724	.001	.002
BPNSF11 <--- F6	.029	.001	.744	-.003	.002
BPNSF6 <--- F6	.033	.002	.678	-.004	.002

Intercepts: (g1 - Measurement weights)

Parameter	SE	SE-SE	Mean	Bias	SE-Bias
**BPNSF19**	.068	.003	5.325	.011	.005
**BPNSF13**	.068	.003	5.202	.006	.005
**BPNSF7**	.076	.004	4.949	.004	.005
**BPNSF1**	.071	.004	4.595	.001	.005
**BPNSF18**	.065	.003	4.379	-.004	.005
**BPNSF15**	.079	.004	3.790	-.003	.006
**BPNSF10**	.075	.004	3.297	.003	.005
**BPNSF5**	.083	.004	3.682	-.006	.006
**BPNSF24**	.062	.003	5.250	.006	.004
**BPNSF16**	.063	.003	5.120	.008	.004
**BPNSF12**	.067	.003	5.238	.008	.005
**BPNSF4**	.059	.003	5.075	.006	.004
**BPNSF22**	.069	.003	3.015	.000	.005
**BPNSF20**	.076	.004	2.342	-.009	.005
**BPNSF8**	.083	.004	2.764	-.004	.006
**BPNSF2**	.076	.004	2.229	-.001	.005
**BPNSF21**	.074	.004	5.364	.006	.005
**BPNSF14**	.069	.003	5.597	.011	.005
**BPNSF9**	.069	.003	5.853	.006	.005
**BPNSF3**	.066	.003	5.723	.007	.005
**BPNSF23**	.072	.004	2.055	-.013	.005
**BPNSF17**	.079	.004	2.435	-.005	.006
**BPNSF11**	.073	.004	2.688	-.003	.005
**BPNSF6**	.082	.004	2.453	-.005	.006

Covariances: (g1 - Measurement weights)

Parameter	SE	SE-SE	Mean	Bias	SE-Bias

Parameter			SE	SE-SE	Mean	Bias	SE-Bias
F1	<-->	F2	.025	.001	-.076	.001	.002
F2	<-->	F3	.021	.001	-.096	.003	.001
F1	<-->	F3	.069	.003	.580	-.001	.005
F2	<-->	F4	.037	.002	.205	-.001	.003
F3	<-->	F4	.051	.003	-.458	.003	.004
F1	<-->	F4	.061	.003	-.390	.007	.004
F2	<-->	F5	.022	.001	-.061	.001	.002
F4	<-->	F5	.061	.003	-.601	.010	.004
F3	<-->	F5	.068	.003	.611	-.001	.005
F1	<-->	F5	.104	.005	.853	-.004	.007
F6	<-->	F5	.083	.004	-.840	.013	.006
F6	<-->	F3	.059	.003	-.502	-.002	.004
F6	<-->	F4	.105	.005	1.125	-.008	.007
F6	<-->	F2	.047	.002	.275	-.001	.003
F6	<-->	F1	.075	.004	-.557	.006	.005

Correlations: (g1 - Measurement weights)

Parameter			SE	SE-SE	Mean	Bias	SE-Bias
F1	<-->	F2	.072	.004	-.237	.001	.005
F2	<-->	F3	.066	.003	-.324	.006	.005
F1	<-->	F3	.054	.003	.746	-.003	.004
F2	<-->	F4	.060	.003	.587	-.001	.004
F3	<-->	F4	.050	.002	-.547	.002	.004
F1	<-->	F4	.056	.003	-.430	.007	.004
F2	<-->	F5	.062	.003	-.179	-.002	.004
F4	<-->	F5	.047	.002	-.626	.004	.003
F3	<-->	F5	.040	.002	.742	.001	.003
F1	<-->	F5	.036	.002	.955	-.001	.003
F6	<-->	F5	.043	.002	-.706	.003	.003
F6	<-->	F3	.049	.002	-.484	-.005	.003
F6	<-->	F4	.032	.002	.926	.001	.002
F6	<-->	F2	.050	.003	.638	.001	.004
F6	<-->	F1	.048	.002	-.495	.003	.003

Variances: (g1 - Measurement weights)

Parameter	SE	SE-SE	Mean	Bias	SE-Bias
**F1**	.114	.006	.846	.004	.008
**F2**	.038	.002	.127	.003	.003
**F3**	.066	.003	.718	.002	.005
**F4**	.098	.005	.983	-.004	.007
**F5**	.112	.006	.947	-.007	.008
**F6**	.143	.007	1.504	-.015	.010
**e1**	.137	.007	1.151	-.026	.010
**e2**	.104	.005	.799	.000	.007
**e3**	.172	.009	1.715	.004	.012
**e4**	.137	.007	1.840	-.015	.010
**e5**	.138	.007	2.065	-.020	.010
**e6**	.148	.007	1.552	-.011	.010
**e7**	.141	.007	1.354	.000	.010
**e8**	.131	.007	1.518	-.005	.009
**e9**	.089	.004	.784	-.006	.006
**e10**	.103	.005	.719	-.015	.007
**e11**	.082	.004	.619	-.001	.006
**e12**	.087	.004	1.000	.006	.006
**e13**	.103	.005	1.152	-.002	.007
**e14**	.125	.006	.992	.004	.009
**e15**	.150	.007	1.239	.005	.011
**e16**	.146	.007	1.298	.018	.010
**e17**	.114	.006	1.061	-.004	.008
**e18**	.155	.008	.795	-.013	.011
**e19**	.158	.008	.995	-.018	.011
**e20**	.165	.008	1.126	-.014	.012
**e21**	.126	.006	.874	-.005	.009
**e22**	.175	.009	1.307	-.013	.012
**e23**	.115	.006	1.142	.004	.008
**e24**	.152	.008	1.339	.010	.011

Matrices (g1 - Measurement weights)

Sample Covariances - Standard Errors (g1 - Measurement weights)

	BPNSF6	BPNSF11	BPNSF17	BPNSF23	BPNSF3	BPNSF9	BPNSF14	BPNSF21	BPNSF2	BPNSF8	BPNSF20	BPNSF22	BPNSF4	BPNSF12	BPNSF16	BPNS[illegible]
BPNSF6	.189															
BPNSF11	.149	.158														
BPNSF17	.153	.153	.188													
BPNSF23	.149	.150	.138	.180												
BPNSF3	.128	.115	.109	.108	.175											
BPNSF9	.116	.104	.108	.109	.116	.182										
BPNSF14	.124	.116	.124	.117	.105	.122	.166									
BPNSF21	.101	.104	.121	.111	.106	.115	.137	.163								
BPNSF2	.154	.150	.138	.147	.101	.110	.120	.116	.159							
BPNSF8	.153	.148	.155	.151	.114	.115	.111	.110	.151	.169						
BPNSF20	.153	.147	.159	.149	.100	.106	.131	.116	.132	.154	.166					
BPNSF22	.121	.128	.124	.125	.095	.091	.102	.105	.112	.126	.114	.119				
BPNSF4	.104	.104	.097	.085	.104	.088	.074	.096	.113	.107	.091	.090	.102			
BPNSF12	.117	.103	.107	.091	.094	.110	.131	.120	.107	.115	.100	.092	.085	.135		
BPNSF16	.122	.104	.107	.108	.098	.107	.123	.114	.109	.109	.102	.082	.080	.102	.123	
BPNSF24	.101	.093	.103	.097	.106	.110	.102	.105	.097	.098	.090	.092	.082	.095	.094	[illegible]
BPNSF5	.124	.125	.135	.113	.092	.091	.103	.109	.112	.136	.118	.109	.088	.098	.099	[illegible]
BPNSF10	.139	.143	.144	.134	.123	.109	.126	.121	.139	.153	.136	.126	.114	.116	.112	[illegible]
BPNSF15	.123	.129	.132	.115	.124	.112	.111	.125	.134	.148	.114	.123	.098	.112	.110	[illegible]
BPNSF18	.120	.122	.124	.110	.104	.112	.117	.113	.113	.134	.102	.112	.088	.117	.111	[illegible]
BPNSF1	.124	.105	.111	.094	.112	.097	.103	.110	.110	.115	.097	.096	.091	.094	.086	[illegible]
BPNSF7	.121	.112	.114	.093	.112	.112	.109	.118	.111	.114	.097	.101	.091	.109	.100	[illegible]
BPNSF13	.117	.118	.112	.112	.098	.116	.130	.133	.116	.117	.108	.095	.088	.132	.113	[illegible]
BPNSF19	.107	.112	.117	.115	.112	.113	.136	.142	.121	.125	.124	.106	.099	.108	.117	[illegible]

Sample Correlations - Standard Errors (g1 - Measurement weights)

	BPNSF6	BPNSF11	BPNSF17	BPNSF23	BPNSF3	BPNSF9	BPNSF14	BPNSF21	BPNSF2	BPNSF8	BPNSF20	BPNSF22	BPNSF4	BPNSF12	BPNSF16	BPNS[illegible]
BPNSF6	.000															
BPNSF11	.044	.000														
BPNSF17	.044	.048	.000													
BPNSF23	.043	.040	.047	.000												
BPNSF3	.052	.051	.049	.043	.000											
BPNSF9	.051	.045	.046	.047	.053	.000										
BPNSF14	.052	.049	.049	.048	.053	.046	.000									
BPNSF21	.043	.042	.053	.044	.052	.051	.051	.000								
BPNSF2	.051	.044	.048	.045	.045	.047	.048	.044	.000							
BPNSF8	.045	.042	.049	.051	.052	.048	.048	.049	.044	.000						
BPNSF20	.050	.047	.049	.043	.044	.045	.052	.051	.047	.048	.000					
BPNSF22	.044	.046	.048	.044	.045	.043	.051	.052	.040	.040	.044	.000				
BPNSF4	.049	.052	.049	.044	.056	.049	.035	.050	.053	.051	.044	.050	.000			
BPNSF12	.050	.049	.049	.043	.048	.050	.055	.051	.048	.053	.047	.047	.046	.000		
BPNSF16	.053	.047	.048	.048	.053	.049	.056	.049	.050	.050	.048	.045	.044	.043	.000	
BPNSF24	.048	.047	.051	.050	.052	.050	.056	.054	.047	.051	.049	.051	.048	.039	.052	[illegible]
BPNSF5	.044	.045	.053	.047	.045	.043	.048	.049	.042	.052	.049	.048	.048	.048	.047	[illegible]
BPNSF10	.050	.041	.052	.048	.056	.049	.054	.051	.047	.050	.051	.050	.058	.053	.052	[illegible]
BPNSF15	.047	.047	.051	.047	.057	.052	.050	.055	.052	.055	.046	.053	.052	.054	.055	[illegible]
BPNSF18	.051	.051	.053	.050	.052	.057	.057	.054	.048	.055	.047	.054	.050	.061	.059	[illegible]
BPNSF1	.051	.044	.047	.042	.045	.047	.050	.047	.045	.047	.044	.045	.045	.046	.045	[illegible]
BPNSF7	.050	.045	.047	.041	.053	.047	.052	.048	.045	.046	.043	.046	.050	.052	.051	[illegible]
BPNSF13	.050	.050	.049	.048	.048	.051	.037	.044	.048	.051	.047	.048	.046	.052	.050	[illegible]
BPNSF19	.045	.046	.049	.043	.053	.052	.053	.045	.048	.054	.050	.051	.054	.048	.054	[illegible]

Sample Means - Standard Errors (g1 - Measurement weights)

	BPNSF6	BPNSF11	BPNSF17	BPNSF23	BPNSF3	BPNSF9	BPNSF14	BPNSF21	BPNSF2	BPNSF8	BPNSF20	BPNSF22	BPNSF4	BPNSF12	BPNSF16	BPNSF[illegible]
BPNSF6	.082	.073	.079	.072	.066	.069	.069	.074	.076	.083	.076	.069	.059	.067	.063	[illegible]

Bootstrap Confidence (g1 - Measurement weights)

Percentile method (g1 - Measurement weights)

90% confidence intervals (percentile method)

Scalar Estimates (g1 - Measurement weights)

Regression Weights: (g1 - Measurement weights)

Parameter	Estimate	Lower	Upper	P
BPNSF19 <--- F1	1.000	1.000	1.000	...
BPNSF13 <--- F1	1.146	1.045	1.234	.010
BPNSF7 <--- F1	.872	.767	.971	.010
BPNSF1 <--- F1	.774	.651	.908	.010
BPNSF18 <--- F2	1.000	1.000	1.000	...
BPNSF15 <--- F2	2.522	2.037	3.351	.010
BPNSF10 <--- F2	3.235	2.580	4.410	.010
BPNSF5 <--- F2	2.579	2.034	3.484	.010
BPNSF24 <--- F3	1.000	1.000	1.000	...
BPNSF16 <--- F3	1.125	1.040	1.218	.010
BPNSF12 <--- F3	1.221	1.134	1.299	.010
BPNSF4 <--- F3	.861	.771	.947	.010
BPNSF22 <--- F4	1.000	1.000	1.000	...
BPNSF20 <--- F4	1.188	1.107	1.281	.010
BPNSF8 <--- F4	1.203	1.117	1.281	.010
BPNSF2 <--- F4	1.115	1.029	1.215	.010
BPNSF21 <--- F5	1.000	1.000	1.000	...
BPNSF14 <--- F5	1.090	1.014	1.183	.010
BPNSF9 <--- F5	1.038	.959	1.137	.010
BPNSF3 <--- F5	.889	.797	.997	.010
BPNSF23 <--- F6	1.000	1.000	1.000	...
BPNSF17 <--- F6	.977	.925	1.044	.010
BPNSF11 <--- F6	.972	.915	1.036	.010
BPNSF6 <--- F6	.870	.809	.937	.010

Standardized Regression Weights: (g1 - Measurement weights)

Parameter	Estimate	Lower	Upper	P
BPNSF19 <--- F1	.646	.580	.704	.010
BPNSF13 <--- F1	.762	.703	.807	.010
BPNSF7 <--- F1	.521	.460	.580	.010
BPNSF1 <--- F1	.462	.409	.518	.010
BPNSF18 <--- F2	.237	.178	.287	.010
BPNSF15 <--- F2	.579	.515	.642	.010
BPNSF10 <--- F2	.700	.639	.765	.010
BPNSF5 <--- F2	.593	.543	.641	.010
BPNSF24 <--- F3	.689	.642	.737	.010
BPNSF16 <--- F3	.743	.693	.799	.010
BPNSF12 <--- F3	.795	.747	.832	.010
BPNSF4 <--- F3	.590	.540	.637	.010
BPNSF22 <--- F4	.679	.630	.727	.010
BPNSF20 <--- F4	.765	.723	.808	.010
BPNSF8 <--- F4	.733	.679	.779	.010
BPNSF2 <--- F4	.700	.641	.748	.010
BPNSF21 <--- F5	.687	.635	.738	.010
BPNSF14 <--- F5	.764	.701	.829	.010
BPNSF9 <--- F5	.710	.654	.769	.010
BPNSF3 <--- F5	.631	.566	.701	.010
BPNSF23 <--- F6	.796	.747	.841	.010
BPNSF17 <--- F6	.723	.677	.773	.010
BPNSF11 <--- F6	.747	.693	.792	.010
BPNSF6 <--- F6	.681	.620	.736	.010

Intercepts: (g1 - Measurement weights)

Parameter	Estimate	Lower	Upper	P
**BPNSF19**	5.314	5.214	5.451	.010
**BPNSF13**	5.196	5.075	5.310	.010
**BPNSF7**	4.945	4.815	5.064	.010
**BPNSF1**	4.595	4.485	4.713	.010
**BPNSF18**	4.383	4.269	4.483	.010
**BPNSF15**	3.793	3.658	3.936	.010
**BPNSF10**	3.294	3.169	3.408	.010
**BPNSF5**	3.688	3.558	3.829	.010
**BPNSF24**	5.244	5.139	5.367	.010
**BPNSF16**	5.112	5.014	5.223	.010
**BPNSF12**	5.230	5.123	5.346	.010
**BPNSF4**	5.068	4.970	5.164	.010
**BPNSF22**	3.015	2.901	3.142	.010
**BPNSF20**	2.351	2.220	2.470	.010
**BPNSF8**	2.768	2.626	2.909	.010

Parameter	Estimate	Lower	Upper	P
**BPNSF2**	2.230	2.105	2.367	.010
**BPNSF21**	5.359	5.242	5.484	.010
**BPNSF14**	5.585	5.485	5.713	.010
**BPNSF9**	5.848	5.729	5.952	.010
**BPNSF3**	5.716	5.618	5.853	.010
**BPNSF23**	2.068	1.943	2.178	.010
**BPNSF17**	2.440	2.304	2.585	.010
**BPNSF11**	2.690	2.572	2.818	.010
**BPNSF6**	2.458	2.319	2.585	.010

Covariances: (g1 - Measurement weights)

Parameter	Estimate	Lower	Upper	P
F1 <--> F2	-.077	-.124	-.037	.010
F2 <--> F3	-.099	-.132	-.064	.010
F1 <--> F3	.582	.478	.697	.010
F2 <--> F4	.206	.148	.269	.010
F3 <--> F4	-.462	-.539	-.377	.010
F1 <--> F4	-.398	-.498	-.298	.010
F2 <--> F5	-.061	-.101	-.025	.010
F4 <--> F5	-.611	-.708	-.505	.010
F3 <--> F5	.612	.510	.724	.010
F1 <--> F5	.857	.686	1.053	.010
F6 <--> F5	-.853	-.987	-.717	.010
F6 <--> F3	-.499	-.608	-.408	.010
F6 <--> F4	1.133	.955	1.326	.010
F6 <--> F2	.277	.207	.361	.010
F6 <--> F1	-.563	-.684	-.441	.010

Correlations: (g1 - Measurement weights)

Parameter	Estimate	Lower	Upper	P
F1 <--> F2	-.238	-.362	-.117	.010
F2 <--> F3	-.330	-.446	-.224	.010
F1 <--> F3	.750	.658	.837	.010
F2 <--> F4	.588	.477	.681	.010
F3 <--> F4	-.549	-.628	-.471	.010
F1 <--> F4	-.436	-.524	-.336	.010
F2 <--> F5	-.178	-.287	-.073	.010
F4 <--> F5	-.630	-.710	-.551	.010
F3 <--> F5	.741	.675	.806	.010
F1 <--> F5	.956	.894	1.013	.010
F6 <--> F5	-.709	-.776	-.640	.010
F6 <--> F3	-.479	-.560	-.404	.010
F6 <--> F4	.925	.867	.975	.010
F6 <--> F2	.637	.544	.718	.010
F6 <--> F1	-.498	-.580	-.411	.010

Variances: (g1 - Measurement weights)

Parameter	Estimate	Lower	Upper	P
**F1**	.841	.646	1.037	.010
**F2**	.124	.070	.186	.010
**F3**	.715	.618	.832	.010
**F4**	.988	.827	1.152	.010
**F5**	.954	.783	1.137	.010
**F6**	1.519	1.274	1.746	.010
**e1**	1.178	.939	1.380	.010
**e2**	.799	.649	1.000	.010
**e3**	1.711	1.451	1.999	.010
**e4**	1.855	1.621	2.099	.010
**e5**	2.085	1.838	2.322	.010
**e6**	1.564	1.314	1.827	.010
**e7**	1.355	1.113	1.563	.010
**e8**	1.524	1.295	1.743	.010
**e9**	.789	.630	.945	.010
**e10**	.734	.563	.941	.010
**e11**	.619	.468	.762	.010
**e12**	.995	.856	1.144	.010
**e13**	1.154	.985	1.327	.010

Parameter	Estimate	Lower	Upper	P
e14	.988	.782	1.199	.010
e15	1.234	1.020	1.458	.010
e16	1.280	1.051	1.550	.010
e17	1.065	.873	1.259	.010
e18	.808	.553	1.059	.010
e19	1.013	.758	1.267	.010
e20	1.140	.863	1.429	.010
e21	.879	.667	1.089	.010
e22	1.321	1.002	1.605	.010
e23	1.138	.969	1.334	.010
e24	1.329	1.097	1.612	.010

Matrices (g1 - Measurement weights)

Sample Covariances (g1 - Measurement weights)

Sample Covariances - Lower Bounds (PC) (g1 - Measurement weights)

	BPNSF6	BPNSF11	BPNSF17	BPNSF23	BPNSF3	BPNSF9	BPNSF14	BPNSF21	BPNSF2	BPNSF8	BPNSF20	BPNSF22	BPNSF4	BPNSF12	BPNSF16	BPNS[illegible]
BPNSF6	2.334															
BPNSF11	1.185	2.407														
BPNSF17	1.144	1.179	2.238													
BPNSF23	1.083	1.165	1.100	2.025												
BPNSF3	-1.068	-.940	-.930	-1.146	1.572											
BPNSF9	-1.074	-.991	-1.039	-1.162	.755	1.632										
BPNSF14	-1.016	-.936	-1.068	-1.118	.759	.897	1.730									
BPNSF21	-.845	-.876	-.773	-1.051	.557	.717	.863	1.770								
BPNSF2	1.006	1.137	.869	1.170	-1.116	-1.062	-1.113	-1.016	2.389							
BPNSF8	1.181	1.236	1.035	.912	-.772	-.997	-.808	-.575	1.133	2.418						
BPNSF20	.879	.939	1.022	1.193	-.958	-.986	-1.046	-.893	1.116	1.029	2.013					
BPNSF22	.684	.840	.617	.844	-.744	-.691	-.576	-.599	.940	1.077	.918	1.856				
BPNSF4	-.668	-.527	-.508	-.514	.481	.299	.568	.270	-.739	-.537	-.597	-.502	1.312			
BPNSF12	-.817	-.566	-.748	-.671	.431	.520	.695	.471	-.793	-.705	-.792	-.745	.523	1.456		
BPNSF16	-.868	-.726	-.816	-.842	.397	.553	.603	.520	-.786	-.767	-.795	-.640	.505	.831	1.413	
BPNSF24	-.650	-.607	-.738	-.718	.437	.548	.435	.368	-.882	-.701	-.760	-.751	.443	.761	.675	[illegible]
BPNSF5	.572	.710	.437	.446	-.334	-.443	-.372	-.458	.608	.513	.413	.420	-.163	-.571	-.639	[illegible]
BPNSF10	.638	1.131	.564	.547	-.402	-.544	-.398	-.481	.656	.811	.435	.337	-.434	-.647	-.557	[illegible]
BPNSF15	.390	.553	.355	.168	-.417	-.334	-.014	-.402	.144	.421	.272	.249	-.286	-.537	-.294	[illegible]
BPNSF18	-.343	-.115	-.066	-.227	.012	.086	-.142	.193	-.121	-.046	-.242	-.055	-.112	-.097	.019	[illegible]
BPNSF1	-.478	-.428	-.264	-.406	.420	.224	.177	.394	-.321	-.300	-.361	-.312	.446	.373	.294	[illegible]
BPNSF7	-.444	-.592	-.288	-.615	.373	.612	.459	.689	-.734	-.254	-.416	-.343	.141	.184	.161	[illegible]
BPNSF13	-.833	-.762	-.771	-.855	.577	.664	1.070	.814	-.840	-.574	-.814	-.531	.424	.715	.567	[illegible]
BPNSF19	-.745	-.870	-.871	-1.037	.561	.558	.818	.996	-1.004	-.625	-.934	-.586	.285	.571	.562	[illegible]

Sample Covariances - Upper Bounds (PC) (g1 - Measurement weights)

	BPNSF6	BPNSF11	BPNSF17	BPNSF23	BPNSF3	BPNSF9	BPNSF14	BPNSF21	BPNSF2	BPNSF8	BPNSF20	BPNSF22	BPNSF4	BPNSF12	BPNSF16	BPNS[illegible]
BPNSF6	2.942															
BPNSF11	1.685	2.909														
BPNSF17	1.663	1.690	2.870													
BPNSF23	1.585	1.683	1.594	2.617												
BPNSF3	-.631	-.574	-.540	-.793	2.154											
BPNSF9	-.701	-.656	-.678	-.829	1.129	2.239										
BPNSF14	-.594	-.546	-.654	-.733	1.096	1.297	2.261									
BPNSF21	-.485	-.539	-.365	-.687	.913	1.099	1.332	2.292								
BPNSF2	1.496	1.646	1.325	1.664	-.753	-.687	-.719	-.634	2.911							
BPNSF8	1.697	1.728	1.570	1.428	-.382	-.587	-.450	-.213	1.636	2.942						
BPNSF20	1.427	1.436	1.562	1.702	-.617	-.607	-.622	-.514	1.533	1.533	2.583					
BPNSF22	1.101	1.260	1.016	1.266	-.428	-.373	-.234	-.223	1.323	1.503	1.293	2.260				
BPNSF4	-.321	-.195	-.202	-.216	.799	.599	.822	.583	-.367	-.185	-.317	-.213	1.639			
BPNSF12	-.398	-.209	-.397	-.373	.753	.892	1.128	.880	-.443	-.344	-.450	-.433	.802	1.923		
BPNSF16	-.449	-.379	-.447	-.485	.718	.905	1.006	.876	-.422	-.394	-.453	-.355	.775	1.160	1.817	
BPNSF24	-.308	-.282	-.365	-.389	.794	.926	.790	.716	-.555	-.362	-.465	-.435	.710	1.074	.985	[illegible]
BPNSF5	1.003	1.133	.875	.841	-.011	-.135	-.062	-.110	.969	.981	.809	.770	.114	-.263	-.290	[illegible]
BPNSF10	1.131	1.597	1.074	.988	-.016	-.161	.034	-.074	1.145	1.312	.869	.767	-.048	-.255	-.188	[illegible]
BPNSF15	.796	.993	.777	.541	-.012	.036	.353	.014	.593	.912	.627	.638	.038	-.172	.100	[illegible]
BPNSF18	.042	.275	.324	.109	.363	.426	.255	.578	.231	.386	.106	.320	.184	.297	.366	[illegible]
BPNSF1	-.083	-.101	.106	-.076	.792	.538	.544	.771	.025	.089	-.056	-.003	.746	.672	.574	[illegible]
BPNSF7	-.055	-.230	.084	-.312	.750	.989	.834	1.079	-.392	.117	-.060	-.006	.438	.562	.493	[illegible]
BPNSF13	-.433	-.360	-.410	-.488	.907	1.033	1.491	1.272	-.449	-.170	-.437	-.201	.727	1.144	.944	[illegible]

	BPNSF6	BPNSF11	BPNSF17	BPNSF23	BPNSF3	BPNSF9	BPNSF14	BPNSF21	BPNSF2	BPNSF8	BPNSF20	BPNSF22	BPNSF4	BPNSF12	BPNSF16	BPNS[illegible]
**BPNSF19**	-.375	-.486	-.491	-.654	.934	.941	1.239	1.468	-.604	-.185	-.519	-.247	.613	.916	.943	[illegible]

Sample Covariances - Two Tailed Significance (PC) (g1 - Measurement weights)

	BPNSF6	BPNSF11	BPNSF17	BPNSF23	BPNSF3	BPNSF9	BPNSF14	BPNSF21	BPNSF2	BPNSF8	BPNSF20	BPNSF22	BPNSF4	BPNSF12	BPNSF16	BPNS[illegible]
**BPNSF6**	.010															
**BPNSF11**	.010	.010														
**BPNSF17**	.010	.010	.010													
**BPNSF23**	.010	.010	.010	.010												
**BPNSF3**	.010	.010	.010	.010	.010											
**BPNSF9**	.010	.010	.010	.010	.010	.010										
**BPNSF14**	.010	.010	.010	.010	.010	.010	.010									
**BPNSF21**	.010	.010	.010	.010	.010	.010	.010	.010								
**BPNSF2**	.010	.010	.010	.010	.010	.010	.010	.010	.010							
**BPNSF8**	.010	.010	.010	.010	.010	.010	.010	.010	.010	.010						
**BPNSF20**	.010	.010	.010	.010	.010	.010	.010	.010	.010	.010	.010					
**BPNSF22**	.010	.010	.010	.010	.010	.010	.010	.010	.010	.010	.010	.010				
**BPNSF4**	.010	.010	.010	.010	.010	.010	.010	.010	.010	.010	.010	.010	.010			
**BPNSF12**	.010	.010	.010	.010	.010	.010	.010	.010	.010	.010	.010	.010	.010	.010		
**BPNSF16**	.010	.010	.010	.010	.010	.010	.010	.010	.010	.010	.010	.010	.010	.010	.010	
**BPNSF24**	.010	.010	.010	.010	.010	.010	.010	.010	.010	.010	.010	.010	.010	.010	.010	[illegible]
**BPNSF5**	.010	.010	.010	.010	.077	.012	.044	.016	.010	.010	.010	.010	.721	.010	.010	[illegible]
**BPNSF10**	.010	.010	.010	.010	.077	.010	.193	.010	.010	.010	.010	.010	.039	.010	.010	[illegible]
**BPNSF15**	.010	.010	.010	.010	.094	.201	.157	.142	.010	.010	.010	.010	.196	.010	.371	[illegible]
**BPNSF18**	.207	.487	.403	.569	.078	.041	.589	.010	.702	.197	.400	.271	.783	.336	.078	[illegible]
**BPNSF1**	.027	.018	.456	.016	.010	.010	.010	.010	.146	.339	.033	.095	.010	.010	.010	[illegible]
**BPNSF7**	.025	.010	.268	.010	.010	.010	.010	.010	.010	.519	.010	.073	.010	.010	.016	[illegible]
**BPNSF13**	.010	.010	.010	.010	.010	.010	.010	.010	.010	.010	.010	.010	.010	.010	.010	[illegible]
**BPNSF19**	.010	.010	.010	.010	.010	.010	.010	.010	.010	.010	.010	.010	.010	.010	.010	[illegible]

Sample Correlations (g1 - Measurement weights)

Sample Correlations - Lower Bounds (PC) (g1 - Measurement weights)

	BPNSF6	BPNSF11	BPNSF17	BPNSF23	BPNSF3	BPNSF9	BPNSF14	BPNSF21	BPNSF2	BPNSF8	BPNSF20	BPNSF22	BPNSF4	BPNSF12	BPNSF16	BPNS[illegible]
**BPNSF6**	1.000															
**BPNSF11**	.469	1.000														
**BPNSF17**	.460	.467	1.000													
**BPNSF23**	.472	.506	.472	1.000												
**BPNSF3**	-.471	-.424	-.417	-.541	1.000											
**BPNSF9**	-.477	-.436	-.460	-.550	.403	1.000										
**BPNSF14**	-.444	-.394	-.456	-.501	.387	.474	1.000									
**BPNSF21**	-.360	-.375	-.341	-.465	.297	.376	.447	1.000								
**BPNSF2**	.395	.437	.335	.502	-.500	-.465	-.470	-.431	1.000							
**BPNSF8**	.458	.476	.412	.376	-.339	-.424	-.347	-.253	.446	1.000						
**BPNSF20**	.389	.398	.448	.542	-.452	-.456	-.475	-.418	.455	.430	1.000					
**BPNSF22**	.309	.363	.273	.409	-.378	-.340	-.287	-.291	.408	.474	.436	1.000				
**BPNSF4**	-.331	-.268	-.265	-.276	.290	.181	.347	.164	-.368	-.265	-.316	-.289	1.000			
**BPNSF12**	-.370	-.261	-.353	-.325	.247	.312	.391	.264	-.371	-.334	-.389	-.387	.343	1.000		
**BPNSF16**	-.407	-.342	-.383	-.426	.232	.326	.349	.306	-.371	-.363	-.411	-.343	.345	.526	1.000	
**BPNSF24**	-.323	-.303	-.351	-.377	.270	.348	.248	.217	-.431	-.349	-.419	-.417	.296	.493	.446	[illegible]
**BPNSF5**	.237	.296	.177	.195	-.158	-.208	-.174	-.213	.246	.210	.178	.194	-.088	-.296	-.310	[illegible]
**BPNSF10**	.250	.440	.218	.220	-.179	-.245	-.174	-.205	.256	.318	.179	.152	-.226	-.302	-.267	[illegible]
**BPNSF15**	.156	.218	.142	.072	-.193	-.160	-.006	-.175	.059	.167	.120	.115	-.153	-.263	-.144	[illegible]
**BPNSF18**	-.143	-.048	-.029	-.106	.007	.042	-.067	.095	-.052	-.021	-.109	-.028	-.065	-.051	.010	[illegible]
**BPNSF1**	-.193	-.184	-.116	-.177	.218	.111	.085	.195	-.132	-.122	-.162	-.144	.256	.190	.155	[illegible]
**BPNSF7**	-.180	-.240	-.121	-.269	.176	.297	.211	.319	-.306	-.104	-.180	-.154	.079	.099	.084	[illegible]
**BPNSF13**	-.373	-.333	-.344	-.390	.311	.356	.589	.438	-.366	-.252	-.384	-.271	.266	.432	.336	[illegible]
**BPNSF19**	-.309	-.374	-.379	-.450	.288	.302	.415	.536	-.426	-.269	-.416	-.287	.162	.317	.320	[illegible]

Sample Correlations - Upper Bounds (PC) (g1 - Measurement weights)

	BPNSF6	BPNSF11	BPNSF17	BPNSF23	BPNSF3	BPNSF9	BPNSF14	BPNSF21	BPNSF2	BPNSF8	BPNSF20	BPNSF22	BPNSF4	BPNSF12	BPNSF16	BPNS[illegible]
**BPNSF6**	1.000															
**BPNSF11**	.617	1.000														
**BPNSF17**	.608	.617	1.000													
**BPNSF23**	.615	.635	.626	1.000												
**BPNSF3**	-.295	-.259	-.254	-.386	1.000											
**BPNSF9**	-.308	-.297	-.309	-.394	.578	1.000										
**BPNSF14**	-.264	-.233	-.294	-.346	.573	.635	1.000									

	BPNSF6	BPNSF11	BPNSF17	BPNSF23	BPNSF3	BPNSF9	BPNSF14	BPNSF21	BPNSF2	BPNSF8	BPNSF20	BPNSF22	BPNSF4	BPNSF12	BPNSF16	BPNS
**BPNSF21**	-.213	-.227	-.166	-.334	.470	.536	.624	1.000								
**BPNSF2**	.557	.589	.496	.651	-.347	-.304	-.318	-.283	1.000							
**BPNSF8**	.607	.618	.579	.550	-.172	-.264	-.196	-.088	.596	1.000						
**BPNSF20**	.559	.555	.612	.691	-.297	-.305	-.294	-.245	.614	.597	1.000					
**BPNSF22**	.453	.516	.434	.554	-.217	-.192	-.110	-.111	.548	.619	.575	1.000				
**BPNSF4**	-.167	-.100	-.104	-.118	.471	.352	.451	.327	-.186	-.097	-.177	-.124	1.000			
**BPNSF12**	-.198	-.102	-.188	-.189	.418	.477	.571	.434	-.211	-.159	-.228	-.231	.508	1.000		
**BPNSF16**	-.224	-.188	-.223	-.259	.409	.499	.536	.466	-.201	-.192	-.236	-.193	.483	.668	1.000	
**BPNSF24**	-.155	-.141	-.189	-.210	.441	.518	.437	.399	-.272	-.178	-.250	-.247	.463	.629	.607	1.0
**BPNSF5**	.395	.442	.364	.354	-.005	-.057	-.029	-.051	.393	.382	.344	.359	.062	-.133	-.149	-.1
**BPNSF10**	.413	.582	.403	.384	-.007	-.071	.016	-.032	.413	.480	.339	.314	-.024	-.117	-.089	-.0
**BPNSF15**	.305	.381	.310	.223	-.006	.018	.158	.007	.230	.354	.264	.285	.020	-.088	.048	-.0
**BPNSF18**	.017	.119	.135	.049	.178	.218	.128	.279	.097	.159	.048	.144	.102	.152	.201	.1
**BPNSF1**	-.033	-.041	.042	-.034	.377	.272	.254	.359	.010	.038	-.026	-.001	.413	.354	.303	.3
**BPNSF7**	-.022	-.093	.033	-.132	.360	.454	.386	.478	-.153	.048	-.026	-.003	.243	.274	.245	.2
**BPNSF13**	-.195	-.156	-.180	-.234	.469	.528	.712	.592	-.202	-.076	-.217	-.109	.416	.600	.511	.4
**BPNSF19**	-.162	-.213	-.212	-.319	.466	.472	.589	.679	-.259	-.075	-.239	-.119	.346	.474	.496	.4

Sample Correlations - Two Tailed Significance (PC) (g1 - Measurement weights)

	BPNSF6	BPNSF11	BPNSF17	BPNSF23	BPNSF3	BPNSF9	BPNSF14	BPNSF21	BPNSF2	BPNSF8	BPNSF20	BPNSF22	BPNSF4	BPNSF12	BPNSF16	BPNS
**BPNSF6**	...															
**BPNSF11**	.010	...														
**BPNSF17**	.010	.010	...													
**BPNSF23**	.010	.010	.010	...												
**BPNSF3**	.010	.010	.010	.010	...											
**BPNSF9**	.010	.010	.010	.010	.010	...										
**BPNSF14**	.010	.010	.010	.010	.010	.010	...									
**BPNSF21**	.010	.010	.010	.010	.010	.010	.010	...								
**BPNSF2**	.010	.010	.010	.010	.010	.010	.010	.010	...							
**BPNSF8**	.010	.010	.010	.010	.010	.010	.010	.010	.010	...						
**BPNSF20**	.010	.010	.010	.010	.010	.010	.010	.010	.010	.010	...					
**BPNSF22**	.010	.010	.010	.010	.010	.010	.010	.010	.010	.010	.010	...				
**BPNSF4**	.010	.010	.010	.010	.010	.010	.010	.010	.010	.010	.010	.010	...			
**BPNSF12**	.010	.010	.010	.010	.010	.010	.010	.010	.010	.010	.010	.010	.010	...		
**BPNSF16**	.010	.010	.010	.010	.010	.010	.010	.010	.010	.010	.010	.010	.010	.010	...	
**BPNSF24**	.010	.010	.010	.010	.010	.010	.010	.010	.010	.010	.010	.010	.010	.010	.010	
**BPNSF5**	.010	.010	.010	.010	.077	.012	.044	.016	.010	.010	.010	.010	.721	.010	.010	.0
**BPNSF10**	.010	.010	.010	.010	.077	.010	.193	.010	.010	.010	.010	.010	.039	.010	.010	.0
**BPNSF15**	.010	.010	.010	.010	.094	.201	.157	.142	.010	.010	.010	.010	.196	.010	.371	.0
**BPNSF18**	.207	.487	.403	.569	.078	.041	.589	.010	.702	.197	.400	.271	.783	.336	.078	.5
**BPNSF1**	.027	.017	.456	.016	.010	.010	.010	.010	.145	.339	.033	.095	.010	.010	.010	.0
**BPNSF7**	.025	.010	.268	.010	.010	.010	.010	.010	.010	.519	.010	.073	.010	.010	.015	.0
**BPNSF13**	.010	.010	.010	.010	.010	.010	.010	.010	.010	.010	.010	.010	.010	.010	.010	.0
**BPNSF19**	.010	.010	.010	.010	.010	.010	.010	.010	.010	.010	.010	.010	.010	.010	.010	.0

Sample Means (g1 - Measurement weights)

Sample Means - Lower Bounds (PC) (g1 - Measurement weights)

	BPNSF6	BPNSF11	BPNSF17	BPNSF23	BPNSF3	BPNSF9	BPNSF14	BPNSF21	BPNSF2	BPNSF8	BPNSF20	BPNSF22	BPNSF4	BPNSF12	BPNSF16	BPNSF
**BPNSF6**	2.319	2.572	2.304	1.943	5.618	5.729	5.485	5.242	2.103	2.626	2.215	2.901	4.970	5.123	5.014	5.13

Sample Means - Upper Bounds (PC) (g1 - Measurement weights)

	BPNSF6	BPNSF11	BPNSF17	BPNSF23	BPNSF3	BPNSF9	BPNSF14	BPNSF21	BPNSF2	BPNSF8	BPNSF20	BPNSF22	BPNSF4	BPNSF12	BPNSF16	BPNSF
**BPNSF6**	2.585	2.818	2.585	2.178	5.853	5.952	5.713	5.486	2.367	2.909	2.470	3.142	5.166	5.354	5.226	5.36

Sample Means - Two Tailed Significance (PC) (g1 - Measurement weights)

	BPNSF6	BPNSF11	BPNSF17	BPNSF23	BPNSF3	BPNSF9	BPNSF14	BPNSF21	BPNSF2	BPNSF8	BPNSF20	BPNSF22	BPNSF4	BPNSF12	BPNSF16	BPNSF
**BPNSF6**	.010	.010	.010	.010	.010	.010	.010	.010	.010	.010	.010	.010	.010	.010	.010	.01

Bias-corrected percentile method (g1 - Measurement weights)

90% confidence intervals (bias-corrected percentile method)

Scalar Estimates (g1 - Measurement weights)

Regression Weights: (g1 - Measurement weights)

Parameter	Estimate	Lower	Upper	P
BPNSF19 <--- F1	1.000	1.000	1.000	...
BPNSF13 <--- F1	1.146	1.055	1.257	.005
BPNSF7 <--- F1	.872	.767	.971	.010
BPNSF1 <--- F1	.774	.649	.884	.014
BPNSF18 <--- F2	1.000	1.000	1.000	...
BPNSF15 <--- F2	2.522	2.055	3.469	.007
BPNSF10 <--- F2	3.235	2.585	4.436	.009
BPNSF5 <--- F2	2.579	2.065	3.564	.008
BPNSF24 <--- F3	1.000	1.000	1.000	...
BPNSF16 <--- F3	1.125	1.037	1.216	.011
BPNSF12 <--- F3	1.221	1.145	1.303	.006
BPNSF4 <--- F3	.861	.766	.946	.011
BPNSF22 <--- F4	1.000	1.000	1.000	...
BPNSF20 <--- F4	1.188	1.115	1.297	.004
BPNSF8 <--- F4	1.203	1.127	1.302	.005
BPNSF2 <--- F4	1.115	1.043	1.226	.004
BPNSF21 <--- F5	1.000	1.000	1.000	...
BPNSF14 <--- F5	1.090	1.028	1.194	.006
BPNSF9 <--- F5	1.038	.967	1.139	.007
BPNSF3 <--- F5	.889	.795	.992	.013
BPNSF23 <--- F6	1.000	1.000	1.000	...
BPNSF17 <--- F6	.977	.910	1.028	.023
BPNSF11 <--- F6	.972	.916	1.036	.009
BPNSF6 <--- F6	.870	.811	.938	.009

Standardized Regression Weights: (g1 - Measurement weights)

Parameter	Estimate	Lower	Upper	P
BPNSF19 <--- F1	.646	.550	.690	.032
BPNSF13 <--- F1	.762	.702	.807	.011
BPNSF7 <--- F1	.521	.456	.578	.012
BPNSF1 <--- F1	.462	.411	.518	.009
BPNSF18 <--- F2	.237	.178	.287	.011
BPNSF15 <--- F2	.579	.515	.642	.010
BPNSF10 <--- F2	.700	.654	.769	.004
BPNSF5 <--- F2	.593	.544	.642	.009
BPNSF24 <--- F3	.689	.632	.730	.018
BPNSF16 <--- F3	.743	.676	.793	.018
BPNSF12 <--- F3	.795	.743	.830	.013
BPNSF4 <--- F3	.590	.548	.647	.005
BPNSF22 <--- F4	.679	.635	.730	.006
BPNSF20 <--- F4	.765	.724	.812	.006
BPNSF8 <--- F4	.733	.682	.783	.006
BPNSF2 <--- F4	.700	.651	.759	.005
BPNSF21 <--- F5	.687	.636	.739	.009
BPNSF14 <--- F5	.764	.683	.821	.021
BPNSF9 <--- F5	.710	.647	.763	.015
BPNSF3 <--- F5	.631	.561	.693	.015
BPNSF23 <--- F6	.796	.747	.841	.009
BPNSF17 <--- F6	.723	.677	.774	.009
BPNSF11 <--- F6	.747	.703	.794	.005
BPNSF6 <--- F6	.681	.622	.737	.007

Intercepts: (g1 - Measurement weights)

Parameter	Estimate	Lower	Upper	P
**BPNSF19**	5.314	5.188	5.415	.020
**BPNSF13**	5.196	5.056	5.298	.020
**BPNSF7**	4.945	4.805	5.057	.016
**BPNSF1**	4.595	4.485	4.712	.012
**BPNSF18**	4.383	4.278	4.496	.007
**BPNSF15**	3.793	3.658	3.939	.009
**BPNSF10**	3.294	3.164	3.407	.012
**BPNSF5**	3.688	3.570	3.830	.006
**BPNSF24**	5.244	5.135	5.362	.014
**BPNSF16**	5.112	5.002	5.212	.019
**BPNSF12**	5.230	5.113	5.330	.021
**BPNSF4**	5.068	4.947	5.150	.032
**BPNSF22**	3.015	2.901	3.142	.010

Parameter	Estimate	Lower	Upper	P
**BPNSF20**	2.351	2.225	2.483	.006
**BPNSF8**	2.768	2.631	2.909	.009
**BPNSF2**	2.230	2.118	2.383	.005
**BPNSF21**	5.359	5.224	5.478	.016
**BPNSF14**	5.585	5.446	5.695	.020
**BPNSF9**	5.848	5.703	5.943	.023
**BPNSF3**	5.716	5.611	5.816	.016
**BPNSF23**	2.068	1.975	2.251	.002
**BPNSF17**	2.440	2.316	2.609	.005
**BPNSF11**	2.690	2.587	2.822	.005
**BPNSF6**	2.458	2.350	2.643	.004

Covariances: (g1 - Measurement weights)

Parameter	Estimate	Lower	Upper	P
F1 <--> F2	-.077	-.130	-.046	.003
F2 <--> F3	-.099	-.153	-.071	.003
F1 <--> F3	.582	.492	.718	.005
F2 <--> F4	.206	.153	.276	.005
F3 <--> F4	-.462	-.545	-.385	.008
F1 <--> F4	-.398	-.525	-.316	.003
F2 <--> F5	-.061	-.105	-.026	.007
F4 <--> F5	-.611	-.733	-.527	.003
F3 <--> F5	.612	.514	.725	.008
F1 <--> F5	.857	.692	1.065	.007
F6 <--> F5	-.853	-1.076	-.750	.002
F6 <--> F3	-.499	-.609	-.414	.008
F6 <--> F4	1.133	.981	1.349	.004
F6 <--> F2	.277	.210	.369	.007
F6 <--> F1	-.563	-.705	-.448	.006

Correlations: (g1 - Measurement weights)

Parameter	Estimate	Lower	Upper	P
F1 <--> F2	-.238	-.368	-.137	.006
F2 <--> F3	-.330	-.486	-.236	.004
F1 <--> F3	.750	.677	.845	.005
F2 <--> F4	.588	.434	.655	.032
F3 <--> F4	-.549	-.630	-.479	.006
F1 <--> F4	-.436	-.536	-.351	.005
F2 <--> F5	-.178	-.286	-.066	.012
F4 <--> F5	-.630	-.721	-.564	.004
F3 <--> F5	.741	.667	.801	.016
F1 <--> F5	.956	.901	1.022	.005
F6 <--> F5	-.709	-.777	-.643	.009
F6 <--> F3	-.479	-.554	-.395	.016
F6 <--> F4	.925	.850	.970	.020
F6 <--> F2	.637	.539	.714	.015
F6 <--> F1	-.498	-.589	-.431	.004

Variances: (g1 - Measurement weights)

Parameter	Estimate	Lower	Upper	P
**F1**	.841	.617	.997	.020
**F2**	.124	.069	.185	.012
**F3**	.715	.621	.837	.007
**F4**	.988	.828	1.164	.009
**F5**	.954	.791	1.170	.007
**F6**	1.519	1.288	1.754	.007
**e1**	1.178	.980	1.470	.003
**e2**	.799	.665	1.025	.006
**e3**	1.711	1.396	1.964	.018
**e4**	1.855	1.649	2.149	.005
**e5**	2.085	1.862	2.364	.005
**e6**	1.564	1.336	1.839	.006
**e7**	1.355	1.081	1.532	.019
**e8**	1.524	1.328	1.790	.005
**e9**	.789	.631	.945	.009
**e10**	.734	.608	.996	.002
**e11**	.619	.496	.779	.006

Parameter	Estimate	Lower	Upper	P
**e12**	.995	.842	1.124	.018
**e13**	1.154	1.001	1.335	.007
**e14**	.988	.776	1.190	.012
**e15**	1.234	1.024	1.460	.009
**e16**	1.280	1.008	1.496	.021
**e17**	1.065	.886	1.259	.007
**e18**	.808	.600	1.191	.003
**e19**	1.013	.788	1.365	.003
**e20**	1.140	.873	1.439	.007
**e21**	.879	.684	1.125	.006
**e22**	1.321	1.030	1.635	.006
**e23**	1.138	.962	1.330	.012
**e24**	1.329	1.072	1.573	.015

Matrices (g1 - Measurement weights)

Sample Covariances (g1 - Measurement weights)

Sample Covariances - Lower Bounds (BC) (g1 - Measurement weights)

	BPNSF6	BPNSF11	BPNSF17	BPNSF23	BPNSF3	BPNSF9	BPNSF14	BPNSF21	BPNSF2	BPNSF8	BPNSF20	BPNSF22	BPNSF4	BPNSF12	BPNSF16	BPNS[illegible]
**BPNSF6**	2.397															
**BPNSF11**	1.225	2.460														
**BPNSF17**	1.121	1.186	2.247													
**BPNSF23**	1.087	1.221	1.115	2.069												
**BPNSF3**	-1.179	-.979	-.974	-1.230	1.656											
**BPNSF9**	-1.161	-1.197	-1.086	-1.237	.792	1.689										
**BPNSF14**	-1.043	-1.010	-1.078	-1.166	.770	.902	1.746									
**BPNSF21**	-.887	-.893	-.796	-1.079	.567	.734	.911	1.776								
**BPNSF2**	1.009	1.161	.906	1.156	-1.117	-1.091	-1.128	-.990	2.387							
**BPNSF8**	1.192	1.251	1.071	.921	-.874	-1.035	-.844	-.617	1.160	2.418						
**BPNSF20**	.974	.969	1.053	1.247	-1.006	-1.003	-1.093	-.914	1.145	1.065	2.026					
**BPNSF22**	.685	.863	.604	.848	-.821	-.709	-.582	-.620	.966	1.104	.936	1.861				
**BPNSF4**	-.702	-.549	-.552	-.519	.488	.321	.579	.282	-.757	-.633	-.628	-.512	1.318			
**BPNSF12**	-.823	-.569	-.747	-.637	.410	.515	.708	.525	-.776	-.753	-.766	-.746	.541	1.467		
**BPNSF16**	-.874	-.753	-.836	-.847	.398	.579	.634	.530	-.809	-.784	-.823	-.646	.506	.842	1.424	
**BPNSF24**	-.650	-.611	-.698	-.713	.459	.587	.435	.392	-.925	-.726	-.760	-.752	.448	.780	.675	1.3[illegible]
**BPNSF5**	.566	.756	.436	.458	-.324	-.451	-.370	-.458	.622	.537	.396	.454	-.195	-.571	-.667	-.5[illegible]
**BPNSF10**	.628	1.119	.577	.570	-.385	-.549	-.408	-.491	.701	.811	.438	.351	-.454	-.664	-.603	-.4[illegible]
**BPNSF15**	.369	.537	.355	.168	-.422	-.334	.012	-.402	.132	.421	.248	.218	-.313	-.587	-.308	-.3[illegible]
**BPNSF18**	-.384	-.161	-.103	-.260	-.001	.090	-.144	.179	-.113	-.134	-.243	-.074	-.098	-.091	.023	-.1[illegible]
**BPNSF1**	-.479	-.430	-.262	-.424	.460	.256	.202	.420	-.373	-.323	-.416	-.324	.472	.393	.320	.3[illegible]
**BPNSF7**	-.479	-.627	-.316	-.658	.388	.613	.479	.702	-.726	-.301	-.445	-.400	.150	.243	.203	.2[illegible]
**BPNSF13**	-.886	-.800	-.778	-.896	.580	.654	1.115	.845	-.832	-.612	-.832	-.537	.434	.715	.604	.4[illegible]
**BPNSF19**	-.754	-.870	-.939	-1.063	.615	.606	.849	1.014	-.997	-.674	-1.004	-.631	.307	.593	.613	.4[illegible]

Sample Covariances - Upper Bounds (BC) (g1 - Measurement weights)

	BPNSF6	BPNSF11	BPNSF17	BPNSF23	BPNSF3	BPNSF9	BPNSF14	BPNSF21	BPNSF2	BPNSF8	BPNSF20	BPNSF22	BPNSF4	BPNSF12	BPNSF16	BPNS[illegible]
**BPNSF6**	2.992															
**BPNSF11**	1.781	3.010														
**BPNSF17**	1.621	1.707	2.876													
**BPNSF23**	1.602	1.695	1.599	2.658												
**BPNSF3**	-.710	-.598	-.603	-.819	2.220											
**BPNSF9**	-.756	-.736	-.704	-.848	1.174	2.344										
**BPNSF14**	-.635	-.578	-.659	-.773	1.108	1.312	2.339									
**BPNSF21**	-.549	-.555	-.404	-.713	.936	1.125	1.382	2.304								
**BPNSF2**	1.506	1.702	1.414	1.658	-.761	-.732	-.731	-.612	2.909							
**BPNSF8**	1.711	1.764	1.593	1.437	-.438	-.661	-.478	-.251	1.664	2.943						
**BPNSF20**	1.507	1.467	1.568	1.752	-.669	-.656	-.643	-.542	1.607	1.572	2.595					
**BPNSF22**	1.101	1.292	.994	1.272	-.467	-.411	-.254	-.277	1.359	1.563	1.330	2.270				
**BPNSF4**	-.343	-.224	-.225	-.216	.812	.611	.830	.599	-.378	-.254	-.329	-.227	1.656			
**BPNSF12**	-.409	-.213	-.397	-.316	.734	.882	1.147	.914	-.436	-.360	-.444	-.437	.831	1.928		
**BPNSF16**	-.471	-.399	-.468	-.487	.720	.974	1.037	.882	-.437	-.415	-.463	-.359	.794	1.178	1.827	
**BPNSF24**	-.308	-.294	-.355	-.387	.811	.972	.790	.723	-.575	-.391	-.465	-.440	.712	1.087	.985	1.7[illegible]
**BPNSF5**	.987	1.163	.866	.845	.000	-.136	-.061	-.108	.997	.988	.792	.824	.104	-.263	-.315	-.2[illegible]
**BPNSF10**	1.103	1.591	1.079	1.008	.000	-.167	.029	-.077	1.180	1.312	.883	.773	-.060	-.265	-.217	-.0[illegible]
**BPNSF15**	.765	.992	.777	.541	-.016	.038	.419	.013	.581	.912	.613	.614	.023	-.200	.067	-.0[illegible]
**BPNSF18**	.011	.242	.313	.106	.336	.448	.246	.555	.244	.342	.099	.300	.185	.310	.367	.2[illegible]
**BPNSF1**	-.094	-.111	.106	-.115	.865	.573	.568	.824	-.004	.026	-.098	-.035	.753	.710	.595	.6[illegible]

	BPNSF6	BPNSF11	BPNSF17	BPNSF23	BPNSF3	BPNSF9	BPNSF14	BPNSF21	BPNSF2	BPNSF8	BPNSF20	BPNSF22	BPNSF4	BPNSF12	BPNSF16	BPNS[illegible]
**BPNSF7**	-.091	-.263	.034	-.341	.771	.990	.853	1.082	-.358	.073	-.106	-.032	.453	.610	.518	.5[illegible]
**BPNSF13**	-.477	-.427	-.410	-.512	.928	1.016	1.585	1.295	-.447	-.233	-.492	-.229	.740	1.144	.980	.8[illegible]
**BPNSF19**	-.386	-.486	-.521	-.680	1.009	.975	1.306	1.489	-.588	-.240	-.584	-.255	.625	.952	1.036	.7[illegible]

Sample Covariances - Two Tailed Significance (BC) (g1 - Measurement weights)

	BPNSF6	BPNSF11	BPNSF17	BPNSF23	BPNSF3	BPNSF9	BPNSF14	BPNSF21	BPNSF2	BPNSF8	BPNSF20	BPNSF22	BPNSF4	BPNSF12	BPNSF16	BPNS[illegible]
**BPNSF6**	.006															
**BPNSF11**	.003	.003														
**BPNSF17**	.014	.007	.008													
**BPNSF23**	.008	.005	.007	.005												
**BPNSF3**	.002	.005	.003	.004	.003											
**BPNSF9**	.002	.001	.005	.003	.003	.003										
**BPNSF14**	.004	.002	.008	.004	.007	.007	.005									
**BPNSF21**	.002	.006	.004	.005	.006	.005	.002	.006								
**BPNSF2**	.007	.004	.004	.012	.009	.005	.006	.018	.012							
**BPNSF8**	.007	.006	.005	.008	.002	.002	.004	.005	.006	.009						
**BPNSF20**	.003	.005	.006	.002	.003	.004	.005	.004	.003	.006	.006					
**BPNSF22**	.009	.006	.014	.009	.002	.003	.006	.003	.004	.005	.005	.009				
**BPNSF4**	.005	.005	.004	.009	.005	.006	.006	.007	.006	.002	.004	.005	.006			
**BPNSF12**	.007	.009	.011	.019	.016	.013	.006	.003	.013	.004	.012	.008	.005	.009		
**BPNSF16**	.007	.005	.004	.009	.009	.004	.004	.007	.006	.005	.006	.007	.007	.006	.007	
**BPNSF24**	.010	.007	.016	.012	.005	.005	.010	.006	.004	.004	.010	.008	.008	.007	.010	.0[illegible]
**BPNSF5**	.012	.004	.012	.008	.100	.010	.047	.019	.006	.007	.012	.004	.577	.010	.005	.0[illegible]
**BPNSF10**	.013	.012	.008	.007	.101	.008	.169	.007	.005	.010	.006	.007	.031	.009	.004	.0[illegible]
**BPNSF15**	.021	.014	.010	.011	.089	.210	.086	.136	.012	.010	.019	.025	.142	.005	.284	.0[illegible]
**BPNSF18**	.137	.732	.493	.535	.102	.036	.624	.019	.611	.362	.372	.330	.671	.299	.074	.5[illegible]
**BPNSF1**	.018	.013	.471	.008	.003	.004	.006	.005	.093	.174	.010	.046	.006	.004	.004	.0[illegible]
**BPNSF7**	.007	.004	.174	.002	.005	.008	.005	.008	.018	.250	.004	.021	.008	.003	.004	.0[illegible]
**BPNSF13**	.003	.003	.009	.005	.007	.014	.004	.005	.012	.002	.004	.005	.005	.010	.004	.0[illegible]
**BPNSF19**	.007	.010	.003	.006	.002	.004	.004	.006	.012	.004	.003	.006	.004	.006	.002	.0[illegible]

Sample Correlations (g1 - Measurement weights)

Sample Correlations - Lower Bounds (BC) (g1 - Measurement weights)

	BPNSF6	BPNSF11	BPNSF17	BPNSF23	BPNSF3	BPNSF9	BPNSF14	BPNSF21	BPNSF2	BPNSF8	BPNSF20	BPNSF22	BPNSF4	BPNSF12	BPNSF16	BPNS[illegible]
**BPNSF6**	1.000															
**BPNSF11**	.487	1.000														
**BPNSF17**	.444	.462	1.000													
**BPNSF23**	.479	.510	.472	1.000												
**BPNSF3**	-.498	-.425	-.430	-.546	1.000											
**BPNSF9**	-.496	-.455	-.465	-.550	.415	1.000										
**BPNSF14**	-.447	-.410	-.458	-.507	.379	.462	1.000									
**BPNSF21**	-.366	-.373	-.347	-.464	.311	.379	.447	1.000								
**BPNSF2**	.395	.448	.337	.488	-.500	-.465	-.465	-.425	1.000							
**BPNSF8**	.458	.468	.419	.375	-.348	-.434	-.361	-.261	.464	1.000						
**BPNSF20**	.412	.406	.458	.545	-.462	-.458	-.478	-.418	.458	.449	1.000					
**BPNSF22**	.309	.364	.257	.403	-.402	-.349	-.288	-.293	.430	.491	.441	1.000				
**BPNSF4**	-.334	-.273	-.279	-.264	.296	.183	.342	.168	-.370	-.283	-.331	-.291	1.000			
**BPNSF12**	-.371	-.261	-.341	-.314	.237	.309	.374	.281	-.370	-.346	-.383	-.387	.337	1.000		
**BPNSF16**	-.417	-.347	-.380	-.426	.220	.323	.341	.294	-.370	-.371	-.399	-.347	.345	.525	1.000	
**BPNSF24**	-.317	-.284	-.335	-.371	.261	.354	.244	.222	-.444	-.359	-.418	-.417	.298	.491	.430	1.0[illegible]
**BPNSF5**	.235	.295	.174	.193	-.153	-.204	-.174	-.202	.243	.210	.163	.206	-.104	-.294	-.323	-.2[illegible]
**BPNSF10**	.249	.440	.227	.232	-.178	-.239	-.175	-.204	.256	.319	.184	.152	-.234	-.303	-.285	-.2[illegible]
**BPNSF15**	.152	.212	.138	.072	-.194	-.161	-.001	-.179	.064	.169	.106	.102	-.162	-.270	-.153	-.1[illegible]
**BPNSF18**	-.159	-.069	-.048	-.113	.001	.051	-.067	.088	-.047	-.060	-.110	-.031	-.056	-.051	.014	-.0[illegible]
**BPNSF1**	-.199	-.184	-.114	-.190	.225	.116	.074	.197	-.148	-.134	-.181	-.150	.256	.221	.164	.1[illegible]
**BPNSF7**	-.208	-.253	-.129	-.281	.204	.297	.211	.317	-.296	-.123	-.190	-.176	.080	.114	.088	.1[illegible]
**BPNSF13**	-.378	-.342	-.343	-.387	.311	.349	.592	.437	-.371	-.262	-.399	-.273	.266	.432	.353	.2[illegible]
**BPNSF19**	-.312	-.373	-.390	-.448	.298	.302	.413	.521	-.416	-.286	-.422	-.287	.177	.317	.330	.2[illegible]

Sample Correlations - Upper Bounds (BC) (g1 - Measurement weights)

	BPNSF6	BPNSF11	BPNSF17	BPNSF23	BPNSF3	BPNSF9	BPNSF14	BPNSF21	BPNSF2	BPNSF8	BPNSF20	BPNSF22	BPNSF4	BPNSF12	BPNSF16	BPNS[illegible]
**BPNSF6**	1.000															
**BPNSF11**	.641	1.000														
**BPNSF17**	.600	.616	1.000													
**BPNSF23**	.629	.639	.627	1.000												
**BPNSF3**	-.315	-.264	-.256	-.401	1.000											

	BPNSF6	BPNSF11	BPNSF17	BPNSF23	BPNSF3	BPNSF9	BPNSF14	BPNSF21	BPNSF2	BPNSF8	BPNSF20	BPNSF22	BPNSF4	BPNSF12	BPNSF16	BPNS
**BPNSF9**	-.329	-.308	-.310	-.394	.589	1.000										
**BPNSF14**	-.266	-.248	-.295	-.348	.563	.627	1.000									
**BPNSF21**	-.223	-.227	-.174	-.334	.479	.549	.624	1.000								
**BPNSF2**	.558	.592	.507	.637	-.348	-.304	-.312	-.276	1.000							
**BPNSF8**	.607	.615	.587	.549	-.177	-.277	-.204	-.106	.609	1.000						
**BPNSF20**	.582	.567	.617	.694	-.323	-.310	-.302	-.245	.614	.602	1.000					
**BPNSF22**	.453	.519	.421	.548	-.237	-.207	-.112	-.117	.559	.630	.580	1.000				
**BPNSF4**	-.168	-.114	-.116	-.109	.476	.352	.450	.343	-.193	-.119	-.193	-.125	1.000			
**BPNSF12**	-.204	-.102	-.185	-.162	.411	.477	.566	.443	-.206	-.168	-.225	-.231	.499	1.000		
**BPNSF16**	-.250	-.192	-.221	-.261	.398	.486	.532	.455	-.200	-.201	-.235	-.196	.484	.666	1.000	
**BPNSF24**	-.153	-.133	-.178	-.205	.427	.524	.430	.409	-.287	-.189	-.249	-.244	.467	.628	.599	1.0
**BPNSF5**	.393	.441	.349	.348	.004	-.052	-.029	-.041	.389	.383	.336	.368	.057	-.127	-.163	-.1
**BPNSF10**	.412	.582	.407	.388	-.007	-.069	.013	-.031	.412	.483	.350	.316	-.040	-.119	-.107	-.0
**BPNSF15**	.294	.371	.303	.223	-.009	.018	.173	.002	.233	.355	.256	.268	.018	-.091	.034	-.0
**BPNSF18**	.005	.101	.128	.046	.165	.223	.127	.266	.101	.143	.046	.144	.108	.152	.205	.1
**BPNSF1**	-.043	-.042	.045	-.047	.380	.275	.253	.360	-.002	.010	-.040	-.015	.413	.386	.304	.3
**BPNSF7**	-.035	-.103	.013	-.141	.372	.455	.386	.475	-.150	.030	-.034	-.012	.244	.280	.250	.2
**BPNSF13**	-.203	-.179	-.180	-.229	.469	.520	.713	.589	-.203	-.095	-.234	-.116	.416	.600	.516	.4
**BPNSF19**	-.166	-.208	-.224	-.316	.474	.472	.588	.673	-.256	-.101	-.242	-.119	.349	.474	.506	.4

Sample Correlations - Two Tailed Significance (BC) (g1 - Measurement weights)

	BPNSF6	BPNSF11	BPNSF17	BPNSF23	BPNSF3	BPNSF9	BPNSF14	BPNSF21	BPNSF2	BPNSF8	BPNSF20	BPNSF22	BPNSF4	BPNSF12	BPNSF16	BPNS
**BPNSF6**	...															
**BPNSF11**	.003	...														
**BPNSF17**	.019	.012	...													
**BPNSF23**	.004	.007	.009	...												
**BPNSF3**	.003	.008	.007	.005	...											
**BPNSF9**	.003	.004	.008	.010	.007	...										
**BPNSF14**	.008	.003	.009	.007	.016	.021	...									
**BPNSF21**	.005	.011	.006	.012	.005	.008	.010	...								
**BPNSF2**	.009	.006	.006	.023	.009	.010	.013	.014	...							
**BPNSF8**	.010	.016	.005	.012	.005	.006	.004	.005	.003	...						
**BPNSF20**	.002	.005	.004	.007	.004	.006	.007	.010	.009	.005	...					
**BPNSF22**	.010	.008	.019	.016	.003	.003	.009	.007	.004	.006	.005	...				
**BPNSF4**	.007	.005	.005	.018	.006	.008	.023	.006	.007	.002	.003	.009	...			
**BPNSF12**	.008	.010	.019	.023	.019	.011	.012	.006	.013	.006	.015	.010	.012	...		
**BPNSF16**	.005	.005	.012	.009	.014	.013	.012	.014	.011	.009	.013	.007	.009	.012	...	
**BPNSF24**	.012	.016	.021	.013	.015	.006	.013	.006	.005	.005	.012	.011	.009	.012	.019	
**BPNSF5**	.013	.011	.014	.012	.111	.015	.044	.023	.011	.009	.019	.005	.594	.012	.006	.0
**BPNSF10**	.011	.010	.006	.007	.081	.012	.177	.011	.011	.009	.005	.009	.027	.009	.003	.0
**BPNSF15**	.021	.026	.012	.010	.085	.192	.117	.111	.009	.009	.018	.026	.164	.009	.284	.0
**BPNSF18**	.144	.732	.525	.519	.096	.030	.606	.021	.611	.349	.372	.294	.670	.349	.070	.5
**BPNSF1**	.015	.016	.503	.009	.006	.007	.014	.009	.093	.166	.011	.049	.010	.003	.007	.0
**BPNSF7**	.007	.004	.174	.004	.005	.009	.009	.013	.012	.283	.006	.028	.009	.004	.012	.0
**BPNSF13**	.005	.004	.011	.012	.010	.019	.007	.011	.009	.004	.003	.006	.009	.010	.005	.0
**BPNSF19**	.007	.012	.005	.012	.005	.011	.011	.018	.013	.005	.007	.010	.007	.010	.005	.0

Sample Means (g1 - Measurement weights)

Sample Means - Lower Bounds (BC) (g1 - Measurement weights)

	BPNSF6	BPNSF11	BPNSF17	BPNSF23	BPNSF3	BPNSF9	BPNSF14	BPNSF21	BPNSF2	BPNSF8	BPNSF20	BPNSF22	BPNSF4	BPNSF12	BPNSF16	BPNSF
**BPNSF6**	2.352	2.588	2.316	1.968	5.611	5.703	5.446	5.227	2.118	2.631	2.225	2.898	4.947	5.113	4.999	5.12

Sample Means - Upper Bounds (BC) (g1 - Measurement weights)

	BPNSF6	BPNSF11	BPNSF17	BPNSF23	BPNSF3	BPNSF9	BPNSF14	BPNSF21	BPNSF2	BPNSF8	BPNSF20	BPNSF22	BPNSF4	BPNSF12	BPNSF16	BPNSF
**BPNSF6**	2.645	2.823	2.609	2.236	5.816	5.943	5.696	5.480	2.383	2.909	2.483	3.139	5.151	5.333	5.213	5.35

Sample Means - Two Tailed Significance (BC) (g1 - Measurement weights)

	BPNSF6	BPNSF11	BPNSF17	BPNSF23	BPNSF3	BPNSF9	BPNSF14	BPNSF21	BPNSF2	BPNSF8	BPNSF20	BPNSF22	BPNSF4	BPNSF12	BPNSF16	BPNSF
**BPNSF6**	.003	.005	.005	.003	.016	.023	.020	.015	.005	.009	.006	.011	.032	.019	.019	.01

g2 (g2 - Measurement weights)

Estimates (g2 - Measurement weights)

Scalar Estimates (g2 - Measurement weights)

Maximum Likelihood Estimates

Regression Weights: (g2 - Measurement weights)

			Estimate	S.E.	C.R.	P	Label
BPNSF19	<---	F1	1.000				
BPNSF13	<---	F1	1.146	.052	21.901	***	a1_1
BPNSF7	<---	F1	.872	.051	16.991	***	a2_1
BPNSF1	<---	F1	.774	.051	15.177	***	a3_1
BPNSF18	<---	F2	1.000				
BPNSF15	<---	F2	2.522	.319	7.905	***	a4_1
BPNSF10	<---	F2	3.235	.395	8.191	***	a5_1
BPNSF5	<---	F2	2.579	.322	8.003	***	a6_1
BPNSF24	<---	F3	1.000				
BPNSF16	<---	F3	1.125	.047	24.171	***	a7_1
BPNSF12	<---	F3	1.221	.048	25.585	***	a8_1
BPNSF4	<---	F3	.861	.044	19.667	***	a9_1
BPNSF22	<---	F4	1.000				
BPNSF20	<---	F4	1.188	.049	24.383	***	a10_1
BPNSF8	<---	F4	1.203	.050	23.925	***	a11_1
BPNSF2	<---	F4	1.115	.049	22.903	***	a12_1
BPNSF21	<---	F5	1.000				
BPNSF14	<---	F5	1.090	.042	25.682	***	a13_1
BPNSF9	<---	F5	1.038	.043	24.375	***	a14_1
BPNSF3	<---	F5	.889	.042	20.973	***	a15_1
BPNSF23	<---	F6	1.000				
BPNSF17	<---	F6	.977	.035	27.907	***	a16_1
BPNSF11	<---	F6	.972	.034	28.226	***	a17_1
BPNSF6	<---	F6	.870	.036	24.502	***	a18_1

Standardized Regression Weights: (g2 - Measurement weights)

			Estimate
BPNSF19	<---	F1	.595
BPNSF13	<---	F1	.758
BPNSF7	<---	F1	.558
BPNSF1	<---	F1	.512
BPNSF18	<---	F2	.261
BPNSF15	<---	F2	.581
BPNSF10	<---	F2	.738
BPNSF5	<---	F2	.640
BPNSF24	<---	F3	.696
BPNSF16	<---	F3	.779
BPNSF12	<---	F3	.841
BPNSF4	<---	F3	.618
BPNSF22	<---	F4	.680
BPNSF20	<---	F4	.763
BPNSF8	<---	F4	.725
BPNSF2	<---	F4	.728
BPNSF21	<---	F5	.706
BPNSF14	<---	F5	.778
BPNSF9	<---	F5	.715
BPNSF3	<---	F5	.614
BPNSF23	<---	F6	.777
BPNSF17	<---	F6	.748
BPNSF11	<---	F6	.700
BPNSF6	<---	F6	.632

Intercepts: (g2 - Measurement weights)

	Estimate	S.E.	C.R.	P	Label
**BPNSF19**	5.243	.074	70.896	***	i1_2
**BPNSF13**	5.197	.067	78.113	***	i2_2
**BPNSF7**	4.850	.069	70.528	***	i3_2
**BPNSF1**	4.867	.067	73.091	***	i4_2
**BPNSF18**	4.106	.072	57.107	***	i5_2
**BPNSF15**	3.605	.082	44.228	***	i6_2
**BPNSF10**	3.152	.082	38.272	***	i7_2
**BPNSF5**	3.637	.076	48.070	***	i8_2

	Estimate	S.E.	C.R.	P	Label
**BPNSF24**	5.311	.070	76.142	***	i9_2
**BPNSF16**	5.218	.070	74.394	***	i10_2
**BPNSF12**	5.314	.070	75.389	***	i11_2
**BPNSF4**	5.077	.068	75.129	***	i12_2
**BPNSF22**	3.078	.076	40.395	***	i13_2
**BPNSF20**	2.439	.081	30.239	***	i14_2
**BPNSF8**	2.731	.086	31.759	***	i15_2
**BPNSF2**	2.309	.079	29.076	***	i16_2
**BPNSF21**	5.240	.070	74.890	***	i17_2
**BPNSF14**	5.607	.069	81.051	***	i18_2
**BPNSF9**	5.725	.072	79.898	***	i19_2
**BPNSF3**	5.619	.071	78.594	***	i20_2
**BPNSF23**	2.173	.078	27.829	***	i21_2
**BPNSF17**	2.474	.079	31.207	***	i22_2
**BPNSF11**	2.761	.084	32.747	***	i23_2
**BPNSF6**	2.616	.084	31.295	***	i24_2

Covariances: (g2 - Measurement weights)

	Estimate	S.E.	C.R.	P	Label
F1 <--> F2	-.204	.036	-5.707	***	ccc1_2
F2 <--> F3	-.187	.034	-5.429	***	ccc2_2
F1 <--> F3	.810	.078	10.401	***	ccc3_2
F2 <--> F4	.353	.053	6.688	***	ccc4_2
F3 <--> F4	-.738	.079	-9.396	***	ccc5_2
F1 <--> F4	-.605	.073	-8.255	***	ccc6_2
F2 <--> F5	-.187	.035	-5.329	***	ccc7_2
F4 <--> F5	-.679	.078	-8.710	***	ccc8_2
F3 <--> F5	.866	.081	10.697	***	ccc9_2
F1 <--> F5	.940	.085	11.120	***	ccc10_2
F6 <--> F5	-.903	.093	-9.700	***	ccc11_2
F6 <--> F3	-.677	.084	-8.036	***	ccc12_2
F6 <--> F4	1.218	.108	11.295	***	ccc13_2
F6 <--> F2	.374	.057	6.570	***	ccc14_2
F6 <--> F1	-.632	.082	-7.690	***	ccc15_2

Correlations: (g2 - Measurement weights)

	Estimate
F1 <--> F2	-.567
F2 <--> F3	-.470
F1 <--> F3	.869
F2 <--> F4	.831
F3 <--> F4	-.672
F1 <--> F4	-.608
F2 <--> F5	-.462
F4 <--> F5	-.609
F3 <--> F5	.829
F1 <--> F5	.992
F6 <--> F5	-.691
F6 <--> F3	-.527
F6 <--> F4	.888
F6 <--> F2	.752
F6 <--> F1	-.542

Variances: (g2 - Measurement weights)

	Estimate	S.E.	C.R.	P	Label
**F1**	.845	.098	8.598	***	vvv1_2
**F2**	.154	.039	3.975	***	vvv2_2
**F3**	1.028	.102	10.037	***	vvv3_2
**F4**	1.172	.118	9.891	***	vvv4_2
**F5**	1.062	.105	10.074	***	vvv5_2
**F6**	1.606	.148	10.887	***	vvv6_2
**e1**	1.540	.111	13.837	***	v1_2
**e2**	.821	.070	11.688	***	v2_2
**e3**	1.421	.101	14.033	***	v3_2
**e4**	1.427	.100	14.215	***	v4_2
**e5**	2.100	.144	14.540	***	v5_2
**e6**	1.918	.146	13.120	***	v6_2

	Estimate	S.E.	C.R.	P	Label
**e7**	1.348	.128	10.545	***	v7_2
**e8**	1.473	.118	12.469	***	v8_2
**e9**	1.094	.084	13.064	***	v9_2
**e10**	.845	.071	11.924	***	v10_2
**e11**	.635	.062	10.215	***	v11_2
**e12**	1.230	.090	13.654	***	v12_2
**e13**	1.360	.102	13.288	***	v13_2
**e14**	1.184	.096	12.276	***	v14_2
**e15**	1.528	.119	12.831	***	v15_2
**e16**	1.292	.101	12.789	***	v16_2
**e17**	1.072	.081	13.233	***	v17_2
**e18**	.823	.067	12.215	***	v18_2
**e19**	1.094	.083	13.138	***	v19_2
**e20**	1.390	.100	13.879	***	v20_2
**e21**	1.052	.089	11.789	***	v21_2
**e22**	1.208	.098	12.327	***	v22_2
**e23**	1.581	.122	12.964	***	v23_2
**e24**	1.831	.135	13.538	***	v24_2

Matrices (g2 - Measurement weights)

Residual Covariances (g2 - Measurement weights)

	BPNSF6	BPNSF11	BPNSF17	BPNSF23	BPNSF3	BPNSF9	BPNSF14	BPNSF21	BPNSF2	BPNSF8	BPNSF20	BPNSF22	BPNSF4	BPNSF12	BPNSF16	BPNS[illegible]
**BPNSF6**	-.113															
**BPNSF11**	-.174	-.208														
**BPNSF17**	-.020	.013	.153													
**BPNSF23**	-.076	-.122	.167	.070												
**BPNSF3**	-.156	.115	-.080	-.086	.047											
**BPNSF9**	.031	-.019	-.240	-.110	.150	-.039										
**BPNSF14**	.180	.172	-.167	.127	-.051	.052	-.068									
**BPNSF21**	.086	.029	.099	.014	.105	-.109	-.041	.090								
**BPNSF2**	.007	-.204	.038	.014	-.092	.037	.024	.003	-.139							
**BPNSF8**	.016	-.171	-.006	-.155	.044	.056	.180	.065	-.092	-.173						
**BPNSF20**	-.183	-.114	.066	.293	-.134	.012	-.009	-.192	-.040	-.064	.080					
**BPNSF22**	-.059	-.021	.105	.081	.142	-.055	.027	-.141	-.001	-.005	.217	.207				
**BPNSF4**	-.138	-.025	-.098	-.116	.742	.101	.013	.345	-.148	-.012	-.204	-.129	.162			
**BPNSF12**	.141	.096	-.044	.030	-.060	-.120	-.097	.023	.100	.239	.140	-.138	.064	-.116		
**BPNSF16**	.144	.047	-.039	.067	-.108	-.155	-.033	.202	.117	.197	-.014	-.132	.030	.003	.011	
**BPNSF24**	.041	.011	-.259	-.052	-.140	-.108	.030	.115	-.059	-.018	-.179	-.397	.050	-.069	.094	[illegible]
**BPNSF5**	.062	-.274	-.205	-.069	-.015	.176	.168	-.036	-.178	.023	-.066	.043	-.312	.100	.142	[illegible]
**BPNSF10**	-.165	.226	.022	.041	-.092	-.146	-.084	-.285	-.155	-.037	.017	.138	-.188	.038	.027	[illegible]
**BPNSF15**	.047	.250	.080	-.070	.023	.171	.214	.023	-.137	.029	.057	.277	-.124	.086	.085	[illegible]
**BPNSF18**	-.108	.277	.216	-.008	.177	.064	.132	-.060	-.041	.079	.244	.169	-.009	.052	.008	[illegible]
**BPNSF1**	.139	.125	.105	.077	.169	-.092	-.029	.167	-.118	.091	-.077	-.073	.324	-.057	-.066	[illegible]
**BPNSF7**	.438	.021	-.147	.210	-.037	.100	-.043	-.006	.090	.028	.098	-.172	.156	-.109	-.045	[illegible]
**BPNSF13**	.096	-.073	.004	.043	-.051	-.197	-.037	.139	.139	.053	.044	-.147	.119	.072	-.004	[illegible]
**BPNSF19**	-.059	-.241	-.281	-.241	-.063	.005	.007	.214	.112	.034	-.125	-.192	.123	-.165	-.135	[illegible]

Residual Means (g2 - Measurement weights)

	BPNSF6	BPNSF11	BPNSF17	BPNSF23	BPNSF3	BPNSF9	BPNSF14	BPNSF21	BPNSF2	BPNSF8	BPNSF20	BPNSF22	BPNSF4	BPNSF12	BPNSF16	BPNSF24	BP[illegible]
	.000	.000	.000	.000	.000	.000	.000	.000	.000	.000	.000	.000	.000	.000	.000	.000	[illegible]

Standardized Residual Covariances (g2 - Measurement weights)

	BPNSF6	BPNSF11	BPNSF17	BPNSF23	BPNSF3	BPNSF9	BPNSF14	BPNSF21	BPNSF2	BPNSF8	BPNSF20	BPNSF22	BPNSF4	BPNSF12	BPNSF16	BPNS[illegible]
**BPNSF6**	-.546															
**BPNSF11**	-1.080	-.993														
**BPNSF17**	-.130	.081	.825													
**BPNSF23**	-.499	-.782	1.116	.389												
**BPNSF3**	-1.211	.874	-.642	-.700	.308											
**BPNSF9**	.239	-.139	-1.899	-.881	1.282	-.254										
**BPNSF14**	1.410	1.324	-1.357	1.037	-.445	.437	-.481									
**BPNSF21**	.676	.226	.806	.113	.918	-.930	-.355	.625								
**BPNSF2**	.045	-1.332	.262	.097	-.752	.299	.195	.027	-.746							
**BPNSF8**	.099	-1.029	-.035	-.988	.333	.413	1.370	.491	-.571	-.790						
**BPNSF20**	-1.195	-.728	.444	1.969	-1.073	.092	-.072	-1.547	-.260	-.389	.419					
**BPNSF22**	-.411	-.148	.755	.591	1.211	-.461	.232	-1.215	-.004	-.032	1.499	1.209				
**BPNSF4**	-1.144	-.204	-.852	-1.025	7.018	.936	.126	3.282	-1.268	-.097	-1.711	-1.151	1.199			

	BPNSF6	BPNSF11	BPNSF17	BPNSF23	BPNSF3	BPNSF9	BPNSF14	BPNSF21	BPNSF2	BPNSF8	BPNSF20	BPNSF22	BPNSF4	BPNSF12	BPNSF16	BPNS[illegible]
**BPNSF12**	1.107	.736	-.357	.251	-.522	-1.019	-.839	.199	.793	1.743	1.081	-1.145	.572	-.788		
**BPNSF16**	1.142	.363	-.317	.560	-.959	-1.338	-.292	1.791	.939	1.463	-.110	-1.112	.275	.023	.075	
**BPNSF24**	.324	.084	-2.162	-.443	-1.271	-.957	.269	1.042	-.480	-.134	-1.432	-3.412	.464	-.579	.813	.2[illegible]
**BPNSF5**	.452	-1.948	-1.543	-.524	-.132	1.521	1.495	-.322	-1.325	.160	-.482	.334	-2.869	.871	1.251	-.3[illegible]
**BPNSF10**	-1.080	1.455	.147	.283	-.735	-1.155	-.679	-2.301	-1.037	-.228	.113	.970	-1.586	.300	.212	-1.2[illegible]
**BPNSF15**	.320	1.664	.561	-.497	.188	1.377	1.782	.192	-.957	.186	.386	2.030	-1.063	.700	.700	-.8[illegible]
**BPNSF18**	-.851	2.170	1.796	-.067	1.649	.590	1.262	-.572	-.338	.606	1.984	1.460	-.092	.487	.073	-.4[illegible]
**BPNSF1**	1.174	1.043	.933	.692	1.626	-.868	-.279	1.614	-1.041	.739	-.666	-.678	3.323	-.548	-.644	.2[illegible]
**BPNSF7**	3.585	.173	-1.260	1.822	-.344	.903	-.394	-.059	.770	.221	.823	-1.534	1.540	-.999	-.417	.3[illegible]
**BPNSF13**	.800	-.598	.033	.377	-.462	-1.741	-.329	1.259	1.191	.421	.367	-1.322	1.171	.642	-.036	.7[illegible]
**BPNSF19**	-.450	-1.806	-2.229	-1.939	-.537	.043	.061	1.826	.886	.248	-.969	-1.584	1.122	-1.393	-1.158	-1.2[illegible]

Standardized Residual Means (g2 - Measurement weights)

	BPNSF6	BPNSF11	BPNSF17	BPNSF23	BPNSF3	BPNSF9	BPNSF14	BPNSF21	BPNSF2	BPNSF8	BPNSF20	BPNSF22	BPNSF4	BPNSF12	BPNSF16	BPNSF24	BP[illegible]
	.000	.000	.000	.000	.000	.000	.000	.000	.000	.000	.000	.000	.000	.000	.000	.000	[illegible]

Notes for Group/Model (g2 - Measurement weights)

The following covariance matrix is not positive definite (g2 - Measurement weights)

	F5	F4	F3	F2	F1	F6
**F5**	1.062					
**F4**	-.679	1.172				
**F3**	.866	-.738	1.028			
**F2**	-.187	.353	-.187	.154		
**F1**	.940	-.605	.810	-.204	.845	
**F6**	-.903	1.218	-.677	.374	-.632	1.606

This solution is not admissible.

Modification Indices (g2 - Measurement weights)

Covariances: (g2 - Measurement weights)

			M.I.	Par Change
e24	<-->	F1	4.581	.092
e23	<-->	F4	4.469	-.094
e23	<-->	F2	6.285	.055
e23	<-->	F1	5.866	-.098
e20	<-->	e24	7.689	-.227
e19	<-->	F6	4.597	-.096
e19	<-->	e20	6.428	.161
e18	<-->	e22	8.734	-.164
e18	<-->	e19	6.635	.131
e17	<-->	F5	5.269	-.070
e17	<-->	F1	5.673	.077
e17	<-->	e22	13.016	.223
e17	<-->	e19	8.398	-.165
e15	<-->	e24	4.917	.197
e15	<-->	e21	5.082	-.160
e14	<-->	e24	6.792	-.207
e14	<-->	e21	17.699	.267
e13	<-->	F5	9.437	.115
e13	<-->	F3	5.863	-.100
e13	<-->	F1	5.348	-.086
e13	<-->	e20	15.269	.277
e12	<-->	F5	14.759	.134
e12	<-->	F3	6.043	-.094
e12	<-->	F2	6.434	-.048
e12	<-->	F6	4.529	.102
e12	<-->	e22	4.233	.135
e12	<-->	e20	100.597	.670
e12	<-->	e18	8.470	-.156
e12	<-->	e17	4.318	.124
e11	<-->	e14	7.140	.141
e10	<-->	e20	5.921	-.143
e10	<-->	e17	7.765	.146
e9	<-->	F4	4.873	-.082
e9	<-->	e22	4.001	-.126
e9	<-->	e20	9.367	-.197

			M.I.	Par Change
e9	<-->	e13	9.701	-.202
e8	<-->	e24	9.015	.264
e8	<-->	e23	8.649	-.245
e8	<-->	e22	4.398	-.156
e8	<-->	e12	13.402	-.263
e7	<-->	F5	7.893	-.113
e7	<-->	e24	4.127	-.181
e7	<-->	e23	7.970	.238
e6	<-->	F5	5.947	.108
e6	<-->	e23	7.251	.251
e5	<-->	e23	5.308	.215
e5	<-->	e6	7.516	.279
e4	<-->	F6	10.450	.162
e4	<-->	e22	4.703	.150
e4	<-->	e20	5.226	.161
e4	<-->	e16	5.672	-.168
e4	<-->	e12	8.617	.196
e4	<-->	e7	8.326	.221
e4	<-->	e6	13.775	-.314
e4	<-->	e5	5.575	-.200
e3	<-->	e24	18.670	.354
e3	<-->	e22	7.798	-.193
e3	<-->	e21	6.123	.163
e3	<-->	e19	6.668	.164
e3	<-->	e13	5.044	-.160
e2	<-->	F3	5.857	.078
e2	<-->	e23	6.092	-.152
e2	<-->	e19	11.487	-.170
e2	<-->	e12	4.372	-.111
e2	<-->	e11	11.911	.148
e1	<-->	F6	6.824	-.137
e1	<-->	e17	5.274	.151
e1	<-->	e16	4.967	.164

Variances: (g2 - Measurement weights)

	M.I.	Par Change

Regression Weights: (g2 - Measurement weights)

			M.I.	Par Change
BPNSF3	<---	BPNSF4	4.393	.023
BPNSF21	<---	F3	4.972	.121
BPNSF2	<---	F2	4.200	-.336
BPNSF22	<---	F3	6.797	-.160
BPNSF22	<---	F2	4.980	.370
BPNSF4	<---	F5	9.169	.170
BPNSF4	<---	F2	7.330	-.423
BPNSF4	<---	F1	13.079	.226
BPNSF4	<---	F6	5.585	-.109
BPNSF4	<---	BPNSF3	5.924	.023
BPNSF24	<---	F4	4.336	-.108
BPNSF24	<---	F2	4.349	-.313
BPNSF7	<---	BPNSF6	4.553	.039
BPNSF19	<---	F6	5.767	-.123

Means: (g2 - Measurement weights)

	M.I.	Par Change

Intercepts: (g2 - Measurement weights)

	M.I.	Par Change

Bootstrap (g2 - Measurement weights)

Bootstrap standard errors (g2 - Measurement weights)

Scalar Estimates (g2 - Measurement weights)

Regression Weights: (g2 - Measurement weights)

Parameter			SE	SE-SE	Mean	Bias	SE-Bias
BPNSF19	<---	F1	.000	.000	1.000	.000	.000
BPNSF13	<---	F1	.058	.003	1.141	-.005	.004
BPNSF7	<---	F1	.060	.003	.871	.000	.004
BPNSF1	<---	F1	.073	.004	.774	.000	.005
BPNSF18	<---	F2	.000	.000	1.000	.000	.000
BPNSF15	<---	F2	.413	.021	2.575	.053	.029
BPNSF10	<---	F2	.549	.027	3.306	.072	.039
BPNSF5	<---	F2	.437	.022	2.636	.057	.031
BPNSF24	<---	F3	.000	.000	1.000	.000	.000
BPNSF16	<---	F3	.054	.003	1.126	.001	.004
BPNSF12	<---	F3	.049	.002	1.216	-.005	.003
BPNSF4	<---	F3	.052	.003	.860	-.001	.004
BPNSF22	<---	F4	.000	.000	1.000	.000	.000
BPNSF20	<---	F4	.051	.003	1.186	-.001	.004
BPNSF8	<---	F4	.050	.003	1.198	-.005	.004
BPNSF2	<---	F4	.055	.003	1.114	-.002	.004
BPNSF21	<---	F5	.000	.000	1.000	.000	.000
BPNSF14	<---	F5	.047	.002	1.092	.002	.003
BPNSF9	<---	F5	.054	.003	1.040	.002	.004
BPNSF3	<---	F5	.061	.003	.894	.005	.004
BPNSF23	<---	F6	.000	.000	1.000	.000	.000
BPNSF17	<---	F6	.034	.002	.980	.004	.002
BPNSF11	<---	F6	.038	.002	.973	.001	.003
BPNSF6	<---	F6	.037	.002	.870	.000	.003

Standardized Regression Weights: (g2 - Measurement weights)

Parameter			SE	SE-SE	Mean	Bias	SE-Bias
BPNSF19	<---	F1	.039	.002	.597	.002	.003
BPNSF13	<---	F1	.029	.001	.758	.000	.002
BPNSF7	<---	F1	.042	.002	.559	.001	.003
BPNSF1	<---	F1	.037	.002	.513	.001	.003
BPNSF18	<---	F2	.043	.002	.262	.001	.003
BPNSF15	<---	F2	.037	.002	.582	.001	.003
BPNSF10	<---	F2	.037	.002	.740	.002	.003
BPNSF5	<---	F2	.038	.002	.640	.000	.003
BPNSF24	<---	F3	.032	.002	.698	.002	.002
BPNSF16	<---	F3	.027	.001	.780	.002	.002
BPNSF12	<---	F3	.019	.001	.841	.001	.001
BPNSF4	<---	F3	.039	.002	.618	-.001	.003
BPNSF22	<---	F4	.028	.001	.679	-.001	.002
BPNSF20	<---	F4	.031	.002	.765	.002	.002
BPNSF8	<---	F4	.028	.001	.723	-.003	.002
BPNSF2	<---	F4	.030	.002	.730	.002	.002
BPNSF21	<---	F5	.030	.001	.705	-.001	.002
BPNSF14	<---	F5	.035	.002	.779	.001	.002
BPNSF9	<---	F5	.036	.002	.717	.002	.003
BPNSF3	<---	F5	.044	.002	.617	.004	.003
BPNSF23	<---	F6	.029	.001	.777	.000	.002
BPNSF17	<---	F6	.032	.002	.751	.003	.002
BPNSF11	<---	F6	.030	.001	.699	-.001	.002
BPNSF6	<---	F6	.029	.001	.631	-.001	.002

Intercepts: (g2 - Measurement weights)

Parameter	SE	SE-SE	Mean	Bias	SE-Bias
**BPNSF19**	.075	.004	5.248	.005	.005
**BPNSF13**	.069	.003	5.209	.011	.005
**BPNSF7**	.071	.004	4.865	.014	.005
**BPNSF1**	.070	.003	4.879	.012	.005
**BPNSF18**	.074	.004	4.102	-.004	.005
**BPNSF15**	.094	.005	3.603	-.002	.007
**BPNSF10**	.092	.005	3.138	-.014	.007
**BPNSF5**	.077	.004	3.641	.004	.005
**BPNSF24**	.077	.004	5.317	.006	.005
**BPNSF16**	.072	.004	5.222	.004	.005
**BPNSF12**	.067	.003	5.318	.003	.005
**BPNSF4**	.073	.004	5.080	.003	.005
**BPNSF22**	.088	.004	3.066	-.013	.006

Parameter	SE	SE-SE	Mean	Bias	SE-Bias
**BPNSF20**	.087	.004	2.426	-.014	.006
**BPNSF8**	.091	.005	2.726	-.006	.006
**BPNSF2**	.078	.004	2.299	-.009	.006
**BPNSF21**	.076	.004	5.254	.014	.005
**BPNSF14**	.067	.003	5.620	.013	.005
**BPNSF9**	.074	.004	5.731	.006	.005
**BPNSF3**	.073	.004	5.627	.007	.005
**BPNSF23**	.076	.004	2.166	-.007	.005
**BPNSF17**	.088	.004	2.462	-.012	.006
**BPNSF11**	.090	.005	2.753	-.007	.006
**BPNSF6**	.079	.004	2.613	-.003	.006

Covariances: (g2 - Measurement weights)

Parameter	SE	SE-SE	Mean	Bias	SE-Bias
F1 <--> F2	.042	.002	-.209	-.004	.003
F2 <--> F3	.046	.002	-.192	-.005	.003
F1 <--> F3	.086	.004	.812	.003	.006
F2 <--> F4	.069	.003	.355	.002	.005
F3 <--> F4	.086	.004	-.746	-.009	.006
F1 <--> F4	.073	.004	-.609	-.005	.005
F2 <--> F5	.042	.002	-.189	-.002	.003
F4 <--> F5	.073	.004	-.678	.001	.005
F3 <--> F5	.096	.005	.864	-.002	.007
F1 <--> F5	.103	.005	.941	.001	.007
F6 <--> F5	.092	.005	-.901	.001	.007
F6 <--> F3	.090	.005	-.683	-.006	.006
F6 <--> F4	.102	.005	1.213	-.005	.007
F6 <--> F2	.073	.004	.373	-.001	.005
F6 <--> F1	.086	.004	-.641	-.009	.006

Correlations: (g2 - Measurement weights)

Parameter	SE	SE-SE	Mean	Bias	SE-Bias
F1 <--> F2	.053	.003	-.574	-.007	.004
F2 <--> F3	.060	.003	-.476	-.006	.004
F1 <--> F3	.040	.002	.866	-.003	.003
F2 <--> F4	.032	.002	.829	-.002	.002
F3 <--> F4	.046	.002	-.677	-.005	.003
F1 <--> F4	.052	.003	-.611	-.003	.004
F2 <--> F5	.059	.003	-.466	-.004	.004
F4 <--> F5	.049	.002	-.608	.000	.003
F3 <--> F5	.039	.002	.825	-.004	.003
F1 <--> F5	.035	.002	.991	-.001	.002
F6 <--> F5	.046	.002	-.693	-.002	.003
F6 <--> F3	.056	.003	-.531	-.004	.004
F6 <--> F4	.035	.002	.885	-.002	.002
F6 <--> F2	.047	.002	.747	-.005	.003
F6 <--> F1	.059	.003	-.550	-.008	.004

Variances: (g2 - Measurement weights)

Parameter	SE	SE-SE	Mean	Bias	SE-Bias
**F1**	.104	.005	.852	.007	.007
**F2**	.053	.003	.160	.006	.004
**F3**	.117	.006	1.036	.008	.008
**F4**	.110	.005	1.175	.003	.008
**F5**	.122	.006	1.062	-.001	.009
**F6**	.153	.008	1.604	-.003	.011
**e1**	.186	.009	1.536	-.004	.013
**e2**	.095	.005	.813	-.007	.007
**e3**	.157	.008	1.414	-.006	.011
**e4**	.114	.006	1.415	-.012	.008
**e5**	.132	.007	2.109	.009	.009
**e6**	.168	.008	1.918	.000	.012
**e7**	.182	.009	1.331	-.017	.013
**e8**	.152	.008	1.481	.008	.011
**e9**	.129	.006	1.088	-.006	.009
**e10**	.103	.005	.838	-.007	.007
**e11**	.072	.004	.626	-.009	.005

Parameter	SE	SE-SE	Mean	Bias	SE-Bias
**e12**	.138	.007	1.234	.004	.010
**e13**	.123	.006	1.367	.007	.009
**e14**	.175	.009	1.166	-.018	.012
**e15**	.190	.009	1.541	.012	.013
**e16**	.180	.009	1.273	-.019	.013
**e17**	.114	.006	1.073	.001	.008
**e18**	.134	.007	.816	-.008	.009
**e19**	.169	.008	1.083	-.011	.012
**e20**	.199	.010	1.374	-.016	.014
**e21**	.150	.008	1.048	-.004	.011
**e22**	.177	.009	1.191	-.017	.013
**e23**	.192	.010	1.582	.001	.014
**e24**	.197	.010	1.836	.005	.014

Matrices (g2 - Measurement weights)

Sample Covariances - Standard Errors (g2 - Measurement weights)

	BPNSF6	BPNSF11	BPNSF17	BPNSF23	BPNSF3	BPNSF9	BPNSF14	BPNSF21	BPNSF2	BPNSF8	BPNSF20	BPNSF22	BPNSF4	BPNSF12	BPNSF16	BPNS[illegible]
**BPNSF6**	.181															
**BPNSF11**	.156	.172														
**BPNSF17**	.159	.159	.203													
**BPNSF23**	.150	.147	.164	.192												
**BPNSF3**	.134	.128	.124	.133	.183											
**BPNSF9**	.125	.137	.149	.135	.133	.194										
**BPNSF14**	.115	.139	.136	.123	.134	.156	.159									
**BPNSF21**	.120	.132	.136	.132	.123	.132	.139	.158								
**BPNSF2**	.143	.130	.136	.149	.107	.111	.112	.104	.169							
**BPNSF8**	.159	.151	.162	.146	.128	.128	.131	.134	.137	.169						
**BPNSF20**	.161	.141	.153	.171	.141	.137	.144	.144	.140	.157	.199					
**BPNSF22**	.147	.136	.136	.139	.120	.128	.133	.137	.125	.148	.139	.144				
**BPNSF4**	.132	.127	.120	.139	.136	.121	.123	.120	.111	.126	.147	.131	.143			
**BPNSF12**	.129	.132	.136	.135	.137	.136	.141	.133	.116	.129	.137	.127	.121	.140		
**BPNSF16**	.122	.126	.128	.133	.138	.132	.121	.138	.118	.136	.158	.134	.127	.133	.162	
**BPNSF24**	.122	.124	.135	.129	.132	.130	.124	.126	.129	.138	.142	.133	.128	.135	.134	.1[illegible]
**BPNSF5**	.137	.135	.143	.150	.135	.119	.110	.132	.111	.147	.163	.151	.139	.133	.122	.1[illegible]
**BPNSF10**	.145	.141	.146	.148	.138	.130	.122	.133	.128	.144	.152	.149	.140	.133	.135	.1[illegible]
**BPNSF15**	.136	.144	.159	.146	.136	.120	.121	.135	.136	.159	.153	.151	.138	.137	.134	.1[illegible]
**BPNSF18**	.138	.141	.140	.137	.119	.113	.103	.121	.128	.142	.135	.142	.133	.138	.122	.1[illegible]
**BPNSF1**	.104	.114	.101	.117	.106	.111	.109	.109	.113	.142	.142	.136	.118	.110	.105	.1[illegible]
**BPNSF7**	.129	.130	.138	.116	.134	.137	.123	.119	.102	.141	.119	.125	.115	.129	.121	.1[illegible]
**BPNSF13**	.119	.125	.124	.119	.112	.124	.131	.119	.104	.119	.124	.119	.106	.116	.115	.1[illegible]
**BPNSF19**	.130	.138	.134	.136	.122	.152	.134	.135	.103	.132	.141	.127	.125	.132	.120	.1[illegible]

Sample Correlations - Standard Errors (g2 - Measurement weights)

	BPNSF6	BPNSF11	BPNSF17	BPNSF23	BPNSF3	BPNSF9	BPNSF14	BPNSF21	BPNSF2	BPNSF8	BPNSF20	BPNSF22	BPNSF4	BPNSF12	BPNSF16	BPNS[illegible]
**BPNSF6**	.000															
**BPNSF11**	.049	.000														
**BPNSF17**	.052	.047	.000													
**BPNSF23**	.046	.044	.045	.000												
**BPNSF3**	.053	.052	.050	.053	.000											
**BPNSF9**	.051	.047	.052	.049	.058	.000										
**BPNSF14**	.047	.054	.047	.050	.059	.052	.000									
**BPNSF21**	.045	.049	.052	.046	.046	.050	.052	.000								
**BPNSF2**	.048	.047	.046	.048	.046	.047	.047	.041	.000							
**BPNSF8**	.049	.050	.046	.045	.049	.047	.051	.049	.043	.000						
**BPNSF20**	.052	.045	.045	.043	.052	.051	.054	.048	.047	.045	.000					
**BPNSF22**	.050	.047	.045	.043	.050	.049	.054	.051	.042	.045	.038	.000				
**BPNSF4**	.050	.051	.045	.051	.036	.048	.049	.039	.045	.048	.050	.051	.000			
**BPNSF12**	.052	.053	.051	.050	.058	.053	.049	.042	.048	.050	.050	.045	.047	.000		
**BPNSF16**	.049	.048	.049	.052	.062	.056	.044	.046	.046	.049	.052	.046	.051	.036	.000	
**BPNSF24**	.050	.049	.050	.050	.060	.055	.047	.047	.047	.048	.049	.043	.051	.045	.042	.0[illegible]
**BPNSF5**	.046	.050	.051	.051	.054	.050	.048	.054	.040	.048	.053	.052	.052	.059	.052	.0[illegible]
**BPNSF10**	.048	.043	.043	.042	.053	.049	.047	.045	.046	.044	.045	.047	.053	.053	.051	.0[illegible]
**BPNSF15**	.044	.045	.052	.047	.052	.046	.049	.051	.047	.049	.047	.047	.052	.054	.050	.0[illegible]
**BPNSF18**	.053	.050	.051	.053	.051	.049	.048	.052	.051	.052	.048	.054	.059	.063	.053	.0[illegible]
**BPNSF1**	.043	.048	.042	.049	.047	.051	.047	.045	.047	.055	.055	.055	.051	.051	.048	.0[illegible]
**BPNSF7**	.052	.054	.052	.050	.059	.051	.051	.050	.046	.054	.049	.052	.050	.055	.054	.0[illegible]
**BPNSF13**	.050	.050	.050	.049	.050	.052	.047	.038	.045	.046	.047	.046	.043	.035	.046	.0[illegible]
**BPNSF19**	.050	.052	.049	.051	.051	.056	.054	.047	.043	.048	.050	.050	.048	.052	.051	.0[illegible]

Sample Means - Standard Errors (g2 - Measurement weights)

	BPNSF6	BPNSF11	BPNSF17	BPNSF23	BPNSF3	BPNSF9	BPNSF14	BPNSF21	BPNSF2	BPNSF8	BPNSF20	BPNSF22	BPNSF4	BPNSF12	BPNSF16	BPNSF[illegible]
**BPNSF6**	.079	.090	.088	.076	.073	.074	.067	.076	.078	.091	.087	.088	.073	.067	.072	.07[illegible]

Bootstrap Confidence (g2 - Measurement weights)

Percentile method (g2 - Measurement weights)

90% confidence intervals (percentile method)

Scalar Estimates (g2 - Measurement weights)

Regression Weights: (g2 - Measurement weights)

Parameter			Estimate	Lower	Upper	P
BPNSF19	<---	F1	1.000	1.000	1.000	...
BPNSF13	<---	F1	1.146	1.045	1.234	.010
BPNSF7	<---	F1	.872	.767	.971	.010
BPNSF1	<---	F1	.774	.651	.908	.010
BPNSF18	<---	F2	1.000	1.000	1.000	...
BPNSF15	<---	F2	2.522	2.037	3.351	.010
BPNSF10	<---	F2	3.235	2.580	4.410	.010
BPNSF5	<---	F2	2.579	2.034	3.484	.010
BPNSF24	<---	F3	1.000	1.000	1.000	...
BPNSF16	<---	F3	1.125	1.040	1.218	.010
BPNSF12	<---	F3	1.221	1.134	1.299	.010
BPNSF4	<---	F3	.861	.771	.947	.010
BPNSF22	<---	F4	1.000	1.000	1.000	...
BPNSF20	<---	F4	1.188	1.107	1.281	.010
BPNSF8	<---	F4	1.203	1.117	1.281	.010
BPNSF2	<---	F4	1.115	1.029	1.215	.010
BPNSF21	<---	F5	1.000	1.000	1.000	...
BPNSF14	<---	F5	1.090	1.014	1.183	.010
BPNSF9	<---	F5	1.038	.959	1.137	.010
BPNSF3	<---	F5	.889	.797	.997	.010
BPNSF23	<---	F6	1.000	1.000	1.000	...
BPNSF17	<---	F6	.977	.925	1.044	.010
BPNSF11	<---	F6	.972	.915	1.036	.010
BPNSF6	<---	F6	.870	.809	.937	.010

Standardized Regression Weights: (g2 - Measurement weights)

Parameter			Estimate	Lower	Upper	P
BPNSF19	<---	F1	.595	.529	.662	.010
BPNSF13	<---	F1	.758	.710	.806	.010
BPNSF7	<---	F1	.558	.491	.634	.010
BPNSF1	<---	F1	.512	.452	.574	.010
BPNSF18	<---	F2	.261	.191	.336	.010
BPNSF15	<---	F2	.581	.517	.641	.010
BPNSF10	<---	F2	.738	.677	.792	.010
BPNSF5	<---	F2	.640	.572	.707	.010
BPNSF24	<---	F3	.696	.641	.747	.010
BPNSF16	<---	F3	.779	.730	.820	.010
BPNSF12	<---	F3	.841	.806	.871	.010
BPNSF4	<---	F3	.618	.557	.691	.010
BPNSF22	<---	F4	.680	.633	.723	.010
BPNSF20	<---	F4	.763	.714	.816	.010
BPNSF8	<---	F4	.725	.673	.764	.010
BPNSF2	<---	F4	.728	.678	.787	.010
BPNSF21	<---	F5	.706	.653	.757	.010
BPNSF14	<---	F5	.778	.722	.835	.010
BPNSF9	<---	F5	.715	.650	.773	.010
BPNSF3	<---	F5	.614	.541	.688	.010
BPNSF23	<---	F6	.777	.729	.825	.010
BPNSF17	<---	F6	.748	.705	.811	.010
BPNSF11	<---	F6	.700	.653	.750	.010
BPNSF6	<---	F6	.632	.580	.682	.010

Intercepts: (g2 - Measurement weights)

Parameter	Estimate	Lower	Upper	P
**BPNSF19**	5.243	5.111	5.366	.010
**BPNSF13**	5.197	5.086	5.319	.010
**BPNSF7**	4.850	4.741	4.976	.010
**BPNSF1**	4.867	4.773	4.990	.010
**BPNSF18**	4.106	3.979	4.230	.010
**BPNSF15**	3.605	3.416	3.752	.010
**BPNSF10**	3.152	2.976	3.296	.010
**BPNSF5**	3.637	3.513	3.763	.010
**BPNSF24**	5.311	5.192	5.438	.010
**BPNSF16**	5.218	5.104	5.337	.010
**BPNSF12**	5.314	5.201	5.424	.010
**BPNSF4**	5.077	4.965	5.211	.010
**BPNSF22**	3.078	2.913	3.216	.010
**BPNSF20**	2.439	2.279	2.604	.010
**BPNSF8**	2.731	2.584	2.889	.010
**BPNSF2**	2.309	2.160	2.414	.010
**BPNSF21**	5.240	5.118	5.391	.010
**BPNSF14**	5.607	5.518	5.728	.010
**BPNSF9**	5.725	5.608	5.857	.010
**BPNSF3**	5.619	5.508	5.742	.010
**BPNSF23**	2.173	2.042	2.284	.010
**BPNSF17**	2.474	2.325	2.613	.010
**BPNSF11**	2.761	2.604	2.904	.010
**BPNSF6**	2.616	2.464	2.725	.010

Covariances: (g2 - Measurement weights)

Parameter	Estimate	Lower	Upper	P
F1 <--> F2	-.204	-.287	-.143	.010
F2 <--> F3	-.187	-.281	-.122	.010
F1 <--> F3	.810	.685	.950	.010
F2 <--> F4	.353	.243	.466	.010
F3 <--> F4	-.738	-.883	-.612	.010
F1 <--> F4	-.605	-.739	-.497	.010
F2 <--> F5	-.187	-.272	-.123	.010
F4 <--> F5	-.679	-.808	-.564	.010
F3 <--> F5	.866	.719	1.038	.010
F1 <--> F5	.940	.801	1.148	.010
F6 <--> F5	-.903	-1.082	-.765	.010
F6 <--> F3	-.677	-.821	-.528	.010
F6 <--> F4	1.218	1.046	1.383	.010
F6 <--> F2	.374	.264	.505	.010
F6 <--> F1	-.632	-.791	-.509	.010

Correlations: (g2 - Measurement weights)

Parameter	Estimate	Lower	Upper	P
F1 <--> F2	-.567	-.651	-.475	.010
F2 <--> F3	-.470	-.577	-.376	.010
F1 <--> F3	.869	.795	.926	.010
F2 <--> F4	.831	.777	.879	.010
F3 <--> F4	-.672	-.746	-.595	.010
F1 <--> F4	-.608	-.696	-.524	.010
F2 <--> F5	-.462	-.572	-.370	.010
F4 <--> F5	-.609	-.684	-.523	.010
F3 <--> F5	.829	.765	.882	.010
F1 <--> F5	.992	.918	1.042	.010
F6 <--> F5	-.691	-.764	-.616	.010
F6 <--> F3	-.527	-.622	-.434	.010
F6 <--> F4	.888	.826	.945	.010
F6 <--> F2	.752	.673	.828	.010
F6 <--> F1	-.542	-.653	-.452	.010

Variances: (g2 - Measurement weights)

Parameter	Estimate	Lower	Upper	P
**F1**	.845	.700	1.025	.010
**F2**	.154	.082	.252	.010
**F3**	1.028	.842	1.233	.010
**F4**	1.172	1.007	1.364	.010

Parameter	Estimate	Lower	Upper	P
**F5**	1.062	.895	1.284	.010
**F6**	1.606	1.360	1.880	.010
**e1**	1.540	1.218	1.860	.010
**e2**	.821	.630	.981	.010
**e3**	1.421	1.121	1.697	.010
**e4**	1.427	1.231	1.606	.010
**e5**	2.100	1.882	2.342	.010
**e6**	1.918	1.627	2.194	.010
**e7**	1.348	1.053	1.680	.010
**e8**	1.473	1.242	1.737	.010
**e9**	1.094	.844	1.297	.010
**e10**	.845	.660	1.001	.010
**e11**	.635	.519	.742	.010
**e12**	1.230	1.000	1.456	.010
**e13**	1.360	1.162	1.561	.010
**e14**	1.184	.889	1.492	.010
**e15**	1.528	1.271	1.882	.010
**e16**	1.292	.995	1.588	.010
**e17**	1.072	.904	1.263	.010
**e18**	.823	.590	1.055	.010
**e19**	1.094	.857	1.389	.010
**e20**	1.390	1.027	1.716	.010
**e21**	1.052	.815	1.313	.010
**e22**	1.208	.899	1.494	.010
**e23**	1.581	1.279	1.924	.010
**e24**	1.831	1.487	2.181	.010

Matrices (g2 - Measurement weights)

Sample Covariances (g2 - Measurement weights)

Sample Covariances - Lower Bounds (PC) (g2 - Measurement weights)

	BPNSF6	BPNSF11	BPNSF17	BPNSF23	BPNSF3	BPNSF9	BPNSF14	BPNSF21	BPNSF2	BPNSF8	BPNSF20	BPNSF22	BPNSF4	BPNSF12	BPNSF16	BPNS[illegible]
**BPNSF6**	2.618															
**BPNSF11**	.891	2.597														
**BPNSF17**	1.099	1.287	2.541													
**BPNSF23**	1.081	1.203	1.450	2.385												
**BPNSF3**	-1.078	-.866	-1.056	-1.127	1.965											
**BPNSF9**	-1.015	-1.177	-1.431	-1.265	.913	1.897										
**BPNSF14**	-.874	-1.012	-1.348	-1.044	.754	1.010	1.748									
**BPNSF21**	-.915	-1.066	-1.031	-1.106	.857	.778	.899	1.959								
**BPNSF2**	.965	.899	1.151	1.121	-.956	-.940	-1.002	-.931	2.284							
**BPNSF8**	1.011	.971	1.151	1.055	-.895	-.996	-.946	-.985	1.223	2.758						
**BPNSF20**	.801	1.035	1.212	1.446	-1.053	-1.035	-1.137	-1.258	1.290	1.348	2.521					
**BPNSF22**	.752	.931	1.041	1.068	-.676	-1.006	-.936	-1.043	1.090	1.169	1.364	2.498				
**BPNSF4**	-.885	-.814	-.880	-.941	1.173	.673	.637	.916	-1.049	-.999	-1.237	-1.002	1.923			
**BPNSF12**	-.778	-.940	-1.092	-1.000	.654	.739	.819	.888	-1.124	-1.045	-1.155	-1.267	.938	1.827		
**BPNSF16**	-.719	-.926	-.978	-.913	.538	.631	.831	.934	-1.028	-1.033	-1.288	-1.198	.810	1.198	1.895	
**BPNSF24**	-.737	-.847	-1.146	-.926	.429	.575	.735	.777	-1.115	-1.137	-1.285	-1.369	.738	.941	1.007	1.8[illegible]
**BPNSF5**	.684	.456	.496	.656	-.665	-.538	-.550	-.775	.645	.884	.725	.739	-.987	-.715	-.620	-.7[illegible]
**BPNSF10**	.650	1.183	.956	1.030	-.873	-.978	-.940	-1.126	.892	1.084	1.097	1.050	-.987	-.940	-.880	-.9[illegible]
**BPNSF15**	.651	.919	.724	.599	-.635	-.542	-.504	-.671	.609	.824	.881	.943	-.793	-.735	-.697	-.7[illegible]
**BPNSF18**	-.024	.403	.363	.122	-.204	-.336	-.254	-.435	.137	.257	.410	.275	-.392	-.396	-.398	-.4[illegible]
**BPNSF1**	-.470	-.552	-.523	-.597	.643	.475	.604	.740	-.827	-.743	-.863	-.792	.644	.520	.468	.4[illegible]
**BPNSF7**	-.244	-.762	-.915	-.539	.442	.741	.658	.623	-.661	-.827	-.754	-.921	.583	.530	.540	.5[illegible]
**BPNSF13**	-.722	-.995	-.929	-.885	.720	.729	.930	1.031	-.832	-.991	-.971	-1.061	.744	1.025	.841	.7[illegible]
**BPNSF19**	-.851	-1.074	-1.109	-1.157	.574	.754	.806	.933	-.719	-.930	-1.087	-.986	.648	.610	.556	.4[illegible]

Sample Covariances - Upper Bounds (PC) (g2 - Measurement weights)

	BPNSF6	BPNSF11	BPNSF17	BPNSF23	BPNSF3	BPNSF9	BPNSF14	BPNSF21	BPNSF2	BPNSF8	BPNSF20	BPNSF22	BPNSF4	BPNSF12	BPNSF16	BPNS[illegible]
**BPNSF6**	3.251															
**BPNSF11**	1.425	3.169														
**BPNSF17**	1.595	1.810	3.245													
**BPNSF23**	1.584	1.676	2.000	3.053												
**BPNSF3**	-.650	-.448	-.648	-.676	2.566											
**BPNSF9**	-.586	-.744	-.942	-.817	1.345	2.548										
**BPNSF14**	-.493	-.572	-.893	-.643	1.201	1.519	2.302									
**BPNSF21**	-.503	-.620	-.574	-.653	1.279	1.249	1.355	2.472								

	BPNSF6	BPNSF11	BPNSF17	BPNSF23	BPNSF3	BPNSF9	BPNSF14	BPNSF21	BPNSF2	BPNSF8	BPNSF20	BPNSF22	BPNSF4	BPNSF12	BPNSF16	BPNS[illegible]
**BPNSF2**	1.441	1.349	1.577	1.621	-.598	-.572	-.618	-.594	2.898							
**BPNSF8**	1.553	1.500	1.644	1.551	-.483	-.577	-.500	-.546	1.696	3.333						
**BPNSF20**	1.316	1.532	1.729	1.999	-.607	-.574	-.639	-.792	1.753	1.857	3.246					
**BPNSF22**	1.237	1.372	1.534	1.533	-.291	-.563	-.475	-.601	1.499	1.645	1.824	2.983				
**BPNSF4**	-.437	-.371	-.489	-.480	1.640	1.085	1.067	1.304	-.653	-.554	-.730	-.547	2.385			
**BPNSF12**	-.340	-.507	-.655	-.582	1.082	1.200	1.305	1.332	-.734	-.612	-.695	-.844	1.341	2.296		
**BPNSF16**	-.306	-.496	-.583	-.467	.968	1.086	1.222	1.390	-.639	-.581	-.747	-.758	1.232	1.657	2.452	
**BPNSF24**	-.327	-.452	-.719	-.506	.844	.994	1.163	1.190	-.688	-.684	-.821	-.928	1.159	1.393	1.469	[illegible]
**BPNSF5**	1.136	.911	.966	1.162	-.245	-.151	-.178	-.314	1.011	1.377	1.261	1.229	-.509	-.283	-.202	[illegible]
**BPNSF10**	1.137	1.621	1.428	1.500	-.390	-.556	-.553	-.667	1.337	1.589	1.645	1.547	-.490	-.512	-.434	[illegible]
**BPNSF15**	1.106	1.386	1.251	1.116	-.142	-.128	-.100	-.216	1.052	1.355	1.367	1.438	-.316	-.252	-.244	[illegible]
**BPNSF18**	.438	.903	.819	.590	.215	.062	.090	-.035	.554	.739	.862	.751	.053	.064	.006	[illegible]
**BPNSF1**	-.131	-.178	-.188	-.209	1.001	.837	.963	1.074	-.462	-.269	-.387	-.324	1.052	.889	.831	[illegible]
**BPNSF7**	.184	-.300	-.440	-.154	.911	1.185	1.051	1.006	-.318	-.376	-.329	-.487	.966	.980	.950	[illegible]
**BPNSF13**	-.356	-.563	-.515	-.489	1.090	1.143	1.358	1.452	-.481	-.584	-.595	-.644	1.103	1.400	1.247	[illegible]
**BPNSF19**	-.412	-.587	-.661	-.677	.975	1.255	1.271	1.407	-.380	-.494	-.630	-.586	1.049	1.075	.966	[illegible]

Sample Covariances - Two Tailed Significance (PC) (g2 - Measurement weights)

	BPNSF6	BPNSF11	BPNSF17	BPNSF23	BPNSF3	BPNSF9	BPNSF14	BPNSF21	BPNSF2	BPNSF8	BPNSF20	BPNSF22	BPNSF4	BPNSF12	BPNSF16	BPNS[illegible]
**BPNSF6**	.010															
**BPNSF11**	.010	.010														
**BPNSF17**	.010	.010	.010													
**BPNSF23**	.010	.010	.010	.010												
**BPNSF3**	.010	.010	.010	.010	.010											
**BPNSF9**	.010	.010	.010	.010	.010	.010										
**BPNSF14**	.010	.010	.010	.010	.010	.010	.010									
**BPNSF21**	.010	.010	.010	.010	.010	.010	.010	.010								
**BPNSF2**	.010	.010	.010	.010	.010	.010	.010	.010	.010							
**BPNSF8**	.010	.010	.010	.010	.010	.010	.010	.010	.010	.010						
**BPNSF20**	.010	.010	.010	.010	.010	.010	.010	.010	.010	.010	.010					
**BPNSF22**	.010	.010	.010	.010	.010	.010	.010	.010	.010	.010	.010	.010				
**BPNSF4**	.010	.010	.010	.010	.010	.010	.010	.010	.010	.010	.010	.010	.010			
**BPNSF12**	.010	.010	.010	.010	.010	.010	.010	.010	.010	.010	.010	.010	.010	.010		
**BPNSF16**	.010	.010	.010	.010	.010	.010	.010	.010	.010	.010	.010	.010	.010	.010	.010	
**BPNSF24**	.010	.010	.010	.010	.010	.010	.010	.010	.010	.010	.010	.010	.010	.010	.010	[illegible]
**BPNSF5**	.010	.010	.010	.010	.010	.021	.010	.010	.010	.010	.010	.010	.010	.010	.013	[illegible]
**BPNSF10**	.010	.010	.010	.010	.010	.010	.010	.010	.010	.010	.010	.010	.010	.010	.010	[illegible]
**BPNSF15**	.010	.010	.010	.010	.016	.010	.023	.010	.010	.010	.010	.010	.010	.010	.010	[illegible]
**BPNSF18**	.131	.010	.010	.016	.946	.259	.576	.040	.010	.010	.010	.010	.162	.274	.108	[illegible]
**BPNSF1**	.010	.010	.010	.010	.010	.010	.010	.010	.010	.010	.010	.010	.010	.010	.010	[illegible]
**BPNSF7**	.692	.010	.010	.014	.010	.010	.010	.010	.010	.010	.010	.010	.010	.010	.010	[illegible]
**BPNSF13**	.010	.010	.010	.010	.010	.010	.010	.010	.010	.010	.010	.010	.010	.010	.010	[illegible]
**BPNSF19**	.010	.010	.010	.010	.010	.010	.010	.010	.010	.010	.010	.010	.010	.010	.010	[illegible]

Sample Correlations (g2 - Measurement weights)

Sample Correlations - Lower Bounds (PC) (g2 - Measurement weights)

	BPNSF6	BPNSF11	BPNSF17	BPNSF23	BPNSF3	BPNSF9	BPNSF14	BPNSF21	BPNSF2	BPNSF8	BPNSF20	BPNSF22	BPNSF4	BPNSF12	BPNSF16	BPNS[illegible]
**BPNSF6**	1.000															
**BPNSF11**	.309	1.000														
**BPNSF17**	.379	.452	1.000													
**BPNSF23**	.390	.438	.543	1.000												
**BPNSF3**	-.420	-.355	-.418	-.454	1.000											
**BPNSF9**	-.397	-.453	-.547	-.507	.412	1.000										
**BPNSF14**	-.359	-.418	-.538	-.449	.363	.496	1.000									
**BPNSF21**	-.355	-.407	-.399	-.436	.387	.361	.447	1.000								
**BPNSF2**	.355	.334	.414	.432	-.402	-.399	-.437	-.386	1.000							
**BPNSF8**	.346	.325	.405	.376	-.355	-.389	-.368	-.371	.454	1.000						
**BPNSF20**	.278	.367	.425	.539	-.408	-.406	-.452	-.473	.474	.466	1.000					
**BPNSF22**	.262	.323	.377	.399	-.271	-.388	-.386	-.417	.421	.418	.501	1.000				
**BPNSF4**	-.344	-.336	-.347	-.374	.566	.323	.319	.437	-.430	-.379	-.467	-.398	1.000			
**BPNSF12**	-.325	-.383	-.434	-.413	.306	.359	.435	.438	-.472	-.416	-.459	-.511	.458	1.000		
**BPNSF16**	-.285	-.360	-.390	-.377	.247	.301	.416	.457	-.421	-.397	-.492	-.474	.385	.620	1.000	
**BPNSF24**	-.296	-.348	-.443	-.382	.191	.265	.389	.359	-.453	-.430	-.507	-.538	.354	.482	.508	[illegible]
**BPNSF5**	.266	.169	.187	.266	-.281	-.222	-.243	-.324	.266	.335	.288	.291	-.416	-.320	-.261	[illegible]
**BPNSF10**	.227	.415	.351	.380	-.341	-.386	-.389	-.426	.333	.382	.397	.378	-.380	-.385	-.348	[illegible]
**BPNSF15**	.218	.311	.250	.211	-.241	-.205	-.206	-.252	.220	.278	.295	.329	-.304	-.296	-.264	[illegible]
**BPNSF18**	-.009	.160	.135	.050	-.085	-.145	-.119	-.191	.057	.093	.161	.109	-.173	-.182	-.174	[illegible]
**BPNSF1**	-.193	-.234	-.220	-.255	.307	.226	.310	.351	-.361	-.300	-.356	-.335	.313	.261	.231	[illegible]

	BPNSF6	BPNSF11	BPNSF17	BPNSF23	BPNSF3	BPNSF9	BPNSF14	BPNSF21	BPNSF2	BPNSF8	BPNSF20	BPNSF22	BPNSF4	BPNSF12	BPNSF16	BPNS[illegible]
**BPNSF7**	-.099	-.309	-.363	-.228	.207	.363	.329	.296	-.289	-.333	-.297	-.376	.277	.263	.257	.2[illegible]
**BPNSF13**	-.314	-.421	-.391	-.386	.352	.362	.500	.528	-.368	-.398	-.411	-.444	.379	.542	.432	.4[illegible]
**BPNSF19**	-.323	-.417	-.431	-.444	.252	.341	.387	.434	-.296	-.339	-.414	-.385	.294	.294	.258	.1[illegible]

Sample Correlations - Upper Bounds (PC) (g2 - Measurement weights)

	BPNSF6	BPNSF11	BPNSF17	BPNSF23	BPNSF3	BPNSF9	BPNSF14	BPNSF21	BPNSF2	BPNSF8	BPNSF20	BPNSF22	BPNSF4	BPNSF12	BPNSF16	BPNS[illegible]
**BPNSF6**	1.000															
**BPNSF11**	.480	1.000														
**BPNSF17**	.548	.610	1.000													
**BPNSF23**	.540	.589	.697	1.000												
**BPNSF3**	-.248	-.178	-.248	-.272	1.000											
**BPNSF9**	-.230	-.291	-.372	-.348	.605	1.000										
**BPNSF14**	-.205	-.239	-.388	-.283	.548	.674	1.000									
**BPNSF21**	-.196	-.252	-.221	-.267	.543	.532	.618	1.000								
**BPNSF2**	.510	.489	.572	.589	-.245	-.238	-.271	-.242	1.000							
**BPNSF8**	.509	.499	.546	.525	-.189	-.234	-.202	-.208	.595	1.000						
**BPNSF20**	.447	.525	.586	.681	-.241	-.237	-.281	-.317	.629	.605	1.000					
**BPNSF22**	.432	.482	.529	.547	-.113	-.227	-.210	-.240	.559	.554	.625	1.000				
**BPNSF4**	-.170	-.153	-.198	-.205	.693	.481	.473	.558	-.285	-.224	-.300	-.228	1.000			
**BPNSF12**	-.143	-.213	-.276	-.254	.501	.546	.590	.578	-.314	-.251	-.287	-.364	.620	1.000		
**BPNSF16**	-.118	-.204	-.239	-.188	.441	.490	.569	.606	-.273	-.227	-.308	-.322	.564	.732	1.000	
**BPNSF24**	-.124	-.178	-.281	-.214	.376	.437	.541	.513	-.298	-.271	-.347	-.397	.521	.633	.646	1.0[illegible]
**BPNSF5**	.423	.335	.357	.431	-.112	-.065	-.084	-.134	.399	.497	.469	.463	-.235	-.128	-.090	-.1[illegible]
**BPNSF10**	.383	.556	.481	.515	-.151	-.224	-.233	-.277	.481	.527	.552	.537	-.200	-.214	-.178	-.2[illegible]
**BPNSF15**	.361	.467	.421	.379	-.054	-.047	-.038	-.085	.376	.439	.454	.484	-.130	-.100	-.099	-.1[illegible]
**BPNSF18**	.163	.336	.313	.230	.091	.028	.043	-.015	.220	.272	.324	.289	.025	.029	.003	-.0[illegible]
**BPNSF1**	-.052	-.074	-.077	-.090	.464	.409	.463	.495	-.205	-.111	-.164	-.146	.495	.441	.387	.4[illegible]
**BPNSF7**	.071	-.125	-.195	-.066	.413	.537	.499	.467	-.135	-.151	-.134	-.208	.443	.455	.438	.4[illegible]
**BPNSF13**	-.149	-.243	-.220	-.219	.525	.537	.652	.657	-.216	-.246	-.257	-.288	.525	.661	.585	.5[illegible]
**BPNSF19**	-.156	-.226	-.260	-.269	.421	.532	.572	.582	-.158	-.185	-.248	-.227	.451	.466	.420	.3[illegible]

Sample Correlations - Two Tailed Significance (PC) (g2 - Measurement weights)

	BPNSF6	BPNSF11	BPNSF17	BPNSF23	BPNSF3	BPNSF9	BPNSF14	BPNSF21	BPNSF2	BPNSF8	BPNSF20	BPNSF22	BPNSF4	BPNSF12	BPNSF16	BPNS[illegible]
**BPNSF6**	...															
**BPNSF11**	.010	...														
**BPNSF17**	.010	.010	...													
**BPNSF23**	.010	.010	.010	...												
**BPNSF3**	.010	.010	.010	.010	...											
**BPNSF9**	.010	.010	.010	.010	.010	...										
**BPNSF14**	.010	.010	.010	.010	.010	.010	...									
**BPNSF21**	.010	.010	.010	.010	.010	.010	.010	...								
**BPNSF2**	.010	.010	.010	.010	.010	.010	.010	.010	...							
**BPNSF8**	.010	.010	.010	.010	.010	.010	.010	.010	.010	...						
**BPNSF20**	.010	.010	.010	.010	.010	.010	.010	.010	.010	.010	...					
**BPNSF22**	.010	.010	.010	.010	.010	.010	.010	.010	.010	.010	.010	...				
**BPNSF4**	.010	.010	.010	.010	.010	.010	.010	.010	.010	.010	.010	.010	...			
**BPNSF12**	.010	.010	.010	.010	.010	.010	.010	.010	.010	.010	.010	.010	.010	...		
**BPNSF16**	.010	.010	.010	.010	.010	.010	.010	.010	.010	.010	.010	.010	.010	.010	...	
**BPNSF24**	.010	.010	.010	.010	.010	.010	.010	.010	.010	.010	.010	.010	.010	.010	.010	
**BPNSF5**	.010	.010	.010	.010	.010	.021	.010	.010	.010	.010	.010	.010	.010	.010	.013	.0[illegible]
**BPNSF10**	.010	.010	.010	.010	.010	.010	.010	.010	.010	.010	.010	.010	.010	.010	.010	.0[illegible]
**BPNSF15**	.010	.010	.010	.010	.015	.010	.023	.010	.010	.010	.010	.010	.010	.010	.010	.0[illegible]
**BPNSF18**	.131	.010	.010	.016	.946	.259	.576	.040	.010	.010	.010	.010	.162	.274	.108	.0[illegible]
**BPNSF1**	.010	.010	.010	.010	.010	.010	.010	.010	.010	.010	.010	.010	.010	.010	.010	.0[illegible]
**BPNSF7**	.692	.010	.010	.014	.010	.010	.010	.010	.010	.010	.010	.010	.010	.010	.010	.0[illegible]
**BPNSF13**	.010	.010	.010	.010	.010	.010	.010	.010	.010	.010	.010	.010	.010	.010	.010	.0[illegible]
**BPNSF19**	.010	.010	.010	.010	.010	.010	.010	.010	.010	.010	.010	.010	.010	.010	.010	.0[illegible]

Sample Means (g2 - Measurement weights)

Sample Means - Lower Bounds (PC) (g2 - Measurement weights)

	BPNSF6	BPNSF11	BPNSF17	BPNSF23	BPNSF3	BPNSF9	BPNSF14	BPNSF21	BPNSF2	BPNSF8	BPNSF20	BPNSF22	BPNSF4	BPNSF12	BPNSF16	BPNSF[illegible]
**BPNSF6**	2.464	2.604	2.325	2.042	5.508	5.608	5.518	5.118	2.160	2.584	2.279	2.913	4.965	5.201	5.104	5.19[illegible]

Sample Means - Upper Bounds (PC) (g2 - Measurement weights)

	BPNSF6	BPNSF11	BPNSF17	BPNSF23	BPNSF3	BPNSF9	BPNSF14	BPNSF21	BPNSF2	BPNSF8	BPNSF20	BPNSF22	BPNSF4	BPNSF12	BPNSF16	BPNSF[illegible]

	BPNSF6	BPNSF11	BPNSF17	BPNSF23	BPNSF3	BPNSF9	BPNSF14	BPNSF21	BPNSF2	BPNSF8	BPNSF20	BPNSF22	BPNSF4	BPNSF12	BPNSF16	BPNSF[illegible]
**BPNSF6**	2.725	2.904	2.613	2.284	5.742	5.857	5.728	5.391	2.414	2.889	2.604	3.216	5.211	5.424	5.337	5.43[illegible]

Sample Means - Two Tailed Significance (PC) (g2 - Measurement weights)

	BPNSF6	BPNSF11	BPNSF17	BPNSF23	BPNSF3	BPNSF9	BPNSF14	BPNSF21	BPNSF2	BPNSF8	BPNSF20	BPNSF22	BPNSF4	BPNSF12	BPNSF16	BPNSF[illegible]
**BPNSF6**	.010	.010	.010	.010	.010	.010	.010	.010	.010	.010	.010	.010	.010	.010	.010	.01[illegible]

Bias-corrected percentile method (g2 - Measurement weights)

90% confidence intervals (bias-corrected percentile method)

Scalar Estimates (g2 - Measurement weights)

Regression Weights: (g2 - Measurement weights)

Parameter			Estimate	Lower	Upper	P
BPNSF19	<---	F1	1.000	1.000	1.000	...
BPNSF13	<---	F1	1.146	1.055	1.257	.005
BPNSF7	<---	F1	.872	.767	.971	.010
BPNSF1	<---	F1	.774	.649	.884	.014
BPNSF18	<---	F2	1.000	1.000	1.000	...
BPNSF15	<---	F2	2.522	2.055	3.469	.007
BPNSF10	<---	F2	3.235	2.585	4.436	.009
BPNSF5	<---	F2	2.579	2.065	3.564	.008
BPNSF24	<---	F3	1.000	1.000	1.000	...
BPNSF16	<---	F3	1.125	1.037	1.216	.011
BPNSF12	<---	F3	1.221	1.145	1.303	.006
BPNSF4	<---	F3	.861	.766	.946	.011
BPNSF22	<---	F4	1.000	1.000	1.000	...
BPNSF20	<---	F4	1.188	1.115	1.297	.004
BPNSF8	<---	F4	1.203	1.127	1.302	.005
BPNSF2	<---	F4	1.115	1.043	1.226	.004
BPNSF21	<---	F5	1.000	1.000	1.000	...
BPNSF14	<---	F5	1.090	1.028	1.194	.006
BPNSF9	<---	F5	1.038	.967	1.139	.007
BPNSF3	<---	F5	.889	.795	.992	.013
BPNSF23	<---	F6	1.000	1.000	1.000	...
BPNSF17	<---	F6	.977	.910	1.028	.023
BPNSF11	<---	F6	.972	.916	1.036	.009
BPNSF6	<---	F6	.870	.811	.938	.009

Standardized Regression Weights: (g2 - Measurement weights)

Parameter			Estimate	Lower	Upper	P
BPNSF19	<---	F1	.595	.528	.662	.012
BPNSF13	<---	F1	.758	.705	.800	.014
BPNSF7	<---	F1	.558	.507	.647	.004
BPNSF1	<---	F1	.512	.440	.567	.020
BPNSF18	<---	F2	.261	.190	.334	.011
BPNSF15	<---	F2	.581	.521	.641	.009
BPNSF10	<---	F2	.738	.665	.788	.019
BPNSF5	<---	F2	.640	.570	.699	.014
BPNSF24	<---	F3	.696	.630	.736	.025
BPNSF16	<---	F3	.779	.727	.816	.018
BPNSF12	<---	F3	.841	.800	.866	.021
BPNSF4	<---	F3	.618	.564	.696	.007
BPNSF22	<---	F4	.680	.637	.732	.006
BPNSF20	<---	F4	.763	.709	.810	.021
BPNSF8	<---	F4	.725	.676	.766	.008
BPNSF2	<---	F4	.728	.675	.782	.014
BPNSF21	<---	F5	.706	.657	.762	.007
BPNSF14	<---	F5	.778	.707	.833	.015
BPNSF9	<---	F5	.715	.641	.767	.021
BPNSF3	<---	F5	.614	.535	.675	.019
BPNSF23	<---	F6	.777	.729	.826	.009
BPNSF17	<---	F6	.748	.692	.797	.018
BPNSF11	<---	F6	.700	.654	.752	.009
BPNSF6	<---	F6	.632	.588	.683	.007

Intercepts: (g2 - Measurement weights)

Parameter	Estimate	Lower	Upper	P
**BPNSF19**	5.243	5.097	5.359	.018
**BPNSF13**	5.197	5.048	5.289	.036
**BPNSF7**	4.850	4.674	4.940	.044
**BPNSF1**	4.867	4.714	4.963	.032
**BPNSF18**	4.106	3.992	4.258	.005
**BPNSF15**	3.605	3.459	3.774	.005
**BPNSF10**	3.152	3.016	3.343	.004
**BPNSF5**	3.637	3.491	3.747	.018
**BPNSF24**	5.311	5.174	5.425	.018
**BPNSF16**	5.218	5.101	5.335	.011
**BPNSF12**	5.314	5.201	5.422	.012
**BPNSF4**	5.077	4.964	5.195	.013
**BPNSF22**	3.078	2.940	3.225	.006
**BPNSF20**	2.439	2.319	2.620	.003
**BPNSF8**	2.731	2.604	2.908	.005
**BPNSF2**	2.309	2.184	2.437	.005
**BPNSF21**	5.240	5.096	5.362	.025
**BPNSF14**	5.607	5.463	5.693	.044
**BPNSF9**	5.725	5.608	5.857	.010
**BPNSF3**	5.619	5.494	5.736	.015
**BPNSF23**	2.173	2.061	2.334	.004
**BPNSF17**	2.474	2.333	2.622	.007
**BPNSF11**	2.761	2.619	2.929	.005
**BPNSF6**	2.616	2.451	2.720	.014

Covariances: (g2 - Measurement weights)

Parameter	Estimate	Lower	Upper	P
F1 <--> F2	-.204	-.285	-.142	.012
F2 <--> F3	-.187	-.271	-.121	.015
F1 <--> F3	.810	.685	.953	.009
F2 <--> F4	.353	.243	.466	.010
F3 <--> F4	-.738	-.896	-.616	.009
F1 <--> F4	-.605	-.736	-.497	.011
F2 <--> F5	-.187	-.271	-.123	.011
F4 <--> F5	-.679	-.823	-.583	.005
F3 <--> F5	.866	.726	1.049	.007
F1 <--> F5	.940	.813	1.169	.004
F6 <--> F5	-.903	-1.104	-.770	.006
F6 <--> F3	-.677	-.805	-.498	.020
F6 <--> F4	1.218	1.063	1.394	.007
F6 <--> F2	.374	.272	.517	.005
F6 <--> F1	-.632	-.815	-.519	.006

Correlations: (g2 - Measurement weights)

Parameter	Estimate	Lower	Upper	P
F1 <--> F2	-.567	-.636	-.456	.032
F2 <--> F3	-.470	-.566	-.353	.019
F1 <--> F3	.869	.789	.922	.012
F2 <--> F4	.831	.778	.880	.009
F3 <--> F4	-.672	-.738	-.582	.020
F1 <--> F4	-.608	-.696	-.524	.010
F2 <--> F5	-.462	-.554	-.350	.020
F4 <--> F5	-.609	-.681	-.519	.014
F3 <--> F5	.829	.765	.882	.010
F1 <--> F5	.992	.911	1.036	.016
F6 <--> F5	-.691	-.767	-.616	.009
F6 <--> F3	-.527	-.601	-.401	.028
F6 <--> F4	.888	.837	.960	.003
F6 <--> F2	.752	.680	.835	.004
F6 <--> F1	-.542	-.635	-.431	.020

Variances: (g2 - Measurement weights)

Parameter	Estimate	Lower	Upper	P
**F1**	.845	.709	1.047	.008
**F2**	.154	.080	.247	.012

Parameter	Estimate	Lower	Upper	P
**F3**	1.028	.837	1.221	.012
**F4**	1.172	1.018	1.382	.006
**F5**	1.062	.915	1.332	.005
**F6**	1.606	1.361	1.880	.009
**e1**	1.540	1.234	1.899	.007
**e2**	.821	.692	1.009	.004
**e3**	1.421	1.121	1.697	.010
**e4**	1.427	1.261	1.646	.004
**e5**	2.100	1.862	2.323	.015
**e6**	1.918	1.627	2.194	.010
**e7**	1.348	1.119	1.733	.003
**e8**	1.473	1.272	1.801	.005
**e9**	1.094	.872	1.324	.007
**e10**	.845	.679	1.012	.007
**e11**	.635	.531	.757	.005
**e12**	1.230	.999	1.451	.011
**e13**	1.360	1.135	1.533	.018
**e14**	1.184	.909	1.507	.005
**e15**	1.528	1.299	1.912	.005
**e16**	1.292	1.068	1.675	.004
**e17**	1.072	.923	1.273	.005
**e18**	.823	.634	1.064	.005
**e19**	1.094	.868	1.410	.006
**e20**	1.390	1.110	1.731	.004
**e21**	1.052	.838	1.341	.005
**e22**	1.208	.928	1.590	.005
**e23**	1.581	1.279	1.924	.010
**e24**	1.831	1.544	2.222	.009

Matrices (g2 - Measurement weights)

Sample Covariances (g2 - Measurement weights)

Sample Covariances - Lower Bounds (BC) (g2 - Measurement weights)

	BPNSF6	BPNSF11	BPNSF17	BPNSF23	BPNSF3	BPNSF9	BPNSF14	BPNSF21	BPNSF2	BPNSF8	BPNSF20	BPNSF22	BPNSF4	BPNSF12	BPNSF16	BPNS[illegible]
**BPNSF6**	2.589															
**BPNSF11**	.958	2.587														
**BPNSF17**	1.116	1.287	2.611													
**BPNSF23**	1.112	1.226	1.490	2.372												
**BPNSF3**	-1.069	-.875	-1.049	-1.127	1.965											
**BPNSF9**	-1.025	-1.195	-1.458	-1.308	.907	1.905										
**BPNSF14**	-.865	-1.036	-1.409	-1.084	.760	1.027	1.795									
**BPNSF21**	-.909	-1.102	-1.058	-1.099	.814	.807	.909	1.924								
**BPNSF2**	.974	.895	1.184	1.129	-.959	-.921	-1.067	-.935	2.371							
**BPNSF8**	1.012	.971	1.158	1.115	-.895	-.996	-.951	-.985	1.224	2.806						
**BPNSF20**	.812	1.076	1.258	1.500	-1.042	-1.030	-1.200	-1.266	1.312	1.403	2.598					
**BPNSF22**	.770	.968	1.127	1.068	-.675	-1.012	-.944	-1.043	1.093	1.181	1.364	2.481				
**BPNSF4**	-.851	-.827	-.881	-.941	1.168	.686	.644	.923	-1.038	-.970	-1.215	-.961	1.913			
**BPNSF12**	-.763	-.915	-1.092	-.995	.654	.755	.873	.916	-1.093	-1.056	-1.155	-1.220	.941	1.846		
**BPNSF16**	-.698	-.913	-.976	-.913	.550	.635	.834	.932	-1.028	-1.045	-1.253	-1.167	.820	1.198	1.863	
**BPNSF24**	-.701	-.847	-1.171	-.951	.434	.611	.791	.777	-1.149	-1.152	-1.285	-1.375	.733	.990	.999	1.8[illegible]
**BPNSF5**	.681	.463	.496	.640	-.646	-.500	-.543	-.727	.632	.901	.720	.755	-.956	-.715	-.620	-.7[illegible]
**BPNSF10**	.666	1.211	.958	1.080	-.886	-.985	-.964	-1.126	.917	1.120	1.103	1.072	-.955	-.956	-.872	-1.0[illegible]
**BPNSF15**	.657	.935	.758	.671	-.586	-.539	-.497	-.671	.632	.748	.886	.964	-.730	-.705	-.686	-.7[illegible]
**BPNSF18**	-.024	.386	.320	.121	-.192	-.330	-.268	-.469	.132	.257	.408	.277	-.392	-.396	-.424	-.4[illegible]
**BPNSF1**	-.422	-.549	-.520	-.597	.649	.506	.620	.741	-.826	-.743	-.840	-.782	.617	.538	.453	.4[illegible]
**BPNSF7**	-.218	-.773	-.961	-.545	.436	.743	.668	.630	-.652	-.814	-.752	-.936	.620	.569	.578	.5[illegible]
**BPNSF13**	-.711	-.995	-.940	-.866	.720	.753	.943	1.047	-.845	-.970	-.970	-1.056	.755	1.036	.832	.8[illegible]
**BPNSF19**	-.854	-1.087	-1.157	-1.157	.560	.742	.846	.942	-.716	-.930	-1.093	-.984	.649	.634	.555	.4[illegible]

Sample Covariances - Upper Bounds (BC) (g2 - Measurement weights)

	BPNSF6	BPNSF11	BPNSF17	BPNSF23	BPNSF3	BPNSF9	BPNSF14	BPNSF21	BPNSF2	BPNSF8	BPNSF20	BPNSF22	BPNSF4	BPNSF12	BPNSF16	BPNS[illegible]
**BPNSF6**	3.240															
**BPNSF11**	1.497	3.167														
**BPNSF17**	1.619	1.810	3.314													
**BPNSF23**	1.631	1.705	2.028	3.050												
**BPNSF3**	-.633	-.465	-.642	-.676	2.566											
**BPNSF9**	-.593	-.749	-.957	-.841	1.344	2.552										

	BPNSF6	BPNSF11	BPNSF17	BPNSF23	BPNSF3	BPNSF9	BPNSF14	BPNSF21	BPNSF2	BPNSF8	BPNSF20	BPNSF22	BPNSF4	BPNSF12	BPNSF16	BPNS[illegible]
**BPNSF14**	-.486	-.584	-.948	-.690	1.202	1.569	2.368									
**BPNSF21**	-.489	-.648	-.599	-.641	1.239	1.273	1.415	2.454								
**BPNSF2**	1.445	1.343	1.624	1.622	-.604	-.569	-.655	-.596	2.916							
**BPNSF8**	1.562	1.500	1.667	1.597	-.483	-.577	-.503	-.546	1.696	3.371						
**BPNSF20**	1.332	1.554	1.764	2.029	-.579	-.573	-.689	-.797	1.790	1.919	3.293					
**BPNSF22**	1.254	1.406	1.593	1.533	-.286	-.574	-.484	-.599	1.529	1.670	1.824	2.973				
**BPNSF4**	-.391	-.408	-.490	-.480	1.634	1.102	1.096	1.339	-.653	-.547	-.706	-.518	2.384			
**BPNSF12**	-.323	-.505	-.655	-.571	1.082	1.249	1.366	1.363	-.718	-.614	-.695	-.822	1.350	2.354		
**BPNSF16**	-.264	-.489	-.568	-.467	.987	1.094	1.222	1.389	-.639	-.592	-.732	-.737	1.234	1.657	2.418	
**BPNSF24**	-.304	-.450	-.727	-.536	.848	1.042	1.194	1.190	-.708	-.690	-.818	-.936	1.132	1.430	1.460	[illegible]
**BPNSF5**	1.132	.922	.977	1.134	-.213	-.114	-.167	-.292	.998	1.382	1.251	1.246	-.489	-.282	-.202	[illegible]
**BPNSF10**	1.144	1.680	1.429	1.554	-.396	-.564	-.569	-.667	1.355	1.607	1.646	1.596	-.469	-.521	-.397	[illegible]
**BPNSF15**	1.116	1.398	1.272	1.161	-.120	-.127	-.083	-.216	1.071	1.329	1.380	1.449	-.272	-.244	-.238	[illegible]
**BPNSF18**	.438	.893	.784	.588	.225	.072	.062	-.048	.552	.739	.858	.751	.053	.064	-.024	[illegible]
**BPNSF1**	-.043	-.170	-.186	-.209	1.008	.881	1.001	1.075	-.460	-.269	-.365	-.306	1.048	.922	.784	[illegible]
**BPNSF7**	.224	-.304	-.483	-.168	.876	1.186	1.060	1.016	-.309	-.355	-.327	-.517	1.004	.996	1.009	[illegible]
**BPNSF13**	-.334	-.563	-.521	-.466	1.090	1.175	1.373	1.460	-.484	-.577	-.589	-.643	1.119	1.446	1.230	[illegible]
**BPNSF19**	-.428	-.624	-.713	-.677	.961	1.251	1.285	1.420	-.374	-.494	-.631	-.585	1.057	1.084	.951	[illegible]

Sample Covariances - Two Tailed Significance (BC) (g2 - Measurement weights)

	BPNSF6	BPNSF11	BPNSF17	BPNSF23	BPNSF3	BPNSF9	BPNSF14	BPNSF21	BPNSF2	BPNSF8	BPNSF20	BPNSF22	BPNSF4	BPNSF12	BPNSF16	BPNS[illegible]
**BPNSF6**	.012															
**BPNSF11**	.003	.011														
**BPNSF17**	.006	.010	.004													
**BPNSF23**	.003	.006	.005	.011												
**BPNSF3**	.012	.007	.011	.010	.010											
**BPNSF9**	.008	.008	.005	.005	.011	.008										
**BPNSF14**	.012	.007	.002	.004	.009	.005	.003									
**BPNSF21**	.013	.006	.005	.013	.019	.006	.007	.021								
**BPNSF2**	.007	.012	.005	.009	.009	.012	.003	.009	.003							
**BPNSF8**	.009	.010	.007	.002	.010	.010	.008	.010	.009	.005						
**BPNSF20**	.008	.007	.004	.004	.012	.012	.003	.007	.004	.003	.006					
**BPNSF22**	.006	.005	.003	.010	.011	.009	.008	.012	.008	.006	.010	.012				
**BPNSF4**	.020	.006	.009	.010	.012	.007	.007	.006	.011	.015	.012	.021	.011			
**BPNSF12**	.018	.013	.010	.012	.010	.005	.005	.004	.019	.009	.010	.018	.008	.004		
**BPNSF16**	.016	.013	.013	.010	.007	.009	.009	.011	.009	.007	.014	.020	.008	.010	.020	
**BPNSF24**	.019	.011	.006	.004	.009	.003	.004	.010	.004	.009	.012	.009	.012	.004	.013	[illegible]
**BPNSF5**	.012	.006	.009	.014	.025	.044	.012	.020	.013	.007	.012	.006	.019	.011	.013	[illegible]
**BPNSF10**	.007	.004	.009	.003	.009	.008	.005	.010	.006	.005	.009	.005	.013	.006	.013	[illegible]
**BPNSF15**	.007	.006	.007	.003	.027	.011	.034	.010	.006	.021	.007	.006	.025	.012	.013	[illegible]
**BPNSF18**	.131	.013	.018	.017	.926	.292	.403	.027	.011	.010	.012	.009	.162	.274	.063	[illegible]
**BPNSF1**	.023	.012	.015	.010	.007	.005	.005	.009	.011	.010	.016	.013	.012	.005	.025	[illegible]
**BPNSF7**	.965	.009	.005	.010	.015	.009	.006	.008	.015	.013	.011	.004	.004	.006	.005	[illegible]
**BPNSF13**	.014	.010	.008	.018	.010	.006	.009	.007	.007	.012	.011	.011	.006	.004	.013	[illegible]
**BPNSF19**	.007	.006	.004	.010	.014	.012	.005	.007	.012	.010	.009	.011	.009	.006	.012	[illegible]

Sample Correlations (g2 - Measurement weights)

Sample Correlations - Lower Bounds (BC) (g2 - Measurement weights)

	BPNSF6	BPNSF11	BPNSF17	BPNSF23	BPNSF3	BPNSF9	BPNSF14	BPNSF21	BPNSF2	BPNSF8	BPNSF20	BPNSF22	BPNSF4	BPNSF12	BPNSF16	BPNS[illegible]
**BPNSF6**	1.000															
**BPNSF11**	.340	1.000														
**BPNSF17**	.373	.447	1.000													
**BPNSF23**	.407	.440	.556	1.000												
**BPNSF3**	-.409	-.357	-.413	-.439	1.000											
**BPNSF9**	-.396	-.444	-.545	-.507	.416	1.000										
**BPNSF14**	-.347	-.418	-.539	-.444	.356	.496	1.000									
**BPNSF21**	-.353	-.418	-.401	-.430	.361	.375	.446	1.000								
**BPNSF2**	.363	.337	.414	.424	-.397	-.374	-.426	-.386	1.000							
**BPNSF8**	.353	.319	.405	.391	-.347	-.378	-.370	-.374	.453	1.000						
**BPNSF20**	.277	.370	.443	.539	-.398	-.398	-.469	-.473	.470	.468	1.000					
**BPNSF22**	.261	.329	.405	.398	-.269	-.384	-.386	-.425	.421	.420	.494	1.000				
**BPNSF4**	-.332	-.339	-.351	-.374	.565	.322	.321	.435	-.426	-.374	-.450	-.390	1.000			
**BPNSF12**	-.300	-.374	-.430	-.403	.303	.360	.432	.430	-.462	-.411	-.452	-.507	.452	1.000		
**BPNSF16**	-.285	-.357	-.389	-.385	.245	.286	.424	.459	-.412	-.395	-.480	-.464	.389	.616	1.000	
**BPNSF24**	-.285	-.332	-.443	-.391	.191	.270	.395	.352	-.453	-.430	-.492	-.537	.351	.478	.469	[illegible]
**BPNSF5**	.262	.181	.175	.261	-.262	-.216	-.241	-.303	.263	.336	.278	.299	-.394	-.317	-.256	[illegible]
**BPNSF10**	.234	.415	.344	.392	-.339	-.392	-.389	-.426	.325	.388	.395	.384	-.365	-.398	-.346	[illegible]
**BPNSF15**	.227	.320	.255	.231	-.218	-.204	-.197	-.251	.223	.267	.303	.331	-.289	-.271	-.262	[illegible]

	BPNSF6	BPNSF11	BPNSF17	BPNSF23	BPNSF3	BPNSF9	BPNSF14	BPNSF21	BPNSF2	BPNSF8	BPNSF20	BPNSF22	BPNSF4	BPNSF12	BPNSF16	BPNS
**BPNSF18**	-.009	.147	.125	.048	-.085	-.142	-.127	-.201	.067	.092	.161	.109	-.175	-.194	-.183	[illegible]
**BPNSF1**	-.173	-.228	-.213	-.253	.306	.226	.306	.350	-.356	-.281	-.338	-.327	.298	.258	.221	[illegible]
**BPNSF7**	-.093	-.308	-.367	-.227	.202	.366	.344	.302	-.274	-.318	-.311	-.406	.294	.264	.262	[illegible]
**BPNSF13**	-.298	-.414	-.383	-.367	.349	.366	.494	.528	-.363	-.395	-.406	-.444	.378	.544	.429	[illegible]
**BPNSF19**	-.318	-.419	-.447	-.434	.250	.329	.386	.425	-.279	-.327	-.407	-.382	.294	.294	.257	[illegible]

Sample Correlations - Upper Bounds (BC) (g2 - Measurement weights)

	BPNSF6	BPNSF11	BPNSF17	BPNSF23	BPNSF3	BPNSF9	BPNSF14	BPNSF21	BPNSF2	BPNSF8	BPNSF20	BPNSF22	BPNSF4	BPNSF12	BPNSF16	BPNS
**BPNSF6**	1.000															
**BPNSF11**	.500	1.000														
**BPNSF17**	.547	.603	1.000													
**BPNSF23**	.550	.593	.704	1.000												
**BPNSF3**	-.235	-.179	-.246	-.260	1.000											
**BPNSF9**	-.226	-.288	-.371	-.340	.608	1.000										
**BPNSF14**	-.191	-.249	-.390	-.278	.531	.672	1.000									
**BPNSF21**	-.196	-.255	-.230	-.261	.530	.536	.613	1.000								
**BPNSF2**	.512	.491	.572	.583	-.241	-.229	-.261	-.242	1.000							
**BPNSF8**	.510	.497	.545	.535	-.183	-.232	-.202	-.212	.594	1.000						
**BPNSF20**	.446	.529	.598	.681	-.224	-.235	-.286	-.317	.623	.608	1.000					
**BPNSF22**	.429	.487	.549	.544	-.110	-.222	-.210	-.253	.559	.564	.622	1.000				
**BPNSF4**	-.160	-.164	-.200	-.205	.692	.481	.501	.557	-.273	-.216	-.287	-.218	1.000			
**BPNSF12**	-.111	-.207	-.258	-.245	.494	.547	.588	.574	-.305	-.249	-.282	-.344	.618	1.000		
**BPNSF16**	-.118	-.202	-.228	-.202	.441	.483	.570	.610	-.266	-.224	-.295	-.312	.568	.725	1.000	
**BPNSF24**	-.113	-.170	-.280	-.219	.374	.445	.557	.512	-.298	-.271	-.335	-.397	.520	.630	.628	[illegible]
**BPNSF5**	.418	.353	.348	.430	-.093	-.050	-.082	-.128	.399	.500	.460	.478	-.217	-.118	-.085	[illegible]
**BPNSF10**	.389	.556	.480	.528	-.149	-.228	-.234	-.277	.476	.531	.549	.538	-.185	-.216	-.168	[illegible]
**BPNSF15**	.365	.472	.429	.386	-.043	-.047	-.031	-.084	.381	.438	.454	.490	-.112	-.087	-.093	[illegible]
**BPNSF18**	.163	.326	.301	.227	.091	.030	.029	-.023	.229	.264	.324	.289	.011	.028	-.008	[illegible]
**BPNSF1**	-.016	-.070	-.074	-.084	.462	.411	.458	.491	-.203	-.108	-.145	-.126	.482	.434	.381	[illegible]
**BPNSF7**	.087	-.122	-.202	-.057	.403	.540	.517	.467	-.131	-.130	-.145	-.215	.467	.458	.459	[illegible]
**BPNSF13**	-.138	-.237	-.204	-.205	.509	.544	.649	.656	-.211	-.245	-.256	-.288	.521	.661	.581	[illegible]
**BPNSF19**	-.147	-.234	-.285	-.259	.416	.515	.572	.582	-.133	-.178	-.241	-.219	.451	.466	.418	[illegible]

Sample Correlations - Two Tailed Significance (BC) (g2 - Measurement weights)

	BPNSF6	BPNSF11	BPNSF17	BPNSF23	BPNSF3	BPNSF9	BPNSF14	BPNSF21	BPNSF2	BPNSF8	BPNSF20	BPNSF22	BPNSF4	BPNSF12	BPNSF16	BPNS
**BPNSF6**	...															
**BPNSF11**	.004	...														
**BPNSF17**	.011	.016	...													
**BPNSF23**	.004	.008	.006	...												
**BPNSF3**	.019	.009	.012	.014	...											
**BPNSF9**	.012	.019	.013	.011	.008	...										
**BPNSF14**	.016	.009	.007	.013	.019	.011	...									
**BPNSF21**	.012	.007	.008	.015	.026	.007	.011	...								
**BPNSF2**	.006	.008	.010	.014	.012	.019	.019	.012	...							
**BPNSF8**	.006	.014	.012	.004	.015	.013	.009	.008	.011	...						
**BPNSF20**	.011	.008	.006	.010	.018	.015	.006	.010	.013	.006	...					
**BPNSF22**	.011	.007	.002	.012	.012	.014	.009	.008	.011	.006	.018	...				
**BPNSF4**	.020	.006	.007	.010	.011	.011	.007	.012	.015	.013	.021	.019	...			
**BPNSF12**	.032	.015	.020	.019	.014	.009	.012	.012	.025	.012	.021	.016	.013	...		
**BPNSF16**	.011	.012	.014	.007	.012	.013	.007	.008	.016	.011	.014	.018	.007	.014	...	
**BPNSF24**	.021	.020	.011	.006	.011	.008	.004	.015	.010	.010	.023	.011	.012	.012	.034	[illegible]
**BPNSF5**	.012	.004	.013	.011	.036	.041	.012	.026	.012	.009	.015	.006	.026	.013	.018	[illegible]
**BPNSF10**	.007	.010	.013	.006	.014	.008	.009	.010	.015	.006	.012	.008	.021	.007	.012	[illegible]
**BPNSF15**	.005	.007	.007	.004	.030	.011	.036	.012	.008	.013	.009	.008	.023	.019	.012	[illegible]
**BPNSF18**	.131	.016	.020	.018	.946	.292	.403	.027	.007	.013	.010	.010	.141	.252	.075	[illegible]
**BPNSF1**	.025	.013	.018	.013	.012	.009	.012	.014	.012	.012	.020	.016	.016	.013	.019	[illegible]
**BPNSF7**	.945	.011	.006	.020	.020	.009	.004	.009	.019	.018	.006	.004	.004	.009	.006	[illegible]
**BPNSF13**	.018	.016	.016	.023	.015	.009	.016	.012	.014	.013	.012	.010	.012	.009	.014	[illegible]
**BPNSF19**	.015	.007	.003	.014	.012	.021	.011	.012	.026	.021	.013	.014	.011	.009	.012	[illegible]

Sample Means (g2 - Measurement weights)

Sample Means - Lower Bounds (BC) (g2 - Measurement weights)

	BPNSF6	BPNSF11	BPNSF17	BPNSF23	BPNSF3	BPNSF9	BPNSF14	BPNSF21	BPNSF2	BPNSF8	BPNSF20	BPNSF22	BPNSF4	BPNSF12	BPNSF16	BPNS
**BPNSF6**	2.451	2.619	2.333	2.062	5.494	5.608	5.463	5.096	2.184	2.604	2.319	2.940	4.964	5.201	5.101	[illegible]

Sample Means - Upper Bounds (BC) (g2 - Measurement weights)

	BPNSF6	BPNSF11	BPNSF17	BPNSF23	BPNSF3	BPNSF9	BPNSF14	BPNSF21	BPNSF2	BPNSF8	BPNSF20	BPNSF22	BPNSF4	BPNSF12	BPNSF16	BPNSF[illegible]
**BPNSF6**	2.720	2.929	2.622	2.341	5.737	5.857	5.693	5.362	2.437	2.908	2.620	3.225	5.195	5.422	5.335	5.42[illegible]

Sample Means - Two Tailed Significance (BC) (g2 - Measurement weights)

	BPNSF6	BPNSF11	BPNSF17	BPNSF23	BPNSF3	BPNSF9	BPNSF14	BPNSF21	BPNSF2	BPNSF8	BPNSF20	BPNSF22	BPNSF4	BPNSF12	BPNSF16	BPNSF[illegible]
**BPNSF6**	.014	.005	.007	.004	.014	.010	.044	.025	.005	.005	.003	.006	.013	.012	.011	.01[illegible]

g3 (g3 - Measurement weights)

Estimates (g3 - Measurement weights)

Scalar Estimates (g3 - Measurement weights)

Maximum Likelihood Estimates

Regression Weights: (g3 - Measurement weights)

			Estimate	S.E.	C.R.	P	Label
BPNSF19	<---	F1	1.000				
BPNSF13	<---	F1	1.146	.052	21.901	***	a1_1
BPNSF7	<---	F1	.872	.051	16.991	***	a2_1
BPNSF1	<---	F1	.774	.051	15.177	***	a3_1
BPNSF18	<---	F2	1.000				
BPNSF15	<---	F2	2.522	.319	7.905	***	a4_1
BPNSF10	<---	F2	3.235	.395	8.191	***	a5_1
BPNSF5	<---	F2	2.579	.322	8.003	***	a6_1
BPNSF24	<---	F3	1.000				
BPNSF16	<---	F3	1.125	.047	24.171	***	a7_1
BPNSF12	<---	F3	1.221	.048	25.585	***	a8_1
BPNSF4	<---	F3	.861	.044	19.667	***	a9_1
BPNSF22	<---	F4	1.000				
BPNSF20	<---	F4	1.188	.049	24.383	***	a10_1
BPNSF8	<---	F4	1.203	.050	23.925	***	a11_1
BPNSF2	<---	F4	1.115	.049	22.903	***	a12_1
BPNSF21	<---	F5	1.000				
BPNSF14	<---	F5	1.090	.042	25.682	***	a13_1
BPNSF9	<---	F5	1.038	.043	24.375	***	a14_1
BPNSF3	<---	F5	.889	.042	20.973	***	a15_1
BPNSF23	<---	F6	1.000				
BPNSF17	<---	F6	.977	.035	27.907	***	a16_1
BPNSF11	<---	F6	.972	.034	28.226	***	a17_1
BPNSF6	<---	F6	.870	.036	24.502	***	a18_1

Standardized Regression Weights: (g3 - Measurement weights)

			Estimate
BPNSF19	<---	F1	.640
BPNSF13	<---	F1	.723
BPNSF7	<---	F1	.546
BPNSF1	<---	F1	.448
BPNSF18	<---	F2	.265
BPNSF15	<---	F2	.563
BPNSF10	<---	F2	.771
BPNSF5	<---	F2	.612
BPNSF24	<---	F3	.677
BPNSF16	<---	F3	.764
BPNSF12	<---	F3	.819
BPNSF4	<---	F3	.606
BPNSF22	<---	F4	.693
BPNSF20	<---	F4	.750
BPNSF8	<---	F4	.768
BPNSF2	<---	F4	.692
BPNSF21	<---	F5	.713
BPNSF14	<---	F5	.741
BPNSF9	<---	F5	.736
BPNSF3	<---	F5	.606
BPNSF23	<---	F6	.786
BPNSF17	<---	F6	.752
BPNSF11	<---	F6	.785

	Estimate
BPNSF6 <--- F6	.672

Intercepts: (g3 - Measurement weights)

	Estimate	S.E.	C.R.	P	Label
**BPNSF19**	4.926	.068	72.520	***	i1_3
**BPNSF13**	4.783	.069	69.381	***	i2_3
**BPNSF7**	4.717	.069	67.936	***	i3_3
**BPNSF1**	4.672	.075	62.209	***	i4_3
**BPNSF18**	4.474	.071	62.963	***	i5_3
**BPNSF15**	3.730	.085	44.107	***	i6_3
**BPNSF10**	3.118	.079	39.405	***	i7_3
**BPNSF5**	3.901	.080	49.062	***	i8_3
**BPNSF24**	5.117	.067	75.962	***	i9_3
**BPNSF16**	5.019	.067	74.795	***	i10_3
**BPNSF12**	5.107	.068	75.183	***	i11_3
**BPNSF4**	5.204	.065	80.425	***	i12_3
**BPNSF22**	3.274	.078	42.157	***	i13_3
**BPNSF20**	2.762	.085	32.371	***	i14_3
**BPNSF8**	2.916	.084	34.567	***	i15_3
**BPNSF2**	2.644	.087	30.467	***	i16_3
**BPNSF21**	4.999	.068	73.149	***	i17_3
**BPNSF14**	5.183	.072	72.248	***	i18_3
**BPNSF9**	5.461	.069	79.460	***	i19_3
**BPNSF3**	5.491	.071	76.800	***	i20_3
**BPNSF23**	2.503	.084	29.837	***	i21_3
**BPNSF17**	2.828	.086	33.025	***	i22_3
**BPNSF11**	2.806	.082	34.346	***	i23_3
**BPNSF6**	2.632	.085	30.834	***	i24_3

Covariances: (g3 - Measurement weights)

	Estimate	S.E.	C.R.	P	Label
F1 <--> F2	-.167	.032	-5.198	***	ccc1_3
F2 <--> F3	-.150	.030	-4.971	***	ccc2_3
F1 <--> F3	.765	.073	10.524	***	ccc3_3
F2 <--> F4	.365	.055	6.694	***	ccc4_3
F3 <--> F4	-.584	.072	-8.121	***	ccc5_3
F1 <--> F4	-.403	.069	-5.885	***	ccc6_3
F2 <--> F5	-.174	.034	-5.176	***	ccc7_3
F4 <--> F5	-.659	.078	-8.400	***	ccc8_3
F3 <--> F5	.823	.076	10.812	***	ccc9_3
F1 <--> F5	.827	.078	10.617	***	ccc10_3
F6 <--> F5	-1.046	.101	-10.320	***	ccc11_3
F6 <--> F3	-.723	.086	-8.385	***	ccc12_3
F6 <--> F4	1.387	.121	11.464	***	ccc13_3
F6 <--> F2	.434	.065	6.727	***	ccc14_3
F6 <--> F1	-.584	.085	-6.909	***	ccc15_3

Correlations: (g3 - Measurement weights)

	Estimate
F1 <--> F2	-.474
F2 <--> F3	-.405
F1 <--> F3	.898
F2 <--> F4	.836
F3 <--> F4	-.553
F1 <--> F4	-.401
F2 <--> F5	-.440
F4 <--> F5	-.584
F3 <--> F5	.861
F1 <--> F5	.907
F6 <--> F5	-.757
F6 <--> F3	-.560
F6 <--> F4	.909
F6 <--> F2	.812
F6 <--> F1	-.474

Variances: (g3 - Measurement weights)

	Estimate	S.E.	C.R.	P	Label
**F1**	.812	.094	8.667	***	vvv1_3
**F2**	.153	.038	3.975	***	vvv2_3
**F3**	.894	.091	9.866	***	vvv3_3
**F4**	1.247	.126	9.907	***	vvv4_3
**F5**	1.022	.102	10.035	***	vvv5_3
**F6**	1.869	.170	11.018	***	vvv6_3
**e1**	1.172	.090	13.087	***	v1_3
**e2**	.977	.082	11.847	***	v2_3
**e3**	1.456	.106	13.787	***	v3_3
**e4**	1.938	.137	14.179	***	v4_3
**e5**	2.018	.140	14.463	***	v5_3
**e6**	2.102	.157	13.382	***	v6_3
**e7**	1.092	.111	9.867	***	v7_3
**e8**	1.701	.131	12.972	***	v8_3
**e9**	1.057	.081	13.098	***	v9_3
**e10**	.805	.067	11.999	***	v10_3
**e11**	.652	.061	10.681	***	v11_3
**e12**	1.138	.084	13.595	***	v12_3
**e13**	1.347	.103	13.020	***	v13_3
**e14**	1.371	.111	12.342	***	v14_3
**e15**	1.255	.104	12.025	***	v15_3
**e16**	1.688	.129	13.039	***	v16_3
**e17**	.986	.076	13.012	***	v17_3
**e18**	.999	.079	12.680	***	v18_3
**e19**	.931	.073	12.739	***	v19_3
**e20**	1.391	.101	13.798	***	v20_3
**e21**	1.158	.094	12.316	***	v21_3
**e22**	1.370	.107	12.807	***	v22_3
**e23**	1.103	.089	12.327	***	v23_3
**e24**	1.719	.127	13.524	***	v24_3

Matrices (g3 - Measurement weights)

Residual Covariances (g3 - Measurement weights)

	BPNSF6	BPNSF11	BPNSF17	BPNSF23	BPNSF3	BPNSF9	BPNSF14	BPNSF21	BPNSF2	BPNSF8	BPNSF20	BPNSF22	BPNSF4	BPNSF12	BPNSF16	BPNS[illegible]
**BPNSF6**	-.123															
**BPNSF11**	.066	.040														
**BPNSF17**	-.030	-.009	.050													
**BPNSF23**	-.091	-.042	.076	-.002												
**BPNSF3**	-.060	.018	-.117	-.144	-.042											
**BPNSF9**	-.133	-.232	-.317	-.087	.184	.138										
**BPNSF14**	.231	.107	-.053	.061	-.037	.201	-.014									
**BPNSF21**	.258	.092	.345	.080	-.146	-.122	-.121	-.103								
**BPNSF2**	-.173	-.024	-.093	.105	-.286	.001	.063	.242	-.007							
**BPNSF8**	-.019	.129	.010	-.062	-.086	-.220	.192	.133	.081	.100						
**BPNSF20**	-.149	-.049	.286	.313	-.240	-.256	.028	.051	-.006	.004	.025					
**BPNSF22**	-.292	-.142	-.233	.029	-.097	.043	.211	.022	-.161	.034	-.024	-.126				
**BPNSF4**	-.012	.018	.105	.036	.409	-.048	-.209	-.073	.151	.004	-.001	-.092	-.079			
**BPNSF12**	.151	.036	-.032	-.077	-.078	.119	.108	-.004	.054	.128	.009	-.114	-.063	.106		
**BPNSF16**	.183	-.007	-.077	.037	-.196	-.060	.029	.011	.100	.125	-.109	-.081	-.076	.100	-.011	
**BPNSF24**	-.013	-.156	.053	-.138	-.150	-.068	-.074	.159	-.047	-.103	-.114	-.009	-.088	-.027	-.073	-.0[illegible]
**BPNSF5**	.095	.157	-.059	-.046	.003	-.072	.071	.106	.414	.217	-.064	.083	-.134	-.102	-.141	-.1[illegible]
**BPNSF10**	.063	.324	.074	-.043	-.178	-.326	.022	.035	.054	.192	-.123	-.063	-.112	.010	-.007	-.0[illegible]
**BPNSF15**	-.249	-.179	-.094	-.449	.049	.029	.121	.217	-.223	-.025	-.317	-.181	-.119	.186	.088	.0[illegible]
**BPNSF18**	-.239	-.122	-.014	-.330	.055	.337	.445	.258	-.197	-.193	-.192	-.003	-.014	.361	.276	.4[illegible]
**BPNSF1**	.016	-.016	.039	.063	.387	.015	-.188	-.049	.171	-.060	-.056	-.274	.262	-.057	-.129	-.1[illegible]
**BPNSF7**	.279	-.071	.023	.008	-.104	.000	.009	.111	.119	.164	.060	-.065	-.100	.010	-.081	-.1[illegible]
**BPNSF13**	.092	-.065	-.036	.123	-.237	-.005	.028	-.065	.075	.051	-.017	-.191	-.035	.233	.034	-.0[illegible]
**BPNSF19**	-.018	-.123	.021	-.117	-.097	.000	-.015	.297	.132	-.025	-.023	-.054	-.019	-.104	-.010	-.0[illegible]

Residual Means (g3 - Measurement weights)

	BPNSF6	BPNSF11	BPNSF17	BPNSF23	BPNSF3	BPNSF9	BPNSF14	BPNSF21	BPNSF2	BPNSF8	BPNSF20	BPNSF22	BPNSF4	BPNSF12	BPNSF16	BPNSF24	BP[illegible]
	.000	.000	.000	.000	.000	.000	.000	.000	.000	.000	.000	.000	.000	.000	.000	.000	[illegible]

Standardized Residual Covariances (g3 - Measurement weights)

	BPNSF6	BPNSF11	BPNSF17	BPNSF23	BPNSF3	BPNSF9	BPNSF14	BPNSF21	BPNSF2	BPNSF8	BPNSF20	BPNSF22	BPNSF4	BPNSF12	BPNSF16	BPNS[illegible]
**BPNSF6**	-.574															

	BPNSF6	BPNSF11	BPNSF17	BPNSF23	BPNSF3	BPNSF9	BPNSF14	BPNSF21	BPNSF2	BPNSF8	BPNSF20	BPNSF22	BPNSF4	BPNSF12	BPNSF16	BPNS
**BPNSF11**	.403	.204														
**BPNSF17**	-.178	-.055	.235													
**BPNSF23**	-.540	-.252	.441	-.009												
**BPNSF3**	-.453	.138	-.869	-1.085	-.277											
**BPNSF9**	-1.022	-1.828	-2.398	-.667	1.653	.993										
**BPNSF14**	1.702	.809	-.385	.450	-.322	1.725	-.090									
**BPNSF21**	2.004	.731	2.631	.620	-1.319	-1.110	-1.048	-.751								
**BPNSF2**	-1.035	-.145	-.543	.623	-2.158	.010	.466	1.891	-.032							
**BPNSF8**	-.117	.790	.059	-.370	-.662	-1.734	1.453	1.059	.469	.477						
**BPNSF20**	-.898	-.302	1.683	1.862	-1.831	-2.002	.212	.405	-.032	.025	.117					
**BPNSF22**	-1.956	-.970	-1.528	.191	-.815	.369	1.749	.191	-1.039	.220	-.156	-.711				
**BPNSF4**	-.103	.160	.888	.308	4.067	-.485	-2.024	-.745	1.259	.038	-.011	-.856	-.644			
**BPNSF12**	1.199	.298	-.247	-.613	-.710	1.089	.943	-.041	.419	1.019	.071	-.998	-.623	.786		
**BPNSF16**	1.483	-.058	-.615	.302	-1.832	-.565	.260	.105	.797	1.014	-.873	-.723	-.764	.896	-.081	
**BPNSF24**	-.102	-1.312	.426	-1.132	-1.420	-.653	-.679	1.541	-.374	-.840	-.919	-.083	-.899	-.251	-.688	-.6
**BPNSF5**	.640	1.086	-.390	-.310	.027	-.622	.593	.921	2.727	1.455	-.424	.612	-1.238	-.892	-1.256	-1.2
**BPNSF10**	.416	2.170	.477	-.281	-1.482	-2.807	.183	.307	.347	1.243	-.793	-.449	-1.035	.086	-.063	-.2
**BPNSF15**	-1.590	-1.176	-.595	-2.874	.383	.237	.948	1.780	-1.393	-.162	-2.000	-1.264	-1.038	1.532	.741	.5
**BPNSF18**	-1.881	-1.002	-.109	-2.636	.517	3.313	4.197	2.556	-1.526	-1.533	-1.503	-.022	-.146	3.596	2.778	4.3
**BPNSF1**	.121	-.122	.286	.472	3.376	.132	-1.613	-.442	1.256	-.456	-.418	-2.252	2.529	-.516	-1.183	-1.0
**BPNSF7**	2.232	-.590	.186	.068	-.972	-.003	.080	1.063	.944	1.333	.485	-.574	-1.033	.096	-.783	-1.4
**BPNSF13**	.737	-.535	-.283	.987	-2.153	-.046	.247	-.607	.591	.414	-.134	-1.689	-.354	2.115	.319	-.4
**BPNSF19**	-.147	-1.038	.171	-.966	-.910	-.002	-.137	2.848	1.066	-.202	-.188	-.487	-.196	-.986	-.092	-.4

Standardized Residual Means (g3 - Measurement weights)

	BPNSF6	BPNSF11	BPNSF17	BPNSF23	BPNSF3	BPNSF9	BPNSF14	BPNSF21	BPNSF2	BPNSF8	BPNSF20	BPNSF22	BPNSF4	BPNSF12	BPNSF16	BPNSF24	BP
	.000	.000	.000	.000	.000	.000	.000	.000	.000	.000	.000	.000	.000	.000	.000	.000	.

Notes for Group/Model (g3 - Measurement weights)

The following covariance matrix is not positive definite (g3 - Measurement weights)

	F5	F4	F3	F2	F1	F6
**F5**	1.022					
**F4**	-.659	1.247				
**F3**	.823	-.584	.894			
**F2**	-.174	.365	-.150	.153		
**F1**	.827	-.403	.765	-.167	.812	
**F6**	-1.046	1.387	-.723	.434	-.584	1.869

This solution is not admissible.

Modification Indices (g3 - Measurement weights)

Covariances: (g3 - Measurement weights)

			M.I.	Par Change
e24	<-->	F4	4.846	-.106
e23	<-->	F2	9.184	.053
e21	<-->	F4	10.077	.129
e21	<-->	F2	13.604	-.066
e20	<-->	F4	5.468	-.102
e20	<-->	e23	5.441	.154
e19	<-->	e22	6.587	-.157
e19	<-->	e21	5.675	.136
e19	<-->	e20	8.759	.176
e18	<-->	e22	4.428	-.133
e18	<-->	e19	10.481	.168
e17	<-->	F5	11.308	-.099
e17	<-->	F1	11.501	.114
e17	<-->	e22	14.731	.239
e17	<-->	e19	10.438	-.165
e16	<-->	F2	4.254	.044
e16	<-->	F1	5.266	.103
e16	<-->	e20	11.312	-.269
e15	<-->	F2	5.258	.043
e15	<-->	e21	7.683	-.189
e15	<-->	e19	6.146	-.149
e14	<-->	F2	13.184	-.071
e14	<-->	F6	7.918	.136

			M.I.	Par Change
e14	<-->	e23	5.842	-.166
e14	<-->	e22	14.002	.283
e14	<-->	e21	18.238	.301
e13	<-->	F5	7.976	.106
e13	<-->	F1	10.774	-.131
e13	<-->	e22	4.322	-.152
e13	<-->	e21	5.807	.165
e13	<-->	e19	10.980	.200
e13	<-->	e18	6.384	.158
e12	<-->	F2	6.984	-.045
e12	<-->	e20	69.263	.538
e12	<-->	e18	11.348	-.190
e11	<-->	e20	4.062	-.109
e11	<-->	e18	4.169	.096
e10	<-->	e22	4.217	-.121
e10	<-->	e20	9.503	-.177
e9	<-->	e22	5.063	.146
e9	<-->	e17	15.711	.216
e8	<-->	F5	4.766	.091
e8	<-->	F3	4.386	-.091
e8	<-->	e16	16.651	.370
e7	<-->	e23	11.875	.225
e7	<-->	e19	7.649	-.163
e7	<-->	e14	4.202	-.150
e6	<-->	e21	8.103	-.240
e6	<-->	e12	4.884	-.178
e5	<-->	F2	5.319	.051
e5	<-->	e22	4.494	.182
e5	<-->	e18	4.431	.154
e5	<-->	e12	8.388	-.221
e5	<-->	e9	8.949	.223
e5	<-->	e8	17.022	.391
e5	<-->	e6	5.689	.248
e4	<-->	F2	18.203	-.093
e4	<-->	F6	6.702	.143
e4	<-->	e20	36.243	.497
e4	<-->	e18	5.978	-.176
e4	<-->	e16	5.732	.224
e4	<-->	e13	6.085	-.206
e4	<-->	e12	17.707	.317
e4	<-->	e6	4.428	-.216
e4	<-->	e5	26.782	-.504
e3	<-->	e24	6.735	.211
e3	<-->	e17	4.842	.137
e3	<-->	e7	6.879	-.188
e2	<-->	F5	6.714	-.081
e2	<-->	F3	7.392	.088
e2	<-->	e20	12.185	-.215
e2	<-->	e13	6.636	-.161
e2	<-->	e11	17.733	.197
e1	<-->	e17	34.165	.331
e1	<-->	e11	10.851	-.165

Variances: (g3 - Measurement weights)

	M.I.	Par Change

Regression Weights: (g3 - Measurement weights)

			M.I.	Par Change
BPNSF9	<---	F4	6.501	-.119
BPNSF9	<---	F2	6.677	-.356
BPNSF9	<---	F6	9.389	-.115
BPNSF9	<---	BPNSF17	4.094	-.030
BPNSF21	<---	F4	4.677	.103
BPNSF21	<---	F6	8.598	.113
BPNSF21	<---	BPNSF17	5.726	.036
BPNSF5	<---	BPNSF2	4.566	.044
BPNSF15	<---	F4	4.915	-.153
BPNSF15	<---	F2	5.102	-.456
BPNSF15	<---	F6	5.586	-.132

	M.I.	Par Change
BPNSF18 <--- F5	15.218	.279
BPNSF18 <--- F3	15.562	.306
BPNSF18 <--- F1	11.557	.276

Means: (g3 - Measurement weights)

	M.I.	Par Change

Intercepts: (g3 - Measurement weights)

	M.I.	Par Change

Bootstrap (g3 - Measurement weights)

Bootstrap standard errors (g3 - Measurement weights)

Scalar Estimates (g3 - Measurement weights)

Regression Weights: (g3 - Measurement weights)

Parameter	SE	SE-SE	Mean	Bias	SE-Bias
BPNSF19 <--- F1	.000	.000	1.000	.000	.000
BPNSF13 <--- F1	.058	.003	1.141	-.005	.004
BPNSF7 <--- F1	.060	.003	.871	.000	.004
BPNSF1 <--- F1	.073	.004	.774	.000	.005
BPNSF18 <--- F2	.000	.000	1.000	.000	.000
BPNSF15 <--- F2	.413	.021	2.575	.053	.029
BPNSF10 <--- F2	.549	.027	3.306	.072	.039
BPNSF5 <--- F2	.437	.022	2.636	.057	.031
BPNSF24 <--- F3	.000	.000	1.000	.000	.000
BPNSF16 <--- F3	.054	.003	1.126	.001	.004
BPNSF12 <--- F3	.049	.002	1.216	-.005	.003
BPNSF4 <--- F3	.052	.003	.860	-.001	.004
BPNSF22 <--- F4	.000	.000	1.000	.000	.000
BPNSF20 <--- F4	.051	.003	1.186	-.001	.004
BPNSF8 <--- F4	.050	.003	1.198	-.005	.004
BPNSF2 <--- F4	.055	.003	1.114	-.002	.004
BPNSF21 <--- F5	.000	.000	1.000	.000	.000
BPNSF14 <--- F5	.047	.002	1.092	.002	.003
BPNSF9 <--- F5	.054	.003	1.040	.002	.004
BPNSF3 <--- F5	.061	.003	.894	.005	.004
BPNSF23 <--- F6	.000	.000	1.000	.000	.000
BPNSF17 <--- F6	.034	.002	.980	.004	.002
BPNSF11 <--- F6	.038	.002	.973	.001	.003
BPNSF6 <--- F6	.037	.002	.870	.000	.003

Standardized Regression Weights: (g3 - Measurement weights)

Parameter	SE	SE-SE	Mean	Bias	SE-Bias
BPNSF19 <--- F1	.035	.002	.641	.002	.002
BPNSF13 <--- F1	.029	.001	.719	-.003	.002
BPNSF7 <--- F1	.038	.002	.546	.000	.003
BPNSF1 <--- F1	.039	.002	.448	.000	.003
BPNSF18 <--- F2	.041	.002	.267	.001	.003
BPNSF15 <--- F2	.030	.001	.562	-.001	.002
BPNSF10 <--- F2	.034	.002	.771	.000	.002
BPNSF5 <--- F2	.035	.002	.611	-.001	.002
BPNSF24 <--- F3	.030	.001	.677	.000	.002
BPNSF16 <--- F3	.032	.002	.766	.002	.002
BPNSF12 <--- F3	.022	.001	.818	-.001	.002
BPNSF4 <--- F3	.036	.002	.605	-.002	.003
BPNSF22 <--- F4	.025	.001	.695	.002	.002
BPNSF20 <--- F4	.031	.002	.752	.002	.002
BPNSF8 <--- F4	.029	.001	.765	-.003	.002
BPNSF2 <--- F4	.032	.002	.692	.000	.002
BPNSF21 <--- F5	.032	.002	.717	.004	.002
BPNSF14 <--- F5	.035	.002	.744	.004	.002
BPNSF9 <--- F5	.033	.002	.740	.004	.002
BPNSF3 <--- F5	.045	.002	.611	.005	.003

Parameter			SE	SE-SE	Mean	Bias	SE-Bias
BPNSF23	<---	F6	.023	.001	.786	.001	.002
BPNSF17	<---	F6	.028	.001	.754	.002	.002
BPNSF11	<---	F6	.025	.001	.783	-.001	.002
BPNSF6	<---	F6	.033	.002	.670	-.002	.002

Intercepts: (g3 - Measurement weights)

Parameter	SE	SE-SE	Mean	Bias	SE-Bias
**BPNSF19**	.075	.004	4.922	-.004	.005
**BPNSF13**	.066	.003	4.780	-.003	.005
**BPNSF7**	.076	.004	4.715	-.002	.005
**BPNSF1**	.077	.004	4.675	.003	.005
**BPNSF18**	.071	.004	4.477	.003	.005
**BPNSF15**	.076	.004	3.736	.006	.005
**BPNSF10**	.079	.004	3.120	.001	.006
**BPNSF5**	.081	.004	3.896	-.005	.006
**BPNSF24**	.071	.004	5.114	-.003	.005
**BPNSF16**	.071	.004	5.022	.003	.005
**BPNSF12**	.070	.003	5.113	.005	.005
**BPNSF4**	.063	.003	5.201	-.003	.004
**BPNSF22**	.074	.004	3.276	.002	.005
**BPNSF20**	.083	.004	2.756	-.006	.006
**BPNSF8**	.081	.004	2.920	.004	.006
**BPNSF2**	.088	.004	2.647	.002	.006
**BPNSF21**	.063	.003	4.999	.001	.004
**BPNSF14**	.070	.004	5.178	-.005	.005
**BPNSF9**	.078	.004	5.462	.000	.006
**BPNSF3**	.073	.004	5.486	-.005	.005
**BPNSF23**	.074	.004	2.503	.000	.005
**BPNSF17**	.086	.004	2.825	-.003	.006
**BPNSF11**	.077	.004	2.812	.006	.005
**BPNSF6**	.080	.004	2.642	.010	.006

Covariances: (g3 - Measurement weights)

Parameter			SE	SE-SE	Mean	Bias	SE-Bias
F1	<-->	F2	.032	.002	-.166	.001	.002
F2	<-->	F3	.027	.001	-.148	.002	.002
F1	<-->	F3	.063	.003	.759	-.005	.004
F2	<-->	F4	.063	.003	.364	-.001	.004
F3	<-->	F4	.071	.004	-.586	-.002	.005
F1	<-->	F4	.066	.003	-.406	-.003	.005
F2	<-->	F5	.032	.002	-.171	.003	.002
F4	<-->	F5	.071	.004	-.657	.001	.005
F3	<-->	F5	.077	.004	.819	-.004	.005
F1	<-->	F5	.084	.004	.825	-.002	.006
F6	<-->	F5	.089	.004	-1.049	-.003	.006
F6	<-->	F3	.081	.004	-.722	.001	.006
F6	<-->	F4	.100	.005	1.384	-.004	.007
F6	<-->	F2	.071	.004	.432	-.002	.005
F6	<-->	F1	.078	.004	-.588	-.004	.006

Correlations: (g3 - Measurement weights)

Parameter			SE	SE-SE	Mean	Bias	SE-Bias
F1	<-->	F2	.062	.003	-.473	.000	.004
F2	<-->	F3	.054	.003	-.403	.001	.004
F1	<-->	F3	.036	.002	.895	-.003	.003
F2	<-->	F4	.038	.002	.834	-.002	.003
F3	<-->	F4	.055	.003	-.556	-.003	.004
F1	<-->	F4	.060	.003	-.404	-.003	.004
F2	<-->	F5	.061	.003	-.435	.005	.004
F4	<-->	F5	.051	.003	-.581	.002	.004
F3	<-->	F5	.042	.002	.857	-.005	.003
F1	<-->	F5	.047	.002	.904	-.004	.003
F6	<-->	F5	.036	.002	-.758	-.001	.003
F6	<-->	F3	.047	.002	-.560	-.001	.003
F6	<-->	F4	.031	.002	.906	-.002	.002
F6	<-->	F2	.046	.002	.810	-.001	.003
F6	<-->	F1	.052	.003	-.478	-.004	.004

Variances: (g3 - Measurement weights)

Parameter	SE	SE-SE	Mean	Bias	SE-Bias
**F1**	.091	.005	.813	.001	.006
**F2**	.049	.002	.157	.004	.003
**F3**	.077	.004	.891	-.003	.005
**F4**	.106	.005	1.250	.003	.008
**F5**	.105	.005	1.029	.007	.007
**F6**	.141	.007	1.868	.000	.010
**e1**	.134	.007	1.162	-.011	.009
**e2**	.105	.005	.981	.004	.007
**e3**	.163	.008	1.447	-.009	.012
**e4**	.173	.009	1.922	-.016	.012
**e5**	.146	.007	1.996	-.023	.010
**e6**	.159	.008	2.096	-.006	.011
**e7**	.155	.008	1.085	-.007	.011
**e8**	.157	.008	1.698	-.003	.011
**e9**	.128	.006	1.057	.000	.009
**e10**	.114	.006	.793	-.013	.008
**e11**	.080	.004	.646	-.006	.006
**e12**	.147	.007	1.142	.004	.010
**e13**	.120	.006	1.333	-.014	.009
**e14**	.189	.009	1.353	-.018	.013
**e15**	.151	.008	1.265	.009	.011
**e16**	.199	.010	1.683	-.006	.014
**e17**	.133	.007	.970	-.016	.009
**e18**	.145	.007	.984	-.014	.010
**e19**	.125	.006	.917	-.013	.009
**e20**	.222	.011	1.382	-.009	.016
**e21**	.131	.007	1.151	-.007	.009
**e22**	.166	.008	1.358	-.012	.012
**e23**	.134	.007	1.110	.007	.010
**e24**	.217	.011	1.735	.016	.015

Matrices (g3 - Measurement weights)

Sample Covariances - Standard Errors (g3 - Measurement weights)

	BPNSF6	BPNSF11	BPNSF17	BPNSF23	BPNSF3	BPNSF9	BPNSF14	BPNSF21	BPNSF2	BPNSF8	BPNSF20	BPNSF22	BPNSF4	BPNSF12	BPNSF16	BPNS[illegible]
**BPNSF6**	.174															
**BPNSF11**	.152	.149														
**BPNSF17**	.156	.158	.157													
**BPNSF23**	.148	.139	.145	.159												
**BPNSF3**	.126	.127	.121	.132	.192											
**BPNSF9**	.138	.124	.143	.135	.141	.177										
**BPNSF14**	.122	.118	.144	.129	.125	.136	.147									
**BPNSF21**	.111	.130	.132	.113	.123	.120	.119	.134								
**BPNSF2**	.166	.157	.165	.138	.132	.126	.130	.112	.180							
**BPNSF8**	.149	.146	.165	.145	.142	.145	.130	.129	.170	.163						
**BPNSF20**	.157	.154	.159	.156	.127	.141	.134	.133	.159	.160	.176					
**BPNSF22**	.142	.130	.152	.137	.133	.134	.135	.120	.131	.138	.140	.132				
**BPNSF4**	.114	.118	.113	.099	.135	.106	.103	.114	.122	.113	.116	.114	.127			
**BPNSF12**	.119	.124	.137	.125	.122	.131	.127	.103	.123	.137	.135	.122	.095	.138		
**BPNSF16**	.123	.117	.123	.119	.110	.130	.117	.112	.108	.134	.138	.117	.094	.114	.120	
**BPNSF24**	.112	.108	.121	.111	.120	.113	.111	.106	.124	.123	.120	.117	.104	.104	.095	.1[illegible]
**BPNSF5**	.141	.131	.140	.129	.130	.112	.127	.118	.161	.151	.154	.140	.112	.123	.113	.1[illegible]
**BPNSF10**	.160	.140	.151	.132	.124	.132	.116	.113	.148	.149	.139	.134	.111	.108	.117	.1[illegible]
**BPNSF15**	.166	.134	.158	.133	.126	.120	.130	.131	.130	.151	.157	.139	.112	.120	.115	.1[illegible]
**BPNSF18**	.129	.121	.138	.115	.109	.110	.121	.103	.139	.144	.131	.121	.102	.117	.110	.1[illegible]
**BPNSF1**	.145	.127	.148	.126	.141	.125	.133	.128	.140	.150	.143	.129	.117	.123	.125	.1[illegible]
**BPNSF7**	.129	.123	.135	.113	.134	.123	.121	.107	.138	.153	.122	.137	.114	.111	.117	.1[illegible]
**BPNSF13**	.132	.114	.130	.118	.124	.118	.123	.101	.125	.149	.135	.127	.097	.117	.111	.1[illegible]
**BPNSF19**	.108	.103	.128	.100	.098	.119	.118	.107	.116	.133	.125	.104	.095	.108	.102	.0[illegible]

Sample Correlations - Standard Errors (g3 - Measurement weights)

	BPNSF6	BPNSF11	BPNSF17	BPNSF23	BPNSF3	BPNSF9	BPNSF14	BPNSF21	BPNSF2	BPNSF8	BPNSF20	BPNSF22	BPNSF4	BPNSF12	BPNSF16	BPNS[illegible]
**BPNSF6**	.000															
**BPNSF11**	.046	.000														
**BPNSF17**	.047	.045	.000													
**BPNSF23**	.045	.035	.039	.000												

	BPNSF6	BPNSF11	BPNSF17	BPNSF23	BPNSF3	BPNSF9	BPNSF14	BPNSF21	BPNSF2	BPNSF8	BPNSF20	BPNSF22	BPNSF4	BPNSF12	BPNSF16	BPNS[illegible]
**BPNSF3**	.052	.053	.048	.053	.000											
**BPNSF9**	.050	.042	.044	.045	.059	.000										
**BPNSF14**	.047	.047	.050	.046	.052	.041	.000									
**BPNSF21**	.048	.051	.052	.044	.056	.053	.055	.000								
**BPNSF2**	.052	.048	.049	.043	.051	.049	.050	.049	.000							
**BPNSF8**	.045	.039	.046	.040	.053	.049	.051	.052	.048	.000						
**BPNSF20**	.048	.045	.038	.038	.049	.048	.048	.054	.045	.045	.000					
**BPNSF22**	.051	.042	.051	.041	.054	.058	.058	.054	.041	.040	.047	.000				
**BPNSF4**	.050	.053	.048	.043	.054	.053	.052	.059	.055	.050	.050	.056	.000			
**BPNSF12**	.046	.048	.050	.049	.054	.041	.048	.043	.048	.054	.051	.049	.049	.000		
**BPNSF16**	.052	.048	.048	.050	.056	.054	.048	.051	.044	.055	.052	.052	.053	.038	.000	
**BPNSF24**	.045	.044	.050	.044	.058	.051	.051	.046	.051	.050	.049	.054	.058	.043	.047	[illegible]
**BPNSF5**	.044	.039	.043	.040	.053	.043	.050	.051	.043	.043	.047	.047	.050	.048	.047	[illegible]
**BPNSF10**	.054	.038	.044	.041	.054	.049	.048	.050	.048	.044	.045	.049	.052	.046	.051	[illegible]
**BPNSF15**	.054	.043	.050	.045	.051	.047	.051	.057	.043	.047	.049	.049	.051	.050	.050	[illegible]
**BPNSF18**	.051	.048	.050	.045	.050	.050	.055	.051	.053	.055	.049	.050	.052	.055	.054	[illegible]
**BPNSF1**	.054	.049	.053	.047	.051	.052	.057	.060	.050	.054	.052	.051	.053	.053	.058	[illegible]
**BPNSF7**	.052	.051	.052	.045	.065	.056	.056	.049	.054	.060	.049	.061	.058	.051	.058	[illegible]
**BPNSF13**	.052	.048	.049	.050	.058	.046	.052	.048	.050	.061	.054	.054	.048	.037	.047	[illegible]
**BPNSF19**	.046	.041	.051	.042	.049	.050	.056	.038	.047	.052	.052	.048	.049	.050	.046	[illegible]

Sample Means - Standard Errors (g3 - Measurement weights)

	BPNSF6	BPNSF11	BPNSF17	BPNSF23	BPNSF3	BPNSF9	BPNSF14	BPNSF21	BPNSF2	BPNSF8	BPNSF20	BPNSF22	BPNSF4	BPNSF12	BPNSF16	BPNSF[illegible]
**BPNSF6**	.080	.077	.086	.074	.073	.078	.070	.063	.088	.081	.083	.074	.063	.070	.071	[illegible]

Bootstrap Confidence (g3 - Measurement weights)

Percentile method (g3 - Measurement weights)

90% confidence intervals (percentile method)

Scalar Estimates (g3 - Measurement weights)

Regression Weights: (g3 - Measurement weights)

Parameter			Estimate	Lower	Upper	P
BPNSF19	<---	F1	1.000	1.000	1.000	...
BPNSF13	<---	F1	1.146	1.045	1.234	.010
BPNSF7	<---	F1	.872	.767	.971	.010
BPNSF1	<---	F1	.774	.651	.908	.010
BPNSF18	<---	F2	1.000	1.000	1.000	...
BPNSF15	<---	F2	2.522	2.037	3.351	.010
BPNSF10	<---	F2	3.235	2.580	4.410	.010
BPNSF5	<---	F2	2.579	2.034	3.484	.010
BPNSF24	<---	F3	1.000	1.000	1.000	...
BPNSF16	<---	F3	1.125	1.040	1.218	.010
BPNSF12	<---	F3	1.221	1.134	1.299	.010
BPNSF4	<---	F3	.861	.771	.947	.010
BPNSF22	<---	F4	1.000	1.000	1.000	...
BPNSF20	<---	F4	1.188	1.107	1.281	.010
BPNSF8	<---	F4	1.203	1.117	1.281	.010
BPNSF2	<---	F4	1.115	1.029	1.215	.010
BPNSF21	<---	F5	1.000	1.000	1.000	...
BPNSF14	<---	F5	1.090	1.014	1.183	.010
BPNSF9	<---	F5	1.038	.959	1.137	.010
BPNSF3	<---	F5	.889	.797	.997	.010
BPNSF23	<---	F6	1.000	1.000	1.000	...
BPNSF17	<---	F6	.977	.925	1.044	.010
BPNSF11	<---	F6	.972	.915	1.036	.010
BPNSF6	<---	F6	.870	.809	.937	.010

Standardized Regression Weights: (g3 - Measurement weights)

Parameter			Estimate	Lower	Upper	P
BPNSF19	<---	F1	.640	.580	.703	.010
BPNSF13	<---	F1	.723	.671	.767	.010
BPNSF7	<---	F1	.546	.481	.610	.010
BPNSF1	<---	F1	.448	.387	.512	.010

Parameter			Estimate	Lower	Upper	P
BPNSF18	<---	F2	.265	.202	.332	.010
BPNSF15	<---	F2	.563	.508	.608	.010
BPNSF10	<---	F2	.771	.715	.827	.010
BPNSF5	<---	F2	.612	.556	.675	.010
BPNSF24	<---	F3	.677	.627	.733	.010
BPNSF16	<---	F3	.764	.714	.816	.010
BPNSF12	<---	F3	.819	.782	.856	.010
BPNSF4	<---	F3	.606	.547	.673	.010
BPNSF22	<---	F4	.693	.652	.737	.010
BPNSF20	<---	F4	.750	.698	.800	.010
BPNSF8	<---	F4	.768	.716	.815	.010
BPNSF2	<---	F4	.692	.637	.744	.010
BPNSF21	<---	F5	.713	.657	.774	.010
BPNSF14	<---	F5	.741	.685	.803	.010
BPNSF9	<---	F5	.736	.686	.794	.010
BPNSF3	<---	F5	.606	.529	.682	.010
BPNSF23	<---	F6	.786	.750	.829	.010
BPNSF17	<---	F6	.752	.712	.799	.010
BPNSF11	<---	F6	.785	.734	.824	.010
BPNSF6	<---	F6	.672	.618	.730	.010

Intercepts: (g3 - Measurement weights)

Parameter	Estimate	Lower	Upper	P
**BPNSF19**	4.926	4.800	5.057	.010
**BPNSF13**	4.783	4.674	4.885	.010
**BPNSF7**	4.717	4.587	4.832	.010
**BPNSF1**	4.672	4.530	4.800	.010
**BPNSF18**	4.474	4.362	4.598	.010
**BPNSF15**	3.730	3.608	3.849	.010
**BPNSF10**	3.118	2.989	3.241	.010
**BPNSF5**	3.901	3.773	4.058	.010
**BPNSF24**	5.117	5.001	5.227	.010
**BPNSF16**	5.019	4.900	5.137	.010
**BPNSF12**	5.107	4.998	5.234	.010
**BPNSF4**	5.204	5.091	5.299	.010
**BPNSF22**	3.274	3.146	3.394	.010
**BPNSF20**	2.762	2.612	2.898	.010
**BPNSF8**	2.916	2.777	3.057	.010
**BPNSF2**	2.644	2.510	2.809	.010
**BPNSF21**	4.999	4.893	5.097	.010
**BPNSF14**	5.183	5.052	5.294	.010
**BPNSF9**	5.461	5.333	5.584	.010
**BPNSF3**	5.491	5.371	5.613	.010
**BPNSF23**	2.503	2.387	2.629	.010
**BPNSF17**	2.828	2.685	2.973	.010
**BPNSF11**	2.806	2.681	2.931	.010
**BPNSF6**	2.632	2.512	2.774	.010

Covariances: (g3 - Measurement weights)

Parameter			Estimate	Lower	Upper	P
F1	<-->	F2	-.167	-.223	-.115	.010
F2	<-->	F3	-.150	-.203	-.107	.010
F1	<-->	F3	.765	.653	.873	.010
F2	<-->	F4	.365	.265	.479	.010
F3	<-->	F4	-.584	-.708	-.467	.010
F1	<-->	F4	-.403	-.515	-.298	.010
F2	<-->	F5	-.174	-.230	-.123	.010
F4	<-->	F5	-.659	-.776	-.548	.010
F3	<-->	F5	.823	.700	.963	.010
F1	<-->	F5	.827	.682	.974	.010
F6	<-->	F5	-1.046	-1.196	-.898	.010
F6	<-->	F3	-.723	-.849	-.574	.010
F6	<-->	F4	1.387	1.208	1.550	.010
F6	<-->	F2	.434	.311	.557	.010
F6	<-->	F1	-.584	-.716	-.459	.010

Correlations: (g3 - Measurement weights)

Parameter	Estimate	Lower	Upper	P

Parameter	Estimate	Lower	Upper	P
F1 <--> F2	-.474	-.575	-.362	.010
F2 <--> F3	-.405	-.500	-.319	.010
F1 <--> F3	.898	.831	.950	.010
F2 <--> F4	.836	.767	.893	.010
F3 <--> F4	-.553	-.647	-.468	.010
F1 <--> F4	-.401	-.510	-.304	.010
F2 <--> F5	-.440	-.529	-.321	.010
F4 <--> F5	-.584	-.672	-.497	.010
F3 <--> F5	.861	.778	.921	.010
F1 <--> F5	.907	.826	.981	.010
F6 <--> F5	-.757	-.826	-.706	.010
F6 <--> F3	-.560	-.634	-.473	.010
F6 <--> F4	.909	.847	.955	.010
F6 <--> F2	.812	.723	.886	.010
F6 <--> F1	-.474	-.554	-.395	.010

Variances: (g3 - Measurement weights)

Parameter	Estimate	Lower	Upper	P
**F1**	.812	.664	.975	.010
**F2**	.153	.085	.244	.010
**F3**	.894	.778	1.027	.010
**F4**	1.247	1.060	1.415	.010
**F5**	1.022	.846	1.200	.010
**F6**	1.869	1.635	2.096	.010
**e1**	1.172	.951	1.383	.010
**e2**	.977	.802	1.161	.010
**e3**	1.456	1.194	1.727	.010
**e4**	1.938	1.645	2.214	.010
**e5**	2.018	1.768	2.239	.010
**e6**	2.102	1.844	2.362	.010
**e7**	1.092	.843	1.359	.010
**e8**	1.701	1.453	1.955	.010
**e9**	1.057	.824	1.269	.010
**e10**	.805	.606	.997	.010
**e11**	.652	.521	.770	.010
**e12**	1.138	.913	1.447	.010
**e13**	1.347	1.120	1.539	.010
**e14**	1.371	1.061	1.659	.010
**e15**	1.255	.993	1.516	.010
**e16**	1.688	1.366	2.044	.010
**e17**	.986	.762	1.181	.010
**e18**	.999	.744	1.237	.010
**e19**	.931	.706	1.097	.010
**e20**	1.391	1.043	1.791	.010
**e21**	1.158	.939	1.361	.010
**e22**	1.370	1.067	1.613	.010
**e23**	1.103	.894	1.352	.010
**e24**	1.719	1.389	2.084	.010

Matrices (g3 - Measurement weights)

Sample Covariances (g3 - Measurement weights)

Sample Covariances - Lower Bounds (PC) (g3 - Measurement weights)

	BPNSF6	BPNSF11	BPNSF17	BPNSF23	BPNSF3	BPNSF9	BPNSF14	BPNSF21	BPNSF2	BPNSF8	BPNSF20	BPNSF22	BPNSF4	BPNSF12	BPNSF16	BPNSI
**BPNSF6**	2.741															
**BPNSF11**	1.342	2.674														
**BPNSF17**	1.302	1.484	2.920													
**BPNSF23**	1.275	1.528	1.661	2.755												
**BPNSF3**	-1.083	-1.079	-1.205	-1.282	1.804											
**BPNSF9**	-1.293	-1.467	-1.607	-1.391	.893	1.845										
**BPNSF14**	-.980	-1.195	-1.410	-1.275	.753	1.117	1.969									
**BPNSF21**	-.839	-1.160	-.933	-1.145	.571	.751	.818	1.654								
**BPNSF2**	.894	1.227	1.122	1.401	-1.146	-.928	-.951	-.654	2.898							
**BPNSF8**	1.159	1.513	1.360	1.362	-1.027	-1.306	-.899	-.865	1.434	2.851						
**BPNSF20**	.993	1.299	1.625	1.706	-1.151	-1.333	-1.074	-.967	1.341	1.505	2.817					
**BPNSF22**	.635	.987	.861	1.198	-.906	-.860	-.732	-.816	1.025	1.283	1.224	2.221				
**BPNSF4**	-.745	-.794	-.705	-.753	.824	.507	.377	.458	-.612	-.789	-.810	-.773	1.524			

	BPNSF6	BPNSF11	BPNSF17	BPNSF23	BPNSF3	BPNSF9	BPNSF14	BPNSF21	BPNSF2	BPNSF8	BPNSF20	BPNSF22	BPNSF4	BPNSF12	BPNSF16	BPNS[illegible]
**BPNSF12**	-.815	-.997	-1.115	-1.163	.629	.912	.984	.836	-.924	-.947	-1.094	-1.015	.722	1.826		
**BPNSF16**	-.728	-1.013	-1.080	-.975	.430	.672	.836	.733	-.817	-.918	-1.156	-.940	.632	1.137	1.696	
**BPNSF24**	-.831	-1.018	-.843	-1.042	.391	.601	.675	.789	-.907	-1.015	-1.006	-.763	.519	.900	.763	[illegible]
**BPNSF5**	.830	1.039	.779	.864	-.603	-.720	-.633	-.517	1.190	1.098	.797	.771	-.639	-.784	-.736	[illegible]
**BPNSF10**	1.012	1.468	1.207	1.133	-.882	-1.125	-.782	-.721	1.116	1.360	1.047	.885	-.706	-.766	-.742	[illegible]
**BPNSF15**	.424	.676	.684	.406	-.548	-.608	-.554	-.428	.560	.819	.519	.523	-.627	-.439	-.509	[illegible]
**BPNSF18**	-.082	.079	.155	-.094	-.271	-.010	.080	-.052	-.049	.007	.017	.161	-.295	.009	-.078	[illegible]
**BPNSF1**	-.629	-.678	-.641	-.604	.708	.478	.301	.389	-.440	-.686	-.675	-.834	.583	.465	.322	[illegible]
**BPNSF7**	-.373	-.775	-.674	-.707	.311	.517	.612	.652	-.495	-.493	-.542	-.638	.291	.630	.475	[illegible]
**BPNSF13**	-.698	-.902	-.905	-.729	.405	.776	.858	.681	-.643	-.759	-.808	-.870	.538	1.081	.807	[illegible]
**BPNSF19**	-.721	-.859	-.762	-.880	.464	.669	.695	.945	-.523	-.721	-.740	-.637	.475	.612	.649	[illegible]

Sample Covariances - Upper Bounds (PC) (g3 - Measurement weights)

	BPNSF6	BPNSF11	BPNSF17	BPNSF23	BPNSF3	BPNSF9	BPNSF14	BPNSF21	BPNSF2	BPNSF8	BPNSF20	BPNSF22	BPNSF4	BPNSF12	BPNSF16	BPNS[illegible]
**BPNSF6**	3.323															
**BPNSF11**	1.864	3.156														
**BPNSF17**	1.813	2.010	3.441													
**BPNSF23**	1.796	1.996	2.122	3.260												
**BPNSF3**	-.667	-.682	-.836	-.868	2.439											
**BPNSF9**	-.838	-1.065	-1.124	-.945	1.357	2.458										
**BPNSF14**	-.559	-.812	-.894	-.884	1.177	1.571	2.426									
**BPNSF21**	-.464	-.703	-.468	-.773	.974	1.142	1.225	2.085								
**BPNSF2**	1.433	1.722	1.665	1.867	-.712	-.538	-.503	-.290	3.519							
**BPNSF8**	1.668	2.010	1.907	1.840	-.549	-.798	-.480	-.435	2.002	3.409						
**BPNSF20**	1.524	1.790	2.155	2.210	-.758	-.850	-.623	-.528	1.912	2.039	3.413					
**BPNSF22**	1.124	1.403	1.360	1.642	-.464	-.405	-.282	-.416	1.452	1.757	1.712	2.691				
**BPNSF4**	-.361	-.388	-.311	-.429	1.288	.862	.723	.835	-.205	-.408	-.427	-.384	1.950			
**BPNSF12**	-.429	-.598	-.641	-.739	1.005	1.373	1.415	1.160	-.526	-.492	-.635	-.615	1.038	2.284		
**BPNSF16**	-.332	-.588	-.662	-.596	.821	1.106	1.227	1.091	-.455	-.432	-.678	-.542	.947	1.496	2.081	
**BPNSF24**	-.450	-.664	-.434	-.656	.777	.976	1.024	1.165	-.511	-.588	-.620	-.386	.854	1.240	1.097	[illegible]
**BPNSF5**	1.294	1.442	1.256	1.280	-.159	-.356	-.211	-.127	1.709	1.574	1.312	1.256	-.268	-.367	-.367	[illegible]
**BPNSF10**	1.559	1.919	1.691	1.606	-.449	-.698	-.390	-.335	1.635	1.871	1.538	1.344	-.313	-.410	-.373	[illegible]
**BPNSF15**	.959	1.134	1.223	.858	-.137	-.205	-.131	-.007	.998	1.297	1.016	.972	-.272	-.030	-.132	[illegible]
**BPNSF18**	.343	.497	.627	.305	.093	.350	.466	.271	.410	.495	.474	.564	.020	.384	.299	[illegible]
**BPNSF1**	-.131	-.251	-.144	-.182	1.181	.880	.742	.810	.024	-.185	-.192	-.379	.984	.867	.732	[illegible]
**BPNSF7**	.060	-.376	-.236	-.313	.769	.925	1.011	1.017	-.030	-.013	-.149	-.177	.662	.982	.849	[illegible]
**BPNSF13**	-.240	-.526	-.450	-.360	.823	1.163	1.256	1.038	-.231	-.244	-.352	-.444	.867	1.454	1.177	[illegible]
**BPNSF19**	-.334	-.527	-.352	-.548	.796	1.060	1.088	1.298	-.136	-.275	-.311	-.280	.788	.993	1.003	[illegible]

Sample Covariances - Two Tailed Significance (PC) (g3 - Measurement weights)

	BPNSF6	BPNSF11	BPNSF17	BPNSF23	BPNSF3	BPNSF9	BPNSF14	BPNSF21	BPNSF2	BPNSF8	BPNSF20	BPNSF22	BPNSF4	BPNSF12	BPNSF16	BPNS[illegible]
**BPNSF6**	.010															
**BPNSF11**	.010	.010														
**BPNSF17**	.010	.010	.010													
**BPNSF23**	.010	.010	.010	.010												
**BPNSF3**	.010	.010	.010	.010	.010											
**BPNSF9**	.010	.010	.010	.010	.010	.010										
**BPNSF14**	.010	.010	.010	.010	.010	.010	.010									
**BPNSF21**	.010	.010	.010	.010	.010	.010	.010	.010								
**BPNSF2**	.010	.010	.010	.010	.010	.010	.010	.010	.010							
**BPNSF8**	.010	.010	.010	.010	.010	.010	.010	.010	.010	.010						
**BPNSF20**	.010	.010	.010	.010	.010	.010	.010	.010	.010	.010	.010					
**BPNSF22**	.010	.010	.010	.010	.010	.010	.010	.010	.010	.010	.010	.010				
**BPNSF4**	.010	.010	.010	.010	.010	.010	.010	.010	.010	.010	.010	.010	.010			
**BPNSF12**	.010	.010	.010	.010	.010	.010	.010	.010	.010	.010	.010	.010	.010	.010		
**BPNSF16**	.010	.010	.010	.010	.010	.010	.010	.010	.010	.010	.010	.010	.010	.010	.010	
**BPNSF24**	.010	.010	.010	.010	.010	.010	.010	.010	.010	.010	.010	.010	.010	.010	.010	[illegible]
**BPNSF5**	.010	.010	.010	.010	.010	.010	.010	.010	.010	.010	.010	.010	.010	.010	.010	[illegible]
**BPNSF10**	.010	.010	.010	.010	.010	.010	.010	.010	.010	.010	.010	.010	.010	.010	.010	[illegible]
**BPNSF15**	.010	.010	.010	.010	.010	.010	.016	.097	.010	.010	.010	.010	.010	.066	.014	[illegible]
**BPNSF18**	.373	.022	.010	.386	.431	.135	.010	.336	.197	.090	.074	.012	.149	.095	.281	[illegible]
**BPNSF1**	.019	.010	.010	.010	.010	.010	.010	.010	.175	.010	.010	.010	.010	.010	.010	[illegible]
**BPNSF7**	.178	.010	.010	.010	.010	.010	.010	.010	.062	.094	.010	.010	.010	.010	.010	[illegible]
**BPNSF13**	.010	.010	.010	.010	.010	.010	.010	.010	.010	.010	.010	.010	.010	.010	.010	[illegible]
**BPNSF19**	.010	.010	.010	.010	.010	.010	.010	.010	.016	.010	.010	.010	.010	.010	.010	[illegible]

Sample Correlations (g3 - Measurement weights)

Sample Correlations - Lower Bounds (PC) (g3 - Measurement weights)

	BPNSF6	BPNSF11	BPNSF17	BPNSF23	BPNSF3	BPNSF9	BPNSF14	BPNSF21	BPNSF2	BPNSF8	BPNSF20	BPNSF22	BPNSF4	BPNSF12	BPNSF16	BPNS[illegible]
**BPNSF6**	1.000															
**BPNSF11**	.468	1.000														
**BPNSF17**	.419	.488	1.000													
**BPNSF23**	.425	.543	.550	1.000												
**BPNSF3**	-.426	-.445	-.482	-.508	1.000											
**BPNSF9**	-.501	-.576	-.601	-.533	.424	1.000										
**BPNSF14**	-.381	-.472	-.526	-.499	.352	.546	1.000									
**BPNSF21**	-.352	-.477	-.371	-.484	.283	.389	.400	1.000								
**BPNSF2**	.289	.410	.347	.462	-.439	-.360	-.359	-.278	1.000							
**BPNSF8**	.386	.506	.435	.443	-.386	-.482	-.349	-.349	.466	1.000						
**BPNSF20**	.335	.440	.532	.576	-.445	-.498	-.401	-.390	.441	.496	1.000					
**BPNSF22**	.235	.384	.312	.444	-.380	-.366	-.320	-.374	.372	.483	.454	1.000				
**BPNSF4**	-.327	-.353	-.303	-.327	.445	.267	.191	.254	-.268	-.346	-.352	-.376	1.000			
**BPNSF12**	-.326	-.409	-.421	-.465	.305	.464	.476	.434	-.366	-.382	-.423	-.450	.377	1.000		
**BPNSF16**	-.305	-.423	-.438	-.403	.211	.332	.420	.399	-.329	-.376	-.462	-.430	.355	.591	1.000	
**BPNSF24**	-.342	-.437	-.344	-.430	.195	.311	.335	.447	-.374	-.418	-.410	-.360	.278	.473	.410	1.0[illegible]
**BPNSF5**	.285	.374	.265	.302	-.244	-.286	-.247	-.221	.403	.377	.273	.305	-.287	-.308	-.320	-.2[illegible]
**BPNSF10**	.357	.538	.409	.407	-.372	-.453	-.320	-.310	.382	.473	.368	.349	-.330	-.322	-.318	-.2[illegible]
**BPNSF15**	.143	.240	.235	.141	-.217	-.239	-.222	-.191	.185	.281	.179	.204	-.280	-.187	-.221	-.2[illegible]
**BPNSF18**	-.030	.031	.057	-.036	-.120	-.005	.040	-.025	-.019	.003	.007	.072	-.156	.004	-.039	.0[illegible]
**BPNSF1**	-.222	-.258	-.233	-.223	.327	.205	.133	.177	-.160	-.248	-.243	-.331	.293	.211	.151	.1[illegible]
**BPNSF7**	-.151	-.314	-.264	-.275	.140	.251	.281	.332	-.191	-.193	-.219	-.278	.153	.310	.248	.1[illegible]
**BPNSF13**	-.279	-.383	-.348	-.298	.189	.382	.415	.360	-.253	-.305	-.319	-.377	.295	.559	.431	.3[illegible]
**BPNSF19**	-.292	-.361	-.307	-.363	.227	.332	.339	.517	-.210	-.289	-.300	-.289	.272	.313	.348	.3[illegible]

Sample Correlations - Upper Bounds (PC) (g3 - Measurement weights)

	BPNSF6	BPNSF11	BPNSF17	BPNSF23	BPNSF3	BPNSF9	BPNSF14	BPNSF21	BPNSF2	BPNSF8	BPNSF20	BPNSF22	BPNSF4	BPNSF12	BPNSF16	BPNS[illegible]
**BPNSF6**	1.000															
**BPNSF11**	.627	1.000														
**BPNSF17**	.579	.644	1.000													
**BPNSF23**	.577	.652	.680	1.000												
**BPNSF3**	-.258	-.268	-.304	-.333	1.000											
**BPNSF9**	-.333	-.438	-.443	-.380	.613	1.000										
**BPNSF14**	-.221	-.321	-.341	-.346	.529	.682	1.000									
**BPNSF21**	-.189	-.314	-.192	-.332	.475	.562	.595	1.000								
**BPNSF2**	.452	.552	.514	.602	-.263	-.201	-.190	-.116	1.000							
**BPNSF8**	.525	.645	.582	.584	-.213	-.324	-.184	-.177	.617	1.000						
**BPNSF20**	.495	.583	.661	.706	-.279	-.337	-.242	-.208	.590	.640	1.000					
**BPNSF22**	.410	.519	.484	.584	-.205	-.174	-.125	-.195	.506	.617	.601	1.000				
**BPNSF4**	-.156	-.175	-.130	-.184	.626	.442	.370	.450	-.083	-.167	-.175	-.188	1.000			
**BPNSF12**	-.175	-.249	-.254	-.299	.470	.603	.630	.569	-.202	-.198	-.251	-.284	.537	1.000		
**BPNSF16**	-.132	-.262	-.271	-.255	.413	.531	.575	.576	-.173	-.174	-.284	-.256	.522	.721	1.000	
**BPNSF24**	-.192	-.290	-.179	-.283	.387	.484	.498	.594	-.205	-.241	-.256	-.173	.475	.613	.572	1.0[illegible]
**BPNSF5**	.435	.497	.409	.428	-.065	-.138	-.084	-.051	.550	.511	.431	.465	-.116	-.155	-.166	-.1[illegible]
**BPNSF10**	.541	.658	.567	.542	-.190	-.296	-.159	-.145	.541	.616	.517	.508	-.146	-.167	-.163	-.1[illegible]
**BPNSF15**	.332	.382	.405	.299	-.058	-.085	-.052	-.003	.329	.436	.339	.368	-.127	-.012	-.057	-.0[illegible]
**BPNSF18**	.132	.194	.231	.116	.042	.160	.214	.138	.152	.186	.180	.243	.011	.185	.145	.2[illegible]
**BPNSF1**	-.047	-.095	-.051	-.067	.497	.378	.315	.377	.008	-.066	-.071	-.154	.468	.384	.337	.3[illegible]
**BPNSF7**	.024	-.152	-.089	-.121	.364	.442	.468	.501	-.013	-.005	-.061	-.083	.346	.473	.429	.3[illegible]
**BPNSF13**	-.094	-.214	-.183	-.137	.387	.534	.587	.521	-.090	-.096	-.136	-.205	.457	.678	.584	.5[illegible]
**BPNSF19**	-.139	-.222	-.145	-.225	.393	.494	.522	.639	-.058	-.117	-.124	-.126	.421	.486	.502	.4[illegible]

Sample Correlations - Two Tailed Significance (PC) (g3 - Measurement weights)

	BPNSF6	BPNSF11	BPNSF17	BPNSF23	BPNSF3	BPNSF9	BPNSF14	BPNSF21	BPNSF2	BPNSF8	BPNSF20	BPNSF22	BPNSF4	BPNSF12	BPNSF16	BPNS[illegible]
**BPNSF6**	...															
**BPNSF11**	.010	...														
**BPNSF17**	.010	.010	...													
**BPNSF23**	.010	.010	.010	...												
**BPNSF3**	.010	.010	.010	.010	...											
**BPNSF9**	.010	.010	.010	.010	.010	...										
**BPNSF14**	.010	.010	.010	.010	.010	.010	...									
**BPNSF21**	.010	.010	.010	.010	.010	.010	.010	...								
**BPNSF2**	.010	.010	.010	.010	.010	.010	.010	.010	...							
**BPNSF8**	.010	.010	.010	.010	.010	.010	.010	.010	.010	...						
**BPNSF20**	.010	.010	.010	.010	.010	.010	.010	.010	.010	.010	...					
**BPNSF22**	.010	.010	.010	.010	.010	.010	.010	.010	.010	.010	.010	...				
**BPNSF4**	.010	.010	.010	.010	.010	.010	.010	.010	.010	.010	.010	.010	...			

	BPNSF6	BPNSF11	BPNSF17	BPNSF23	BPNSF3	BPNSF9	BPNSF14	BPNSF21	BPNSF2	BPNSF8	BPNSF20	BPNSF22	BPNSF4	BPNSF12	BPNSF16	BPNS[illegible]
**BPNSF12**	.010	.010	.010	.010	.010	.010	.010	.010	.010	.010	.010	.010	.010	...		
**BPNSF16**	.010	.010	.010	.010	.010	.010	.010	.010	.010	.010	.010	.010	.010	.010	...	
**BPNSF24**	.010	.010	.010	.010	.010	.010	.010	.010	.010	.010	.010	.010	.010	.010	.010	
**BPNSF5**	.010	.010	.010	.010	.010	.010	.010	.010	.010	.010	.010	.010	.010	.010	.010	.0[illegible]
**BPNSF10**	.010	.010	.010	.010	.010	.010	.010	.010	.010	.010	.010	.010	.010	.010	.010	.0[illegible]
**BPNSF15**	.010	.010	.010	.010	.010	.010	.016	.097	.010	.010	.010	.010	.010	.066	.014	.0[illegible]
**BPNSF18**	.373	.022	.010	.387	.431	.135	.010	.336	.197	.090	.074	.012	.149	.095	.281	.0[illegible]
**BPNSF1**	.019	.010	.010	.010	.010	.010	.010	.010	.175	.010	.010	.010	.010	.010	.010	.0[illegible]
**BPNSF7**	.178	.010	.010	.010	.010	.010	.010	.010	.062	.094	.010	.010	.010	.010	.010	.0[illegible]
**BPNSF13**	.010	.010	.010	.010	.010	.010	.010	.010	.010	.010	.010	.010	.010	.010	.010	.0[illegible]
**BPNSF19**	.010	.010	.010	.010	.010	.010	.010	.010	.016	.010	.010	.010	.010	.010	.010	.0[illegible]

Sample Means (g3 - Measurement weights)

Sample Means - Lower Bounds (PC) (g3 - Measurement weights)

	BPNSF6	BPNSF11	BPNSF17	BPNSF23	BPNSF3	BPNSF9	BPNSF14	BPNSF21	BPNSF2	BPNSF8	BPNSF20	BPNSF22	BPNSF4	BPNSF12	BPNSF16	BPNSF[illegible]
**BPNSF6**	2.512	2.681	2.684	2.387	5.371	5.333	5.052	4.893	2.510	2.777	2.612	3.146	5.091	4.998	4.900	5.00[illegible]

Sample Means - Upper Bounds (PC) (g3 - Measurement weights)

	BPNSF6	BPNSF11	BPNSF17	BPNSF23	BPNSF3	BPNSF9	BPNSF14	BPNSF21	BPNSF2	BPNSF8	BPNSF20	BPNSF22	BPNSF4	BPNSF12	BPNSF16	BPNSF[illegible]
**BPNSF6**	2.774	2.931	2.973	2.629	5.613	5.584	5.294	5.097	2.809	3.057	2.898	3.394	5.299	5.234	5.137	5.23[illegible]

Sample Means - Two Tailed Significance (PC) (g3 - Measurement weights)

	BPNSF6	BPNSF11	BPNSF17	BPNSF23	BPNSF3	BPNSF9	BPNSF14	BPNSF21	BPNSF2	BPNSF8	BPNSF20	BPNSF22	BPNSF4	BPNSF12	BPNSF16	BPNSF[illegible]
**BPNSF6**	.010	.010	.010	.010	.010	.010	.010	.010	.010	.010	.010	.010	.010	.010	.010	.01[illegible]

Bias-corrected percentile method (g3 - Measurement weights)

90% confidence intervals (bias-corrected percentile method)

Scalar Estimates (g3 - Measurement weights)

Regression Weights: (g3 - Measurement weights)

Parameter			Estimate	Lower	Upper	P
BPNSF19	<---	F1	1.000	1.000	1.000	...
BPNSF13	<---	F1	1.146	1.055	1.257	.005
BPNSF7	<---	F1	.872	.767	.971	.010
BPNSF1	<---	F1	.774	.649	.884	.014
BPNSF18	<---	F2	1.000	1.000	1.000	...
BPNSF15	<---	F2	2.522	2.055	3.469	.007
BPNSF10	<---	F2	3.235	2.585	4.436	.009
BPNSF5	<---	F2	2.579	2.065	3.564	.008
BPNSF24	<---	F3	1.000	1.000	1.000	...
BPNSF16	<---	F3	1.125	1.037	1.216	.011
BPNSF12	<---	F3	1.221	1.145	1.303	.006
BPNSF4	<---	F3	.861	.766	.946	.011
BPNSF22	<---	F4	1.000	1.000	1.000	...
BPNSF20	<---	F4	1.188	1.115	1.297	.004
BPNSF8	<---	F4	1.203	1.127	1.302	.005
BPNSF2	<---	F4	1.115	1.043	1.226	.004
BPNSF21	<---	F5	1.000	1.000	1.000	...
BPNSF14	<---	F5	1.090	1.028	1.194	.006
BPNSF9	<---	F5	1.038	.967	1.139	.007
BPNSF3	<---	F5	.889	.795	.992	.013
BPNSF23	<---	F6	1.000	1.000	1.000	...
BPNSF17	<---	F6	.977	.910	1.028	.023
BPNSF11	<---	F6	.972	.916	1.036	.009
BPNSF6	<---	F6	.870	.811	.938	.009

Standardized Regression Weights: (g3 - Measurement weights)

Parameter			Estimate	Lower	Upper	P
BPNSF19	<---	F1	.640	.571	.683	.021
BPNSF13	<---	F1	.723	.678	.769	.005

Parameter			Estimate	Lower	Upper	P
BPNSF7	<---	F1	.546	.481	.611	.008
BPNSF1	<---	F1	.448	.389	.517	.008
BPNSF18	<---	F2	.265	.198	.326	.015
BPNSF15	<---	F2	.563	.508	.608	.010
BPNSF10	<---	F2	.771	.717	.827	.009
BPNSF5	<---	F2	.612	.563	.684	.004
BPNSF24	<---	F3	.677	.636	.736	.007
BPNSF16	<---	F3	.764	.701	.807	.021
BPNSF12	<---	F3	.819	.781	.852	.013
BPNSF4	<---	F3	.606	.548	.674	.007
BPNSF22	<---	F4	.693	.649	.735	.013
BPNSF20	<---	F4	.750	.697	.798	.012
BPNSF8	<---	F4	.768	.717	.821	.007
BPNSF2	<---	F4	.692	.642	.748	.008
BPNSF21	<---	F5	.713	.644	.756	.039
BPNSF14	<---	F5	.741	.674	.793	.023
BPNSF9	<---	F5	.736	.688	.795	.008
BPNSF3	<---	F5	.606	.523	.674	.020
BPNSF23	<---	F6	.786	.756	.830	.005
BPNSF17	<---	F6	.752	.707	.798	.014
BPNSF11	<---	F6	.785	.741	.825	.008
BPNSF6	<---	F6	.672	.621	.732	.006

Intercepts: (g3 - Measurement weights)

Parameter	Estimate	Lower	Upper	P
**BPNSF19**	4.926	4.800	5.057	.010
**BPNSF13**	4.783	4.686	4.891	.005
**BPNSF7**	4.717	4.584	4.828	.012
**BPNSF1**	4.672	4.526	4.791	.014
**BPNSF18**	4.474	4.362	4.596	.012
**BPNSF15**	3.730	3.601	3.846	.014
**BPNSF10**	3.118	2.981	3.240	.012
**BPNSF5**	3.901	3.786	4.070	.004
**BPNSF24**	5.117	4.982	5.215	.014
**BPNSF16**	5.019	4.898	5.117	.018
**BPNSF12**	5.107	4.983	5.207	.021
**BPNSF4**	5.204	5.103	5.307	.006
**BPNSF22**	3.274	3.122	3.385	.015
**BPNSF20**	2.762	2.639	2.912	.005
**BPNSF8**	2.916	2.767	3.047	.019
**BPNSF2**	2.644	2.511	2.820	.009
**BPNSF21**	4.999	4.869	5.078	.021
**BPNSF14**	5.183	5.067	5.299	.007
**BPNSF9**	5.461	5.320	5.569	.020
**BPNSF3**	5.491	5.381	5.626	.007
**BPNSF23**	2.503	2.397	2.650	.006
**BPNSF17**	2.828	2.698	3.010	.005
**BPNSF11**	2.806	2.672	2.910	.016
**BPNSF6**	2.632	2.488	2.760	.019

Covariances: (g3 - Measurement weights)

Parameter			Estimate	Lower	Upper	P
F1	<-->	F2	-.167	-.233	-.122	.005
F2	<-->	F3	-.150	-.208	-.111	.004
F1	<-->	F3	.765	.663	.878	.006
F2	<-->	F4	.365	.269	.480	.009
F3	<-->	F4	-.584	-.703	-.463	.013
F1	<-->	F4	-.403	-.516	-.298	.009
F2	<-->	F5	-.174	-.252	-.137	.003
F4	<-->	F5	-.659	-.796	-.560	.005
F3	<-->	F5	.823	.719	.994	.005
F1	<-->	F5	.827	.687	.981	.008
F6	<-->	F5	-1.046	-1.193	-.895	.012
F6	<-->	F3	-.723	-.847	-.572	.012
F6	<-->	F4	1.387	1.204	1.540	.013
F6	<-->	F2	.434	.319	.558	.008
F6	<-->	F1	-.584	-.692	-.426	.021

Correlations: (g3 - Measurement weights)

Parameter	Estimate	Lower	Upper	P
F1 <--> F2	-.474	-.579	-.366	.008
F2 <--> F3	-.405	-.497	-.316	.011
F1 <--> F3	.898	.840	.956	.006
F2 <--> F4	.836	.769	.893	.009
F3 <--> F4	-.553	-.646	-.455	.013
F1 <--> F4	-.401	-.505	-.297	.014
F2 <--> F5	-.440	-.529	-.326	.009
F4 <--> F5	-.584	-.678	-.502	.007
F3 <--> F5	.861	.795	.936	.006
F1 <--> F5	.907	.828	.982	.009
F6 <--> F5	-.757	-.825	-.704	.011
F6 <--> F3	-.560	-.629	-.459	.015
F6 <--> F4	.909	.852	.958	.007
F6 <--> F2	.812	.732	.887	.009
F6 <--> F1	-.474	-.552	-.391	.016

Variances: (g3 - Measurement weights)

Parameter	Estimate	Lower	Upper	P
**F1**	.812	.661	.972	.012
**F2**	.153	.084	.235	.012
**F3**	.894	.784	1.040	.004
**F4**	1.247	1.056	1.407	.012
**F5**	1.022	.829	1.183	.020
**F6**	1.869	1.608	2.083	.013
**e1**	1.172	.945	1.379	.012
**e2**	.977	.798	1.153	.011
**e3**	1.456	1.203	1.786	.005
**e4**	1.938	1.679	2.232	.007
**e5**	2.018	1.810	2.313	.003
**e6**	2.102	1.854	2.373	.007
**e7**	1.092	.851	1.369	.007
**e8**	1.701	1.459	1.970	.006
**e9**	1.057	.838	1.273	.008
**e10**	.805	.630	1.020	.005
**e11**	.652	.535	.798	.005
**e12**	1.138	.926	1.475	.008
**e13**	1.347	1.138	1.565	.006
**e14**	1.371	1.083	1.701	.005
**e15**	1.255	.969	1.474	.020
**e16**	1.688	1.367	2.067	.009
**e17**	.986	.791	1.228	.004
**e18**	.999	.788	1.273	.005
**e19**	.931	.714	1.107	.007
**e20**	1.391	1.076	1.808	.006
**e21**	1.158	.931	1.361	.011
**e22**	1.370	1.067	1.613	.010
**e23**	1.103	.875	1.321	.014
**e24**	1.719	1.306	2.066	.014

Matrices (g3 - Measurement weights)

Sample Covariances (g3 - Measurement weights)

Sample Covariances - Lower Bounds (BC) (g3 - Measurement weights)

	BPNSF6	BPNSF11	BPNSF17	BPNSF23	BPNSF3	BPNSF9	BPNSF14	BPNSF21	BPNSF2	BPNSF8	BPNSF20	BPNSF22	BPNSF4	BPNSF12	BPNSF16	BPNS[illegible]
**BPNSF6**	2.719															
**BPNSF11**	1.383	2.681														
**BPNSF17**	1.316	1.451	2.933													
**BPNSF23**	1.313	1.523	1.661	2.755												
**BPNSF3**	-1.124	-1.094	-1.233	-1.263	1.765											
**BPNSF9**	-1.290	-1.469	-1.622	-1.362	.893	1.844										
**BPNSF14**	-.942	-1.200	-1.418	-1.268	.773	1.118	1.983									
**BPNSF21**	-.844	-1.167	-.933	-1.143	.570	.729	.799	1.700								
**BPNSF2**	.910	1.248	1.130	1.396	-1.194	-.935	-.953	-.664	2.935							
**BPNSF8**	1.220	1.534	1.364	1.411	-1.051	-1.306	-.914	-.937	1.434	2.883						
**BPNSF20**	1.017	1.296	1.638	1.695	-1.137	-1.327	-1.089	-.926	1.341	1.506	2.845					
**BPNSF22**	.664	.991	.860	1.236	-.886	-.906	-.765	-.858	1.036	1.306	1.225	2.252				
**BPNSF4**	-.757	-.749	-.708	-.790	.816	.524	.377	.463	-.625	-.837	-.830	-.774	1.514			

	BPNSF6	BPNSF11	BPNSF17	BPNSF23	BPNSF3	BPNSF9	BPNSF14	BPNSF21	BPNSF2	BPNSF8	BPNSF20	BPNSF22	BPNSF4	BPNSF12	BPNSF16	BPNS[illegible]
**BPNSF12**	-.914	-1.017	-1.125	-1.185	.642	.973	1.027	.858	-.964	-.966	-1.085	-1.027	.727	1.900		
**BPNSF16**	-.728	-1.013	-1.073	-.986	.487	.683	.884	.736	-.822	-.922	-1.167	-.963	.633	1.153	1.739	
**BPNSF24**	-.852	-1.004	-.821	-1.042	.370	.608	.663	.803	-.899	-1.030	-.977	-.780	.523	.924	.778	[illegible]
**BPNSF5**	.815	1.065	.784	.877	-.591	-.749	-.683	-.517	1.226	1.090	.832	.743	-.674	-.794	-.769	[illegible]
**BPNSF10**	1.019	1.503	1.221	1.176	-.882	-1.131	-.766	-.703	1.116	1.416	1.067	.908	-.696	-.768	-.719	[illegible]
**BPNSF15**	.453	.685	.732	.448	-.553	-.624	-.584	-.549	.590	.795	.524	.525	-.629	-.492	-.539	[illegible]
**BPNSF18**	-.060	.114	.218	-.054	-.319	-.012	.079	-.102	-.017	-.004	.067	.177	-.295	-.020	-.110	[illegible]
**BPNSF1**	-.568	-.643	-.672	-.604	.708	.509	.305	.380	-.484	-.774	-.616	-.854	.593	.473	.336	[illegible]
**BPNSF7**	-.367	-.741	-.668	-.713	.302	.515	.611	.637	-.498	-.493	-.535	-.670	.290	.659	.473	[illegible]
**BPNSF13**	-.697	-.911	-.932	-.742	.420	.811	.868	.747	-.648	-.788	-.808	-.897	.574	1.109	.843	[illegible]
**BPNSF19**	-.661	-.859	-.740	-.872	.462	.670	.709	.966	-.490	-.696	-.707	-.640	.465	.612	.701	[illegible]

Sample Covariances - Upper Bounds (BC) (g3 - Measurement weights)

	BPNSF6	BPNSF11	BPNSF17	BPNSF23	BPNSF3	BPNSF9	BPNSF14	BPNSF21	BPNSF2	BPNSF8	BPNSF20	BPNSF22	BPNSF4	BPNSF12	BPNSF16	BPNS[illegible]
**BPNSF6**	3.289															
**BPNSF11**	1.900	3.183														
**BPNSF17**	1.833	2.009	3.462													
**BPNSF23**	1.818	1.990	2.131	3.261												
**BPNSF3**	-.688	-.683	-.837	-.827	2.431											
**BPNSF9**	-.838	-1.073	-1.150	-.901	1.357	2.456										
**BPNSF14**	-.540	-.821	-.930	-.874	1.188	1.577	2.454									
**BPNSF21**	-.472	-.705	-.488	-.769	.973	1.117	1.182	2.181								
**BPNSF2**	1.453	1.750	1.679	1.864	-.747	-.543	-.505	-.317	3.531							
**BPNSF8**	1.711	2.046	1.914	1.878	-.589	-.805	-.498	-.473	2.002	3.426						
**BPNSF20**	1.557	1.781	2.156	2.208	-.706	-.849	-.636	-.471	1.912	2.042	3.446					
**BPNSF22**	1.150	1.440	1.360	1.718	-.460	-.433	-.316	-.466	1.513	1.778	1.716	2.720				
**BPNSF4**	-.385	-.350	-.315	-.435	1.278	.886	.723	.839	-.218	-.446	-.443	-.388	1.945			
**BPNSF12**	-.442	-.612	-.659	-.759	1.043	1.409	1.440	1.200	-.565	-.505	-.629	-.637	1.054	2.368		
**BPNSF16**	-.334	-.588	-.641	-.600	.867	1.113	1.258	1.110	-.455	-.444	-.682	-.559	.949	1.531	2.147	
**BPNSF24**	-.480	-.647	-.410	-.656	.768	.994	1.017	1.174	-.509	-.635	-.549	-.405	.872	1.259	1.107	[illegible]
**BPNSF5**	1.292	1.503	1.258	1.290	-.148	-.372	-.251	-.127	1.716	1.564	1.355	1.232	-.279	-.373	-.414	[illegible]
**BPNSF10**	1.570	1.985	1.697	1.614	-.449	-.705	-.369	-.330	1.635	1.918	1.557	1.354	-.311	-.411	-.333	[illegible]
**BPNSF15**	1.001	1.166	1.270	.918	-.153	-.228	-.176	-.054	1.037	1.283	1.029	.973	-.275	-.091	-.155	[illegible]
**BPNSF18**	.379	.516	.679	.353	.054	.344	.459	.233	.440	.478	.512	.573	.020	.370	.256	[illegible]
**BPNSF1**	-.123	-.224	-.175	-.182	1.181	.923	.760	.804	.013	-.225	-.161	-.385	.989	.879	.759	[illegible]
**BPNSF7**	.076	-.339	-.227	-.322	.763	.924	1.000	1.010	-.038	-.014	-.143	-.201	.652	1.013	.849	[illegible]
**BPNSF13**	-.233	-.528	-.497	-.370	.840	1.196	1.267	1.079	-.232	-.308	-.354	-.464	.891	1.468	1.202	[illegible]
**BPNSF19**	-.277	-.527	-.317	-.540	.794	1.061	1.091	1.314	-.112	-.267	-.263	-.291	.783	.993	1.018	[illegible]

Sample Covariances - Two Tailed Significance (BC) (g3 - Measurement weights)

	BPNSF6	BPNSF11	BPNSF17	BPNSF23	BPNSF3	BPNSF9	BPNSF14	BPNSF21	BPNSF2	BPNSF8	BPNSF20	BPNSF22	BPNSF4	BPNSF12	BPNSF16	BPNS[illegible]
**BPNSF6**	.014															
**BPNSF11**	.007	.007														
**BPNSF17**	.006	.012	.007													
**BPNSF23**	.005	.012	.009	.009												
**BPNSF3**	.006	.009	.008	.019	.015											
**BPNSF9**	.011	.009	.005	.021	.010	.012										
**BPNSF14**	.023	.009	.007	.015	.007	.009	.006									
**BPNSF21**	.008	.009	.009	.011	.012	.025	.015	.004								
**BPNSF2**	.008	.006	.008	.012	.006	.007	.009	.006	.006							
**BPNSF8**	.003	.005	.009	.002	.005	.009	.006	.004	.010	.007						
**BPNSF20**	.005	.012	.008	.012	.018	.011	.008	.020	.010	.009	.005					
**BPNSF22**	.007	.009	.011	.003	.012	.005	.005	.003	.006	.007	.009	.004				
**BPNSF4**	.008	.025	.008	.006	.011	.005	.010	.009	.007	.004	.005	.009	.012			
**BPNSF12**	.003	.006	.008	.006	.005	.003	.004	.004	.005	.007	.012	.006	.007	.002		
**BPNSF16**	.009	.010	.012	.008	.004	.009	.002	.007	.009	.009	.009	.007	.009	.004	.004	
**BPNSF24**	.005	.013	.019	.010	.015	.008	.012	.007	.011	.005	.034	.006	.006	.005	.006	[illegible]
**BPNSF5**	.011	.005	.009	.006	.012	.005	.004	.010	.005	.012	.005	.018	.006	.007	.005	[illegible]
**BPNSF10**	.008	.005	.009	.009	.010	.009	.016	.012	.010	.003	.006	.009	.012	.009	.015	[illegible]
**BPNSF15**	.006	.006	.004	.004	.007	.005	.005	.039	.005	.013	.008	.009	.009	.032	.006	[illegible]
**BPNSF18**	.321	.012	.004	.233	.223	.156	.012	.595	.150	.105	.042	.008	.149	.149	.468	[illegible]
**BPNSF1**	.034	.016	.008	.010	.010	.004	.007	.014	.125	.005	.025	.005	.007	.007	.006	[illegible]
**BPNSF7**	.222	.026	.014	.008	.012	.011	.011	.012	.049	.089	.015	.006	.012	.003	.011	[illegible]
**BPNSF13**	.011	.009	.005	.006	.004	.004	.007	.003	.009	.005	.009	.005	.004	.004	.005	[illegible]
**BPNSF19**	.028	.010	.019	.016	.012	.009	.007	.006	.035	.013	.023	.006	.012	.010	.005	[illegible]

Sample Correlations (g3 - Measurement weights)

Sample Correlations - Lower Bounds (BC) (g3 - Measurement weights)

	BPNSF6	BPNSF11	BPNSF17	BPNSF23	BPNSF3	BPNSF9	BPNSF14	BPNSF21	BPNSF2	BPNSF8	BPNSF20	BPNSF22	BPNSF4	BPNSF12	BPNSF16	BPNS[illegible]
**BPNSF6**	1.000															
**BPNSF11**	.477	1.000														
**BPNSF17**	.403	.492	1.000													
**BPNSF23**	.445	.543	.545	1.000												
**BPNSF3**	-.434	-.445	-.483	-.505	1.000											
**BPNSF9**	-.488	-.567	-.579	-.529	.398	1.000										
**BPNSF14**	-.366	-.467	-.523	-.485	.342	.546	1.000									
**BPNSF21**	-.346	-.469	-.371	-.481	.272	.364	.374	1.000								
**BPNSF2**	.292	.412	.343	.470	-.431	-.350	-.357	-.285	1.000							
**BPNSF8**	.400	.509	.443	.474	-.392	-.485	-.352	-.373	.463	1.000						
**BPNSF20**	.340	.439	.529	.561	-.431	-.489	-.401	-.368	.441	.496	1.000					
**BPNSF22**	.237	.393	.312	.447	-.376	-.367	-.340	-.387	.372	.486	.454	1.000				
**BPNSF4**	-.327	-.338	-.303	-.343	.436	.270	.200	.256	-.269	-.352	-.355	-.378	1.000			
**BPNSF12**	-.327	-.410	-.424	-.466	.308	.464	.477	.436	-.361	-.382	-.404	-.459	.377	1.000		
**BPNSF16**	-.304	-.421	-.423	-.408	.224	.330	.425	.396	-.331	-.360	-.454	-.438	.356	.593	1.000	
**BPNSF24**	-.355	-.429	-.342	-.429	.180	.314	.323	.446	-.370	-.423	-.397	-.366	.292	.474	.408	1.0[illegible]
**BPNSF5**	.279	.381	.258	.306	-.239	-.295	-.265	-.227	.401	.375	.279	.298	-.287	-.308	-.326	-.2[illegible]
**BPNSF10**	.352	.538	.408	.407	-.346	-.454	-.305	-.307	.382	.483	.369	.349	-.324	-.321	-.317	-.2[illegible]
**BPNSF15**	.148	.240	.248	.154	-.221	-.240	-.231	-.232	.195	.284	.181	.202	-.279	-.195	-.225	-.2[illegible]
**BPNSF18**	-.025	.046	.083	-.028	-.137	-.012	.030	-.055	-.005	-.004	.011	.074	-.155	-.013	-.053	.0[illegible]
**BPNSF1**	-.206	-.243	-.233	-.219	.332	.205	.134	.171	-.166	-.267	-.220	-.343	.296	.214	.150	.1[illegible]
**BPNSF7**	-.149	-.307	-.252	-.274	.121	.240	.284	.313	-.193	-.201	-.218	-.291	.150	.316	.231	.1[illegible]
**BPNSF13**	-.282	-.386	-.350	-.315	.207	.389	.407	.367	-.254	-.310	-.305	-.388	.309	.563	.434	.3[illegible]
**BPNSF19**	-.280	-.357	-.295	-.359	.219	.332	.329	.517	-.200	-.285	-.288	-.303	.272	.309	.337	.3[illegible]

Sample Correlations - Upper Bounds (BC) (g3 - Measurement weights)

	BPNSF6	BPNSF11	BPNSF17	BPNSF23	BPNSF3	BPNSF9	BPNSF14	BPNSF21	BPNSF2	BPNSF8	BPNSF20	BPNSF22	BPNSF4	BPNSF12	BPNSF16	BPNS[illegible]
**BPNSF6**	1.000															
**BPNSF11**	.629	1.000														
**BPNSF17**	.569	.647	1.000													
**BPNSF23**	.592	.652	.674	1.000												
**BPNSF3**	-.258	-.270	-.308	-.324	1.000											
**BPNSF9**	-.322	-.433	-.432	-.375	.596	1.000										
**BPNSF14**	-.205	-.316	-.340	-.343	.518	.686	1.000									
**BPNSF21**	-.182	-.306	-.192	-.331	.460	.553	.561	1.000								
**BPNSF2**	.458	.559	.512	.605	-.252	-.195	-.186	-.121	1.000							
**BPNSF8**	.544	.645	.583	.613	-.224	-.324	-.185	-.185	.615	1.000						
**BPNSF20**	.498	.580	.651	.695	-.262	-.324	-.242	-.176	.593	.642	1.000					
**BPNSF22**	.413	.530	.484	.588	-.204	-.174	-.140	-.208	.506	.621	.601	1.000				
**BPNSF4**	-.158	-.158	-.130	-.192	.616	.444	.378	.454	-.086	-.184	-.184	-.188	1.000			
**BPNSF12**	-.177	-.249	-.256	-.299	.473	.601	.631	.571	-.198	-.198	-.245	-.292	.537	1.000		
**BPNSF16**	-.132	-.259	-.255	-.257	.418	.529	.582	.569	-.176	-.172	-.282	-.262	.523	.722	1.000	
**BPNSF24**	-.208	-.278	-.168	-.281	.372	.487	.492	.593	-.203	-.256	-.228	-.188	.488	.615	.569	1.0[illegible]
**BPNSF5**	.433	.509	.398	.436	-.058	-.147	-.102	-.054	.544	.509	.440	.460	-.116	-.156	-.173	-.1[illegible]
**BPNSF10**	.537	.658	.561	.541	-.158	-.296	-.135	-.131	.533	.619	.518	.508	-.135	-.167	-.158	-.1[illegible]
**BPNSF15**	.337	.382	.416	.316	-.059	-.085	-.073	-.023	.344	.436	.353	.357	-.123	-.037	-.061	-.0[illegible]
**BPNSF18**	.136	.210	.244	.131	.029	.152	.212	.113	.175	.179	.188	.244	.012	.165	.127	.2[illegible]
**BPNSF1**	-.037	-.086	-.051	-.066	.501	.378	.325	.366	.006	-.076	-.056	-.162	.470	.390	.337	.3[illegible]
**BPNSF7**	.029	-.139	-.082	-.120	.353	.433	.469	.480	-.015	-.006	-.057	-.086	.344	.482	.424	.3[illegible]
**BPNSF13**	-.100	-.216	-.187	-.147	.401	.543	.579	.527	-.091	-.110	-.117	-.210	.459	.683	.593	.5[illegible]
**BPNSF19**	-.114	-.220	-.120	-.214	.384	.493	.510	.639	-.044	-.109	-.098	-.137	.421	.474	.497	.4[illegible]

Sample Correlations - Two Tailed Significance (BC) (g3 - Measurement weights)

	BPNSF6	BPNSF11	BPNSF17	BPNSF23	BPNSF3	BPNSF9	BPNSF14	BPNSF21	BPNSF2	BPNSF8	BPNSF20	BPNSF22	BPNSF4	BPNSF12	BPNSF16	BPNS[illegible]
**BPNSF6**	...															
**BPNSF11**	.007	...														
**BPNSF17**	.018	.009	...													
**BPNSF23**	.006	.010	.014	...												
**BPNSF3**	.009	.009	.007	.015	...											
**BPNSF9**	.018	.014	.019	.016	.023	...										
**BPNSF14**	.025	.012	.012	.020	.016	.009	...									
**BPNSF21**	.015	.018	.010	.013	.019	.021	.025	...								
**BPNSF2**	.007	.007	.014	.007	.013	.014	.012	.007	...							
**BPNSF8**	.003	.009	.008	.002	.006	.009	.009	.006	.012	...						
**BPNSF20**	.007	.012	.016	.016	.023	.023	.010	.032	.009	.009	...					
**BPNSF22**	.009	.005	.010	.007	.011	.009	.004	.005	.010	.007	.011	...				
**BPNSF4**	.009	.021	.010	.006	.014	.007	.008	.009	.008	.005	.006	.009	...			

	BPNSF6	BPNSF11	BPNSF17	BPNSF23	BPNSF3	BPNSF9	BPNSF14	BPNSF21	BPNSF2	BPNSF8	BPNSF20	BPNSF22	BPNSF4	BPNSF12	BPNSF16	BPNS
**BPNSF12**	.009	.009	.009	.009	.007	.012	.009	.008	.013	.010	.020	.006	.010	...		
**BPNSF16**	.011	.011	.020	.008	.007	.012	.007	.012	.009	.013	.012	.006	.009	.009	...	
**BPNSF24**	.003	.015	.013	.012	.018	.008	.014	.011	.012	.007	.034	.007	.007	.008	.013	
**BPNSF5**	.012	.004	.016	.006	.014	.006	.004	.008	.012	.013	.007	.014	.010	.009	.005	.0
**BPNSF10**	.015	.010	.012	.012	.021	.009	.026	.013	.012	.005	.009	.010	.016	.012	.012	.0
**BPNSF15**	.008	.010	.005	.005	.009	.009	.005	.042	.005	.009	.009	.012	.011	.037	.010	.0
**BPNSF18**	.334	.012	.005	.264	.254	.214	.015	.612	.142	.116	.056	.010	.157	.188	.468	.0
**BPNSF1**	.038	.014	.010	.012	.008	.010	.007	.018	.138	.007	.028	.005	.008	.007	.011	.0
**BPNSF7**	.213	.021	.021	.012	.019	.015	.009	.030	.049	.084	.012	.007	.012	.006	.015	.0
**BPNSF13**	.008	.008	.008	.006	.004	.006	.018	.007	.009	.007	.019	.005	.006	.006	.005	.0
**BPNSF19**	.026	.012	.025	.016	.018	.012	.015	.012	.035	.012	.026	.005	.010	.019	.016	.0

Sample Means (g3 - Measurement weights)

Sample Means - Lower Bounds (BC) (g3 - Measurement weights)

	BPNSF6	BPNSF11	BPNSF17	BPNSF23	BPNSF3	BPNSF9	BPNSF14	BPNSF21	BPNSF2	BPNSF8	BPNSF20	BPNSF22	BPNSF4	BPNSF12	BPNSF16	BPNSF
**BPNSF6**	2.488	2.672	2.698	2.395	5.381	5.325	5.067	4.869	2.511	2.767	2.639	3.128	5.101	4.983	4.896	4.98

Sample Means - Upper Bounds (BC) (g3 - Measurement weights)

	BPNSF6	BPNSF11	BPNSF17	BPNSF23	BPNSF3	BPNSF9	BPNSF14	BPNSF21	BPNSF2	BPNSF8	BPNSF20	BPNSF22	BPNSF4	BPNSF12	BPNSF16	BPNSF
**BPNSF6**	2.760	2.910	3.010	2.644	5.626	5.571	5.299	5.078	2.820	3.048	2.912	3.386	5.308	5.207	5.117	5.21

Sample Means - Two Tailed Significance (BC) (g3 - Measurement weights)

	BPNSF6	BPNSF11	BPNSF17	BPNSF23	BPNSF3	BPNSF9	BPNSF14	BPNSF21	BPNSF2	BPNSF8	BPNSF20	BPNSF22	BPNSF4	BPNSF12	BPNSF16	BPNSF
**BPNSF6**	.019	.016	.005	.006	.007	.019	.007	.021	.009	.019	.005	.014	.006	.021	.018	.01

Minimization History (Measurement weights)

Iteration		Negative eigenvalues	Condition #	Smallest eigenvalue	Diameter	F	NTries	Ratio
**0**	e	32		-9.204	9999.000	104288.606	0	9999.000
**1**	e	33		-1.033	3.814	35236.999	16	.259
**2**	e	44		-.339	3.028	16933.545	6	.532
**3**	e*	28		-.228	1.441	12036.289	5	.862
**4**	e*	13		-.047	.879	9327.639	4	1.014
**5**	e	6		-.022	.750	7855.951	5	.914
**6**	e	3		-.014	1.430	6219.075	6	.898
**7**	e	3		-.013	3.094	4194.774	6	.901
**8**	e*	2		-.007	3.584	2839.827	5	.808
**9**	e	0	560.237		1.197	2439.662	5	.854
**10**	e	0	811.154		.748	2370.908	2	.000
**11**	e	0	939.224		.611	2323.628	1	1.121
**12**	e	0	1869.775		.532	2315.878	1	.987
**13**	e	0	5289.489		.299	2311.231	1	1.137
**14**	e	0	6620.359		.379	2310.352	1	.833
**15**	e	0	14421.284		.104	2309.900	1	1.037
**16**	e	0	15868.064		.065	2309.884	1	1.013
**17**	e	0	16023.886		.003	2309.884	1	1.001
**18**	e	0	16563.092		.000	2309.884	1	1.000

Pairwise Parameter Comparisons (Measurement weights)

Variance-covariance Matrix of Estimates (Measurement weights)

	a1_1	a2_1	a3_1	a4_1	a5_1	a6_1	a7_1	a8_1	a9_1	a10_1	a11_1	a12_1	a13_1	a14_1	a15_1	a16_1	a17_1	a18_1	ccc1_1	ccc2_1
**a1_1**	.003																			
**a2_1**	.001	.003																		
**a3_1**	.001	.001	.003																	
**a4_1**	.000	.000	.000	.102																
**a5_1**	.000	.000	.000	.113	.156															
**a6_1**	.000	.000	.000	.090	.116	.104														
**a7_1**	.000	.000	.000	.000	.000	.000	.002													
**a8_1**	.000	.000	.000	.000	.000	.000	.001	.002												
**a9_1**	.000	.000	.000	.000	.000	.000	.001	.001	.002											
**a10_1**	.000	.000	.000	.000	.000	.000	.000	.000	.000	.002										
**a11_1**	.000	.000	.000	.000	.000	.000	.000	.000	.000	.001	.003									

	a1_1	a2_1	a3_1	a4_1	a5_1	a6_1	a7_1	a8_1	a9_1	a10_1	a11_1	a12_1	a13_1	a14_1	a15_1	a16_1	a17_1	a18_1	ccc1_1	ccc2_1
**a12_1**	.000	.000	.000	.000	.000	.000	.000	.000	.000	.001	.001	.002								
**a13_1**	.000	.000	.000	.000	.000	.000	.000	.000	.000	.000	.000	.000	.002							
**a14_1**	.000	.000	.000	.000	.000	.000	.000	.000	.000	.000	.000	.000	.001	.002						
**a15_1**	.000	.000	.000	.000	.000	.000	.000	.000	.000	.000	.000	.000	.001	.001	.002					
**a16_1**	.000	.000	.000	.000	.000	.000	.000	.000	.000	.000	.000	.000	.000	.000	.000	.001				
**a17_1**	.000	.000	.000	.000	.000	.000	.000	.000	.000	.000	.000	.000	.000	.000	.000	.001	.001			
**a18_1**	.000	.000	.000	.000	.000	.000	.000	.000	.000	.000	.000	.000	.000	.000	.000	.000	.000	.001		
**ccc1_1**	.000	.000	.000	.003	.003	.003	.000	.000	.000	.000	.000	.000	.000	.000	.000	.000	.000	.000	.001	
**ccc2_1**	.000	.000	.000	.003	.004	.004	.000	.000	.000	.000	.000	.000	.000	.000	.000	.000	.000	.000	.000	.001
**ccc3_1**	-.001	-.001	-.001	.000	.000	.000	-.001	-.001	-.001	.000	.000	.000	.000	.000	.000	.000	.000	.000	.000	.000
**ccc4_1**	.000	.000	.000	-.007	-.009	-.007	.000	.000	.000	.000	.000	.000	.000	.000	.000	.000	.000	.000	.000	-.001
**ccc5_1**	.000	.000	.000	.000	.000	.000	.001	.001	.000	.001	.001	.001	.000	.000	.000	.000	.000	.000	.000	.001
**ccc6_1**	.001	.000	.000	.000	.000	.000	.000	.000	.000	.000	.000	.000	.000	.000	.000	.000	.000	.000	.001	.000
**ccc7_1**	.000	.000	.000	.002	.003	.002	.000	.000	.000	.000	.000	.000	.000	.000	.000	.000	.000	.000	.000	.000
**ccc8_1**	.000	.000	.000	.000	.000	.000	.000	.000	.000	.001	.001	.001	.001	.001	.000	.000	.000	.000	.000	.000
**ccc9_1**	.000	.000	.000	.000	.000	.000	-.001	-.001	-.001	.000	.000	.000	-.001	-.001	.000	.000	.000	.000	.000	.000
**ccc10_1**	-.001	-.001	-.001	.000	.000	.000	.000	.000	.000	.000	.000	.000	-.001	-.001	-.001	.000	.000	.000	.000	.000
**ccc11_1**	.000	.000	.000	.000	.000	.000	.000	.000	.000	.000	.000	.000	.001	.001	.001	.000	.000	.000	.001	.000
**ccc12_1**	.000	.000	.000	.000	.000	.000	.001	.001	.000	.000	.000	.000	.000	.000	.000	.000	.000	.000	.000	.001
**ccc13_1**	.000	.000	.000	.000	.000	.000	.000	.000	.000	-.001	-.001	-.001	.000	.000	.000	-.001	-.001	-.001	-.001	-.001
**ccc14_1**	.000	.000	.000	-.010	-.012	-.010	.000	.000	.000	.000	.000	.000	.000	.000	.000	.000	.000	.000	-.001	-.001
**ccc15_1**	.001	.001	.001	.000	.000	.000	.000	.000	.000	.000	.000	.000	.000	.000	.000	.000	.000	.000	.001	.000
**vvv1_1**	-.003	-.002	-.002	.000	.000	.000	.000	.000	.000	.000	.000	.000	.000	.000	.000	.000	.000	.000	-.001	.000
**v1_1**	.000	.000	.000	.000	.000	.000	.000	.000	.000	.000	.000	.000	.000	.000	.000	.000	.000	.000	.000	.000
**v2_1**	.000	.000	.000	.000	.000	.000	.000	.000	.000	.000	.000	.000	.000	.000	.000	.000	.000	.000	.000	.000
**v3_1**	.000	.000	.000	.000	.000	.000	.000	.000	.000	.000	.000	.000	.000	.000	.000	.000	.000	.000	.000	.000
**v4_1**	.000	.000	.000	.000	.000	.000	.000	.000	.000	.000	.000	.000	.000	.000	.000	.000	.000	.000	.000	.000
**vvv2_1**	.000	.000	.000	-.009	-.011	-.009	.000	.000	.000	.000	.000	.000	.000	.000	.000	.000	.000	.000	.000	.000
**v5_1**	.000	.000	.000	.002	.003	.002	.000	.000	.000	.000	.000	.000	.000	.000	.000	.000	.000	.000	.000	.000
**v6_1**	.000	.000	.000	-.003	.000	.000	.000	.000	.000	.000	.000	.000	.000	.000	.000	.000	.000	.000	.000	.000
**v7_1**	.000	.000	.000	.000	-.004	.000	.000	.000	.000	.000	.000	.000	.000	.000	.000	.000	.000	.000	.000	.000
**v8_1**	.000	.000	.000	.000	.000	-.003	.000	.000	.000	.000	.000	.000	.000	.000	.000	.000	.000	.000	.000	.000
**vvv3_1**	.000	.000	.000	.000	.000	.000	-.002	-.002	-.001	.000	.000	.000	.000	.000	.000	.000	.000	.000	.000	-.001
**v9_1**	.000	.000	.000	.000	.000	.000	.000	.000	.000	.000	.000	.000	.000	.000	.000	.000	.000	.000	.000	.000
**v10_1**	.000	.000	.000	.000	.000	.000	.000	.000	.000	.000	.000	.000	.000	.000	.000	.000	.000	.000	.000	.000
**v11_1**	.000	.000	.000	.000	.000	.000	.000	.000	.000	.000	.000	.000	.000	.000	.000	.000	.000	.000	.000	.000
**v12_1**	.000	.000	.000	.000	.000	.000	.000	.000	.000	.000	.000	.000	.000	.000	.000	.000	.000	.000	.000	.000
**vvv4_1**	.000	.000	.000	.000	.000	.000	.000	.000	.000	-.002	-.002	-.002	.000	.000	.000	.000	.000	.000	.000	.000
**v13_1**	.000	.000	.000	.000	.000	.000	.000	.000	.000	.000	.000	.000	.000	.000	.000	.000	.000	.000	.000	.000
**v14_1**	.000	.000	.000	.000	.000	.000	.000	.000	.000	.000	.000	.000	.000	.000	.000	.000	.000	.000	.000	.000
**v15_1**	.000	.000	.000	.000	.000	.000	.000	.000	.000	.000	.000	.000	.000	.000	.000	.000	.000	.000	.000	.000
**v16_1**	.000	.000	.000	.000	.000	.000	.000	.000	.000	.000	.000	.000	.000	.000	.000	.000	.000	.000	.000	.000
**vvv5_1**	.000	.000	.000	.000	.000	.000	.000	.000	.000	.000	.000	.000	-.002	-.002	-.002	.000	.000	.000	.000	.000
**v17_1**	.000	.000	.000	.000	.000	.000	.000	.000	.000	.000	.000	.000	.000	.000	.000	.000	.000	.000	.000	.000
**v18_1**	.000	.000	.000	.000	.000	.000	.000	.000	.000	.000	.000	.000	.000	.000	.000	.000	.000	.000	.000	.000
**v19_1**	.000	.000	.000	.000	.000	.000	.000	.000	.000	.000	.000	.000	.000	.000	.000	.000	.000	.000	.000	.000
**v20_1**	.000	.000	.000	.000	.000	.000	.000	.000	.000	.000	.000	.000	.000	.000	.000	.000	.000	.000	.000	.000
**vvv6_1**	.000	.000	.000	.000	.000	.000	.000	.000	.000	.000	.000	.000	.000	.000	.000	-.002	-.002	-.001	-.001	-.001
**v21_1**	.000	.000	.000	.000	.000	.000	.000	.000	.000	.000	.000	.000	.000	.000	.000	.000	.000	.000	.000	.000
**v22_1**	.000	.000	.000	.000	.000	.000	.000	.000	.000	.000	.000	.000	.000	.000	.000	.000	.000	.000	.000	.000
**v23_1**	.000	.000	.000	.000	.000	.000	.000	.000	.000	.000	.000	.000	.000	.000	.000	.000	.000	.000	.000	.000
**v24_1**	.000	.000	.000	.000	.000	.000	.000	.000	.000	.000	.000	.000	.000	.000	.000	.000	.000	.000	.000	.000
**i1_1**	.000	.000	.000	.000	.000	.000	.000	.000	.000	.000	.000	.000	.000	.000	.000	.000	.000	.000	.000	.000
**i2_1**	.000	.000	.000	.000	.000	.000	.000	.000	.000	.000	.000	.000	.000	.000	.000	.000	.000	.000	.000	.000
**i3_1**	.000	.000	.000	.000	.000	.000	.000	.000	.000	.000	.000	.000	.000	.000	.000	.000	.000	.000	.000	.000
**i4_1**	.000	.000	.000	.000	.000	.000	.000	.000	.000	.000	.000	.000	.000	.000	.000	.000	.000	.000	.000	.000
**i5_1**	.000	.000	.000	.000	.000	.000	.000	.000	.000	.000	.000	.000	.000	.000	.000	.000	.000	.000	.000	.000
**i6_1**	.000	.000	.000	.000	.000	.000	.000	.000	.000	.000	.000	.000	.000	.000	.000	.000	.000	.000	.000	.000
**i7_1**	.000	.000	.000	.000	.000	.000	.000	.000	.000	.000	.000	.000	.000	.000	.000	.000	.000	.000	.000	.000
**i8_1**	.000	.000	.000	.000	.000	.000	.000	.000	.000	.000	.000	.000	.000	.000	.000	.000	.000	.000	.000	.000
**i9_1**	.000	.000	.000	.000	.000	.000	.000	.000	.000	.000	.000	.000	.000	.000	.000	.000	.000	.000	.000	.000
**i10_1**	.000	.000	.000	.000	.000	.000	.000	.000	.000	.000	.000	.000	.000	.000	.000	.000	.000	.000	.000	.000
**i11_1**	.000	.000	.000	.000	.000	.000	.000	.000	.000	.000	.000	.000	.000	.000	.000	.000	.000	.000	.000	.000
**i12_1**	.000	.000	.000	.000	.000	.000	.000	.000	.000	.000	.000	.000	.000	.000	.000	.000	.000	.000	.000	.000
**i13_1**	.000	.000	.000	.000	.000	.000	.000	.000	.000	.000	.000	.000	.000	.000	.000	.000	.000	.000	.000	.000
**i14_1**	.000	.000	.000	.000	.000	.000	.000	.000	.000	.000	.000	.000	.000	.000	.000	.000	.000	.000	.000	.000
**i15_1**	.000	.000	.000	.000	.000	.000	.000	.000	.000	.000	.000	.000	.000	.000	.000	.000	.000	.000	.000	.000
**i16_1**	.000	.000	.000	.000	.000	.000	.000	.000	.000	.000	.000	.000	.000	.000	.000	.000	.000	.000	.000	.000
**i17_1**	.000	.000	.000	.000	.000	.000	.000	.000	.000	.000	.000	.000	.000	.000	.000	.000	.000	.000	.000	.000
**i18_1**	.000	.000	.000	.000	.000	.000	.000	.000	.000	.000	.000	.000	.000	.000	.000	.000	.000	.000	.000	.000
**i19_1**	.000	.000	.000	.000	.000	.000	.000	.000	.000	.000	.000	.000	.000	.000	.000	.000	.000	.000	.000	.000
**i20_1**	.000	.000	.000	.000	.000	.000	.000	.000	.000	.000	.000	.000	.000	.000	.000	.000	.000	.000	.000	.000

	a1_1	a2_1	a3_1	a4_1	a5_1	a6_1	a7_1	a8_1	a9_1	a10_1	a11_1	a12_1	a13_1	a14_1	a15_1	a16_1	a17_1	a18_1	ccc1_1	ccc2_1
i21_1	.000	.000	.000	.000	.000	.000	.000	.000	.000	.000	.000	.000	.000	.000	.000	.000	.000	.000	.000	.000
i22_1	.000	.000	.000	.000	.000	.000	.000	.000	.000	.000	.000	.000	.000	.000	.000	.000	.000	.000	.000	.000
i23_1	.000	.000	.000	.000	.000	.000	.000	.000	.000	.000	.000	.000	.000	.000	.000	.000	.000	.000	.000	.000
i24_1	.000	.000	.000	.000	.000	.000	.000	.000	.000	.000	.000	.000	.000	.000	.000	.000	.000	.000	.000	.000
ccc1_2	.000	.000	.000	.007	.009	.007	.000	.000	.000	.000	.000	.000	.000	.000	.000	.000	.000	.000	.000	.000
ccc2_2	.000	.000	.000	.007	.008	.007	.000	.000	.000	.000	.000	.000	.000	.000	.000	.000	.000	.000	.000	.000
ccc3_2	-.001	-.001	-.001	.000	.000	.000	-.001	-.001	-.001	.000	.000	.000	.000	.000	.000	.000	.000	.000	.000	.000
ccc4_2	.000	.000	.000	-.012	-.016	-.013	.000	.000	.000	.000	.000	.000	.000	.000	.000	.000	.000	.000	.000	.000
ccc5_2	.000	.000	.000	.000	.000	.000	.001	.001	.001	.001	.001	.001	.000	.000	.000	.000	.000	.000	.000	.000
ccc6_2	.001	.001	.001	.000	.000	.000	.000	.000	.000	.001	.001	.001	.000	.000	.000	.000	.000	.000	.000	.000
ccc7_2	.000	.000	.000	.007	.008	.007	.000	.000	.000	.000	.000	.000	.000	.000	.000	.000	.000	.000	.000	.000
ccc8_2	.000	.000	.000	.000	.000	.000	.000	.000	.000	.001	.001	.001	.001	.001	.001	.000	.000	.000	.000	.000
ccc9_2	.000	.000	.000	.000	.000	.000	-.001	-.001	-.001	.000	.000	.000	-.001	-.001	-.001	.000	.000	.000	.000	.000
ccc10_2	-.002	-.001	-.001	.000	.000	.000	.000	.000	.000	.000	.000	.000	-.001	-.001	-.001	.000	.000	.000	.000	.000
ccc11_2	.000	.000	.000	.000	.000	.000	.000	.000	.000	.000	.000	.000	.001	.001	.001	.001	.000	.000	.000	.000
ccc12_2	.000	.000	.000	.000	.000	.000	.001	.001	.001	.000	.000	.000	.000	.000	.000	.000	.000	.000	.000	.000
ccc13_2	.000	.000	.000	.000	.000	.000	.000	.000	.000	-.001	-.001	-.001	.000	.000	.000	-.001	-.001	-.001	.000	.000
ccc14_2	.000	.000	.000	-.013	-.017	-.013	.000	.000	.000	.000	.000	.000	.000	.000	.000	.000	.000	.000	.000	.000
ccc15_2	.001	.001	.001	.000	.000	.000	.000	.000	.000	.000	.000	.000	.000	.000	.000	.000	.000	.000	.000	.000
vvv1_2	-.003	-.002	-.002	.000	.000	.000	.000	.000	.000	.000	.000	.000	.000	.000	.000	.000	.000	.000	.000	.000
v1_2	.000	.000	.000	.000	.000	.000	.000	.000	.000	.000	.000	.000	.000	.000	.000	.000	.000	.000	.000	.000
v2_2	.000	.000	.000	.000	.000	.000	.000	.000	.000	.000	.000	.000	.000	.000	.000	.000	.000	.000	.000	.000
v3_2	.000	.000	.000	.000	.000	.000	.000	.000	.000	.000	.000	.000	.000	.000	.000	.000	.000	.000	.000	.000
v4_2	.000	.000	.000	.000	.000	.000	.000	.000	.000	.000	.000	.000	.000	.000	.000	.000	.000	.000	.000	.000
vvv2_2	.000	.000	.000	-.011	-.014	-.011	.000	.000	.000	.000	.000	.000	.000	.000	.000	.000	.000	.000	.000	.000
v5_2	.000	.000	.000	.002	.003	.002	.000	.000	.000	.000	.000	.000	.000	.000	.000	.000	.000	.000	.000	.000
v6_2	.000	.000	.000	-.002	.000	.000	.000	.000	.000	.000	.000	.000	.000	.000	.000	.000	.000	.000	.000	.000
v7_2	.000	.000	.000	.000	-.003	.000	.000	.000	.000	.000	.000	.000	.000	.000	.000	.000	.000	.000	.000	.000
v8_2	.000	.000	.000	.000	.000	-.002	.000	.000	.000	.000	.000	.000	.000	.000	.000	.000	.000	.000	.000	.000
vvv3_2	.000	.000	.000	.000	.000	.000	-.002	-.003	-.002	.000	.000	.000	.000	.000	.000	.000	.000	.000	.000	.000
v9_2	.000	.000	.000	.000	.000	.000	.000	.000	.000	.000	.000	.000	.000	.000	.000	.000	.000	.000	.000	.000
v10_2	.000	.000	.000	.000	.000	.000	.000	.000	.000	.000	.000	.000	.000	.000	.000	.000	.000	.000	.000	.000
v11_2	.000	.000	.000	.000	.000	.000	.000	.000	.000	.000	.000	.000	.000	.000	.000	.000	.000	.000	.000	.000
v12_2	.000	.000	.000	.000	.000	.000	.000	.000	.000	.000	.000	.000	.000	.000	.000	.000	.000	.000	.000	.000
vvv4_2	.000	.000	.000	.000	.000	.000	.000	.000	.000	-.003	-.003	-.003	.000	.000	.000	.000	.000	.000	.000	.000
v13_2	.000	.000	.000	.000	.000	.000	.000	.000	.000	.000	.000	.000	.000	.000	.000	.000	.000	.000	.000	.000
v14_2	.000	.000	.000	.000	.000	.000	.000	.000	.000	.000	.000	.000	.000	.000	.000	.000	.000	.000	.000	.000
v15_2	.000	.000	.000	.000	.000	.000	.000	.000	.000	.000	.000	.000	.000	.000	.000	.000	.000	.000	.000	.000
v16_2	.000	.000	.000	.000	.000	.000	.000	.000	.000	.000	.000	.000	.000	.000	.000	.000	.000	.000	.000	.000
vvv5_2	.000	.000	.000	.000	.000	.000	.000	.000	.000	.000	.000	.000	-.002	-.002	-.002	.000	.000	.000	.000	.000
v17_2	.000	.000	.000	.000	.000	.000	.000	.000	.000	.000	.000	.000	.000	.000	.000	.000	.000	.000	.000	.000
v18_2	.000	.000	.000	.000	.000	.000	.000	.000	.000	.000	.000	.000	.000	.000	.000	.000	.000	.000	.000	.000
v19_2	.000	.000	.000	.000	.000	.000	.000	.000	.000	.000	.000	.000	.000	.000	.000	.000	.000	.000	.000	.000
v20_2	.000	.000	.000	.000	.000	.000	.000	.000	.000	.000	.000	.000	.000	.000	.000	.000	.000	.000	.000	.000
vvv6_2	.000	.000	.000	.000	.000	.000	.000	.000	.000	.000	.000	.000	.000	.000	.000	-.002	-.002	-.002	.000	.000
v21_2	.000	.000	.000	.000	.000	.000	.000	.000	.000	.000	.000	.000	.000	.000	.000	.000	.000	.000	.000	.000
v22_2	.000	.000	.000	.000	.000	.000	.000	.000	.000	.000	.000	.000	.000	.000	.000	.000	.000	.000	.000	.000
v23_2	.000	.000	.000	.000	.000	.000	.000	.000	.000	.000	.000	.000	.000	.000	.000	.000	.000	.000	.000	.000
v24_2	.000	.000	.000	.000	.000	.000	.000	.000	.000	.000	.000	.000	.000	.000	.000	.000	.000	.000	.000	.000
i1_2	.000	.000	.000	.000	.000	.000	.000	.000	.000	.000	.000	.000	.000	.000	.000	.000	.000	.000	.000	.000
i2_2	.000	.000	.000	.000	.000	.000	.000	.000	.000	.000	.000	.000	.000	.000	.000	.000	.000	.000	.000	.000
i3_2	.000	.000	.000	.000	.000	.000	.000	.000	.000	.000	.000	.000	.000	.000	.000	.000	.000	.000	.000	.000
i4_2	.000	.000	.000	.000	.000	.000	.000	.000	.000	.000	.000	.000	.000	.000	.000	.000	.000	.000	.000	.000
i5_2	.000	.000	.000	.000	.000	.000	.000	.000	.000	.000	.000	.000	.000	.000	.000	.000	.000	.000	.000	.000
i6_2	.000	.000	.000	.000	.000	.000	.000	.000	.000	.000	.000	.000	.000	.000	.000	.000	.000	.000	.000	.000
i7_2	.000	.000	.000	.000	.000	.000	.000	.000	.000	.000	.000	.000	.000	.000	.000	.000	.000	.000	.000	.000
i8_2	.000	.000	.000	.000	.000	.000	.000	.000	.000	.000	.000	.000	.000	.000	.000	.000	.000	.000	.000	.000
i9_2	.000	.000	.000	.000	.000	.000	.000	.000	.000	.000	.000	.000	.000	.000	.000	.000	.000	.000	.000	.000
i10_2	.000	.000	.000	.000	.000	.000	.000	.000	.000	.000	.000	.000	.000	.000	.000	.000	.000	.000	.000	.000
i11_2	.000	.000	.000	.000	.000	.000	.000	.000	.000	.000	.000	.000	.000	.000	.000	.000	.000	.000	.000	.000
i12_2	.000	.000	.000	.000	.000	.000	.000	.000	.000	.000	.000	.000	.000	.000	.000	.000	.000	.000	.000	.000
i13_2	.000	.000	.000	.000	.000	.000	.000	.000	.000	.000	.000	.000	.000	.000	.000	.000	.000	.000	.000	.000
i14_2	.000	.000	.000	.000	.000	.000	.000	.000	.000	.000	.000	.000	.000	.000	.000	.000	.000	.000	.000	.000
i15_2	.000	.000	.000	.000	.000	.000	.000	.000	.000	.000	.000	.000	.000	.000	.000	.000	.000	.000	.000	.000
i16_2	.000	.000	.000	.000	.000	.000	.000	.000	.000	.000	.000	.000	.000	.000	.000	.000	.000	.000	.000	.000
i17_2	.000	.000	.000	.000	.000	.000	.000	.000	.000	.000	.000	.000	.000	.000	.000	.000	.000	.000	.000	.000
i18_2	.000	.000	.000	.000	.000	.000	.000	.000	.000	.000	.000	.000	.000	.000	.000	.000	.000	.000	.000	.000
i19_2	.000	.000	.000	.000	.000	.000	.000	.000	.000	.000	.000	.000	.000	.000	.000	.000	.000	.000	.000	.000
i20_2	.000	.000	.000	.000	.000	.000	.000	.000	.000	.000	.000	.000	.000	.000	.000	.000	.000	.000	.000	.000
i21_2	.000	.000	.000	.000	.000	.000	.000	.000	.000	.000	.000	.000	.000	.000	.000	.000	.000	.000	.000	.000
i22_2	.000	.000	.000	.000	.000	.000	.000	.000	.000	.000	.000	.000	.000	.000	.000	.000	.000	.000	.000	.000
i23_2	.000	.000	.000	.000	.000	.000	.000	.000	.000	.000	.000	.000	.000	.000	.000	.000	.000	.000	.000	.000

	a1_1	a2_1	a3_1	a4_1	a5_1	a6_1	a7_1	a8_1	a9_1	a10_1	a11_1	a12_1	a13_1	a14_1	a15_1	a16_1	a17_1	a18_1	ccc1_1	ccc2_1
i24_2	.000	.000	.000	.000	.000	.000	.000	.000	.000	.000	.000	.000	.000	.000	.000	.000	.000	.000	.000	.000
ccc1_3	.000	.000	.000	.006	.008	.006	.000	.000	.000	.000	.000	.000	.000	.000	.000	.000	.000	.000	.000	.000
ccc2_3	.000	.000	.000	.005	.007	.005	.000	.000	.000	.000	.000	.000	.000	.000	.000	.000	.000	.000	.000	.000
ccc3_3	-.001	-.001	-.001	.000	.000	.000	-.001	-.001	-.001	.000	.000	.000	.000	.000	.000	.000	.000	.000	.000	.000
ccc4_3	.000	.000	.000	-.013	-.017	-.013	.000	.000	.000	.000	.000	.000	.000	.000	.000	.000	.000	.000	.000	.000
ccc5_3	.000	.000	.000	.000	.000	.000	.001	.001	.001	.001	.001	.001	.000	.000	.000	.000	.000	.000	.000	.000
ccc6_3	.001	.000	.000	.000	.000	.000	.000	.000	.000	.000	.001	.000	.000	.000	.000	.000	.000	.000	.000	.000
ccc7_3	.000	.000	.000	.006	.008	.006	.000	.000	.000	.000	.000	.000	.000	.000	.000	.000	.000	.000	.000	.000
ccc8_3	.000	.000	.000	.000	.000	.000	.000	.000	.000	.001	.001	.001	.001	.001	.001	.000	.000	.000	.000	.000
ccc9_3	.000	.000	.000	.000	.000	.000	-.001	-.001	-.001	.000	.000	.000	-.001	-.001	-.001	.000	.000	.000	.000	.000
ccc10_3	-.001	-.001	-.001	.000	.000	.000	.000	.000	.000	.000	.000	.000	-.001	-.001	-.001	.000	.000	.000	.000	.000
ccc11_3	.000	.000	.000	.000	.000	.000	.000	.000	.000	.000	.000	.000	.001	.001	.001	.001	.001	.001	.000	.000
ccc12_3	.000	.000	.000	.000	.000	.000	.001	.001	.001	.000	.000	.000	.000	.000	.000	.000	.000	.000	.000	.000
ccc13_3	.000	.000	.000	.000	.000	.000	.000	.000	.000	-.002	-.002	-.002	.000	.000	.000	-.001	-.001	-.001	.000	.000
ccc14_3	.000	.000	.000	-.015	-.020	-.016	.000	.000	.000	.000	.000	.000	.000	.000	.000	.000	.000	.000	.000	-.001
ccc15_3	.001	.001	.001	.000	.000	.000	.000	.000	.000	.000	.000	.000	.000	.000	.000	.000	.000	.000	.000	.000
vvv1_3	-.002	-.002	-.002	.000	.000	.000	.000	.000	.000	.000	.000	.000	.000	.000	.000	.000	.000	.000	.000	.000
v1_3	.000	.000	.000	.000	.000	.000	.000	.000	.000	.000	.000	.000	.000	.000	.000	.000	.000	.000	.000	.000
v2_3	.000	.000	.000	.000	.000	.000	.000	.000	.000	.000	.000	.000	.000	.000	.000	.000	.000	.000	.000	.000
v3_3	.000	.000	.000	.000	.000	.000	.000	.000	.000	.000	.000	.000	.000	.000	.000	.000	.000	.000	.000	.000
v4_3	.000	.000	.000	.000	.000	.000	.000	.000	.000	.000	.000	.000	.000	.000	.000	.000	.000	.000	.000	.000
vvv2_3	.000	.000	.000	-.011	-.014	-.011	.000	.000	.000	.000	.000	.000	.000	.000	.000	.000	.000	.000	.000	.000
v5_3	.000	.000	.000	.002	.002	.002	.000	.000	.000	.000	.000	.000	.000	.000	.000	.000	.000	.000	.000	.000
v6_3	.000	.000	.000	-.002	.000	.000	.000	.000	.000	.000	.000	.000	.000	.000	.000	.000	.000	.000	.000	.000
v7_3	.000	.000	.000	.000	-.003	.000	.000	.000	.000	.000	.000	.000	.000	.000	.000	.000	.000	.000	.000	.000
v8_3	.000	.000	.000	.000	.000	-.002	.000	.000	.000	.000	.000	.000	.000	.000	.000	.000	.000	.000	.000	.000
vvv3_3	.000	.000	.000	.000	.000	.000	-.002	-.002	-.002	.000	.000	.000	.000	.000	.000	.000	.000	.000	.000	.000
v9_3	.000	.000	.000	.000	.000	.000	.000	.000	.000	.000	.000	.000	.000	.000	.000	.000	.000	.000	.000	.000
v10_3	.000	.000	.000	.000	.000	.000	.000	.000	.000	.000	.000	.000	.000	.000	.000	.000	.000	.000	.000	.000
v11_3	.000	.000	.000	.000	.000	.000	.000	.000	.000	.000	.000	.000	.000	.000	.000	.000	.000	.000	.000	.000
v12_3	.000	.000	.000	.000	.000	.000	.000	.000	.000	.000	.000	.000	.000	.000	.000	.000	.000	.000	.000	.000
vvv4_3	.000	.000	.000	.000	.000	.000	.000	.000	.000	-.003	-.003	-.003	.000	.000	.000	.000	.000	.000	.000	.000
v13_3	.000	.000	.000	.000	.000	.000	.000	.000	.000	.000	.000	.000	.000	.000	.000	.000	.000	.000	.000	.000
v14_3	.000	.000	.000	.000	.000	.000	.000	.000	.000	.000	.000	.000	.000	.000	.000	.000	.000	.000	.000	.000
v15_3	.000	.000	.000	.000	.000	.000	.000	.000	.000	.000	-.001	.000	.000	.000	.000	.000	.000	.000	.000	.000
v16_3	.000	.000	.000	.000	.000	.000	.000	.000	.000	.000	.000	.000	.000	.000	.000	.000	.000	.000	.000	.000
vvv5_3	.000	.000	.000	.000	.000	.000	.000	.000	.000	.000	.000	.000	-.002	-.002	-.002	.000	.000	.000	.000	.000
v17_3	.000	.000	.000	.000	.000	.000	.000	.000	.000	.000	.000	.000	.000	.000	.000	.000	.000	.000	.000	.000
v18_3	.000	.000	.000	.000	.000	.000	.000	.000	.000	.000	.000	.000	.000	.000	.000	.000	.000	.000	.000	.000
v19_3	.000	.000	.000	.000	.000	.000	.000	.000	.000	.000	.000	.000	.000	.000	.000	.000	.000	.000	.000	.000
v20_3	.000	.000	.000	.000	.000	.000	.000	.000	.000	.000	.000	.000	.000	.000	.000	.000	.000	.000	.000	.000
vvv6_3	.000	.000	.000	.000	.000	.000	.000	.000	.000	.000	.000	.000	.000	.000	.000	-.002	-.002	-.002	.000	.000
v21_3	.000	.000	.000	.000	.000	.000	.000	.000	.000	.000	.000	.000	.000	.000	.000	.000	.000	.000	.000	.000
v22_3	.000	.000	.000	.000	.000	.000	.000	.000	.000	.000	.000	.000	.000	.000	.000	.000	.000	.000	.000	.000
v23_3	.000	.000	.000	.000	.000	.000	.000	.000	.000	.000	.000	.000	.000	.000	.000	.000	.000	.000	.000	.000
v24_3	.000	.000	.000	.000	.000	.000	.000	.000	.000	.000	.000	.000	.000	.000	.000	.000	.000	.000	.000	.000
i1_3	.000	.000	.000	.000	.000	.000	.000	.000	.000	.000	.000	.000	.000	.000	.000	.000	.000	.000	.000	.000
i2_3	.000	.000	.000	.000	.000	.000	.000	.000	.000	.000	.000	.000	.000	.000	.000	.000	.000	.000	.000	.000
i3_3	.000	.000	.000	.000	.000	.000	.000	.000	.000	.000	.000	.000	.000	.000	.000	.000	.000	.000	.000	.000
i4_3	.000	.000	.000	.000	.000	.000	.000	.000	.000	.000	.000	.000	.000	.000	.000	.000	.000	.000	.000	.000
i5_3	.000	.000	.000	.000	.000	.000	.000	.000	.000	.000	.000	.000	.000	.000	.000	.000	.000	.000	.000	.000
i6_3	.000	.000	.000	.000	.000	.000	.000	.000	.000	.000	.000	.000	.000	.000	.000	.000	.000	.000	.000	.000
i7_3	.000	.000	.000	.000	.000	.000	.000	.000	.000	.000	.000	.000	.000	.000	.000	.000	.000	.000	.000	.000
i8_3	.000	.000	.000	.000	.000	.000	.000	.000	.000	.000	.000	.000	.000	.000	.000	.000	.000	.000	.000	.000
i9_3	.000	.000	.000	.000	.000	.000	.000	.000	.000	.000	.000	.000	.000	.000	.000	.000	.000	.000	.000	.000
i10_3	.000	.000	.000	.000	.000	.000	.000	.000	.000	.000	.000	.000	.000	.000	.000	.000	.000	.000	.000	.000
i11_3	.000	.000	.000	.000	.000	.000	.000	.000	.000	.000	.000	.000	.000	.000	.000	.000	.000	.000	.000	.000
i12_3	.000	.000	.000	.000	.000	.000	.000	.000	.000	.000	.000	.000	.000	.000	.000	.000	.000	.000	.000	.000
i13_3	.000	.000	.000	.000	.000	.000	.000	.000	.000	.000	.000	.000	.000	.000	.000	.000	.000	.000	.000	.000
i14_3	.000	.000	.000	.000	.000	.000	.000	.000	.000	.000	.000	.000	.000	.000	.000	.000	.000	.000	.000	.000
i15_3	.000	.000	.000	.000	.000	.000	.000	.000	.000	.000	.000	.000	.000	.000	.000	.000	.000	.000	.000	.000
i16_3	.000	.000	.000	.000	.000	.000	.000	.000	.000	.000	.000	.000	.000	.000	.000	.000	.000	.000	.000	.000
i17_3	.000	.000	.000	.000	.000	.000	.000	.000	.000	.000	.000	.000	.000	.000	.000	.000	.000	.000	.000	.000
i18_3	.000	.000	.000	.000	.000	.000	.000	.000	.000	.000	.000	.000	.000	.000	.000	.000	.000	.000	.000	.000
i19_3	.000	.000	.000	.000	.000	.000	.000	.000	.000	.000	.000	.000	.000	.000	.000	.000	.000	.000	.000	.000
i20_3	.000	.000	.000	.000	.000	.000	.000	.000	.000	.000	.000	.000	.000	.000	.000	.000	.000	.000	.000	.000
i21_3	.000	.000	.000	.000	.000	.000	.000	.000	.000	.000	.000	.000	.000	.000	.000	.000	.000	.000	.000	.000
i22_3	.000	.000	.000	.000	.000	.000	.000	.000	.000	.000	.000	.000	.000	.000	.000	.000	.000	.000	.000	.000
i23_3	.000	.000	.000	.000	.000	.000	.000	.000	.000	.000	.000	.000	.000	.000	.000	.000	.000	.000	.000	.000
i24_3	.000	.000	.000	.000	.000	.000	.000	.000	.000	.000	.000	.000	.000	.000	.000	.000	.000	.000	.000	.000

Correlations of Estimates (Measurement weights)

	a1_1	a2_1	a3_1	a4_1	a5_1	a6_1	a7_1	a8_1	a9_1	a10_1	a11_1	a12_1	a13_1	a14_1	a15_1	a16_1	a17_1	a18_1	ccc1_1
**a1_1**	1.000																		
**a2_1**	.509	1.000																	
**a3_1**	.457	.354	1.000																
**a4_1**	.000	.000	.000	1.000															
**a5_1**	.000	.000	.000	.899	1.000														
**a6_1**	.000	.000	.000	.879	.910	1.000													
**a7_1**	.000	.000	.000	.000	.000	.000	1.000												
**a8_1**	.000	.000	.000	.000	.000	.000	.635	1.000											
**a9_1**	.000	.000	.000	.000	.000	.000	.488	.517	1.000										
**a10_1**	.000	.000	.000	.000	.000	.000	.000	.000	.000	1.000									
**a11_1**	.000	.000	.000	.000	.000	.000	.000	.000	.000	.589	1.000								
**a12_1**	.000	.000	.000	.000	.000	.000	.000	.000	.000	.565	.553	1.000							
**a13_1**	.000	.000	.000	.000	.000	.000	.000	.000	.000	.000	.000	.000	1.000						
**a14_1**	.000	.000	.000	.000	.000	.000	.000	.000	.000	.000	.000	.000	.553	1.000					
**a15_1**	.000	.000	.000	.000	.000	.000	.000	.000	.000	.000	.000	.000	.477	.452	1.000				
**a16_1**	.000	.000	.000	.000	.000	.000	.000	.000	.000	.000	.000	.000	.000	.000	.000	1.000			
**a17_1**	.000	.000	.000	.000	.000	.000	.000	.000	.000	.000	.000	.000	.000	.000	.000	.438	1.000		
**a18_1**	.000	.000	.000	.000	.000	.000	.000	.000	.000	.000	.000	.000	.000	.000	.000	.380	.385	1.000	
**ccc1_1**	.096	.070	.063	.353	.365	.356	.000	.000	.000	.000	.000	.000	.000	.000	.000	.000	.000	.000	1.000
**ccc2_1**	.000	.000	.000	.473	.489	.477	.104	.111	.084	.000	.000	.000	.000	.000	.000	.000	.000	.000	.656
**ccc3_1**	-.288	-.211	-.191	.000	.000	.000	-.233	-.248	-.189	.000	.000	.000	.000	.000	.000	.000	.000	.000	-.277
**ccc4_1**	.000	.000	.000	-.654	-.675	-.659	.000	.000	.000	-.148	-.142	-.136	.000	.000	.000	.000	.000	.000	-.497
**ccc5_1**	.000	.000	.000	.000	.000	.000	.197	.209	.160	.203	.195	.187	.000	.000	.000	.000	.000	.000	.263
**ccc6_1**	.193	.142	.128	.000	.000	.000	.000	.000	.000	.161	.155	.149	.000	.000	.000	.000	.000	.000	.429
**ccc7_1**	.000	.000	.000	.285	.294	.287	.000	.000	.000	.000	.000	.000	.060	.056	.049	.000	.000	.000	.759
**ccc8_1**	.000	.000	.000	.000	.000	.000	.000	.000	.000	.223	.214	.206	.207	.193	.171	.000	.000	.000	.276
**ccc9_1**	.000	.000	.000	.000	.000	.000	-.241	-.257	-.196	.000	.000	.000	-.231	-.215	-.191	.000	.000	.000	-.184
**ccc10_1**	-.331	-.243	-.219	.000	.000	.000	.000	.000	.000	.000	.000	.000	-.257	-.238	-.212	.000	.000	.000	-.201
**ccc11_1**	.000	.000	.000	.000	.000	.000	.000	.000	.000	.000	.000	.000	.231	.215	.191	.145	.151	.135	.298
**ccc12_1**	.000	.000	.000	.000	.000	.000	.181	.192	.147	.000	.000	.000	.000	.000	.000	.108	.113	.101	.304
**ccc13_1**	.000	.000	.000	.000	.000	.000	.000	.000	.000	-.292	-.280	-.269	.000	.000	.000	-.170	-.177	-.158	-.219
**ccc14_1**	.000	.000	.000	-.683	-.706	-.689	.000	.000	.000	.000	.000	.000	.000	.000	.000	-.090	-.094	-.084	-.531
**ccc15_1**	.220	.161	.145	.000	.000	.000	.000	.000	.000	.000	.000	.000	.000	.000	.000	.107	.111	.100	.466
**vvv1_1**	-.522	-.392	-.354	.000	.000	.000	.000	.000	.000	.000	.000	.000	.000	.000	.000	.000	.000	.000	-.231
**v1_1**	.089	.068	.062	.000	.000	.000	.000	.000	.000	.000	.000	.000	.000	.000	.000	.000	.000	.000	.010
**v2_1**	-.107	-.006	-.005	.000	.000	.000	.000	.000	.000	.000	.000	.000	.000	.000	.000	.000	.000	.000	-.008
**v3_1**	-.002	-.057	-.001	.000	.000	.000	.000	.000	.000	.000	.000	.000	.000	.000	.000	.000	.000	.000	-.002
**v4_1**	-.001	-.001	-.051	.000	.000	.000	.000	.000	.000	.000	.000	.000	.000	.000	.000	.000	.000	.000	-.001
**vvv2_1**	.000	.000	.000	-.871	-.894	-.877	.000	.000	.000	.000	.000	.000	.000	.000	.000	.000	.000	.000	-.424
**v5_1**	.000	.000	.000	.051	.052	.051	.000	.000	.000	.000	.000	.000	.000	.000	.000	.000	.000	.000	.020
**v6_1**	.000	.000	.000	-.069	.003	.002	.000	.000	.000	.000	.000	.000	.000	.000	.000	.000	.000	.000	-.006
**v7_1**	.000	.000	.000	.010	-.076	.005	.000	.000	.000	.000	.000	.000	.000	.000	.000	.000	.000	.000	-.012
**v8_1**	.000	.000	.000	.004	.004	-.063	.000	.000	.000	.000	.000	.000	.000	.000	.000	.000	.000	.000	-.006
**vvv3_1**	.000	.000	.000	.000	.000	.000	-.481	-.507	-.394	.000	.000	.000	.000	.000	.000	.000	.000	.000	-.150
**v9_1**	.000	.000	.000	.000	.000	.000	.117	.127	.093	.000	.000	.000	.000	.000	.000	.000	.000	.000	.000
**v10_1**	.000	.000	.000	.000	.000	.000	-.118	.003	-.004	.000	.000	.000	.000	.000	.000	.000	.000	.000	.000
**v11_1**	.000	.000	.000	.000	.000	.000	-.003	-.143	-.007	.000	.000	.000	.000	.000	.000	.000	.000	.000	.000
**v12_1**	.000	.000	.000	.000	.000	.000	-.001	.001	-.081	.000	.000	.000	.000	.000	.000	.000	.000	.000	.000
**vvv4_1**	.000	.000	.000	.000	.000	.000	.000	.000	.000	-.494	-.475	-.457	.000	.000	.000	.000	.000	.000	-.155
**v13_1**	.000	.000	.000	.000	.000	.000	.000	.000	.000	.084	.080	.077	.000	.000	.000	.000	.000	.000	.000
**v14_1**	.000	.000	.000	.000	.000	.000	.000	.000	.000	-.095	.000	.000	.000	.000	.000	.000	.000	.000	.000
**v15_1**	.000	.000	.000	.000	.000	.000	.000	.000	.000	.002	-.084	.000	.000	.000	.000	.000	.000	.000	.000
**v16_1**	.000	.000	.000	.000	.000	.000	.000	.000	.000	.002	.000	-.078	.000	.000	.000	.000	.000	.000	.000
**vvv5_1**	.000	.000	.000	.000	.000	.000	.000	.000	.000	.000	.000	.000	-.463	-.432	-.384	.000	.000	.000	-.104
**v17_1**	.000	.000	.000	.000	.000	.000	.000	.000	.000	.000	.000	.000	.059	.055	.049	.000	.000	.000	.000
**v18_1**	.000	.000	.000	.000	.000	.000	.000	.000	.000	.000	.000	.000	-.066	.000	.003	.000	.000	.000	.000
**v19_1**	.000	.000	.000	.000	.000	.000	.000	.000	.000	.000	.000	.000	.001	-.057	.002	.000	.000	.000	.000
**v20_1**	.000	.000	.000	.000	.000	.000	.000	.000	.000	.000	.000	.000	.000	.000	-.052	.000	.000	.000	.000
**vvv6_1**	.000	.000	.000	.000	.000	.000	.000	.000	.000	.000	.000	.000	.000	.000	.000	-.327	-.341	-.306	-.216
**v21_1**	.000	.000	.000	.000	.000	.000	.000	.000	.000	.000	.000	.000	.000	.000	.000	.090	.094	.084	.000
**v22_1**	.000	.000	.000	.000	.000	.000	.000	.000	.000	.000	.000	.000	.000	.000	.000	-.076	-.002	.000	.000
**v23_1**	.000	.000	.000	.000	.000	.000	.000	.000	.000	.000	.000	.000	.000	.000	.000	-.004	-.085	.000	.000
**v24_1**	.000	.000	.000	.000	.000	.000	.000	.000	.000	.000	.000	.000	.000	.000	.000	-.002	-.001	-.075	.000
**i1_1**	.000	.000	.000	.000	.000	.000	.000	.000	.000	.000	.000	.000	.000	.000	.000	.000	.000	.000	.000
**i2_1**	.000	.000	.000	.000	.000	.000	.000	.000	.000	.000	.000	.000	.000	.000	.000	.000	.000	.000	.000
**i3_1**	.000	.000	.000	.000	.000	.000	.000	.000	.000	.000	.000	.000	.000	.000	.000	.000	.000	.000	.000
**i4_1**	.000	.000	.000	.000	.000	.000	.000	.000	.000	.000	.000	.000	.000	.000	.000	.000	.000	.000	.000
**i5_1**	.000	.000	.000	.000	.000	.000	.000	.000	.000	.000	.000	.000	.000	.000	.000	.000	.000	.000	.000
**i6_1**	.000	.000	.000	.000	.000	.000	.000	.000	.000	.000	.000	.000	.000	.000	.000	.000	.000	.000	.000
**i7_1**	.000	.000	.000	.000	.000	.000	.000	.000	.000	.000	.000	.000	.000	.000	.000	.000	.000	.000	.000
**i8_1**	.000	.000	.000	.000	.000	.000	.000	.000	.000	.000	.000	.000	.000	.000	.000	.000	.000	.000	.000
**i9_1**	.000	.000	.000	.000	.000	.000	.000	.000	.000	.000	.000	.000	.000	.000	.000	.000	.000	.000	.000

	a1_1	a2_1	a3_1	a4_1	a5_1	a6_1	a7_1	a8_1	a9_1	a10_1	a11_1	a12_1	a13_1	a14_1	a15_1	a16_1	a17_1	a18_1	ccc1_1
**i10_1**	.000	.000	.000	.000	.000	.000	.000	.000	.000	.000	.000	.000	.000	.000	.000	.000	.000	.000	.000
**i11_1**	.000	.000	.000	.000	.000	.000	.000	.000	.000	.000	.000	.000	.000	.000	.000	.000	.000	.000	.000
**i12_1**	.000	.000	.000	.000	.000	.000	.000	.000	.000	.000	.000	.000	.000	.000	.000	.000	.000	.000	.000
**i13_1**	.000	.000	.000	.000	.000	.000	.000	.000	.000	.000	.000	.000	.000	.000	.000	.000	.000	.000	.000
**i14_1**	.000	.000	.000	.000	.000	.000	.000	.000	.000	.000	.000	.000	.000	.000	.000	.000	.000	.000	.000
**i15_1**	.000	.000	.000	.000	.000	.000	.000	.000	.000	.000	.000	.000	.000	.000	.000	.000	.000	.000	.000
**i16_1**	.000	.000	.000	.000	.000	.000	.000	.000	.000	.000	.000	.000	.000	.000	.000	.000	.000	.000	.000
**i17_1**	.000	.000	.000	.000	.000	.000	.000	.000	.000	.000	.000	.000	.000	.000	.000	.000	.000	.000	.000
**i18_1**	.000	.000	.000	.000	.000	.000	.000	.000	.000	.000	.000	.000	.000	.000	.000	.000	.000	.000	.000
**i19_1**	.000	.000	.000	.000	.000	.000	.000	.000	.000	.000	.000	.000	.000	.000	.000	.000	.000	.000	.000
**i20_1**	.000	.000	.000	.000	.000	.000	.000	.000	.000	.000	.000	.000	.000	.000	.000	.000	.000	.000	.000
**i21_1**	.000	.000	.000	.000	.000	.000	.000	.000	.000	.000	.000	.000	.000	.000	.000	.000	.000	.000	.000
**i22_1**	.000	.000	.000	.000	.000	.000	.000	.000	.000	.000	.000	.000	.000	.000	.000	.000	.000	.000	.000
**i23_1**	.000	.000	.000	.000	.000	.000	.000	.000	.000	.000	.000	.000	.000	.000	.000	.000	.000	.000	.000
**i24_1**	.000	.000	.000	.000	.000	.000	.000	.000	.000	.000	.000	.000	.000	.000	.000	.000	.000	.000	.000
**ccc1_2**	.181	.140	.129	.628	.653	.638	.000	.000	.000	.000	.000	.000	.000	.000	.000	.000	.000	.000	.266
**ccc2_2**	.000	.000	.000	.597	.621	.607	.137	.149	.110	.000	.000	.000	.000	.000	.000	.000	.000	.000	.234
**ccc3_2**	-.330	-.255	-.234	.000	.000	.000	-.262	-.285	-.210	.000	.000	.000	.000	.000	.000	.000	.000	.000	-.036
**ccc4_2**	.000	.000	.000	-.735	-.765	-.747	.000	.000	.000	-.167	-.160	-.160	.000	.000	.000	.000	.000	.000	-.289
**ccc5_2**	.000	.000	.000	.000	.000	.000	.237	.257	.190	.235	.224	.224	.000	.000	.000	.000	.000	.000	.000
**ccc6_2**	.262	.202	.186	.000	.000	.000	.000	.000	.000	.206	.197	.197	.000	.000	.000	.000	.000	.000	.028
**ccc7_2**	.000	.000	.000	.586	.610	.595	.000	.000	.000	.000	.000	.000	.124	.114	.098	.000	.000	.000	.230
**ccc8_2**	.000	.000	.000	.000	.000	.000	.000	.000	.000	.218	.208	.208	.203	.186	.160	.000	.000	.000	.000
**ccc9_2**	.000	.000	.000	.000	.000	.000	-.270	-.293	-.216	.000	.000	.000	-.249	-.228	-.196	.000	.000	.000	.000
**ccc10_2**	-.353	-.272	-.250	.000	.000	.000	.000	.000	.000	.000	.000	.000	-.259	-.237	-.204	.000	.000	.000	-.038
**ccc11_2**	.000	.000	.000	.000	.000	.000	.000	.000	.000	.000	.000	.000	.226	.207	.178	.155	.141	.126	.000
**ccc12_2**	.000	.000	.000	.000	.000	.000	.203	.220	.162	.000	.000	.000	.000	.000	.000	.128	.117	.104	.000
**ccc13_2**	.000	.000	.000	.000	.000	.000	.000	.000	.000	-.282	-.270	-.270	.000	.000	.000	-.181	-.165	-.147	.000
**ccc14_2**	.000	.000	.000	-.723	-.752	-.734	.000	.000	.000	.000	.000	.000	.000	.000	.000	-.105	-.096	-.085	-.284
**ccc15_2**	.244	.188	.173	.000	.000	.000	.000	.000	.000	.000	.000	.000	.000	.000	.000	.123	.112	.100	.026
**vvv1_2**	-.540	-.422	-.389	.000	.000	.000	.000	.000	.000	.000	.000	.000	.000	.000	.000	.000	.000	.000	-.059
**v1_2**	.041	.035	.032	.000	.000	.000	.000	.000	.000	.000	.000	.000	.000	.000	.000	.000	.000	.000	.005
**v2_2**	-.055	.005	.008	.000	.000	.000	.000	.000	.000	.000	.000	.000	.000	.000	.000	.000	.000	.000	-.004
**v3_2**	.000	-.039	.003	.000	.000	.000	.000	.000	.000	.000	.000	.000	.000	.000	.000	.000	.000	.000	-.001
**v4_2**	.000	.001	-.038	.000	.000	.000	.000	.000	.000	.000	.000	.000	.000	.000	.000	.000	.000	.000	-.001
**vvv2_2**	.000	.000	.000	-.873	-.905	-.887	.000	.000	.000	.000	.000	.000	.000	.000	.000	.000	.000	.000	-.342
**v5_2**	.000	.000	.000	.046	.048	.047	.000	.000	.000	.000	.000	.000	.000	.000	.000	.000	.000	.000	.018
**v6_2**	.000	.000	.000	-.052	.002	.001	.000	.000	.000	.000	.000	.000	.000	.000	.000	.000	.000	.000	-.005
**v7_2**	.000	.000	.000	.002	-.069	.005	.000	.000	.000	.000	.000	.000	.000	.000	.000	.000	.000	.000	-.012
**v8_2**	.000	.000	.000	.000	.002	-.059	.000	.000	.000	.000	.000	.000	.000	.000	.000	.000	.000	.000	-.006
**vvv3_2**	.000	.000	.000	.000	.000	.000	-.504	-.543	-.406	.000	.000	.000	.000	.000	.000	.000	.000	.000	.000
**v9_2**	.000	.000	.000	.000	.000	.000	.091	.101	.072	.000	.000	.000	.000	.000	.000	.000	.000	.000	.000
**v10_2**	.000	.000	.000	.000	.000	.000	-.103	.013	.000	.000	.000	.000	.000	.000	.000	.000	.000	.000	.000
**v11_2**	.000	.000	.000	.000	.000	.000	.007	-.136	-.001	.000	.000	.000	.000	.000	.000	.000	.000	.000	.000
**v12_2**	.000	.000	.000	.000	.000	.000	.001	.004	-.069	.000	.000	.000	.000	.000	.000	.000	.000	.000	.000
**vvv4_2**	.000	.000	.000	.000	.000	.000	.000	.000	.000	-.493	-.472	-.471	.000	.000	.000	.000	.000	.000	.000
**v13_2**	.000	.000	.000	.000	.000	.000	.000	.000	.000	.074	.071	.071	.000	.000	.000	.000	.000	.000	.000
**v14_2**	.000	.000	.000	.000	.000	.000	.000	.000	.000	-.083	-.002	.003	.000	.000	.000	.000	.000	.000	.000
**v15_2**	.000	.000	.000	.000	.000	.000	.000	.000	.000	.001	-.071	.003	.000	.000	.000	.000	.000	.000	.000
**v16_2**	.000	.000	.000	.000	.000	.000	.000	.000	.000	.001	-.001	-.079	.000	.000	.000	.000	.000	.000	.000
**vvv5_2**	.000	.000	.000	.000	.000	.000	.000	.000	.000	.000	.000	.000	-.467	-.429	-.371	.000	.000	.000	.000
**v17_2**	.000	.000	.000	.000	.000	.000	.000	.000	.000	.000	.000	.000	.065	.060	.053	.000	.000	.000	.000
**v18_2**	.000	.000	.000	.000	.000	.000	.000	.000	.000	.000	.000	.000	-.074	-.001	.000	.000	.000	.000	.000
**v19_2**	.000	.000	.000	.000	.000	.000	.000	.000	.000	.000	.000	.000	.001	-.060	.000	.000	.000	.000	.000
**v20_2**	.000	.000	.000	.000	.000	.000	.000	.000	.000	.000	.000	.000	.000	.000	-.049	.000	.000	.000	.000
**vvv6_2**	.000	.000	.000	.000	.000	.000	.000	.000	.000	.000	.000	.000	.000	.000	.000	-.350	-.320	-.287	.000
**v21_2**	.000	.000	.000	.000	.000	.000	.000	.000	.000	.000	.000	.000	.000	.000	.000	.104	.095	.085	.000
**v22_2**	.000	.000	.000	.000	.000	.000	.000	.000	.000	.000	.000	.000	.000	.000	.000	-.103	-.006	-.003	.000
**v23_2**	.000	.000	.000	.000	.000	.000	.000	.000	.000	.000	.000	.000	.000	.000	.000	.002	-.078	-.003	.000
**v24_2**	.000	.000	.000	.000	.000	.000	.000	.000	.000	.000	.000	.000	.000	.000	.000	.001	-.003	-.069	.000
**i1_2**	.000	.000	.000	.000	.000	.000	.000	.000	.000	.000	.000	.000	.000	.000	.000	.000	.000	.000	.000
**i2_2**	.000	.000	.000	.000	.000	.000	.000	.000	.000	.000	.000	.000	.000	.000	.000	.000	.000	.000	.000
**i3_2**	.000	.000	.000	.000	.000	.000	.000	.000	.000	.000	.000	.000	.000	.000	.000	.000	.000	.000	.000
**i4_2**	.000	.000	.000	.000	.000	.000	.000	.000	.000	.000	.000	.000	.000	.000	.000	.000	.000	.000	.000
**i5_2**	.000	.000	.000	.000	.000	.000	.000	.000	.000	.000	.000	.000	.000	.000	.000	.000	.000	.000	.000
**i6_2**	.000	.000	.000	.000	.000	.000	.000	.000	.000	.000	.000	.000	.000	.000	.000	.000	.000	.000	.000
**i7_2**	.000	.000	.000	.000	.000	.000	.000	.000	.000	.000	.000	.000	.000	.000	.000	.000	.000	.000	.000
**i8_2**	.000	.000	.000	.000	.000	.000	.000	.000	.000	.000	.000	.000	.000	.000	.000	.000	.000	.000	.000
**i9_2**	.000	.000	.000	.000	.000	.000	.000	.000	.000	.000	.000	.000	.000	.000	.000	.000	.000	.000	.000
**i10_2**	.000	.000	.000	.000	.000	.000	.000	.000	.000	.000	.000	.000	.000	.000	.000	.000	.000	.000	.000
**i11_2**	.000	.000	.000	.000	.000	.000	.000	.000	.000	.000	.000	.000	.000	.000	.000	.000	.000	.000	.000
**i12_2**	.000	.000	.000	.000	.000	.000	.000	.000	.000	.000	.000	.000	.000	.000	.000	.000	.000	.000	.000

	a1_1	a2_1	a3_1	a4_1	a5_1	a6_1	a7_1	a8_1	a9_1	a10_1	a11_1	a12_1	a13_1	a14_1	a15_1	a16_1	a17_1	a18_1	ccc1_1
i13_2	.000	.000	.000	.000	.000	.000	.000	.000	.000	.000	.000	.000	.000	.000	.000	.000	.000	.000	.000
i14_2	.000	.000	.000	.000	.000	.000	.000	.000	.000	.000	.000	.000	.000	.000	.000	.000	.000	.000	.000
i15_2	.000	.000	.000	.000	.000	.000	.000	.000	.000	.000	.000	.000	.000	.000	.000	.000	.000	.000	.000
i16_2	.000	.000	.000	.000	.000	.000	.000	.000	.000	.000	.000	.000	.000	.000	.000	.000	.000	.000	.000
i17_2	.000	.000	.000	.000	.000	.000	.000	.000	.000	.000	.000	.000	.000	.000	.000	.000	.000	.000	.000
i18_2	.000	.000	.000	.000	.000	.000	.000	.000	.000	.000	.000	.000	.000	.000	.000	.000	.000	.000	.000
i19_2	.000	.000	.000	.000	.000	.000	.000	.000	.000	.000	.000	.000	.000	.000	.000	.000	.000	.000	.000
i20_2	.000	.000	.000	.000	.000	.000	.000	.000	.000	.000	.000	.000	.000	.000	.000	.000	.000	.000	.000
i21_2	.000	.000	.000	.000	.000	.000	.000	.000	.000	.000	.000	.000	.000	.000	.000	.000	.000	.000	.000
i22_2	.000	.000	.000	.000	.000	.000	.000	.000	.000	.000	.000	.000	.000	.000	.000	.000	.000	.000	.000
i23_2	.000	.000	.000	.000	.000	.000	.000	.000	.000	.000	.000	.000	.000	.000	.000	.000	.000	.000	.000
i24_2	.000	.000	.000	.000	.000	.000	.000	.000	.000	.000	.000	.000	.000	.000	.000	.000	.000	.000	.000
ccc1_3	.148	.119	.102	.569	.598	.577	.000	.000	.000	.000	.000	.000	.000	.000	.000	.000	.000	.000	.241
ccc2_3	.000	.000	.000	.545	.572	.552	.126	.135	.101	.000	.000	.000	.000	.000	.000	.000	.000	.000	.215
ccc3_3	-.300	-.240	-.206	.000	.000	.000	-.266	-.285	-.214	.000	.000	.000	.000	.000	.000	.000	.000	.000	-.032
ccc4_3	.000	.000	.000	-.733	-.771	-.744	.000	.000	.000	-.162	-.166	-.151	.000	.000	.000	.000	.000	.000	-.289
ccc5_3	.000	.000	.000	.000	.000	.000	.205	.220	.165	.197	.202	.183	.000	.000	.000	.000	.000	.000	.000
ccc6_3	.168	.134	.115	.000	.000	.000	.000	.000	.000	.143	.146	.133	.000	.000	.000	.000	.000	.000	.018
ccc7_3	.000	.000	.000	.567	.596	.575	.000	.000	.000	.000	.000	.000	.113	.112	.093	.000	.000	.000	.224
ccc8_3	.000	.000	.000	.000	.000	.000	.000	.000	.000	.204	.208	.190	.183	.182	.151	.000	.000	.000	.000
ccc9_3	.000	.000	.000	.000	.000	.000	-.273	-.293	-.220	.000	.000	.000	-.235	-.235	-.194	.000	.000	.000	.000
ccc10_3	-.303	-.242	-.208	.000	.000	.000	.000	.000	.000	.000	.000	.000	-.231	-.231	-.191	.000	.000	.000	-.033
ccc11_3	.000	.000	.000	.000	.000	.000	.000	.000	.000	.000	.000	.000	.225	.224	.186	.163	.174	.141	.000
ccc12_3	.000	.000	.000	.000	.000	.000	.212	.227	.171	.000	.000	.000	.000	.000	.000	.132	.142	.115	.000
ccc13_3	.000	.000	.000	.000	.000	.000	.000	.000	.000	-.278	-.284	-.259	.000	.000	.000	-.181	-.194	-.157	.000
ccc14_3	.000	.000	.000	-.737	-.774	-.747	.000	.000	.000	.000	.000	.000	.000	.000	.000	-.106	-.114	-.092	-.291
ccc15_3	.197	.158	.135	.000	.000	.000	.000	.000	.000	.000	.000	.000	.000	.000	.000	.109	.117	.095	.021
vvv1_3	-.489	-.398	-.342	.000	.000	.000	.000	.000	.000	.000	.000	.000	.000	.000	.000	.000	.000	.000	-.053
v1_3	.068	.058	.050	.000	.000	.000	.000	.000	.000	.000	.000	.000	.000	.000	.000	.000	.000	.000	.008
v2_3	-.072	.000	-.003	.000	.000	.000	.000	.000	.000	.000	.000	.000	.000	.000	.000	.000	.000	.000	-.005
v3_3	-.004	-.053	-.001	.000	.000	.000	.000	.000	.000	.000	.000	.000	.000	.000	.000	.000	.000	.000	-.002
v4_3	-.002	.000	-.040	.000	.000	.000	.000	.000	.000	.000	.000	.000	.000	.000	.000	.000	.000	.000	-.001
vvv2_3	.000	.000	.000	-.870	-.909	-.882	.000	.000	.000	.000	.000	.000	.000	.000	.000	.000	.000	.000	-.342
v5_3	.000	.000	.000	.041	.043	.041	.000	.000	.000	.000	.000	.000	.000	.000	.000	.000	.000	.000	.016
v6_3	.000	.000	.000	-.041	.002	-.001	.000	.000	.000	.000	.000	.000	.000	.000	.000	.000	.000	.000	-.004
v7_3	.000	.000	.000	-.003	-.071	-.002	.000	.000	.000	.000	.000	.000	.000	.000	.000	.000	.000	.000	-.013
v8_3	.000	.000	.000	-.001	.002	-.045	.000	.000	.000	.000	.000	.000	.000	.000	.000	.000	.000	.000	-.004
vvv3_3	.000	.000	.000	.000	.000	.000	-.497	-.528	-.403	.000	.000	.000	.000	.000	.000	.000	.000	.000	.000
v9_3	.000	.000	.000	.000	.000	.000	.083	.089	.066	.000	.000	.000	.000	.000	.000	.000	.000	.000	.000
v10_3	.000	.000	.000	.000	.000	.000	-.094	.007	.001	.000	.000	.000	.000	.000	.000	.000	.000	.000	.000
v11_3	.000	.000	.000	.000	.000	.000	.006	-.115	.001	.000	.000	.000	.000	.000	.000	.000	.000	.000	.000
v12_3	.000	.000	.000	.000	.000	.000	.001	.003	-.066	.000	.000	.000	.000	.000	.000	.000	.000	.000	.000
vvv4_3	.000	.000	.000	.000	.000	.000	.000	.000	.000	-.479	-.489	-.447	.000	.000	.000	.000	.000	.000	.000
v13_3	.000	.000	.000	.000	.000	.000	.000	.000	.000	.081	.083	.076	.000	.000	.000	.000	.000	.000	.000
v14_3	.000	.000	.000	.000	.000	.000	.000	.000	.000	-.082	.003	-.003	.000	.000	.000	.000	.000	.000	.000
v15_3	.000	.000	.000	.000	.000	.000	.000	.000	.000	-.002	-.095	-.003	.000	.000	.000	.000	.000	.000	.000
v16_3	.000	.000	.000	.000	.000	.000	.000	.000	.000	-.001	.002	-.070	.000	.000	.000	.000	.000	.000	.000
vvv5_3	.000	.000	.000	.000	.000	.000	.000	.000	.000	.000	.000	.000	-.436	-.435	-.362	.000	.000	.000	.000
v17_3	.000	.000	.000	.000	.000	.000	.000	.000	.000	.000	.000	.000	.065	.065	.055	.000	.000	.000	.000
v18_3	.000	.000	.000	.000	.000	.000	.000	.000	.000	.000	.000	.000	-.062	.000	-.001	.000	.000	.000	.000
v19_3	.000	.000	.000	.000	.000	.000	.000	.000	.000	.000	.000	.000	-.004	-.068	-.001	.000	.000	.000	.000
v20_3	.000	.000	.000	.000	.000	.000	.000	.000	.000	.000	.000	.000	-.002	.000	-.048	.000	.000	.000	.000
vvv6_3	.000	.000	.000	.000	.000	.000	.000	.000	.000	.000	.000	.000	.000	.000	.000	-.349	-.372	-.304	.000
v21_3	.000	.000	.000	.000	.000	.000	.000	.000	.000	.000	.000	.000	.000	.000	.000	.076	.081	.066	.000
v22_3	.000	.000	.000	.000	.000	.000	.000	.000	.000	.000	.000	.000	.000	.000	.000	-.075	.004	.001	.000
v23_3	.000	.000	.000	.000	.000	.000	.000	.000	.000	.000	.000	.000	.000	.000	.000	.001	-.089	.001	.000
v24_3	.000	.000	.000	.000	.000	.000	.000	.000	.000	.000	.000	.000	.000	.000	.000	.001	.003	-.061	.000
i1_3	.000	.000	.000	.000	.000	.000	.000	.000	.000	.000	.000	.000	.000	.000	.000	.000	.000	.000	.000
i2_3	.000	.000	.000	.000	.000	.000	.000	.000	.000	.000	.000	.000	.000	.000	.000	.000	.000	.000	.000
i3_3	.000	.000	.000	.000	.000	.000	.000	.000	.000	.000	.000	.000	.000	.000	.000	.000	.000	.000	.000
i4_3	.000	.000	.000	.000	.000	.000	.000	.000	.000	.000	.000	.000	.000	.000	.000	.000	.000	.000	.000
i5_3	.000	.000	.000	.000	.000	.000	.000	.000	.000	.000	.000	.000	.000	.000	.000	.000	.000	.000	.000
i6_3	.000	.000	.000	.000	.000	.000	.000	.000	.000	.000	.000	.000	.000	.000	.000	.000	.000	.000	.000
i7_3	.000	.000	.000	.000	.000	.000	.000	.000	.000	.000	.000	.000	.000	.000	.000	.000	.000	.000	.000
i8_3	.000	.000	.000	.000	.000	.000	.000	.000	.000	.000	.000	.000	.000	.000	.000	.000	.000	.000	.000
i9_3	.000	.000	.000	.000	.000	.000	.000	.000	.000	.000	.000	.000	.000	.000	.000	.000	.000	.000	.000
i10_3	.000	.000	.000	.000	.000	.000	.000	.000	.000	.000	.000	.000	.000	.000	.000	.000	.000	.000	.000
i11_3	.000	.000	.000	.000	.000	.000	.000	.000	.000	.000	.000	.000	.000	.000	.000	.000	.000	.000	.000
i12_3	.000	.000	.000	.000	.000	.000	.000	.000	.000	.000	.000	.000	.000	.000	.000	.000	.000	.000	.000
i13_3	.000	.000	.000	.000	.000	.000	.000	.000	.000	.000	.000	.000	.000	.000	.000	.000	.000	.000	.000
i14_3	.000	.000	.000	.000	.000	.000	.000	.000	.000	.000	.000	.000	.000	.000	.000	.000	.000	.000	.000
i15_3	.000	.000	.000	.000	.000	.000	.000	.000	.000	.000	.000	.000	.000	.000	.000	.000	.000	.000	.000

	a1_1	a2_1	a3_1	a4_1	a5_1	a6_1	a7_1	a8_1	a9_1	a10_1	a11_1	a12_1	a13_1	a14_1	a15_1	a16_1	a17_1	a18_1	ccc1_1
**i16_3**	.000	.000	.000	.000	.000	.000	.000	.000	.000	.000	.000	.000	.000	.000	.000	.000	.000	.000	.000
**i17_3**	.000	.000	.000	.000	.000	.000	.000	.000	.000	.000	.000	.000	.000	.000	.000	.000	.000	.000	.000
**i18_3**	.000	.000	.000	.000	.000	.000	.000	.000	.000	.000	.000	.000	.000	.000	.000	.000	.000	.000	.000
**i19_3**	.000	.000	.000	.000	.000	.000	.000	.000	.000	.000	.000	.000	.000	.000	.000	.000	.000	.000	.000
**i20_3**	.000	.000	.000	.000	.000	.000	.000	.000	.000	.000	.000	.000	.000	.000	.000	.000	.000	.000	.000
**i21_3**	.000	.000	.000	.000	.000	.000	.000	.000	.000	.000	.000	.000	.000	.000	.000	.000	.000	.000	.000
**i22_3**	.000	.000	.000	.000	.000	.000	.000	.000	.000	.000	.000	.000	.000	.000	.000	.000	.000	.000	.000
**i23_3**	.000	.000	.000	.000	.000	.000	.000	.000	.000	.000	.000	.000	.000	.000	.000	.000	.000	.000	.000
**i24_3**	.000	.000	.000	.000	.000	.000	.000	.000	.000	.000	.000	.000	.000	.000	.000	.000	.000	.000	.000

Critical Ratios for Differences between Parameters (Measurement weights)

	a1_1	a2_1	a3_1	a4_1	a5_1	a6_1	a7_1	a8_1	a9_1	a10_1	a11_1	a12_1	a13_1	a14_1	a15_1
**a1_1**	.000														
**a2_1**	-5.346	.000													
**a3_1**	-6.901	-1.672	.000												
**a4_1**	4.256	5.108	5.410	.000											
**a5_1**	5.243	5.934	6.179	4.030	.000										
**a6_1**	4.389	5.233	5.532	.358	-3.910	.000									
**a7_1**	-.305	3.657	5.076	-4.334	-5.306	-4.466	.000								
**a8_1**	1.055	4.987	6.394	-4.034	-5.062	-4.169	2.386	.000							
**a9_1**	-4.184	-.160	1.286	-5.159	-5.975	-5.284	-5.773	-7.992	.000						
**a10_1**	.581	4.470	5.862	-4.135	-5.144	-4.269	.934	-.486	4.994	.000					
**a11_1**	.787	4.619	5.989	-4.083	-5.103	-4.218	1.146	-.254	5.138	.347	.000				
**a12_1**	-.434	3.445	4.834	-4.360	-5.327	-4.491	-.143	-1.551	3.887	-1.598	-1.883	.000			
**a13_1**	-.829	3.286	4.761	-4.449	-5.399	-4.580	-.548	-2.045	3.765	-1.508	-1.717	-.385	.000		
**a14_1**	-1.606	2.495	3.966	-4.612	-5.531	-4.741	-1.378	-2.862	2.901	-2.317	-2.511	-1.196	-1.304	.000	
**a15_1**	-3.824	.258	1.725	-5.075	-5.907	-5.201	-3.752	-5.206	.458	-4.633	-4.785	-3.510	-4.649	-3.355	.000
**a16_1**	-2.690	1.696	3.273	-4.815	-5.695	-4.943	-2.541	-4.125	2.071	-3.517	-3.697	-2.308	-2.063	-1.108	1.604
**a17_1**	-2.775	1.632	3.217	-4.830	-5.707	-4.957	-2.633	-4.223	2.004	-3.610	-3.789	-2.395	-2.157	-1.196	1.533
**a18_1**	-4.364	-.022	1.542	-5.146	-5.963	-5.271	-4.350	-5.896	.167	-5.268	-5.411	-4.066	-3.977	-3.024	-.334
**ccc1_1**	-22.041	-17.203	-15.466	-8.348	-8.563	-8.446	-22.923	-24.268	-18.760	-23.263	-22.948	-21.935	-23.900	-22.776	-19.800
**ccc2_1**	-21.766	-17.251	-15.591	-8.486	-8.676	-8.584	-24.597	-26.052	-20.106	-23.869	-23.531	-22.530	-24.610	-23.471	-20.466
**ccc3_1**	-6.215	-3.320	-2.230	-5.975	-6.640	-6.091	-6.417	-7.434	-3.435	-7.789	-7.888	-6.858	-6.869	-6.155	-4.149
**ccc4_1**	-14.946	-10.727	-9.193	-6.755	-7.224	-6.854	-15.795	-17.167	-11.696	-15.342	-15.303	-14.282	-16.090	-15.110	-12.436
**ccc5_1**	-20.751	-17.363	-16.135	-9.205	-9.263	-9.291	-23.960	-25.361	-19.978	-24.565	-24.351	-23.259	-21.803	-21.044	-18.983
**ccc6_1**	-21.179	-17.023	-15.629	-8.985	-9.088	-9.072	-19.666	-20.716	-16.606	-21.927	-21.801	-20.775	-19.831	-19.114	-17.154
**ccc7_1**	-21.007	-16.500	-14.845	-8.251	-8.482	-8.349	-22.699	-24.052	-18.513	-23.045	-22.733	-21.713	-24.299	-23.096	-19.979
**ccc8_1**	-20.340	-17.287	-16.184	-9.601	-9.595	-9.682	-20.912	-21.893	-18.062	-24.030	-23.880	-22.825	-23.331	-22.416	-20.176
**ccc9_1**	-6.595	-3.228	-2.021	-5.877	-6.561	-5.994	-5.971	-6.979	-3.015	-7.316	-7.420	-6.394	-5.784	-5.177	-3.399
**ccc10_1**	-2.702	-.145	.807	-5.072	-5.908	-5.196	-2.959	-3.992	-.046	-3.608	-3.743	-2.818	-2.392	-1.865	-.334
**ccc11_1**	-19.852	-17.216	-16.268	-10.214	-10.114	-10.290	-20.219	-21.079	-17.753	-20.640	-20.633	-19.908	-22.415	-21.629	-19.709
**ccc12_1**	-19.316	-16.213	-15.093	-9.266	-9.321	-9.351	-21.796	-23.066	-18.223	-20.323	-20.281	-19.452	-19.995	-19.319	-17.468
**ccc13_1**	-.116	2.378	3.265	-4.163	-5.166	-4.294	.079	-.807	2.553	-.449	-.576	.151	.405	.898	2.303
**ccc14_1**	-12.621	-8.734	-7.329	-6.394	-6.916	-6.493	-13.129	-14.425	-9.325	-13.765	-13.757	-12.672	-13.181	-12.315	-9.924
**ccc15_1**	-20.569	-16.793	-15.546	-9.399	-9.439	-9.483	-18.748	-19.681	-16.065	-19.204	-19.194	-18.410	-18.791	-18.183	-16.507
**vvv1_1**	-2.338	-.241	.547	-5.048	-5.892	-5.171	-2.676	-3.566	-.184	-3.241	-3.363	-2.562	-2.389	-1.885	-.453
**v1_1**	.315	3.047	4.007	-4.056	-5.078	-4.188	.522	-.424	3.167	-.098	-.248	.611	.878	1.405	2.906
**v2_1**	-3.691	-.812	.278	-5.266	-6.066	-5.388	-3.773	-4.851	-.727	-4.441	-4.573	-3.613	-3.459	-2.834	-1.065
**v3_1**	4.239	6.199	7.057	-2.373	-3.684	-2.517	4.475	3.729	6.538	3.971	3.835	4.521	4.789	5.192	6.345
**v4_1**	5.035	7.006	7.578	-1.936	-3.318	-2.083	5.266	4.560	7.218	4.786	4.656	5.307	5.568	5.948	7.038
**vvv2_1**	-16.725	-12.411	-10.838	-6.913	-7.347	-7.009	-17.799	-19.173	-13.653	-18.327	-18.177	-17.082	-18.267	-17.243	-14.472
**v5_1**	6.153	7.973	8.617	-1.276	-2.784	-1.429	6.373	5.720	8.171	5.928	5.805	6.407	6.655	7.004	8.006
**v6_1**	3.096	5.148	5.875	-2.736	-4.040	-2.941	3.307	2.575	5.334	2.816	2.688	3.360	3.604	4.002	5.140
**v7_1**	1.486	3.453	4.150	-3.399	-4.422	-3.528	1.663	.966	3.596	1.202	1.085	1.723	1.931	2.313	3.403
**v8_1**	2.831	4.907	5.643	-2.926	-4.142	-2.998	3.041	2.301	5.092	2.546	2.417	3.096	3.340	3.743	4.895
**vvv3_1**	-4.838	-1.766	-.667	-5.524	-6.276	-5.644	-3.980	-4.831	-1.484	-5.432	-5.554	-4.598	-4.484	-3.854	-2.073
**v9_1**	-4.369	-1.014	.187	-5.329	-6.115	-5.451	-4.558	-5.846	-.976	-5.017	-5.150	-4.104	-3.974	-3.278	-1.311
**v10_1**	-5.068	-1.706	-.502	-5.501	-6.255	-5.622	-4.766	-6.215	-1.663	-5.739	-5.863	-4.823	-4.728	-4.029	-2.054
**v11_1**	-6.696	-3.233	-1.991	-5.865	-6.551	-5.983	-6.736	-7.445	-3.284	-7.448	-7.551	-6.498	-6.497	-5.768	-3.718
**v12_1**	-1.679	1.372	2.461	-4.666	-5.576	-4.793	-1.495	-2.582	1.511	-2.189	-2.342	-1.367	-1.127	-.509	1.248
**vvv4_1**	-1.405	1.035	1.904	-4.590	-5.516	-4.717	-1.244	-2.106	1.165	-1.527	-1.640	-.983	-.945	-.461	.914
**v13_1**	.076	2.782	3.744	-4.136	-5.144	-4.267	.294	-.671	2.994	-.350	-.507	.400	.654	1.192	2.726
**v14_1**	-1.622	1.204	2.211	-4.656	-5.569	-4.784	-1.447	-2.449	1.368	-2.007	-2.233	-1.330	-1.104	-.537	1.076
**v15_1**	.790	3.271	4.153	-3.858	-4.916	-3.992	1.006	.121	3.471	.423	.270	1.085	1.344	1.833	3.228
**v16_1**	1.200	3.673	4.552	-3.719	-4.801	-3.853	1.424	.541	3.884	.841	.694	1.455	1.767	2.254	3.644
**vvv5_1**	-1.765	.758	1.657	-4.709	-5.613	-4.835	-1.608	-2.499	.885	-2.180	-2.309	-1.504	-1.125	-.699	.550
**v17_1**	-.857	2.043	3.076	-4.433	-5.387	-4.563	-.653	-1.686	2.249	-1.321	-1.475	-.542	-.292	.304	1.996
**v18_1**	-4.048	-.762	.413	-5.263	-6.062	-5.386	-3.958	-5.116	-.668	-4.670	-4.805	-3.777	-3.525	-2.953	-1.036
**v19_1**	-1.433	1.532	2.590	-4.599	-5.522	-4.727	-1.244	-2.299	1.723	-1.921	-2.072	-1.123	-.881	-.276	1.420
**v20_1**	-.061	2.763	3.769	-4.193	-5.191	-4.325	.163	-.846	2.990	-.496	-.652	.261	.537	1.101	2.654

	a1_1	a2_1	a3_1	a4_1	a5_1	a6_1	a7_1	a8_1	a9_1	a10_1	a11_1	a12_1	a13_1	a14_1	a15_1
vvv6_1	2.550	4.438	5.107	-2.889	-4.104	-3.027	2.733	2.062	4.590	2.286	2.170	2.786	2.998	3.364	4.408
v21_1	-2.953	.083	1.167	-5.018	-5.864	-5.142	-2.817	-3.890	.213	-3.491	-3.631	-2.671	-2.482	-1.865	-.114
v22_1	1.531	3.956	4.818	-3.589	-4.695	-3.725	1.758	.891	4.169	1.183	1.038	1.829	2.098	2.574	3.935
v23_1	-.082	2.586	3.536	-4.179	-5.179	-4.310	.128	-.822	2.787	-.492	-.640	.222	.480	1.010	2.521
v24_1	1.631	4.101	4.979	-3.573	-4.681	-3.709	1.865	.981	4.324	1.278	1.130	1.936	2.214	2.700	4.087
i1_1	48.617	52.206	53.453	8.557	5.190	8.306	50.893	49.320	55.128	49.379	48.649	50.254	52.745	53.356	55.293
i2_1	48.104	51.763	53.034	8.206	4.899	7.957	50.443	48.835	54.776	48.891	48.143	49.783	52.350	52.973	54.952
i3_1	42.200	45.555	46.725	7.401	4.260	7.161	44.023	42.601	47.867	42.714	42.113	43.545	45.532	46.117	47.937
i4_1	38.256	41.580	42.741	6.329	3.386	6.099	39.928	38.538	43.696	38.676	38.117	39.505	41.331	41.919	43.730
i5_1	36.688	40.081	41.267	5.691	2.862	5.467	38.372	36.954	42.221	37.101	36.536	37.948	39.793	40.396	42.252
i6_1	29.382	32.650	33.796	3.880	1.390	3.673	30.725	29.401	34.341	29.596	29.126	30.425	31.900	32.496	34.297
i7_1	22.896	25.984	27.071	2.349	.147	2.157	23.916	22.703	27.245	22.935	22.557	23.728	24.851	25.426	27.137
i8_1	28.236	31.498	32.643	3.560	1.129	3.356	29.538	28.222	33.136	28.424	27.965	29.253	30.685	31.281	33.083
i9_1	52.146	56.135	57.517	8.389	5.033	8.137	55.036	53.224	59.918	53.220	52.314	54.181	57.389	58.055	60.215
i10_1	49.258	53.115	54.453	7.970	4.698	7.722	51.872	50.150	56.517	50.181	49.353	51.117	54.007	54.660	56.751
i11_1	50.316	54.147	55.475	8.330	4.992	8.079	52.937	51.224	57.551	51.250	50.421	52.178	55.070	55.716	57.788
i12_1	49.732	53.684	55.054	7.846	4.593	7.600	52.482	50.704	57.279	50.722	49.855	51.679	54.730	55.396	57.542
i13_1	21.398	24.718	25.888	1.509	-.547	1.323	22.505	21.194	26.116	21.443	21.034	22.297	23.529	24.151	26.008
i14_1	13.324	16.471	17.584	-.523	-2.199	-.689	14.063	12.866	17.379	13.163	12.858	13.985	14.816	15.421	17.194
i15_1	17.263	20.312	21.389	.747	-1.160	.569	18.089	16.916	21.322	17.181	16.856	17.972	18.889	19.467	21.169
i16_1	11.780	14.860	15.950	-.891	-2.498	-1.054	12.441	11.280	15.663	11.581	11.300	12.389	13.135	13.730	15.466
i17_1	49.144	52.738	53.986	8.695	5.301	8.442	51.442	49.865	55.688	49.920	49.184	50.795	53.310	53.921	55.859
i18_1	52.414	56.081	57.352	9.397	5.871	9.138	54.907	53.275	59.292	53.302	52.512	54.189	56.920	57.535	59.507
i19_1	54.662	58.283	59.537	10.191	6.521	9.924	57.172	55.556	61.506	55.572	54.779	56.446	59.186	59.789	61.728
i20_1	54.376	58.090	59.377	9.802	6.198	9.538	56.993	55.326	61.468	55.335	54.510	56.230	59.099	59.718	61.711
i21_1	10.174	13.293	14.398	-1.386	-2.903	-1.544	10.794	9.624	14.047	9.940	9.668	10.760	11.464	12.070	13.835
i22_1	13.585	16.569	17.624	-.252	-1.973	-.420	14.269	13.139	17.392	13.423	13.138	14.202	14.967	15.538	17.211
i23_1	16.634	19.717	20.806	.512	-1.353	.336	17.455	16.271	20.725	16.542	16.217	17.343	18.257	18.842	20.567
i24_1	14.312	17.422	18.521	-.197	-1.932	-.366	15.069	13.884	18.350	14.171	13.863	14.983	15.831	16.426	18.173
ccc1_2	-23.359	-18.450	-16.745	-9.153	-9.232	-9.258	-22.634	-23.887	-18.833	-23.024	-22.798	-21.830	-23.308	-22.326	-19.700
ccc2_2	-21.280	-17.133	-15.617	-9.037	-9.136	-9.140	-24.306	-25.817	-19.905	-23.045	-22.809	-21.835	-23.367	-22.368	-19.700
ccc3_2	-3.141	-.599	.344	-5.215	-6.025	-5.338	-3.134	-4.024	-.528	-4.119	-4.250	-3.329	-3.167	-2.574	-.893
ccc4_2	-10.674	-7.047	-5.741	-6.032	-6.600	-6.126	-10.971	-12.200	-7.407	-10.762	-10.834	-9.861	-10.888	-10.102	-7.917
ccc5_2	-19.966	-17.159	-16.148	-9.921	-9.866	-10.000	-22.928	-24.272	-19.420	-23.454	-23.331	-22.436	-20.481	-19.880	-18.229
ccc6_2	-22.426	-18.341	-17.002	-9.552	-9.559	-9.634	-19.929	-20.883	-17.174	-22.645	-22.526	-21.615	-20.021	-19.387	-17.647
ccc7_2	-21.162	-17.035	-15.526	-9.034	-9.134	-9.137	-22.513	-23.775	-18.682	-22.904	-22.676	-21.701	-24.753	-23.557	-20.570
ccc8_2	-19.437	-16.615	-15.598	-9.747	-9.723	-9.827	-19.867	-20.785	-17.222	-22.643	-22.536	-21.645	-21.881	-21.039	-18.987
ccc9_2	-2.909	-.060	.957	-5.032	-5.876	-5.156	-2.498	-3.371	.051	-3.408	-3.542	-2.640	-2.238	-1.726	-.232
ccc10_2	-1.809	.620	1.517	-4.794	-5.682	-4.920	-1.917	-2.896	.831	-2.541	-2.679	-1.798	-1.448	-.949	.502
ccc11_2	-19.189	-16.696	-15.801	-10.305	-10.198	-10.380	-19.486	-20.305	-17.147	-19.901	-19.908	-19.212	-21.393	-20.644	-18.829
ccc12_2	-18.381	-15.699	-14.734	-9.695	-9.688	-9.776	-20.565	-21.762	-17.392	-19.160	-19.162	-18.416	-18.731	-18.165	-16.601
ccc13_2	.599	2.902	3.720	-3.873	-4.926	-4.005	.794	-.025	3.070	.232	.112	.793	1.102	1.554	2.843
ccc14_2	-9.988	-6.494	-5.238	-5.929	-6.512	-6.024	-10.213	-11.404	-6.780	-10.863	-10.920	-9.896	-10.089	-9.341	-7.254
ccc15_2	-20.687	-17.031	-15.821	-9.572	-9.585	-9.655	-18.607	-19.504	-16.036	-19.054	-19.054	-18.296	-18.625	-18.047	-16.451
vvv1_2	-2.251	-.209	.554	-5.025	-5.873	-5.148	-2.577	-3.445	-.149	-3.129	-3.250	-2.467	-2.295	-1.804	-.411
v1_2	3.255	5.529	6.333	-2.907	-4.130	-3.047	3.443	2.636	5.681	2.900	2.758	3.498	3.776	4.215	5.470
v2_2	-3.624	-.587	.536	-5.209	-6.019	-5.331	-3.611	-4.715	-.485	-4.296	-4.431	-3.447	-3.287	-2.645	-.830
v3_2	2.408	4.764	5.708	-3.291	-4.449	-3.429	2.656	1.785	5.077	2.073	1.923	2.719	3.009	3.486	4.848
v4_2	2.480	4.930	5.708	-3.275	-4.436	-3.413	2.731	1.854	5.170	2.144	1.992	2.794	3.089	3.568	4.940
vvv2_2	-15.246	-11.170	-9.689	-6.703	-7.161	-6.793	-16.043	-17.368	-12.100	-16.619	-16.538	-15.457	-16.302	-15.364	-12.805
v5_2	6.209	8.015	8.655	-1.227	-2.741	-1.381	6.427	5.779	8.212	5.985	5.863	6.461	6.707	7.054	8.048
v6_2	4.970	6.754	7.386	-1.689	-3.129	-1.869	5.169	4.532	6.928	4.738	4.622	5.209	5.436	5.780	6.762
v7_2	1.461	3.459	4.168	-3.419	-4.456	-3.557	1.640	.931	3.606	1.171	1.053	1.702	1.913	2.301	3.410
v8_2	2.531	4.672	5.431	-3.084	-4.275	-3.161	2.744	1.980	4.861	2.234	2.102	2.802	3.050	3.467	4.657
vvv3_2	-1.031	1.363	2.215	-4.460	-5.410	-4.588	-.735	-1.438	1.318	-1.412	-1.540	-.772	-.565	-.092	1.254
v9_2	-.533	2.262	3.257	-4.331	-5.304	-4.461	-.339	-1.383	2.541	-.972	-1.124	-.223	.035	.593	2.184
v10_2	-3.420	-.304	.809	-5.132	-5.956	-5.256	-3.156	-4.428	-.190	-3.987	-4.125	-3.144	-2.971	-2.334	-.530
v11_2	-6.297	-2.940	-1.737	-5.807	-6.504	-5.925	-6.336	-7.036	-2.973	-7.006	-7.114	-6.088	-6.055	-5.353	-3.377
v12_2	.802	3.456	4.400	-3.899	-4.950	-4.032	1.036	.087	3.589	.410	.256	1.119	1.401	1.926	3.427
vvv4_2	.199	2.327	3.083	-3.967	-5.003	-4.098	.370	-.383	2.464	-.106	-.211	.384	.649	1.065	2.251
v13_2	1.862	4.268	5.123	-3.468	-4.595	-3.604	2.094	1.234	4.487	1.567	1.416	2.224	2.436	2.908	4.257
v14_2	.347	2.862	3.757	-4.014	-5.044	-4.146	.555	-.341	3.054	-.031	-.175	.640	.891	1.388	2.806
v15_2	2.937	5.065	5.819	-2.919	-4.137	-3.058	3.155	2.396	5.261	2.648	2.452	3.214	3.464	3.877	5.060
v16_2	1.279	3.709	4.573	-3.677	-4.766	-3.812	1.501	.634	3.915	.928	.783	1.528	1.838	2.316	3.680
vvv5_2	-.711	1.628	2.460	-4.344	-5.314	-4.473	-.541	-1.369	1.766	-1.079	-1.206	-.454	-.213	.190	1.364
v17_2	-.769	2.091	3.110	-4.406	-5.365	-4.535	-.565	-1.584	2.295	-1.224	-1.377	-.457	-.206	.383	2.050
v18_2	-3.784	-.570	.580	-5.210	-6.019	-5.333	-3.681	-4.815	-.466	-4.383	-4.519	-3.511	-3.246	-2.690	-.821
v19_2	-.529	2.276	3.275	-4.331	-5.304	-4.461	-.321	-1.321	2.481	-.970	-1.122	-.218	.041	.588	2.199
v20_2	2.157	4.607	5.478	-3.386	-4.528	-3.524	2.401	1.523	4.842	1.815	1.665	2.467	2.755	3.235	4.531
vvv6_2	2.938	4.703	5.329	-2.606	-3.863	-2.745	3.111	2.484	4.844	2.693	2.584	3.160	3.360	3.701	4.674
v21_2	-.913	1.752	2.700	-4.439	-5.392	-4.567	-.725	-1.672	1.923	-1.337	-1.479	-.624	-.390	.141	1.652
v22_2	.556	3.042	3.925	-3.938	-4.981	-4.071	.767	-.119	3.235	.185	.042	.848	1.102	1.592	2.991

	a1_1	a2_1	a3_1	a4_1	a5_1	a6_1	a7_1	a8_1	a9_1	a10_1	a11_1	a12_1	a13_1	a14_1	a15_1
v23_2	3.274	5.361	6.101	-2.757	-4.002	-2.898	3.493	2.747	5.557	2.992	2.861	3.545	3.798	4.203	5.361
v24_2	4.723	6.634	7.311	-1.994	-3.362	-2.140	4.938	4.255	6.826	4.476	4.351	4.980	5.226	5.594	6.649
i1_2	45.221	48.574	49.741	8.307	5.000	8.059	47.134	45.701	50.998	45.796	45.171	46.621	48.701	49.280	51.090
i2_2	47.857	51.492	52.754	8.207	4.902	7.959	50.159	48.566	54.454	48.625	47.888	49.512	52.037	52.657	54.623
i3_2	42.863	46.378	47.602	7.133	4.032	6.895	44.868	43.360	48.943	43.461	42.807	44.328	46.525	47.135	49.046
i4_2	43.934	47.536	48.790	7.194	4.077	6.954	46.066	44.508	50.278	44.596	43.905	45.482	47.825	48.448	50.406
i5_2	33.281	36.621	37.790	4.841	2.171	4.625	34.807	33.432	38.554	33.601	33.080	34.441	36.116	36.716	38.550
i6_2	25.383	28.383	29.437	3.287	.918	3.087	26.424	25.241	29.662	25.456	25.075	26.223	27.362	27.915	29.568
i7_2	20.555	23.504	24.543	1.911	-.205	1.723	21.430	20.288	24.567	20.528	20.193	21.289	22.251	22.803	24.437
i8_2	27.077	30.256	31.373	3.400	1.002	3.198	28.284	27.012	31.764	27.221	26.789	28.030	29.356	29.939	31.695
i9_2	47.760	51.276	52.497	8.538	5.178	8.287	49.925	48.396	54.044	48.465	47.768	49.325	51.689	52.290	54.187
i10_2	46.528	50.021	51.236	8.251	4.946	8.003	48.629	47.117	52.705	47.196	46.516	48.052	50.346	50.946	52.833
i11_2	47.475	50.962	52.174	8.544	5.185	8.293	49.600	48.087	53.675	48.160	47.474	49.013	51.331	51.928	53.810
i12_2	45.989	49.571	50.817	7.833	4.599	7.588	48.169	46.613	52.369	46.689	45.985	47.566	49.955	50.571	52.511
i13_2	20.902	24.025	25.125	1.695	-.388	1.509	21.880	20.659	25.236	20.905	20.537	21.710	22.791	23.376	25.114
i14_2	13.449	16.402	17.446	-.252	-1.973	-.420	14.116	13.001	17.202	13.283	13.003	14.054	14.800	15.365	17.019
i15_2	15.746	18.573	19.572	.633	-1.245	.458	16.430	15.358	19.386	15.618	15.338	16.354	17.111	17.647	19.220
i16_2	12.226	15.205	16.259	-.649	-2.298	-.814	12.865	11.744	15.972	12.035	11.762	12.815	13.533	14.106	15.779
i17_2	46.852	50.353	51.571	8.319	5.000	8.070	48.972	47.455	53.063	47.531	46.845	48.388	50.704	51.304	53.195
i18_2	51.422	54.985	56.221	9.447	5.917	9.187	53.758	52.188	57.978	52.230	51.485	53.095	55.645	56.246	58.160
i19_2	51.603	55.078	56.284	9.793	6.206	9.531	53.843	52.319	57.935	52.368	51.651	53.212	55.648	56.235	58.099
i20_2	50.484	53.957	55.162	9.472	5.943	9.212	52.687	51.169	56.766	51.225	50.518	52.070	54.467	55.056	56.921
i21_2	10.922	13.930	14.995	-1.064	-2.638	-1.225	11.530	10.403	14.659	10.704	10.439	11.494	12.180	12.762	14.456
i22_2	13.979	16.972	18.030	-.147	-1.888	-.316	14.678	13.543	17.816	13.825	13.535	14.606	15.387	15.960	17.637
i23_2	16.272	19.145	20.160	.723	-1.173	.546	16.990	15.896	20.003	16.157	15.866	16.903	17.698	18.243	19.842
i24_2	14.905	17.789	18.808	.285	-1.532	.112	15.588	14.496	18.604	14.764	14.483	15.516	16.275	16.824	18.433
ccc1_3	-22.954	-18.156	-16.382	-8.907	-9.034	-9.009	-22.844	-24.126	-18.930	-23.217	-22.961	-21.980	-23.616	-22.589	-19.853
ccc2_3	-21.463	-17.170	-15.598	-8.797	-8.942	-8.898	-24.439	-25.918	-19.990	-23.356	-23.083	-22.096	-23.826	-22.774	-19.977
ccc3_3	-3.759	-1.084	-.099	-5.371	-6.151	-5.492	-3.745	-4.672	-1.038	-4.836	-4.963	-4.007	-3.869	-3.243	-1.473
ccc4_3	-10.334	-6.765	-5.480	-5.976	-6.547	-6.072	-10.597	-11.811	-7.089	-10.440	-10.466	-9.567	-10.494	-9.724	-7.582
ccc5_3	-19.453	-16.478	-15.405	-9.497	-9.513	-9.580	-22.128	-23.421	-18.580	-22.565	-22.619	-21.479	-20.049	-19.406	-17.643
ccc6_3	-19.625	-15.955	-14.612	-8.965	-9.077	-9.053	-18.444	-19.447	-15.543	-20.340	-20.369	-19.312	-18.524	-17.860	-16.032
ccc7_3	-21.226	-17.050	-15.522	-8.949	-9.069	-9.051	-22.627	-23.900	-18.752	-23.011	-22.770	-21.791	-24.746	-23.673	-20.605
ccc8_3	-19.145	-16.331	-15.318	-9.681	-9.670	-9.762	-19.560	-20.477	-16.920	-22.123	-22.201	-21.095	-21.313	-20.660	-18.573
ccc9_3	-3.495	-.526	.534	-5.180	-5.996	-5.302	-3.030	-3.937	-.391	-4.032	-4.165	-3.230	-2.796	-2.245	-.695
ccc10_3	-3.011	-.436	.515	-5.163	-5.983	-5.286	-3.288	-4.318	-.382	-3.933	-4.065	-3.143	-2.721	-2.179	-.650
ccc11_3	-19.217	-16.880	-16.041	-10.658	-10.500	-10.731	-19.464	-20.235	-17.270	-19.863	-19.879	-19.219	-21.212	-20.682	-18.902
ccc12_3	-18.529	-15.892	-14.944	-9.819	-9.792	-9.899	-20.787	-21.947	-17.637	-19.292	-19.295	-18.561	-18.864	-18.309	-16.773
ccc13_3	1.828	3.924	4.668	-3.326	-4.473	-3.462	2.024	1.279	4.092	1.400	1.280	1.921	2.316	2.724	3.889
ccc14_3	-8.569	-5.305	-4.134	-5.656	-6.269	-5.751	-8.680	-9.802	-5.470	-9.320	-9.401	-8.424	-8.494	-7.808	-5.887
ccc15_3	-19.175	-15.874	-14.663	-9.411	-9.456	-9.494	-17.710	-18.595	-15.178	-18.161	-18.172	-17.419	-17.701	-17.136	-15.575
vvv1_3	-2.615	-.481	.312	-5.143	-5.969	-5.265	-2.989	-3.888	-.470	-3.557	-3.679	-2.870	-2.704	-2.193	-.744
v1_3	.257	2.987	3.945	-4.074	-5.094	-4.206	.469	-.481	3.123	-.154	-.304	.558	.825	1.354	2.861
v2_3	-1.681	1.084	2.086	-4.690	-5.597	-4.817	-1.563	-2.562	1.244	-2.203	-2.345	-1.445	-1.224	-.658	.951
v3_3	2.624	4.877	5.809	-3.173	-4.351	-3.312	2.869	2.028	5.207	2.306	2.159	2.930	3.212	3.671	4.985
v4_3	5.408	7.306	7.876	-1.682	-3.102	-1.830	5.634	4.955	7.507	5.172	5.046	5.672	5.924	6.289	7.334
vvv2_3	-15.287	-11.204	-9.721	-6.711	-7.163	-6.802	-16.091	-17.418	-12.142	-16.667	-16.583	-15.502	-16.356	-15.416	-12.851
v5_3	5.851	7.713	8.372	-1.470	-2.944	-1.621	6.074	5.406	7.915	5.619	5.494	6.110	6.362	6.720	7.745
v6_3	5.773	7.447	8.039	-1.163	-2.666	-1.330	5.965	5.367	7.612	5.559	5.449	6.001	6.218	6.539	7.458
v7_3	-.440	1.809	2.609	-4.231	-5.130	-4.361	-.271	-1.067	1.945	-.789	-.913	-.189	.017	.459	1.718
v8_3	3.931	5.892	6.587	-2.379	-3.688	-2.484	4.143	3.442	6.079	3.671	3.545	4.189	4.432	4.811	5.896
vvv3_3	-2.410	.216	1.151	-4.909	-5.777	-5.034	-1.912	-2.664	.288	-2.856	-2.985	-2.150	-1.962	-1.437	.053
v9_3	-.928	1.939	2.961	-4.452	-5.403	-4.582	-.756	-1.822	2.199	-1.388	-1.539	-.618	-.366	.209	1.846
v10_3	-4.007	-.785	.367	-5.266	-6.065	-5.389	-3.752	-5.066	-.694	-4.614	-4.748	-3.739	-3.591	-2.928	-1.052
v11_3	-6.151	-2.757	-1.540	-5.758	-6.464	-5.876	-6.182	-6.970	-2.784	-6.865	-6.975	-5.936	-5.900	-5.189	-3.189
v12_3	-.078	2.718	3.714	-4.195	-5.193	-4.327	.143	-.856	2.863	-.509	-.664	.240	.513	1.071	2.662
vvv4_3	.739	2.762	3.480	-3.718	-4.796	-3.850	.910	.193	2.898	.381	.278	.856	1.179	1.573	2.698
v13_3	1.730	4.116	4.963	-3.505	-4.625	-3.641	1.957	1.105	4.327	1.436	1.290	2.087	2.294	2.761	4.098
v14_3	1.831	4.083	4.882	-3.407	-4.543	-3.544	2.045	1.242	4.274	1.468	1.378	2.108	2.361	2.801	4.058
v15_3	.934	3.300	4.140	-3.774	-4.846	-3.908	1.142	.300	3.486	.586	.433	1.215	1.464	1.929	3.254
v16_3	3.882	5.865	6.568	-2.422	-3.720	-2.564	4.096	3.387	6.055	3.617	3.495	4.051	4.389	4.772	5.870
vvv5_3	-1.086	1.318	2.173	-4.480	-5.426	-4.608	-.920	-1.770	1.453	-1.470	-1.598	-.827	-.542	-.127	1.077
v17_3	-1.738	1.252	2.319	-4.684	-5.592	-4.812	-1.559	-2.622	1.433	-2.238	-2.388	-1.433	-1.234	-.613	1.150
v18_3	-1.562	1.351	2.390	-4.637	-5.553	-4.764	-1.380	-2.415	1.529	-2.043	-2.192	-1.260	-1.000	-.439	1.228
v19_3	-2.398	.663	1.755	-4.863	-5.737	-4.988	-2.241	-3.326	.821	-2.928	-3.074	-2.102	-1.886	-1.232	.497
v20_3	2.157	4.594	5.460	-3.380	-4.523	-3.518	2.399	1.527	4.827	1.817	1.668	2.465	2.749	3.229	4.518
vvv6_3	4.070	5.627	6.179	-1.809	-3.179	-1.951	4.229	3.676	5.754	3.858	3.761	4.270	4.452	4.751	5.606
v21_3	.111	2.676	3.588	-4.101	-5.115	-4.233	.318	-.595	2.868	-.279	-.423	.406	.658	1.165	2.613
v22_3	1.880	4.202	5.027	-3.424	-4.557	-3.560	2.102	1.273	4.406	1.551	1.410	2.168	2.431	2.885	4.184
v23_3	-.418	2.243	3.190	-4.284	-5.265	-4.414	-.218	-1.165	2.431	-.833	-.979	-.121	.127	.656	2.164
v24_3	4.169	6.185	6.899	-2.338	-3.652	-2.481	4.392	3.671	6.386	3.905	3.775	4.438	4.694	5.083	6.199
i1_3	44.081	47.635	48.873	7.368	4.222	7.128	46.167	44.634	50.311	44.724	44.046	45.599	47.886	48.501	50.431

	a1_1	a2_1	a3_1	a4_1	a5_1	a6_1	a7_1	a8_1	a9_1	a10_1	a11_1	a12_1	a13_1	a14_1	a15_1	
**i2_3**	42.018	45.521	46.742	6.925	3.863	6.689	43.983	42.485	48.034	42.592	41.949	43.458	45.610	46.221	48.127	[illegible]
**i3_3**	41.066	44.545	45.758	6.720	3.696	6.485	42.975	41.494	46.983	41.609	40.981	42.470	44.561	45.169	47.064	[illegible]
**i4_3**	38.516	41.788	42.930	6.557	3.576	6.326	40.149	38.784	43.846	38.923	38.375	39.738	41.516	42.094	43.874	[illegible]
**i5_3**	37.709	41.108	42.295	5.970	3.089	5.743	39.431	38.006	43.297	38.146	37.571	38.993	40.879	41.481	43.337	[illegible]
**i6_3**	25.982	28.902	29.928	3.659	1.227	3.456	26.991	25.841	30.134	26.051	25.681	26.797	27.898	28.435	30.040	[illegible]
**i7_3**	20.787	23.826	24.896	1.813	-.288	1.626	21.716	20.533	24.965	20.776	20.424	21.560	22.583	23.152	24.840	[illegible]
**i8_3**	28.941	32.018	33.098	4.193	1.655	3.984	30.135	28.902	33.498	29.099	28.674	29.880	31.184	31.745	33.435	[illegible]
**i9_3**	46.546	50.140	51.390	7.955	4.698	7.709	48.758	47.193	52.982	47.264	46.552	48.144	50.569	51.185	53.131	[illegible]
**i10_3**	45.508	49.104	50.355	7.657	4.455	7.413	47.687	46.125	51.904	46.204	45.500	47.086	49.476	50.094	52.044	[illegible]
**i11_3**	46.192	49.762	51.003	7.924	4.674	7.678	48.365	46.814	52.551	46.890	46.188	47.764	50.146	50.759	52.692	[illegible]
**i12_3**	48.762	52.473	53.762	8.238	4.923	7.989	51.184	49.544	55.602	49.592	48.819	50.495	53.158	53.788	55.796	[illegible]
**i13_3**	22.722	25.814	26.902	2.289	.098	2.097	23.739	22.525	27.072	22.758	22.380	23.552	24.673	25.248	26.963	[illegible]
**i14_3**	16.142	18.989	19.994	.725	-1.170	.549	16.845	15.763	19.826	16.022	15.737	16.763	17.540	18.080	19.664	[illegible]
**i15_3**	17.830	20.710	21.726	1.194	-.788	1.013	18.594	17.492	21.628	17.744	17.441	18.491	19.334	19.878	21.478	[illegible]
**i16_3**	14.781	17.584	18.575	.369	-1.460	.196	15.429	14.371	18.349	14.635	14.365	15.365	16.084	16.617	18.178	[illegible]
**i17_3**	44.757	48.302	49.536	7.589	4.402	7.346	46.856	45.325	50.992	45.411	44.731	46.282	48.582	49.193	51.116	[illegible]
**i18_3**	45.461	48.890	50.083	8.136	4.855	7.889	47.460	45.986	51.436	46.076	45.425	46.919	49.098	49.690	51.543	[illegible]
**i19_3**	49.951	53.519	54.758	9.004	5.556	8.749	52.246	50.679	56.462	50.730	49.996	51.599	54.107	54.712	56.633	[illegible]
**i20_3**	49.036	52.498	53.701	9.079	5.623	8.823	51.182	49.676	55.235	49.741	49.050	50.586	52.924	53.514	55.377	[illegible]
**i21_3**	13.724	16.593	17.608	-.058	-1.812	-.227	14.368	13.286	17.358	13.560	13.289	14.309	15.027	15.575	17.178	[illegible]
**i22_3**	16.757	19.600	20.603	.925	-1.006	.747	17.475	16.393	20.456	16.649	16.359	17.387	18.180	18.718	20.298	[illegible]
**i23_3**	17.105	20.052	21.092	.860	-1.064	.682	17.879	16.751	20.987	17.011	16.704	17.776	18.633	19.191	20.832	[illegible]
**i24_3**	14.842	17.680	18.683	.333	-1.490	.161	15.506	14.432	18.467	14.698	14.423	15.438	16.174	16.714	18.296	[illegible]

Bootstrap (Measurement weights)

Summary of Bootstrap Iterations (Measurement weights)

(Measurement weights)

Iterations	Method 0	Method 1	Method 2
**1**	0	0	0
**2**	0	0	0
**3**	0	0	0
**4**	0	0	0
**5**	0	0	0
**6**	0	0	0
**7**	0	0	0
**8**	0	0	0
**9**	0	1	0
**10**	0	8	0
**11**	0	25	0
**12**	0	26	0
**13**	0	24	0
**14**	0	19	0
**15**	0	25	0
**16**	0	19	0
**17**	0	11	0
**18**	0	7	0
**19**	0	35	0
**Total**	0	200	0

0 bootstrap samples were unused because of a singular covariance matrix.
0 bootstrap samples were unused because a solution was not found.
200 usable bootstrap samples were obtained.

Bootstrap Distributions (Measurement weights)

ML discrepancy (implied vs sample) (Measurement weights)

```
                            |--------------------
                 2919.415   |*
                 2984.320   |*
                 3049.226   |**
                 3114.131   |*******
                 3179.036   |*********
                 3243.942   |******
                 3308.847   |*********
N = 200          3373.753   |**************
Mean = 3324.047  3438.658   |*******
S. e. = 11.920   3503.563   |******
```

```
                    3568.469   |***
                    3633.374   |**
                    3698.280   |*
                    3763.185   |*
                    3828.090   |*
                               |--------------------
```

ML discrepancy (implied vs pop) (Measurement weights)

```
                               |--------------------
                    2559.617   |*
                    2579.017   |*****
                    2598.417   |**********
                    2617.818   |*************
                    2637.218   |***************
                    2656.618   |**********
                    2676.018   |***************
N = 200             2695.419   |************
Mean = 2664.258     2714.819   |**********
S. e. = 3.629       2734.219   |********
                    2753.619   |**
                    2773.020   |**
                    2792.420   |**
                    2811.820   |
                    2831.220   |*
                               |--------------------
```

K-L overoptimism (unstabilized) (Measurement weights)

```
                               |--------------------
                   -1412.314   |*
                   -1085.190   |*
                    -758.066   |***
                    -430.942   |*******
                    -103.818   |*******
                     223.307   |****************
                     550.431   |******************
N = 200              877.555   |********************
Mean = 710.565      1204.679   |**********
S. e. = 52.787      1531.803   |*********
                    1858.927   |*******
                    2186.051   |**
                    2513.175   |**
                    2840.299   |
                    3167.423   |*
                               |--------------------
```

K-L overoptimism (stabilized) (Measurement weights)

```
                               |--------------------
                     258.555   |*
                     326.735   |***
                     394.916   |****
                     463.097   |*******
                     531.277   |**********
                     599.458   |**************
                     667.639   |*****************
N = 200              735.819   |*****************
Mean = 691.090       804.000   |******
S. e. = 12.855       872.181   |***********
                     940.362   |*****
                    1008.542   |****
                    1076.723   |**
                    1144.904   |*
                    1213.084   |*
                               |--------------------
```

Measurement intercepts (Measurement intercepts)

Notes for Model (Measurement intercepts)

Computation of degrees of freedom (Measurement intercepts)

**Number of distinct sample moments:**	972
**Number of distinct parameters to be estimated:**	177
**Degrees of freedom (972 - 177):**	795

Result (Measurement intercepts)

Minimum was achieved
Chi-square = 2457.111
Degrees of freedom = 795
Probability level = .000

g1 (g1 - Measurement intercepts)

Estimates (g1 - Measurement intercepts)

Scalar Estimates (g1 - Measurement intercepts)

Maximum Likelihood Estimates

Regression Weights: (g1 - Measurement intercepts)

			Estimate	S.E.	C.R.	P	Label
BPNSF19	<---	F1	1.000				
BPNSF13	<---	F1	1.144	.052	22.216	***	a1_1
BPNSF7	<---	F1	.863	.050	17.162	***	a2_1
BPNSF1	<---	F1	.759	.050	15.166	***	a3_1
BPNSF18	<---	F2	1.000				
BPNSF15	<---	F2	2.454	.303	8.092	***	a4_1
BPNSF10	<---	F2	3.105	.370	8.386	***	a5_1
BPNSF5	<---	F2	2.516	.307	8.199	***	a6_1
BPNSF24	<---	F3	1.000				
BPNSF16	<---	F3	1.124	.046	24.274	***	a7_1
BPNSF12	<---	F3	1.221	.048	25.694	***	a8_1
BPNSF4	<---	F3	.846	.044	19.431	***	a9_1
BPNSF22	<---	F4	1.000				
BPNSF20	<---	F4	1.192	.049	24.555	***	a10_1
BPNSF8	<---	F4	1.197	.050	23.990	***	a11_1
BPNSF2	<---	F4	1.120	.049	23.055	***	a12_1
BPNSF21	<---	F5	1.000				
BPNSF14	<---	F5	1.094	.042	26.011	***	a13_1
BPNSF9	<---	F5	1.038	.042	24.673	***	a14_1
BPNSF3	<---	F5	.882	.042	21.109	***	a15_1
BPNSF23	<---	F6	1.000				
BPNSF17	<---	F6	.976	.035	28.159	***	a16_1
BPNSF11	<---	F6	.959	.034	28.209	***	a17_1
BPNSF6	<---	F6	.861	.035	24.551	***	a18_1

Standardized Regression Weights: (g1 - Measurement intercepts)

			Estimate
BPNSF19	<---	F1	.650
BPNSF13	<---	F1	.765
BPNSF7	<---	F1	.522
BPNSF1	<---	F1	.456
BPNSF18	<---	F2	.244
BPNSF15	<---	F2	.582
BPNSF10	<---	F2	.693
BPNSF5	<---	F2	.596
BPNSF24	<---	F3	.691
BPNSF16	<---	F3	.744
BPNSF12	<---	F3	.797
BPNSF4	<---	F3	.582
BPNSF22	<---	F4	.681
BPNSF20	<---	F4	.768
BPNSF8	<---	F4	.731
BPNSF2	<---	F4	.703
BPNSF21	<---	F5	.692
BPNSF14	<---	F5	.770

			Estimate
BPNSF9	<---	F5	.714
BPNSF3	<---	F5	.632
BPNSF23	<---	F6	.799
BPNSF17	<---	F6	.727
BPNSF11	<---	F6	.744
BPNSF6	<---	F6	.681

Intercepts: (g1 - Measurement intercepts)

	Estimate	S.E.	C.R.	P	Label
**BPNSF19**	5.140	.040	127.940	***	i1_1
**BPNSF13**	5.043	.039	130.871	***	i2_1
**BPNSF7**	4.817	.040	119.340	***	i3_1
**BPNSF1**	4.705	.041	114.698	***	i4_1
**BPNSF18**	4.316	.041	104.417	***	i5_1
**BPNSF15**	3.699	.046	81.096	***	i6_1
**BPNSF10**	3.158	.045	69.442	***	i7_1
**BPNSF5**	3.721	.044	85.234	***	i8_1
**BPNSF24**	5.225	.037	140.058	***	i9_1
**BPNSF16**	5.118	.038	134.993	***	i10_1
**BPNSF12**	5.220	.038	136.344	***	i11_1
**BPNSF4**	5.116	.037	139.813	***	i12_1
**BPNSF22**	3.135	.043	73.443	***	i13_1
**BPNSF20**	2.524	.046	55.295	***	i14_1
**BPNSF8**	2.823	.047	59.807	***	i15_1
**BPNSF2**	2.400	.046	52.041	***	i16_1
**BPNSF21**	5.178	.039	131.278	***	i17_1
**BPNSF14**	5.441	.040	137.076	***	i18_1
**BPNSF9**	5.657	.040	141.770	***	i19_1
**BPNSF3**	5.591	.040	140.422	***	i20_1
**BPNSF23**	2.250	.045	49.691	***	i21_1
**BPNSF17**	2.586	.047	55.085	***	i22_1
**BPNSF11**	2.754	.046	59.623	***	i23_1
**BPNSF6**	2.574	.047	55.227	***	i24_1

Covariances: (g1 - Measurement intercepts)

			Estimate	S.E.	C.R.	P	Label
F1	<-->	F2	-.076	.025	-3.043	.002	ccc1_1
F2	<-->	F3	-.102	.024	-4.298	***	ccc2_1
F1	<-->	F3	.585	.061	9.560	***	ccc3_1
F2	<-->	F4	.209	.035	5.916	***	ccc4_1
F3	<-->	F4	-.462	.057	-8.050	***	ccc5_1
F1	<-->	F4	-.415	.063	-6.576	***	ccc6_1
F2	<-->	F5	-.058	.025	-2.370	.018	ccc7_1
F4	<-->	F5	-.629	.070	-8.987	***	ccc8_1
F3	<-->	F5	.613	.062	9.860	***	ccc9_1
F1	<-->	F5	.880	.079	11.080	***	ccc10_1
F6	<-->	F5	-.879	.088	-9.992	***	ccc11_1
F6	<-->	F3	-.503	.068	-7.401	***	ccc12_1
F6	<-->	F4	1.155	.099	11.669	***	ccc13_1
F6	<-->	F2	.282	.045	6.200	***	ccc14_1
F6	<-->	F1	-.586	.079	-7.429	***	ccc15_1

Correlations: (g1 - Measurement intercepts)

			Estimate
F1	<-->	F2	-.224
F2	<-->	F3	-.330
F1	<-->	F3	.743
F2	<-->	F4	.574
F3	<-->	F4	-.545
F1	<-->	F4	-.447
F2	<-->	F5	-.161
F4	<-->	F5	-.636
F3	<-->	F5	.731
F1	<-->	F5	.958
F6	<-->	F5	-.713
F6	<-->	F3	-.476
F6	<-->	F4	.927

			Estimate
F6	<-->	F2	.621
F6	<-->	F1	-.506

Variances: (g1 - Measurement intercepts)

	Estimate	S.E.	C.R.	P	Label
**F1**	.863	.097	8.916	***	vvv1_1
**F2**	.133	.033	4.029	***	vvv2_1
**F3**	.719	.072	9.950	***	vvv3_1
**F4**	.999	.101	9.921	***	vvv4_1
**F5**	.979	.097	10.046	***	vvv5_1
**F6**	1.553	.139	11.156	***	vvv6_1
**e1**	1.182	.090	13.072	***	v1_1
**e2**	.798	.073	10.951	***	v2_1
**e3**	1.715	.123	13.979	***	v3_1
**e4**	1.896	.133	14.237	***	v4_1
**e5**	2.086	.144	14.531	***	v5_1
**e6**	1.562	.125	12.527	***	v6_1
**e7**	1.384	.131	10.532	***	v7_1
**e8**	1.521	.123	12.355	***	v8_1
**e9**	.788	.063	12.566	***	v9_1
**e10**	.734	.062	11.749	***	v10_1
**e11**	.614	.059	10.467	***	v11_1
**e12**	1.003	.074	13.564	***	v12_1
**e13**	1.156	.088	13.169	***	v13_1
**e14**	.988	.082	11.993	***	v14_1
**e15**	1.244	.099	12.597	***	v15_1
**e16**	1.282	.099	12.945	***	v16_1
**e17**	1.066	.080	13.401	***	v17_1
**e18**	.805	.065	12.399	***	v18_1
**e19**	1.017	.077	13.190	***	v19_1
**e20**	1.142	.083	13.818	***	v20_1
**e21**	.878	.074	11.864	***	v21_1
**e22**	1.321	.101	13.020	***	v22_1
**e23**	1.150	.090	12.808	***	v23_1
**e24**	1.332	.099	13.438	***	v24_1

Matrices (g1 - Measurement intercepts)

Residual Covariances (g1 - Measurement intercepts)

	BPNSF6	BPNSF11	BPNSF17	BPNSF23	BPNSF3	BPNSF9	BPNSF14	BPNSF21	BPNSF2	BPNSF8	BPNSF20	BPNSF22	BPNSF4	BPNSF12	BPNSF16	BPNS[illegible]
**BPNSF6**	.170															
**BPNSF11**	.183	.106														
**BPNSF17**	.102	-.054	-.244													
**BPNSF23**	.036	-.028	-.190	-.090												
**BPNSF3**	-.191	-.032	.010	-.205	-.014											
**BPNSF9**	-.121	.027	.035	-.086	.041	-.143										
**BPNSF14**	.025	.204	.082	.041	-.015	-.020	.043									
**BPNSF21**	.070	.114	.273	-.007	-.122	-.107	.022	-.006								
**BPNSF2**	.150	.140	-.176	.147	-.318	-.151	-.129	-.115	.117							
**BPNSF8**	.256	.173	-.078	-.193	.073	-.044	.192	.331	.070	.032						
**BPNSF20**	.000	-.133	-.062	.072	-.152	-.029	-.016	.015	-.006	-.121	-.112					
**BPNSF22**	-.085	-.075	-.330	-.077	-.035	.113	.270	.204	.009	.113	-.089	-.083				
**BPNSF4**	-.136	.040	.054	.072	.169	-.093	.126	-.095	-.120	.090	-.002	.031	-.055			
**BPNSF12**	-.082	.195	.041	.109	-.057	-.068	.073	-.082	.009	.139	.066	-.024	-.068	.012		
**BPNSF16**	-.204	-.013	-.061	-.093	-.050	.002	.061	.010	-.022	.037	.002	.031	-.042	.014	-.004	
**BPNSF24**	-.077	.038	-.037	-.053	.071	.113	-.078	-.070	-.201	.008	-.080	-.129	-.028	.032	.027	[illegible]
**BPNSF5**	.174	.253	-.046	-.059	-.055	-.142	-.054	-.136	.199	.146	-.014	.085	.175	-.105	-.178	[illegible]
**BPNSF10**	.132	.555	-.059	-.114	-.051	-.192	.023	-.093	.184	.317	-.125	-.095	.018	-.059	-.025	[illegible]
**BPNSF15**	.005	.115	-.118	-.325	-.084	-.011	.323	-.054	-.198	.052	-.149	-.063	.082	-.057	.175	[illegible]
**BPNSF18**	-.400	-.209	-.166	-.347	.238	.315	.120	.447	-.183	-.104	-.340	-.078	.112	.232	.307	[illegible]
**BPNSF1**	.103	.158	.348	.200	.019	-.301	-.372	-.082	.188	.242	.157	.132	.235	.009	-.050	[illegible]
**BPNSF7**	.167	.053	.363	.038	-.073	.011	-.179	.120	-.154	.338	.197	.172	-.118	-.230	-.231	[illegible]
**BPNSF13**	-.063	.068	.074	.001	-.163	-.196	.193	.043	-.117	.185	-.081	.096	.017	.121	.018	[illegible]
**BPNSF19**	-.061	-.117	-.112	-.262	-.036	-.143	.060	.372	-.347	.071	-.245	-.014	-.045	.020	.098	[illegible]

Residual Means (g1 - Measurement intercepts)

	BPNSF6	BPNSF11	BPNSF17	BPNSF23	BPNSF3	BPNSF9	BPNSF14	BPNSF21	BPNSF2	BPNSF8	BPNSF20	BPNSF22	BPNSF4	BPNSF12	BPNSF16	BPNSF24	BP[illegible]

	BPNSF6	BPNSF11	BPNSF17	BPNSF23	BPNSF3	BPNSF9	BPNSF14	BPNSF21	BPNSF2	BPNSF8	BPNSF20	BPNSF22	BPNSF4	BPNSF12	BPNSF16	BPNSF24	BP[illegible]
	-.116	-.064	-.146	-.181	.125	.191	.144	.180	-.170	-.055	-.173	-.119	-.048	.010	-.006	.018	-.[illegible]

Standardized Residual Covariances (g1 - Measurement intercepts)

	BPNSF6	BPNSF11	BPNSF17	BPNSF23	BPNSF3	BPNSF9	BPNSF14	BPNSF21	BPNSF2	BPNSF8	BPNSF20	BPNSF22	BPNSF4	BPNSF12	BPNSF16	BPNS[illegible]
**BPNSF6**	1.012															
**BPNSF11**	1.350	.611														
**BPNSF17**	.729	-.371	-1.289													
**BPNSF23**	.272	-.202	-1.317	-.549												
**BPNSF3**	-1.756	-.289	.089	-1.880	-.112											
**BPNSF9**	-1.057	.228	.283	-.741	.389	-1.021										
**BPNSF14**	.224	1.750	.677	.355	-.144	-.182	.322									
**BPNSF21**	.620	.973	2.250	-.063	-1.181	-.972	.201	-.047								
**BPNSF2**	1.147	1.030	-1.247	1.101	-2.918	-1.313	-1.142	-1.008	.685							
**BPNSF8**	1.887	1.232	-.534	-1.390	.653	-.370	1.648	2.819	.504	.178						
**BPNSF20**	-.002	-.985	-.441	.539	-1.420	-.260	-.145	.130	-.042	-.869	-.688					
**BPNSF22**	-.704	-.606	-2.557	-.631	-.344	1.072	2.600	1.950	.071	.879	-.721	-.568				
**BPNSF4**	-1.445	.410	.541	.769	2.014	-1.055	1.445	-1.078	-1.252	.907	-.021	.346	-.537			
**BPNSF12**	-.812	1.888	.384	1.076	-.630	-.705	.765	-.858	.091	1.303	.650	-.252	-.809	.106		
**BPNSF16**	-2.054	-.129	-.572	-.941	-.556	.025	.658	.112	-.221	.358	.021	.335	-.516	.153	-.037	
**BPNSF24**	-.812	.396	-.363	-.564	.838	1.260	-.883	-.787	-2.085	.084	-.842	-1.446	-.362	.363	.323	.2[illegible]
**BPNSF5**	1.461	2.070	-.360	-.497	-.545	-1.343	-.517	-1.291	1.654	1.181	-.116	.772	1.917	-1.085	-1.870	-1.8[illegible]
**BPNSF10**	1.030	4.222	-.432	-.889	-.469	-1.704	.212	-.833	1.425	2.390	-.988	-.805	.182	-.575	-.249	.7[illegible]
**BPNSF15**	.039	.945	-.932	-2.725	-.829	-.105	3.123	-.511	-1.650	.417	-1.268	-.567	.904	-.589	1.838	.3[illegible]
**BPNSF18**	-3.545	-1.815	-1.385	-3.102	2.419	3.070	1.197	4.392	-1.607	-.889	-3.061	-.745	1.281	2.508	3.354	1.9[illegible]
**BPNSF1**	.877	1.309	2.775	1.702	.178	-2.697	-3.394	-.739	1.585	1.977	1.350	1.209	2.535	.089	-.511	.4[illegible]
**BPNSF7**	1.419	.443	2.907	.324	-.692	.098	-1.622	1.084	-1.303	2.777	1.706	1.581	-1.274	-2.309	-2.364	-1.3[illegible]
**BPNSF13**	-.585	.615	.638	.013	-1.613	-1.816	1.805	.407	-1.082	1.655	-.765	.963	.198	1.282	.192	-.5[illegible]
**BPNSF19**	-.554	-1.038	-.955	-2.379	-.357	-1.331	.567	3.494	-3.125	.619	-2.259	-.132	-.509	.214	1.058	.0[illegible]

Standardized Residual Means (g1 - Measurement intercepts)

	BPNSF6	BPNSF11	BPNSF17	BPNSF23	BPNSF3	BPNSF9	BPNSF14	BPNSF21	BPNSF2	BPNSF8	BPNSF20	BPNSF22	BPNSF4	BPNSF12	BPNSF16	BPNSF24	BP[illegible]
	-1.542	-.830	-1.826	-2.434	1.892	2.775	2.151	2.639	-2.229	-.706	-2.338	-1.702	-.807	.166	-.097	.312	-.[illegible]

Notes for Group/Model (g1 - Measurement intercepts)

The following covariance matrix is not positive definite (g1 - Measurement intercepts)

	F5	F4	F3	F2	F1	F6
**F5**	.979					
**F4**	-.629	.999				
**F3**	.613	-.462	.719			
**F2**	-.058	.209	-.102	.133		
**F1**	.880	-.415	.585	-.076	.863	
**F6**	-.879	1.155	-.503	.282	-.586	1.553

This solution is not admissible.

Modification Indices (g1 - Measurement intercepts)

Covariances: (g1 - Measurement intercepts)

			M.I.	Par Change
e24	<-->	F3	4.927	-.090
e23	<-->	F3	4.914	.085
e23	<-->	F2	27.194	.105
e21	<-->	F2	12.328	-.063
e19	<-->	F1	12.115	-.119
e19	<-->	e20	5.195	.126
e17	<-->	F3	6.134	-.089
e17	<-->	F1	21.192	.161
e17	<-->	e22	5.447	.146
e17	<-->	e20	5.108	-.128
e16	<-->	F5	5.054	-.079
e16	<-->	e22	8.491	-.203
e15	<-->	F2	7.523	.058
e15	<-->	F1	5.845	.097
e15	<-->	e24	6.599	.177
e15	<-->	e23	6.884	.171

			M.I.	Par Change
e15	<-->	e21	18.165	-.250
e15	<-->	e19	7.676	-.168
e15	<-->	e17	5.070	.139
e14	<-->	F2	5.752	-.046
e14	<-->	e23	8.993	-.178
e14	<-->	e21	14.559	.203
e13	<-->	F5	7.398	.090
e13	<-->	F4	4.066	.071
e13	<-->	F3	4.056	-.077
e13	<-->	e22	6.022	-.162
e13	<-->	e18	8.257	.150
e13	<-->	e15	8.155	.184
e12	<-->	e20	10.835	.181
e12	<-->	e19	4.503	-.112
e12	<-->	e18	5.102	.108
e11	<-->	F2	5.488	-.038
e11	<-->	F6	4.457	.072
e11	<-->	e23	6.728	.130
e11	<-->	e18	5.162	.095
e11	<-->	e13	4.355	-.103
e10	<-->	F4	5.481	.073
e10	<-->	F6	10.943	-.117
e10	<-->	e16	4.276	.113
e9	<-->	F4	5.349	-.072
e9	<-->	e19	11.590	.164
e9	<-->	e18	11.344	-.148
e8	<-->	e12	12.643	.236
e7	<-->	e23	30.048	.403
e7	<-->	e15	7.010	.204
e7	<-->	e14	5.563	-.166
e7	<-->	e13	7.355	-.198
e7	<-->	e9	5.885	.149
e7	<-->	e8	5.806	-.202
e6	<-->	F2	5.652	.053
e6	<-->	e21	7.746	-.184
e6	<-->	e18	16.037	.246
e6	<-->	e16	5.897	-.187
e6	<-->	e11	5.249	-.134
e6	<-->	e10	10.948	.202
e5	<-->	F1	6.026	.119
e5	<-->	e24	7.462	-.229
e5	<-->	e18	13.102	-.242
e5	<-->	e17	6.983	.198
e4	<-->	F3	5.359	.109
e4	<-->	F2	27.855	-.131
e4	<-->	e22	4.235	.168
e4	<-->	e20	10.164	.235
e4	<-->	e18	12.469	-.226
e4	<-->	e16	5.926	.196
e4	<-->	e12	15.375	.274
e4	<-->	e7	5.429	-.210
e4	<-->	e6	15.199	-.350
e3	<-->	F3	15.861	-.179
e3	<-->	F1	8.622	.126
e3	<-->	e22	8.311	.225
e3	<-->	e18	10.399	-.198
e3	<-->	e17	4.368	.144
e3	<-->	e16	7.573	-.212
e3	<-->	e11	5.063	-.132
e3	<-->	e10	4.475	-.129
e3	<-->	e8	4.604	-.183
e3	<-->	e4	4.490	.190
e2	<-->	F2	7.605	.048
e2	<-->	e20	9.365	-.158
e2	<-->	e19	10.140	-.157
e2	<-->	e18	19.705	.198
e2	<-->	e11	10.642	.141
e1	<-->	e19	5.986	-.141
e1	<-->	e17	34.035	.341
e1	<-->	e5	7.098	.212

Variances: (g1 - Measurement intercepts)

	M.I.	Par Change

Regression Weights: (g1 - Measurement intercepts)

			M.I.	Par Change
BPNSF6	<---	F3	4.250	-.152
BPNSF11	<---	F2	19.707	.772
BPNSF11	<---	BPNSF8	4.567	.036
BPNSF11	<---	BPNSF5	4.592	.029
BPNSF11	<---	BPNSF10	15.494	.061
BPNSF11	<---	BPNSF15	4.926	.030
BPNSF17	<---	BPNSF2	4.169	-.041
BPNSF23	<---	F2	13.133	-.567
BPNSF23	<---	BPNSF8	6.623	-.039
BPNSF23	<---	BPNSF10	6.553	-.035
BPNSF23	<---	BPNSF15	7.126	-.033
BPNSF3	<---	F4	4.145	-.114
BPNSF14	<---	F2	4.631	.314
BPNSF21	<---	BPNSF17	4.156	.034
BPNSF21	<---	BPNSF8	4.278	.033
BPNSF21	<---	BPNSF19	5.058	.022
BPNSF2	<---	F5	9.162	-.184
BPNSF2	<---	F1	11.019	-.220
BPNSF2	<---	BPNSF3	4.138	-.020
BPNSF2	<---	BPNSF21	4.149	-.022
BPNSF2	<---	BPNSF7	5.764	-.027
BPNSF2	<---	BPNSF19	4.327	-.022
BPNSF8	<---	F5	7.671	.168
BPNSF8	<---	F2	6.097	.451
BPNSF8	<---	F1	8.305	.191
BPNSF8	<---	BPNSF6	4.006	.038
BPNSF8	<---	BPNSF21	4.011	.021
BPNSF8	<---	BPNSF10	6.031	.040
BPNSF20	<---	F2	5.521	-.390
BPNSF20	<---	BPNSF11	5.478	-.038
BPNSF20	<---	BPNSF8	4.618	-.034
BPNSF20	<---	BPNSF10	6.738	-.038
BPNSF5	<---	F5	4.460	-.142
BPNSF10	<---	BPNSF6	4.191	.044
BPNSF10	<---	BPNSF11	15.603	.080
BPNSF10	<---	BPNSF3	4.343	.023
BPNSF10	<---	BPNSF2	7.088	.060
BPNSF10	<---	BPNSF8	8.892	.059
BPNSF10	<---	BPNSF24	5.578	.028
BPNSF10	<---	BPNSF15	6.291	.040
BPNSF10	<---	BPNSF7	5.595	.030
BPNSF10	<---	BPNSF13	4.057	.025
BPNSF10	<---	BPNSF19	5.165	.027
BPNSF15	<---	BPNSF14	4.066	.023
BPNSF18	<---	F5	19.177	.322
BPNSF18	<---	F4	8.515	-.216
BPNSF18	<---	F3	12.952	.321
BPNSF18	<---	F1	18.263	.344
BPNSF18	<---	F6	10.398	-.190
BPNSF18	<---	BPNSF21	4.170	.026
BPNSF1	<---	F5	11.183	-.236
BPNSF1	<---	F4	4.909	.158
BPNSF1	<---	F2	7.734	-.594
BPNSF1	<---	F6	5.528	.133
BPNSF1	<---	BPNSF3	7.070	-.031
BPNSF1	<---	BPNSF9	12.020	-.040
BPNSF1	<---	BPNSF14	15.100	-.046
BPNSF1	<---	BPNSF21	9.771	-.039
BPNSF1	<---	BPNSF22	4.750	-.042
BPNSF1	<---	BPNSF4	4.171	-.026
BPNSF1	<---	BPNSF12	7.533	-.034
BPNSF1	<---	BPNSF16	8.363	-.037
BPNSF1	<---	BPNSF24	6.768	-.032
BPNSF1	<---	BPNSF5	11.471	-.056
BPNSF1	<---	BPNSF10	15.606	-.074
BPNSF1	<---	BPNSF15	18.341	-.072
BPNSF1	<---	BPNSF18	8.891	-.044

			M.I.	Par Change
BPNSF1	<---	BPNSF7	5.944	-.032
BPNSF1	<---	BPNSF13	10.102	-.041
BPNSF1	<---	BPNSF19	11.649	-.043
BPNSF7	<---	F4	4.059	.138
BPNSF7	<---	F3	6.141	-.204
BPNSF7	<---	BPNSF17	4.810	.046
BPNSF19	<---	F4	7.816	-.163
BPNSF19	<---	F6	8.246	-.133
BPNSF19	<---	BPNSF21	8.247	.029
BPNSF19	<---	BPNSF18	4.915	.027

Means: (g1 - Measurement intercepts)

	M.I.	Par Change

Intercepts: (g1 - Measurement intercepts)

	M.I.	Par Change
**BPNSF10**	4.283	.133
**BPNSF1**	8.017	-.190

Bootstrap (g1 - Measurement intercepts)

Bootstrap standard errors (g1 - Measurement intercepts)

Scalar Estimates (g1 - Measurement intercepts)

Regression Weights: (g1 - Measurement intercepts)

Parameter			SE	SE-SE	Mean	Bias	SE-Bias
BPNSF19	<---	F1	.000	.000	1.000	.000	.000
BPNSF13	<---	F1	.057	.003	1.139	-.005	.004
BPNSF7	<---	F1	.059	.003	.862	.000	.004
BPNSF1	<---	F1	.071	.004	.758	-.001	.005
BPNSF18	<---	F2	.000	.000	1.000	.000	.000
BPNSF15	<---	F2	.387	.019	2.498	.043	.027
BPNSF10	<---	F2	.513	.026	3.163	.058	.036
BPNSF5	<---	F2	.410	.021	2.563	.047	.029
BPNSF24	<---	F3	.000	.000	1.000	.000	.000
BPNSF16	<---	F3	.054	.003	1.125	.000	.004
BPNSF12	<---	F3	.049	.002	1.215	-.006	.003
BPNSF4	<---	F3	.051	.003	.844	-.002	.004
BPNSF22	<---	F4	.000	.000	1.000	.000	.000
BPNSF20	<---	F4	.051	.003	1.191	-.001	.004
BPNSF8	<---	F4	.050	.002	1.191	-.005	.004
BPNSF2	<---	F4	.054	.003	1.118	-.002	.004
BPNSF21	<---	F5	.000	.000	1.000	.000	.000
BPNSF14	<---	F5	.045	.002	1.096	.002	.003
BPNSF9	<---	F5	.052	.003	1.040	.002	.004
BPNSF3	<---	F5	.059	.003	.887	.005	.004
BPNSF23	<---	F6	.000	.000	1.000	.000	.000
BPNSF17	<---	F6	.034	.002	.979	.003	.002
BPNSF11	<---	F6	.038	.002	.959	.000	.003
BPNSF6	<---	F6	.037	.002	.861	.000	.003

Standardized Regression Weights: (g1 - Measurement intercepts)

Parameter			SE	SE-SE	Mean	Bias	SE-Bias
BPNSF19	<---	F1	.039	.002	.654	.004	.003
BPNSF13	<---	F1	.031	.002	.763	-.002	.002
BPNSF7	<---	F1	.038	.002	.521	-.001	.003
BPNSF1	<---	F1	.033	.002	.455	.000	.002
BPNSF18	<---	F2	.036	.002	.246	.002	.003
BPNSF15	<---	F2	.038	.002	.583	.001	.003
BPNSF10	<---	F2	.036	.002	.692	-.001	.003
BPNSF5	<---	F2	.029	.001	.597	.000	.002
BPNSF24	<---	F3	.029	.001	.693	.002	.002
BPNSF16	<---	F3	.032	.002	.748	.004	.002
BPNSF12	<---	F3	.025	.001	.796	-.001	.002

Parameter	SE	SE-SE	Mean	Bias	SE-Bias
BPNSF4 <--- F3	.029	.001	.580	-.002	.002
BPNSF22 <--- F4	.027	.001	.680	-.001	.002
BPNSF20 <--- F4	.026	.001	.766	-.002	.002
BPNSF8 <--- F4	.031	.002	.728	-.004	.002
BPNSF2 <--- F4	.032	.002	.698	-.005	.002
BPNSF21 <--- F5	.030	.001	.690	-.001	.002
BPNSF14 <--- F5	.040	.002	.772	.002	.003
BPNSF9 <--- F5	.034	.002	.716	.002	.002
BPNSF3 <--- F5	.039	.002	.635	.003	.003
BPNSF23 <--- F6	.026	.001	.798	-.001	.002
BPNSF17 <--- F6	.029	.001	.728	.001	.002
BPNSF11 <--- F6	.029	.001	.741	-.003	.002
BPNSF6 <--- F6	.033	.002	.677	-.004	.002

Intercepts: (g1 - Measurement intercepts)

Parameter	SE	SE-SE	Mean	Bias	SE-Bias
**BPNSF19**	.045	.002	5.148	.008	.003
**BPNSF13**	.042	.002	5.052	.009	.003
**BPNSF7**	.042	.002	4.827	.009	.003
**BPNSF1**	.040	.002	4.713	.008	.003
**BPNSF18**	.041	.002	4.315	-.001	.003
**BPNSF15**	.047	.002	3.698	-.002	.003
**BPNSF10**	.051	.003	3.153	-.005	.004
**BPNSF5**	.047	.002	3.717	-.004	.003
**BPNSF24**	.041	.002	5.232	.006	.003
**BPNSF16**	.044	.002	5.125	.008	.003
**BPNSF12**	.039	.002	5.228	.008	.003
**BPNSF4**	.039	.002	5.121	.006	.003
**BPNSF22**	.047	.002	3.128	-.007	.003
**BPNSF20**	.054	.003	2.512	-.013	.004
**BPNSF8**	.055	.003	2.817	-.006	.004
**BPNSF2**	.048	.002	2.393	-.007	.003
**BPNSF21**	.042	.002	5.189	.011	.003
**BPNSF14**	.044	.002	5.452	.011	.003
**BPNSF9**	.046	.002	5.666	.009	.003
**BPNSF3**	.043	.002	5.600	.009	.003
**BPNSF23**	.046	.002	2.239	-.010	.003
**BPNSF17**	.053	.003	2.574	-.011	.004
**BPNSF11**	.049	.002	2.749	-.005	.003
**BPNSF6**	.053	.003	2.570	-.004	.004

Covariances: (g1 - Measurement intercepts)

Parameter	SE	SE-SE	Mean	Bias	SE-Bias
F1 <--> F2	.026	.001	-.075	.001	.002
F2 <--> F3	.022	.001	-.100	.002	.002
F1 <--> F3	.068	.003	.585	.000	.005
F2 <--> F4	.038	.002	.208	-.001	.003
F3 <--> F4	.051	.003	-.460	.002	.004
F1 <--> F4	.063	.003	-.408	.007	.004
F2 <--> F5	.023	.001	-.058	.001	.002
F4 <--> F5	.061	.003	-.620	.010	.004
F3 <--> F5	.066	.003	.614	.000	.005
F1 <--> F5	.102	.005	.878	-.002	.007
F6 <--> F5	.082	.004	-.867	.012	.006
F6 <--> F3	.060	.003	-.506	-.003	.004
F6 <--> F4	.102	.005	1.148	-.007	.007
F6 <--> F2	.048	.002	.281	-.001	.003
F6 <--> F1	.076	.004	-.581	.005	.005

Correlations: (g1 - Measurement intercepts)

Parameter	SE	SE-SE	Mean	Bias	SE-Bias
F1 <--> F2	.074	.004	-.222	.002	.005
F2 <--> F3	.066	.003	-.324	.007	.005
F1 <--> F3	.053	.003	.740	-.003	.004
F2 <--> F4	.062	.003	.573	-.001	.004
F3 <--> F4	.049	.002	-.544	.002	.003
F1 <--> F4	.056	.003	-.440	.007	.004

Parameter			SE	SE-SE	Mean	Bias	SE-Bias
F2	<-->	F5	.063	.003	-.163	-.001	.004
F4	<-->	F5	.047	.002	-.632	.004	.003
F3	<-->	F5	.041	.002	.732	.001	.003
F1	<-->	F5	.035	.002	.957	-.001	.003
F6	<-->	F5	.043	.002	-.711	.003	.003
F6	<-->	F3	.049	.002	-.481	-.005	.003
F6	<-->	F4	.032	.002	.928	.001	.002
F6	<-->	F2	.053	.003	.622	.001	.004
F6	<-->	F1	.049	.002	-.503	.003	.003

Variances: (g1 - Measurement intercepts)

Parameter	SE	SE-SE	Mean	Bias	SE-Bias
**F1**	.114	.006	.869	.006	.008
**F2**	.039	.002	.136	.003	.003
**F3**	.067	.003	.723	.004	.005
**F4**	.096	.005	.996	-.003	.007
**F5**	.109	.005	.973	-.006	.008
**F6**	.139	.007	1.540	-.013	.010
**e1**	.135	.007	1.158	-.024	.010
**e2**	.103	.005	.799	.001	.007
**e3**	.171	.009	1.720	.006	.012
**e4**	.146	.007	1.886	-.009	.010
**e5**	.139	.007	2.069	-.017	.010
**e6**	.149	.007	1.552	-.010	.011
**e7**	.143	.007	1.388	.004	.010
**e8**	.131	.007	1.519	-.002	.009
**e9**	.090	.004	.784	-.005	.006
**e10**	.103	.005	.720	-.013	.007
**e11**	.082	.004	.615	.000	.006
**e12**	.089	.004	1.010	.007	.006
**e13**	.103	.005	1.155	-.001	.007
**e14**	.125	.006	.994	.005	.009
**e15**	.153	.008	1.251	.007	.011
**e16**	.144	.007	1.301	.019	.010
**e17**	.113	.006	1.064	-.002	.008
**e18**	.155	.008	.792	-.012	.011
**e19**	.157	.008	1.000	-.017	.011
**e20**	.166	.008	1.129	-.013	.012
**e21**	.125	.006	.875	-.003	.009
**e22**	.175	.009	1.310	-.011	.012
**e23**	.117	.006	1.156	.006	.008
**e24**	.152	.008	1.343	.012	.011

Matrices (g1 - Measurement intercepts)

Sample Covariances - Standard Errors (g1 - Measurement intercepts)

	BPNSF6	BPNSF11	BPNSF17	BPNSF23	BPNSF3	BPNSF9	BPNSF14	BPNSF21	BPNSF2	BPNSF8	BPNSF20	BPNSF22	BPNSF4	BPNSF12	BPNSF16	BPNS[illegible]
**BPNSF6**	.189															
**BPNSF11**	.149	.158														
**BPNSF17**	.153	.153	.188													
**BPNSF23**	.149	.150	.138	.180												
**BPNSF3**	.128	.115	.109	.108	.175											
**BPNSF9**	.116	.104	.108	.109	.116	.182										
**BPNSF14**	.124	.116	.124	.117	.105	.122	.166									
**BPNSF21**	.101	.104	.121	.111	.106	.115	.137	.163								
**BPNSF2**	.154	.150	.138	.147	.101	.110	.120	.116	.159							
**BPNSF8**	.153	.148	.155	.151	.114	.115	.111	.110	.151	.169						
**BPNSF20**	.153	.147	.159	.149	.100	.106	.131	.116	.132	.154	.166					
**BPNSF22**	.121	.128	.124	.125	.095	.091	.102	.105	.112	.126	.114	.119				
**BPNSF4**	.104	.104	.097	.085	.104	.088	.074	.096	.113	.107	.091	.090	.102			
**BPNSF12**	.117	.103	.107	.091	.094	.110	.131	.120	.107	.115	.100	.092	.085	.135		
**BPNSF16**	.122	.104	.107	.108	.098	.107	.123	.114	.109	.109	.102	.082	.080	.102	.123	
**BPNSF24**	.101	.093	.103	.097	.106	.110	.102	.105	.097	.098	.090	.092	.082	.095	.094	.1[illegible]
**BPNSF5**	.124	.125	.135	.113	.092	.091	.103	.109	.112	.136	.118	.109	.088	.098	.099	.0[illegible]
**BPNSF10**	.139	.143	.144	.134	.123	.109	.126	.121	.139	.153	.136	.126	.114	.116	.112	.1[illegible]
**BPNSF15**	.123	.129	.132	.115	.124	.112	.111	.125	.134	.148	.114	.123	.098	.112	.110	.1[illegible]
**BPNSF18**	.120	.122	.124	.110	.104	.112	.117	.113	.113	.134	.102	.112	.088	.117	.111	.1[illegible]
**BPNSF1**	.124	.105	.111	.094	.112	.097	.103	.110	.110	.115	.097	.096	.091	.094	.086	.0[illegible]
**BPNSF7**	.121	.112	.114	.093	.112	.112	.109	.118	.111	.114	.097	.101	.091	.109	.100	.1[illegible]

	BPNSF6	BPNSF11	BPNSF17	BPNSF23	BPNSF3	BPNSF9	BPNSF14	BPNSF21	BPNSF2	BPNSF8	BPNSF20	BPNSF22	BPNSF4	BPNSF12	BPNSF16	BPNS[illegible]
**BPNSF13**	.117	.118	.112	.112	.098	.116	.130	.133	.116	.117	.108	.095	.088	.132	.113	[illegible]
**BPNSF19**	.107	.112	.117	.115	.112	.113	.136	.142	.121	.125	.124	.106	.099	.108	.117	[illegible]

Sample Correlations - Standard Errors (g1 - Measurement intercepts)

	BPNSF6	BPNSF11	BPNSF17	BPNSF23	BPNSF3	BPNSF9	BPNSF14	BPNSF21	BPNSF2	BPNSF8	BPNSF20	BPNSF22	BPNSF4	BPNSF12	BPNSF16	BPNS[illegible]
**BPNSF6**	.000															
**BPNSF11**	.044	.000														
**BPNSF17**	.044	.048	.000													
**BPNSF23**	.043	.040	.047	.000												
**BPNSF3**	.052	.051	.049	.043	.000											
**BPNSF9**	.051	.045	.046	.047	.053	.000										
**BPNSF14**	.052	.049	.049	.048	.053	.046	.000									
**BPNSF21**	.043	.042	.053	.044	.052	.051	.051	.000								
**BPNSF2**	.051	.044	.048	.045	.045	.047	.048	.044	.000							
**BPNSF8**	.045	.042	.049	.051	.052	.048	.048	.049	.044	.000						
**BPNSF20**	.050	.047	.049	.043	.044	.045	.052	.051	.047	.048	.000					
**BPNSF22**	.044	.046	.048	.044	.045	.043	.051	.052	.040	.040	.044	.000				
**BPNSF4**	.049	.052	.049	.044	.056	.049	.035	.050	.053	.051	.044	.050	.000			
**BPNSF12**	.050	.049	.049	.043	.048	.050	.055	.051	.048	.053	.047	.047	.046	.000		
**BPNSF16**	.053	.047	.048	.048	.053	.049	.056	.049	.050	.050	.048	.045	.044	.043	.000	
**BPNSF24**	.048	.047	.051	.050	.052	.050	.056	.054	.047	.051	.049	.051	.048	.039	.052	[illegible]
**BPNSF5**	.044	.045	.053	.047	.045	.043	.048	.049	.042	.052	.049	.048	.048	.048	.047	[illegible]
**BPNSF10**	.050	.041	.052	.048	.056	.049	.054	.051	.047	.050	.051	.050	.058	.053	.052	[illegible]
**BPNSF15**	.047	.047	.051	.047	.057	.052	.050	.055	.052	.055	.046	.053	.052	.054	.055	[illegible]
**BPNSF18**	.051	.051	.053	.050	.052	.057	.057	.054	.048	.055	.047	.054	.050	.061	.059	[illegible]
**BPNSF1**	.051	.044	.047	.042	.045	.047	.050	.047	.045	.047	.044	.045	.045	.046	.045	[illegible]
**BPNSF7**	.050	.045	.047	.041	.053	.047	.052	.048	.045	.046	.043	.046	.050	.052	.051	[illegible]
**BPNSF13**	.050	.050	.049	.048	.048	.051	.037	.044	.048	.051	.047	.048	.046	.052	.050	[illegible]
**BPNSF19**	.045	.046	.049	.043	.053	.052	.053	.045	.048	.054	.050	.051	.054	.048	.054	[illegible]

Sample Means - Standard Errors (g1 - Measurement intercepts)

	BPNSF6	BPNSF11	BPNSF17	BPNSF23	BPNSF3	BPNSF9	BPNSF14	BPNSF21	BPNSF2	BPNSF8	BPNSF20	BPNSF22	BPNSF4	BPNSF12	BPNSF16	BPNSF[illegible]
**BPNSF6**	.082	.073	.079	.072	.066	.069	.069	.074	.076	.083	.076	.069	.059	.067	.063	[illegible]

Bootstrap Confidence (g1 - Measurement intercepts)

Percentile method (g1 - Measurement intercepts)

90% confidence intervals (percentile method)

Scalar Estimates (g1 - Measurement intercepts)

Regression Weights: (g1 - Measurement intercepts)

Parameter			Estimate	Lower	Upper	P
BPNSF19	<---	F1	1.000	1.000	1.000	...
BPNSF13	<---	F1	1.144	1.043	1.237	.010
BPNSF7	<---	F1	.863	.764	.963	.010
BPNSF1	<---	F1	.759	.644	.885	.010
BPNSF18	<---	F2	1.000	1.000	1.000	...
BPNSF15	<---	F2	2.454	1.993	3.280	.010
BPNSF10	<---	F2	3.105	2.497	4.232	.010
BPNSF5	<---	F2	2.516	2.001	3.370	.010
BPNSF24	<---	F3	1.000	1.000	1.000	...
BPNSF16	<---	F3	1.124	1.038	1.214	.010
BPNSF12	<---	F3	1.221	1.136	1.296	.010
BPNSF4	<---	F3	.846	.752	.934	.010
BPNSF22	<---	F4	1.000	1.000	1.000	...
BPNSF20	<---	F4	1.192	1.111	1.283	.010
BPNSF8	<---	F4	1.197	1.114	1.272	.010
BPNSF2	<---	F4	1.120	1.035	1.222	.010
BPNSF21	<---	F5	1.000	1.000	1.000	...
BPNSF14	<---	F5	1.094	1.022	1.177	.010
BPNSF9	<---	F5	1.038	.963	1.130	.010
BPNSF3	<---	F5	.882	.793	.989	.010
BPNSF23	<---	F6	1.000	1.000	1.000	...
BPNSF17	<---	F6	.976	.925	1.043	.010

Parameter	Estimate	Lower	Upper	P
BPNSF11 <--- F6	.959	.899	1.022	.010
BPNSF6 <--- F6	.861	.801	.930	.010

Standardized Regression Weights: (g1 - Measurement intercepts)

Parameter	Estimate	Lower	Upper	P
BPNSF19 <--- F1	.650	.587	.710	.010
BPNSF13 <--- F1	.765	.705	.813	.010
BPNSF7 <--- F1	.522	.460	.583	.010
BPNSF1 <--- F1	.456	.404	.510	.010
BPNSF18 <--- F2	.244	.186	.298	.010
BPNSF15 <--- F2	.582	.517	.646	.010
BPNSF10 <--- F2	.693	.630	.752	.010
BPNSF5 <--- F2	.596	.546	.647	.010
BPNSF24 <--- F3	.691	.644	.744	.010
BPNSF16 <--- F3	.744	.688	.799	.010
BPNSF12 <--- F3	.797	.749	.833	.010
BPNSF4 <--- F3	.582	.535	.631	.010
BPNSF22 <--- F4	.681	.633	.728	.010
BPNSF20 <--- F4	.768	.725	.811	.010
BPNSF8 <--- F4	.731	.676	.780	.010
BPNSF2 <--- F4	.703	.644	.750	.010
BPNSF21 <--- F5	.692	.641	.741	.010
BPNSF14 <--- F5	.770	.707	.835	.010
BPNSF9 <--- F5	.714	.657	.773	.010
BPNSF3 <--- F5	.632	.569	.704	.010
BPNSF23 <--- F6	.799	.750	.842	.010
BPNSF17 <--- F6	.727	.682	.778	.010
BPNSF11 <--- F6	.744	.693	.788	.010
BPNSF6 <--- F6	.681	.621	.735	.010

Intercepts: (g1 - Measurement intercepts)

Parameter	Estimate	Lower	Upper	P
**BPNSF19**	5.140	5.067	5.217	.010
**BPNSF13**	5.043	4.979	5.123	.010
**BPNSF7**	4.817	4.755	4.891	.010
**BPNSF1**	4.705	4.648	4.786	.010
**BPNSF18**	4.316	4.250	4.387	.010
**BPNSF15**	3.699	3.616	3.773	.010
**BPNSF10**	3.158	3.066	3.227	.010
**BPNSF5**	3.721	3.641	3.789	.010
**BPNSF24**	5.225	5.160	5.302	.010
**BPNSF16**	5.118	5.059	5.200	.010
**BPNSF12**	5.220	5.163	5.293	.010
**BPNSF4**	5.116	5.057	5.187	.010
**BPNSF22**	3.135	3.051	3.203	.010
**BPNSF20**	2.524	2.403	2.600	.010
**BPNSF8**	2.823	2.727	2.907	.010
**BPNSF2**	2.400	2.308	2.469	.010
**BPNSF21**	5.178	5.107	5.260	.010
**BPNSF14**	5.441	5.379	5.529	.010
**BPNSF9**	5.657	5.589	5.737	.010
**BPNSF3**	5.591	5.534	5.683	.010
**BPNSF23**	2.250	2.163	2.317	.010
**BPNSF17**	2.586	2.485	2.662	.010
**BPNSF11**	2.754	2.667	2.833	.010
**BPNSF6**	2.574	2.483	2.656	.010

Covariances: (g1 - Measurement intercepts)

Parameter	Estimate	Lower	Upper	P
F1 <--> F2	-.076	-.126	-.037	.010
F2 <--> F3	-.102	-.139	-.067	.010
F1 <--> F3	.585	.481	.699	.010
F2 <--> F4	.209	.150	.272	.010
F3 <--> F4	-.462	-.542	-.381	.010
F1 <--> F4	-.415	-.523	-.310	.010
F2 <--> F5	-.058	-.098	-.019	.010
F4 <--> F5	-.629	-.729	-.531	.010

Parameter	Estimate	Lower	Upper	P
F3 <--> F5	.613	.504	.724	.010
F1 <--> F5	.880	.716	1.055	.010
F6 <--> F5	-.879	-1.020	-.747	.010
F6 <--> F3	-.503	-.613	-.408	.010
F6 <--> F4	1.155	.971	1.341	.010
F6 <--> F2	.282	.210	.367	.010
F6 <--> F1	-.586	-.713	-.465	.010

Correlations: (g1 - Measurement intercepts)

Parameter	Estimate	Lower	Upper	P
F1 <--> F2	-.224	-.352	-.099	.010
F2 <--> F3	-.330	-.450	-.223	.010
F1 <--> F3	.743	.655	.831	.010
F2 <--> F4	.574	.456	.669	.010
F3 <--> F4	-.545	-.623	-.467	.010
F1 <--> F4	-.447	-.539	-.346	.010
F2 <--> F5	-.161	-.277	-.049	.010
F4 <--> F5	-.636	-.717	-.555	.010
F3 <--> F5	.731	.664	.798	.010
F1 <--> F5	.958	.894	1.015	.010
F6 <--> F5	-.713	-.781	-.642	.010
F6 <--> F3	-.476	-.561	-.398	.010
F6 <--> F4	.927	.868	.976	.010
F6 <--> F2	.621	.531	.702	.010
F6 <--> F1	-.506	-.591	-.418	.010

Variances: (g1 - Measurement intercepts)

Parameter	Estimate	Lower	Upper	P
**F1**	.863	.672	1.056	.010
**F2**	.133	.079	.195	.010
**F3**	.719	.620	.840	.010
**F4**	.999	.841	1.166	.010
**F5**	.979	.815	1.165	.010
**F6**	1.553	1.318	1.785	.010
**e1**	1.182	.944	1.383	.010
**e2**	.798	.649	.996	.010
**e3**	1.715	1.459	2.007	.010
**e4**	1.896	1.655	2.180	.010
**e5**	2.086	1.839	2.321	.010
**e6**	1.562	1.314	1.822	.010
**e7**	1.384	1.157	1.612	.010
**e8**	1.521	1.288	1.736	.010
**e9**	.788	.625	.945	.010
**e10**	.734	.571	.940	.010
**e11**	.614	.467	.760	.010
**e12**	1.003	.862	1.159	.010
**e13**	1.156	.986	1.330	.010
**e14**	.988	.780	1.200	.010
**e15**	1.244	1.026	1.473	.010
**e16**	1.282	1.046	1.553	.010
**e17**	1.066	.880	1.259	.010
**e18**	.805	.547	1.055	.010
**e19**	1.017	.763	1.269	.010
**e20**	1.142	.868	1.431	.010
**e21**	.878	.671	1.092	.010
**e22**	1.321	1.012	1.606	.010
**e23**	1.150	.978	1.347	.010
**e24**	1.332	1.098	1.616	.010

Matrices (g1 - Measurement intercepts)

Sample Covariances (g1 - Measurement intercepts)

Sample Covariances - Lower Bounds (PC) (g1 - Measurement intercepts)

	BPNSF6	BPNSF11	BPNSF17	BPNSF23	BPNSF3	BPNSF9	BPNSF14	BPNSF21	BPNSF2	BPNSF8	BPNSF20	BPNSF22	BPNSF4	BPNSF12	BPNSF16	BPNS[illegible]
**BPNSF6**	2.334															
**BPNSF11**	1.185	2.407														

	BPNSF6	BPNSF11	BPNSF17	BPNSF23	BPNSF3	BPNSF9	BPNSF14	BPNSF21	BPNSF2	BPNSF8	BPNSF20	BPNSF22	BPNSF4	BPNSF12	BPNSF16	BPNS[illegible]
**BPNSF17**	1.144	1.179	2.238													
**BPNSF23**	1.083	1.165	1.100	2.025												
**BPNSF3**	-1.068	-.940	-.930	-1.146	1.572											
**BPNSF9**	-1.074	-.991	-1.039	-1.162	.755	1.632										
**BPNSF14**	-1.016	-.936	-1.068	-1.118	.759	.897	1.730									
**BPNSF21**	-.845	-.876	-.773	-1.051	.557	.717	.863	1.770								
**BPNSF2**	1.006	1.137	.869	1.170	-1.116	-1.062	-1.113	-1.016	2.389							
**BPNSF8**	1.181	1.236	1.035	.912	-.772	-.997	-.808	-.575	1.133	2.418						
**BPNSF20**	.879	.939	1.022	1.193	-.958	-.986	-1.046	-.893	1.116	1.029	2.013					
**BPNSF22**	.684	.840	.617	.844	-.744	-.691	-.576	-.599	.940	1.077	.918	1.856				
**BPNSF4**	-.668	-.527	-.508	-.514	.481	.299	.568	.270	-.739	-.537	-.597	-.502	1.312			
**BPNSF12**	-.817	-.566	-.748	-.671	.431	.520	.695	.471	-.793	-.705	-.792	-.745	.523	1.456		
**BPNSF16**	-.868	-.726	-.816	-.842	.397	.553	.603	.520	-.786	-.767	-.795	-.640	.505	.831	1.413	
**BPNSF24**	-.650	-.607	-.738	-.718	.437	.548	.435	.368	-.882	-.701	-.760	-.751	.443	.761	.675	[illegible]
**BPNSF5**	.572	.710	.437	.446	-.334	-.443	-.372	-.458	.608	.513	.413	.420	-.163	-.571	-.639	[illegible]
**BPNSF10**	.638	1.131	.564	.547	-.402	-.544	-.398	-.481	.656	.811	.435	.337	-.434	-.647	-.557	[illegible]
**BPNSF15**	.390	.553	.355	.168	-.417	-.334	-.014	-.402	.144	.421	.272	.249	-.286	-.537	-.294	[illegible]
**BPNSF18**	-.343	-.115	-.066	-.227	.012	.086	-.142	.193	-.121	-.046	-.242	-.055	-.112	-.097	.019	[illegible]
**BPNSF1**	-.478	-.428	-.264	-.406	.420	.224	.177	.394	-.321	-.300	-.361	-.312	.446	.373	.294	[illegible]
**BPNSF7**	-.444	-.592	-.288	-.615	.373	.612	.459	.689	-.734	-.254	-.416	-.343	.141	.184	.161	[illegible]
**BPNSF13**	-.833	-.762	-.771	-.855	.577	.664	1.070	.814	-.840	-.574	-.814	-.531	.424	.715	.567	[illegible]
**BPNSF19**	-.745	-.870	-.871	-1.037	.561	.558	.818	.996	-1.004	-.625	-.934	-.586	.285	.571	.562	[illegible]

Sample Covariances - Upper Bounds (PC) (g1 - Measurement intercepts)

	BPNSF6	BPNSF11	BPNSF17	BPNSF23	BPNSF3	BPNSF9	BPNSF14	BPNSF21	BPNSF2	BPNSF8	BPNSF20	BPNSF22	BPNSF4	BPNSF12	BPNSF16	BPNS[illegible]
**BPNSF6**	2.942															
**BPNSF11**	1.685	2.909														
**BPNSF17**	1.663	1.690	2.870													
**BPNSF23**	1.585	1.683	1.594	2.617												
**BPNSF3**	-.631	-.574	-.540	-.793	2.154											
**BPNSF9**	-.701	-.656	-.678	-.829	1.129	2.239										
**BPNSF14**	-.594	-.546	-.654	-.733	1.096	1.297	2.261									
**BPNSF21**	-.485	-.539	-.365	-.687	.913	1.099	1.332	2.292								
**BPNSF2**	1.496	1.646	1.325	1.664	-.753	-.687	-.719	-.634	2.911							
**BPNSF8**	1.697	1.728	1.570	1.428	-.382	-.587	-.450	-.213	1.636	2.942						
**BPNSF20**	1.427	1.436	1.562	1.702	-.617	-.607	-.622	-.514	1.533	1.533	2.583					
**BPNSF22**	1.101	1.260	1.016	1.266	-.428	-.373	-.234	-.223	1.323	1.503	1.293	2.260				
**BPNSF4**	-.321	-.195	-.202	-.216	.799	.599	.822	.583	-.367	-.185	-.317	-.213	1.639			
**BPNSF12**	-.398	-.209	-.397	-.373	.753	.892	1.128	.880	-.443	-.344	-.450	-.433	.802	1.923		
**BPNSF16**	-.449	-.379	-.447	-.485	.718	.905	1.006	.876	-.422	-.394	-.453	-.355	.775	1.160	1.817	
**BPNSF24**	-.308	-.282	-.365	-.389	.794	.926	.790	.716	-.555	-.362	-.465	-.435	.710	1.074	.985	[illegible]
**BPNSF5**	1.003	1.133	.875	.841	-.011	-.135	-.062	-.110	.969	.981	.809	.770	.114	-.263	-.290	[illegible]
**BPNSF10**	1.131	1.597	1.074	.988	-.016	-.161	.034	-.074	1.145	1.312	.869	.767	-.048	-.255	-.188	[illegible]
**BPNSF15**	.796	.993	.777	.541	-.012	.036	.353	.014	.593	.912	.627	.638	.038	-.172	.100	[illegible]
**BPNSF18**	.042	.275	.324	.109	.363	.426	.255	.578	.231	.386	.106	.320	.184	.297	.366	[illegible]
**BPNSF1**	-.083	-.101	.106	-.076	.792	.538	.544	.771	.025	.089	-.056	-.003	.746	.672	.574	[illegible]
**BPNSF7**	-.055	-.230	.084	-.312	.750	.989	.834	1.079	-.392	.117	-.060	-.006	.438	.562	.493	[illegible]
**BPNSF13**	-.433	-.360	-.410	-.488	.907	1.033	1.491	1.272	-.449	-.170	-.437	-.201	.727	1.144	.944	[illegible]
**BPNSF19**	-.375	-.486	-.491	-.654	.934	.941	1.239	1.468	-.604	-.185	-.519	-.247	.613	.916	.943	[illegible]

Sample Covariances - Two Tailed Significance (PC) (g1 - Measurement intercepts)

	BPNSF6	BPNSF11	BPNSF17	BPNSF23	BPNSF3	BPNSF9	BPNSF14	BPNSF21	BPNSF2	BPNSF8	BPNSF20	BPNSF22	BPNSF4	BPNSF12	BPNSF16	BPNS[illegible]
**BPNSF6**	.010															
**BPNSF11**	.010	.010														
**BPNSF17**	.010	.010	.010													
**BPNSF23**	.010	.010	.010	.010												
**BPNSF3**	.010	.010	.010	.010	.010											
**BPNSF9**	.010	.010	.010	.010	.010	.010										
**BPNSF14**	.010	.010	.010	.010	.010	.010	.010									
**BPNSF21**	.010	.010	.010	.010	.010	.010	.010	.010								
**BPNSF2**	.010	.010	.010	.010	.010	.010	.010	.010	.010							
**BPNSF8**	.010	.010	.010	.010	.010	.010	.010	.010	.010	.010						
**BPNSF20**	.010	.010	.010	.010	.010	.010	.010	.010	.010	.010	.010					
**BPNSF22**	.010	.010	.010	.010	.010	.010	.010	.010	.010	.010	.010	.010				
**BPNSF4**	.010	.010	.010	.010	.010	.010	.010	.010	.010	.010	.010	.010	.010			
**BPNSF12**	.010	.010	.010	.010	.010	.010	.010	.010	.010	.010	.010	.010	.010	.010		
**BPNSF16**	.010	.010	.010	.010	.010	.010	.010	.010	.010	.010	.010	.010	.010	.010	.010	
**BPNSF24**	.010	.010	.010	.010	.010	.010	.010	.010	.010	.010	.010	.010	.010	.010	.010	[illegible]
**BPNSF5**	.010	.010	.010	.010	.077	.012	.044	.016	.010	.010	.010	.010	.721	.010	.010	[illegible]

	BPNSF6	BPNSF11	BPNSF17	BPNSF23	BPNSF3	BPNSF9	BPNSF14	BPNSF21	BPNSF2	BPNSF8	BPNSF20	BPNSF22	BPNSF4	BPNSF12	BPNSF16	BPNS
**BPNSF10**	.010	.010	.010	.010	.077	.010	.193	.010	.010	.010	.010	.010	.039	.010	.010	.0
**BPNSF15**	.010	.010	.010	.010	.094	.201	.157	.142	.010	.010	.010	.010	.196	.010	.371	.0
**BPNSF18**	.207	.487	.403	.569	.078	.041	.589	.010	.702	.197	.400	.271	.783	.336	.078	.5
**BPNSF1**	.027	.018	.456	.016	.010	.010	.010	.010	.146	.339	.033	.095	.010	.010	.010	.0
**BPNSF7**	.025	.010	.268	.010	.010	.010	.010	.010	.010	.519	.010	.073	.010	.010	.016	.0
**BPNSF13**	.010	.010	.010	.010	.010	.010	.010	.010	.010	.010	.010	.010	.010	.010	.010	.0
**BPNSF19**	.010	.010	.010	.010	.010	.010	.010	.010	.010	.010	.010	.010	.010	.010	.010	.0

Sample Correlations (g1 - Measurement intercepts)

Sample Correlations - Lower Bounds (PC) (g1 - Measurement intercepts)

	BPNSF6	BPNSF11	BPNSF17	BPNSF23	BPNSF3	BPNSF9	BPNSF14	BPNSF21	BPNSF2	BPNSF8	BPNSF20	BPNSF22	BPNSF4	BPNSF12	BPNSF16	BPNS
**BPNSF6**	1.000															
**BPNSF11**	.469	1.000														
**BPNSF17**	.460	.467	1.000													
**BPNSF23**	.472	.506	.472	1.000												
**BPNSF3**	-.471	-.424	-.417	-.541	1.000											
**BPNSF9**	-.477	-.436	-.460	-.550	.403	1.000										
**BPNSF14**	-.444	-.394	-.456	-.501	.387	.474	1.000									
**BPNSF21**	-.360	-.375	-.341	-.465	.297	.376	.447	1.000								
**BPNSF2**	.395	.437	.335	.502	-.500	-.465	-.470	-.431	1.000							
**BPNSF8**	.458	.476	.412	.376	-.339	-.424	-.347	-.253	.446	1.000						
**BPNSF20**	.389	.398	.448	.542	-.452	-.456	-.475	-.418	.455	.430	1.000					
**BPNSF22**	.309	.363	.273	.409	-.378	-.340	-.287	-.291	.408	.474	.436	1.000				
**BPNSF4**	-.331	-.268	-.265	-.276	.290	.181	.347	.164	-.368	-.265	-.316	-.289	1.000			
**BPNSF12**	-.370	-.261	-.353	-.325	.247	.312	.391	.264	-.371	-.334	-.389	-.387	.343	1.000		
**BPNSF16**	-.407	-.342	-.383	-.426	.232	.326	.349	.306	-.371	-.363	-.411	-.343	.345	.526	1.000	
**BPNSF24**	-.323	-.303	-.351	-.377	.270	.348	.248	.217	-.431	-.349	-.419	-.417	.296	.493	.446	1.0
**BPNSF5**	.237	.296	.177	.195	-.158	-.208	-.174	-.213	.246	.210	.178	.194	-.088	-.296	-.310	-.2
**BPNSF10**	.250	.440	.218	.220	-.179	-.245	-.174	-.205	.256	.318	.179	.152	-.226	-.302	-.267	-.2
**BPNSF15**	.156	.218	.142	.072	-.193	-.160	-.006	-.175	.059	.167	.120	.115	-.153	-.263	-.144	-.1
**BPNSF18**	-.143	-.048	-.029	-.106	.007	.042	-.067	.095	-.052	-.021	-.109	-.028	-.065	-.051	.010	-.0
**BPNSF1**	-.193	-.184	-.116	-.177	.218	.111	.085	.195	-.132	-.122	-.162	-.144	.256	.190	.155	.1
**BPNSF7**	-.180	-.240	-.121	-.269	.176	.297	.211	.319	-.306	-.104	-.180	-.154	.079	.099	.084	.1
**BPNSF13**	-.373	-.333	-.344	-.390	.311	.356	.589	.438	-.366	-.252	-.384	-.271	.266	.432	.336	.2
**BPNSF19**	-.309	-.374	-.379	-.450	.288	.302	.415	.536	-.426	-.269	-.416	-.287	.162	.317	.320	.2

Sample Correlations - Upper Bounds (PC) (g1 - Measurement intercepts)

	BPNSF6	BPNSF11	BPNSF17	BPNSF23	BPNSF3	BPNSF9	BPNSF14	BPNSF21	BPNSF2	BPNSF8	BPNSF20	BPNSF22	BPNSF4	BPNSF12	BPNSF16	BPNS
**BPNSF6**	1.000															
**BPNSF11**	.617	1.000														
**BPNSF17**	.608	.617	1.000													
**BPNSF23**	.615	.635	.626	1.000												
**BPNSF3**	-.295	-.259	-.254	-.386	1.000											
**BPNSF9**	-.308	-.297	-.309	-.394	.578	1.000										
**BPNSF14**	-.264	-.233	-.294	-.346	.573	.635	1.000									
**BPNSF21**	-.213	-.227	-.166	-.334	.470	.536	.624	1.000								
**BPNSF2**	.557	.589	.496	.651	-.347	-.304	-.318	-.283	1.000							
**BPNSF8**	.607	.618	.579	.550	-.172	-.264	-.196	-.088	.596	1.000						
**BPNSF20**	.559	.555	.612	.691	-.297	-.305	-.294	-.245	.614	.597	1.000					
**BPNSF22**	.453	.516	.434	.554	-.217	-.192	-.110	-.111	.548	.619	.575	1.000				
**BPNSF4**	-.167	-.100	-.104	-.118	.471	.352	.451	.327	-.186	-.097	-.177	-.124	1.000			
**BPNSF12**	-.198	-.102	-.188	-.189	.418	.477	.571	.434	-.211	-.159	-.228	-.231	.508	1.000		
**BPNSF16**	-.224	-.188	-.223	-.259	.409	.499	.536	.466	-.201	-.192	-.236	-.193	.483	.668	1.000	
**BPNSF24**	-.155	-.141	-.189	-.210	.441	.518	.437	.399	-.272	-.178	-.250	-.247	.463	.629	.607	1.0
**BPNSF5**	.395	.442	.364	.354	-.005	-.057	-.029	-.051	.393	.382	.344	.359	.062	-.133	-.149	-.1
**BPNSF10**	.413	.582	.403	.384	-.007	-.071	.016	-.032	.413	.480	.339	.314	-.024	-.117	-.089	-.0
**BPNSF15**	.305	.381	.310	.223	-.006	.018	.158	.007	.230	.354	.264	.285	.020	-.088	.048	-.0
**BPNSF18**	.017	.119	.135	.049	.178	.218	.128	.279	.097	.159	.048	.144	.102	.152	.201	.1
**BPNSF1**	-.033	-.041	.042	-.034	.377	.272	.254	.359	.010	.038	-.026	-.001	.413	.354	.303	.3
**BPNSF7**	-.022	-.093	.033	-.132	.360	.454	.386	.478	-.153	.048	-.026	-.003	.243	.274	.245	.2
**BPNSF13**	-.195	-.156	-.180	-.234	.469	.528	.712	.592	-.202	-.076	-.217	-.109	.416	.600	.511	.4
**BPNSF19**	-.162	-.213	-.212	-.319	.466	.472	.589	.679	-.259	-.075	-.239	-.119	.346	.474	.496	.4

Sample Correlations - Two Tailed Significance (PC) (g1 - Measurement intercepts)

	BPNSF6	BPNSF11	BPNSF17	BPNSF23	BPNSF3	BPNSF9	BPNSF14	BPNSF21	BPNSF2	BPNSF8	BPNSF20	BPNSF22	BPNSF4	BPNSF12	BPNSF16	BPNS
**BPNSF6**	...															

	BPNSF6	BPNSF11	BPNSF17	BPNSF23	BPNSF3	BPNSF9	BPNSF14	BPNSF21	BPNSF2	BPNSF8	BPNSF20	BPNSF22	BPNSF4	BPNSF12	BPNSF16	BPNS
**BPNSF11**	.010	...														
**BPNSF17**	.010	.010	...													
**BPNSF23**	.010	.010	.010	...												
**BPNSF3**	.010	.010	.010	.010	...											
**BPNSF9**	.010	.010	.010	.010	.010	...										
**BPNSF14**	.010	.010	.010	.010	.010	.010	...									
**BPNSF21**	.010	.010	.010	.010	.010	.010	.010	...								
**BPNSF2**	.010	.010	.010	.010	.010	.010	.010	.010	...							
**BPNSF8**	.010	.010	.010	.010	.010	.010	.010	.010	.010	...						
**BPNSF20**	.010	.010	.010	.010	.010	.010	.010	.010	.010	.010	...					
**BPNSF22**	.010	.010	.010	.010	.010	.010	.010	.010	.010	.010	.010	...				
**BPNSF4**	.010	.010	.010	.010	.010	.010	.010	.010	.010	.010	.010	.010	...			
**BPNSF12**	.010	.010	.010	.010	.010	.010	.010	.010	.010	.010	.010	.010	.010	...		
**BPNSF16**	.010	.010	.010	.010	.010	.010	.010	.010	.010	.010	.010	.010	.010	.010	...	
**BPNSF24**	.010	.010	.010	.010	.010	.010	.010	.010	.010	.010	.010	.010	.010	.010	.010	
**BPNSF5**	.010	.010	.010	.010	.077	.012	.044	.016	.010	.010	.010	.010	.721	.010	.010	.0
**BPNSF10**	.010	.010	.010	.010	.077	.010	.193	.010	.010	.010	.010	.010	.039	.010	.010	.0
**BPNSF15**	.010	.010	.010	.010	.094	.201	.157	.142	.010	.010	.010	.010	.196	.010	.371	.0
**BPNSF18**	.207	.487	.403	.569	.078	.041	.589	.010	.702	.197	.400	.271	.783	.336	.078	.5
**BPNSF1**	.027	.017	.456	.016	.010	.010	.010	.010	.145	.339	.033	.095	.010	.010	.010	.0
**BPNSF7**	.025	.010	.268	.010	.010	.010	.010	.010	.010	.519	.010	.073	.010	.010	.015	.0
**BPNSF13**	.010	.010	.010	.010	.010	.010	.010	.010	.010	.010	.010	.010	.010	.010	.010	.0
**BPNSF19**	.010	.010	.010	.010	.010	.010	.010	.010	.010	.010	.010	.010	.010	.010	.010	.0

Sample Means (g1 - Measurement intercepts)

Sample Means - Lower Bounds (PC) (g1 - Measurement intercepts)

	BPNSF6	BPNSF11	BPNSF17	BPNSF23	BPNSF3	BPNSF9	BPNSF14	BPNSF21	BPNSF2	BPNSF8	BPNSF20	BPNSF22	BPNSF4	BPNSF12	BPNSF16	BPNSF
**BPNSF6**	2.319	2.572	2.304	1.943	5.618	5.729	5.485	5.242	2.103	2.626	2.215	2.901	4.970	5.123	5.014	5.13

Sample Means - Upper Bounds (PC) (g1 - Measurement intercepts)

	BPNSF6	BPNSF11	BPNSF17	BPNSF23	BPNSF3	BPNSF9	BPNSF14	BPNSF21	BPNSF2	BPNSF8	BPNSF20	BPNSF22	BPNSF4	BPNSF12	BPNSF16	BPNSF
**BPNSF6**	2.585	2.818	2.585	2.178	5.853	5.952	5.713	5.486	2.367	2.909	2.470	3.142	5.166	5.354	5.226	5.36

Sample Means - Two Tailed Significance (PC) (g1 - Measurement intercepts)

	BPNSF6	BPNSF11	BPNSF17	BPNSF23	BPNSF3	BPNSF9	BPNSF14	BPNSF21	BPNSF2	BPNSF8	BPNSF20	BPNSF22	BPNSF4	BPNSF12	BPNSF16	BPNSF
**BPNSF6**	.010	.010	.010	.010	.010	.010	.010	.010	.010	.010	.010	.010	.010	.010	.010	.01

Bias-corrected percentile method (g1 - Measurement intercepts)

90% confidence intervals (bias-corrected percentile method)

Scalar Estimates (g1 - Measurement intercepts)

Regression Weights: (g1 - Measurement intercepts)

Parameter			Estimate	Lower	Upper	P
BPNSF19	<---	F1	1.000	1.000	1.000	...
BPNSF13	<---	F1	1.144	1.053	1.252	.004
BPNSF7	<---	F1	.863	.764	.963	.010
BPNSF1	<---	F1	.759	.643	.876	.012
BPNSF18	<---	F2	1.000	1.000	1.000	...
BPNSF15	<---	F2	2.454	2.008	3.361	.006
BPNSF10	<---	F2	3.105	2.512	4.256	.008
BPNSF5	<---	F2	2.516	2.030	3.412	.008
BPNSF24	<---	F3	1.000	1.000	1.000	...
BPNSF16	<---	F3	1.124	1.040	1.219	.009
BPNSF12	<---	F3	1.221	1.151	1.306	.004
BPNSF4	<---	F3	.846	.746	.934	.012
BPNSF22	<---	F4	1.000	1.000	1.000	...
BPNSF20	<---	F4	1.192	1.118	1.295	.005
BPNSF8	<---	F4	1.197	1.120	1.292	.005
BPNSF2	<---	F4	1.120	1.043	1.226	.005
BPNSF21	<---	F5	1.000	1.000	1.000	...
BPNSF14	<---	F5	1.094	1.023	1.179	.009
BPNSF9	<---	F5	1.038	.967	1.135	.007

Parameter			Estimate	Lower	Upper	P
BPNSF3	<---	F5	.882	.787	.982	.015
BPNSF23	<---	F6	1.000	1.000	1.000	...
BPNSF17	<---	F6	.976	.914	1.026	.021
BPNSF11	<---	F6	.959	.910	1.027	.005
BPNSF6	<---	F6	.861	.801	.930	.010

Standardized Regression Weights: (g1 - Measurement intercepts)

Parameter			Estimate	Lower	Upper	P
BPNSF19	<---	F1	.650	.559	.693	.030
BPNSF13	<---	F1	.765	.705	.812	.011
BPNSF7	<---	F1	.522	.457	.582	.012
BPNSF1	<---	F1	.456	.406	.511	.007
BPNSF18	<---	F2	.244	.188	.301	.009
BPNSF15	<---	F2	.582	.517	.646	.011
BPNSF10	<---	F2	.693	.642	.760	.005
BPNSF5	<---	F2	.596	.548	.648	.009
BPNSF24	<---	F3	.691	.634	.731	.018
BPNSF16	<---	F3	.744	.679	.794	.018
BPNSF12	<---	F3	.797	.745	.832	.014
BPNSF4	<---	F3	.582	.542	.643	.004
BPNSF22	<---	F4	.681	.636	.732	.007
BPNSF20	<---	F4	.768	.727	.813	.007
BPNSF8	<---	F4	.731	.681	.781	.006
BPNSF2	<---	F4	.703	.657	.761	.004
BPNSF21	<---	F5	.692	.642	.741	.009
BPNSF14	<---	F5	.770	.690	.828	.020
BPNSF9	<---	F5	.714	.655	.771	.013
BPNSF3	<---	F5	.632	.566	.700	.014
BPNSF23	<---	F6	.799	.749	.842	.011
BPNSF17	<---	F6	.727	.681	.778	.011
BPNSF11	<---	F6	.744	.701	.794	.004
BPNSF6	<---	F6	.681	.624	.740	.006

Intercepts: (g1 - Measurement intercepts)

Parameter	Estimate	Lower	Upper	P
**BPNSF19**	5.140	5.046	5.203	.028
**BPNSF13**	5.043	4.967	5.102	.034
**BPNSF7**	4.817	4.726	4.873	.044
**BPNSF1**	4.705	4.630	4.766	.030
**BPNSF18**	4.316	4.252	4.390	.009
**BPNSF15**	3.699	3.606	3.770	.012
**BPNSF10**	3.158	3.068	3.229	.009
**BPNSF5**	3.721	3.650	3.798	.006
**BPNSF24**	5.225	5.135	5.281	.034
**BPNSF16**	5.118	5.049	5.195	.019
**BPNSF12**	5.220	5.151	5.275	.034
**BPNSF4**	5.116	5.041	5.175	.026
**BPNSF22**	3.135	3.062	3.213	.004
**BPNSF20**	2.524	2.445	2.626	.003
**BPNSF8**	2.823	2.738	2.909	.005
**BPNSF2**	2.400	2.330	2.498	.003
**BPNSF21**	5.178	5.054	5.218	.095
**BPNSF14**	5.441	5.353	5.498	.053
**BPNSF9**	5.657	5.564	5.726	.032
**BPNSF3**	5.591	5.530	5.666	.018
**BPNSF23**	2.250	2.189	2.341	.002
**BPNSF17**	2.586	2.504	2.680	.003
**BPNSF11**	2.754	2.681	2.846	.004
**BPNSF6**	2.574	2.487	2.664	.007

Covariances: (g1 - Measurement intercepts)

Parameter			Estimate	Lower	Upper	P
F1	<-->	F2	-.076	-.131	-.043	.004
F2	<-->	F3	-.102	-.156	-.073	.003
F1	<-->	F3	.585	.496	.718	.006
F2	<-->	F4	.209	.156	.283	.005
F3	<-->	F4	-.462	-.542	-.381	.010

Parameter	Estimate	Lower	Upper	P
F1 <--> F4	-.415	-.548	-.332	.002
F2 <--> F5	-.058	-.100	-.021	.008
F4 <--> F5	-.629	-.763	-.548	.002
F3 <--> F5	.613	.525	.729	.006
F1 <--> F5	.880	.716	1.055	.010
F6 <--> F5	-.879	-1.063	-.769	.003
F6 <--> F3	-.503	-.614	-.411	.009
F6 <--> F4	1.155	1.006	1.375	.004
F6 <--> F2	.282	.214	.381	.006
F6 <--> F1	-.586	-.719	-.467	.008

Correlations: (g1 - Measurement intercepts)

Parameter	Estimate	Lower	Upper	P
F1 <--> F2	-.224	-.363	-.126	.005
F2 <--> F3	-.330	-.483	-.236	.004
F1 <--> F3	.743	.671	.846	.004
F2 <--> F4	.574	.421	.652	.023
F3 <--> F4	-.545	-.625	-.476	.006
F1 <--> F4	-.447	-.556	-.362	.004
F2 <--> F5	-.161	-.278	-.051	.009
F4 <--> F5	-.636	-.728	-.570	.004
F3 <--> F5	.731	.650	.794	.015
F1 <--> F5	.958	.904	1.024	.005
F6 <--> F5	-.713	-.782	-.649	.009
F6 <--> F3	-.476	-.553	-.387	.019
F6 <--> F4	.927	.853	.972	.020
F6 <--> F2	.621	.509	.700	.015
F6 <--> F1	-.506	-.599	-.434	.005

Variances: (g1 - Measurement intercepts)

Parameter	Estimate	Lower	Upper	P
**F1**	.863	.643	1.021	.016
**F2**	.133	.078	.195	.011
**F3**	.719	.620	.835	.012
**F4**	.999	.849	1.222	.006
**F5**	.979	.815	1.165	.010
**F6**	1.553	1.322	1.786	.008
**e1**	1.182	.984	1.475	.003
**e2**	.798	.662	1.015	.007
**e3**	1.715	1.403	1.966	.016
**e4**	1.896	1.701	2.211	.004
**e5**	2.086	1.859	2.368	.005
**e6**	1.562	1.333	1.838	.005
**e7**	1.384	1.117	1.568	.020
**e8**	1.521	1.320	1.750	.007
**e9**	.788	.633	.946	.009
**e10**	.734	.605	.980	.003
**e11**	.614	.493	.771	.006
**e12**	1.003	.853	1.140	.015
**e13**	1.156	1.002	1.337	.008
**e14**	.988	.777	1.197	.012
**e15**	1.244	1.022	1.471	.011
**e16**	1.282	1.007	1.489	.023
**e17**	1.066	.893	1.268	.007
**e18**	.805	.597	1.182	.003
**e19**	1.017	.802	1.387	.003
**e20**	1.142	.874	1.441	.009
**e21**	.878	.689	1.123	.006
**e22**	1.321	1.032	1.630	.006
**e23**	1.150	.955	1.319	.018
**e24**	1.332	1.059	1.570	.018

Matrices (g1 - Measurement intercepts)

Sample Covariances (g1 - Measurement intercepts)

Sample Covariances - Lower Bounds (BC) (g1 - Measurement intercepts)

	BPNSF6	BPNSF11	BPNSF17	BPNSF23	BPNSF3	BPNSF9	BPNSF14	BPNSF21	BPNSF2	BPNSF8	BPNSF20	BPNSF22	BPNSF4	BPNSF12	BPNSF16	BPNS[illegible]
**BPNSF6**	2.397															
**BPNSF11**	1.225	2.460														
**BPNSF17**	1.121	1.186	2.247													
**BPNSF23**	1.087	1.221	1.115	2.069												
**BPNSF3**	-1.179	-.979	-.974	-1.230	1.656											
**BPNSF9**	-1.161	-1.197	-1.086	-1.237	.792	1.689										
**BPNSF14**	-1.043	-1.010	-1.078	-1.166	.770	.902	1.746									
**BPNSF21**	-.887	-.893	-.796	-1.079	.567	.734	.911	1.776								
**BPNSF2**	1.009	1.161	.906	1.156	-1.117	-1.091	-1.128	-.990	2.387							
**BPNSF8**	1.192	1.251	1.071	.921	-.874	-1.035	-.844	-.617	1.160	2.418						
**BPNSF20**	.974	.969	1.053	1.247	-1.006	-1.003	-1.093	-.914	1.145	1.065	2.026					
**BPNSF22**	.685	.863	.604	.848	-.821	-.709	-.582	-.620	.966	1.104	.936	1.861				
**BPNSF4**	-.702	-.549	-.552	-.519	.488	.321	.579	.282	-.757	-.633	-.628	-.512	1.318			
**BPNSF12**	-.823	-.569	-.747	-.637	.410	.515	.708	.525	-.776	-.753	-.766	-.746	.541	1.467		
**BPNSF16**	-.874	-.753	-.836	-.847	.398	.579	.634	.530	-.809	-.784	-.823	-.646	.506	.842	1.424	
**BPNSF24**	-.650	-.611	-.698	-.713	.459	.587	.435	.392	-.925	-.726	-.760	-.752	.448	.780	.675	1.3[illegible]
**BPNSF5**	.566	.756	.436	.458	-.324	-.451	-.370	-.458	.622	.537	.396	.454	-.195	-.571	-.667	-.5[illegible]
**BPNSF10**	.628	1.119	.577	.570	-.385	-.549	-.408	-.491	.701	.811	.438	.351	-.454	-.664	-.603	-.4[illegible]
**BPNSF15**	.369	.537	.355	.168	-.422	-.334	.012	-.402	.132	.421	.248	.218	-.313	-.587	-.308	-.3[illegible]
**BPNSF18**	-.384	-.161	-.103	-.260	-.001	.090	-.144	.179	-.113	-.134	-.243	-.074	-.098	-.091	.023	-.1[illegible]
**BPNSF1**	-.479	-.430	-.262	-.424	.460	.256	.202	.420	-.373	-.323	-.416	-.324	.472	.393	.320	.3[illegible]
**BPNSF7**	-.479	-.627	-.316	-.658	.388	.613	.479	.702	-.726	-.301	-.445	-.400	.150	.243	.203	.2[illegible]
**BPNSF13**	-.886	-.800	-.778	-.896	.580	.654	1.115	.845	-.832	-.612	-.832	-.537	.434	.715	.604	.4[illegible]
**BPNSF19**	-.754	-.870	-.939	-1.063	.615	.606	.849	1.014	-.997	-.674	-1.004	-.631	.307	.593	.613	.4[illegible]

Sample Covariances - Upper Bounds (BC) (g1 - Measurement intercepts)

	BPNSF6	BPNSF11	BPNSF17	BPNSF23	BPNSF3	BPNSF9	BPNSF14	BPNSF21	BPNSF2	BPNSF8	BPNSF20	BPNSF22	BPNSF4	BPNSF12	BPNSF16	BPNS[illegible]
**BPNSF6**	2.992															
**BPNSF11**	1.781	3.010														
**BPNSF17**	1.621	1.707	2.876													
**BPNSF23**	1.602	1.695	1.599	2.658												
**BPNSF3**	-.710	-.598	-.603	-.819	2.220											
**BPNSF9**	-.756	-.736	-.704	-.848	1.174	2.344										
**BPNSF14**	-.635	-.578	-.659	-.773	1.108	1.312	2.339									
**BPNSF21**	-.549	-.555	-.404	-.713	.936	1.125	1.382	2.304								
**BPNSF2**	1.506	1.702	1.414	1.658	-.761	-.732	-.731	-.612	2.909							
**BPNSF8**	1.711	1.764	1.593	1.437	-.438	-.661	-.478	-.251	1.664	2.943						
**BPNSF20**	1.507	1.467	1.568	1.752	-.669	-.656	-.643	-.542	1.607	1.572	2.595					
**BPNSF22**	1.101	1.292	.994	1.272	-.467	-.411	-.254	-.277	1.359	1.563	1.330	2.270				
**BPNSF4**	-.343	-.224	-.225	-.216	.812	.611	.830	.599	-.378	-.254	-.329	-.227	1.656			
**BPNSF12**	-.409	-.213	-.397	-.316	.734	.882	1.147	.914	-.436	-.360	-.444	-.437	.831	1.928		
**BPNSF16**	-.471	-.399	-.468	-.487	.720	.974	1.037	.882	-.437	-.415	-.463	-.359	.794	1.178	1.827	
**BPNSF24**	-.308	-.294	-.355	-.387	.811	.972	.790	.723	-.575	-.391	-.465	-.440	.712	1.087	.985	1.7[illegible]
**BPNSF5**	.987	1.163	.866	.845	.000	-.136	-.061	-.108	.997	.988	.792	.824	.104	-.263	-.315	-.2[illegible]
**BPNSF10**	1.103	1.591	1.079	1.008	.000	-.167	.029	-.077	1.180	1.312	.883	.773	-.060	-.265	-.217	-.0[illegible]
**BPNSF15**	.765	.992	.777	.541	-.016	.038	.419	.013	.581	.912	.613	.614	.023	-.200	.067	-.0[illegible]
**BPNSF18**	.011	.242	.313	.106	.336	.448	.246	.555	.244	.342	.099	.300	.185	.310	.367	.2[illegible]
**BPNSF1**	-.094	-.111	.106	-.115	.865	.573	.568	.824	-.004	.026	-.098	-.035	.753	.710	.595	.6[illegible]
**BPNSF7**	-.091	-.263	.034	-.341	.771	.990	.853	1.082	-.358	.073	-.106	-.032	.453	.610	.518	.5[illegible]
**BPNSF13**	-.477	-.427	-.410	-.512	.928	1.016	1.585	1.295	-.447	-.233	-.492	-.229	.740	1.144	.980	.8[illegible]
**BPNSF19**	-.386	-.486	-.521	-.680	1.009	.975	1.306	1.489	-.588	-.240	-.584	-.255	.625	.952	1.036	.7[illegible]

Sample Covariances - Two Tailed Significance (BC) (g1 - Measurement intercepts)

	BPNSF6	BPNSF11	BPNSF17	BPNSF23	BPNSF3	BPNSF9	BPNSF14	BPNSF21	BPNSF2	BPNSF8	BPNSF20	BPNSF22	BPNSF4	BPNSF12	BPNSF16	BPNS[illegible]
**BPNSF6**	.006															
**BPNSF11**	.003	.003														
**BPNSF17**	.014	.007	.008													
**BPNSF23**	.008	.005	.007	.005												
**BPNSF3**	.002	.005	.003	.004	.003											
**BPNSF9**	.002	.001	.005	.003	.003	.003										
**BPNSF14**	.004	.002	.008	.004	.007	.007	.005									
**BPNSF21**	.002	.006	.004	.005	.006	.005	.002	.006								
**BPNSF2**	.007	.004	.004	.012	.009	.005	.006	.018	.012							
**BPNSF8**	.007	.006	.005	.008	.002	.002	.004	.005	.006	.009						
**BPNSF20**	.003	.005	.006	.002	.003	.004	.005	.004	.003	.006	.006					
**BPNSF22**	.009	.006	.014	.009	.002	.003	.006	.003	.004	.005	.005	.009				
**BPNSF4**	.005	.005	.004	.009	.005	.006	.006	.007	.006	.002	.004	.005	.006			
**BPNSF12**	.007	.009	.011	.019	.016	.013	.006	.003	.013	.004	.012	.008	.005	.009		
**BPNSF16**	.007	.005	.004	.009	.009	.004	.004	.007	.006	.005	.006	.007	.007	.006	.007	

	BPNSF6	BPNSF11	BPNSF17	BPNSF23	BPNSF3	BPNSF9	BPNSF14	BPNSF21	BPNSF2	BPNSF8	BPNSF20	BPNSF22	BPNSF4	BPNSF12	BPNSF16	BPNS[illegible]
**BPNSF24**	.010	.007	.016	.012	.005	.005	.010	.006	.004	.004	.010	.008	.008	.007	.010	[illegible]
**BPNSF5**	.012	.004	.012	.008	.100	.010	.047	.019	.006	.007	.012	.004	.577	.010	.005	[illegible]
**BPNSF10**	.013	.012	.008	.007	.101	.008	.169	.007	.005	.010	.006	.007	.031	.009	.004	[illegible]
**BPNSF15**	.021	.014	.010	.011	.089	.210	.086	.136	.012	.010	.019	.025	.142	.005	.284	[illegible]
**BPNSF18**	.137	.732	.493	.535	.102	.036	.624	.019	.611	.362	.372	.330	.671	.299	.074	[illegible]
**BPNSF1**	.018	.013	.471	.008	.003	.004	.006	.005	.093	.174	.010	.046	.006	.004	.004	[illegible]
**BPNSF7**	.007	.004	.174	.002	.005	.008	.005	.008	.018	.250	.004	.021	.008	.003	.004	[illegible]
**BPNSF13**	.003	.003	.009	.005	.007	.014	.004	.005	.012	.002	.004	.005	.005	.010	.004	[illegible]
**BPNSF19**	.007	.010	.003	.006	.002	.004	.004	.006	.012	.004	.003	.006	.004	.006	.002	[illegible]

Sample Correlations (g1 - Measurement intercepts)

Sample Correlations - Lower Bounds (BC) (g1 - Measurement intercepts)

	BPNSF6	BPNSF11	BPNSF17	BPNSF23	BPNSF3	BPNSF9	BPNSF14	BPNSF21	BPNSF2	BPNSF8	BPNSF20	BPNSF22	BPNSF4	BPNSF12	BPNSF16	BPNS[illegible]
**BPNSF6**	1.000															
**BPNSF11**	.487	1.000														
**BPNSF17**	.444	.462	1.000													
**BPNSF23**	.479	.510	.472	1.000												
**BPNSF3**	-.498	-.425	-.430	-.546	1.000											
**BPNSF9**	-.496	-.455	-.465	-.550	.415	1.000										
**BPNSF14**	-.447	-.410	-.458	-.507	.379	.462	1.000									
**BPNSF21**	-.366	-.373	-.347	-.464	.311	.379	.447	1.000								
**BPNSF2**	.395	.448	.337	.488	-.500	-.465	-.465	-.425	1.000							
**BPNSF8**	.458	.468	.419	.375	-.348	-.434	-.361	-.261	.464	1.000						
**BPNSF20**	.412	.406	.458	.545	-.462	-.458	-.478	-.418	.458	.449	1.000					
**BPNSF22**	.309	.364	.257	.403	-.402	-.349	-.288	-.293	.430	.491	.441	1.000				
**BPNSF4**	-.334	-.273	-.279	-.264	.296	.183	.342	.168	-.370	-.283	-.331	-.291	1.000			
**BPNSF12**	-.371	-.261	-.341	-.314	.237	.309	.374	.281	-.370	-.346	-.383	-.387	.337	1.000		
**BPNSF16**	-.417	-.347	-.380	-.426	.220	.323	.341	.294	-.370	-.371	-.399	-.347	.345	.525	1.000	
**BPNSF24**	-.317	-.284	-.335	-.371	.261	.354	.244	.222	-.444	-.359	-.418	-.417	.298	.491	.430	[illegible]
**BPNSF5**	.235	.295	.174	.193	-.153	-.204	-.174	-.202	.243	.210	.163	.206	-.104	-.294	-.323	[illegible]
**BPNSF10**	.249	.440	.227	.232	-.178	-.239	-.175	-.204	.256	.319	.184	.152	-.234	-.303	-.285	[illegible]
**BPNSF15**	.152	.212	.138	.072	-.194	-.161	-.001	-.179	.064	.169	.106	.102	-.162	-.270	-.153	[illegible]
**BPNSF18**	-.159	-.069	-.048	-.113	.001	.051	-.067	.088	-.047	-.060	-.110	-.031	-.056	-.051	.014	[illegible]
**BPNSF1**	-.199	-.184	-.114	-.190	.225	.116	.074	.197	-.148	-.134	-.181	-.150	.256	.221	.164	[illegible]
**BPNSF7**	-.208	-.253	-.129	-.281	.204	.297	.211	.317	-.296	-.123	-.190	-.176	.080	.114	.088	[illegible]
**BPNSF13**	-.378	-.342	-.343	-.387	.311	.349	.592	.437	-.371	-.262	-.399	-.273	.266	.432	.353	[illegible]
**BPNSF19**	-.312	-.373	-.390	-.448	.298	.302	.413	.521	-.416	-.286	-.422	-.287	.177	.317	.330	[illegible]

Sample Correlations - Upper Bounds (BC) (g1 - Measurement intercepts)

	BPNSF6	BPNSF11	BPNSF17	BPNSF23	BPNSF3	BPNSF9	BPNSF14	BPNSF21	BPNSF2	BPNSF8	BPNSF20	BPNSF22	BPNSF4	BPNSF12	BPNSF16	BPNS[illegible]
**BPNSF6**	1.000															
**BPNSF11**	.641	1.000														
**BPNSF17**	.600	.616	1.000													
**BPNSF23**	.629	.639	.627	1.000												
**BPNSF3**	-.315	-.264	-.256	-.401	1.000											
**BPNSF9**	-.329	-.308	-.310	-.394	.589	1.000										
**BPNSF14**	-.266	-.248	-.295	-.348	.563	.627	1.000									
**BPNSF21**	-.223	-.227	-.174	-.334	.479	.549	.624	1.000								
**BPNSF2**	.558	.592	.507	.637	-.348	-.304	-.312	-.276	1.000							
**BPNSF8**	.607	.615	.587	.549	-.177	-.277	-.204	-.106	.609	1.000						
**BPNSF20**	.582	.567	.617	.694	-.323	-.310	-.302	-.245	.614	.602	1.000					
**BPNSF22**	.453	.519	.421	.548	-.237	-.207	-.112	-.117	.559	.630	.580	1.000				
**BPNSF4**	-.168	-.114	-.116	-.109	.476	.352	.450	.343	-.193	-.119	-.193	-.125	1.000			
**BPNSF12**	-.204	-.102	-.185	-.162	.411	.477	.566	.443	-.206	-.168	-.225	-.231	.499	1.000		
**BPNSF16**	-.250	-.192	-.221	-.261	.398	.486	.532	.455	-.200	-.201	-.235	-.196	.484	.666	1.000	
**BPNSF24**	-.153	-.133	-.178	-.205	.427	.524	.430	.409	-.287	-.189	-.249	-.244	.467	.628	.599	[illegible]
**BPNSF5**	.393	.441	.349	.348	.004	-.052	-.029	-.041	.389	.383	.336	.368	.057	-.127	-.163	[illegible]
**BPNSF10**	.412	.582	.407	.388	-.007	-.069	.013	-.031	.412	.483	.350	.316	-.040	-.119	-.107	[illegible]
**BPNSF15**	.294	.371	.303	.223	-.009	.018	.173	.002	.233	.355	.256	.268	.018	-.091	.034	[illegible]
**BPNSF18**	.005	.101	.128	.046	.165	.223	.127	.266	.101	.143	.046	.144	.108	.152	.205	[illegible]
**BPNSF1**	-.043	-.042	.045	-.047	.380	.275	.253	.360	-.002	.010	-.040	-.015	.413	.386	.304	[illegible]
**BPNSF7**	-.035	-.103	.013	-.141	.372	.455	.386	.475	-.150	.030	-.034	-.012	.244	.280	.250	[illegible]
**BPNSF13**	-.203	-.179	-.180	-.229	.469	.520	.713	.589	-.203	-.095	-.234	-.116	.416	.600	.516	[illegible]
**BPNSF19**	-.166	-.208	-.224	-.316	.474	.472	.588	.673	-.256	-.101	-.242	-.119	.349	.474	.506	[illegible]

Sample Correlations - Two Tailed Significance (BC) (g1 - Measurement intercepts)

	BPNSF6	BPNSF11	BPNSF17	BPNSF23	BPNSF3	BPNSF9	BPNSF14	BPNSF21	BPNSF2	BPNSF8	BPNSF20	BPNSF22	BPNSF4	BPNSF12	BPNSF16	BPNS[illegible]
**BPNSF6**	...															
**BPNSF11**	.003	...														
**BPNSF17**	.019	.012	...													
**BPNSF23**	.004	.007	.009	...												
**BPNSF3**	.003	.008	.007	.005	...											
**BPNSF9**	.003	.004	.008	.010	.007	...										
**BPNSF14**	.008	.003	.009	.007	.016	.021	...									
**BPNSF21**	.005	.011	.006	.012	.005	.008	.010	...								
**BPNSF2**	.009	.006	.006	.023	.009	.010	.013	.014	...							
**BPNSF8**	.010	.016	.005	.012	.005	.006	.004	.005	.003	...						
**BPNSF20**	.002	.005	.004	.007	.004	.006	.007	.010	.009	.005	...					
**BPNSF22**	.010	.008	.019	.016	.003	.003	.009	.007	.004	.006	.005	...				
**BPNSF4**	.007	.005	.005	.018	.006	.008	.023	.006	.007	.002	.003	.009	...			
**BPNSF12**	.008	.010	.019	.023	.019	.011	.012	.006	.013	.006	.015	.010	.012	...		
**BPNSF16**	.005	.005	.012	.009	.014	.013	.012	.014	.011	.009	.013	.007	.009	.012	...	
**BPNSF24**	.012	.016	.021	.013	.015	.006	.013	.006	.005	.005	.012	.011	.009	.012	.019	
**BPNSF5**	.013	.011	.014	.012	.111	.015	.044	.023	.011	.009	.019	.005	.594	.012	.006	.0[illegible]
**BPNSF10**	.011	.010	.006	.007	.081	.012	.177	.011	.011	.009	.005	.009	.027	.009	.003	.0[illegible]
**BPNSF15**	.021	.026	.012	.010	.085	.192	.117	.111	.009	.009	.018	.026	.164	.009	.284	.0[illegible]
**BPNSF18**	.144	.732	.525	.519	.096	.030	.606	.021	.611	.349	.372	.294	.670	.349	.070	.5[illegible]
**BPNSF1**	.015	.016	.503	.009	.006	.007	.014	.009	.093	.166	.011	.049	.010	.003	.007	.0[illegible]
**BPNSF7**	.007	.004	.174	.004	.005	.009	.009	.013	.012	.283	.006	.028	.009	.004	.012	.0[illegible]
**BPNSF13**	.005	.004	.011	.012	.010	.019	.007	.011	.009	.004	.003	.006	.009	.010	.005	.0[illegible]
**BPNSF19**	.007	.012	.005	.012	.005	.011	.011	.018	.013	.005	.007	.010	.007	.010	.005	.0[illegible]

Sample Means (g1 - Measurement intercepts)

Sample Means - Lower Bounds (BC) (g1 - Measurement intercepts)

	BPNSF6	BPNSF11	BPNSF17	BPNSF23	BPNSF3	BPNSF9	BPNSF14	BPNSF21	BPNSF2	BPNSF8	BPNSF20	BPNSF22	BPNSF4	BPNSF12	BPNSF16	BPNSF[illegible]
**BPNSF6**	2.352	2.588	2.316	1.968	5.611	5.703	5.446	5.227	2.118	2.631	2.225	2.898	4.947	5.113	4.999	5.12[illegible]

Sample Means - Upper Bounds (BC) (g1 - Measurement intercepts)

	BPNSF6	BPNSF11	BPNSF17	BPNSF23	BPNSF3	BPNSF9	BPNSF14	BPNSF21	BPNSF2	BPNSF8	BPNSF20	BPNSF22	BPNSF4	BPNSF12	BPNSF16	BPNSF[illegible]
**BPNSF6**	2.645	2.823	2.609	2.236	5.816	5.943	5.696	5.480	2.383	2.909	2.483	3.139	5.151	5.333	5.213	5.35[illegible]

Sample Means - Two Tailed Significance (BC) (g1 - Measurement intercepts)

	BPNSF6	BPNSF11	BPNSF17	BPNSF23	BPNSF3	BPNSF9	BPNSF14	BPNSF21	BPNSF2	BPNSF8	BPNSF20	BPNSF22	BPNSF4	BPNSF12	BPNSF16	BPNSF[illegible]
**BPNSF6**	.003	.005	.005	.003	.016	.023	.020	.015	.005	.009	.006	.011	.032	.019	.019	.01[illegible]

g2 (g2 - Measurement intercepts)

Estimates (g2 - Measurement intercepts)

Scalar Estimates (g2 - Measurement intercepts)

Maximum Likelihood Estimates

Regression Weights: (g2 - Measurement intercepts)

			Estimate	S.E.	C.R.	P	Label
BPNSF19	<---	F1	1.000				
BPNSF13	<---	F1	1.144	.052	22.216	***	a1_1
BPNSF7	<---	F1	.863	.050	17.162	***	a2_1
BPNSF1	<---	F1	.759	.050	15.166	***	a3_1
BPNSF18	<---	F2	1.000				
BPNSF15	<---	F2	2.454	.303	8.092	***	a4_1
BPNSF10	<---	F2	3.105	.370	8.386	***	a5_1
BPNSF5	<---	F2	2.516	.307	8.199	***	a6_1
BPNSF24	<---	F3	1.000				
BPNSF16	<---	F3	1.124	.046	24.274	***	a7_1
BPNSF12	<---	F3	1.221	.048	25.694	***	a8_1
BPNSF4	<---	F3	.846	.044	19.431	***	a9_1
BPNSF22	<---	F4	1.000				
BPNSF20	<---	F4	1.192	.049	24.555	***	a10_1
BPNSF8	<---	F4	1.197	.050	23.990	***	a11_1

			Estimate	S.E.	C.R.	P	Label
BPNSF2	<---	F4	1.120	.049	23.055	***	a12_1
BPNSF21	<---	F5	1.000				
BPNSF14	<---	F5	1.094	.042	26.011	***	a13_1
BPNSF9	<---	F5	1.038	.042	24.673	***	a14_1
BPNSF3	<---	F5	.882	.042	21.109	***	a15_1
BPNSF23	<---	F6	1.000				
BPNSF17	<---	F6	.976	.035	28.159	***	a16_1
BPNSF11	<---	F6	.959	.034	28.209	***	a17_1
BPNSF6	<---	F6	.861	.035	24.551	***	a18_1

Standardized Regression Weights: (g2 - Measurement intercepts)

			Estimate
BPNSF19	<---	F1	.600
BPNSF13	<---	F1	.761
BPNSF7	<---	F1	.558
BPNSF1	<---	F1	.508
BPNSF18	<---	F2	.268
BPNSF15	<---	F2	.584
BPNSF10	<---	F2	.733
BPNSF5	<---	F2	.644
BPNSF24	<---	F3	.698
BPNSF16	<---	F3	.780
BPNSF12	<---	F3	.842
BPNSF4	<---	F3	.610
BPNSF22	<---	F4	.681
BPNSF20	<---	F4	.765
BPNSF8	<---	F4	.724
BPNSF2	<---	F4	.730
BPNSF21	<---	F5	.706
BPNSF14	<---	F5	.779
BPNSF9	<---	F5	.716
BPNSF3	<---	F5	.611
BPNSF23	<---	F6	.780
BPNSF17	<---	F6	.749
BPNSF11	<---	F6	.696
BPNSF6	<---	F6	.629

Intercepts: (g2 - Measurement intercepts)

	Estimate	S.E.	C.R.	P	Label
**BPNSF19**	5.140	.040	127.940	***	i1_1
**BPNSF13**	5.043	.039	130.871	***	i2_1
**BPNSF7**	4.817	.040	119.340	***	i3_1
**BPNSF1**	4.705	.041	114.698	***	i4_1
**BPNSF18**	4.316	.041	104.417	***	i5_1
**BPNSF15**	3.699	.046	81.096	***	i6_1
**BPNSF10**	3.158	.045	69.442	***	i7_1
**BPNSF5**	3.721	.044	85.234	***	i8_1
**BPNSF24**	5.225	.037	140.058	***	i9_1
**BPNSF16**	5.118	.038	134.993	***	i10_1
**BPNSF12**	5.220	.038	136.344	***	i11_1
**BPNSF4**	5.116	.037	139.813	***	i12_1
**BPNSF22**	3.135	.043	73.443	***	i13_1
**BPNSF20**	2.524	.046	55.295	***	i14_1
**BPNSF8**	2.823	.047	59.807	***	i15_1
**BPNSF2**	2.400	.046	52.041	***	i16_1
**BPNSF21**	5.178	.039	131.278	***	i17_1
**BPNSF14**	5.441	.040	137.076	***	i18_1
**BPNSF9**	5.657	.040	141.770	***	i19_1
**BPNSF3**	5.591	.040	140.422	***	i20_1
**BPNSF23**	2.250	.045	49.691	***	i21_1
**BPNSF17**	2.586	.047	55.085	***	i22_1
**BPNSF11**	2.754	.046	59.623	***	i23_1
**BPNSF6**	2.574	.047	55.227	***	i24_1

Covariances: (g2 - Measurement intercepts)

			Estimate	S.E.	C.R.	P	Label
F1	<-->	F2	-.215	.037	-5.792	***	ccc1_2

			Estimate	S.E.	C.R.	P	Label
F2	<-->	F3	-.195	.035	-5.495	***	ccc2_2
F1	<-->	F3	.823	.079	10.436	***	ccc3_2
F2	<-->	F4	.366	.054	6.801	***	ccc4_2
F3	<-->	F4	-.743	.079	-9.418	***	ccc5_2
F1	<-->	F4	-.616	.074	-8.305	***	ccc6_2
F2	<-->	F5	-.194	.036	-5.395	***	ccc7_2
F4	<-->	F5	-.685	.078	-8.749	***	ccc8_2
F3	<-->	F5	.872	.081	10.718	***	ccc9_2
F1	<-->	F5	.955	.086	11.173	***	ccc10_2
F6	<-->	F5	-.912	.094	-9.721	***	ccc11_2
F6	<-->	F3	-.685	.085	-8.054	***	ccc12_2
F6	<-->	F4	1.228	.108	11.314	***	ccc13_2
F6	<-->	F2	.389	.058	6.674	***	ccc14_2
F6	<-->	F1	-.645	.083	-7.726	***	ccc15_2

Correlations: (g2 - Measurement intercepts)

			Estimate
F1	<-->	F2	-.569
F2	<-->	F3	-.471
F1	<-->	F3	.868
F2	<-->	F4	.832
F3	<-->	F4	-.673
F1	<-->	F4	-.610
F2	<-->	F5	-.464
F4	<-->	F5	-.611
F3	<-->	F5	.829
F1	<-->	F5	.993
F6	<-->	F5	-.692
F6	<-->	F3	-.528
F6	<-->	F4	.888
F6	<-->	F2	.752
F6	<-->	F1	-.543

Variances: (g2 - Measurement intercepts)

	Estimate	S.E.	C.R.	P	Label
**F1**	.867	.100	8.669	***	vvv1_2
**F2**	.164	.040	4.067	***	vvv2_2
**F3**	1.036	.103	10.057	***	vvv3_2
**F4**	1.175	.119	9.914	***	vvv4_2
**F5**	1.069	.106	10.113	***	vvv5_2
**F6**	1.626	.149	10.914	***	vvv6_2
**e1**	1.539	.111	13.817	***	v1_2
**e2**	.823	.071	11.653	***	v2_2
**e3**	1.423	.101	14.038	***	v3_2
**e4**	1.439	.101	14.234	***	v4_2
**e5**	2.130	.147	14.528	***	v5_2
**e6**	1.916	.146	13.097	***	v6_2
**e7**	1.365	.128	10.673	***	v7_2
**e8**	1.469	.118	12.417	***	v8_2
**e9**	1.093	.084	13.054	***	v9_2
**e10**	.843	.071	11.903	***	v10_2
**e11**	.633	.062	10.176	***	v11_2
**e12**	1.249	.091	13.705	***	v12_2
**e13**	1.360	.102	13.291	***	v13_2
**e14**	1.182	.096	12.254	***	v14_2
**e15**	1.531	.119	12.861	***	v15_2
**e16**	1.292	.101	12.777	***	v16_2
**e17**	1.073	.081	13.238	***	v17_2
**e18**	.828	.068	12.214	***	v18_2
**e19**	1.095	.083	13.140	***	v19_2
**e20**	1.396	.100	13.899	***	v20_2
**e21**	1.048	.089	11.739	***	v21_2
**e22**	1.209	.098	12.305	***	v22_2
**e23**	1.589	.122	13.006	***	v23_2
**e24**	1.843	.136	13.558	***	v24_2

Matrices (g2 - Measurement intercepts)

Residual Covariances (g2 - Measurement intercepts)

	BPNSF6	BPNSF11	BPNSF17	BPNSF23	BPNSF3	BPNSF9	BPNSF14	BPNSF21	BPNSF2	BPNSF8	BPNSF20	BPNSF22	BPNSF4	BPNSF12	BPNSF16	BPNS[illegible]
**BPNSF6**	-.114															
**BPNSF11**	-.157	-.192														
**BPNSF17**	-.021	.018	.137													
**BPNSF23**	-.078	-.119	.149	.055												
**BPNSF3**	-.162	.106	-.079	-.084	.048											
**BPNSF9**	.032	-.022	-.231	-.100	.151	-.048										
**BPNSF14**	.183	.172	-.155	.140	-.053	.039	-.090									
**BPNSF21**	.086	.026	.107	.023	.106	-.116	-.052	.082								
**BPNSF2**	.005	-.201	.024	-.002	-.089	.048	.037	.013	-.155							
**BPNSF8**	.026	-.154	-.007	-.158	.041	.059	.186	.067	-.094	-.161						
**BPNSF20**	-.185	-.111	.051	.276	-.131	.023	.006	-.181	-.057	-.067	.065					
**BPNSF22**	-.056	-.014	.097	.072	.143	-.048	.036	-.135	-.010	-.001	.207	.204				
**BPNSF4**	-.146	-.036	-.102	-.120	.754	.108	.018	.352	-.153	-.024	-.209	-.135	.163			
**BPNSF12**	.143	.094	-.035	.040	-.060	-.129	-.110	.015	.111	.240	.152	-.131	.074	-.126		
**BPNSF16**	.145	.045	-.031	.076	-.108	-.163	-.045	.194	.127	.199	-.003	-.126	.040	-.008	.003	
**BPNSF24**	.041	.009	-.252	-.044	-.140	-.115	.019	.108	-.049	-.016	-.169	-.392	.058	-.079	.085	[illegible]
**BPNSF5**	.059	-.274	-.218	-.083	-.012	.184	.178	-.029	-.194	.017	-.083	.032	-.312	.109	.151	[illegible]
**BPNSF10**	-.152	.244	.024	.043	-.097	-.147	-.082	-.285	-.154	-.023	.018	.143	-.198	.038	.026	[illegible]
**BPNSF15**	.046	.252	.069	-.081	.025	.178	.223	.029	-.150	.025	.043	.269	-.126	.094	.092	[illegible]
**BPNSF18**	-.117	.268	.202	-.023	.183	.072	.141	-.053	-.057	.066	.226	.156	-.006	.061	.016	[illegible]
**BPNSF1**	.135	.119	.105	.078	.176	-.090	-.029	.169	-.116	.087	-.075	-.074	.335	-.055	-.064	[illegible]
**BPNSF7**	.438	.020	-.142	.216	-.036	.094	-.051	-.011	.098	.030	.106	-.168	.163	-.114	-.049	[illegible]
**BPNSF13**	.102	-.069	.017	.057	-.057	-.214	-.058	.123	.155	.062	.061	-.135	.121	.055	-.019	[illegible]
**BPNSF19**	-.053	-.237	-.268	-.228	-.070	-.011	-.013	.198	.128	.044	-.109	-.181	.124	-.181	-.150	[illegible]

Residual Means (g2 - Measurement intercepts)

	BPNSF6	BPNSF11	BPNSF17	BPNSF23	BPNSF3	BPNSF9	BPNSF14	BPNSF21	BPNSF2	BPNSF8	BPNSF20	BPNSF22	BPNSF4	BPNSF12	BPNSF16	BPNSF24	BP[illegible]
	.042	.007	-.111	-.077	.028	.068	.166	.061	-.091	-.091	-.085	-.056	-.039	.094	.100	.085	[illegible]

Standardized Residual Covariances (g2 - Measurement intercepts)

	BPNSF6	BPNSF11	BPNSF17	BPNSF23	BPNSF3	BPNSF9	BPNSF14	BPNSF21	BPNSF2	BPNSF8	BPNSF20	BPNSF22	BPNSF4	BPNSF12	BPNSF16	BPNS[illegible]
**BPNSF6**	-.552															
**BPNSF11**	-.980	-.918														
**BPNSF17**	-.137	.112	.735													
**BPNSF23**	-.515	-.761	.992	.302												
**BPNSF3**	-1.254	.807	-.631	-.682	.317											
**BPNSF9**	.240	-.163	-1.819	-.796	1.288	-.316										
**BPNSF14**	1.426	1.317	-1.247	1.138	-.461	.330	-.630									
**BPNSF21**	.674	.198	.866	.188	.929	-.989	-.450	.568								
**BPNSF2**	.034	-1.310	.164	-.013	-.722	.380	.303	.106	-.828							
**BPNSF8**	.163	-.931	-.046	-1.008	.310	.436	1.411	.511	-.583	-.739						
**BPNSF20**	-1.204	-.706	.340	1.841	-1.041	.179	.045	-1.455	-.368	-.403	.336					
**BPNSF22**	-.393	-.095	.695	.520	1.217	-.404	.310	-1.160	-.067	-.008	1.426	1.188				
**BPNSF4**	-1.212	-.297	-.884	-1.052	7.142	1.003	.175	3.351	-1.304	-.192	-1.747	-1.210	1.207			
**BPNSF12**	1.113	.722	-.284	.330	-.520	-1.089	-.943	.128	.877	1.755	1.167	-1.087	.663	-.855		
**BPNSF16**	1.146	.347	-.251	.630	-.956	-1.401	-.391	1.720	1.015	1.473	-.025	-1.060	.363	-.066	.022	
**BPNSF24**	.331	.071	-2.092	-.373	-1.269	-1.017	.172	.978	-.402	-.123	-1.345	-3.356	.542	-.662	.731	[illegible]
**BPNSF5**	.423	-1.952	-1.630	-.629	-.103	1.581	1.571	-.255	-1.433	.114	-.601	.250	-2.869	.947	1.318	[illegible]
**BPNSF10**	-1.003	1.580	.166	.293	-.772	-1.155	-.661	-2.302	-1.030	-.144	.117	1.007	-1.667	.297	.209	[illegible]
**BPNSF15**	.309	1.679	.487	-.576	.205	1.424	1.838	.241	-1.040	.163	.291	1.966	-1.077	.758	.753	[illegible]
**BPNSF18**	-.918	2.084	1.657	-.192	1.685	.657	1.330	-.494	-.466	.501	1.821	1.334	-.055	.569	.153	[illegible]
**BPNSF1**	1.142	.996	.932	.697	1.692	-.848	-.282	1.630	-1.023	.710	-.648	-.680	3.438	-.524	-.619	[illegible]
**BPNSF7**	3.582	.158	-1.210	1.866	-.333	.848	-.471	-.105	.827	.234	.883	-1.492	1.609	-1.042	-.457	[illegible]
**BPNSF13**	.842	-.566	.145	.497	-.520	-1.872	-.516	1.102	1.315	.493	.506	-1.206	1.189	.489	-.171	[illegible]
**BPNSF19**	-.402	-1.771	-2.113	-1.816	-.597	-.095	-.113	1.680	.998	.316	-.836	-1.482	1.127	-1.516	-1.274	[illegible]

Standardized Residual Means (g2 - Measurement intercepts)

	BPNSF6	BPNSF11	BPNSF17	BPNSF23	BPNSF3	BPNSF9	BPNSF14	BPNSF21	BPNSF2	BPNSF8	BPNSF20	BPNSF22	BPNSF4	BPNSF12	BPNSF16	BPNSF24	BP[illegible]
	.505	.083	-1.401	-.982	.399	.950	2.381	.877	-1.140	-1.064	-1.049	-.734	-.575	1.336	1.430	1.224	[illegible]

Notes for Group/Model (g2 - Measurement intercepts)

The following covariance matrix is not positive definite (g2 - Measurement intercepts)

	F5	F4	F3	F2	F1	F6
F5	1.069					
F4	-.685	1.175				
F3	.872	-.743	1.036			
F2	-.194	.366	-.195	.164		
F1	.955	-.616	.823	-.215	.867	
F6	-.912	1.228	-.685	.389	-.645	1.626

This solution is not admissible.

Modification Indices (g2 - Measurement intercepts)

Covariances: (g2 - Measurement intercepts)

			M.I.	Par Change
e24	<-->	F1	4.970	.096
e23	<-->	F4	4.367	-.093
e23	<-->	F2	6.267	.057
e23	<-->	F1	5.432	-.095
e20	<-->	e24	8.007	-.232
e19	<-->	F6	4.656	-.098
e19	<-->	e20	6.698	.165
e18	<-->	e22	9.112	-.168
e18	<-->	e19	6.441	.130
e17	<-->	F5	5.175	-.070
e17	<-->	F1	5.348	.075
e17	<-->	e22	13.234	.225
e17	<-->	e19	8.225	-.163
e15	<-->	e24	4.846	.196
e15	<-->	e21	5.031	-.159
e14	<-->	e24	6.788	-.207
e14	<-->	e21	17.427	.265
e13	<-->	F5	9.447	.115
e13	<-->	F3	5.967	-.101
e13	<-->	F1	5.197	-.085
e13	<-->	e20	15.106	.276
e12	<-->	F5	14.773	.135
e12	<-->	F3	5.443	-.090
e12	<-->	F2	6.380	-.050
e12	<-->	F6	4.327	.100
e12	<-->	e22	4.575	.141
e12	<-->	e20	101.483	.678
e12	<-->	e18	9.094	-.164
e12	<-->	e17	4.758	.131
e11	<-->	e14	7.147	.141
e10	<-->	e20	5.783	-.141
e10	<-->	e17	7.723	.146
e9	<-->	F4	4.903	-.082
e9	<-->	e20	9.175	-.195
e9	<-->	e13	9.635	-.201
e8	<-->	e24	8.743	.261
e8	<-->	e23	8.812	-.247
e8	<-->	e22	4.369	-.155
e8	<-->	e12	12.796	-.258
e7	<-->	F5	7.693	-.112
e7	<-->	e23	8.538	.247
e6	<-->	F5	5.707	.106
e6	<-->	e23	7.159	.249
e5	<-->	e23	4.573	.201
e5	<-->	e20	4.246	.176
e5	<-->	e6	7.598	.283
e4	<-->	F6	10.800	.167
e4	<-->	e22	4.297	.144
e4	<-->	e20	5.025	.158
e4	<-->	e16	5.730	-.169
e4	<-->	e12	7.815	.189
e4	<-->	e7	8.658	.227
e4	<-->	e6	13.875	-.316
e4	<-->	e5	6.443	-.218
e3	<-->	e24	18.122	.350
e3	<-->	e22	7.632	-.192
e3	<-->	e21	6.155	.163
e3	<-->	e19	6.725	.165

			M.I.	Par Change
e3	<-->	e13	5.059	-.160
e2	<-->	F3	5.800	.078
e2	<-->	e23	5.624	-.146
e2	<-->	e19	11.742	-.172
e2	<-->	e12	4.830	-.117
e2	<-->	e11	11.931	.149
e1	<-->	F6	6.844	-.138
e1	<-->	e17	5.090	.148
e1	<-->	e16	4.933	.164

Variances: (g2 - Measurement intercepts)

	M.I.	Par Change

Regression Weights: (g2 - Measurement intercepts)

			M.I.	Par Change
BPNSF6	<---	BPNSF7	7.069	.036
BPNSF11	<---	BPNSF15	4.007	.031
BPNSF3	<---	BPNSF6	4.892	-.041
BPNSF14	<---	BPNSF6	4.072	.030
BPNSF14	<---	BPNSF11	5.822	.035
BPNSF14	<---	BPNSF23	5.739	.040
BPNSF14	<---	BPNSF22	5.204	.030
BPNSF14	<---	BPNSF5	4.218	.024
BPNSF14	<---	BPNSF15	5.607	.027
BPNSF14	<---	BPNSF18	4.083	.021
BPNSF21	<---	F3	4.753	.118
BPNSF2	<---	F2	4.414	-.333
BPNSF22	<---	F3	6.734	-.159
BPNSF22	<---	F2	4.953	.357
BPNSF4	<---	F5	9.124	.170
BPNSF4	<---	F4	4.048	-.109
BPNSF4	<---	F2	7.325	-.411
BPNSF4	<---	F1	13.030	.224
BPNSF4	<---	F6	5.755	-.111
BPNSF4	<---	BPNSF6	8.849	-.053
BPNSF4	<---	BPNSF11	4.373	-.035
BPNSF4	<---	BPNSF23	5.875	-.048
BPNSF4	<---	BPNSF2	8.064	-.054
BPNSF4	<---	BPNSF8	5.226	-.038
BPNSF4	<---	BPNSF20	8.314	-.053
BPNSF4	<---	BPNSF5	11.827	-.047
BPNSF4	<---	BPNSF10	6.828	-.040
BPNSF4	<---	BPNSF15	6.280	-.034
BPNSF24	<---	F4	4.339	-.108
BPNSF24	<---	F2	4.409	-.305
BPNSF5	<---	BPNSF11	4.153	-.039
BPNSF10	<---	BPNSF11	6.369	.049
BPNSF18	<---	BPNSF6	7.269	-.061
BPNSF18	<---	BPNSF23	4.898	-.056
BPNSF18	<---	BPNSF3	4.563	-.026
BPNSF18	<---	BPNSF9	6.094	-.030
BPNSF18	<---	BPNSF14	5.618	-.030
BPNSF18	<---	BPNSF21	7.134	-.035
BPNSF18	<---	BPNSF2	4.827	-.053
BPNSF18	<---	BPNSF4	5.950	-.032
BPNSF18	<---	BPNSF12	6.376	-.033
BPNSF18	<---	BPNSF16	7.009	-.035
BPNSF18	<---	BPNSF24	7.028	-.034
BPNSF18	<---	BPNSF10	4.646	-.042
BPNSF18	<---	BPNSF1	9.902	-.045
BPNSF18	<---	BPNSF7	6.413	-.035
BPNSF18	<---	BPNSF13	5.838	-.033
BPNSF18	<---	BPNSF19	6.339	-.033
BPNSF1	<---	BPNSF11	4.203	.037
BPNSF1	<---	BPNSF17	5.048	.043
BPNSF1	<---	BPNSF3	4.649	.022
BPNSF1	<---	BPNSF21	4.234	.022
BPNSF1	<---	BPNSF4	5.418	.026
BPNSF1	<---	BPNSF10	4.422	.034

			M.I.	Par Change
BPNSF1	<---	BPNSF13	4.099	.023
BPNSF19	<---	F6	5.436	-.118

Means: (g2 - Measurement intercepts)

	M.I.	Par Change

Intercepts: (g2 - Measurement intercepts)

	M.I.	Par Change
**BPNSF18**	7.190	-.189

Bootstrap (g2 - Measurement intercepts)

Bootstrap standard errors (g2 - Measurement intercepts)

Scalar Estimates (g2 - Measurement intercepts)

Regression Weights: (g2 - Measurement intercepts)

Parameter			SE	SE-SE	Mean	Bias	SE-Bias
BPNSF19	<---	F1	.000	.000	1.000	.000	.000
BPNSF13	<---	F1	.057	.003	1.139	-.005	.004
BPNSF7	<---	F1	.059	.003	.862	.000	.004
BPNSF1	<---	F1	.071	.004	.758	-.001	.005
BPNSF18	<---	F2	.000	.000	1.000	.000	.000
BPNSF15	<---	F2	.387	.019	2.498	.043	.027
BPNSF10	<---	F2	.513	.026	3.163	.058	.036
BPNSF5	<---	F2	.410	.021	2.563	.047	.029
BPNSF24	<---	F3	.000	.000	1.000	.000	.000
BPNSF16	<---	F3	.054	.003	1.125	.000	.004
BPNSF12	<---	F3	.049	.002	1.215	-.006	.003
BPNSF4	<---	F3	.051	.003	.844	-.002	.004
BPNSF22	<---	F4	.000	.000	1.000	.000	.000
BPNSF20	<---	F4	.051	.003	1.191	-.001	.004
BPNSF8	<---	F4	.050	.002	1.191	-.005	.004
BPNSF2	<---	F4	.054	.003	1.118	-.002	.004
BPNSF21	<---	F5	.000	.000	1.000	.000	.000
BPNSF14	<---	F5	.045	.002	1.096	.002	.003
BPNSF9	<---	F5	.052	.003	1.040	.002	.004
BPNSF3	<---	F5	.059	.003	.887	.005	.004
BPNSF23	<---	F6	.000	.000	1.000	.000	.000
BPNSF17	<---	F6	.034	.002	.979	.003	.002
BPNSF11	<---	F6	.038	.002	.959	.000	.003
BPNSF6	<---	F6	.037	.002	.861	.000	.003

Standardized Regression Weights: (g2 - Measurement intercepts)

Parameter			SE	SE-SE	Mean	Bias	SE-Bias
BPNSF19	<---	F1	.038	.002	.603	.002	.003
BPNSF13	<---	F1	.028	.001	.761	.000	.002
BPNSF7	<---	F1	.042	.002	.560	.002	.003
BPNSF1	<---	F1	.037	.002	.509	.001	.003
BPNSF18	<---	F2	.043	.002	.269	.001	.003
BPNSF15	<---	F2	.038	.002	.585	.001	.003
BPNSF10	<---	F2	.037	.002	.736	.003	.003
BPNSF5	<---	F2	.038	.002	.644	.000	.003
BPNSF24	<---	F3	.032	.002	.700	.002	.002
BPNSF16	<---	F3	.027	.001	.782	.002	.002
BPNSF12	<---	F3	.019	.001	.843	.001	.001
BPNSF4	<---	F3	.040	.002	.609	-.001	.003
BPNSF22	<---	F4	.028	.001	.680	-.001	.002
BPNSF20	<---	F4	.031	.002	.768	.002	.002
BPNSF8	<---	F4	.029	.001	.721	-.003	.002
BPNSF2	<---	F4	.030	.002	.732	.002	.002
BPNSF21	<---	F5	.030	.001	.706	-.001	.002
BPNSF14	<---	F5	.034	.002	.780	.001	.002
BPNSF9	<---	F5	.036	.002	.718	.002	.003
BPNSF3	<---	F5	.045	.002	.615	.004	.003

Parameter			SE	SE-SE	Mean	Bias	SE-Bias
BPNSF23	<---	F6	.029	.001	.780	.000	.002
BPNSF17	<---	F6	.032	.002	.752	.003	.002
BPNSF11	<---	F6	.030	.002	.696	-.001	.002
BPNSF6	<---	F6	.030	.001	.628	-.001	.002

Intercepts: (g2 - Measurement intercepts)

Parameter	SE	SE-SE	Mean	Bias	SE-Bias
**BPNSF19**	.045	.002	5.148	.008	.003
**BPNSF13**	.042	.002	5.052	.009	.003
**BPNSF7**	.042	.002	4.827	.009	.003
**BPNSF1**	.040	.002	4.713	.008	.003
**BPNSF18**	.041	.002	4.315	-.001	.003
**BPNSF15**	.047	.002	3.698	-.002	.003
**BPNSF10**	.051	.003	3.153	-.005	.004
**BPNSF5**	.047	.002	3.717	-.004	.003
**BPNSF24**	.041	.002	5.232	.006	.003
**BPNSF16**	.044	.002	5.125	.008	.003
**BPNSF12**	.039	.002	5.228	.008	.003
**BPNSF4**	.039	.002	5.121	.006	.003
**BPNSF22**	.047	.002	3.128	-.007	.003
**BPNSF20**	.054	.003	2.512	-.013	.004
**BPNSF8**	.055	.003	2.817	-.006	.004
**BPNSF2**	.048	.002	2.393	-.007	.003
**BPNSF21**	.042	.002	5.189	.011	.003
**BPNSF14**	.044	.002	5.452	.011	.003
**BPNSF9**	.046	.002	5.666	.009	.003
**BPNSF3**	.043	.002	5.600	.009	.003
**BPNSF23**	.046	.002	2.239	-.010	.003
**BPNSF17**	.053	.003	2.574	-.011	.004
**BPNSF11**	.049	.002	2.749	-.005	.003
**BPNSF6**	.053	.003	2.570	-.004	.004

Covariances: (g2 - Measurement intercepts)

Parameter			SE	SE-SE	Mean	Bias	SE-Bias
F1	<-->	F2	.044	.002	-.220	-.005	.003
F2	<-->	F3	.048	.002	-.200	-.006	.003
F1	<-->	F3	.085	.004	.827	.004	.006
F2	<-->	F4	.070	.003	.370	.004	.005
F3	<-->	F4	.084	.004	-.754	-.011	.006
F1	<-->	F4	.073	.004	-.623	-.007	.005
F2	<-->	F5	.043	.002	-.198	-.003	.003
F4	<-->	F5	.072	.004	-.685	.000	.005
F3	<-->	F5	.095	.005	.872	.000	.007
F1	<-->	F5	.102	.005	.959	.004	.007
F6	<-->	F5	.091	.005	-.912	.000	.006
F6	<-->	F3	.090	.005	-.692	-.007	.006
F6	<-->	F4	.100	.005	1.225	-.002	.007
F6	<-->	F2	.074	.004	.390	.001	.005
F6	<-->	F1	.087	.004	-.656	-.011	.006

Correlations: (g2 - Measurement intercepts)

Parameter			SE	SE-SE	Mean	Bias	SE-Bias
F1	<-->	F2	.053	.003	-.576	-.007	.004
F2	<-->	F3	.061	.003	-.477	-.006	.004
F1	<-->	F3	.040	.002	.866	-.003	.003
F2	<-->	F4	.032	.002	.830	-.002	.002
F3	<-->	F4	.046	.002	-.679	-.005	.003
F1	<-->	F4	.052	.003	-.613	-.003	.004
F2	<-->	F5	.059	.003	-.467	-.004	.004
F4	<-->	F5	.049	.002	-.611	.000	.003
F3	<-->	F5	.039	.002	.825	-.004	.003
F1	<-->	F5	.035	.002	.992	-.001	.002
F6	<-->	F5	.046	.002	-.693	-.001	.003
F6	<-->	F3	.056	.003	-.532	-.004	.004
F6	<-->	F4	.035	.002	.886	-.002	.002
F6	<-->	F2	.047	.002	.747	-.005	.003
F6	<-->	F1	.059	.003	-.551	-.008	.004

Variances: (g2 - Measurement intercepts)

Parameter	SE	SE-SE	Mean	Bias	SE-Bias
**F1**	.104	.005	.877	.010	.007
**F2**	.056	.003	.172	.007	.004
**F3**	.116	.006	1.047	.011	.008
**F4**	.108	.005	1.181	.006	.008
**F5**	.119	.006	1.070	.001	.008
**F6**	.151	.008	1.626	.000	.011
**e1**	.186	.009	1.537	-.002	.013
**e2**	.095	.005	.817	-.006	.007
**e3**	.158	.008	1.418	-.005	.011
**e4**	.115	.006	1.429	-.010	.008
**e5**	.138	.007	2.143	.012	.010
**e6**	.168	.008	1.919	.003	.012
**e7**	.183	.009	1.349	-.016	.013
**e8**	.152	.008	1.479	.009	.011
**e9**	.129	.006	1.088	-.004	.009
**e10**	.103	.005	.837	-.006	.007
**e11**	.073	.004	.625	-.007	.005
**e12**	.143	.007	1.256	.007	.010
**e13**	.123	.006	1.369	.009	.009
**e14**	.175	.009	1.165	-.016	.012
**e15**	.190	.009	1.545	.014	.013
**e16**	.180	.009	1.275	-.017	.013
**e17**	.115	.006	1.076	.002	.008
**e18**	.134	.007	.822	-.007	.009
**e19**	.171	.009	1.086	-.009	.012
**e20**	.202	.010	1.383	-.013	.014
**e21**	.151	.008	1.045	-.003	.011
**e22**	.175	.009	1.194	-.015	.012
**e23**	.196	.010	1.593	.004	.014
**e24**	.203	.010	1.852	.009	.014

Matrices (g2 - Measurement intercepts)

Sample Covariances - Standard Errors (g2 - Measurement intercepts)

	BPNSF6	BPNSF11	BPNSF17	BPNSF23	BPNSF3	BPNSF9	BPNSF14	BPNSF21	BPNSF2	BPNSF8	BPNSF20	BPNSF22	BPNSF4	BPNSF12	BPNSF16	BPNS[illegible]
**BPNSF6**	.181															
**BPNSF11**	.156	.172														
**BPNSF17**	.159	.159	.203													
**BPNSF23**	.150	.147	.164	.192												
**BPNSF3**	.134	.128	.124	.133	.183											
**BPNSF9**	.125	.137	.149	.135	.133	.194										
**BPNSF14**	.115	.139	.136	.123	.134	.156	.159									
**BPNSF21**	.120	.132	.136	.132	.123	.132	.139	.158								
**BPNSF2**	.143	.130	.136	.149	.107	.111	.112	.104	.169							
**BPNSF8**	.159	.151	.162	.146	.128	.128	.131	.134	.137	.169						
**BPNSF20**	.161	.141	.153	.171	.141	.137	.144	.144	.140	.157	.199					
**BPNSF22**	.147	.136	.136	.139	.120	.128	.133	.137	.125	.148	.139	.144				
**BPNSF4**	.132	.127	.120	.139	.136	.121	.123	.120	.111	.126	.147	.131	.143			
**BPNSF12**	.129	.132	.136	.135	.137	.136	.141	.133	.116	.129	.137	.127	.121	.140		
**BPNSF16**	.122	.126	.128	.133	.138	.132	.121	.138	.118	.136	.158	.134	.127	.133	.162	
**BPNSF24**	.122	.124	.135	.129	.132	.130	.124	.126	.129	.138	.142	.133	.128	.135	.134	.1[illegible]
**BPNSF5**	.137	.135	.143	.150	.135	.119	.110	.132	.111	.147	.163	.151	.139	.133	.122	.1[illegible]
**BPNSF10**	.145	.141	.146	.148	.138	.130	.122	.133	.128	.144	.152	.149	.140	.133	.135	.1[illegible]
**BPNSF15**	.136	.144	.159	.146	.136	.120	.121	.135	.136	.159	.153	.151	.138	.137	.134	.1[illegible]
**BPNSF18**	.138	.141	.140	.137	.119	.113	.103	.121	.128	.142	.135	.142	.133	.138	.122	.1[illegible]
**BPNSF1**	.104	.114	.101	.117	.106	.111	.109	.109	.113	.142	.142	.136	.118	.110	.105	.1[illegible]
**BPNSF7**	.129	.130	.138	.116	.134	.137	.123	.119	.102	.141	.119	.125	.115	.129	.121	.1[illegible]
**BPNSF13**	.119	.125	.124	.119	.112	.124	.131	.119	.104	.119	.124	.119	.106	.116	.115	.1[illegible]
**BPNSF19**	.130	.138	.134	.136	.122	.152	.134	.135	.103	.132	.141	.127	.125	.132	.120	.1[illegible]

Sample Correlations - Standard Errors (g2 - Measurement intercepts)

	BPNSF6	BPNSF11	BPNSF17	BPNSF23	BPNSF3	BPNSF9	BPNSF14	BPNSF21	BPNSF2	BPNSF8	BPNSF20	BPNSF22	BPNSF4	BPNSF12	BPNSF16	BPNS[illegible]
**BPNSF6**	.000															
**BPNSF11**	.049	.000														
**BPNSF17**	.052	.047	.000													
**BPNSF23**	.046	.044	.045	.000												

	BPNSF6	BPNSF11	BPNSF17	BPNSF23	BPNSF3	BPNSF9	BPNSF14	BPNSF21	BPNSF2	BPNSF8	BPNSF20	BPNSF22	BPNSF4	BPNSF12	BPNSF16	BPNS[illegible]
**BPNSF3**	.053	.052	.050	.053	.000											
**BPNSF9**	.051	.047	.052	.049	.058	.000										
**BPNSF14**	.047	.054	.047	.050	.059	.052	.000									
**BPNSF21**	.045	.049	.052	.046	.046	.050	.052	.000								
**BPNSF2**	.048	.047	.046	.048	.046	.047	.047	.041	.000							
**BPNSF8**	.049	.050	.046	.045	.049	.047	.051	.049	.043	.000						
**BPNSF20**	.052	.045	.045	.043	.052	.051	.054	.048	.047	.045	.000					
**BPNSF22**	.050	.047	.045	.043	.050	.049	.054	.051	.042	.045	.038	.000				
**BPNSF4**	.050	.051	.045	.051	.036	.048	.049	.039	.045	.048	.050	.051	.000			
**BPNSF12**	.052	.053	.051	.050	.058	.053	.049	.042	.048	.050	.050	.045	.047	.000		
**BPNSF16**	.049	.048	.049	.052	.062	.056	.044	.046	.046	.049	.052	.046	.051	.036	.000	
**BPNSF24**	.050	.049	.050	.050	.060	.055	.047	.047	.047	.048	.049	.043	.051	.045	.042	.0[illegible]
**BPNSF5**	.046	.050	.051	.051	.054	.050	.048	.054	.040	.048	.053	.052	.052	.059	.052	.0[illegible]
**BPNSF10**	.048	.043	.043	.042	.053	.049	.047	.045	.046	.044	.045	.047	.053	.053	.051	.0[illegible]
**BPNSF15**	.044	.045	.052	.047	.052	.046	.049	.051	.047	.049	.047	.047	.052	.054	.050	.0[illegible]
**BPNSF18**	.053	.050	.051	.053	.051	.049	.048	.052	.051	.052	.048	.054	.059	.063	.053	.0[illegible]
**BPNSF1**	.043	.048	.042	.049	.047	.051	.047	.045	.047	.055	.055	.055	.051	.051	.048	.0[illegible]
**BPNSF7**	.052	.054	.052	.050	.059	.051	.051	.050	.046	.054	.049	.052	.050	.055	.054	.0[illegible]
**BPNSF13**	.050	.050	.050	.049	.050	.052	.047	.038	.045	.046	.047	.046	.043	.035	.046	.0[illegible]
**BPNSF19**	.050	.052	.049	.051	.051	.056	.054	.047	.043	.048	.050	.050	.048	.052	.051	.0[illegible]

Sample Means - Standard Errors (g2 - Measurement intercepts)

	BPNSF6	BPNSF11	BPNSF17	BPNSF23	BPNSF3	BPNSF9	BPNSF14	BPNSF21	BPNSF2	BPNSF8	BPNSF20	BPNSF22	BPNSF4	BPNSF12	BPNSF16	BPNSF[illegible]
**BPNSF6**	.079	.090	.088	.076	.073	.074	.067	.076	.078	.091	.087	.088	.073	.067	.072	.07[illegible]

Bootstrap Confidence (g2 - Measurement intercepts)

Percentile method (g2 - Measurement intercepts)

90% confidence intervals (percentile method)

Scalar Estimates (g2 - Measurement intercepts)

Regression Weights: (g2 - Measurement intercepts)

Parameter			Estimate	Lower	Upper	P
BPNSF19	<---	F1	1.000	1.000	1.000	...
BPNSF13	<---	F1	1.144	1.043	1.237	.010
BPNSF7	<---	F1	.863	.764	.963	.010
BPNSF1	<---	F1	.759	.644	.885	.010
BPNSF18	<---	F2	1.000	1.000	1.000	...
BPNSF15	<---	F2	2.454	1.993	3.280	.010
BPNSF10	<---	F2	3.105	2.497	4.232	.010
BPNSF5	<---	F2	2.516	2.001	3.370	.010
BPNSF24	<---	F3	1.000	1.000	1.000	...
BPNSF16	<---	F3	1.124	1.038	1.214	.010
BPNSF12	<---	F3	1.221	1.136	1.296	.010
BPNSF4	<---	F3	.846	.752	.934	.010
BPNSF22	<---	F4	1.000	1.000	1.000	...
BPNSF20	<---	F4	1.192	1.111	1.283	.010
BPNSF8	<---	F4	1.197	1.114	1.272	.010
BPNSF2	<---	F4	1.120	1.035	1.222	.010
BPNSF21	<---	F5	1.000	1.000	1.000	...
BPNSF14	<---	F5	1.094	1.022	1.177	.010
BPNSF9	<---	F5	1.038	.963	1.130	.010
BPNSF3	<---	F5	.882	.793	.989	.010
BPNSF23	<---	F6	1.000	1.000	1.000	...
BPNSF17	<---	F6	.976	.925	1.043	.010
BPNSF11	<---	F6	.959	.899	1.022	.010
BPNSF6	<---	F6	.861	.801	.930	.010

Standardized Regression Weights: (g2 - Measurement intercepts)

Parameter			Estimate	Lower	Upper	P
BPNSF19	<---	F1	.600	.540	.666	.010
BPNSF13	<---	F1	.761	.712	.807	.010
BPNSF7	<---	F1	.558	.490	.634	.010
BPNSF1	<---	F1	.508	.448	.569	.010

Parameter			Estimate	Lower	Upper	P
BPNSF18	<---	F2	.268	.196	.344	.010
BPNSF15	<---	F2	.584	.521	.644	.010
BPNSF10	<---	F2	.733	.672	.790	.010
BPNSF5	<---	F2	.644	.578	.711	.010
BPNSF24	<---	F3	.698	.643	.746	.010
BPNSF16	<---	F3	.780	.733	.820	.010
BPNSF12	<---	F3	.842	.808	.872	.010
BPNSF4	<---	F3	.610	.548	.686	.010
BPNSF22	<---	F4	.681	.634	.723	.010
BPNSF20	<---	F4	.765	.718	.817	.010
BPNSF8	<---	F4	.724	.670	.764	.010
BPNSF2	<---	F4	.730	.682	.788	.010
BPNSF21	<---	F5	.706	.656	.759	.010
BPNSF14	<---	F5	.779	.722	.836	.010
BPNSF9	<---	F5	.716	.653	.775	.010
BPNSF3	<---	F5	.611	.538	.688	.010
BPNSF23	<---	F6	.780	.732	.826	.010
BPNSF17	<---	F6	.749	.707	.813	.010
BPNSF11	<---	F6	.696	.647	.748	.010
BPNSF6	<---	F6	.629	.579	.679	.010

Intercepts: (g2 - Measurement intercepts)

Parameter	Estimate	Lower	Upper	P
**BPNSF19**	5.140	5.067	5.217	.010
**BPNSF13**	5.043	4.979	5.123	.010
**BPNSF7**	4.817	4.755	4.891	.010
**BPNSF1**	4.705	4.648	4.786	.010
**BPNSF18**	4.316	4.250	4.387	.010
**BPNSF15**	3.699	3.616	3.773	.010
**BPNSF10**	3.158	3.066	3.227	.010
**BPNSF5**	3.721	3.641	3.789	.010
**BPNSF24**	5.225	5.160	5.302	.010
**BPNSF16**	5.118	5.059	5.200	.010
**BPNSF12**	5.220	5.163	5.293	.010
**BPNSF4**	5.116	5.057	5.187	.010
**BPNSF22**	3.135	3.051	3.203	.010
**BPNSF20**	2.524	2.403	2.600	.010
**BPNSF8**	2.823	2.727	2.907	.010
**BPNSF2**	2.400	2.308	2.469	.010
**BPNSF21**	5.178	5.107	5.260	.010
**BPNSF14**	5.441	5.379	5.529	.010
**BPNSF9**	5.657	5.589	5.737	.010
**BPNSF3**	5.591	5.534	5.683	.010
**BPNSF23**	2.250	2.163	2.317	.010
**BPNSF17**	2.586	2.485	2.662	.010
**BPNSF11**	2.754	2.667	2.833	.010
**BPNSF6**	2.574	2.483	2.656	.010

Covariances: (g2 - Measurement intercepts)

Parameter			Estimate	Lower	Upper	P
F1	<-->	F2	-.215	-.300	-.151	.010
F2	<-->	F3	-.195	-.291	-.129	.010
F1	<-->	F3	.823	.695	.961	.010
F2	<-->	F4	.366	.257	.483	.010
F3	<-->	F4	-.743	-.895	-.610	.010
F1	<-->	F4	-.616	-.748	-.508	.010
F2	<-->	F5	-.194	-.280	-.130	.010
F4	<-->	F5	-.685	-.810	-.577	.010
F3	<-->	F5	.872	.723	1.048	.010
F1	<-->	F5	.955	.818	1.160	.010
F6	<-->	F5	-.912	-1.084	-.774	.010
F6	<-->	F3	-.685	-.832	-.534	.010
F6	<-->	F4	1.228	1.060	1.390	.010
F6	<-->	F2	.389	.275	.520	.010
F6	<-->	F1	-.645	-.818	-.516	.010

Correlations: (g2 - Measurement intercepts)

Parameter	Estimate	Lower	Upper	P

Parameter	Estimate	Lower	Upper	P
F1 <--> F2	-.569	-.653	-.475	.010
F2 <--> F3	-.471	-.578	-.376	.010
F1 <--> F3	.868	.792	.924	.010
F2 <--> F4	.832	.778	.880	.010
F3 <--> F4	-.673	-.746	-.598	.010
F1 <--> F4	-.610	-.697	-.523	.010
F2 <--> F5	-.464	-.570	-.365	.010
F4 <--> F5	-.611	-.687	-.526	.010
F3 <--> F5	.829	.764	.882	.010
F1 <--> F5	.993	.919	1.040	.010
F6 <--> F5	-.692	-.766	-.617	.010
F6 <--> F3	-.528	-.621	-.435	.010
F6 <--> F4	.888	.827	.943	.010
F6 <--> F2	.752	.673	.830	.010
F6 <--> F1	-.543	-.654	-.451	.010

Variances: (g2 - Measurement intercepts)

Parameter	Estimate	Lower	Upper	P
**F1**	.867	.720	1.061	.010
**F2**	.164	.089	.270	.010
**F3**	1.036	.858	1.245	.010
**F4**	1.175	1.020	1.357	.010
**F5**	1.069	.911	1.290	.010
**F6**	1.626	1.377	1.896	.010
**e1**	1.539	1.220	1.858	.010
**e2**	.823	.632	.983	.010
**e3**	1.423	1.123	1.694	.010
**e4**	1.439	1.245	1.623	.010
**e5**	2.130	1.907	2.385	.010
**e6**	1.916	1.626	2.192	.010
**e7**	1.365	1.060	1.702	.010
**e8**	1.469	1.247	1.732	.010
**e9**	1.093	.843	1.297	.010
**e10**	.843	.661	1.001	.010
**e11**	.633	.517	.740	.010
**e12**	1.249	1.018	1.490	.010
**e13**	1.360	1.161	1.562	.010
**e14**	1.182	.890	1.490	.010
**e15**	1.531	1.280	1.886	.010
**e16**	1.292	.997	1.587	.010
**e17**	1.073	.903	1.267	.010
**e18**	.828	.598	1.060	.010
**e19**	1.095	.858	1.396	.010
**e20**	1.396	1.028	1.735	.010
**e21**	1.048	.809	1.310	.010
**e22**	1.209	.898	1.495	.010
**e23**	1.589	1.285	1.942	.010
**e24**	1.843	1.488	2.227	.010

Matrices (g2 - Measurement intercepts)

Sample Covariances (g2 - Measurement intercepts)

Sample Covariances - Lower Bounds (PC) (g2 - Measurement intercepts)

	BPNSF6	BPNSF11	BPNSF17	BPNSF23	BPNSF3	BPNSF9	BPNSF14	BPNSF21	BPNSF2	BPNSF8	BPNSF20	BPNSF22	BPNSF4	BPNSF12	BPNSF16	BPNSI
**BPNSF6**	2.618															
**BPNSF11**	.891	2.597														
**BPNSF17**	1.099	1.287	2.541													
**BPNSF23**	1.081	1.203	1.450	2.385												
**BPNSF3**	-1.078	-.866	-1.056	-1.127	1.965											
**BPNSF9**	-1.015	-1.177	-1.431	-1.265	.913	1.897										
**BPNSF14**	-.874	-1.012	-1.348	-1.044	.754	1.010	1.748									
**BPNSF21**	-.915	-1.066	-1.031	-1.106	.857	.778	.899	1.959								
**BPNSF2**	.965	.899	1.151	1.121	-.956	-.940	-1.002	-.931	2.284							
**BPNSF8**	1.011	.971	1.151	1.055	-.895	-.996	-.946	-.985	1.223	2.758						
**BPNSF20**	.801	1.035	1.212	1.446	-1.053	-1.035	-1.137	-1.258	1.290	1.348	2.521					
**BPNSF22**	.752	.931	1.041	1.068	-.676	-1.006	-.936	-1.043	1.090	1.169	1.364	2.498				
**BPNSF4**	-.885	-.814	-.880	-.941	1.173	.673	.637	.916	-1.049	-.999	-1.237	-1.002	1.923			

	BPNSF6	BPNSF11	BPNSF17	BPNSF23	BPNSF3	BPNSF9	BPNSF14	BPNSF21	BPNSF2	BPNSF8	BPNSF20	BPNSF22	BPNSF4	BPNSF12	BPNSF16	BPNS
**BPNSF12**	-.778	-.940	-1.092	-1.000	.654	.739	.819	.888	-1.124	-1.045	-1.155	-1.267	.938	1.827		
**BPNSF16**	-.719	-.926	-.978	-.913	.538	.631	.831	.934	-1.028	-1.033	-1.288	-1.198	.810	1.198	1.895	
**BPNSF24**	-.737	-.847	-1.146	-.926	.429	.575	.735	.777	-1.115	-1.137	-1.285	-1.369	.738	.941	1.007	1.8
**BPNSF5**	.684	.456	.496	.656	-.665	-.538	-.550	-.775	.645	.884	.725	.739	-.987	-.715	-.620	-.7
**BPNSF10**	.650	1.183	.956	1.030	-.873	-.978	-.940	-1.126	.892	1.084	1.097	1.050	-.987	-.940	-.880	-.9
**BPNSF15**	.651	.919	.724	.599	-.635	-.542	-.504	-.671	.609	.824	.881	.943	-.793	-.735	-.697	-.7
**BPNSF18**	-.024	.403	.363	.122	-.204	-.336	-.254	-.435	.137	.257	.410	.275	-.392	-.396	-.398	-.4
**BPNSF1**	-.470	-.552	-.523	-.597	.643	.475	.604	.740	-.827	-.743	-.863	-.792	.644	.520	.468	.4
**BPNSF7**	-.244	-.762	-.915	-.539	.442	.741	.658	.623	-.661	-.827	-.754	-.921	.583	.530	.540	.5
**BPNSF13**	-.722	-.995	-.929	-.885	.720	.729	.930	1.031	-.832	-.991	-.971	-1.061	.744	1.025	.841	.7
**BPNSF19**	-.851	-1.074	-1.109	-1.157	.574	.754	.806	.933	-.719	-.930	-1.087	-.986	.648	.610	.556	.4

Sample Covariances - Upper Bounds (PC) (g2 - Measurement intercepts)

	BPNSF6	BPNSF11	BPNSF17	BPNSF23	BPNSF3	BPNSF9	BPNSF14	BPNSF21	BPNSF2	BPNSF8	BPNSF20	BPNSF22	BPNSF4	BPNSF12	BPNSF16	BPNS
**BPNSF6**	3.251															
**BPNSF11**	1.425	3.169														
**BPNSF17**	1.595	1.810	3.245													
**BPNSF23**	1.584	1.676	2.000	3.053												
**BPNSF3**	-.650	-.448	-.648	-.676	2.566											
**BPNSF9**	-.586	-.744	-.942	-.817	1.345	2.548										
**BPNSF14**	-.493	-.572	-.893	-.643	1.201	1.519	2.302									
**BPNSF21**	-.503	-.620	-.574	-.653	1.279	1.249	1.355	2.472								
**BPNSF2**	1.441	1.349	1.577	1.621	-.598	-.572	-.618	-.594	2.898							
**BPNSF8**	1.553	1.500	1.644	1.551	-.483	-.577	-.500	-.546	1.696	3.333						
**BPNSF20**	1.316	1.532	1.729	1.999	-.607	-.574	-.639	-.792	1.753	1.857	3.246					
**BPNSF22**	1.237	1.372	1.534	1.533	-.291	-.563	-.475	-.601	1.499	1.645	1.824	2.983				
**BPNSF4**	-.437	-.371	-.489	-.480	1.640	1.085	1.067	1.304	-.653	-.554	-.730	-.547	2.385			
**BPNSF12**	-.340	-.507	-.655	-.582	1.082	1.200	1.305	1.332	-.734	-.612	-.695	-.844	1.341	2.296		
**BPNSF16**	-.306	-.496	-.583	-.467	.968	1.086	1.222	1.390	-.639	-.581	-.747	-.758	1.232	1.657	2.452	
**BPNSF24**	-.327	-.452	-.719	-.506	.844	.994	1.163	1.190	-.688	-.684	-.821	-.928	1.159	1.393	1.469	2.4
**BPNSF5**	1.136	.911	.966	1.162	-.245	-.151	-.178	-.314	1.011	1.377	1.261	1.229	-.509	-.283	-.202	-.3
**BPNSF10**	1.137	1.621	1.428	1.500	-.390	-.556	-.553	-.667	1.337	1.589	1.645	1.547	-.490	-.512	-.434	-.5
**BPNSF15**	1.106	1.386	1.251	1.116	-.142	-.128	-.100	-.216	1.052	1.355	1.367	1.438	-.316	-.252	-.244	-.3
**BPNSF18**	.438	.903	.819	.590	.215	.062	.090	-.035	.554	.739	.862	.751	.053	.064	.006	-.0
**BPNSF1**	-.131	-.178	-.188	-.209	1.001	.837	.963	1.074	-.462	-.269	-.387	-.324	1.052	.889	.831	.8
**BPNSF7**	.184	-.300	-.440	-.154	.911	1.185	1.051	1.006	-.318	-.376	-.329	-.487	.966	.980	.950	.9
**BPNSF13**	-.356	-.563	-.515	-.489	1.090	1.143	1.358	1.452	-.481	-.584	-.595	-.644	1.103	1.400	1.247	1.1
**BPNSF19**	-.412	-.587	-.661	-.677	.975	1.255	1.271	1.407	-.380	-.494	-.630	-.586	1.049	1.075	.966	.8

Sample Covariances - Two Tailed Significance (PC) (g2 - Measurement intercepts)

	BPNSF6	BPNSF11	BPNSF17	BPNSF23	BPNSF3	BPNSF9	BPNSF14	BPNSF21	BPNSF2	BPNSF8	BPNSF20	BPNSF22	BPNSF4	BPNSF12	BPNSF16	BPNS
**BPNSF6**	.010															
**BPNSF11**	.010	.010														
**BPNSF17**	.010	.010	.010													
**BPNSF23**	.010	.010	.010	.010												
**BPNSF3**	.010	.010	.010	.010	.010											
**BPNSF9**	.010	.010	.010	.010	.010	.010										
**BPNSF14**	.010	.010	.010	.010	.010	.010	.010									
**BPNSF21**	.010	.010	.010	.010	.010	.010	.010	.010								
**BPNSF2**	.010	.010	.010	.010	.010	.010	.010	.010	.010							
**BPNSF8**	.010	.010	.010	.010	.010	.010	.010	.010	.010	.010						
**BPNSF20**	.010	.010	.010	.010	.010	.010	.010	.010	.010	.010	.010					
**BPNSF22**	.010	.010	.010	.010	.010	.010	.010	.010	.010	.010	.010	.010				
**BPNSF4**	.010	.010	.010	.010	.010	.010	.010	.010	.010	.010	.010	.010	.010			
**BPNSF12**	.010	.010	.010	.010	.010	.010	.010	.010	.010	.010	.010	.010	.010	.010		
**BPNSF16**	.010	.010	.010	.010	.010	.010	.010	.010	.010	.010	.010	.010	.010	.010	.010	
**BPNSF24**	.010	.010	.010	.010	.010	.010	.010	.010	.010	.010	.010	.010	.010	.010	.010	.0
**BPNSF5**	.010	.010	.010	.010	.010	.021	.010	.010	.010	.010	.010	.010	.010	.010	.013	.0
**BPNSF10**	.010	.010	.010	.010	.010	.010	.010	.010	.010	.010	.010	.010	.010	.010	.010	.0
**BPNSF15**	.010	.010	.010	.010	.016	.010	.023	.010	.010	.010	.010	.010	.010	.010	.010	.0
**BPNSF18**	.131	.010	.010	.016	.946	.259	.576	.040	.010	.010	.010	.010	.162	.274	.108	.0
**BPNSF1**	.010	.010	.010	.010	.010	.010	.010	.010	.010	.010	.010	.010	.010	.010	.010	.0
**BPNSF7**	.692	.010	.010	.014	.010	.010	.010	.010	.010	.010	.010	.010	.010	.010	.010	.0
**BPNSF13**	.010	.010	.010	.010	.010	.010	.010	.010	.010	.010	.010	.010	.010	.010	.010	.0
**BPNSF19**	.010	.010	.010	.010	.010	.010	.010	.010	.010	.010	.010	.010	.010	.010	.010	.0

Sample Correlations (g2 - Measurement intercepts)

Sample Correlations - Lower Bounds (PC) (g2 - Measurement intercepts)

	BPNSF6	BPNSF11	BPNSF17	BPNSF23	BPNSF3	BPNSF9	BPNSF14	BPNSF21	BPNSF2	BPNSF8	BPNSF20	BPNSF22	BPNSF4	BPNSF12	BPNSF16	BPNS
**BPNSF6**	1.000															
**BPNSF11**	.309	1.000														
**BPNSF17**	.379	.452	1.000													
**BPNSF23**	.390	.438	.543	1.000												
**BPNSF3**	-.420	-.355	-.418	-.454	1.000											
**BPNSF9**	-.397	-.453	-.547	-.507	.412	1.000										
**BPNSF14**	-.359	-.418	-.538	-.449	.363	.496	1.000									
**BPNSF21**	-.355	-.407	-.399	-.436	.387	.361	.447	1.000								
**BPNSF2**	.355	.334	.414	.432	-.402	-.399	-.437	-.386	1.000							
**BPNSF8**	.346	.325	.405	.376	-.355	-.389	-.368	-.371	.454	1.000						
**BPNSF20**	.278	.367	.425	.539	-.408	-.406	-.452	-.473	.474	.466	1.000					
**BPNSF22**	.262	.323	.377	.399	-.271	-.388	-.386	-.417	.421	.418	.501	1.000				
**BPNSF4**	-.344	-.336	-.347	-.374	.566	.323	.319	.437	-.430	-.379	-.467	-.398	1.000			
**BPNSF12**	-.325	-.383	-.434	-.413	.306	.359	.435	.438	-.472	-.416	-.459	-.511	.458	1.000		
**BPNSF16**	-.285	-.360	-.390	-.377	.247	.301	.416	.457	-.421	-.397	-.492	-.474	.385	.620	1.000	
**BPNSF24**	-.296	-.348	-.443	-.382	.191	.265	.389	.359	-.453	-.430	-.507	-.538	.354	.482	.508	1.0
**BPNSF5**	.266	.169	.187	.266	-.281	-.222	-.243	-.324	.266	.335	.288	.291	-.416	-.320	-.261	-.3
**BPNSF10**	.227	.415	.351	.380	-.341	-.386	-.389	-.426	.333	.382	.397	.378	-.380	-.385	-.348	-.3
**BPNSF15**	.218	.311	.250	.211	-.241	-.205	-.206	-.252	.220	.278	.295	.329	-.304	-.296	-.264	-.3
**BPNSF18**	-.009	.160	.135	.050	-.085	-.145	-.119	-.191	.057	.093	.161	.109	-.173	-.182	-.174	-.2
**BPNSF1**	-.193	-.234	-.220	-.255	.307	.226	.310	.351	-.361	-.300	-.356	-.335	.313	.261	.231	.2
**BPNSF7**	-.099	-.309	-.363	-.228	.207	.363	.329	.296	-.289	-.333	-.297	-.376	.277	.263	.257	.2
**BPNSF13**	-.314	-.421	-.391	-.386	.352	.362	.500	.528	-.368	-.398	-.411	-.444	.379	.542	.432	.4
**BPNSF19**	-.323	-.417	-.431	-.444	.252	.341	.387	.434	-.296	-.339	-.414	-.385	.294	.294	.258	.1

Sample Correlations - Upper Bounds (PC) (g2 - Measurement intercepts)

	BPNSF6	BPNSF11	BPNSF17	BPNSF23	BPNSF3	BPNSF9	BPNSF14	BPNSF21	BPNSF2	BPNSF8	BPNSF20	BPNSF22	BPNSF4	BPNSF12	BPNSF16	BPNS
**BPNSF6**	1.000															
**BPNSF11**	.480	1.000														
**BPNSF17**	.548	.610	1.000													
**BPNSF23**	.540	.589	.697	1.000												
**BPNSF3**	-.248	-.178	-.248	-.272	1.000											
**BPNSF9**	-.230	-.291	-.372	-.348	.605	1.000										
**BPNSF14**	-.205	-.239	-.388	-.283	.548	.674	1.000									
**BPNSF21**	-.196	-.252	-.221	-.267	.543	.532	.618	1.000								
**BPNSF2**	.510	.489	.572	.589	-.245	-.238	-.271	-.242	1.000							
**BPNSF8**	.509	.499	.546	.525	-.189	-.234	-.202	-.208	.595	1.000						
**BPNSF20**	.447	.525	.586	.681	-.241	-.237	-.281	-.317	.629	.605	1.000					
**BPNSF22**	.432	.482	.529	.547	-.113	-.227	-.210	-.240	.559	.554	.625	1.000				
**BPNSF4**	-.170	-.153	-.198	-.205	.693	.481	.473	.558	-.285	-.224	-.300	-.228	1.000			
**BPNSF12**	-.143	-.213	-.276	-.254	.501	.546	.590	.578	-.314	-.251	-.287	-.364	.620	1.000		
**BPNSF16**	-.118	-.204	-.239	-.188	.441	.490	.569	.606	-.273	-.227	-.308	-.322	.564	.732	1.000	
**BPNSF24**	-.124	-.178	-.281	-.214	.376	.437	.541	.513	-.298	-.271	-.347	-.397	.521	.633	.646	1.0
**BPNSF5**	.423	.335	.357	.431	-.112	-.065	-.084	-.134	.399	.497	.469	.463	-.235	-.128	-.090	-.1
**BPNSF10**	.383	.556	.481	.515	-.151	-.224	-.233	-.277	.481	.527	.552	.537	-.200	-.214	-.178	-.2
**BPNSF15**	.361	.467	.421	.379	-.054	-.047	-.038	-.085	.376	.439	.454	.484	-.130	-.100	-.099	-.1
**BPNSF18**	.163	.336	.313	.230	.091	.028	.043	-.015	.220	.272	.324	.289	.025	.029	.003	-.0
**BPNSF1**	-.052	-.074	-.077	-.090	.464	.409	.463	.495	-.205	-.111	-.164	-.146	.495	.441	.387	.4
**BPNSF7**	.071	-.125	-.195	-.066	.413	.537	.499	.467	-.135	-.151	-.134	-.208	.443	.455	.438	.4
**BPNSF13**	-.149	-.243	-.220	-.219	.525	.537	.652	.657	-.216	-.246	-.257	-.288	.525	.661	.585	.5
**BPNSF19**	-.156	-.226	-.260	-.269	.421	.532	.572	.582	-.158	-.185	-.248	-.227	.451	.466	.420	.3

Sample Correlations - Two Tailed Significance (PC) (g2 - Measurement intercepts)

	BPNSF6	BPNSF11	BPNSF17	BPNSF23	BPNSF3	BPNSF9	BPNSF14	BPNSF21	BPNSF2	BPNSF8	BPNSF20	BPNSF22	BPNSF4	BPNSF12	BPNSF16	BPNS
**BPNSF6**	...															
**BPNSF11**	.010	...														
**BPNSF17**	.010	.010	...													
**BPNSF23**	.010	.010	.010	...												
**BPNSF3**	.010	.010	.010	.010	...											
**BPNSF9**	.010	.010	.010	.010	.010	...										
**BPNSF14**	.010	.010	.010	.010	.010	.010	...									
**BPNSF21**	.010	.010	.010	.010	.010	.010	.010	...								
**BPNSF2**	.010	.010	.010	.010	.010	.010	.010	.010	...							
**BPNSF8**	.010	.010	.010	.010	.010	.010	.010	.010	.010	...						
**BPNSF20**	.010	.010	.010	.010	.010	.010	.010	.010	.010	.010	...					
**BPNSF22**	.010	.010	.010	.010	.010	.010	.010	.010	.010	.010	.010	...				
**BPNSF4**	.010	.010	.010	.010	.010	.010	.010	.010	.010	.010	.010	.010	...			

	BPNSF6	BPNSF11	BPNSF17	BPNSF23	BPNSF3	BPNSF9	BPNSF14	BPNSF21	BPNSF2	BPNSF8	BPNSF20	BPNSF22	BPNSF4	BPNSF12	BPNSF16	BPNS
**BPNSF12**	.010	.010	.010	.010	.010	.010	.010	.010	.010	.010	.010	.010	.010	...		
**BPNSF16**	.010	.010	.010	.010	.010	.010	.010	.010	.010	.010	.010	.010	.010	.010	...	
**BPNSF24**	.010	.010	.010	.010	.010	.010	.010	.010	.010	.010	.010	.010	.010	.010	.010	
**BPNSF5**	.010	.010	.010	.010	.010	.021	.010	.010	.010	.010	.010	.010	.010	.010	.013	.0
**BPNSF10**	.010	.010	.010	.010	.010	.010	.010	.010	.010	.010	.010	.010	.010	.010	.010	.0
**BPNSF15**	.010	.010	.010	.010	.015	.010	.023	.010	.010	.010	.010	.010	.010	.010	.010	.0
**BPNSF18**	.131	.010	.010	.016	.946	.259	.576	.040	.010	.010	.010	.010	.162	.274	.108	.0
**BPNSF1**	.010	.010	.010	.010	.010	.010	.010	.010	.010	.010	.010	.010	.010	.010	.010	.0
**BPNSF7**	.692	.010	.010	.014	.010	.010	.010	.010	.010	.010	.010	.010	.010	.010	.010	.0
**BPNSF13**	.010	.010	.010	.010	.010	.010	.010	.010	.010	.010	.010	.010	.010	.010	.010	.0
**BPNSF19**	.010	.010	.010	.010	.010	.010	.010	.010	.010	.010	.010	.010	.010	.010	.010	.0

Sample Means (g2 - Measurement intercepts)

Sample Means - Lower Bounds (PC) (g2 - Measurement intercepts)

	BPNSF6	BPNSF11	BPNSF17	BPNSF23	BPNSF3	BPNSF9	BPNSF14	BPNSF21	BPNSF2	BPNSF8	BPNSF20	BPNSF22	BPNSF4	BPNSF12	BPNSF16	BPNSF
**BPNSF6**	2.464	2.604	2.325	2.042	5.508	5.608	5.518	5.118	2.160	2.584	2.279	2.913	4.965	5.201	5.104	5.19

Sample Means - Upper Bounds (PC) (g2 - Measurement intercepts)

	BPNSF6	BPNSF11	BPNSF17	BPNSF23	BPNSF3	BPNSF9	BPNSF14	BPNSF21	BPNSF2	BPNSF8	BPNSF20	BPNSF22	BPNSF4	BPNSF12	BPNSF16	BPNSF
**BPNSF6**	2.725	2.904	2.613	2.284	5.742	5.857	5.728	5.391	2.414	2.889	2.604	3.216	5.211	5.424	5.337	5.43

Sample Means - Two Tailed Significance (PC) (g2 - Measurement intercepts)

	BPNSF6	BPNSF11	BPNSF17	BPNSF23	BPNSF3	BPNSF9	BPNSF14	BPNSF21	BPNSF2	BPNSF8	BPNSF20	BPNSF22	BPNSF4	BPNSF12	BPNSF16	BPNSF
**BPNSF6**	.010	.010	.010	.010	.010	.010	.010	.010	.010	.010	.010	.010	.010	.010	.010	.01

Bias-corrected percentile method (g2 - Measurement intercepts)

90% confidence intervals (bias-corrected percentile method)

Scalar Estimates (g2 - Measurement intercepts)

Regression Weights: (g2 - Measurement intercepts)

Parameter			Estimate	Lower	Upper	P
BPNSF19	<---	F1	1.000	1.000	1.000	...
BPNSF13	<---	F1	1.144	1.053	1.252	.004
BPNSF7	<---	F1	.863	.764	.963	.010
BPNSF1	<---	F1	.759	.643	.876	.012
BPNSF18	<---	F2	1.000	1.000	1.000	...
BPNSF15	<---	F2	2.454	2.008	3.361	.006
BPNSF10	<---	F2	3.105	2.512	4.256	.008
BPNSF5	<---	F2	2.516	2.030	3.412	.008
BPNSF24	<---	F3	1.000	1.000	1.000	...
BPNSF16	<---	F3	1.124	1.040	1.219	.009
BPNSF12	<---	F3	1.221	1.151	1.306	.004
BPNSF4	<---	F3	.846	.746	.934	.012
BPNSF22	<---	F4	1.000	1.000	1.000	...
BPNSF20	<---	F4	1.192	1.118	1.295	.005
BPNSF8	<---	F4	1.197	1.120	1.292	.005
BPNSF2	<---	F4	1.120	1.043	1.226	.005
BPNSF21	<---	F5	1.000	1.000	1.000	...
BPNSF14	<---	F5	1.094	1.023	1.179	.009
BPNSF9	<---	F5	1.038	.967	1.135	.007
BPNSF3	<---	F5	.882	.787	.982	.015
BPNSF23	<---	F6	1.000	1.000	1.000	...
BPNSF17	<---	F6	.976	.914	1.026	.021
BPNSF11	<---	F6	.959	.910	1.027	.005
BPNSF6	<---	F6	.861	.801	.930	.010

Standardized Regression Weights: (g2 - Measurement intercepts)

Parameter			Estimate	Lower	Upper	P
BPNSF19	<---	F1	.600	.540	.666	.010
BPNSF13	<---	F1	.761	.708	.804	.012

Parameter			Estimate	Lower	Upper	P
BPNSF7	<---	F1	.558	.497	.645	.006
BPNSF1	<---	F1	.508	.437	.561	.021
BPNSF18	<---	F2	.268	.196	.341	.012
BPNSF15	<---	F2	.584	.528	.648	.007
BPNSF10	<---	F2	.733	.663	.788	.018
BPNSF5	<---	F2	.644	.575	.704	.014
BPNSF24	<---	F3	.698	.630	.737	.026
BPNSF16	<---	F3	.780	.728	.817	.019
BPNSF12	<---	F3	.842	.801	.868	.020
BPNSF4	<---	F3	.610	.556	.690	.006
BPNSF22	<---	F4	.681	.637	.731	.007
BPNSF20	<---	F4	.765	.712	.813	.020
BPNSF8	<---	F4	.724	.674	.765	.008
BPNSF2	<---	F4	.730	.680	.784	.012
BPNSF21	<---	F5	.706	.662	.762	.006
BPNSF14	<---	F5	.779	.705	.829	.019
BPNSF9	<---	F5	.716	.639	.767	.025
BPNSF3	<---	F5	.611	.531	.670	.021
BPNSF23	<---	F6	.780	.734	.830	.009
BPNSF17	<---	F6	.749	.686	.796	.021
BPNSF11	<---	F6	.696	.651	.754	.007
BPNSF6	<---	F6	.629	.581	.681	.007

Intercepts: (g2 - Measurement intercepts)

Parameter	Estimate	Lower	Upper	P
**BPNSF19**	5.140	5.046	5.203	.028
**BPNSF13**	5.043	4.967	5.102	.034
**BPNSF7**	4.817	4.726	4.873	.044
**BPNSF1**	4.705	4.630	4.766	.030
**BPNSF18**	4.316	4.252	4.390	.009
**BPNSF15**	3.699	3.606	3.770	.012
**BPNSF10**	3.158	3.068	3.229	.009
**BPNSF5**	3.721	3.650	3.798	.006
**BPNSF24**	5.225	5.135	5.281	.034
**BPNSF16**	5.118	5.049	5.195	.019
**BPNSF12**	5.220	5.151	5.275	.034
**BPNSF4**	5.116	5.041	5.175	.026
**BPNSF22**	3.135	3.062	3.213	.004
**BPNSF20**	2.524	2.445	2.626	.003
**BPNSF8**	2.823	2.738	2.909	.005
**BPNSF2**	2.400	2.330	2.498	.003
**BPNSF21**	5.178	5.054	5.218	.095
**BPNSF14**	5.441	5.353	5.498	.053
**BPNSF9**	5.657	5.564	5.726	.032
**BPNSF3**	5.591	5.530	5.666	.018
**BPNSF23**	2.250	2.189	2.341	.002
**BPNSF17**	2.586	2.504	2.680	.003
**BPNSF11**	2.754	2.681	2.846	.004
**BPNSF6**	2.574	2.487	2.664	.007

Covariances: (g2 - Measurement intercepts)

Parameter			Estimate	Lower	Upper	P
F1	<-->	F2	-.215	-.300	-.151	.011
F2	<-->	F3	-.195	-.278	-.124	.018
F1	<-->	F3	.823	.691	.957	.013
F2	<-->	F4	.366	.249	.477	.014
F3	<-->	F4	-.743	-.895	-.610	.010
F1	<-->	F4	-.616	-.746	-.506	.011
F2	<-->	F5	-.194	-.279	-.130	.012
F4	<-->	F5	-.685	-.814	-.586	.007
F3	<-->	F5	.872	.728	1.049	.008
F1	<-->	F5	.955	.830	1.179	.005
F6	<-->	F5	-.912	-1.112	-.784	.006
F6	<-->	F3	-.685	-.807	-.503	.025
F6	<-->	F4	1.228	1.083	1.403	.006
F6	<-->	F2	.389	.284	.527	.005
F6	<-->	F1	-.645	-.824	-.520	.008

Correlations: (g2 - Measurement intercepts)

Parameter			Estimate	Lower	Upper	P
F1	<-->	F2	-.569	-.638	-.461	.030
F2	<-->	F3	-.471	-.565	-.350	.021
F1	<-->	F3	.868	.790	.923	.012
F2	<-->	F4	.832	.781	.881	.007
F3	<-->	F4	-.673	-.736	-.582	.025
F1	<-->	F4	-.610	-.696	-.522	.011
F2	<-->	F5	-.464	-.550	-.341	.023
F4	<-->	F5	-.611	-.684	-.524	.013
F3	<-->	F5	.829	.764	.882	.010
F1	<-->	F5	.993	.913	1.034	.020
F6	<-->	F5	-.692	-.765	-.615	.011
F6	<-->	F3	-.528	-.600	-.400	.028
F6	<-->	F4	.888	.835	.959	.004
F6	<-->	F2	.752	.682	.835	.004
F6	<-->	F1	-.543	-.638	-.437	.020

Variances: (g2 - Measurement intercepts)

Parameter	Estimate	Lower	Upper	P
**F1**	.867	.713	1.047	.012
**F2**	.164	.082	.262	.016
**F3**	1.036	.851	1.239	.012
**F4**	1.175	1.024	1.379	.007
**F5**	1.069	.922	1.324	.005
**F6**	1.626	1.377	1.896	.010
**e1**	1.539	1.228	1.887	.009
**e2**	.823	.695	1.009	.004
**e3**	1.423	1.123	1.692	.011
**e4**	1.439	1.277	1.658	.004
**e5**	2.130	1.869	2.364	.019
**e6**	1.916	1.623	2.190	.012
**e7**	1.365	1.122	1.724	.004
**e8**	1.469	1.269	1.803	.005
**e9**	1.093	.871	1.322	.008
**e10**	.843	.677	1.009	.007
**e11**	.633	.530	.755	.005
**e12**	1.249	1.008	1.477	.014
**e13**	1.360	1.134	1.536	.019
**e14**	1.182	.907	1.511	.005
**e15**	1.531	1.297	1.914	.006
**e16**	1.292	1.067	1.676	.004
**e17**	1.073	.924	1.287	.005
**e18**	.828	.631	1.066	.006
**e19**	1.095	.866	1.405	.007
**e20**	1.396	1.122	1.750	.004
**e21**	1.048	.836	1.340	.005
**e22**	1.209	.927	1.566	.006
**e23**	1.589	1.285	1.942	.010
**e24**	1.843	1.488	2.227	.010

Matrices (g2 - Measurement intercepts)

Sample Covariances (g2 - Measurement intercepts)

Sample Covariances - Lower Bounds (BC) (g2 - Measurement intercepts)

	BPNSF6	BPNSF11	BPNSF17	BPNSF23	BPNSF3	BPNSF9	BPNSF14	BPNSF21	BPNSF2	BPNSF8	BPNSF20	BPNSF22	BPNSF4	BPNSF12	BPNSF16	BPNS[illegible]
**BPNSF6**	2.589															
**BPNSF11**	.958	2.587														
**BPNSF17**	1.116	1.287	2.611													
**BPNSF23**	1.112	1.226	1.490	2.372												
**BPNSF3**	-1.069	-.875	-1.049	-1.127	1.965											
**BPNSF9**	-1.025	-1.195	-1.458	-1.308	.907	1.905										
**BPNSF14**	-.865	-1.036	-1.409	-1.084	.760	1.027	1.795									
**BPNSF21**	-.909	-1.102	-1.058	-1.099	.814	.807	.909	1.924								
**BPNSF2**	.974	.895	1.184	1.129	-.959	-.921	-1.067	-.935	2.371							
**BPNSF8**	1.012	.971	1.158	1.115	-.895	-.996	-.951	-.985	1.224	2.806						
**BPNSF20**	.812	1.076	1.258	1.500	-1.042	-1.030	-1.200	-1.266	1.312	1.403	2.598					
**BPNSF22**	.770	.968	1.127	1.068	-.675	-1.012	-.944	-1.043	1.093	1.181	1.364	2.481				
**BPNSF4**	-.851	-.827	-.881	-.941	1.168	.686	.644	.923	-1.038	-.970	-1.215	-.961	1.913			

	BPNSF6	BPNSF11	BPNSF17	BPNSF23	BPNSF3	BPNSF9	BPNSF14	BPNSF21	BPNSF2	BPNSF8	BPNSF20	BPNSF22	BPNSF4	BPNSF12	BPNSF16	BPNS[illegible]
**BPNSF12**	-.763	-.915	-1.092	-.995	.654	.755	.873	.916	-1.093	-1.056	-1.155	-1.220	.941	1.846		
**BPNSF16**	-.698	-.913	-.976	-.913	.550	.635	.834	.932	-1.028	-1.045	-1.253	-1.167	.820	1.198	1.863	
**BPNSF24**	-.701	-.847	-1.171	-.951	.434	.611	.791	.777	-1.149	-1.152	-1.285	-1.375	.733	.990	.999	[illegible]
**BPNSF5**	.681	.463	.496	.640	-.646	-.500	-.543	-.727	.632	.901	.720	.755	-.956	-.715	-.620	[illegible]
**BPNSF10**	.666	1.211	.958	1.080	-.886	-.985	-.964	-1.126	.917	1.120	1.103	1.072	-.955	-.956	-.872	[illegible]
**BPNSF15**	.657	.935	.758	.671	-.586	-.539	-.497	-.671	.632	.748	.886	.964	-.730	-.705	-.686	[illegible]
**BPNSF18**	-.024	.386	.320	.121	-.192	-.330	-.268	-.469	.132	.257	.408	.277	-.392	-.396	-.424	[illegible]
**BPNSF1**	-.422	-.549	-.520	-.597	.649	.506	.620	.741	-.826	-.743	-.840	-.782	.617	.538	.453	[illegible]
**BPNSF7**	-.218	-.773	-.961	-.545	.436	.743	.668	.630	-.652	-.814	-.752	-.936	.620	.569	.578	[illegible]
**BPNSF13**	-.711	-.995	-.940	-.866	.720	.753	.943	1.047	-.845	-.970	-.970	-1.056	.755	1.036	.832	[illegible]
**BPNSF19**	-.854	-1.087	-1.157	-1.157	.560	.742	.846	.942	-.716	-.930	-1.093	-.984	.649	.634	.555	[illegible]

Sample Covariances - Upper Bounds (BC) (g2 - Measurement intercepts)

	BPNSF6	BPNSF11	BPNSF17	BPNSF23	BPNSF3	BPNSF9	BPNSF14	BPNSF21	BPNSF2	BPNSF8	BPNSF20	BPNSF22	BPNSF4	BPNSF12	BPNSF16	BPNS[illegible]
**BPNSF6**	3.240															
**BPNSF11**	1.497	3.167														
**BPNSF17**	1.619	1.810	3.314													
**BPNSF23**	1.631	1.705	2.028	3.050												
**BPNSF3**	-.633	-.465	-.642	-.676	2.566											
**BPNSF9**	-.593	-.749	-.957	-.841	1.344	2.552										
**BPNSF14**	-.486	-.584	-.948	-.690	1.202	1.569	2.368									
**BPNSF21**	-.489	-.648	-.599	-.641	1.239	1.273	1.415	2.454								
**BPNSF2**	1.445	1.343	1.624	1.622	-.604	-.569	-.655	-.596	2.916							
**BPNSF8**	1.562	1.500	1.667	1.597	-.483	-.577	-.503	-.546	1.696	3.371						
**BPNSF20**	1.332	1.554	1.764	2.029	-.579	-.573	-.689	-.797	1.790	1.919	3.293					
**BPNSF22**	1.254	1.406	1.593	1.533	-.286	-.574	-.484	-.599	1.529	1.670	1.824	2.973				
**BPNSF4**	-.391	-.408	-.490	-.480	1.634	1.102	1.096	1.339	-.653	-.547	-.706	-.518	2.384			
**BPNSF12**	-.323	-.505	-.655	-.571	1.082	1.249	1.366	1.363	-.718	-.614	-.695	-.822	1.350	2.354		
**BPNSF16**	-.264	-.489	-.568	-.467	.987	1.094	1.222	1.389	-.639	-.592	-.732	-.737	1.234	1.657	2.418	
**BPNSF24**	-.304	-.450	-.727	-.536	.848	1.042	1.194	1.190	-.708	-.690	-.818	-.936	1.132	1.430	1.460	[illegible]
**BPNSF5**	1.132	.922	.977	1.134	-.213	-.114	-.167	-.292	.998	1.382	1.251	1.246	-.489	-.282	-.202	[illegible]
**BPNSF10**	1.144	1.680	1.429	1.554	-.396	-.564	-.569	-.667	1.355	1.607	1.646	1.596	-.469	-.521	-.397	[illegible]
**BPNSF15**	1.116	1.398	1.272	1.161	-.120	-.127	-.083	-.216	1.071	1.329	1.380	1.449	-.272	-.244	-.238	[illegible]
**BPNSF18**	.438	.893	.784	.588	.225	.072	.062	-.048	.552	.739	.858	.751	.053	.064	-.024	[illegible]
**BPNSF1**	-.043	-.170	-.186	-.209	1.008	.881	1.001	1.075	-.460	-.269	-.365	-.306	1.048	.922	.784	[illegible]
**BPNSF7**	.224	-.304	-.483	-.168	.876	1.186	1.060	1.016	-.309	-.355	-.327	-.517	1.004	.996	1.009	[illegible]
**BPNSF13**	-.334	-.563	-.521	-.466	1.090	1.175	1.373	1.460	-.484	-.577	-.589	-.643	1.119	1.446	1.230	[illegible]
**BPNSF19**	-.428	-.624	-.713	-.677	.961	1.251	1.285	1.420	-.374	-.494	-.631	-.585	1.057	1.084	.951	[illegible]

Sample Covariances - Two Tailed Significance (BC) (g2 - Measurement intercepts)

	BPNSF6	BPNSF11	BPNSF17	BPNSF23	BPNSF3	BPNSF9	BPNSF14	BPNSF21	BPNSF2	BPNSF8	BPNSF20	BPNSF22	BPNSF4	BPNSF12	BPNSF16	BPNS[illegible]
**BPNSF6**	.012															
**BPNSF11**	.003	.011														
**BPNSF17**	.006	.010	.004													
**BPNSF23**	.003	.006	.005	.011												
**BPNSF3**	.012	.007	.011	.010	.010											
**BPNSF9**	.008	.008	.005	.005	.011	.008										
**BPNSF14**	.012	.007	.002	.004	.009	.005	.003									
**BPNSF21**	.013	.006	.005	.013	.019	.006	.007	.021								
**BPNSF2**	.007	.012	.005	.009	.009	.012	.003	.009	.003							
**BPNSF8**	.009	.010	.007	.002	.010	.010	.008	.010	.009	.005						
**BPNSF20**	.008	.007	.004	.004	.012	.012	.003	.007	.004	.003	.006					
**BPNSF22**	.006	.005	.003	.010	.011	.009	.008	.012	.008	.006	.010	.012				
**BPNSF4**	.020	.006	.009	.010	.012	.007	.007	.006	.011	.015	.012	.021	.011			
**BPNSF12**	.018	.013	.010	.012	.010	.005	.005	.004	.019	.009	.010	.018	.008	.004		
**BPNSF16**	.016	.013	.013	.010	.007	.009	.009	.011	.009	.007	.014	.020	.008	.010	.020	
**BPNSF24**	.019	.011	.006	.004	.009	.003	.004	.010	.004	.009	.012	.009	.012	.004	.013	[illegible]
**BPNSF5**	.012	.006	.009	.014	.025	.044	.012	.020	.013	.007	.012	.006	.019	.011	.013	[illegible]
**BPNSF10**	.007	.004	.009	.003	.009	.008	.005	.010	.006	.005	.009	.005	.013	.006	.013	[illegible]
**BPNSF15**	.007	.006	.007	.003	.027	.011	.034	.010	.006	.021	.007	.006	.025	.012	.013	[illegible]
**BPNSF18**	.131	.013	.018	.017	.926	.292	.403	.027	.011	.010	.012	.009	.162	.274	.063	[illegible]
**BPNSF1**	.023	.012	.015	.010	.007	.005	.005	.009	.011	.010	.016	.013	.012	.005	.025	[illegible]
**BPNSF7**	.965	.009	.005	.010	.015	.009	.006	.008	.015	.013	.011	.004	.004	.006	.005	[illegible]
**BPNSF13**	.014	.010	.008	.018	.010	.006	.009	.007	.007	.012	.011	.011	.006	.004	.013	[illegible]
**BPNSF19**	.007	.006	.004	.010	.014	.012	.005	.007	.012	.010	.009	.011	.009	.006	.012	[illegible]

Sample Correlations (g2 - Measurement intercepts)

Sample Correlations - Lower Bounds (BC) (g2 - Measurement intercepts)

	BPNSF6	BPNSF11	BPNSF17	BPNSF23	BPNSF3	BPNSF9	BPNSF14	BPNSF21	BPNSF2	BPNSF8	BPNSF20	BPNSF22	BPNSF4	BPNSF12	BPNSF16	BPNS
**BPNSF6**	1.000															
**BPNSF11**	.340	1.000														
**BPNSF17**	.373	.447	1.000													
**BPNSF23**	.407	.440	.556	1.000												
**BPNSF3**	-.409	-.357	-.413	-.439	1.000											
**BPNSF9**	-.396	-.444	-.545	-.507	.416	1.000										
**BPNSF14**	-.347	-.418	-.539	-.444	.356	.496	1.000									
**BPNSF21**	-.353	-.418	-.401	-.430	.361	.375	.446	1.000								
**BPNSF2**	.363	.337	.414	.424	-.397	-.374	-.426	-.386	1.000							
**BPNSF8**	.353	.319	.405	.391	-.347	-.378	-.370	-.374	.453	1.000						
**BPNSF20**	.277	.370	.443	.539	-.398	-.398	-.469	-.473	.470	.468	1.000					
**BPNSF22**	.261	.329	.405	.398	-.269	-.384	-.386	-.425	.421	.420	.494	1.000				
**BPNSF4**	-.332	-.339	-.351	-.374	.565	.322	.321	.435	-.426	-.374	-.450	-.390	1.000			
**BPNSF12**	-.300	-.374	-.430	-.403	.303	.360	.432	.430	-.462	-.411	-.452	-.507	.452	1.000		
**BPNSF16**	-.285	-.357	-.389	-.385	.245	.286	.424	.459	-.412	-.395	-.480	-.464	.389	.616	1.000	
**BPNSF24**	-.285	-.332	-.443	-.391	.191	.270	.395	.352	-.453	-.430	-.492	-.537	.351	.478	.469	1.0
**BPNSF5**	.262	.181	.175	.261	-.262	-.216	-.241	-.303	.263	.336	.278	.299	-.394	-.317	-.256	-.3
**BPNSF10**	.234	.415	.344	.392	-.339	-.392	-.389	-.426	.325	.388	.395	.384	-.365	-.398	-.346	-.3
**BPNSF15**	.227	.320	.255	.231	-.218	-.204	-.197	-.251	.223	.267	.303	.331	-.289	-.271	-.262	-.3
**BPNSF18**	-.009	.147	.125	.048	-.085	-.142	-.127	-.201	.067	.092	.161	.109	-.175	-.194	-.183	-.1
**BPNSF1**	-.173	-.228	-.213	-.253	.306	.226	.306	.350	-.356	-.281	-.338	-.327	.298	.258	.221	.2
**BPNSF7**	-.093	-.308	-.367	-.227	.202	.366	.344	.302	-.274	-.318	-.311	-.406	.294	.264	.262	.2
**BPNSF13**	-.298	-.414	-.383	-.367	.349	.366	.494	.528	-.363	-.395	-.406	-.444	.378	.544	.429	.4
**BPNSF19**	-.318	-.419	-.447	-.434	.250	.329	.386	.425	-.279	-.327	-.407	-.382	.294	.294	.257	.1

Sample Correlations - Upper Bounds (BC) (g2 - Measurement intercepts)

	BPNSF6	BPNSF11	BPNSF17	BPNSF23	BPNSF3	BPNSF9	BPNSF14	BPNSF21	BPNSF2	BPNSF8	BPNSF20	BPNSF22	BPNSF4	BPNSF12	BPNSF16	BPNS
**BPNSF6**	1.000															
**BPNSF11**	.500	1.000														
**BPNSF17**	.547	.603	1.000													
**BPNSF23**	.550	.593	.704	1.000												
**BPNSF3**	-.235	-.179	-.246	-.260	1.000											
**BPNSF9**	-.226	-.288	-.371	-.340	.608	1.000										
**BPNSF14**	-.191	-.249	-.390	-.278	.531	.672	1.000									
**BPNSF21**	-.196	-.255	-.230	-.261	.530	.536	.613	1.000								
**BPNSF2**	.512	.491	.572	.583	-.241	-.229	-.261	-.242	1.000							
**BPNSF8**	.510	.497	.545	.535	-.183	-.232	-.202	-.212	.594	1.000						
**BPNSF20**	.446	.529	.598	.681	-.224	-.235	-.286	-.317	.623	.608	1.000					
**BPNSF22**	.429	.487	.549	.544	-.110	-.222	-.210	-.253	.559	.564	.622	1.000				
**BPNSF4**	-.160	-.164	-.200	-.205	.692	.481	.501	.557	-.273	-.216	-.287	-.218	1.000			
**BPNSF12**	-.111	-.207	-.258	-.245	.494	.547	.588	.574	-.305	-.249	-.282	-.344	.618	1.000		
**BPNSF16**	-.118	-.202	-.228	-.202	.441	.483	.570	.610	-.266	-.224	-.295	-.312	.568	.725	1.000	
**BPNSF24**	-.113	-.170	-.280	-.219	.374	.445	.557	.512	-.298	-.271	-.335	-.397	.520	.630	.628	1.0
**BPNSF5**	.418	.353	.348	.430	-.093	-.050	-.082	-.128	.399	.500	.460	.478	-.217	-.118	-.085	-.1
**BPNSF10**	.389	.556	.480	.528	-.149	-.228	-.234	-.277	.476	.531	.549	.538	-.185	-.216	-.168	-.2
**BPNSF15**	.365	.472	.429	.386	-.043	-.047	-.031	-.084	.381	.438	.454	.490	-.112	-.087	-.093	-.1
**BPNSF18**	.163	.326	.301	.227	.091	.030	.029	-.023	.229	.264	.324	.289	.011	.028	-.008	-.0
**BPNSF1**	-.016	-.070	-.074	-.084	.462	.411	.458	.491	-.203	-.108	-.145	-.126	.482	.434	.381	.4
**BPNSF7**	.087	-.122	-.202	-.057	.403	.540	.517	.467	-.131	-.130	-.145	-.215	.467	.458	.459	.4
**BPNSF13**	-.138	-.237	-.204	-.205	.509	.544	.649	.656	-.211	-.245	-.256	-.288	.521	.661	.581	.5
**BPNSF19**	-.147	-.234	-.285	-.259	.416	.515	.572	.582	-.133	-.178	-.241	-.219	.451	.466	.418	.3

Sample Correlations - Two Tailed Significance (BC) (g2 - Measurement intercepts)

	BPNSF6	BPNSF11	BPNSF17	BPNSF23	BPNSF3	BPNSF9	BPNSF14	BPNSF21	BPNSF2	BPNSF8	BPNSF20	BPNSF22	BPNSF4	BPNSF12	BPNSF16	BPNS
**BPNSF6**	...															
**BPNSF11**	.004	...														
**BPNSF17**	.011	.016	...													
**BPNSF23**	.004	.008	.006	...												
**BPNSF3**	.019	.009	.012	.014	...											
**BPNSF9**	.012	.019	.013	.011	.008	...										
**BPNSF14**	.016	.009	.007	.013	.019	.011	...									
**BPNSF21**	.012	.007	.008	.015	.026	.007	.011	...								
**BPNSF2**	.006	.008	.010	.014	.012	.019	.019	.012	...							
**BPNSF8**	.006	.014	.012	.004	.015	.013	.009	.008	.011	...						
**BPNSF20**	.011	.008	.006	.010	.018	.015	.006	.010	.013	.006	...					
**BPNSF22**	.011	.007	.002	.012	.012	.014	.009	.008	.011	.006	.018	...				
**BPNSF4**	.020	.006	.007	.010	.011	.011	.007	.012	.015	.013	.021	.019	...			

	BPNSF6	BPNSF11	BPNSF17	BPNSF23	BPNSF3	BPNSF9	BPNSF14	BPNSF21	BPNSF2	BPNSF8	BPNSF20	BPNSF22	BPNSF4	BPNSF12	BPNSF16	BPNS[illegible]
**BPNSF12**	.032	.015	.020	.019	.014	.009	.012	.012	.025	.012	.021	.016	.013	...		
**BPNSF16**	.011	.012	.014	.007	.012	.013	.007	.008	.016	.011	.014	.018	.007	.014	...	
**BPNSF24**	.021	.020	.011	.006	.011	.008	.004	.015	.010	.010	.023	.011	.012	.012	.034	
**BPNSF5**	.012	.004	.013	.011	.036	.041	.012	.026	.012	.009	.015	.006	.026	.013	.018	.0[illegible]
**BPNSF10**	.007	.010	.013	.006	.014	.008	.009	.010	.015	.006	.012	.008	.021	.007	.012	.0[illegible]
**BPNSF15**	.005	.007	.007	.004	.030	.011	.036	.012	.008	.013	.009	.008	.023	.019	.012	.0[illegible]
**BPNSF18**	.131	.016	.020	.018	.946	.292	.403	.027	.007	.013	.010	.010	.141	.252	.075	.0[illegible]
**BPNSF1**	.025	.013	.018	.013	.012	.009	.012	.014	.012	.012	.020	.016	.016	.013	.019	.0[illegible]
**BPNSF7**	.945	.011	.006	.020	.020	.009	.004	.009	.019	.018	.006	.004	.004	.009	.006	.0[illegible]
**BPNSF13**	.018	.016	.016	.023	.015	.009	.016	.012	.014	.013	.012	.010	.012	.009	.014	.0[illegible]
**BPNSF19**	.015	.007	.003	.014	.012	.021	.011	.012	.026	.021	.013	.014	.011	.009	.012	.0[illegible]

Sample Means (g2 - Measurement intercepts)

Sample Means - Lower Bounds (BC) (g2 - Measurement intercepts)

	BPNSF6	BPNSF11	BPNSF17	BPNSF23	BPNSF3	BPNSF9	BPNSF14	BPNSF21	BPNSF2	BPNSF8	BPNSF20	BPNSF22	BPNSF4	BPNSF12	BPNSF16	BPNSF[illegible]
**BPNSF6**	2.451	2.619	2.333	2.062	5.494	5.608	5.463	5.096	2.184	2.604	2.319	2.940	4.964	5.201	5.101	5.17[illegible]

Sample Means - Upper Bounds (BC) (g2 - Measurement intercepts)

	BPNSF6	BPNSF11	BPNSF17	BPNSF23	BPNSF3	BPNSF9	BPNSF14	BPNSF21	BPNSF2	BPNSF8	BPNSF20	BPNSF22	BPNSF4	BPNSF12	BPNSF16	BPNSF[illegible]
**BPNSF6**	2.720	2.929	2.622	2.341	5.737	5.857	5.693	5.362	2.437	2.908	2.620	3.225	5.195	5.422	5.335	5.42[illegible]

Sample Means - Two Tailed Significance (BC) (g2 - Measurement intercepts)

	BPNSF6	BPNSF11	BPNSF17	BPNSF23	BPNSF3	BPNSF9	BPNSF14	BPNSF21	BPNSF2	BPNSF8	BPNSF20	BPNSF22	BPNSF4	BPNSF12	BPNSF16	BPNSF[illegible]
**BPNSF6**	.014	.005	.007	.004	.014	.010	.044	.025	.005	.005	.003	.006	.013	.012	.011	.01[illegible]

g3 (g3 - Measurement intercepts)

Estimates (g3 - Measurement intercepts)

Scalar Estimates (g3 - Measurement intercepts)

Maximum Likelihood Estimates

Regression Weights: (g3 - Measurement intercepts)

			Estimate	S.E.	C.R.	P	Label
BPNSF19	<---	F1	1.000				
BPNSF13	<---	F1	1.144	.052	22.216	***	a1_1
BPNSF7	<---	F1	.863	.050	17.162	***	a2_1
BPNSF1	<---	F1	.759	.050	15.166	***	a3_1
BPNSF18	<---	F2	1.000				
BPNSF15	<---	F2	2.454	.303	8.092	***	a4_1
BPNSF10	<---	F2	3.105	.370	8.386	***	a5_1
BPNSF5	<---	F2	2.516	.307	8.199	***	a6_1
BPNSF24	<---	F3	1.000				
BPNSF16	<---	F3	1.124	.046	24.274	***	a7_1
BPNSF12	<---	F3	1.221	.048	25.694	***	a8_1
BPNSF4	<---	F3	.846	.044	19.431	***	a9_1
BPNSF22	<---	F4	1.000				
BPNSF20	<---	F4	1.192	.049	24.555	***	a10_1
BPNSF8	<---	F4	1.197	.050	23.990	***	a11_1
BPNSF2	<---	F4	1.120	.049	23.055	***	a12_1
BPNSF21	<---	F5	1.000				
BPNSF14	<---	F5	1.094	.042	26.011	***	a13_1
BPNSF9	<---	F5	1.038	.042	24.673	***	a14_1
BPNSF3	<---	F5	.882	.042	21.109	***	a15_1
BPNSF23	<---	F6	1.000				
BPNSF17	<---	F6	.976	.035	28.159	***	a16_1
BPNSF11	<---	F6	.959	.034	28.209	***	a17_1
BPNSF6	<---	F6	.861	.035	24.551	***	a18_1

Standardized Regression Weights: (g3 - Measurement intercepts)

	Estimate

	Estimate
BPNSF19 <--- F1	.648
BPNSF13 <--- F1	.727
BPNSF7 <--- F1	.551
BPNSF1 <--- F1	.449
BPNSF18 <--- F2	.272
BPNSF15 <--- F2	.563
BPNSF10 <--- F2	.760
BPNSF5 <--- F2	.613
BPNSF24 <--- F3	.679
BPNSF16 <--- F3	.767
BPNSF12 <--- F3	.822
BPNSF4 <--- F3	.596
BPNSF22 <--- F4	.697
BPNSF20 <--- F4	.753
BPNSF8 <--- F4	.768
BPNSF2 <--- F4	.695
BPNSF21 <--- F5	.719
BPNSF14 <--- F5	.747
BPNSF9 <--- F5	.742
BPNSF3 <--- F5	.609
BPNSF23 <--- F6	.789
BPNSF17 <--- F6	.755
BPNSF11 <--- F6	.781
BPNSF6 <--- F6	.671

Intercepts: (g3 - Measurement intercepts)

	Estimate	S.E.	C.R.	P	Label
**BPNSF19**	5.140	.040	127.940	***	i1_1
**BPNSF13**	5.043	.039	130.871	***	i2_1
**BPNSF7**	4.817	.040	119.340	***	i3_1
**BPNSF1**	4.705	.041	114.698	***	i4_1
**BPNSF18**	4.316	.041	104.417	***	i5_1
**BPNSF15**	3.699	.046	81.096	***	i6_1
**BPNSF10**	3.158	.045	69.442	***	i7_1
**BPNSF5**	3.721	.044	85.234	***	i8_1
**BPNSF24**	5.225	.037	140.058	***	i9_1
**BPNSF16**	5.118	.038	134.993	***	i10_1
**BPNSF12**	5.220	.038	136.344	***	i11_1
**BPNSF4**	5.116	.037	139.813	***	i12_1
**BPNSF22**	3.135	.043	73.443	***	i13_1
**BPNSF20**	2.524	.046	55.295	***	i14_1
**BPNSF8**	2.823	.047	59.807	***	i15_1
**BPNSF2**	2.400	.046	52.041	***	i16_1
**BPNSF21**	5.178	.039	131.278	***	i17_1
**BPNSF14**	5.441	.040	137.076	***	i18_1
**BPNSF9**	5.657	.040	141.770	***	i19_1
**BPNSF3**	5.591	.040	140.422	***	i20_1
**BPNSF23**	2.250	.045	49.691	***	i21_1
**BPNSF17**	2.586	.047	55.085	***	i22_1
**BPNSF11**	2.754	.046	59.623	***	i23_1
**BPNSF6**	2.574	.047	55.227	***	i24_1

Covariances: (g3 - Measurement intercepts)

	Estimate	S.E.	C.R.	P	Label
F1 <--> F2	-.176	.034	-5.250	***	ccc1_3
F2 <--> F3	-.156	.031	-5.018	***	ccc2_3
F1 <--> F3	.783	.074	10.546	***	ccc3_3
F2 <--> F4	.380	.056	6.806	***	ccc4_3
F3 <--> F4	-.596	.073	-8.177	***	ccc5_3
F1 <--> F4	-.434	.071	-6.126	***	ccc6_3
F2 <--> F5	-.182	.035	-5.219	***	ccc7_3
F4 <--> F5	-.687	.080	-8.542	***	ccc8_3
F3 <--> F5	.839	.077	10.826	***	ccc9_3
F1 <--> F5	.866	.081	10.729	***	ccc10_3
F6 <--> F5	-1.084	.104	-10.397	***	ccc11_3
F6 <--> F3	-.741	.088	-8.438	***	ccc12_3
F6 <--> F4	1.421	.123	11.509	***	ccc13_3
F6 <--> F2	.453	.066	6.837	***	ccc14_3
F6 <--> F1	-.621	.088	-7.087	***	ccc15_3

Correlations: (g3 - Measurement intercepts)

	Estimate
F1 <--> F2	-.473
F2 <--> F3	-.406
F1 <--> F3	.892
F2 <--> F4	.837
F3 <--> F4	-.557
F1 <--> F4	-.417
F2 <--> F5	-.439
F4 <--> F5	-.594
F3 <--> F5	.858
F1 <--> F5	.913
F6 <--> F5	-.762
F6 <--> F3	-.563
F6 <--> F4	.912
F6 <--> F2	.812
F6 <--> F1	-.486

Variances: (g3 - Measurement intercepts)

	Estimate	S.E.	C.R.	P	Label
**F1**	.851	.097	8.772	***	vvv1_3
**F2**	.162	.040	4.066	***	vvv2_3
**F3**	.904	.091	9.891	***	vvv3_3
**F4**	1.269	.128	9.945	***	vvv4_3
**F5**	1.057	.104	10.117	***	vvv5_3
**F6**	1.915	.173	11.051	***	vvv6_3
**e1**	1.176	.090	13.033	***	v1_3
**e2**	.992	.084	11.820	***	v2_3
**e3**	1.454	.106	13.772	***	v3_3
**e4**	1.944	.137	14.180	***	v4_3
**e5**	2.030	.141	14.449	***	v5_3
**e6**	2.101	.157	13.348	***	v6_3
**e7**	1.142	.113	10.117	***	v7_3
**e8**	1.704	.132	12.919	***	v8_3
**e9**	1.055	.081	13.066	***	v9_3
**e10**	.800	.067	11.936	***	v10_3
**e11**	.647	.061	10.587	***	v11_3
**e12**	1.176	.086	13.649	***	v12_3
**e13**	1.345	.103	13.004	***	v13_3
**e14**	1.377	.112	12.313	***	v14_3
**e15**	1.261	.105	12.051	***	v15_3
**e16**	1.703	.131	13.028	***	v16_3
**e17**	.985	.076	13.006	***	v17_3
**e18**	1.005	.079	12.673	***	v18_3
**e19**	.931	.073	12.732	***	v19_3
**e20**	1.395	.101	13.813	***	v20_3
**e21**	1.164	.095	12.291	***	v21_3
**e22**	1.375	.108	12.789	***	v22_3
**e23**	1.125	.091	12.411	***	v23_3
**e24**	1.730	.128	13.540	***	v24_3

Matrices (g3 - Measurement intercepts)

Residual Covariances (g3 - Measurement intercepts)

	BPNSF6	BPNSF11	BPNSF17	BPNSF23	BPNSF3	BPNSF9	BPNSF14	BPNSF21	BPNSF2	BPNSF8	BPNSF20	BPNSF22	BPNSF4	BPNSF12	BPNSF16	BPNS[illegible]
**BPNSF6**	-.139															
**BPNSF11**	.066	.025														
**BPNSF17**	-.052	-.026	.005													
**BPNSF23**	-.114	-.061	.033	-.055												
**BPNSF3**	-.046	.030	-.092	-.117	-.061											
**BPNSF9**	-.108	-.209	-.280	-.047	.159	.099										
**BPNSF14**	.260	.135	-.010	.107	-.067	.156	-.071									
**BPNSF21**	.281	.114	.380	.118	-.170	-.159	-.163	-.137								
**BPNSF2**	-.197	-.045	-.134	.061	-.260	.038	.104	.277	-.061							
**BPNSF8**	-.031	.122	-.019	-.093	-.065	-.188	.228	.163	.054	.082						
**BPNSF20**	-.175	-.072	.242	.267	-.212	-.216	.072	.089	-.048	-.024	-.025					
**BPNSF22**	-.309	-.156	-.265	-.005	-.076	.073	.245	.051	-.191	.016	-.056	-.146				
**BPNSF4**	-.014	.014	.109	.040	.413	-.049	-.213	-.074	.155	.003	.003	-.090	-.101			

	BPNSF6	BPNSF11	BPNSF17	BPNSF23	BPNSF3	BPNSF9	BPNSF14	BPNSF21	BPNSF2	BPNSF8	BPNSF20	BPNSF22	BPNSF4	BPNSF12	BPNSF16	BPNSI
**BPNSF12**	.162	.045	-.011	-.055	-.088	.099	.083	-.023	.074	.141	.030	-.099	-.058	.096		
**BPNSF16**	.193	.001	-.058	.057	-.205	-.078	.006	-.006	.118	.137	-.089	-.068	-.070	.086	-.018	
**BPNSF24**	-.003	-.149	.070	-.120	-.159	-.085	-.094	.144	-.030	-.092	-.096	.003	-.083	-.040	-.084	-.0
**BPNSF5**	.089	.154	-.076	-.065	.008	-.063	.083	.115	.395	.207	-.085	.070	-.135	-.095	-.135	-.1
**BPNSF10**	.075	.342	.075	-.044	-.180	-.324	.026	.037	.051	.202	-.127	-.061	-.120	.009	-.008	-.0
**BPNSF15**	-.253	-.179	-.109	-.465	.052	.037	.131	.224	-.239	-.032	-.334	-.192	-.121	.191	.093	.0
**BPNSF18**	-.251	-.134	-.032	-.349	.060	.345	.455	.266	-.215	-.208	-.211	-.017	-.011	.369	.282	.4
**BPNSF1**	.029	-.004	.057	.082	.376	-.004	-.210	-.067	.192	-.042	-.034	-.257	.269	-.061	-.132	-.1
**BPNSF7**	.297	-.052	.049	.035	-.123	-.028	-.023	.084	.146	.189	.089	-.042	-.098	-.001	-.091	-.1
**BPNSF13**	.122	-.035	.003	.163	-.269	-.051	-.023	-.109	.115	.089	.026	-.157	-.038	.209	.013	-.0
**BPNSF19**	.008	-.096	.056	-.081	-.126	-.042	-.061	.257	.168	.009	.015	-.024	-.023	-.127	-.030	-.0

Residual Means (g3 - Measurement intercepts)

	BPNSF6	BPNSF11	BPNSF17	BPNSF23	BPNSF3	BPNSF9	BPNSF14	BPNSF21	BPNSF2	BPNSF8	BPNSF20	BPNSF22	BPNSF4	BPNSF12	BPNSF16	BPNSF24	BPI
	.058	.052	.242	.253	-.100	-.196	-.258	-.180	.245	.094	.237	.140	.089	-.113	-.099	-.109	.

Standardized Residual Covariances (g3 - Measurement intercepts)

	BPNSF6	BPNSF11	BPNSF17	BPNSF23	BPNSF3	BPNSF9	BPNSF14	BPNSF21	BPNSF2	BPNSF8	BPNSF20	BPNSF22	BPNSF4	BPNSF12	BPNSF16	BPNSI
**BPNSF6**	-.648															
**BPNSF11**	.400	.127														
**BPNSF17**	-.300	-.153	.022													
**BPNSF23**	-.672	-.362	.185	-.262												
**BPNSF3**	-.344	.231	-.678	-.875	-.401											
**BPNSF9**	-.822	-1.624	-2.074	-.356	1.402	.698										
**BPNSF14**	1.881	1.000	-.073	.762	-.567	1.307	-.457									
**BPNSF21**	2.156	.892	2.850	.893	-1.515	-1.417	-1.383	-.985								
**BPNSF2**	-1.165	-.268	-.771	.356	-1.935	.289	.754	2.125	-.273							
**BPNSF8**	-.188	.744	-.109	-.549	-.495	-1.463	1.693	1.281	.313	.393						
**BPNSF20**	-1.040	-.433	1.399	1.553	-1.597	-1.660	.529	.687	-.270	-.136	-.117					
**BPNSF22**	-2.053	-1.053	-1.712	-.033	-.633	.621	1.993	.436	-1.213	.106	-.356	-.819				
**BPNSF4**	-.116	.121	.908	.341	4.072	-.491	-2.024	-.743	1.275	.027	.026	-.832	-.816			
**BPNSF12**	1.280	.369	-.085	-.431	-.794	.892	.715	-.215	.568	1.117	.237	-.860	-.563	.703		
**BPNSF16**	1.556	.009	-.460	.460	-1.902	-.728	.058	-.058	.931	1.105	-.709	-.597	-.704	.767	-.137	
**BPNSF24**	-.027	-1.243	.556	-.972	-1.487	-.801	-.850	1.374	-.240	-.747	-.768	.027	-.846	-.365	-.791	-.7
**BPNSF5**	.594	1.061	-.501	-.433	.066	-.535	.676	.987	2.570	1.380	-.555	.508	-1.238	-.829	-1.196	-1.2
**BPNSF10**	.491	2.289	.476	-.286	-1.490	-2.755	.215	.320	.326	1.306	-.806	-.432	-1.101	.076	-.073	-.2
**BPNSF15**	-1.609	-1.172	-.678	-2.945	.409	.299	1.011	1.824	-1.479	-.204	-2.089	-1.332	-1.048	1.571	.779	.5
**BPNSF18**	-1.960	-1.089	-.244	-2.743	.566	3.345	4.208	2.598	-1.641	-1.637	-1.630	-.146	-.115	3.639	2.825	4.3
**BPNSF1**	.213	-.027	.417	.611	3.255	-.035	-1.771	-.595	1.391	-.316	-.252	-2.098	2.576	-.541	-1.202	-1.0
**BPNSF7**	2.362	-.434	.383	.279	-1.134	-.264	-.206	.796	1.142	1.521	.705	-.371	-.999	-.007	-.872	-1.5
**BPNSF13**	.952	-.282	.027	1.282	-2.390	-.451	-.196	-.982	.885	.703	.203	-1.361	-.379	1.865	.116	-.5
**BPNSF19**	.068	-.797	.446	-.649	-1.162	-.385	-.542	2.414	1.326	.074	.121	-.211	-.233	-1.179	-.284	-.5

Standardized Residual Means (g3 - Measurement intercepts)

	BPNSF6	BPNSF11	BPNSF17	BPNSF23	BPNSF3	BPNSF9	BPNSF14	BPNSF21	BPNSF2	BPNSF8	BPNSF20	BPNSF22	BPNSF4	BPNSF12	BPNSF16	BPNSF24	BPI
	.682	.632	2.810	2.995	-1.392	-2.818	-3.547	-2.610	2.795	1.105	2.761	1.790	1.361	-1.653	-1.468	-1.614	2.

Notes for Group/Model (g3 - Measurement intercepts)

The following covariance matrix is not positive definite (g3 - Measurement intercepts)

	F5	F4	F3	F2	F1	F6
**F5**	1.057					
**F4**	-.687	1.269				
**F3**	.839	-.596	.904			
**F2**	-.182	.380	-.156	.162		
**F1**	.866	-.434	.783	-.176	.851	
**F6**	-1.084	1.421	-.741	.453	-.621	1.915

This solution is not admissible.

Modification Indices (g3 - Measurement intercepts)

Covariances: (g3 - Measurement intercepts)

			M.I.	Par Change
e24	<-->	F4	5.339	-.112

			M.I.	Par Change
e23	<-->	F2	10.548	.059
e23	<-->	e24	4.727	.162
e21	<-->	F4	10.838	.135
e21	<-->	F2	14.749	-.071
e20	<-->	F4	4.957	-.097
e20	<-->	e23	4.744	.145
e19	<-->	e22	6.668	-.158
e19	<-->	e21	5.630	.136
e19	<-->	e20	8.777	.176
e18	<-->	e22	4.841	-.140
e18	<-->	e19	10.661	.170
e17	<-->	F5	11.275	-.099
e17	<-->	F1	11.623	.116
e17	<-->	e22	14.511	.238
e17	<-->	e19	10.536	-.166
e16	<-->	F1	4.400	.096
e16	<-->	e20	10.625	-.263
e15	<-->	F2	6.027	.048
e15	<-->	e21	8.123	-.195
e15	<-->	e19	5.978	-.147
e14	<-->	F2	13.807	-.075
e14	<-->	F6	7.910	.138
e14	<-->	e23	6.605	-.179
e14	<-->	e22	14.560	.290
e14	<-->	e21	19.029	.310
e13	<-->	F5	8.091	.107
e13	<-->	F1	10.642	-.132
e13	<-->	e22	4.310	-.152
e13	<-->	e21	5.848	.166
e13	<-->	e19	11.091	.201
e13	<-->	e18	6.303	.158
e12	<-->	F2	8.222	-.052
e12	<-->	e22	4.521	.143
e12	<-->	e20	70.002	.549
e12	<-->	e18	12.578	-.204
e10	<-->	e22	4.042	-.119
e10	<-->	e20	8.926	-.171
e9	<-->	e22	5.078	.147
e9	<-->	e17	15.860	.217
e8	<-->	e16	17.658	.384
e7	<-->	e23	14.997	.258
e7	<-->	e19	7.201	-.161
e7	<-->	e14	5.067	-.167
e6	<-->	e21	8.643	-.249
e6	<-->	e12	5.151	-.185
e5	<-->	F2	4.535	.049
e5	<-->	e22	4.963	.192
e5	<-->	e12	6.078	-.191
e5	<-->	e9	8.965	.224
e5	<-->	e8	17.471	.398
e5	<-->	e6	5.169	.237
e4	<-->	F2	19.907	-.102
e4	<-->	F6	6.721	.144
e4	<-->	e20	36.494	.500
e4	<-->	e18	6.231	-.181
e4	<-->	e16	6.219	.234
e4	<-->	e13	5.921	-.203
e4	<-->	e12	18.868	.332
e4	<-->	e6	4.652	-.222
e4	<-->	e5	25.665	-.496
e3	<-->	e24	6.564	.209
e3	<-->	e17	4.658	.134
e3	<-->	e7	7.139	-.194
e2	<-->	F5	6.143	-.078
e2	<-->	F3	6.900	.087
e2	<-->	e20	12.755	-.222
e2	<-->	e13	6.655	-.163
e2	<-->	e11	17.604	.199
e2	<-->	e7	4.518	.133
e1	<-->	e17	33.930	.330
e1	<-->	e11	10.934	-.167

Variances: (g3 - Measurement intercepts)

	M.I.	Par Change

Regression Weights: (g3 - Measurement intercepts)

			M.I.	Par Change
BPNSF6	<---	BPNSF2	4.468	-.046
BPNSF6	<---	BPNSF20	5.200	-.049
BPNSF6	<---	BPNSF22	4.528	-.040
BPNSF11	<---	BPNSF17	4.695	-.038
BPNSF11	<---	BPNSF23	4.742	-.042
BPNSF11	<---	BPNSF9	5.681	-.022
BPNSF11	<---	BPNSF21	5.989	-.025
BPNSF11	<---	BPNSF2	4.210	-.037
BPNSF11	<---	BPNSF20	7.160	-.048
BPNSF11	<---	BPNSF22	4.762	-.034
BPNSF11	<---	BPNSF4	4.909	-.023
BPNSF11	<---	BPNSF12	4.242	-.021
BPNSF11	<---	BPNSF16	5.239	-.024
BPNSF11	<---	BPNSF24	6.594	-.026
BPNSF11	<---	BPNSF1	4.109	-.022
BPNSF11	<---	BPNSF7	6.740	-.028
BPNSF11	<---	BPNSF13	6.195	-.026
BPNSF11	<---	BPNSF19	5.794	-.025
BPNSF17	<---	BPNSF21	4.057	.023
BPNSF17	<---	BPNSF20	7.111	.052
BPNSF17	<---	BPNSF18	4.466	.028
BPNSF23	<---	BPNSF9	4.100	.019
BPNSF23	<---	BPNSF2	5.133	.042
BPNSF23	<---	BPNSF20	10.455	.059
BPNSF23	<---	BPNSF22	6.351	.040
BPNSF23	<---	BPNSF1	4.713	.025
BPNSF3	<---	BPNSF4	5.404	.026
BPNSF3	<---	BPNSF1	5.219	.027
BPNSF9	<---	F4	6.367	-.117
BPNSF9	<---	F2	6.448	-.340
BPNSF9	<---	F6	9.149	-.112
BPNSF9	<---	BPNSF6	4.074	-.032
BPNSF9	<---	BPNSF11	4.088	-.031
BPNSF9	<---	BPNSF17	6.955	-.041
BPNSF9	<---	BPNSF8	4.938	-.033
BPNSF9	<---	BPNSF10	4.174	-.028
BPNSF14	<---	BPNSF4	5.687	-.023
BPNSF14	<---	BPNSF1	5.335	-.024
BPNSF21	<---	F4	4.706	.103
BPNSF21	<---	F6	8.624	.111
BPNSF21	<---	BPNSF17	5.078	.036
BPNSF2	<---	BPNSF23	4.230	.048
BPNSF2	<---	BPNSF9	4.922	.025
BPNSF2	<---	BPNSF21	4.922	.027
BPNSF2	<---	BPNSF4	5.584	.030
BPNSF2	<---	BPNSF16	4.571	.027
BPNSF2	<---	BPNSF5	9.455	.050
BPNSF2	<---	BPNSF1	6.881	.035
BPNSF2	<---	BPNSF7	4.128	.027
BPNSF2	<---	BPNSF13	4.173	.026
BPNSF2	<---	BPNSF19	5.084	.028
BPNSF20	<---	BPNSF17	10.770	.064
BPNSF20	<---	BPNSF23	11.951	.074
BPNSF4	<---	BPNSF6	5.478	.040
BPNSF4	<---	BPNSF11	10.171	.053
BPNSF4	<---	BPNSF17	14.515	.066
BPNSF4	<---	BPNSF23	11.745	.065
BPNSF4	<---	BPNSF3	24.130	.046
BPNSF4	<---	BPNSF9	9.944	.029
BPNSF4	<---	BPNSF14	6.748	.025
BPNSF4	<---	BPNSF21	9.468	.031
BPNSF4	<---	BPNSF2	13.629	.066
BPNSF4	<---	BPNSF8	8.067	.046
BPNSF4	<---	BPNSF20	10.847	.058
BPNSF4	<---	BPNSF22	8.846	.046

			M.I.	Par Change
BPNSF4	<---	BPNSF12	9.223	.030
BPNSF4	<---	BPNSF16	9.842	.032
BPNSF4	<---	BPNSF24	10.308	.032
BPNSF4	<---	BPNSF5	9.267	.040
BPNSF4	<---	BPNSF10	6.736	.039
BPNSF4	<---	BPNSF15	5.144	.030
BPNSF4	<---	BPNSF18	6.392	.030
BPNSF4	<---	BPNSF1	20.358	.049
BPNSF4	<---	BPNSF7	9.536	.033
BPNSF4	<---	BPNSF13	9.188	.031
BPNSF4	<---	BPNSF19	11.160	.034
BPNSF5	<---	BPNSF6	4.314	.044
BPNSF5	<---	BPNSF2	13.546	.081
BPNSF5	<---	BPNSF8	4.811	.044
BPNSF5	<---	BPNSF22	4.018	.038
BPNSF5	<---	BPNSF18	8.639	.043
BPNSF10	<---	BPNSF17	4.812	-.041
BPNSF10	<---	BPNSF23	6.037	-.051
BPNSF10	<---	BPNSF3	11.960	-.035
BPNSF10	<---	BPNSF9	14.402	-.038
BPNSF10	<---	BPNSF14	10.855	-.034
BPNSF10	<---	BPNSF21	10.390	-.035
BPNSF10	<---	BPNSF2	5.320	-.045
BPNSF10	<---	BPNSF20	9.744	-.059
BPNSF10	<---	BPNSF22	9.228	-.051
BPNSF10	<---	BPNSF4	11.625	-.038
BPNSF10	<---	BPNSF12	9.800	-.034
BPNSF10	<---	BPNSF16	9.636	-.034
BPNSF10	<---	BPNSF24	9.194	-.033
BPNSF10	<---	BPNSF5	9.382	-.044
BPNSF10	<---	BPNSF15	6.731	-.037
BPNSF10	<---	BPNSF18	12.732	-.046
BPNSF10	<---	BPNSF1	12.429	-.042
BPNSF10	<---	BPNSF7	14.546	-.045
BPNSF10	<---	BPNSF13	8.174	-.032
BPNSF10	<---	BPNSF19	10.182	-.035
BPNSF15	<---	F4	5.151	-.155
BPNSF15	<---	F2	5.167	-.447
BPNSF15	<---	F6	5.681	-.131
BPNSF15	<---	BPNSF23	7.516	-.070
BPNSF18	<---	F5	12.900	.253
BPNSF18	<---	F3	14.036	.290
BPNSF18	<---	F1	9.968	.252
BPNSF18	<---	BPNSF9	7.077	.032
BPNSF18	<---	BPNSF14	7.577	.034
BPNSF18	<---	BPNSF21	5.449	.030
BPNSF18	<---	BPNSF12	7.220	.034
BPNSF18	<---	BPNSF16	6.216	.033
BPNSF18	<---	BPNSF24	8.528	.037
BPNSF18	<---	BPNSF5	8.479	.049
BPNSF18	<---	BPNSF15	5.739	.040
BPNSF18	<---	BPNSF7	7.050	.037
BPNSF18	<---	BPNSF13	5.862	.032
BPNSF18	<---	BPNSF19	5.678	.031
BPNSF13	<---	BPNSF6	4.606	-.035
BPNSF13	<---	BPNSF11	5.376	-.037
BPNSF13	<---	BPNSF17	6.437	-.042
BPNSF13	<---	BPNSF3	11.234	-.030
BPNSF13	<---	BPNSF9	7.688	-.024
BPNSF13	<---	BPNSF14	6.279	-.023
BPNSF13	<---	BPNSF21	8.306	-.028
BPNSF13	<---	BPNSF2	4.618	-.037
BPNSF13	<---	BPNSF8	4.449	-.033
BPNSF13	<---	BPNSF20	5.597	-.039
BPNSF13	<---	BPNSF22	10.826	-.048
BPNSF13	<---	BPNSF4	7.963	-.027
BPNSF13	<---	BPNSF16	5.862	-.023
BPNSF13	<---	BPNSF24	6.401	-.024
BPNSF13	<---	BPNSF5	6.450	-.032
BPNSF13	<---	BPNSF15	5.040	-.028
BPNSF13	<---	BPNSF18	5.932	-.027
BPNSF13	<---	BPNSF1	8.564	-.030

			M.I.	Par Change
BPNSF13	<---	BPNSF7	4.851	-.023
BPNSF13	<---	BPNSF19	7.050	-.026
BPNSF19	<---	BPNSF6	4.281	-.036
BPNSF19	<---	BPNSF23	6.266	-.048
BPNSF19	<---	BPNSF12	5.506	-.024
BPNSF19	<---	BPNSF5	4.139	-.027
BPNSF19	<---	BPNSF7	4.230	-.022

Means: (g3 - Measurement intercepts)

	M.I.	Par Change

Intercepts: (g3 - Measurement intercepts)

	M.I.	Par Change
**BPNSF11**	5.162	-.125
**BPNSF4**	12.563	.192
**BPNSF10**	10.097	-.187
**BPNSF13**	7.526	-.141

Bootstrap (g3 - Measurement intercepts)

Bootstrap standard errors (g3 - Measurement intercepts)

Scalar Estimates (g3 - Measurement intercepts)

Regression Weights: (g3 - Measurement intercepts)

Parameter			SE	SE-SE	Mean	Bias	SE-Bias
BPNSF19	<---	F1	.000	.000	1.000	.000	.000
BPNSF13	<---	F1	.057	.003	1.139	-.005	.004
BPNSF7	<---	F1	.059	.003	.862	.000	.004
BPNSF1	<---	F1	.071	.004	.758	-.001	.005
BPNSF18	<---	F2	.000	.000	1.000	.000	.000
BPNSF15	<---	F2	.387	.019	2.498	.043	.027
BPNSF10	<---	F2	.513	.026	3.163	.058	.036
BPNSF5	<---	F2	.410	.021	2.563	.047	.029
BPNSF24	<---	F3	.000	.000	1.000	.000	.000
BPNSF16	<---	F3	.054	.003	1.125	.000	.004
BPNSF12	<---	F3	.049	.002	1.215	-.006	.003
BPNSF4	<---	F3	.051	.003	.844	-.002	.004
BPNSF22	<---	F4	.000	.000	1.000	.000	.000
BPNSF20	<---	F4	.051	.003	1.191	-.001	.004
BPNSF8	<---	F4	.050	.002	1.191	-.005	.004
BPNSF2	<---	F4	.054	.003	1.118	-.002	.004
BPNSF21	<---	F5	.000	.000	1.000	.000	.000
BPNSF14	<---	F5	.045	.002	1.096	.002	.003
BPNSF9	<---	F5	.052	.003	1.040	.002	.004
BPNSF3	<---	F5	.059	.003	.887	.005	.004
BPNSF23	<---	F6	.000	.000	1.000	.000	.000
BPNSF17	<---	F6	.034	.002	.979	.003	.002
BPNSF11	<---	F6	.038	.002	.959	.000	.003
BPNSF6	<---	F6	.037	.002	.861	.000	.003

Standardized Regression Weights: (g3 - Measurement intercepts)

Parameter			SE	SE-SE	Mean	Bias	SE-Bias
BPNSF19	<---	F1	.035	.002	.650	.002	.002
BPNSF13	<---	F1	.028	.001	.725	-.002	.002
BPNSF7	<---	F1	.037	.002	.552	.001	.003
BPNSF1	<---	F1	.039	.002	.450	.001	.003
BPNSF18	<---	F2	.042	.002	.274	.002	.003
BPNSF15	<---	F2	.030	.001	.563	.000	.002
BPNSF10	<---	F2	.034	.002	.760	.000	.002
BPNSF5	<---	F2	.036	.002	.613	-.001	.003
BPNSF24	<---	F3	.029	.001	.679	.000	.002
BPNSF16	<---	F3	.031	.002	.769	.002	.002
BPNSF12	<---	F3	.022	.001	.821	-.001	.002
BPNSF4	<---	F3	.034	.002	.594	-.002	.002

Parameter	SE	SE-SE	Mean	Bias	SE-Bias
BPNSF22 <--- F4	.025	.001	.699	.002	.002
BPNSF20 <--- F4	.031	.002	.755	.002	.002
BPNSF8 <--- F4	.028	.001	.766	-.002	.002
BPNSF2 <--- F4	.032	.002	.695	.000	.002
BPNSF21 <--- F5	.032	.002	.724	.005	.002
BPNSF14 <--- F5	.034	.002	.751	.004	.002
BPNSF9 <--- F5	.032	.002	.746	.004	.002
BPNSF3 <--- F5	.043	.002	.615	.006	.003
BPNSF23 <--- F6	.023	.001	.790	.001	.002
BPNSF17 <--- F6	.028	.001	.758	.003	.002
BPNSF11 <--- F6	.025	.001	.780	-.001	.002
BPNSF6 <--- F6	.032	.002	.670	-.001	.002

Intercepts: (g3 - Measurement intercepts)

Parameter	SE	SE-SE	Mean	Bias	SE-Bias
**BPNSF19**	.045	.002	5.148	.008	.003
**BPNSF13**	.042	.002	5.052	.009	.003
**BPNSF7**	.042	.002	4.827	.009	.003
**BPNSF1**	.040	.002	4.713	.008	.003
**BPNSF18**	.041	.002	4.315	-.001	.003
**BPNSF15**	.047	.002	3.698	-.002	.003
**BPNSF10**	.051	.003	3.153	-.005	.004
**BPNSF5**	.047	.002	3.717	-.004	.003
**BPNSF24**	.041	.002	5.232	.006	.003
**BPNSF16**	.044	.002	5.125	.008	.003
**BPNSF12**	.039	.002	5.228	.008	.003
**BPNSF4**	.039	.002	5.121	.006	.003
**BPNSF22**	.047	.002	3.128	-.007	.003
**BPNSF20**	.054	.003	2.512	-.013	.004
**BPNSF8**	.055	.003	2.817	-.006	.004
**BPNSF2**	.048	.002	2.393	-.007	.003
**BPNSF21**	.042	.002	5.189	.011	.003
**BPNSF14**	.044	.002	5.452	.011	.003
**BPNSF9**	.046	.002	5.666	.009	.003
**BPNSF3**	.043	.002	5.600	.009	.003
**BPNSF23**	.046	.002	2.239	-.010	.003
**BPNSF17**	.053	.003	2.574	-.011	.004
**BPNSF11**	.049	.002	2.749	-.005	.003
**BPNSF6**	.053	.003	2.570	-.004	.004

Covariances: (g3 - Measurement intercepts)

Parameter	SE	SE-SE	Mean	Bias	SE-Bias
F1 <--> F2	.034	.002	-.176	.000	.002
F2 <--> F3	.028	.001	-.154	.001	.002
F1 <--> F3	.066	.003	.781	-.002	.005
F2 <--> F4	.064	.003	.381	.001	.005
F3 <--> F4	.073	.004	-.601	-.005	.005
F1 <--> F4	.068	.003	-.440	-.007	.005
F2 <--> F5	.033	.002	-.180	.002	.002
F4 <--> F5	.073	.004	-.690	-.003	.005
F3 <--> F5	.079	.004	.838	.000	.006
F1 <--> F5	.089	.004	.870	.004	.006
F6 <--> F5	.093	.005	-1.093	-.009	.007
F6 <--> F3	.083	.004	-.744	-.003	.006
F6 <--> F4	.105	.005	1.423	.002	.007
F6 <--> F2	.073	.004	.453	.000	.005
F6 <--> F1	.081	.004	-.630	-.009	.006

Correlations: (g3 - Measurement intercepts)

Parameter	SE	SE-SE	Mean	Bias	SE-Bias
F1 <--> F2	.062	.003	-.473	.000	.004
F2 <--> F3	.054	.003	-.405	.001	.004
F1 <--> F3	.036	.002	.889	-.003	.003
F2 <--> F4	.037	.002	.835	-.002	.003
F3 <--> F4	.055	.003	-.560	-.004	.004
F1 <--> F4	.058	.003	-.422	-.004	.004
F2 <--> F5	.060	.003	-.434	.005	.004

Parameter			SE	SE-SE	Mean	Bias	SE-Bias
F4	<-->	F5	.049	.002	-.592	.002	.003
F3	<-->	F5	.040	.002	.853	-.005	.003
F1	<-->	F5	.045	.002	.910	-.003	.003
F6	<-->	F5	.035	.002	-.763	-.001	.002
F6	<-->	F3	.046	.002	-.565	-.001	.003
F6	<-->	F4	.030	.002	.909	-.002	.002
F6	<-->	F2	.045	.002	.810	-.002	.003
F6	<-->	F1	.051	.003	-.491	-.005	.004

Variances: (g3 - Measurement intercepts)

Parameter	SE	SE-SE	Mean	Bias	SE-Bias
**F1**	.097	.005	.858	.007	.007
**F2**	.051	.003	.167	.005	.004
**F3**	.079	.004	.905	.000	.006
**F4**	.109	.005	1.276	.008	.008
**F5**	.109	.005	1.070	.013	.008
**F6**	.152	.008	1.923	.008	.011
**e1**	.137	.007	1.168	-.008	.010
**e2**	.110	.005	.999	.007	.008
**e3**	.163	.008	1.447	-.007	.012
**e4**	.170	.008	1.931	-.013	.012
**e5**	.149	.007	2.011	-.019	.011
**e6**	.159	.008	2.097	-.004	.011
**e7**	.153	.008	1.138	-.005	.011
**e8**	.162	.008	1.703	-.001	.011
**e9**	.128	.006	1.057	.001	.009
**e10**	.113	.006	.789	-.011	.008
**e11**	.080	.004	.643	-.004	.006
**e12**	.142	.007	1.180	.004	.010
**e13**	.121	.006	1.333	-.012	.009
**e14**	.194	.010	1.360	-.016	.014
**e15**	.150	.007	1.273	.011	.011
**e16**	.208	.010	1.702	-.001	.015
**e17**	.133	.007	.971	-.015	.009
**e18**	.149	.007	.993	-.012	.011
**e19**	.125	.006	.919	-.012	.009
**e20**	.219	.011	1.388	-.007	.016
**e21**	.132	.007	1.159	-.005	.009
**e22**	.172	.009	1.365	-.010	.012
**e23**	.132	.007	1.132	.008	.009
**e24**	.213	.011	1.747	.017	.015

Matrices (g3 - Measurement intercepts)

Sample Covariances - Standard Errors (g3 - Measurement intercepts)

	BPNSF6	BPNSF11	BPNSF17	BPNSF23	BPNSF3	BPNSF9	BPNSF14	BPNSF21	BPNSF2	BPNSF8	BPNSF20	BPNSF22	BPNSF4	BPNSF12	BPNSF16	BPNS[illegible]
**BPNSF6**	.174															
**BPNSF11**	.152	.149														
**BPNSF17**	.156	.158	.157													
**BPNSF23**	.148	.139	.145	.159												
**BPNSF3**	.126	.127	.121	.132	.192											
**BPNSF9**	.138	.124	.143	.135	.141	.177										
**BPNSF14**	.122	.118	.144	.129	.125	.136	.147									
**BPNSF21**	.111	.130	.132	.113	.123	.120	.119	.134								
**BPNSF2**	.166	.157	.165	.138	.132	.126	.130	.112	.180							
**BPNSF8**	.149	.146	.165	.145	.142	.145	.130	.129	.170	.163						
**BPNSF20**	.157	.154	.159	.156	.127	.141	.134	.133	.159	.160	.176					
**BPNSF22**	.142	.130	.152	.137	.133	.134	.135	.120	.131	.138	.140	.132				
**BPNSF4**	.114	.118	.113	.099	.135	.106	.103	.114	.122	.113	.116	.114	.127			
**BPNSF12**	.119	.124	.137	.125	.122	.131	.127	.103	.123	.137	.135	.122	.095	.138		
**BPNSF16**	.123	.117	.123	.119	.110	.130	.117	.112	.108	.134	.138	.117	.094	.114	.120	
**BPNSF24**	.112	.108	.121	.111	.120	.113	.111	.106	.124	.123	.120	.117	.104	.104	.095	[illegible]
**BPNSF5**	.141	.131	.140	.129	.130	.112	.127	.118	.161	.151	.154	.140	.112	.123	.113	[illegible]
**BPNSF10**	.160	.140	.151	.132	.124	.132	.116	.113	.148	.149	.139	.134	.111	.108	.117	[illegible]
**BPNSF15**	.166	.134	.158	.133	.126	.120	.130	.131	.130	.151	.157	.139	.112	.120	.115	[illegible]
**BPNSF18**	.129	.121	.138	.115	.109	.110	.121	.103	.139	.144	.131	.121	.102	.117	.110	[illegible]
**BPNSF1**	.145	.127	.148	.126	.141	.125	.133	.128	.140	.150	.143	.129	.117	.123	.125	[illegible]
**BPNSF7**	.129	.123	.135	.113	.134	.123	.121	.107	.138	.153	.122	.137	.114	.111	.117	[illegible]
**BPNSF13**	.132	.114	.130	.118	.124	.118	.123	.101	.125	.149	.135	.127	.097	.117	.111	[illegible]

	BPNSF6	BPNSF11	BPNSF17	BPNSF23	BPNSF3	BPNSF9	BPNSF14	BPNSF21	BPNSF2	BPNSF8	BPNSF20	BPNSF22	BPNSF4	BPNSF12	BPNSF16	BPNS[illegible]
**BPNSF19**	.108	.103	.128	.100	.098	.119	.118	.107	.116	.133	.125	.104	.095	.108	.102	.0[illegible]

Sample Correlations - Standard Errors (g3 - Measurement intercepts)

	BPNSF6	BPNSF11	BPNSF17	BPNSF23	BPNSF3	BPNSF9	BPNSF14	BPNSF21	BPNSF2	BPNSF8	BPNSF20	BPNSF22	BPNSF4	BPNSF12	BPNSF16	BPNS[illegible]
**BPNSF6**	.000															
**BPNSF11**	.046	.000														
**BPNSF17**	.047	.045	.000													
**BPNSF23**	.045	.035	.039	.000												
**BPNSF3**	.052	.053	.048	.053	.000											
**BPNSF9**	.050	.042	.044	.045	.059	.000										
**BPNSF14**	.047	.047	.050	.046	.052	.041	.000									
**BPNSF21**	.048	.051	.052	.044	.056	.053	.055	.000								
**BPNSF2**	.052	.048	.049	.043	.051	.049	.050	.049	.000							
**BPNSF8**	.045	.039	.046	.040	.053	.049	.051	.052	.048	.000						
**BPNSF20**	.048	.045	.038	.038	.049	.048	.048	.054	.045	.045	.000					
**BPNSF22**	.051	.042	.051	.041	.054	.058	.058	.054	.041	.040	.047	.000				
**BPNSF4**	.050	.053	.048	.043	.054	.053	.052	.059	.055	.050	.050	.056	.000			
**BPNSF12**	.046	.048	.050	.049	.054	.041	.048	.043	.048	.054	.051	.049	.049	.000		
**BPNSF16**	.052	.048	.048	.050	.056	.054	.048	.051	.044	.055	.052	.052	.053	.038	.000	
**BPNSF24**	.045	.044	.050	.044	.058	.051	.051	.046	.051	.050	.049	.054	.058	.043	.047	.0[illegible]
**BPNSF5**	.044	.039	.043	.040	.053	.043	.050	.051	.043	.043	.047	.047	.050	.048	.047	.0[illegible]
**BPNSF10**	.054	.038	.044	.041	.054	.049	.048	.050	.048	.044	.045	.049	.052	.046	.051	.0[illegible]
**BPNSF15**	.054	.043	.050	.045	.051	.047	.051	.057	.043	.047	.049	.049	.051	.050	.050	.0[illegible]
**BPNSF18**	.051	.048	.050	.045	.050	.050	.055	.051	.053	.055	.049	.050	.052	.055	.054	.0[illegible]
**BPNSF1**	.054	.049	.053	.047	.051	.052	.057	.060	.050	.054	.052	.051	.053	.053	.058	.0[illegible]
**BPNSF7**	.052	.051	.052	.045	.065	.056	.056	.049	.054	.060	.049	.061	.058	.051	.058	.0[illegible]
**BPNSF13**	.052	.048	.049	.050	.058	.046	.052	.048	.050	.061	.054	.054	.048	.037	.047	.0[illegible]
**BPNSF19**	.046	.041	.051	.042	.049	.050	.056	.038	.047	.052	.052	.048	.049	.050	.046	.0[illegible]

Sample Means - Standard Errors (g3 - Measurement intercepts)

	BPNSF6	BPNSF11	BPNSF17	BPNSF23	BPNSF3	BPNSF9	BPNSF14	BPNSF21	BPNSF2	BPNSF8	BPNSF20	BPNSF22	BPNSF4	BPNSF12	BPNSF16	BPNSF[illegible]
**BPNSF6**	.080	.077	.086	.074	.073	.078	.070	.063	.088	.081	.083	.074	.063	.070	.071	.07[illegible]

Bootstrap Confidence (g3 - Measurement intercepts)

Percentile method (g3 - Measurement intercepts)

90% confidence intervals (percentile method)

Scalar Estimates (g3 - Measurement intercepts)

Regression Weights: (g3 - Measurement intercepts)

Parameter			Estimate	Lower	Upper	P
BPNSF19	<---	F1	1.000	1.000	1.000	...
BPNSF13	<---	F1	1.144	1.043	1.237	.010
BPNSF7	<---	F1	.863	.764	.963	.010
BPNSF1	<---	F1	.759	.644	.885	.010
BPNSF18	<---	F2	1.000	1.000	1.000	...
BPNSF15	<---	F2	2.454	1.993	3.280	.010
BPNSF10	<---	F2	3.105	2.497	4.232	.010
BPNSF5	<---	F2	2.516	2.001	3.370	.010
BPNSF24	<---	F3	1.000	1.000	1.000	...
BPNSF16	<---	F3	1.124	1.038	1.214	.010
BPNSF12	<---	F3	1.221	1.136	1.296	.010
BPNSF4	<---	F3	.846	.752	.934	.010
BPNSF22	<---	F4	1.000	1.000	1.000	...
BPNSF20	<---	F4	1.192	1.111	1.283	.010
BPNSF8	<---	F4	1.197	1.114	1.272	.010
BPNSF2	<---	F4	1.120	1.035	1.222	.010
BPNSF21	<---	F5	1.000	1.000	1.000	...
BPNSF14	<---	F5	1.094	1.022	1.177	.010
BPNSF9	<---	F5	1.038	.963	1.130	.010
BPNSF3	<---	F5	.882	.793	.989	.010
BPNSF23	<---	F6	1.000	1.000	1.000	...
BPNSF17	<---	F6	.976	.925	1.043	.010
BPNSF11	<---	F6	.959	.899	1.022	.010

Parameter			Estimate	Lower	Upper	P
BPNSF6	<---	F6	.861	.801	.930	.010

Standardized Regression Weights: (g3 - Measurement intercepts)

Parameter			Estimate	Lower	Upper	P
BPNSF19	<---	F1	.648	.589	.711	.010
BPNSF13	<---	F1	.727	.674	.770	.010
BPNSF7	<---	F1	.551	.488	.613	.010
BPNSF1	<---	F1	.449	.388	.514	.010
BPNSF18	<---	F2	.272	.207	.337	.010
BPNSF15	<---	F2	.563	.511	.610	.010
BPNSF10	<---	F2	.760	.702	.820	.010
BPNSF5	<---	F2	.613	.555	.679	.010
BPNSF24	<---	F3	.679	.631	.735	.010
BPNSF16	<---	F3	.767	.719	.819	.010
BPNSF12	<---	F3	.822	.785	.859	.010
BPNSF4	<---	F3	.596	.538	.656	.010
BPNSF22	<---	F4	.697	.655	.740	.010
BPNSF20	<---	F4	.753	.701	.804	.010
BPNSF8	<---	F4	.768	.715	.814	.010
BPNSF2	<---	F4	.695	.638	.745	.010
BPNSF21	<---	F5	.719	.668	.777	.010
BPNSF14	<---	F5	.747	.690	.808	.010
BPNSF9	<---	F5	.742	.692	.797	.010
BPNSF3	<---	F5	.609	.537	.682	.010
BPNSF23	<---	F6	.789	.753	.832	.010
BPNSF17	<---	F6	.755	.717	.802	.010
BPNSF11	<---	F6	.781	.733	.821	.010
BPNSF6	<---	F6	.671	.619	.730	.010

Intercepts: (g3 - Measurement intercepts)

Parameter	Estimate	Lower	Upper	P
**BPNSF19**	5.140	5.067	5.217	.010
**BPNSF13**	5.043	4.979	5.123	.010
**BPNSF7**	4.817	4.755	4.891	.010
**BPNSF1**	4.705	4.648	4.786	.010
**BPNSF18**	4.316	4.250	4.387	.010
**BPNSF15**	3.699	3.616	3.773	.010
**BPNSF10**	3.158	3.066	3.227	.010
**BPNSF5**	3.721	3.641	3.789	.010
**BPNSF24**	5.225	5.160	5.302	.010
**BPNSF16**	5.118	5.059	5.200	.010
**BPNSF12**	5.220	5.163	5.293	.010
**BPNSF4**	5.116	5.057	5.187	.010
**BPNSF22**	3.135	3.051	3.203	.010
**BPNSF20**	2.524	2.403	2.600	.010
**BPNSF8**	2.823	2.727	2.907	.010
**BPNSF2**	2.400	2.308	2.469	.010
**BPNSF21**	5.178	5.107	5.260	.010
**BPNSF14**	5.441	5.379	5.529	.010
**BPNSF9**	5.657	5.589	5.737	.010
**BPNSF3**	5.591	5.534	5.683	.010
**BPNSF23**	2.250	2.163	2.317	.010
**BPNSF17**	2.586	2.485	2.662	.010
**BPNSF11**	2.754	2.667	2.833	.010
**BPNSF6**	2.574	2.483	2.656	.010

Covariances: (g3 - Measurement intercepts)

Parameter			Estimate	Lower	Upper	P
F1	<-->	F2	-.176	-.235	-.123	.010
F2	<-->	F3	-.156	-.206	-.112	.010
F1	<-->	F3	.783	.675	.910	.010
F2	<-->	F4	.380	.280	.497	.010
F3	<-->	F4	-.596	-.735	-.489	.010
F1	<-->	F4	-.434	-.562	-.338	.010
F2	<-->	F5	-.182	-.239	-.128	.010
F4	<-->	F5	-.687	-.809	-.585	.010
F3	<-->	F5	.839	.708	.988	.010

Parameter	Estimate	Lower	Upper	P
F1 <--> F5	.866	.714	1.016	.010
F6 <--> F5	-1.084	-1.238	-.945	.010
F6 <--> F3	-.741	-.877	-.597	.010
F6 <--> F4	1.421	1.237	1.588	.010
F6 <--> F2	.453	.335	.577	.010
F6 <--> F1	-.621	-.764	-.494	.010

Correlations: (g3 - Measurement intercepts)

Parameter	Estimate	Lower	Upper	P
F1 <--> F2	-.473	-.574	-.365	.010
F2 <--> F3	-.406	-.500	-.312	.010
F1 <--> F3	.892	.832	.948	.010
F2 <--> F4	.837	.772	.895	.010
F3 <--> F4	-.557	-.656	-.480	.010
F1 <--> F4	-.417	-.526	-.331	.010
F2 <--> F5	-.439	-.529	-.319	.010
F4 <--> F5	-.594	-.680	-.507	.010
F3 <--> F5	.858	.778	.916	.010
F1 <--> F5	.913	.836	.983	.010
F6 <--> F5	-.762	-.826	-.712	.010
F6 <--> F3	-.563	-.637	-.476	.010
F6 <--> F4	.912	.850	.958	.010
F6 <--> F2	.812	.729	.884	.010
F6 <--> F1	-.486	-.570	-.411	.010

Variances: (g3 - Measurement intercepts)

Parameter	Estimate	Lower	Upper	P
**F1**	.851	.700	1.033	.010
**F2**	.162	.092	.252	.010
**F3**	.904	.786	1.050	.010
**F4**	1.269	1.084	1.451	.010
**F5**	1.057	.885	1.268	.010
**F6**	1.915	1.664	2.173	.010
**e1**	1.176	.956	1.398	.010
**e2**	.992	.814	1.183	.010
**e3**	1.454	1.192	1.731	.010
**e4**	1.944	1.654	2.219	.010
**e5**	2.030	1.790	2.268	.010
**e6**	2.101	1.845	2.370	.010
**e7**	1.142	.883	1.396	.010
**e8**	1.704	1.446	1.966	.010
**e9**	1.055	.822	1.265	.010
**e10**	.800	.600	.990	.010
**e11**	.647	.517	.763	.010
**e12**	1.176	.960	1.482	.010
**e13**	1.345	1.120	1.538	.010
**e14**	1.377	1.057	1.693	.010
**e15**	1.261	1.007	1.515	.010
**e16**	1.703	1.372	2.092	.010
**e17**	.985	.761	1.192	.010
**e18**	1.005	.750	1.244	.010
**e19**	.931	.712	1.101	.010
**e20**	1.395	1.048	1.793	.010
**e21**	1.164	.935	1.378	.010
**e22**	1.375	1.063	1.639	.010
**e23**	1.125	.925	1.362	.010
**e24**	1.730	1.402	2.090	.010

Matrices (g3 - Measurement intercepts)

Sample Covariances (g3 - Measurement intercepts)

Sample Covariances - Lower Bounds (PC) (g3 - Measurement intercepts)

	BPNSF6	BPNSF11	BPNSF17	BPNSF23	BPNSF3	BPNSF9	BPNSF14	BPNSF21	BPNSF2	BPNSF8	BPNSF20	BPNSF22	BPNSF4	BPNSF12	BPNSF16	BPNS[illegible]
**BPNSF6**	2.741															
**BPNSF11**	1.342	2.674														
**BPNSF17**	1.302	1.484	2.920													

	BPNSF6	BPNSF11	BPNSF17	BPNSF23	BPNSF3	BPNSF9	BPNSF14	BPNSF21	BPNSF2	BPNSF8	BPNSF20	BPNSF22	BPNSF4	BPNSF12	BPNSF16	[illegible]
BPNSF23	1.275	1.528	1.661	2.755												
BPNSF3	-1.083	-1.079	-1.205	-1.282	1.804											
BPNSF9	-1.293	-1.467	-1.607	-1.391	.893	1.845										
BPNSF14	-.980	-1.195	-1.410	-1.275	.753	1.117	1.969									
BPNSF21	-.839	-1.160	-.933	-1.145	.571	.751	.818	1.654								
BPNSF2	.894	1.227	1.122	1.401	-1.146	-.928	-.951	-.654	2.898							
BPNSF8	1.159	1.513	1.360	1.362	-1.027	-1.306	-.899	-.865	1.434	2.851						
BPNSF20	.993	1.299	1.625	1.706	-1.151	-1.333	-1.074	-.967	1.341	1.505	2.817					
BPNSF22	.635	.987	.861	1.198	-.906	-.860	-.732	-.816	1.025	1.283	1.224	2.221				
BPNSF4	-.745	-.794	-.705	-.753	.824	.507	.377	.458	-.612	-.789	-.810	-.773	1.524			
BPNSF12	-.815	-.997	-1.115	-1.163	.629	.912	.984	.836	-.924	-.947	-1.094	-1.015	.722	1.826		
BPNSF16	-.728	-1.013	-1.080	-.975	.430	.672	.836	.733	-.817	-.918	-1.156	-.940	.632	1.137	1.696	
BPNSF24	-.831	-1.018	-.843	-1.042	.391	.601	.675	.789	-.907	-1.015	-1.006	-.763	.519	.900	.763	[illegible]
BPNSF5	.830	1.039	.779	.864	-.603	-.720	-.633	-.517	1.190	1.098	.797	.771	-.639	-.784	-.736	[illegible]
BPNSF10	1.012	1.468	1.207	1.133	-.882	-1.125	-.782	-.721	1.116	1.360	1.047	.885	-.706	-.766	-.742	[illegible]
BPNSF15	.424	.676	.684	.406	-.548	-.608	-.554	-.428	.560	.819	.519	.523	-.627	-.439	-.509	[illegible]
BPNSF18	-.082	.079	.155	-.094	-.271	-.010	.080	-.052	-.049	.007	.017	.161	-.295	.009	-.078	[illegible]
BPNSF1	-.629	-.678	-.641	-.604	.708	.478	.301	.389	-.440	-.686	-.675	-.834	.583	.465	.322	[illegible]
BPNSF7	-.373	-.775	-.674	-.707	.311	.517	.612	.652	-.495	-.493	-.542	-.638	.291	.630	.475	[illegible]
BPNSF13	-.698	-.902	-.905	-.729	.405	.776	.858	.681	-.643	-.759	-.808	-.870	.538	1.081	.807	[illegible]
BPNSF19	-.721	-.859	-.762	-.880	.464	.669	.695	.945	-.523	-.721	-.740	-.637	.475	.612	.649	[illegible]

Sample Covariances - Upper Bounds (PC) (g3 - Measurement intercepts)

	BPNSF6	BPNSF11	BPNSF17	BPNSF23	BPNSF3	BPNSF9	BPNSF14	BPNSF21	BPNSF2	BPNSF8	BPNSF20	BPNSF22	BPNSF4	BPNSF12	BPNSF16	[illegible]
BPNSF6	3.323															
BPNSF11	1.864	3.156														
BPNSF17	1.813	2.010	3.441													
BPNSF23	1.796	1.996	2.122	3.260												
BPNSF3	-.667	-.682	-.836	-.868	2.439											
BPNSF9	-.838	-1.065	-1.124	-.945	1.357	2.458										
BPNSF14	-.559	-.812	-.894	-.884	1.177	1.571	2.426									
BPNSF21	-.464	-.703	-.468	-.773	.974	1.142	1.225	2.085								
BPNSF2	1.433	1.722	1.665	1.867	-.712	-.538	-.503	-.290	3.519							
BPNSF8	1.668	2.010	1.907	1.840	-.549	-.798	-.480	-.435	2.002	3.409						
BPNSF20	1.524	1.790	2.155	2.210	-.758	-.850	-.623	-.528	1.912	2.039	3.413					
BPNSF22	1.124	1.403	1.360	1.642	-.464	-.405	-.282	-.416	1.452	1.757	1.712	2.691				
BPNSF4	-.361	-.388	-.311	-.429	1.288	.862	.723	.835	-.205	-.408	-.427	-.384	1.950			
BPNSF12	-.429	-.598	-.641	-.739	1.005	1.373	1.415	1.160	-.526	-.492	-.635	-.615	1.038	2.284		
BPNSF16	-.332	-.588	-.662	-.596	.821	1.106	1.227	1.091	-.455	-.432	-.678	-.542	.947	1.496	2.081	
BPNSF24	-.450	-.664	-.434	-.656	.777	.976	1.024	1.165	-.511	-.588	-.620	-.386	.854	1.240	1.097	[illegible]
BPNSF5	1.294	1.442	1.256	1.280	-.159	-.356	-.211	-.127	1.709	1.574	1.312	1.256	-.268	-.367	-.367	[illegible]
BPNSF10	1.559	1.919	1.691	1.606	-.449	-.698	-.390	-.335	1.635	1.871	1.538	1.344	-.313	-.410	-.373	[illegible]
BPNSF15	.959	1.134	1.223	.858	-.137	-.205	-.131	-.007	.998	1.297	1.016	.972	-.272	-.030	-.132	[illegible]
BPNSF18	.343	.497	.627	.305	.093	.350	.466	.271	.410	.495	.474	.564	.020	.384	.299	[illegible]
BPNSF1	-.131	-.251	-.144	-.182	1.181	.880	.742	.810	.024	-.185	-.192	-.379	.984	.867	.732	[illegible]
BPNSF7	.060	-.376	-.236	-.313	.769	.925	1.011	1.017	-.030	-.013	-.149	-.177	.662	.982	.849	[illegible]
BPNSF13	-.240	-.526	-.450	-.360	.823	1.163	1.256	1.038	-.231	-.244	-.352	-.444	.867	1.454	1.177	[illegible]
BPNSF19	-.334	-.527	-.352	-.548	.796	1.060	1.088	1.298	-.136	-.275	-.311	-.280	.788	.993	1.003	[illegible]

Sample Covariances - Two Tailed Significance (PC) (g3 - Measurement intercepts)

	BPNSF6	BPNSF11	BPNSF17	BPNSF23	BPNSF3	BPNSF9	BPNSF14	BPNSF21	BPNSF2	BPNSF8	BPNSF20	BPNSF22	BPNSF4	BPNSF12	BPNSF16	[illegible]
BPNSF6	.010															
BPNSF11	.010	.010														
BPNSF17	.010	.010	.010													
BPNSF23	.010	.010	.010	.010												
BPNSF3	.010	.010	.010	.010	.010											
BPNSF9	.010	.010	.010	.010	.010	.010										
BPNSF14	.010	.010	.010	.010	.010	.010	.010									
BPNSF21	.010	.010	.010	.010	.010	.010	.010	.010								
BPNSF2	.010	.010	.010	.010	.010	.010	.010	.010	.010							
BPNSF8	.010	.010	.010	.010	.010	.010	.010	.010	.010	.010						
BPNSF20	.010	.010	.010	.010	.010	.010	.010	.010	.010	.010	.010					
BPNSF22	.010	.010	.010	.010	.010	.010	.010	.010	.010	.010	.010	.010				
BPNSF4	.010	.010	.010	.010	.010	.010	.010	.010	.010	.010	.010	.010	.010			
BPNSF12	.010	.010	.010	.010	.010	.010	.010	.010	.010	.010	.010	.010	.010	.010		
BPNSF16	.010	.010	.010	.010	.010	.010	.010	.010	.010	.010	.010	.010	.010	.010	.010	
BPNSF24	.010	.010	.010	.010	.010	.010	.010	.010	.010	.010	.010	.010	.010	.010	.010	[illegible]
BPNSF5	.010	.010	.010	.010	.010	.010	.010	.010	.010	.010	.010	.010	.010	.010	.010	[illegible]
BPNSF10	.010	.010	.010	.010	.010	.010	.010	.010	.010	.010	.010	.010	.010	.010	.010	[illegible]

	BPNSF6	BPNSF11	BPNSF17	BPNSF23	BPNSF3	BPNSF9	BPNSF14	BPNSF21	BPNSF2	BPNSF8	BPNSF20	BPNSF22	BPNSF4	BPNSF12	BPNSF16	BPNS
**BPNSF15**	.010	.010	.010	.010	.010	.010	.016	.097	.010	.010	.010	.010	.010	.066	.014	.0
**BPNSF18**	.373	.022	.010	.386	.431	.135	.010	.336	.197	.090	.074	.012	.149	.095	.281	.0
**BPNSF1**	.019	.010	.010	.010	.010	.010	.010	.010	.175	.010	.010	.010	.010	.010	.010	.0
**BPNSF7**	.178	.010	.010	.010	.010	.010	.010	.010	.062	.094	.010	.010	.010	.010	.010	.0
**BPNSF13**	.010	.010	.010	.010	.010	.010	.010	.010	.010	.010	.010	.010	.010	.010	.010	.0
**BPNSF19**	.010	.010	.010	.010	.010	.010	.010	.010	.016	.010	.010	.010	.010	.010	.010	.0

Sample Correlations (g3 - Measurement intercepts)

Sample Correlations - Lower Bounds (PC) (g3 - Measurement intercepts)

	BPNSF6	BPNSF11	BPNSF17	BPNSF23	BPNSF3	BPNSF9	BPNSF14	BPNSF21	BPNSF2	BPNSF8	BPNSF20	BPNSF22	BPNSF4	BPNSF12	BPNSF16	BPNS
**BPNSF6**	1.000															
**BPNSF11**	.468	1.000														
**BPNSF17**	.419	.488	1.000													
**BPNSF23**	.425	.543	.550	1.000												
**BPNSF3**	-.426	-.445	-.482	-.508	1.000											
**BPNSF9**	-.501	-.576	-.601	-.533	.424	1.000										
**BPNSF14**	-.381	-.472	-.526	-.499	.352	.546	1.000									
**BPNSF21**	-.352	-.477	-.371	-.484	.283	.389	.400	1.000								
**BPNSF2**	.289	.410	.347	.462	-.439	-.360	-.359	-.278	1.000							
**BPNSF8**	.386	.506	.435	.443	-.386	-.482	-.349	-.349	.466	1.000						
**BPNSF20**	.335	.440	.532	.576	-.445	-.498	-.401	-.390	.441	.496	1.000					
**BPNSF22**	.235	.384	.312	.444	-.380	-.366	-.320	-.374	.372	.483	.454	1.000				
**BPNSF4**	-.327	-.353	-.303	-.327	.445	.267	.191	.254	-.268	-.346	-.352	-.376	1.000			
**BPNSF12**	-.326	-.409	-.421	-.465	.305	.464	.476	.434	-.366	-.382	-.423	-.450	.377	1.000		
**BPNSF16**	-.305	-.423	-.438	-.403	.211	.332	.420	.399	-.329	-.376	-.462	-.430	.355	.591	1.000	
**BPNSF24**	-.342	-.437	-.344	-.430	.195	.311	.335	.447	-.374	-.418	-.410	-.360	.278	.473	.410	1.0
**BPNSF5**	.285	.374	.265	.302	-.244	-.286	-.247	-.221	.403	.377	.273	.305	-.287	-.308	-.320	-.2
**BPNSF10**	.357	.538	.409	.407	-.372	-.453	-.320	-.310	.382	.473	.368	.349	-.330	-.322	-.318	-.2
**BPNSF15**	.143	.240	.235	.141	-.217	-.239	-.222	-.191	.185	.281	.179	.204	-.280	-.187	-.221	-.2
**BPNSF18**	-.030	.031	.057	-.036	-.120	-.005	.040	-.025	-.019	.003	.007	.072	-.156	.004	-.039	.0
**BPNSF1**	-.222	-.258	-.233	-.223	.327	.205	.133	.177	-.160	-.248	-.243	-.331	.293	.211	.151	.1
**BPNSF7**	-.151	-.314	-.264	-.275	.140	.251	.281	.332	-.191	-.193	-.219	-.278	.153	.310	.248	.1
**BPNSF13**	-.279	-.383	-.348	-.298	.189	.382	.415	.360	-.253	-.305	-.319	-.377	.295	.559	.431	.3
**BPNSF19**	-.292	-.361	-.307	-.363	.227	.332	.339	.517	-.210	-.289	-.300	-.289	.272	.313	.348	.3

Sample Correlations - Upper Bounds (PC) (g3 - Measurement intercepts)

	BPNSF6	BPNSF11	BPNSF17	BPNSF23	BPNSF3	BPNSF9	BPNSF14	BPNSF21	BPNSF2	BPNSF8	BPNSF20	BPNSF22	BPNSF4	BPNSF12	BPNSF16	BPNS
**BPNSF6**	1.000															
**BPNSF11**	.627	1.000														
**BPNSF17**	.579	.644	1.000													
**BPNSF23**	.577	.652	.680	1.000												
**BPNSF3**	-.258	-.268	-.304	-.333	1.000											
**BPNSF9**	-.333	-.438	-.443	-.380	.613	1.000										
**BPNSF14**	-.221	-.321	-.341	-.346	.529	.682	1.000									
**BPNSF21**	-.189	-.314	-.192	-.332	.475	.562	.595	1.000								
**BPNSF2**	.452	.552	.514	.602	-.263	-.201	-.190	-.116	1.000							
**BPNSF8**	.525	.645	.582	.584	-.213	-.324	-.184	-.177	.617	1.000						
**BPNSF20**	.495	.583	.661	.706	-.279	-.337	-.242	-.208	.590	.640	1.000					
**BPNSF22**	.410	.519	.484	.584	-.205	-.174	-.125	-.195	.506	.617	.601	1.000				
**BPNSF4**	-.156	-.175	-.130	-.184	.626	.442	.370	.450	-.083	-.167	-.175	-.188	1.000			
**BPNSF12**	-.175	-.249	-.254	-.299	.470	.603	.630	.569	-.202	-.198	-.251	-.284	.537	1.000		
**BPNSF16**	-.132	-.262	-.271	-.255	.413	.531	.575	.576	-.173	-.174	-.284	-.256	.522	.721	1.000	
**BPNSF24**	-.192	-.290	-.179	-.283	.387	.484	.498	.594	-.205	-.241	-.256	-.173	.475	.613	.572	1.0
**BPNSF5**	.435	.497	.409	.428	-.065	-.138	-.084	-.051	.550	.511	.431	.465	-.116	-.155	-.166	-.1
**BPNSF10**	.541	.658	.567	.542	-.190	-.296	-.159	-.145	.541	.616	.517	.508	-.146	-.167	-.163	-.1
**BPNSF15**	.332	.382	.405	.299	-.058	-.085	-.052	-.003	.329	.436	.339	.368	-.127	-.012	-.057	-.0
**BPNSF18**	.132	.194	.231	.116	.042	.160	.214	.138	.152	.186	.180	.243	.011	.185	.145	.2
**BPNSF1**	-.047	-.095	-.051	-.067	.497	.378	.315	.377	.008	-.066	-.071	-.154	.468	.384	.337	.3
**BPNSF7**	.024	-.152	-.089	-.121	.364	.442	.468	.501	-.013	-.005	-.061	-.083	.346	.473	.429	.3
**BPNSF13**	-.094	-.214	-.183	-.137	.387	.534	.587	.521	-.090	-.096	-.136	-.205	.457	.678	.584	.5
**BPNSF19**	-.139	-.222	-.145	-.225	.393	.494	.522	.639	-.058	-.117	-.124	-.126	.421	.486	.502	.4

Sample Correlations - Two Tailed Significance (PC) (g3 - Measurement intercepts)

	BPNSF6	BPNSF11	BPNSF17	BPNSF23	BPNSF3	BPNSF9	BPNSF14	BPNSF21	BPNSF2	BPNSF8	BPNSF20	BPNSF22	BPNSF4	BPNSF12	BPNSF16	BPNS
**BPNSF6**	...															
**BPNSF11**	.010	...														

	BPNSF6	BPNSF11	BPNSF17	BPNSF23	BPNSF3	BPNSF9	BPNSF14	BPNSF21	BPNSF2	BPNSF8	BPNSF20	BPNSF22	BPNSF4	BPNSF12	BPNSF16	BPNS
**BPNSF17**	.010	.010	...													
**BPNSF23**	.010	.010	.010	...												
**BPNSF3**	.010	.010	.010	.010	...											
**BPNSF9**	.010	.010	.010	.010	.010	...										
**BPNSF14**	.010	.010	.010	.010	.010	.010	...									
**BPNSF21**	.010	.010	.010	.010	.010	.010	.010	...								
**BPNSF2**	.010	.010	.010	.010	.010	.010	.010	.010	...							
**BPNSF8**	.010	.010	.010	.010	.010	.010	.010	.010	.010	...						
**BPNSF20**	.010	.010	.010	.010	.010	.010	.010	.010	.010	.010	...					
**BPNSF22**	.010	.010	.010	.010	.010	.010	.010	.010	.010	.010	.010	...				
**BPNSF4**	.010	.010	.010	.010	.010	.010	.010	.010	.010	.010	.010	.010	...			
**BPNSF12**	.010	.010	.010	.010	.010	.010	.010	.010	.010	.010	.010	.010	.010	...		
**BPNSF16**	.010	.010	.010	.010	.010	.010	.010	.010	.010	.010	.010	.010	.010	.010	...	
**BPNSF24**	.010	.010	.010	.010	.010	.010	.010	.010	.010	.010	.010	.010	.010	.010	.010	
**BPNSF5**	.010	.010	.010	.010	.010	.010	.010	.010	.010	.010	.010	.010	.010	.010	.010	.0
**BPNSF10**	.010	.010	.010	.010	.010	.010	.010	.010	.010	.010	.010	.010	.010	.010	.010	.0
**BPNSF15**	.010	.010	.010	.010	.010	.010	.016	.097	.010	.010	.010	.010	.010	.066	.014	.0
**BPNSF18**	.373	.022	.010	.387	.431	.135	.010	.336	.197	.090	.074	.012	.149	.095	.281	.0
**BPNSF1**	.019	.010	.010	.010	.010	.010	.010	.010	.175	.010	.010	.010	.010	.010	.010	.0
**BPNSF7**	.178	.010	.010	.010	.010	.010	.010	.010	.062	.094	.010	.010	.010	.010	.010	.0
**BPNSF13**	.010	.010	.010	.010	.010	.010	.010	.010	.010	.010	.010	.010	.010	.010	.010	.0
**BPNSF19**	.010	.010	.010	.010	.010	.010	.010	.010	.016	.010	.010	.010	.010	.010	.010	.0

Sample Means (g3 - Measurement intercepts)

Sample Means - Lower Bounds (PC) (g3 - Measurement intercepts)

	BPNSF6	BPNSF11	BPNSF17	BPNSF23	BPNSF3	BPNSF9	BPNSF14	BPNSF21	BPNSF2	BPNSF8	BPNSF20	BPNSF22	BPNSF4	BPNSF12	BPNSF16	BPNSF
**BPNSF6**	2.512	2.681	2.684	2.387	5.371	5.333	5.052	4.893	2.510	2.777	2.612	3.146	5.091	4.998	4.900	5.00

Sample Means - Upper Bounds (PC) (g3 - Measurement intercepts)

	BPNSF6	BPNSF11	BPNSF17	BPNSF23	BPNSF3	BPNSF9	BPNSF14	BPNSF21	BPNSF2	BPNSF8	BPNSF20	BPNSF22	BPNSF4	BPNSF12	BPNSF16	BPNSF
**BPNSF6**	2.774	2.931	2.973	2.629	5.613	5.584	5.294	5.097	2.809	3.057	2.898	3.394	5.299	5.234	5.137	5.23

Sample Means - Two Tailed Significance (PC) (g3 - Measurement intercepts)

	BPNSF6	BPNSF11	BPNSF17	BPNSF23	BPNSF3	BPNSF9	BPNSF14	BPNSF21	BPNSF2	BPNSF8	BPNSF20	BPNSF22	BPNSF4	BPNSF12	BPNSF16	BPNSF
**BPNSF6**	.010	.010	.010	.010	.010	.010	.010	.010	.010	.010	.010	.010	.010	.010	.010	.01

Bias-corrected percentile method (g3 - Measurement intercepts)

90% confidence intervals (bias-corrected percentile method)

Scalar Estimates (g3 - Measurement intercepts)

Regression Weights: (g3 - Measurement intercepts)

Parameter			Estimate	Lower	Upper	P
BPNSF19	<---	F1	1.000	1.000	1.000	...
BPNSF13	<---	F1	1.144	1.053	1.252	.004
BPNSF7	<---	F1	.863	.764	.963	.010
BPNSF1	<---	F1	.759	.643	.876	.012
BPNSF18	<---	F2	1.000	1.000	1.000	...
BPNSF15	<---	F2	2.454	2.008	3.361	.006
BPNSF10	<---	F2	3.105	2.512	4.256	.008
BPNSF5	<---	F2	2.516	2.030	3.412	.008
BPNSF24	<---	F3	1.000	1.000	1.000	...
BPNSF16	<---	F3	1.124	1.040	1.219	.009
BPNSF12	<---	F3	1.221	1.151	1.306	.004
BPNSF4	<---	F3	.846	.746	.934	.012
BPNSF22	<---	F4	1.000	1.000	1.000	...
BPNSF20	<---	F4	1.192	1.118	1.295	.005
BPNSF8	<---	F4	1.197	1.120	1.292	.005
BPNSF2	<---	F4	1.120	1.043	1.226	.005
BPNSF21	<---	F5	1.000	1.000	1.000	...
BPNSF14	<---	F5	1.094	1.023	1.179	.009
BPNSF9	<---	F5	1.038	.967	1.135	.007
BPNSF3	<---	F5	.882	.787	.982	.015

Parameter			Estimate	Lower	Upper	P
BPNSF23	<---	F6	1.000	1.000	1.000	...
BPNSF17	<---	F6	.976	.914	1.026	.021
BPNSF11	<---	F6	.959	.910	1.027	.005
BPNSF6	<---	F6	.861	.801	.930	.010

Standardized Regression Weights: (g3 - Measurement intercepts)

Parameter			Estimate	Lower	Upper	P
BPNSF19	<---	F1	.648	.578	.696	.021
BPNSF13	<---	F1	.727	.683	.774	.005
BPNSF7	<---	F1	.551	.487	.609	.012
BPNSF1	<---	F1	.449	.389	.514	.009
BPNSF18	<---	F2	.272	.190	.331	.019
BPNSF15	<---	F2	.563	.509	.607	.013
BPNSF10	<---	F2	.760	.706	.821	.007
BPNSF5	<---	F2	.613	.564	.687	.005
BPNSF24	<---	F3	.679	.640	.738	.007
BPNSF16	<---	F3	.767	.704	.810	.021
BPNSF12	<---	F3	.822	.784	.856	.012
BPNSF4	<---	F3	.596	.543	.664	.005
BPNSF22	<---	F4	.697	.650	.739	.016
BPNSF20	<---	F4	.753	.701	.804	.012
BPNSF8	<---	F4	.768	.719	.820	.007
BPNSF2	<---	F4	.695	.640	.745	.009
BPNSF21	<---	F5	.719	.651	.760	.046
BPNSF14	<---	F5	.747	.675	.798	.026
BPNSF9	<---	F5	.742	.691	.797	.011
BPNSF3	<---	F5	.609	.529	.675	.021
BPNSF23	<---	F6	.789	.756	.833	.008
BPNSF17	<---	F6	.755	.707	.799	.018
BPNSF11	<---	F6	.781	.733	.821	.010
BPNSF6	<---	F6	.671	.620	.731	.008

Intercepts: (g3 - Measurement intercepts)

Parameter	Estimate	Lower	Upper	P
**BPNSF19**	5.140	5.046	5.203	.028
**BPNSF13**	5.043	4.967	5.102	.034
**BPNSF7**	4.817	4.726	4.873	.044
**BPNSF1**	4.705	4.630	4.766	.030
**BPNSF18**	4.316	4.252	4.390	.009
**BPNSF15**	3.699	3.606	3.770	.012
**BPNSF10**	3.158	3.068	3.229	.009
**BPNSF5**	3.721	3.650	3.798	.006
**BPNSF24**	5.225	5.135	5.281	.034
**BPNSF16**	5.118	5.049	5.195	.019
**BPNSF12**	5.220	5.151	5.275	.034
**BPNSF4**	5.116	5.041	5.175	.026
**BPNSF22**	3.135	3.062	3.213	.004
**BPNSF20**	2.524	2.445	2.626	.003
**BPNSF8**	2.823	2.738	2.909	.005
**BPNSF2**	2.400	2.330	2.498	.003
**BPNSF21**	5.178	5.054	5.218	.095
**BPNSF14**	5.441	5.353	5.498	.053
**BPNSF9**	5.657	5.564	5.726	.032
**BPNSF3**	5.591	5.530	5.666	.018
**BPNSF23**	2.250	2.189	2.341	.002
**BPNSF17**	2.586	2.504	2.680	.003
**BPNSF11**	2.754	2.681	2.846	.004
**BPNSF6**	2.574	2.487	2.664	.007

Covariances: (g3 - Measurement intercepts)

Parameter			Estimate	Lower	Upper	P
F1	<-->	F2	-.176	-.243	-.132	.005
F2	<-->	F3	-.156	-.213	-.116	.006
F1	<-->	F3	.783	.676	.910	.009
F2	<-->	F4	.380	.284	.504	.008
F3	<-->	F4	-.596	-.730	-.488	.012
F1	<-->	F4	-.434	-.551	-.316	.015

Parameter	Estimate	Lower	Upper	P
F2 <--> F5	-.182	-.257	-.142	.003
F4 <--> F5	-.687	-.812	-.590	.009
F3 <--> F5	.839	.731	1.005	.006
F1 <--> F5	.866	.709	1.011	.012
F6 <--> F5	-1.084	-1.234	-.939	.012
F6 <--> F3	-.741	-.864	-.581	.015
F6 <--> F4	1.421	1.227	1.578	.015
F6 <--> F2	.453	.335	.577	.010
F6 <--> F1	-.621	-.735	-.458	.023

Correlations: (g3 - Measurement intercepts)

Parameter	Estimate	Lower	Upper	P
F1 <--> F2	-.473	-.574	-.366	.009
F2 <--> F3	-.406	-.500	-.312	.010
F1 <--> F3	.892	.833	.952	.005
F2 <--> F4	.837	.774	.907	.006
F3 <--> F4	-.557	-.651	-.459	.014
F1 <--> F4	-.417	-.512	-.319	.016
F2 <--> F5	-.439	-.531	-.321	.009
F4 <--> F5	-.594	-.681	-.508	.009
F3 <--> F5	.858	.792	.928	.006
F1 <--> F5	.913	.837	.984	.009
F6 <--> F5	-.762	-.826	-.712	.010
F6 <--> F3	-.563	-.635	-.471	.012
F6 <--> F4	.912	.857	.960	.006
F6 <--> F2	.812	.731	.886	.008
F6 <--> F1	-.486	-.565	-.399	.019

Variances: (g3 - Measurement intercepts)

Parameter	Estimate	Lower	Upper	P
**F1**	.851	.688	1.005	.019
**F2**	.162	.088	.245	.014
**F3**	.904	.795	1.059	.005
**F4**	1.269	1.078	1.430	.016
**F5**	1.057	.859	1.224	.020
**F6**	1.915	1.633	2.152	.016
**e1**	1.176	.954	1.395	.012
**e2**	.992	.805	1.173	.013
**e3**	1.454	1.201	1.778	.006
**e4**	1.944	1.688	2.234	.007
**e5**	2.030	1.808	2.301	.004
**e6**	2.101	1.857	2.374	.007
**e7**	1.142	.882	1.394	.011
**e8**	1.704	1.458	1.976	.006
**e9**	1.055	.839	1.274	.008
**e10**	.800	.627	1.014	.005
**e11**	.647	.530	.791	.006
**e12**	1.176	.970	1.506	.007
**e13**	1.345	1.136	1.559	.007
**e14**	1.377	1.084	1.716	.005
**e15**	1.261	.975	1.486	.020
**e16**	1.703	1.359	2.087	.012
**e17**	.985	.793	1.228	.004
**e18**	1.005	.778	1.288	.006
**e19**	.931	.717	1.110	.007
**e20**	1.395	1.085	1.813	.006
**e21**	1.164	.930	1.376	.011
**e22**	1.375	1.062	1.637	.011
**e23**	1.125	.909	1.349	.012
**e24**	1.730	1.316	2.079	.015

Matrices (g3 - Measurement intercepts)

Sample Covariances (g3 - Measurement intercepts)

Sample Covariances - Lower Bounds (BC) (g3 - Measurement intercepts)

	BPNSF6	BPNSF11	BPNSF17	BPNSF23	BPNSF3	BPNSF9	BPNSF14	BPNSF21	BPNSF2	BPNSF8	BPNSF20	BPNSF22	BPNSF4	BPNSF12	BPNSF16	BPNS[illegible]

	BPNSF6	BPNSF11	BPNSF17	BPNSF23	BPNSF3	BPNSF9	BPNSF14	BPNSF21	BPNSF2	BPNSF8	BPNSF20	BPNSF22	BPNSF4	BPNSF12	BPNSF16	BPNS[illegible]
**BPNSF6**	2.719															
**BPNSF11**	1.383	2.681														
**BPNSF17**	1.316	1.451	2.933													
**BPNSF23**	1.313	1.523	1.661	2.755												
**BPNSF3**	-1.124	-1.094	-1.233	-1.263	1.765											
**BPNSF9**	-1.290	-1.469	-1.622	-1.362	.893	1.844										
**BPNSF14**	-.942	-1.200	-1.418	-1.268	.773	1.118	1.983									
**BPNSF21**	-.844	-1.167	-.933	-1.143	.570	.729	.799	1.700								
**BPNSF2**	.910	1.248	1.130	1.396	-1.194	-.935	-.953	-.664	2.935							
**BPNSF8**	1.220	1.534	1.364	1.411	-1.051	-1.306	-.914	-.937	1.434	2.883						
**BPNSF20**	1.017	1.296	1.638	1.695	-1.137	-1.327	-1.089	-.926	1.341	1.506	2.845					
**BPNSF22**	.664	.991	.860	1.236	-.886	-.906	-.765	-.858	1.036	1.306	1.225	2.252				
**BPNSF4**	-.757	-.749	-.708	-.790	.816	.524	.377	.463	-.625	-.837	-.830	-.774	1.514			
**BPNSF12**	-.914	-1.017	-1.125	-1.185	.642	.973	1.027	.858	-.964	-.966	-1.085	-1.027	.727	1.900		
**BPNSF16**	-.728	-1.013	-1.073	-.986	.487	.683	.884	.736	-.822	-.922	-1.167	-.963	.633	1.153	1.739	
**BPNSF24**	-.852	-1.004	-.821	-1.042	.370	.608	.663	.803	-.899	-1.030	-.977	-.780	.523	.924	.778	1.6[illegible]
**BPNSF5**	.815	1.065	.784	.877	-.591	-.749	-.683	-.517	1.226	1.090	.832	.743	-.674	-.794	-.769	-.7[illegible]
**BPNSF10**	1.019	1.503	1.221	1.176	-.882	-1.131	-.766	-.703	1.116	1.416	1.067	.908	-.696	-.768	-.719	-.6[illegible]
**BPNSF15**	.453	.685	.732	.448	-.553	-.624	-.584	-.549	.590	.795	.524	.525	-.629	-.492	-.539	-.4[illegible]
**BPNSF18**	-.060	.114	.218	-.054	-.319	-.012	.079	-.102	-.017	-.004	.067	.177	-.295	-.020	-.110	.0[illegible]
**BPNSF1**	-.568	-.643	-.672	-.604	.708	.509	.305	.380	-.484	-.774	-.616	-.854	.593	.473	.336	.3[illegible]
**BPNSF7**	-.367	-.741	-.668	-.713	.302	.515	.611	.637	-.498	-.493	-.535	-.670	.290	.659	.473	.3[illegible]
**BPNSF13**	-.697	-.911	-.932	-.742	.420	.811	.868	.747	-.648	-.788	-.808	-.897	.574	1.109	.843	.6[illegible]
**BPNSF19**	-.661	-.859	-.740	-.872	.462	.670	.709	.966	-.490	-.696	-.707	-.640	.465	.612	.701	.5[illegible]

Sample Covariances - Upper Bounds (BC) (g3 - Measurement intercepts)

	BPNSF6	BPNSF11	BPNSF17	BPNSF23	BPNSF3	BPNSF9	BPNSF14	BPNSF21	BPNSF2	BPNSF8	BPNSF20	BPNSF22	BPNSF4	BPNSF12	BPNSF16	BPNS[illegible]
**BPNSF6**	3.289															
**BPNSF11**	1.900	3.183														
**BPNSF17**	1.833	2.009	3.462													
**BPNSF23**	1.818	1.990	2.131	3.261												
**BPNSF3**	-.688	-.683	-.837	-.827	2.431											
**BPNSF9**	-.838	-1.073	-1.150	-.901	1.357	2.456										
**BPNSF14**	-.540	-.821	-.930	-.874	1.188	1.577	2.454									
**BPNSF21**	-.472	-.705	-.488	-.769	.973	1.117	1.182	2.181								
**BPNSF2**	1.453	1.750	1.679	1.864	-.747	-.543	-.505	-.317	3.531							
**BPNSF8**	1.711	2.046	1.914	1.878	-.589	-.805	-.498	-.473	2.002	3.426						
**BPNSF20**	1.557	1.781	2.156	2.208	-.706	-.849	-.636	-.471	1.912	2.042	3.446					
**BPNSF22**	1.150	1.440	1.360	1.718	-.460	-.433	-.316	-.466	1.513	1.778	1.716	2.720				
**BPNSF4**	-.385	-.350	-.315	-.435	1.278	.886	.723	.839	-.218	-.446	-.443	-.388	1.945			
**BPNSF12**	-.442	-.612	-.659	-.759	1.043	1.409	1.440	1.200	-.565	-.505	-.629	-.637	1.054	2.368		
**BPNSF16**	-.334	-.588	-.641	-.600	.867	1.113	1.258	1.110	-.455	-.444	-.682	-.559	.949	1.531	2.147	
**BPNSF24**	-.480	-.647	-.410	-.656	.768	.994	1.017	1.174	-.509	-.635	-.549	-.405	.872	1.259	1.107	2.0[illegible]
**BPNSF5**	1.292	1.503	1.258	1.290	-.148	-.372	-.251	-.127	1.716	1.564	1.355	1.232	-.279	-.373	-.414	-.3[illegible]
**BPNSF10**	1.570	1.985	1.697	1.614	-.449	-.705	-.369	-.330	1.635	1.918	1.557	1.354	-.311	-.411	-.333	-.3[illegible]
**BPNSF15**	1.001	1.166	1.270	.918	-.153	-.228	-.176	-.054	1.037	1.283	1.029	.973	-.275	-.091	-.155	-.1[illegible]
**BPNSF18**	.379	.516	.679	.353	.054	.344	.459	.233	.440	.478	.512	.573	.020	.370	.256	.4[illegible]
**BPNSF1**	-.123	-.224	-.175	-.182	1.181	.923	.760	.804	.013	-.225	-.161	-.385	.989	.879	.759	.7[illegible]
**BPNSF7**	.076	-.339	-.227	-.322	.763	.924	1.000	1.010	-.038	-.014	-.143	-.201	.652	1.013	.849	.6[illegible]
**BPNSF13**	-.233	-.528	-.497	-.370	.840	1.196	1.267	1.079	-.232	-.308	-.354	-.464	.891	1.468	1.202	1.0[illegible]
**BPNSF19**	-.277	-.527	-.317	-.540	.794	1.061	1.091	1.314	-.112	-.267	-.263	-.291	.783	.993	1.018	.9[illegible]

Sample Covariances - Two Tailed Significance (BC) (g3 - Measurement intercepts)

	BPNSF6	BPNSF11	BPNSF17	BPNSF23	BPNSF3	BPNSF9	BPNSF14	BPNSF21	BPNSF2	BPNSF8	BPNSF20	BPNSF22	BPNSF4	BPNSF12	BPNSF16	BPNS[illegible]
**BPNSF6**	.014															
**BPNSF11**	.007	.007														
**BPNSF17**	.006	.012	.007													
**BPNSF23**	.005	.012	.009	.009												
**BPNSF3**	.006	.009	.008	.019	.015											
**BPNSF9**	.011	.009	.005	.021	.010	.012										
**BPNSF14**	.023	.009	.007	.015	.007	.009	.006									
**BPNSF21**	.008	.009	.009	.011	.012	.025	.015	.004								
**BPNSF2**	.008	.006	.008	.012	.006	.007	.009	.006	.006							
**BPNSF8**	.003	.005	.009	.002	.005	.009	.006	.004	.010	.007						
**BPNSF20**	.005	.012	.008	.012	.018	.011	.008	.020	.010	.009	.005					
**BPNSF22**	.007	.009	.011	.003	.012	.005	.005	.003	.006	.007	.009	.004				
**BPNSF4**	.008	.025	.008	.006	.011	.005	.010	.009	.007	.004	.005	.009	.012			
**BPNSF12**	.003	.006	.008	.006	.005	.003	.004	.004	.005	.007	.012	.006	.007	.002		
**BPNSF16**	.009	.010	.012	.008	.004	.009	.002	.007	.009	.009	.009	.007	.009	.004	.004	

	BPNSF6	BPNSF11	BPNSF17	BPNSF23	BPNSF3	BPNSF9	BPNSF14	BPNSF21	BPNSF2	BPNSF8	BPNSF20	BPNSF22	BPNSF4	BPNSF12	BPNSF16	BPNS[illegible]
**BPNSF24**	.005	.013	.019	.010	.015	.008	.012	.007	.011	.005	.034	.006	.006	.005	.006	[illegible]
**BPNSF5**	.011	.005	.009	.006	.012	.005	.004	.010	.005	.012	.005	.018	.006	.007	.005	[illegible]
**BPNSF10**	.008	.005	.009	.009	.010	.009	.016	.012	.010	.003	.006	.009	.012	.009	.015	[illegible]
**BPNSF15**	.006	.006	.004	.004	.007	.005	.005	.039	.005	.013	.008	.009	.009	.032	.006	[illegible]
**BPNSF18**	.321	.012	.004	.233	.223	.156	.012	.595	.150	.105	.042	.008	.149	.149	.468	[illegible]
**BPNSF1**	.034	.016	.008	.010	.010	.004	.007	.014	.125	.005	.025	.005	.007	.007	.006	[illegible]
**BPNSF7**	.222	.026	.014	.008	.012	.011	.011	.012	.049	.089	.015	.006	.012	.003	.011	[illegible]
**BPNSF13**	.011	.009	.005	.006	.004	.004	.007	.003	.009	.005	.009	.005	.004	.004	.005	[illegible]
**BPNSF19**	.028	.010	.019	.016	.012	.009	.007	.006	.035	.013	.023	.006	.012	.010	.005	[illegible]

Sample Correlations (g3 - Measurement intercepts)

Sample Correlations - Lower Bounds (BC) (g3 - Measurement intercepts)

	BPNSF6	BPNSF11	BPNSF17	BPNSF23	BPNSF3	BPNSF9	BPNSF14	BPNSF21	BPNSF2	BPNSF8	BPNSF20	BPNSF22	BPNSF4	BPNSF12	BPNSF16	BPNS[illegible]
**BPNSF6**	1.000															
**BPNSF11**	.477	1.000														
**BPNSF17**	.403	.492	1.000													
**BPNSF23**	.445	.543	.545	1.000												
**BPNSF3**	-.434	-.445	-.483	-.505	1.000											
**BPNSF9**	-.488	-.567	-.579	-.529	.398	1.000										
**BPNSF14**	-.366	-.467	-.523	-.485	.342	.546	1.000									
**BPNSF21**	-.346	-.469	-.371	-.481	.272	.364	.374	1.000								
**BPNSF2**	.292	.412	.343	.470	-.431	-.350	-.357	-.285	1.000							
**BPNSF8**	.400	.509	.443	.474	-.392	-.485	-.352	-.373	.463	1.000						
**BPNSF20**	.340	.439	.529	.561	-.431	-.489	-.401	-.368	.441	.496	1.000					
**BPNSF22**	.237	.393	.312	.447	-.376	-.367	-.340	-.387	.372	.486	.454	1.000				
**BPNSF4**	-.327	-.338	-.303	-.343	.436	.270	.200	.256	-.269	-.352	-.355	-.378	1.000			
**BPNSF12**	-.327	-.410	-.424	-.466	.308	.464	.477	.436	-.361	-.382	-.404	-.459	.377	1.000		
**BPNSF16**	-.304	-.421	-.423	-.408	.224	.330	.425	.396	-.331	-.360	-.454	-.438	.356	.593	1.000	
**BPNSF24**	-.355	-.429	-.342	-.429	.180	.314	.323	.446	-.370	-.423	-.397	-.366	.292	.474	.408	[illegible]
**BPNSF5**	.279	.381	.258	.306	-.239	-.295	-.265	-.227	.401	.375	.279	.298	-.287	-.308	-.326	[illegible]
**BPNSF10**	.352	.538	.408	.407	-.346	-.454	-.305	-.307	.382	.483	.369	.349	-.324	-.321	-.317	[illegible]
**BPNSF15**	.148	.240	.248	.154	-.221	-.240	-.231	-.232	.195	.284	.181	.202	-.279	-.195	-.225	[illegible]
**BPNSF18**	-.025	.046	.083	-.028	-.137	-.012	.030	-.055	-.005	-.004	.011	.074	-.155	-.013	-.053	[illegible]
**BPNSF1**	-.206	-.243	-.233	-.219	.332	.205	.134	.171	-.166	-.267	-.220	-.343	.296	.214	.150	[illegible]
**BPNSF7**	-.149	-.307	-.252	-.274	.121	.240	.284	.313	-.193	-.201	-.218	-.291	.150	.316	.231	[illegible]
**BPNSF13**	-.282	-.386	-.350	-.315	.207	.389	.407	.367	-.254	-.310	-.305	-.388	.309	.563	.434	[illegible]
**BPNSF19**	-.280	-.357	-.295	-.359	.219	.332	.329	.517	-.200	-.285	-.288	-.303	.272	.309	.337	[illegible]

Sample Correlations - Upper Bounds (BC) (g3 - Measurement intercepts)

	BPNSF6	BPNSF11	BPNSF17	BPNSF23	BPNSF3	BPNSF9	BPNSF14	BPNSF21	BPNSF2	BPNSF8	BPNSF20	BPNSF22	BPNSF4	BPNSF12	BPNSF16	BPNS[illegible]
**BPNSF6**	1.000															
**BPNSF11**	.629	1.000														
**BPNSF17**	.569	.647	1.000													
**BPNSF23**	.592	.652	.674	1.000												
**BPNSF3**	-.258	-.270	-.308	-.324	1.000											
**BPNSF9**	-.322	-.433	-.432	-.375	.596	1.000										
**BPNSF14**	-.205	-.316	-.340	-.343	.518	.686	1.000									
**BPNSF21**	-.182	-.306	-.192	-.331	.460	.553	.561	1.000								
**BPNSF2**	.458	.559	.512	.605	-.252	-.195	-.186	-.121	1.000							
**BPNSF8**	.544	.645	.583	.613	-.224	-.324	-.185	-.185	.615	1.000						
**BPNSF20**	.498	.580	.651	.695	-.262	-.324	-.242	-.176	.593	.642	1.000					
**BPNSF22**	.413	.530	.484	.588	-.204	-.174	-.140	-.208	.506	.621	.601	1.000				
**BPNSF4**	-.158	-.158	-.130	-.192	.616	.444	.378	.454	-.086	-.184	-.184	-.188	1.000			
**BPNSF12**	-.177	-.249	-.256	-.299	.473	.601	.631	.571	-.198	-.198	-.245	-.292	.537	1.000		
**BPNSF16**	-.132	-.259	-.255	-.257	.418	.529	.582	.569	-.176	-.172	-.282	-.262	.523	.722	1.000	
**BPNSF24**	-.208	-.278	-.168	-.281	.372	.487	.492	.593	-.203	-.256	-.228	-.188	.488	.615	.569	[illegible]
**BPNSF5**	.433	.509	.398	.436	-.058	-.147	-.102	-.054	.544	.509	.440	.460	-.116	-.156	-.173	[illegible]
**BPNSF10**	.537	.658	.561	.541	-.158	-.296	-.135	-.131	.533	.619	.518	.508	-.135	-.167	-.158	[illegible]
**BPNSF15**	.337	.382	.416	.316	-.059	-.085	-.073	-.023	.344	.436	.353	.357	-.123	-.037	-.061	[illegible]
**BPNSF18**	.136	.210	.244	.131	.029	.152	.212	.113	.175	.179	.188	.244	.012	.165	.127	[illegible]
**BPNSF1**	-.037	-.086	-.051	-.066	.501	.378	.325	.366	.006	-.076	-.056	-.162	.470	.390	.337	[illegible]
**BPNSF7**	.029	-.139	-.082	-.120	.353	.433	.469	.480	-.015	-.006	-.057	-.086	.344	.482	.424	[illegible]
**BPNSF13**	-.100	-.216	-.187	-.147	.401	.543	.579	.527	-.091	-.110	-.117	-.210	.459	.683	.593	[illegible]
**BPNSF19**	-.114	-.220	-.120	-.214	.384	.493	.510	.639	-.044	-.109	-.098	-.137	.421	.474	.497	[illegible]

Sample Correlations - Two Tailed Significance (BC) (g3 - Measurement intercepts)

	BPNSF6	BPNSF11	BPNSF17	BPNSF23	BPNSF3	BPNSF9	BPNSF14	BPNSF21	BPNSF2	BPNSF8	BPNSF20	BPNSF22	BPNSF4	BPNSF12	BPNSF16	BPNS[illegible]
**BPNSF6**	...															
**BPNSF11**	.007	...														
**BPNSF17**	.018	.009	...													
**BPNSF23**	.006	.010	.014	...												
**BPNSF3**	.009	.009	.007	.015	...											
**BPNSF9**	.018	.014	.019	.016	.023	...										
**BPNSF14**	.025	.012	.012	.020	.016	.009	...									
**BPNSF21**	.015	.018	.010	.013	.019	.021	.025	...								
**BPNSF2**	.007	.007	.014	.007	.013	.014	.012	.007	...							
**BPNSF8**	.003	.009	.008	.002	.006	.009	.009	.006	.012	...						
**BPNSF20**	.007	.012	.016	.016	.023	.023	.010	.032	.009	.009	...					
**BPNSF22**	.009	.005	.010	.007	.011	.009	.004	.005	.010	.007	.011	...				
**BPNSF4**	.009	.021	.010	.006	.014	.007	.008	.009	.008	.005	.006	.009	...			
**BPNSF12**	.009	.009	.009	.009	.007	.012	.009	.008	.013	.010	.020	.006	.010	...		
**BPNSF16**	.011	.011	.020	.008	.007	.012	.007	.012	.009	.013	.012	.006	.009	.009	...	
**BPNSF24**	.003	.015	.013	.012	.018	.008	.014	.011	.012	.007	.034	.007	.007	.008	.013	
**BPNSF5**	.012	.004	.016	.006	.014	.006	.004	.008	.012	.013	.007	.014	.010	.009	.005	[illegible]
**BPNSF10**	.015	.010	.012	.012	.021	.009	.026	.013	.012	.005	.009	.010	.016	.012	.012	[illegible]
**BPNSF15**	.008	.010	.005	.005	.009	.009	.005	.042	.005	.009	.009	.012	.011	.037	.010	[illegible]
**BPNSF18**	.334	.012	.005	.264	.254	.214	.015	.612	.142	.116	.056	.010	.157	.188	.468	[illegible]
**BPNSF1**	.038	.014	.010	.012	.008	.010	.007	.018	.138	.007	.028	.005	.008	.007	.011	[illegible]
**BPNSF7**	.213	.021	.021	.012	.019	.015	.009	.030	.049	.084	.012	.007	.012	.006	.015	[illegible]
**BPNSF13**	.008	.008	.008	.006	.004	.006	.018	.007	.009	.007	.019	.005	.006	.006	.005	[illegible]
**BPNSF19**	.026	.012	.025	.016	.018	.012	.015	.012	.035	.012	.026	.005	.010	.019	.016	[illegible]

Sample Means (g3 - Measurement intercepts)

Sample Means - Lower Bounds (BC) (g3 - Measurement intercepts)

	BPNSF6	BPNSF11	BPNSF17	BPNSF23	BPNSF3	BPNSF9	BPNSF14	BPNSF21	BPNSF2	BPNSF8	BPNSF20	BPNSF22	BPNSF4	BPNSF12	BPNSF16	BPNSF[illegible]
**BPNSF6**	2.488	2.672	2.698	2.395	5.381	5.325	5.067	4.869	2.511	2.767	2.639	3.128	5.101	4.983	4.896	[illegible]

Sample Means - Upper Bounds (BC) (g3 - Measurement intercepts)

	BPNSF6	BPNSF11	BPNSF17	BPNSF23	BPNSF3	BPNSF9	BPNSF14	BPNSF21	BPNSF2	BPNSF8	BPNSF20	BPNSF22	BPNSF4	BPNSF12	BPNSF16	BPNSF[illegible]
**BPNSF6**	2.760	2.910	3.010	2.644	5.626	5.571	5.299	5.078	2.820	3.048	2.912	3.386	5.308	5.207	5.117	[illegible]

Sample Means - Two Tailed Significance (BC) (g3 - Measurement intercepts)

	BPNSF6	BPNSF11	BPNSF17	BPNSF23	BPNSF3	BPNSF9	BPNSF14	BPNSF21	BPNSF2	BPNSF8	BPNSF20	BPNSF22	BPNSF4	BPNSF12	BPNSF16	BPNSF[illegible]
**BPNSF6**	.019	.016	.005	.006	.007	.019	.007	.021	.009	.019	.005	.014	.006	.021	.018	[illegible]

Minimization History (Measurement intercepts)

Iteration		Negative eigenvalues	Condition #	Smallest eigenvalue	Diameter	F	NTries	Ratio
**0**	e	27		-.831	9999.000	16979.917	0	9999.000
**1**	e	25		-.152	3.022	7285.827	19	.436
**2**	e*	10		-.129	1.677	4483.401	5	.965
**3**	e*	1		-.003	.718	3615.453	5	.913
**4**	e*	0	1456.356		.945	3043.009	5	.719
**5**	e	0	896.735		1.035	2826.584	3	.000
**6**	e	1		-.008	1.164	2527.127	1	.888
**7**	e	0	1786.773		.363	2487.436	6	.718
**8**	e	0	3412.411		.488	2463.265	1	1.129
**9**	e	0	2973.790		.506	2460.006	1	.786
**10**	e	0	9450.281		.204	2457.414	1	1.062
**11**	e	0	9737.572		.226	2457.156	1	.933
**12**	e	0	13150.881		.031	2457.111	1	1.010
**13**	e	0	12887.617		.006	2457.111	1	1.002
**14**	e	0	12942.049		.000	2457.111	1	1.000

Pairwise Parameter Comparisons (Measurement intercepts)

Variance-covariance Matrix of Estimates (Measurement intercepts)

	a1_1	a2_1	a3_1	a4_1	a5_1	a6_1	a7_1	a8_1	a9_1	a10_1	a11_1	a12_1	a13_1	a14_1	a15_1	a16_1	a17_1	a18_1	ccc1_1	ccc2_1
**a1_1**	.003																			
**a2_1**	.001	.003																		

	a1_1	a2_1	a3_1	a4_1	a5_1	a6_1	a7_1	a8_1	a9_1	a10_1	a11_1	a12_1	a13_1	a14_1	a15_1	a16_1	a17_1	a18_1	ccc1_1	ccc2_1
**a3_1**	.001	.001	.003																	
**a4_1**	.000	.000	.000	.092																
**a5_1**	.000	.000	.000	.100	.137															
**a6_1**	.000	.000	.000	.081	.103	.094														
**a7_1**	.000	.000	.000	.000	.000	.000	.002													
**a8_1**	.000	.000	.000	.000	.000	.000	.001	.002												
**a9_1**	.000	.000	.000	.000	.000	.000	.001	.001	.002											
**a10_1**	.000	.000	.000	.000	.000	.000	.000	.000	.000	.002										
**a11_1**	.000	.000	.000	.000	.000	.000	.000	.000	.000	.001	.002									
**a12_1**	.000	.000	.000	.000	.000	.000	.000	.000	.000	.001	.001	.002								
**a13_1**	.000	.000	.000	.000	.000	.000	.000	.000	.000	.000	.000	.000	.002							
**a14_1**	.000	.000	.000	.000	.000	.000	.000	.000	.000	.000	.000	.000	.001	.002						
**a15_1**	.000	.000	.000	.000	.000	.000	.000	.000	.000	.000	.000	.000	.001	.001	.002					
**a16_1**	.000	.000	.000	.000	.000	.000	.000	.000	.000	.000	.000	.000	.000	.000	.000	.001				
**a17_1**	.000	.000	.000	.000	.000	.000	.000	.000	.000	.000	.000	.000	.000	.000	.000	.001	.001			
**a18_1**	.000	.000	.000	.000	.000	.000	.000	.000	.000	.000	.000	.000	.000	.000	.000	.000	.000	.001		
**ccc1_1**	.000	.000	.000	.002	.003	.003	.000	.000	.000	.000	.000	.000	.000	.000	.000	.000	.000	.000	.001	
**ccc2_1**	.000	.000	.000	.003	.004	.003	.000	.000	.000	.000	.000	.000	.000	.000	.000	.000	.000	.000	.000	.001
**ccc3_1**	-.001	-.001	-.001	.000	.000	.000	-.001	-.001	.000	.000	.000	.000	.000	.000	.000	.000	.000	.000	.000	.000
**ccc4_1**	.000	.000	.000	-.007	-.009	-.007	.000	.000	.000	.000	.000	.000	.000	.000	.000	.000	.000	.000	.000	-.001
**ccc5_1**	.000	.000	.000	.000	.000	.000	.001	.001	.000	.001	.001	.001	.000	.000	.000	.000	.000	.000	.000	.001
**ccc6_1**	.001	.000	.000	.000	.000	.000	.000	.000	.000	.001	.000	.000	.000	.000	.000	.000	.000	.000	.001	.000
**ccc7_1**	.000	.000	.000	.002	.002	.002	.000	.000	.000	.000	.000	.000	.000	.000	.000	.000	.000	.000	.000	.000
**ccc8_1**	.000	.000	.000	.000	.000	.000	.000	.000	.000	.001	.001	.001	.001	.001	.000	.000	.000	.000	.000	.000
**ccc9_1**	.000	.000	.000	.000	.000	.000	-.001	-.001	-.001	.000	.000	.000	-.001	-.001	.000	.000	.000	.000	.000	.000
**ccc10_1**	-.001	-.001	-.001	.000	.000	.000	.000	.000	.000	.000	.000	.000	-.001	-.001	-.001	.000	.000	.000	.000	.000
**ccc11_1**	.000	.000	.000	.000	.000	.000	.000	.000	.000	.000	.000	.000	.001	.001	.001	.000	.000	.000	.001	.000
**ccc12_1**	.000	.000	.000	.000	.000	.000	.001	.001	.000	.000	.000	.000	.000	.000	.000	.000	.000	.000	.001	.001
**ccc13_1**	.000	.000	.000	.000	.000	.000	.000	.000	.000	-.001	-.001	-.001	.000	.000	.000	-.001	-.001	-.001	-.001	-.001
**ccc14_1**	.000	.000	.000	-.009	-.012	-.009	.000	.000	.000	.000	.000	.000	.000	.000	.000	.000	.000	.000	-.001	-.001
**ccc15_1**	.001	.001	.001	.000	.000	.000	.000	.000	.000	.000	.000	.000	.000	.000	.000	.000	.000	.000	.001	.000
**vvv1_1**	-.003	-.002	-.002	.000	.000	.000	.000	.000	.000	.000	.000	.000	.000	.000	.000	.000	.000	.000	-.001	.000
**v1_1**	.000	.000	.000	.000	.000	.000	.000	.000	.000	.000	.000	.000	.000	.000	.000	.000	.000	.000	.000	.000
**v2_1**	.000	.000	.000	.000	.000	.000	.000	.000	.000	.000	.000	.000	.000	.000	.000	.000	.000	.000	.000	.000
**v3_1**	.000	.000	.000	.000	.000	.000	.000	.000	.000	.000	.000	.000	.000	.000	.000	.000	.000	.000	.000	.000
**v4_1**	.000	.000	.000	.000	.000	.000	.000	.000	.000	.000	.000	.000	.000	.000	.000	.000	.000	.000	.000	.000
**vvv2_1**	.000	.000	.000	-.009	-.011	-.009	.000	.000	.000	.000	.000	.000	.000	.000	.000	.000	.000	.000	.000	.000
**v5_1**	.000	.000	.000	.002	.003	.002	.000	.000	.000	.000	.000	.000	.000	.000	.000	.000	.000	.000	.000	.000
**v6_1**	.000	.000	.000	-.003	.000	.000	.000	.000	.000	.000	.000	.000	.000	.000	.000	.000	.000	.000	.000	.000
**v7_1**	.000	.000	.000	.000	-.004	.000	.000	.000	.000	.000	.000	.000	.000	.000	.000	.000	.000	.000	.000	.000
**v8_1**	.000	.000	.000	.000	.000	-.003	.000	.000	.000	.000	.000	.000	.000	.000	.000	.000	.000	.000	.000	.000
**vvv3_1**	.000	.000	.000	.000	.000	.000	-.002	-.002	-.001	.000	.000	.000	.000	.000	.000	.000	.000	.000	.000	-.001
**v9_1**	.000	.000	.000	.000	.000	.000	.000	.000	.000	.000	.000	.000	.000	.000	.000	.000	.000	.000	.000	.000
**v10_1**	.000	.000	.000	.000	.000	.000	.000	.000	.000	.000	.000	.000	.000	.000	.000	.000	.000	.000	.000	.000
**v11_1**	.000	.000	.000	.000	.000	.000	.000	.000	.000	.000	.000	.000	.000	.000	.000	.000	.000	.000	.000	.000
**v12_1**	.000	.000	.000	.000	.000	.000	.000	.000	.000	.000	.000	.000	.000	.000	.000	.000	.000	.000	.000	.000
**vvv4_1**	.000	.000	.000	.000	.000	.000	.000	.000	.000	-.002	-.002	-.002	.000	.000	.000	.000	.000	.000	.000	.000
**v13_1**	.000	.000	.000	.000	.000	.000	.000	.000	.000	.000	.000	.000	.000	.000	.000	.000	.000	.000	.000	.000
**v14_1**	.000	.000	.000	.000	.000	.000	.000	.000	.000	.000	.000	.000	.000	.000	.000	.000	.000	.000	.000	.000
**v15_1**	.000	.000	.000	.000	.000	.000	.000	.000	.000	.000	.000	.000	.000	.000	.000	.000	.000	.000	.000	.000
**v16_1**	.000	.000	.000	.000	.000	.000	.000	.000	.000	.000	.000	.000	.000	.000	.000	.000	.000	.000	.000	.000
**vvv5_1**	.000	.000	.000	.000	.000	.000	.000	.000	.000	.000	.000	.000	-.002	-.002	-.002	.000	.000	.000	.000	.000
**v17_1**	.000	.000	.000	.000	.000	.000	.000	.000	.000	.000	.000	.000	.000	.000	.000	.000	.000	.000	.000	.000
**v18_1**	.000	.000	.000	.000	.000	.000	.000	.000	.000	.000	.000	.000	.000	.000	.000	.000	.000	.000	.000	.000
**v19_1**	.000	.000	.000	.000	.000	.000	.000	.000	.000	.000	.000	.000	.000	.000	.000	.000	.000	.000	.000	.000
**v20_1**	.000	.000	.000	.000	.000	.000	.000	.000	.000	.000	.000	.000	.000	.000	.000	.000	.000	.000	.000	.000
**vvv6_1**	.000	.000	.000	.000	.000	.000	.000	.000	.000	.000	.000	.000	.000	.000	.000	-.002	-.002	-.001	-.001	-.001
**v21_1**	.000	.000	.000	.000	.000	.000	.000	.000	.000	.000	.000	.000	.000	.000	.000	.000	.000	.000	.000	.000
**v22_1**	.000	.000	.000	.000	.000	.000	.000	.000	.000	.000	.000	.000	.000	.000	.000	.000	.000	.000	.000	.000
**v23_1**	.000	.000	.000	.000	.000	.000	.000	.000	.000	.000	.000	.000	.000	.000	.000	.000	.000	.000	.000	.000
**v24_1**	.000	.000	.000	.000	.000	.000	.000	.000	.000	.000	.000	.000	.000	.000	.000	.000	.000	.000	.000	.000
**i1_1**	.000	.000	.000	.000	.000	.000	.000	.000	.000	.000	.000	.000	.000	.000	.000	.000	.000	.000	.000	.000
**i2_1**	.000	.000	.000	.000	.000	.000	.000	.000	.000	.000	.000	.000	.000	.000	.000	.000	.000	.000	.000	.000
**i3_1**	.000	.000	.000	.000	.000	.000	.000	.000	.000	.000	.000	.000	.000	.000	.000	.000	.000	.000	.000	.000
**i4_1**	.000	.000	.000	.000	.000	.000	.000	.000	.000	.000	.000	.000	.000	.000	.000	.000	.000	.000	.000	.000
**i5_1**	.000	.000	.000	.000	.000	.000	.000	.000	.000	.000	.000	.000	.000	.000	.000	.000	.000	.000	.000	.000
**i6_1**	.000	.000	.000	.000	.000	.000	.000	.000	.000	.000	.000	.000	.000	.000	.000	.000	.000	.000	.000	.000
**i7_1**	.000	.000	.000	.000	.000	.000	.000	.000	.000	.000	.000	.000	.000	.000	.000	.000	.000	.000	.000	.000
**i8_1**	.000	.000	.000	.000	.000	.000	.000	.000	.000	.000	.000	.000	.000	.000	.000	.000	.000	.000	.000	.000
**i9_1**	.000	.000	.000	.000	.000	.000	.000	.000	.000	.000	.000	.000	.000	.000	.000	.000	.000	.000	.000	.000
**i10_1**	.000	.000	.000	.000	.000	.000	.000	.000	.000	.000	.000	.000	.000	.000	.000	.000	.000	.000	.000	.000
**i11_1**	.000	.000	.000	.000	.000	.000	.000	.000	.000	.000	.000	.000	.000	.000	.000	.000	.000	.000	.000	.000

	a1_1	a2_1	a3_1	a4_1	a5_1	a6_1	a7_1	a8_1	a9_1	a10_1	a11_1	a12_1	a13_1	a14_1	a15_1	a16_1	a17_1	a18_1	ccc1_1	ccc2_1
**i12_1**	.000	.000	.000	.000	.000	.000	.000	.000	.000	.000	.000	.000	.000	.000	.000	.000	.000	.000	.000	.000
**i13_1**	.000	.000	.000	.000	.000	.000	.000	.000	.000	.000	.000	.000	.000	.000	.000	.000	.000	.000	.000	.000
**i14_1**	.000	.000	.000	.000	.000	.000	.000	.000	.000	.000	.000	.000	.000	.000	.000	.000	.000	.000	.000	.000
**i15_1**	.000	.000	.000	.000	.000	.000	.000	.000	.000	.000	.000	.000	.000	.000	.000	.000	.000	.000	.000	.000
**i16_1**	.000	.000	.000	.000	.000	.000	.000	.000	.000	.000	.000	.000	.000	.000	.000	.000	.000	.000	.000	.000
**i17_1**	.000	.000	.000	.000	.000	.000	.000	.000	.000	.000	.000	.000	.000	.000	.000	.000	.000	.000	.000	.000
**i18_1**	.000	.000	.000	.000	.000	.000	.000	.000	.000	.000	.000	.000	.000	.000	.000	.000	.000	.000	.000	.000
**i19_1**	.000	.000	.000	.000	.000	.000	.000	.000	.000	.000	.000	.000	.000	.000	.000	.000	.000	.000	.000	.000
**i20_1**	.000	.000	.000	.000	.000	.000	.000	.000	.000	.000	.000	.000	.000	.000	.000	.000	.000	.000	.000	.000
**i21_1**	.000	.000	.000	.000	.000	.000	.000	.000	.000	.000	.000	.000	.000	.000	.000	.000	.000	.000	.000	.000
**i22_1**	.000	.000	.000	.000	.000	.000	.000	.000	.000	.000	.000	.000	.000	.000	.000	.000	.000	.000	.000	.000
**i23_1**	.000	.000	.000	.000	.000	.000	.000	.000	.000	.000	.000	.000	.000	.000	.000	.000	.000	.000	.000	.000
**i24_1**	.000	.000	.000	.000	.000	.000	.000	.000	.000	.000	.000	.000	.000	.000	.000	.000	.000	.000	.000	.000
**ccc1_2**	.000	.000	.000	.007	.009	.007	.000	.000	.000	.000	.000	.000	.000	.000	.000	.000	.000	.000	.000	.000
**ccc2_2**	.000	.000	.000	.006	.008	.006	.000	.000	.000	.000	.000	.000	.000	.000	.000	.000	.000	.000	.000	.000
**ccc3_2**	-.001	-.001	-.001	.000	.000	.000	-.001	-.001	-.001	.000	.000	.000	.000	.000	.000	.000	.000	.000	.000	.000
**ccc4_2**	.000	.000	.000	-.012	-.015	-.012	.000	.000	.000	.000	.000	.000	.000	.000	.000	.000	.000	.000	.000	.000
**ccc5_2**	.000	.000	.000	.000	.000	.000	.001	.001	.001	.001	.001	.001	.000	.000	.000	.000	.000	.000	.000	.000
**ccc6_2**	.001	.001	.001	.000	.000	.000	.000	.000	.000	.001	.001	.001	.000	.000	.000	.000	.000	.000	.000	.000
**ccc7_2**	.000	.000	.000	.006	.008	.006	.000	.000	.000	.000	.000	.000	.000	.000	.000	.000	.000	.000	.000	.000
**ccc8_2**	.000	.000	.000	.000	.000	.000	.000	.000	.000	.001	.001	.001	.001	.001	.001	.000	.000	.000	.000	.000
**ccc9_2**	.000	.000	.000	.000	.000	.000	-.001	-.001	-.001	.000	.000	.000	-.001	-.001	-.001	.000	.000	.000	.000	.000
**ccc10_2**	-.002	-.001	-.001	.000	.000	.000	.000	.000	.000	.000	.000	.000	-.001	-.001	-.001	.000	.000	.000	.000	.000
**ccc11_2**	.000	.000	.000	.000	.000	.000	.000	.000	.000	.000	.000	.000	.001	.001	.001	.000	.000	.000	.000	.000
**ccc12_2**	.000	.000	.000	.000	.000	.000	.001	.001	.001	.000	.000	.000	.000	.000	.000	.000	.000	.000	.000	.000
**ccc13_2**	.000	.000	.000	.000	.000	.000	.000	.000	.000	-.001	-.001	-.001	.000	.000	.000	-.001	-.001	-.001	.000	.000
**ccc14_2**	.000	.000	.000	-.013	-.016	-.013	.000	.000	.000	.000	.000	.000	.000	.000	.000	.000	.000	.000	.000	-.001
**ccc15_2**	.001	.001	.001	.000	.000	.000	.000	.000	.000	.000	.000	.000	.000	.000	.000	.000	.000	.000	.000	.000
**vvv1_2**	-.003	-.002	-.002	.000	.000	.000	.000	.000	.000	.000	.000	.000	.000	.000	.000	.000	.000	.000	.000	.000
**v1_2**	.000	.000	.000	.000	.000	.000	.000	.000	.000	.000	.000	.000	.000	.000	.000	.000	.000	.000	.000	.000
**v2_2**	.000	.000	.000	.000	.000	.000	.000	.000	.000	.000	.000	.000	.000	.000	.000	.000	.000	.000	.000	.000
**v3_2**	.000	.000	.000	.000	.000	.000	.000	.000	.000	.000	.000	.000	.000	.000	.000	.000	.000	.000	.000	.000
**v4_2**	.000	.000	.000	.000	.000	.000	.000	.000	.000	.000	.000	.000	.000	.000	.000	.000	.000	.000	.000	.000
**vvv2_2**	.000	.000	.000	-.011	-.013	-.011	.000	.000	.000	.000	.000	.000	.000	.000	.000	.000	.000	.000	.000	.000
**v5_2**	.000	.000	.000	.002	.003	.002	.000	.000	.000	.000	.000	.000	.000	.000	.000	.000	.000	.000	.000	.000
**v6_2**	.000	.000	.000	-.002	.000	.000	.000	.000	.000	.000	.000	.000	.000	.000	.000	.000	.000	.000	.000	.000
**v7_2**	.000	.000	.000	.000	-.003	.000	.000	.000	.000	.000	.000	.000	.000	.000	.000	.000	.000	.000	.000	.000
**v8_2**	.000	.000	.000	.000	.000	-.002	.000	.000	.000	.000	.000	.000	.000	.000	.000	.000	.000	.000	.000	.000
**vvv3_2**	.000	.000	.000	.000	.000	.000	-.002	-.003	-.002	.000	.000	.000	.000	.000	.000	.000	.000	.000	.000	.000
**v9_2**	.000	.000	.000	.000	.000	.000	.000	.000	.000	.000	.000	.000	.000	.000	.000	.000	.000	.000	.000	.000
**v10_2**	.000	.000	.000	.000	.000	.000	.000	.000	.000	.000	.000	.000	.000	.000	.000	.000	.000	.000	.000	.000
**v11_2**	.000	.000	.000	.000	.000	.000	.000	.000	.000	.000	.000	.000	.000	.000	.000	.000	.000	.000	.000	.000
**v12_2**	.000	.000	.000	.000	.000	.000	.000	.000	.000	.000	.000	.000	.000	.000	.000	.000	.000	.000	.000	.000
**vvv4_2**	.000	.000	.000	.000	.000	.000	.000	.000	.000	-.003	-.003	-.003	.000	.000	.000	.000	.000	.000	.000	.000
**v13_2**	.000	.000	.000	.000	.000	.000	.000	.000	.000	.000	.000	.000	.000	.000	.000	.000	.000	.000	.000	.000
**v14_2**	.000	.000	.000	.000	.000	.000	.000	.000	.000	.000	.000	.000	.000	.000	.000	.000	.000	.000	.000	.000
**v15_2**	.000	.000	.000	.000	.000	.000	.000	.000	.000	.000	.000	.000	.000	.000	.000	.000	.000	.000	.000	.000
**v16_2**	.000	.000	.000	.000	.000	.000	.000	.000	.000	.000	.000	.000	.000	.000	.000	.000	.000	.000	.000	.000
**vvv5_2**	.000	.000	.000	.000	.000	.000	.000	.000	.000	.000	.000	.000	-.002	-.002	-.002	.000	.000	.000	.000	.000
**v17_2**	.000	.000	.000	.000	.000	.000	.000	.000	.000	.000	.000	.000	.000	.000	.000	.000	.000	.000	.000	.000
**v18_2**	.000	.000	.000	.000	.000	.000	.000	.000	.000	.000	.000	.000	.000	.000	.000	.000	.000	.000	.000	.000
**v19_2**	.000	.000	.000	.000	.000	.000	.000	.000	.000	.000	.000	.000	.000	.000	.000	.000	.000	.000	.000	.000
**v20_2**	.000	.000	.000	.000	.000	.000	.000	.000	.000	.000	.000	.000	.000	.000	.000	.000	.000	.000	.000	.000
**vvv6_2**	.000	.000	.000	.000	.000	.000	.000	.000	.000	.000	.000	.000	.000	.000	.000	-.002	-.002	-.001	.000	.000
**v21_2**	.000	.000	.000	.000	.000	.000	.000	.000	.000	.000	.000	.000	.000	.000	.000	.000	.000	.000	.000	.000
**v22_2**	.000	.000	.000	.000	.000	.000	.000	.000	.000	.000	.000	.000	.000	.000	.000	.000	.000	.000	.000	.000
**v23_2**	.000	.000	.000	.000	.000	.000	.000	.000	.000	.000	.000	.000	.000	.000	.000	.000	.000	.000	.000	.000
**v24_2**	.000	.000	.000	.000	.000	.000	.000	.000	.000	.000	.000	.000	.000	.000	.000	.000	.000	.000	.000	.000
**ccc1_3**	.000	.000	.000	.006	.007	.006	.000	.000	.000	.000	.000	.000	.000	.000	.000	.000	.000	.000	.000	.000
**ccc2_3**	.000	.000	.000	.005	.006	.005	.000	.000	.000	.000	.000	.000	.000	.000	.000	.000	.000	.000	.000	.000
**ccc3_3**	-.001	-.001	-.001	.000	.000	.000	-.001	-.001	-.001	.000	.000	.000	.000	.000	.000	.000	.000	.000	.000	.000
**ccc4_3**	.000	.000	.000	-.012	-.016	-.013	.000	.000	.000	.000	.000	.000	.000	.000	.000	.000	.000	.000	.000	.000
**ccc5_3**	.000	.000	.000	.000	.000	.000	.001	.001	.001	.001	.001	.001	.000	.000	.000	.000	.000	.000	.000	.000
**ccc6_3**	.001	.000	.000	.000	.000	.000	.000	.000	.000	.001	.001	.000	.000	.000	.000	.000	.000	.000	.000	.000
**ccc7_3**	.000	.000	.000	.006	.008	.006	.000	.000	.000	.000	.000	.000	.000	.000	.000	.000	.000	.000	.000	.000
**ccc8_3**	.000	.000	.000	.000	.000	.000	.000	.000	.000	.001	.001	.001	.001	.001	.001	.000	.000	.000	.000	.000
**ccc9_3**	.000	.000	.000	.000	.000	.000	-.001	-.001	-.001	.000	.000	.000	-.001	-.001	-.001	.000	.000	.000	.000	.000
**ccc10_3**	-.001	-.001	-.001	.000	.000	.000	.000	.000	.000	.000	.000	.000	-.001	-.001	-.001	.000	.000	.000	.000	.000
**ccc11_3**	.000	.000	.000	.000	.000	.000	.000	.000	.000	.000	.000	.000	.001	.001	.001	.001	.001	.001	.000	.000
**ccc12_3**	.000	.000	.000	.000	.000	.000	.001	.001	.001	.000	.000	.000	.000	.000	.000	.000	.000	.000	.000	.000
**ccc13_3**	.000	.000	.000	.000	.000	.000	.000	.000	.000	-.002	-.002	-.002	.000	.000	.000	-.001	-.001	-.001	.000	.000
**ccc14_3**	.000	.000	.000	-.015	-.019	-.015	.000	.000	.000	.000	.000	.000	.000	.000	.000	.000	.000	.000	.000	-.001

	a1_1	a2_1	a3_1	a4_1	a5_1	a6_1	a7_1	a8_1	a9_1	a10_1	a11_1	a12_1	a13_1	a14_1	a15_1	a16_1	a17_1	a18_1	ccc1_1	ccc2_1
**ccc15_3**	.001	.001	.001	.000	.000	.000	.000	.000	.000	.000	.000	.000	.000	.000	.000	.000	.000	.000	.000	.000
**vvv1_3**	-.002	-.002	-.002	.000	.000	.000	.000	.000	.000	.000	.000	.000	.000	.000	.000	.000	.000	.000	.000	.000
**v1_3**	.000	.000	.000	.000	.000	.000	.000	.000	.000	.000	.000	.000	.000	.000	.000	.000	.000	.000	.000	.000
**v2_3**	.000	.000	.000	.000	.000	.000	.000	.000	.000	.000	.000	.000	.000	.000	.000	.000	.000	.000	.000	.000
**v3_3**	.000	.000	.000	.000	.000	.000	.000	.000	.000	.000	.000	.000	.000	.000	.000	.000	.000	.000	.000	.000
**v4_3**	.000	.000	.000	.000	.000	.000	.000	.000	.000	.000	.000	.000	.000	.000	.000	.000	.000	.000	.000	.000
**vvv2_3**	.000	.000	.000	-.010	-.013	-.011	.000	.000	.000	.000	.000	.000	.000	.000	.000	.000	.000	.000	.000	.000
**v5_3**	.000	.000	.000	.002	.002	.002	.000	.000	.000	.000	.000	.000	.000	.000	.000	.000	.000	.000	.000	.000
**v6_3**	.000	.000	.000	-.002	.000	.000	.000	.000	.000	.000	.000	.000	.000	.000	.000	.000	.000	.000	.000	.000
**v7_3**	.000	.000	.000	.000	-.003	.000	.000	.000	.000	.000	.000	.000	.000	.000	.000	.000	.000	.000	.000	.000
**v8_3**	.000	.000	.000	.000	.000	-.002	.000	.000	.000	.000	.000	.000	.000	.000	.000	.000	.000	.000	.000	.000
**vvv3_3**	.000	.000	.000	.000	.000	.000	-.002	-.002	-.002	.000	.000	.000	.000	.000	.000	.000	.000	.000	.000	.000
**v9_3**	.000	.000	.000	.000	.000	.000	.000	.000	.000	.000	.000	.000	.000	.000	.000	.000	.000	.000	.000	.000
**v10_3**	.000	.000	.000	.000	.000	.000	.000	.000	.000	.000	.000	.000	.000	.000	.000	.000	.000	.000	.000	.000
**v11_3**	.000	.000	.000	.000	.000	.000	.000	.000	.000	.000	.000	.000	.000	.000	.000	.000	.000	.000	.000	.000
**v12_3**	.000	.000	.000	.000	.000	.000	.000	.000	.000	.000	.000	.000	.000	.000	.000	.000	.000	.000	.000	.000
**vvv4_3**	.000	.000	.000	.000	.000	.000	.000	.000	.000	-.003	-.003	-.003	.000	.000	.000	.000	.000	.000	.000	.000
**v13_3**	.000	.000	.000	.000	.000	.000	.000	.000	.000	.000	.000	.000	.000	.000	.000	.000	.000	.000	.000	.000
**v14_3**	.000	.000	.000	.000	.000	.000	.000	.000	.000	.000	.000	.000	.000	.000	.000	.000	.000	.000	.000	.000
**v15_3**	.000	.000	.000	.000	.000	.000	.000	.000	.000	.000	.000	.000	.000	.000	.000	.000	.000	.000	.000	.000
**v16_3**	.000	.000	.000	.000	.000	.000	.000	.000	.000	.000	.000	.000	.000	.000	.000	.000	.000	.000	.000	.000
**vvv5_3**	.000	.000	.000	.000	.000	.000	.000	.000	.000	.000	.000	.000	-.002	-.002	-.002	.000	.000	.000	.000	.000
**v17_3**	.000	.000	.000	.000	.000	.000	.000	.000	.000	.000	.000	.000	.000	.000	.000	.000	.000	.000	.000	.000
**v18_3**	.000	.000	.000	.000	.000	.000	.000	.000	.000	.000	.000	.000	.000	.000	.000	.000	.000	.000	.000	.000
**v19_3**	.000	.000	.000	.000	.000	.000	.000	.000	.000	.000	.000	.000	.000	.000	.000	.000	.000	.000	.000	.000
**v20_3**	.000	.000	.000	.000	.000	.000	.000	.000	.000	.000	.000	.000	.000	.000	.000	.000	.000	.000	.000	.000
**vvv6_3**	.000	.000	.000	.000	.000	.000	.000	.000	.000	.000	.000	.000	.000	.000	.000	-.002	-.002	-.002	.000	.000
**v21_3**	.000	.000	.000	.000	.000	.000	.000	.000	.000	.000	.000	.000	.000	.000	.000	.000	.000	.000	.000	.000
**v22_3**	.000	.000	.000	.000	.000	.000	.000	.000	.000	.000	.000	.000	.000	.000	.000	.000	.000	.000	.000	.000
**v23_3**	.000	.000	.000	.000	.000	.000	.000	.000	.000	.000	.000	.000	.000	.000	.000	.000	.000	.000	.000	.000
**v24_3**	.000	.000	.000	.000	.000	.000	.000	.000	.000	.000	.000	.000	.000	.000	.000	.000	.000	.000	.000	.000

Correlations of Estimates (Measurement intercepts)

	a1_1	a2_1	a3_1	a4_1	a5_1	a6_1	a7_1	a8_1	a9_1	a10_1	a11_1	a12_1	a13_1	a14_1	a15_1	a16_1	a17_1	a18_1	ccc1_1
**a1_1**	1.000																		
**a2_1**	.505	1.000																	
**a3_1**	.448	.346	1.000																
**a4_1**	.000	.000	.000	1.000															
**a5_1**	.000	.000	.000	.893	1.000														
**a6_1**	.000	.000	.000	.874	.905	1.000													
**a7_1**	.000	.000	.000	.000	.000	.000	1.000												
**a8_1**	.000	.000	.000	.000	.000	.000	.635	1.000											
**a9_1**	.000	.000	.000	.000	.000	.000	.480	.509	1.000										
**a10_1**	.000	.000	.000	.000	.000	.000	.000	.000	.000	1.000									
**a11_1**	.000	.000	.000	.000	.000	.000	.000	.000	.000	.588	1.000								
**a12_1**	.000	.000	.000	.000	.000	.000	.000	.000	.000	.567	.552	1.000							
**a13_1**	.000	.000	.000	.000	.000	.000	.000	.000	.000	.000	.000	.000	1.000						
**a14_1**	.000	.000	.000	.000	.000	.000	.000	.000	.000	.000	.000	.000	.553	1.000					
**a15_1**	.000	.000	.000	.000	.000	.000	.000	.000	.000	.000	.000	.000	.475	.449	1.000				
**a16_1**	.000	.000	.000	.000	.000	.000	.000	.000	.000	.000	.000	.000	.000	.000	.000	1.000			
**a17_1**	.000	.000	.000	.000	.000	.000	.000	.000	.000	.000	.000	.000	.000	.000	.000	.433	1.000		
**a18_1**	.000	.000	.000	.000	.000	.000	.000	.000	.000	.000	.000	.000	.000	.000	.000	.376	.378	1.000	
**ccc1_1**	.090	.066	.058	.327	.338	.330	.000	.000	.000	.000	.000	.000	.000	.000	.000	.000	.000	.000	1.000
**ccc2_1**	.000	.000	.000	.462	.477	.466	.104	.111	.083	.000	.000	.000	.000	.000	.000	.000	.000	.000	.644
**ccc3_1**	-.283	-.206	-.184	.000	.000	.000	-.232	-.247	-.185	.000	.000	.000	.000	.000	.000	.000	.000	.000	-.274
**ccc4_1**	.000	.000	.000	-.636	-.657	-.641	.000	.000	.000	-.148	-.141	-.136	.000	.000	.000	.000	.000	.000	-.484
**ccc5_1**	.000	.000	.000	.000	.000	.000	.195	.208	.156	.201	.192	.185	.000	.000	.000	.000	.000	.000	.263
**ccc6_1**	.195	.142	.126	.000	.000	.000	.000	.000	.000	.164	.157	.151	.000	.000	.000	.000	.000	.000	.421
**ccc7_1**	.000	.000	.000	.255	.263	.257	.000	.000	.000	.000	.000	.000	.055	.051	.045	.000	.000	.000	.757
**ccc8_1**	.000	.000	.000	.000	.000	.000	.000	.000	.000	.225	.215	.207	.208	.192	.170	.000	.000	.000	.272
**ccc9_1**	.000	.000	.000	.000	.000	.000	-.239	-.254	-.191	.000	.000	.000	-.228	-.211	-.186	.000	.000	.000	-.182
**ccc10_1**	-.328	-.239	-.213	.000	.000	.000	.000	.000	.000	.000	.000	.000	-.256	-.237	-.209	.000	.000	.000	-.188
**ccc11_1**	.000	.000	.000	.000	.000	.000	.000	.000	.000	.000	.000	.000	.231	.214	.189	.144	.149	.134	.294
**ccc12_1**	.000	.000	.000	.000	.000	.000	.179	.191	.143	.000	.000	.000	.000	.000	.000	.107	.111	.099	.303
**ccc13_1**	.000	.000	.000	.000	.000	.000	.000	.000	.000	-.292	-.279	-.269	.000	.000	.000	-.168	-.174	-.156	-.222
**ccc14_1**	.000	.000	.000	-.666	-.688	-.672	.000	.000	.000	.000	.000	.000	.000	.000	.000	-.089	-.093	-.083	-.518
**ccc15_1**	.220	.160	.143	.000	.000	.000	.000	.000	.000	.000	.000	.000	.000	.000	.000	.107	.111	.099	.458
**vvv1_1**	-.518	-.386	-.345	.000	.000	.000	.000	.000	.000	.000	.000	.000	.000	.000	.000	.000	.000	.000	-.220
**v1_1**	.088	.067	.060	.000	.000	.000	.000	.000	.000	.000	.000	.000	.000	.000	.000	.000	.000	.000	.009
**v2_1**	-.107	-.006	-.005	.000	.000	.000	.000	.000	.000	.000	.000	.000	.000	.000	.000	.000	.000	.000	-.008

	a1_1	a2_1	a3_1	a4_1	a5_1	a6_1	a7_1	a8_1	a9_1	a10_1	a11_1	a12_1	a13_1	a14_1	a15_1	a16_1	a17_1	a18_1	ccc1_1
**v3_1**	-.001	-.056	-.001	.000	.000	.000	.000	.000	.000	.000	.000	.000	.000	.000	.000	.000	.000	.000	-.001
**v4_1**	-.001	-.001	-.049	.000	.000	.000	.000	.000	.000	.000	.000	.000	.000	.000	.000	.000	.000	.000	-.001
**vvv2_1**	.000	.000	.000	-.865	-.888	-.871	.000	.000	.000	.000	.000	.000	.000	.000	.000	.000	.000	.000	-.397
**v5_1**	.000	.000	.000	.053	.055	.053	.000	.000	.000	.000	.000	.000	.000	.000	.000	.000	.000	.000	.019
**v6_1**	.000	.000	.000	-.072	.003	.002	.000	.000	.000	.000	.000	.000	.000	.000	.000	.000	.000	.000	-.006
**v7_1**	.000	.000	.000	.010	-.078	.005	.000	.000	.000	.000	.000	.000	.000	.000	.000	.000	.000	.000	-.011
**v8_1**	.000	.000	.000	.005	.004	-.067	.000	.000	.000	.000	.000	.000	.000	.000	.000	.000	.000	.000	-.006
**vvv3_1**	.000	.000	.000	.000	.000	.000	-.480	-.506	-.387	.000	.000	.000	.000	.000	.000	.000	.000	.000	-.151
**v9_1**	.000	.000	.000	.000	.000	.000	.118	.128	.093	.000	.000	.000	.000	.000	.000	.000	.000	.000	.000
**v10_1**	.000	.000	.000	.000	.000	.000	-.119	.003	-.004	.000	.000	.000	.000	.000	.000	.000	.000	.000	.000
**v11_1**	.000	.000	.000	.000	.000	.000	-.003	-.145	-.007	.000	.000	.000	.000	.000	.000	.000	.000	.000	.000
**v12_1**	.000	.000	.000	.000	.000	.000	-.001	.001	-.081	.000	.000	.000	.000	.000	.000	.000	.000	.000	.000
**vvv4_1**	.000	.000	.000	.000	.000	.000	.000	.000	.000	-.493	-.473	-.457	.000	.000	.000	.000	.000	.000	-.158
**v13_1**	.000	.000	.000	.000	.000	.000	.000	.000	.000	.084	.080	.077	.000	.000	.000	.000	.000	.000	.000
**v14_1**	.000	.000	.000	.000	.000	.000	.000	.000	.000	-.095	.000	.000	.000	.000	.000	.000	.000	.000	.000
**v15_1**	.000	.000	.000	.000	.000	.000	.000	.000	.000	.002	-.082	.000	.000	.000	.000	.000	.000	.000	.000
**v16_1**	.000	.000	.000	.000	.000	.000	.000	.000	.000	.002	.000	-.078	.000	.000	.000	.000	.000	.000	.000
**vvv5_1**	.000	.000	.000	.000	.000	.000	.000	.000	.000	.000	.000	.000	-.462	-.429	-.380	.000	.000	.000	-.096
**v17_1**	.000	.000	.000	.000	.000	.000	.000	.000	.000	.000	.000	.000	.058	.055	.049	.000	.000	.000	.000
**v18_1**	.000	.000	.000	.000	.000	.000	.000	.000	.000	.000	.000	.000	-.066	.000	.003	.000	.000	.000	.000
**v19_1**	.000	.000	.000	.000	.000	.000	.000	.000	.000	.000	.000	.000	.001	-.056	.002	.000	.000	.000	.000
**v20_1**	.000	.000	.000	.000	.000	.000	.000	.000	.000	.000	.000	.000	.000	.000	-.052	.000	.000	.000	.000
**vvv6_1**	.000	.000	.000	.000	.000	.000	.000	.000	.000	.000	.000	.000	.000	.000	.000	-.325	-.336	-.302	-.218
**v21_1**	.000	.000	.000	.000	.000	.000	.000	.000	.000	.000	.000	.000	.000	.000	.000	.091	.094	.084	.000
**v22_1**	.000	.000	.000	.000	.000	.000	.000	.000	.000	.000	.000	.000	.000	.000	.000	-.077	-.002	.000	.000
**v23_1**	.000	.000	.000	.000	.000	.000	.000	.000	.000	.000	.000	.000	.000	.000	.000	-.004	-.084	.000	.000
**v24_1**	.000	.000	.000	.000	.000	.000	.000	.000	.000	.000	.000	.000	.000	.000	.000	-.002	-.001	-.075	.000
**i1_1**	.000	.000	.000	.000	.000	.000	.000	.000	.000	.000	.000	.000	.000	.000	.000	.000	.000	.000	.000
**i2_1**	.000	.000	.000	.000	.000	.000	.000	.000	.000	.000	.000	.000	.000	.000	.000	.000	.000	.000	.000
**i3_1**	.000	.000	.000	.000	.000	.000	.000	.000	.000	.000	.000	.000	.000	.000	.000	.000	.000	.000	.000
**i4_1**	.000	.000	.000	.000	.000	.000	.000	.000	.000	.000	.000	.000	.000	.000	.000	.000	.000	.000	.000
**i5_1**	.000	.000	.000	.000	.000	.000	.000	.000	.000	.000	.000	.000	.000	.000	.000	.000	.000	.000	.000
**i6_1**	.000	.000	.000	.000	.000	.000	.000	.000	.000	.000	.000	.000	.000	.000	.000	.000	.000	.000	.000
**i7_1**	.000	.000	.000	.000	.000	.000	.000	.000	.000	.000	.000	.000	.000	.000	.000	.000	.000	.000	.000
**i8_1**	.000	.000	.000	.000	.000	.000	.000	.000	.000	.000	.000	.000	.000	.000	.000	.000	.000	.000	.000
**i9_1**	.000	.000	.000	.000	.000	.000	.000	.000	.000	.000	.000	.000	.000	.000	.000	.000	.000	.000	.000
**i10_1**	.000	.000	.000	.000	.000	.000	.000	.000	.000	.000	.000	.000	.000	.000	.000	.000	.000	.000	.000
**i11_1**	.000	.000	.000	.000	.000	.000	.000	.000	.000	.000	.000	.000	.000	.000	.000	.000	.000	.000	.000
**i12_1**	.000	.000	.000	.000	.000	.000	.000	.000	.000	.000	.000	.000	.000	.000	.000	.000	.000	.000	.000
**i13_1**	.000	.000	.000	.000	.000	.000	.000	.000	.000	.000	.000	.000	.000	.000	.000	.000	.000	.000	.000
**i14_1**	.000	.000	.000	.000	.000	.000	.000	.000	.000	.000	.000	.000	.000	.000	.000	.000	.000	.000	.000
**i15_1**	.000	.000	.000	.000	.000	.000	.000	.000	.000	.000	.000	.000	.000	.000	.000	.000	.000	.000	.000
**i16_1**	.000	.000	.000	.000	.000	.000	.000	.000	.000	.000	.000	.000	.000	.000	.000	.000	.000	.000	.000
**i17_1**	.000	.000	.000	.000	.000	.000	.000	.000	.000	.000	.000	.000	.000	.000	.000	.000	.000	.000	.000
**i18_1**	.000	.000	.000	.000	.000	.000	.000	.000	.000	.000	.000	.000	.000	.000	.000	.000	.000	.000	.000
**i19_1**	.000	.000	.000	.000	.000	.000	.000	.000	.000	.000	.000	.000	.000	.000	.000	.000	.000	.000	.000
**i20_1**	.000	.000	.000	.000	.000	.000	.000	.000	.000	.000	.000	.000	.000	.000	.000	.000	.000	.000	.000
**i21_1**	.000	.000	.000	.000	.000	.000	.000	.000	.000	.000	.000	.000	.000	.000	.000	.000	.000	.000	.000
**i22_1**	.000	.000	.000	.000	.000	.000	.000	.000	.000	.000	.000	.000	.000	.000	.000	.000	.000	.000	.000
**i23_1**	.000	.000	.000	.000	.000	.000	.000	.000	.000	.000	.000	.000	.000	.000	.000	.000	.000	.000	.000
**i24_1**	.000	.000	.000	.000	.000	.000	.000	.000	.000	.000	.000	.000	.000	.000	.000	.000	.000	.000	.000
**ccc1_2**	.181	.139	.126	.618	.644	.629	.000	.000	.000	.000	.000	.000	.000	.000	.000	.000	.000	.000	.244
**ccc2_2**	.000	.000	.000	.587	.611	.597	.138	.150	.109	.000	.000	.000	.000	.000	.000	.000	.000	.000	.214
**ccc3_2**	-.326	-.250	-.228	.000	.000	.000	-.262	-.285	-.207	.000	.000	.000	.000	.000	.000	.000	.000	.000	-.033
**ccc4_2**	.000	.000	.000	-.726	-.756	-.739	.000	.000	.000	-.169	-.161	-.162	.000	.000	.000	.000	.000	.000	-.265
**ccc5_2**	.000	.000	.000	.000	.000	.000	.236	.257	.187	.235	.223	.224	.000	.000	.000	.000	.000	.000	.000
**ccc6_2**	.259	.199	.181	.000	.000	.000	.000	.000	.000	.207	.197	.197	.000	.000	.000	.000	.000	.000	.026
**ccc7_2**	.000	.000	.000	.576	.599	.586	.000	.000	.000	.000	.000	.000	.124	.114	.097	.000	.000	.000	.211
**ccc8_2**	.000	.000	.000	.000	.000	.000	.000	.000	.000	.218	.207	.208	.201	.184	.158	.000	.000	.000	.000
**ccc9_2**	.000	.000	.000	.000	.000	.000	-.269	-.293	-.212	.000	.000	.000	-.247	-.226	-.193	.000	.000	.000	.000
**ccc10_2**	-.349	-.268	-.244	.000	.000	.000	.000	.000	.000	.000	.000	.000	-.257	-.235	-.202	.000	.000	.000	-.035
**ccc11_2**	.000	.000	.000	.000	.000	.000	.000	.000	.000	.000	.000	.000	.224	.205	.175	.154	.139	.124	.000
**ccc12_2**	.000	.000	.000	.000	.000	.000	.202	.220	.160	.000	.000	.000	.000	.000	.000	.127	.115	.103	.000
**ccc13_2**	.000	.000	.000	.000	.000	.000	.000	.000	.000	-.282	-.268	-.269	.000	.000	.000	-.179	-.161	-.144	.000
**ccc14_2**	.000	.000	.000	-.713	-.742	-.725	.000	.000	.000	.000	.000	.000	.000	.000	.000	-.105	-.095	-.085	-.260
**ccc15_2**	.241	.185	.169	.000	.000	.000	.000	.000	.000	.000	.000	.000	.000	.000	.000	.122	.110	.098	.024
**vvv1_2**	-.536	-.416	-.380	.000	.000	.000	.000	.000	.000	.000	.000	.000	.000	.000	.000	.000	.000	.000	-.054
**v1_2**	.041	.035	.032	.000	.000	.000	.000	.000	.000	.000	.000	.000	.000	.000	.000	.000	.000	.000	.004
**v2_2**	-.055	.005	.008	.000	.000	.000	.000	.000	.000	.000	.000	.000	.000	.000	.000	.000	.000	.000	-.004
**v3_2**	.000	-.039	.003	.000	.000	.000	.000	.000	.000	.000	.000	.000	.000	.000	.000	.000	.000	.000	-.001
**v4_2**	.000	.001	-.038	.000	.000	.000	.000	.000	.000	.000	.000	.000	.000	.000	.000	.000	.000	.000	-.001
**vvv2_2**	.000	.000	.000	-.868	-.899	-.882	.000	.000	.000	.000	.000	.000	.000	.000	.000	.000	.000	.000	-.316

	a1_1	a2_1	a3_1	a4_1	a5_1	a6_1	a7_1	a8_1	a9_1	a10_1	a11_1	a12_1	a13_1	a14_1	a15_1	a16_1	a17_1	a18_1	ccc1_1
v5_2	.000	.000	.000	.047	.049	.048	.000	.000	.000	.000	.000	.000	.000	.000	.000	.000	.000	.000	.017
v6_2	.000	.000	.000	-.054	.002	.002	.000	.000	.000	.000	.000	.000	.000	.000	.000	.000	.000	.000	-.005
v7_2	.000	.000	.000	.002	-.070	.005	.000	.000	.000	.000	.000	.000	.000	.000	.000	.000	.000	.000	-.011
v8_2	.000	.000	.000	.001	.002	-.061	.000	.000	.000	.000	.000	.000	.000	.000	.000	.000	.000	.000	-.006
vvv3_2	.000	.000	.000	.000	.000	.000	-.503	-.542	-.399	.000	.000	.000	.000	.000	.000	.000	.000	.000	.000
v9_2	.000	.000	.000	.000	.000	.000	.091	.101	.071	.000	.000	.000	.000	.000	.000	.000	.000	.000	.000
v10_2	.000	.000	.000	.000	.000	.000	-.104	.013	.000	.000	.000	.000	.000	.000	.000	.000	.000	.000	.000
v11_2	.000	.000	.000	.000	.000	.000	.007	-.137	-.001	.000	.000	.000	.000	.000	.000	.000	.000	.000	.000
v12_2	.000	.000	.000	.000	.000	.000	.001	.004	-.068	.000	.000	.000	.000	.000	.000	.000	.000	.000	.000
vvv4_2	.000	.000	.000	.000	.000	.000	.000	.000	.000	-.492	-.469	-.470	.000	.000	.000	.000	.000	.000	.000
v13_2	.000	.000	.000	.000	.000	.000	.000	.000	.000	.074	.070	.070	.000	.000	.000	.000	.000	.000	.000
v14_2	.000	.000	.000	.000	.000	.000	.000	.000	.000	-.082	-.001	.004	.000	.000	.000	.000	.000	.000	.000
v15_2	.000	.000	.000	.000	.000	.000	.000	.000	.000	.001	-.070	.003	.000	.000	.000	.000	.000	.000	.000
v16_2	.000	.000	.000	.000	.000	.000	.000	.000	.000	.001	-.001	-.079	.000	.000	.000	.000	.000	.000	.000
vvv5_2	.000	.000	.000	.000	.000	.000	.000	.000	.000	.000	.000	.000	-.463	-.426	-.366	.000	.000	.000	.000
v17_2	.000	.000	.000	.000	.000	.000	.000	.000	.000	.000	.000	.000	.064	.059	.051	.000	.000	.000	.000
v18_2	.000	.000	.000	.000	.000	.000	.000	.000	.000	.000	.000	.000	-.072	-.001	.000	.000	.000	.000	.000
v19_2	.000	.000	.000	.000	.000	.000	.000	.000	.000	.000	.000	.000	.001	-.059	.000	.000	.000	.000	.000
v20_2	.000	.000	.000	.000	.000	.000	.000	.000	.000	.000	.000	.000	.000	.000	-.048	.000	.000	.000	.000
vvv6_2	.000	.000	.000	.000	.000	.000	.000	.000	.000	.000	.000	.000	.000	.000	.000	-.347	-.315	-.282	.000
v21_2	.000	.000	.000	.000	.000	.000	.000	.000	.000	.000	.000	.000	.000	.000	.000	.105	.095	.084	.000
v22_2	.000	.000	.000	.000	.000	.000	.000	.000	.000	.000	.000	.000	.000	.000	.000	-.103	-.006	-.004	.000
v23_2	.000	.000	.000	.000	.000	.000	.000	.000	.000	.000	.000	.000	.000	.000	.000	.002	-.078	-.003	.000
v24_2	.000	.000	.000	.000	.000	.000	.000	.000	.000	.000	.000	.000	.000	.000	.000	.001	-.003	-.068	.000
ccc1_3	.147	.117	.100	.558	.586	.566	.000	.000	.000	.000	.000	.000	.000	.000	.000	.000	.000	.000	.220
ccc2_3	.000	.000	.000	.533	.561	.541	.126	.136	.100	.000	.000	.000	.000	.000	.000	.000	.000	.000	.196
ccc3_3	-.295	-.236	-.200	.000	.000	.000	-.266	-.285	-.210	.000	.000	.000	.000	.000	.000	.000	.000	.000	-.030
ccc4_3	.000	.000	.000	-.724	-.760	-.734	.000	.000	.000	-.164	-.167	-.153	.000	.000	.000	.000	.000	.000	-.265
ccc5_3	.000	.000	.000	.000	.000	.000	.206	.221	.163	.197	.201	.184	.000	.000	.000	.000	.000	.000	.000
ccc6_3	.171	.137	.116	.000	.000	.000	.000	.000	.000	.148	.151	.137	.000	.000	.000	.000	.000	.000	.017
ccc7_3	.000	.000	.000	.555	.583	.563	.000	.000	.000	.000	.000	.000	.112	.112	.092	.000	.000	.000	.204
ccc8_3	.000	.000	.000	.000	.000	.000	.000	.000	.000	.206	.210	.192	.184	.183	.151	.000	.000	.000	.000
ccc9_3	.000	.000	.000	.000	.000	.000	-.273	-.292	-.215	.000	.000	.000	-.233	-.232	-.191	.000	.000	.000	.000
ccc10_3	-.300	-.240	-.204	.000	.000	.000	.000	.000	.000	.000	.000	.000	-.231	-.230	-.189	.000	.000	.000	-.030
ccc11_3	.000	.000	.000	.000	.000	.000	.000	.000	.000	.000	.000	.000	.224	.223	.184	.163	.172	.140	.000
ccc12_3	.000	.000	.000	.000	.000	.000	.213	.228	.168	.000	.000	.000	.000	.000	.000	.132	.139	.114	.000
ccc13_3	.000	.000	.000	.000	.000	.000	.000	.000	.000	-.278	-.283	-.258	.000	.000	.000	-.180	-.190	-.155	.000
ccc14_3	.000	.000	.000	-.727	-.764	-.738	.000	.000	.000	.000	.000	.000	.000	.000	.000	-.107	-.113	-.092	-.267
ccc15_3	.198	.158	.134	.000	.000	.000	.000	.000	.000	.000	.000	.000	.000	.000	.000	.111	.117	.095	.020
vvv1_3	-.486	-.394	-.335	.000	.000	.000	.000	.000	.000	.000	.000	.000	.000	.000	.000	.000	.000	.000	-.050
v1_3	.070	.059	.051	.000	.000	.000	.000	.000	.000	.000	.000	.000	.000	.000	.000	.000	.000	.000	.007
v2_3	-.074	.000	-.003	.000	.000	.000	.000	.000	.000	.000	.000	.000	.000	.000	.000	.000	.000	.000	-.005
v3_3	-.004	-.054	-.001	.000	.000	.000	.000	.000	.000	.000	.000	.000	.000	.000	.000	.000	.000	.000	-.002
v4_3	-.002	.000	-.040	.000	.000	.000	.000	.000	.000	.000	.000	.000	.000	.000	.000	.000	.000	.000	-.001
vvv2_3	.000	.000	.000	-.863	-.902	-.876	.000	.000	.000	.000	.000	.000	.000	.000	.000	.000	.000	.000	-.316
v5_3	.000	.000	.000	.042	.044	.043	.000	.000	.000	.000	.000	.000	.000	.000	.000	.000	.000	.000	.016
v6_3	.000	.000	.000	-.043	.002	-.001	.000	.000	.000	.000	.000	.000	.000	.000	.000	.000	.000	.000	-.004
v7_3	.000	.000	.000	-.003	-.071	-.002	.000	.000	.000	.000	.000	.000	.000	.000	.000	.000	.000	.000	-.012
v8_3	.000	.000	.000	-.001	.002	-.047	.000	.000	.000	.000	.000	.000	.000	.000	.000	.000	.000	.000	-.004
vvv3_3	.000	.000	.000	.000	.000	.000	-.496	-.528	-.394	.000	.000	.000	.000	.000	.000	.000	.000	.000	.000
v9_3	.000	.000	.000	.000	.000	.000	.084	.091	.066	.000	.000	.000	.000	.000	.000	.000	.000	.000	.000
v10_3	.000	.000	.000	.000	.000	.000	-.097	.008	.000	.000	.000	.000	.000	.000	.000	.000	.000	.000	.000
v11_3	.000	.000	.000	.000	.000	.000	.006	-.118	.000	.000	.000	.000	.000	.000	.000	.000	.000	.000	.000
v12_3	.000	.000	.000	.000	.000	.000	.001	.003	-.065	.000	.000	.000	.000	.000	.000	.000	.000	.000	.000
vvv4_3	.000	.000	.000	.000	.000	.000	.000	.000	.000	-.478	-.487	-.446	.000	.000	.000	.000	.000	.000	.000
v13_3	.000	.000	.000	.000	.000	.000	.000	.000	.000	.082	.083	.076	.000	.000	.000	.000	.000	.000	.000
v14_3	.000	.000	.000	.000	.000	.000	.000	.000	.000	-.082	.003	-.003	.000	.000	.000	.000	.000	.000	.000
v15_3	.000	.000	.000	.000	.000	.000	.000	.000	.000	-.002	-.095	-.003	.000	.000	.000	.000	.000	.000	.000
v16_3	.000	.000	.000	.000	.000	.000	.000	.000	.000	-.001	.002	-.070	.000	.000	.000	.000	.000	.000	.000
vvv5_3	.000	.000	.000	.000	.000	.000	.000	.000	.000	.000	.000	.000	-.434	-.434	-.359	.000	.000	.000	.000
v17_3	.000	.000	.000	.000	.000	.000	.000	.000	.000	.000	.000	.000	.065	.065	.054	.000	.000	.000	.000
v18_3	.000	.000	.000	.000	.000	.000	.000	.000	.000	.000	.000	.000	-.062	.000	-.001	.000	.000	.000	.000
v19_3	.000	.000	.000	.000	.000	.000	.000	.000	.000	.000	.000	.000	-.004	-.067	-.001	.000	.000	.000	.000
v20_3	.000	.000	.000	.000	.000	.000	.000	.000	.000	.000	.000	.000	-.002	.000	-.047	.000	.000	.000	.000
vvv6_3	.000	.000	.000	.000	.000	.000	.000	.000	.000	.000	.000	.000	.000	.000	.000	-.346	-.365	-.300	.000
v21_3	.000	.000	.000	.000	.000	.000	.000	.000	.000	.000	.000	.000	.000	.000	.000	.076	.080	.066	.000
v22_3	.000	.000	.000	.000	.000	.000	.000	.000	.000	.000	.000	.000	.000	.000	.000	-.075	.004	.001	.000
v23_3	.000	.000	.000	.000	.000	.000	.000	.000	.000	.000	.000	.000	.000	.000	.000	.001	-.087	.001	.000
v24_3	.000	.000	.000	.000	.000	.000	.000	.000	.000	.000	.000	.000	.000	.000	.000	.001	.003	-.061	.000

Critical Ratios for Differences between Parameters (Measurement intercepts)

	a1_1	a2_1	a3_1	a4_1	a5_1	a6_1	a7_1	a8_1	a9_1	a10_1	a11_1	a12_1	a13_1	a14_1	a15_1
**a1_1**	.000														
**a2_1**	-5.560	.000													
**a3_1**	-7.210	-1.798	.000												
**a4_1**	4.258	5.177	5.513	.000											
**a5_1**	5.245	6.001	6.278	3.858	.000										
**a6_1**	4.408	5.317	5.649	.404	-3.685	.000									
**a7_1**	-.286	3.831	5.350	-4.334	-5.308	-4.484	.000								
**a8_1**	1.094	5.180	6.684	-4.017	-5.047	-4.171	2.404	.000							
**a9_1**	-4.427	-.254	1.300	-5.250	-6.060	-5.389	-6.078	-8.293	.000						
**a10_1**	.682	4.720	6.208	-4.108	-5.121	-4.260	1.014	-.418	5.318	.000					
**a11_1**	.731	4.717	6.185	-4.091	-5.108	-4.244	1.060	-.352	5.301	.092	.000				
**a12_1**	-.349	3.676	5.162	-4.345	-5.317	-4.495	-.073	-1.492	4.199	-1.614	-1.654	.000			
**a13_1**	-.753	3.533	5.117	-4.442	-5.396	-4.590	-.484	-1.997	4.104	-1.531	-1.571	-.395	.000		
**a14_1**	-1.592	2.681	4.264	-4.624	-5.546	-4.770	-1.376	-2.876	3.182	-2.399	-2.425	-1.263	-1.403	.000	
**a15_1**	-3.957	.294	1.877	-5.136	-5.966	-5.276	-3.889	-5.359	.599	-4.850	-4.838	-3.711	-4.942	-3.557	.000
**a16_1**	-2.714	1.853	3.551	-4.843	-5.726	-4.987	-2.571	-4.168	2.337	-3.633	-3.637	-2.410	-2.172	-1.149	1.730
**a17_1**	-3.009	1.581	3.290	-4.901	-5.773	-5.044	-2.888	-4.491	2.044	-3.947	-3.945	-2.716	-2.507	-1.475	1.424
**a18_1**	-4.541	-.022	1.664	-5.218	-6.033	-5.357	-4.530	-6.089	.278	-5.530	-5.500	-4.312	-4.252	-3.233	-.378
**ccc1_1**	-22.121	-17.183	-15.294	-8.545	-8.772	-8.651	-22.820	-24.169	-18.376	-23.237	-22.822	-21.902	-23.931	-22.782	-19.689
**ccc2_1**	-21.976	-17.355	-15.546	-8.722	-8.919	-8.828	-24.629	-26.091	-19.825	-23.950	-23.511	-22.602	-24.768	-23.604	-20.479
**ccc3_1**	-6.176	-3.193	-2.027	-6.040	-6.714	-6.170	-6.350	-7.368	-3.198	-7.770	-7.741	-6.836	-6.850	-6.098	-4.001
**ccc4_1**	-14.980	-10.644	-8.987	-6.869	-7.344	-6.978	-15.721	-17.097	-11.365	-15.340	-15.185	-14.273	-16.122	-15.102	-12.305
**ccc5_1**	-20.826	-17.360	-16.034	-9.448	-9.520	-9.539	-23.905	-25.310	-19.692	-24.576	-24.237	-23.267	-21.864	-21.077	-18.927
**ccc6_1**	-21.286	-17.061	-15.569	-9.261	-9.370	-9.355	-19.669	-20.713	-16.449	-22.017	-21.771	-20.863	-19.903	-19.165	-17.139
**ccc7_1**	-21.075	-16.462	-14.661	-8.431	-8.676	-8.538	-22.560	-23.917	-18.090	-22.985	-22.572	-21.646	-24.246	-23.021	-19.793
**ccc8_1**	-20.403	-17.309	-16.132	-9.906	-9.909	-9.993	-20.889	-21.864	-17.891	-24.061	-23.788	-22.858	-23.347	-22.403	-20.095
**ccc9_1**	-6.571	-3.115	-1.828	-5.945	-6.636	-6.076	-5.942	-6.952	-2.816	-7.336	-7.313	-6.412	-5.815	-5.173	-3.307
**ccc10_1**	-2.445	.170	1.178	-5.020	-5.875	-5.160	-2.655	-3.680	.381	-3.353	-3.373	-2.570	-2.161	-1.608	-.016
**ccc11_1**	-19.845	-17.188	-16.185	-10.556	-10.469	-10.636	-20.148	-20.999	-17.570	-20.612	-20.522	-19.886	-22.338	-21.530	-19.566
**ccc12_1**	-19.315	-16.154	-14.954	-9.515	-9.584	-9.605	-21.677	-22.945	-17.918	-20.296	-20.159	-19.423	-19.980	-19.280	-17.358
**ccc13_1**	.093	2.631	3.563	-4.074	-5.089	-4.222	.276	-.604	2.858	-.310	-.343	.289	.563	1.081	2.540
**ccc14_1**	-12.550	-8.566	-7.059	-6.478	-7.007	-6.588	-12.978	-14.275	-8.956	-13.686	-13.551	-12.590	-13.111	-12.208	-9.714
**ccc15_1**	-20.552	-16.749	-15.429	-9.702	-9.750	-9.791	-18.696	-19.619	-15.890	-19.197	-19.098	-18.410	-18.792	-18.166	-16.443
**vvv1_1**	-2.148	.001	.837	-4.999	-5.859	-5.138	-2.440	-3.323	.160	-3.046	-3.067	-2.372	-2.194	-1.665	-.182
**v1_1**	.376	3.179	4.196	-4.020	-5.046	-4.170	.565	-.382	3.350	-.104	-.143	.607	.880	1.439	3.012
**v2_1**	-3.692	-.723	.439	-5.308	-6.112	-5.445	-3.775	-4.855	-.557	-4.499	-4.508	-3.666	-3.513	-2.850	-.993
**v3_1**	4.285	6.305	7.206	-2.261	-3.565	-2.425	4.501	3.753	6.676	3.957	3.912	4.511	4.785	5.215	6.427
**v4_1**	5.261	7.256	7.861	-1.686	-3.074	-1.854	5.470	4.773	7.495	4.961	4.916	5.476	5.740	6.139	7.265
**vvv2_1**	-16.552	-12.152	-10.462	-6.990	-7.435	-7.095	-17.456	-18.829	-13.070	-18.068	-17.806	-16.825	-18.004	-16.955	-14.089
**v5_1**	6.176	8.045	8.726	-1.119	-2.614	-1.295	6.376	5.723	8.269	5.897	5.854	6.379	6.632	7.004	8.055
**v6_1**	3.097	5.203	5.973	-2.654	-3.953	-2.882	3.290	2.557	5.424	2.762	2.721	3.307	3.556	3.980	5.173
**v7_1**	1.697	3.705	4.440	-3.250	-4.278	-3.398	1.861	1.165	3.887	1.365	1.331	1.886	2.099	2.503	3.640
**v8_1**	2.823	4.951	5.730	-2.855	-4.064	-2.942	3.015	2.274	5.172	2.482	2.442	3.034	3.281	3.710	4.917
**vvv3_1**	-4.787	-1.627	-.456	-5.564	-6.324	-5.699	-3.937	-4.791	-1.293	-5.433	-5.434	-4.595	-4.481	-3.814	-1.946
**v9_1**	-4.386	-.925	.358	-5.379	-6.169	-5.516	-4.578	-5.873	-.788	-5.096	-5.096	-4.177	-4.051	-3.312	-1.242
**v10_1**	-5.073	-1.609	-.323	-5.557	-6.315	-5.692	-4.764	-6.220	-1.470	-5.801	-5.794	-4.879	-4.789	-4.047	-1.973
**v11_1**	-6.783	-3.210	-1.879	-5.955	-6.643	-6.086	-6.810	-7.513	-3.154	-7.586	-7.556	-6.628	-6.641	-5.868	-3.710
**v12_1**	-1.568	1.569	2.726	-4.649	-5.567	-4.793	-1.392	-2.481	1.771	-2.143	-2.172	-1.318	-1.072	-.416	1.426
**vvv4_1**	-1.284	1.212	2.130	-4.554	-5.489	-4.697	-1.132	-1.993	1.397	-1.470	-1.499	-.925	-.872	-.361	1.075
**v13_1**	.117	2.902	3.925	-4.111	-5.121	-4.260	.320	-.648	3.168	-.376	-.415	.378	.637	1.210	2.822
**v14_1**	-1.603	1.304	2.374	-4.664	-5.580	-4.807	-1.439	-2.443	1.531	-2.050	-2.161	-1.370	-1.142	-.539	1.154
**v15_1**	.898	3.444	4.378	-3.793	-4.856	-3.945	1.099	.214	3.693	.471	.417	1.134	1.399	1.918	3.380
**v16_1**	1.231	3.774	4.706	-3.675	-4.758	-3.828	1.438	.553	4.031	.809	.767	1.427	1.743	2.261	3.720
**vvv5_1**	-1.501	1.060	2.002	-4.632	-5.553	-4.774	-1.350	-2.233	1.247	-1.963	-1.990	-1.293	-.940	-.490	.810
**v17_1**	-.828	2.160	3.259	-4.428	-5.385	-4.575	-.637	-1.674	2.427	-1.360	-1.394	-.577	-.323	.312	2.090
**v18_1**	-4.098	-.705	.552	-5.318	-6.119	-5.456	-4.010	-5.174	-.524	-4.784	-4.788	-3.884	-3.633	-3.021	-1.001
**v19_1**	-1.376	1.674	2.798	-4.594	-5.522	-4.739	-1.198	-2.255	1.932	-1.930	-1.960	-1.129	-.882	-.241	1.540
**v20_1**	-.021	2.890	3.960	-4.174	-5.174	-4.323	.187	-.826	3.173	-.525	-.564	.236	.518	1.119	2.754
**vvv6_1**	2.756	4.666	5.365	-2.699	-3.923	-2.856	2.923	2.260	4.851	2.447	2.412	2.942	3.157	3.540	4.619
**v21_1**	-2.947	.177	1.331	-5.047	-5.897	-5.187	-2.817	-3.893	.381	-3.547	-3.564	-2.723	-2.533	-1.878	-.040
**v22_1**	1.553	4.048	4.963	-3.544	-4.647	-3.697	1.762	.893	4.305	1.142	1.100	1.791	2.065	2.573	4.002
**v23_1**	.051	2.789	3.795	-4.125	-5.133	-4.274	.248	-.703	3.046	-.421	-.459	.294	.559	1.121	2.704
**v24_1**	1.678	4.221	5.153	-3.518	-4.627	-3.673	1.894	1.008	4.490	1.261	1.217	1.922	2.206	2.724	4.183
**i1_1**	61.168	66.473	68.229	8.778	5.462	8.477	65.485	62.981	72.498	62.628	61.567	63.790	69.552	70.494	73.466
**i2_1**	60.615	66.011	67.796	8.469	5.206	8.172	65.038	62.481	72.209	62.115	61.028	63.296	69.227	70.186	73.222
**i3_1**	56.130	61.347	63.088	7.724	4.596	7.435	60.103	57.684	66.906	57.400	56.426	58.559	63.863	64.803	67.747
**i4_1**	54.078	59.226	60.950	7.354	4.294	7.070	57.867	55.503	64.528	55.254	54.325	56.405	61.456	62.390	65.299
**i5_1**	48.027	53.067	54.772	6.082	3.249	5.813	51.407	49.145	57.815	48.978	48.152	50.125	54.631	55.563	58.435
**i6_1**	37.137	41.793	43.399	4.060	1.592	3.814	39.606	37.627	45.260	37.624	37.026	38.721	41.985	42.874	45.550
**i7_1**	29.308	33.864	35.457	2.295	.141	2.069	31.326	29.452	36.733	29.541	29.057	30.640	33.316	34.209	36.859
**i8_1**	38.161	42.933	44.575	4.134	1.651	3.887	40.790	38.743	46.640	38.717	38.080	39.837	43.327	44.237	46.985
**i9_1**	64.172	69.699	71.516	9.069	5.697	8.765	68.949	66.286	76.401	65.854	64.679	67.050	73.477	74.449	77.551

	a1_1	a2_1	a3_1	a4_1	a5_1	a6_1	a7_1	a8_1	a9_1	a10_1	a11_1	a12_1	a13_1	a14_1	a15_1	[illegible]
**i10_1**	62.129	67.587	69.387	8.714	5.406	8.414	66.710	64.105	74.012	63.710	62.584	64.898	71.052	72.018	75.086	[illegible]
**i11_1**	63.507	68.963	70.760	9.047	5.680	8.743	68.148	65.535	75.461	65.125	63.984	66.309	72.536	73.497	76.556	[illegible]
**i12_1**	62.862	68.413	70.240	8.713	5.403	8.413	67.616	64.946	75.098	64.523	63.355	65.727	72.136	73.117	76.242	[illegible]
**i13_1**	29.754	34.456	36.097	2.221	.078	1.996	31.913	29.962	37.548	30.038	29.520	31.168	34.049	34.972	37.720	[illegible]
**i14_1**	20.052	24.473	26.045	.229	-1.557	.027	21.524	19.781	26.613	19.982	19.636	21.078	23.039	23.933	26.543	[illegible]
**i15_1**	24.028	28.430	29.984	1.201	-.757	.988	25.682	23.919	30.795	24.074	23.681	25.153	27.343	28.219	30.794	[illegible]
**i16_1**	18.160	22.534	24.094	-.178	-1.891	-.375	19.510	17.803	24.507	18.026	17.710	19.116	20.917	21.806	24.394	[illegible]
**i17_1**	62.184	67.548	69.320	8.908	5.567	8.605	66.631	64.083	73.762	63.708	62.614	64.878	70.826	71.775	74.781	[illegible]
**i18_1**	66.077	71.487	73.262	9.765	6.271	9.453	70.760	68.160	78.011	67.735	66.582	68.903	75.158	76.104	79.117	[illegible]
**i19_1**	69.261	74.706	76.485	10.470	6.851	10.149	74.133	71.492	81.481	71.027	69.826	72.193	78.696	79.638	82.656	[illegible]
**i20_1**	68.305	73.741	75.519	10.255	6.674	9.937	73.123	70.494	80.445	70.040	68.853	71.206	77.639	78.582	81.600	[illegible]
**i21_1**	16.121	20.506	22.075	-.667	-2.293	-.858	17.373	15.676	22.357	15.924	15.633	17.024	18.699	19.597	22.205	[illegible]
**i22_1**	20.683	25.053	26.605	.428	-1.392	.224	22.156	20.432	27.180	20.626	20.279	21.707	23.663	24.542	27.114	[illegible]
**i23_1**	23.267	27.707	29.276	.977	-.942	.767	24.909	23.134	30.067	23.297	22.908	24.387	26.568	27.455	30.060	[illegible]
**i24_1**	20.583	24.967	26.524	.391	-1.423	.187	22.059	20.330	27.102	20.524	20.177	21.610	23.571	24.454	27.036	[illegible]
**ccc1_2**	-23.523	-18.517	-16.674	-9.470	-9.552	-9.582	-22.565	-23.814	-18.543	-23.027	-22.704	-21.834	-23.336	-22.336	-19.628	[illegible]
**ccc2_2**	-21.420	-17.195	-15.555	-9.328	-9.433	-9.440	-24.297	-25.814	-19.615	-23.079	-22.743	-21.866	-23.438	-22.417	-19.656	[illegible]
**ccc3_2**	-2.996	-.385	.617	-5.206	-6.029	-5.344	-2.976	-3.865	-.235	-3.993	-4.007	-3.205	-3.037	-2.412	-.662	[illegible]
**ccc4_2**	-10.449	-6.746	-5.354	-6.064	-6.641	-6.169	-10.683	-11.910	-6.933	-10.549	-10.509	-9.651	-10.662	-9.844	-7.574	[illegible]
**ccc5_2**	-20.031	-17.164	-16.079	-10.202	-10.164	-10.285	-22.914	-24.259	-19.213	-23.498	-23.255	-22.476	-20.548	-19.922	-18.201	[illegible]
**ccc6_2**	-22.407	-18.278	-16.853	-9.833	-9.853	-9.920	-19.904	-20.855	-16.998	-22.663	-22.426	-21.633	-20.058	-19.401	-17.597	[illegible]
**ccc7_2**	-21.295	-17.091	-15.461	-9.324	-9.430	-9.436	-22.472	-23.732	-18.407	-22.934	-22.605	-21.729	-24.836	-23.614	-20.520	[illegible]
**ccc8_2**	-19.518	-16.633	-15.541	-10.022	-10.014	-10.107	-19.889	-20.809	-17.087	-22.716	-22.489	-21.712	-21.943	-21.073	-18.941	[illegible]
**ccc9_2**	-2.822	.103	1.182	-5.037	-5.889	-5.177	-2.425	-3.300	.267	-3.377	-3.396	-2.607	-2.208	-1.664	-.095	[illegible]
**ccc10_2**	-1.654	.842	1.795	-4.756	-5.657	-4.899	-1.738	-2.714	1.143	-2.411	-2.437	-1.669	-1.327	-.799	.718	
**ccc11_2**	-19.214	-16.674	-15.718	-10.603	-10.516	-10.682	-19.464	-20.283	-16.997	-19.922	-19.846	-19.232	-21.380	-20.610	-18.732	[illegible]
**ccc12_2**	-18.395	-15.664	-14.634	-9.967	-9.976	-10.052	-20.503	-21.697	-17.169	-19.168	-19.082	-18.423	-18.748	-18.159	-16.534	[illegible]
**ccc13_2**	.693	3.052	3.917	-3.808	-4.866	-3.959	.874	.056	3.266	.268	.236	.829	1.146	1.625	2.973	[illegible]
**ccc14_2**	-9.710	-6.154	-4.821	-5.947	-6.539	-6.052	-9.879	-11.063	-6.279	-10.592	-10.528	-9.630	-9.810	-9.033	-6.873	[illegible]
**ccc15_2**	-20.594	-16.915	-15.636	-9.852	-9.879	-9.939	-18.532	-19.423	-15.833	-19.025	-18.936	-18.270	-18.603	-18.005	-16.355	[illegible]
**vvv1_2**	-2.059	.031	.839	-4.971	-5.836	-5.110	-2.340	-3.200	.192	-2.932	-2.953	-2.275	-2.097	-1.583	-.140	[illegible]
**v1_2**	3.268	5.608	6.460	-2.833	-4.051	-2.993	3.436	2.626	5.797	2.851	2.805	3.451	3.736	4.204	5.524	[illegible]
**v2_2**	-3.578	-.455	.740	-5.237	-6.053	-5.375	-3.565	-4.670	-.270	-4.307	-4.317	-3.456	-3.294	-2.615	-.713	[illegible]
**v3_2**	2.450	4.877	5.873	-3.225	-4.382	-3.383	2.677	1.804	5.232	2.049	2.002	2.698	2.995	3.503	4.934	[illegible]
**v4_2**	2.594	5.105	5.932	-3.177	-4.342	-3.335	2.825	1.949	5.387	2.194	2.146	2.845	3.146	3.655	5.091	[illegible]
**vvv2_2**	-14.961	-10.821	-9.243	-6.755	-7.224	-6.854	-15.611	-16.930	-11.466	-16.266	-16.073	-15.112	-15.931	-14.972	-12.337	[illegible]
**v5_2**	6.345	8.178	8.847	-.979	-2.490	-1.156	6.541	5.900	8.399	6.071	6.028	6.544	6.793	7.158	8.189	[illegible]
**v6_2**	4.975	6.809	7.479	-1.566	-2.989	-1.767	5.157	4.518	7.011	4.692	4.653	5.166	5.398	5.764	6.796	[illegible]
**v7_2**	1.602	3.657	4.409	-3.311	-4.348	-3.468	1.769	1.057	3.845	1.262	1.228	1.795	2.013	2.427	3.592	[illegible]
**v8_2**	2.520	4.720	5.525	-3.025	-4.211	-3.118	2.715	1.949	4.947	2.165	2.125	2.736	2.989	3.432	4.682	[illegible]
**vvv3_2**	-.940	1.513	2.414	-4.428	-5.383	-4.572	-.667	-1.371	1.501	-1.374	-1.403	-.733	-.522	-.021	1.387	
**v9_2**	-.526	2.355	3.415	-4.328	-5.301	-4.475	-.347	-1.395	2.696	-1.033	-1.068	-.279	-.017	.579	2.253	
**v10_2**	-3.443	-.229	.960	-5.174	-6.001	-5.313	-3.182	-4.462	-.036	-4.074	-4.086	-3.224	-3.053	-2.375	-.476	[illegible]
**v11_2**	-6.339	-2.878	-1.590	-5.884	-6.585	-6.015	-6.368	-7.069	-2.808	-7.099	-7.078	-6.174	-6.150	-5.406	-3.329	[illegible]
**v12_2**	1.002	3.713	4.708	-3.805	-4.867	-3.958	1.220	.275	3.892	.548	.505	1.255	1.544	2.099	3.663	[illegible]
**vvv4_2**	.241	2.429	3.232	-3.927	-4.963	-4.075	.400	-.356	2.610	-.115	-.143	.378	.646	1.089	2.335	
**v13_2**	1.885	4.364	5.273	-3.418	-4.542	-3.573	2.099	1.235	4.626	1.525	1.478	2.185	2.405	2.909	4.328	[illegible]
**v14_2**	.341	2.933	3.885	-3.999	-5.027	-4.148	.534	-.366	3.175	-.098	-.139	.575	.831	1.361	2.852	[illegible]
**v15_2**	2.979	5.171	5.972	-2.835	-4.049	-2.994	3.180	2.417	5.405	2.631	2.526	3.201	3.458	3.899	5.143	[illegible]
**v16_2**	1.303	3.803	4.720	-3.635	-4.723	-3.788	1.507	.637	4.055	.888	.846	1.493	1.808	2.317	3.750	[illegible]
**vvv5_2**	-.639	1.764	2.647	-4.312	-5.287	-4.458	-.480	-1.310	1.954	-1.061	-1.091	-.434	-.192	.237	1.473	
**v17_2**	-.739	2.209	3.293	-4.399	-5.360	-4.545	-.548	-1.571	2.473	-1.261	-1.296	-.489	-.235	.392	2.144	
**v18_2**	-3.709	-.406	.817	-5.231	-6.048	-5.370	-3.605	-4.740	-.215	-4.365	-4.374	-3.491	-3.227	-2.630	-.671	[illegible]
**v19_2**	-.498	2.391	3.454	-4.320	-5.295	-4.467	-.305	-1.308	2.655	-1.007	-1.042	-.250	.013	.597	2.290	
**v20_2**	2.233	4.751	5.674	-3.311	-4.454	-3.468	2.458	1.579	5.029	1.827	1.781	2.481	2.775	3.286	4.651	[illegible]
**vvv6_2**	3.055	4.854	5.512	-2.452	-3.707	-2.610	3.213	2.589	5.026	2.765	2.732	3.231	3.434	3.795	4.808	[illegible]
**v21_2**	-.938	1.806	2.815	-4.449	-5.402	-4.595	-.765	-1.714	2.033	-1.427	-1.458	-.708	-.472	.093	1.682	
**v22_2**	.580	3.136	4.074	-3.907	-4.951	-4.058	.775	-.113	3.378	.147	.109	.813	1.071	1.593	3.062	[illegible]
**v23_2**	3.352	5.496	6.280	-2.648	-3.890	-2.808	3.553	2.805	5.729	3.013	2.971	3.568	3.827	4.259	5.475	[illegible]
**v24_2**	4.806	6.764	7.479	-1.840	-3.201	-2.006	5.002	4.319	6.986	4.505	4.463	5.011	5.262	5.653	6.758	[illegible]
**ccc1_3**	-23.089	-18.207	-16.293	-9.198	-9.329	-9.307	-22.746	-24.024	-18.600	-23.193	-22.842	-21.958	-23.617	-22.573	-19.752	[illegible]
**ccc2_3**	-21.622	-17.241	-15.536	-9.064	-9.215	-9.172	-24.437	-25.922	-19.690	-23.398	-23.024	-22.133	-23.916	-22.841	-19.942	[illegible]
**ccc3_3**	-3.539	-.805	.241	-5.352	-6.149	-5.489	-3.506	-4.428	-.670	-4.616	-4.625	-3.794	-3.647	-2.993	-1.161	[illegible]
**ccc4_3**	-10.072	-6.434	-5.068	-6.000	-6.579	-6.106	-10.274	-11.482	-6.589	-10.195	-10.111	-9.326	-10.229	-9.429	-7.208	[illegible]
**ccc5_3**	-19.495	-16.472	-15.325	-9.780	-9.808	-9.867	-22.081	-23.370	-18.343	-22.572	-22.510	-21.487	-20.079	-19.414	-17.587	[illegible]
**ccc6_3**	-19.695	-15.998	-14.580	-9.273	-9.387	-9.366	-18.414	-19.402	-15.393	-20.397	-20.318	-19.375	-18.550	-17.870	-16.001	[illegible]
**ccc7_3**	-21.327	-17.079	-15.431	-9.235	-9.361	-9.344	-22.539	-23.809	-18.433	-22.996	-22.658	-21.776	-24.769	-23.676	-20.507	[illegible]
**ccc8_3**	-19.170	-16.336	-15.264	-10.012	-10.008	-10.097	-19.512	-20.418	-16.756	-22.117	-22.083	-21.096	-21.289	-20.617	-18.484	[illegible]
**ccc9_3**	-3.283	-.258	.860	-5.160	-5.991	-5.299	-2.841	-3.744	-.071	-3.868	-3.883	-3.070	-2.649	-2.070	-.454	[illegible]
**ccc10_3**	-2.577	.032	1.031	-5.061	-5.908	-5.200	-2.778	-3.789	.221	-3.467	-3.485	-2.692	-2.299	-1.737	-.163	[illegible]
**ccc11_3**	-19.164	-16.820	-15.940	-11.032	-10.889	-11.107	-19.360	-20.119	-17.082	-19.796	-19.735	-19.161	-21.081	-20.537	-18.732	[illegible]
**ccc12_3**	-18.515	-15.847	-14.841	-10.120	-10.107	-10.205	-20.688	-21.838	-17.390	-19.264	-19.183	-18.538	-18.844	-18.269	-16.685	[illegible]

	a1_1	a2_1	a3_1	a4_1	a5_1	a6_1	a7_1	a8_1	a9_1	a10_1	a11_1	a12_1	a13_1	a14_1	a15_1
**ccc13_3**	2.069	4.189	4.965	-3.155	-4.315	-3.310	2.249	1.513	4.395	1.580	1.541	2.096	2.506	2.934	4.137
**ccc14_3**	-8.246	-4.933	-3.697	-5.649	-6.271	-5.756	-8.315	-9.428	-4.962	-9.012	-8.976	-8.123	-8.179	-7.467	-5.483
**ccc15_3**	-19.103	-15.811	-14.550	-9.741	-9.792	-9.829	-17.614	-18.483	-14.994	-18.106	-18.031	-17.378	-17.650	-17.075	-15.485
**vvv1_3**	-2.252	-.090	.745	-5.034	-5.888	-5.172	-2.540	-3.420	.053	-3.144	-3.165	-2.472	-2.296	-1.768	-.289
**v1_3**	.311	3.110	4.123	-4.041	-5.063	-4.191	.504	-.444	3.294	-.165	-.204	.547	.818	1.379	2.955
**v2_3**	-1.498	1.323	2.376	-4.647	-5.566	-4.790	-1.382	-2.373	1.547	-2.068	-2.096	-1.315	-1.088	-.494	1.175
**v3_3**	2.632	4.953	5.940	-3.115	-4.289	-3.273	2.857	2.012	5.325	2.249	2.203	2.877	3.165	3.656	5.038
**v4_3**	5.456	7.405	8.011	-1.534	-2.942	-1.703	5.662	4.983	7.635	5.166	5.122	5.668	5.925	6.314	7.410
**vvv2_3**	-15.073	-10.914	-9.328	-6.774	-7.237	-6.875	-15.739	-17.062	-11.576	-16.392	-16.194	-15.232	-16.074	-15.108	-12.457
**v5_3**	5.921	7.824	8.519	-1.289	-2.755	-1.463	6.122	5.457	8.053	5.635	5.591	6.126	6.382	6.762	7.834
**v6_3**	5.778	7.495	8.122	-1.016	-2.497	-1.203	5.952	5.353	7.687	5.516	5.478	5.959	6.180	6.523	7.487
**v7_3**	-.014	2.264	3.100	-4.049	-4.973	-4.198	.147	-.640	2.452	-.407	-.439	.186	.401	.864	2.164
**v8_3**	3.954	5.961	6.695	-2.267	-3.567	-2.390	4.146	3.447	6.180	3.640	3.599	4.159	4.406	4.808	5.943
**vvv3_3**	-2.287	.399	1.389	-4.893	-5.770	-5.033	-1.815	-2.568	.506	-2.784	-2.807	-2.079	-1.886	-1.332	.223
**v9_3**	-.931	2.024	3.111	-4.458	-5.409	-4.604	-.774	-1.845	2.348	-1.458	-1.491	-.684	-.429	.184	1.906
**v10_3**	-4.069	-.744	.487	-5.325	-6.125	-5.462	-3.810	-5.139	-.569	-4.739	-4.744	-3.857	-3.714	-3.008	-1.033
**v11_3**	-6.218	-2.722	-1.421	-5.841	-6.549	-5.972	-6.242	-7.020	-2.646	-6.985	-6.964	-6.051	-6.024	-5.271	-3.170
**v12_3**	.320	3.144	4.182	-4.053	-5.073	-4.203	.530	-.453	3.339	-.164	-.204	.574	.857	1.438	3.075
**vvv4_3**	.904	2.961	3.715	-3.604	-4.690	-3.754	1.062	.350	3.137	.485	.455	.959	1.298	1.714	2.881
**v13_3**	1.740	4.197	5.097	-3.461	-4.578	-3.615	1.948	1.093	4.451	1.381	1.339	2.036	2.249	2.748	4.154
**v14_3**	1.887	4.193	5.037	-3.334	-4.469	-3.489	2.083	1.281	4.425	1.466	1.471	2.106	2.364	2.831	4.145
**v15_3**	1.005	3.435	4.326	-3.717	-4.791	-3.869	1.197	.353	3.668	.597	.540	1.229	1.483	1.978	3.368
**v16_3**	3.979	6.002	6.742	-2.273	-3.570	-2.436	4.174	3.468	6.224	3.661	3.624	4.093	4.436	4.842	5.985
**vvv5_3**	-.749	1.677	2.568	-4.356	-5.323	-4.501	-.590	-1.428	1.867	-1.176	-1.206	-.543	-.289	.145	1.394
**v17_3**	-1.737	1.348	2.486	-4.699	-5.609	-4.843	-1.569	-2.636	1.596	-2.304	-2.331	-1.493	-1.294	-.631	1.223
**v18_3**	-1.472	1.517	2.618	-4.623	-5.546	-4.767	-1.300	-2.335	1.761	-2.016	-2.045	-1.231	-.968	-.371	1.374
**v19_3**	-2.389	.766	1.931	-4.884	-5.761	-5.026	-2.240	-3.330	.998	-2.985	-3.006	-2.153	-1.936	-1.242	.579
**v20_3**	2.213	4.721	5.639	-3.313	-4.455	-3.469	2.436	1.562	4.996	1.809	1.763	2.460	2.750	3.261	4.620
**vvv6_3**	4.265	5.834	6.407	-1.543	-2.910	-1.704	4.409	3.864	5.986	4.016	3.985	4.422	4.605	4.917	5.797
**v21_3**	.187	2.814	3.779	-4.059	-5.078	-4.208	.379	-.533	3.057	-.264	-.301	.422	.678	1.216	2.729
**v22_3**	1.936	4.317	5.190	-3.354	-4.487	-3.509	2.140	1.311	4.563	1.547	1.505	2.166	2.433	2.916	4.276
**v23_3**	-.188	2.529	3.527	-4.200	-5.195	-4.348	.002	-.940	2.775	-.660	-.696	.050	.306	.864	2.434
**v24_3**	4.253	6.318	7.073	-2.200	-3.510	-2.364	4.456	3.736	6.552	3.933	3.890	4.467	4.728	5.142	6.310

Bootstrap (Measurement intercepts)

Summary of Bootstrap Iterations (Measurement intercepts)

(Measurement intercepts)

Iterations	Method 0	Method 1	Method 2
**1**	0	0	0
**2**	0	0	0
**3**	0	0	0
**4**	0	0	0
**5**	0	0	0
**6**	0	0	0
**7**	0	0	0
**8**	0	0	0
**9**	0	0	0
**10**	0	12	0
**11**	0	22	0
**12**	0	25	0
**13**	0	23	0
**14**	0	29	0
**15**	0	29	0
**16**	0	12	0
**17**	0	5	0
**18**	0	8	0
**19**	0	35	0
**Total**	0	200	0

0 bootstrap samples were unused because of a singular covariance matrix.
0 bootstrap samples were unused because a solution was not found.
200 usable bootstrap samples were obtained.

Bootstrap Distributions (Measurement intercepts)

ML discrepancy (implied vs sample) (Measurement intercepts)

|--------------------

```
                        3091.650   |*
                        3160.598   |**
                        3229.547   |**
                        3298.495   |*********
                        3367.443   |*************
                        3436.391   |***********
                        3505.340   |***************
N = 200                 3574.288   |******************
Mean = 3524.158         3643.236   |************
S. e. = 12.139          3712.184   |*********
                        3781.132   |****
                        3850.081   |****
                        3919.029   |*
                        3987.977   |
                        4056.925   |*
                                   |--------------------
```

ML discrepancy (implied vs pop) (Measurement intercepts)

```
                                   |--------------------
                        2669.319   |**
                        2687.077   |******
                        2704.835   |***********
                        2722.593   |***************
                        2740.351   |*************
                        2758.109   |************
                        2775.867   |***********
N = 200                 2793.625   |**************
Mean = 2759.034         2811.383   |*******
S. e. = 3.411           2829.141   |****
                        2846.899   |***
                        2864.657   |***
                        2882.416   |
                        2900.174   |*
                        2917.932   |*
                                   |--------------------
```

K-L overoptimism (unstabilized) (Measurement intercepts)

```
                                   |--------------------
                       -1490.133   |*
                       -1167.903   |*
                        -845.673   |***
                        -523.442   |******
                        -201.212   |********
                         121.018   |*****************
                         443.248   |****************
N = 200                  765.479   |********************
Mean = 605.230          1087.709   |**********
S. e. = 52.332          1409.939   |**********
                        1732.169   |*******
                        2054.400   |**
                        2376.630   |**
                        2698.860   |
                        3021.090   |*
                                   |--------------------
```

K-L overoptimism (stabilized) (Measurement intercepts)

```
                                   |--------------------
                         156.576   |*
                         224.645   |****
                         292.715   |****
                         360.784   |********
                         428.853   |**********
                         496.923   |***********
                         564.992   |******************
N = 200                  633.062   |*****************
Mean = 585.756           701.131   |**********
S. e. = 12.822           769.200   |*********
                         837.270   |******
                         905.339   |****
```

```
973.409    |**
1041.478   |
1109.547   |*
           |--------------------
```

Structural covariances (Structural covariances)

Notes for Model (Structural covariances)

Computation of degrees of freedom (Structural covariances)

**Number of distinct sample moments:**	972
**Number of distinct parameters to be estimated:**	135
**Degrees of freedom (972 - 135):**	837

Result (Structural covariances)

Minimum was achieved
Chi-square = 2573.086
Degrees of freedom = 837
Probability level = .000

g1 (g1 - Structural covariances)

Estimates (g1 - Structural covariances)

Scalar Estimates (g1 - Structural covariances)

Maximum Likelihood Estimates

Regression Weights: (g1 - Structural covariances)

			Estimate	S.E.	C.R.	P	Label
BPNSF19	<---	F1	1.000				
BPNSF13	<---	F1	1.137	.051	22.120	***	a1_1
BPNSF7	<---	F1	.855	.050	17.033	***	a2_1
BPNSF1	<---	F1	.763	.050	15.221	***	a3_1
BPNSF18	<---	F2	1.000				
BPNSF15	<---	F2	2.472	.310	7.981	***	a4_1
BPNSF10	<---	F2	3.106	.377	8.240	***	a5_1
BPNSF5	<---	F2	2.568	.317	8.091	***	a6_1
BPNSF24	<---	F3	1.000				
BPNSF16	<---	F3	1.130	.046	24.395	***	a7_1
BPNSF12	<---	F3	1.224	.048	25.722	***	a8_1
BPNSF4	<---	F3	.850	.043	19.567	***	a9_1
BPNSF22	<---	F4	1.000				
BPNSF20	<---	F4	1.196	.049	24.557	***	a10_1
BPNSF8	<---	F4	1.200	.050	23.963	***	a11_1
BPNSF2	<---	F4	1.121	.049	23.044	***	a12_1
BPNSF21	<---	F5	1.000				
BPNSF14	<---	F5	1.094	.042	25.957	***	a13_1
BPNSF9	<---	F5	1.039	.042	24.620	***	a14_1
BPNSF3	<---	F5	.881	.042	21.077	***	a15_1
BPNSF23	<---	F6	1.000				
BPNSF17	<---	F6	.976	.035	28.242	***	a16_1
BPNSF11	<---	F6	.959	.034	28.176	***	a17_1
BPNSF6	<---	F6	.863	.035	24.658	***	a18_1

Standardized Regression Weights: (g1 - Structural covariances)

			Estimate
BPNSF19	<---	F1	.645
BPNSF13	<---	F1	.752
BPNSF7	<---	F1	.514
BPNSF1	<---	F1	.462
BPNSF18	<---	F2	.258
BPNSF15	<---	F2	.601
BPNSF10	<---	F2	.707
BPNSF5	<---	F2	.629
BPNSF24	<---	F3	.725

			Estimate
BPNSF16	<---	F3	.777
BPNSF12	<---	F3	.821
BPNSF4	<---	F3	.623
BPNSF22	<---	F4	.708
BPNSF20	<---	F4	.784
BPNSF8	<---	F4	.758
BPNSF2	<---	F4	.725
BPNSF21	<---	F5	.702
BPNSF14	<---	F5	.778
BPNSF9	<---	F5	.725
BPNSF3	<---	F5	.643
BPNSF23	<---	F6	.812
BPNSF17	<---	F6	.744
BPNSF11	<---	F6	.758
BPNSF6	<---	F6	.699

Intercepts: (g1 - Structural covariances)

	Estimate	S.E.	C.R.	P	Label
**BPNSF19**	5.161	.041	127.263	***	i1_1
**BPNSF13**	5.068	.039	129.843	***	i2_1
**BPNSF7**	4.834	.041	118.944	***	i3_1
**BPNSF1**	4.719	.041	114.400	***	i4_1
**BPNSF18**	4.323	.041	104.415	***	i5_1
**BPNSF15**	3.715	.046	80.790	***	i6_1
**BPNSF10**	3.176	.046	68.997	***	i7_1
**BPNSF5**	3.738	.044	84.870	***	i8_1
**BPNSF24**	5.223	.038	138.988	***	i9_1
**BPNSF16**	5.115	.038	133.720	***	i10_1
**BPNSF12**	5.218	.039	134.852	***	i11_1
**BPNSF4**	5.114	.037	139.031	***	i12_1
**BPNSF22**	3.120	.043	72.371	***	i13_1
**BPNSF20**	2.508	.046	54.169	***	i14_1
**BPNSF8**	2.806	.048	58.739	***	i15_1
**BPNSF2**	2.383	.047	51.118	***	i16_1
**BPNSF21**	5.200	.040	130.424	***	i17_1
**BPNSF14**	5.464	.040	135.863	***	i18_1
**BPNSF9**	5.679	.040	140.701	***	i19_1
**BPNSF3**	5.610	.040	139.703	***	i20_1
**BPNSF23**	2.241	.046	49.111	***	i21_1
**BPNSF17**	2.577	.047	54.540	***	i22_1
**BPNSF11**	2.744	.047	58.985	***	i23_1
**BPNSF6**	2.565	.047	54.736	***	i24_1

Covariances: (g1 - Structural covariances)

			Estimate	S.E.	C.R.	P	Label
F1	<-->	F2	-.156	.024	-6.584	***	ccc1_1
F2	<-->	F3	-.149	.022	-6.643	***	ccc2_1
F1	<-->	F3	.728	.048	15.066	***	ccc3_1
F2	<-->	F4	.314	.041	7.635	***	ccc4_1
F3	<-->	F4	-.596	.045	-13.381	***	ccc5_1
F1	<-->	F4	-.490	.043	-11.286	***	ccc6_1
F2	<-->	F5	-.143	.023	-6.324	***	ccc7_1
F4	<-->	F5	-.668	.049	-13.722	***	ccc8_1
F3	<-->	F5	.770	.049	15.782	***	ccc9_1
F1	<-->	F5	.903	.056	16.114	***	ccc10_1
F6	<-->	F5	-.958	.060	-15.868	***	ccc11_1
F6	<-->	F3	-.641	.050	-12.931	***	ccc12_1
F6	<-->	F4	1.266	.073	17.426	***	ccc13_1
F6	<-->	F2	.372	.048	7.715	***	ccc14_1
F6	<-->	F1	-.622	.052	-12.029	***	ccc15_1

Correlations: (g1 - Structural covariances)

			Estimate
F1	<-->	F2	-.433
F2	<-->	F3	-.411
F1	<-->	F3	.836
F2	<-->	F4	.758

	Estimate
F3 <--> F4	-.595
F1 <--> F4	-.494
F2 <--> F5	-.363
F4 <--> F5	-.615
F3 <--> F5	.808
F1 <--> F5	.956
F6 <--> F5	-.723
F6 <--> F3	-.525
F6 <--> F4	.910
F6 <--> F2	.737
F6 <--> F1	-.514

Variances: (g1 - Structural covariances)

	Estimate	S.E.	C.R.	P	Label
**F1**	.863	.072	12.009	***	vvv1_1
**F2**	.150	.036	4.231	***	vvv2_1
**F3**	.879	.065	13.450	***	vvv3_1
**F4**	1.141	.085	13.446	***	vvv4_1
**F5**	1.033	.073	14.100	***	vvv5_1
**F6**	1.696	.103	16.468	***	vvv6_1
**e1**	1.211	.090	13.434	***	v1_1
**e2**	.855	.071	12.103	***	v2_1
**e3**	1.759	.124	14.144	***	v3_1
**e4**	1.859	.130	14.309	***	v4_1
**e5**	2.109	.145	14.555	***	v5_1
**e6**	1.623	.127	12.807	***	v6_1
**e7**	1.452	.129	11.246	***	v7_1
**e8**	1.513	.121	12.501	***	v8_1
**e9**	.791	.063	12.627	***	v9_1
**e10**	.737	.062	11.819	***	v10_1
**e11**	.635	.059	10.739	***	v11_1
**e12**	.998	.074	13.570	***	v12_1
**e13**	1.137	.087	13.127	***	v13_1
**e14**	1.020	.084	12.103	***	v14_1
**e15**	1.214	.097	12.530	***	v15_1
**e16**	1.295	.100	12.953	***	v16_1
**e17**	1.065	.079	13.444	***	v17_1
**e18**	.806	.064	12.585	***	v18_1
**e19**	1.005	.076	13.236	***	v19_1
**e20**	1.138	.082	13.823	***	v20_1
**e21**	.873	.073	11.899	***	v21_1
**e22**	1.303	.100	12.983	***	v22_1
**e23**	1.156	.090	12.815	***	v23_1
**e24**	1.322	.099	13.400	***	v24_1

Matrices (g1 - Structural covariances)

Residual Covariances (g1 - Structural covariances)

	BPNSF6	BPNSF11	BPNSF17	BPNSF23	BPNSF3	BPNSF9	BPNSF14	BPNSF21	BPNSF2	BPNSF8	BPNSF20	BPNSF22	BPNSF4	BPNSF12	BPNSF16	BPNS[illegible]
**BPNSF6**	.070															
**BPNSF11**	.063	-.032														
**BPNSF17**	-.019	-.188	-.362													
**BPNSF23**	-.089	-.165	-.329	-.227												
**BPNSF3**	-.131	.033	.077	-.137	-.052											
**BPNSF9**	-.050	.105	.115	-.004	-.009	-.191										
**BPNSF14**	.100	.286	.165	.126	-.066	-.082	-.023									
**BPNSF21**	.139	.189	.350	.071	-.169	-.164	-.037	-.060								
**BPNSF2**	.040	.018	-.299	.021	-.280	-.105	-.081	-.071	-.078							
**BPNSF8**	.135	.041	-.212	-.330	.115	.006	.245	.379	-.126	-.151						
**BPNSF20**	-.120	-.264	-.196	-.066	-.110	.021	.036	.063	-.202	-.333	-.355					
**BPNSF22**	-.182	-.182	-.439	-.189	-.001	.153	.312	.242	-.152	-.061	-.262	-.206				
**BPNSF4**	-.033	.154	.170	.191	.050	-.234	-.022	-.230	.010	.230	.138	.146	-.171			
**BPNSF12**	.066	.359	.207	.279	-.227	-.270	-.138	-.275	.196	.339	.266	.142	-.240	-.254		
**BPNSF16**	-.067	.139	.094	.065	-.208	-.185	-.136	-.170	.151	.224	.188	.185	-.203	-.215	-.221	
**BPNSF24**	.042	.170	.098	.084	-.066	-.050	-.249	-.226	-.051	.171	.082	.005	-.167	-.166	-.157	-.1[illegible]
**BPNSF5**	-.039	.017	-.285	-.305	.139	.088	.188	.085	-.117	-.193	-.351	-.195	.283	.051	-.033	-.0[illegible]
**BPNSF10**	-.110	.287	-.332	-.394	.182	.082	.312	.171	-.183	-.077	-.518	-.422	.144	.122	.143	.2[illegible]
**BPNSF15**	-.192	-.103	-.340	-.552	.102	.208	.554	.157	-.494	-.266	-.466	-.326	.184	.089	.310	.1[illegible]
**BPNSF18**	-.478	-.295	-.254	-.437	.312	.403	.213	.532	-.301	-.231	-.467	-.183	.153	.291	.361	.2[illegible]

	BPNSF6	BPNSF11	BPNSF17	BPNSF23	BPNSF3	BPNSF9	BPNSF14	BPNSF21	BPNSF2	BPNSF8	BPNSF20	BPNSF22	BPNSF4	BPNSF12	BPNSF16	BPNS[illegible]
**BPNSF1**	.130	.186	.377	.229	.001	-.323	-.395	-.102	.255	.314	.228	.191	.139	-.129	-.179	-.0[illegible]
**BPNSF7**	.190	.079	.389	.065	-.085	-.003	-.194	.107	-.085	.413	.272	.234	-.221	-.377	-.368	-.2[illegible]
**BPNSF13**	-.031	.103	.109	.038	-.179	-.217	.172	.024	-.024	.286	.019	.179	-.120	-.075	-.165	-.2[illegible]
**BPNSF19**	-.029	-.083	-.077	-.226	-.056	-.167	.036	.349	-.262	.162	-.154	.062	-.169	-.157	-.067	-.1[illegible]

Residual Means (g1 - Structural covariances)

	BPNSF6	BPNSF11	BPNSF17	BPNSF23	BPNSF3	BPNSF9	BPNSF14	BPNSF21	BPNSF2	BPNSF8	BPNSF20	BPNSF22	BPNSF4	BPNSF12	BPNSF16	BPNSF24	BP[illegible]
	-.107	-.054	-.137	-.172	.106	.168	.121	.159	-.153	-.039	-.157	-.105	-.046	.012	-.004	.021	-.[illegible]

Standardized Residual Covariances (g1 - Structural covariances)

	BPNSF6	BPNSF11	BPNSF17	BPNSF23	BPNSF3	BPNSF9	BPNSF14	BPNSF21	BPNSF2	BPNSF8	BPNSF20	BPNSF22	BPNSF4	BPNSF12	BPNSF16	BPNS[illegible]
**BPNSF6**	.398															
**BPNSF11**	.441	-.173														
**BPNSF17**	-.131	-1.215	-1.834													
**BPNSF23**	-.626	-1.113	-2.152	-1.310												
**BPNSF3**	-1.163	.286	.640	-1.201	-.396											
**BPNSF9**	-.416	.854	.898	-.033	-.085	-1.332										
**BPNSF14**	.850	2.337	1.307	1.048	-.624	-.721	-.163									
**BPNSF21**	1.178	1.545	2.766	.592	-1.596	-1.448	-.332	-.422								
**BPNSF2**	.284	.127	-1.991	.145	-2.449	-.870	-.683	-.593	-.421							
**BPNSF8**	.939	.273	-1.368	-2.226	.981	.052	1.993	3.079	-.829	-.781						
**BPNSF20**	-.862	-1.814	-1.299	-.455	-.972	.174	.304	.525	-1.364	-2.177	-1.983					
**BPNSF22**	-1.435	-1.378	-3.213	-1.447	-.012	1.393	2.868	2.219	-1.135	-.444	-1.951	-1.339				
**BPNSF4**	-.329	1.482	1.588	1.889	.564	-2.476	-.236	-2.451	.096	2.143	1.330	1.535	-1.546			
**BPNSF12**	.585	3.098	1.732	2.460	-2.249	-2.498	-1.288	-2.582	1.672	2.819	2.283	1.328	-2.503	-1.925		
**BPNSF16**	-.611	1.235	.808	.591	-2.125	-1.777	-1.308	-1.645	1.330	1.919	1.666	1.790	-2.190	-1.986	-1.758	
**BPNSF24**	.413	1.609	.891	.813	-.720	-.508	-2.568	-2.339	-.474	1.554	.772	.056	-1.926	-1.656	-1.627	-1.1[illegible]
**BPNSF5**	-.301	.132	-2.089	-2.355	1.309	.784	1.716	.769	-.882	-1.417	-2.672	-1.622	2.894	.475	-.315	-.4[illegible]
**BPNSF10**	-.793	1.988	-2.226	-2.781	1.581	.683	2.630	1.425	-1.272	-.518	-3.601	-3.213	1.361	1.042	1.254	2.0[illegible]
**BPNSF15**	-1.500	-.775	-2.483	-4.255	.949	1.853	5.016	1.408	-3.729	-1.956	-3.537	-2.700	1.870	.823	2.933	1.4[illegible]
**BPNSF18**	-4.102	-2.467	-2.048	-3.750	3.116	3.844	2.067	5.103	-2.512	-1.881	-3.942	-1.675	1.664	2.884	3.669	2.3[illegible]
**BPNSF1**	1.084	1.516	2.961	1.914	.007	-2.874	-3.559	-.920	2.074	2.489	1.880	1.705	1.437	-1.201	-1.708	-.6[illegible]
**BPNSF7**	1.576	.635	3.028	.533	-.785	-.028	-1.712	.946	-.684	3.246	2.216	2.063	-2.257	-3.441	-3.463	-2.4[illegible]
**BPNSF13**	-.274	.898	.918	.338	-1.742	-1.966	1.566	.218	-.213	2.424	.165	1.707	-1.307	-.710	-1.622	-2.1[illegible]
**BPNSF19**	-.257	-.706	-.636	-1.978	-.541	-1.526	.326	3.211	-2.246	1.356	-1.334	.579	-1.817	-1.491	-.655	-1.4[illegible]

Standardized Residual Means (g1 - Structural covariances)

	BPNSF6	BPNSF11	BPNSF17	BPNSF23	BPNSF3	BPNSF9	BPNSF14	BPNSF21	BPNSF2	BPNSF8	BPNSF20	BPNSF22	BPNSF4	BPNSF12	BPNSF16	BPNSF24	BP[illegible]
	-1.398	-.688	-1.683	-2.252	1.588	2.419	1.774	2.294	-1.942	-.478	-2.021	-1.451	-.751	.185	-.055	.336	-.[illegible]

Notes for Group/Model (g1 - Structural covariances)

The following covariance matrix is not positive definite (g1 - Structural covariances)

	F5	F4	F3	F2	F1	F6
**F5**	1.033					
**F4**	-.668	1.141				
**F3**	.770	-.596	.879			
**F2**	-.143	.314	-.149	.150		
**F1**	.903	-.490	.728	-.156	.863	
**F6**	-.958	1.266	-.641	.372	-.622	1.696

This solution is not admissible.

Modification Indices (g1 - Structural covariances)

Covariances: (g1 - Structural covariances)

			M.I.	Par Change
F4	<-->	F5	4.450	-.044
F2	<-->	F4	8.479	-.033
F2	<-->	F3	8.623	-.035
F1	<-->	F2	4.447	.024
e24	<-->	F3	4.163	-.081
e23	<-->	F2	29.513	.106
e21	<-->	F4	9.145	.101
e21	<-->	F3	4.729	.074

			M.I.	Par Change
e21	<-->	F2	16.018	-.070
e21	<-->	F1	4.744	-.073
e19	<-->	F1	11.861	-.116
e19	<-->	e20	4.632	.119
e18	<-->	F2	5.315	.038
e17	<-->	F3	8.752	-.105
e17	<-->	F1	25.730	.175
e17	<-->	e22	5.629	.148
e17	<-->	e20	5.406	-.132
e16	<-->	F5	8.470	-.104
e16	<-->	F3	4.255	.082
e16	<-->	e22	7.517	-.192
e16	<-->	e21	5.713	.142
e16	<-->	e20	4.971	-.142
e15	<-->	F2	4.088	.041
e15	<-->	F1	9.466	.120
e15	<-->	e24	6.992	.181
e15	<-->	e23	5.635	.155
e15	<-->	e21	15.979	-.234
e15	<-->	e19	9.497	-.185
e15	<-->	e17	4.621	.132
e14	<-->	F2	14.393	-.071
e14	<-->	e23	10.201	-.194
e14	<-->	e22	4.336	.133
e14	<-->	e21	18.738	.235
e13	<-->	e22	5.502	-.153
e13	<-->	e18	5.504	.122
e13	<-->	e15	6.037	.156
e12	<-->	e20	10.668	.179
e12	<-->	e19	4.460	-.111
e12	<-->	e18	4.680	.104
e12	<-->	e17	4.152	-.109
e11	<-->	F2	13.873	-.059
e11	<-->	F6	4.390	.074
e11	<-->	e23	5.166	.115
e11	<-->	e21	5.206	.104
e11	<-->	e16	4.058	.108
e10	<-->	F4	6.534	.081
e10	<-->	F6	8.907	-.108
e10	<-->	e16	5.360	.128
e9	<-->	e19	12.085	.168
e9	<-->	e18	12.284	-.154
e8	<-->	e12	12.402	.232
e8	<-->	e10	4.944	-.134
e7	<-->	F1	4.450	.093
e7	<-->	e23	31.338	.417
e7	<-->	e15	6.019	.189
e7	<-->	e14	8.780	-.212
e7	<-->	e13	8.563	-.214
e7	<-->	e9	4.203	.127
e6	<-->	F4	4.709	-.098
e6	<-->	F2	11.222	.072
e6	<-->	e21	8.473	-.195
e6	<-->	e18	18.531	.269
e6	<-->	e16	10.027	-.249
e6	<-->	e11	8.800	-.178
e6	<-->	e10	6.979	.163
e6	<-->	e7	8.060	.245
e5	<-->	F1	9.979	.151
e5	<-->	e24	7.737	-.234
e5	<-->	e18	12.678	-.240
e5	<-->	e17	7.201	.202
e5	<-->	e6	5.103	.214
e4	<-->	F4	8.941	.138
e4	<-->	F3	7.970	.129
e4	<-->	F2	31.977	-.133
e4	<-->	e20	9.360	.223
e4	<-->	e18	13.611	-.235
e4	<-->	e16	7.714	.223
e4	<-->	e12	17.073	.284
e4	<-->	e7	7.305	-.242
e4	<-->	e6	16.207	-.362

			M.I.	Par Change
e3	<-->	F3	21.943	-.208
e3	<-->	F1	14.182	.159
e3	<-->	e22	7.077	.209
e3	<-->	e18	8.315	-.179
e3	<-->	e17	5.400	.162
e3	<-->	e16	8.141	-.224
e3	<-->	e11	6.744	-.155
e3	<-->	e10	5.318	-.142
e3	<-->	e7	4.750	.191
e3	<-->	e4	4.331	.186
e2	<-->	F2	16.566	.069
e2	<-->	e20	7.532	-.144
e2	<-->	e19	9.216	-.152
e2	<-->	e18	23.167	.219
e2	<-->	e11	5.508	.103
e2	<-->	e9	4.109	-.093
e2	<-->	e8	4.808	.139
e2	<-->	e6	4.645	.140
e2	<-->	e3	6.601	.166
e1	<-->	e21	6.413	-.143
e1	<-->	e19	6.479	-.147
e1	<-->	e17	35.705	.352
e1	<-->	e15	4.330	.136
e1	<-->	e7	4.999	.166
e1	<-->	e5	9.136	.243
e1	<-->	e4	4.569	-.162

Variances: (g1 - Structural covariances)

	M.I.	Par Change

Regression Weights: (g1 - Structural covariances)

			M.I.	Par Change
BPNSF6	<---	F3	4.170	-.134
BPNSF11	<---	F2	17.023	.657
BPNSF11	<---	BPNSF8	4.896	.037
BPNSF11	<---	BPNSF5	4.780	.030
BPNSF11	<---	BPNSF10	15.811	.061
BPNSF11	<---	BPNSF15	5.209	.031
BPNSF23	<---	F2	5.386	-.332
BPNSF23	<---	BPNSF8	6.243	-.038
BPNSF23	<---	BPNSF10	5.999	-.034
BPNSF23	<---	BPNSF15	6.688	-.032
BPNSF14	<---	F2	4.746	.291
BPNSF21	<---	BPNSF17	4.649	.036
BPNSF21	<---	BPNSF19	5.093	.022
BPNSF2	<---	F5	12.118	-.207
BPNSF2	<---	F1	10.771	-.217
BPNSF2	<---	BPNSF3	4.936	-.022
BPNSF2	<---	BPNSF14	4.703	-.022
BPNSF2	<---	BPNSF21	4.940	-.024
BPNSF2	<---	BPNSF15	4.627	-.031
BPNSF2	<---	BPNSF7	6.237	-.028
BPNSF2	<---	BPNSF19	4.755	-.024
BPNSF8	<---	F5	4.346	.122
BPNSF8	<---	F1	5.124	.147
BPNSF8	<---	BPNSF6	4.102	.038
BPNSF20	<---	F2	8.096	-.437
BPNSF20	<---	BPNSF11	5.835	-.040
BPNSF20	<---	BPNSF14	4.210	-.019
BPNSF20	<---	BPNSF21	4.124	-.020
BPNSF20	<---	BPNSF8	5.634	-.038
BPNSF20	<---	BPNSF5	5.074	-.029
BPNSF20	<---	BPNSF10	9.448	-.045
BPNSF20	<---	BPNSF15	4.789	-.029
BPNSF20	<---	BPNSF18	4.944	-.026
BPNSF20	<---	BPNSF19	4.117	-.020
BPNSF12	<---	F5	4.348	-.095
BPNSF12	<---	F6	4.636	.077
BPNSF10	<---	BPNSF11	13.242	.074

			M.I.	Par Change
BPNSF10	<---	BPNSF3	7.267	.030
BPNSF10	<---	BPNSF9	5.191	.025
BPNSF10	<---	BPNSF14	6.599	.029
BPNSF10	<---	BPNSF21	6.154	.030
BPNSF10	<---	BPNSF8	6.491	.050
BPNSF10	<---	BPNSF4	4.759	.027
BPNSF10	<---	BPNSF12	4.892	.027
BPNSF10	<---	BPNSF16	5.343	.028
BPNSF10	<---	BPNSF24	7.489	.033
BPNSF10	<---	BPNSF15	8.622	.047
BPNSF10	<---	BPNSF18	4.609	.030
BPNSF10	<---	BPNSF7	9.067	.038
BPNSF10	<---	BPNSF13	7.394	.034
BPNSF10	<---	BPNSF19	8.651	.036
BPNSF15	<---	F5	7.302	.180
BPNSF15	<---	F4	5.471	-.150
BPNSF15	<---	F6	6.552	-.134
BPNSF15	<---	BPNSF14	6.269	.029
BPNSF15	<---	BPNSF16	4.122	.025
BPNSF15	<---	BPNSF10	4.527	.038
BPNSF15	<---	BPNSF18	4.187	.029
BPNSF18	<---	F5	22.421	.341
BPNSF18	<---	F4	8.820	-.206
BPNSF18	<---	F3	12.881	.286
BPNSF18	<---	F2	4.908	-.449
BPNSF18	<---	F1	18.221	.341
BPNSF18	<---	F6	11.163	-.188
BPNSF18	<---	BPNSF21	4.819	.028
BPNSF18	<---	BPNSF19	4.598	.028
BPNSF1	<---	F5	6.137	-.169
BPNSF1	<---	F6	4.149	.109
BPNSF1	<---	BPNSF3	6.155	-.028
BPNSF1	<---	BPNSF9	10.824	-.037
BPNSF1	<---	BPNSF14	13.443	-.043
BPNSF1	<---	BPNSF21	8.446	-.036
BPNSF1	<---	BPNSF22	4.277	-.039
BPNSF1	<---	BPNSF12	7.133	-.033
BPNSF1	<---	BPNSF16	7.864	-.035
BPNSF1	<---	BPNSF24	6.409	-.031
BPNSF1	<---	BPNSF5	10.061	-.052
BPNSF1	<---	BPNSF10	13.520	-.068
BPNSF1	<---	BPNSF15	16.282	-.066
BPNSF1	<---	BPNSF18	7.884	-.041
BPNSF1	<---	BPNSF7	4.856	-.029
BPNSF1	<---	BPNSF13	8.554	-.037
BPNSF1	<---	BPNSF19	10.245	-.040
BPNSF7	<---	BPNSF17	4.216	.043
BPNSF13	<---	BPNSF14	5.334	.020
BPNSF13	<---	BPNSF5	4.779	.026
BPNSF13	<---	BPNSF15	4.797	.026
BPNSF13	<---	BPNSF7	4.055	.019
BPNSF19	<---	F5	5.429	.131
BPNSF19	<---	F4	6.826	-.142
BPNSF19	<---	F6	9.934	-.139
BPNSF19	<---	BPNSF14	4.143	.020
BPNSF19	<---	BPNSF21	9.987	.032
BPNSF19	<---	BPNSF16	4.045	.021
BPNSF19	<---	BPNSF18	6.001	.029
BPNSF19	<---	BPNSF7	5.409	.025

Means: (g1 - Structural covariances)

	M.I.	Par Change

Intercepts: (g1 - Structural covariances)

	M.I.	Par Change
**BPNSF10**	5.565	.153
**BPNSF1**	7.519	-.181

Bootstrap (g1 - Structural covariances)

Bootstrap standard errors (g1 - Structural covariances)

Scalar Estimates (g1 - Structural covariances)

Regression Weights: (g1 - Structural covariances)

Parameter	SE	SE-SE	Mean	Bias	SE-Bias
BPNSF19 <--- F1	.000	.000	1.000	.000	.000
BPNSF13 <--- F1	.056	.003	1.132	-.005	.004
BPNSF7 <--- F1	.059	.003	.855	.000	.004
BPNSF1 <--- F1	.071	.004	.762	-.001	.005
BPNSF18 <--- F2	.000	.000	1.000	.000	.000
BPNSF15 <--- F2	.396	.020	2.525	.054	.028
BPNSF10 <--- F2	.522	.026	3.180	.074	.037
BPNSF5 <--- F2	.422	.021	2.629	.061	.030
BPNSF24 <--- F3	.000	.000	1.000	.000	.000
BPNSF16 <--- F3	.053	.003	1.129	-.001	.004
BPNSF12 <--- F3	.049	.002	1.218	-.006	.003
BPNSF4 <--- F3	.051	.003	.848	-.002	.004
BPNSF22 <--- F4	.000	.000	1.000	.000	.000
BPNSF20 <--- F4	.052	.003	1.195	-.001	.004
BPNSF8 <--- F4	.050	.002	1.195	-.005	.004
BPNSF2 <--- F4	.054	.003	1.120	-.001	.004
BPNSF21 <--- F5	.000	.000	1.000	.000	.000
BPNSF14 <--- F5	.045	.002	1.096	.002	.003
BPNSF9 <--- F5	.052	.003	1.041	.002	.004
BPNSF3 <--- F5	.058	.003	.885	.004	.004
BPNSF23 <--- F6	.000	.000	1.000	.000	.000
BPNSF17 <--- F6	.034	.002	.979	.003	.002
BPNSF11 <--- F6	.038	.002	.959	.000	.003
BPNSF6 <--- F6	.037	.002	.862	.000	.003

Standardized Regression Weights: (g1 - Structural covariances)

Parameter	SE	SE-SE	Mean	Bias	SE-Bias
BPNSF19 <--- F1	.033	.002	.651	.006	.002
BPNSF13 <--- F1	.026	.001	.752	-.001	.002
BPNSF7 <--- F1	.033	.002	.514	.000	.002
BPNSF1 <--- F1	.030	.002	.462	.000	.002
BPNSF18 <--- F2	.039	.002	.259	.001	.003
BPNSF15 <--- F2	.032	.002	.603	.002	.002
BPNSF10 <--- F2	.026	.001	.707	.000	.002
BPNSF5 <--- F2	.027	.001	.631	.002	.002
BPNSF24 <--- F3	.025	.001	.728	.002	.002
BPNSF16 <--- F3	.028	.001	.780	.003	.002
BPNSF12 <--- F3	.020	.001	.821	.000	.001
BPNSF4 <--- F3	.028	.001	.622	-.002	.002
BPNSF22 <--- F4	.021	.001	.709	.001	.002
BPNSF20 <--- F4	.023	.001	.784	-.001	.002
BPNSF8 <--- F4	.026	.001	.756	-.002	.002
BPNSF2 <--- F4	.026	.001	.722	-.003	.002
BPNSF21 <--- F5	.024	.001	.703	.001	.002
BPNSF14 <--- F5	.035	.002	.782	.004	.002
BPNSF9 <--- F5	.032	.002	.730	.004	.002
BPNSF3 <--- F5	.038	.002	.648	.005	.003
BPNSF23 <--- F6	.023	.001	.813	.001	.002
BPNSF17 <--- F6	.026	.001	.747	.003	.002
BPNSF11 <--- F6	.022	.001	.757	-.001	.002
BPNSF6 <--- F6	.027	.001	.697	-.002	.002

Intercepts: (g1 - Structural covariances)

Parameter	SE	SE-SE	Mean	Bias	SE-Bias
**BPNSF19**	.044	.002	5.167	.006	.003
**BPNSF13**	.040	.002	5.074	.006	.003
**BPNSF7**	.042	.002	4.842	.008	.003
**BPNSF1**	.039	.002	4.726	.007	.003
**BPNSF18**	.042	.002	4.322	.000	.003
**BPNSF15**	.046	.002	3.714	-.001	.003
**BPNSF10**	.049	.002	3.172	-.004	.003
**BPNSF5**	.045	.002	3.735	-.003	.003

Parameter	SE	SE-SE	Mean	Bias	SE-Bias
**BPNSF24**	.040	.002	5.227	.004	.003
**BPNSF16**	.042	.002	5.121	.006	.003
**BPNSF12**	.037	.002	5.224	.006	.003
**BPNSF4**	.039	.002	5.118	.004	.003
**BPNSF22**	.045	.002	3.115	-.004	.003
**BPNSF20**	.052	.003	2.498	-.010	.004
**BPNSF8**	.052	.003	2.803	-.003	.004
**BPNSF2**	.046	.002	2.379	-.004	.003
**BPNSF21**	.039	.002	5.208	.008	.003
**BPNSF14**	.041	.002	5.473	.008	.003
**BPNSF9**	.044	.002	5.686	.006	.003
**BPNSF3**	.041	.002	5.617	.007	.003
**BPNSF23**	.044	.002	2.233	-.008	.003
**BPNSF17**	.052	.003	2.568	-.009	.004
**BPNSF11**	.048	.002	2.742	-.003	.003
**BPNSF6**	.051	.003	2.563	-.002	.004

Covariances: (g1 - Structural covariances)

Parameter	SE	SE-SE	Mean	Bias	SE-Bias
F1 <--> F2	.025	.001	-.157	-.001	.002
F2 <--> F3	.025	.001	-.150	.000	.002
F1 <--> F3	.047	.002	.730	.001	.003
F2 <--> F4	.051	.003	.314	.000	.004
F3 <--> F4	.044	.002	-.601	-.005	.003
F1 <--> F4	.043	.002	-.492	-.002	.003
F2 <--> F5	.024	.001	-.143	.000	.002
F4 <--> F5	.043	.002	-.666	.002	.003
F3 <--> F5	.052	.003	.770	.000	.004
F1 <--> F5	.075	.004	.906	.003	.005
F6 <--> F5	.058	.003	-.957	.001	.004
F6 <--> F3	.049	.002	-.645	-.005	.003
F6 <--> F4	.070	.003	1.264	-.002	.005
F6 <--> F2	.058	.003	.371	-.001	.004
F6 <--> F1	.055	.003	-.628	-.006	.004

Correlations: (g1 - Structural covariances)

Parameter	SE	SE-SE	Mean	Bias	SE-Bias
F1 <--> F2	.039	.002	-.434	-.002	.003
F2 <--> F3	.036	.002	-.410	.000	.003
F1 <--> F3	.026	.001	.833	-.003	.002
F2 <--> F4	.025	.001	.757	-.001	.002
F3 <--> F4	.027	.001	-.598	-.002	.002
F1 <--> F4	.033	.002	-.494	.000	.002
F2 <--> F5	.038	.002	-.363	.000	.003
F4 <--> F5	.028	.001	-.612	.002	.002
F3 <--> F5	.022	.001	.805	-.003	.002
F1 <--> F5	.023	.001	.954	-.002	.002
F6 <--> F5	.025	.001	-.723	.001	.002
F6 <--> F3	.031	.002	-.528	-.003	.002
F6 <--> F4	.018	.001	.909	-.002	.001
F6 <--> F2	.029	.001	.734	-.002	.002
F6 <--> F1	.033	.002	-.518	-.003	.002

Variances: (g1 - Structural covariances)

Parameter	SE	SE-SE	Mean	Bias	SE-Bias
**F1**	.081	.004	.871	.008	.006
**F2**	.047	.002	.154	.004	.003
**F3**	.065	.003	.885	.006	.005
**F4**	.080	.004	1.144	.003	.006
**F5**	.080	.004	1.037	.003	.006
**F6**	.102	.005	1.694	-.001	.007
**e1**	.136	.007	1.185	-.026	.010
**e2**	.105	.005	.856	.001	.007
**e3**	.171	.009	1.766	.006	.012
**e4**	.141	.007	1.851	-.008	.010
**e5**	.138	.007	2.093	-.016	.010
**e6**	.143	.007	1.613	-.009	.010

Parameter	SE	SE-SE	Mean	Bias	SE-Bias
**e7**	.134	.007	1.458	.006	.009
**e8**	.133	.007	1.507	-.006	.009
**e9**	.088	.004	.786	-.005	.006
**e10**	.104	.005	.725	-.012	.007
**e11**	.081	.004	.634	-.001	.006
**e12**	.089	.004	1.005	.007	.006
**e13**	.099	.005	1.134	-.003	.007
**e14**	.133	.007	1.025	.004	.009
**e15**	.153	.008	1.223	.009	.011
**e16**	.144	.007	1.315	.020	.010
**e17**	.112	.006	1.063	-.002	.008
**e18**	.153	.008	.792	-.014	.011
**e19**	.155	.008	.987	-.017	.011
**e20**	.166	.008	1.126	-.013	.012
**e21**	.124	.006	.867	-.006	.009
**e22**	.172	.009	1.291	-.012	.012
**e23**	.116	.006	1.162	.006	.008
**e24**	.152	.008	1.336	.013	.011

Matrices (g1 - Structural covariances)

Sample Covariances - Standard Errors (g1 - Structural covariances)

	BPNSF6	BPNSF11	BPNSF17	BPNSF23	BPNSF3	BPNSF9	BPNSF14	BPNSF21	BPNSF2	BPNSF8	BPNSF20	BPNSF22	BPNSF4	BPNSF12	BPNSF16	BPNS[illegible]
**BPNSF6**	.189															
**BPNSF11**	.149	.158														
**BPNSF17**	.153	.153	.188													
**BPNSF23**	.149	.150	.138	.180												
**BPNSF3**	.128	.115	.109	.108	.175											
**BPNSF9**	.116	.104	.108	.109	.116	.182										
**BPNSF14**	.124	.116	.124	.117	.105	.122	.166									
**BPNSF21**	.101	.104	.121	.111	.106	.115	.137	.163								
**BPNSF2**	.154	.150	.138	.147	.101	.110	.120	.116	.159							
**BPNSF8**	.153	.148	.155	.151	.114	.115	.111	.110	.151	.169						
**BPNSF20**	.153	.147	.159	.149	.100	.106	.131	.116	.132	.154	.166					
**BPNSF22**	.121	.128	.124	.125	.095	.091	.102	.105	.112	.126	.114	.119				
**BPNSF4**	.104	.104	.097	.085	.104	.088	.074	.096	.113	.107	.091	.090	.102			
**BPNSF12**	.117	.103	.107	.091	.094	.110	.131	.120	.107	.115	.100	.092	.085	.135		
**BPNSF16**	.122	.104	.107	.108	.098	.107	.123	.114	.109	.109	.102	.082	.080	.102	.123	
**BPNSF24**	.101	.093	.103	.097	.106	.110	.102	.105	.097	.098	.090	.092	.082	.095	.094	[illegible]
**BPNSF5**	.124	.125	.135	.113	.092	.091	.103	.109	.112	.136	.118	.109	.088	.098	.099	[illegible]
**BPNSF10**	.139	.143	.144	.134	.123	.109	.126	.121	.139	.153	.136	.126	.114	.116	.112	[illegible]
**BPNSF15**	.123	.129	.132	.115	.124	.112	.111	.125	.134	.148	.114	.123	.098	.112	.110	[illegible]
**BPNSF18**	.120	.122	.124	.110	.104	.112	.117	.113	.113	.134	.102	.112	.088	.117	.111	[illegible]
**BPNSF1**	.124	.105	.111	.094	.112	.097	.103	.110	.110	.115	.097	.096	.091	.094	.086	[illegible]
**BPNSF7**	.121	.112	.114	.093	.112	.112	.109	.118	.111	.114	.097	.101	.091	.109	.100	[illegible]
**BPNSF13**	.117	.118	.112	.112	.098	.116	.130	.133	.116	.117	.108	.095	.088	.132	.113	[illegible]
**BPNSF19**	.107	.112	.117	.115	.112	.113	.136	.142	.121	.125	.124	.106	.099	.108	.117	[illegible]

Sample Correlations - Standard Errors (g1 - Structural covariances)

	BPNSF6	BPNSF11	BPNSF17	BPNSF23	BPNSF3	BPNSF9	BPNSF14	BPNSF21	BPNSF2	BPNSF8	BPNSF20	BPNSF22	BPNSF4	BPNSF12	BPNSF16	BPNS[illegible]
**BPNSF6**	.000															
**BPNSF11**	.044	.000														
**BPNSF17**	.044	.048	.000													
**BPNSF23**	.043	.040	.047	.000												
**BPNSF3**	.052	.051	.049	.043	.000											
**BPNSF9**	.051	.045	.046	.047	.053	.000										
**BPNSF14**	.052	.049	.049	.048	.053	.046	.000									
**BPNSF21**	.043	.042	.053	.044	.052	.051	.051	.000								
**BPNSF2**	.051	.044	.048	.045	.045	.047	.048	.044	.000							
**BPNSF8**	.045	.042	.049	.051	.052	.048	.048	.049	.044	.000						
**BPNSF20**	.050	.047	.049	.043	.044	.045	.052	.051	.047	.048	.000					
**BPNSF22**	.044	.046	.048	.044	.045	.043	.051	.052	.040	.040	.044	.000				
**BPNSF4**	.049	.052	.049	.044	.056	.049	.035	.050	.053	.051	.044	.050	.000			
**BPNSF12**	.050	.049	.049	.043	.048	.050	.055	.051	.048	.053	.047	.047	.046	.000		
**BPNSF16**	.053	.047	.048	.048	.053	.049	.056	.049	.050	.050	.048	.045	.044	.043	.000	
**BPNSF24**	.048	.047	.051	.050	.052	.050	.056	.054	.047	.051	.049	.051	.048	.039	.052	[illegible]
**BPNSF5**	.044	.045	.053	.047	.045	.043	.048	.049	.042	.052	.049	.048	.048	.048	.047	[illegible]
**BPNSF10**	.050	.041	.052	.048	.056	.049	.054	.051	.047	.050	.051	.050	.058	.053	.052	[illegible]
**BPNSF15**	.047	.047	.051	.047	.057	.052	.050	.055	.052	.055	.046	.053	.052	.054	.055	[illegible]

	BPNSF6	BPNSF11	BPNSF17	BPNSF23	BPNSF3	BPNSF9	BPNSF14	BPNSF21	BPNSF2	BPNSF8	BPNSF20	BPNSF22	BPNSF4	BPNSF12	BPNSF16	BPNS[illegible]
**BPNSF18**	.051	.051	.053	.050	.052	.057	.057	.054	.048	.055	.047	.054	.050	.061	.059	.0[illegible]
**BPNSF1**	.051	.044	.047	.042	.045	.047	.050	.047	.045	.047	.044	.045	.045	.046	.045	.0[illegible]
**BPNSF7**	.050	.045	.047	.041	.053	.047	.052	.048	.045	.046	.043	.046	.050	.052	.051	.0[illegible]
**BPNSF13**	.050	.050	.049	.048	.048	.051	.037	.044	.048	.051	.047	.048	.046	.052	.050	.0[illegible]
**BPNSF19**	.045	.046	.049	.043	.053	.052	.053	.045	.048	.054	.050	.051	.054	.048	.054	.0[illegible]

Sample Means - Standard Errors (g1 - Structural covariances)

	BPNSF6	BPNSF11	BPNSF17	BPNSF23	BPNSF3	BPNSF9	BPNSF14	BPNSF21	BPNSF2	BPNSF8	BPNSF20	BPNSF22	BPNSF4	BPNSF12	BPNSF16	BPNSF[illegible]
**BPNSF6**	.082	.073	.079	.072	.066	.069	.069	.074	.076	.083	.076	.069	.059	.067	.063	.06[illegible]

Bootstrap Confidence (g1 - Structural covariances)

Percentile method (g1 - Structural covariances)

90% confidence intervals (percentile method)

Scalar Estimates (g1 - Structural covariances)

Regression Weights: (g1 - Structural covariances)

Parameter			Estimate	Lower	Upper	P
BPNSF19	<---	F1	1.000	1.000	1.000	...
BPNSF13	<---	F1	1.137	1.035	1.224	.010
BPNSF7	<---	F1	.855	.759	.953	.010
BPNSF1	<---	F1	.763	.647	.886	.010
BPNSF18	<---	F2	1.000	1.000	1.000	...
BPNSF15	<---	F2	2.472	2.005	3.283	.010
BPNSF10	<---	F2	3.106	2.481	4.237	.010
BPNSF5	<---	F2	2.568	2.058	3.451	.010
BPNSF24	<---	F3	1.000	1.000	1.000	...
BPNSF16	<---	F3	1.130	1.045	1.218	.010
BPNSF12	<---	F3	1.224	1.141	1.298	.010
BPNSF4	<---	F3	.850	.757	.938	.010
BPNSF22	<---	F4	1.000	1.000	1.000	...
BPNSF20	<---	F4	1.196	1.117	1.287	.010
BPNSF8	<---	F4	1.200	1.118	1.276	.010
BPNSF2	<---	F4	1.121	1.040	1.220	.010
BPNSF21	<---	F5	1.000	1.000	1.000	...
BPNSF14	<---	F5	1.094	1.025	1.171	.010
BPNSF9	<---	F5	1.039	.965	1.132	.010
BPNSF3	<---	F5	.881	.793	.984	.010
BPNSF23	<---	F6	1.000	1.000	1.000	...
BPNSF17	<---	F6	.976	.924	1.040	.010
BPNSF11	<---	F6	.959	.898	1.018	.010
BPNSF6	<---	F6	.863	.803	.929	.010

Standardized Regression Weights: (g1 - Structural covariances)

Parameter			Estimate	Lower	Upper	P
BPNSF19	<---	F1	.645	.595	.703	.010
BPNSF13	<---	F1	.752	.709	.791	.010
BPNSF7	<---	F1	.514	.462	.569	.010
BPNSF1	<---	F1	.462	.414	.511	.010
BPNSF18	<---	F2	.258	.193	.326	.010
BPNSF15	<---	F2	.601	.550	.657	.010
BPNSF10	<---	F2	.707	.663	.753	.010
BPNSF5	<---	F2	.629	.583	.681	.010
BPNSF24	<---	F3	.725	.685	.769	.010
BPNSF16	<---	F3	.777	.730	.823	.010
BPNSF12	<---	F3	.821	.785	.854	.010
BPNSF4	<---	F3	.623	.576	.674	.010
BPNSF22	<---	F4	.708	.674	.744	.010
BPNSF20	<---	F4	.784	.746	.822	.010
BPNSF8	<---	F4	.758	.706	.794	.010
BPNSF2	<---	F4	.725	.676	.767	.010
BPNSF21	<---	F5	.702	.662	.741	.010
BPNSF14	<---	F5	.778	.719	.839	.010
BPNSF9	<---	F5	.725	.675	.777	.010

Parameter			Estimate	Lower	Upper	P
BPNSF3	<---	F5	.643	.581	.706	.010
BPNSF23	<---	F6	.812	.771	.852	.010
BPNSF17	<---	F6	.744	.705	.794	.010
BPNSF11	<---	F6	.758	.721	.792	.010
BPNSF6	<---	F6	.699	.651	.743	.010

Intercepts: (g1 - Structural covariances)

Parameter	Estimate	Lower	Upper	P
**BPNSF19**	5.161	5.091	5.232	.010
**BPNSF13**	5.068	5.007	5.137	.010
**BPNSF7**	4.834	4.769	4.910	.010
**BPNSF1**	4.719	4.664	4.798	.010
**BPNSF18**	4.323	4.258	4.394	.010
**BPNSF15**	3.715	3.630	3.788	.010
**BPNSF10**	3.176	3.091	3.248	.010
**BPNSF5**	3.738	3.659	3.813	.010
**BPNSF24**	5.223	5.159	5.293	.010
**BPNSF16**	5.115	5.050	5.196	.010
**BPNSF12**	5.218	5.165	5.289	.010
**BPNSF4**	5.114	5.059	5.184	.010
**BPNSF22**	3.120	3.039	3.185	.010
**BPNSF20**	2.508	2.405	2.582	.010
**BPNSF8**	2.806	2.712	2.889	.010
**BPNSF2**	2.383	2.304	2.456	.010
**BPNSF21**	5.200	5.131	5.272	.010
**BPNSF14**	5.464	5.408	5.547	.010
**BPNSF9**	5.679	5.606	5.758	.010
**BPNSF3**	5.610	5.551	5.699	.010
**BPNSF23**	2.241	2.158	2.305	.010
**BPNSF17**	2.577	2.481	2.646	.010
**BPNSF11**	2.744	2.665	2.823	.010
**BPNSF6**	2.565	2.477	2.642	.010

Covariances: (g1 - Structural covariances)

Parameter			Estimate	Lower	Upper	P
F1	<-->	F2	-.156	-.195	-.115	.010
F2	<-->	F3	-.149	-.191	-.110	.010
F1	<-->	F3	.728	.655	.809	.010
F2	<-->	F4	.314	.228	.402	.010
F3	<-->	F4	-.596	-.672	-.526	.010
F1	<-->	F4	-.490	-.566	-.422	.010
F2	<-->	F5	-.143	-.184	-.104	.010
F4	<-->	F5	-.668	-.738	-.597	.010
F3	<-->	F5	.770	.687	.857	.010
F1	<-->	F5	.903	.796	1.035	.010
F6	<-->	F5	-.958	-1.064	-.863	.010
F6	<-->	F3	-.641	-.716	-.564	.010
F6	<-->	F4	1.266	1.153	1.379	.010
F6	<-->	F2	.372	.277	.465	.010
F6	<-->	F1	-.622	-.721	-.541	.010

Correlations: (g1 - Structural covariances)

Parameter			Estimate	Lower	Upper	P
F1	<-->	F2	-.433	-.494	-.364	.010
F2	<-->	F3	-.411	-.467	-.354	.010
F1	<-->	F3	.836	.790	.876	.010
F2	<-->	F4	.758	.719	.796	.010
F3	<-->	F4	-.595	-.639	-.553	.010
F1	<-->	F4	-.494	-.545	-.436	.010
F2	<-->	F5	-.363	-.415	-.287	.010
F4	<-->	F5	-.615	-.656	-.564	.010
F3	<-->	F5	.808	.764	.840	.010
F1	<-->	F5	.956	.915	.993	.010
F6	<-->	F5	-.723	-.764	-.679	.010
F6	<-->	F3	-.525	-.574	-.476	.010
F6	<-->	F4	.910	.877	.937	.010
F6	<-->	F2	.737	.684	.779	.010

Parameter	Estimate	Lower	Upper	P
F6 <--> F1	-.514	-.564	-.463	.010

Variances: (g1 - Structural covariances)

Parameter	Estimate	Lower	Upper	P
**F1**	.863	.734	.992	.010
**F2**	.150	.083	.234	.010
**F3**	.879	.772	.991	.010
**F4**	1.141	1.018	1.269	.010
**F5**	1.033	.920	1.173	.010
**F6**	1.696	1.532	1.865	.010
**e1**	1.211	.983	1.457	.010
**e2**	.855	.706	1.054	.010
**e3**	1.759	1.487	2.065	.010
**e4**	1.859	1.640	2.136	.010
**e5**	2.109	1.862	2.346	.010
**e6**	1.623	1.374	1.877	.010
**e7**	1.452	1.226	1.686	.010
**e8**	1.513	1.289	1.735	.010
**e9**	.791	.629	.931	.010
**e10**	.737	.574	.936	.010
**e11**	.635	.491	.775	.010
**e12**	.998	.858	1.154	.010
**e13**	1.137	.981	1.330	.010
**e14**	1.020	.798	1.234	.010
**e15**	1.214	1.018	1.451	.010
**e16**	1.295	1.051	1.572	.010
**e17**	1.065	.871	1.261	.010
**e18**	.806	.565	1.059	.010
**e19**	1.005	.745	1.251	.010
**e20**	1.138	.860	1.438	.010
**e21**	.873	.656	1.078	.010
**e22**	1.303	1.004	1.575	.010
**e23**	1.156	.981	1.359	.010
**e24**	1.322	1.095	1.602	.010

Matrices (g1 - Structural covariances)

Sample Covariances (g1 - Structural covariances)

Sample Covariances - Lower Bounds (PC) (g1 - Structural covariances)

	BPNSF6	BPNSF11	BPNSF17	BPNSF23	BPNSF3	BPNSF9	BPNSF14	BPNSF21	BPNSF2	BPNSF8	BPNSF20	BPNSF22	BPNSF4	BPNSF12	BPNSF16	BPNS[illegible]
**BPNSF6**	2.334															
**BPNSF11**	1.185	2.407														
**BPNSF17**	1.144	1.179	2.238													
**BPNSF23**	1.083	1.165	1.100	2.025												
**BPNSF3**	-1.068	-.940	-.930	-1.146	1.572											
**BPNSF9**	-1.074	-.991	-1.039	-1.162	.755	1.632										
**BPNSF14**	-1.016	-.936	-1.068	-1.118	.759	.897	1.730									
**BPNSF21**	-.845	-.876	-.773	-1.051	.557	.717	.863	1.770								
**BPNSF2**	1.006	1.137	.869	1.170	-1.116	-1.062	-1.113	-1.016	2.389							
**BPNSF8**	1.181	1.236	1.035	.912	-.772	-.997	-.808	-.575	1.133	2.418						
**BPNSF20**	.879	.939	1.022	1.193	-.958	-.986	-1.046	-.893	1.116	1.029	2.013					
**BPNSF22**	.684	.840	.617	.844	-.744	-.691	-.576	-.599	.940	1.077	.918	1.856				
**BPNSF4**	-.668	-.527	-.508	-.514	.481	.299	.568	.270	-.739	-.537	-.597	-.502	1.312			
**BPNSF12**	-.817	-.566	-.748	-.671	.431	.520	.695	.471	-.793	-.705	-.792	-.745	.523	1.456		
**BPNSF16**	-.868	-.726	-.816	-.842	.397	.553	.603	.520	-.786	-.767	-.795	-.640	.505	.831	1.413	
**BPNSF24**	-.650	-.607	-.738	-.718	.437	.548	.435	.368	-.882	-.701	-.760	-.751	.443	.761	.675	1.3[illegible]
**BPNSF5**	.572	.710	.437	.446	-.334	-.443	-.372	-.458	.608	.513	.413	.420	-.163	-.571	-.639	-.5[illegible]
**BPNSF10**	.638	1.131	.564	.547	-.402	-.544	-.398	-.481	.656	.811	.435	.337	-.434	-.647	-.557	-.4[illegible]
**BPNSF15**	.390	.553	.355	.168	-.417	-.334	-.014	-.402	.144	.421	.272	.249	-.286	-.537	-.294	-.3[illegible]
**BPNSF18**	-.343	-.115	-.066	-.227	.012	.086	-.142	.193	-.121	-.046	-.242	-.055	-.112	-.097	.019	-.1[illegible]
**BPNSF1**	-.478	-.428	-.264	-.406	.420	.224	.177	.394	-.321	-.300	-.361	-.312	.446	.373	.294	.3[illegible]
**BPNSF7**	-.444	-.592	-.288	-.615	.373	.612	.459	.689	-.734	-.254	-.416	-.343	.141	.184	.161	.2[illegible]
**BPNSF13**	-.833	-.762	-.771	-.855	.577	.664	1.070	.814	-.840	-.574	-.814	-.531	.424	.715	.567	.4[illegible]
**BPNSF19**	-.745	-.870	-.871	-1.037	.561	.558	.818	.996	-1.004	-.625	-.934	-.586	.285	.571	.562	.4[illegible]

Sample Covariances - Upper Bounds (PC) (g1 - Structural covariances)

	BPNSF6	BPNSF11	BPNSF17	BPNSF23	BPNSF3	BPNSF9	BPNSF14	BPNSF21	BPNSF2	BPNSF8	BPNSF20	BPNSF22	BPNSF4	BPNSF12	BPNSF16	BPNS[illegible]
**BPNSF6**	2.942															
**BPNSF11**	1.685	2.909														
**BPNSF17**	1.663	1.690	2.870													
**BPNSF23**	1.585	1.683	1.594	2.617												
**BPNSF3**	-.631	-.574	-.540	-.793	2.154											
**BPNSF9**	-.701	-.656	-.678	-.829	1.129	2.239										
**BPNSF14**	-.594	-.546	-.654	-.733	1.096	1.297	2.261									
**BPNSF21**	-.485	-.539	-.365	-.687	.913	1.099	1.332	2.292								
**BPNSF2**	1.496	1.646	1.325	1.664	-.753	-.687	-.719	-.634	2.911							
**BPNSF8**	1.697	1.728	1.570	1.428	-.382	-.587	-.450	-.213	1.636	2.942						
**BPNSF20**	1.427	1.436	1.562	1.702	-.617	-.607	-.622	-.514	1.533	1.533	2.583					
**BPNSF22**	1.101	1.260	1.016	1.266	-.428	-.373	-.234	-.223	1.323	1.503	1.293	2.260				
**BPNSF4**	-.321	-.195	-.202	-.216	.799	.599	.822	.583	-.367	-.185	-.317	-.213	1.639			
**BPNSF12**	-.398	-.209	-.397	-.373	.753	.892	1.128	.880	-.443	-.344	-.450	-.433	.802	1.923		
**BPNSF16**	-.449	-.379	-.447	-.485	.718	.905	1.006	.876	-.422	-.394	-.453	-.355	.775	1.160	1.817	
**BPNSF24**	-.308	-.282	-.365	-.389	.794	.926	.790	.716	-.555	-.362	-.465	-.435	.710	1.074	.985	[illegible]
**BPNSF5**	1.003	1.133	.875	.841	-.011	-.135	-.062	-.110	.969	.981	.809	.770	.114	-.263	-.290	[illegible]
**BPNSF10**	1.131	1.597	1.074	.988	-.016	-.161	.034	-.074	1.145	1.312	.869	.767	-.048	-.255	-.188	[illegible]
**BPNSF15**	.796	.993	.777	.541	-.012	.036	.353	.014	.593	.912	.627	.638	.038	-.172	.100	[illegible]
**BPNSF18**	.042	.275	.324	.109	.363	.426	.255	.578	.231	.386	.106	.320	.184	.297	.366	[illegible]
**BPNSF1**	-.083	-.101	.106	-.076	.792	.538	.544	.771	.025	.089	-.056	-.003	.746	.672	.574	[illegible]
**BPNSF7**	-.055	-.230	.084	-.312	.750	.989	.834	1.079	-.392	.117	-.060	-.006	.438	.562	.493	[illegible]
**BPNSF13**	-.433	-.360	-.410	-.488	.907	1.033	1.491	1.272	-.449	-.170	-.437	-.201	.727	1.144	.944	[illegible]
**BPNSF19**	-.375	-.486	-.491	-.654	.934	.941	1.239	1.468	-.604	-.185	-.519	-.247	.613	.916	.943	[illegible]

Sample Covariances - Two Tailed Significance (PC) (g1 - Structural covariances)

	BPNSF6	BPNSF11	BPNSF17	BPNSF23	BPNSF3	BPNSF9	BPNSF14	BPNSF21	BPNSF2	BPNSF8	BPNSF20	BPNSF22	BPNSF4	BPNSF12	BPNSF16	BPNS[illegible]
**BPNSF6**	.010															
**BPNSF11**	.010	.010														
**BPNSF17**	.010	.010	.010													
**BPNSF23**	.010	.010	.010	.010												
**BPNSF3**	.010	.010	.010	.010	.010											
**BPNSF9**	.010	.010	.010	.010	.010	.010										
**BPNSF14**	.010	.010	.010	.010	.010	.010	.010									
**BPNSF21**	.010	.010	.010	.010	.010	.010	.010	.010								
**BPNSF2**	.010	.010	.010	.010	.010	.010	.010	.010	.010							
**BPNSF8**	.010	.010	.010	.010	.010	.010	.010	.010	.010	.010						
**BPNSF20**	.010	.010	.010	.010	.010	.010	.010	.010	.010	.010	.010					
**BPNSF22**	.010	.010	.010	.010	.010	.010	.010	.010	.010	.010	.010	.010				
**BPNSF4**	.010	.010	.010	.010	.010	.010	.010	.010	.010	.010	.010	.010	.010			
**BPNSF12**	.010	.010	.010	.010	.010	.010	.010	.010	.010	.010	.010	.010	.010	.010		
**BPNSF16**	.010	.010	.010	.010	.010	.010	.010	.010	.010	.010	.010	.010	.010	.010	.010	
**BPNSF24**	.010	.010	.010	.010	.010	.010	.010	.010	.010	.010	.010	.010	.010	.010	.010	[illegible]
**BPNSF5**	.010	.010	.010	.010	.077	.012	.044	.016	.010	.010	.010	.010	.721	.010	.010	[illegible]
**BPNSF10**	.010	.010	.010	.010	.077	.010	.193	.010	.010	.010	.010	.010	.039	.010	.010	[illegible]
**BPNSF15**	.010	.010	.010	.010	.094	.201	.157	.142	.010	.010	.010	.010	.196	.010	.371	[illegible]
**BPNSF18**	.207	.487	.403	.569	.078	.041	.589	.010	.702	.197	.400	.271	.783	.336	.078	[illegible]
**BPNSF1**	.027	.018	.456	.016	.010	.010	.010	.010	.146	.339	.033	.095	.010	.010	.010	[illegible]
**BPNSF7**	.025	.010	.268	.010	.010	.010	.010	.010	.010	.519	.010	.073	.010	.010	.016	[illegible]
**BPNSF13**	.010	.010	.010	.010	.010	.010	.010	.010	.010	.010	.010	.010	.010	.010	.010	[illegible]
**BPNSF19**	.010	.010	.010	.010	.010	.010	.010	.010	.010	.010	.010	.010	.010	.010	.010	[illegible]

Sample Correlations (g1 - Structural covariances)

Sample Correlations - Lower Bounds (PC) (g1 - Structural covariances)

	BPNSF6	BPNSF11	BPNSF17	BPNSF23	BPNSF3	BPNSF9	BPNSF14	BPNSF21	BPNSF2	BPNSF8	BPNSF20	BPNSF22	BPNSF4	BPNSF12	BPNSF16	BPNS[illegible]
**BPNSF6**	1.000															
**BPNSF11**	.469	1.000														
**BPNSF17**	.460	.467	1.000													
**BPNSF23**	.472	.506	.472	1.000												
**BPNSF3**	-.471	-.424	-.417	-.541	1.000											
**BPNSF9**	-.477	-.436	-.460	-.550	.403	1.000										
**BPNSF14**	-.444	-.394	-.456	-.501	.387	.474	1.000									
**BPNSF21**	-.360	-.375	-.341	-.465	.297	.376	.447	1.000								
**BPNSF2**	.395	.437	.335	.502	-.500	-.465	-.470	-.431	1.000							
**BPNSF8**	.458	.476	.412	.376	-.339	-.424	-.347	-.253	.446	1.000						
**BPNSF20**	.389	.398	.448	.542	-.452	-.456	-.475	-.418	.455	.430	1.000					
**BPNSF22**	.309	.363	.273	.409	-.378	-.340	-.287	-.291	.408	.474	.436	1.000				
**BPNSF4**	-.331	-.268	-.265	-.276	.290	.181	.347	.164	-.368	-.265	-.316	-.289	1.000			

	BPNSF6	BPNSF11	BPNSF17	BPNSF23	BPNSF3	BPNSF9	BPNSF14	BPNSF21	BPNSF2	BPNSF8	BPNSF20	BPNSF22	BPNSF4	BPNSF12	BPNSF16	BPNS[illegible]
**BPNSF12**	-.370	-.261	-.353	-.325	.247	.312	.391	.264	-.371	-.334	-.389	-.387	.343	1.000		
**BPNSF16**	-.407	-.342	-.383	-.426	.232	.326	.349	.306	-.371	-.363	-.411	-.343	.345	.526	1.000	
**BPNSF24**	-.323	-.303	-.351	-.377	.270	.348	.248	.217	-.431	-.349	-.419	-.417	.296	.493	.446	[illegible]
**BPNSF5**	.237	.296	.177	.195	-.158	-.208	-.174	-.213	.246	.210	.178	.194	-.088	-.296	-.310	[illegible]
**BPNSF10**	.250	.440	.218	.220	-.179	-.245	-.174	-.205	.256	.318	.179	.152	-.226	-.302	-.267	[illegible]
**BPNSF15**	.156	.218	.142	.072	-.193	-.160	-.006	-.175	.059	.167	.120	.115	-.153	-.263	-.144	[illegible]
**BPNSF18**	-.143	-.048	-.029	-.106	.007	.042	-.067	.095	-.052	-.021	-.109	-.028	-.065	-.051	.010	[illegible]
**BPNSF1**	-.193	-.184	-.116	-.177	.218	.111	.085	.195	-.132	-.122	-.162	-.144	.256	.190	.155	[illegible]
**BPNSF7**	-.180	-.240	-.121	-.269	.176	.297	.211	.319	-.306	-.104	-.180	-.154	.079	.099	.084	[illegible]
**BPNSF13**	-.373	-.333	-.344	-.390	.311	.356	.589	.438	-.366	-.252	-.384	-.271	.266	.432	.336	[illegible]
**BPNSF19**	-.309	-.374	-.379	-.450	.288	.302	.415	.536	-.426	-.269	-.416	-.287	.162	.317	.320	[illegible]

Sample Correlations - Upper Bounds (PC) (g1 - Structural covariances)

	BPNSF6	BPNSF11	BPNSF17	BPNSF23	BPNSF3	BPNSF9	BPNSF14	BPNSF21	BPNSF2	BPNSF8	BPNSF20	BPNSF22	BPNSF4	BPNSF12	BPNSF16	BPNS[illegible]
**BPNSF6**	1.000															
**BPNSF11**	.617	1.000														
**BPNSF17**	.608	.617	1.000													
**BPNSF23**	.615	.635	.626	1.000												
**BPNSF3**	-.295	-.259	-.254	-.386	1.000											
**BPNSF9**	-.308	-.297	-.309	-.394	.578	1.000										
**BPNSF14**	-.264	-.233	-.294	-.346	.573	.635	1.000									
**BPNSF21**	-.213	-.227	-.166	-.334	.470	.536	.624	1.000								
**BPNSF2**	.557	.589	.496	.651	-.347	-.304	-.318	-.283	1.000							
**BPNSF8**	.607	.618	.579	.550	-.172	-.264	-.196	-.088	.596	1.000						
**BPNSF20**	.559	.555	.612	.691	-.297	-.305	-.294	-.245	.614	.597	1.000					
**BPNSF22**	.453	.516	.434	.554	-.217	-.192	-.110	-.111	.548	.619	.575	1.000				
**BPNSF4**	-.167	-.100	-.104	-.118	.471	.352	.451	.327	-.186	-.097	-.177	-.124	1.000			
**BPNSF12**	-.198	-.102	-.188	-.189	.418	.477	.571	.434	-.211	-.159	-.228	-.231	.508	1.000		
**BPNSF16**	-.224	-.188	-.223	-.259	.409	.499	.536	.466	-.201	-.192	-.236	-.193	.483	.668	1.000	
**BPNSF24**	-.155	-.141	-.189	-.210	.441	.518	.437	.399	-.272	-.178	-.250	-.247	.463	.629	.607	[illegible]
**BPNSF5**	.395	.442	.364	.354	-.005	-.057	-.029	-.051	.393	.382	.344	.359	.062	-.133	-.149	[illegible]
**BPNSF10**	.413	.582	.403	.384	-.007	-.071	.016	-.032	.413	.480	.339	.314	-.024	-.117	-.089	[illegible]
**BPNSF15**	.305	.381	.310	.223	-.006	.018	.158	.007	.230	.354	.264	.285	.020	-.088	.048	[illegible]
**BPNSF18**	.017	.119	.135	.049	.178	.218	.128	.279	.097	.159	.048	.144	.102	.152	.201	[illegible]
**BPNSF1**	-.033	-.041	.042	-.034	.377	.272	.254	.359	.010	.038	-.026	-.001	.413	.354	.303	[illegible]
**BPNSF7**	-.022	-.093	.033	-.132	.360	.454	.386	.478	-.153	.048	-.026	-.003	.243	.274	.245	[illegible]
**BPNSF13**	-.195	-.156	-.180	-.234	.469	.528	.712	.592	-.202	-.076	-.217	-.109	.416	.600	.511	[illegible]
**BPNSF19**	-.162	-.213	-.212	-.319	.466	.472	.589	.679	-.259	-.075	-.239	-.119	.346	.474	.496	[illegible]

Sample Correlations - Two Tailed Significance (PC) (g1 - Structural covariances)

	BPNSF6	BPNSF11	BPNSF17	BPNSF23	BPNSF3	BPNSF9	BPNSF14	BPNSF21	BPNSF2	BPNSF8	BPNSF20	BPNSF22	BPNSF4	BPNSF12	BPNSF16	BPNS[illegible]
**BPNSF6**	...															
**BPNSF11**	.010	...														
**BPNSF17**	.010	.010	...													
**BPNSF23**	.010	.010	.010	...												
**BPNSF3**	.010	.010	.010	.010	...											
**BPNSF9**	.010	.010	.010	.010	.010	...										
**BPNSF14**	.010	.010	.010	.010	.010	.010	...									
**BPNSF21**	.010	.010	.010	.010	.010	.010	.010	...								
**BPNSF2**	.010	.010	.010	.010	.010	.010	.010	.010	...							
**BPNSF8**	.010	.010	.010	.010	.010	.010	.010	.010	.010	...						
**BPNSF20**	.010	.010	.010	.010	.010	.010	.010	.010	.010	.010	...					
**BPNSF22**	.010	.010	.010	.010	.010	.010	.010	.010	.010	.010	.010	...				
**BPNSF4**	.010	.010	.010	.010	.010	.010	.010	.010	.010	.010	.010	.010	...			
**BPNSF12**	.010	.010	.010	.010	.010	.010	.010	.010	.010	.010	.010	.010	.010	...		
**BPNSF16**	.010	.010	.010	.010	.010	.010	.010	.010	.010	.010	.010	.010	.010	.010	...	
**BPNSF24**	.010	.010	.010	.010	.010	.010	.010	.010	.010	.010	.010	.010	.010	.010	.010	
**BPNSF5**	.010	.010	.010	.010	.077	.012	.044	.016	.010	.010	.010	.010	.721	.010	.010	[illegible]
**BPNSF10**	.010	.010	.010	.010	.077	.010	.193	.010	.010	.010	.010	.010	.039	.010	.010	[illegible]
**BPNSF15**	.010	.010	.010	.010	.094	.201	.157	.142	.010	.010	.010	.010	.196	.010	.371	[illegible]
**BPNSF18**	.207	.487	.403	.569	.078	.041	.589	.010	.702	.197	.400	.271	.783	.336	.078	[illegible]
**BPNSF1**	.027	.017	.456	.016	.010	.010	.010	.010	.145	.339	.033	.095	.010	.010	.010	[illegible]
**BPNSF7**	.025	.010	.268	.010	.010	.010	.010	.010	.010	.519	.010	.073	.010	.010	.015	[illegible]
**BPNSF13**	.010	.010	.010	.010	.010	.010	.010	.010	.010	.010	.010	.010	.010	.010	.010	[illegible]
**BPNSF19**	.010	.010	.010	.010	.010	.010	.010	.010	.010	.010	.010	.010	.010	.010	.010	[illegible]

Sample Means (g1 - Structural covariances)

Sample Means - Lower Bounds (PC) (g1 - Structural covariances)

	BPNSF6	BPNSF11	BPNSF17	BPNSF23	BPNSF3	BPNSF9	BPNSF14	BPNSF21	BPNSF2	BPNSF8	BPNSF20	BPNSF22	BPNSF4	BPNSF12	BPNSF16	BPNSF[illegible]
**BPNSF6**	2.319	2.572	2.304	1.943	5.618	5.729	5.485	5.242	2.103	2.626	2.215	2.901	4.970	5.123	5.014	5.13[illegible]

Sample Means - Upper Bounds (PC) (g1 - Structural covariances)

	BPNSF6	BPNSF11	BPNSF17	BPNSF23	BPNSF3	BPNSF9	BPNSF14	BPNSF21	BPNSF2	BPNSF8	BPNSF20	BPNSF22	BPNSF4	BPNSF12	BPNSF16	BPNSF[illegible]
**BPNSF6**	2.585	2.818	2.585	2.178	5.853	5.952	5.713	5.486	2.367	2.909	2.470	3.142	5.166	5.354	5.226	5.36[illegible]

Sample Means - Two Tailed Significance (PC) (g1 - Structural covariances)

	BPNSF6	BPNSF11	BPNSF17	BPNSF23	BPNSF3	BPNSF9	BPNSF14	BPNSF21	BPNSF2	BPNSF8	BPNSF20	BPNSF22	BPNSF4	BPNSF12	BPNSF16	BPNSF[illegible]
**BPNSF6**	.010	.010	.010	.010	.010	.010	.010	.010	.010	.010	.010	.010	.010	.010	.010	.01[illegible]

Bias-corrected percentile method (g1 - Structural covariances)

90% confidence intervals (bias-corrected percentile method)

Scalar Estimates (g1 - Structural covariances)

Regression Weights: (g1 - Structural covariances)

Parameter			Estimate	Lower	Upper	P
BPNSF19	<---	F1	1.000	1.000	1.000	...
BPNSF13	<---	F1	1.137	1.046	1.235	.005
BPNSF7	<---	F1	.855	.759	.953	.010
BPNSF1	<---	F1	.763	.630	.865	.018
BPNSF18	<---	F2	1.000	1.000	1.000	...
BPNSF15	<---	F2	2.472	2.005	3.283	.010
BPNSF10	<---	F2	3.106	2.501	4.343	.007
BPNSF5	<---	F2	2.568	2.064	3.455	.009
BPNSF24	<---	F3	1.000	1.000	1.000	...
BPNSF16	<---	F3	1.130	1.046	1.219	.009
BPNSF12	<---	F3	1.224	1.152	1.307	.004
BPNSF4	<---	F3	.850	.755	.938	.012
BPNSF22	<---	F4	1.000	1.000	1.000	...
BPNSF20	<---	F4	1.196	1.124	1.298	.005
BPNSF8	<---	F4	1.200	1.128	1.306	.004
BPNSF2	<---	F4	1.121	1.043	1.221	.006
BPNSF21	<---	F5	1.000	1.000	1.000	...
BPNSF14	<---	F5	1.094	1.026	1.178	.009
BPNSF9	<---	F5	1.039	.970	1.136	.007
BPNSF3	<---	F5	.881	.791	.984	.012
BPNSF23	<---	F6	1.000	1.000	1.000	...
BPNSF17	<---	F6	.976	.914	1.026	.019
BPNSF11	<---	F6	.959	.904	1.023	.007
BPNSF6	<---	F6	.863	.805	.932	.008

Standardized Regression Weights: (g1 - Structural covariances)

Parameter			Estimate	Lower	Upper	P
BPNSF19	<---	F1	.645	.579	.685	.044
BPNSF13	<---	F1	.752	.707	.790	.012
BPNSF7	<---	F1	.514	.463	.570	.009
BPNSF1	<---	F1	.462	.409	.507	.016
BPNSF18	<---	F2	.258	.190	.324	.012
BPNSF15	<---	F2	.601	.550	.657	.009
BPNSF10	<---	F2	.707	.663	.752	.011
BPNSF5	<---	F2	.629	.575	.669	.026
BPNSF24	<---	F3	.725	.685	.767	.013
BPNSF16	<---	F3	.777	.706	.810	.034
BPNSF12	<---	F3	.821	.784	.853	.011
BPNSF4	<---	F3	.623	.578	.675	.007
BPNSF22	<---	F4	.708	.673	.741	.012
BPNSF20	<---	F4	.784	.746	.822	.010
BPNSF8	<---	F4	.758	.700	.792	.013
BPNSF2	<---	F4	.725	.682	.770	.005
BPNSF21	<---	F5	.702	.660	.738	.019
BPNSF14	<---	F5	.778	.697	.829	.026

Parameter			Estimate	Lower	Upper	P
BPNSF9	<---	F5	.725	.673	.776	.015
BPNSF3	<---	F5	.643	.561	.698	.020
BPNSF23	<---	F6	.812	.768	.849	.014
BPNSF17	<---	F6	.744	.703	.785	.020
BPNSF11	<---	F6	.758	.728	.802	.004
BPNSF6	<---	F6	.699	.655	.745	.007

Intercepts: (g1 - Structural covariances)

Parameter	Estimate	Lower	Upper	P
**BPNSF19**	5.161	5.069	5.220	.030
**BPNSF13**	5.068	5.002	5.129	.020
**BPNSF7**	4.834	4.747	4.893	.032
**BPNSF1**	4.719	4.652	4.783	.019
**BPNSF18**	4.323	4.257	4.393	.012
**BPNSF15**	3.715	3.618	3.779	.015
**BPNSF10**	3.176	3.096	3.253	.006
**BPNSF5**	3.738	3.667	3.815	.007
**BPNSF24**	5.223	5.143	5.277	.032
**BPNSF16**	5.115	5.046	5.189	.016
**BPNSF12**	5.218	5.160	5.277	.018
**BPNSF4**	5.114	5.037	5.171	.023
**BPNSF22**	3.120	3.047	3.194	.006
**BPNSF20**	2.508	2.432	2.602	.003
**BPNSF8**	2.806	2.721	2.891	.007
**BPNSF2**	2.383	2.314	2.463	.005
**BPNSF21**	5.200	5.118	5.247	.044
**BPNSF14**	5.464	5.388	5.523	.026
**BPNSF9**	5.679	5.589	5.744	.026
**BPNSF3**	5.610	5.553	5.700	.009
**BPNSF23**	2.241	2.176	2.321	.003
**BPNSF17**	2.577	2.497	2.668	.004
**BPNSF11**	2.744	2.670	2.828	.005
**BPNSF6**	2.565	2.479	2.648	.009

Covariances: (g1 - Structural covariances)

Parameter			Estimate	Lower	Upper	P
F1	<-->	F2	-.156	-.195	-.114	.011
F2	<-->	F3	-.149	-.191	-.110	.009
F1	<-->	F3	.728	.639	.801	.016
F2	<-->	F4	.314	.229	.406	.009
F3	<-->	F4	-.596	-.663	-.518	.019
F1	<-->	F4	-.490	-.566	-.422	.012
F2	<-->	F5	-.143	-.187	-.108	.006
F4	<-->	F5	-.668	-.742	-.608	.006
F3	<-->	F5	.770	.686	.857	.012
F1	<-->	F5	.903	.798	1.036	.009
F6	<-->	F5	-.958	-1.081	-.880	.004
F6	<-->	F3	-.641	-.711	-.536	.018
F6	<-->	F4	1.266	1.155	1.381	.008
F6	<-->	F2	.372	.280	.474	.009
F6	<-->	F1	-.622	-.718	-.538	.014

Correlations: (g1 - Structural covariances)

Parameter			Estimate	Lower	Upper	P
F1	<-->	F2	-.433	-.491	-.355	.014
F2	<-->	F3	-.411	-.475	-.357	.006
F1	<-->	F3	.836	.799	.878	.004
F2	<-->	F4	.758	.721	.800	.007
F3	<-->	F4	-.595	-.637	-.544	.015
F1	<-->	F4	-.494	-.540	-.433	.015
F2	<-->	F5	-.363	-.411	-.282	.019
F4	<-->	F5	-.615	-.659	-.566	.006
F3	<-->	F5	.808	.766	.840	.008
F1	<-->	F5	.956	.916	.994	.007
F6	<-->	F5	-.723	-.765	-.679	.009
F6	<-->	F3	-.525	-.565	-.460	.025
F6	<-->	F4	.910	.880	.943	.007

Parameter			Estimate	Lower	Upper	P
F6	<-->	F2	.737	.684	.780	.009
F6	<-->	F1	-.514	-.559	-.456	.023

Variances: (g1 - Structural covariances)

Parameter	Estimate	Lower	Upper	P
**F1**	.863	.726	.981	.016
**F2**	.150	.083	.232	.012
**F3**	.879	.757	.987	.016
**F4**	1.141	1.010	1.268	.012
**F5**	1.033	.918	1.171	.011
**F6**	1.696	1.534	1.876	.008
**e1**	1.211	1.017	1.526	.002
**e2**	.855	.716	1.086	.006
**e3**	1.759	1.461	2.028	.014
**e4**	1.859	1.672	2.158	.004
**e5**	2.109	1.889	2.393	.005
**e6**	1.623	1.395	1.881	.007
**e7**	1.452	1.205	1.654	.018
**e8**	1.513	1.293	1.761	.007
**e9**	.791	.648	.947	.007
**e10**	.737	.618	1.022	.002
**e11**	.635	.520	.786	.005
**e12**	.998	.852	1.140	.013
**e13**	1.137	1.000	1.339	.005
**e14**	1.020	.793	1.232	.012
**e15**	1.214	1.022	1.465	.009
**e16**	1.295	1.035	1.511	.020
**e17**	1.065	.880	1.263	.009
**e18**	.806	.613	1.192	.003
**e19**	1.005	.787	1.357	.004
**e20**	1.138	.861	1.439	.009
**e21**	.873	.692	1.123	.005
**e22**	1.303	1.036	1.636	.005
**e23**	1.156	.969	1.351	.012
**e24**	1.322	1.078	1.561	.015

Matrices (g1 - Structural covariances)

Sample Covariances (g1 - Structural covariances)

Sample Covariances - Lower Bounds (BC) (g1 - Structural covariances)

	BPNSF6	BPNSF11	BPNSF17	BPNSF23	BPNSF3	BPNSF9	BPNSF14	BPNSF21	BPNSF2	BPNSF8	BPNSF20	BPNSF22	BPNSF4	BPNSF12	BPNSF16	BPNS[illegible]
**BPNSF6**	2.397															
**BPNSF11**	1.225	2.460														
**BPNSF17**	1.121	1.186	2.247													
**BPNSF23**	1.087	1.221	1.115	2.069												
**BPNSF3**	-1.179	-.979	-.974	-1.230	1.656											
**BPNSF9**	-1.161	-1.197	-1.086	-1.237	.792	1.689										
**BPNSF14**	-1.043	-1.010	-1.078	-1.166	.770	.902	1.746									
**BPNSF21**	-.887	-.893	-.796	-1.079	.567	.734	.911	1.776								
**BPNSF2**	1.009	1.161	.906	1.156	-1.117	-1.091	-1.128	-.990	2.387							
**BPNSF8**	1.192	1.251	1.071	.921	-.874	-1.035	-.844	-.617	1.160	2.418						
**BPNSF20**	.974	.969	1.053	1.247	-1.006	-1.003	-1.093	-.914	1.145	1.065	2.026					
**BPNSF22**	.685	.863	.604	.848	-.821	-.709	-.582	-.620	.966	1.104	.936	1.861				
**BPNSF4**	-.702	-.549	-.552	-.519	.488	.321	.579	.282	-.757	-.633	-.628	-.512	1.318			
**BPNSF12**	-.823	-.569	-.747	-.637	.410	.515	.708	.525	-.776	-.753	-.766	-.746	.541	1.467		
**BPNSF16**	-.874	-.753	-.836	-.847	.398	.579	.634	.530	-.809	-.784	-.823	-.646	.506	.842	1.424	
**BPNSF24**	-.650	-.611	-.698	-.713	.459	.587	.435	.392	-.925	-.726	-.760	-.752	.448	.780	.675	1.3[illegible]
**BPNSF5**	.566	.756	.436	.458	-.324	-.451	-.370	-.458	.622	.537	.396	.454	-.195	-.571	-.667	-.5[illegible]
**BPNSF10**	.628	1.119	.577	.570	-.385	-.549	-.408	-.491	.701	.811	.438	.351	-.454	-.664	-.603	-.4[illegible]
**BPNSF15**	.369	.537	.355	.168	-.422	-.334	.012	-.402	.132	.421	.248	.218	-.313	-.587	-.308	-.3[illegible]
**BPNSF18**	-.384	-.161	-.103	-.260	-.001	.090	-.144	.179	-.113	-.134	-.243	-.074	-.098	-.091	.023	-.1[illegible]
**BPNSF1**	-.479	-.430	-.262	-.424	.460	.256	.202	.420	-.373	-.323	-.416	-.324	.472	.393	.320	.3[illegible]
**BPNSF7**	-.479	-.627	-.316	-.658	.388	.613	.479	.702	-.726	-.301	-.445	-.400	.150	.243	.203	.2[illegible]
**BPNSF13**	-.886	-.800	-.778	-.896	.580	.654	1.115	.845	-.832	-.612	-.832	-.537	.434	.715	.604	.4[illegible]
**BPNSF19**	-.754	-.870	-.939	-1.063	.615	.606	.849	1.014	-.997	-.674	-1.004	-.631	.307	.593	.613	.4[illegible]

Sample Covariances - Upper Bounds (BC) (g1 - Structural covariances)

	BPNSF6	BPNSF11	BPNSF17	BPNSF23	BPNSF3	BPNSF9	BPNSF14	BPNSF21	BPNSF2	BPNSF8	BPNSF20	BPNSF22	BPNSF4	BPNSF12	BPNSF16	BPNS
**BPNSF6**	2.992															
**BPNSF11**	1.781	3.010														
**BPNSF17**	1.621	1.707	2.876													
**BPNSF23**	1.602	1.695	1.599	2.658												
**BPNSF3**	-.710	-.598	-.603	-.819	2.220											
**BPNSF9**	-.756	-.736	-.704	-.848	1.174	2.344										
**BPNSF14**	-.635	-.578	-.659	-.773	1.108	1.312	2.339									
**BPNSF21**	-.549	-.555	-.404	-.713	.936	1.125	1.382	2.304								
**BPNSF2**	1.506	1.702	1.414	1.658	-.761	-.732	-.731	-.612	2.909							
**BPNSF8**	1.711	1.764	1.593	1.437	-.438	-.661	-.478	-.251	1.664	2.943						
**BPNSF20**	1.507	1.467	1.568	1.752	-.669	-.656	-.643	-.542	1.607	1.572	2.595					
**BPNSF22**	1.101	1.292	.994	1.272	-.467	-.411	-.254	-.277	1.359	1.563	1.330	2.270				
**BPNSF4**	-.343	-.224	-.225	-.216	.812	.611	.830	.599	-.378	-.254	-.329	-.227	1.656			
**BPNSF12**	-.409	-.213	-.397	-.316	.734	.882	1.147	.914	-.436	-.360	-.444	-.437	.831	1.928		
**BPNSF16**	-.471	-.399	-.468	-.487	.720	.974	1.037	.882	-.437	-.415	-.463	-.359	.794	1.178	1.827	
**BPNSF24**	-.308	-.294	-.355	-.387	.811	.972	.790	.723	-.575	-.391	-.465	-.440	.712	1.087	.985	1.7
**BPNSF5**	.987	1.163	.866	.845	.000	-.136	-.061	-.108	.997	.988	.792	.824	.104	-.263	-.315	-.2
**BPNSF10**	1.103	1.591	1.079	1.008	.000	-.167	.029	-.077	1.180	1.312	.883	.773	-.060	-.265	-.217	-.0
**BPNSF15**	.765	.992	.777	.541	-.016	.038	.419	.013	.581	.912	.613	.614	.023	-.200	.067	-.0
**BPNSF18**	.011	.242	.313	.106	.336	.448	.246	.555	.244	.342	.099	.300	.185	.310	.367	.2
**BPNSF1**	-.094	-.111	.106	-.115	.865	.573	.568	.824	-.004	.026	-.098	-.035	.753	.710	.595	.6
**BPNSF7**	-.091	-.263	.034	-.341	.771	.990	.853	1.082	-.358	.073	-.106	-.032	.453	.610	.518	.5
**BPNSF13**	-.477	-.427	-.410	-.512	.928	1.016	1.585	1.295	-.447	-.233	-.492	-.229	.740	1.144	.980	.8
**BPNSF19**	-.386	-.486	-.521	-.680	1.009	.975	1.306	1.489	-.588	-.240	-.584	-.255	.625	.952	1.036	.7

Sample Covariances - Two Tailed Significance (BC) (g1 - Structural covariances)

	BPNSF6	BPNSF11	BPNSF17	BPNSF23	BPNSF3	BPNSF9	BPNSF14	BPNSF21	BPNSF2	BPNSF8	BPNSF20	BPNSF22	BPNSF4	BPNSF12	BPNSF16	BPNS
**BPNSF6**	.006															
**BPNSF11**	.003	.003														
**BPNSF17**	.014	.007	.008													
**BPNSF23**	.008	.005	.007	.005												
**BPNSF3**	.002	.005	.003	.004	.003											
**BPNSF9**	.002	.001	.005	.003	.003	.003										
**BPNSF14**	.004	.002	.008	.004	.007	.007	.005									
**BPNSF21**	.002	.006	.004	.005	.006	.005	.002	.006								
**BPNSF2**	.007	.004	.004	.012	.009	.005	.006	.018	.012							
**BPNSF8**	.007	.006	.005	.008	.002	.002	.004	.005	.006	.009						
**BPNSF20**	.003	.005	.006	.002	.003	.004	.005	.004	.003	.006	.006					
**BPNSF22**	.009	.006	.014	.009	.002	.003	.006	.003	.004	.005	.005	.009				
**BPNSF4**	.005	.005	.004	.009	.005	.006	.006	.007	.006	.002	.004	.005	.006			
**BPNSF12**	.007	.009	.011	.019	.016	.013	.006	.003	.013	.004	.012	.008	.005	.009		
**BPNSF16**	.007	.005	.004	.009	.009	.004	.004	.007	.006	.005	.006	.007	.007	.006	.007	
**BPNSF24**	.010	.007	.016	.012	.005	.005	.010	.006	.004	.004	.010	.008	.008	.007	.010	.0
**BPNSF5**	.012	.004	.012	.008	.100	.010	.047	.019	.006	.007	.012	.004	.577	.010	.005	.0
**BPNSF10**	.013	.012	.008	.007	.101	.008	.169	.007	.005	.010	.006	.007	.031	.009	.004	.0
**BPNSF15**	.021	.014	.010	.011	.089	.210	.086	.136	.012	.010	.019	.025	.142	.005	.284	.0
**BPNSF18**	.137	.732	.493	.535	.102	.036	.624	.019	.611	.362	.372	.330	.671	.299	.074	.5
**BPNSF1**	.018	.013	.471	.008	.003	.004	.006	.005	.093	.174	.010	.046	.006	.004	.004	.0
**BPNSF7**	.007	.004	.174	.002	.005	.008	.005	.008	.018	.250	.004	.021	.008	.003	.004	.0
**BPNSF13**	.003	.003	.009	.005	.007	.014	.004	.005	.012	.002	.004	.005	.005	.010	.004	.0
**BPNSF19**	.007	.010	.003	.006	.002	.004	.004	.006	.012	.004	.003	.006	.004	.006	.002	.0

Sample Correlations (g1 - Structural covariances)

Sample Correlations - Lower Bounds (BC) (g1 - Structural covariances)

	BPNSF6	BPNSF11	BPNSF17	BPNSF23	BPNSF3	BPNSF9	BPNSF14	BPNSF21	BPNSF2	BPNSF8	BPNSF20	BPNSF22	BPNSF4	BPNSF12	BPNSF16	BPNS
**BPNSF6**	1.000															
**BPNSF11**	.487	1.000														
**BPNSF17**	.444	.462	1.000													
**BPNSF23**	.479	.510	.472	1.000												
**BPNSF3**	-.498	-.425	-.430	-.546	1.000											
**BPNSF9**	-.496	-.455	-.465	-.550	.415	1.000										
**BPNSF14**	-.447	-.410	-.458	-.507	.379	.462	1.000									
**BPNSF21**	-.366	-.373	-.347	-.464	.311	.379	.447	1.000								
**BPNSF2**	.395	.448	.337	.488	-.500	-.465	-.465	-.425	1.000							
**BPNSF8**	.458	.468	.419	.375	-.348	-.434	-.361	-.261	.464	1.000						
**BPNSF20**	.412	.406	.458	.545	-.462	-.458	-.478	-.418	.458	.449	1.000					
**BPNSF22**	.309	.364	.257	.403	-.402	-.349	-.288	-.293	.430	.491	.441	1.000				
**BPNSF4**	-.334	-.273	-.279	-.264	.296	.183	.342	.168	-.370	-.283	-.331	-.291	1.000			

	BPNSF6	BPNSF11	BPNSF17	BPNSF23	BPNSF3	BPNSF9	BPNSF14	BPNSF21	BPNSF2	BPNSF8	BPNSF20	BPNSF22	BPNSF4	BPNSF12	BPNSF16	BPNS
**BPNSF12**	-.371	-.261	-.341	-.314	.237	.309	.374	.281	-.370	-.346	-.383	-.387	.337	1.000		
**BPNSF16**	-.417	-.347	-.380	-.426	.220	.323	.341	.294	-.370	-.371	-.399	-.347	.345	.525	1.000	
**BPNSF24**	-.317	-.284	-.335	-.371	.261	.354	.244	.222	-.444	-.359	-.418	-.417	.298	.491	.430	1.0
**BPNSF5**	.235	.295	.174	.193	-.153	-.204	-.174	-.202	.243	.210	.163	.206	-.104	-.294	-.323	-.2
**BPNSF10**	.249	.440	.227	.232	-.178	-.239	-.175	-.204	.256	.319	.184	.152	-.234	-.303	-.285	-.2
**BPNSF15**	.152	.212	.138	.072	-.194	-.161	-.001	-.179	.064	.169	.106	.102	-.162	-.270	-.153	-.1
**BPNSF18**	-.159	-.069	-.048	-.113	.001	.051	-.067	.088	-.047	-.060	-.110	-.031	-.056	-.051	.014	-.0
**BPNSF1**	-.199	-.184	-.114	-.190	.225	.116	.074	.197	-.148	-.134	-.181	-.150	.256	.221	.164	.1
**BPNSF7**	-.208	-.253	-.129	-.281	.204	.297	.211	.317	-.296	-.123	-.190	-.176	.080	.114	.088	.1
**BPNSF13**	-.378	-.342	-.343	-.387	.311	.349	.592	.437	-.371	-.262	-.399	-.273	.266	.432	.353	.2
**BPNSF19**	-.312	-.373	-.390	-.448	.298	.302	.413	.521	-.416	-.286	-.422	-.287	.177	.317	.330	.2

Sample Correlations - Upper Bounds (BC) (g1 - Structural covariances)

	BPNSF6	BPNSF11	BPNSF17	BPNSF23	BPNSF3	BPNSF9	BPNSF14	BPNSF21	BPNSF2	BPNSF8	BPNSF20	BPNSF22	BPNSF4	BPNSF12	BPNSF16	BPNS
**BPNSF6**	1.000															
**BPNSF11**	.641	1.000														
**BPNSF17**	.600	.616	1.000													
**BPNSF23**	.629	.639	.627	1.000												
**BPNSF3**	-.315	-.264	-.256	-.401	1.000											
**BPNSF9**	-.329	-.308	-.310	-.394	.589	1.000										
**BPNSF14**	-.266	-.248	-.295	-.348	.563	.627	1.000									
**BPNSF21**	-.223	-.227	-.174	-.334	.479	.549	.624	1.000								
**BPNSF2**	.558	.592	.507	.637	-.348	-.304	-.312	-.276	1.000							
**BPNSF8**	.607	.615	.587	.549	-.177	-.277	-.204	-.106	.609	1.000						
**BPNSF20**	.582	.567	.617	.694	-.323	-.310	-.302	-.245	.614	.602	1.000					
**BPNSF22**	.453	.519	.421	.548	-.237	-.207	-.112	-.117	.559	.630	.580	1.000				
**BPNSF4**	-.168	-.114	-.116	-.109	.476	.352	.450	.343	-.193	-.119	-.193	-.125	1.000			
**BPNSF12**	-.204	-.102	-.185	-.162	.411	.477	.566	.443	-.206	-.168	-.225	-.231	.499	1.000		
**BPNSF16**	-.250	-.192	-.221	-.261	.398	.486	.532	.455	-.200	-.201	-.235	-.196	.484	.666	1.000	
**BPNSF24**	-.153	-.133	-.178	-.205	.427	.524	.430	.409	-.287	-.189	-.249	-.244	.467	.628	.599	1.0
**BPNSF5**	.393	.441	.349	.348	.004	-.052	-.029	-.041	.389	.383	.336	.368	.057	-.127	-.163	-.1
**BPNSF10**	.412	.582	.407	.388	-.007	-.069	.013	-.031	.412	.483	.350	.316	-.040	-.119	-.107	-.0
**BPNSF15**	.294	.371	.303	.223	-.009	.018	.173	.002	.233	.355	.256	.268	.018	-.091	.034	-.0
**BPNSF18**	.005	.101	.128	.046	.165	.223	.127	.266	.101	.143	.046	.144	.108	.152	.205	.1
**BPNSF1**	-.043	-.042	.045	-.047	.380	.275	.253	.360	-.002	.010	-.040	-.015	.413	.386	.304	.3
**BPNSF7**	-.035	-.103	.013	-.141	.372	.455	.386	.475	-.150	.030	-.034	-.012	.244	.280	.250	.2
**BPNSF13**	-.203	-.179	-.180	-.229	.469	.520	.713	.589	-.203	-.095	-.234	-.116	.416	.600	.516	.4
**BPNSF19**	-.166	-.208	-.224	-.316	.474	.472	.588	.673	-.256	-.101	-.242	-.119	.349	.474	.506	.4

Sample Correlations - Two Tailed Significance (BC) (g1 - Structural covariances)

	BPNSF6	BPNSF11	BPNSF17	BPNSF23	BPNSF3	BPNSF9	BPNSF14	BPNSF21	BPNSF2	BPNSF8	BPNSF20	BPNSF22	BPNSF4	BPNSF12	BPNSF16	BPNS
**BPNSF6**	...															
**BPNSF11**	.003	...														
**BPNSF17**	.019	.012	...													
**BPNSF23**	.004	.007	.009	...												
**BPNSF3**	.003	.008	.007	.005	...											
**BPNSF9**	.003	.004	.008	.010	.007	...										
**BPNSF14**	.008	.003	.009	.007	.016	.021	...									
**BPNSF21**	.005	.011	.006	.012	.005	.008	.010	...								
**BPNSF2**	.009	.006	.006	.023	.009	.010	.013	.014	...							
**BPNSF8**	.010	.016	.005	.012	.005	.006	.004	.005	.003	...						
**BPNSF20**	.002	.005	.004	.007	.004	.006	.007	.010	.009	.005	...					
**BPNSF22**	.010	.008	.019	.016	.003	.003	.009	.007	.004	.006	.005	...				
**BPNSF4**	.007	.005	.005	.018	.006	.008	.023	.006	.007	.002	.003	.009	...			
**BPNSF12**	.008	.010	.019	.023	.019	.011	.012	.006	.013	.006	.015	.010	.012	...		
**BPNSF16**	.005	.005	.012	.009	.014	.013	.012	.014	.011	.009	.013	.007	.009	.012	...	
**BPNSF24**	.012	.016	.021	.013	.015	.006	.013	.006	.005	.005	.012	.011	.009	.012	.019	
**BPNSF5**	.013	.011	.014	.012	.111	.015	.044	.023	.011	.009	.019	.005	.594	.012	.006	.0
**BPNSF10**	.011	.010	.006	.007	.081	.012	.177	.011	.011	.009	.005	.009	.027	.009	.003	.0
**BPNSF15**	.021	.026	.012	.010	.085	.192	.117	.111	.009	.009	.018	.026	.164	.009	.284	.0
**BPNSF18**	.144	.732	.525	.519	.096	.030	.606	.021	.611	.349	.372	.294	.670	.349	.070	.5
**BPNSF1**	.015	.016	.503	.009	.006	.007	.014	.009	.093	.166	.011	.049	.010	.003	.007	.0
**BPNSF7**	.007	.004	.174	.004	.005	.009	.009	.013	.012	.283	.006	.028	.009	.004	.012	.0
**BPNSF13**	.005	.004	.011	.012	.010	.019	.007	.011	.009	.004	.003	.006	.009	.010	.005	.0
**BPNSF19**	.007	.012	.005	.012	.005	.011	.011	.018	.013	.005	.007	.010	.007	.010	.005	.0

Sample Means (g1 - Structural covariances)

Sample Means - Lower Bounds (BC) (g1 - Structural covariances)

	BPNSF6	BPNSF11	BPNSF17	BPNSF23	BPNSF3	BPNSF9	BPNSF14	BPNSF21	BPNSF2	BPNSF8	BPNSF20	BPNSF22	BPNSF4	BPNSF12	BPNSF16	BPNSF[illegible]
**BPNSF6**	2.352	2.588	2.316	1.968	5.611	5.703	5.446	5.227	2.118	2.631	2.225	2.898	4.947	5.113	4.999	5.12[illegible]

Sample Means - Upper Bounds (BC) (g1 - Structural covariances)

	BPNSF6	BPNSF11	BPNSF17	BPNSF23	BPNSF3	BPNSF9	BPNSF14	BPNSF21	BPNSF2	BPNSF8	BPNSF20	BPNSF22	BPNSF4	BPNSF12	BPNSF16	BPNSF[illegible]
**BPNSF6**	2.645	2.823	2.609	2.236	5.816	5.943	5.696	5.480	2.383	2.909	2.483	3.139	5.151	5.333	5.213	5.35[illegible]

Sample Means - Two Tailed Significance (BC) (g1 - Structural covariances)

	BPNSF6	BPNSF11	BPNSF17	BPNSF23	BPNSF3	BPNSF9	BPNSF14	BPNSF21	BPNSF2	BPNSF8	BPNSF20	BPNSF22	BPNSF4	BPNSF12	BPNSF16	BPNSF[illegible]
**BPNSF6**	.003	.005	.005	.003	.016	.023	.020	.015	.005	.009	.006	.011	.032	.019	.019	.01[illegible]

g2 (g2 - Structural covariances)

Estimates (g2 - Structural covariances)

Scalar Estimates (g2 - Structural covariances)

Maximum Likelihood Estimates

Regression Weights: (g2 - Structural covariances)

			Estimate	S.E.	C.R.	P	Label
BPNSF19	<---	F1	1.000				
BPNSF13	<---	F1	1.137	.051	22.120	***	a1_1
BPNSF7	<---	F1	.855	.050	17.033	***	a2_1
BPNSF1	<---	F1	.763	.050	15.221	***	a3_1
BPNSF18	<---	F2	1.000				
BPNSF15	<---	F2	2.472	.310	7.981	***	a4_1
BPNSF10	<---	F2	3.106	.377	8.240	***	a5_1
BPNSF5	<---	F2	2.568	.317	8.091	***	a6_1
BPNSF24	<---	F3	1.000				
BPNSF16	<---	F3	1.130	.046	24.395	***	a7_1
BPNSF12	<---	F3	1.224	.048	25.722	***	a8_1
BPNSF4	<---	F3	.850	.043	19.567	***	a9_1
BPNSF22	<---	F4	1.000				
BPNSF20	<---	F4	1.196	.049	24.557	***	a10_1
BPNSF8	<---	F4	1.200	.050	23.963	***	a11_1
BPNSF2	<---	F4	1.121	.049	23.044	***	a12_1
BPNSF21	<---	F5	1.000				
BPNSF14	<---	F5	1.094	.042	25.957	***	a13_1
BPNSF9	<---	F5	1.039	.042	24.620	***	a14_1
BPNSF3	<---	F5	.881	.042	21.077	***	a15_1
BPNSF23	<---	F6	1.000				
BPNSF17	<---	F6	.976	.035	28.242	***	a16_1
BPNSF11	<---	F6	.959	.034	28.176	***	a17_1
BPNSF6	<---	F6	.863	.035	24.658	***	a18_1

Standardized Regression Weights: (g2 - Structural covariances)

			Estimate
BPNSF19	<---	F1	.600
BPNSF13	<---	F1	.769
BPNSF7	<---	F1	.558
BPNSF1	<---	F1	.511
BPNSF18	<---	F2	.257
BPNSF15	<---	F2	.572
BPNSF10	<---	F2	.713
BPNSF5	<---	F2	.637
BPNSF24	<---	F3	.666
BPNSF16	<---	F3	.755
BPNSF12	<---	F3	.823
BPNSF4	<---	F3	.580
BPNSF22	<---	F4	.671
BPNSF20	<---	F4	.765
BPNSF8	<---	F4	.721

			Estimate
BPNSF2	<---	F4	.730
BPNSF21	<---	F5	.697
BPNSF14	<---	F5	.773
BPNSF9	<---	F5	.711
BPNSF3	<---	F5	.604
BPNSF23	<---	F6	.782
BPNSF17	<---	F6	.754
BPNSF11	<---	F6	.703
BPNSF6	<---	F6	.635

Intercepts: (g2 - Structural covariances)

	Estimate	S.E.	C.R.	P	Label
**BPNSF19**	5.161	.041	127.263	***	i1_1
**BPNSF13**	5.068	.039	129.843	***	i2_1
**BPNSF7**	4.834	.041	118.944	***	i3_1
**BPNSF1**	4.719	.041	114.400	***	i4_1
**BPNSF18**	4.323	.041	104.415	***	i5_1
**BPNSF15**	3.715	.046	80.790	***	i6_1
**BPNSF10**	3.176	.046	68.997	***	i7_1
**BPNSF5**	3.738	.044	84.870	***	i8_1
**BPNSF24**	5.223	.038	138.988	***	i9_1
**BPNSF16**	5.115	.038	133.720	***	i10_1
**BPNSF12**	5.218	.039	134.852	***	i11_1
**BPNSF4**	5.114	.037	139.031	***	i12_1
**BPNSF22**	3.120	.043	72.371	***	i13_1
**BPNSF20**	2.508	.046	54.169	***	i14_1
**BPNSF8**	2.806	.048	58.739	***	i15_1
**BPNSF2**	2.383	.047	51.118	***	i16_1
**BPNSF21**	5.200	.040	130.424	***	i17_1
**BPNSF14**	5.464	.040	135.863	***	i18_1
**BPNSF9**	5.679	.040	140.701	***	i19_1
**BPNSF3**	5.610	.040	139.703	***	i20_1
**BPNSF23**	2.241	.046	49.111	***	i21_1
**BPNSF17**	2.577	.047	54.540	***	i22_1
**BPNSF11**	2.744	.047	58.985	***	i23_1
**BPNSF6**	2.565	.047	54.736	***	i24_1

Covariances: (g2 - Structural covariances)

			Estimate	S.E.	C.R.	P	Label
F1	<-->	F2	-.156	.024	-6.584	***	ccc1_1
F2	<-->	F3	-.149	.022	-6.643	***	ccc2_1
F1	<-->	F3	.728	.048	15.066	***	ccc3_1
F2	<-->	F4	.314	.041	7.635	***	ccc4_1
F3	<-->	F4	-.596	.045	-13.381	***	ccc5_1
F1	<-->	F4	-.490	.043	-11.286	***	ccc6_1
F2	<-->	F5	-.143	.023	-6.324	***	ccc7_1
F4	<-->	F5	-.668	.049	-13.722	***	ccc8_1
F3	<-->	F5	.770	.049	15.782	***	ccc9_1
F1	<-->	F5	.903	.056	16.114	***	ccc10_1
F6	<-->	F5	-.958	.060	-15.868	***	ccc11_1
F6	<-->	F3	-.641	.050	-12.931	***	ccc12_1
F6	<-->	F4	1.266	.073	17.426	***	ccc13_1
F6	<-->	F2	.372	.048	7.715	***	ccc14_1
F6	<-->	F1	-.622	.052	-12.029	***	ccc15_1

Correlations: (g2 - Structural covariances)

			Estimate
F1	<-->	F2	-.433
F2	<-->	F3	-.411
F1	<-->	F3	.836
F2	<-->	F4	.758
F3	<-->	F4	-.595
F1	<-->	F4	-.494
F2	<-->	F5	-.363
F4	<-->	F5	-.615
F3	<-->	F5	.808
F1	<-->	F5	.956

	Estimate
F6 <--> F5	-.723
F6 <--> F3	-.525
F6 <--> F4	.910
F6 <--> F2	.737
F6 <--> F1	-.514

Variances: (g2 - Structural covariances)

	Estimate	S.E.	C.R.	P	Label
**F1**	.863	.072	12.009	***	vvv1_1
**F2**	.150	.036	4.231	***	vvv2_1
**F3**	.879	.065	13.450	***	vvv3_1
**F4**	1.141	.085	13.446	***	vvv4_1
**F5**	1.033	.073	14.100	***	vvv5_1
**F6**	1.696	.103	16.468	***	vvv6_1
**e1**	1.532	.112	13.704	***	v1_2
**e2**	.769	.066	11.729	***	v2_2
**e3**	1.399	.101	13.916	***	v3_2
**e4**	1.427	.101	14.106	***	v4_2
**e5**	2.129	.147	14.520	***	v5_2
**e6**	1.889	.145	13.052	***	v6_2
**e7**	1.399	.127	11.018	***	v7_2
**e8**	1.452	.118	12.324	***	v8_2
**e9**	1.102	.084	13.103	***	v9_2
**e10**	.847	.071	11.965	***	v10_2
**e11**	.626	.061	10.300	***	v11_2
**e12**	1.252	.091	13.714	***	v12_2
**e13**	1.394	.105	13.280	***	v13_2
**e14**	1.156	.095	12.166	***	v14_2
**e15**	1.521	.119	12.797	***	v15_2
**e16**	1.259	.099	12.672	***	v16_2
**e17**	1.095	.082	13.399	***	v17_2
**e18**	.832	.066	12.543	***	v18_2
**e19**	1.089	.082	13.278	***	v19_2
**e20**	1.400	.100	13.951	***	v20_2
**e21**	1.076	.089	12.140	***	v21_2
**e22**	1.227	.098	12.579	***	v22_2
**e23**	1.596	.121	13.169	***	v23_2
**e24**	1.864	.136	13.664	***	v24_2

Matrices (g2 - Structural covariances)

Residual Covariances (g2 - Structural covariances)

	BPNSF6	BPNSF11	BPNSF17	BPNSF23	BPNSF3	BPNSF9	BPNSF14	BPNSF21	BPNSF2	BPNSF8	BPNSF20	BPNSF22	BPNSF4	BPNSF12	BPNSF16	BPNS[illegible]
**BPNSF6**	-.191															
**BPNSF11**	-.217	-.264														
**BPNSF17**	-.082	-.048	.053													
**BPNSF23**	-.141	-.186	.081	-.044												
**BPNSF3**	-.127	.144	-.040	-.044	.073											
**BPNSF9**	.074	.024	-.184	-.052	.184	-.005										
**BPNSF14**	.227	.220	-.107	.190	-.018	.080	-.050									
**BPNSF21**	.127	.070	.152	.069	.138	-.080	-.013	.097								
**BPNSF2**	-.035	-.244	-.020	-.047	-.106	.029	.017	-.006	-.083							
**BPNSF8**	-.019	-.202	-.056	-.209	.024	.040	.166	.049	-.054	-.112						
**BPNSF20**	-.230	-.159	.002	.225	-.148	.004	-.015	-.200	-.017	-.027	.130					
**BPNSF22**	-.091	-.051	.059	.033	.127	-.066	.017	-.152	.027	.036	.244	.204				
**BPNSF4**	-.175	-.070	-.136	-.155	.828	.195	.110	.436	-.289	-.168	-.352	-.257	.266			
**BPNSF12**	.099	.044	-.086	-.012	.049	-.002	.024	.138	-.086	.031	-.057	-.308	.230	.107		
**BPNSF16**	.106	.000	-.076	.030	-.009	-.048	.077	.306	-.053	.007	-.194	-.288	.181	.198	.186	
**BPNSF24**	.004	-.034	-.295	-.089	-.049	-.009	.132	.211	-.213	-.190	-.342	-.539	.187	.110	.257	.1[illegible]
**BPNSF5**	.078	-.252	-.195	-.060	-.119	.058	.044	-.151	-.067	.151	.050	.147	-.401	-.019	.034	-.1[illegible]
**BPNSF10**	-.108	.295	.076	.096	-.238	-.312	-.256	-.444	.025	.166	.207	.304	-.315	-.133	-.129	-.2[illegible]
**BPNSF15**	.075	.286	.104	-.046	-.084	.050	.088	-.094	-.014	.169	.186	.391	-.216	-.038	-.028	-.2[illegible]
**BPNSF18**	-.103	.284	.218	-.006	.138	.019	.085	-.104	.001	.127	.287	.208	-.043	.006	-.034	-.0[illegible]
**BPNSF1**	.123	.105	.091	.063	.208	-.053	.010	.205	-.221	-.024	-.186	-.168	.391	.027	.010	.0[illegible]
**BPNSF7**	.419	-.003	-.165	.192	.010	.147	.005	.040	-.028	-.103	-.026	-.280	.234	-.011	.044	.1[illegible]
**BPNSF13**	.077	-.098	-.013	.027	.002	-.145	.015	.189	-.010	-.112	-.113	-.283	.214	.191	.104	.1[illegible]
**BPNSF19**	-.072	-.258	-.290	-.250	-.024	.042	.044	.251	-.013	-.106	-.258	-.307	.201	-.069	-.048	-.0[illegible]

Residual Means (g2 - Structural covariances)

	BPNSF6	BPNSF11	BPNSF17	BPNSF23	BPNSF3	BPNSF9	BPNSF14	BPNSF21	BPNSF2	BPNSF8	BPNSF20	BPNSF22	BPNSF4	BPNSF12	BPNSF16	BPNSF24	BP
	.051	.016	-.103	-.068	.010	.046	.142	.040	-.075	-.075	-.069	-.041	-.037	.097	.103	.088	-.

Standardized Residual Covariances (g2 - Structural covariances)

	BPNSF6	BPNSF11	BPNSF17	BPNSF23	BPNSF3	BPNSF9	BPNSF14	BPNSF21	BPNSF2	BPNSF8	BPNSF20	BPNSF22	BPNSF4	BPNSF12	BPNSF16	BPNS
**BPNSF6**	-.901															
**BPNSF11**	-1.318	-1.234														
**BPNSF17**	-.518	-.296	.273													
**BPNSF23**	-.896	-1.154	.519	-.234												
**BPNSF3**	-.971	1.088	-.316	-.353	.492											
**BPNSF9**	.561	.181	-1.435	-.408	1.600	-.031										
**BPNSF14**	1.757	1.671	-.846	1.520	-.155	.683	-.357									
**BPNSF21**	.979	.529	1.207	.550	1.229	-.689	-.112	.673								
**BPNSF2**	-.234	-1.584	-.133	-.317	-.877	.234	.139	-.048	-.453							
**BPNSF8**	-.117	-1.214	-.352	-1.309	.185	.302	1.278	.374	-.345	-.523						
**BPNSF20**	-1.490	-1.007	.015	1.485	-1.196	.033	-.121	-1.629	-.115	-.169	.686					
**BPNSF22**	-.628	-.345	.417	.235	1.089	-.560	.146	-1.317	.195	.237	1.704	1.188				
**BPNSF4**	-1.480	-.582	-1.196	-1.375	8.161	1.889	1.094	4.315	-2.592	-1.395	-3.102	-2.389	2.078			
**BPNSF12**	.806	.354	-.727	-.100	.460	-.019	.225	1.281	-.739	.244	-.478	-2.755	2.259	.814		
**BPNSF16**	.868	.002	-.641	.254	-.088	-.440	.719	2.868	-.457	.059	-1.631	-2.573	1.794	1.793	1.393	
**BPNSF24**	.033	-.272	-2.511	-.763	-.469	-.086	1.253	2.006	-1.849	-1.522	-2.905	-4.851	1.886	1.023	2.426	1.3
**BPNSF5**	.562	-1.799	-1.460	-.450	-1.065	.513	.407	-1.362	-.515	1.070	.378	1.170	-3.853	-.179	.313	-1.3
**BPNSF10**	-.720	1.927	.519	.658	-1.956	-2.554	-2.160	-3.708	.174	1.077	1.416	2.220	-2.795	-1.144	-1.114	-2.4
**BPNSF15**	.511	1.921	.733	-.326	-.698	.411	.749	-.796	-.103	1.127	1.315	2.940	-1.943	-.331	-.243	-1.7
**BPNSF18**	-.801	2.195	1.775	-.049	1.281	.173	.811	-.984	.007	.978	2.353	1.790	-.435	.063	-.331	-.8
**BPNSF1**	1.030	.871	.796	.557	2.026	-.507	.101	2.001	-1.989	-.199	-1.640	-1.560	4.151	.276	.103	.9
**BPNSF7**	3.413	-.027	-1.404	1.648	.092	1.360	.049	.378	-.241	-.833	-.225	-2.535	2.406	-.106	.437	1.1
**BPNSF13**	.638	-.812	-.111	.233	.018	-1.320	.134	1.754	-.089	-.927	-.992	-2.617	2.215	1.839	1.011	1.7
**BPNSF19**	-.539	-1.919	-2.263	-1.971	-.204	.356	.377	2.151	-.103	-.783	-2.029	-2.549	1.892	-.615	-.431	-.5

Standardized Residual Means (g2 - Structural covariances)

	BPNSF6	BPNSF11	BPNSF17	BPNSF23	BPNSF3	BPNSF9	BPNSF14	BPNSF21	BPNSF2	BPNSF8	BPNSF20	BPNSF22	BPNSF4	BPNSF12	BPNSF16	BPNSF24	BP
	.602	.194	-1.274	-.853	.134	.642	2.065	.572	-.948	-.878	-.860	-.541	-.565	1.446	1.529	1.305	-1.

Notes for Group/Model (g2 - Structural covariances)

The following covariance matrix is not positive definite (g2 - Structural covariances)

	F5	F4	F3	F2	F1	F6
**F5**	1.033					
**F4**	-.668	1.141				
**F3**	.770	-.596	.879			
**F2**	-.143	.314	-.149	.150		
**F1**	.903	-.490	.728	-.156	.863	
**F6**	-.958	1.266	-.641	.372	-.622	1.696

This solution is not admissible.

Modification Indices (g2 - Structural covariances)

Covariances: (g2 - Structural covariances)

			M.I.	Par Change
e24	<-->	F1	4.902	.101
e20	<-->	e24	7.319	-.223
e19	<-->	F6	4.677	-.095
e19	<-->	e20	6.657	.164
e18	<-->	e22	8.098	-.159
e18	<-->	e19	6.243	.128
e17	<-->	F2	5.691	-.045
e17	<-->	F1	6.550	.089
e17	<-->	F6	5.973	.107
e17	<-->	e22	14.992	.242
e17	<-->	e19	7.149	-.153
e15	<-->	e24	4.283	.185
e15	<-->	e21	5.607	-.169
e14	<-->	e24	7.925	-.224

			M.I.	Par Change
e14	<-->	e21	15.560	.251
e13	<-->	F5	11.355	.127
e13	<-->	F3	9.565	-.130
e13	<-->	F1	7.014	-.106
e13	<-->	e20	15.974	.289
e12	<-->	F5	17.639	.148
e12	<-->	F3	4.636	-.083
e12	<-->	F2	8.701	-.059
e12	<-->	F6	7.241	.126
e12	<-->	e22	5.542	.156
e12	<-->	e20	103.362	.685
e12	<-->	e18	7.769	-.152
e12	<-->	e17	5.348	.140
e11	<-->	e14	4.995	.117
e10	<-->	e20	5.575	-.139
e10	<-->	e17	7.753	.148
e9	<-->	F4	7.870	-.109
e9	<-->	e20	9.303	-.197
e9	<-->	e13	10.606	-.215
e8	<-->	e24	7.207	.237
e8	<-->	e23	11.109	-.277
e8	<-->	e22	5.214	-.170
e8	<-->	e15	4.321	.171
e8	<-->	e12	13.782	-.269
e7	<-->	F5	13.207	-.147
e7	<-->	e24	4.914	-.202
e7	<-->	e23	6.101	.211
e7	<-->	e17	5.137	-.159
e6	<-->	e23	5.857	.224
e6	<-->	e21	4.155	-.161
e5	<-->	e24	4.137	-.203
e5	<-->	e23	4.051	.189
e5	<-->	e20	4.047	.172
e5	<-->	e6	7.256	.275
e4	<-->	F4	5.367	-.099
e4	<-->	F2	4.677	-.045
e4	<-->	F6	14.491	.188
e4	<-->	e22	5.632	.165
e4	<-->	e20	5.868	.172
e4	<-->	e16	7.135	-.188
e4	<-->	e12	8.280	.195
e4	<-->	e7	7.000	.207
e4	<-->	e6	16.144	-.341
e4	<-->	e5	7.128	-.229
e3	<-->	e24	18.396	.354
e3	<-->	e22	6.606	-.178
e3	<-->	e21	6.702	.171
e3	<-->	e19	8.039	.180
e3	<-->	e13	5.453	-.169
e2	<-->	F3	5.781	.077
e2	<-->	e23	4.687	-.133
e2	<-->	e19	11.980	-.173
e2	<-->	e12	5.087	-.120
e2	<-->	e11	11.403	.144
e1	<-->	F3	4.208	-.088
e1	<-->	F1	5.316	-.089
e1	<-->	F6	6.478	-.132
e1	<-->	e17	5.163	.152
e1	<-->	e16	4.946	.164

Variances: (g2 - Structural covariances)

	M.I.	Par Change

Regression Weights: (g2 - Structural covariances)

			M.I.	Par Change
BPNSF6	<---	F5	4.575	.150
BPNSF6	<---	F3	4.505	.165
BPNSF6	<---	F1	5.311	.179
BPNSF6	<---	BPNSF14	4.180	.025

			M.I.	Par Change
BPNSF6	<---	BPNSF16	4.008	.026
BPNSF6	<---	BPNSF24	4.094	.025
BPNSF6	<---	BPNSF1	4.245	.028
BPNSF6	<---	BPNSF7	8.069	.038
BPNSF3	<---	BPNSF6	4.427	-.039
BPNSF14	<---	BPNSF6	4.774	.033
BPNSF14	<---	BPNSF11	6.165	.036
BPNSF14	<---	BPNSF23	6.940	.044
BPNSF14	<---	BPNSF8	5.178	.032
BPNSF14	<---	BPNSF22	6.024	.033
BPNSF14	<---	BPNSF15	4.928	.026
BPNSF21	<---	F3	6.547	.154
BPNSF21	<---	F1	5.017	.134
BPNSF22	<---	F3	11.140	-.227
BPNSF22	<---	F2	7.945	.487
BPNSF22	<---	F1	5.950	-.166
BPNSF4	<---	F5	11.039	.190
BPNSF4	<---	F4	6.081	-.136
BPNSF4	<---	F3	5.984	.155
BPNSF4	<---	F2	6.919	-.425
BPNSF4	<---	F1	15.496	.250
BPNSF4	<---	F6	8.049	-.128
BPNSF4	<---	BPNSF6	8.599	-.052
BPNSF4	<---	BPNSF11	4.121	-.034
BPNSF4	<---	BPNSF23	5.889	-.048
BPNSF4	<---	BPNSF2	8.720	-.057
BPNSF4	<---	BPNSF8	5.532	-.039
BPNSF4	<---	BPNSF20	8.996	-.055
BPNSF4	<---	BPNSF5	10.904	-.045
BPNSF4	<---	BPNSF10	6.228	-.039
BPNSF4	<---	BPNSF15	5.607	-.032
BPNSF24	<---	F4	6.896	-.139
BPNSF24	<---	F2	5.493	-.363
BPNSF24	<---	F6	4.046	-.087
BPNSF5	<---	BPNSF11	5.294	-.044
BPNSF10	<---	F5	11.132	-.221
BPNSF10	<---	F1	9.673	-.228
BPNSF10	<---	BPNSF11	5.508	.046
BPNSF18	<---	F2	4.606	.439
BPNSF18	<---	BPNSF6	7.523	-.062
BPNSF18	<---	BPNSF23	4.919	-.056
BPNSF18	<---	BPNSF3	5.283	-.028
BPNSF18	<---	BPNSF9	6.971	-.032
BPNSF18	<---	BPNSF14	6.500	-.032
BPNSF18	<---	BPNSF21	8.085	-.037
BPNSF18	<---	BPNSF2	4.799	-.053
BPNSF18	<---	BPNSF4	6.738	-.035
BPNSF18	<---	BPNSF12	7.172	-.035
BPNSF18	<---	BPNSF16	7.834	-.037
BPNSF18	<---	BPNSF24	7.856	-.036
BPNSF18	<---	BPNSF10	4.791	-.043
BPNSF18	<---	BPNSF1	10.947	-.047
BPNSF18	<---	BPNSF7	7.287	-.038
BPNSF18	<---	BPNSF13	6.675	-.035
BPNSF18	<---	BPNSF19	7.172	-.035
BPNSF1	<---	BPNSF17	4.433	.040
BPNSF1	<---	BPNSF4	4.738	.024
BPNSF19	<---	F6	6.156	-.123

Means: (g2 - Structural covariances)

	M.I.	Par Change

Intercepts: (g2 - Structural covariances)

	M.I.	Par Change
**BPNSF18**	7.800	-.197

Bootstrap (g2 - Structural covariances)

Bootstrap standard errors (g2 - Structural covariances)

Scalar Estimates (g2 - Structural covariances)

Regression Weights: (g2 - Structural covariances)

Parameter			SE	SE-SE	Mean	Bias	SE-Bias
BPNSF19	<---	F1	.000	.000	1.000	.000	.000
BPNSF13	<---	F1	.056	.003	1.132	-.005	.004
BPNSF7	<---	F1	.059	.003	.855	.000	.004
BPNSF1	<---	F1	.071	.004	.762	-.001	.005
BPNSF18	<---	F2	.000	.000	1.000	.000	.000
BPNSF15	<---	F2	.396	.020	2.525	.054	.028
BPNSF10	<---	F2	.522	.026	3.180	.074	.037
BPNSF5	<---	F2	.422	.021	2.629	.061	.030
BPNSF24	<---	F3	.000	.000	1.000	.000	.000
BPNSF16	<---	F3	.053	.003	1.129	-.001	.004
BPNSF12	<---	F3	.049	.002	1.218	-.006	.003
BPNSF4	<---	F3	.051	.003	.848	-.002	.004
BPNSF22	<---	F4	.000	.000	1.000	.000	.000
BPNSF20	<---	F4	.052	.003	1.195	-.001	.004
BPNSF8	<---	F4	.050	.002	1.195	-.005	.004
BPNSF2	<---	F4	.054	.003	1.120	-.001	.004
BPNSF21	<---	F5	.000	.000	1.000	.000	.000
BPNSF14	<---	F5	.045	.002	1.096	.002	.003
BPNSF9	<---	F5	.052	.003	1.041	.002	.004
BPNSF3	<---	F5	.058	.003	.885	.004	.004
BPNSF23	<---	F6	.000	.000	1.000	.000	.000
BPNSF17	<---	F6	.034	.002	.979	.003	.002
BPNSF11	<---	F6	.038	.002	.959	.000	.003
BPNSF6	<---	F6	.037	.002	.862	.000	.003

Standardized Regression Weights: (g2 - Structural covariances)

Parameter			SE	SE-SE	Mean	Bias	SE-Bias
BPNSF19	<---	F1	.034	.002	.603	.002	.002
BPNSF13	<---	F1	.023	.001	.770	.000	.002
BPNSF7	<---	F1	.038	.002	.560	.002	.003
BPNSF1	<---	F1	.034	.002	.512	.001	.002
BPNSF18	<---	F2	.039	.002	.257	.000	.003
BPNSF15	<---	F2	.032	.002	.572	.000	.002
BPNSF10	<---	F2	.031	.002	.716	.003	.002
BPNSF5	<---	F2	.030	.002	.637	.000	.002
BPNSF24	<---	F3	.029	.001	.668	.002	.002
BPNSF16	<---	F3	.025	.001	.756	.001	.002
BPNSF12	<---	F3	.018	.001	.824	.001	.001
BPNSF4	<---	F3	.036	.002	.580	.000	.003
BPNSF22	<---	F4	.024	.001	.670	-.001	.002
BPNSF20	<---	F4	.028	.001	.767	.002	.002
BPNSF8	<---	F4	.027	.001	.718	-.002	.002
BPNSF2	<---	F4	.029	.001	.733	.003	.002
BPNSF21	<---	F5	.025	.001	.697	.000	.002
BPNSF14	<---	F5	.031	.002	.776	.003	.002
BPNSF9	<---	F5	.033	.002	.714	.003	.002
BPNSF3	<---	F5	.040	.002	.608	.004	.003
BPNSF23	<---	F6	.024	.001	.783	.000	.002
BPNSF17	<---	F6	.027	.001	.757	.003	.002
BPNSF11	<---	F6	.027	.001	.703	.000	.002
BPNSF6	<---	F6	.026	.001	.635	-.001	.002

Intercepts: (g2 - Structural covariances)

Parameter	SE	SE-SE	Mean	Bias	SE-Bias
**BPNSF19**	.044	.002	5.167	.006	.003
**BPNSF13**	.040	.002	5.074	.006	.003
**BPNSF7**	.042	.002	4.842	.008	.003
**BPNSF1**	.039	.002	4.726	.007	.003
**BPNSF18**	.042	.002	4.322	.000	.003
**BPNSF15**	.046	.002	3.714	-.001	.003
**BPNSF10**	.049	.002	3.172	-.004	.003
**BPNSF5**	.045	.002	3.735	-.003	.003

Parameter	SE	SE-SE	Mean	Bias	SE-Bias
**BPNSF24**	.040	.002	5.227	.004	.003
**BPNSF16**	.042	.002	5.121	.006	.003
**BPNSF12**	.037	.002	5.224	.006	.003
**BPNSF4**	.039	.002	5.118	.004	.003
**BPNSF22**	.045	.002	3.115	-.004	.003
**BPNSF20**	.052	.003	2.498	-.010	.004
**BPNSF8**	.052	.003	2.803	-.003	.004
**BPNSF2**	.046	.002	2.379	-.004	.003
**BPNSF21**	.039	.002	5.208	.008	.003
**BPNSF14**	.041	.002	5.473	.008	.003
**BPNSF9**	.044	.002	5.686	.006	.003
**BPNSF3**	.041	.002	5.617	.007	.003
**BPNSF23**	.044	.002	2.233	-.008	.003
**BPNSF17**	.052	.003	2.568	-.009	.004
**BPNSF11**	.048	.002	2.742	-.003	.003
**BPNSF6**	.051	.003	2.563	-.002	.004

Covariances: (g2 - Structural covariances)

Parameter	SE	SE-SE	Mean	Bias	SE-Bias
F1 <--> F2	.025	.001	-.157	-.001	.002
F2 <--> F3	.025	.001	-.150	.000	.002
F1 <--> F3	.047	.002	.730	.001	.003
F2 <--> F4	.051	.003	.314	.000	.004
F3 <--> F4	.044	.002	-.601	-.005	.003
F1 <--> F4	.043	.002	-.492	-.002	.003
F2 <--> F5	.024	.001	-.143	.000	.002
F4 <--> F5	.043	.002	-.666	.002	.003
F3 <--> F5	.052	.003	.770	.000	.004
F1 <--> F5	.075	.004	.906	.003	.005
F6 <--> F5	.058	.003	-.957	.001	.004
F6 <--> F3	.049	.002	-.645	-.005	.003
F6 <--> F4	.070	.003	1.264	-.002	.005
F6 <--> F2	.058	.003	.371	-.001	.004
F6 <--> F1	.055	.003	-.628	-.006	.004

Correlations: (g2 - Structural covariances)

Parameter	SE	SE-SE	Mean	Bias	SE-Bias
F1 <--> F2	.039	.002	-.434	-.002	.003
F2 <--> F3	.036	.002	-.410	.000	.003
F1 <--> F3	.026	.001	.833	-.003	.002
F2 <--> F4	.025	.001	.757	-.001	.002
F3 <--> F4	.027	.001	-.598	-.002	.002
F1 <--> F4	.033	.002	-.494	.000	.002
F2 <--> F5	.038	.002	-.363	.000	.003
F4 <--> F5	.028	.001	-.612	.002	.002
F3 <--> F5	.022	.001	.805	-.003	.002
F1 <--> F5	.023	.001	.954	-.002	.002
F6 <--> F5	.025	.001	-.723	.001	.002
F6 <--> F3	.031	.002	-.528	-.003	.002
F6 <--> F4	.018	.001	.909	-.002	.001
F6 <--> F2	.029	.001	.734	-.002	.002
F6 <--> F1	.033	.002	-.518	-.003	.002

Variances: (g2 - Structural covariances)

Parameter	SE	SE-SE	Mean	Bias	SE-Bias
**F1**	.081	.004	.871	.008	.006
**F2**	.047	.002	.154	.004	.003
**F3**	.065	.003	.885	.006	.005
**F4**	.080	.004	1.144	.003	.006
**F5**	.080	.004	1.037	.003	.006
**F6**	.102	.005	1.694	-.001	.007
**e1**	.187	.009	1.531	-.001	.013
**e2**	.088	.004	.764	-.005	.006
**e3**	.157	.008	1.393	-.006	.011
**e4**	.115	.006	1.416	-.011	.008
**e5**	.138	.007	2.141	.013	.010
**e6**	.164	.008	1.893	.004	.012

Parameter	SE	SE-SE	Mean	Bias	SE-Bias
**e7**	.173	.009	1.383	-.017	.012
**e8**	.147	.007	1.463	.012	.010
**e9**	.132	.007	1.098	-.004	.009
**e10**	.102	.005	.843	-.005	.007
**e11**	.071	.004	.619	-.008	.005
**e12**	.142	.007	1.258	.006	.010
**e13**	.124	.006	1.404	.009	.009
**e14**	.173	.009	1.144	-.013	.012
**e15**	.189	.009	1.534	.013	.013
**e16**	.177	.009	1.240	-.019	.013
**e17**	.114	.006	1.096	.001	.008
**e18**	.131	.007	.825	-.007	.009
**e19**	.168	.008	1.079	-.011	.012
**e20**	.199	.010	1.387	-.012	.014
**e21**	.147	.007	1.075	-.001	.010
**e22**	.172	.009	1.214	-.012	.012
**e23**	.196	.010	1.601	.005	.014
**e24**	.206	.010	1.871	.007	.015

Matrices (g2 - Structural covariances)

Sample Covariances - Standard Errors (g2 - Structural covariances)

	BPNSF6	BPNSF11	BPNSF17	BPNSF23	BPNSF3	BPNSF9	BPNSF14	BPNSF21	BPNSF2	BPNSF8	BPNSF20	BPNSF22	BPNSF4	BPNSF12	BPNSF16	BPNS[illegible]
**BPNSF6**	.181															
**BPNSF11**	.156	.172														
**BPNSF17**	.159	.159	.203													
**BPNSF23**	.150	.147	.164	.192												
**BPNSF3**	.134	.128	.124	.133	.183											
**BPNSF9**	.125	.137	.149	.135	.133	.194										
**BPNSF14**	.115	.139	.136	.123	.134	.156	.159									
**BPNSF21**	.120	.132	.136	.132	.123	.132	.139	.158								
**BPNSF2**	.143	.130	.136	.149	.107	.111	.112	.104	.169							
**BPNSF8**	.159	.151	.162	.146	.128	.128	.131	.134	.137	.169						
**BPNSF20**	.161	.141	.153	.171	.141	.137	.144	.144	.140	.157	.199					
**BPNSF22**	.147	.136	.136	.139	.120	.128	.133	.137	.125	.148	.139	.144				
**BPNSF4**	.132	.127	.120	.139	.136	.121	.123	.120	.111	.126	.147	.131	.143			
**BPNSF12**	.129	.132	.136	.135	.137	.136	.141	.133	.116	.129	.137	.127	.121	.140		
**BPNSF16**	.122	.126	.128	.133	.138	.132	.121	.138	.118	.136	.158	.134	.127	.133	.162	
**BPNSF24**	.122	.124	.135	.129	.132	.130	.124	.126	.129	.138	.142	.133	.128	.135	.134	[illegible]
**BPNSF5**	.137	.135	.143	.150	.135	.119	.110	.132	.111	.147	.163	.151	.139	.133	.122	[illegible]
**BPNSF10**	.145	.141	.146	.148	.138	.130	.122	.133	.128	.144	.152	.149	.140	.133	.135	[illegible]
**BPNSF15**	.136	.144	.159	.146	.136	.120	.121	.135	.136	.159	.153	.151	.138	.137	.134	[illegible]
**BPNSF18**	.138	.141	.140	.137	.119	.113	.103	.121	.128	.142	.135	.142	.133	.138	.122	[illegible]
**BPNSF1**	.104	.114	.101	.117	.106	.111	.109	.109	.113	.142	.142	.136	.118	.110	.105	[illegible]
**BPNSF7**	.129	.130	.138	.116	.134	.137	.123	.119	.102	.141	.119	.125	.115	.129	.121	[illegible]
**BPNSF13**	.119	.125	.124	.119	.112	.124	.131	.119	.104	.119	.124	.119	.106	.116	.115	[illegible]
**BPNSF19**	.130	.138	.134	.136	.122	.152	.134	.135	.103	.132	.141	.127	.125	.132	.120	[illegible]

Sample Correlations - Standard Errors (g2 - Structural covariances)

	BPNSF6	BPNSF11	BPNSF17	BPNSF23	BPNSF3	BPNSF9	BPNSF14	BPNSF21	BPNSF2	BPNSF8	BPNSF20	BPNSF22	BPNSF4	BPNSF12	BPNSF16	BPNS[illegible]
**BPNSF6**	.000															
**BPNSF11**	.049	.000														
**BPNSF17**	.052	.047	.000													
**BPNSF23**	.046	.044	.045	.000												
**BPNSF3**	.053	.052	.050	.053	.000											
**BPNSF9**	.051	.047	.052	.049	.058	.000										
**BPNSF14**	.047	.054	.047	.050	.059	.052	.000									
**BPNSF21**	.045	.049	.052	.046	.046	.050	.052	.000								
**BPNSF2**	.048	.047	.046	.048	.046	.047	.047	.041	.000							
**BPNSF8**	.049	.050	.046	.045	.049	.047	.051	.049	.043	.000						
**BPNSF20**	.052	.045	.045	.043	.052	.051	.054	.048	.047	.045	.000					
**BPNSF22**	.050	.047	.045	.043	.050	.049	.054	.051	.042	.045	.038	.000				
**BPNSF4**	.050	.051	.045	.051	.036	.048	.049	.039	.045	.048	.050	.051	.000			
**BPNSF12**	.052	.053	.051	.050	.058	.053	.049	.042	.048	.050	.050	.045	.047	.000		
**BPNSF16**	.049	.048	.049	.052	.062	.056	.044	.046	.046	.049	.052	.046	.051	.036	.000	
**BPNSF24**	.050	.049	.050	.050	.060	.055	.047	.047	.047	.048	.049	.043	.051	.045	.042	[illegible]
**BPNSF5**	.046	.050	.051	.051	.054	.050	.048	.054	.040	.048	.053	.052	.052	.059	.052	[illegible]
**BPNSF10**	.048	.043	.043	.042	.053	.049	.047	.045	.046	.044	.045	.047	.053	.053	.051	[illegible]
**BPNSF15**	.044	.045	.052	.047	.052	.046	.049	.051	.047	.049	.047	.047	.052	.054	.050	[illegible]

	BPNSF6	BPNSF11	BPNSF17	BPNSF23	BPNSF3	BPNSF9	BPNSF14	BPNSF21	BPNSF2	BPNSF8	BPNSF20	BPNSF22	BPNSF4	BPNSF12	BPNSF16	BPNS[illegible]
**BPNSF18**	.053	.050	.051	.053	.051	.049	.048	.052	.051	.052	.048	.054	.059	.063	.053	.0[illegible]
**BPNSF1**	.043	.048	.042	.049	.047	.051	.047	.045	.047	.055	.055	.055	.051	.051	.048	.0[illegible]
**BPNSF7**	.052	.054	.052	.050	.059	.051	.051	.050	.046	.054	.049	.052	.050	.055	.054	.0[illegible]
**BPNSF13**	.050	.050	.050	.049	.050	.052	.047	.038	.045	.046	.047	.046	.043	.035	.046	.0[illegible]
**BPNSF19**	.050	.052	.049	.051	.051	.056	.054	.047	.043	.048	.050	.050	.048	.052	.051	.0[illegible]

Sample Means - Standard Errors (g2 - Structural covariances)

	BPNSF6	BPNSF11	BPNSF17	BPNSF23	BPNSF3	BPNSF9	BPNSF14	BPNSF21	BPNSF2	BPNSF8	BPNSF20	BPNSF22	BPNSF4	BPNSF12	BPNSF16	BPNSF[illegible]
**BPNSF6**	.079	.090	.088	.076	.073	.074	.067	.076	.078	.091	.087	.088	.073	.067	.072	.07[illegible]

Bootstrap Confidence (g2 - Structural covariances)

Percentile method (g2 - Structural covariances)

90% confidence intervals (percentile method)

Scalar Estimates (g2 - Structural covariances)

Regression Weights: (g2 - Structural covariances)

Parameter			Estimate	Lower	Upper	P
BPNSF19	<---	F1	1.000	1.000	1.000	...
BPNSF13	<---	F1	1.137	1.035	1.224	.010
BPNSF7	<---	F1	.855	.759	.953	.010
BPNSF1	<---	F1	.763	.647	.886	.010
BPNSF18	<---	F2	1.000	1.000	1.000	...
BPNSF15	<---	F2	2.472	2.005	3.283	.010
BPNSF10	<---	F2	3.106	2.481	4.237	.010
BPNSF5	<---	F2	2.568	2.058	3.451	.010
BPNSF24	<---	F3	1.000	1.000	1.000	...
BPNSF16	<---	F3	1.130	1.045	1.218	.010
BPNSF12	<---	F3	1.224	1.141	1.298	.010
BPNSF4	<---	F3	.850	.757	.938	.010
BPNSF22	<---	F4	1.000	1.000	1.000	...
BPNSF20	<---	F4	1.196	1.117	1.287	.010
BPNSF8	<---	F4	1.200	1.118	1.276	.010
BPNSF2	<---	F4	1.121	1.040	1.220	.010
BPNSF21	<---	F5	1.000	1.000	1.000	...
BPNSF14	<---	F5	1.094	1.025	1.171	.010
BPNSF9	<---	F5	1.039	.965	1.132	.010
BPNSF3	<---	F5	.881	.793	.984	.010
BPNSF23	<---	F6	1.000	1.000	1.000	...
BPNSF17	<---	F6	.976	.924	1.040	.010
BPNSF11	<---	F6	.959	.898	1.018	.010
BPNSF6	<---	F6	.863	.803	.929	.010

Standardized Regression Weights: (g2 - Structural covariances)

Parameter			Estimate	Lower	Upper	P
BPNSF19	<---	F1	.600	.553	.659	.010
BPNSF13	<---	F1	.769	.734	.807	.010
BPNSF7	<---	F1	.558	.499	.631	.010
BPNSF1	<---	F1	.511	.448	.565	.010
BPNSF18	<---	F2	.257	.195	.321	.010
BPNSF15	<---	F2	.572	.519	.627	.010
BPNSF10	<---	F2	.713	.665	.766	.010
BPNSF5	<---	F2	.637	.585	.691	.010
BPNSF24	<---	F3	.666	.622	.720	.010
BPNSF16	<---	F3	.755	.711	.797	.010
BPNSF12	<---	F3	.823	.792	.853	.010
BPNSF4	<---	F3	.580	.526	.646	.010
BPNSF22	<---	F4	.671	.633	.709	.010
BPNSF20	<---	F4	.765	.722	.811	.010
BPNSF8	<---	F4	.721	.675	.759	.010
BPNSF2	<---	F4	.730	.678	.779	.010
BPNSF21	<---	F5	.697	.654	.738	.010
BPNSF14	<---	F5	.773	.725	.827	.010
BPNSF9	<---	F5	.711	.653	.764	.010

Parameter	Estimate	Lower	Upper	P
BPNSF3 <--- F5	.604	.546	.673	.010
BPNSF23 <--- F6	.782	.740	.825	.010
BPNSF17 <--- F6	.754	.716	.800	.010
BPNSF11 <--- F6	.703	.660	.743	.010
BPNSF6 <--- F6	.635	.593	.676	.010

Intercepts: (g2 - Structural covariances)

Parameter	Estimate	Lower	Upper	P
**BPNSF19**	5.161	5.091	5.232	.010
**BPNSF13**	5.068	5.007	5.137	.010
**BPNSF7**	4.834	4.769	4.910	.010
**BPNSF1**	4.719	4.664	4.798	.010
**BPNSF18**	4.323	4.258	4.394	.010
**BPNSF15**	3.715	3.630	3.788	.010
**BPNSF10**	3.176	3.091	3.248	.010
**BPNSF5**	3.738	3.659	3.813	.010
**BPNSF24**	5.223	5.159	5.293	.010
**BPNSF16**	5.115	5.050	5.196	.010
**BPNSF12**	5.218	5.165	5.289	.010
**BPNSF4**	5.114	5.059	5.184	.010
**BPNSF22**	3.120	3.039	3.185	.010
**BPNSF20**	2.508	2.405	2.582	.010
**BPNSF8**	2.806	2.712	2.889	.010
**BPNSF2**	2.383	2.304	2.456	.010
**BPNSF21**	5.200	5.131	5.272	.010
**BPNSF14**	5.464	5.408	5.547	.010
**BPNSF9**	5.679	5.606	5.758	.010
**BPNSF3**	5.610	5.551	5.699	.010
**BPNSF23**	2.241	2.158	2.305	.010
**BPNSF17**	2.577	2.481	2.646	.010
**BPNSF11**	2.744	2.665	2.823	.010
**BPNSF6**	2.565	2.477	2.642	.010

Covariances: (g2 - Structural covariances)

Parameter	Estimate	Lower	Upper	P
F1 <--> F2	-.156	-.195	-.115	.010
F2 <--> F3	-.149	-.191	-.110	.010
F1 <--> F3	.728	.655	.809	.010
F2 <--> F4	.314	.228	.402	.010
F3 <--> F4	-.596	-.672	-.526	.010
F1 <--> F4	-.490	-.566	-.422	.010
F2 <--> F5	-.143	-.184	-.104	.010
F4 <--> F5	-.668	-.738	-.597	.010
F3 <--> F5	.770	.687	.857	.010
F1 <--> F5	.903	.796	1.035	.010
F6 <--> F5	-.958	-1.064	-.863	.010
F6 <--> F3	-.641	-.716	-.564	.010
F6 <--> F4	1.266	1.153	1.379	.010
F6 <--> F2	.372	.277	.465	.010
F6 <--> F1	-.622	-.721	-.541	.010

Correlations: (g2 - Structural covariances)

Parameter	Estimate	Lower	Upper	P
F1 <--> F2	-.433	-.494	-.364	.010
F2 <--> F3	-.411	-.467	-.354	.010
F1 <--> F3	.836	.790	.876	.010
F2 <--> F4	.758	.719	.796	.010
F3 <--> F4	-.595	-.639	-.553	.010
F1 <--> F4	-.494	-.545	-.436	.010
F2 <--> F5	-.363	-.415	-.287	.010
F4 <--> F5	-.615	-.656	-.564	.010
F3 <--> F5	.808	.764	.840	.010
F1 <--> F5	.956	.915	.993	.010
F6 <--> F5	-.723	-.764	-.679	.010
F6 <--> F3	-.525	-.574	-.476	.010
F6 <--> F4	.910	.877	.937	.010
F6 <--> F2	.737	.684	.779	.010

Parameter	Estimate	Lower	Upper	P
F6 <--> F1	-.514	-.564	-.463	.010

Variances: (g2 - Structural covariances)

Parameter	Estimate	Lower	Upper	P
**F1**	.863	.734	.992	.010
**F2**	.150	.083	.234	.010
**F3**	.879	.772	.991	.010
**F4**	1.141	1.018	1.269	.010
**F5**	1.033	.920	1.173	.010
**F6**	1.696	1.532	1.865	.010
**e1**	1.532	1.224	1.883	.010
**e2**	.769	.612	.911	.010
**e3**	1.399	1.128	1.658	.010
**e4**	1.427	1.230	1.614	.010
**e5**	2.129	1.902	2.383	.010
**e6**	1.889	1.622	2.173	.010
**e7**	1.399	1.103	1.727	.010
**e8**	1.452	1.250	1.717	.010
**e9**	1.102	.848	1.325	.010
**e10**	.847	.675	1.012	.010
**e11**	.626	.509	.734	.010
**e12**	1.252	1.023	1.491	.010
**e13**	1.394	1.196	1.597	.010
**e14**	1.156	.877	1.466	.010
**e15**	1.521	1.266	1.854	.010
**e16**	1.259	.980	1.577	.010
**e17**	1.095	.928	1.289	.010
**e18**	.832	.613	1.048	.010
**e19**	1.089	.845	1.390	.010
**e20**	1.400	1.034	1.728	.010
**e21**	1.076	.856	1.333	.010
**e22**	1.227	.952	1.498	.010
**e23**	1.596	1.290	1.945	.010
**e24**	1.864	1.503	2.233	.010

Matrices (g2 - Structural covariances)

Sample Covariances (g2 - Structural covariances)

Sample Covariances - Lower Bounds (PC) (g2 - Structural covariances)

	BPNSF6	BPNSF11	BPNSF17	BPNSF23	BPNSF3	BPNSF9	BPNSF14	BPNSF21	BPNSF2	BPNSF8	BPNSF20	BPNSF22	BPNSF4	BPNSF12	BPNSF16	BPNS[illegible]
**BPNSF6**	2.618															
**BPNSF11**	.891	2.597														
**BPNSF17**	1.099	1.287	2.541													
**BPNSF23**	1.081	1.203	1.450	2.385												
**BPNSF3**	-1.078	-.866	-1.056	-1.127	1.965											
**BPNSF9**	-1.015	-1.177	-1.431	-1.265	.913	1.897										
**BPNSF14**	-.874	-1.012	-1.348	-1.044	.754	1.010	1.748									
**BPNSF21**	-.915	-1.066	-1.031	-1.106	.857	.778	.899	1.959								
**BPNSF2**	.965	.899	1.151	1.121	-.956	-.940	-1.002	-.931	2.284							
**BPNSF8**	1.011	.971	1.151	1.055	-.895	-.996	-.946	-.985	1.223	2.758						
**BPNSF20**	.801	1.035	1.212	1.446	-1.053	-1.035	-1.137	-1.258	1.290	1.348	2.521					
**BPNSF22**	.752	.931	1.041	1.068	-.676	-1.006	-.936	-1.043	1.090	1.169	1.364	2.498				
**BPNSF4**	-.885	-.814	-.880	-.941	1.173	.673	.637	.916	-1.049	-.999	-1.237	-1.002	1.923			
**BPNSF12**	-.778	-.940	-1.092	-1.000	.654	.739	.819	.888	-1.124	-1.045	-1.155	-1.267	.938	1.827		
**BPNSF16**	-.719	-.926	-.978	-.913	.538	.631	.831	.934	-1.028	-1.033	-1.288	-1.198	.810	1.198	1.895	
**BPNSF24**	-.737	-.847	-1.146	-.926	.429	.575	.735	.777	-1.115	-1.137	-1.285	-1.369	.738	.941	1.007	1.8[illegible]
**BPNSF5**	.684	.456	.496	.656	-.665	-.538	-.550	-.775	.645	.884	.725	.739	-.987	-.715	-.620	-.7[illegible]
**BPNSF10**	.650	1.183	.956	1.030	-.873	-.978	-.940	-1.126	.892	1.084	1.097	1.050	-.987	-.940	-.880	-.9[illegible]
**BPNSF15**	.651	.919	.724	.599	-.635	-.542	-.504	-.671	.609	.824	.881	.943	-.793	-.735	-.697	-.7[illegible]
**BPNSF18**	-.024	.403	.363	.122	-.204	-.336	-.254	-.435	.137	.257	.410	.275	-.392	-.396	-.398	-.4[illegible]
**BPNSF1**	-.470	-.552	-.523	-.597	.643	.475	.604	.740	-.827	-.743	-.863	-.792	.644	.520	.468	.4[illegible]
**BPNSF7**	-.244	-.762	-.915	-.539	.442	.741	.658	.623	-.661	-.827	-.754	-.921	.583	.530	.540	.5[illegible]
**BPNSF13**	-.722	-.995	-.929	-.885	.720	.729	.930	1.031	-.832	-.991	-.971	-1.061	.744	1.025	.841	.7[illegible]
**BPNSF19**	-.851	-1.074	-1.109	-1.157	.574	.754	.806	.933	-.719	-.930	-1.087	-.986	.648	.610	.556	.4[illegible]

Sample Covariances - Upper Bounds (PC) (g2 - Structural covariances)

	BPNSF6	BPNSF11	BPNSF17	BPNSF23	BPNSF3	BPNSF9	BPNSF14	BPNSF21	BPNSF2	BPNSF8	BPNSF20	BPNSF22	BPNSF4	BPNSF12	BPNSF16	BPNS[illegible]
**BPNSF6**	3.251															
**BPNSF11**	1.425	3.169														
**BPNSF17**	1.595	1.810	3.245													
**BPNSF23**	1.584	1.676	2.000	3.053												
**BPNSF3**	-.650	-.448	-.648	-.676	2.566											
**BPNSF9**	-.586	-.744	-.942	-.817	1.345	2.548										
**BPNSF14**	-.493	-.572	-.893	-.643	1.201	1.519	2.302									
**BPNSF21**	-.503	-.620	-.574	-.653	1.279	1.249	1.355	2.472								
**BPNSF2**	1.441	1.349	1.577	1.621	-.598	-.572	-.618	-.594	2.898							
**BPNSF8**	1.553	1.500	1.644	1.551	-.483	-.577	-.500	-.546	1.696	3.333						
**BPNSF20**	1.316	1.532	1.729	1.999	-.607	-.574	-.639	-.792	1.753	1.857	3.246					
**BPNSF22**	1.237	1.372	1.534	1.533	-.291	-.563	-.475	-.601	1.499	1.645	1.824	2.983				
**BPNSF4**	-.437	-.371	-.489	-.480	1.640	1.085	1.067	1.304	-.653	-.554	-.730	-.547	2.385			
**BPNSF12**	-.340	-.507	-.655	-.582	1.082	1.200	1.305	1.332	-.734	-.612	-.695	-.844	1.341	2.296		
**BPNSF16**	-.306	-.496	-.583	-.467	.968	1.086	1.222	1.390	-.639	-.581	-.747	-.758	1.232	1.657	2.452	
**BPNSF24**	-.327	-.452	-.719	-.506	.844	.994	1.163	1.190	-.688	-.684	-.821	-.928	1.159	1.393	1.469	[illegible]
**BPNSF5**	1.136	.911	.966	1.162	-.245	-.151	-.178	-.314	1.011	1.377	1.261	1.229	-.509	-.283	-.202	[illegible]
**BPNSF10**	1.137	1.621	1.428	1.500	-.390	-.556	-.553	-.667	1.337	1.589	1.645	1.547	-.490	-.512	-.434	[illegible]
**BPNSF15**	1.106	1.386	1.251	1.116	-.142	-.128	-.100	-.216	1.052	1.355	1.367	1.438	-.316	-.252	-.244	[illegible]
**BPNSF18**	.438	.903	.819	.590	.215	.062	.090	-.035	.554	.739	.862	.751	.053	.064	.006	[illegible]
**BPNSF1**	-.131	-.178	-.188	-.209	1.001	.837	.963	1.074	-.462	-.269	-.387	-.324	1.052	.889	.831	[illegible]
**BPNSF7**	.184	-.300	-.440	-.154	.911	1.185	1.051	1.006	-.318	-.376	-.329	-.487	.966	.980	.950	[illegible]
**BPNSF13**	-.356	-.563	-.515	-.489	1.090	1.143	1.358	1.452	-.481	-.584	-.595	-.644	1.103	1.400	1.247	[illegible]
**BPNSF19**	-.412	-.587	-.661	-.677	.975	1.255	1.271	1.407	-.380	-.494	-.630	-.586	1.049	1.075	.966	[illegible]

Sample Covariances - Two Tailed Significance (PC) (g2 - Structural covariances)

	BPNSF6	BPNSF11	BPNSF17	BPNSF23	BPNSF3	BPNSF9	BPNSF14	BPNSF21	BPNSF2	BPNSF8	BPNSF20	BPNSF22	BPNSF4	BPNSF12	BPNSF16	BPNS[illegible]
**BPNSF6**	.010															
**BPNSF11**	.010	.010														
**BPNSF17**	.010	.010	.010													
**BPNSF23**	.010	.010	.010	.010												
**BPNSF3**	.010	.010	.010	.010	.010											
**BPNSF9**	.010	.010	.010	.010	.010	.010										
**BPNSF14**	.010	.010	.010	.010	.010	.010	.010									
**BPNSF21**	.010	.010	.010	.010	.010	.010	.010	.010								
**BPNSF2**	.010	.010	.010	.010	.010	.010	.010	.010	.010							
**BPNSF8**	.010	.010	.010	.010	.010	.010	.010	.010	.010	.010						
**BPNSF20**	.010	.010	.010	.010	.010	.010	.010	.010	.010	.010	.010					
**BPNSF22**	.010	.010	.010	.010	.010	.010	.010	.010	.010	.010	.010	.010				
**BPNSF4**	.010	.010	.010	.010	.010	.010	.010	.010	.010	.010	.010	.010	.010			
**BPNSF12**	.010	.010	.010	.010	.010	.010	.010	.010	.010	.010	.010	.010	.010	.010		
**BPNSF16**	.010	.010	.010	.010	.010	.010	.010	.010	.010	.010	.010	.010	.010	.010	.010	
**BPNSF24**	.010	.010	.010	.010	.010	.010	.010	.010	.010	.010	.010	.010	.010	.010	.010	[illegible]
**BPNSF5**	.010	.010	.010	.010	.010	.021	.010	.010	.010	.010	.010	.010	.010	.010	.013	[illegible]
**BPNSF10**	.010	.010	.010	.010	.010	.010	.010	.010	.010	.010	.010	.010	.010	.010	.010	[illegible]
**BPNSF15**	.010	.010	.010	.010	.016	.010	.023	.010	.010	.010	.010	.010	.010	.010	.010	[illegible]
**BPNSF18**	.131	.010	.010	.016	.946	.259	.576	.040	.010	.010	.010	.010	.162	.274	.108	[illegible]
**BPNSF1**	.010	.010	.010	.010	.010	.010	.010	.010	.010	.010	.010	.010	.010	.010	.010	[illegible]
**BPNSF7**	.692	.010	.010	.014	.010	.010	.010	.010	.010	.010	.010	.010	.010	.010	.010	[illegible]
**BPNSF13**	.010	.010	.010	.010	.010	.010	.010	.010	.010	.010	.010	.010	.010	.010	.010	[illegible]
**BPNSF19**	.010	.010	.010	.010	.010	.010	.010	.010	.010	.010	.010	.010	.010	.010	.010	[illegible]

Sample Correlations (g2 - Structural covariances)

Sample Correlations - Lower Bounds (PC) (g2 - Structural covariances)

	BPNSF6	BPNSF11	BPNSF17	BPNSF23	BPNSF3	BPNSF9	BPNSF14	BPNSF21	BPNSF2	BPNSF8	BPNSF20	BPNSF22	BPNSF4	BPNSF12	BPNSF16	BPNS[illegible]
**BPNSF6**	1.000															
**BPNSF11**	.309	1.000														
**BPNSF17**	.379	.452	1.000													
**BPNSF23**	.390	.438	.543	1.000												
**BPNSF3**	-.420	-.355	-.418	-.454	1.000											
**BPNSF9**	-.397	-.453	-.547	-.507	.412	1.000										
**BPNSF14**	-.359	-.418	-.538	-.449	.363	.496	1.000									
**BPNSF21**	-.355	-.407	-.399	-.436	.387	.361	.447	1.000								
**BPNSF2**	.355	.334	.414	.432	-.402	-.399	-.437	-.386	1.000							
**BPNSF8**	.346	.325	.405	.376	-.355	-.389	-.368	-.371	.454	1.000						
**BPNSF20**	.278	.367	.425	.539	-.408	-.406	-.452	-.473	.474	.466	1.000					
**BPNSF22**	.262	.323	.377	.399	-.271	-.388	-.386	-.417	.421	.418	.501	1.000				
**BPNSF4**	-.344	-.336	-.347	-.374	.566	.323	.319	.437	-.430	-.379	-.467	-.398	1.000			

	BPNSF6	BPNSF11	BPNSF17	BPNSF23	BPNSF3	BPNSF9	BPNSF14	BPNSF21	BPNSF2	BPNSF8	BPNSF20	BPNSF22	BPNSF4	BPNSF12	BPNSF16	BPNS[illegible]
**BPNSF12**	-.325	-.383	-.434	-.413	.306	.359	.435	.438	-.472	-.416	-.459	-.511	.458	1.000		
**BPNSF16**	-.285	-.360	-.390	-.377	.247	.301	.416	.457	-.421	-.397	-.492	-.474	.385	.620	1.000	
**BPNSF24**	-.296	-.348	-.443	-.382	.191	.265	.389	.359	-.453	-.430	-.507	-.538	.354	.482	.508	[illegible]
**BPNSF5**	.266	.169	.187	.266	-.281	-.222	-.243	-.324	.266	.335	.288	.291	-.416	-.320	-.261	[illegible]
**BPNSF10**	.227	.415	.351	.380	-.341	-.386	-.389	-.426	.333	.382	.397	.378	-.380	-.385	-.348	[illegible]
**BPNSF15**	.218	.311	.250	.211	-.241	-.205	-.206	-.252	.220	.278	.295	.329	-.304	-.296	-.264	[illegible]
**BPNSF18**	-.009	.160	.135	.050	-.085	-.145	-.119	-.191	.057	.093	.161	.109	-.173	-.182	-.174	[illegible]
**BPNSF1**	-.193	-.234	-.220	-.255	.307	.226	.310	.351	-.361	-.300	-.356	-.335	.313	.261	.231	[illegible]
**BPNSF7**	-.099	-.309	-.363	-.228	.207	.363	.329	.296	-.289	-.333	-.297	-.376	.277	.263	.257	[illegible]
**BPNSF13**	-.314	-.421	-.391	-.386	.352	.362	.500	.528	-.368	-.398	-.411	-.444	.379	.542	.432	[illegible]
**BPNSF19**	-.323	-.417	-.431	-.444	.252	.341	.387	.434	-.296	-.339	-.414	-.385	.294	.294	.258	[illegible]

Sample Correlations - Upper Bounds (PC) (g2 - Structural covariances)

	BPNSF6	BPNSF11	BPNSF17	BPNSF23	BPNSF3	BPNSF9	BPNSF14	BPNSF21	BPNSF2	BPNSF8	BPNSF20	BPNSF22	BPNSF4	BPNSF12	BPNSF16	BPNS[illegible]
**BPNSF6**	1.000															
**BPNSF11**	.480	1.000														
**BPNSF17**	.548	.610	1.000													
**BPNSF23**	.540	.589	.697	1.000												
**BPNSF3**	-.248	-.178	-.248	-.272	1.000											
**BPNSF9**	-.230	-.291	-.372	-.348	.605	1.000										
**BPNSF14**	-.205	-.239	-.388	-.283	.548	.674	1.000									
**BPNSF21**	-.196	-.252	-.221	-.267	.543	.532	.618	1.000								
**BPNSF2**	.510	.489	.572	.589	-.245	-.238	-.271	-.242	1.000							
**BPNSF8**	.509	.499	.546	.525	-.189	-.234	-.202	-.208	.595	1.000						
**BPNSF20**	.447	.525	.586	.681	-.241	-.237	-.281	-.317	.629	.605	1.000					
**BPNSF22**	.432	.482	.529	.547	-.113	-.227	-.210	-.240	.559	.554	.625	1.000				
**BPNSF4**	-.170	-.153	-.198	-.205	.693	.481	.473	.558	-.285	-.224	-.300	-.228	1.000			
**BPNSF12**	-.143	-.213	-.276	-.254	.501	.546	.590	.578	-.314	-.251	-.287	-.364	.620	1.000		
**BPNSF16**	-.118	-.204	-.239	-.188	.441	.490	.569	.606	-.273	-.227	-.308	-.322	.564	.732	1.000	
**BPNSF24**	-.124	-.178	-.281	-.214	.376	.437	.541	.513	-.298	-.271	-.347	-.397	.521	.633	.646	[illegible]
**BPNSF5**	.423	.335	.357	.431	-.112	-.065	-.084	-.134	.399	.497	.469	.463	-.235	-.128	-.090	[illegible]
**BPNSF10**	.383	.556	.481	.515	-.151	-.224	-.233	-.277	.481	.527	.552	.537	-.200	-.214	-.178	[illegible]
**BPNSF15**	.361	.467	.421	.379	-.054	-.047	-.038	-.085	.376	.439	.454	.484	-.130	-.100	-.099	[illegible]
**BPNSF18**	.163	.336	.313	.230	.091	.028	.043	-.015	.220	.272	.324	.289	.025	.029	.003	[illegible]
**BPNSF1**	-.052	-.074	-.077	-.090	.464	.409	.463	.495	-.205	-.111	-.164	-.146	.495	.441	.387	[illegible]
**BPNSF7**	.071	-.125	-.195	-.066	.413	.537	.499	.467	-.135	-.151	-.134	-.208	.443	.455	.438	[illegible]
**BPNSF13**	-.149	-.243	-.220	-.219	.525	.537	.652	.657	-.216	-.246	-.257	-.288	.525	.661	.585	[illegible]
**BPNSF19**	-.156	-.226	-.260	-.269	.421	.532	.572	.582	-.158	-.185	-.248	-.227	.451	.466	.420	[illegible]

Sample Correlations - Two Tailed Significance (PC) (g2 - Structural covariances)

	BPNSF6	BPNSF11	BPNSF17	BPNSF23	BPNSF3	BPNSF9	BPNSF14	BPNSF21	BPNSF2	BPNSF8	BPNSF20	BPNSF22	BPNSF4	BPNSF12	BPNSF16	BPNS[illegible]
**BPNSF6**	...															
**BPNSF11**	.010	...														
**BPNSF17**	.010	.010	...													
**BPNSF23**	.010	.010	.010	...												
**BPNSF3**	.010	.010	.010	.010	...											
**BPNSF9**	.010	.010	.010	.010	.010	...										
**BPNSF14**	.010	.010	.010	.010	.010	.010	...									
**BPNSF21**	.010	.010	.010	.010	.010	.010	.010	...								
**BPNSF2**	.010	.010	.010	.010	.010	.010	.010	.010	...							
**BPNSF8**	.010	.010	.010	.010	.010	.010	.010	.010	.010	...						
**BPNSF20**	.010	.010	.010	.010	.010	.010	.010	.010	.010	.010	...					
**BPNSF22**	.010	.010	.010	.010	.010	.010	.010	.010	.010	.010	.010	...				
**BPNSF4**	.010	.010	.010	.010	.010	.010	.010	.010	.010	.010	.010	.010	...			
**BPNSF12**	.010	.010	.010	.010	.010	.010	.010	.010	.010	.010	.010	.010	.010	...		
**BPNSF16**	.010	.010	.010	.010	.010	.010	.010	.010	.010	.010	.010	.010	.010	.010	...	
**BPNSF24**	.010	.010	.010	.010	.010	.010	.010	.010	.010	.010	.010	.010	.010	.010	.010	
**BPNSF5**	.010	.010	.010	.010	.010	.021	.010	.010	.010	.010	.010	.010	.010	.010	.013	[illegible]
**BPNSF10**	.010	.010	.010	.010	.010	.010	.010	.010	.010	.010	.010	.010	.010	.010	.010	[illegible]
**BPNSF15**	.010	.010	.010	.010	.015	.010	.023	.010	.010	.010	.010	.010	.010	.010	.010	[illegible]
**BPNSF18**	.131	.010	.010	.016	.946	.259	.576	.040	.010	.010	.010	.010	.162	.274	.108	[illegible]
**BPNSF1**	.010	.010	.010	.010	.010	.010	.010	.010	.010	.010	.010	.010	.010	.010	.010	[illegible]
**BPNSF7**	.692	.010	.010	.014	.010	.010	.010	.010	.010	.010	.010	.010	.010	.010	.010	[illegible]
**BPNSF13**	.010	.010	.010	.010	.010	.010	.010	.010	.010	.010	.010	.010	.010	.010	.010	[illegible]
**BPNSF19**	.010	.010	.010	.010	.010	.010	.010	.010	.010	.010	.010	.010	.010	.010	.010	[illegible]

Sample Means (g2 - Structural covariances)

Sample Means - Lower Bounds (PC) (g2 - Structural covariances)

	BPNSF6	BPNSF11	BPNSF17	BPNSF23	BPNSF3	BPNSF9	BPNSF14	BPNSF21	BPNSF2	BPNSF8	BPNSF20	BPNSF22	BPNSF4	BPNSF12	BPNSF16	BPNSF[illegible]
**BPNSF6**	2.464	2.604	2.325	2.042	5.508	5.608	5.518	5.118	2.160	2.584	2.279	2.913	4.965	5.201	5.104	5.19[illegible]

Sample Means - Upper Bounds (PC) (g2 - Structural covariances)

	BPNSF6	BPNSF11	BPNSF17	BPNSF23	BPNSF3	BPNSF9	BPNSF14	BPNSF21	BPNSF2	BPNSF8	BPNSF20	BPNSF22	BPNSF4	BPNSF12	BPNSF16	BPNSF[illegible]
**BPNSF6**	2.725	2.904	2.613	2.284	5.742	5.857	5.728	5.391	2.414	2.889	2.604	3.216	5.211	5.424	5.337	5.43[illegible]

Sample Means - Two Tailed Significance (PC) (g2 - Structural covariances)

	BPNSF6	BPNSF11	BPNSF17	BPNSF23	BPNSF3	BPNSF9	BPNSF14	BPNSF21	BPNSF2	BPNSF8	BPNSF20	BPNSF22	BPNSF4	BPNSF12	BPNSF16	BPNSF[illegible]
**BPNSF6**	.010	.010	.010	.010	.010	.010	.010	.010	.010	.010	.010	.010	.010	.010	.010	.01[illegible]

Bias-corrected percentile method (g2 - Structural covariances)

90% confidence intervals (bias-corrected percentile method)

Scalar Estimates (g2 - Structural covariances)

Regression Weights: (g2 - Structural covariances)

Parameter			Estimate	Lower	Upper	P
BPNSF19	<---	F1	1.000	1.000	1.000	...
BPNSF13	<---	F1	1.137	1.046	1.235	.005
BPNSF7	<---	F1	.855	.759	.953	.010
BPNSF1	<---	F1	.763	.630	.865	.018
BPNSF18	<---	F2	1.000	1.000	1.000	...
BPNSF15	<---	F2	2.472	2.005	3.283	.010
BPNSF10	<---	F2	3.106	2.501	4.343	.007
BPNSF5	<---	F2	2.568	2.064	3.455	.009
BPNSF24	<---	F3	1.000	1.000	1.000	...
BPNSF16	<---	F3	1.130	1.046	1.219	.009
BPNSF12	<---	F3	1.224	1.152	1.307	.004
BPNSF4	<---	F3	.850	.755	.938	.012
BPNSF22	<---	F4	1.000	1.000	1.000	...
BPNSF20	<---	F4	1.196	1.124	1.298	.005
BPNSF8	<---	F4	1.200	1.128	1.306	.004
BPNSF2	<---	F4	1.121	1.043	1.221	.006
BPNSF21	<---	F5	1.000	1.000	1.000	...
BPNSF14	<---	F5	1.094	1.026	1.178	.009
BPNSF9	<---	F5	1.039	.970	1.136	.007
BPNSF3	<---	F5	.881	.791	.984	.012
BPNSF23	<---	F6	1.000	1.000	1.000	...
BPNSF17	<---	F6	.976	.914	1.026	.019
BPNSF11	<---	F6	.959	.904	1.023	.007
BPNSF6	<---	F6	.863	.805	.932	.008

Standardized Regression Weights: (g2 - Structural covariances)

Parameter			Estimate	Lower	Upper	P
BPNSF19	<---	F1	.600	.549	.654	.014
BPNSF13	<---	F1	.769	.734	.807	.011
BPNSF7	<---	F1	.558	.501	.632	.009
BPNSF1	<---	F1	.511	.447	.563	.012
BPNSF18	<---	F2	.257	.193	.320	.012
BPNSF15	<---	F2	.572	.519	.627	.010
BPNSF10	<---	F2	.713	.648	.752	.030
BPNSF5	<---	F2	.637	.583	.688	.013
BPNSF24	<---	F3	.666	.607	.708	.021
BPNSF16	<---	F3	.755	.708	.790	.015
BPNSF12	<---	F3	.823	.790	.850	.014
BPNSF4	<---	F3	.580	.536	.652	.004
BPNSF22	<---	F4	.671	.631	.707	.012
BPNSF20	<---	F4	.765	.718	.808	.014
BPNSF8	<---	F4	.721	.675	.760	.009
BPNSF2	<---	F4	.730	.674	.776	.015
BPNSF21	<---	F5	.697	.653	.737	.012
BPNSF14	<---	F5	.773	.707	.822	.021

Parameter			Estimate	Lower	Upper	P
BPNSF9	<---	F5	.711	.646	.752	.028
BPNSF3	<---	F5	.604	.542	.667	.018
BPNSF23	<---	F6	.782	.740	.825	.009
BPNSF17	<---	F6	.754	.700	.793	.018
BPNSF11	<---	F6	.703	.658	.743	.012
BPNSF6	<---	F6	.635	.594	.677	.009

Intercepts: (g2 - Structural covariances)

Parameter	Estimate	Lower	Upper	P
**BPNSF19**	5.161	5.069	5.220	.030
**BPNSF13**	5.068	5.002	5.129	.020
**BPNSF7**	4.834	4.747	4.893	.032
**BPNSF1**	4.719	4.652	4.783	.019
**BPNSF18**	4.323	4.257	4.393	.012
**BPNSF15**	3.715	3.618	3.779	.015
**BPNSF10**	3.176	3.096	3.253	.006
**BPNSF5**	3.738	3.667	3.815	.007
**BPNSF24**	5.223	5.143	5.277	.032
**BPNSF16**	5.115	5.046	5.189	.016
**BPNSF12**	5.218	5.160	5.277	.018
**BPNSF4**	5.114	5.037	5.171	.023
**BPNSF22**	3.120	3.047	3.194	.006
**BPNSF20**	2.508	2.432	2.602	.003
**BPNSF8**	2.806	2.721	2.891	.007
**BPNSF2**	2.383	2.314	2.463	.005
**BPNSF21**	5.200	5.118	5.247	.044
**BPNSF14**	5.464	5.388	5.523	.026
**BPNSF9**	5.679	5.589	5.744	.026
**BPNSF3**	5.610	5.553	5.700	.009
**BPNSF23**	2.241	2.176	2.321	.003
**BPNSF17**	2.577	2.497	2.668	.004
**BPNSF11**	2.744	2.670	2.828	.005
**BPNSF6**	2.565	2.479	2.648	.009

Covariances: (g2 - Structural covariances)

Parameter			Estimate	Lower	Upper	P
F1	<-->	F2	-.156	-.195	-.114	.011
F2	<-->	F3	-.149	-.191	-.110	.009
F1	<-->	F3	.728	.639	.801	.016
F2	<-->	F4	.314	.229	.406	.009
F3	<-->	F4	-.596	-.663	-.518	.019
F1	<-->	F4	-.490	-.566	-.422	.012
F2	<-->	F5	-.143	-.187	-.108	.006
F4	<-->	F5	-.668	-.742	-.608	.006
F3	<-->	F5	.770	.686	.857	.012
F1	<-->	F5	.903	.798	1.036	.009
F6	<-->	F5	-.958	-1.081	-.880	.004
F6	<-->	F3	-.641	-.711	-.536	.018
F6	<-->	F4	1.266	1.155	1.381	.008
F6	<-->	F2	.372	.280	.474	.009
F6	<-->	F1	-.622	-.718	-.538	.014

Correlations: (g2 - Structural covariances)

Parameter			Estimate	Lower	Upper	P
F1	<-->	F2	-.433	-.491	-.355	.014
F2	<-->	F3	-.411	-.475	-.357	.006
F1	<-->	F3	.836	.799	.878	.004
F2	<-->	F4	.758	.721	.800	.007
F3	<-->	F4	-.595	-.637	-.544	.015
F1	<-->	F4	-.494	-.540	-.433	.015
F2	<-->	F5	-.363	-.411	-.282	.019
F4	<-->	F5	-.615	-.659	-.566	.006
F3	<-->	F5	.808	.766	.840	.008
F1	<-->	F5	.956	.916	.994	.007
F6	<-->	F5	-.723	-.765	-.679	.009
F6	<-->	F3	-.525	-.565	-.460	.025
F6	<-->	F4	.910	.880	.943	.007

Parameter	Estimate	Lower	Upper	P
F6 <--> F2	.737	.684	.780	.009
F6 <--> F1	-.514	-.559	-.456	.023

Variances: (g2 - Structural covariances)

Parameter	Estimate	Lower	Upper	P
**F1**	.863	.726	.981	.016
**F2**	.150	.083	.232	.012
**F3**	.879	.757	.987	.016
**F4**	1.141	1.010	1.268	.012
**F5**	1.033	.918	1.171	.011
**F6**	1.696	1.534	1.876	.008
**e1**	1.532	1.224	1.885	.009
**e2**	.769	.634	.927	.006
**e3**	1.399	1.128	1.668	.009
**e4**	1.427	1.253	1.634	.005
**e5**	2.129	1.871	2.368	.016
**e6**	1.889	1.625	2.184	.007
**e7**	1.399	1.185	1.763	.002
**e8**	1.452	1.250	1.717	.010
**e9**	1.102	.866	1.332	.008
**e10**	.847	.680	1.014	.007
**e11**	.626	.520	.744	.006
**e12**	1.252	.999	1.474	.016
**e13**	1.394	1.184	1.582	.015
**e14**	1.156	.893	1.482	.005
**e15**	1.521	1.284	1.903	.006
**e16**	1.259	1.038	1.646	.003
**e17**	1.095	.936	1.295	.007
**e18**	.832	.622	1.053	.009
**e19**	1.089	.868	1.431	.005
**e20**	1.400	1.133	1.744	.004
**e21**	1.076	.860	1.364	.007
**e22**	1.227	.981	1.622	.005
**e23**	1.596	1.282	1.938	.012
**e24**	1.864	1.561	2.278	.008

Matrices (g2 - Structural covariances)

Sample Covariances (g2 - Structural covariances)

Sample Covariances - Lower Bounds (BC) (g2 - Structural covariances)

	BPNSF6	BPNSF11	BPNSF17	BPNSF23	BPNSF3	BPNSF9	BPNSF14	BPNSF21	BPNSF2	BPNSF8	BPNSF20	BPNSF22	BPNSF4	BPNSF12	BPNSF16	BPNS[illegible]
**BPNSF6**	2.589															
**BPNSF11**	.958	2.587														
**BPNSF17**	1.116	1.287	2.611													
**BPNSF23**	1.112	1.226	1.490	2.372												
**BPNSF3**	-1.069	-.875	-1.049	-1.127	1.965											
**BPNSF9**	-1.025	-1.195	-1.458	-1.308	.907	1.905										
**BPNSF14**	-.865	-1.036	-1.409	-1.084	.760	1.027	1.795									
**BPNSF21**	-.909	-1.102	-1.058	-1.099	.814	.807	.909	1.924								
**BPNSF2**	.974	.895	1.184	1.129	-.959	-.921	-1.067	-.935	2.371							
**BPNSF8**	1.012	.971	1.158	1.115	-.895	-.996	-.951	-.985	1.224	2.806						
**BPNSF20**	.812	1.076	1.258	1.500	-1.042	-1.030	-1.200	-1.266	1.312	1.403	2.598					
**BPNSF22**	.770	.968	1.127	1.068	-.675	-1.012	-.944	-1.043	1.093	1.181	1.364	2.481				
**BPNSF4**	-.851	-.827	-.881	-.941	1.168	.686	.644	.923	-1.038	-.970	-1.215	-.961	1.913			
**BPNSF12**	-.763	-.915	-1.092	-.995	.654	.755	.873	.916	-1.093	-1.056	-1.155	-1.220	.941	1.846		
**BPNSF16**	-.698	-.913	-.976	-.913	.550	.635	.834	.932	-1.028	-1.045	-1.253	-1.167	.820	1.198	1.863	
**BPNSF24**	-.701	-.847	-1.171	-.951	.434	.611	.791	.777	-1.149	-1.152	-1.285	-1.375	.733	.990	.999	1.8[illegible]
**BPNSF5**	.681	.463	.496	.640	-.646	-.500	-.543	-.727	.632	.901	.720	.755	-.956	-.715	-.620	-.7[illegible]
**BPNSF10**	.666	1.211	.958	1.080	-.886	-.985	-.964	-1.126	.917	1.120	1.103	1.072	-.955	-.956	-.872	-1.0[illegible]
**BPNSF15**	.657	.935	.758	.671	-.586	-.539	-.497	-.671	.632	.748	.886	.964	-.730	-.705	-.686	-.7[illegible]
**BPNSF18**	-.024	.386	.320	.121	-.192	-.330	-.268	-.469	.132	.257	.408	.277	-.392	-.396	-.424	-.4[illegible]
**BPNSF1**	-.422	-.549	-.520	-.597	.649	.506	.620	.741	-.826	-.743	-.840	-.782	.617	.538	.453	.4[illegible]
**BPNSF7**	-.218	-.773	-.961	-.545	.436	.743	.668	.630	-.652	-.814	-.752	-.936	.620	.569	.578	.5[illegible]
**BPNSF13**	-.711	-.995	-.940	-.866	.720	.753	.943	1.047	-.845	-.970	-.970	-1.056	.755	1.036	.832	.8[illegible]
**BPNSF19**	-.854	-1.087	-1.157	-1.157	.560	.742	.846	.942	-.716	-.930	-1.093	-.984	.649	.634	.555	.4[illegible]

Sample Covariances - Upper Bounds (BC) (g2 - Structural covariances)

	BPNSF6	BPNSF11	BPNSF17	BPNSF23	BPNSF3	BPNSF9	BPNSF14	BPNSF21	BPNSF2	BPNSF8	BPNSF20	BPNSF22	BPNSF4	BPNSF12	BPNSF16	BPNS[illegible]
**BPNSF6**	3.240															
**BPNSF11**	1.497	3.167														
**BPNSF17**	1.619	1.810	3.314													
**BPNSF23**	1.631	1.705	2.028	3.050												
**BPNSF3**	-.633	-.465	-.642	-.676	2.566											
**BPNSF9**	-.593	-.749	-.957	-.841	1.344	2.552										
**BPNSF14**	-.486	-.584	-.948	-.690	1.202	1.569	2.368									
**BPNSF21**	-.489	-.648	-.599	-.641	1.239	1.273	1.415	2.454								
**BPNSF2**	1.445	1.343	1.624	1.622	-.604	-.569	-.655	-.596	2.916							
**BPNSF8**	1.562	1.500	1.667	1.597	-.483	-.577	-.503	-.546	1.696	3.371						
**BPNSF20**	1.332	1.554	1.764	2.029	-.579	-.573	-.689	-.797	1.790	1.919	3.293					
**BPNSF22**	1.254	1.406	1.593	1.533	-.286	-.574	-.484	-.599	1.529	1.670	1.824	2.973				
**BPNSF4**	-.391	-.408	-.490	-.480	1.634	1.102	1.096	1.339	-.653	-.547	-.706	-.518	2.384			
**BPNSF12**	-.323	-.505	-.655	-.571	1.082	1.249	1.366	1.363	-.718	-.614	-.695	-.822	1.350	2.354		
**BPNSF16**	-.264	-.489	-.568	-.467	.987	1.094	1.222	1.389	-.639	-.592	-.732	-.737	1.234	1.657	2.418	
**BPNSF24**	-.304	-.450	-.727	-.536	.848	1.042	1.194	1.190	-.708	-.690	-.818	-.936	1.132	1.430	1.460	[illegible]
**BPNSF5**	1.132	.922	.977	1.134	-.213	-.114	-.167	-.292	.998	1.382	1.251	1.246	-.489	-.282	-.202	[illegible]
**BPNSF10**	1.144	1.680	1.429	1.554	-.396	-.564	-.569	-.667	1.355	1.607	1.646	1.596	-.469	-.521	-.397	[illegible]
**BPNSF15**	1.116	1.398	1.272	1.161	-.120	-.127	-.083	-.216	1.071	1.329	1.380	1.449	-.272	-.244	-.238	[illegible]
**BPNSF18**	.438	.893	.784	.588	.225	.072	.062	-.048	.552	.739	.858	.751	.053	.064	-.024	[illegible]
**BPNSF1**	-.043	-.170	-.186	-.209	1.008	.881	1.001	1.075	-.460	-.269	-.365	-.306	1.048	.922	.784	[illegible]
**BPNSF7**	.224	-.304	-.483	-.168	.876	1.186	1.060	1.016	-.309	-.355	-.327	-.517	1.004	.996	1.009	[illegible]
**BPNSF13**	-.334	-.563	-.521	-.466	1.090	1.175	1.373	1.460	-.484	-.577	-.589	-.643	1.119	1.446	1.230	[illegible]
**BPNSF19**	-.428	-.624	-.713	-.677	.961	1.251	1.285	1.420	-.374	-.494	-.631	-.585	1.057	1.084	.951	[illegible]

Sample Covariances - Two Tailed Significance (BC) (g2 - Structural covariances)

	BPNSF6	BPNSF11	BPNSF17	BPNSF23	BPNSF3	BPNSF9	BPNSF14	BPNSF21	BPNSF2	BPNSF8	BPNSF20	BPNSF22	BPNSF4	BPNSF12	BPNSF16	BPNS[illegible]
**BPNSF6**	.012															
**BPNSF11**	.003	.011														
**BPNSF17**	.006	.010	.004													
**BPNSF23**	.003	.006	.005	.011												
**BPNSF3**	.012	.007	.011	.010	.010											
**BPNSF9**	.008	.008	.005	.005	.011	.008										
**BPNSF14**	.012	.007	.002	.004	.009	.005	.003									
**BPNSF21**	.013	.006	.005	.013	.019	.006	.007	.021								
**BPNSF2**	.007	.012	.005	.009	.009	.012	.003	.009	.003							
**BPNSF8**	.009	.010	.007	.002	.010	.010	.008	.010	.009	.005						
**BPNSF20**	.008	.007	.004	.004	.012	.012	.003	.007	.004	.003	.006					
**BPNSF22**	.006	.005	.003	.010	.011	.009	.008	.012	.008	.006	.010	.012				
**BPNSF4**	.020	.006	.009	.010	.012	.007	.007	.006	.011	.015	.012	.021	.011			
**BPNSF12**	.018	.013	.010	.012	.010	.005	.005	.004	.019	.009	.010	.018	.008	.004		
**BPNSF16**	.016	.013	.013	.010	.007	.009	.009	.011	.009	.007	.014	.020	.008	.010	.020	
**BPNSF24**	.019	.011	.006	.004	.009	.003	.004	.010	.004	.009	.012	.009	.012	.004	.013	[illegible]
**BPNSF5**	.012	.006	.009	.014	.025	.044	.012	.020	.013	.007	.012	.006	.019	.011	.013	[illegible]
**BPNSF10**	.007	.004	.009	.003	.009	.008	.005	.010	.006	.005	.009	.005	.013	.006	.013	[illegible]
**BPNSF15**	.007	.006	.007	.003	.027	.011	.034	.010	.006	.021	.007	.006	.025	.012	.013	[illegible]
**BPNSF18**	.131	.013	.018	.017	.926	.292	.403	.027	.011	.010	.012	.009	.162	.274	.063	[illegible]
**BPNSF1**	.023	.012	.015	.010	.007	.005	.005	.009	.011	.010	.016	.013	.012	.005	.025	[illegible]
**BPNSF7**	.965	.009	.005	.010	.015	.009	.006	.008	.015	.013	.011	.004	.004	.006	.005	[illegible]
**BPNSF13**	.014	.010	.008	.018	.010	.006	.009	.007	.007	.012	.011	.011	.006	.004	.013	[illegible]
**BPNSF19**	.007	.006	.004	.010	.014	.012	.005	.007	.012	.010	.009	.011	.009	.006	.012	[illegible]

Sample Correlations (g2 - Structural covariances)

Sample Correlations - Lower Bounds (BC) (g2 - Structural covariances)

	BPNSF6	BPNSF11	BPNSF17	BPNSF23	BPNSF3	BPNSF9	BPNSF14	BPNSF21	BPNSF2	BPNSF8	BPNSF20	BPNSF22	BPNSF4	BPNSF12	BPNSF16	BPNS[illegible]
**BPNSF6**	1.000															
**BPNSF11**	.340	1.000														
**BPNSF17**	.373	.447	1.000													
**BPNSF23**	.407	.440	.556	1.000												
**BPNSF3**	-.409	-.357	-.413	-.439	1.000											
**BPNSF9**	-.396	-.444	-.545	-.507	.416	1.000										
**BPNSF14**	-.347	-.418	-.539	-.444	.356	.496	1.000									
**BPNSF21**	-.353	-.418	-.401	-.430	.361	.375	.446	1.000								
**BPNSF2**	.363	.337	.414	.424	-.397	-.374	-.426	-.386	1.000							
**BPNSF8**	.353	.319	.405	.391	-.347	-.378	-.370	-.374	.453	1.000						
**BPNSF20**	.277	.370	.443	.539	-.398	-.398	-.469	-.473	.470	.468	1.000					
**BPNSF22**	.261	.329	.405	.398	-.269	-.384	-.386	-.425	.421	.420	.494	1.000				
**BPNSF4**	-.332	-.339	-.351	-.374	.565	.322	.321	.435	-.426	-.374	-.450	-.390	1.000			

	BPNSF6	BPNSF11	BPNSF17	BPNSF23	BPNSF3	BPNSF9	BPNSF14	BPNSF21	BPNSF2	BPNSF8	BPNSF20	BPNSF22	BPNSF4	BPNSF12	BPNSF16	BPNS[illegible]
**BPNSF12**	-.300	-.374	-.430	-.403	.303	.360	.432	.430	-.462	-.411	-.452	-.507	.452	1.000		
**BPNSF16**	-.285	-.357	-.389	-.385	.245	.286	.424	.459	-.412	-.395	-.480	-.464	.389	.616	1.000	
**BPNSF24**	-.285	-.332	-.443	-.391	.191	.270	.395	.352	-.453	-.430	-.492	-.537	.351	.478	.469	[illegible]
**BPNSF5**	.262	.181	.175	.261	-.262	-.216	-.241	-.303	.263	.336	.278	.299	-.394	-.317	-.256	[illegible]
**BPNSF10**	.234	.415	.344	.392	-.339	-.392	-.389	-.426	.325	.388	.395	.384	-.365	-.398	-.346	[illegible]
**BPNSF15**	.227	.320	.255	.231	-.218	-.204	-.197	-.251	.223	.267	.303	.331	-.289	-.271	-.262	[illegible]
**BPNSF18**	-.009	.147	.125	.048	-.085	-.142	-.127	-.201	.067	.092	.161	.109	-.175	-.194	-.183	[illegible]
**BPNSF1**	-.173	-.228	-.213	-.253	.306	.226	.306	.350	-.356	-.281	-.338	-.327	.298	.258	.221	[illegible]
**BPNSF7**	-.093	-.308	-.367	-.227	.202	.366	.344	.302	-.274	-.318	-.311	-.406	.294	.264	.262	[illegible]
**BPNSF13**	-.298	-.414	-.383	-.367	.349	.366	.494	.528	-.363	-.395	-.406	-.444	.378	.544	.429	[illegible]
**BPNSF19**	-.318	-.419	-.447	-.434	.250	.329	.386	.425	-.279	-.327	-.407	-.382	.294	.294	.257	[illegible]

Sample Correlations - Upper Bounds (BC) (g2 - Structural covariances)

	BPNSF6	BPNSF11	BPNSF17	BPNSF23	BPNSF3	BPNSF9	BPNSF14	BPNSF21	BPNSF2	BPNSF8	BPNSF20	BPNSF22	BPNSF4	BPNSF12	BPNSF16	BPNS[illegible]
**BPNSF6**	1.000															
**BPNSF11**	.500	1.000														
**BPNSF17**	.547	.603	1.000													
**BPNSF23**	.550	.593	.704	1.000												
**BPNSF3**	-.235	-.179	-.246	-.260	1.000											
**BPNSF9**	-.226	-.288	-.371	-.340	.608	1.000										
**BPNSF14**	-.191	-.249	-.390	-.278	.531	.672	1.000									
**BPNSF21**	-.196	-.255	-.230	-.261	.530	.536	.613	1.000								
**BPNSF2**	.512	.491	.572	.583	-.241	-.229	-.261	-.242	1.000							
**BPNSF8**	.510	.497	.545	.535	-.183	-.232	-.202	-.212	.594	1.000						
**BPNSF20**	.446	.529	.598	.681	-.224	-.235	-.286	-.317	.623	.608	1.000					
**BPNSF22**	.429	.487	.549	.544	-.110	-.222	-.210	-.253	.559	.564	.622	1.000				
**BPNSF4**	-.160	-.164	-.200	-.205	.692	.481	.501	.557	-.273	-.216	-.287	-.218	1.000			
**BPNSF12**	-.111	-.207	-.258	-.245	.494	.547	.588	.574	-.305	-.249	-.282	-.344	.618	1.000		
**BPNSF16**	-.118	-.202	-.228	-.202	.441	.483	.570	.610	-.266	-.224	-.295	-.312	.568	.725	1.000	
**BPNSF24**	-.113	-.170	-.280	-.219	.374	.445	.557	.512	-.298	-.271	-.335	-.397	.520	.630	.628	[illegible]
**BPNSF5**	.418	.353	.348	.430	-.093	-.050	-.082	-.128	.399	.500	.460	.478	-.217	-.118	-.085	[illegible]
**BPNSF10**	.389	.556	.480	.528	-.149	-.228	-.234	-.277	.476	.531	.549	.538	-.185	-.216	-.168	[illegible]
**BPNSF15**	.365	.472	.429	.386	-.043	-.047	-.031	-.084	.381	.438	.454	.490	-.112	-.087	-.093	[illegible]
**BPNSF18**	.163	.326	.301	.227	.091	.030	.029	-.023	.229	.264	.324	.289	.011	.028	-.008	[illegible]
**BPNSF1**	-.016	-.070	-.074	-.084	.462	.411	.458	.491	-.203	-.108	-.145	-.126	.482	.434	.381	[illegible]
**BPNSF7**	.087	-.122	-.202	-.057	.403	.540	.517	.467	-.131	-.130	-.145	-.215	.467	.458	.459	[illegible]
**BPNSF13**	-.138	-.237	-.204	-.205	.509	.544	.649	.656	-.211	-.245	-.256	-.288	.521	.661	.581	[illegible]
**BPNSF19**	-.147	-.234	-.285	-.259	.416	.515	.572	.582	-.133	-.178	-.241	-.219	.451	.466	.418	[illegible]

Sample Correlations - Two Tailed Significance (BC) (g2 - Structural covariances)

	BPNSF6	BPNSF11	BPNSF17	BPNSF23	BPNSF3	BPNSF9	BPNSF14	BPNSF21	BPNSF2	BPNSF8	BPNSF20	BPNSF22	BPNSF4	BPNSF12	BPNSF16	BPNS[illegible]
**BPNSF6**	...															
**BPNSF11**	.004	...														
**BPNSF17**	.011	.016	...													
**BPNSF23**	.004	.008	.006	...												
**BPNSF3**	.019	.009	.012	.014	...											
**BPNSF9**	.012	.019	.013	.011	.008	...										
**BPNSF14**	.016	.009	.007	.013	.019	.011	...									
**BPNSF21**	.012	.007	.008	.015	.026	.007	.011	...								
**BPNSF2**	.006	.008	.010	.014	.012	.019	.019	.012	...							
**BPNSF8**	.006	.014	.012	.004	.015	.013	.009	.008	.011	...						
**BPNSF20**	.011	.008	.006	.010	.018	.015	.006	.010	.013	.006	...					
**BPNSF22**	.011	.007	.002	.012	.012	.014	.009	.008	.011	.006	.018	...				
**BPNSF4**	.020	.006	.007	.010	.011	.011	.007	.012	.015	.013	.021	.019	...			
**BPNSF12**	.032	.015	.020	.019	.014	.009	.012	.012	.025	.012	.021	.016	.013	...		
**BPNSF16**	.011	.012	.014	.007	.012	.013	.007	.008	.016	.011	.014	.018	.007	.014	...	
**BPNSF24**	.021	.020	.011	.006	.011	.008	.004	.015	.010	.010	.023	.011	.012	.012	.034	
**BPNSF5**	.012	.004	.013	.011	.036	.041	.012	.026	.012	.009	.015	.006	.026	.013	.018	[illegible]
**BPNSF10**	.007	.010	.013	.006	.014	.008	.009	.010	.015	.006	.012	.008	.021	.007	.012	[illegible]
**BPNSF15**	.005	.007	.007	.004	.030	.011	.036	.012	.008	.013	.009	.008	.023	.019	.012	[illegible]
**BPNSF18**	.131	.016	.020	.018	.946	.292	.403	.027	.007	.013	.010	.010	.141	.252	.075	[illegible]
**BPNSF1**	.025	.013	.018	.013	.012	.009	.012	.014	.012	.012	.020	.016	.016	.013	.019	[illegible]
**BPNSF7**	.945	.011	.006	.020	.020	.009	.004	.009	.019	.018	.006	.004	.004	.009	.006	[illegible]
**BPNSF13**	.018	.016	.016	.023	.015	.009	.016	.012	.014	.013	.012	.010	.012	.009	.014	[illegible]
**BPNSF19**	.015	.007	.003	.014	.012	.021	.011	.012	.026	.021	.013	.014	.011	.009	.012	[illegible]

Sample Means (g2 - Structural covariances)

Sample Means - Lower Bounds (BC) (g2 - Structural covariances)

	BPNSF6	BPNSF11	BPNSF17	BPNSF23	BPNSF3	BPNSF9	BPNSF14	BPNSF21	BPNSF2	BPNSF8	BPNSF20	BPNSF22	BPNSF4	BPNSF12	BPNSF16	BPNSF[illegible]
**BPNSF6**	2.451	2.619	2.333	2.062	5.494	5.608	5.463	5.096	2.184	2.604	2.319	2.940	4.964	5.201	5.101	5.17[illegible]

Sample Means - Upper Bounds (BC) (g2 - Structural covariances)

	BPNSF6	BPNSF11	BPNSF17	BPNSF23	BPNSF3	BPNSF9	BPNSF14	BPNSF21	BPNSF2	BPNSF8	BPNSF20	BPNSF22	BPNSF4	BPNSF12	BPNSF16	BPNSF[illegible]
**BPNSF6**	2.720	2.929	2.622	2.341	5.737	5.857	5.693	5.362	2.437	2.908	2.620	3.225	5.195	5.422	5.335	5.42[illegible]

Sample Means - Two Tailed Significance (BC) (g2 - Structural covariances)

	BPNSF6	BPNSF11	BPNSF17	BPNSF23	BPNSF3	BPNSF9	BPNSF14	BPNSF21	BPNSF2	BPNSF8	BPNSF20	BPNSF22	BPNSF4	BPNSF12	BPNSF16	BPNSF[illegible]
**BPNSF6**	.014	.005	.007	.004	.014	.010	.044	.025	.005	.005	.003	.006	.013	.012	.011	.01[illegible]

g3 (g3 - Structural covariances)

Estimates (g3 - Structural covariances)

Scalar Estimates (g3 - Structural covariances)

Maximum Likelihood Estimates

Regression Weights: (g3 - Structural covariances)

			Estimate	S.E.	C.R.	P	Label
BPNSF19	<---	F1	1.000				
BPNSF13	<---	F1	1.137	.051	22.120	***	a1_1
BPNSF7	<---	F1	.855	.050	17.033	***	a2_1
BPNSF1	<---	F1	.763	.050	15.221	***	a3_1
BPNSF18	<---	F2	1.000				
BPNSF15	<---	F2	2.472	.310	7.981	***	a4_1
BPNSF10	<---	F2	3.106	.377	8.240	***	a5_1
BPNSF5	<---	F2	2.568	.317	8.091	***	a6_1
BPNSF24	<---	F3	1.000				
BPNSF16	<---	F3	1.130	.046	24.395	***	a7_1
BPNSF12	<---	F3	1.224	.048	25.722	***	a8_1
BPNSF4	<---	F3	.850	.043	19.567	***	a9_1
BPNSF22	<---	F4	1.000				
BPNSF20	<---	F4	1.196	.049	24.557	***	a10_1
BPNSF8	<---	F4	1.200	.050	23.963	***	a11_1
BPNSF2	<---	F4	1.121	.049	23.044	***	a12_1
BPNSF21	<---	F5	1.000				
BPNSF14	<---	F5	1.094	.042	25.957	***	a13_1
BPNSF9	<---	F5	1.039	.042	24.620	***	a14_1
BPNSF3	<---	F5	.881	.042	21.077	***	a15_1
BPNSF23	<---	F6	1.000				
BPNSF17	<---	F6	.976	.035	28.242	***	a16_1
BPNSF11	<---	F6	.959	.034	28.176	***	a17_1
BPNSF6	<---	F6	.863	.035	24.658	***	a18_1

Standardized Regression Weights: (g3 - Structural covariances)

			Estimate
BPNSF19	<---	F1	.654
BPNSF13	<---	F1	.723
BPNSF7	<---	F1	.551
BPNSF1	<---	F1	.452
BPNSF18	<---	F2	.263
BPNSF15	<---	F2	.556
BPNSF10	<---	F2	.751
BPNSF5	<---	F2	.611
BPNSF24	<---	F3	.675
BPNSF16	<---	F3	.769
BPNSF12	<---	F3	.820
BPNSF4	<---	F3	.593
BPNSF22	<---	F4	.678
BPNSF20	<---	F4	.739
BPNSF8	<---	F4	.748

			Estimate
BPNSF2	<---	F4	.673
BPNSF21	<---	F5	.719
BPNSF14	<---	F5	.743
BPNSF9	<---	F5	.736
BPNSF3	<---	F5	.604
BPNSF23	<---	F6	.773
BPNSF17	<---	F6	.736
BPNSF11	<---	F6	.762
BPNSF6	<---	F6	.651

Intercepts: (g3 - Structural covariances)

	Estimate	S.E.	C.R.	P	Label
**BPNSF19**	5.161	.041	127.263	***	i1_1
**BPNSF13**	5.068	.039	129.843	***	i2_1
**BPNSF7**	4.834	.041	118.944	***	i3_1
**BPNSF1**	4.719	.041	114.400	***	i4_1
**BPNSF18**	4.323	.041	104.415	***	i5_1
**BPNSF15**	3.715	.046	80.790	***	i6_1
**BPNSF10**	3.176	.046	68.997	***	i7_1
**BPNSF5**	3.738	.044	84.870	***	i8_1
**BPNSF24**	5.223	.038	138.988	***	i9_1
**BPNSF16**	5.115	.038	133.720	***	i10_1
**BPNSF12**	5.218	.039	134.852	***	i11_1
**BPNSF4**	5.114	.037	139.031	***	i12_1
**BPNSF22**	3.120	.043	72.371	***	i13_1
**BPNSF20**	2.508	.046	54.169	***	i14_1
**BPNSF8**	2.806	.048	58.739	***	i15_1
**BPNSF2**	2.383	.047	51.118	***	i16_1
**BPNSF21**	5.200	.040	130.424	***	i17_1
**BPNSF14**	5.464	.040	135.863	***	i18_1
**BPNSF9**	5.679	.040	140.701	***	i19_1
**BPNSF3**	5.610	.040	139.703	***	i20_1
**BPNSF23**	2.241	.046	49.111	***	i21_1
**BPNSF17**	2.577	.047	54.540	***	i22_1
**BPNSF11**	2.744	.047	58.985	***	i23_1
**BPNSF6**	2.565	.047	54.736	***	i24_1

Covariances: (g3 - Structural covariances)

			Estimate	S.E.	C.R.	P	Label
F1	<-->	F2	-.156	.024	-6.584	***	ccc1_1
F2	<-->	F3	-.149	.022	-6.643	***	ccc2_1
F1	<-->	F3	.728	.048	15.066	***	ccc3_1
F2	<-->	F4	.314	.041	7.635	***	ccc4_1
F3	<-->	F4	-.596	.045	-13.381	***	ccc5_1
F1	<-->	F4	-.490	.043	-11.286	***	ccc6_1
F2	<-->	F5	-.143	.023	-6.324	***	ccc7_1
F4	<-->	F5	-.668	.049	-13.722	***	ccc8_1
F3	<-->	F5	.770	.049	15.782	***	ccc9_1
F1	<-->	F5	.903	.056	16.114	***	ccc10_1
F6	<-->	F5	-.958	.060	-15.868	***	ccc11_1
F6	<-->	F3	-.641	.050	-12.931	***	ccc12_1
F6	<-->	F4	1.266	.073	17.426	***	ccc13_1
F6	<-->	F2	.372	.048	7.715	***	ccc14_1
F6	<-->	F1	-.622	.052	-12.029	***	ccc15_1

Correlations: (g3 - Structural covariances)

			Estimate
F1	<-->	F2	-.433
F2	<-->	F3	-.411
F1	<-->	F3	.836
F2	<-->	F4	.758
F3	<-->	F4	-.595
F1	<-->	F4	-.494
F2	<-->	F5	-.363
F4	<-->	F5	-.615
F3	<-->	F5	.808
F1	<-->	F5	.956

			Estimate
F6	<-->	F5	-.723
F6	<-->	F3	-.525
F6	<-->	F4	.910
F6	<-->	F2	.737
F6	<-->	F1	-.514

Variances: (g3 - Structural covariances)

	Estimate	S.E.	C.R.	P	Label
**F1**	.863	.072	12.009	***	vvv1_1
**F2**	.150	.036	4.231	***	vvv2_1
**F3**	.879	.065	13.450	***	vvv3_1
**F4**	1.141	.085	13.446	***	vvv4_1
**F5**	1.033	.073	14.100	***	vvv5_1
**F6**	1.696	.103	16.468	***	vvv6_1
**e1**	1.153	.088	13.173	***	v1_3
**e2**	1.020	.082	12.402	***	v2_3
**e3**	1.452	.105	13.828	***	v3_3
**e4**	1.961	.138	14.183	***	v4_3
**e5**	2.018	.140	14.413	***	v5_3
**e6**	2.056	.156	13.152	***	v6_3
**e7**	1.123	.111	10.087	***	v7_3
**e8**	1.663	.132	12.625	***	v8_3
**e9**	1.050	.081	12.953	***	v9_3
**e10**	.778	.067	11.670	***	v10_3
**e11**	.643	.062	10.413	***	v11_3
**e12**	1.168	.086	13.558	***	v12_3
**e13**	1.343	.103	13.085	***	v13_3
**e14**	1.358	.109	12.421	***	v14_3
**e15**	1.295	.105	12.279	***	v15_3
**e16**	1.731	.132	13.134	***	v16_3
**e17**	.966	.074	13.085	***	v17_3
**e18**	1.005	.078	12.842	***	v18_3
**e19**	.943	.073	12.909	***	v19_3
**e20**	1.400	.101	13.840	***	v20_3
**e21**	1.144	.093	12.271	***	v21_3
**e22**	1.368	.107	12.768	***	v22_3
**e23**	1.125	.091	12.419	***	v23_3
**e24**	1.720	.127	13.497	***	v24_3

Matrices (g3 - Structural covariances)

Residual Covariances (g3 - Structural covariances)

	BPNSF6	BPNSF11	BPNSF17	BPNSF23	BPNSF3	BPNSF9	BPNSF14	BPNSF21	BPNSF2	BPNSF8	BPNSF20	BPNSF22	BPNSF4	BPNSF12	BPNSF16	BPNS[illegible]
**BPNSF6**	.030															
**BPNSF11**	.245	.226														
**BPNSF17**	.131	.179	.221													
**BPNSF23**	.072	.149	.247	.185												
**BPNSF3**	-.141	-.077	-.201	-.229	-.046											
**BPNSF9**	-.219	-.334	-.407	-.178	.181	.111										
**BPNSF14**	.142	.003	-.145	-.031	-.043	.183	-.042									
**BPNSF21**	.174	-.007	.257	-.008	-.148	-.135	-.137	-.095								
**BPNSF2**	-.050	.120	.034	.233	-.279	.016	.081	.256	.067							
**BPNSF8**	.123	.295	.158	.088	-.084	-.210	.204	.142	.219	.222						
**BPNSF20**	-.021	.101	.418	.447	-.231	-.238	.049	.067	.116	.149	.165					
**BPNSF22**	-.177	-.007	-.114	.150	-.094	.053	.223	.031	-.050	.165	.092	-.017				
**BPNSF4**	-.084	-.065	.029	-.042	.463	.008	-.152	-.018	.158	.008	.008	-.088	-.081			
**BPNSF12**	.059	-.070	-.129	-.175	-.015	.183	.172	.058	.077	.146	.035	-.098	-.038	.130		
**BPNSF16**	.100	-.104	-.165	-.052	-.140	-.003	.087	.067	.123	.143	-.083	-.065	-.054	.111	.025	
**BPNSF24**	-.089	-.245	-.028	-.221	-.097	-.014	-.019	.213	-.030	-.090	-.094	.003	-.065	-.012	-.061	-.0[illegible]
**BPNSF5**	.246	.330	.103	.119	-.071	-.156	-.015	.025	.560	.383	.090	.218	-.140	-.104	-.142	-.1[illegible]
**BPNSF10**	.289	.582	.319	.206	-.286	-.448	-.105	-.083	.278	.443	.113	.143	-.135	-.013	-.028	-.0[illegible]
**BPNSF15**	-.089	.005	.078	-.273	-.030	-.059	.030	.132	-.066	.151	-.152	-.036	-.130	.177	.081	.0[illegible]
**BPNSF18**	-.182	-.057	.047	-.268	.026	.305	.412	.227	-.142	-.131	-.134	.049	-.016	.361	.276	.4[illegible]
**BPNSF1**	.033	.000	.060	.086	.348	-.037	-.245	-.098	.242	.012	.020	-.213	.299	-.015	-.092	-.0[illegible]
**BPNSF7**	.295	-.055	.046	.032	-.145	-.055	-.051	.059	.197	.244	.144	.003	-.056	.061	-.035	-.1[illegible]
**BPNSF13**	.120	-.037	.001	.161	-.300	-.089	-.062	-.145	.183	.163	.100	-.097	.015	.289	.084	.0[illegible]
**BPNSF19**	.011	-.094	.058	-.079	-.158	-.081	-.102	.220	.231	.078	.084	.032	.020	-.062	.027	-.0[illegible]

Residual Means (g3 - Structural covariances)

	BPNSF6	BPNSF11	BPNSF17	BPNSF23	BPNSF3	BPNSF9	BPNSF14	BPNSF21	BPNSF2	BPNSF8	BPNSF20	BPNSF22	BPNSF4	BPNSF12	BPNSF16	BPNSF24	BPN
	.067	.061	.251	.262	-.119	-.218	-.281	-.201	.261	.110	.254	.154	.090	-.110	-.096	-.106	.

Standardized Residual Covariances (g3 - Structural covariances)

	BPNSF6	BPNSF11	BPNSF17	BPNSF23	BPNSF3	BPNSF9	BPNSF14	BPNSF21	BPNSF2	BPNSF8	BPNSF20	BPNSF22	BPNSF4	BPNSF12	BPNSF16	BPNS
**BPNSF6**	.148															
**BPNSF11**	1.607	1.236														
**BPNSF17**	.820	1.146	1.086													
**BPNSF23**	.462	.965	1.529	.956												
**BPNSF3**	-1.097	-.624	-1.550	-1.800	-.305											
**BPNSF9**	-1.734	-2.731	-3.171	-1.410	1.610	.788										
**BPNSF14**	1.077	.021	-1.082	-.239	-.370	1.549	-.275									
**BPNSF21**	1.397	-.060	2.040	-.067	-1.342	-1.220	-1.181	-.693								
**BPNSF2**	-.317	.774	.209	1.456	-2.128	.127	.600	2.021	.310							
**BPNSF8**	.786	1.936	.990	.559	-.661	-1.676	1.563	1.150	1.329	1.109						
**BPNSF20**	-.136	.659	2.604	2.822	-1.802	-1.888	.370	.543	.701	.909	.807					
**BPNSF22**	-1.252	-.052	-.788	1.056	-.805	.461	1.873	.275	-.335	1.128	.626	-.098				
**BPNSF4**	-.737	-.598	.249	-.376	4.624	.079	-1.477	-.191	1.333	.066	.065	-.835	-.662			
**BPNSF12**	.489	-.603	-1.052	-1.464	-.140	1.697	1.530	.551	.606	1.186	.283	-.870	-.381	.974		
**BPNSF16**	.844	-.910	-1.378	-.444	-1.329	-.027	.791	.651	.994	1.190	-.685	-.588	-.555	1.012	.190	
**BPNSF24**	-.751	-2.157	-.237	-1.888	-.929	-.131	-.172	2.091	-.242	-.753	-.781	.026	-.675	-.111	-.582	-.4
**BPNSF5**	1.737	2.426	.721	.846	-.608	-1.362	-.130	.219	3.825	2.685	.626	1.681	-1.312	-.925	-1.289	-1.3
**BPNSF10**	2.035	4.235	2.210	1.454	-2.456	-3.961	-.887	-.741	1.885	3.071	.777	1.093	-1.274	-.120	-.251	-.4
**BPNSF15**	-.599	.032	.522	-1.860	-.238	-.487	.238	1.108	-.429	1.012	-1.007	-.266	-1.156	1.490	.695	.4
**BPNSF18**	-1.474	-.482	.381	-2.217	.247	2.986	3.867	2.259	-1.116	-1.063	-1.075	.430	-.166	3.620	2.809	4.3
**BPNSF1**	.247	.000	.456	.660	3.000	-.327	-2.055	-.878	1.776	.094	.152	-1.765	2.875	-.139	-.844	-.7
**BPNSF7**	2.413	-.474	.373	.265	-1.339	-.516	-.455	.558	1.564	2.003	1.172	.023	-.581	.585	-.348	-1.0
**BPNSF13**	.960	-.311	.007	1.301	-2.645	-.781	-.524	-1.301	1.422	1.304	.794	-.847	.153	2.622	.784	.0
**BPNSF19**	.088	-.812	.475	-.662	-1.458	-.745	-.901	2.074	1.857	.645	.687	.292	.209	-.594	.267	-.0

Standardized Residual Means (g3 - Structural covariances)

	BPNSF6	BPNSF11	BPNSF17	BPNSF23	BPNSF3	BPNSF9	BPNSF14	BPNSF21	BPNSF2	BPNSF8	BPNSF20	BPNSF22	BPNSF4	BPNSF12	BPNSF16	BPNSF24	BPN
	.807	.776	3.013	3.229	-1.662	-3.152	-3.893	-2.953	3.041	1.332	3.040	2.030	1.393	-1.636	-1.450	-1.589	2.

Notes for Group/Model (g3 - Structural covariances)

The following covariance matrix is not positive definite (g3 - Structural covariances)

	F5	F4	F3	F2	F1	F6
**F5**	1.033					
**F4**	-.668	1.141				
**F3**	.770	-.596	.879			
**F2**	-.143	.314	-.149	.150		
**F1**	.903	-.490	.728	-.156	.863	
**F6**	-.958	1.266	-.641	.372	-.622	1.696

This solution is not admissible.

Modification Indices (g3 - Structural covariances)

Covariances: (g3 - Structural covariances)

			M.I.	Par Change
e24	<-->	F4	5.469	-.112
e23	<-->	F2	13.624	.072
e23	<-->	e24	4.404	.157
e21	<-->	F4	8.313	.117
e21	<-->	F2	10.542	-.064
e20	<-->	F4	5.465	-.101
e20	<-->	F1	5.073	-.091
e20	<-->	e23	4.095	.136
e19	<-->	F1	4.796	-.074
e19	<-->	F6	4.512	-.087
e19	<-->	e22	8.695	-.182
e19	<-->	e21	4.227	.118
e19	<-->	e20	9.633	.187
e18	<-->	e22	6.614	-.164

			M.I.	Par Change
e18	<-->	e19	11.629	.179
e17	<-->	F5	13.245	-.107
e17	<-->	F1	11.223	.114
e17	<-->	e22	13.810	.231
e17	<-->	e19	11.248	-.171
e16	<-->	F2	5.285	.054
e16	<-->	F1	5.520	.110
e16	<-->	e20	10.978	-.269
e15	<-->	F2	9.875	.066
e15	<-->	e21	8.917	-.206
e15	<-->	e19	5.605	-.145
e14	<-->	F2	8.615	-.063
e14	<-->	F6	6.168	.124
e14	<-->	e23	7.366	-.188
e14	<-->	e22	13.388	.276
e14	<-->	e21	17.135	.291
e13	<-->	F5	11.051	.125
e13	<-->	F1	11.424	-.139
e13	<-->	e22	4.933	-.163
e13	<-->	e21	5.009	.153
e13	<-->	e19	12.151	.212
e13	<-->	e18	7.899	.177
e12	<-->	F2	6.145	-.048
e12	<-->	e22	4.087	.136
e12	<-->	e20	70.293	.551
e12	<-->	e18	12.705	-.205
e11	<-->	e18	4.757	.105
e10	<-->	e22	4.998	-.132
e10	<-->	e20	9.223	-.174
e9	<-->	e22	5.035	.147
e9	<-->	e17	17.651	.228
e8	<-->	F5	4.168	.086
e8	<-->	F4	5.722	.117
e8	<-->	e16	18.815	.399
e7	<-->	F6	6.142	.122
e7	<-->	e23	17.388	.285
e7	<-->	e19	8.042	-.174
e7	<-->	e15	4.381	.154
e6	<-->	e21	7.293	-.228
e6	<-->	e12	4.340	-.170
e5	<-->	F3	6.666	.126
e5	<-->	e22	4.855	.190
e5	<-->	e12	5.499	-.182
e5	<-->	e9	9.946	.236
e5	<-->	e8	16.205	.383
e5	<-->	e7	5.516	-.201
e5	<-->	e6	4.261	.214
e4	<-->	F2	21.607	-.113
e4	<-->	F6	7.172	.154
e4	<-->	e20	33.383	.481
e4	<-->	e18	7.364	-.198
e4	<-->	e16	5.724	.227
e4	<-->	e13	6.696	-.217
e4	<-->	e12	19.593	.340
e4	<-->	e6	4.869	-.228
e4	<-->	e5	26.359	-.504
e3	<-->	e24	7.070	.216
e3	<-->	e17	4.188	.126
e3	<-->	e7	9.001	-.222
e2	<-->	F5	9.281	-.097
e2	<-->	F3	14.676	.139
e2	<-->	e20	15.012	-.245
e2	<-->	e13	6.814	-.167
e2	<-->	e11	22.335	.231
e1	<-->	e17	33.840	.325
e1	<-->	e11	8.216	-.146

Variances: (g3 - Structural covariances)

	M.I.	Par Change

Regression Weights: (g3 - Structural covariances)

			M.I.	Par Change
BPNSF6	<---	BPNSF2	4.418	-.046
BPNSF6	<---	BPNSF20	5.390	-.050
BPNSF6	<---	BPNSF22	4.626	-.040
BPNSF11	<---	F2	4.763	.349
BPNSF11	<---	BPNSF17	4.659	-.038
BPNSF11	<---	BPNSF23	4.917	-.044
BPNSF11	<---	BPNSF9	6.553	-.024
BPNSF11	<---	BPNSF14	4.738	-.021
BPNSF11	<---	BPNSF21	6.756	-.027
BPNSF11	<---	BPNSF20	7.306	-.049
BPNSF11	<---	BPNSF22	4.790	-.035
BPNSF11	<---	BPNSF4	5.633	-.025
BPNSF11	<---	BPNSF12	5.113	-.023
BPNSF11	<---	BPNSF16	6.138	-.026
BPNSF11	<---	BPNSF24	7.414	-.028
BPNSF11	<---	BPNSF18	4.069	-.024
BPNSF11	<---	BPNSF1	4.609	-.024
BPNSF11	<---	BPNSF7	7.226	-.029
BPNSF11	<---	BPNSF13	6.718	-.027
BPNSF11	<---	BPNSF19	6.284	-.026
BPNSF17	<---	BPNSF20	7.447	.054
BPNSF17	<---	BPNSF18	4.371	.028
BPNSF23	<---	BPNSF2	5.760	.045
BPNSF23	<---	BPNSF20	11.157	.061
BPNSF23	<---	BPNSF22	6.704	.042
BPNSF23	<---	BPNSF1	4.232	.023
BPNSF3	<---	BPNSF4	5.425	.026
BPNSF3	<---	BPNSF1	4.896	.026
BPNSF9	<---	F4	9.301	-.151
BPNSF9	<---	F2	8.608	-.422
BPNSF9	<---	F6	13.603	-.148
BPNSF9	<---	BPNSF6	5.340	-.037
BPNSF9	<---	BPNSF11	5.279	-.036
BPNSF9	<---	BPNSF17	8.761	-.047
BPNSF9	<---	BPNSF8	5.597	-.036
BPNSF9	<---	BPNSF10	4.517	-.030
BPNSF14	<---	BPNSF17	4.201	-.034
BPNSF14	<---	BPNSF4	5.649	-.023
BPNSF14	<---	BPNSF1	5.735	-.025
BPNSF21	<---	F6	6.710	.105
BPNSF21	<---	BPNSF17	4.095	.033
BPNSF2	<---	BPNSF9	5.259	.026
BPNSF2	<---	BPNSF21	5.374	.029
BPNSF2	<---	BPNSF4	5.730	.030
BPNSF2	<---	BPNSF12	4.198	.025
BPNSF2	<---	BPNSF16	4.886	.028
BPNSF2	<---	BPNSF5	10.034	.052
BPNSF2	<---	BPNSF1	7.037	.036
BPNSF2	<---	BPNSF7	4.525	.028
BPNSF2	<---	BPNSF13	4.667	.027
BPNSF2	<---	BPNSF19	5.529	.029
BPNSF20	<---	BPNSF17	10.519	.063
BPNSF20	<---	BPNSF23	11.557	.074
BPNSF4	<---	BPNSF6	5.177	.040
BPNSF4	<---	BPNSF11	9.877	.053
BPNSF4	<---	BPNSF17	14.166	.066
BPNSF4	<---	BPNSF23	11.378	.065
BPNSF4	<---	BPNSF3	24.161	.046
BPNSF4	<---	BPNSF9	10.030	.029
BPNSF4	<---	BPNSF14	6.839	.025
BPNSF4	<---	BPNSF21	9.550	.031
BPNSF4	<---	BPNSF2	14.180	.068
BPNSF4	<---	BPNSF8	8.256	.047
BPNSF4	<---	BPNSF20	10.925	.059
BPNSF4	<---	BPNSF22	8.901	.046
BPNSF4	<---	BPNSF12	8.838	.030
BPNSF4	<---	BPNSF16	9.299	.031
BPNSF4	<---	BPNSF24	9.911	.032
BPNSF4	<---	BPNSF5	9.623	.041
BPNSF4	<---	BPNSF10	6.990	.040

			M.I.	Par Change
BPNSF4	<---	BPNSF15	5.349	.031
BPNSF4	<---	BPNSF18	6.379	.030
BPNSF4	<---	BPNSF1	20.265	.049
BPNSF4	<---	BPNSF7	9.737	.033
BPNSF4	<---	BPNSF13	9.332	.031
BPNSF4	<---	BPNSF19	11.371	.034
BPNSF12	<---	F5	5.299	.107
BPNSF12	<---	F1	4.729	.112
BPNSF5	<---	F4	4.524	.141
BPNSF5	<---	BPNSF6	5.597	.051
BPNSF5	<---	BPNSF11	4.587	.045
BPNSF5	<---	BPNSF2	16.071	.090
BPNSF5	<---	BPNSF8	6.254	.051
BPNSF5	<---	BPNSF22	5.198	.043
BPNSF5	<---	BPNSF18	8.947	.044
BPNSF10	<---	F5	6.825	-.161
BPNSF10	<---	F6	6.997	.128
BPNSF10	<---	BPNSF3	12.331	-.036
BPNSF10	<---	BPNSF9	14.992	-.040
BPNSF10	<---	BPNSF14	11.119	-.035
BPNSF10	<---	BPNSF21	10.735	-.036
BPNSF10	<---	BPNSF20	6.005	-.048
BPNSF10	<---	BPNSF22	6.761	-.044
BPNSF10	<---	BPNSF4	10.696	-.037
BPNSF10	<---	BPNSF12	9.084	-.033
BPNSF10	<---	BPNSF16	8.773	-.033
BPNSF10	<---	BPNSF24	8.659	-.033
BPNSF10	<---	BPNSF5	9.609	-.045
BPNSF10	<---	BPNSF15	7.071	-.039
BPNSF10	<---	BPNSF18	12.815	-.047
BPNSF10	<---	BPNSF1	12.006	-.042
BPNSF10	<---	BPNSF7	15.064	-.046
BPNSF10	<---	BPNSF13	8.461	-.033
BPNSF10	<---	BPNSF19	10.586	-.036
BPNSF15	<---	F4	4.200	-.148
BPNSF15	<---	F2	4.657	-.448
BPNSF15	<---	F6	4.536	-.125
BPNSF15	<---	BPNSF23	5.942	-.063
BPNSF18	<---	F5	11.637	.243
BPNSF18	<---	F3	13.661	.292
BPNSF18	<---	F1	10.147	.252
BPNSF18	<---	BPNSF9	7.069	.031
BPNSF18	<---	BPNSF14	7.594	.034
BPNSF18	<---	BPNSF21	5.452	.030
BPNSF18	<---	BPNSF12	7.486	.035
BPNSF18	<---	BPNSF16	6.487	.033
BPNSF18	<---	BPNSF24	8.786	.038
BPNSF18	<---	BPNSF5	8.533	.049
BPNSF18	<---	BPNSF15	5.752	.040
BPNSF18	<---	BPNSF7	7.060	.036
BPNSF18	<---	BPNSF13	5.831	.032
BPNSF18	<---	BPNSF19	5.664	.031
BPNSF1	<---	F2	4.454	-.420
BPNSF13	<---	BPNSF3	11.336	-.030
BPNSF13	<---	BPNSF9	7.935	-.025
BPNSF13	<---	BPNSF14	6.417	-.023
BPNSF13	<---	BPNSF21	8.226	-.028
BPNSF13	<---	BPNSF22	8.590	-.044
BPNSF13	<---	BPNSF4	6.660	-.025
BPNSF13	<---	BPNSF16	4.692	-.021
BPNSF13	<---	BPNSF24	5.457	-.023
BPNSF13	<---	BPNSF5	6.084	-.032
BPNSF13	<---	BPNSF15	4.777	-.028
BPNSF13	<---	BPNSF18	5.466	-.027
BPNSF13	<---	BPNSF1	7.406	-.029
BPNSF13	<---	BPNSF7	4.300	-.021
BPNSF13	<---	BPNSF19	6.583	-.025
BPNSF19	<---	BPNSF3	4.184	-.019
BPNSF19	<---	BPNSF12	4.826	-.022

Means: (g3 - Structural covariances)

	M.I.	Par Change

Intercepts: (g3 - Structural covariances)

	M.I.	Par Change
**BPNSF11**	5.312	-.127
**BPNSF4**	12.218	.189
**BPNSF10**	9.436	-.184
**BPNSF13**	6.623	-.134

Bootstrap (g3 - Structural covariances)

Bootstrap standard errors (g3 - Structural covariances)

Scalar Estimates (g3 - Structural covariances)

Regression Weights: (g3 - Structural covariances)

Parameter	SE	SE-SE	Mean	Bias	SE-Bias
BPNSF19 <--- F1	.000	.000	1.000	.000	.000
BPNSF13 <--- F1	.056	.003	1.132	-.005	.004
BPNSF7 <--- F1	.059	.003	.855	.000	.004
BPNSF1 <--- F1	.071	.004	.762	-.001	.005
BPNSF18 <--- F2	.000	.000	1.000	.000	.000
BPNSF15 <--- F2	.396	.020	2.525	.054	.028
BPNSF10 <--- F2	.522	.026	3.180	.074	.037
BPNSF5 <--- F2	.422	.021	2.629	.061	.030
BPNSF24 <--- F3	.000	.000	1.000	.000	.000
BPNSF16 <--- F3	.053	.003	1.129	-.001	.004
BPNSF12 <--- F3	.049	.002	1.218	-.006	.003
BPNSF4 <--- F3	.051	.003	.848	-.002	.004
BPNSF22 <--- F4	.000	.000	1.000	.000	.000
BPNSF20 <--- F4	.052	.003	1.195	-.001	.004
BPNSF8 <--- F4	.050	.002	1.195	-.005	.004
BPNSF2 <--- F4	.054	.003	1.120	-.001	.004
BPNSF21 <--- F5	.000	.000	1.000	.000	.000
BPNSF14 <--- F5	.045	.002	1.096	.002	.003
BPNSF9 <--- F5	.052	.003	1.041	.002	.004
BPNSF3 <--- F5	.058	.003	.885	.004	.004
BPNSF23 <--- F6	.000	.000	1.000	.000	.000
BPNSF17 <--- F6	.034	.002	.979	.003	.002
BPNSF11 <--- F6	.038	.002	.959	.000	.003
BPNSF6 <--- F6	.037	.002	.862	.000	.003

Standardized Regression Weights: (g3 - Structural covariances)

Parameter	SE	SE-SE	Mean	Bias	SE-Bias
BPNSF19 <--- F1	.030	.002	.657	.003	.002
BPNSF13 <--- F1	.023	.001	.721	-.002	.002
BPNSF7 <--- F1	.034	.002	.553	.002	.002
BPNSF1 <--- F1	.033	.002	.453	.001	.002
BPNSF18 <--- F2	.041	.002	.265	.002	.003
BPNSF15 <--- F2	.026	.001	.557	.001	.002
BPNSF10 <--- F2	.030	.001	.753	.002	.002
BPNSF5 <--- F2	.030	.001	.613	.002	.002
BPNSF24 <--- F3	.028	.001	.677	.002	.002
BPNSF16 <--- F3	.029	.001	.771	.003	.002
BPNSF12 <--- F3	.020	.001	.820	.000	.001
BPNSF4 <--- F3	.034	.002	.593	.000	.002
BPNSF22 <--- F4	.024	.001	.679	.002	.002
BPNSF20 <--- F4	.028	.001	.741	.003	.002
BPNSF8 <--- F4	.025	.001	.745	-.003	.002
BPNSF2 <--- F4	.029	.001	.674	.001	.002
BPNSF21 <--- F5	.030	.001	.723	.004	.002
BPNSF14 <--- F5	.029	.001	.746	.003	.002
BPNSF9 <--- F5	.027	.001	.739	.003	.002
BPNSF3 <--- F5	.041	.002	.607	.004	.003
BPNSF23 <--- F6	.022	.001	.774	.001	.002
BPNSF17 <--- F6	.026	.001	.738	.002	.002
BPNSF11 <--- F6	.025	.001	.761	-.001	.002

Parameter	SE	SE-SE	Mean	Bias	SE-Bias
BPNSF6 <--- F6	.030	.002	.649	-.002	.002

Intercepts: (g3 - Structural covariances)

Parameter	SE	SE-SE	Mean	Bias	SE-Bias
**BPNSF19**	.044	.002	5.167	.006	.003
**BPNSF13**	.040	.002	5.074	.006	.003
**BPNSF7**	.042	.002	4.842	.008	.003
**BPNSF1**	.039	.002	4.726	.007	.003
**BPNSF18**	.042	.002	4.322	.000	.003
**BPNSF15**	.046	.002	3.714	-.001	.003
**BPNSF10**	.049	.002	3.172	-.004	.003
**BPNSF5**	.045	.002	3.735	-.003	.003
**BPNSF24**	.040	.002	5.227	.004	.003
**BPNSF16**	.042	.002	5.121	.006	.003
**BPNSF12**	.037	.002	5.224	.006	.003
**BPNSF4**	.039	.002	5.118	.004	.003
**BPNSF22**	.045	.002	3.115	-.004	.003
**BPNSF20**	.052	.003	2.498	-.010	.004
**BPNSF8**	.052	.003	2.803	-.003	.004
**BPNSF2**	.046	.002	2.379	-.004	.003
**BPNSF21**	.039	.002	5.208	.008	.003
**BPNSF14**	.041	.002	5.473	.008	.003
**BPNSF9**	.044	.002	5.686	.006	.003
**BPNSF3**	.041	.002	5.617	.007	.003
**BPNSF23**	.044	.002	2.233	-.008	.003
**BPNSF17**	.052	.003	2.568	-.009	.004
**BPNSF11**	.048	.002	2.742	-.003	.003
**BPNSF6**	.051	.003	2.563	-.002	.004

Covariances: (g3 - Structural covariances)

Parameter	SE	SE-SE	Mean	Bias	SE-Bias
F1 <--> F2	.025	.001	-.157	-.001	.002
F2 <--> F3	.025	.001	-.150	.000	.002
F1 <--> F3	.047	.002	.730	.001	.003
F2 <--> F4	.051	.003	.314	.000	.004
F3 <--> F4	.044	.002	-.601	-.005	.003
F1 <--> F4	.043	.002	-.492	-.002	.003
F2 <--> F5	.024	.001	-.143	.000	.002
F4 <--> F5	.043	.002	-.666	.002	.003
F3 <--> F5	.052	.003	.770	.000	.004
F1 <--> F5	.075	.004	.906	.003	.005
F6 <--> F5	.058	.003	-.957	.001	.004
F6 <--> F3	.049	.002	-.645	-.005	.003
F6 <--> F4	.070	.003	1.264	-.002	.005
F6 <--> F2	.058	.003	.371	-.001	.004
F6 <--> F1	.055	.003	-.628	-.006	.004

Correlations: (g3 - Structural covariances)

Parameter	SE	SE-SE	Mean	Bias	SE-Bias
F1 <--> F2	.039	.002	-.434	-.002	.003
F2 <--> F3	.036	.002	-.410	.000	.003
F1 <--> F3	.026	.001	.833	-.003	.002
F2 <--> F4	.025	.001	.757	-.001	.002
F3 <--> F4	.027	.001	-.598	-.002	.002
F1 <--> F4	.033	.002	-.494	.000	.002
F2 <--> F5	.038	.002	-.363	.000	.003
F4 <--> F5	.028	.001	-.612	.002	.002
F3 <--> F5	.022	.001	.805	-.003	.002
F1 <--> F5	.023	.001	.954	-.002	.002
F6 <--> F5	.025	.001	-.723	.001	.002
F6 <--> F3	.031	.002	-.528	-.003	.002
F6 <--> F4	.018	.001	.909	-.002	.001
F6 <--> F2	.029	.001	.734	-.002	.002
F6 <--> F1	.033	.002	-.518	-.003	.002

Variances: (g3 - Structural covariances)

Parameter	SE	SE-SE	Mean	Bias	SE-Bias
**F1**	.081	.004	.871	.008	.006
**F2**	.047	.002	.154	.004	.003
**F3**	.065	.003	.885	.006	.005
**F4**	.080	.004	1.144	.003	.006
**F5**	.080	.004	1.037	.003	.006
**F6**	.102	.005	1.694	-.001	.007
**e1**	.129	.006	1.145	-.008	.009
**e2**	.109	.005	1.028	.008	.008
**e3**	.165	.008	1.444	-.007	.012
**e4**	.170	.009	1.948	-.012	.012
**e5**	.149	.007	2.000	-.019	.011
**e6**	.154	.008	2.051	-.004	.011
**e7**	.148	.007	1.115	-.008	.010
**e8**	.159	.008	1.659	-.004	.011
**e9**	.128	.006	1.051	.001	.009
**e10**	.111	.006	.768	-.010	.008
**e11**	.079	.004	.637	-.006	.006
**e12**	.142	.007	1.173	.004	.010
**e13**	.120	.006	1.335	-.008	.008
**e14**	.186	.009	1.340	-.019	.013
**e15**	.148	.007	1.307	.012	.010
**e16**	.210	.010	1.729	-.003	.015
**e17**	.129	.006	.951	-.016	.009
**e18**	.146	.007	.994	-.011	.010
**e19**	.123	.006	.932	-.011	.009
**e20**	.216	.011	1.394	-.006	.015
**e21**	.126	.006	1.138	-.006	.009
**e22**	.169	.008	1.358	-.010	.012
**e23**	.129	.006	1.132	.008	.009
**e24**	.208	.010	1.737	.017	.015

Matrices (g3 - Structural covariances)

Sample Covariances - Standard Errors (g3 - Structural covariances)

	BPNSF6	BPNSF11	BPNSF17	BPNSF23	BPNSF3	BPNSF9	BPNSF14	BPNSF21	BPNSF2	BPNSF8	BPNSF20	BPNSF22	BPNSF4	BPNSF12	BPNSF16	BPNS
**BPNSF6**	.174															
**BPNSF11**	.152	.149														
**BPNSF17**	.156	.158	.157													
**BPNSF23**	.148	.139	.145	.159												
**BPNSF3**	.126	.127	.121	.132	.192											
**BPNSF9**	.138	.124	.143	.135	.141	.177										
**BPNSF14**	.122	.118	.144	.129	.125	.136	.147									
**BPNSF21**	.111	.130	.132	.113	.123	.120	.119	.134								
**BPNSF2**	.166	.157	.165	.138	.132	.126	.130	.112	.180							
**BPNSF8**	.149	.146	.165	.145	.142	.145	.130	.129	.170	.163						
**BPNSF20**	.157	.154	.159	.156	.127	.141	.134	.133	.159	.160	.176					
**BPNSF22**	.142	.130	.152	.137	.133	.134	.135	.120	.131	.138	.140	.132				
**BPNSF4**	.114	.118	.113	.099	.135	.106	.103	.114	.122	.113	.116	.114	.127			
**BPNSF12**	.119	.124	.137	.125	.122	.131	.127	.103	.123	.137	.135	.122	.095	.138		
**BPNSF16**	.123	.117	.123	.119	.110	.130	.117	.112	.108	.134	.138	.117	.094	.114	.120	
**BPNSF24**	.112	.108	.121	.111	.120	.113	.111	.106	.124	.123	.120	.117	.104	.104	.095	[illegible]
**BPNSF5**	.141	.131	.140	.129	.130	.112	.127	.118	.161	.151	.154	.140	.112	.123	.113	[illegible]
**BPNSF10**	.160	.140	.151	.132	.124	.132	.116	.113	.148	.149	.139	.134	.111	.108	.117	[illegible]
**BPNSF15**	.166	.134	.158	.133	.126	.120	.130	.131	.130	.151	.157	.139	.112	.120	.115	[illegible]
**BPNSF18**	.129	.121	.138	.115	.109	.110	.121	.103	.139	.144	.131	.121	.102	.117	.110	[illegible]
**BPNSF1**	.145	.127	.148	.126	.141	.125	.133	.128	.140	.150	.143	.129	.117	.123	.125	[illegible]
**BPNSF7**	.129	.123	.135	.113	.134	.123	.121	.107	.138	.153	.122	.137	.114	.111	.117	[illegible]
**BPNSF13**	.132	.114	.130	.118	.124	.118	.123	.101	.125	.149	.135	.127	.097	.117	.111	[illegible]
**BPNSF19**	.108	.103	.128	.100	.098	.119	.118	.107	.116	.133	.125	.104	.095	.108	.102	[illegible]

Sample Correlations - Standard Errors (g3 - Structural covariances)

	BPNSF6	BPNSF11	BPNSF17	BPNSF23	BPNSF3	BPNSF9	BPNSF14	BPNSF21	BPNSF2	BPNSF8	BPNSF20	BPNSF22	BPNSF4	BPNSF12	BPNSF16	BPNS
**BPNSF6**	.000															
**BPNSF11**	.046	.000														
**BPNSF17**	.047	.045	.000													
**BPNSF23**	.045	.035	.039	.000												
**BPNSF3**	.052	.053	.048	.053	.000											
**BPNSF9**	.050	.042	.044	.045	.059	.000										
**BPNSF14**	.047	.047	.050	.046	.052	.041	.000									

	BPNSF6	BPNSF11	BPNSF17	BPNSF23	BPNSF3	BPNSF9	BPNSF14	BPNSF21	BPNSF2	BPNSF8	BPNSF20	BPNSF22	BPNSF4	BPNSF12	BPNSF16	BPNS[illegible]
**BPNSF21**	.048	.051	.052	.044	.056	.053	.055	.000								
**BPNSF2**	.052	.048	.049	.043	.051	.049	.050	.049	.000							
**BPNSF8**	.045	.039	.046	.040	.053	.049	.051	.052	.048	.000						
**BPNSF20**	.048	.045	.038	.038	.049	.048	.048	.054	.045	.045	.000					
**BPNSF22**	.051	.042	.051	.041	.054	.058	.058	.054	.041	.040	.047	.000				
**BPNSF4**	.050	.053	.048	.043	.054	.053	.052	.059	.055	.050	.050	.056	.000			
**BPNSF12**	.046	.048	.050	.049	.054	.041	.048	.043	.048	.054	.051	.049	.049	.000		
**BPNSF16**	.052	.048	.048	.050	.056	.054	.048	.051	.044	.055	.052	.052	.053	.038	.000	
**BPNSF24**	.045	.044	.050	.044	.058	.051	.051	.046	.051	.050	.049	.054	.058	.043	.047	.0[illegible]
**BPNSF5**	.044	.039	.043	.040	.053	.043	.050	.051	.043	.043	.047	.047	.050	.048	.047	.0[illegible]
**BPNSF10**	.054	.038	.044	.041	.054	.049	.048	.050	.048	.044	.045	.049	.052	.046	.051	.0[illegible]
**BPNSF15**	.054	.043	.050	.045	.051	.047	.051	.057	.043	.047	.049	.049	.051	.050	.050	.0[illegible]
**BPNSF18**	.051	.048	.050	.045	.050	.050	.055	.051	.053	.055	.049	.050	.052	.055	.054	.0[illegible]
**BPNSF1**	.054	.049	.053	.047	.051	.052	.057	.060	.050	.054	.052	.051	.053	.053	.058	.0[illegible]
**BPNSF7**	.052	.051	.052	.045	.065	.056	.056	.049	.054	.060	.049	.061	.058	.051	.058	.0[illegible]
**BPNSF13**	.052	.048	.049	.050	.058	.046	.052	.048	.050	.061	.054	.054	.048	.037	.047	.0[illegible]
**BPNSF19**	.046	.041	.051	.042	.049	.050	.056	.038	.047	.052	.052	.048	.049	.050	.046	.0[illegible]

Sample Means - Standard Errors (g3 - Structural covariances)

	BPNSF6	BPNSF11	BPNSF17	BPNSF23	BPNSF3	BPNSF9	BPNSF14	BPNSF21	BPNSF2	BPNSF8	BPNSF20	BPNSF22	BPNSF4	BPNSF12	BPNSF16	BPNSF[illegible]
**BPNSF6**	.080	.077	.086	.074	.073	.078	.070	.063	.088	.081	.083	.074	.063	.070	.071	.07[illegible]

Bootstrap Confidence (g3 - Structural covariances)

Percentile method (g3 - Structural covariances)

90% confidence intervals (percentile method)

Scalar Estimates (g3 - Structural covariances)

Regression Weights: (g3 - Structural covariances)

Parameter			Estimate	Lower	Upper	P
BPNSF19	<---	F1	1.000	1.000	1.000	...
BPNSF13	<---	F1	1.137	1.035	1.224	.010
BPNSF7	<---	F1	.855	.759	.953	.010
BPNSF1	<---	F1	.763	.647	.886	.010
BPNSF18	<---	F2	1.000	1.000	1.000	...
BPNSF15	<---	F2	2.472	2.005	3.283	.010
BPNSF10	<---	F2	3.106	2.481	4.237	.010
BPNSF5	<---	F2	2.568	2.058	3.451	.010
BPNSF24	<---	F3	1.000	1.000	1.000	...
BPNSF16	<---	F3	1.130	1.045	1.218	.010
BPNSF12	<---	F3	1.224	1.141	1.298	.010
BPNSF4	<---	F3	.850	.757	.938	.010
BPNSF22	<---	F4	1.000	1.000	1.000	...
BPNSF20	<---	F4	1.196	1.117	1.287	.010
BPNSF8	<---	F4	1.200	1.118	1.276	.010
BPNSF2	<---	F4	1.121	1.040	1.220	.010
BPNSF21	<---	F5	1.000	1.000	1.000	...
BPNSF14	<---	F5	1.094	1.025	1.171	.010
BPNSF9	<---	F5	1.039	.965	1.132	.010
BPNSF3	<---	F5	.881	.793	.984	.010
BPNSF23	<---	F6	1.000	1.000	1.000	...
BPNSF17	<---	F6	.976	.924	1.040	.010
BPNSF11	<---	F6	.959	.898	1.018	.010
BPNSF6	<---	F6	.863	.803	.929	.010

Standardized Regression Weights: (g3 - Structural covariances)

Parameter			Estimate	Lower	Upper	P
BPNSF19	<---	F1	.654	.601	.710	.010
BPNSF13	<---	F1	.723	.681	.757	.010
BPNSF7	<---	F1	.551	.494	.608	.010
BPNSF1	<---	F1	.452	.396	.505	.010
BPNSF18	<---	F2	.263	.198	.327	.010
BPNSF15	<---	F2	.556	.511	.599	.010
BPNSF10	<---	F2	.751	.704	.803	.010

Parameter			Estimate	Lower	Upper	P
BPNSF5	<---	F2	.611	.566	.664	.010
BPNSF24	<---	F3	.675	.634	.731	.010
BPNSF16	<---	F3	.769	.725	.818	.010
BPNSF12	<---	F3	.820	.789	.852	.010
BPNSF4	<---	F3	.593	.538	.652	.010
BPNSF22	<---	F4	.678	.636	.720	.010
BPNSF20	<---	F4	.739	.695	.786	.010
BPNSF8	<---	F4	.748	.701	.784	.010
BPNSF2	<---	F4	.673	.626	.721	.010
BPNSF21	<---	F5	.719	.674	.768	.010
BPNSF14	<---	F5	.743	.691	.792	.010
BPNSF9	<---	F5	.736	.698	.784	.010
BPNSF3	<---	F5	.604	.537	.673	.010
BPNSF23	<---	F6	.773	.742	.812	.010
BPNSF17	<---	F6	.736	.696	.783	.010
BPNSF11	<---	F6	.762	.721	.802	.010
BPNSF6	<---	F6	.651	.598	.699	.010

Intercepts: (g3 - Structural covariances)

Parameter	Estimate	Lower	Upper	P
**BPNSF19**	5.161	5.091	5.232	.010
**BPNSF13**	5.068	5.007	5.137	.010
**BPNSF7**	4.834	4.769	4.910	.010
**BPNSF1**	4.719	4.664	4.798	.010
**BPNSF18**	4.323	4.258	4.394	.010
**BPNSF15**	3.715	3.630	3.788	.010
**BPNSF10**	3.176	3.091	3.248	.010
**BPNSF5**	3.738	3.659	3.813	.010
**BPNSF24**	5.223	5.159	5.293	.010
**BPNSF16**	5.115	5.050	5.196	.010
**BPNSF12**	5.218	5.165	5.289	.010
**BPNSF4**	5.114	5.059	5.184	.010
**BPNSF22**	3.120	3.039	3.185	.010
**BPNSF20**	2.508	2.405	2.582	.010
**BPNSF8**	2.806	2.712	2.889	.010
**BPNSF2**	2.383	2.304	2.456	.010
**BPNSF21**	5.200	5.131	5.272	.010
**BPNSF14**	5.464	5.408	5.547	.010
**BPNSF9**	5.679	5.606	5.758	.010
**BPNSF3**	5.610	5.551	5.699	.010
**BPNSF23**	2.241	2.158	2.305	.010
**BPNSF17**	2.577	2.481	2.646	.010
**BPNSF11**	2.744	2.665	2.823	.010
**BPNSF6**	2.565	2.477	2.642	.010

Covariances: (g3 - Structural covariances)

Parameter			Estimate	Lower	Upper	P
F1	<-->	F2	-.156	-.195	-.115	.010
F2	<-->	F3	-.149	-.191	-.110	.010
F1	<-->	F3	.728	.655	.809	.010
F2	<-->	F4	.314	.228	.402	.010
F3	<-->	F4	-.596	-.672	-.526	.010
F1	<-->	F4	-.490	-.566	-.422	.010
F2	<-->	F5	-.143	-.184	-.104	.010
F4	<-->	F5	-.668	-.738	-.597	.010
F3	<-->	F5	.770	.687	.857	.010
F1	<-->	F5	.903	.796	1.035	.010
F6	<-->	F5	-.958	-1.064	-.863	.010
F6	<-->	F3	-.641	-.716	-.564	.010
F6	<-->	F4	1.266	1.153	1.379	.010
F6	<-->	F2	.372	.277	.465	.010
F6	<-->	F1	-.622	-.721	-.541	.010

Correlations: (g3 - Structural covariances)

Parameter			Estimate	Lower	Upper	P
F1	<-->	F2	-.433	-.494	-.364	.010
F2	<-->	F3	-.411	-.467	-.354	.010

Parameter			Estimate	Lower	Upper	P
F1	<-->	F3	.836	.790	.876	.010
F2	<-->	F4	.758	.719	.796	.010
F3	<-->	F4	-.595	-.639	-.553	.010
F1	<-->	F4	-.494	-.545	-.436	.010
F2	<-->	F5	-.363	-.415	-.287	.010
F4	<-->	F5	-.615	-.656	-.564	.010
F3	<-->	F5	.808	.764	.840	.010
F1	<-->	F5	.956	.915	.993	.010
F6	<-->	F5	-.723	-.764	-.679	.010
F6	<-->	F3	-.525	-.574	-.476	.010
F6	<-->	F4	.910	.877	.937	.010
F6	<-->	F2	.737	.684	.779	.010
F6	<-->	F1	-.514	-.564	-.463	.010

Variances: (g3 - Structural covariances)

Parameter	Estimate	Lower	Upper	P
**F1**	.863	.734	.992	.010
**F2**	.150	.083	.234	.010
**F3**	.879	.772	.991	.010
**F4**	1.141	1.018	1.269	.010
**F5**	1.033	.920	1.173	.010
**F6**	1.696	1.532	1.865	.010
**e1**	1.153	.937	1.361	.010
**e2**	1.020	.848	1.209	.010
**e3**	1.452	1.188	1.741	.010
**e4**	1.961	1.682	2.229	.010
**e5**	2.018	1.767	2.251	.010
**e6**	2.056	1.793	2.302	.010
**e7**	1.123	.872	1.372	.010
**e8**	1.663	1.400	1.923	.010
**e9**	1.050	.827	1.256	.010
**e10**	.778	.578	.966	.010
**e11**	.643	.514	.768	.010
**e12**	1.168	.964	1.467	.010
**e13**	1.343	1.128	1.534	.010
**e14**	1.358	1.050	1.659	.010
**e15**	1.295	1.047	1.560	.010
**e16**	1.731	1.394	2.135	.010
**e17**	.966	.749	1.170	.010
**e18**	1.005	.774	1.249	.010
**e19**	.943	.726	1.143	.010
**e20**	1.400	1.057	1.771	.010
**e21**	1.144	.907	1.338	.010
**e22**	1.368	1.059	1.627	.010
**e23**	1.125	.921	1.340	.010
**e24**	1.720	1.411	2.090	.010

Matrices (g3 - Structural covariances)

Sample Covariances (g3 - Structural covariances)

Sample Covariances - Lower Bounds (PC) (g3 - Structural covariances)

	BPNSF6	BPNSF11	BPNSF17	BPNSF23	BPNSF3	BPNSF9	BPNSF14	BPNSF21	BPNSF2	BPNSF8	BPNSF20	BPNSF22	BPNSF4	BPNSF12	BPNSF16	BPNS[illegible]
**BPNSF6**	2.741															
**BPNSF11**	1.342	2.674														
**BPNSF17**	1.302	1.484	2.920													
**BPNSF23**	1.275	1.528	1.661	2.755												
**BPNSF3**	-1.083	-1.079	-1.205	-1.282	1.804											
**BPNSF9**	-1.293	-1.467	-1.607	-1.391	.893	1.845										
**BPNSF14**	-.980	-1.195	-1.410	-1.275	.753	1.117	1.969									
**BPNSF21**	-.839	-1.160	-.933	-1.145	.571	.751	.818	1.654								
**BPNSF2**	.894	1.227	1.122	1.401	-1.146	-.928	-.951	-.654	2.898							
**BPNSF8**	1.159	1.513	1.360	1.362	-1.027	-1.306	-.899	-.865	1.434	2.851						
**BPNSF20**	.993	1.299	1.625	1.706	-1.151	-1.333	-1.074	-.967	1.341	1.505	2.817					
**BPNSF22**	.635	.987	.861	1.198	-.906	-.860	-.732	-.816	1.025	1.283	1.224	2.221				
**BPNSF4**	-.745	-.794	-.705	-.753	.824	.507	.377	.458	-.612	-.789	-.810	-.773	1.524			
**BPNSF12**	-.815	-.997	-1.115	-1.163	.629	.912	.984	.836	-.924	-.947	-1.094	-1.015	.722	1.826		
**BPNSF16**	-.728	-1.013	-1.080	-.975	.430	.672	.836	.733	-.817	-.918	-1.156	-.940	.632	1.137	1.696	

	BPNSF6	BPNSF11	BPNSF17	BPNSF23	BPNSF3	BPNSF9	BPNSF14	BPNSF21	BPNSF2	BPNSF8	BPNSF20	BPNSF22	BPNSF4	BPNSF12	BPNSF16	BPNS[illegible]
**BPNSF24**	-.831	-1.018	-.843	-1.042	.391	.601	.675	.789	-.907	-1.015	-1.006	-.763	.519	.900	.763	[illegible]
**BPNSF5**	.830	1.039	.779	.864	-.603	-.720	-.633	-.517	1.190	1.098	.797	.771	-.639	-.784	-.736	[illegible]
**BPNSF10**	1.012	1.468	1.207	1.133	-.882	-1.125	-.782	-.721	1.116	1.360	1.047	.885	-.706	-.766	-.742	[illegible]
**BPNSF15**	.424	.676	.684	.406	-.548	-.608	-.554	-.428	.560	.819	.519	.523	-.627	-.439	-.509	[illegible]
**BPNSF18**	-.082	.079	.155	-.094	-.271	-.010	.080	-.052	-.049	.007	.017	.161	-.295	.009	-.078	[illegible]
**BPNSF1**	-.629	-.678	-.641	-.604	.708	.478	.301	.389	-.440	-.686	-.675	-.834	.583	.465	.322	[illegible]
**BPNSF7**	-.373	-.775	-.674	-.707	.311	.517	.612	.652	-.495	-.493	-.542	-.638	.291	.630	.475	[illegible]
**BPNSF13**	-.698	-.902	-.905	-.729	.405	.776	.858	.681	-.643	-.759	-.808	-.870	.538	1.081	.807	[illegible]
**BPNSF19**	-.721	-.859	-.762	-.880	.464	.669	.695	.945	-.523	-.721	-.740	-.637	.475	.612	.649	[illegible]

Sample Covariances - Upper Bounds (PC) (g3 - Structural covariances)

	BPNSF6	BPNSF11	BPNSF17	BPNSF23	BPNSF3	BPNSF9	BPNSF14	BPNSF21	BPNSF2	BPNSF8	BPNSF20	BPNSF22	BPNSF4	BPNSF12	BPNSF16	BPNS[illegible]
**BPNSF6**	3.323															
**BPNSF11**	1.864	3.156														
**BPNSF17**	1.813	2.010	3.441													
**BPNSF23**	1.796	1.996	2.122	3.260												
**BPNSF3**	-.667	-.682	-.836	-.868	2.439											
**BPNSF9**	-.838	-1.065	-1.124	-.945	1.357	2.458										
**BPNSF14**	-.559	-.812	-.894	-.884	1.177	1.571	2.426									
**BPNSF21**	-.464	-.703	-.468	-.773	.974	1.142	1.225	2.085								
**BPNSF2**	1.433	1.722	1.665	1.867	-.712	-.538	-.503	-.290	3.519							
**BPNSF8**	1.668	2.010	1.907	1.840	-.549	-.798	-.480	-.435	2.002	3.409						
**BPNSF20**	1.524	1.790	2.155	2.210	-.758	-.850	-.623	-.528	1.912	2.039	3.413					
**BPNSF22**	1.124	1.403	1.360	1.642	-.464	-.405	-.282	-.416	1.452	1.757	1.712	2.691				
**BPNSF4**	-.361	-.388	-.311	-.429	1.288	.862	.723	.835	-.205	-.408	-.427	-.384	1.950			
**BPNSF12**	-.429	-.598	-.641	-.739	1.005	1.373	1.415	1.160	-.526	-.492	-.635	-.615	1.038	2.284		
**BPNSF16**	-.332	-.588	-.662	-.596	.821	1.106	1.227	1.091	-.455	-.432	-.678	-.542	.947	1.496	2.081	
**BPNSF24**	-.450	-.664	-.434	-.656	.777	.976	1.024	1.165	-.511	-.588	-.620	-.386	.854	1.240	1.097	[illegible]
**BPNSF5**	1.294	1.442	1.256	1.280	-.159	-.356	-.211	-.127	1.709	1.574	1.312	1.256	-.268	-.367	-.367	[illegible]
**BPNSF10**	1.559	1.919	1.691	1.606	-.449	-.698	-.390	-.335	1.635	1.871	1.538	1.344	-.313	-.410	-.373	[illegible]
**BPNSF15**	.959	1.134	1.223	.858	-.137	-.205	-.131	-.007	.998	1.297	1.016	.972	-.272	-.030	-.132	[illegible]
**BPNSF18**	.343	.497	.627	.305	.093	.350	.466	.271	.410	.495	.474	.564	.020	.384	.299	[illegible]
**BPNSF1**	-.131	-.251	-.144	-.182	1.181	.880	.742	.810	.024	-.185	-.192	-.379	.984	.867	.732	[illegible]
**BPNSF7**	.060	-.376	-.236	-.313	.769	.925	1.011	1.017	-.030	-.013	-.149	-.177	.662	.982	.849	[illegible]
**BPNSF13**	-.240	-.526	-.450	-.360	.823	1.163	1.256	1.038	-.231	-.244	-.352	-.444	.867	1.454	1.177	[illegible]
**BPNSF19**	-.334	-.527	-.352	-.548	.796	1.060	1.088	1.298	-.136	-.275	-.311	-.280	.788	.993	1.003	[illegible]

Sample Covariances - Two Tailed Significance (PC) (g3 - Structural covariances)

	BPNSF6	BPNSF11	BPNSF17	BPNSF23	BPNSF3	BPNSF9	BPNSF14	BPNSF21	BPNSF2	BPNSF8	BPNSF20	BPNSF22	BPNSF4	BPNSF12	BPNSF16	BPNS[illegible]
**BPNSF6**	.010															
**BPNSF11**	.010	.010														
**BPNSF17**	.010	.010	.010													
**BPNSF23**	.010	.010	.010	.010												
**BPNSF3**	.010	.010	.010	.010	.010											
**BPNSF9**	.010	.010	.010	.010	.010	.010										
**BPNSF14**	.010	.010	.010	.010	.010	.010	.010									
**BPNSF21**	.010	.010	.010	.010	.010	.010	.010	.010								
**BPNSF2**	.010	.010	.010	.010	.010	.010	.010	.010	.010							
**BPNSF8**	.010	.010	.010	.010	.010	.010	.010	.010	.010	.010						
**BPNSF20**	.010	.010	.010	.010	.010	.010	.010	.010	.010	.010	.010					
**BPNSF22**	.010	.010	.010	.010	.010	.010	.010	.010	.010	.010	.010	.010				
**BPNSF4**	.010	.010	.010	.010	.010	.010	.010	.010	.010	.010	.010	.010	.010			
**BPNSF12**	.010	.010	.010	.010	.010	.010	.010	.010	.010	.010	.010	.010	.010	.010		
**BPNSF16**	.010	.010	.010	.010	.010	.010	.010	.010	.010	.010	.010	.010	.010	.010	.010	
**BPNSF24**	.010	.010	.010	.010	.010	.010	.010	.010	.010	.010	.010	.010	.010	.010	.010	[illegible]
**BPNSF5**	.010	.010	.010	.010	.010	.010	.010	.010	.010	.010	.010	.010	.010	.010	.010	[illegible]
**BPNSF10**	.010	.010	.010	.010	.010	.010	.010	.010	.010	.010	.010	.010	.010	.010	.010	[illegible]
**BPNSF15**	.010	.010	.010	.010	.010	.010	.016	.097	.010	.010	.010	.010	.010	.066	.014	[illegible]
**BPNSF18**	.373	.022	.010	.386	.431	.135	.010	.336	.197	.090	.074	.012	.149	.095	.281	[illegible]
**BPNSF1**	.019	.010	.010	.010	.010	.010	.010	.010	.175	.010	.010	.010	.010	.010	.010	[illegible]
**BPNSF7**	.178	.010	.010	.010	.010	.010	.010	.010	.062	.094	.010	.010	.010	.010	.010	[illegible]
**BPNSF13**	.010	.010	.010	.010	.010	.010	.010	.010	.010	.010	.010	.010	.010	.010	.010	[illegible]
**BPNSF19**	.010	.010	.010	.010	.010	.010	.010	.010	.016	.010	.010	.010	.010	.010	.010	[illegible]

Sample Correlations (g3 - Structural covariances)

Sample Correlations - Lower Bounds (PC) (g3 - Structural covariances)

	BPNSF6	BPNSF11	BPNSF17	BPNSF23	BPNSF3	BPNSF9	BPNSF14	BPNSF21	BPNSF2	BPNSF8	BPNSF20	BPNSF22	BPNSF4	BPNSF12	BPNSF16	BPNS[illegible]
**BPNSF6**	1.000															
**BPNSF11**	.468	1.000														
**BPNSF17**	.419	.488	1.000													
**BPNSF23**	.425	.543	.550	1.000												
**BPNSF3**	-.426	-.445	-.482	-.508	1.000											
**BPNSF9**	-.501	-.576	-.601	-.533	.424	1.000										
**BPNSF14**	-.381	-.472	-.526	-.499	.352	.546	1.000									
**BPNSF21**	-.352	-.477	-.371	-.484	.283	.389	.400	1.000								
**BPNSF2**	.289	.410	.347	.462	-.439	-.360	-.359	-.278	1.000							
**BPNSF8**	.386	.506	.435	.443	-.386	-.482	-.349	-.349	.466	1.000						
**BPNSF20**	.335	.440	.532	.576	-.445	-.498	-.401	-.390	.441	.496	1.000					
**BPNSF22**	.235	.384	.312	.444	-.380	-.366	-.320	-.374	.372	.483	.454	1.000				
**BPNSF4**	-.327	-.353	-.303	-.327	.445	.267	.191	.254	-.268	-.346	-.352	-.376	1.000			
**BPNSF12**	-.326	-.409	-.421	-.465	.305	.464	.476	.434	-.366	-.382	-.423	-.450	.377	1.000		
**BPNSF16**	-.305	-.423	-.438	-.403	.211	.332	.420	.399	-.329	-.376	-.462	-.430	.355	.591	1.000	
**BPNSF24**	-.342	-.437	-.344	-.430	.195	.311	.335	.447	-.374	-.418	-.410	-.360	.278	.473	.410	1.0[illegible]
**BPNSF5**	.285	.374	.265	.302	-.244	-.286	-.247	-.221	.403	.377	.273	.305	-.287	-.308	-.320	-.2[illegible]
**BPNSF10**	.357	.538	.409	.407	-.372	-.453	-.320	-.310	.382	.473	.368	.349	-.330	-.322	-.318	-.2[illegible]
**BPNSF15**	.143	.240	.235	.141	-.217	-.239	-.222	-.191	.185	.281	.179	.204	-.280	-.187	-.221	-.2[illegible]
**BPNSF18**	-.030	.031	.057	-.036	-.120	-.005	.040	-.025	-.019	.003	.007	.072	-.156	.004	-.039	.0[illegible]
**BPNSF1**	-.222	-.258	-.233	-.223	.327	.205	.133	.177	-.160	-.248	-.243	-.331	.293	.211	.151	.1[illegible]
**BPNSF7**	-.151	-.314	-.264	-.275	.140	.251	.281	.332	-.191	-.193	-.219	-.278	.153	.310	.248	.1[illegible]
**BPNSF13**	-.279	-.383	-.348	-.298	.189	.382	.415	.360	-.253	-.305	-.319	-.377	.295	.559	.431	.3[illegible]
**BPNSF19**	-.292	-.361	-.307	-.363	.227	.332	.339	.517	-.210	-.289	-.300	-.289	.272	.313	.348	.3[illegible]

Sample Correlations - Upper Bounds (PC) (g3 - Structural covariances)

	BPNSF6	BPNSF11	BPNSF17	BPNSF23	BPNSF3	BPNSF9	BPNSF14	BPNSF21	BPNSF2	BPNSF8	BPNSF20	BPNSF22	BPNSF4	BPNSF12	BPNSF16	BPNS[illegible]
**BPNSF6**	1.000															
**BPNSF11**	.627	1.000														
**BPNSF17**	.579	.644	1.000													
**BPNSF23**	.577	.652	.680	1.000												
**BPNSF3**	-.258	-.268	-.304	-.333	1.000											
**BPNSF9**	-.333	-.438	-.443	-.380	.613	1.000										
**BPNSF14**	-.221	-.321	-.341	-.346	.529	.682	1.000									
**BPNSF21**	-.189	-.314	-.192	-.332	.475	.562	.595	1.000								
**BPNSF2**	.452	.552	.514	.602	-.263	-.201	-.190	-.116	1.000							
**BPNSF8**	.525	.645	.582	.584	-.213	-.324	-.184	-.177	.617	1.000						
**BPNSF20**	.495	.583	.661	.706	-.279	-.337	-.242	-.208	.590	.640	1.000					
**BPNSF22**	.410	.519	.484	.584	-.205	-.174	-.125	-.195	.506	.617	.601	1.000				
**BPNSF4**	-.156	-.175	-.130	-.184	.626	.442	.370	.450	-.083	-.167	-.175	-.188	1.000			
**BPNSF12**	-.175	-.249	-.254	-.299	.470	.603	.630	.569	-.202	-.198	-.251	-.284	.537	1.000		
**BPNSF16**	-.132	-.262	-.271	-.255	.413	.531	.575	.576	-.173	-.174	-.284	-.256	.522	.721	1.000	
**BPNSF24**	-.192	-.290	-.179	-.283	.387	.484	.498	.594	-.205	-.241	-.256	-.173	.475	.613	.572	1.0[illegible]
**BPNSF5**	.435	.497	.409	.428	-.065	-.138	-.084	-.051	.550	.511	.431	.465	-.116	-.155	-.166	-.1[illegible]
**BPNSF10**	.541	.658	.567	.542	-.190	-.296	-.159	-.145	.541	.616	.517	.508	-.146	-.167	-.163	-.1[illegible]
**BPNSF15**	.332	.382	.405	.299	-.058	-.085	-.052	-.003	.329	.436	.339	.368	-.127	-.012	-.057	-.0[illegible]
**BPNSF18**	.132	.194	.231	.116	.042	.160	.214	.138	.152	.186	.180	.243	.011	.185	.145	.2[illegible]
**BPNSF1**	-.047	-.095	-.051	-.067	.497	.378	.315	.377	.008	-.066	-.071	-.154	.468	.384	.337	.3[illegible]
**BPNSF7**	.024	-.152	-.089	-.121	.364	.442	.468	.501	-.013	-.005	-.061	-.083	.346	.473	.429	.3[illegible]
**BPNSF13**	-.094	-.214	-.183	-.137	.387	.534	.587	.521	-.090	-.096	-.136	-.205	.457	.678	.584	.5[illegible]
**BPNSF19**	-.139	-.222	-.145	-.225	.393	.494	.522	.639	-.058	-.117	-.124	-.126	.421	.486	.502	.4[illegible]

Sample Correlations - Two Tailed Significance (PC) (g3 - Structural covariances)

	BPNSF6	BPNSF11	BPNSF17	BPNSF23	BPNSF3	BPNSF9	BPNSF14	BPNSF21	BPNSF2	BPNSF8	BPNSF20	BPNSF22	BPNSF4	BPNSF12	BPNSF16	BPNS[illegible]
**BPNSF6**	...															
**BPNSF11**	.010	...														
**BPNSF17**	.010	.010	...													
**BPNSF23**	.010	.010	.010	...												
**BPNSF3**	.010	.010	.010	.010	...											
**BPNSF9**	.010	.010	.010	.010	.010	...										
**BPNSF14**	.010	.010	.010	.010	.010	.010	...									
**BPNSF21**	.010	.010	.010	.010	.010	.010	.010	...								
**BPNSF2**	.010	.010	.010	.010	.010	.010	.010	.010	...							
**BPNSF8**	.010	.010	.010	.010	.010	.010	.010	.010	.010	...						
**BPNSF20**	.010	.010	.010	.010	.010	.010	.010	.010	.010	.010	...					
**BPNSF22**	.010	.010	.010	.010	.010	.010	.010	.010	.010	.010	.010	...				
**BPNSF4**	.010	.010	.010	.010	.010	.010	.010	.010	.010	.010	.010	.010	...			
**BPNSF12**	.010	.010	.010	.010	.010	.010	.010	.010	.010	.010	.010	.010	.010	...		
**BPNSF16**	.010	.010	.010	.010	.010	.010	.010	.010	.010	.010	.010	.010	.010	.010	...	

	BPNSF6	BPNSF11	BPNSF17	BPNSF23	BPNSF3	BPNSF9	BPNSF14	BPNSF21	BPNSF2	BPNSF8	BPNSF20	BPNSF22	BPNSF4	BPNSF12	BPNSF16	BPNSI
**BPNSF24**	.010	.010	.010	.010	.010	.010	.010	.010	.010	.010	.010	.010	.010	.010	.010	
**BPNSF5**	.010	.010	.010	.010	.010	.010	.010	.010	.010	.010	.010	.010	.010	.010	.010	.0
**BPNSF10**	.010	.010	.010	.010	.010	.010	.010	.010	.010	.010	.010	.010	.010	.010	.010	.0
**BPNSF15**	.010	.010	.010	.010	.010	.010	.016	.097	.010	.010	.010	.010	.010	.066	.014	.0
**BPNSF18**	.373	.022	.010	.387	.431	.135	.010	.336	.197	.090	.074	.012	.149	.095	.281	.0
**BPNSF1**	.019	.010	.010	.010	.010	.010	.010	.010	.175	.010	.010	.010	.010	.010	.010	.0
**BPNSF7**	.178	.010	.010	.010	.010	.010	.010	.010	.062	.094	.010	.010	.010	.010	.010	.0
**BPNSF13**	.010	.010	.010	.010	.010	.010	.010	.010	.010	.010	.010	.010	.010	.010	.010	.0
**BPNSF19**	.010	.010	.010	.010	.010	.010	.010	.010	.016	.010	.010	.010	.010	.010	.010	.0

Sample Means (g3 - Structural covariances)

Sample Means - Lower Bounds (PC) (g3 - Structural covariances)

	BPNSF6	BPNSF11	BPNSF17	BPNSF23	BPNSF3	BPNSF9	BPNSF14	BPNSF21	BPNSF2	BPNSF8	BPNSF20	BPNSF22	BPNSF4	BPNSF12	BPNSF16	BPNSF
**BPNSF6**	2.512	2.681	2.684	2.387	5.371	5.333	5.052	4.893	2.510	2.777	2.612	3.146	5.091	4.998	4.900	5.00

Sample Means - Upper Bounds (PC) (g3 - Structural covariances)

	BPNSF6	BPNSF11	BPNSF17	BPNSF23	BPNSF3	BPNSF9	BPNSF14	BPNSF21	BPNSF2	BPNSF8	BPNSF20	BPNSF22	BPNSF4	BPNSF12	BPNSF16	BPNSF
**BPNSF6**	2.774	2.931	2.973	2.629	5.613	5.584	5.294	5.097	2.809	3.057	2.898	3.394	5.299	5.234	5.137	5.23

Sample Means - Two Tailed Significance (PC) (g3 - Structural covariances)

	BPNSF6	BPNSF11	BPNSF17	BPNSF23	BPNSF3	BPNSF9	BPNSF14	BPNSF21	BPNSF2	BPNSF8	BPNSF20	BPNSF22	BPNSF4	BPNSF12	BPNSF16	BPNSF
**BPNSF6**	.010	.010	.010	.010	.010	.010	.010	.010	.010	.010	.010	.010	.010	.010	.010	.01

Bias-corrected percentile method (g3 - Structural covariances)

90% confidence intervals (bias-corrected percentile method)

Scalar Estimates (g3 - Structural covariances)

Regression Weights: (g3 - Structural covariances)

Parameter			Estimate	Lower	Upper	P
BPNSF19	<---	F1	1.000	1.000	1.000	...
BPNSF13	<---	F1	1.137	1.046	1.235	.005
BPNSF7	<---	F1	.855	.759	.953	.010
BPNSF1	<---	F1	.763	.630	.865	.018
BPNSF18	<---	F2	1.000	1.000	1.000	...
BPNSF15	<---	F2	2.472	2.005	3.283	.010
BPNSF10	<---	F2	3.106	2.501	4.343	.007
BPNSF5	<---	F2	2.568	2.064	3.455	.009
BPNSF24	<---	F3	1.000	1.000	1.000	...
BPNSF16	<---	F3	1.130	1.046	1.219	.009
BPNSF12	<---	F3	1.224	1.152	1.307	.004
BPNSF4	<---	F3	.850	.755	.938	.012
BPNSF22	<---	F4	1.000	1.000	1.000	...
BPNSF20	<---	F4	1.196	1.124	1.298	.005
BPNSF8	<---	F4	1.200	1.128	1.306	.004
BPNSF2	<---	F4	1.121	1.043	1.221	.006
BPNSF21	<---	F5	1.000	1.000	1.000	...
BPNSF14	<---	F5	1.094	1.026	1.178	.009
BPNSF9	<---	F5	1.039	.970	1.136	.007
BPNSF3	<---	F5	.881	.791	.984	.012
BPNSF23	<---	F6	1.000	1.000	1.000	...
BPNSF17	<---	F6	.976	.914	1.026	.019
BPNSF11	<---	F6	.959	.904	1.023	.007
BPNSF6	<---	F6	.863	.805	.932	.008

Standardized Regression Weights: (g3 - Structural covariances)

Parameter			Estimate	Lower	Upper	P
BPNSF19	<---	F1	.654	.598	.700	.018
BPNSF13	<---	F1	.723	.688	.764	.004
BPNSF7	<---	F1	.551	.492	.607	.012
BPNSF1	<---	F1	.452	.393	.503	.013

Parameter	Estimate	Lower	Upper	P
BPNSF18 <--- F2	.263	.196	.324	.013
BPNSF15 <--- F2	.556	.510	.596	.012
BPNSF10 <--- F2	.751	.704	.803	.010
BPNSF5 <--- F2	.611	.566	.663	.012
BPNSF24 <--- F3	.675	.629	.724	.014
BPNSF16 <--- F3	.769	.713	.810	.020
BPNSF12 <--- F3	.820	.786	.851	.012
BPNSF4 <--- F3	.593	.538	.652	.009
BPNSF22 <--- F4	.678	.634	.714	.016
BPNSF20 <--- F4	.739	.695	.786	.010
BPNSF8 <--- F4	.748	.709	.791	.004
BPNSF2 <--- F4	.673	.625	.720	.013
BPNSF21 <--- F5	.719	.660	.764	.023
BPNSF14 <--- F5	.743	.683	.780	.032
BPNSF9 <--- F5	.736	.693	.780	.015
BPNSF3 <--- F5	.604	.526	.666	.018
BPNSF23 <--- F6	.773	.742	.811	.011
BPNSF17 <--- F6	.736	.695	.783	.012
BPNSF11 <--- F6	.762	.722	.804	.009
BPNSF6 <--- F6	.651	.603	.703	.007

Intercepts: (g3 - Structural covariances)

Parameter	Estimate	Lower	Upper	P
**BPNSF19**	5.161	5.069	5.220	.030
**BPNSF13**	5.068	5.002	5.129	.020
**BPNSF7**	4.834	4.747	4.893	.032
**BPNSF1**	4.719	4.652	4.783	.019
**BPNSF18**	4.323	4.257	4.393	.012
**BPNSF15**	3.715	3.618	3.779	.015
**BPNSF10**	3.176	3.096	3.253	.006
**BPNSF5**	3.738	3.667	3.815	.007
**BPNSF24**	5.223	5.143	5.277	.032
**BPNSF16**	5.115	5.046	5.189	.016
**BPNSF12**	5.218	5.160	5.277	.018
**BPNSF4**	5.114	5.037	5.171	.023
**BPNSF22**	3.120	3.047	3.194	.006
**BPNSF20**	2.508	2.432	2.602	.003
**BPNSF8**	2.806	2.721	2.891	.007
**BPNSF2**	2.383	2.314	2.463	.005
**BPNSF21**	5.200	5.118	5.247	.044
**BPNSF14**	5.464	5.388	5.523	.026
**BPNSF9**	5.679	5.589	5.744	.026
**BPNSF3**	5.610	5.553	5.700	.009
**BPNSF23**	2.241	2.176	2.321	.003
**BPNSF17**	2.577	2.497	2.668	.004
**BPNSF11**	2.744	2.670	2.828	.005
**BPNSF6**	2.565	2.479	2.648	.009

Covariances: (g3 - Structural covariances)

Parameter	Estimate	Lower	Upper	P
F1 <--> F2	-.156	-.195	-.114	.011
F2 <--> F3	-.149	-.191	-.110	.009
F1 <--> F3	.728	.639	.801	.016
F2 <--> F4	.314	.229	.406	.009
F3 <--> F4	-.596	-.663	-.518	.019
F1 <--> F4	-.490	-.566	-.422	.012
F2 <--> F5	-.143	-.187	-.108	.006
F4 <--> F5	-.668	-.742	-.608	.006
F3 <--> F5	.770	.686	.857	.012
F1 <--> F5	.903	.798	1.036	.009
F6 <--> F5	-.958	-1.081	-.880	.004
F6 <--> F3	-.641	-.711	-.536	.018
F6 <--> F4	1.266	1.155	1.381	.008
F6 <--> F2	.372	.280	.474	.009
F6 <--> F1	-.622	-.718	-.538	.014

Correlations: (g3 - Structural covariances)

Parameter	Estimate	Lower	Upper	P

Parameter			Estimate	Lower	Upper	P
F1	<-->	F2	-.433	-.491	-.355	.014
F2	<-->	F3	-.411	-.475	-.357	.006
F1	<-->	F3	.836	.799	.878	.004
F2	<-->	F4	.758	.721	.800	.007
F3	<-->	F4	-.595	-.637	-.544	.015
F1	<-->	F4	-.494	-.540	-.433	.015
F2	<-->	F5	-.363	-.411	-.282	.019
F4	<-->	F5	-.615	-.659	-.566	.006
F3	<-->	F5	.808	.766	.840	.008
F1	<-->	F5	.956	.916	.994	.007
F6	<-->	F5	-.723	-.765	-.679	.009
F6	<-->	F3	-.525	-.565	-.460	.025
F6	<-->	F4	.910	.880	.943	.007
F6	<-->	F2	.737	.684	.780	.009
F6	<-->	F1	-.514	-.559	-.456	.023

Variances: (g3 - Structural covariances)

Parameter	Estimate	Lower	Upper	P
**F1**	.863	.726	.981	.016
**F2**	.150	.083	.232	.012
**F3**	.879	.757	.987	.016
**F4**	1.141	1.010	1.268	.012
**F5**	1.033	.918	1.171	.011
**F6**	1.696	1.534	1.876	.008
**e1**	1.153	.943	1.362	.009
**e2**	1.020	.828	1.182	.020
**e3**	1.452	1.207	1.788	.006
**e4**	1.961	1.698	2.261	.007
**e5**	2.018	1.799	2.292	.004
**e6**	2.056	1.807	2.312	.008
**e7**	1.123	.886	1.377	.008
**e8**	1.663	1.413	1.944	.007
**e9**	1.050	.834	1.259	.009
**e10**	.778	.613	.989	.004
**e11**	.643	.520	.779	.006
**e12**	1.168	.967	1.496	.007
**e13**	1.343	1.129	1.540	.008
**e14**	1.358	1.074	1.687	.005
**e15**	1.295	1.017	1.545	.015
**e16**	1.731	1.402	2.138	.008
**e17**	.966	.786	1.205	.003
**e18**	1.005	.809	1.289	.004
**e19**	.943	.762	1.206	.005
**e20**	1.400	1.061	1.785	.009
**e21**	1.144	.913	1.342	.009
**e22**	1.368	1.081	1.639	.007
**e23**	1.125	.899	1.327	.016
**e24**	1.720	1.318	2.059	.014

Matrices (g3 - Structural covariances)

Sample Covariances (g3 - Structural covariances)

Sample Covariances - Lower Bounds (BC) (g3 - Structural covariances)

	BPNSF6	BPNSF11	BPNSF17	BPNSF23	BPNSF3	BPNSF9	BPNSF14	BPNSF21	BPNSF2	BPNSF8	BPNSF20	BPNSF22	BPNSF4	BPNSF12	BPNSF16	BPNS[illegible]
**BPNSF6**	2.719															
**BPNSF11**	1.383	2.681														
**BPNSF17**	1.316	1.451	2.933													
**BPNSF23**	1.313	1.523	1.661	2.755												
**BPNSF3**	-1.124	-1.094	-1.233	-1.263	1.765											
**BPNSF9**	-1.290	-1.469	-1.622	-1.362	.893	1.844										
**BPNSF14**	-.942	-1.200	-1.418	-1.268	.773	1.118	1.983									
**BPNSF21**	-.844	-1.167	-.933	-1.143	.570	.729	.799	1.700								
**BPNSF2**	.910	1.248	1.130	1.396	-1.194	-.935	-.953	-.664	2.935							
**BPNSF8**	1.220	1.534	1.364	1.411	-1.051	-1.306	-.914	-.937	1.434	2.883						
**BPNSF20**	1.017	1.296	1.638	1.695	-1.137	-1.327	-1.089	-.926	1.341	1.506	2.845					
**BPNSF22**	.664	.991	.860	1.236	-.886	-.906	-.765	-.858	1.036	1.306	1.225	2.252				
**BPNSF4**	-.757	-.749	-.708	-.790	.816	.524	.377	.463	-.625	-.837	-.830	-.774	1.514			

	BPNSF6	BPNSF11	BPNSF17	BPNSF23	BPNSF3	BPNSF9	BPNSF14	BPNSF21	BPNSF2	BPNSF8	BPNSF20	BPNSF22	BPNSF4	BPNSF12	BPNSF16	BPNS[illegible]
**BPNSF12**	-.914	-1.017	-1.125	-1.185	.642	.973	1.027	.858	-.964	-.966	-1.085	-1.027	.727	1.900		
**BPNSF16**	-.728	-1.013	-1.073	-.986	.487	.683	.884	.736	-.822	-.922	-1.167	-.963	.633	1.153	1.739	
**BPNSF24**	-.852	-1.004	-.821	-1.042	.370	.608	.663	.803	-.899	-1.030	-.977	-.780	.523	.924	.778	[illegible]
**BPNSF5**	.815	1.065	.784	.877	-.591	-.749	-.683	-.517	1.226	1.090	.832	.743	-.674	-.794	-.769	[illegible]
**BPNSF10**	1.019	1.503	1.221	1.176	-.882	-1.131	-.766	-.703	1.116	1.416	1.067	.908	-.696	-.768	-.719	[illegible]
**BPNSF15**	.453	.685	.732	.448	-.553	-.624	-.584	-.549	.590	.795	.524	.525	-.629	-.492	-.539	[illegible]
**BPNSF18**	-.060	.114	.218	-.054	-.319	-.012	.079	-.102	-.017	-.004	.067	.177	-.295	-.020	-.110	[illegible]
**BPNSF1**	-.568	-.643	-.672	-.604	.708	.509	.305	.380	-.484	-.774	-.616	-.854	.593	.473	.336	[illegible]
**BPNSF7**	-.367	-.741	-.668	-.713	.302	.515	.611	.637	-.498	-.493	-.535	-.670	.290	.659	.473	[illegible]
**BPNSF13**	-.697	-.911	-.932	-.742	.420	.811	.868	.747	-.648	-.788	-.808	-.897	.574	1.109	.843	[illegible]
**BPNSF19**	-.661	-.859	-.740	-.872	.462	.670	.709	.966	-.490	-.696	-.707	-.640	.465	.612	.701	[illegible]

Sample Covariances - Upper Bounds (BC) (g3 - Structural covariances)

	BPNSF6	BPNSF11	BPNSF17	BPNSF23	BPNSF3	BPNSF9	BPNSF14	BPNSF21	BPNSF2	BPNSF8	BPNSF20	BPNSF22	BPNSF4	BPNSF12	BPNSF16	BPNS[illegible]
**BPNSF6**	3.289															
**BPNSF11**	1.900	3.183														
**BPNSF17**	1.833	2.009	3.462													
**BPNSF23**	1.818	1.990	2.131	3.261												
**BPNSF3**	-.688	-.683	-.837	-.827	2.431											
**BPNSF9**	-.838	-1.073	-1.150	-.901	1.357	2.456										
**BPNSF14**	-.540	-.821	-.930	-.874	1.188	1.577	2.454									
**BPNSF21**	-.472	-.705	-.488	-.769	.973	1.117	1.182	2.181								
**BPNSF2**	1.453	1.750	1.679	1.864	-.747	-.543	-.505	-.317	3.531							
**BPNSF8**	1.711	2.046	1.914	1.878	-.589	-.805	-.498	-.473	2.002	3.426						
**BPNSF20**	1.557	1.781	2.156	2.208	-.706	-.849	-.636	-.471	1.912	2.042	3.446					
**BPNSF22**	1.150	1.440	1.360	1.718	-.460	-.433	-.316	-.466	1.513	1.778	1.716	2.720				
**BPNSF4**	-.385	-.350	-.315	-.435	1.278	.886	.723	.839	-.218	-.446	-.443	-.388	1.945			
**BPNSF12**	-.442	-.612	-.659	-.759	1.043	1.409	1.440	1.200	-.565	-.505	-.629	-.637	1.054	2.368		
**BPNSF16**	-.334	-.588	-.641	-.600	.867	1.113	1.258	1.110	-.455	-.444	-.682	-.559	.949	1.531	2.147	
**BPNSF24**	-.480	-.647	-.410	-.656	.768	.994	1.017	1.174	-.509	-.635	-.549	-.405	.872	1.259	1.107	[illegible]
**BPNSF5**	1.292	1.503	1.258	1.290	-.148	-.372	-.251	-.127	1.716	1.564	1.355	1.232	-.279	-.373	-.414	[illegible]
**BPNSF10**	1.570	1.985	1.697	1.614	-.449	-.705	-.369	-.330	1.635	1.918	1.557	1.354	-.311	-.411	-.333	[illegible]
**BPNSF15**	1.001	1.166	1.270	.918	-.153	-.228	-.176	-.054	1.037	1.283	1.029	.973	-.275	-.091	-.155	[illegible]
**BPNSF18**	.379	.516	.679	.353	.054	.344	.459	.233	.440	.478	.512	.573	.020	.370	.256	[illegible]
**BPNSF1**	-.123	-.224	-.175	-.182	1.181	.923	.760	.804	.013	-.225	-.161	-.385	.989	.879	.759	[illegible]
**BPNSF7**	.076	-.339	-.227	-.322	.763	.924	1.000	1.010	-.038	-.014	-.143	-.201	.652	1.013	.849	[illegible]
**BPNSF13**	-.233	-.528	-.497	-.370	.840	1.196	1.267	1.079	-.232	-.308	-.354	-.464	.891	1.468	1.202	[illegible]
**BPNSF19**	-.277	-.527	-.317	-.540	.794	1.061	1.091	1.314	-.112	-.267	-.263	-.291	.783	.993	1.018	[illegible]

Sample Covariances - Two Tailed Significance (BC) (g3 - Structural covariances)

	BPNSF6	BPNSF11	BPNSF17	BPNSF23	BPNSF3	BPNSF9	BPNSF14	BPNSF21	BPNSF2	BPNSF8	BPNSF20	BPNSF22	BPNSF4	BPNSF12	BPNSF16	BPNS[illegible]
**BPNSF6**	.014															
**BPNSF11**	.007	.007														
**BPNSF17**	.006	.012	.007													
**BPNSF23**	.005	.012	.009	.009												
**BPNSF3**	.006	.009	.008	.019	.015											
**BPNSF9**	.011	.009	.005	.021	.010	.012										
**BPNSF14**	.023	.009	.007	.015	.007	.009	.006									
**BPNSF21**	.008	.009	.009	.011	.012	.025	.015	.004								
**BPNSF2**	.008	.006	.008	.012	.006	.007	.009	.006	.006							
**BPNSF8**	.003	.005	.009	.002	.005	.009	.006	.004	.010	.007						
**BPNSF20**	.005	.012	.008	.012	.018	.011	.008	.020	.010	.009	.005					
**BPNSF22**	.007	.009	.011	.003	.012	.005	.005	.003	.006	.007	.009	.004				
**BPNSF4**	.008	.025	.008	.006	.011	.005	.010	.009	.007	.004	.005	.009	.012			
**BPNSF12**	.003	.006	.008	.006	.005	.003	.004	.004	.005	.007	.012	.006	.007	.002		
**BPNSF16**	.009	.010	.012	.008	.004	.009	.002	.007	.009	.009	.009	.007	.009	.004	.004	
**BPNSF24**	.005	.013	.019	.010	.015	.008	.012	.007	.011	.005	.034	.006	.006	.005	.006	[illegible]
**BPNSF5**	.011	.005	.009	.006	.012	.005	.004	.010	.005	.012	.005	.018	.006	.007	.005	[illegible]
**BPNSF10**	.008	.005	.009	.009	.010	.009	.016	.012	.010	.003	.006	.009	.012	.009	.015	[illegible]
**BPNSF15**	.006	.006	.004	.004	.007	.005	.005	.039	.005	.013	.008	.009	.009	.032	.006	[illegible]
**BPNSF18**	.321	.012	.004	.233	.223	.156	.012	.595	.150	.105	.042	.008	.149	.149	.468	[illegible]
**BPNSF1**	.034	.016	.008	.010	.010	.004	.007	.014	.125	.005	.025	.005	.007	.007	.006	[illegible]
**BPNSF7**	.222	.026	.014	.008	.012	.011	.011	.012	.049	.089	.015	.006	.012	.003	.011	[illegible]
**BPNSF13**	.011	.009	.005	.006	.004	.004	.007	.003	.009	.005	.009	.005	.004	.004	.005	[illegible]
**BPNSF19**	.028	.010	.019	.016	.012	.009	.007	.006	.035	.013	.023	.006	.012	.010	.005	[illegible]

Sample Correlations (g3 - Structural covariances)

Sample Correlations - Lower Bounds (BC) (g3 - Structural covariances)

	BPNSF6	BPNSF11	BPNSF17	BPNSF23	BPNSF3	BPNSF9	BPNSF14	BPNSF21	BPNSF2	BPNSF8	BPNSF20	BPNSF22	BPNSF4	BPNSF12	BPNSF16	BPNS
**BPNSF6**	1.000															
**BPNSF11**	.477	1.000														
**BPNSF17**	.403	.492	1.000													
**BPNSF23**	.445	.543	.545	1.000												
**BPNSF3**	-.434	-.445	-.483	-.505	1.000											
**BPNSF9**	-.488	-.567	-.579	-.529	.398	1.000										
**BPNSF14**	-.366	-.467	-.523	-.485	.342	.546	1.000									
**BPNSF21**	-.346	-.469	-.371	-.481	.272	.364	.374	1.000								
**BPNSF2**	.292	.412	.343	.470	-.431	-.350	-.357	-.285	1.000							
**BPNSF8**	.400	.509	.443	.474	-.392	-.485	-.352	-.373	.463	1.000						
**BPNSF20**	.340	.439	.529	.561	-.431	-.489	-.401	-.368	.441	.496	1.000					
**BPNSF22**	.237	.393	.312	.447	-.376	-.367	-.340	-.387	.372	.486	.454	1.000				
**BPNSF4**	-.327	-.338	-.303	-.343	.436	.270	.200	.256	-.269	-.352	-.355	-.378	1.000			
**BPNSF12**	-.327	-.410	-.424	-.466	.308	.464	.477	.436	-.361	-.382	-.404	-.459	.377	1.000		
**BPNSF16**	-.304	-.421	-.423	-.408	.224	.330	.425	.396	-.331	-.360	-.454	-.438	.356	.593	1.000	
**BPNSF24**	-.355	-.429	-.342	-.429	.180	.314	.323	.446	-.370	-.423	-.397	-.366	.292	.474	.408	1.0
**BPNSF5**	.279	.381	.258	.306	-.239	-.295	-.265	-.227	.401	.375	.279	.298	-.287	-.308	-.326	-.2
**BPNSF10**	.352	.538	.408	.407	-.346	-.454	-.305	-.307	.382	.483	.369	.349	-.324	-.321	-.317	-.2
**BPNSF15**	.148	.240	.248	.154	-.221	-.240	-.231	-.232	.195	.284	.181	.202	-.279	-.195	-.225	-.2
**BPNSF18**	-.025	.046	.083	-.028	-.137	-.012	.030	-.055	-.005	-.004	.011	.074	-.155	-.013	-.053	.0
**BPNSF1**	-.206	-.243	-.233	-.219	.332	.205	.134	.171	-.166	-.267	-.220	-.343	.296	.214	.150	.1
**BPNSF7**	-.149	-.307	-.252	-.274	.121	.240	.284	.313	-.193	-.201	-.218	-.291	.150	.316	.231	.1
**BPNSF13**	-.282	-.386	-.350	-.315	.207	.389	.407	.367	-.254	-.310	-.305	-.388	.309	.563	.434	.3
**BPNSF19**	-.280	-.357	-.295	-.359	.219	.332	.329	.517	-.200	-.285	-.288	-.303	.272	.309	.337	.3

Sample Correlations - Upper Bounds (BC) (g3 - Structural covariances)

	BPNSF6	BPNSF11	BPNSF17	BPNSF23	BPNSF3	BPNSF9	BPNSF14	BPNSF21	BPNSF2	BPNSF8	BPNSF20	BPNSF22	BPNSF4	BPNSF12	BPNSF16	BPNS
**BPNSF6**	1.000															
**BPNSF11**	.629	1.000														
**BPNSF17**	.569	.647	1.000													
**BPNSF23**	.592	.652	.674	1.000												
**BPNSF3**	-.258	-.270	-.308	-.324	1.000											
**BPNSF9**	-.322	-.433	-.432	-.375	.596	1.000										
**BPNSF14**	-.205	-.316	-.340	-.343	.518	.686	1.000									
**BPNSF21**	-.182	-.306	-.192	-.331	.460	.553	.561	1.000								
**BPNSF2**	.458	.559	.512	.605	-.252	-.195	-.186	-.121	1.000							
**BPNSF8**	.544	.645	.583	.613	-.224	-.324	-.185	-.185	.615	1.000						
**BPNSF20**	.498	.580	.651	.695	-.262	-.324	-.242	-.176	.593	.642	1.000					
**BPNSF22**	.413	.530	.484	.588	-.204	-.174	-.140	-.208	.506	.621	.601	1.000				
**BPNSF4**	-.158	-.158	-.130	-.192	.616	.444	.378	.454	-.086	-.184	-.184	-.188	1.000			
**BPNSF12**	-.177	-.249	-.256	-.299	.473	.601	.631	.571	-.198	-.198	-.245	-.292	.537	1.000		
**BPNSF16**	-.132	-.259	-.255	-.257	.418	.529	.582	.569	-.176	-.172	-.282	-.262	.523	.722	1.000	
**BPNSF24**	-.208	-.278	-.168	-.281	.372	.487	.492	.593	-.203	-.256	-.228	-.188	.488	.615	.569	1.0
**BPNSF5**	.433	.509	.398	.436	-.058	-.147	-.102	-.054	.544	.509	.440	.460	-.116	-.156	-.173	-.1
**BPNSF10**	.537	.658	.561	.541	-.158	-.296	-.135	-.131	.533	.619	.518	.508	-.135	-.167	-.158	-.1
**BPNSF15**	.337	.382	.416	.316	-.059	-.085	-.073	-.023	.344	.436	.353	.357	-.123	-.037	-.061	-.0
**BPNSF18**	.136	.210	.244	.131	.029	.152	.212	.113	.175	.179	.188	.244	.012	.165	.127	.2
**BPNSF1**	-.037	-.086	-.051	-.066	.501	.378	.325	.366	.006	-.076	-.056	-.162	.470	.390	.337	.3
**BPNSF7**	.029	-.139	-.082	-.120	.353	.433	.469	.480	-.015	-.006	-.057	-.086	.344	.482	.424	.3
**BPNSF13**	-.100	-.216	-.187	-.147	.401	.543	.579	.527	-.091	-.110	-.117	-.210	.459	.683	.593	.5
**BPNSF19**	-.114	-.220	-.120	-.214	.384	.493	.510	.639	-.044	-.109	-.098	-.137	.421	.474	.497	.4

Sample Correlations - Two Tailed Significance (BC) (g3 - Structural covariances)

	BPNSF6	BPNSF11	BPNSF17	BPNSF23	BPNSF3	BPNSF9	BPNSF14	BPNSF21	BPNSF2	BPNSF8	BPNSF20	BPNSF22	BPNSF4	BPNSF12	BPNSF16	BPNS
**BPNSF6**	...															
**BPNSF11**	.007	...														
**BPNSF17**	.018	.009	...													
**BPNSF23**	.006	.010	.014	...												
**BPNSF3**	.009	.009	.007	.015	...											
**BPNSF9**	.018	.014	.019	.016	.023	...										
**BPNSF14**	.025	.012	.012	.020	.016	.009	...									
**BPNSF21**	.015	.018	.010	.013	.019	.021	.025	...								
**BPNSF2**	.007	.007	.014	.007	.013	.014	.012	.007	...							
**BPNSF8**	.003	.009	.008	.002	.006	.009	.009	.006	.012	...						
**BPNSF20**	.007	.012	.016	.016	.023	.023	.010	.032	.009	.009	...					
**BPNSF22**	.009	.005	.010	.007	.011	.009	.004	.005	.010	.007	.011	...				
**BPNSF4**	.009	.021	.010	.006	.014	.007	.008	.009	.008	.005	.006	.009	...			

	BPNSF6	BPNSF11	BPNSF17	BPNSF23	BPNSF3	BPNSF9	BPNSF14	BPNSF21	BPNSF2	BPNSF8	BPNSF20	BPNSF22	BPNSF4	BPNSF12	BPNSF16	BPNS
**BPNSF12**	.009	.009	.009	.009	.007	.012	.009	.008	.013	.010	.020	.006	.010	...		
**BPNSF16**	.011	.011	.020	.008	.007	.012	.007	.012	.009	.013	.012	.006	.009	.009	...	
**BPNSF24**	.003	.015	.013	.012	.018	.008	.014	.011	.012	.007	.034	.007	.007	.008	.013	
**BPNSF5**	.012	.004	.016	.006	.014	.006	.004	.008	.012	.013	.007	.014	.010	.009	.005	.0
**BPNSF10**	.015	.010	.012	.012	.021	.009	.026	.013	.012	.005	.009	.010	.016	.012	.012	.0
**BPNSF15**	.008	.010	.005	.005	.009	.009	.005	.042	.005	.009	.009	.012	.011	.037	.010	.0
**BPNSF18**	.334	.012	.005	.264	.254	.214	.015	.612	.142	.116	.056	.010	.157	.188	.468	.0
**BPNSF1**	.038	.014	.010	.012	.008	.010	.007	.018	.138	.007	.028	.005	.008	.007	.011	.0
**BPNSF7**	.213	.021	.021	.012	.019	.015	.009	.030	.049	.084	.012	.007	.012	.006	.015	.0
**BPNSF13**	.008	.008	.008	.006	.004	.006	.018	.007	.009	.007	.019	.005	.006	.006	.005	.0
**BPNSF19**	.026	.012	.025	.016	.018	.012	.015	.012	.035	.012	.026	.005	.010	.019	.016	.0

Sample Means (g3 - Structural covariances)

Sample Means - Lower Bounds (BC) (g3 - Structural covariances)

	BPNSF6	BPNSF11	BPNSF17	BPNSF23	BPNSF3	BPNSF9	BPNSF14	BPNSF21	BPNSF2	BPNSF8	BPNSF20	BPNSF22	BPNSF4	BPNSF12	BPNSF16	BPNSF
**BPNSF6**	2.488	2.672	2.698	2.395	5.381	5.325	5.067	4.869	2.511	2.767	2.639	3.128	5.101	4.983	4.896	4.98

Sample Means - Upper Bounds (BC) (g3 - Structural covariances)

	BPNSF6	BPNSF11	BPNSF17	BPNSF23	BPNSF3	BPNSF9	BPNSF14	BPNSF21	BPNSF2	BPNSF8	BPNSF20	BPNSF22	BPNSF4	BPNSF12	BPNSF16	BPNSF
**BPNSF6**	2.760	2.910	3.010	2.644	5.626	5.571	5.299	5.078	2.820	3.048	2.912	3.386	5.308	5.207	5.117	5.21

Sample Means - Two Tailed Significance (BC) (g3 - Structural covariances)

	BPNSF6	BPNSF11	BPNSF17	BPNSF23	BPNSF3	BPNSF9	BPNSF14	BPNSF21	BPNSF2	BPNSF8	BPNSF20	BPNSF22	BPNSF4	BPNSF12	BPNSF16	BPNSF
**BPNSF6**	.019	.016	.005	.006	.007	.019	.007	.021	.009	.019	.005	.014	.006	.021	.018	.01

Minimization History (Structural covariances)

Iteration		Negative eigenvalues	Condition #	Smallest eigenvalue	Diameter	F	NTries	Ratio
**0**	e	24		-.847	9999.000	17202.586	0	9999.000
**1**	e*	13		-1.832	3.549	7097.246	19	.325
**2**	e	11		-.677	.228	6274.816	6	.960
**3**	e*	5		-.152	.542	5407.119	6	.569
**4**	e	1		-.069	.606	4166.517	5	.903
**5**	e	0	4763.574		.559	3466.367	5	.854
**6**	e	0	1270.791		1.104	3091.445	3	.000
**7**	e	0	2095.611		1.241	2678.397	1	.991
**8**	e	0	2195.030		.488	2625.704	2	.000
**9**	e	0	7534.292		.438	2584.770	1	1.131
**10**	e	0	9462.687		.618	2583.175	1	.192
**11**	e	0	41398.311		.273	2573.603	1	1.027
**12**	e	0	48566.148		.269	2573.188	1	.909
**13**	e	0	74601.276		.049	2573.087	1	1.017
**14**	e	0	79119.072		.014	2573.086	1	1.005
**15**	e	0	79951.159		.000	2573.086	1	1.000

Pairwise Parameter Comparisons (Structural covariances)

Variance-covariance Matrix of Estimates (Structural covariances)

	a1_1	a2_1	a3_1	a4_1	a5_1	a6_1	a7_1	a8_1	a9_1	a10_1	a11_1	a12_1	a13_1	a14_1	a15_1	a16_1	a17_1	a18_1	ccc1_1	ccc2_1
**a1_1**	.003																			
**a2_1**	.001	.003																		
**a3_1**	.001	.001	.003																	
**a4_1**	.000	.000	.000	.096																
**a5_1**	.000	.000	.000	.104	.142															
**a6_1**	.000	.000	.000	.086	.108	.101														
**a7_1**	.000	.000	.000	.000	.000	.000	.002													
**a8_1**	.000	.000	.000	.000	.000	.000	.001	.002												
**a9_1**	.000	.000	.000	.000	.000	.000	.001	.001	.002											
**a10_1**	.000	.000	.000	.000	.000	.000	.000	.000	.000	.002										
**a11_1**	.000	.000	.000	.000	.000	.000	.000	.000	.000	.001	.003									
**a12_1**	.000	.000	.000	.000	.000	.000	.000	.000	.000	.001	.001	.002								
**a13_1**	.000	.000	.000	.000	.000	.000	.000	.000	.000	.000	.000	.000	.002							
**a14_1**	.000	.000	.000	.000	.000	.000	.000	.000	.000	.000	.000	.000	.001	.002						

	a1_1	a2_1	a3_1	a4_1	a5_1	a6_1	a7_1	a8_1	a9_1	a10_1	a11_1	a12_1	a13_1	a14_1	a15_1	a16_1	a17_1	a18_1	ccc1_1	ccc2_1
**a15_1**	.000	.000	.000	.000	.000	.000	.000	.000	.000	.000	.000	.000	.001	.001	.002					
**a16_1**	.000	.000	.000	.000	.000	.000	.000	.000	.000	.000	.000	.000	.000	.000	.000	.001				
**a17_1**	.000	.000	.000	.000	.000	.000	.000	.000	.000	.000	.000	.000	.000	.000	.000	.001	.001			
**a18_1**	.000	.000	.000	.000	.000	.000	.000	.000	.000	.000	.000	.000	.000	.000	.000	.000	.000	.001		
**ccc1_1**	.000	.000	.000	.005	.007	.005	.000	.000	.000	.000	.000	.000	.000	.000	.000	.000	.000	.000	.001	
**ccc2_1**	.000	.000	.000	.005	.006	.005	.000	.000	.000	.000	.000	.000	.000	.000	.000	.000	.000	.000	.000	.001
**ccc3_1**	-.001	-.001	-.001	.000	.000	.000	-.001	-.001	-.001	.000	.000	.000	.000	.000	.000	.000	.000	.000	.000	.000
**ccc4_1**	.000	.000	.000	-.011	-.013	-.011	.000	.000	.000	.000	.000	.000	.000	.000	.000	.000	.000	.000	-.001	-.001
**ccc5_1**	.000	.000	.000	.000	.000	.000	.001	.001	.001	.001	.001	.001	.000	.000	.000	.000	.000	.000	.000	.000
**ccc6_1**	.001	.001	.000	.000	.000	.000	.000	.000	.000	.001	.001	.001	.000	.000	.000	.000	.000	.000	.000	.000
**ccc7_1**	.000	.000	.000	.005	.006	.005	.000	.000	.000	.000	.000	.000	.000	.000	.000	.000	.000	.000	.000	.000
**ccc8_1**	.000	.000	.000	.000	.000	.000	.000	.000	.000	.001	.001	.001	.001	.001	.001	.000	.000	.000	.000	.000
**ccc9_1**	.000	.000	.000	.000	.000	.000	-.001	-.001	-.001	.000	.000	.000	-.001	-.001	-.001	.000	.000	.000	.000	.000
**ccc10_1**	-.001	-.001	-.001	.000	.000	.000	.000	.000	.000	.000	.000	.000	-.001	-.001	-.001	.000	.000	.000	.000	.000
**ccc11_1**	.000	.000	.000	.000	.000	.000	.000	.000	.000	.000	.000	.000	.001	.001	.001	.001	.000	.000	.000	.000
**ccc12_1**	.000	.000	.000	.000	.000	.000	.001	.001	.001	.000	.000	.000	.000	.000	.000	.000	.000	.000	.000	.000
**ccc13_1**	.000	.000	.000	.000	.000	.000	.000	.000	.000	-.002	-.002	-.001	.000	.000	.000	-.001	-.001	-.001	.000	.000
**ccc14_1**	.000	.000	.000	-.013	-.016	-.013	.000	.000	.000	.000	.000	.000	.000	.000	.000	.000	.000	.000	-.001	-.001
**ccc15_1**	.001	.001	.001	.000	.000	.000	.000	.000	.000	.000	.000	.000	.000	.000	.000	.000	.000	.000	.001	.000
**vvv1_1**	-.003	-.002	-.002	.000	.000	.000	.000	.000	.000	.000	.000	.000	.000	.000	.000	.000	.000	.000	-.001	.000
**v1_1**	.000	.000	.000	.000	.000	.000	.000	.000	.000	.000	.000	.000	.000	.000	.000	.000	.000	.000	.000	.000
**v2_1**	.000	.000	.000	.000	.000	.000	.000	.000	.000	.000	.000	.000	.000	.000	.000	.000	.000	.000	.000	.000
**v3_1**	.000	.000	.000	.000	.000	.000	.000	.000	.000	.000	.000	.000	.000	.000	.000	.000	.000	.000	.000	.000
**v4_1**	.000	.000	.000	.000	.000	.000	.000	.000	.000	.000	.000	.000	.000	.000	.000	.000	.000	.000	.000	.000
**vvv2_1**	.000	.000	.000	-.010	-.013	-.011	.000	.000	.000	.000	.000	.000	.000	.000	.000	.000	.000	.000	-.001	-.001
**v5_1**	.000	.000	.000	.002	.003	.002	.000	.000	.000	.000	.000	.000	.000	.000	.000	.000	.000	.000	.000	.000
**v6_1**	.000	.000	.000	-.003	.000	.000	.000	.000	.000	.000	.000	.000	.000	.000	.000	.000	.000	.000	.000	.000
**v7_1**	.000	.000	.000	.000	-.004	.000	.000	.000	.000	.000	.000	.000	.000	.000	.000	.000	.000	.000	.000	.000
**v8_1**	.000	.000	.000	.000	.000	-.002	.000	.000	.000	.000	.000	.000	.000	.000	.000	.000	.000	.000	.000	.000
**vvv3_1**	.000	.000	.000	.000	.000	.000	-.002	-.002	-.002	.000	.000	.000	.000	.000	.000	.000	.000	.000	.000	.000
**v9_1**	.000	.000	.000	.000	.000	.000	.000	.000	.000	.000	.000	.000	.000	.000	.000	.000	.000	.000	.000	.000
**v10_1**	.000	.000	.000	.000	.000	.000	.000	.000	.000	.000	.000	.000	.000	.000	.000	.000	.000	.000	.000	.000
**v11_1**	.000	.000	.000	.000	.000	.000	.000	.000	.000	.000	.000	.000	.000	.000	.000	.000	.000	.000	.000	.000
**v12_1**	.000	.000	.000	.000	.000	.000	.000	.000	.000	.000	.000	.000	.000	.000	.000	.000	.000	.000	.000	.000
**vvv4_1**	.000	.000	.000	.000	.000	.000	.000	.000	.000	-.003	-.003	-.003	.000	.000	.000	.000	.000	.000	.000	.000
**v13_1**	.000	.000	.000	.000	.000	.000	.000	.000	.000	.000	.000	.000	.000	.000	.000	.000	.000	.000	.000	.000
**v14_1**	.000	.000	.000	.000	.000	.000	.000	.000	.000	.000	.000	.000	.000	.000	.000	.000	.000	.000	.000	.000
**v15_1**	.000	.000	.000	.000	.000	.000	.000	.000	.000	.000	.000	.000	.000	.000	.000	.000	.000	.000	.000	.000
**v16_1**	.000	.000	.000	.000	.000	.000	.000	.000	.000	.000	.000	.000	.000	.000	.000	.000	.000	.000	.000	.000
**vvv5_1**	.000	.000	.000	.000	.000	.000	.000	.000	.000	.000	.000	.000	-.002	-.002	-.002	.000	.000	.000	.000	.000
**v17_1**	.000	.000	.000	.000	.000	.000	.000	.000	.000	.000	.000	.000	.000	.000	.000	.000	.000	.000	.000	.000
**v18_1**	.000	.000	.000	.000	.000	.000	.000	.000	.000	.000	.000	.000	.000	.000	.000	.000	.000	.000	.000	.000
**v19_1**	.000	.000	.000	.000	.000	.000	.000	.000	.000	.000	.000	.000	.000	.000	.000	.000	.000	.000	.000	.000
**v20_1**	.000	.000	.000	.000	.000	.000	.000	.000	.000	.000	.000	.000	.000	.000	.000	.000	.000	.000	.000	.000
**vvv6_1**	.000	.000	.000	.000	.000	.000	.000	.000	.000	.000	.000	.000	.000	.000	.000	-.002	-.002	-.002	.000	.000
**v21_1**	.000	.000	.000	.000	.000	.000	.000	.000	.000	.000	.000	.000	.000	.000	.000	.000	.000	.000	.000	.000
**v22_1**	.000	.000	.000	.000	.000	.000	.000	.000	.000	.000	.000	.000	.000	.000	.000	.000	.000	.000	.000	.000
**v23_1**	.000	.000	.000	.000	.000	.000	.000	.000	.000	.000	.000	.000	.000	.000	.000	.000	.000	.000	.000	.000
**v24_1**	.000	.000	.000	.000	.000	.000	.000	.000	.000	.000	.000	.000	.000	.000	.000	.000	.000	.000	.000	.000
**i1_1**	.000	.000	.000	.000	.000	.000	.000	.000	.000	.000	.000	.000	.000	.000	.000	.000	.000	.000	.000	.000
**i2_1**	.000	.000	.000	.000	.000	.000	.000	.000	.000	.000	.000	.000	.000	.000	.000	.000	.000	.000	.000	.000
**i3_1**	.000	.000	.000	.000	.000	.000	.000	.000	.000	.000	.000	.000	.000	.000	.000	.000	.000	.000	.000	.000
**i4_1**	.000	.000	.000	.000	.000	.000	.000	.000	.000	.000	.000	.000	.000	.000	.000	.000	.000	.000	.000	.000
**i5_1**	.000	.000	.000	.000	.000	.000	.000	.000	.000	.000	.000	.000	.000	.000	.000	.000	.000	.000	.000	.000
**i6_1**	.000	.000	.000	.000	.000	.000	.000	.000	.000	.000	.000	.000	.000	.000	.000	.000	.000	.000	.000	.000
**i7_1**	.000	.000	.000	.000	.000	.000	.000	.000	.000	.000	.000	.000	.000	.000	.000	.000	.000	.000	.000	.000
**i8_1**	.000	.000	.000	.000	.000	.000	.000	.000	.000	.000	.000	.000	.000	.000	.000	.000	.000	.000	.000	.000
**i9_1**	.000	.000	.000	.000	.000	.000	.000	.000	.000	.000	.000	.000	.000	.000	.000	.000	.000	.000	.000	.000
**i10_1**	.000	.000	.000	.000	.000	.000	.000	.000	.000	.000	.000	.000	.000	.000	.000	.000	.000	.000	.000	.000
**i11_1**	.000	.000	.000	.000	.000	.000	.000	.000	.000	.000	.000	.000	.000	.000	.000	.000	.000	.000	.000	.000
**i12_1**	.000	.000	.000	.000	.000	.000	.000	.000	.000	.000	.000	.000	.000	.000	.000	.000	.000	.000	.000	.000
**i13_1**	.000	.000	.000	.000	.000	.000	.000	.000	.000	.000	.000	.000	.000	.000	.000	.000	.000	.000	.000	.000
**i14_1**	.000	.000	.000	.000	.000	.000	.000	.000	.000	.000	.000	.000	.000	.000	.000	.000	.000	.000	.000	.000
**i15_1**	.000	.000	.000	.000	.000	.000	.000	.000	.000	.000	.000	.000	.000	.000	.000	.000	.000	.000	.000	.000
**i16_1**	.000	.000	.000	.000	.000	.000	.000	.000	.000	.000	.000	.000	.000	.000	.000	.000	.000	.000	.000	.000
**i17_1**	.000	.000	.000	.000	.000	.000	.000	.000	.000	.000	.000	.000	.000	.000	.000	.000	.000	.000	.000	.000
**i18_1**	.000	.000	.000	.000	.000	.000	.000	.000	.000	.000	.000	.000	.000	.000	.000	.000	.000	.000	.000	.000
**i19_1**	.000	.000	.000	.000	.000	.000	.000	.000	.000	.000	.000	.000	.000	.000	.000	.000	.000	.000	.000	.000
**i20_1**	.000	.000	.000	.000	.000	.000	.000	.000	.000	.000	.000	.000	.000	.000	.000	.000	.000	.000	.000	.000
**i21_1**	.000	.000	.000	.000	.000	.000	.000	.000	.000	.000	.000	.000	.000	.000	.000	.000	.000	.000	.000	.000
**i22_1**	.000	.000	.000	.000	.000	.000	.000	.000	.000	.000	.000	.000	.000	.000	.000	.000	.000	.000	.000	.000
**i23_1**	.000	.000	.000	.000	.000	.000	.000	.000	.000	.000	.000	.000	.000	.000	.000	.000	.000	.000	.000	.000

	a1_1	a2_1	a3_1	a4_1	a5_1	a6_1	a7_1	a8_1	a9_1	a10_1	a11_1	a12_1	a13_1	a14_1	a15_1	a16_1	a17_1	a18_1	ccc1_1	ccc2_1
i24_1	.000	.000	.000	.000	.000	.000	.000	.000	.000	.000	.000	.000	.000	.000	.000	.000	.000	.000	.000	.000
v1_2	.000	.000	.000	.000	.000	.000	.000	.000	.000	.000	.000	.000	.000	.000	.000	.000	.000	.000	.000	.000
v2_2	.000	.000	.000	.000	.000	.000	.000	.000	.000	.000	.000	.000	.000	.000	.000	.000	.000	.000	.000	.000
v3_2	.000	.000	.000	.000	.000	.000	.000	.000	.000	.000	.000	.000	.000	.000	.000	.000	.000	.000	.000	.000
v4_2	.000	.000	.000	.000	.000	.000	.000	.000	.000	.000	.000	.000	.000	.000	.000	.000	.000	.000	.000	.000
v5_2	.000	.000	.000	.002	.003	.002	.000	.000	.000	.000	.000	.000	.000	.000	.000	.000	.000	.000	.000	.000
v6_2	.000	.000	.000	-.003	.000	.000	.000	.000	.000	.000	.000	.000	.000	.000	.000	.000	.000	.000	.000	.000
v7_2	.000	.000	.000	.000	-.004	.000	.000	.000	.000	.000	.000	.000	.000	.000	.000	.000	.000	.000	.000	.000
v8_2	.000	.000	.000	.000	.000	-.003	.000	.000	.000	.000	.000	.000	.000	.000	.000	.000	.000	.000	.000	.000
v9_2	.000	.000	.000	.000	.000	.000	.000	.000	.000	.000	.000	.000	.000	.000	.000	.000	.000	.000	.000	.000
v10_2	.000	.000	.000	.000	.000	.000	.000	.000	.000	.000	.000	.000	.000	.000	.000	.000	.000	.000	.000	.000
v11_2	.000	.000	.000	.000	.000	.000	.000	.000	.000	.000	.000	.000	.000	.000	.000	.000	.000	.000	.000	.000
v12_2	.000	.000	.000	.000	.000	.000	.000	.000	.000	.000	.000	.000	.000	.000	.000	.000	.000	.000	.000	.000
v13_2	.000	.000	.000	.000	.000	.000	.000	.000	.000	.000	.000	.000	.000	.000	.000	.000	.000	.000	.000	.000
v14_2	.000	.000	.000	.000	.000	.000	.000	.000	.000	.000	.000	.000	.000	.000	.000	.000	.000	.000	.000	.000
v15_2	.000	.000	.000	.000	.000	.000	.000	.000	.000	.000	.000	.000	.000	.000	.000	.000	.000	.000	.000	.000
v16_2	.000	.000	.000	.000	.000	.000	.000	.000	.000	.000	.000	.000	.000	.000	.000	.000	.000	.000	.000	.000
v17_2	.000	.000	.000	.000	.000	.000	.000	.000	.000	.000	.000	.000	.000	.000	.000	.000	.000	.000	.000	.000
v18_2	.000	.000	.000	.000	.000	.000	.000	.000	.000	.000	.000	.000	.000	.000	.000	.000	.000	.000	.000	.000
v19_2	.000	.000	.000	.000	.000	.000	.000	.000	.000	.000	.000	.000	.000	.000	.000	.000	.000	.000	.000	.000
v20_2	.000	.000	.000	.000	.000	.000	.000	.000	.000	.000	.000	.000	.000	.000	.000	.000	.000	.000	.000	.000
v21_2	.000	.000	.000	.000	.000	.000	.000	.000	.000	.000	.000	.000	.000	.000	.000	.000	.000	.000	.000	.000
v22_2	.000	.000	.000	.000	.000	.000	.000	.000	.000	.000	.000	.000	.000	.000	.000	.000	.000	.000	.000	.000
v23_2	.000	.000	.000	.000	.000	.000	.000	.000	.000	.000	.000	.000	.000	.000	.000	.000	.000	.000	.000	.000
v24_2	.000	.000	.000	.000	.000	.000	.000	.000	.000	.000	.000	.000	.000	.000	.000	.000	.000	.000	.000	.000
v1_3	.000	.000	.000	.000	.000	.000	.000	.000	.000	.000	.000	.000	.000	.000	.000	.000	.000	.000	.000	.000
v2_3	.000	.000	.000	.000	.000	.000	.000	.000	.000	.000	.000	.000	.000	.000	.000	.000	.000	.000	.000	.000
v3_3	.000	.000	.000	.000	.000	.000	.000	.000	.000	.000	.000	.000	.000	.000	.000	.000	.000	.000	.000	.000
v4_3	.000	.000	.000	.000	.000	.000	.000	.000	.000	.000	.000	.000	.000	.000	.000	.000	.000	.000	.000	.000
v5_3	.000	.000	.000	.002	.003	.002	.000	.000	.000	.000	.000	.000	.000	.000	.000	.000	.000	.000	.000	.000
v6_3	.000	.000	.000	-.002	.000	.000	.000	.000	.000	.000	.000	.000	.000	.000	.000	.000	.000	.000	.000	.000
v7_3	.000	.000	.000	.000	-.004	.000	.000	.000	.000	.000	.000	.000	.000	.000	.000	.000	.000	.000	.000	.000
v8_3	.000	.000	.000	.000	.000	-.002	.000	.000	.000	.000	.000	.000	.000	.000	.000	.000	.000	.000	.000	.000
v9_3	.000	.000	.000	.000	.000	.000	.000	.000	.000	.000	.000	.000	.000	.000	.000	.000	.000	.000	.000	.000
v10_3	.000	.000	.000	.000	.000	.000	.000	.000	.000	.000	.000	.000	.000	.000	.000	.000	.000	.000	.000	.000
v11_3	.000	.000	.000	.000	.000	.000	.000	.000	.000	.000	.000	.000	.000	.000	.000	.000	.000	.000	.000	.000
v12_3	.000	.000	.000	.000	.000	.000	.000	.000	.000	.000	.000	.000	.000	.000	.000	.000	.000	.000	.000	.000
v13_3	.000	.000	.000	.000	.000	.000	.000	.000	.000	.000	.000	.000	.000	.000	.000	.000	.000	.000	.000	.000
v14_3	.000	.000	.000	.000	.000	.000	.000	.000	.000	.000	.000	.000	.000	.000	.000	.000	.000	.000	.000	.000
v15_3	.000	.000	.000	.000	.000	.000	.000	.000	.000	.000	.000	.000	.000	.000	.000	.000	.000	.000	.000	.000
v16_3	.000	.000	.000	.000	.000	.000	.000	.000	.000	.000	.000	.000	.000	.000	.000	.000	.000	.000	.000	.000
v17_3	.000	.000	.000	.000	.000	.000	.000	.000	.000	.000	.000	.000	.000	.000	.000	.000	.000	.000	.000	.000
v18_3	.000	.000	.000	.000	.000	.000	.000	.000	.000	.000	.000	.000	.000	.000	.000	.000	.000	.000	.000	.000
v19_3	.000	.000	.000	.000	.000	.000	.000	.000	.000	.000	.000	.000	.000	.000	.000	.000	.000	.000	.000	.000
v20_3	.000	.000	.000	.000	.000	.000	.000	.000	.000	.000	.000	.000	.000	.000	.000	.000	.000	.000	.000	.000
v21_3	.000	.000	.000	.000	.000	.000	.000	.000	.000	.000	.000	.000	.000	.000	.000	.000	.000	.000	.000	.000
v22_3	.000	.000	.000	.000	.000	.000	.000	.000	.000	.000	.000	.000	.000	.000	.000	.000	.000	.000	.000	.000
v23_3	.000	.000	.000	.000	.000	.000	.000	.000	.000	.000	.000	.000	.000	.000	.000	.000	.000	.000	.000	.000
v24_3	.000	.000	.000	.000	.000	.000	.000	.000	.000	.000	.000	.000	.000	.000	.000	.000	.000	.000	.000	.000

Correlations of Estimates (Structural covariances)

	a1_1	a2_1	a3_1	a4_1	a5_1	a6_1	a7_1	a8_1	a9_1	a10_1	a11_1	a12_1	a13_1	a14_1	a15_1	a16_1	a17_1	a18_1	ccc1_1
a1_1	1.000																		
a2_1	.498	1.000																	
a3_1	.446	.343	1.000																
a4_1	.000	.000	.000	1.000															
a5_1	.000	.000	.000	.894	1.000														
a6_1	.000	.000	.000	.878	.906	1.000													
a7_1	.000	.000	.000	.000	.000	.000	1.000												
a8_1	.000	.000	.000	.000	.000	.000	.631	1.000											
a9_1	.000	.000	.000	.000	.000	.000	.481	.508	1.000										
a10_1	.000	.000	.000	.000	.000	.000	.000	.000	.000	1.000									
a11_1	.000	.000	.000	.000	.000	.000	.000	.000	.000	.590	1.000								
a12_1	.000	.000	.000	.000	.000	.000	.000	.000	.000	.568	.554	1.000							
a13_1	.001	.000	.001	.000	.000	.000	.000	.000	.000	.000	.000	.000	1.000						
a14_1	.000	.000	.000	.000	.000	.000	.000	.000	.000	.000	.000	.000	.554	1.000					
a15_1	.000	.000	.000	.000	.000	.000	.000	.000	.000	.000	.000	.000	.475	.450	1.000				
a16_1	.000	.000	.000	.000	.000	.000	.000	.000	.000	.000	.000	.000	.000	.000	.000	1.000			
a17_1	.000	.000	.000	.000	.000	.000	.000	.000	.000	.000	.000	-.001	.000	.000	.000	.431	1.000		
a18_1	.000	.000	.000	.000	.000	.000	.000	.000	.000	.000	.000	.000	.000	.000	.000	.377	.376	1.000	

	a1_1	a2_1	a3_1	a4_1	a5_1	a6_1	a7_1	a8_1	a9_1	a10_1	a11_1	a12_1	a13_1	a14_1	a15_1	a16_1	a17_1	a18_1	ccc1_1
ccc1_1	.194	.148	.132	.716	.744	.727	.000	.000	.000	.000	.000	.000	.000	.000	.000	.000	.000	.000	1.000
ccc2_1	.000	.000	.000	.722	.750	.733	.165	.176	.131	.000	.000	.000	.000	.000	.000	.000	.000	.000	.839
ccc3_1	-.445	-.338	-.302	.000	.000	.000	-.373	-.400	-.296	.000	.000	.000	.000	.000	.000	.000	.000	.000	-.293
ccc4_1	.000	.000	.000	-.830	-.863	-.843	.000	.000	.000	-.189	-.184	-.177	.000	.000	.000	.000	.000	.000	-.787
ccc5_1	.000	.000	.000	.000	.000	.000	.332	.356	.263	.332	.323	.310	.000	.000	.000	.000	.000	.000	.220
ccc6_1	.333	.253	.226	.000	.000	.000	.000	.000	.000	.280	.272	.261	.000	.000	.000	.000	.000	.000	.409
ccc7_1	.000	.000	.000	.688	.714	.698	.000	.000	.000	.000	.000	.000	.143	.135	.115	.000	.000	.000	.862
ccc8_1	.000	.000	.000	.000	.000	.000	.000	.000	.000	.340	.331	.318	.310	.293	.250	.000	.000	.000	.236
ccc9_1	.000	.000	.000	.000	.000	.000	-.391	-.419	-.310	.000	.000	.000	-.356	-.337	-.288	.000	.000	.000	-.159
ccc10_1	-.476	-.362	-.323	.000	.000	.000	.000	.000	.000	.000	.000	.000	-.364	-.344	-.294	.000	.000	.000	-.285
ccc11_1	.000	.000	.000	.000	.000	.000	.000	.000	.000	.000	.000	.000	.358	.339	.289	.242	.241	.209	.228
ccc12_1	.000	.000	.000	.000	.000	.000	.320	.343	.254	.000	.000	.000	.000	.000	.000	.197	.196	.170	.238
ccc13_1	.000	.000	.000	.000	.000	.000	.000	.000	.000	-.432	-.421	-.404	.000	.000	.000	-.265	-.264	-.229	-.168
ccc14_1	.000	.000	.000	-.839	-.872	-.851	.000	.000	.000	.000	.000	.000	.000	.000	.000	-.117	-.117	-.102	-.802
ccc15_1	.355	.270	.241	.000	.000	.000	.000	.000	.000	.000	.000	.000	.000	.000	.000	.183	.182	.158	.416
vvv1_1	-.699	-.542	-.485	.000	.000	.000	.000	.000	.000	.000	.000	.000	.000	.000	.000	.000	.000	.000	-.323
v1_1	.073	.059	.053	.000	.000	.000	.000	.000	.000	.000	.000	.000	.001	.000	.001	.000	.000	.000	.017
v2_1	-.088	.000	.001	.000	.000	.000	.000	-.001	.000	.000	.000	.000	.001	.000	.002	.000	.000	.000	-.012
v3_1	-.002	-.046	.000	.000	.000	.000	.000	.000	.000	.000	.000	.000	.000	.000	.000	.000	.000	.000	-.003
v4_1	-.001	.000	-.043	.000	.000	.000	.000	.000	.000	.000	.000	.000	.000	.000	.000	.000	.000	.000	-.002
vvv2_1	.000	.000	.000	-.919	-.950	-.932	.000	.000	.000	.000	.000	.000	.000	.000	.000	.000	.000	.000	-.796
v5_1	.000	.000	.000	.049	.051	.050	.000	.000	.000	.000	.000	.000	.000	.000	.000	.000	.000	.000	.039
v6_1	.000	.000	.000	-.065	.002	.001	.000	.000	.000	.000	.000	.000	.000	.000	.000	.000	.000	.000	-.011
v7_1	.000	.000	.000	.004	-.073	.003	.000	.000	.000	.000	.000	.000	.000	.000	.000	.000	.000	.000	-.026
v8_1	.000	.000	.000	.002	.003	-.063	.000	.000	.000	.000	.000	.000	.000	.000	.000	.000	.000	.000	-.013
vvv3_1	.000	.000	.000	.000	.000	.000	-.663	-.704	-.530	.000	.000	.000	.000	.000	.000	.000	.000	.000	-.108
v9_1	.000	.000	.000	.000	.000	.000	.116	.125	.091	.000	.000	.000	.000	.000	.000	.000	.000	.000	.000
v10_1	.000	.000	.000	.000	.000	.000	-.116	.004	-.003	.000	.000	.000	.000	.000	.000	.000	.000	.000	.000
v11_1	.000	.000	.000	.000	.000	.000	.000	-.138	-.005	.000	.000	.000	.000	.000	.000	.000	.000	.000	.000
v12_1	.000	.000	.000	.000	.000	.000	.000	.001	-.080	.000	.000	.000	.000	.000	.000	.000	.000	.000	.000
vvv4_1	.000	.000	.000	.000	.000	.000	.000	.000	.000	-.663	-.647	-.622	.000	.000	.000	.000	.000	.000	-.117
v13_1	.000	.000	.000	.000	.000	.000	.000	.000	.000	.088	.085	.082	.000	.000	.000	.000	.000	.000	.000
v14_1	.000	.000	.000	.000	.000	.000	.000	.000	.000	-.098	.000	-.001	.000	.000	.000	-.001	.000	.000	.000
v15_1	.000	.000	.000	.000	.000	.000	.000	.000	.000	.001	-.089	.000	.000	.000	.000	-.001	.000	.000	.000
v16_1	.000	.000	.000	.000	.000	.000	.000	.000	.000	.001	.000	-.081	.000	.000	.000	.000	.000	.000	.000
vvv5_1	.000	.000	.000	.000	.000	.000	.000	.000	.000	.000	.000	.000	-.635	-.602	-.516	.000	.000	.000	-.114
v17_1	.000	-.001	-.001	.000	.000	.000	.000	.000	.000	.000	.000	.000	.062	.059	.051	.000	.000	.000	.000
v18_1	.000	-.002	-.001	.000	.000	.000	.000	.000	.000	.000	.000	.000	-.074	.000	.002	-.001	.000	.000	.000
v19_1	.000	-.001	-.001	.000	.000	.000	.000	.000	.000	.000	.000	.000	.000	-.061	.001	.000	.000	.000	.000
v20_1	.000	-.001	.000	.000	.000	.000	.000	.000	.000	.000	.000	.000	.000	.000	-.056	.000	.000	.000	.000
vvv6_1	.000	.000	.000	.000	.000	.000	.000	.000	.000	.000	.000	.000	.000	.000	.000	-.504	-.502	-.439	-.146
v21_1	.000	.000	.000	.000	.000	.000	.000	.000	.000	.000	.000	.000	.000	.000	.000	.099	.101	.090	.000
v22_1	.000	.000	.000	.000	.000	.000	.000	.000	.000	.000	.000	.000	.000	.000	.000	-.081	-.001	.000	.000
v23_1	.000	.000	.000	.000	.000	.000	.000	.000	.000	.000	.000	.000	.000	.000	.000	-.003	-.089	-.001	.000
v24_1	.000	.000	.000	.000	.000	.000	.000	.000	.000	.000	.000	.000	.000	.000	.000	-.002	-.001	-.080	.000
i1_1	.000	.000	.000	.000	.000	.000	.000	.000	.000	.000	.000	.000	.000	.000	.000	.000	.000	.000	.000
i2_1	.000	.000	.000	.000	.000	.000	.000	.000	.000	.000	.000	.000	.000	.000	.000	.000	.000	.000	.000
i3_1	.000	.000	.000	.000	.000	.000	.000	.000	.000	.000	.000	.000	.000	.000	.000	.000	.000	.000	.000
i4_1	.000	.000	.000	.000	.000	.000	.000	.000	.000	.000	.000	.000	.000	.000	.000	.000	.000	.000	.000
i5_1	.000	.000	.000	.000	.000	.000	.000	.000	.000	.000	.000	.000	.000	.000	.000	.000	.000	.000	.000
i6_1	.000	.000	.000	.000	.000	.000	.000	.000	.000	.000	.000	.000	.000	.000	.000	.000	.000	.000	.000
i7_1	.000	.000	.000	.000	.000	.000	.000	.000	.000	.000	.000	.000	.000	.000	.000	.000	.000	.000	.000
i8_1	.000	.000	.000	.000	.000	.000	.000	.000	.000	.000	.000	.000	.000	.000	.000	.000	.000	.000	.000
i9_1	.000	.000	.000	.000	.000	.000	.000	.000	.000	.000	.000	.000	.000	.000	.000	.000	.000	.000	.000
i10_1	.000	.000	.000	.000	.000	.000	.000	.000	.000	.000	.000	.000	.000	.000	.000	.000	.000	.000	.000
i11_1	.000	.000	.000	.000	.000	.000	.000	.000	.000	.000	.000	.000	.000	.000	.000	.000	.000	.000	.000
i12_1	.000	.000	.000	.000	.000	.000	.000	.000	.000	.000	.000	.000	.000	.000	.000	.000	.000	.000	.000
i13_1	.000	.000	.000	.000	.000	.000	.000	.000	.000	.000	.000	.000	.000	.000	.000	.000	.000	.000	.000
i14_1	.000	.000	.000	.000	.000	.000	.000	.000	.000	.000	.000	.000	.000	.000	.000	.000	.000	.000	.000
i15_1	.000	.000	.000	.000	.000	.000	.000	.000	.000	.000	.000	.000	.000	.000	.000	.000	.000	.000	.000
i16_1	.000	.000	.000	.000	.000	.000	.000	.000	.000	.000	.000	.000	.000	.000	.000	.000	.000	.000	.000
i17_1	.000	.000	.000	.000	.000	.000	.000	.000	.000	.000	.000	.000	.000	.000	.000	.000	.000	.000	.000
i18_1	.000	.000	.000	.000	.000	.000	.000	.000	.000	.000	.000	.000	.000	.000	.000	.000	.000	.000	.000
i19_1	.000	.000	.000	.000	.000	.000	.000	.000	.000	.000	.000	.000	.000	.000	.000	.000	.000	.000	.000
i20_1	.000	.000	.000	.000	.000	.000	.000	.000	.000	.000	.000	.000	.000	.000	.000	.000	.000	.000	.000
i21_1	.000	.000	.000	.000	.000	.000	.000	.000	.000	.000	.000	.000	.000	.000	.000	.000	.000	.000	.000
i22_1	.000	.000	.000	.000	.000	.000	.000	.000	.000	.000	.000	.000	.000	.000	.000	.000	.000	.000	.000
i23_1	.000	.000	.000	.000	.000	.000	.000	.000	.000	.000	.000	.000	.000	.000	.000	.000	.000	.000	.000
i24_1	.000	.000	.000	.000	.000	.000	.000	.000	.000	.000	.000	.000	.000	.000	.000	.000	.000	.000	.000
v1_2	.061	.048	.044	.000	.000	.000	.000	.000	.000	.000	.000	.000	.001	.000	.000	.000	.000	.000	.014
v2_2	-.096	.005	.006	.000	.000	.000	.000	.000	.000	.000	.000	.000	.001	.000	.000	.000	.000	.000	-.013
v3_2	.000	-.058	.002	.000	.000	.000	.000	.000	.000	.000	.000	.000	.000	.000	.000	.000	.000	.000	-.003

	a1_1	a2_1	a3_1	a4_1	a5_1	a6_1	a7_1	a8_1	a9_1	a10_1	a11_1	a12_1	a13_1	a14_1	a15_1	a16_1	a17_1	a18_1	ccc1_1
**v4_2**	.000	.001	-.056	.000	.000	.000	.000	.000	.000	.000	.000	.000	.000	.000	.000	.000	.000	.000	-.003
**v5_2**	.000	.000	.000	.049	.051	.050	.000	.000	.000	.000	.000	.000	.000	.000	.000	.000	.000	.000	.040
**v6_2**	.000	.000	.000	-.057	.002	.002	.000	.000	.000	.000	.000	.000	.000	.000	.000	.000	.000	.000	-.009
**v7_2**	.001	.000	.000	.002	-.076	.005	.000	.000	.000	.000	.000	.000	.000	.000	.000	.000	-.001	.000	-.028
**v8_2**	.000	.000	.000	.001	.004	-.067	.000	.000	.000	.000	.000	.000	.000	.000	.000	.000	.000	.000	-.014
**v9_2**	.000	.000	.000	.000	.000	.000	.088	.099	.069	.000	.000	.000	.000	.000	.000	.000	.000	.000	.000
**v10_2**	.001	.000	.000	.000	.000	.000	-.103	.014	.000	.000	.000	.000	.000	.000	.000	.000	.000	.000	.000
**v11_2**	.001	.001	.001	.000	.000	.000	.008	-.141	-.001	.000	.000	.000	.000	.000	.000	.000	.000	.000	.000
**v12_2**	.000	.000	.000	.000	.000	.000	.001	.004	-.067	.000	.000	.000	.000	.000	.000	.000	.000	.000	.000
**v13_2**	.000	.000	.000	.000	.000	.000	.000	.000	.000	.078	.075	.074	.000	.000	.000	.000	.000	.000	.000
**v14_2**	.000	.000	.000	.000	.000	.000	.000	.000	.000	-.093	.000	.004	.000	.000	.000	.001	-.001	.000	.000
**v15_2**	.000	.000	.000	.000	.000	.000	.000	.000	.000	.002	-.076	.003	.000	.000	.000	.000	-.001	.000	.000
**v16_2**	.000	.000	.000	.000	.000	.000	.000	.000	.000	.002	.000	-.090	.000	.000	.000	.000	.000	.000	.000
**v17_2**	.002	.001	.002	.000	.000	.000	.000	.000	.000	.000	.000	.000	.063	.059	.051	.000	.000	.000	.001
**v18_2**	.004	.002	.003	.000	.000	.000	.000	.000	.000	.000	.000	.000	-.074	.000	.000	.000	-.001	.000	.001
**v19_2**	.003	.002	.002	.000	.000	.000	.000	.000	.000	.000	.000	.000	.000	-.059	.000	.000	.000	.000	.001
**v20_2**	.001	.001	.001	.000	.000	.000	.000	.000	.000	.000	.000	.000	.000	.000	-.047	.000	.000	.000	.000
**v21_2**	.000	.000	.000	.000	.000	.000	.000	.000	.000	.000	.000	.001	.000	.000	.000	.094	.088	.078	.000
**v22_2**	.000	.000	.000	.000	.000	.000	.000	.000	.000	.000	.000	.001	.000	.000	.000	-.095	-.003	-.002	.000
**v23_2**	.000	.000	.000	.000	.000	.000	.000	.000	.000	.000	.000	.001	.000	.000	.000	.001	-.071	-.001	.000
**v24_2**	.000	.000	.000	.000	.000	.000	.000	.000	.000	.000	.000	.000	.000	.000	.000	.001	-.001	-.063	.000
**v1_3**	.079	.065	.058	.000	.000	.000	.000	.000	.000	.000	.000	.000	-.002	.000	-.001	.000	.000	.000	.018
**v2_3**	-.078	.001	.000	.000	.000	.000	.000	.000	.000	.000	.000	.000	-.003	.000	-.001	.000	.000	.000	-.011
**v3_3**	-.003	-.058	.000	.000	.000	.000	.000	.000	.000	.000	.000	.000	-.001	.000	.000	.000	.000	.000	-.004
**v4_3**	-.002	.000	-.042	.000	.000	.000	.000	.000	.000	.000	.000	.000	-.001	.000	.000	.000	.000	.000	-.002
**v5_3**	.000	.000	.000	.050	.053	.051	.000	.000	.000	.000	.000	.000	.000	.000	.000	.000	.000	.000	.041
**v6_3**	.000	.000	.000	-.051	.005	.000	.000	.000	.000	.000	.000	.000	.000	.000	.000	.000	.000	.000	-.007
**v7_3**	-.001	.000	.000	.000	-.092	.002	.000	.000	.000	.000	.000	.000	-.001	.000	.000	.000	.001	.000	-.034
**v8_3**	.000	.000	.000	.000	.007	-.057	.000	.000	.000	.000	.000	.000	.000	.000	.000	.000	.000	.000	-.010
**v9_3**	.000	.000	.000	.000	.000	.000	.092	.101	.072	.000	.000	.000	.000	.000	.000	.000	.000	.000	.000
**v10_3**	.000	.000	.000	.000	.000	.000	-.111	.012	.000	.000	.000	.000	.000	.000	.000	.000	.000	.000	.000
**v11_3**	-.001	.000	-.001	.000	.000	.000	.010	-.136	.000	.000	.000	.000	.000	.000	.000	.000	.000	.000	.000
**v12_3**	.000	.000	.000	.000	.000	.000	.002	.004	-.071	.000	.000	.000	.000	.000	.000	.000	.000	.000	.000
**v13_3**	.000	.000	.000	.000	.000	.000	.000	.000	.000	.081	.081	.076	.000	.000	.000	.000	.001	.000	.000
**v14_3**	.000	.000	.000	.000	.000	.000	.000	.000	.000	-.081	.002	-.002	.000	.000	.000	.000	.001	.000	.000
**v15_3**	.000	.000	.000	.000	.000	.000	.000	.000	.000	-.001	-.093	-.002	.000	.000	.000	.000	.001	.000	.000
**v16_3**	.000	.000	.000	.000	.000	.000	.000	.000	.000	-.001	.001	-.067	.000	.000	.000	.000	.001	.000	.000
**v17_3**	-.002	.000	-.001	.000	.000	.000	.000	.000	.000	.000	.000	.000	.071	.069	.059	.000	.001	.000	.000
**v18_3**	-.003	-.001	-.002	.000	.000	.000	.000	.000	.000	.000	.000	.000	-.063	.000	-.001	.000	.001	.000	.000
**v19_3**	-.003	.000	-.001	.000	.000	.000	.000	.000	.000	.000	.000	.000	-.002	-.070	-.001	.000	.001	.000	.000
**v20_3**	-.001	.000	-.001	.000	.000	.000	.000	.000	.000	.000	.000	.000	-.001	.000	-.048	.000	.000	.000	.000
**v21_3**	.000	.000	.000	.000	.000	.000	.000	.000	.000	.000	.000	-.001	-.001	.000	.000	.084	.087	.072	.000
**v22_3**	.000	.000	.000	.000	.000	.000	.000	.000	.000	.000	.000	.000	-.001	.000	.000	-.082	.003	.000	.000
**v23_3**	.000	.000	.000	.000	.000	.000	.000	.000	.000	.000	.000	.000	-.001	.000	.000	.001	-.097	.000	.000
**v24_3**	.000	.000	.000	.000	.000	.000	.000	.000	.000	.000	.000	.000	.000	.000	.000	.000	.002	-.066	.000

Critical Ratios for Differences between Parameters (Structural covariances)

	a1_1	a2_1	a3_1	a4_1	a5_1	a6_1	a7_1	a8_1	a9_1	a10_1	a11_1	a12_1	a13_1	a14_1	a15_1	
**a1_1**	.000															
**a2_1**	-5.530	.000														
**a3_1**	-6.988	-1.601	.000													
**a4_1**	4.252	5.151	5.445	.000												
**a5_1**	5.176	5.918	6.160	3.707	.000											
**a6_1**	4.450	5.329	5.616	.621	-3.335	.000										
**a7_1**	-.098	4.021	5.372	-4.284	-5.202	-4.482	.000									
**a8_1**	1.247	5.331	6.667	-3.981	-4.953	-4.186	2.331	.000								
**a9_1**	-4.267	-.086	1.303	-5.186	-5.946	-5.363	-6.123	-8.265	.000							
**a10_1**	.833	4.867	6.188	-4.069	-5.025	-4.273	.978	-.416	5.305	.000						
**a11_1**	.880	4.859	6.161	-4.053	-5.012	-4.257	1.024	-.350	5.284	.092	.000					
**a12_1**	-.227	3.796	5.117	-4.309	-5.223	-4.507	-.139	-1.520	4.157	-1.660	-1.698	.000				
**a13_1**	-.646	3.638	5.048	-4.408	-5.305	-4.603	-.578	-2.049	4.035	-1.583	-1.620	-.417	.000			
**a14_1**	-1.473	2.797	4.204	-4.584	-5.450	-4.775	-1.456	-2.913	3.124	-2.436	-2.460	-1.272	-1.382	.000		
**a15_1**	-3.860	.394	1.805	-5.089	-5.866	-5.269	-3.989	-5.414	.522	-4.903	-4.887	-3.736	-4.946	-3.580	.000	
**a16_1**	-2.603	1.973	3.487	-4.800	-5.628	-4.987	-2.673	-4.226	2.269	-3.688	-3.687	-2.433	-2.170	-1.159	1.742	
**a17_1**	-2.893	1.700	3.222	-4.856	-5.674	-5.041	-2.985	-4.540	1.973	-3.994	-3.988	-2.732	-2.499	-1.482	1.435	
**a18_1**	-4.414	.116	1.622	-5.163	-5.926	-5.341	-4.610	-6.124	.229	-5.560	-5.525	-4.311	-4.225	-3.218	-.343	[illegible]
**ccc1_1**	-24.747	-19.352	-17.491	-8.961	-9.069	-9.060	-24.719	-25.964	-20.332	-24.965	-24.478	-23.602	-25.857	-24.694	-21.585	[illegible]
**ccc2_1**	-22.930	-18.262	-16.607	-8.919	-9.033	-9.018	-26.628	-28.079	-21.615	-25.081	-24.582	-23.706	-26.029	-24.853	-21.709	[illegible]
**ccc3_1**	-4.817	-1.575	-.439	-5.561	-6.256	-5.729	-5.120	-6.175	-1.641	-6.812	-6.774	-5.721	-5.698	-4.838	-2.390	[illegible]
**ccc4_1**	-12.502	-8.342	-6.929	-6.261	-6.761	-6.390	-13.175	-14.471	-8.958	-12.702	-12.583	-11.689	-13.246	-12.303	-9.672	[illegible]

	a1_1	a2_1	a3_1	a4_1	a5_1	a6_1	a7_1	a8_1	a9_1	a10_1	a11_1	a12_1	a13_1	a14_1	a15_1	[illegible]
**ccc5_1**	-25.481	-21.624	-20.268	-9.805	-9.754	-9.872	-32.844	-34.763	-27.069	-33.177	-32.516	-31.311	-27.558	-26.645	-24.179	[illegible]
**ccc6_1**	-29.508	-23.414	-21.449	-9.470	-9.477	-9.545	-25.519	-26.613	-21.818	-30.407	-29.838	-28.717	-26.180	-25.253	-22.750	[illegible]
**ccc7_1**	-22.795	-18.128	-16.476	-8.876	-8.997	-8.976	-24.695	-25.945	-20.274	-24.935	-24.441	-23.562	-27.552	-26.204	-22.668	[illegible]
**ccc8_1**	-25.500	-21.782	-20.481	-10.014	-9.929	-10.076	-26.760	-27.797	-23.267	-33.322	-32.705	-31.476	-32.870	-31.452	-27.832	[illegible]
**ccc9_1**	-5.180	-1.223	.092	-5.428	-6.146	-5.599	-4.543	-5.598	-1.071	-6.183	-6.155	-5.096	-4.324	-3.613	-1.531	[illegible]
**ccc10_1**	-2.533	.543	1.616	-4.984	-5.780	-5.165	-3.123	-4.367	.752	-3.944	-3.951	-2.934	-2.343	-1.678	.276	[illegible]
**ccc11_1**	-26.426	-23.093	-21.934	-10.869	-10.645	-10.912	-27.441	-28.387	-24.308	-27.770	-27.514	-26.816	-34.215	-32.841	-29.331	[illegible]
**ccc12_1**	-24.900	-21.208	-19.917	-9.924	-9.855	-9.989	-31.647	-33.493	-26.154	-26.435	-26.129	-25.371	-26.664	-25.806	-23.471	[illegible]
**ccc13_1**	1.451	4.649	5.694	-3.790	-4.793	-3.998	1.577	.482	4.918	.678	.634	1.418	2.049	2.703	4.591	[illegible]
**ccc14_1**	-10.855	-6.945	-5.627	-5.980	-6.515	-6.111	-11.340	-12.581	-7.364	-12.024	-11.913	-10.935	-11.276	-10.410	-7.981	[illegible]
**ccc15_1**	-30.039	-23.988	-22.076	-9.854	-9.799	-9.921	-25.235	-26.268	-21.794	-25.591	-25.310	-24.549	-25.720	-24.882	-22.602	[illegible]
**vvv1_1**	-2.402	.072	.945	-5.059	-5.844	-5.238	-3.121	-4.187	.160	-3.832	-3.844	-2.968	-2.768	-2.108	-.216	[illegible]
**v1_1**	.739	3.536	4.442	-3.908	-4.889	-4.112	.799	-.128	3.611	.149	.108	.882	1.178	1.730	3.321	[illegible]
**v2_1**	-3.096	-.002	1.061	-5.088	-5.869	-5.267	-3.253	-4.329	.066	-3.969	-3.980	-3.095	-2.903	-2.232	-.316	[illegible]
**v3_1**	4.622	6.634	7.427	-2.134	-3.392	-2.371	4.740	4.018	6.904	4.218	4.171	4.781	5.067	5.485	6.693	[illegible]
**v4_1**	5.165	7.204	7.756	-1.825	-3.128	-2.068	5.283	4.586	7.366	4.778	4.731	5.320	5.600	6.002	7.164	[illegible]
**vvv2_1**	-15.787	-11.460	-9.972	-6.775	-7.194	-6.893	-16.780	-18.078	-12.463	-17.342	-17.093	-16.110	-17.115	-16.105	-13.319	[illegible]
**v5_1**	6.324	8.175	8.777	-1.080	-2.511	-1.340	6.435	5.802	8.325	5.974	5.930	6.466	6.727	7.091	8.142	[illegible]
**v6_1**	3.552	5.629	6.306	-2.481	-3.732	-2.767	3.651	2.944	5.770	3.144	3.102	3.697	3.959	4.371	5.557	[illegible]
**v7_1**	2.265	4.304	4.970	-3.045	-4.063	-3.262	2.344	1.653	4.419	1.854	1.817	2.398	2.634	3.039	4.204	[illegible]
**v8_1**	2.862	5.019	5.723	-2.884	-4.026	-3.041	2.956	2.222	5.159	2.432	2.391	3.008	3.271	3.700	4.935	[illegible]
**vvv3_1**	-3.102	.285	1.403	-5.032	-5.821	-5.212	-2.459	-3.304	.305	-3.889	-3.899	-2.968	-2.765	-2.056	-.029	[illegible]
**v9_1**	-4.264	-.798	.349	-5.318	-6.057	-5.491	-4.611	-5.866	-.801	-5.098	-5.095	-4.153	-4.007	-3.277	-1.193	[illegible]
**v10_1**	-4.944	-1.475	-.326	-5.490	-6.200	-5.659	-4.797	-6.218	-1.478	-5.795	-5.784	-4.848	-4.738	-4.005	-1.917	[illegible]
**v11_1**	-6.398	-2.835	-1.649	-5.824	-6.475	-5.985	-6.584	-7.279	-2.914	-7.314	-7.284	-6.337	-6.313	-5.552	-3.393	[illegible]
**v12_1**	-1.546	1.602	2.637	-4.629	-5.488	-4.818	-1.518	-2.582	1.679	-2.242	-2.269	-1.391	-1.130	-.481	1.382	
**vvv4_1**	.044	2.898	3.833	-4.143	-5.085	-4.342	.115	-.853	3.057	-.446	-.477	.168	.499	1.079	2.748	
**v13_1**	.003	2.814	3.735	-4.149	-5.090	-4.348	.072	-.880	2.966	-.614	-.652	.171	.449	1.020	2.661	
**v14_1**	-1.182	1.679	2.619	-4.522	-5.400	-4.713	-1.143	-2.107	1.798	-1.732	-1.834	-1.033	-.782	-.198	1.477	
**v15_1**	.704	3.286	4.131	-3.875	-4.861	-4.079	.782	-.094	3.431	.168	.125	.860	1.137	1.658	3.154	[illegible]
**v16_1**	1.405	3.928	4.752	-3.616	-4.644	-3.825	1.495	.638	4.084	.890	.848	1.518	1.852	2.359	3.818	[illegible]
**vvv5_1**	-1.159	2.000	3.038	-4.520	-5.398	-4.712	-1.119	-2.187	2.153	-1.850	-1.881	-.997	-.578	-.056	1.498	[illegible]
**v17_1**	-.764	2.231	3.215	-4.401	-5.299	-4.595	-.712	-1.726	2.380	-1.410	-1.443	-.603	-.334	.295	2.094	
**v18_1**	-4.036	-.613	.519	-5.268	-6.017	-5.443	-4.108	-5.248	-.572	-4.853	-4.854	-3.922	-3.642	-3.044	-.991	[illegible]
**v19_1**	-1.444	1.637	2.650	-4.601	-5.465	-4.791	-1.413	-2.452	1.770	-2.123	-2.150	-1.290	-1.031	-.386	1.424	
**v20_1**	.016	2.933	3.889	-4.160	-5.099	-4.359	.088	-.901	3.101	-.600	-.639	.185	.481	1.076	2.724	
**vvv6_1**	4.856	7.335	8.141	-2.377	-3.608	-2.613	5.010	4.157	7.570	4.389	4.330	5.049	5.410	5.903	7.330	[illegible]
**v21_1**	-2.945	.198	1.235	-5.023	-5.814	-5.202	-2.962	-4.015	.274	-3.666	-3.680	-2.814	-2.611	-1.959	-.096	[illegible]
**v22_1**	1.474	3.989	4.811	-3.589	-4.622	-3.799	1.565	.710	4.145	.961	.919	1.634	1.921	2.426	3.881	[illegible]
**v23_1**	.188	2.915	3.808	-4.077	-5.029	-4.277	.260	-.664	3.062	-.385	-.422	.348	.628	1.180	2.768	
**v24_1**	1.667	4.217	5.050	-3.535	-4.577	-3.747	1.764	.896	4.384	1.150	1.106	1.832	2.129	2.642	4.117	[illegible]
**i1_1**	61.462	66.696	68.181	8.610	5.421	8.104	65.464	62.956	72.552	62.563	61.464	63.796	69.532	70.427	73.473	[illegible]
**i2_1**	60.911	66.228	67.735	8.319	5.178	7.819	65.004	62.446	72.240	62.041	60.919	63.293	69.181	70.092	73.198	[illegible]
**i3_1**	56.428	61.586	63.064	7.565	4.560	7.084	60.104	57.681	66.989	57.360	56.349	58.588	63.882	64.781	67.796	[illegible]
**i4_1**	54.354	59.447	60.915	7.194	4.255	6.721	57.855	55.487	64.597	55.201	54.237	56.420	61.468	62.362	65.342	[illegible]
**i5_1**	48.272	53.272	54.732	5.925	3.209	5.483	51.384	49.119	57.882	48.918	48.059	50.130	54.652	55.548	58.490	[illegible]
**i6_1**	37.382	41.993	43.379	3.972	1.604	3.577	39.598	37.635	45.299	37.609	36.990	38.756	42.018	42.877	45.595	[illegible]
**i7_1**	29.555	34.065	35.443	2.251	.185	1.897	31.328	29.479	36.760	29.550	29.051	30.692	33.362	34.224	36.904	[illegible]
**i8_1**	38.425	43.150	44.564	4.048	1.665	3.651	40.794	38.762	46.691	38.712	38.052	39.883	43.370	44.247	47.039	[illegible]
**i9_1**	64.176	69.629	71.162	8.820	5.589	8.308	68.611	65.940	76.147	65.467	64.253	66.741	73.122	74.046	77.233	[illegible]
**i10_1**	62.093	67.475	68.995	8.473	5.304	7.969	66.329	63.722	73.702	63.289	62.128	64.552	70.650	71.568	74.714	[illegible]
**i11_1**	63.432	68.807	70.321	8.799	5.574	8.288	67.719	65.106	75.095	64.659	63.485	65.918	72.076	72.989	76.123	[illegible]
**i12_1**	62.927	68.411	69.954	8.474	5.303	7.970	67.348	64.668	74.927	64.201	62.992	65.485	71.865	72.797	76.012	[illegible]
**i13_1**	29.559	34.211	35.631	2.073	.037	1.723	31.440	29.518	37.096	29.580	29.052	30.757	33.602	34.494	37.276	[illegible]
**i14_1**	19.823	24.195	25.563	.117	-1.574	-.186	21.039	19.337	26.124	19.529	19.180	20.660	22.588	23.453	26.080	[illegible]
**i15_1**	23.789	28.144	29.494	1.068	-.788	.743	25.186	23.459	30.303	23.605	23.207	24.721	26.877	27.726	30.322	[illegible]
**i16_1**	17.962	22.296	23.658	-.282	-1.902	-.575	19.066	17.397	24.068	17.612	17.294	18.739	20.515	21.379	23.986	[illegible]
**i17_1**	62.462	67.751	69.247	8.738	5.525	8.229	66.585	64.034	73.787	63.618	62.487	64.860	70.772	71.674	74.753	[illegible]
**i18_1**	66.307	71.631	73.124	9.584	6.222	9.054	70.645	68.045	77.958	67.579	66.390	68.820	75.017	75.913	78.999	[illegible]
**i19_1**	69.507	74.867	76.362	10.272	6.789	9.726	74.036	71.389	81.455	70.881	69.640	72.122	78.574	79.465	82.562	[illegible]
**i20_1**	68.579	73.939	75.436	10.050	6.606	9.509	73.068	70.428	80.476	69.930	68.699	71.173	77.575	78.469	81.570	[illegible]
**i21_1**	16.061	20.416	21.790	-.737	-2.278	-1.020	17.080	15.418	22.082	15.657	15.362	16.793	18.463	19.338	21.968	[illegible]
**i22_1**	20.626	24.966	26.321	.337	-1.392	.028	21.864	20.170	26.913	20.353	19.998	21.474	23.422	24.278	26.877	[illegible]
**i23_1**	23.186	27.591	28.958	.871	-.952	.550	24.585	22.839	29.767	22.990	22.592	24.121	26.289	27.151	29.785	[illegible]
**i24_1**	20.535	24.890	26.251	.299	-1.423	-.008	21.777	20.076	26.848	20.260	19.904	21.385	23.342	24.202	26.813	[illegible]
**v1_2**	3.286	5.622	6.377	-2.854	-4.004	-3.079	3.320	2.532	5.688	2.755	2.709	3.371	3.666	4.125	5.451	[illegible]
**v2_2**	-4.217	-1.043	.074	-5.377	-6.107	-5.549	-4.492	-5.613	-1.021	-5.220	-5.217	-4.303	-4.165	-3.454	-1.437	[illegible]
**v3_2**	2.318	4.726	5.659	-3.295	-4.376	-3.512	2.426	1.568	5.013	1.815	1.768	2.488	2.796	3.300	4.753	[illegible]
**v4_2**	2.554	5.060	5.748	-3.207	-4.303	-3.426	2.665	1.811	5.241	2.055	2.008	2.725	3.036	3.538	4.984	[illegible]
**v5_2**	6.384	8.216	8.811	-1.021	-2.459	-1.281	6.494	5.867	8.364	6.038	5.994	6.525	6.783	7.143	8.182	[illegible]
**v6_2**	4.896	6.746	7.348	-1.669	-3.017	-1.948	4.993	4.362	6.877	4.538	4.498	5.030	5.273	5.638	6.689	[illegible]
**v7_2**	1.914	3.981	4.657	-3.207	-4.196	-3.425	1.989	1.289	4.093	1.493	1.458	2.046	2.280	2.691	3.873	[illegible]

	a1_1	a2_1	a3_1	a4_1	a5_1	a6_1	a7_1	a8_1	a9_1	a10_1	a11_1	a12_1	a13_1	a14_1	a15_1
**v8_2**	2.449	4.656	5.377	-3.079	-4.193	-3.227	2.540	1.790	4.794	2.006	1.966	2.596	2.859	3.298	4.564
**v9_2**	-.352	2.519	3.460	-4.267	-5.188	-4.464	-.302	-1.320	2.745	-.964	-.999	-.191	.088	.673	2.353
**v10_2**	-3.309	-.093	.969	-5.113	-5.889	-5.291	-3.194	-4.445	-.029	-4.055	-4.065	-3.183	-2.992	-2.323	-.411
**v11_2**	-6.420	-2.909	-1.742	-5.847	-6.495	-6.009	-6.619	-7.264	-2.992	-7.316	-7.287	-6.355	-6.325	-5.578	-3.458
**v12_2**	1.102	3.808	4.694	-3.776	-4.779	-3.983	1.194	.273	3.881	.545	.502	1.271	1.575	2.121	3.695
**v13_2**	2.202	4.630	5.422	-3.294	-4.374	-3.510	2.302	1.475	4.792	1.768	1.721	2.433	2.654	3.140	4.540
**v14_2**	.178	2.798	3.656	-4.061	-5.016	-4.261	.246	-.640	2.933	-.359	-.408	.332	.598	1.128	2.648
**v15_2**	2.967	5.159	5.873	-2.865	-4.010	-3.088	3.065	2.319	5.305	2.533	2.424	3.120	3.387	3.823	5.078
**v16_2**	1.093	3.626	4.455	-3.728	-4.737	-3.935	1.177	.317	3.775	.572	.531	1.208	1.531	2.040	3.506
**v17_2**	-.438	2.496	3.459	-4.299	-5.215	-4.495	-.378	-1.371	2.647	-1.065	-1.100	-.275	.008	.621	2.375
**v18_2**	-3.644	-.285	.824	-5.178	-5.942	-5.354	-3.689	-4.808	-.227	-4.426	-4.432	-3.515	-3.230	-2.635	-.631
**v19_2**	-.492	2.433	3.393	-4.314	-5.227	-4.510	-.433	-1.422	2.581	-1.117	-1.152	-.330	-.050	.534	2.260
**v20_2**	2.333	4.853	5.676	-3.293	-4.374	-3.509	2.439	1.580	5.030	1.827	1.781	2.501	2.810	3.314	4.692
**v21_2**	-.595	2.165	3.070	-4.332	-5.242	-4.527	-.542	-1.474	2.292	-1.186	-1.219	-.444	-.184	.377	1.987
**v22_2**	.813	3.383	4.224	-3.835	-4.827	-4.040	.892	.021	3.529	.281	.241	.970	1.248	1.765	3.254
**v23_2**	3.486	5.644	6.347	-2.633	-3.814	-2.861	3.589	2.854	5.795	3.062	3.018	3.638	3.912	4.340	5.574
**v24_2**	4.986	6.937	7.572	-1.796	-3.099	-2.038	5.092	4.427	7.083	4.611	4.567	5.131	5.392	5.777	6.887
**v1_3**	.169	3.039	3.966	-4.096	-5.046	-4.296	.235	-.710	3.107	-.424	-.462	.326	.612	1.178	2.805
**v2_3**	-1.169	1.705	2.661	-4.531	-5.408	-4.722	-1.171	-2.154	1.827	-1.845	-1.874	-1.059	-.804	-.209	1.500
**v3_3**	2.689	5.011	5.915	-3.119	-4.228	-3.339	2.801	1.972	5.297	2.209	2.163	2.859	3.160	3.647	5.047
**v4_3**	5.583	7.515	8.034	-1.506	-2.852	-1.753	5.697	5.038	7.667	5.218	5.174	5.731	5.997	6.378	7.474
**v5_3**	5.908	7.816	8.436	-1.360	-2.753	-1.615	6.021	5.368	7.970	5.546	5.501	6.054	6.320	6.696	7.780
**v6_3**	5.583	7.310	7.872	-1.175	-2.578	-1.448	5.677	5.088	7.433	5.251	5.213	5.710	5.940	6.280	7.258
**v7_3**	-.117	2.188	2.942	-4.099	-4.924	-4.299	-.063	-.840	2.283	-.604	-.635	.014	.240	.703	2.030
**v8_3**	3.721	5.729	6.383	-2.402	-3.621	-2.581	3.817	3.134	5.864	3.327	3.286	3.862	4.115	4.513	5.657
**v9_3**	-.904	2.041	3.008	-4.440	-5.332	-4.633	-.894	-1.939	2.246	-1.542	-1.574	-.748	-.480	.122	1.851
**v10_3**	-4.261	-.927	.177	-5.346	-6.081	-5.519	-4.127	-5.478	-.901	-5.060	-5.060	-4.153	-4.004	-3.306	-1.310
**v11_3**	-6.144	-2.668	-1.512	-5.790	-6.448	-5.953	-6.339	-7.008	-2.738	-7.029	-7.006	-6.077	-6.030	-5.293	-3.193
**v12_3**	.312	3.136	4.060	-4.055	-5.011	-4.256	.389	-.570	3.210	-.280	-.320	.479	.774	1.347	2.996
**v13_3**	1.797	4.268	5.075	-3.459	-4.512	-3.671	1.892	1.051	4.427	1.339	1.295	2.018	2.246	2.741	4.168
**v14_3**	1.831	4.178	4.945	-3.390	-4.453	-3.603	1.920	1.123	4.321	1.316	1.316	1.982	2.255	2.724	4.074
**v15_3**	1.344	3.760	4.550	-3.598	-4.628	-3.807	1.428	.608	3.901	.849	.782	1.495	1.767	2.251	3.645
**v16_3**	4.201	6.209	6.863	-2.199	-3.442	-2.434	4.302	3.618	6.352	3.809	3.769	4.253	4.605	5.002	6.147
**v17_3**	-1.893	1.242	2.273	-4.728	-5.570	-4.914	-1.878	-2.935	1.361	-2.594	-2.618	-1.746	-1.548	-.879	1.030
**v18_3**	-1.405	1.609	2.599	-4.591	-5.457	-4.780	-1.374	-2.392	1.736	-2.069	-2.097	-1.255	-.974	-.380	1.396
**v19_3**	-2.168	.988	2.026	-4.804	-5.633	-4.989	-2.163	-3.225	1.098	-2.880	-2.901	-2.026	-1.788	-1.103	.734
**v20_3**	2.318	4.822	5.637	-3.289	-4.371	-3.506	2.426	1.573	4.998	1.818	1.772	2.488	2.792	3.294	4.661
**v21_3**	.064	2.722	3.593	-4.106	-5.054	-4.305	.130	-.770	2.858	-.497	-.533	.217	.486	1.024	2.569
**v22_3**	1.944	4.331	5.111	-3.368	-4.435	-3.582	2.037	1.226	4.482	1.462	1.420	2.100	2.379	2.857	4.232
**v23_3**	-.116	2.601	3.491	-4.174	-5.110	-4.372	-.052	-.971	2.739	-.691	-.727	.039	.309	.860	2.442
**v24_3**	4.242	6.311	6.984	-2.245	-3.484	-2.479	4.349	3.643	6.463	3.840	3.796	4.391	4.662	5.072	6.253

Bootstrap (Structural covariances)

Summary of Bootstrap Iterations (Structural covariances)

(Structural covariances)

Iterations	Method 0	Method 1	Method 2
**1**	0	0	0
**2**	0	0	0
**3**	0	0	0
**4**	0	0	0
**5**	0	0	0
**6**	0	0	0
**7**	0	1	0
**8**	0	8	0
**9**	0	15	0
**10**	0	25	0
**11**	0	29	0
**12**	0	22	0
**13**	0	25	0
**14**	0	21	0
**15**	0	15	0
**16**	0	7	0
**17**	0	7	0
**18**	0	5	0
**19**	0	20	0
**Total**	0	200	0

0 bootstrap samples were unused because of a singular covariance matrix.
0 bootstrap samples were unused because a solution was not found.

200 usable bootstrap samples were obtained.

Bootstrap Distributions (Structural covariances)

ML discrepancy (implied vs sample) (Structural covariances)

```
                          |--------------------
              3248.718    |*
              3319.124    |**
              3389.530    |***
              3459.936    |******
              3530.341    |*************
              3600.747    |**************
              3671.153    |***************
N = 200       3741.559    |*****************
Mean = 3694.437  3811.964 |************
S. e. = 12.344   3882.370 |********
              3952.776    |*****
              4023.182    |****
              4093.587    |*
              4163.993    |*
              4234.399    |*
                          |--------------------
```

ML discrepancy (implied vs pop) (Structural covariances)

```
                          |--------------------
              2734.537    |***
              2751.038    |*******
              2767.539    |**********
              2784.040    |*************
              2800.541    |*************
              2817.042    |*****************
              2833.543    |************
N = 200       2850.044    |************
Mean = 2816.193  2866.545 |******
S. e. = 3.072    2883.046 |******
              2899.547    |**
              2916.048    |*
              2932.549    |*
              2949.050    |*
              2965.551    |*
                          |--------------------
```

K-L overoptimism (unstabilized) (Structural covariances)

```
                          |--------------------
             -1637.699    |*
             -1313.992    |*
              -990.285    |**
              -666.577    |******
              -342.870    |*********
               -19.162    |**************
               304.545    |*****************
N = 200        628.252    |*******************
Mean = 492.110   951.960  |*************
S. e. = 52.028  1275.667  |**********
              1599.374    |*******
              1923.082    |**
              2246.789    |**
              2570.497    |
              2894.204    |*
                          |--------------------
```

K-L overoptimism (stabilized) (Structural covariances)

```
                          |--------------------
                 4.122    |*
                77.236    |*
               150.349    |****
               223.462    |********
```

	296.576	|**********
	369.689	|****************
	442.802	|**************
N = 200	515.916	|***************
Mean = 472.636	589.029	|***************
S. e. = 12.681	662.143	|*********
	735.256	|*****
	808.369	|***
	881.483	|*
	954.596	|*
	1027.710	|*
		|--------------------

Measurement residuals (Measurement residuals)

Notes for Model (Measurement residuals)

Computation of degrees of freedom (Measurement residuals)

**Number of distinct sample moments:**	972
**Number of distinct parameters to be estimated:**	87
**Degrees of freedom (972 - 87):**	885

Result (Measurement residuals)

Minimum was achieved
Chi-square = 2703.756
Degrees of freedom = 885
Probability level = .000

g1 (g1 - Measurement residuals)

Estimates (g1 - Measurement residuals)

Scalar Estimates (g1 - Measurement residuals)

Maximum Likelihood Estimates

Regression Weights: (g1 - Measurement residuals)

			Estimate	S.E.	C.R.	P	Label
BPNSF19	<---	F1	1.000				
BPNSF13	<---	F1	1.143	.052	21.904	***	a1_1
BPNSF7	<---	F1	.863	.051	16.943	***	a2_1
BPNSF1	<---	F1	.762	.051	14.998	***	a3_1
BPNSF18	<---	F2	1.000				
BPNSF15	<---	F2	2.428	.301	8.066	***	a4_1
BPNSF10	<---	F2	3.040	.365	8.335	***	a5_1
BPNSF5	<---	F2	2.544	.311	8.191	***	a6_1
BPNSF24	<---	F3	1.000				
BPNSF16	<---	F3	1.131	.047	24.240	***	a7_1
BPNSF12	<---	F3	1.223	.048	25.542	***	a8_1
BPNSF4	<---	F3	.856	.044	19.522	***	a9_1
BPNSF22	<---	F4	1.000				
BPNSF20	<---	F4	1.199	.049	24.508	***	a10_1
BPNSF8	<---	F4	1.193	.050	23.824	***	a11_1
BPNSF2	<---	F4	1.118	.049	22.901	***	a12_1
BPNSF21	<---	F5	1.000				
BPNSF14	<---	F5	1.094	.042	25.847	***	a13_1
BPNSF9	<---	F5	1.040	.042	24.600	***	a14_1
BPNSF3	<---	F5	.883	.042	21.014	***	a15_1
BPNSF23	<---	F6	1.000				
BPNSF17	<---	F6	.974	.034	28.266	***	a16_1
BPNSF11	<---	F6	.946	.034	27.785	***	a17_1
BPNSF6	<---	F6	.848	.035	24.209	***	a18_1

Standardized Regression Weights: (g1 - Measurement residuals)

			Estimate
BPNSF19	<---	F1	.630

	Estimate
BPNSF13 <--- F1	.748
BPNSF7 <--- F1	.542
BPNSF1 <--- F1	.471
BPNSF18 <--- F2	.263
BPNSF15 <--- F2	.574
BPNSF10 <--- F2	.718
BPNSF5 <--- F2	.628
BPNSF24 <--- F3	.687
BPNSF16 <--- F3	.767
BPNSF12 <--- F3	.821
BPNSF4 <--- F3	.601
BPNSF22 <--- F4	.685
BPNSF20 <--- F4	.764
BPNSF8 <--- F4	.740
BPNSF2 <--- F4	.708
BPNSF21 <--- F5	.705
BPNSF14 <--- F5	.764
BPNSF9 <--- F5	.726
BPNSF3 <--- F5	.617
BPNSF23 <--- F6	.791
BPNSF17 <--- F6	.746
BPNSF11 <--- F6	.735
BPNSF6 <--- F6	.654

Intercepts: (g1 - Measurement residuals)

	Estimate	S.E.	C.R.	P	Label
**BPNSF19**	5.162	.041	127.012	***	i1_1
**BPNSF13**	5.060	.039	129.318	***	i2_1
**BPNSF7**	4.838	.041	118.588	***	i3_1
**BPNSF1**	4.711	.041	113.532	***	i4_1
**BPNSF18**	4.320	.041	104.324	***	i5_1
**BPNSF15**	3.709	.046	80.542	***	i6_1
**BPNSF10**	3.189	.046	69.185	***	i7_1
**BPNSF5**	3.741	.044	84.780	***	i8_1
**BPNSF24**	5.224	.038	138.282	***	i9_1
**BPNSF16**	5.117	.038	133.590	***	i10_1
**BPNSF12**	5.218	.039	134.827	***	i11_1
**BPNSF4**	5.116	.037	138.378	***	i12_1
**BPNSF22**	3.122	.043	72.197	***	i13_1
**BPNSF20**	2.516	.046	54.118	***	i14_1
**BPNSF8**	2.805	.048	58.684	***	i15_1
**BPNSF2**	2.393	.047	51.088	***	i16_1
**BPNSF21**	5.200	.040	130.235	***	i17_1
**BPNSF14**	5.460	.040	135.532	***	i18_1
**BPNSF9**	5.679	.040	140.700	***	i19_1
**BPNSF3**	5.609	.040	139.288	***	i20_1
**BPNSF23**	2.247	.046	49.100	***	i21_1
**BPNSF17**	2.579	.047	54.570	***	i22_1
**BPNSF11**	2.752	.047	59.079	***	i23_1
**BPNSF6**	2.568	.047	54.729	***	i24_1

Covariances: (g1 - Measurement residuals)

	Estimate	S.E.	C.R.	P	Label
F1 <--> F2	-.158	.024	-6.630	***	ccc1_1
F2 <--> F3	-.152	.023	-6.704	***	ccc2_1
F1 <--> F3	.726	.048	14.975	***	ccc3_1
F2 <--> F4	.321	.042	7.723	***	ccc4_1
F3 <--> F4	-.598	.045	-13.357	***	ccc5_1
F1 <--> F4	-.486	.043	-11.201	***	ccc6_1
F2 <--> F5	-.145	.023	-6.365	***	ccc7_1
F4 <--> F5	-.665	.049	-13.651	***	ccc8_1
F3 <--> F5	.771	.049	15.737	***	ccc9_1
F1 <--> F5	.896	.056	16.010	***	ccc10_1
F6 <--> F5	-.959	.061	-15.831	***	ccc11_1
F6 <--> F3	-.642	.050	-12.886	***	ccc12_1
F6 <--> F4	1.272	.073	17.405	***	ccc13_1
F6 <--> F2	.377	.048	7.795	***	ccc14_1
F6 <--> F1	-.618	.052	-11.931	***	ccc15_1

Correlations: (g1 - Measurement residuals)

	Estimate
F1 <--> F2	-.434
F2 <--> F3	-.413
F1 <--> F3	.836
F2 <--> F4	.763
F3 <--> F4	-.596
F1 <--> F4	-.491
F2 <--> F5	-.363
F4 <--> F5	-.611
F3 <--> F5	.808
F1 <--> F5	.953
F6 <--> F5	-.722
F6 <--> F3	-.524
F6 <--> F4	.909
F6 <--> F2	.734
F6 <--> F1	-.511

Variances: (g1 - Measurement residuals)

	Estimate	S.E.	C.R.	P	Label
**F1**	.856	.072	11.898	***	vvv1_1
**F2**	.155	.036	4.287	***	vvv2_1
**F3**	.880	.066	13.358	***	vvv3_1
**F4**	1.146	.085	13.419	***	vvv4_1
**F5**	1.034	.073	14.069	***	vvv5_1
**F6**	1.709	.104	16.468	***	vvv6_1
**e1**	1.298	.057	22.852	***	v1_1
**e2**	.878	.045	19.534	***	v2_1
**e3**	1.533	.064	23.941	***	v3_1
**e4**	1.748	.071	24.477	***	v4_1
**e5**	2.082	.083	25.017	***	v5_1
**e6**	1.854	.084	22.006	***	v6_1
**e7**	1.341	.076	17.533	***	v7_1
**e8**	1.538	.074	20.806	***	v8_1
**e9**	.982	.044	22.064	***	v9_1
**e10**	.788	.040	19.923	***	v10_1
**e11**	.638	.037	17.379	***	v11_1
**e12**	1.138	.049	23.364	***	v12_1
**e13**	1.292	.057	22.516	***	v13_1
**e14**	1.171	.057	20.631	***	v14_1
**e15**	1.348	.063	21.354	***	v15_1
**e16**	1.428	.065	22.100	***	v16_1
**e17**	1.045	.046	22.682	***	v17_1
**e18**	.880	.041	21.286	***	v18_1
**e19**	1.005	.045	22.279	***	v19_1
**e20**	1.309	.055	23.832	***	v20_1
**e21**	1.022	.050	20.293	***	v21_1
**e22**	1.293	.060	21.690	***	v22_1
**e23**	1.300	.059	21.938	***	v23_1
**e24**	1.642	.070	23.290	***	v24_1

Matrices (g1 - Measurement residuals)

Residual Covariances (g1 - Measurement residuals)

	BPNSF6	BPNSF11	BPNSF17	BPNSF23	BPNSF3	BPNSF9	BPNSF14	BPNSF21	BPNSF2	BPNSF8	BPNSF20	BPNSF22	BPNSF4	BPNSF12	BPNSF16	BPNS[illegible]
**BPNSF6**	-.218															
**BPNSF11**	.094	-.146														
**BPNSF17**	-.004	-.176	-.357													
**BPNSF23**	-.076	-.156	-.338	-.389												
**BPNSF3**	-.140	.026	.078	-.134	-.226											
**BPNSF9**	-.061	.096	.116	-.001	-.013	-.196										
**BPNSF14**	.087	.274	.165	.127	-.069	-.085	-.097									
**BPNSF21**	.127	.178	.349	.073	-.171	-.166	-.038	-.041								
**BPNSF2**	.057	.033	-.300	.018	-.283	-.109	-.087	-.076	-.210							
**BPNSF8**	.158	.062	-.207	-.328	.109	-.001	.236	.371	-.120	-.272						
**BPNSF20**	-.108	-.256	-.203	-.076	-.110	.021	.034	.061	-.208	-.334	-.522					
**BPNSF22**	-.169	-.172	-.442	-.195	-.003	.152	.309	.240	-.154	-.059	-.271	-.366				
**BPNSF4**	-.036	.152	.174	.197	.045	-.241	-.028	-.235	.015	.233	.146	.152	-.320			

	BPNSF6	BPNSF11	BPNSF17	BPNSF23	BPNSF3	BPNSF9	BPNSF14	BPNSF21	BPNSF2	BPNSF8	BPNSF20	BPNSF22	BPNSF4	BPNSF12	BPNSF16	BPNS
**BPNSF12**	.055	.350	.207	.280	-.229	-.271	-.138	-.276	.196	.336	.270	.143	-.246	-.254		
**BPNSF16**	-.075	.132	.095	.067	-.211	-.189	-.137	-.172	.152	.223	.194	.188	-.210	-.215	-.274	
**BPNSF24**	.035	.164	.098	.086	-.068	-.052	-.250	-.227	-.050	.169	.086	.008	-.173	-.166	-.159	-.3
**BPNSF5**	-.030	.024	-.289	-.310	.142	.090	.191	.088	-.127	-.201	-.367	-.207	.290	.056	-.028	-.0
**BPNSF10**	-.087	.308	-.322	-.386	.180	.080	.309	.168	-.183	-.072	-.523	-.424	.146	.121	.143	.2
**BPNSF15**	-.177	-.089	-.336	-.549	.101	.208	.553	.156	-.497	-.266	-.474	-.330	.187	.090	.312	.1
**BPNSF18**	-.477	-.295	-.258	-.442	.315	.405	.215	.534	-.308	-.238	-.476	-.191	.157	.294	.364	.2
**BPNSF1**	.120	.177	.373	.226	.005	-.317	-.388	-.096	.250	.307	.225	.188	.138	-.125	-.176	-.0
**BPNSF7**	.184	.073	.389	.066	-.086	-.005	-.194	.106	-.086	.410	.274	.234	-.227	-.380	-.372	-.2
**BPNSF13**	-.041	.094	.107	.037	-.179	-.216	.175	.026	-.027	.280	.019	.177	-.127	-.076	-.167	-.2
**BPNSF19**	-.042	-.094	-.082	-.230	-.051	-.162	.043	.356	-.268	.154	-.157	.058	-.171	-.153	-.064	-.1

Residual Means (g1 - Measurement residuals)

	BPNSF6	BPNSF11	BPNSF17	BPNSF23	BPNSF3	BPNSF9	BPNSF14	BPNSF21	BPNSF2	BPNSF8	BPNSF20	BPNSF22	BPNSF4	BPNSF12	BPNSF16	BPNSF24	BP
	-.111	-.062	-.140	-.178	.106	.168	.126	.158	-.163	-.037	-.165	-.107	-.048	.012	-.005	.019	-.

Standardized Residual Covariances (g1 - Measurement residuals)

	BPNSF6	BPNSF11	BPNSF17	BPNSF23	BPNSF3	BPNSF9	BPNSF14	BPNSF21	BPNSF2	BPNSF8	BPNSF20	BPNSF22	BPNSF4	BPNSF12	BPNSF16	BPNS
**BPNSF6**	-1.124															
**BPNSF11**	.619	-.765														
**BPNSF17**	-.026	-1.123	-1.814													
**BPNSF23**	-.502	-1.015	-2.161	-2.107												
**BPNSF3**	-1.144	.208	.626	-1.102	-1.581											
**BPNSF9**	-.487	.766	.907	-.006	-.117	-1.362										
**BPNSF14**	.692	2.173	1.283	1.017	-.613	-.730	-.676									
**BPNSF21**	1.032	1.442	2.778	.593	-1.566	-1.474	-.333	-.291								
**BPNSF2**	.383	.220	-1.958	.117	-2.328	-.881	-.703	-.622	-1.087							
**BPNSF8**	1.037	.399	-1.317	-2.127	.879	-.004	1.857	2.974	-.761	-1.349						
**BPNSF20**	-.726	-1.689	-1.318	-.506	-.907	.169	.278	.504	-1.350	-2.103	-2.738					
**BPNSF22**	-1.241	-1.245	-3.148	-1.415	-.024	1.333	2.709	2.136	-1.098	-.406	-1.916	-2.219				
**BPNSF4**	-.330	1.378	1.560	1.809	.460	-2.446	-.282	-2.422	.132	2.043	1.313	1.478	-2.658			
**BPNSF12**	.472	2.970	1.729	2.403	-2.183	-2.512	-1.269	-2.595	1.638	2.745	2.259	1.303	-2.474	-1.926		
**BPNSF16**	-.646	1.138	.805	.588	-2.049	-1.785	-1.289	-1.650	1.298	1.850	1.647	1.739	-2.158	-1.968	-2.119	
**BPNSF24**	.307	1.445	.851	.767	-.682	-.508	-2.426	-2.253	-.434	1.439	.754	.071	-1.833	-1.586	-1.557	-2.5
**BPNSF5**	-.219	.180	-2.101	-2.317	1.269	.804	1.695	.787	-.936	-1.438	-2.699	-1.651	2.814	.519	-.257	-.3
**BPNSF10**	-.613	2.150	-2.208	-2.711	1.536	.680	2.617	1.440	-1.267	-.486	-3.612	-3.194	1.358	1.058	1.266	2.0
**BPNSF15**	-1.267	-.633	-2.361	-3.968	.870	1.773	4.722	1.350	-3.531	-1.846	-3.366	-2.550	1.750	.799	2.793	1.3
**BPNSF18**	-3.910	-2.434	-2.097	-3.703	3.022	3.883	2.063	5.174	-2.527	-1.905	-3.925	-1.693	1.639	2.936	3.674	2.2
**BPNSF1**	.975	1.446	3.000	1.873	.048	-2.892	-3.523	-.890	2.039	2.448	1.847	1.661	1.402	-1.190	-1.700	-.6
**BPNSF7**	1.515	.605	3.173	.550	-.804	-.047	-1.762	.985	-.707	3.313	2.268	2.094	-2.329	-3.616	-3.607	-2.4
**BPNSF13**	-.351	.794	.894	.319	-1.668	-1.948	1.564	.238	-.232	2.319	.160	1.631	-1.314	-.712	-1.616	-2.0
**BPNSF19**	-.342	-.778	-.669	-1.925	-.469	-1.450	.387	3.239	-2.204	1.242	-1.300	.515	-1.737	-1.428	-.613	-1.2

Standardized Residual Means (g1 - Measurement residuals)

	BPNSF6	BPNSF11	BPNSF17	BPNSF23	BPNSF3	BPNSF9	BPNSF14	BPNSF21	BPNSF2	BPNSF8	BPNSF20	BPNSF22	BPNSF4	BPNSF12	BPNSF16	BPNSF24	BP
	-1.365	-.768	-1.712	-2.259	1.529	2.418	1.807	2.300	-2.016	-.448	-2.058	-1.429	-.749	.185	-.075	.299	-.

Notes for Group/Model (g1 - Measurement residuals)

The following covariance matrix is not positive definite (g1 - Measurement residuals)

	F5	F4	F3	F2	F1	F6
**F5**	1.034					
**F4**	-.665	1.146				
**F3**	.771	-.598	.880			
**F2**	-.145	.321	-.152	.155		
**F1**	.896	-.486	.726	-.158	.856	
**F6**	-.959	1.272	-.642	.377	-.618	1.709

This solution is not admissible.

Modification Indices (g1 - Measurement residuals)

Covariances: (g1 - Measurement residuals)

	M.I.	Par Change
F2 <--> F4	8.214	-.035

			M.I.	Par Change
F2	<-->	F3	8.782	-.036
F1	<-->	F2	4.216	.024
e23	<-->	F2	28.798	.113
e21	<-->	F4	11.920	.129
e21	<-->	F3	4.727	.081
e21	<-->	F2	11.727	-.065
e21	<-->	F6	5.674	-.096
e19	<-->	F1	10.523	-.110
e19	<-->	e20	4.628	.128
e18	<-->	F2	5.851	.042
e17	<-->	F3	8.675	-.105
e17	<-->	F1	24.066	.169
e17	<-->	e22	6.182	.155
e17	<-->	e20	4.378	-.126
e16	<-->	F5	7.559	-.106
e16	<-->	F3	4.123	.086
e16	<-->	e22	6.971	-.195
e16	<-->	e21	5.873	.163
e16	<-->	e20	4.293	-.148
e15	<-->	F1	8.544	.120
e15	<-->	e24	6.167	.197
e15	<-->	e23	5.033	.162
e15	<-->	e21	9.897	-.208
e15	<-->	e19	8.551	-.185
e15	<-->	e17	4.366	.134
e14	<-->	F2	13.570	-.075
e14	<-->	F6	4.848	.101
e14	<-->	e23	6.965	-.180
e14	<-->	e21	16.889	.256
e13	<-->	e22	4.802	-.153
e13	<-->	e18	4.663	.124
e13	<-->	e15	6.994	.188
e12	<-->	e20	8.865	.186
e12	<-->	e18	4.094	.108
e11	<-->	F2	12.833	-.058
e11	<-->	F6	4.227	.076
e11	<-->	e23	5.299	.125
e11	<-->	e21	4.915	.110
e10	<-->	F4	4.883	.075
e10	<-->	F6	6.582	-.101
e10	<-->	e16	4.448	.126
e9	<-->	e19	10.206	.170
e9	<-->	e18	8.329	-.146
e8	<-->	e12	10.816	.233
e8	<-->	e10	4.595	-.135
e7	<-->	F4	4.335	-.094
e7	<-->	F1	5.277	.099
e7	<-->	e23	30.209	.420
e7	<-->	e15	5.180	.179
e7	<-->	e14	9.001	-.223
e7	<-->	e13	8.591	-.221
e7	<-->	e8	4.931	-.183
e6	<-->	F2	5.550	.056
e6	<-->	e21	5.617	-.181
e6	<-->	e18	17.203	.287
e6	<-->	e16	8.273	-.251
e6	<-->	e11	7.560	-.176
e6	<-->	e10	5.676	.161
e6	<-->	e7	8.697	.263
e5	<-->	F1	9.148	.145
e5	<-->	e24	6.484	-.236
e5	<-->	e18	11.170	-.233
e5	<-->	e17	7.544	.205
e5	<-->	e6	4.777	.218
e4	<-->	F4	8.979	.139
e4	<-->	F3	7.121	.120
e4	<-->	F2	32.005	-.132
e4	<-->	e20	8.877	.225
e4	<-->	e18	13.251	-.235
e4	<-->	e16	7.670	.226
e4	<-->	e12	16.326	.288
e4	<-->	e7	8.201	-.242

			M.I.	Par Change
e4	<-->	e6	15.185	-.360
e3	<-->	F3	28.579	-.226
e3	<-->	F1	22.282	.187
e3	<-->	e22	7.772	.206
e3	<-->	e18	9.475	-.187
e3	<-->	e17	4.982	.145
e3	<-->	e16	8.812	-.229
e3	<-->	e11	9.394	-.173
e3	<-->	e10	6.632	-.153
e3	<-->	e7	6.435	.203
e3	<-->	e4	4.304	.169
e2	<-->	F2	15.684	.069
e2	<-->	e20	4.838	-.125
e2	<-->	e19	8.481	-.148
e2	<-->	e18	24.494	.239
e2	<-->	e11	4.974	.101
e2	<-->	e8	4.528	.138
e2	<-->	e3	5.422	.143
e1	<-->	e21	4.665	-.136
e1	<-->	e19	5.587	-.141
e1	<-->	e17	34.339	.355
e1	<-->	e7	4.680	.161
e1	<-->	e5	8.245	.237
e1	<-->	e4	4.722	-.166

Variances: (g1 - Measurement residuals)

	M.I.	Par Change
**e24**	7.229	-.323
**e9**	6.631	-.193

Regression Weights: (g1 - Measurement residuals)

			M.I.	Par Change
BPNSF11	<---	F2	16.534	.676
BPNSF11	<---	BPNSF8	4.473	.037
BPNSF11	<---	BPNSF5	4.042	.029
BPNSF11	<---	BPNSF10	13.633	.060
BPNSF11	<---	BPNSF15	4.349	.030
BPNSF23	<---	F2	5.051	-.341
BPNSF23	<---	BPNSF8	5.212	-.037
BPNSF23	<---	BPNSF10	5.399	-.034
BPNSF23	<---	BPNSF15	5.703	-.031
BPNSF21	<---	BPNSF17	4.458	.035
BPNSF21	<---	BPNSF19	5.177	.022
BPNSF2	<---	F5	11.486	-.212
BPNSF2	<---	F1	10.001	-.221
BPNSF2	<---	BPNSF3	5.257	-.024
BPNSF2	<---	BPNSF14	4.975	-.024
BPNSF2	<---	BPNSF21	5.236	-.026
BPNSF2	<---	BPNSF15	4.847	-.033
BPNSF2	<---	BPNSF7	6.532	-.031
BPNSF2	<---	BPNSF19	5.021	-.025
BPNSF8	<---	F1	4.259	.142
BPNSF8	<---	BPNSF6	4.245	.040
BPNSF20	<---	F2	8.403	-.468
BPNSF20	<---	BPNSF11	5.386	-.041
BPNSF20	<---	BPNSF14	4.139	-.020
BPNSF20	<---	BPNSF21	4.094	-.021
BPNSF20	<---	BPNSF8	4.925	-.038
BPNSF20	<---	BPNSF5	5.033	-.031
BPNSF20	<---	BPNSF10	9.491	-.048
BPNSF20	<---	BPNSF15	4.753	-.030
BPNSF20	<---	BPNSF18	4.933	-.027
BPNSF20	<---	BPNSF19	4.055	-.021
BPNSF12	<---	F5	4.293	-.095
BPNSF12	<---	F6	4.398	.075
BPNSF10	<---	BPNSF11	13.799	.072
BPNSF10	<---	BPNSF3	7.032	.029
BPNSF10	<---	BPNSF9	4.918	.024
BPNSF10	<---	BPNSF14	6.513	.028

			M.I.	Par Change
BPNSF10	<---	BPNSF21	5.812	.028
BPNSF10	<---	BPNSF8	6.514	.049
BPNSF10	<---	BPNSF4	4.474	.025
BPNSF10	<---	BPNSF12	4.574	.025
BPNSF10	<---	BPNSF16	5.033	.027
BPNSF10	<---	BPNSF24	7.147	.031
BPNSF10	<---	BPNSF15	9.483	.048
BPNSF10	<---	BPNSF18	4.317	.029
BPNSF10	<---	BPNSF7	9.000	.037
BPNSF10	<---	BPNSF13	7.145	.032
BPNSF10	<---	BPNSF19	8.213	.034
BPNSF15	<---	F5	5.729	.170
BPNSF15	<---	F4	4.600	-.146
BPNSF15	<---	F6	5.678	-.132
BPNSF15	<---	BPNSF14	6.151	.030
BPNSF15	<---	BPNSF16	4.048	.026
BPNSF15	<---	BPNSF10	4.216	.039
BPNSF15	<---	BPNSF18	4.099	.030
BPNSF18	<---	F5	22.976	.344
BPNSF18	<---	F4	9.052	-.208
BPNSF18	<---	F3	13.668	.293
BPNSF18	<---	F2	5.113	-.449
BPNSF18	<---	F1	19.498	.353
BPNSF18	<---	F6	11.412	-.190
BPNSF18	<---	BPNSF21	5.134	.029
BPNSF18	<---	BPNSF19	4.871	.029
BPNSF1	<---	F5	5.511	-.155
BPNSF1	<---	F6	4.270	.107
BPNSF1	<---	BPNSF3	5.767	-.027
BPNSF1	<---	BPNSF9	10.642	-.036
BPNSF1	<---	BPNSF14	13.162	-.041
BPNSF1	<---	BPNSF21	8.265	-.034
BPNSF1	<---	BPNSF22	4.122	-.037
BPNSF1	<---	BPNSF12	6.900	-.031
BPNSF1	<---	BPNSF16	7.622	-.033
BPNSF1	<---	BPNSF24	6.075	-.029
BPNSF1	<---	BPNSF5	10.013	-.050
BPNSF1	<---	BPNSF10	13.579	-.066
BPNSF1	<---	BPNSF15	16.340	-.064
BPNSF1	<---	BPNSF18	7.819	-.039
BPNSF1	<---	BPNSF7	4.836	-.028
BPNSF1	<---	BPNSF13	8.376	-.035
BPNSF1	<---	BPNSF19	9.995	-.038
BPNSF7	<---	BPNSF17	4.503	.042
BPNSF13	<---	BPNSF14	6.250	.022
BPNSF13	<---	BPNSF5	5.341	.028
BPNSF13	<---	BPNSF10	4.698	.029
BPNSF13	<---	BPNSF15	5.253	.027
BPNSF13	<---	BPNSF7	4.239	.020
BPNSF19	<---	F5	5.226	.133
BPNSF19	<---	F4	6.661	-.145
BPNSF19	<---	F6	9.913	-.144
BPNSF19	<---	BPNSF21	9.087	.032
BPNSF19	<---	BPNSF18	5.335	.029
BPNSF19	<---	BPNSF7	4.644	.024

Means: (g1 - Measurement residuals)

	M.I.	Par Change

Intercepts: (g1 - Measurement residuals)

	M.I.	Par Change
**BPNSF10**	5.262	.144
**BPNSF1**	7.362	-.174

Bootstrap (g1 - Measurement residuals)

Bootstrap standard errors (g1 - Measurement residuals)

Scalar Estimates (g1 - Measurement residuals)

Regression Weights: (g1 - Measurement residuals)

Parameter			SE	SE-SE	Mean	Bias	SE-Bias
BPNSF19	<---	F1	.000	.000	1.000	.000	.000
BPNSF13	<---	F1	.057	.003	1.140	-.004	.004
BPNSF7	<---	F1	.059	.003	.862	-.001	.004
BPNSF1	<---	F1	.071	.004	.761	-.002	.005
BPNSF18	<---	F2	.000	.000	1.000	.000	.000
BPNSF15	<---	F2	.376	.019	2.472	.044	.027
BPNSF10	<---	F2	.495	.025	3.100	.060	.035
BPNSF5	<---	F2	.411	.021	2.595	.051	.029
BPNSF24	<---	F3	.000	.000	1.000	.000	.000
BPNSF16	<---	F3	.053	.003	1.131	.000	.004
BPNSF12	<---	F3	.049	.002	1.219	-.003	.003
BPNSF4	<---	F3	.051	.003	.854	-.002	.004
BPNSF22	<---	F4	.000	.000	1.000	.000	.000
BPNSF20	<---	F4	.051	.003	1.197	-.002	.004
BPNSF8	<---	F4	.049	.002	1.187	-.006	.003
BPNSF2	<---	F4	.054	.003	1.117	-.002	.004
BPNSF21	<---	F5	.000	.000	1.000	.000	.000
BPNSF14	<---	F5	.045	.002	1.094	.001	.003
BPNSF9	<---	F5	.053	.003	1.042	.001	.004
BPNSF3	<---	F5	.059	.003	.885	.002	.004
BPNSF23	<---	F6	.000	.000	1.000	.000	.000
BPNSF17	<---	F6	.034	.002	.976	.003	.002
BPNSF11	<---	F6	.038	.002	.947	.000	.003
BPNSF6	<---	F6	.036	.002	.847	-.001	.003

Standardized Regression Weights: (g1 - Measurement residuals)

Parameter			SE	SE-SE	Mean	Bias	SE-Bias
BPNSF19	<---	F1	.028	.001	.632	.002	.002
BPNSF13	<---	F1	.018	.001	.747	-.002	.001
BPNSF7	<---	F1	.031	.002	.542	.000	.002
BPNSF1	<---	F1	.031	.002	.470	-.001	.002
BPNSF18	<---	F2	.039	.002	.264	.001	.003
BPNSF15	<---	F2	.027	.001	.574	.000	.002
BPNSF10	<---	F2	.026	.001	.719	.000	.002
BPNSF5	<---	F2	.025	.001	.628	.000	.002
BPNSF24	<---	F3	.021	.001	.688	.001	.001
BPNSF16	<---	F3	.021	.001	.768	.001	.001
BPNSF12	<---	F3	.014	.001	.821	.000	.001
BPNSF4	<---	F3	.027	.001	.599	-.002	.002
BPNSF22	<---	F4	.019	.001	.685	.000	.001
BPNSF20	<---	F4	.019	.001	.765	.000	.001
BPNSF8	<---	F4	.021	.001	.737	-.003	.001
BPNSF2	<---	F4	.022	.001	.707	-.001	.002
BPNSF21	<---	F5	.021	.001	.706	.001	.001
BPNSF14	<---	F5	.025	.001	.766	.002	.002
BPNSF9	<---	F5	.024	.001	.728	.002	.002
BPNSF3	<---	F5	.033	.002	.619	.002	.002
BPNSF23	<---	F6	.018	.001	.791	.000	.001
BPNSF17	<---	F6	.020	.001	.747	.002	.001
BPNSF11	<---	F6	.021	.001	.734	-.002	.001
BPNSF6	<---	F6	.022	.001	.651	-.003	.002

Intercepts: (g1 - Measurement residuals)

Parameter	SE	SE-SE	Mean	Bias	SE-Bias
**BPNSF19**	.043	.002	5.166	.004	.003
**BPNSF13**	.040	.002	5.065	.005	.003
**BPNSF7**	.043	.002	4.844	.005	.003
**BPNSF1**	.040	.002	4.716	.005	.003
**BPNSF18**	.042	.002	4.319	-.001	.003
**BPNSF15**	.045	.002	3.710	.000	.003
**BPNSF10**	.049	.002	3.185	-.003	.003
**BPNSF5**	.045	.002	3.739	-.003	.003
**BPNSF24**	.040	.002	5.227	.003	.003
**BPNSF16**	.042	.002	5.122	.005	.003

Parameter	SE	SE-SE	Mean	Bias	SE-Bias
**BPNSF12**	.037	.002	5.223	.006	.003
**BPNSF4**	.038	.002	5.118	.002	.003
**BPNSF22**	.045	.002	3.118	-.004	.003
**BPNSF20**	.050	.003	2.507	-.009	.004
**BPNSF8**	.052	.003	2.803	-.002	.004
**BPNSF2**	.047	.002	2.390	-.003	.003
**BPNSF21**	.039	.002	5.207	.007	.003
**BPNSF14**	.041	.002	5.466	.007	.003
**BPNSF9**	.045	.002	5.683	.004	.003
**BPNSF3**	.040	.002	5.613	.003	.003
**BPNSF23**	.043	.002	2.240	-.007	.003
**BPNSF17**	.051	.003	2.573	-.006	.004
**BPNSF11**	.049	.002	2.750	-.001	.003
**BPNSF6**	.050	.003	2.569	.001	.004

Covariances: (g1 - Measurement residuals)

Parameter	SE	SE-SE	Mean	Bias	SE-Bias
F1 <--> F2	.025	.001	-.158	.000	.002
F2 <--> F3	.024	.001	-.152	.000	.002
F1 <--> F3	.046	.002	.724	-.002	.003
F2 <--> F4	.051	.003	.322	.000	.004
F3 <--> F4	.044	.002	-.601	-.003	.003
F1 <--> F4	.042	.002	-.487	-.001	.003
F2 <--> F5	.024	.001	-.145	.000	.002
F4 <--> F5	.042	.002	-.663	.002	.003
F3 <--> F5	.053	.003	.770	-.001	.004
F1 <--> F5	.074	.004	.897	.001	.005
F6 <--> F5	.058	.003	-.957	.002	.004
F6 <--> F3	.049	.002	-.645	-.002	.003
F6 <--> F4	.068	.003	1.268	-.004	.005
F6 <--> F2	.057	.003	.376	-.001	.004
F6 <--> F1	.055	.003	-.621	-.002	.004

Correlations: (g1 - Measurement residuals)

Parameter	SE	SE-SE	Mean	Bias	SE-Bias
F1 <--> F2	.040	.002	-.435	-.001	.003
F2 <--> F3	.036	.002	-.412	.001	.003
F1 <--> F3	.026	.001	.833	-.003	.002
F2 <--> F4	.023	.001	.762	-.002	.002
F3 <--> F4	.028	.001	-.598	-.002	.002
F1 <--> F4	.032	.002	-.491	.000	.002
F2 <--> F5	.037	.002	-.363	.001	.003
F4 <--> F5	.027	.001	-.609	.002	.002
F3 <--> F5	.023	.001	.806	-.002	.002
F1 <--> F5	.023	.001	.951	-.001	.002
F6 <--> F5	.025	.001	-.721	.001	.002
F6 <--> F3	.031	.002	-.526	-.003	.002
F6 <--> F4	.018	.001	.907	-.002	.001
F6 <--> F2	.029	.001	.732	-.002	.002
F6 <--> F1	.033	.002	-.513	-.002	.002

Variances: (g1 - Measurement residuals)

Parameter	SE	SE-SE	Mean	Bias	SE-Bias
**F1**	.078	.004	.860	.004	.006
**F2**	.047	.002	.159	.004	.003
**F3**	.065	.003	.882	.002	.005
**F4**	.079	.004	1.147	.002	.006
**F5**	.081	.004	1.036	.002	.006
**F6**	.100	.005	1.704	-.004	.007
**e1**	.098	.005	1.287	-.011	.007
**e2**	.056	.003	.880	.001	.004
**e3**	.101	.005	1.531	-.002	.007
**e4**	.088	.004	1.737	-.010	.006
**e5**	.084	.004	2.074	-.007	.006
**e6**	.096	.005	1.851	-.003	.007
**e7**	.108	.005	1.336	-.005	.008
**e8**	.091	.005	1.539	.001	.006

Parameter	SE	SE-SE	Mean	Bias	SE-Bias
**e9**	.066	.003	.979	-.003	.005
**e10**	.063	.003	.780	-.008	.004
**e11**	.047	.002	.633	-.005	.003
**e12**	.068	.003	1.144	.006	.005
**e13**	.068	.003	1.292	.000	.005
**e14**	.096	.005	1.163	-.009	.007
**e15**	.101	.005	1.359	.011	.007
**e16**	.107	.005	1.428	.000	.008
**e17**	.069	.003	1.040	-.005	.005
**e18**	.095	.005	.870	-.009	.007
**e19**	.094	.005	.993	-.012	.007
**e20**	.118	.006	1.300	-.009	.008
**e21**	.086	.004	1.018	-.004	.006
**e22**	.105	.005	1.281	-.011	.007
**e23**	.097	.005	1.306	.006	.007
**e24**	.107	.005	1.655	.014	.008

Matrices (g1 - Measurement residuals)

Sample Covariances - Standard Errors (g1 - Measurement residuals)

	BPNSF6	BPNSF11	BPNSF17	BPNSF23	BPNSF3	BPNSF9	BPNSF14	BPNSF21	BPNSF2	BPNSF8	BPNSF20	BPNSF22	BPNSF4	BPNSF12	BPNSF16	BPNS[illegible]
**BPNSF6**	.189															
**BPNSF11**	.149	.158														
**BPNSF17**	.153	.153	.188													
**BPNSF23**	.149	.150	.138	.180												
**BPNSF3**	.128	.115	.109	.108	.175											
**BPNSF9**	.116	.104	.108	.109	.116	.182										
**BPNSF14**	.124	.116	.124	.117	.105	.122	.166									
**BPNSF21**	.101	.104	.121	.111	.106	.115	.137	.163								
**BPNSF2**	.154	.150	.138	.147	.101	.110	.120	.116	.159							
**BPNSF8**	.153	.148	.155	.151	.114	.115	.111	.110	.151	.169						
**BPNSF20**	.153	.147	.159	.149	.100	.106	.131	.116	.132	.154	.166					
**BPNSF22**	.121	.128	.124	.125	.095	.091	.102	.105	.112	.126	.114	.119				
**BPNSF4**	.104	.104	.097	.085	.104	.088	.074	.096	.113	.107	.091	.090	.102			
**BPNSF12**	.117	.103	.107	.091	.094	.110	.131	.120	.107	.115	.100	.092	.085	.135		
**BPNSF16**	.122	.104	.107	.108	.098	.107	.123	.114	.109	.109	.102	.082	.080	.102	.123	
**BPNSF24**	.101	.093	.103	.097	.106	.110	.102	.105	.097	.098	.090	.092	.082	.095	.094	.1[illegible]
**BPNSF5**	.124	.125	.135	.113	.092	.091	.103	.109	.112	.136	.118	.109	.088	.098	.099	.0[illegible]
**BPNSF10**	.139	.143	.144	.134	.123	.109	.126	.121	.139	.153	.136	.126	.114	.116	.112	.1[illegible]
**BPNSF15**	.123	.129	.132	.115	.124	.112	.111	.125	.134	.148	.114	.123	.098	.112	.110	.1[illegible]
**BPNSF18**	.120	.122	.124	.110	.104	.112	.117	.113	.113	.134	.102	.112	.088	.117	.111	.1[illegible]
**BPNSF1**	.124	.105	.111	.094	.112	.097	.103	.110	.110	.115	.097	.096	.091	.094	.086	.0[illegible]
**BPNSF7**	.121	.112	.114	.093	.112	.112	.109	.118	.111	.114	.097	.101	.091	.109	.100	.1[illegible]
**BPNSF13**	.117	.118	.112	.112	.098	.116	.130	.133	.116	.117	.108	.095	.088	.132	.113	.1[illegible]
**BPNSF19**	.107	.112	.117	.115	.112	.113	.136	.142	.121	.125	.124	.106	.099	.108	.117	.1[illegible]

Sample Correlations - Standard Errors (g1 - Measurement residuals)

	BPNSF6	BPNSF11	BPNSF17	BPNSF23	BPNSF3	BPNSF9	BPNSF14	BPNSF21	BPNSF2	BPNSF8	BPNSF20	BPNSF22	BPNSF4	BPNSF12	BPNSF16	BPNS[illegible]
**BPNSF6**	.000															
**BPNSF11**	.044	.000														
**BPNSF17**	.044	.048	.000													
**BPNSF23**	.043	.040	.047	.000												
**BPNSF3**	.052	.051	.049	.043	.000											
**BPNSF9**	.051	.045	.046	.047	.053	.000										
**BPNSF14**	.052	.049	.049	.048	.053	.046	.000									
**BPNSF21**	.043	.042	.053	.044	.052	.051	.051	.000								
**BPNSF2**	.051	.044	.048	.045	.045	.047	.048	.044	.000							
**BPNSF8**	.045	.042	.049	.051	.052	.048	.048	.049	.044	.000						
**BPNSF20**	.050	.047	.049	.043	.044	.045	.052	.051	.047	.048	.000					
**BPNSF22**	.044	.046	.048	.044	.045	.043	.051	.052	.040	.040	.044	.000				
**BPNSF4**	.049	.052	.049	.044	.056	.049	.035	.050	.053	.051	.044	.050	.000			
**BPNSF12**	.050	.049	.049	.043	.048	.050	.055	.051	.048	.053	.047	.047	.046	.000		
**BPNSF16**	.053	.047	.048	.048	.053	.049	.056	.049	.050	.050	.048	.045	.044	.043	.000	
**BPNSF24**	.048	.047	.051	.050	.052	.050	.056	.054	.047	.051	.049	.051	.048	.039	.052	.0[illegible]
**BPNSF5**	.044	.045	.053	.047	.045	.043	.048	.049	.042	.052	.049	.048	.048	.048	.047	.0[illegible]
**BPNSF10**	.050	.041	.052	.048	.056	.049	.054	.051	.047	.050	.051	.050	.058	.053	.052	.0[illegible]
**BPNSF15**	.047	.047	.051	.047	.057	.052	.050	.055	.052	.055	.046	.053	.052	.054	.055	.0[illegible]
**BPNSF18**	.051	.051	.053	.050	.052	.057	.057	.054	.048	.055	.047	.054	.050	.061	.059	.0[illegible]
**BPNSF1**	.051	.044	.047	.042	.045	.047	.050	.047	.045	.047	.044	.045	.045	.046	.045	.0[illegible]

	BPNSF6	BPNSF11	BPNSF17	BPNSF23	BPNSF3	BPNSF9	BPNSF14	BPNSF21	BPNSF2	BPNSF8	BPNSF20	BPNSF22	BPNSF4	BPNSF12	BPNSF16	BPNS[illegible]
**BPNSF7**	.050	.045	.047	.041	.053	.047	.052	.048	.045	.046	.043	.046	.050	.052	.051	.0[illegible]
**BPNSF13**	.050	.050	.049	.048	.048	.051	.037	.044	.048	.051	.047	.048	.046	.052	.050	.0[illegible]
**BPNSF19**	.045	.046	.049	.043	.053	.052	.053	.045	.048	.054	.050	.051	.054	.048	.054	.0[illegible]

Sample Means - Standard Errors (g1 - Measurement residuals)

	BPNSF6	BPNSF11	BPNSF17	BPNSF23	BPNSF3	BPNSF9	BPNSF14	BPNSF21	BPNSF2	BPNSF8	BPNSF20	BPNSF22	BPNSF4	BPNSF12	BPNSF16	BPNSF[illegible]
**BPNSF6**	.082	.073	.079	.072	.066	.069	.069	.074	.076	.083	.076	.069	.059	.067	.063	.06[illegible]

Bootstrap Confidence (g1 - Measurement residuals)

Percentile method (g1 - Measurement residuals)

90% confidence intervals (percentile method)

Scalar Estimates (g1 - Measurement residuals)

Regression Weights: (g1 - Measurement residuals)

Parameter			Estimate	Lower	Upper	P
BPNSF19	<---	F1	1.000	1.000	1.000	...
BPNSF13	<---	F1	1.143	1.043	1.243	.010
BPNSF7	<---	F1	.863	.769	.962	.010
BPNSF1	<---	F1	.762	.645	.879	.010
BPNSF18	<---	F2	1.000	1.000	1.000	...
BPNSF15	<---	F2	2.428	1.960	3.174	.010
BPNSF10	<---	F2	3.040	2.425	4.012	.010
BPNSF5	<---	F2	2.544	2.047	3.419	.010
BPNSF24	<---	F3	1.000	1.000	1.000	...
BPNSF16	<---	F3	1.131	1.045	1.220	.010
BPNSF12	<---	F3	1.223	1.146	1.296	.010
BPNSF4	<---	F3	.856	.763	.942	.010
BPNSF22	<---	F4	1.000	1.000	1.000	...
BPNSF20	<---	F4	1.199	1.122	1.293	.010
BPNSF8	<---	F4	1.193	1.112	1.272	.010
BPNSF2	<---	F4	1.118	1.036	1.212	.010
BPNSF21	<---	F5	1.000	1.000	1.000	...
BPNSF14	<---	F5	1.094	1.017	1.175	.010
BPNSF9	<---	F5	1.040	.953	1.132	.010
BPNSF3	<---	F5	.883	.790	.987	.010
BPNSF23	<---	F6	1.000	1.000	1.000	...
BPNSF17	<---	F6	.974	.922	1.038	.010
BPNSF11	<---	F6	.946	.888	1.006	.010
BPNSF6	<---	F6	.848	.791	.910	.010

Standardized Regression Weights: (g1 - Measurement residuals)

Parameter			Estimate	Lower	Upper	P
BPNSF19	<---	F1	.630	.585	.677	.010
BPNSF13	<---	F1	.748	.714	.776	.010
BPNSF7	<---	F1	.542	.494	.594	.010
BPNSF1	<---	F1	.471	.416	.520	.010
BPNSF18	<---	F2	.263	.198	.327	.010
BPNSF15	<---	F2	.574	.530	.616	.010
BPNSF10	<---	F2	.718	.678	.757	.010
BPNSF5	<---	F2	.628	.583	.672	.010
BPNSF24	<---	F3	.687	.651	.721	.010
BPNSF16	<---	F3	.767	.733	.801	.010
BPNSF12	<---	F3	.821	.797	.844	.010
BPNSF4	<---	F3	.601	.553	.646	.010
BPNSF22	<---	F4	.685	.656	.716	.010
BPNSF20	<---	F4	.764	.728	.798	.010
BPNSF8	<---	F4	.740	.700	.772	.010
BPNSF2	<---	F4	.708	.669	.741	.010
BPNSF21	<---	F5	.705	.670	.741	.010
BPNSF14	<---	F5	.764	.717	.806	.010
BPNSF9	<---	F5	.726	.690	.769	.010
BPNSF3	<---	F5	.617	.566	.670	.010
BPNSF23	<---	F6	.791	.763	.821	.010

Parameter			Estimate	Lower	Upper	P
BPNSF17	<---	F6	.746	.709	.779	.010
BPNSF11	<---	F6	.735	.698	.768	.010
BPNSF6	<---	F6	.654	.613	.686	.010

Intercepts: (g1 - Measurement residuals)

Parameter	Estimate	Lower	Upper	P
**BPNSF19**	5.162	5.089	5.232	.010
**BPNSF13**	5.060	4.995	5.131	.010
**BPNSF7**	4.838	4.770	4.911	.010
**BPNSF1**	4.711	4.655	4.790	.010
**BPNSF18**	4.320	4.255	4.391	.010
**BPNSF15**	3.709	3.626	3.783	.010
**BPNSF10**	3.189	3.106	3.258	.010
**BPNSF5**	3.741	3.666	3.819	.010
**BPNSF24**	5.224	5.158	5.294	.010
**BPNSF16**	5.117	5.054	5.195	.010
**BPNSF12**	5.218	5.162	5.288	.010
**BPNSF4**	5.116	5.057	5.177	.010
**BPNSF22**	3.122	3.040	3.191	.010
**BPNSF20**	2.516	2.413	2.589	.010
**BPNSF8**	2.805	2.714	2.884	.010
**BPNSF2**	2.393	2.314	2.474	.010
**BPNSF21**	5.200	5.127	5.270	.010
**BPNSF14**	5.460	5.399	5.539	.010
**BPNSF9**	5.679	5.606	5.757	.010
**BPNSF3**	5.609	5.551	5.689	.010
**BPNSF23**	2.247	2.168	2.312	.010
**BPNSF17**	2.579	2.489	2.653	.010
**BPNSF11**	2.752	2.665	2.830	.010
**BPNSF6**	2.568	2.482	2.654	.010

Covariances: (g1 - Measurement residuals)

Parameter			Estimate	Lower	Upper	P
F1	<-->	F2	-.158	-.198	-.117	.010
F2	<-->	F3	-.152	-.192	-.114	.010
F1	<-->	F3	.726	.649	.794	.010
F2	<-->	F4	.321	.238	.410	.010
F3	<-->	F4	-.598	-.676	-.524	.010
F1	<-->	F4	-.486	-.558	-.414	.010
F2	<-->	F5	-.145	-.184	-.109	.010
F4	<-->	F5	-.665	-.732	-.597	.010
F3	<-->	F5	.771	.689	.859	.010
F1	<-->	F5	.896	.788	1.025	.010
F6	<-->	F5	-.959	-1.074	-.865	.010
F6	<-->	F3	-.642	-.719	-.561	.010
F6	<-->	F4	1.272	1.152	1.382	.010
F6	<-->	F2	.377	.285	.474	.010
F6	<-->	F1	-.618	-.715	-.533	.010

Correlations: (g1 - Measurement residuals)

Parameter			Estimate	Lower	Upper	P
F1	<-->	F2	-.434	-.499	-.363	.010
F2	<-->	F3	-.413	-.470	-.356	.010
F1	<-->	F3	.836	.792	.877	.010
F2	<-->	F4	.763	.724	.800	.010
F3	<-->	F4	-.596	-.639	-.553	.010
F1	<-->	F4	-.491	-.541	-.435	.010
F2	<-->	F5	-.363	-.415	-.291	.010
F4	<-->	F5	-.611	-.651	-.561	.010
F3	<-->	F5	.808	.765	.842	.010
F1	<-->	F5	.953	.909	.988	.010
F6	<-->	F5	-.722	-.765	-.677	.010
F6	<-->	F3	-.524	-.574	-.476	.010
F6	<-->	F4	.909	.877	.938	.010
F6	<-->	F2	.734	.677	.775	.010
F6	<-->	F1	-.511	-.565	-.457	.010

Variances: (g1 - Measurement residuals)

Parameter	Estimate	Lower	Upper	P
**F1**	.856	.726	.983	.010
**F2**	.155	.088	.239	.010
**F3**	.880	.769	.995	.010
**F4**	1.146	1.018	1.270	.010
**F5**	1.034	.908	1.179	.010
**F6**	1.709	1.547	1.870	.010
**e1**	1.298	1.144	1.463	.010
**e2**	.878	.791	.969	.010
**e3**	1.533	1.361	1.693	.010
**e4**	1.748	1.588	1.875	.010
**e5**	2.082	1.935	2.228	.010
**e6**	1.854	1.694	2.019	.010
**e7**	1.341	1.179	1.515	.010
**e8**	1.538	1.391	1.690	.010
**e9**	.982	.868	1.088	.010
**e10**	.788	.678	.890	.010
**e11**	.638	.561	.715	.010
**e12**	1.138	1.020	1.263	.010
**e13**	1.292	1.173	1.401	.010
**e14**	1.171	1.005	1.314	.010
**e15**	1.348	1.194	1.529	.010
**e16**	1.428	1.255	1.612	.010
**e17**	1.045	.914	1.149	.010
**e18**	.880	.720	1.038	.010
**e19**	1.005	.849	1.152	.010
**e20**	1.309	1.100	1.517	.010
**e21**	1.022	.887	1.164	.010
**e22**	1.293	1.114	1.464	.010
**e23**	1.300	1.144	1.468	.010
**e24**	1.642	1.484	1.845	.010

Matrices (g1 - Measurement residuals)

Sample Covariances (g1 - Measurement residuals)

Sample Covariances - Lower Bounds (PC) (g1 - Measurement residuals)

	BPNSF6	BPNSF11	BPNSF17	BPNSF23	BPNSF3	BPNSF9	BPNSF14	BPNSF21
**BPNSF6**	2.334							
**BPNSF11**	1.185	2.407						
**BPNSF17**	1.144	1.179	2.238					
**BPNSF23**	1.083	1.165	1.100	2.025				
**BPNSF3**	-1.068	-.940	-.930	-1.146	1.572			
**BPNSF9**	-1.074	-.991	-1.039	-1.162	.755	1.632		
**BPNSF14**	-1.016	-.936	-1.068	-1.118	.759	.897	1.730	
**BPNSF21**	-.845	-.876	-.773	-1.051	.557	.717	.863	1.770
**BPNSF2**	1.006	1.137	.869	1.170	-1.116	-1.062	-1.113	-1.016
**BPNSF8**	1.181	1.236	1.035	.912	-.772	-.997	-.808	-.575
**BPNSF20**	.879	.939	1.022	1.193	-.958	-.986	-1.046	-.893
**BPNSF22**	.684	.840	.617	.844	-.744	-.691	-.576	-.599
**BPNSF4**	-.668	-.527	-.508	-.514	.481	.299	.568	.270
**BPNSF12**	-.817	-.566	-.748	-.671	.431	.520	.695	.471
**BPNSF16**	-.868	-.726	-.816	-.842	.397	.553	.603	.520
**BPNSF24**	-.650	-.607	-.738	-.718	.437	.548	.435	.368
**BPNSF5**	.572	.710	.437	.446	-.334	-.443	-.372	-.458
**BPNSF10**	.638	1.131	.564	.547	-.402	-.544	-.398	-.481
**BPNSF15**	.390	.553	.355	.168	-.417	-.334	-.014	-.402
**BPNSF18**	-.343	-.115	-.066	-.227	.012	.086	-.142	.193
**BPNSF1**	-.478	-.428	-.264	-.406	.420	.224	.177	.394
**BPNSF7**	-.444	-.592	-.288	-.615	.373	.612	.459	.689
**BPNSF13**	-.833	-.762	-.771	-.855	.577	.664	1.070	.814
**BPNSF19**	-.745	-.870	-.871	-1.037	.561	.558	.818	.996

	BPNSF2	BPNSF8	BPNSF20	BPNSF22	BPNSF4	BPNSF12	BPNSF16	BPNS[illegible]
**BPNSF6**								
**BPNSF11**								
**BPNSF17**								
**BPNSF23**								
**BPNSF3**								
**BPNSF9**								
**BPNSF14**								
**BPNSF21**								
**BPNSF2**	2.389							
**BPNSF8**	1.133	2.418						
**BPNSF20**	1.116	1.029	2.013					
**BPNSF22**	.940	1.077	.918	1.856				
**BPNSF4**	-.739	-.537	-.597	-.502	1.312			
**BPNSF12**	-.793	-.705	-.792	-.745	.523	1.456		
**BPNSF16**	-.786	-.767	-.795	-.640	.505	.831	1.413	
**BPNSF24**	-.882	-.701	-.760	-.751	.443	.761	.675	1.3[illegible]
**BPNSF5**	.608	.513	.413	.420	-.163	-.571	-.639	-.5[illegible]
**BPNSF10**	.656	.811	.435	.337	-.434	-.647	-.557	-.4[illegible]
**BPNSF15**	.144	.421	.272	.249	-.286	-.537	-.294	-.3[illegible]
**BPNSF18**	-.121	-.046	-.242	-.055	-.112	-.097	.019	-.1[illegible]
**BPNSF1**	-.321	-.300	-.361	-.312	.446	.373	.294	.3[illegible]
**BPNSF7**	-.734	-.254	-.416	-.343	.141	.184	.161	.2[illegible]
**BPNSF13**	-.840	-.574	-.814	-.531	.424	.715	.567	.4[illegible]
**BPNSF19**	-1.004	-.625	-.934	-.586	.285	.571	.562	.4[illegible]

Sample Covariances - Upper Bounds (PC) (g1 - Measurement residuals)

	BPNSF6	BPNSF11	BPNSF17	BPNSF23	BPNSF3	BPNSF9	BPNSF14	BPNSF21	BPNSF2	BPNSF8	BPNSF20	BPNSF22	BPNSF4	BPNSF12	BPNSF16	BPNS[illegible]
**BPNSF6**	2.942															
**BPNSF11**	1.685	2.909														

	BPNSF6	BPNSF11	BPNSF17	BPNSF23	BPNSF3	BPNSF9	BPNSF14	BPNSF21	BPNSF2	BPNSF8	BPNSF20	BPNSF22	BPNSF4	BPNSF12	BPNSF16	BPNS[illegible]
**BPNSF17**	1.663	1.690	2.870													
**BPNSF23**	1.585	1.683	1.594	2.617												
**BPNSF3**	-.631	-.574	-.540	-.793	2.154											
**BPNSF9**	-.701	-.656	-.678	-.829	1.129	2.239										
**BPNSF14**	-.594	-.546	-.654	-.733	1.096	1.297	2.261									
**BPNSF21**	-.485	-.539	-.365	-.687	.913	1.099	1.332	2.292								
**BPNSF2**	1.496	1.646	1.325	1.664	-.753	-.687	-.719	-.634	2.911							
**BPNSF8**	1.697	1.728	1.570	1.428	-.382	-.587	-.450	-.213	1.636	2.942						
**BPNSF20**	1.427	1.436	1.562	1.702	-.617	-.607	-.622	-.514	1.533	1.533	2.583					
**BPNSF22**	1.101	1.260	1.016	1.266	-.428	-.373	-.234	-.223	1.323	1.503	1.293	2.260				
**BPNSF4**	-.321	-.195	-.202	-.216	.799	.599	.822	.583	-.367	-.185	-.317	-.213	1.639			
**BPNSF12**	-.398	-.209	-.397	-.373	.753	.892	1.128	.880	-.443	-.344	-.450	-.433	.802	1.923		
**BPNSF16**	-.449	-.379	-.447	-.485	.718	.905	1.006	.876	-.422	-.394	-.453	-.355	.775	1.160	1.817	
**BPNSF24**	-.308	-.282	-.365	-.389	.794	.926	.790	.716	-.555	-.362	-.465	-.435	.710	1.074	.985	1.7[illegible]
**BPNSF5**	1.003	1.133	.875	.841	-.011	-.135	-.062	-.110	.969	.981	.809	.770	.114	-.263	-.290	-.2[illegible]
**BPNSF10**	1.131	1.597	1.074	.988	-.016	-.161	.034	-.074	1.145	1.312	.869	.767	-.048	-.255	-.188	-.0[illegible]
**BPNSF15**	.796	.993	.777	.541	-.012	.036	.353	.014	.593	.912	.627	.638	.038	-.172	.100	-.0[illegible]
**BPNSF18**	.042	.275	.324	.109	.363	.426	.255	.578	.231	.386	.106	.320	.184	.297	.366	.2[illegible]
**BPNSF1**	-.083	-.101	.106	-.076	.792	.538	.544	.771	.025	.089	-.056	-.003	.746	.672	.574	.6[illegible]
**BPNSF7**	-.055	-.230	.084	-.312	.750	.989	.834	1.079	-.392	.117	-.060	-.006	.438	.562	.493	.5[illegible]
**BPNSF13**	-.433	-.360	-.410	-.488	.907	1.033	1.491	1.272	-.449	-.170	-.437	-.201	.727	1.144	.944	.8[illegible]
**BPNSF19**	-.375	-.486	-.491	-.654	.934	.941	1.239	1.468	-.604	-.185	-.519	-.247	.613	.916	.943	.7[illegible]

Sample Covariances - Two Tailed Significance (PC) (g1 - Measurement residuals)

	BPNSF6	BPNSF11	BPNSF17	BPNSF23	BPNSF3	BPNSF9	BPNSF14	BPNSF21	BPNSF2	BPNSF8	BPNSF20	BPNSF22	BPNSF4	BPNSF12	BPNSF16	BPNS[illegible]
**BPNSF6**	.010															
**BPNSF11**	.010	.010														
**BPNSF17**	.010	.010	.010													
**BPNSF23**	.010	.010	.010	.010												
**BPNSF3**	.010	.010	.010	.010	.010											
**BPNSF9**	.010	.010	.010	.010	.010	.010										
**BPNSF14**	.010	.010	.010	.010	.010	.010	.010									
**BPNSF21**	.010	.010	.010	.010	.010	.010	.010	.010								
**BPNSF2**	.010	.010	.010	.010	.010	.010	.010	.010	.010							
**BPNSF8**	.010	.010	.010	.010	.010	.010	.010	.010	.010	.010						
**BPNSF20**	.010	.010	.010	.010	.010	.010	.010	.010	.010	.010	.010					
**BPNSF22**	.010	.010	.010	.010	.010	.010	.010	.010	.010	.010	.010	.010				
**BPNSF4**	.010	.010	.010	.010	.010	.010	.010	.010	.010	.010	.010	.010	.010			
**BPNSF12**	.010	.010	.010	.010	.010	.010	.010	.010	.010	.010	.010	.010	.010	.010		
**BPNSF16**	.010	.010	.010	.010	.010	.010	.010	.010	.010	.010	.010	.010	.010	.010	.010	
**BPNSF24**	.010	.010	.010	.010	.010	.010	.010	.010	.010	.010	.010	.010	.010	.010	.010	.0[illegible]
**BPNSF5**	.010	.010	.010	.010	.077	.012	.044	.016	.010	.010	.010	.010	.721	.010	.010	.0[illegible]
**BPNSF10**	.010	.010	.010	.010	.077	.010	.193	.010	.010	.010	.010	.010	.039	.010	.010	.0[illegible]
**BPNSF15**	.010	.010	.010	.010	.094	.201	.157	.142	.010	.010	.010	.010	.196	.010	.371	.0[illegible]
**BPNSF18**	.207	.487	.403	.569	.078	.041	.589	.010	.702	.197	.400	.271	.783	.336	.078	.5[illegible]
**BPNSF1**	.027	.018	.456	.016	.010	.010	.010	.010	.146	.339	.033	.095	.010	.010	.010	.0[illegible]
**BPNSF7**	.025	.010	.268	.010	.010	.010	.010	.010	.010	.519	.010	.073	.010	.010	.016	.0[illegible]
**BPNSF13**	.010	.010	.010	.010	.010	.010	.010	.010	.010	.010	.010	.010	.010	.010	.010	.0[illegible]
**BPNSF19**	.010	.010	.010	.010	.010	.010	.010	.010	.010	.010	.010	.010	.010	.010	.010	.0[illegible]

Sample Correlations (g1 - Measurement residuals)

Sample Correlations - Lower Bounds (PC) (g1 - Measurement residuals)

	BPNSF6	BPNSF11	BPNSF17	BPNSF23	BPNSF3	BPNSF9	BPNSF14	BPNSF21	BPNSF2	BPNSF8	BPNSF20	BPNSF22	BPNSF4	BPNSF12	BPNSF16	BPNS[illegible]
**BPNSF6**	1.000															
**BPNSF11**	.469	1.000														
**BPNSF17**	.460	.467	1.000													
**BPNSF23**	.472	.506	.472	1.000												
**BPNSF3**	-.471	-.424	-.417	-.541	1.000											
**BPNSF9**	-.477	-.436	-.460	-.550	.403	1.000										
**BPNSF14**	-.444	-.394	-.456	-.501	.387	.474	1.000									
**BPNSF21**	-.360	-.375	-.341	-.465	.297	.376	.447	1.000								
**BPNSF2**	.395	.437	.335	.502	-.500	-.465	-.470	-.431	1.000							
**BPNSF8**	.458	.476	.412	.376	-.339	-.424	-.347	-.253	.446	1.000						
**BPNSF20**	.389	.398	.448	.542	-.452	-.456	-.475	-.418	.455	.430	1.000					
**BPNSF22**	.309	.363	.273	.409	-.378	-.340	-.287	-.291	.408	.474	.436	1.000				
**BPNSF4**	-.331	-.268	-.265	-.276	.290	.181	.347	.164	-.368	-.265	-.316	-.289	1.000			
**BPNSF12**	-.370	-.261	-.353	-.325	.247	.312	.391	.264	-.371	-.334	-.389	-.387	.343	1.000		
**BPNSF16**	-.407	-.342	-.383	-.426	.232	.326	.349	.306	-.371	-.363	-.411	-.343	.345	.526	1.000	

	BPNSF6	BPNSF11	BPNSF17	BPNSF23	BPNSF3	BPNSF9	BPNSF14	BPNSF21	BPNSF2	BPNSF8	BPNSF20	BPNSF22	BPNSF4	BPNSF12	BPNSF16	BPNS
**BPNSF24**	-.323	-.303	-.351	-.377	.270	.348	.248	.217	-.431	-.349	-.419	-.417	.296	.493	.446	1.0
**BPNSF5**	.237	.296	.177	.195	-.158	-.208	-.174	-.213	.246	.210	.178	.194	-.088	-.296	-.310	-.2
**BPNSF10**	.250	.440	.218	.220	-.179	-.245	-.174	-.205	.256	.318	.179	.152	-.226	-.302	-.267	-.2
**BPNSF15**	.156	.218	.142	.072	-.193	-.160	-.006	-.175	.059	.167	.120	.115	-.153	-.263	-.144	-.1
**BPNSF18**	-.143	-.048	-.029	-.106	.007	.042	-.067	.095	-.052	-.021	-.109	-.028	-.065	-.051	.010	-.0
**BPNSF1**	-.193	-.184	-.116	-.177	.218	.111	.085	.195	-.132	-.122	-.162	-.144	.256	.190	.155	.1
**BPNSF7**	-.180	-.240	-.121	-.269	.176	.297	.211	.319	-.306	-.104	-.180	-.154	.079	.099	.084	.1
**BPNSF13**	-.373	-.333	-.344	-.390	.311	.356	.589	.438	-.366	-.252	-.384	-.271	.266	.432	.336	.2
**BPNSF19**	-.309	-.374	-.379	-.450	.288	.302	.415	.536	-.426	-.269	-.416	-.287	.162	.317	.320	.2

Sample Correlations - Upper Bounds (PC) (g1 - Measurement residuals)

	BPNSF6	BPNSF11	BPNSF17	BPNSF23	BPNSF3	BPNSF9	BPNSF14	BPNSF21	BPNSF2	BPNSF8	BPNSF20	BPNSF22	BPNSF4	BPNSF12	BPNSF16	BPNS
**BPNSF6**	1.000															
**BPNSF11**	.617	1.000														
**BPNSF17**	.608	.617	1.000													
**BPNSF23**	.615	.635	.626	1.000												
**BPNSF3**	-.295	-.259	-.254	-.386	1.000											
**BPNSF9**	-.308	-.297	-.309	-.394	.578	1.000										
**BPNSF14**	-.264	-.233	-.294	-.346	.573	.635	1.000									
**BPNSF21**	-.213	-.227	-.166	-.334	.470	.536	.624	1.000								
**BPNSF2**	.557	.589	.496	.651	-.347	-.304	-.318	-.283	1.000							
**BPNSF8**	.607	.618	.579	.550	-.172	-.264	-.196	-.088	.596	1.000						
**BPNSF20**	.559	.555	.612	.691	-.297	-.305	-.294	-.245	.614	.597	1.000					
**BPNSF22**	.453	.516	.434	.554	-.217	-.192	-.110	-.111	.548	.619	.575	1.000				
**BPNSF4**	-.167	-.100	-.104	-.118	.471	.352	.451	.327	-.186	-.097	-.177	-.124	1.000			
**BPNSF12**	-.198	-.102	-.188	-.189	.418	.477	.571	.434	-.211	-.159	-.228	-.231	.508	1.000		
**BPNSF16**	-.224	-.188	-.223	-.259	.409	.499	.536	.466	-.201	-.192	-.236	-.193	.483	.668	1.000	
**BPNSF24**	-.155	-.141	-.189	-.210	.441	.518	.437	.399	-.272	-.178	-.250	-.247	.463	.629	.607	1.0
**BPNSF5**	.395	.442	.364	.354	-.005	-.057	-.029	-.051	.393	.382	.344	.359	.062	-.133	-.149	-.1
**BPNSF10**	.413	.582	.403	.384	-.007	-.071	.016	-.032	.413	.480	.339	.314	-.024	-.117	-.089	-.0
**BPNSF15**	.305	.381	.310	.223	-.006	.018	.158	.007	.230	.354	.264	.285	.020	-.088	.048	-.0
**BPNSF18**	.017	.119	.135	.049	.178	.218	.128	.279	.097	.159	.048	.144	.102	.152	.201	.1
**BPNSF1**	-.033	-.041	.042	-.034	.377	.272	.254	.359	.010	.038	-.026	-.001	.413	.354	.303	.3
**BPNSF7**	-.022	-.093	.033	-.132	.360	.454	.386	.478	-.153	.048	-.026	-.003	.243	.274	.245	.2
**BPNSF13**	-.195	-.156	-.180	-.234	.469	.528	.712	.592	-.202	-.076	-.217	-.109	.416	.600	.511	.4
**BPNSF19**	-.162	-.213	-.212	-.319	.466	.472	.589	.679	-.259	-.075	-.239	-.119	.346	.474	.496	.4

Sample Correlations - Two Tailed Significance (PC) (g1 - Measurement residuals)

	BPNSF6	BPNSF11	BPNSF17	BPNSF23	BPNSF3	BPNSF9	BPNSF14	BPNSF21	BPNSF2	BPNSF8	BPNSF20	BPNSF22	BPNSF4	BPNSF12	BPNSF16	BPNS
**BPNSF6**	...															
**BPNSF11**	.010	...														
**BPNSF17**	.010	.010	...													
**BPNSF23**	.010	.010	.010	...												
**BPNSF3**	.010	.010	.010	.010	...											
**BPNSF9**	.010	.010	.010	.010	.010	...										
**BPNSF14**	.010	.010	.010	.010	.010	.010	...									
**BPNSF21**	.010	.010	.010	.010	.010	.010	.010	...								
**BPNSF2**	.010	.010	.010	.010	.010	.010	.010	.010	...							
**BPNSF8**	.010	.010	.010	.010	.010	.010	.010	.010	.010	...						
**BPNSF20**	.010	.010	.010	.010	.010	.010	.010	.010	.010	.010	...					
**BPNSF22**	.010	.010	.010	.010	.010	.010	.010	.010	.010	.010	.010	...				
**BPNSF4**	.010	.010	.010	.010	.010	.010	.010	.010	.010	.010	.010	.010	...			
**BPNSF12**	.010	.010	.010	.010	.010	.010	.010	.010	.010	.010	.010	.010	.010	...		
**BPNSF16**	.010	.010	.010	.010	.010	.010	.010	.010	.010	.010	.010	.010	.010	.010	...	
**BPNSF24**	.010	.010	.010	.010	.010	.010	.010	.010	.010	.010	.010	.010	.010	.010	.010	
**BPNSF5**	.010	.010	.010	.010	.077	.012	.044	.016	.010	.010	.010	.010	.721	.010	.010	.0
**BPNSF10**	.010	.010	.010	.010	.077	.010	.193	.010	.010	.010	.010	.010	.039	.010	.010	.0
**BPNSF15**	.010	.010	.010	.010	.094	.201	.157	.142	.010	.010	.010	.010	.196	.010	.371	.0
**BPNSF18**	.207	.487	.403	.569	.078	.041	.589	.010	.702	.197	.400	.271	.783	.336	.078	.5
**BPNSF1**	.027	.017	.456	.016	.010	.010	.010	.010	.145	.339	.033	.095	.010	.010	.010	.0
**BPNSF7**	.025	.010	.268	.010	.010	.010	.010	.010	.010	.519	.010	.073	.010	.010	.015	.0
**BPNSF13**	.010	.010	.010	.010	.010	.010	.010	.010	.010	.010	.010	.010	.010	.010	.010	.0
**BPNSF19**	.010	.010	.010	.010	.010	.010	.010	.010	.010	.010	.010	.010	.010	.010	.010	.0

Sample Means (g1 - Measurement residuals)

Sample Means - Lower Bounds (PC) (g1 - Measurement residuals)

	BPNSF6	BPNSF11	BPNSF17	BPNSF23	BPNSF3	BPNSF9	BPNSF14	BPNSF21	BPNSF2	BPNSF8	BPNSF20	BPNSF22	BPNSF4	BPNSF12	BPNSF16	BPNSF[illegible]
**BPNSF6**	2.319	2.572	2.304	1.943	5.618	5.729	5.485	5.242	2.103	2.626	2.215	2.901	4.970	5.123	5.014	5.13[illegible]

Sample Means - Upper Bounds (PC) (g1 - Measurement residuals)

	BPNSF6	BPNSF11	BPNSF17	BPNSF23	BPNSF3	BPNSF9	BPNSF14	BPNSF21	BPNSF2	BPNSF8	BPNSF20	BPNSF22	BPNSF4	BPNSF12	BPNSF16	BPNSF[illegible]
**BPNSF6**	2.585	2.818	2.585	2.178	5.853	5.952	5.713	5.486	2.367	2.909	2.470	3.142	5.166	5.354	5.226	5.36[illegible]

Sample Means - Two Tailed Significance (PC) (g1 - Measurement residuals)

	BPNSF6	BPNSF11	BPNSF17	BPNSF23	BPNSF3	BPNSF9	BPNSF14	BPNSF21	BPNSF2	BPNSF8	BPNSF20	BPNSF22	BPNSF4	BPNSF12	BPNSF16	BPNSF[illegible]
**BPNSF6**	.010	.010	.010	.010	.010	.010	.010	.010	.010	.010	.010	.010	.010	.010	.010	.01[illegible]

Bias-corrected percentile method (g1 - Measurement residuals)

90% confidence intervals (bias-corrected percentile method)

Scalar Estimates (g1 - Measurement residuals)

Regression Weights: (g1 - Measurement residuals)

Parameter			Estimate	Lower	Upper	P
BPNSF19	<---	F1	1.000	1.000	1.000	...
BPNSF13	<---	F1	1.143	1.053	1.251	.006
BPNSF7	<---	F1	.863	.769	.962	.010
BPNSF1	<---	F1	.762	.630	.861	.019
BPNSF18	<---	F2	1.000	1.000	1.000	...
BPNSF15	<---	F2	2.428	1.988	3.270	.005
BPNSF10	<---	F2	3.040	2.429	4.137	.009
BPNSF5	<---	F2	2.544	2.058	3.497	.006
BPNSF24	<---	F3	1.000	1.000	1.000	...
BPNSF16	<---	F3	1.131	1.047	1.226	.008
BPNSF12	<---	F3	1.223	1.148	1.306	.006
BPNSF4	<---	F3	.856	.761	.941	.011
BPNSF22	<---	F4	1.000	1.000	1.000	...
BPNSF20	<---	F4	1.199	1.124	1.303	.005
BPNSF8	<---	F4	1.193	1.126	1.307	.004
BPNSF2	<---	F4	1.118	1.043	1.221	.006
BPNSF21	<---	F5	1.000	1.000	1.000	...
BPNSF14	<---	F5	1.094	1.032	1.184	.006
BPNSF9	<---	F5	1.040	.960	1.133	.008
BPNSF3	<---	F5	.883	.790	.978	.012
BPNSF23	<---	F6	1.000	1.000	1.000	...
BPNSF17	<---	F6	.974	.910	1.029	.016
BPNSF11	<---	F6	.946	.892	1.012	.006
BPNSF6	<---	F6	.848	.794	.912	.007

Standardized Regression Weights: (g1 - Measurement residuals)

Parameter			Estimate	Lower	Upper	P
BPNSF19	<---	F1	.630	.579	.673	.018
BPNSF13	<---	F1	.748	.714	.776	.009
BPNSF7	<---	F1	.542	.497	.603	.006
BPNSF1	<---	F1	.471	.412	.510	.016
BPNSF18	<---	F2	.263	.194	.322	.014
BPNSF15	<---	F2	.574	.534	.619	.008
BPNSF10	<---	F2	.718	.672	.755	.016
BPNSF5	<---	F2	.628	.584	.675	.009
BPNSF24	<---	F3	.687	.650	.721	.012
BPNSF16	<---	F3	.767	.729	.800	.012
BPNSF12	<---	F3	.821	.797	.845	.009
BPNSF4	<---	F3	.601	.560	.651	.006
BPNSF22	<---	F4	.685	.656	.716	.010
BPNSF20	<---	F4	.764	.726	.792	.019
BPNSF8	<---	F4	.740	.704	.775	.006
BPNSF2	<---	F4	.708	.674	.744	.007
BPNSF21	<---	F5	.705	.667	.738	.013
BPNSF14	<---	F5	.764	.710	.800	.016
BPNSF9	<---	F5	.726	.687	.762	.015
BPNSF3	<---	F5	.617	.552	.665	.020

Parameter	Estimate	Lower	Upper	P
BPNSF23 <--- F6	.791	.763	.820	.013
BPNSF17 <--- F6	.746	.706	.777	.015
BPNSF11 <--- F6	.735	.701	.769	.007
BPNSF6 <--- F6	.654	.621	.693	.004

Intercepts: (g1 - Measurement residuals)

Parameter	Estimate	Lower	Upper	P
**BPNSF19**	5.162	5.083	5.225	.023
**BPNSF13**	5.060	4.994	5.124	.016
**BPNSF7**	4.838	4.762	4.899	.021
**BPNSF1**	4.711	4.645	4.781	.014
**BPNSF18**	4.320	4.260	4.394	.008
**BPNSF15**	3.709	3.608	3.773	.018
**BPNSF10**	3.189	3.106	3.258	.010
**BPNSF5**	3.741	3.670	3.825	.006
**BPNSF24**	5.224	5.147	5.286	.020
**BPNSF16**	5.117	5.052	5.191	.013
**BPNSF12**	5.218	5.157	5.277	.018
**BPNSF4**	5.116	5.047	5.170	.015
**BPNSF22**	3.122	3.041	3.193	.009
**BPNSF20**	2.516	2.436	2.598	.006
**BPNSF8**	2.805	2.715	2.887	.009
**BPNSF2**	2.393	2.321	2.481	.005
**BPNSF21**	5.200	5.118	5.253	.034
**BPNSF14**	5.460	5.374	5.512	.032
**BPNSF9**	5.679	5.593	5.744	.020
**BPNSF3**	5.609	5.553	5.695	.008
**BPNSF23**	2.247	2.185	2.324	.004
**BPNSF17**	2.579	2.497	2.657	.006
**BPNSF11**	2.752	2.669	2.831	.008
**BPNSF6**	2.568	2.478	2.646	.012

Covariances: (g1 - Measurement residuals)

Parameter	Estimate	Lower	Upper	P
F1 <--> F2	-.158	-.203	-.123	.006
F2 <--> F3	-.152	-.193	-.115	.008
F1 <--> F3	.726	.648	.794	.011
F2 <--> F4	.321	.237	.407	.012
F3 <--> F4	-.598	-.664	-.517	.019
F1 <--> F4	-.486	-.558	-.416	.009
F2 <--> F5	-.145	-.185	-.109	.009
F4 <--> F5	-.665	-.743	-.602	.005
F3 <--> F5	.771	.688	.859	.011
F1 <--> F5	.896	.802	1.050	.005
F6 <--> F5	-.959	-1.085	-.880	.004
F6 <--> F3	-.642	-.715	-.553	.012
F6 <--> F4	1.272	1.166	1.392	.007
F6 <--> F2	.377	.281	.472	.012
F6 <--> F1	-.618	-.716	-.533	.009

Correlations: (g1 - Measurement residuals)

Parameter	Estimate	Lower	Upper	P
F1 <--> F2	-.434	-.490	-.354	.014
F2 <--> F3	-.413	-.478	-.359	.007
F1 <--> F3	.836	.800	.882	.004
F2 <--> F4	.763	.725	.800	.009
F3 <--> F4	-.596	-.637	-.540	.021
F1 <--> F4	-.491	-.541	-.433	.013
F2 <--> F5	-.363	-.412	-.286	.013
F4 <--> F5	-.611	-.651	-.563	.008
F3 <--> F5	.808	.765	.841	.011
F1 <--> F5	.953	.913	.993	.007
F6 <--> F5	-.722	-.765	-.678	.009
F6 <--> F3	-.524	-.566	-.466	.018
F6 <--> F4	.909	.878	.939	.008
F6 <--> F2	.734	.673	.771	.015
F6 <--> F1	-.511	-.560	-.447	.016

Variances: (g1 - Measurement residuals)

Parameter	Estimate	Lower	Upper	P
**F1**	.856	.719	.976	.015
**F2**	.155	.087	.236	.012
**F3**	.880	.762	.989	.012
**F4**	1.146	1.017	1.269	.011
**F5**	1.034	.908	1.179	.009
**F6**	1.709	1.565	1.899	.004
**e1**	1.298	1.153	1.482	.005
**e2**	.878	.791	.969	.010
**e3**	1.533	1.375	1.707	.007
**e4**	1.748	1.600	1.878	.007
**e5**	2.082	1.958	2.240	.006
**e6**	1.854	1.712	2.027	.005
**e7**	1.341	1.182	1.525	.007
**e8**	1.538	1.396	1.710	.007
**e9**	.982	.872	1.091	.006
**e10**	.788	.692	.907	.004
**e11**	.638	.573	.731	.004
**e12**	1.138	1.015	1.255	.014
**e13**	1.292	1.173	1.401	.010
**e14**	1.171	1.005	1.314	.010
**e15**	1.348	1.172	1.493	.023
**e16**	1.428	1.241	1.591	.015
**e17**	1.045	.927	1.156	.007
**e18**	.880	.743	1.079	.004
**e19**	1.005	.859	1.176	.005
**e20**	1.309	1.113	1.528	.006
**e21**	1.022	.895	1.166	.007
**e22**	1.293	1.129	1.486	.005
**e23**	1.300	1.128	1.446	.020
**e24**	1.642	1.475	1.810	.019

Matrices (g1 - Measurement residuals)

Sample Covariances (g1 - Measurement residuals)

Sample Covariances - Lower Bounds (BC) (g1 - Measurement residuals)

	BPNSF6	BPNSF11	BPNSF17	BPNSF23	BPNSF3	BPNSF9	BPNSF14	BPNSF21	BPNSF2	BPNSF8	BPNSF20	BPNSF22	BPNSF4	BPNSF12	BPNSF16	BPNS[illegible]
**BPNSF6**	2.397															
**BPNSF11**	1.225	2.460														
**BPNSF17**	1.121	1.186	2.247													
**BPNSF23**	1.087	1.221	1.115	2.069												
**BPNSF3**	-1.179	-.979	-.974	-1.230	1.656											
**BPNSF9**	-1.161	-1.197	-1.086	-1.237	.792	1.689										
**BPNSF14**	-1.043	-1.010	-1.078	-1.166	.770	.902	1.746									
**BPNSF21**	-.887	-.893	-.796	-1.079	.567	.734	.911	1.776								
**BPNSF2**	1.009	1.161	.906	1.156	-1.117	-1.091	-1.128	-.990	2.387							
**BPNSF8**	1.192	1.251	1.071	.921	-.874	-1.035	-.844	-.617	1.160	2.418						
**BPNSF20**	.974	.969	1.053	1.247	-1.006	-1.003	-1.093	-.914	1.145	1.065	2.026					
**BPNSF22**	.685	.863	.604	.848	-.821	-.709	-.582	-.620	.966	1.104	.936	1.861				
**BPNSF4**	-.702	-.549	-.552	-.519	.488	.321	.579	.282	-.757	-.633	-.628	-.512	1.318			
**BPNSF12**	-.823	-.569	-.747	-.637	.410	.515	.708	.525	-.776	-.753	-.766	-.746	.541	1.467		
**BPNSF16**	-.874	-.753	-.836	-.847	.398	.579	.634	.530	-.809	-.784	-.823	-.646	.506	.842	1.424	
**BPNSF24**	-.650	-.611	-.698	-.713	.459	.587	.435	.392	-.925	-.726	-.760	-.752	.448	.780	.675	1.3[illegible]
**BPNSF5**	.566	.756	.436	.458	-.324	-.451	-.370	-.458	.622	.537	.396	.454	-.195	-.571	-.667	-.5[illegible]
**BPNSF10**	.628	1.119	.577	.570	-.385	-.549	-.408	-.491	.701	.811	.438	.351	-.454	-.664	-.603	-.4[illegible]
**BPNSF15**	.369	.537	.355	.168	-.422	-.334	.012	-.402	.132	.421	.248	.218	-.313	-.587	-.308	-.3[illegible]
**BPNSF18**	-.384	-.161	-.103	-.260	-.001	.090	-.144	.179	-.113	-.134	-.243	-.074	-.098	-.091	.023	-.1[illegible]
**BPNSF1**	-.479	-.430	-.262	-.424	.460	.256	.202	.420	-.373	-.323	-.416	-.324	.472	.393	.320	.3[illegible]
**BPNSF7**	-.479	-.627	-.316	-.658	.388	.613	.479	.702	-.726	-.301	-.445	-.400	.150	.243	.203	.2[illegible]
**BPNSF13**	-.886	-.800	-.778	-.896	.580	.654	1.115	.845	-.832	-.612	-.832	-.537	.434	.715	.604	.4[illegible]
**BPNSF19**	-.754	-.870	-.939	-1.063	.615	.606	.849	1.014	-.997	-.674	-1.004	-.631	.307	.593	.613	.4[illegible]

Sample Covariances - Upper Bounds (BC) (g1 - Measurement residuals)

	BPNSF6	BPNSF11	BPNSF17	BPNSF23	BPNSF3	BPNSF9	BPNSF14	BPNSF21	BPNSF2	BPNSF8	BPNSF20	BPNSF22	BPNSF4	BPNSF12	BPNSF16	BPNS[illegible]
**BPNSF6**	2.992															
**BPNSF11**	1.781	3.010														

	BPNSF6	BPNSF11	BPNSF17	BPNSF23	BPNSF3	BPNSF9	BPNSF14	BPNSF21	BPNSF2	BPNSF8	BPNSF20	BPNSF22	BPNSF4	BPNSF12	BPNSF16	BPNS[illegible]
**BPNSF17**	1.621	1.707	2.876													
**BPNSF23**	1.602	1.695	1.599	2.658												
**BPNSF3**	-.710	-.598	-.603	-.819	2.220											
**BPNSF9**	-.756	-.736	-.704	-.848	1.174	2.344										
**BPNSF14**	-.635	-.578	-.659	-.773	1.108	1.312	2.339									
**BPNSF21**	-.549	-.555	-.404	-.713	.936	1.125	1.382	2.304								
**BPNSF2**	1.506	1.702	1.414	1.658	-.761	-.732	-.731	-.612	2.909							
**BPNSF8**	1.711	1.764	1.593	1.437	-.438	-.661	-.478	-.251	1.664	2.943						
**BPNSF20**	1.507	1.467	1.568	1.752	-.669	-.656	-.643	-.542	1.607	1.572	2.595					
**BPNSF22**	1.101	1.292	.994	1.272	-.467	-.411	-.254	-.277	1.359	1.563	1.330	2.270				
**BPNSF4**	-.343	-.224	-.225	-.216	.812	.611	.830	.599	-.378	-.254	-.329	-.227	1.656			
**BPNSF12**	-.409	-.213	-.397	-.316	.734	.882	1.147	.914	-.436	-.360	-.444	-.437	.831	1.928		
**BPNSF16**	-.471	-.399	-.468	-.487	.720	.974	1.037	.882	-.437	-.415	-.463	-.359	.794	1.178	1.827	
**BPNSF24**	-.308	-.294	-.355	-.387	.811	.972	.790	.723	-.575	-.391	-.465	-.440	.712	1.087	.985	[illegible]
**BPNSF5**	.987	1.163	.866	.845	.000	-.136	-.061	-.108	.997	.988	.792	.824	.104	-.263	-.315	[illegible]
**BPNSF10**	1.103	1.591	1.079	1.008	.000	-.167	.029	-.077	1.180	1.312	.883	.773	-.060	-.265	-.217	[illegible]
**BPNSF15**	.765	.992	.777	.541	-.016	.038	.419	.013	.581	.912	.613	.614	.023	-.200	.067	[illegible]
**BPNSF18**	.011	.242	.313	.106	.336	.448	.246	.555	.244	.342	.099	.300	.185	.310	.367	[illegible]
**BPNSF1**	-.094	-.111	.106	-.115	.865	.573	.568	.824	-.004	.026	-.098	-.035	.753	.710	.595	[illegible]
**BPNSF7**	-.091	-.263	.034	-.341	.771	.990	.853	1.082	-.358	.073	-.106	-.032	.453	.610	.518	[illegible]
**BPNSF13**	-.477	-.427	-.410	-.512	.928	1.016	1.585	1.295	-.447	-.233	-.492	-.229	.740	1.144	.980	[illegible]
**BPNSF19**	-.386	-.486	-.521	-.680	1.009	.975	1.306	1.489	-.588	-.240	-.584	-.255	.625	.952	1.036	[illegible]

Sample Covariances - Two Tailed Significance (BC) (g1 - Measurement residuals)

	BPNSF6	BPNSF11	BPNSF17	BPNSF23	BPNSF3	BPNSF9	BPNSF14	BPNSF21	BPNSF2	BPNSF8	BPNSF20	BPNSF22	BPNSF4	BPNSF12	BPNSF16	BPNS[illegible]
**BPNSF6**	.006															
**BPNSF11**	.003	.003														
**BPNSF17**	.014	.007	.008													
**BPNSF23**	.008	.005	.007	.005												
**BPNSF3**	.002	.005	.003	.004	.003											
**BPNSF9**	.002	.001	.005	.003	.003	.003										
**BPNSF14**	.004	.002	.008	.004	.007	.007	.005									
**BPNSF21**	.002	.006	.004	.005	.006	.005	.002	.006								
**BPNSF2**	.007	.004	.004	.012	.009	.005	.006	.018	.012							
**BPNSF8**	.007	.006	.005	.008	.002	.002	.004	.005	.006	.009						
**BPNSF20**	.003	.005	.006	.002	.003	.004	.005	.004	.003	.006	.006					
**BPNSF22**	.009	.006	.014	.009	.002	.003	.006	.003	.004	.005	.005	.009				
**BPNSF4**	.005	.005	.004	.009	.005	.006	.006	.007	.006	.002	.004	.005	.006			
**BPNSF12**	.007	.009	.011	.019	.016	.013	.006	.003	.013	.004	.012	.008	.005	.009		
**BPNSF16**	.007	.005	.004	.009	.009	.004	.004	.007	.006	.005	.006	.007	.007	.006	.007	
**BPNSF24**	.010	.007	.016	.012	.005	.005	.010	.006	.004	.004	.010	.008	.008	.007	.010	[illegible]
**BPNSF5**	.012	.004	.012	.008	.100	.010	.047	.019	.006	.007	.012	.004	.577	.010	.005	[illegible]
**BPNSF10**	.013	.012	.008	.007	.101	.008	.169	.007	.005	.010	.006	.007	.031	.009	.004	[illegible]
**BPNSF15**	.021	.014	.010	.011	.089	.210	.086	.136	.012	.010	.019	.025	.142	.005	.284	[illegible]
**BPNSF18**	.137	.732	.493	.535	.102	.036	.624	.019	.611	.362	.372	.330	.671	.299	.074	[illegible]
**BPNSF1**	.018	.013	.471	.008	.003	.004	.006	.005	.093	.174	.010	.046	.006	.004	.004	[illegible]
**BPNSF7**	.007	.004	.174	.002	.005	.008	.005	.008	.018	.250	.004	.021	.008	.003	.004	[illegible]
**BPNSF13**	.003	.003	.009	.005	.007	.014	.004	.005	.012	.002	.004	.005	.005	.010	.004	[illegible]
**BPNSF19**	.007	.010	.003	.006	.002	.004	.004	.006	.012	.004	.003	.006	.004	.006	.002	[illegible]

Sample Correlations (g1 - Measurement residuals)

Sample Correlations - Lower Bounds (BC) (g1 - Measurement residuals)

	BPNSF6	BPNSF11	BPNSF17	BPNSF23	BPNSF3	BPNSF9	BPNSF14	BPNSF21	BPNSF2	BPNSF8	BPNSF20	BPNSF22	BPNSF4	BPNSF12	BPNSF16	BPNS[illegible]
**BPNSF6**	1.000															
**BPNSF11**	.487	1.000														
**BPNSF17**	.444	.462	1.000													
**BPNSF23**	.479	.510	.472	1.000												
**BPNSF3**	-.498	-.425	-.430	-.546	1.000											
**BPNSF9**	-.496	-.455	-.465	-.550	.415	1.000										
**BPNSF14**	-.447	-.410	-.458	-.507	.379	.462	1.000									
**BPNSF21**	-.366	-.373	-.347	-.464	.311	.379	.447	1.000								
**BPNSF2**	.395	.448	.337	.488	-.500	-.465	-.465	-.425	1.000							
**BPNSF8**	.458	.468	.419	.375	-.348	-.434	-.361	-.261	.464	1.000						
**BPNSF20**	.412	.406	.458	.545	-.462	-.458	-.478	-.418	.458	.449	1.000					
**BPNSF22**	.309	.364	.257	.403	-.402	-.349	-.288	-.293	.430	.491	.441	1.000				
**BPNSF4**	-.334	-.273	-.279	-.264	.296	.183	.342	.168	-.370	-.283	-.331	-.291	1.000			
**BPNSF12**	-.371	-.261	-.341	-.314	.237	.309	.374	.281	-.370	-.346	-.383	-.387	.337	1.000		
**BPNSF16**	-.417	-.347	-.380	-.426	.220	.323	.341	.294	-.370	-.371	-.399	-.347	.345	.525	1.000	

	BPNSF6	BPNSF11	BPNSF17	BPNSF23	BPNSF3	BPNSF9	BPNSF14	BPNSF21	BPNSF2	BPNSF8	BPNSF20	BPNSF22	BPNSF4	BPNSF12	BPNSF16	BPNSI
**BPNSF24**	-.317	-.284	-.335	-.371	.261	.354	.244	.222	-.444	-.359	-.418	-.417	.298	.491	.430	1.0
**BPNSF5**	.235	.295	.174	.193	-.153	-.204	-.174	-.202	.243	.210	.163	.206	-.104	-.294	-.323	-.2
**BPNSF10**	.249	.440	.227	.232	-.178	-.239	-.175	-.204	.256	.319	.184	.152	-.234	-.303	-.285	-.2
**BPNSF15**	.152	.212	.138	.072	-.194	-.161	-.001	-.179	.064	.169	.106	.102	-.162	-.270	-.153	-.1
**BPNSF18**	-.159	-.069	-.048	-.113	.001	.051	-.067	.088	-.047	-.060	-.110	-.031	-.056	-.051	.014	-.0
**BPNSF1**	-.199	-.184	-.114	-.190	.225	.116	.074	.197	-.148	-.134	-.181	-.150	.256	.221	.164	.1
**BPNSF7**	-.208	-.253	-.129	-.281	.204	.297	.211	.317	-.296	-.123	-.190	-.176	.080	.114	.088	.1
**BPNSF13**	-.378	-.342	-.343	-.387	.311	.349	.592	.437	-.371	-.262	-.399	-.273	.266	.432	.353	.2
**BPNSF19**	-.312	-.373	-.390	-.448	.298	.302	.413	.521	-.416	-.286	-.422	-.287	.177	.317	.330	.2

Sample Correlations - Upper Bounds (BC) (g1 - Measurement residuals)

	BPNSF6	BPNSF11	BPNSF17	BPNSF23	BPNSF3	BPNSF9	BPNSF14	BPNSF21	BPNSF2	BPNSF8	BPNSF20	BPNSF22	BPNSF4	BPNSF12	BPNSF16	BPNSI
**BPNSF6**	1.000															
**BPNSF11**	.641	1.000														
**BPNSF17**	.600	.616	1.000													
**BPNSF23**	.629	.639	.627	1.000												
**BPNSF3**	-.315	-.264	-.256	-.401	1.000											
**BPNSF9**	-.329	-.308	-.310	-.394	.589	1.000										
**BPNSF14**	-.266	-.248	-.295	-.348	.563	.627	1.000									
**BPNSF21**	-.223	-.227	-.174	-.334	.479	.549	.624	1.000								
**BPNSF2**	.558	.592	.507	.637	-.348	-.304	-.312	-.276	1.000							
**BPNSF8**	.607	.615	.587	.549	-.177	-.277	-.204	-.106	.609	1.000						
**BPNSF20**	.582	.567	.617	.694	-.323	-.310	-.302	-.245	.614	.602	1.000					
**BPNSF22**	.453	.519	.421	.548	-.237	-.207	-.112	-.117	.559	.630	.580	1.000				
**BPNSF4**	-.168	-.114	-.116	-.109	.476	.352	.450	.343	-.193	-.119	-.193	-.125	1.000			
**BPNSF12**	-.204	-.102	-.185	-.162	.411	.477	.566	.443	-.206	-.168	-.225	-.231	.499	1.000		
**BPNSF16**	-.250	-.192	-.221	-.261	.398	.486	.532	.455	-.200	-.201	-.235	-.196	.484	.666	1.000	
**BPNSF24**	-.153	-.133	-.178	-.205	.427	.524	.430	.409	-.287	-.189	-.249	-.244	.467	.628	.599	1.0
**BPNSF5**	.393	.441	.349	.348	.004	-.052	-.029	-.041	.389	.383	.336	.368	.057	-.127	-.163	-.1
**BPNSF10**	.412	.582	.407	.388	-.007	-.069	.013	-.031	.412	.483	.350	.316	-.040	-.119	-.107	-.0
**BPNSF15**	.294	.371	.303	.223	-.009	.018	.173	.002	.233	.355	.256	.268	.018	-.091	.034	-.0
**BPNSF18**	.005	.101	.128	.046	.165	.223	.127	.266	.101	.143	.046	.144	.108	.152	.205	.1
**BPNSF1**	-.043	-.042	.045	-.047	.380	.275	.253	.360	-.002	.010	-.040	-.015	.413	.386	.304	.3
**BPNSF7**	-.035	-.103	.013	-.141	.372	.455	.386	.475	-.150	.030	-.034	-.012	.244	.280	.250	.2
**BPNSF13**	-.203	-.179	-.180	-.229	.469	.520	.713	.589	-.203	-.095	-.234	-.116	.416	.600	.516	.4
**BPNSF19**	-.166	-.208	-.224	-.316	.474	.472	.588	.673	-.256	-.101	-.242	-.119	.349	.474	.506	.4

Sample Correlations - Two Tailed Significance (BC) (g1 - Measurement residuals)

	BPNSF6	BPNSF11	BPNSF17	BPNSF23	BPNSF3	BPNSF9	BPNSF14	BPNSF21	BPNSF2	BPNSF8	BPNSF20	BPNSF22	BPNSF4	BPNSF12	BPNSF16	BPNSI
**BPNSF6**	...															
**BPNSF11**	.003	...														
**BPNSF17**	.019	.012	...													
**BPNSF23**	.004	.007	.009	...												
**BPNSF3**	.003	.008	.007	.005	...											
**BPNSF9**	.003	.004	.008	.010	.007	...										
**BPNSF14**	.008	.003	.009	.007	.016	.021	...									
**BPNSF21**	.005	.011	.006	.012	.005	.008	.010	...								
**BPNSF2**	.009	.006	.006	.023	.009	.010	.013	.014	...							
**BPNSF8**	.010	.016	.005	.012	.005	.006	.004	.005	.003	...						
**BPNSF20**	.002	.005	.004	.007	.004	.006	.007	.010	.009	.005	...					
**BPNSF22**	.010	.008	.019	.016	.003	.003	.009	.007	.004	.006	.005	...				
**BPNSF4**	.007	.005	.005	.018	.006	.008	.023	.006	.007	.002	.003	.009	...			
**BPNSF12**	.008	.010	.019	.023	.019	.011	.012	.006	.013	.006	.015	.010	.012	...		
**BPNSF16**	.005	.005	.012	.009	.014	.013	.012	.014	.011	.009	.013	.007	.009	.012	...	
**BPNSF24**	.012	.016	.021	.013	.015	.006	.013	.006	.005	.005	.012	.011	.009	.012	.019	
**BPNSF5**	.013	.011	.014	.012	.111	.015	.044	.023	.011	.009	.019	.005	.594	.012	.006	.0
**BPNSF10**	.011	.010	.006	.007	.081	.012	.177	.011	.011	.009	.005	.009	.027	.009	.003	.0
**BPNSF15**	.021	.026	.012	.010	.085	.192	.117	.111	.009	.009	.018	.026	.164	.009	.284	.0
**BPNSF18**	.144	.732	.525	.519	.096	.030	.606	.021	.611	.349	.372	.294	.670	.349	.070	.5
**BPNSF1**	.015	.016	.503	.009	.006	.007	.014	.009	.093	.166	.011	.049	.010	.003	.007	.0
**BPNSF7**	.007	.004	.174	.004	.005	.009	.009	.013	.012	.283	.006	.028	.009	.004	.012	.0
**BPNSF13**	.005	.004	.011	.012	.010	.019	.007	.011	.009	.004	.003	.006	.009	.010	.005	.0
**BPNSF19**	.007	.012	.005	.012	.005	.011	.011	.018	.013	.005	.007	.010	.007	.010	.005	.0

Sample Means (g1 - Measurement residuals)

Sample Means - Lower Bounds (BC) (g1 - Measurement residuals)

	BPNSF6	BPNSF11	BPNSF17	BPNSF23	BPNSF3	BPNSF9	BPNSF14	BPNSF21	BPNSF2	BPNSF8	BPNSF20	BPNSF22	BPNSF4	BPNSF12	BPNSF16	BPNSF[illegible]
**BPNSF6**	2.352	2.588	2.316	1.968	5.611	5.703	5.446	5.227	2.118	2.631	2.225	2.898	4.947	5.113	4.999	5.12[illegible]

Sample Means - Upper Bounds (BC) (g1 - Measurement residuals)

	BPNSF6	BPNSF11	BPNSF17	BPNSF23	BPNSF3	BPNSF9	BPNSF14	BPNSF21	BPNSF2	BPNSF8	BPNSF20	BPNSF22	BPNSF4	BPNSF12	BPNSF16	BPNSF[illegible]
**BPNSF6**	2.645	2.823	2.609	2.236	5.816	5.943	5.696	5.480	2.383	2.909	2.483	3.139	5.151	5.333	5.213	5.35[illegible]

Sample Means - Two Tailed Significance (BC) (g1 - Measurement residuals)

	BPNSF6	BPNSF11	BPNSF17	BPNSF23	BPNSF3	BPNSF9	BPNSF14	BPNSF21	BPNSF2	BPNSF8	BPNSF20	BPNSF22	BPNSF4	BPNSF12	BPNSF16	BPNSF[illegible]
**BPNSF6**	.003	.005	.005	.003	.016	.023	.020	.015	.005	.009	.006	.011	.032	.019	.019	.01[illegible]

g2 (g2 - Measurement residuals)

Estimates (g2 - Measurement residuals)

Scalar Estimates (g2 - Measurement residuals)

Maximum Likelihood Estimates

Regression Weights: (g2 - Measurement residuals)

			Estimate	S.E.	C.R.	P	Label
BPNSF19	<---	F1	1.000				
BPNSF13	<---	F1	1.143	.052	21.904	***	a1_1
BPNSF7	<---	F1	.863	.051	16.943	***	a2_1
BPNSF1	<---	F1	.762	.051	14.998	***	a3_1
BPNSF18	<---	F2	1.000				
BPNSF15	<---	F2	2.428	.301	8.066	***	a4_1
BPNSF10	<---	F2	3.040	.365	8.335	***	a5_1
BPNSF5	<---	F2	2.544	.311	8.191	***	a6_1
BPNSF24	<---	F3	1.000				
BPNSF16	<---	F3	1.131	.047	24.240	***	a7_1
BPNSF12	<---	F3	1.223	.048	25.542	***	a8_1
BPNSF4	<---	F3	.856	.044	19.522	***	a9_1
BPNSF22	<---	F4	1.000				
BPNSF20	<---	F4	1.199	.049	24.508	***	a10_1
BPNSF8	<---	F4	1.193	.050	23.824	***	a11_1
BPNSF2	<---	F4	1.118	.049	22.901	***	a12_1
BPNSF21	<---	F5	1.000				
BPNSF14	<---	F5	1.094	.042	25.847	***	a13_1
BPNSF9	<---	F5	1.040	.042	24.600	***	a14_1
BPNSF3	<---	F5	.883	.042	21.014	***	a15_1
BPNSF23	<---	F6	1.000				
BPNSF17	<---	F6	.974	.034	28.266	***	a16_1
BPNSF11	<---	F6	.946	.034	27.785	***	a17_1
BPNSF6	<---	F6	.848	.035	24.209	***	a18_1

Standardized Regression Weights: (g2 - Measurement residuals)

			Estimate
BPNSF19	<---	F1	.630
BPNSF13	<---	F1	.748
BPNSF7	<---	F1	.542
BPNSF1	<---	F1	.471
BPNSF18	<---	F2	.263
BPNSF15	<---	F2	.574
BPNSF10	<---	F2	.718
BPNSF5	<---	F2	.628
BPNSF24	<---	F3	.687
BPNSF16	<---	F3	.767
BPNSF12	<---	F3	.821
BPNSF4	<---	F3	.601
BPNSF22	<---	F4	.685
BPNSF20	<---	F4	.764
BPNSF8	<---	F4	.740
BPNSF2	<---	F4	.708
BPNSF21	<---	F5	.705
BPNSF14	<---	F5	.764

			Estimate
BPNSF9	<---	F5	.726
BPNSF3	<---	F5	.617
BPNSF23	<---	F6	.791
BPNSF17	<---	F6	.746
BPNSF11	<---	F6	.735
BPNSF6	<---	F6	.654

Intercepts: (g2 - Measurement residuals)

	Estimate	S.E.	C.R.	P	Label
**BPNSF19**	5.162	.041	127.012	***	i1_1
**BPNSF13**	5.060	.039	129.318	***	i2_1
**BPNSF7**	4.838	.041	118.588	***	i3_1
**BPNSF1**	4.711	.041	113.532	***	i4_1
**BPNSF18**	4.320	.041	104.324	***	i5_1
**BPNSF15**	3.709	.046	80.542	***	i6_1
**BPNSF10**	3.189	.046	69.185	***	i7_1
**BPNSF5**	3.741	.044	84.780	***	i8_1
**BPNSF24**	5.224	.038	138.282	***	i9_1
**BPNSF16**	5.117	.038	133.590	***	i10_1
**BPNSF12**	5.218	.039	134.827	***	i11_1
**BPNSF4**	5.116	.037	138.378	***	i12_1
**BPNSF22**	3.122	.043	72.197	***	i13_1
**BPNSF20**	2.516	.046	54.118	***	i14_1
**BPNSF8**	2.805	.048	58.684	***	i15_1
**BPNSF2**	2.393	.047	51.088	***	i16_1
**BPNSF21**	5.200	.040	130.235	***	i17_1
**BPNSF14**	5.460	.040	135.532	***	i18_1
**BPNSF9**	5.679	.040	140.700	***	i19_1
**BPNSF3**	5.609	.040	139.288	***	i20_1
**BPNSF23**	2.247	.046	49.100	***	i21_1
**BPNSF17**	2.579	.047	54.570	***	i22_1
**BPNSF11**	2.752	.047	59.079	***	i23_1
**BPNSF6**	2.568	.047	54.729	***	i24_1

Covariances: (g2 - Measurement residuals)

			Estimate	S.E.	C.R.	P	Label
F1	<-->	F2	-.158	.024	-6.630	***	ccc1_1
F2	<-->	F3	-.152	.023	-6.704	***	ccc2_1
F1	<-->	F3	.726	.048	14.975	***	ccc3_1
F2	<-->	F4	.321	.042	7.723	***	ccc4_1
F3	<-->	F4	-.598	.045	-13.357	***	ccc5_1
F1	<-->	F4	-.486	.043	-11.201	***	ccc6_1
F2	<-->	F5	-.145	.023	-6.365	***	ccc7_1
F4	<-->	F5	-.665	.049	-13.651	***	ccc8_1
F3	<-->	F5	.771	.049	15.737	***	ccc9_1
F1	<-->	F5	.896	.056	16.010	***	ccc10_1
F6	<-->	F5	-.959	.061	-15.831	***	ccc11_1
F6	<-->	F3	-.642	.050	-12.886	***	ccc12_1
F6	<-->	F4	1.272	.073	17.405	***	ccc13_1
F6	<-->	F2	.377	.048	7.795	***	ccc14_1
F6	<-->	F1	-.618	.052	-11.931	***	ccc15_1

Correlations: (g2 - Measurement residuals)

			Estimate
F1	<-->	F2	-.434
F2	<-->	F3	-.413
F1	<-->	F3	.836
F2	<-->	F4	.763
F3	<-->	F4	-.596
F1	<-->	F4	-.491
F2	<-->	F5	-.363
F4	<-->	F5	-.611
F3	<-->	F5	.808
F1	<-->	F5	.953
F6	<-->	F5	-.722
F6	<-->	F3	-.524
F6	<-->	F4	.909

			Estimate
F6	<-->	F2	.734
F6	<-->	F1	-.511

Variances: (g2 - Measurement residuals)

	Estimate	S.E.	C.R.	P	Label
**F1**	.856	.072	11.898	***	vvv1_1
**F2**	.155	.036	4.287	***	vvv2_1
**F3**	.880	.066	13.358	***	vvv3_1
**F4**	1.146	.085	13.419	***	vvv4_1
**F5**	1.034	.073	14.069	***	vvv5_1
**F6**	1.709	.104	16.468	***	vvv6_1
**e1**	1.298	.057	22.852	***	v1_1
**e2**	.878	.045	19.534	***	v2_1
**e3**	1.533	.064	23.941	***	v3_1
**e4**	1.748	.071	24.477	***	v4_1
**e5**	2.082	.083	25.017	***	v5_1
**e6**	1.854	.084	22.006	***	v6_1
**e7**	1.341	.076	17.533	***	v7_1
**e8**	1.538	.074	20.806	***	v8_1
**e9**	.982	.044	22.064	***	v9_1
**e10**	.788	.040	19.923	***	v10_1
**e11**	.638	.037	17.379	***	v11_1
**e12**	1.138	.049	23.364	***	v12_1
**e13**	1.292	.057	22.516	***	v13_1
**e14**	1.171	.057	20.631	***	v14_1
**e15**	1.348	.063	21.354	***	v15_1
**e16**	1.428	.065	22.100	***	v16_1
**e17**	1.045	.046	22.682	***	v17_1
**e18**	.880	.041	21.286	***	v18_1
**e19**	1.005	.045	22.279	***	v19_1
**e20**	1.309	.055	23.832	***	v20_1
**e21**	1.022	.050	20.293	***	v21_1
**e22**	1.293	.060	21.690	***	v22_1
**e23**	1.300	.059	21.938	***	v23_1
**e24**	1.642	.070	23.290	***	v24_1

Matrices (g2 - Measurement residuals)

Residual Covariances (g2 - Measurement residuals)

	BPNSF6	BPNSF11	BPNSF17	BPNSF23	BPNSF3	BPNSF9	BPNSF14	BPNSF21	BPNSF2	BPNSF8	BPNSF20	BPNSF22	BPNSF4	BPNSF12	BPNSF16	BPNS[illegible]
**BPNSF6**	.063															
**BPNSF11**	-.187	.061														
**BPNSF17**	-.067	-.036	-.019													
**BPNSF23**	-.128	-.177	.072	-.002												
**BPNSF3**	-.136	.136	-.039	-.041	.161											
**BPNSF9**	.063	.015	-.183	-.049	.180	.075										
**BPNSF14**	.214	.208	-.107	.192	-.020	.077	-.098									
**BPNSF21**	.115	.059	.152	.070	.136	-.082	-.013	.146								
**BPNSF2**	-.018	-.229	-.020	-.050	-.109	.025	.011	-.010	-.251							
**BPNSF8**	.004	-.182	-.052	-.207	.018	.033	.156	.041	-.048	.074						
**BPNSF20**	-.218	-.151	-.006	.214	-.147	.004	-.016	-.201	-.024	-.029	.099					
**BPNSF22**	-.078	-.040	.056	.027	.126	-.068	.014	-.155	.025	.039	.235	.302				
**BPNSF4**	-.179	-.071	-.132	-.150	.822	.188	.104	.430	-.284	-.165	-.344	-.252	.370			
**BPNSF12**	.089	.035	-.086	-.011	.047	-.004	.024	.137	-.086	.028	-.053	-.307	.224	.098		
**BPNSF16**	.098	-.007	-.075	.032	-.012	-.051	.075	.304	-.052	.006	-.188	-.285	.174	.198	.243	
**BPNSF24**	-.004	-.040	-.295	-.087	-.052	-.012	.131	.210	-.212	-.191	-.337	-.537	.181	.110	.255	[illegible]
**BPNSF5**	.087	-.245	-.199	-.065	-.117	.061	.047	-.148	-.078	.143	.034	.135	-.394	-.014	.039	[illegible]
**BPNSF10**	-.086	.317	.086	.104	-.239	-.314	-.259	-.447	.025	.171	.202	.302	-.312	-.133	-.129	[illegible]
**BPNSF15**	.090	.300	.108	-.043	-.084	.049	.086	-.095	-.017	.169	.178	.387	-.213	-.037	-.026	[illegible]
**BPNSF18**	-.102	.284	.214	-.011	.140	.021	.087	-.102	-.007	.120	.277	.200	-.040	.010	-.030	[illegible]
**BPNSF1**	.113	.095	.086	.059	.213	-.048	.017	.211	-.225	-.031	-.188	-.171	.390	.031	.013	[illegible]
**BPNSF7**	.412	-.009	-.165	.193	.008	.145	.005	.039	-.028	-.106	-.025	-.280	.227	-.014	.040	[illegible]
**BPNSF13**	.066	-.108	-.015	.026	.002	-.145	.017	.191	-.013	-.118	-.113	-.284	.207	.190	.101	[illegible]
**BPNSF19**	-.084	-.270	-.296	-.254	-.019	.048	.052	.257	-.018	-.114	-.261	-.311	.199	-.064	-.045	[illegible]

Residual Means (g2 - Measurement residuals)

	BPNSF6	BPNSF11	BPNSF17	BPNSF23	BPNSF3	BPNSF9	BPNSF14	BPNSF21	BPNSF2	BPNSF8	BPNSF20	BPNSF22	BPNSF4	BPNSF12	BPNSF16	BPNSF24	BP[illegible]

	BPNSF6	BPNSF11	BPNSF17	BPNSF23	BPNSF3	BPNSF9	BPNSF14	BPNSF21	BPNSF2	BPNSF8	BPNSF20	BPNSF22	BPNSF4	BPNSF12	BPNSF16	BPNSF24	BPN
	.048	.009	-.105	-.074	.010	.046	.147	.040	-.084	-.073	-.077	-.043	-.039	.097	.101	.087	-.

Standardized Residual Covariances (g2 - Measurement residuals)

	BPNSF6	BPNSF11	BPNSF17	BPNSF23	BPNSF3	BPNSF9	BPNSF14	BPNSF21	BPNSF2	BPNSF8	BPNSF20	BPNSF22	BPNSF4	BPNSF12	BPNSF16	BPNS
**BPNSF6**	.323															
**BPNSF11**	-1.232	.320														
**BPNSF17**	-.432	-.231	-.098													
**BPNSF23**	-.848	-1.152	.458	-.013												
**BPNSF3**	-1.106	1.103	-.308	-.338	1.121											
**BPNSF9**	.504	.119	-1.432	-.391	1.617	.524										
**BPNSF14**	1.701	1.644	-.834	1.525	-.175	.667	-.684									
**BPNSF21**	.931	.479	1.204	.573	1.241	-.728	-.117	1.035								
**BPNSF2**	-.122	-1.523	-.131	-.333	-.894	.202	.089	-.086	-1.294							
**BPNSF8**	.026	-1.170	-.327	-1.337	.147	.261	1.230	.325	-.305	.368						
**BPNSF20**	-1.458	-.993	-.036	1.413	-1.211	.034	-.132	-1.648	-.155	-.179	.520					
**BPNSF22**	-.568	-.293	.397	.199	1.118	-.596	.118	-1.380	.179	.266	1.658	1.827				
**BPNSF4**	-1.614	-.647	-1.178	-1.374	8.468	1.900	1.051	4.412	-2.544	-1.448	-3.094	-2.447	3.067			
**BPNSF12**	.752	.297	-.721	-.092	.452	-.036	.224	1.288	-.716	.226	-.438	-2.781	2.244	.740		
**BPNSF16**	.845	-.057	-.636	.279	-.118	-.483	.706	2.913	-.438	.050	-1.597	-2.628	1.785	1.807	1.876	
**BPNSF24**	-.032	-.351	-2.552	-.776	-.513	-.113	1.265	2.071	-1.842	-1.625	-2.934	-5.065	1.923	1.050	2.501	2.3
**BPNSF5**	.642	-1.806	-1.442	-.484	-1.043	.539	.415	-1.331	-.571	1.023	.251	1.078	-3.825	-.128	.363	-1.2
**BPNSF10**	-.599	2.203	.587	.726	-2.039	-2.655	-2.193	-3.827	.176	1.153	1.391	2.275	-2.888	-1.164	-1.143	-2.6
**BPNSF15**	.645	2.136	.761	-.310	-.720	.419	.737	-.818	-.121	1.169	1.263	2.980	-1.982	-.323	-.233	-1.8
**BPNSF18**	-.838	2.332	1.731	-.096	1.340	.201	.832	-.983	-.054	.964	2.280	1.776	-.414	.100	-.302	-.8
**BPNSF1**	.917	.776	.694	.490	1.965	-.434	.157	1.945	-1.833	-.244	-1.540	-1.507	3.959	.296	.123	.9
**BPNSF7**	3.389	-.075	-1.345	1.615	.075	1.324	.044	.365	-.233	-.852	-.203	-2.500	2.329	-.129	.392	1.1
**BPNSF13**	.556	-.914	-.126	.220	.022	-1.304	.155	1.751	-.109	-.975	-.961	-2.608	2.148	1.788	.977	1.7
**BPNSF19**	-.693	-2.224	-2.396	-2.123	-.174	.430	.461	2.338	-.151	-.913	-2.149	-2.769	2.018	-.602	-.434	-.5

Standardized Residual Means (g2 - Measurement residuals)

	BPNSF6	BPNSF11	BPNSF17	BPNSF23	BPNSF3	BPNSF9	BPNSF14	BPNSF21	BPNSF2	BPNSF8	BPNSF20	BPNSF22	BPNSF4	BPNSF12	BPNSF16	BPNSF24	BPN
	.590	.111	-1.285	-.933	.144	.656	2.106	.575	-1.039	-.885	-.952	-.578	-.611	1.442	1.531	1.327	-1.

Notes for Group/Model (g2 - Measurement residuals)

The following covariance matrix is not positive definite (g2 - Measurement residuals)

	F5	F4	F3	F2	F1	F6
**F5**	1.034					
**F4**	-.665	1.146				
**F3**	.771	-.598	.880			
**F2**	-.145	.321	-.152	.155		
**F1**	.896	-.486	.726	-.158	.856	
**F6**	-.959	1.272	-.642	.377	-.618	1.709

This solution is not admissible.

Modification Indices (g2 - Measurement residuals)

Covariances: (g2 - Measurement residuals)

			M.I.	Par Change
e24	<-->	F1	5.152	.100
e23	<-->	F5	4.602	.079
e23	<-->	F4	6.938	-.109
e23	<-->	F1	5.314	-.092
e20	<-->	e24	8.183	-.215
e20	<-->	e23	5.282	.157
e19	<-->	e20	6.033	.146
e18	<-->	e22	8.933	-.175
e18	<-->	e19	5.763	.121
e17	<-->	F2	5.938	-.045
e17	<-->	F1	7.212	.093
e17	<-->	F6	6.921	.111
e17	<-->	e22	14.590	.238
e17	<-->	e19	10.402	-.174
e15	<-->	e24	4.501	.169
e15	<-->	e21	7.678	-.183

			M.I.	Par Change
e14	<-->	e24	9.165	-.228
e14	<-->	e21	17.245	.259
e13	<-->	F5	11.222	.122
e13	<-->	F3	10.088	-.127
e13	<-->	F1	6.757	-.103
e13	<-->	e20	17.660	.284
e12	<-->	F5	20.762	.154
e12	<-->	F3	4.546	-.078
e12	<-->	F2	9.327	-.059
e12	<-->	F6	7.635	.122
e12	<-->	e22	5.773	.155
e12	<-->	e20	119.420	.683
e12	<-->	e18	8.892	-.159
e12	<-->	e17	5.249	.130
e11	<-->	e14	4.904	.117
e10	<-->	F3	4.936	.069
e10	<-->	e20	7.220	-.149
e10	<-->	e17	7.853	.141
e10	<-->	e12	5.195	-.118
e9	<-->	F4	8.486	-.106
e9	<-->	e22	4.155	-.125
e9	<-->	e20	11.608	-.202
e9	<-->	e13	12.295	-.212
e8	<-->	e24	7.946	.240
e8	<-->	e23	13.889	-.290
e8	<-->	e15	4.410	.168
e8	<-->	e12	14.443	-.270
e7	<-->	F5	14.494	-.152
e7	<-->	e24	6.308	-.211
e7	<-->	e23	6.230	.191
e7	<-->	e19	4.035	-.134
e7	<-->	e17	5.601	-.160
e6	<-->	e23	6.387	.212
e6	<-->	e21	4.776	-.167
e5	<-->	e24	4.814	-.204
e5	<-->	e23	4.952	.188
e5	<-->	e20	4.233	.169
e5	<-->	e6	7.215	.269
e4	<-->	F5	4.949	.090
e4	<-->	F2	4.643	-.050
e4	<-->	F6	12.696	.192
e4	<-->	e22	4.297	.163
e4	<-->	e20	5.718	.181
e4	<-->	e17	4.035	.139
e4	<-->	e16	5.285	-.188
e4	<-->	e12	7.408	.194
e4	<-->	e7	5.238	.194
e4	<-->	e6	14.011	-.347
e4	<-->	e5	6.165	-.233
e3	<-->	F6	4.819	.111
e3	<-->	e24	19.025	.354
e3	<-->	e22	5.508	-.174
e3	<-->	e21	6.551	.173
e3	<-->	e19	8.209	.184
e3	<-->	e13	5.377	-.169
e2	<-->	F3	7.251	.090
e2	<-->	e23	4.743	-.129
e2	<-->	e19	10.377	-.165
e2	<-->	e12	5.007	-.121
e2	<-->	e11	12.237	.158
e1	<-->	F3	8.220	-.113
e1	<-->	F6	8.916	-.142
e1	<-->	e17	4.220	.125
e1	<-->	e16	5.052	.162
e1	<-->	e2	4.235	-.118

Variances: (g2 - Measurement residuals)

	M.I.	Par Change
**e23**	8.789	.297
**e4**	6.739	-.318

	M.I.	Par Change
e1	5.595	.226

Regression Weights: (g2 - Measurement residuals)

			M.I.	Par Change
BPNSF6	<---	F3	4.231	.150
BPNSF6	<---	F1	5.203	.168
BPNSF6	<---	BPNSF14	4.303	.023
BPNSF6	<---	BPNSF16	4.210	.025
BPNSF6	<---	BPNSF24	4.283	.024
BPNSF6	<---	BPNSF5	4.054	.032
BPNSF6	<---	BPNSF1	4.478	.027
BPNSF6	<---	BPNSF7	8.709	.037
BPNSF11	<---	BPNSF15	4.055	.029
BPNSF3	<---	BPNSF6	4.431	-.039
BPNSF14	<---	BPNSF6	5.640	.037
BPNSF14	<---	BPNSF11	7.186	.040
BPNSF14	<---	BPNSF23	7.542	.047
BPNSF14	<---	BPNSF8	5.432	.034
BPNSF14	<---	BPNSF22	6.215	.034
BPNSF14	<---	BPNSF15	5.281	.027
BPNSF14	<---	BPNSF18	4.071	.021
BPNSF21	<---	F3	7.432	.160
BPNSF21	<---	F1	5.989	.144
BPNSF22	<---	F3	12.088	-.228
BPNSF22	<---	F2	8.132	.469
BPNSF22	<---	F1	6.193	-.165
BPNSF4	<---	F5	12.041	.190
BPNSF4	<---	F4	5.784	-.127
BPNSF4	<---	F3	6.565	.154
BPNSF4	<---	F2	6.589	-.391
BPNSF4	<---	F1	16.905	.251
BPNSF4	<---	F6	7.982	-.121
BPNSF4	<---	BPNSF6	9.700	-.054
BPNSF4	<---	BPNSF11	4.694	-.036
BPNSF4	<---	BPNSF23	6.417	-.048
BPNSF4	<---	BPNSF2	9.355	-.055
BPNSF4	<---	BPNSF8	6.059	-.040
BPNSF4	<---	BPNSF20	9.586	-.054
BPNSF4	<---	BPNSF5	11.978	-.045
BPNSF4	<---	BPNSF10	6.832	-.039
BPNSF4	<---	BPNSF15	6.251	-.033
BPNSF24	<---	F4	7.640	-.138
BPNSF24	<---	F2	5.973	-.353
BPNSF5	<---	BPNSF11	5.666	-.047
BPNSF10	<---	F5	12.092	-.226
BPNSF10	<---	F3	4.512	-.153
BPNSF10	<---	F1	11.078	-.241
BPNSF10	<---	BPNSF11	4.841	.043
BPNSF15	<---	F2	4.175	.397
BPNSF18	<---	F2	4.390	.417
BPNSF18	<---	BPNSF6	7.788	-.063
BPNSF18	<---	BPNSF23	5.006	-.056
BPNSF18	<---	BPNSF3	5.158	-.027
BPNSF18	<---	BPNSF9	6.820	-.031
BPNSF18	<---	BPNSF14	6.313	-.031
BPNSF18	<---	BPNSF21	7.915	-.036
BPNSF18	<---	BPNSF2	4.683	-.051
BPNSF18	<---	BPNSF4	6.602	-.034
BPNSF18	<---	BPNSF12	6.988	-.034
BPNSF18	<---	BPNSF16	7.654	-.036
BPNSF18	<---	BPNSF24	7.695	-.036
BPNSF18	<---	BPNSF10	4.905	-.043
BPNSF18	<---	BPNSF1	10.736	-.046
BPNSF18	<---	BPNSF7	7.086	-.037
BPNSF18	<---	BPNSF13	6.501	-.034
BPNSF18	<---	BPNSF19	6.990	-.034
BPNSF1	<---	BPNSF17	4.027	.042
BPNSF1	<---	BPNSF4	4.390	.026
BPNSF19	<---	F2	4.393	-.341
BPNSF19	<---	F6	7.212	-.123

Means: (g2 - Measurement residuals)

	M.I.	Par Change

Intercepts: (g2 - Measurement residuals)

	M.I.	Par Change
**BPNSF18**	7.665	-.193

Bootstrap (g2 - Measurement residuals)

Bootstrap standard errors (g2 - Measurement residuals)

Scalar Estimates (g2 - Measurement residuals)

Regression Weights: (g2 - Measurement residuals)

Parameter			SE	SE-SE	Mean	Bias	SE-Bias
BPNSF19	<---	F1	.000	.000	1.000	.000	.000
BPNSF13	<---	F1	.057	.003	1.140	-.004	.004
BPNSF7	<---	F1	.059	.003	.862	-.001	.004
BPNSF1	<---	F1	.071	.004	.761	-.002	.005
BPNSF18	<---	F2	.000	.000	1.000	.000	.000
BPNSF15	<---	F2	.376	.019	2.472	.044	.027
BPNSF10	<---	F2	.495	.025	3.100	.060	.035
BPNSF5	<---	F2	.411	.021	2.595	.051	.029
BPNSF24	<---	F3	.000	.000	1.000	.000	.000
BPNSF16	<---	F3	.053	.003	1.131	.000	.004
BPNSF12	<---	F3	.049	.002	1.219	-.003	.003
BPNSF4	<---	F3	.051	.003	.854	-.002	.004
BPNSF22	<---	F4	.000	.000	1.000	.000	.000
BPNSF20	<---	F4	.051	.003	1.197	-.002	.004
BPNSF8	<---	F4	.049	.002	1.187	-.006	.003
BPNSF2	<---	F4	.054	.003	1.117	-.002	.004
BPNSF21	<---	F5	.000	.000	1.000	.000	.000
BPNSF14	<---	F5	.045	.002	1.094	.001	.003
BPNSF9	<---	F5	.053	.003	1.042	.001	.004
BPNSF3	<---	F5	.059	.003	.885	.002	.004
BPNSF23	<---	F6	.000	.000	1.000	.000	.000
BPNSF17	<---	F6	.034	.002	.976	.003	.002
BPNSF11	<---	F6	.038	.002	.947	.000	.003
BPNSF6	<---	F6	.036	.002	.847	-.001	.003

Standardized Regression Weights: (g2 - Measurement residuals)

Parameter			SE	SE-SE	Mean	Bias	SE-Bias
BPNSF19	<---	F1	.028	.001	.632	.002	.002
BPNSF13	<---	F1	.018	.001	.747	-.002	.001
BPNSF7	<---	F1	.031	.002	.542	.000	.002
BPNSF1	<---	F1	.031	.002	.470	-.001	.002
BPNSF18	<---	F2	.039	.002	.264	.001	.003
BPNSF15	<---	F2	.027	.001	.574	.000	.002
BPNSF10	<---	F2	.026	.001	.719	.000	.002
BPNSF5	<---	F2	.025	.001	.628	.000	.002
BPNSF24	<---	F3	.021	.001	.688	.001	.001
BPNSF16	<---	F3	.021	.001	.768	.001	.001
BPNSF12	<---	F3	.014	.001	.821	.000	.001
BPNSF4	<---	F3	.027	.001	.599	-.002	.002
BPNSF22	<---	F4	.019	.001	.685	.000	.001
BPNSF20	<---	F4	.019	.001	.765	.000	.001
BPNSF8	<---	F4	.021	.001	.737	-.003	.001
BPNSF2	<---	F4	.022	.001	.707	-.001	.002
BPNSF21	<---	F5	.021	.001	.706	.001	.001
BPNSF14	<---	F5	.025	.001	.766	.002	.002
BPNSF9	<---	F5	.024	.001	.728	.002	.002
BPNSF3	<---	F5	.033	.002	.619	.002	.002
BPNSF23	<---	F6	.018	.001	.791	.000	.001
BPNSF17	<---	F6	.020	.001	.747	.002	.001
BPNSF11	<---	F6	.021	.001	.734	-.002	.001
BPNSF6	<---	F6	.022	.001	.651	-.003	.002

Intercepts: (g2 - Measurement residuals)

Parameter	SE	SE-SE	Mean	Bias	SE-Bias
**BPNSF19**	.043	.002	5.166	.004	.003
**BPNSF13**	.040	.002	5.065	.005	.003
**BPNSF7**	.043	.002	4.844	.005	.003
**BPNSF1**	.040	.002	4.716	.005	.003
**BPNSF18**	.042	.002	4.319	-.001	.003
**BPNSF15**	.045	.002	3.710	.000	.003
**BPNSF10**	.049	.002	3.185	-.003	.003
**BPNSF5**	.045	.002	3.739	-.003	.003
**BPNSF24**	.040	.002	5.227	.003	.003
**BPNSF16**	.042	.002	5.122	.005	.003
**BPNSF12**	.037	.002	5.223	.006	.003
**BPNSF4**	.038	.002	5.118	.002	.003
**BPNSF22**	.045	.002	3.118	-.004	.003
**BPNSF20**	.050	.003	2.507	-.009	.004
**BPNSF8**	.052	.003	2.803	-.002	.004
**BPNSF2**	.047	.002	2.390	-.003	.003
**BPNSF21**	.039	.002	5.207	.007	.003
**BPNSF14**	.041	.002	5.466	.007	.003
**BPNSF9**	.045	.002	5.683	.004	.003
**BPNSF3**	.040	.002	5.613	.003	.003
**BPNSF23**	.043	.002	2.240	-.007	.003
**BPNSF17**	.051	.003	2.573	-.006	.004
**BPNSF11**	.049	.002	2.750	-.001	.003
**BPNSF6**	.050	.003	2.569	.001	.004

Covariances: (g2 - Measurement residuals)

Parameter	SE	SE-SE	Mean	Bias	SE-Bias
F1 <--> F2	.025	.001	-.158	.000	.002
F2 <--> F3	.024	.001	-.152	.000	.002
F1 <--> F3	.046	.002	.724	-.002	.003
F2 <--> F4	.051	.003	.322	.000	.004
F3 <--> F4	.044	.002	-.601	-.003	.003
F1 <--> F4	.042	.002	-.487	-.001	.003
F2 <--> F5	.024	.001	-.145	.000	.002
F4 <--> F5	.042	.002	-.663	.002	.003
F3 <--> F5	.053	.003	.770	-.001	.004
F1 <--> F5	.074	.004	.897	.001	.005
F6 <--> F5	.058	.003	-.957	.002	.004
F6 <--> F3	.049	.002	-.645	-.002	.003
F6 <--> F4	.068	.003	1.268	-.004	.005
F6 <--> F2	.057	.003	.376	-.001	.004
F6 <--> F1	.055	.003	-.621	-.002	
