# Supplementary material for: The basic psychological needs satisfaction and frustration scale at work: A validation in the Polish language
Source: PLoS One. 2021 Nov 4;16(11):e0258948. doi: 10.1371/journal.pone.0258948 (PMC8568341; doi:10.1371/journal.pone.0258948)
Supplement: Attachment — Submitted filename: Comments to the Authors.pdf [file pone.0258948.s003.pdf]

In their manuscript, the authors validate the BPNSF scale within a Polish context. I enjoyed the use of large samples and the strong statistical background. My only larger concern centers around missing a replicable code to rerun the analysis (see comment 1).

I structured my review along the following lines: General comments and minor comments. Each section is structured by perceived importance of the comment. Parts I copy-pasted from the manuscript are in quotation marks.

General comments

1. Some parts of the results section were not 100% clear to me. How exactly was invariance established? When I think of measurement invariance I think of multilevel SEMs (Fischer & Karl, 2019). The approach you are using seems sounds but for less experienced readers an additional explanation (+ a reference at the beginning of the invariance section) that explains the approach or publishing the analysis code (maybe just the AmostOutput?) next to the dataset could be helpful?
2. Related to the above point I was wondering whether a cross-cultural sample would be needed to really establish measurement invariance? At the moment it seems that we can conclude that asking different samples within Poland will produce solid scales across those samples. However, can we compare the findings from Poland to other countries? Maybe this is not the goal of the study though :)
3. I was wondering in which language the questionnaires were distributed. I imagine the BPNSFS was presented in Polish. Were the other items also presented in Polish? Are they validated in that language? If not, would that need to be discussed in the limitations?
4. Within the first paragraph, I was wondering what low-quality vs. high-quality motivation stands for? What Vansteenkiste and colleagues (2009) would term good vs. poor quality motivation groups?
5. Very nice literature review on need frustrations within the organizational context. If the authors would like to broaden their literature review to other areas of research on need frustration some additional references could give the paper a broader audience e.g. (Leander et al., 2019, 2020; Stollberg et al., 2015; Williams, 2009)
6. Nice limitations; great picking up on the gender distribution. Does the dropout (and completion rates leading to them) also need a short discussion?
7. I understand the reason behind summing up the items (Table 1); as there has been some recent debate around obtaining multiple-item scale scores through summing (McNeish & Wolf, 2020), alternative strategies could have been used as well. I am not sure though whether the authors need to engage with this comment; just a thought :)
8. Unfortunately, some of the tables were cut off. Maybe an explanation underneath could help the reader understand the different concepts (e.g. in Table 1 what does the *s* stand for?)

Minor comments

1. On page 16 it could be handy to refer to the exact number of cases that were excluded? Six I believe?

2. Some sentences could be streamlined (e.g. in the abstract (p. 9) “has been validated” could be “was validated” as the process of validation is now complete)
3. There is still the one or other typo; maybe a fresh pair of eyes could help finding those
    a. (e.g. (p. 10) “yet he or she may not feel [incompetence or failure regarding their work]”
    b. (p. 11) …, the purpose of this project was to validate the BSNSFS-Work Domain [in Poland].

References

Fischer, R., & Karl, J. A. (2019). A primer to (cross-cultural) multi-group invariance testing possibilities in R. *Frontiers in Psychology*, *10*(JULY), 1–18. https://doi.org/10.3389/fpsyg.2019.01507

Leander, N. P., Agostini, M., Stroebe, W., Kreienkamp, J., Spears, R., Kuppens, T., Van Zomeren, M., Otten, S., & Kruglanski, A. W. (2020). Frustration-affirmation? Thwarted goals motivate compliance with social norms for violence and nonviolence. *Journal of Personality and Social Psychology*. https://doi.org/10.1037/pspa0000190

Leander, N. P., Stroebe, W., Kreienkamp, J., Agostini, M., Gordijn, E., & Kruglanski, A. W. (2019). Mass shootings and the salience of guns as means of compensation for thwarted goals. *Journal of Personality and Social Psychology*, *116*(5), 704–723. https://doi.org/10.1037/pspa0000150

McNeish, D., & Wolf, M. G. (2020). Thinking twice about sum scores. *Behavior Research Methods*, *52*(6), 2287–2305. https://doi.org/10.3758/s13428-020-01398-0

Stollberg, J., Fritsche, I., & Bäcker, A. (2015). Striving for group agency: threat to personal control increases the attractiveness of agentic groups. *Frontiers in Psychology*, *6*(May), 1–13. https://doi.org/10.3389/fpsyg.2015.00649

Vansteenkiste, M., Sierens, E., Soenens, B., Luyckx, K., & Lens, W. (2009). Motivational Profiles From a Self-Determination Perspective: The Quality of Motivation Matters. *Journal of Educational Psychology*, *101*(3), 671–688. https://doi.org/10.1037/a0015083

Williams, K. D. (2009). Ostracism: A temporal need-threat model. In M. P. Zanna (Ed.), *Advances in experimental social psychology, Vol 41.* (pp. 275–314). Elsevier Academic Press. https://doi.org/10.1016/S0065-2601(08)00406-1
