# Supplementary material for: The basic psychological needs satisfaction and frustration scale at work: A validation in the Polish language
Source: PLoS One. 2021 Nov 4;16(11):e0258948. doi: 10.1371/journal.pone.0258948 (PMC8568341; doi:10.1371/journal.pone.0258948)
Supplement: Attachment — Submitted filename: RESPONSE TO REVIEWERS.doc [file pone.0258948.s004.doc]

Dear Reviewers,

Thank you for all valuable comments, which enriched the article. We have implemented the suggested changes. We feel that the text has become clearer. We hope that this will enable the publication of our manuscript. Below are our responses.

Reviewer #1: This paper seems to be a piece of valuable work to provide a Polish language validated BPNSFS to future researchers. The authors also acknowledged limitations of this study.

As mentioned in the paper that the scale has been validated in Polish by Kuzma et al (2020), is it possible to add a few sentences on how is this validation different to theirs or the further contributions?

Re: We added text fragments which are comparing the general version of the scale with the current BPNSFS adaptation at work domain in introduction and in further research sections of the manuscript. We emphasized the differences between the two scales and their functionality.

I understand that this BPNSFS has 6 dimensions like previous findings, is there any comparisons between the criterion validity of this scale with previous findings?

Re: The general version of the BPNSFS scale has been to date adopted and validated into many different languages, such as Japanese, Portuguese, Hebrew, Polish and Arabic (Nishimura & Suzuki, 2016, Costa et al., 2017, Benita et al., 2020; Kuźma et al., 2020; Zayed et al., 2021). However, the BPNSFS at work domain has only one validation in two languages English and Norwegian (Olafsen, 2021). The criterion validity for both Polish and Norwegian/English BPNSFS at work validations were checked by measuring associations between the subscales of BPNSFS and other constructs which are connected with positive and negative phenomena at work. In Polish adaptation the criterion validity was assessed on the basis of associations with the level of stress, emotional exhaustion (burnout), self-efficacy, job crafting and work engagement. The author of the English and Norwegian validation checked for associations with managerial need support, motivation (different forms), vigor, emotional exhaustion (burnout), affective commitment and turnover intention. Both in Polish and in Norwegian and English validations it was expected that satisfaction of needs will be positively associated with positive work phenomena (motivation, self-efficacy) and negatively with negative work phenomena (burnout, stress), and with the frustration of needs the relations will be inverted. In both adaptations the results confirmed the researchers’ assumptions.

Olafsen, A. H. (2021). The Basic Psychological Need Satisfaction and Need Frustration at Work Scale: A Validation Study. *Frontiers in Psychology*, *12*, 2708.

Reviewer #2: In their manuscript, the authors validate the BPNSF scale within a Polish context. I enjoyed the use of large samples and the strong statistical background. My only larger concern centers around missing a replicable code to rerun the analysis (see comment 1).
I structured my review along the following lines: General comments and minor comments. Each section is structured by perceived importance of the comment. Parts I copy-pasted from the manuscript are in quotation marks.

General comments
1.Some parts of the results section were not 100% clear to me. How exactly was invariance established? When I think of measurement invariance I think of multilevel SEMs (Fischer & Karl, 2019). The approach you are using seems sounds but for less experienced readers an additional explanation (+ a reference at the beginning of the invariance section) that explains the approach or publishing the analysis code (maybe just the AmostOutput?) next to the dataset could be helpful?

Re: Indeed there are various approaches describing the steps necessary to establish invariance. On one of the pages of our manuscript, just before the results section, there is a reference at the beginning explaining the approach that was undertaken in our paper.

…we also conducted invariance analysis including different types of invariance testing (e.g., configural and metric) following steps recommended by the Vandenberg and Lance approach (2000) and summarized by Milfont and Fischer (2010)…

However, we also added additional information within invariance section agreeing with your comment.

We have also prepared the Amos output file in pdf format for the most important analysis (invariance). It would not be possible to attach all output files due to limited frames/space available, however all of them could be send on request.

2.Related to the above point I was wondering whether a cross-cultural sample would be needed to really establish measurement invariance? At the moment it seems that we can conclude that asking different samples within Poland will produce solid scales across those samples. However, can we compare the findings from Poland to other countries? Maybe this is not the goal of the study though :)

Re: Indeed, the goal of the study was to prepare a solid validation of the scale within Polish settings. Invariance analysis is widely used in various subpopulations and not only for multicultural comparisons. We believe that it would be really desirable also to do some multicultural comparisons, however it was not the aim of our study at the moment. We are open for further international cooperation to compare the results across the countries. Perhaps in the short future it will be possible.

3.I was wondering in which language the questionnaires were distributed. I imagine the BPNSFS was presented in Polish. Were the other items also presented in Polish? Are they validated in that language? If not, would that need to be discussed in the limitations?

Re: All questionnaires used in the validity analysis were distributed in Polish language. These questionnaires were validated in Poland, in the previous studies. In the manuscript, we have added references to Polish-language publications devoted to the tools used in the research: COPSOQ (Baka, 2019), OLBI (Baka & Basińska, 2020), UWES (Szabowska-Walaszczyk et al., 2011), JCS (Rogala & Cieslak, 2019), OSS-SF (Grala & Baka, 2021, in preparation). Thank you for the comment.

4. Within the first paragraph, I was wondering what low-quality vs. high-quality motivation stands for? What Vansteenkiste and colleagues (2009) would term good vs. poor quality motivation groups?

Re: According to SDT, and more precisely one of its subtheories – Organismic Integration Theory (OIT; Ryan & Deci, 2017) – there are six different types of motivational regulation, which vary in their antecedents, the degree of perceived autonomy, and effects on behavior. The six types of motivation are amotivation, external regulation, introjection, identification, integration and intrinsic motivation. The differentiation between low (poor) or high (good) quality motivations is more general (than in OIT theory) which assumes that all controlled types of motivation (external regulation, intojection) are considered low (poor) quality motivation and all autonomic types of motivation (identification, integration and intrinsic motivation) are considered high (good) quality motivation. As this work is set more in Basic Psychological Needs Theory (OIT; Ryan & Deci, 2017) we only used more general terms to introduce the topic.

5. Very nice literature review on need frustrations within the organizational context. If the authors would like to broaden their literature review to other areas of research on need frustration some additional references could give the paper a broader audience e.g. (Leander et al., 2019, 2020; Stollberg et al., 2015; Williams, 2009)
6. Re: Thank you for the recommendations, we have added some of the reference to the manuscript.
6. Nice limitations; great picking up on the gender distribution. Does the dropout (and completion rates leading to them) also need a short discussion?
   Re: Unfortunately, we do not have access to incomplete questionnaires (we did not include them in the database), therefore it will be difficult to make any comparisons. However, in our opinion there is no need to mention about dropouts in the discussion as the problem is less relevant in the case of the cross-sectional study (contrary to longitudinal studies where it is essential).
7. I understand the reason behind summing up the items (Table 1); as there has been some recent debate around obtaining multiple-item scale scores through summing (McNeish & Wolf, 2020), alternative strategies could have been used as well. I am not sure though whether the authors need to engage with this comment; just a thought :)
   Re: We believe that we are aware of advantages and disadvantages of calculations of indices based on the sum, average, and/or factor scores. Obviously, calculations based on sum are probably the most problematic especially with larger number of missing data. However, even calculations based on average might be problematic, as mean could be calculated dividing by the total number of questions in the questionnaire (or within particular subscale) vs. number of questions which were answered by particular person in the study within particular scale. This accommodates possible missing answers to the better extent. The last approach is probably the most sophisticated (indices based on factor scores) as it takes into consideration a relative weight/contribution of particular item while calculating an index representing the final score. This comes, however with a price, as indices based on factor scores are more difficult to interpret, especially in terms of descriptive statistics. Obviously, it is not always easy to decide but the decision has to be made anyway. So, in our paper we were mostly operating on the raw scores for particular items as we used CFA analysis including invariance analysis so the problem of type of the indices used was not relevant, but in the descriptive statistics table we decided to present indices based on sum for the ease of interpretation for the readers especially that particular scales had various ranges.
8. Unfortunately, some of the tables were cut off. Maybe an explanation underneath could help the reader understand the different concepts (e.g. in Table 1 what does the s stand for?)

Re: We have corrected the tables, and we hope they will not change during saving into Plos pdf format. The word which was cut off was skewness, and only the letter “s” was left in the table.

Minor comments
1.On page 16 it could be handy to refer to the exact number of cases that were excluded? Six I believe?
Re: Indeed six cases were eliminated. The process of elimination of cases was mentioned and described on page 11. However, we only mentioned that few cases were eliminated without specifying the exact number. On the other hand the information on the preliminary and final sample were given so it was possible to conclude the number of cases excluded. Anyway, we added the precise information on number of removed cases.

2.Some sentences could be streamlined (e.g. in the abstract (p. 9) “has been validated” could be “was validated” as the process of validation is now complete)
Re: we used „has been validated” as it implies that the work is complete now and the scale is ready to use.

3.There is still the one or other typo; maybe a fresh pair of eyes could help finding those
a.(e.g. (p. 10) “yet he or she may not feel [incompetence or failure regarding their work]”
b.(p. 11) …, the purpose of this project was to validate the BSNSFS-Work Domain [in Poland].-
Re: We checked the typos and other small mistakes, thank you for pointing at some of them .
